# New paradigms for understanding and step changes in treating active and chronic, persistent apicomplexan infections

**DOI:** 10.1038/srep29179

**Published:** 2016-07-14

**Authors:** Martin McPhillie, Ying Zhou, Kamal El Bissati, Jitender Dubey, Hernan Lorenzi, Michael Capper, Amanda K Lukens, Mark Hickman, Stephen Muench, Shiv Kumar Verma, Christopher R. Weber, Kelsey Wheeler, James Gordon, Justin Sanders, Hong Moulton, Kai Wang, Taek-Kyun Kim, Yuqing He, Tatiana Santos, Stuart Woods, Patty Lee, David Donkin, Eric Kim, Laura Fraczek, Joseph Lykins, Farida Esaa, Fatima Alibana-Clouser, Sarah Dovgin, Louis Weiss, Gael Brasseur, Dyann Wirth, Michael Kent, Leroy Hood, Brigitte Meunieur, Craig W. Roberts, S. Samar Hasnain, Svetlana V. Antonyuk, Colin Fishwick, Rima McLeod

**Affiliations:** 1 University of Leeds, Leeds, UK; 2 University of Chicago, Chicago, USA; 3 USDA, Beltsville, Maryland, USA; 4 J Craig Venter Institute, Rockville Maryland, USA; 5 University of Liverpool, Liverpool, UK; 6 Harvard School of Public Health, Boston, Massachusetts, USA; 7 The Broad Institute, Boston, Massachusetts, USA; 8 Walter Reed Army Institute of Research, Silver Spring, Maryland, USA; 9 Oregon State University, Corvallis, Oregon, USA; 10 Institute for Systems Biology, Seattle, Washington, USA; 11 Albert Einstein College of Medicine, Bronx, New York, USA; 12 Strathclyde University, Glasgow, Scotland, UK; 13 CNRS, Marseilles, France; 14 Institute for Integrative Biology of the Cell (12BC), Gif-sur-Yvette, France

## Abstract

*Toxoplasma gondii*, the most common parasitic infection of human brain and eye, persists across lifetimes, can progressively damage sight, and is currently incurable. New, curative medicines are needed urgently. Herein, we develop novel models to facilitate drug development: EGS strain *T. gondii* forms cysts *in vitro* that induce oocysts in cats, the gold standard criterion for cysts. These cysts highly express *cytochrome b*. Using these models, we envisioned, and then created, novel 4-(1*H*)-quinolone scaffolds that target the cytochrome *bc*_*1*_ complex Q_i_ site, of which, a substituted 5,6,7,8-tetrahydroquinolin-4-one inhibits active infection (IC_50_, 30 nM) and cysts (IC_50_, 4 μM) *in vitro,* and *in vivo* (25 mg/kg), and drug resistant *Plasmodium falciparum* (IC_50_, <30 nM), with clinically relevant synergy. Mutant yeast and co-crystallographic studies demonstrate binding to the *bc*_*1*_ complex Q_i_ site. Our results have direct impact on improving outcomes for those with toxoplasmosis, malaria, and ~2 billion persons chronically infected with encysted bradyzoites.

*Toxoplasma gondii* infections can cause systemic symptoms, damage and destroy tissues^1^,^2^,^3^,^4^,^5^,^6^,^7^,^8^,^9^,^10^, especially eye and brain^1^,^2^,^3^,^4^,^5^,^6^,^7^,^8^,^9^,^10^ and cause fatalities^S1–20^. Primary infections may be asymptomatic, or cause fever, headache, malaise, lymphadenopathy, and rarely meningoencephalitis, myocarditis, or pericarditis^9^,^11^,^12^. Retinochoroiditis and retinal scars develop in up to 30% of infected persons^1^,^7^,^13^, and epilepsy may occur^6^,^14^. In immune-compromised and congenitally infected persons, active infection frequently is harmful^1^,^2^,^3^,^4^,^5^,^6^,^7^,^8^,^9^,^10^. Recrudescence arises from incurable, dormant cysts throughout life^6^,^7^,^9^,^10^. In rodents, chronic infection alters fear, smell, reward pathways, neurotransmitters such as GABA and dopamine, and causes abnormal neurologic functions^15^. Although this parasite is present in the brains of 2–3 billion persons worldwide, consequences are unknown. Neurobehavioral abnormalities and differences in serum cytokines, chemokines, and growth factors were associated with seropositivity in humans^16^,^17^. Estimates of costs for available suboptimal medicines to treat active, primary ocular, gestational, and congenital infections, in just the U.S. and Brazil, exceed $5 billion per year (Table 1).

Current treatments against active *T. gondii* tachyzoites can have side effects such as hypersensitivity, kidney stones, and bone marrow suppression, limiting their use^10^. Latent bradyzoites are not significantly affected by any medicines^6^. Atovaquone partially, and transiently, limits cyst burden in mice^18^, but resistance develops with clinical use^19^,^20^,^21^,^22^,^23^,^24^. Thus, *T. gondii* infection is incurable with recrudescence from latent parasites posing a continual threat. Improved medicines are needed urgently. Molecular targets shared by *T. gondii* and *Plasmodia* make re-purposing compounds a productive strategy.

Critical flaws and limitations of available methods and models for developing medicines to cure *T. gondii* infections include lack of *in vitro* culture systems for cysts and scalable, easy to use animal models for screening compounds. To address these challenges, we characterized the EGS parasite (Fig. 1), isolated in 1994 from amniotic fluid of a congenitally infected Brazilian fetus^25^, that form cyst-like structures *in vitro.* In our characterization of EGS *in vitro*, herein, we discovered that true cysts develop, making EGS especially useful for drug development. EGS parasites can infect zebrafish, and we have characterized this, as well as a fluorescent tachyzoite and cyst assay in this new model^26^. Further, cytochrome *bc*_*1*_ expression is markedly increased in encysted EGS bradyzoites suggesting cytochrome *bc*_*1*_ might be a viable drug target for this life stage. This mitochondrial membrane bound protein complex cytochome *bc*_1_, part of the electron transport chain responsible for generating ubiquinone for pyrimidine biosynthesis in *Plasmodium*, is the molecular target of the naphthoquinone, atovaquone^27^,^28^,^29^,^30^,^31^,^32^,^33^,^34^,^35^,^36^,^37^,^38^,^39^,^40^,^41^,^42^,^43^,^44^,^45^,^46^,^47^,^48^,^49^,^50^,^51^,^52^. Partial efficacy, rapid emergence of drug resistance in malaria and toxoplasmosis limit clinical usefulness of atovaquone. We present new 4-(1*H*)-quinolone scaffolds that target the Q_i_ site of cytochrome *bc1* in apicomplexan parasites. Our lead 5,6,7,8-tetrahydroquinolin-4-one compound, MJM170, is highly effective against apicomplexan parasites and has substantially enhanced solubility compared with other reported quinolones due to its’ new scaffold. Direct visualisation in the crystallographic structure opens the way to design a new generation of compounds for both parasites.

## Results

### Characterization of EGS strain develops novel *in vitro* models to test compounds

#### Genotyping and Phylogenetic Analysis of EGS

**Figure 1:**
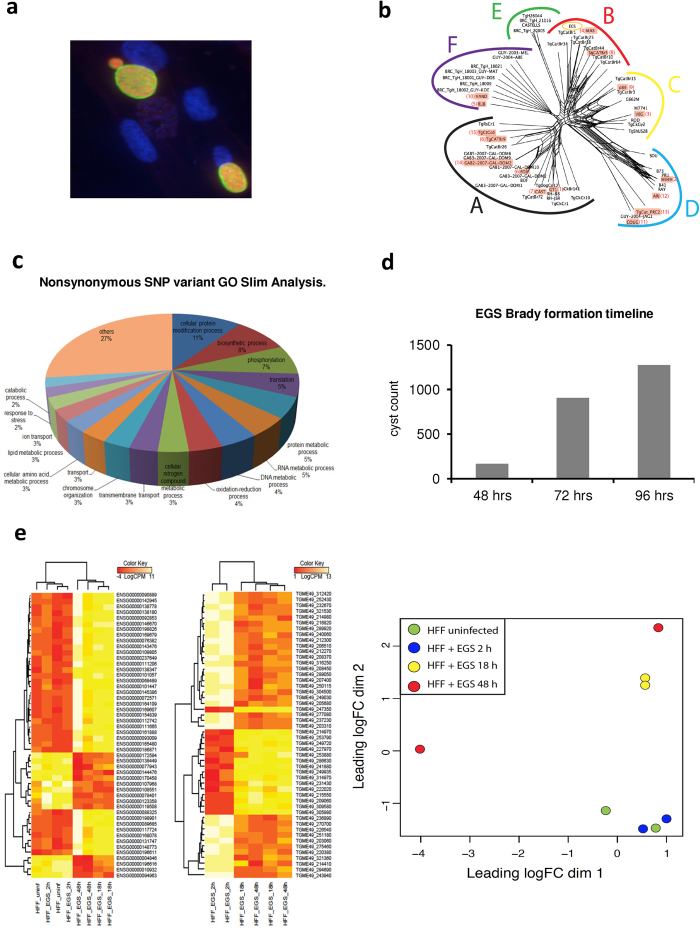
**Characterization of Brazilian (TgBr EGS) *T. gondii*, originally isolated from amniotic fluid in 1994, establishes novel *in vitro* model**. (**a**) *In vitro* EGS cultures in HFF cells form cysts. Note green dolichos immunstaining of the perimeter cyst wall, red bradyzoite antigen 1 (BAG1) immunostaining, and blue DAPI staining of DNA. These cultures produce oocysts when fed to cats (see Fig. 2). (**b**) Genetic characterization of EGS strain. Neighbor-net analysis based on 790,168 genome-wide SNPs common to EGS strain plus 62 parasite isolates representing all major population haplogroups that have been described for *T. gondii* demonstrated that EGS belongs to clade B, haplogroup 4. Groups A to F indicate major population clades of *T. gondii*. Haplogroup numbers are indicated within parentheses based on previous designation. Names within red rectangles denote the representative strains from each haplogroup. (**c**). Full genome sequence analysis of EGS compared with canonical and geographically closely related parasite genomic sequences reveal a non-synonymous mutation and disordered c terminal sequence in Apetela 2(AP2) IV-iv, a bradyzoite repressor. Because AP2 IV-iv is a bradyzoite repressor, a mutation could create a parasite like EGS that remains as an encysted bradyzoite. Biological process GO representation among EGS genes carrying non-synonymous SNPs not present in other canonical and phylogenetically closely related parasite genomic sequences. This analysis revealed unique non-synonymous SNPs in Apetela 2 transcription factor genes and other genes shown in Supplement A: Figure S1 and Supplement B: Excel Table S1 which shows the Go Slim Analysis. The effect on transcription may reflect the disordered region and nonsynomous mutation in AP2 IV-iv. (**d**) Cysts of EGS enlarge with time in culture in HFF. This enlargement is visible over the initial 96 hours in culture. (**e**) DGE analysis of EGS cultures in HFF reveals changes over time in both host and parasite transcriptomes. Left and middle panels depict heat maps of the 50 most upregulated HFF and EGS genes respectively. Right panel: MDS plot of HFF gene expression profiles from uninfected human fibroblast or infected with EGS parasites for 2, 18 or 48 hours. The full data set is in Supplement B: Excel Table S2.

We isolated and sequenced genomic DNA from the EGS^25^ parasite, which formed cysts when grown in human foreskin fibroblasts (HFF) in culture, Fig. 1a (left panel). Phylogenetic analysis based on 796,168 SNPs across 62 *T. gondii* genomes revealed that EGS is closely related to other Brazilian strains including TgCatBr1, TgCatBr18 and TgCatBr25 and ancient South American MAS (Fig. 1b). All these grouped to clade B, haplogroup 4 and 8. EGS differs from other isolates by non-synonymous SNPs in Apetela 2 IV-iv, M=>I (570) and a disordered area beginning at 821, GGNRPHYHVAKQEWRVRYYMNGKRKMRTYSAKFY GYETAHTMAEDFAHYVDKHE. AP2 IV-iv is a member of the plant-like transcription factor family unique to apicomplexan parasites. This AP2 represses bradyzoite to tachyzoite conversion, among other differences (Table 1; Fig. 1c; Supplement A: Box and Figure S2, Supplement B: Excel Table S1).

### Phenotypes of EGS in Human cells *in vitro*, and in Cats and Mice

#### EGS in human foreskin fibroblasts (HFF)

*In vitro*, these EGS parasites form cysts that enlarge over ~96 hours and then destroy monolayers as single cell organisms (Fig. 1d). This created novel, useful *in vitro* models. Cyst walls are thick in electron micrographs (data not shown). Cyst-like structures’ perimeters demonstrate dolichos (green), with bradyzoites within them staining with BAG1 (red) and nuclei with Dapi (blue), Fig. 1a. Kinetic analysis of EGS in HFF cultures, 2, 18, and 72 hours after infection, RNA-seq and MiR-seq results demonstrated varied expression signatures over time in culture (Fig. 1e and Supplement B: Excel Tables S2 and S3) with expression of bradyzoite markers by 18 hours and Apetela 2 signatures by 2 hours.

#### Cats fed EGS in HFF cultures or mouse brain produce oocysts

**Figure 2:**
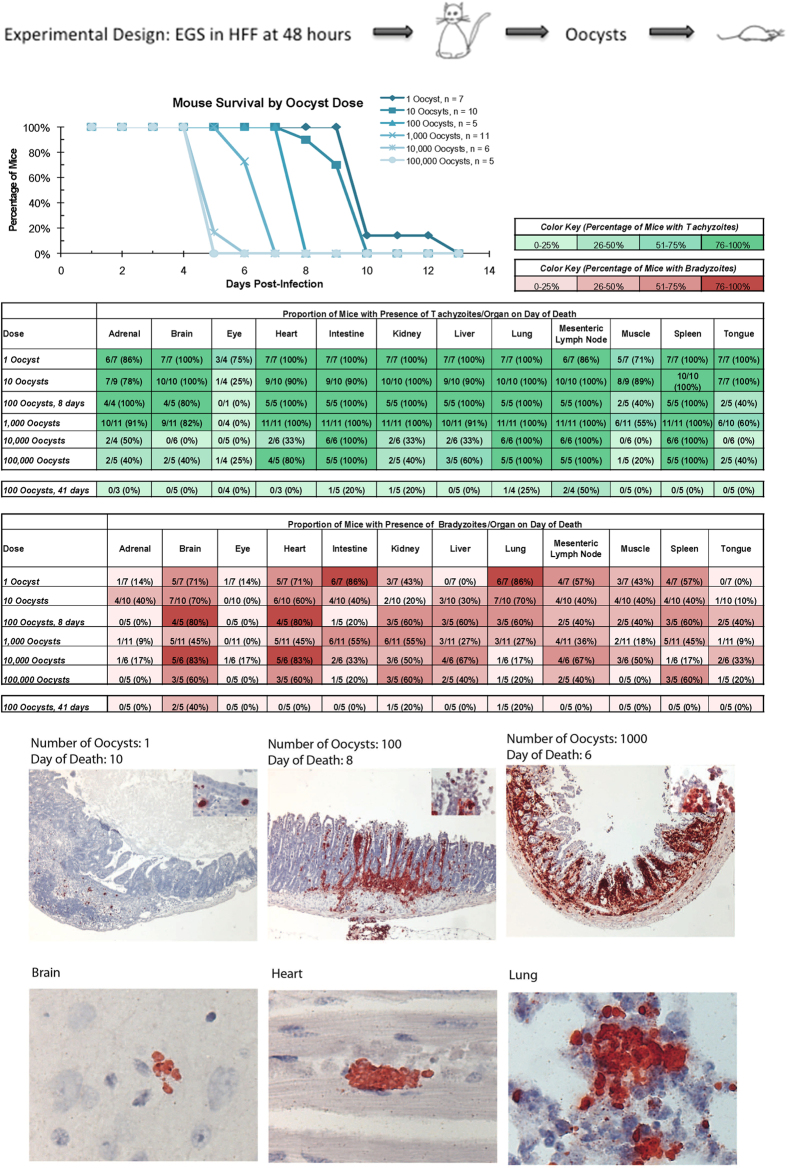
EGS is the only *T. gondii* cultured for more than 30 passages demonstrated to form an encysted bradyzoite when cultures of these parasites are tested by gold standard *in vivo* assay. This EGS given as oocysts produced from feeding EGS cysts in HFF to mice behaves as a typical virulent and pathogenic Brazilian parasite. The dose related infection of terminal ileum, dissemination to lung, ear and brain, presence of tachyzoites and then bradyzoites in multiple organs, and dose related mortality resembles infection of mice with other Brazilian strains characteristic of a typical virulent organism.

When HFF tissue cultures with these cyst structures as seen in Fig. 1a were fed to cats, they developed the classic, gold standard bradyzoite phenotype of producing oocysts (Fig. 2) in two replicate experiments. All other *T. gondii* strains cultured for more than 30 passages, as EGS was since the 1990s, lose the ability to produce oocysts when fed to cats (JP Dubey, personal observations, 1975–2015). This experiment established that these were true bradyzoites in cysts formed *in vitro* under standard culture conditions. Oocysts also formed 10 days after feeding cats mouse brains infected with EGS stably transfected with tachyzoite SAG1 promoter-driven mcherry, and bradyzoite BAG1 promoter-driven green fluorescent protein (GFP), and merozoite promoter-driven blue fluorescent protein, engineered to facilitate creation of automated, scalable *in vitro* and *in vivo* assays. *In vitro*, these promoters did not provide a fluorescence signal robust enough to detect differences between 2 × 10^5^ and 650 parasites useful in scalable assays (data not shown).

#### EGS is virulent in Mice

When these EGS oocysts were fed to mice they produced disease indistinguishable from other virulent Brazilian strains. Oocysts given to mice per-orally created an illness and histopathology phenotypically characteristic for typical, virulent parasites causing dose related proliferation of *T. gondii* with necrosis in terminal ileum, pneumonia at 9–10 days, with brain parasites by 17 days and dose-related mortality (Fig. 2).

#### EGS has a bradyzoite/cyst morphology and alters the transcriptomes of the biologically relevant human monocytic cell line MM6 and human primary neuronal stem cells (NSC)

**Figure 3:**
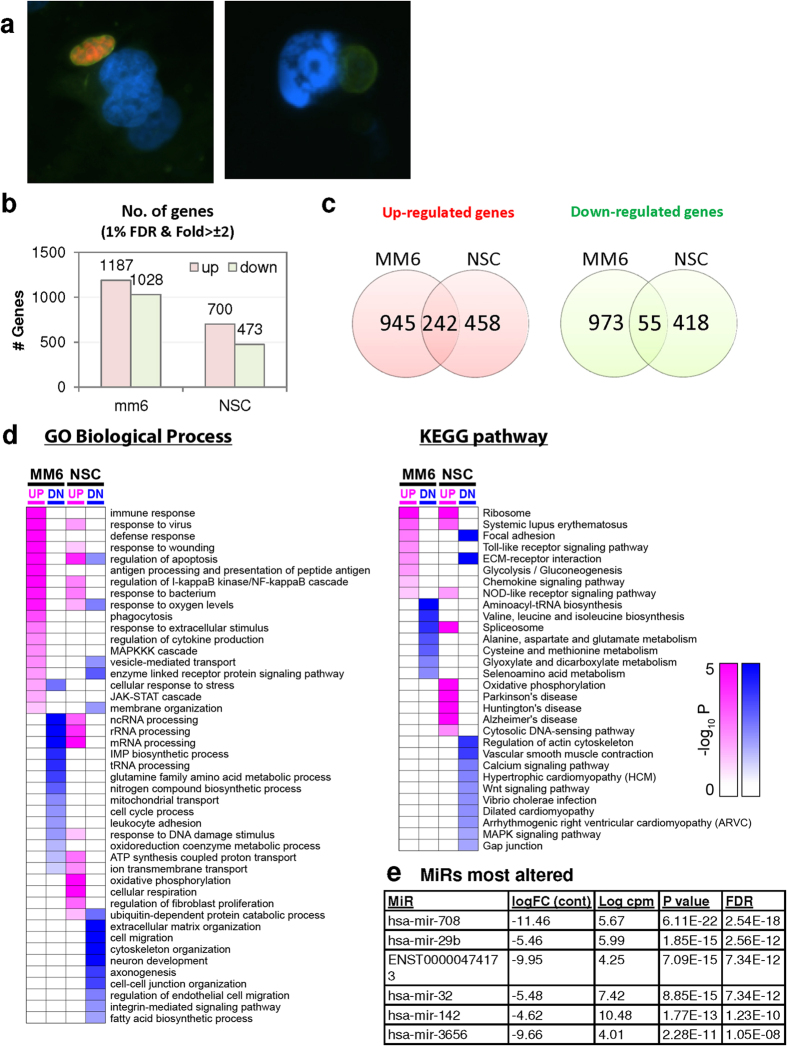
EGS morphology and effect on host cell transcriptomes. (**a**) EGS in human MM6 cells and NSC form cysts. Left NSC with EGS. Right MM6 with EGS. Note green dolichos cyst walls and BAG1(red) in NSC. DAPI stained nuclei(blue). (**b**–**d**) Effects of EGS infection on MM6 and NSC transcriptomes: EGS transcripts in MM6 compared with NSC shows overlap of, as well as unique patterns of, transcripts. Differentially expressed genes in MM6 and NSC cells infected with EGS parasite were identified based on criteria of 1% FDR and absolute fold-change ≥2. Number of DEGs in each cell line are presented with bar graph (**b**) and Venn diagram are used to show general comparison of DEGs identified between the two cell lines (**c**). There is both commonality, overlap in genes modulated and independence in others between cell types indicating cell type also influences transcriptome. Red and green colors were used to represent up- and down-regulated genes, cell line used is indicated on bottom (**b–d**). Functional enrichment analysis was performed for gene ontology (GO) biological process and KEGG pathways (**d**). P-values derived from analysis were -log10 transformed and presented as a heat map. Pink and blue colors indicate GO terms or KEGG pathways enriched by up- and down-regulated genes, respectively. Enriched pathways or biological processes are listed on right of panels and cell lines are indicated on top. **e.** Host cell miR-seq analysis reveals that EGS regulates host cell miRNAs critical in pathogenesis and latency. Data are in Supplement B: Table S8. An especially interesting down-modulated miRNA is hsa-miR-708-5p which is expressed particularly in brain and retina cells causing apoptosis^65^. When *T. gondii* downmodulates this as an encysted bradyzoite in neuronal cells, it would prevent hosts from initiating apoptosis to eliminate chronically infected neurons. (**f**). Parasite genetics and human host cell type have a profound influence on *T. gondii* gene expression. MDS plot comparing *T. gondii* gene expression profiles from MM6 and NSC cells infected with EGS, GT1, ME49 and VEG strains for 18 hours and HFF cell cultures infected with EGS strain for 2, 18 and 48 hours.

**Figure 4:**
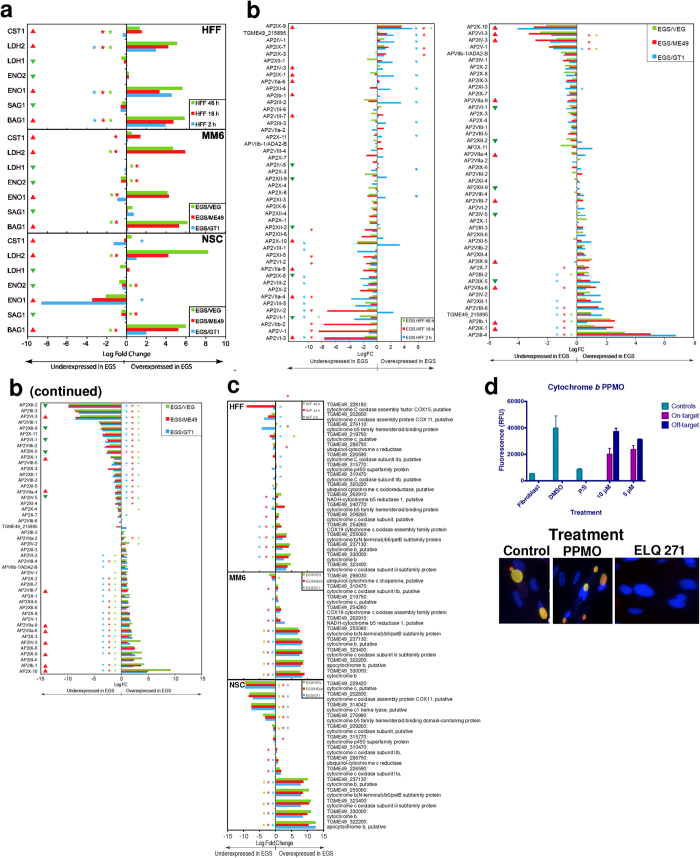
Differential Gene Expression (DGE) analyses and effects of inhibition of cytochrome *bc1*. (**a**) DGE analysis of bradyzoite- and tachyzoite-specific markers during EGS infections of HFF cultures at 2, 18 and 48 hours (top panel), MM6 cells at 18 hours (middle panel) or NSC cultures at 18 hours (bottom panel) versus infections of same host cells with canonical strains GT1, ME49 or VEG at 18 hours (averaged across the three canonical strains for HFF infections). Genes reported as being over- or under-expressed during bradyzoite differentiation is indicated with red or green arrows respectively. “*”, q-value ≤ 0.05; LogFC, logarithm of the fold change in gene expression. CST1, SAG-related sequence SRS44^s114^; LDH2, lactate dehydrogenase 2^S115^; LDH1, lactate dehydrogenase 1^S115^; ENO2, enolase 2; ENO1, enolase 1^S116^; SAG1, SAG-related sequence SRS29B; BAG1, bradyzoite antigen BAG1^S115^. (**b**) DGE analysis of genes encoding AP2 family of transcription factor during the same infection experiments as described in (**a**). Red and green arrows denote AP2 genes found to be over- or under-expressed during bradyzoite development^S117^. “*”, q-value ≤ 0.05. (**c**) *T. gondii* cytochrome family genes differentially expressed during same experimental infections as described in (**a**). “*”, q-value ≤ 0.05. (**d**) Effect of known cytochrome b inhibitors on EGS. Morpholino conjugated to a Vivoporter (called PPMO) designed to knock down cytochrome *b* compared with off target control has a significant effect in reducing replication of YFP RH strain tachyzoites at 5 and 10 μM (p < 0.05) but only a very small effect on size and number of EGS cysts in HFF. As a poorly soluble inhibitor of cytochrome *b,* ELQ271 was reported to partially reduce cyst numbers in mice^27^ and is shown herein also to reduce the EGS cysts *in vitro* at 10 μM in this novel model. This demonstrates the utility of this novel *in vitro* model by indicating that inhibition of cytochrome *b* Qi is associated with reduction of cysts *in vivo* in a mouse model, even when there are serious limitations caused by insolubility of this inhibitory compound. This poor solubility significantly limits ELQ271 as a candidate for progression to a medicine. Increasing selectivity for the parasite enzyme with our new scaffold is another critical challenge.

Human cells particularly relevant to human toxoplasmosis were infected with different strains of *T. gondii* to better characterize EGS parasites. Immunofluorescence staining of EGS-infected MM6 and NSC cultures revealed the development of cysts (Fig. 3a) and accordingly, EGS gene expression resembled that of bradyzoites when compared to equivalent infections done with GT1, ME49 or VEG strains. Interestingly, EGS transcription was influenced by host cell type (Fig. 3). Transcriptomics using host mRNA and miR profiling of EGS cultures in MM6, and NSC cells for 18 hours demonstrated that this parasite modulates host transcripts involved in protein misfolding, neurodegeneration, endoplasmic reticulum stress, splicesosome alteration, ribosome biogenesis, cell cycle, epilepsy, and brain cancer among others (Fig. 3 and Supplement B: Excel Tables S4–S8). The number of genes significantly up or down regulated in MM6 and NSC cells compared to uninfected controls are depicted in Fig. 3. Overexpressed genes differ from those of GT1, ME49 and VEG tachyzoite-infected human NSC cells (Fig. 4a), but modify the same or connected pathways (McLeod *et al.* unpublished observations). Hsa-miR-708-5p was the most affected miRNA (down-modulated) by EGS (Fig. 3; Supplement B: Table S8). miR-708-5p is a regulator that promotes apoptosis in neuronal and retinal cells, which could maintain a niche for EGS-like encysted bradyzoites to persist.

#### EGS transcripts demonstrate importance of cytochromes and key Apetela 2 transcription factors in this life cycle stage

EGS transcripts in HHF, MM6, and NSC cells were enriched for genes transcribed in bradyzoites, including known bradyzoite transcripts, certain Apetela 2s and cytochrome *b* and other cytochromes (Fig. 4b,c). Among transcripts with the most increased fold change in EGS across all three cell lines were: cytochrome b; cytochrome c oxidase subunit III subfamily protein; apocytochrome *b*; cytochrome *b*, putative; and cytochrome *b* (N-terminal)/b6/petB subfamily protein. Other over-expressed genes include bradyzoite transcription factor AP2IX-9 and plant-like heat-shock protein BAG1 (Fig. 4a–c).

### Identifying novel and efficacious compounds against *T. gondii* cytochrome *bc*_1_

Increased expression of cytochromes in EGS (Fig. 4c) made it pertinent to synthesize and test an endochin-like quinolone (ELQ) 271, which was previously reported to inhibit *T. gondii* cytochrome *bc*_*1*_ Q_i_ site and reduce, but not eliminate, brain cyst numbers in mice^27^. ELQ271 also inhibited EGS *in vitro* (Fig. 4d bottom) demonstrating that our *in vitro* model correlates with previously reported partial activity of ELQ271 against bradyzoites in cysts in mouse brain. Vivoporter-PMOs inhibiting cytochrome *bc*_*1*_ had a modest effect on tachyzoite replication and a small effect on size and number of EGS cysts (Fig. 4d top). Minimal effect might be related to limited entry of vivoporter into cysts or mitochondria.

**Figure 5:**
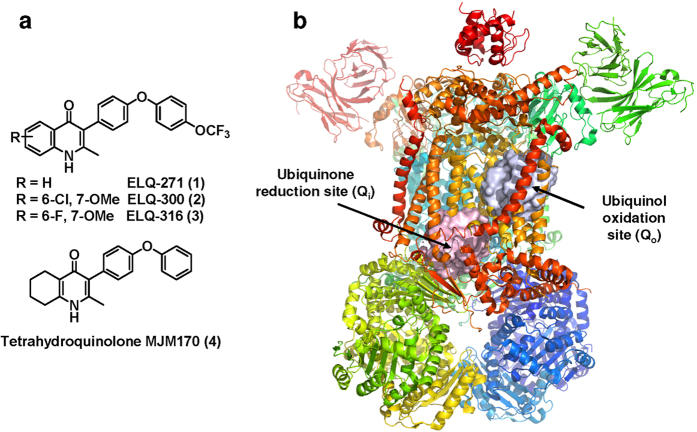
(**a**) Structures of the ELQ class (1–3) and the tetrahydroquinolone scaffold (4)^27^,^45^,^49^,^53^ . Low solubility of the ELQs has been a serious concern going into preclinical evaluation for treatment of malaria^27^. (**b**) *Saccharomyces cerevisiae* cytochrome *bc*_1_ X-ray structure (PDB ID: 1KB9)^5^ The complex contains 11 subunits and 3 respiratory subunits (cytochrome *b*, cytochrome *c1* and Rieske protein). The cytochrome *b* subunit provides both quinone binding sites (Q_o_ and Q_i_) highlighted as grey and pink surfaces respectively.

ELQs have been a focus for drug development for malaria (ELQ 300) and toxoplasmosis (ELQ 271 and 316) as they were reported to be potent and selective (versus human cytochrome *bc*_*1*_) inhibitors of *P. falciparum* cytochrome *bc*_1_ at nanomolar concentrations^27^. ELQs are part of the 4-(1*H*)-quinolone class of cytochrome *bc*_*1*_ inhibitor^36^,^40^,^42^,^45^,^49^,^52^,^53^,^54^,^55^,^56^,^57^,^58^,^59^,^60^,^61^ and (Doggett *et al.*, 13th International Toxoplasmosis Meeting Abstract. Gettysburg Pennsylvania, June 2015), a scaffold that suffers from limited aqueous solubility. Another critical aspect of inhibitor design for this system is minimizing the inhibition of mammalian cytochrome bc1, which shares ~40% sequence identity to the *T. gondii* ortholog within the Q substrate sites. Thus, we set out to design potent and selective inhibitors of *T. gondii* cytochrome *bc*_*1*_ with improved solubility (Fig. 5) compared to known quinolone-based inhibitors. Noting the previous work of GSK on the preclinical development of Clopidol derivatives which led to terminating studies secondary to toxicity in the rat models, as another serious deficiency^62^ and the incorporation of the diphenyl ether group onto the central 4-(1H)-quinolone core as reported by Riscoe *et al.*^27^, we focused on the central core ring system. Doggett, Riscoe *et al.*’s^27^ ELQ 271 (Figs 1 and 5) was reported to be ineffective against yeast with a mutation in the Q_i_ site. Nonetheless, it recently was shown that ELQs can bind both Q_i_ and Q_o_ depending on subtle chemical changes^60^,^61^,^62^. As a result of our initial efforts, a 5,6,7,8-tetrahydroquinolone (MJM170, 2) displayed promising results. (Fig. 5; Table 2). We chose ELQ271 for comparison because it had the greatest activity at the lowest dose (5mg/kg) in the mouse model of Doggett despite the higher cytotoxicity toward human fibroblasts in the *in vitro* toxicity studies^27^.

**Figure 6:**
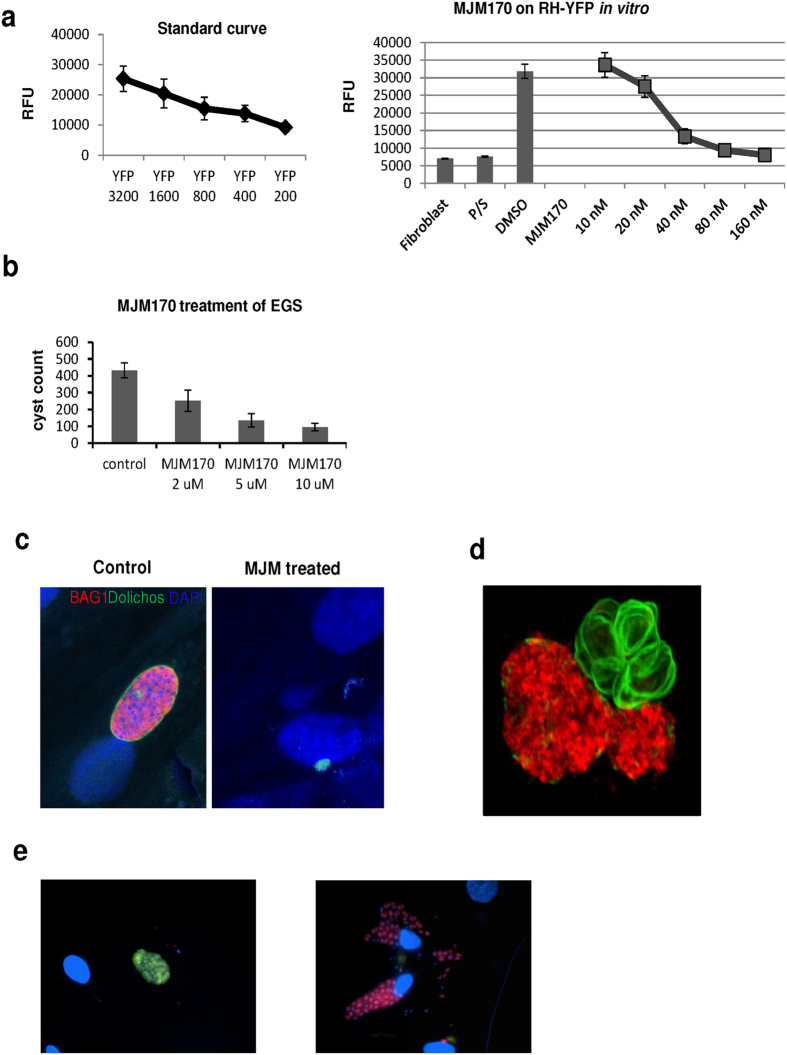
ELQ inhibitors provide a new scaffold and approach yielding compounds that are potent inhibitors of tachyzoites and cysts *in vitro*. (**a–e**) Study of Inhibitors *in vitro* is summarized in Table 2 and led to selection of MJM170 as a promising novel scaffold for both tachyzoites and bradyzoites. (**a**) MJM170 markedly reduces RH YFP tachyzoites in tissue culture robustly at low nanoM levels. (Standard curve left and effect on RH YFP, right panel). (**b**,**c**) MJM170 markedly reduces EGS bradyzoites in cysts *in vitro*. Inhibition of cytochrome *b Qi* eliminates cysts in HFF infected with EGS. Without inhibitory compound in HFF (note, oval cyst with green border staining dolichos) and adjacent panel with inhibitory MJM170 compound (note absence of cysts with small amount of amorphous residual dolichos). MJM170 eliminated tachyzoites followed to 10 days of culture and bradyzoites in cysts *in vitro*. Summary comparison of each of the compounds tested *in vitro* and their ADMET is in Table 2. Note improvement in solubility, properties amenable for compounds to cross blood brain barrier with new scaffold. (**d**) EGS transfected with stage specific reporters for fluors, red tachyzoite SAG1, Green bradyzoite LDH2.

MJM170 is a potent inhibitor of *T. gondii* tachyzoites (RH-YFP strain, IC_50_ 0.03 μM) and bradyzoites (EGS strain, IC_50_ 4 μM), equipotent to ELQ271 (Table 2, Fig. 6).

MJM170 showed 10-fold improved aqueous kinetic solubility (pH 7.4) over ELQ 271 (Table 2), 3-fold improved FaSSIF (pH 6.5) solubility, with similar human microsomal stability profiles (146 vs 172 minutes). A different method from that in ref. 49 was used. MJM170 has a significantly decreased mouse microsomal stability compared to ELQ 271 (20 vs >200 minutes). MJM170 was further evaluated with MDCK-MDR1 cells (a measure of blood-brain barrier permeability) and results suggest that MJM170 could cross the blood brain barrier and not suffer from P-glycoprotein efflux These data highlight the potential of the 5,6,7,8-tetrahydroquinolin-4-one scaffold for further hit-to-lead development.

### MJM 170 is effective *in vivo* against tachyzoites, and modestly against bradyzoites in cysts of mice, and development of a scalable zebrafish assay

**Figure 7:**
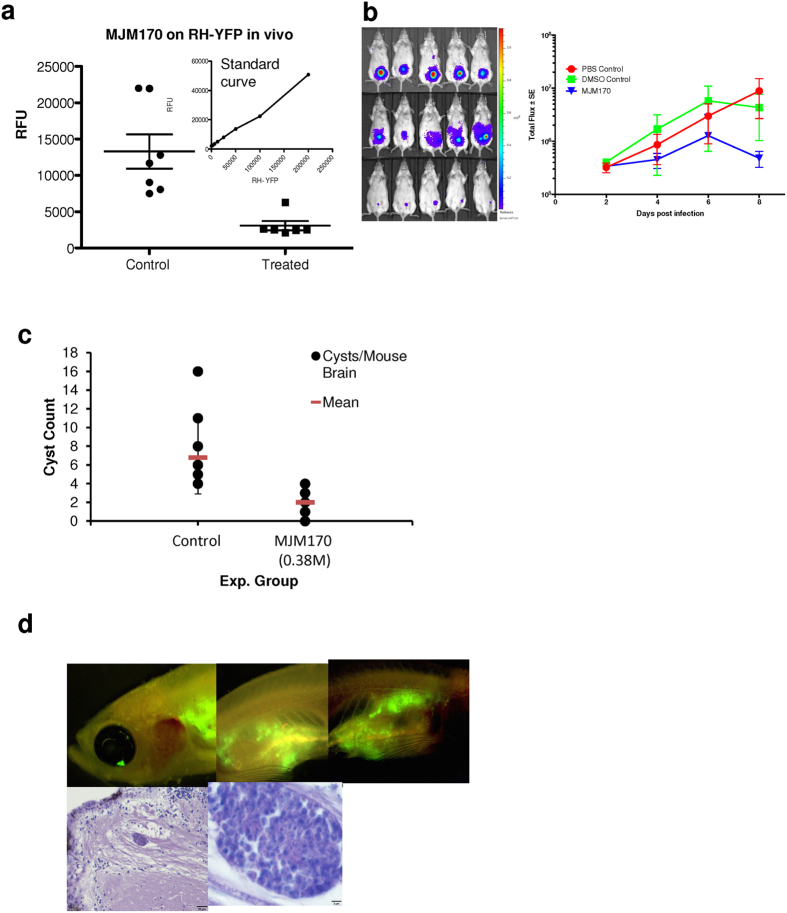
MJM170 is also effective against RH and Prugniaud tachyzoites and Me49 bradyzoites, *in vivo* with translucent zebrafish providing a novel model with potential for scalable *in vivo* assays in which tachyzoites with fluorescent reporters and bradyzoites in cysts can be visualized efficiently. (**a**) 25 mg/kg daily MJM170 administered intraperitoneally eliminates active infection due to RH tachyzoites stably transfected with YFP in mice (RFU control vs rx with MJM 170, p < 0.004) . For the standard curve in the inset, RFU increase with increasing concentrations of fluorescent tachyzoites (R^2^ = 0.99). (**b**) MJM 170 25 mg/kg daily reduces Type 2 parasites. (**c**) MJM 170 reduces cysts in mice infected 2.5 months earlier and treated for 17 days with 12.5 mg/kg daily then without compound for 3 days:cyst count of wet prep of brain homogenate. (**d**) Zebra fish can be used to visualize fluorescent tachyzoites and cysts in more chronic infections.

MJM170 was highly efficacious against RH (Fig. 7a) and Prugniaud (Fig. 7b) strain tachyzoites in mice at 25 mg/kg without toxicity for 5 days (p < 0.00), and modestly reduced numbers of Me49 strain cysts established >2months earlier when treated with 12.5–25 mg mg/kg for 17 days (p < 0.002) (Fig. 7c). In analysis of parallel histopathology, there was a similar trend (data not shown). Translucent zebra fish can be infected with EGS, other *T. gondii* that make cysts, and RH YFP preparing for a novel model for scalable screening (Fig. 7d).

### Cytochrome *bc*_*1*_ Q_i_ is the binding site of MJM170 which is potent against *Plasmodium falciparum* and yeast

#### Tetrahydroquinolone binds to the Q_i_ site of cytochrome *bc*_*1*_

**Figure 8:**
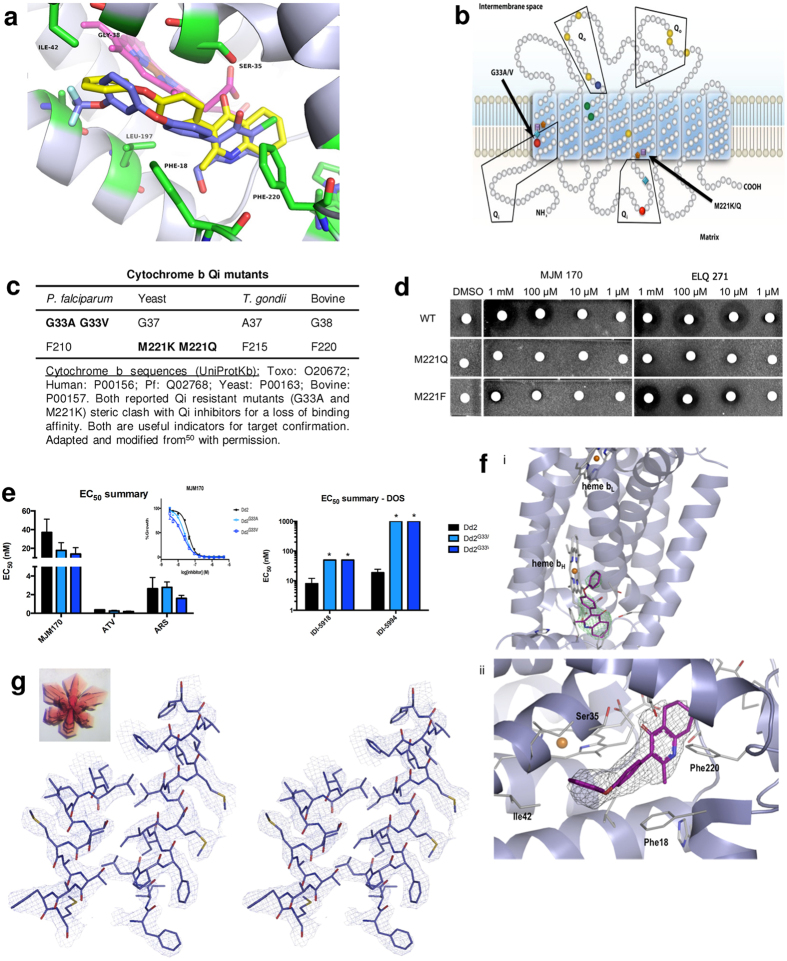
MJM170 targets apicomplexan cytochrome *bc*_*1*_ Qi : modelling, yeast surrogate assays, target validation, co-crystallography and nanoM inhibition of *P. falciparum* and *T. gondii.* (**a**) Modeling: MJM170 (yellow) modelled within cytochrome *b* Q_i_ site (grey) highlighting residues (green) involved in binding. (**b**) Mutations for yeast, *P. falciparum*, predicted for *T. gondii* and bovine enzyme. Relevant mutations are indicated by colored dots in Q_i_ domains on the bottom of the image of mitochondrion membrane for *S. cerevesiae and P. falciparum,* and where those amino acids are in *T. gondii*, human and bovine enzymes. Red dot marks G33A/V in Q_i_ domain of *P. falciparum.* (**c**) Cytochrome b mutants and sequence accession numbers. (**d**) MJM 170 inhibits wild-type but not mutant yeast. Compounds MJM 170 and ELQ 271 with wild type and mutant yeast validate predictions that M221 K/Q would create a steric clash and resistance. (**e**) MJM170 is a potent low nM inhibitor of *Plasmodium falciparum*. In Table 2, wild type *P. falciparum* also are tested and is inhibited at <50 nM by this scaffold. D6 is a drug sensitive strain from Sierra Leone, C235 is a multi-drug resistant strain from Thailand, W2 is a chloroquine resistant strain from Thailand, and C2B has resistance to a variety of drugs including atovaquone. Mutant G33V did not confirm prediction of a steric clash. (**f**) MJM170 binds within Q_i_ site of bovine cytochrome *bc*_1_ as shown by X-ray crystallography. (**f**(i)). An omit Fo-Fc electron density map (green) at 5σ allows unambiguous positioning of MJM170 (magenta) within the Q_i_ site with the tetrahydroquinolone group near heme b_H_ (white) and diphenyl ether directed out of the channel. (**f**(ii)) MJM 170 molecule is included into the structure, the 2Fo-Fc electron density map at 1σ (grey) allows placement of the planar head between heme b_H_ and Phe220 with the carbonyl group positioned in a polar region surrounded by Ser35 and Asp228. (**g**) A stereo picture of the 2Fo–Fc electron density map. Electron density at 1σ level around cytochrome *b* α-helixes 118–128 and 183–199 close to MJM180 compound in X-ray structure of cytochrome*bc1*. Inset: Crystal of mammalian cytochrome bc1 complex.

Studies to determine whether cytochrome *bc*_*1*_ Q_i_ is the molecular target of MJM170 initially included studies of resistance of yeast and *P. falciparum* with known cytochrome *b* Q_i_ mutations predicted to cause a steric clash with MJM170 (Fig. 8). Recently, we reported co-crystal structures of GSK’s cytochrome *bc*_*1*_ inhibitors bound to bovine cytochrome *bc*_*1*_ at the Q_i_ site^52^ demonstrating that these pyridone inhibitors and other structurally related inhibitors bind to an alternative site to atovaquone on cytochrome *bc*_*1*_. This structure allowed us to model MJM170 within the Q_i_ site using the Maestro Suite from Schrödinger. This molecular modelling predicted steric clashes in mutant yeast and *P. falciparum* cytochrome *bc*_*1*_ with MJM170 (Fig. 8a).

#### Co-crystallization of MJM170 with bovine cytochrome *bc*_*1*_ and modelling of the *T. gondii* enzyme confirm the target

Co-crystallization validates predictions made with modelling and confirmed using assays with *S. cerevesiae* mutants (Fig. 8d). There was no steric clash for *P. falciparum*- model based upon this crystal stucture, consistent with *in vitro* assays (Fig. 8e,f). MJM170 was co-crystallized with bovine cytochrome *bc*_1_ (Supplement A: Box with co-crystallography data) and the resulting good quality electron density maps allowed for unambiguous placement of MJM170 within the Q_i_ site (Fig. 8f). The planar region of the quinolone group is held between heme b_H_ and Phe220 and the additional ring further extends into the hydrophobic cavity at the apex of the binding site towards Pro24 and Ile27. The carbonyl group of the compound is surrounded by Ser35, Asp228 and the carbonyl of Trp31, while it’s amine moiety lies between His201 and Ser205. The diphenyl ether group extends outwards towards the hydrophobic residues Ile39 and Ile42 and forms a stacking interaction with Phe18 (Fig. 8f). An example of a stereo electron density involving Ile118-Met129 and Phe183-Phe199 is shown in (Fig. 8g).

#### Surrogate assays demonstrate efficacy of compounds, providing target validation and added value as MJM170 is effective against wild type but not M221Q(F) mutant yeast

Mutants of *S. cerevesiae* were used to further confirm the molecular target of MJM170 (Fig. 8d), and documented that the Q_i_ domain in cytochrome *b* is essential for its efficacy. This approach provided insight into binding of compounds to the enzyme. Crystallographic structure of bovine cytochrome *bc*_*1*_ with GSK932121^52^ indicates that certain amino acids are critical in tetrahydroquinolone binding and explains why there is inhibition by certain compounds. Previous studies reported that no cross-resistance is observed between ELQs and atovaquone in *P. falciparum*. This is rationalised as atovaquone binds the Q_o_ site on cytochrome *bc*_*1*_. A yeast M221Q substitution within the Q_i_ site displayed resistance to ELQ inhibition further confirming that this to be the target site^27^. MJM170 and ELQ271 were effective against *S. cerevisiae* wild type parental AD1-9 strain at 1mM, 100, 5 and 1 μM when grown on non-fermentable, glycerol medium forcing reliance on ATP production for respiration. Yeast strains with point mutations in the cytochrome *b* gene that substitute methionine by glutamine (M221Q) or phenylalanine (M221F) at position 221 in the Q_i_ site predicted to yield a steric clash upon inhibitor binding were resistant to MJM170 (Fig. 8d).

#### Tetrahydroquinolones are potent against wild type *P. falciparum*, and *P. falciparum* G33A/V and other drug resistant mutants but not DHODH mutant

**Figure 9:**
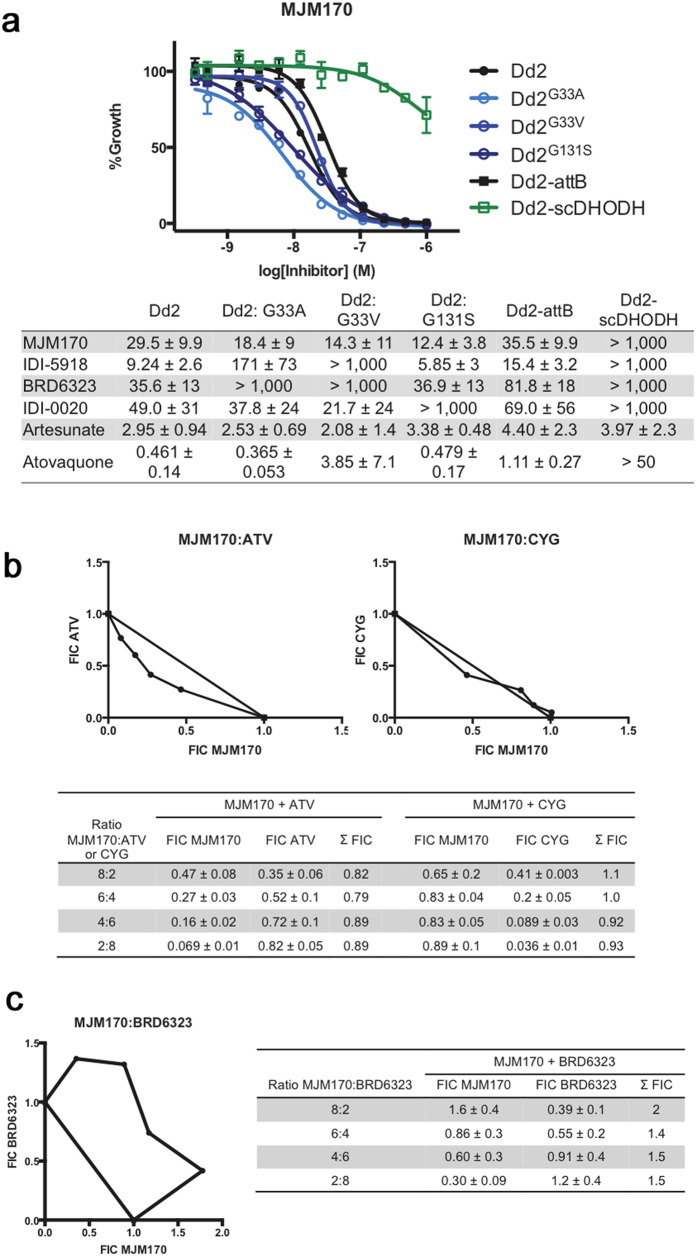
MJM170 potently inhibits *P. falciparum* mitochondrial electron transport important for synthesis of pyrimidines, is modestly synergistic with atovaquone, additive with cycloguanil and antagonistic with Q_i_ inhibitor. (**a**) MJM170 is highly potent (Dd2, black curve, EC_50_ = 29.5nM) without cross-resistance in previously reported cytochrome *b* drug-resistant mutant parasite lines including ubiquinone reduction site mutants^S76,S77^ (Dd2^G33A^ and Dd2^G33V^, light blue and dark blue curves, respectively). Dose-response curve from representative assay. MJM170 cannot inhibit a parasite supplemented with a yeast cytosolic DHODH (scDHODH, green curve) demonstrating that its primary activity in *P. falciparum* is to inhibit electron transport necessary for pyrimidine biosynthesis. Inset Table. Dose-response phenotypes of a panel of *P. falciparum* cytochrome *b* mutant parasite lines. EC_50_ values were calculated using whole-cell SYBR Green^S78^ assay and listed as mean ± standard deviation of three biological replicates, each with triplicate measurements. (**b**,**c**) Isobolograms with MJM170 plus atovaquone or cycloguanil or Qi inhibitor BRD6323^S76^: (**b**) Combinations were with atovaquone (ATV) or cycloguanil (CYG) at multiple fixed volumetric ratios (10:0, 8:2, 6:4, 4:6, 2:8, and 0:10) in Dd2 parasites. Slight synergy observed with combinations of MJM170 and atovaquone while MJM170 and cycloguanil dosed in combination showed additive effect. Fractional inhibitory concentrations (FIC) for each drug were calculated and plotted. Shown is a representative isobologram for each combination of compounds. Table below lists FICs for each compound and ratio tested (values are mean from three independent assays ± standard deviation). Synergy was defined as a combined FIC < 1.0, addivity as FIC = 1.0, and antagonism as FIC > 1.0^S79^. (**c**) Isobologram Figure: MJM170 was tested in combination with previously reported reduction site inhibitor BRD6323^S76^ at multiple fixed volumetric ratios (10:0, 8:2, 6:4, 4:6, 2:8, and 0:10) in Dd2 parasites. Antagonism was observed with combinations of MJM170 and BRD6323 as opposed to synergy observed with oxidation site inhibitor atovaquone. Fractional inhibitory concentrations (FIC) for each drug were calculated and plotted. Representative isobologram of three independent assays is shown. Table below lists FICs for each compound and ratio tested (values are means from three independent assays ± standard deviation). Definitions as in (**b**).

MJM170 is highly effective against *P. falciparum* (Table 2) including multiple strains resistant to available antimicrobials and a cytochrome *bc*_*1*_ Q_i_ mutant (Fig. 9). Resistance against transgenic *P. falciparum* yeast DHODH mutant strain indicates MJM170 affects mitochondria suggesting that the mode of action against *P. falciparum* is through inhibition of electron transport (Fig. 9a).

### Potentially clinically useful combinations with tetrahydroquinolone demonstrated in synergy studies

To determine whether there might be clinically relevant synergies and additive effects, combinations of MJM170 with other clinically available and useful compounds also were tested. Earlier, we had found cycloguanil and related biguanide triazines^63^ were active against *T. gondii* tachyzoites and *P. falciparum* making it relevant to test them in combination with MJM170. We observed modest synergy *in vitro* for atovaquone, additive effect with cycloguanil, and antagonism with BRD6323, a Q_i_ inhibitor for *P. falciparum* (Fig. 9b). Combining atovaquone with proguanil (active component cycloguanil) as Malarone^R^ for malaria provides an approach to reduce selection of drug resistant *plasmodium* mutants.

## Discussion

The results presented here offer a molecular understanding and therapeutic strategies for one of the most common parasitic infections of human brain and eye, and that persists across lifetimes in around 2 billion people worldwide. We have developed new models to facilitate discovery of curative treatments for toxoplasmosis. We have characterized the Brazilian *T. gondii* isolate called EGS that was known to be morphologically similar to encysted bradyzoites in tissue culture. We further validate the cystic nature of these EGS infected cultures, since they are able to induce the intra-intestinal life cycle when fed to cats ultimately resulting in oocyst secretion. This is the first such description of this phenotype and provides definitive proof that this unique parasite has a true cyst phenotype when maintained *in vitro*. Our data also provide a number of other major conceptual advances on EGS by demonstrating the following: (i) Genome sequencing of this EGS isolate demonstrates that EGS has a typical Brazilian virulent genotype and phylogeny, (ii) EGS is a haplogroup 4 *T. gondii.* Consistent with a genotype that is known to be pathogenic and virulent for mice, we demonstrate that EGS oocyst induced infection is similar to that of other virulent Brazilian parasites. For example, mice fed EGS oocysts demonstrated ileal parasites causing necrosis, as well as pneumonitis, encephalitis and systemic infection leading to death. This indicates that the ability to form cysts in culture does not alter the pathogenicity of EGS in mice. However, potentially relevant to its *in vitro* bradyzoite phenotype, full genome sequencing revealed that it has nonsynonymous single nucleotide repeat sequence differences from other Brazilian and canonical U.S. and European parasites which do not share its *in vitro* bradyzoite phenotype. EGS has a non-synonymous mutation in a bradyzoite repressor, Apetela 2(AP2) IV-iv, plant like transcription factor. AP2s interact with HATs and HDACs to modulate transcriptional signatures in apicomplexan parasites^64^. This Apetela 2 plant like transcription factor gene is known to drive tachyzoite switch by repressing bradyzoite genes. If the observed substitution results in a defective or non-functional molecule, this could provide an explanation for the observed bradyzoite phenotype of EGS parasites. This is consistent with our findings that EGS in HFF forms cysts by 24 hours, characterized by BAG1, and Dolichos staining at 24, 48, and 96 hours after infection. These cysts gradually enlarge until 48–96 hrs in culture, when single *T. gondii* begin to destroy HFF monolayers. These are the cultures of bradyzoites in cysts that when fed to cats ~48 hours after infection form oocysts which are virulent in mice, providing definitive proof of an *in vitro* bradyzoite phenotype for the EGS strain of *T. gondii.*

The transcriptomic studies with this EGS isolate have provided critical insights into host cell mechanisms that are a prominent part of the ability of the encysted parasite to persist in this untreatable life cycle stage, and biologic consequences of such persistent infection. RNAseq and miR seq of EGS infected human host cells included human fibroblasts, monocytic and neuronal stem cells with this encysted EGS strain parasite. These provide an understanding of the types of perturbations of biologically relevant host cells this bradyzoite life cycle stage can cause, providing insights into unique aspects of pathogenesis of this infection with untreatable cysts and its consequences. We found that EGS modifies critical host cell pathways. For example we find *in vitro* modulations of host cell pathways in human, primary neuronal stem cells are the same as those associated with modulation of host cell replication as seen with malignancies, and in neurodegenerative diseases. Further, it is noteworthy that the level of a microRNA that specifies apoptosis in eye and brain cells is markedly down modulated by this EGS bradyzoite which would inhibit host protective apoptotic mechanisms allowing parasites to persist in brain and eye without a critical protective mechanism. EGS, as an encysted bradyzoite, clearly alters biologic processes including cell cycle, cell death, alternative splicing, protein synthesis, protein folding and ubiquitination and down regulates hsa-miR-708-5p that specifies apoptosis in neuronal and retinal cells^65^.

RNA and MiR sequencing and transcriptomic analyses of the EGS parasites also identified molecular targets that are critical for the bradyzoite life cycle stage in the parasite as well. These molecular targets include cytochrome *b,* as critically increased in dormant, encysted parasites. Cytochrome *b* was increased along with known cyst constituents like enolase 1, Cyst wall protein, Lactate dehydrogenase 2, bradyzoite antigen 1, Apetela 2 plant like transcription factors not present in animals, such as AP2 IX-ix, and cytochrome oxidase. Our work provides a new means to identify stage specific molecular targets, and emphasizes that cytochrome *bc 1* complex is a critical target. The transcriptome of EGS parasites in HFF over time are similar to those of *in vivo* bradyzoites in terms of known critical genes modified. Finally, EGS presents a much-needed assay for identifying novel molecular targets present in bradyzoites *in vitro*. EGS was also useful to evaluate the effect of inhibitors on encysted bradyzoites *in vitro.*

Recent crystallographic studies with the bovine cytochrome *bc*_1_ complex allowed us to rationally design a novel compound to target the Q_i_ site of cytochrome *b*. Our novel compound was designed to address issues with poor solubility of existing quinolone/pyridone Q_i_ inhibitors. One of these compounds MJM170, a substituted 5,6,7,8-tetrahydroquinolin-4-one inhibits active infection (IC_50_ 30 nM) and cysts (IC_50_ 4 μM) *in vitro,* and *in vivo* (25 mg/kg). It is predicted to cross the blood brain barrier with no efflux as demonstrated in an *in vitro* MDR1-MDCK permeability assay (Table 2), indicating this class of compounds have promise for treatment of central nervous system infections. When we tested MJM170 against wild type and multi-drug resistant *P. falciparum*, we found it was also potent (IC_50_ <30 nM against all strains). In combination studies, MJM170 was identified as additive with cycloguanil and modestly synergistic with atovoquone. Studies of yeast and malaria mutants, as surrogate assays, and co-crystallography studies with bovine cytochrome *bc*_1_ confirm the mechanism of action/target for MJM170. The co-crystal structure of MJM170 in complex with bovine cytochrome *bc*_1_ reveals a clear binding mode within the Q_i_ site. Using homology models of the apicomplexan Qi sites, there are clear differences between the binding sites of the apicomplexan and mammalian orthologs which can be used to fine-tune the selectivity of our scaffold towards apicomplexan *bc*_1_. This is important in moving this work forward since other Q_i_ inhibitor programs have reported toxicity due to potential inhibition of mammalian *bc*_1_^62^. The larger binding pocket of the apicomplexan versus the mammalian *bc*_*1*_ may provide a way forward to increase selectivity. Our work provides a conceptual and a practical step change forward that provides a foundation for further testing and improvements to efficacy, toxicity, solubility, oral absorption, large animal toxicology that will be needed to reach the clinic. Protecting the diphenyl ether side chain of MJM170 would make it less vulnerable to metabolic attack^49^. Our work reported herein not only provides new and important insights into the biology of *T. gondii,* especially the bradyzoite life cycle stage and the remarkable effects of this parasite on its human host’s cells, but also provides critical molecular targets and new methods to identify others. Armed with this information, a novel scaffold with intrinsically higher solubility than the equivalent quinolone has been designed with holds promise towards developing a much-needed curative medicine for those with toxoplasmosis, malaria, and ~2 billion persons chronically infected with presently incurable, encysted bradyzoites which persist and can recrudesce lifelong.

## Methods

All methods were carried out in accordance with approved guidelines set at the University of Leeds by the Education & Training Resources office and all experimental protocols were approved by the IRB committees; University of Chicago Institutional Animal Care and Use Committee (IACUC) and all experimental protocols were approved by the IRB committee; United States Department of Agriculture IACUC and all experimental protocols were approved by the IRB committees; J Craig Venter Institute Research ethics committee; University of Liverpool UK Office for Research Integrity (UKRIO) and all experimental protocols were approved by the IRB committees; Harvard School of Public Health HMS IACUC and all experimental protocols were approved by the IRB committees; The Broad Institute IACUC and all experimental protocols were approved by the IRB committees; Walter Reed Army Institute of Research Division of Human Subjects Protection (DHSP) and all experimental protocols were approved by the IRB committees; Oregon State University IACUC and all experimental protocols were approved by the IRB committees; Institute for Systems Biology ethics committee; Albert Einstein College of Medicine IACUC and all experimental protocols were approved by the IRB committees; Strathclyde University Ethics Committee (UEC) and all experimental protocols were approved by the IRB committees; Institute for Integrative Biology of the Cell IACUC and all experimental protocols were approved by the IRB committees, and the Centre national de la recherche scientifique IACUC and all experimental protocols were approved by the IRB committees.

### Cells and Parasites for work with T. gondii

#### Cells

The cells utilized for *T. gondii* assays included human foreskin fibroblasts (HFF)^S1–21^, Human MonoMac 6 cells (MM6)^S22,S23^, and Neuronal Stem cells (NSC) from a temporal lobe biopsy^S24^.

#### Toxoplasma gondii

The strains of *T. gondii* utilized in this work were: RH-YFP Tachyzoites of the RH-YFP strain were passaged in human foreskin fibroblasts (HFF cells)^S1–21,S25^; EGS-Bradyzoite assays use the EGS strain^S26,S27^, isolated from amniotic fluid of human with congenital toxoplasmosis^S1,S26,S27^; Other strains used are: Me49^S28^; Prugniaud^S14,S15^; Beverly^S29^; Veg; GT1. All other than EGS are *T. gondii* tachyzoites. These parasites are passaged in HFF^S1–21^.

#### Isolation of DNA and RNA

EGS single celled organisms were grown in Human Foreskin Fibroblasts, filtered free of host cells. gDNA^S30^ was isolated and processed for sequencing as described. For isolation of RNA RIN scores were >8.

### Gene sequencing, genomics, RNA and MiR sequencing, Systems analysis, Metabolomics

#### Genome sequencing of *T. gondii* EGS strain

A single Illumina paired-end barcoded library was prepared from tachyzoite gDNA^S30^ with Illumina TrueSeq library preparation kit. The library was then sequenced using 100 bp pared-end reads in one ninth of a lane of an Illumina HiSeq 2000 machine to generate ~2 Gbp of genome sequence.

#### Single nucleotide polymorphism (SNP) identification and annotation

Illumina genome sequencing reads from EGS or downloaded from GenBank SRA database for GT1 (SRR516419), VEG (SRR516406) and TgCatBr1 (SRR350737) were aligned to the *T. gondii* ME49 reference genome assembly (ABPA02000000, ToxoDB^S31^ release 13.0) with Bowtie2^S32^ and realigned around gaps using the GATK toolkit^S33^. SNP calls were done simultaneously across all four strains with samtools utility mpileup^S34^, requiring a minimum SNP coverage of 5 reads and an alternative allele frequency of 0.8 or higher, given the haploid nature of these genomes. Thereafter, SnpEff^S35^ and a gff3 file containing the annotation of *T. gondii* ME49 downloaded from ToxoDB v13.0 were used to classify the different types of mutations identified in each strain. Allelic variants that were different between EGS and the rest of the strain were considered EGS-specific.

#### Phylogenetic network analysis

A total of 790,168 single nucleotide polymorphisms spanning the entire *T. gondii* genome from 62 different strains representing all major haplogroups were downloaded from ToxoDB, combined with SNP data from the same sites from the EGS strain and directly incorporated as a FASTA file into SplitsTree^S36^ v4.13.1 to generate unrooted phylogenetic networks using a neighbor-net method.

#### Differential gene expression (DGE) analysis

Total RNA extracted from human cell cultures infected (or not) with a number of *T. gondii* strains for 2 h, 18 h or 48 h was treated with miRNeasy Mini Kit columns (Qiagen) following manufacturer instructions to separate mRNA and miRNA fractions. Afterwards, Illumina barcoded sequencing libraries were constructed with TruSeq RNA Sample Preparation Kits v2 (Illumina) for mRNA and miRNA TruSeq Small RNA Library Preparation Kit (Illumina) for miRNA. Libraries were sequenced as 100 bp single reads with Illumina HiSeq 2000 apparatus in pulls of 6 or 9 samples per lane for mRNA (yield ~3 Gbp per sample) and miRNA (yield ~2 Gbp per sample) libraries respectively. For protein coding genes, reads were mapped to the human (release GRCh38) and *T. gondii* ME49 strain (ToxoDB release 13.0) reference genome assemblies and annotations with CLC Genomic Workbench software (CLC Bio-Qiagen, Aarhus, Denmark) and raw read counts per gene were then analyzed with the R package EdgeR^S37^ using a generalized linear model likelihood ratio test to identify genes that are differentially expressed among samples.

For miRNA DGE analysis, reads were depleted of adaptor and primer sequences and mapped to the human reference genome assembly (GRCh38) and the miRNA annotation from miRBase^S38^ v21 (www.mirbase.org) with CLC Genomic Workbench software. Identification of human miRNA genes that are differentially expressed across treatments was carried out with EdgeR from raw read counts per miRNA gene using a generalized linear model likelihood ratio test.

For both mRNA and miRNA DGE analyses p-values were adjusted for multiple hypotheses testing using the False Discovery Rate method. MDS plots and heat maps were generated with the plotMDS tool from EdgeR and the R tool heatmap. Differentially expressed genes (DEGs) in MM6 and NSC cell lines infected with EGS parasites were identified under the criteria of 1% FDR and absolute log2-fold-change >1.5 (i.e. fold-change >2 and <0.5 for up- and down-regulated genes, respectively).

#### Functional enrichment analysis

GO enrichment analyses were performed for up- or down-regulated genes, by using the Database for Annotation, Visualization and Integrated Discovery (DAVID) v6.7. GO slim enrichment analysis^S38a^ was performed for genes carrying potential change-of-function mutations in EGS that were absent in strains ME49, VAND or TgCatBr1. GO slim database was downloaded from QuickGO provided by EMBL-EBI. Using taxonomy id “508771” for the ME49 strain, relevant GO slim terms were retrieved. GO slim enrichment analysis was performed with Fisher’s exact test based on the GO slim terms.

### Assay for oocyst development in cats

Oocysts were collected from feces of Toxoplasma-free cats 3–14 days after feeding infected cell cultures or infected mouse brains. Oocysts were separated from feces by sugar floatation, sporulated in 2% sulfuric acid by aeration at room temperature for 1 week. After removing sulfuric acid oocysts were inoculated orally in to Swiss Webster albino mice. All tissues of mice that died or euthanized were studied histologically after staining with hematoxylin and eosin and by BAG1 antibodies to *T. gondii* as described^S39^.

## Chemical Synthesis

**Figure 10:**
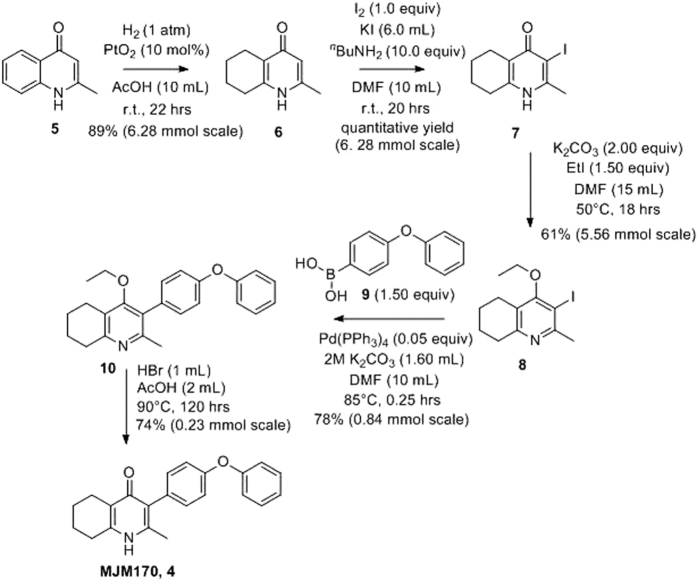
Synthesis of tetrahydroquinolones. **Synthesis of 2-methyl-5,6,7,8-tetrahydroquinolin-4-one (6)** Platinum oxide (100 mg, 10 mol%) was added to a solution of 4-hydroxy-2-methylquinoline (**5**, 1.00 g, 6.28 mmol, 1.00 eq) in glacial acetic acid (10.0 ml). The heterogeneous mixture was catalytically hydrogenated under a balloon of hydrogen. After 22 hrs, TLC (10% MeOH–DCM) confirmed complete reaction. The mixture was filtered through celite under vacuum, washing thoroughly with EtOAc. The filtrate was concentrated and the resulting residue purified by column chromatography (10% MeOH–DCM) to give the desired product as a pale yellow oil (917 mg, 5.65 mmol, 89%); ***R***_**f**_ 0.14 (10% MeOH–DCM); **δ**_**H**_ **(300 MHz, CDCl**_**3**_) 1.74–1.76 (4H, m, CH_2_), 2.29 (3H, s, Me), 2.49–2.52 (2H, m, CH_2_), 2.67–2.70 (2H, m, CH_2_), 6.16 (1H, s, Ar-H); **δ**_**C**_ **(125 MHz, CDCl**_**3**_) 19.0 (Me), 21.8 (CH_2_), 22.1 (CH_2_), 27.1 (CH_2_), 112.5 (CH), 122.4 (_**C**_q), 146.4 (Cq), 147.0 (Cq), 178.3 (Cq); Spectroscopic data consistent with literature values (JMC, 1993, 36, 1245–54). **Synthesis of 2-methyl-3-iodo-5,6,7,8-tetrahydroquinolin-4-one (7)** Butylamine (6.20 ml, 62.8 mmol, 10.0 eq) was added to a suspension of 2-methyl-5,6,7,8-tetrahydroquinolin-4-one (**6**, 1.02 g, 6.28 mmol, 1.00 eq) in DMF (10.0 ml). To this heterogeneous mixture was added I_2_ (1.60 g, 6.28 mmol, 1.00 eq) in a saturated solution of KI (6.00 ml). After 20 hrs stirring at R.T., a precipitate formed in the orange solution, Excess iodine was quenched with 0.1 M sodium thiosulfate solution. The precipitate was filtered by vacuum filtration, washed with distilled H_2_O and dried (Na_2_SO_4_) to give the desired product as a colourless solid (1.76 g, 6.09 mmol, quantative yield); **δ**_**H**_ **(300 MHz, DMSO-*****d***_***6***_) 1.61–1.70 (4H, m, CH_2_), 2.29 (2H, t, *J* 6.0, CH_2_), 2.43 (2H, s, C_**H**2_), CH_3_ under DMSO peak. **Synthesis of 2-methyl-3-iodo-4-ethoxy-5,6,7,8-tetrahydroquinoline (8)** Potassium carbonate (1.53 g, 11.1 mmol, 2.00 eq) was added to a heterogeneous mixture of 2-methyl-3-iodo-5,6,7,8-tetrahydroquinolin-4-one (**7**, 1.60 g, 5.56 mmol, 1.00 eq) in DMF (15.0 ml), and the reaction heated to 50 °C for 30 mins. The R.B. flask was removed from the heating mantle and ethyl iodide was added dropwise. The reaction was then heated at 50 °C for 18 hrs. The reaction was cooled to R.T., quenched with water (40 ml). The resulting emulsion formed which was extracted with EtOAc (50 ml). EtOAc layer were washed with water (3 × 30 ml), brine (3 × 30 ml), dried (Na_2_SO_4_) and concentrated to give a pale yellow oil (1.09 g, 3.44 mmol, 61%); ***R***_**f**_ 0.88 (1:1 Pet–EtOAc); **HPLC** (RT = 1.67 mins); **LCMS** (Method A), (RT = 1.6 min, *m/z* (ES) Found MH^+^ 318.0); **δ**_**H**_ **(500 MHz, CDCl**_**3**_) 1.49 (3H, t, *J* 7.0, ethoxy CH_3_), 1.73–1.78 (2H, m, CH_2_) 1.84–1.88 (2_**H**_, m, CH_2_), 2.78**–**2.69 (5H, m, CH_2_ & CH_3_), 2.84 (2H, t, *J* 6.5, CH_2_), 3.97 (2H, q, *J* 7.0, OCH_2_); **δ**_**C**_ **(125 MHz, CDCl**_**3**_) 15.6 (CH_3_), 22.3 (CH_2_), 22.8 (CH_2_), _2_3.6 (CH_2_), 29.3 (CH_3_), 3_2_.0 (_**C**_H_2_), 68.4 (OCH_2_), 90.9 (Cq), 124.5 (Cq), 158.3 (Cq), 158.9 (Cq), 163.9 (Cq). **Synthesis of 2-methyl-3-(4-phenoxyphenyl)-4-ethoxy-5,6,7,8-tetrahydroquinoline (10)** 2-Methyl-3-iodo-4-ethoxy-5,6,7,8-tetrahydroquinoline (**8**, 0.266 g, 0.839 mmol, 1.00 eq), Pd(PPh_3_)_4_ (0.048 mg, 0.0419 mmol, 5 mol%) and 4-phenoxyphenylboronic acid (**9**, 0.270 mg, 1.26 mmol, 1.50 eq) were charged to a R.B. flask under N_2_(g)^49^. Degassed DMF (10.0 ml) was added to the flask followed by 2M K_2_CO_3_ (1.60 ml). The flask was heated to 85 °C under N_2_(g). After 15 mins, TLC (4:1 Pet–EtOAc) confirmed reaction was complete. The reaction was cooled and diluted with EtOAc (15 ml), filtered through celite and partitioned between EtOAc (10 ml) and H_2_O (25 ml). Combined organics were washed with H_2_O (3 × 30 ml), then brine (3 × 30 ml), dried (Na_2_SO_4_) and concentrated to give a red oil which was purified by column chromatography (3:1 Pet–EtOAc), to give the desired product as a pale yellow oil (0.235 mg, 0.655 mmol, 78%); ***R***_**f**_ 0.31 (3:1 Pet–EtOAc); **HPLC** (RT = 3.08 mins); **δ**_**H**_ **(300 MHz, CDCl**_**3**_) 1.04 (3H, t, *J* 7.0, ethoxy CH_3_), 1.76–1.93 (4H, m, 2xCH_2_), 2.32 (3_**H**_, s, CH_3_) 2.72 (2H, t, *J* 6.0, CH_2_), 2.91 (2H, t, *J* 6.5, CH_2_), 3.50 (2H, q, *J* 7.0, OCH_2_), 7.05–7.16 (5H, m, Ar-H), 7.20–7.29 (2H, m, Ar-H), 7.31–7.43 (2H, m, Ar-H); **δ**_**C**_ **(125 MHz, CDCl**_**3**_) 15.7 (CH_3_), 22.5 (CH_2_), 23.0 (CH_3_), 23.3 (CH_2_), 23.4 (CH_2_), 32.7 (_**C**_H_2_), 68.2 (OCH_2_), 118.6 (CH), 118.9 (CH), 123.4 (CH), 126.8 (Cq), 129.8 (CH), 131.5 (CH), 154.9 (Cq), 156.5 (Cq), 157.1 (Cq), 157.3 (Cq); ***m/z*** (***ES***) (Found: MH^+^, 360.1973. C_24_H_26_NO_2_ requires *MH*, 360.1964). **Synthesis of 2-methyl-3-(4-phenoxyphenyl**)**-4-ethoxy-5,6,7,8-tetrahydroquinoline (MJM170, 4)**^49^ Aqueous hydrobromic acid (>48%) (1.00 ml) was added to a solution of 2-methyl-3-(4-phenoxyphenyl)-4-ethoxy-5,6,7,8-tetrahydroquinoline (**10**, 0.226 mg, 0.630 mmol, 1.00 eq) in glacial acetic acid (2 ml). The reaction was stirred at 90 °C for 5 days, monitoring by LMCS. The reaction was cooled to R.T. and the pH adjusted to pH5 with 2M NaOH. The precipitate was collected by vacuum filtration and recrystallized from MeOH:H_2_O to give the desired product as an off-white solid (0.155 g, 0.467 mmol, 74%); **HPLC** (RT = 2.56 mins); **δ**_**H**_ **(500 MHz, DMSO-*****d***_***6***_) 1.66–1.72 (4H, m, 2xCH_2_), 2.08 (3H, s, CH_3_) 2.31 (2H, t, *J* 6.0, CH_2_), 2.56 (2_**H**_, t, *J* 6.0, CH_2_), 6.99 (2H, d, *J* 8.5, Ar-H), 7.06 (2H, d, *J* 7.5, Ar-H), 7.14–7.18 (3H, m, Ar-H), 7.40–7.43 (2H, m, Ar-H), 11.0 (1H, s, NH); **δ**_**C**_ **(125 MHz, DMSO-*****d***_***6***_) 17.7 (CH_3_), 21.5 (CH_2_), 21.8 (CH_2_), 21.9 (CH_2_), 26.2 (CH_2_), 117.8 (CH), 118.6 (CH), 121.2 (Cq), 123.3 (CH), 123.7 (Cq), 130.0 (CH), 131.4 (Cq), 132.3 (CH), 142.3 (Cq), 143.2 (Cq), 155.0 (Cq), 156.8 (Cq), 175.4 (Cq); ***m/z*** (***ES***) (Found: MH^+^, 332.1654. C_22_H_22_NO_2_ requires *MH*, 332.1645).

Final compounds had >95% purity determined by high performance liquid chromatography (HPLC) and 300 and/or 500 MHz NMR spectrometers. Liquid chromatography-mass spectrometry (LC-MS) and high resolution mass spectrometers (HRMS) analytical systems were used to determine integrity and purity of all intermediates and final compounds. Chemical synthesis is shown in Fig. 10.

### ADME studies of inhibitors

Compounds that were highly effective *in vitro* (IC_50_ < 1 μM) were tested for ADME profiling^S43–58^ by Shanghai ChemPartner Ltd. Initial studies focused on aqueous kinetic solubility pH 7.4, microsomal metabolic stability (human and mouse) and Blood-Brain Barrier (BBB) permeability^S43–58^ (performed with MDCK-MDR1 cells as described^S52^).

## *In Vitro* assays

### Cytotoxicity Assay

#### Toxicity Analysis

Lack of toxicity for mammalian host cells was demonstrated first by visual inspection of monolayers following giemsa staining, in separate methods by incorporation of a mitochondrial cell death reagent called WST we used successfully for this purpose and in separate experiments^S12–15;S18,19^.

Toxicity assays were conducted using WST-1 cell proliferation reagent (Roche) as described^S12–15;S18,19^. HFF were grown on a flat, clear-bottomed, black 96-well plate. Confluent HFF were treated with inhibitory compounds at concentrations equal to those being tested in challenge assays. Compounds were diluted in IMDM-C, and 20 μl were added to each designated well, with triplicates for each condition. A gradient of 2 fold-decreasing concentrations of DMSO in clear IMDM-C was used as a control. The plate was incubated for 72 hours at 37 **°**C. 10 μl of WST-1 reagent (Roche) were added to each well and the cells were incubated for 30 to 60 minutes. Absorbance was read using a fluorometer at 420 nm. A higher degree of color change (and absorbance) indicated mitochondrial activity and cell viability.

## *In Vitro* Cellular Assays for Effects on *T. gondii*

### Vivo PMO

Vivo-PMO (Vivo porter linked to morpholinos) to knock down cytochrome *b* and an off-target PPMO (Vivo porter) were utilized at concentrations of 5 and 10 μM as previously described with both cultures of RH-YFP tachyzoites and EGS^S14^. Morpholino sequence for cytochrome b/c knockdown is 5′ AGTGTTCTCGAAACCATGCTAACAC 3′, and for unrelated sequence, off target, is 5′ CCTCTTACCTCAG-TTACAATTTATA 3′.

#### Tetrahydroquinolone Compounds

Compounds synthesized at the University of Leeds were initially prepared in 10 mM Stock solutions made with 100% Dimethyl Sulfoxide (DMSO) [Sigma Aldrich], and working concentrations were made with IMDM-C (1x, [+] glutamine, [+] 25 mM HEPES, [−] Phenol red, 10% FBS) [Gibco, Denmark]).

### Tachyzoite assays

#### Type 1 parasites

Human foreskin fibroblasts (HFF) were cultured on a flat, clear-bottomed, black 96-well plate to 90% to 100% confluence. IMDM (1x, [+] glutamine, [+] 25 mM HEPES, [+] Phenol red, 10% FBS [gibco, Denmark]) was removed from each well and replaced with IMDM-C(1x, [+] glutamine, [+] 25 mM HEPES, [−] Phenol red, 10% FBS)[Gibco, Denmark]). Type I RH parasites expressing Yellow Fluorescent Protein (RH-YFP) were lysed from host cells by double passage through a 27-gauge needle. Parasites were counted and diluted to 32,000/mL in IMDM-C. Fibroblast cultures were infected with 3200 tachyzoites of the Type I RH-YFP strain and returned to incubator at 37 °C for 1–2 hours to allow for infection ^S12–15;S18,19;S25^. Diluted solutions of the compounds were made using IMDM-C, and 20 μl were added to each designated well, with triplicates for each condition. Controls included pyrimethamine/sulfadiazine (current standard of treatment), DMSO only, fibroblast only, and an untreated YFP gradient with 2 fold dilutions of the parasite. Cells were incubated at 37 °C for 72 hours. The plates were read using a fluorimeter (Synergy H4 Hybrid Reader, BioTek) To ascertain the amount of yellow fluorescent protein, in relative fluorescence units (RFU), as a measure of parasite burden after treatment. Compounds were not considered effective or pursued for further analysis if there were no signs of inhibition at 1 μM. Data was collected using Gen5 software and analyzed with Excel.

#### Type II parasites

To test type II parasites, *T. gondii* ME49 and Prugniaud parasites expressing luciferase or GFP. We tested them *in vitro* and *in vivo* as we have described.

#### EGS strain Bradyzoite Assay

HFF cells were grown in IMDM (1x, [+] glutamine, [+] 25 mM HEPES, [+] Phenol red, 10% FBS, [Gibco, Denmark]) on removable, sterile glass disks in the bottom of a clear, flat-bottomed 24-well plate. Cultures were infected with 3 × 10^4^ parasites (EGS strain) per well, in 0.5 mL media and plate was returned to incubator at 37 °C overnight. The following day, the media was removed and clear IMDM and compounds were added to making various concentrations of the drug, to a total volume of 0.5 mL. Two wells were filled with media only, as a control. Plates were returned to the 37 °C incubator for 72 hours.

Efficacy was determined following fixation. Staining was used to determine the numbers of cysts in cultures without and with treatment with the test compounds. Cells were fixed using 4% paraformaldehyde and stained with Fluorescein-labeled Dolichos Biflorus Agglutinin, DAPI, and anti-BAG1, and anti-SAG1. Disks were removed and mounted onto glass slides and visualized using microscopy (Nikon Tl7). Slides were also scanned using a CRi Pannoramic Scan Whole Slide Scanner and viewed using Pannoramic Viewer Software.

When cysts that had dolichos in their cyst wall were eliminated or markedly reduced in size and number, a compound was considered efficacious against bradyzoites in cysts.

#### Statistical Analyses

Significance of differences were determined using Student’s t-test. P < 0.05 was considered significant. Every experiment was replicated at least twice ^S12–15;S18,19^.

A Pearson test was used to confirm a correlation between increasing dose and increasing inhibition. An ANOVA and subsequent pair wise comparison with Dunnett correction was used to determine whether or not inhibition or toxicity at a given concentration was statistically significant. Stata/SE 12.1 was used for this analysis.

This study was approved by the University of Chicago IRB, IBC, and IACUC.

## *In vivo* Analysis (mice and zebra fish)

### Initial screening with tachyzoites using IVIS, fluorescence, and histopathology^S12–15;S59–62^

Ability of compounds to abrogate tachyzoites multiplication was assessed using an *in vivo* imaging system (IVIS). To facilitate this we have *T. gondii* strains from each of the 3 major lineages expressing the luciferase gene. In these studies mice are injected intraperitoneally with tachyzoites and parasite proliferation followed up to 30 days post infection. Removal of brains at 30 days allows parasite quantitation by bioluminescence *ex vivo* using the IVIS. As an alternative method to improve screening efficiency and scalability it is possible for initial screening to use zebrafish with histopathology and visualization as shown in Fig. 3. Quantitation also was performed using QT PCR as described for mice or in translucent Casper zebrafish with parasites with fluors or luciferase to screen rapidly. Tachyzoites and bradyzoites in cysts were used for IP infection and compounds given intraperitoneally.

### Type II parasites

To test type II parasites, we used *T. gondii* Me49 and Prugniaud parasites^S28,S39^.

### Encephalitis

The ability of compounds to reduce cyst burden and prevent encephalitis induced by the Type II strain of *T. gondii* were tested. Encephalitis was assessed by histological analyses and parasite burdens evaluated by quantitation of cysts^S59^.

### Oocyst induced disease^S39^

The oocyst challenge model is ideal for this study because oocysts can be diluted at one time and stored at 4 °C for 12 months without loss of infectivity titer. For treatment of chronic infection there were 5 to 10 mice per group treated 2 months after infection was established by compound in DMSO for parenteral administration administered once per day. Treatment was for 17 days.

### Zebrafish

Zebrafish were acclimatized to 37 degrees a degree a day and then infected with tachyzoites or cysts of RH YFP, Me49, *Veg T. gondii* as described^S62^. The use of RH YFP was performed for the first time herein in order to develop a rapidly scalable assay for drug development. This is the initial demonstration of cyst formation by 10 days in Zebrafish.

### Tissue processing and histopathology

All organs including eyes and brains were fixed in 0.1 M phosphate buffer (pH 7.4) containing 4% formaldehyde. Sections were cut from paraffin-embedded tissues and stained with Hematoxylin and Eosin (H&E) or immunoperoxidase stained. All sections were examined and assessed without knowledge of the group from which they originated^S39^.

## Testing of cytochrome b Qi mutant yeast

### Target Validation with Mutant *S. cerevisiae* (Growth Inhibition)^S63–73^

Three *S. cerevisiae* strains were used: M221Q and M221F cytochrome b mutants and wild type. They share the same nuclear genetic background deriving from AD1–9 (kindly given by M.Ghislain, UCL, Belgium). AD1–9 harbors multiple deletions in the ABC transporter genes that render the strain more sensitive to drugs than standard yeast strains^S65^.

Cytochrome b mutant M221F was generated by mitochondrial transformation as described^S65^. M221Q was selected as suppressor from a respiratory deficient mutant^S68^. Analysis of revertants from respiratory deficient mutants within the center N of cytochrome *b* in Saccharomyces cerevisiae.

Protocol from^S68^: Yeast strains were grown over 48 hours at 33 °C in liquid YPG medium [1% yeast extract, 2%(wt/vol) peptone, and 3%(vol/vol) (glycerol). Cultures were diluted to an OD_600_ of 0.05 and grown for 2 hrs. Cultures were then combined with YPG containing 6% melted agar for a total volume of 15–20 mL and poured onto OmniTray single-well rectangular plates that measured 86 mm by 128 mm (ThermoScientific) . Filter paper disks (7 mm diameter, 3 um thick) were placed onto the cooled agar plates. Compounds were dissolved in DMSO in diluted concentrations (1 mM, 500 μM, 100 μM, and 10 μM) and 10 microliters were applied to a disk. A single disk with DMSO on each plate was used as a control. Plates were incubated at 33 °C. Images were obtained after 4 days using GelDoc XR Imaging System (BioRad) and Quantity One software. Drug effect was assessed by the presence and size of a zone of inhibition around the disks.

### Testing of *P. falciparum*

D6 is a drug sensitive strain from Sierra Leone, C235 is a multi-drug resistant strain from Thailand, W2 is a chloroquine resistant strain from Thailand, and C2B has resistance to a variety of drugs including atovaquone. These assays were performed as described^S74^.

### Testing of *P. falciparum* cytochrome b Qi and DHODH mutants and drug combinations for *P. falciparum*

#### Parasite Strains and Culture Maintenance

We used the following parasite line from the MR4 repository of the American Type Culture Collection (ATCC): Dd2 (MRA-156). Mutant Dd2 parasites harboring a G33A or G33V substitution in cytochrome b were as reported^S77^. Dd2 parasites with a G131S mutation in cytochrome b and transgenic lines expressing a chromosomally integrated copy of the *S. cerevisiae* DHODH were utilized as previously described^S78^. Parasites were cultured by standard methods in RPMI media supplemented with 5% human O^+^ serum and 0.25% AlbuMAX^®^ II (Life Technologies 11021–045)^S79^.

### *In vitro* drug sensitivity and EC_50_ determinations

Drug susceptibility was measured using the SYBR Green method^S75^. Twelve point curves based on 2-fold dilutions of the test compound were carried out in triplicate each day and replicated on at least three different days. EC_50_ values were calculated using a nonlinear regression curve fit in Prism 6.0 for Mac (GraphPad Software, Inc.).

#### Studies of compound, drug combinations *in vitro*

Isobologram experiments were performed in similar fashion utilizing the modified fixed ratio methodology^S76^. Briefly, MJM170 and either atovaquone or cycloguanil or BRD6323^S79^ were mixed at multiple fixed volumetric ratios (10:0, 8:2, 6:4, 4:6, 2:8, and 0:10) and then serially diluted in 12-point 2-fold dilutions and dispensed in triplicate to 384-well assay plates and replicated on three different days. EC_50_ values were calculated as above, and FICs were calculated for each drug combination as described^S76^. Synergy was defined as an FIC < 1.0, additivity as FIC = 1.0, and antagonism as FIC > 1.0.

#### Molecular Modelling/Chemogenomics

X-ray structures of the cytochrome *bc*_1_ complex are available from the Protein DataBank^S80^. A homology model of the *T. gondii* cytochrome *bc*_1_ complex was generated using the Phyre^S81^ webserver. Molecular modelling and docking was performed on high performance Linux clusters at the University of Leeds, using specialist software: SPROUT^S82^ & eHiTs^S83^ (SymBioSis), Maestro & Glide^S84^ (Schrodinger), AutoDock^S84^ (Scripps Institute), ROCS/EON^S85^ & VIDA^S86^ (OpenEye) and the Marvin/JChem suites (ChemAxon)^S87^.

### X-ray crystallography

Cytochrome *bc*_*1*_ was purified as previously described^S88^. Crude bovine mitochondria were isolated from fresh cow heart and solubilised in DDM. The solution was clarified by ultracentrifugation at 200,000 g for 1 hour at 4 °C and the supernatant applied to a DEAE CL-6B sepharose column ca. 50 ml pre-equilibrated in 50 mM KPi (pH 7.5), 250 mM NaCl, 3 mM NaN_3_, 0.1 g/L DDM, washed with two CV and eluted along a gradient from 250 mM to 500 mM NaCl. Cyt. *bc*_*1*_ containing fractions were pooled and concentrated before loading on a Sepharose S300 column ca. 120 ml equilibrated with 20 mM KMOPS (pH 7.2), 100 mM NaCl, 0.5 mM EDTA, 0.1 g/L DDM at 0.5 ml/min. 10 mM MJM170 stock in DMSO was added to the eluted protein in a two-fold molar excess and allowed to incubate at 4 °C for 1 hour. Increasing amounts of PEG4000 were then added to precipitate cyt. bc_1_ and separate remaining contaminants. The cyt. *bc*_*1*_ was then resuspended before buffer exchange into a final buffer (25 mM KPi (pH 7.5), 3 mM NaN_3_, 0.015% DDM) and concentrated to 40 mg/ml. 1.6% HECAMEG was added to the protein solution prior to crystals growing by the hanging drop vapour diffusion method against a reservoir of 50 mM KPi (pH 6.8), 100 mM NaCl, 3 mM NaN_3_, 9% PEG4000, 0.16% HECMAEG. Crystals were flash frozen in 23% glycerol in reservoir solution as a cryoprotectant. Multiple wedges of data were collected at 100K from different points on the same crystal at I24 Diamond Light Source using 0.9686 Å X-rays with a Pilatus3 6M detector .

Datasets were processed in iMosflm and combined using Blend^S89^ to produce a complete merged dataset. Refinement was carried out with Refmac^S91^ using Prosmart^S90^ to generate secondary structure restraints to assist in the low-resolution refinement. The ligand MJM170 was produced using JLigand^S92^ and modelled in the Q_i_ site of cyt. *bc*_*1*_ using Coot^S93^. Cycles of alternating Refmac5 and manual modelling resulted in a completed model. Data collection and refinement statistics are summarised In Table 1. For 3715 residues 95.2% are Ramachandran favored, 4.6% allowed and 0.3% outliers.

## Interpretation of Data and Statistical Analyses

### Sample size and number of experiments

There were 3 replicate samples per group for *in vitro* experiments. All experiments were performed with sufficient sample sizes to have an 80% power to detect differences at the 5% level of significance.

### Statistics

Groups included untreated or mock treated controls. Results were compared using students T test, Chi square analysis or Fisher’s exact test as appropriate for the data set. When there were more than two groups, pairwise comparisons were made only when F-test for the ANOVA was significant at the 5% levels using protected least significant difference (LSD) test approach.

These methods were utilized to address the clinical problems presented in human toxoplasmosis^S94–S113^

## Additional Information

**Accession codes:** The data for this work presently in Supplements A and B including DNA sequence for EGS, RNA and miiR sequencing data and all analyses with large data sets will be deposited in Toxo db and/or at the Toxoplasmosis Research Institute website at toxoplasmosis.org (currently under revision).

**How to cite this article**: McPhillie, M. *et al.* New paradigms for understanding and step changes in treating active and chronic, persistent apicomplexan infections. *Sci. Rep.* **6**, 29179; doi: 10.1038/srep29179 (2016).

## Supplementary Material

Supplement A

Supplement B

## Figures and Tables

**Table 1:**
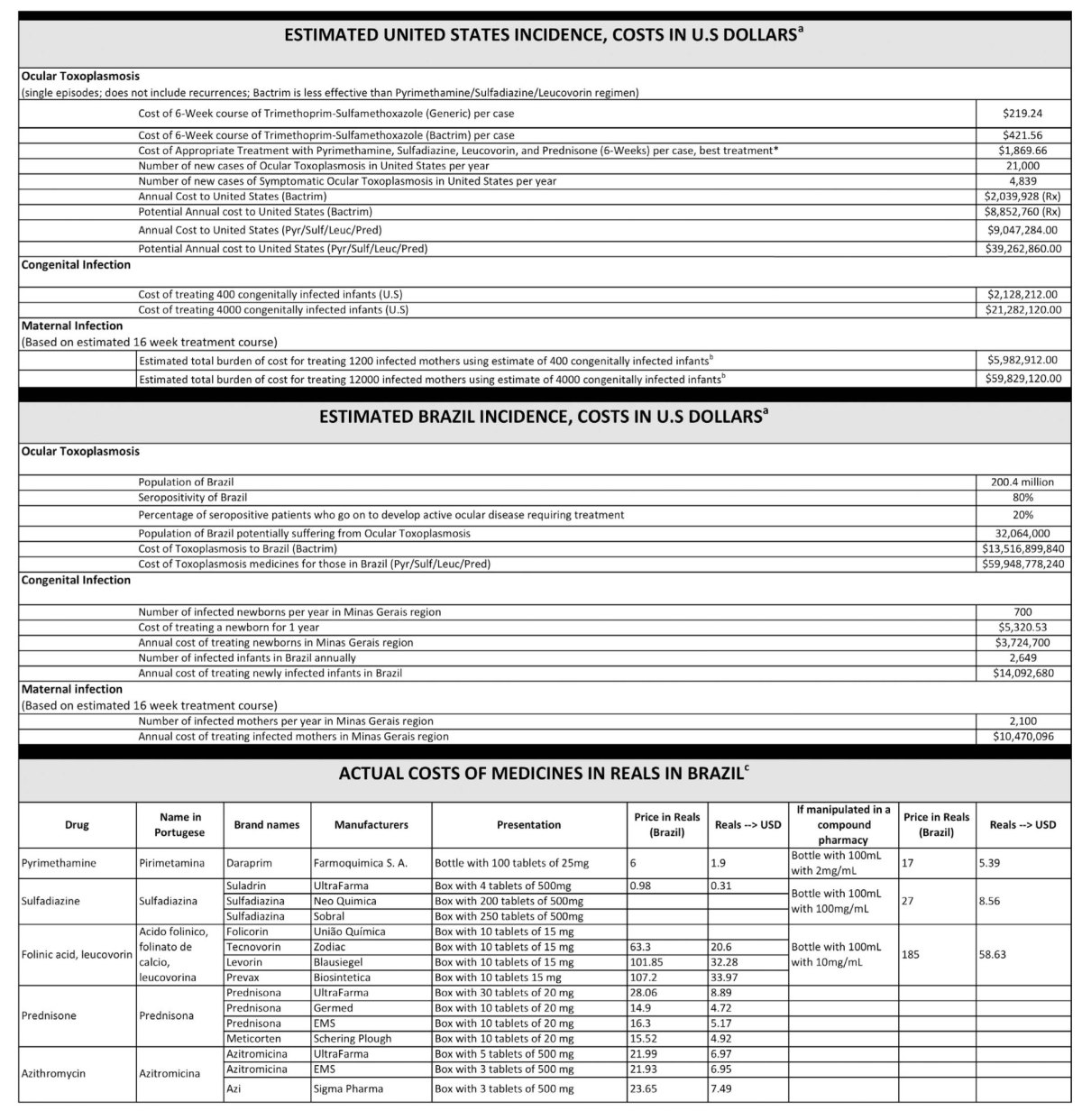
Cost Analysis of Treatment.

Note: Immune compromised not reported, assume cost similar to mothers multiplied by number of unknown cases.

^a^aCost of medicines, manufactured in the United States, prior to August 2015.

^b^Approximately 1/3 of infected mothers transmit to the fetus. cCost analysis data for Brazil provided by Eleonor G.Lago, MD, PhD

**Table 2:**
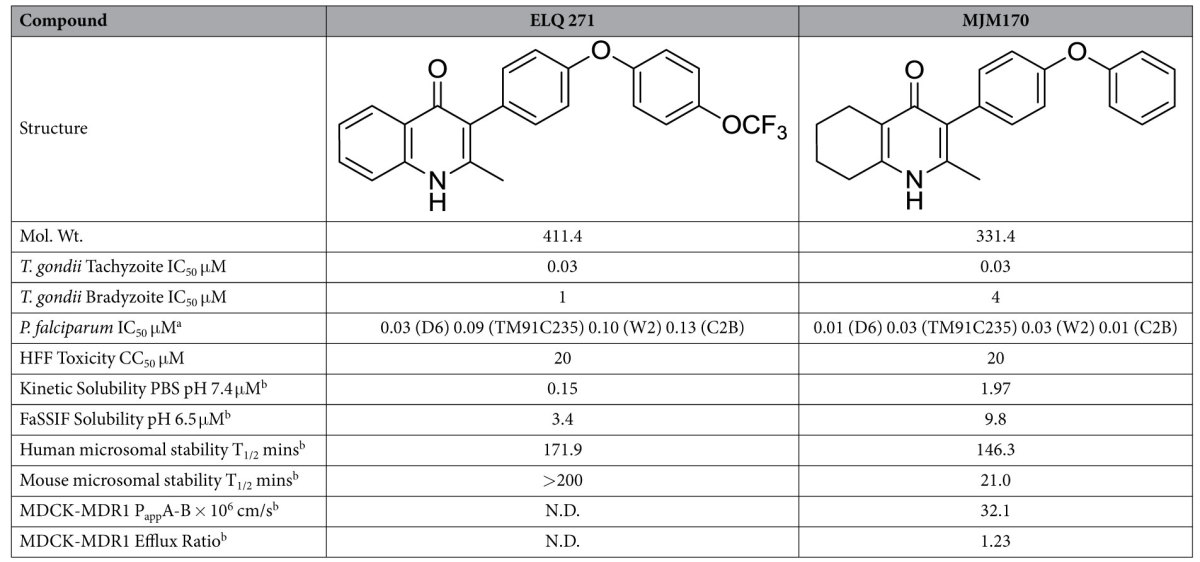
Comparison of ELQ 271 and MJM170 in our biological assays: inhibition of apicomplexan parasites and ADME/Tox. ELQ 271 was synthesised in-house.

^a^The D6 strain (Sierra Leone) is drug sensitive, the TM91C235 (Thailand) is multi-drug resistant, the W2 strain (Thailand) is chloroquine resistant, and the C2B strain is multi-drug resistant with pronounced resistance to atovaquone.

^b^ADME carried out by ChemPartner Shanghai Ltd. N.D. not determined. Human and mouse microsomal stability differs as is known to occur for other compounds such as TMP/SMX.
