# Author Correction: New paradigms for understanding and step changes in treating active and chronic, persistent apicomplexan infections

**DOI:** 10.1038/s41598-020-62323-1

**Published:** 2020-03-23

**Authors:** Martin McPhillie, Ying Zhou, Kamal El Bissati, Jitender Dubey, Hernan Lorenzi, Michael Capper, Amanda K. Lukens, Mark Hickman, Stephen Muench, Shiv Kumar Verma, Christopher R. Weber, Kelsey Wheeler, James Gordon, Justin Sanders, Hong Moulton, Kai Wang, Taek-Kyun Kim, Yuqing He, Tatiana Santos, Stuart Woods, Patty Lee, David Donkin, Eric Kim, Laura Fraczek, Joseph Lykins, Farida Esaa, Fatima Alibana-Clouser, Sarah Dovgin, Louis Weiss, Gael Brasseur, Dyann Wirth, Michael Kent, Leroy Hood, Brigitte Meunieur, Craig W. Roberts, S. Samar Hasnain, Svetlana V. Antonyuk, Colin Fishwick, Rima McLeod

**Affiliations:** 1 University of Leeds, Leeds, UK; 2 University of Chicago, Chicago, USA; 3 USDA, Beltsville, Maryland USA; 4 J Craig Venter Institute, Rockville Maryland, USA; 5 University of Liverpool, Liverpool, UK; 6 Harvard School of Public Health, Boston, Massachusetts, USA; 7 The Broad Institute, Boston, Massachusetts, USA; 8 Walter Reed Army Institute of Research, Silver Spring, Maryland USA; 9 Oregon State University, Corvallis, Oregon, USA; 10 Institute for Systems Biology, Seattle, Washington, USA; 11 Albert Einstein College of Medicine, Bronx, New York USA; 12 Strathclyde University, Glasgow, Scotland UK; 13 CNRS, Marseilles, France; 14 Institute for Integrative Biology of the Cell (12BC), Gif-sur-Yvette, France

Correction to: *Scientific Reports* 10.1038/srep29179, published online 14 July 2016

This Author Correction corrects the following errors in the Article. The authors neglected to cite a relevant study related to the isolation of the EGS strain of *Toxoplasma gondii* from amniotic fluid from a congenitally infected Brazilian fetus. As a result, in the Introduction,

“To address these challenges, we characterized the EGS parasite (Fig. 1), isolated in 1994 from amniotic fluid of a congenitally infected Brazilian fetus^25^, that form cyst-like structures *in vitro*.”

should read:

“To address these challenges, we characterized the EGS parasite (Fig. 1; ATCC® PRA-396), isolated in 1994 from amniotic fluid of a congenitally infected Brazilian fetus^1^ that form cyst-like structures *in vitro*^25^.”

In the Results section, under subheading ‘Characterization of EGS strain develops novel *in vitro* models to test compounds’,

“This AP2 represses bradyzoite to tachyzoite conversion, among other differences (Table 1; Fig. 1c; Supplement A: Box and Figure S2, Supplement B: Excel Table S1).”

should read:

“This AP2 represses tachyzoite to bradyzoite conversion^2^, among other differences (Table 1; Fig. 1c; Supplement A: Box and Figure S2, Supplement B: Excel Table S1).”

In Figure 6d, the incorrect image for transfected EGS was used. The correct Figure 6d appears below as Figure 1.

**Figure 1:**
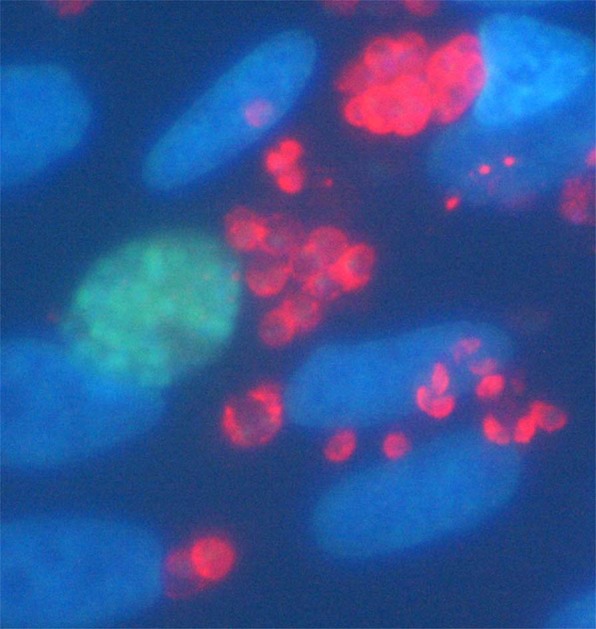
.

Finally, in the Discussion section,

“This Apetela 2 plant like transcription factor gene is known to drive tachyzoite switch by repressing bradyzoite genes.”

should read:

“This Apetela 2, plant-like transcription factor gene, AP2IV-4, represses bradyzoite genes during the tachyzoite cell cycle, thereby preventing commitment to the bradyzoite developmental pathway^2^.”
