# Supplementary material for: New paradigms for understanding and step changes in treating active and chronic, persistent apicomplexan infections
Source: Sci Rep. 2016 Jul 14;6:29179. doi: 10.1038/srep29179 (PMC4944145; doi:10.1038/srep29179)
Supplement: Supplement B [file srep29179-s2.pdf]

Supplement B

New paradigms for understanding and step changes in treating active and chronic, persistent apicomplexan infections

Martin McPhillie[1]*, Ying Zhou[2]*, Kamal El Bissati[2], Jitender Dubey[3], Hernan Lorenzi[4], Michael Capper[5], Amanda K Lukens[6,7], Mark Hickman[8], Stephen Muench[1], Shiv Kumar Verma[3] , Christopher R. Weber[2] , Kelsey Wheeler[2], James Gordon[1], Justin Sanders[9], Hong Moulton[9] , Kai Wang[10] , Taek-Kyun Kim[10], Yuqing He[10], Tatiana Santos [11], Stuart Woods[12] , Patty Lee[8], David Donkin[8], Eric Kim [8], Laura Fraczek[2], Joseph Lykins[2], Farida Esaa[2], Fatima Alibana-Clouser[2] , Sarah Dovgin[2], Louis Weiss[11], Gael Brasseur[14], Dyann Wirth[6,7] , Michael Kent [9], Leroy Hood[10], Brigitte Meunieur[13] , Craig W. Roberts[12], S. Samar Hasnain[5], Svetlana V. Antonyuk[5,+], Colin Fishwick[1, +], and Rima McLeod[2+]

[1] University of Leeds, Leeds, UK

[2] University of Chicago, Chicago, USA

[3] USDA, Beltsville, Maryland, USA

[4] J Craig Venter Institute, Rockville Maryland, USA

[5]University of Liverpool, Liverpool, UK

[6]Harvard School of Public Health, Boston, Massachusetts, USA

[7]The Broad Institute, Boston, Massachusetts, USA

[8] Walter Reed Army Institute of Research, Silver Spring, Maryland, USA

[9]Oregon State University, Corvallis, Oregon, USA

[10]Institute for Systems Biology, Seattle, Washington, USA

[11] Albert Einstein College of Medicine, Bronx, New York, USA

[12] Strathclyde University, Glasgow, Scotland, UK

[13] Institute for Integrative Biology of the Cell (12BC), Gif-sur-Yvette, France

[14] CNRS, Marseilles, France

*Equal contributions

[+] To whom correspondence should be addressed: Rima McLeod , rmcleod@uchicago.edu; Colin G. Fishwick, c.w.g-fishwick@leeds.ac.uk; Svetlana V. Antonyuk, antonyuk@liverpool.ac.uk

Competing financial interests: none

Author contribution statement: M.M conceived the idea of the study, contributed to concept and design, interpreted the results and drafted a part of the manuscript. Y.Z contributed to concept and design, performed experiments, interpreted experimental data, drafted and revised her part of the manuscript. K.E.B. contributed to concept and design, performed experiments, interpreted experimental data, drafted and revised his part of the manuscript. J.D. performed experiments and interpreted experimental data. H.L. carried out the statistical and bioinformatics data analyses, interpretation, and drafted his part of the manuscript. M.C. performed experiments and interpreted experimental data. A.L. performed experiments and interpreted experimental data. M.H. performed experiments and interpreted experimental data. S.M performed experiments and interpreted experimental data. S.K.V performed experiments and interpreted experimental data. C.W. interpreted experimental. K.WH. performed experiments and

interpreted experimental data. J.G. performed experiments and interpreted experimental data. J.S. performed experiments and interpreted experimental data. H.M. carried out the statistical and bioinformatics data analyses, interpretation, and drafted his part of the manuscript. K.WA carried out the statistical and bioinformatics data analyses, interpretation, and drafted his part of the manuscript. T-K.K carried out the statistical and bioinformatics data analyses, interpretation, and drafted his part of the manuscript Y.H. performed experiments and interpreted experimental data. T.S. performed experiments and interpreted experimental data. S.W. performed experiments and interpreted experimental data. P.L. performed experiments and interpreted experimental data. D.D. performed experiments and interpreted experimental data. E.K. performed experiments and interpreted experimental data. L.F. performed experiments and interpreted experimental data. J.L performed experiments and interpreted experimental data. F.E. performed experiments and interpreted experimental data. F.A-C. performed experiments and interpreted experimental data. S.D performed experiments and interpreted experimental data. L.W. interpreted experimental data. G.B. designed components of the experiment. D.W. interpreted experimental data. M.K. interpreted experimental data. L.H. performed experiments and interpreted experimental data. B.M. designed components of experiments. C.W.R. performed experiments and interpreted experimental data. S.S.H. contributed to concept and design, performed experiments, interpreted experimental data, drafted and revised his part of the manuscript. S.V.A performed experiments and interpreted experimental data. C.F. contributed to concept and design, performed experiments, interpreted experimental data, drafted and revised his part of the manuscript. R.M. conceived the idea of the study, contributed to all concepts and designs, coordinated the study, interpreted the results and drafted the manuscript. All authors reviewed the manuscript.

Supplemental Large Supporting data in Excel Tables INDEX:

1-Genes with nonsynonymous snps; Go SLIM Analysis
2-Transcriptomics of infected HFF over time-HFF
3-Transcriptomics of infected HFF over time-EGS
4-Transcriptomics-MM6 uninfected/infected EGS
5-Transcriptomics-MM6-EGS
6-Transcriptomics NSC uninfected/infected EGS
7-Transcriptomics NSC-EGS
8-MIR SEQ

Supplementary Table S1: T. gondii genes carrying potential change-of-function mutations in EGS but not in GT1, VEG or TgCatBr1 strains compared to reference strain ME49

Gene ID	Mutation	Mutation type	Product Name
TGME49_214850	p.Asn536Ser	missense_variant	1-deoxy-D-xylulose 5-phosphate reductoisomerase, putative
TGME49_214850	p.Asp273Gly	missense_variant	1-deoxy-D-xylulose 5-phosphate reductoisomerase, putative
TGME49_214850	p.Pro111Thr	missense_variant	1-deoxy-D-xylulose 5-phosphate reductoisomerase, putative
TGME49_278110	p.Ser2717Ile	missense_variant	1,3-beta-glucan synthase component protein
TGME49_278110	p.Ser2526Cys	missense_variant	1,3-beta-glucan synthase component protein
TGME49_278110	p.Glu2108Asp	missense_variant	1,3-beta-glucan synthase component protein
TGME49_278110	p.Val514Ala	missense_variant	1,3-beta-glucan synthase component protein
TGME49_319920	p.Glu302Asp	missense_variant	2-oxo acid dehydrogenases acyltransferase (catalytic domain) domain-containing protein
TGME49_319920	p.Asp301Gly	missense_variant	2-oxo acid dehydrogenases acyltransferase (catalytic domain) domain-containing protein
TGME49_280710	c.740+4C>G	splice_region_variant	20S proteasome subunit beta 7, putative
TGME49_277500	c.-996A>G	5_prime_UTR_premature_start_codon_gain_variant	26S proteasome regulatory subunit 7, putative
TGME49_246970	p.Thr1292Ile	missense_variant	3'-5' exonuclease domain-containing protein
TGME49_246970	p.Lys1266Thr	missense_variant	3'-5' exonuclease domain-containing protein
TGME49_246970	c.1910+7G>A	splice_region_variant	3'-5' exonuclease domain-containing protein
TGME49_246970	p.Glu510Asp	missense_variant	3'-5' exonuclease domain-containing protein
TGME49_246970	p.Gln126Lys	missense_variant	3'-5' exonuclease domain-containing protein
TGME49_246970	c.-197C>T	5_prime_UTR_premature_start_codon_gain_variant	3'-5' exonuclease domain-containing protein
TGME49_250890	p.Arg10Gly	missense_variant	3'-5' exonuclease domain-containing protein
TGME49_318675	c.2233-3C>T	splice_region_variant	3'5'-cyclic nucleotide phosphodiesterase domain-containing protein
TGME49_224840	p.Asp198Asn	missense_variant	3'5'-cyclic nucleotide phosphodiesterase domain-containing protein
TGME49_224840	p.Tyr204His	missense_variant	3'5'-cyclic nucleotide phosphodiesterase domain-containing protein
TGME49_239620	p.Ser494Ala	missense_variant	5'-nucleotidase, C-terminal domain-containing protein
TGME49_239620	c.-1182C>G	5_prime_UTR_premature_start_codon_gain_variant	5'-nucleotidase, C-terminal domain-containing protein
TGME49_217590	p.Val227Glu	missense_variant	50S ribosomal protein L17, putative
TGME49_217590	p.Glu300Lys	missense_variant	50S ribosomal protein L17, putative
TGME49_217590	p.Lys301Asn	missense_variant	50S ribosomal protein L17, putative
TGME49_223660	p.Pro83Ser	missense_variant	50S ribosomal protein L4, putative
TGME49_223660	p.His161Pro	missense_variant	50S ribosomal protein L4, putative
TGME49_223660	c.2200+8C>T	splice_region_variant	50S ribosomal protein L4, putative
TGME49_211690	c.3885-6A>G	splice_region_variant	AAA family protein
TGME49_239020	p.Glu1153Asp	missense_variant	ABC transporter transmembrane region domain-containing protein
TGME49_239020	p.Val799Met	missense_variant	ABC transporter transmembrane region domain-containing protein
TGME49_239020	p.Ala773Thr	missense_variant	ABC transporter transmembrane region domain-containing protein
TGME49_239020	p.Ala16Thr	missense_variant	ABC transporter transmembrane region domain-containing protein
TGME49_246600	c.-1618C>T	5_prime_UTR_premature_start_codon_gain_variant	ABC1 family protein
TGME49_246600	c.-174T>C	5_prime_UTR_premature_start_codon_gain_variant	ABC1 family protein
TGME49_246600	p.Lys712Gln	missense_variant	ABC1 family protein
TGME49_246600	p.Pro840Leu	missense_variant	ABC1 family protein
TGME49_301120	p.Ala11Thr	missense_variant	acetyl-CoA acetyltransferase
TGME49_301120	c.-387G>T	5_prime_UTR_premature_start_codon_gain_variant	acetyl-CoA acetyltransferase
TGME49_301120	c.-450C>G	5_prime_UTR_premature_start_codon_gain_variant	acetyl-CoA acetyltransferase
TGME49_221320	c.7568+8T>C	splice_region_variant	acetyl-CoA carboxylase ACC1
TGME49_221320	p.Ala1810Gly	missense_variant	acetyl-CoA carboxylase ACC1
TGME49_221320	p.Ala1068Thr	missense_variant	acetyl-CoA carboxylase ACC1
TGME49_218560	c.9338-6T>G	splice_region_variant	acetyl-coA carboxylase ACC2
TGME49_218560	c.8743+7A>G	splice_region_variant	acetyl-coA carboxylase ACC2
TGME49_218560	p.Ser2194Asn	missense_variant	acetyl-coA carboxylase ACC2
TGME49_218560	p.Pro2044Thr	missense_variant	acetyl-coA carboxylase ACC2
TGME49_221160	p.Arg152Gly	missense_variant	acetyltransferase, GNAT family protein
TGME49_221160	p.Gly110Glu	missense_variant	acetyltransferase, GNAT family protein
TGME49_221160	p.Gly103Glu	missense_variant	acetyltransferase, GNAT family protein
TGME49_219250	p.Arg721His	missense_variant	acetyltransferase, GNAT family protein
TGME49_219250	p.Leu617Pro	missense_variant	acetyltransferase, GNAT family protein
TGME49_219250	p.Gly426Ser	missense_variant	acetyltransferase, GNAT family protein
TGME49_219180	p.Asn643Ser	missense_variant	acetyltransferase, GNAT family protein
TGME49_219180	p.Thr173Met	missense_variant	acetyltransferase, GNAT family protein
TGME49_251490	p.Pro432Thr	missense_variant	ACR, COG2135 domain-containing protein
TGME49_251490	p.Pro28Ala	missense_variant	ACR, COG2135 domain-containing protein
TGME49_251490	p.Arg25Leu	missense_variant	ACR, COG2135 domain-containing protein
TGME49_251490	c.-353C>G	5_prime_UTR_premature_start_codon_gain_variant	ACR, COG2135 domain-containing protein
TGME49_251490	c.-393C>T	5_prime_UTR_premature_start_codon_gain_variant	ACR, COG2135 domain-containing protein
TGME49_251490	c.-509C>A	5_prime_UTR_premature_start_codon_gain_variant	ACR, COG2135 domain-containing protein
TGME49_234670	p.Leu90Phe	missense_variant	actin-like family protein
TGME49_234670	p.Asp227Ala	missense_variant	actin-like family protein
TGME49_234670	p.Leu416Val	missense_variant	actin-like family protein
TGME49_248890	c.-392C>G	5_prime_UTR_premature_start_codon_gain_variant	actin-like protein ALP3b
TGME49_248890	p.Val507Leu	missense_variant	actin-like protein ALP3b
TGME49_221410	c.-1237+1G>A	splice_donor_variant	actin-like protein ALP4
TGME49_253040	p.Glu594Gly	missense_variant	actin-related protein ARP4A
TGME49_253040	p.Asn410Ser	missense_variant	actin-related protein ARP4A
TGME49_253040	p.Pro365Thr	missense_variant	actin-related protein ARP4A
TGME49_253040	p.Ser364Phe	missense_variant	actin-related protein ARP4A
TGME49_253040	p.Gln61Glu	missense_variant	actin-related protein ARP4A
TGME49_253040	p.Val60Leu	missense_variant	actin-related protein ARP4A
TGME49_253040	p.Val39Ile	missense_variant	actin-related protein ARP4A
TGME49_249810	p.Leu33Phe	missense_variant	activating signal cointegrator 1 complex subunit 3, putative
TGME49_249810	p.Ala386Thr	missense_variant	activating signal cointegrator 1 complex subunit 3, putative
TGME49_249810	p.Leu405His	missense_variant	activating signal cointegrator 1 complex subunit 3, putative
TGME49_249810	p.Ala494Val	missense_variant	activating signal cointegrator 1 complex subunit 3, putative
TGME49_249810	c.3649+8G>A	splice_region_variant	activating signal cointegrator 1 complex subunit 3, putative
TGME49_249810	p.Met1547Arg	missense_variant	activating signal cointegrator 1 complex subunit 3, putative

TGME49_249810	p.Ala1659Thr	missense_variant	activating signal cointegrator 1 complex subunit 3, putative
TGME49_249810	p.Ser2028Leu	missense_variant	activating signal cointegrator 1 complex subunit 3, putative
TGME49_210800	c.728+6G>C	splice_region_variant	activator of hsp90 ATPase, putative
TGME49_247500	p.Ala692Ser	missense_variant	acyl-CoA dehydrogenase, middle domain-containing protein
TGME49_251550	c.*324T>C	splice_region_variant	acyl-coa-binding protein
TGME49_246800	c.-562C>T	5_prime_UTR_premature_start_codon_gain_variant	acylaminoacyl-peptidase, putative
TGME49_246800	p.Val444Ile	missense_variant	acylaminoacyl-peptidase, putative
TGME49_246800	p.Gln766Lys	missense_variant	acylaminoacyl-peptidase, putative
TGME49_246800	p.Leu851Phe	missense_variant	acylaminoacyl-peptidase, putative
TGME49_297640	p.Pro788Ala	missense_variant	acyltransferase domain-containing protein
TGME49_297640	p.Val317Ala	missense_variant	acyltransferase domain-containing protein
TGME49_297640	p.Val317Ile	missense_variant	acyltransferase domain-containing protein
TGME49_297640	p.His83Gln	missense_variant	acyltransferase domain-containing protein
TGME49_221940	c.-1008C>G	5_prime_UTR_premature_start_codon_gain_variant	adaptin n terminal region domain-containing protein
TGME49_221522	c.1297-6C>T	splice_region_variant	adaptor complexes medium subunit family protein
TGME49_215480	p.Asp456Glu	missense_variant	Adenosine/AMP deaminase domain-containing protein
TGME49_224900	c.-240C>T	5_prime_UTR_premature_start_codon_gain_variant	adenylate kinase, putative
TGME49_277760	p.Asn149Thr	missense_variant	adenylosuccinate lyase, putative
TGME49_277760	p.Ala153Thr	missense_variant	adenylosuccinate lyase, putative
TGME49_224110	p.Cys286Tyr	missense_variant	adhesion regulating molecule region protein, putative
TGME49_239530	p.Ser374Thr	missense_variant	alanine-glyoxylate aminotransferase
TGME49_221380	c.-867C>G	5_prime_UTR_premature_start_codon_gain_variant	alba 1
TGME49_222160	c.462+6A>G	splice_region_variant	aldehyde dehydrogenase
TGME49_222160	p.Lys58Thr	missense_variant	aldehyde dehydrogenase
TGME49_246690	c.-1282T>C	5_prime_UTR_premature_start_codon_gain_variant	alpha amylase, catalytic domain-containing protein
TGME49_246690	p.Arg59Ser	missense_variant	alpha amylase, catalytic domain-containing protein
TGME49_246690	p.Leu236Phe	missense_variant	alpha amylase, catalytic domain-containing protein
TGME49_246690	p.Thr262Ala	missense_variant	alpha amylase, catalytic domain-containing protein
TGME49_246690	p.Glu277Gly	missense_variant	alpha amylase, catalytic domain-containing protein
TGME49_246690	p.Gln281His	missense_variant	alpha amylase, catalytic domain-containing protein
TGME49_246690	c.1213+6T>G	splice_region_variant	alpha amylase, catalytic domain-containing protein
TGME49_246690	p.Ala883Gly	missense_variant	alpha amylase, catalytic domain-containing protein
TGME49_254880	p.Glu349Lys	missense_variant	Alpha-galactosidase
TGME49_254880	p.Phe714Leu	missense_variant	Alpha-galactosidase
TGME49_214260	c.-390C>T	5_prime_UTR_premature_start_codon_gain_variant	alpha-glucan water dikinase 1, putative
TGME49_214260	p.Pro12Ser	missense_variant	alpha-glucan water dikinase 1, putative
TGME49_214260	p.Ala746Thr	missense_variant	alpha-glucan water dikinase 1, putative
TGME49_214260	c.3002-4G>T	splice_region_variant	alpha-glucan water dikinase 1, putative
TGME49_319600	p.Thr644Lys	missense_variant	alpha-tubulin N-acetyltransferase, putative
TGME49_319600	p.Ser464Pro	missense_variant	alpha-tubulin N-acetyltransferase, putative
TGME49_319600	p.Asp319Asn	missense_variant	alpha-tubulin N-acetyltransferase, putative
TGME49_319600	p.Arg284Leu	missense_variant	alpha-tubulin N-acetyltransferase, putative
TGME49_319600	p.Glu2Asp	missense_variant	alpha-tubulin N-acetyltransferase, putative
TGME49_319600	c.-900C>G	5_prime_UTR_premature_start_codon_gain_variant	alpha-tubulin N-acetyltransferase, putative
TGME49_319600	c.-1283G>A	5_prime_UTR_premature_start_codon_gain_variant	alpha-tubulin N-acetyltransferase, putative
TGME49_210980	c.-759G>T	5_prime_UTR_premature_start_codon_gain_variant	alternative splicing type 3 and, putative
TGME49_248700	p.Glu4Asp	missense_variant	alveolin domain containing intermediate filament IMC12
TGME49_275670	p.Asn686Ser	missense_variant	alveolin domain containing intermediate filament IMC15
TGME49_275670	p.Ser622Cys	missense_variant	alveolin domain containing intermediate filament IMC15
TGME49_275670	p.Ala621Thr	missense_variant	alveolin domain containing intermediate filament IMC15
TGME49_275670	p.Gln608His	missense_variant&splice_region_variant	alveolin domain containing intermediate filament IMC15
TGME49_224530	p.Val49Leu	missense_variant	alveolin domain containing intermediate filament IMC5
TGME49_224520	c.-168C>T	5_prime_UTR_premature_start_codon_gain_variant	alveolin domain containing intermediate filament IMC8
TGME49_221310	p.Val86Gly	missense_variant	aminopeptidase N protein
TGME49_224460	c.-350G>A	5_prime_UTR_premature_start_codon_gain_variant	aminopeptidase n, putative
TGME49_224460	p.Asn66Asp	missense_variant	aminopeptidase n, putative
TGME49_224460	c.1753-4T>C	splice_region_variant	aminopeptidase n, putative
TGME49_224350	p.Glu623Ala	missense_variant	aminopeptidase N, putative
TGME49_224350	p.Gly141Val	missense_variant	aminopeptidase N, putative
TGME49_211090	p.Pro171Ser	missense_variant	aminotransferase, class V superfamily protein
TGME49_211090	p.Pro165Ser	missense_variant	aminotransferase, class V superfamily protein
TGME49_211090	p.Ser104Phe	missense_variant	aminotransferase, class V superfamily protein
TGME49_211090	p.Ala5Thr	missense_variant	aminotransferase, class V superfamily protein
TGME49_211090	c.-1215T>G	5_prime_UTR_premature_start_codon_gain_variant	aminotransferase, class V superfamily protein
TGME49_300120	p.Ala84Gly	missense_variant	aminotransferase, class V superfamily protein
TGME49_234280	p.Gly962Ser	missense_variant	AMP deaminase
TGME49_234280	p.Ala916Ser	missense_variant	AMP deaminase
TGME49_236980	p.Gly834Ser	missense_variant	AMP-binding enzyme domain-containing protein
TGME49_219230	p.Thr731Ala	missense_variant	AMP-binding enzyme domain-containing protein
TGME49_219230	p.Gln641His	missense_variant	AMP-binding enzyme domain-containing protein
TGME49_247760	c.1510-5C>T	splice_region_variant	AMP-binding enzyme domain-containing protein
TGME49_221910	c.28-4G>A	splice_region_variant	AN1 family Zinc finger domain-containing protein
TGME49_214500	p.Val73Met	missense_variant	ankyrin repeat-containing protein
TGME49_219210	p.Gly134Ala	missense_variant	ankyrin repeat-containing protein
TGME49_219210	p.Ser33Pro	missense_variant	ankyrin repeat-containing protein
TGME49_219210	c.-559T>C	5_prime_UTR_premature_start_codon_gain_variant	ankyrin repeat-containing protein
TGME49_219210	c.-1074G>T	5_prime_UTR_premature_start_codon_gain_variant	ankyrin repeat-containing protein
TGME49_254200	p.Arg921Lys	missense_variant	anticodon binding domain-containing protein
TGME49_254200	p.Thr744Ala	missense_variant	anticodon binding domain-containing protein
TGME49_254200	c.1814-5C>T	splice_region_variant	anticodon binding domain-containing protein
TGME49_254200	p.Ala413Val	missense_variant	anticodon binding domain-containing protein
TGME49_254200	p.Pro290Ser	missense_variant&splice_region_variant	anticodon binding domain-containing protein
TGME49_254200	p.Ile254Val	missense_variant	anticodon binding domain-containing protein
TGME49_254200	c.-399T>C	5_prime_UTR_premature_start_codon_gain_variant	anticodon binding domain-containing protein
TGME49_252370	p.Gly1813Cys	missense_variant	AP2 domain transcription factor AP2III-1

TGME49_252370	p.Glu1688Asp	missense_variant	AP2 domain transcription factor AP2III-1
TGME49_252370	p.Glu1632Asp	missense_variant	AP2 domain transcription factor AP2III-1
TGME49_252370	p.Ala1507Ser	missense_variant	AP2 domain transcription factor AP2III-1
TGME49_252370	p.Pro1365Ser	missense_variant	AP2 domain transcription factor AP2III-1
TGME49_252370	p.Pro1353Gln	missense_variant	AP2 domain transcription factor AP2III-1
TGME49_252370	p.Val922Ala	missense_variant	AP2 domain transcription factor AP2III-1
TGME49_252370	p.Gly607Ala	missense_variant	AP2 domain transcription factor AP2III-1
TGME49_252370	p.Thr207Met	missense_variant	AP2 domain transcription factor AP2III-1
TGME49_252370	p.Gly122Asp	missense_variant	AP2 domain transcription factor AP2III-1
TGME49_253380	c.-3324G>T	5_prime_UTR_premature_start_codon_gain_variant	AP2 domain transcription factor AP2III-2
TGME49_253380	c.-2969C>T	5_prime_UTR_premature_start_codon_gain_variant	AP2 domain transcription factor AP2III-2
TGME49_253380	p.Ala90Pro	missense_variant	AP2 domain transcription factor AP2III-2
TGME49_253380	p.Ala210Thr	missense_variant	AP2 domain transcription factor AP2III-2
TGME49_253380	p.Ala339Val	missense_variant	AP2 domain transcription factor AP2III-2
TGME49_253380	p.Asn419Lys	missense_variant	AP2 domain transcription factor AP2III-2
TGME49_253380	p.Ala459Val	missense_variant	AP2 domain transcription factor AP2III-2
TGME49_253380	p.Val775Leu	missense_variant	AP2 domain transcription factor AP2III-2
TGME49_253380	p.Ile1317Val	missense_variant	AP2 domain transcription factor AP2III-2
TGME49_253380	p.Leu1646Ser	missense_variant	AP2 domain transcription factor AP2III-2
TGME49_299150	p.Arg1877Met	missense_variant	AP2 domain transcription factor AP2III-3
TGME49_299150	p.Arg1817Thr	missense_variant	AP2 domain transcription factor AP2III-3
TGME49_299150	p.Lys1768Arg	missense_variant	AP2 domain transcription factor AP2III-3
TGME49_299150	p.Ser1608Asn	missense_variant	AP2 domain transcription factor AP2III-3
TGME49_299150	p.Ala1328Ser	missense_variant	AP2 domain transcription factor AP2III-3
TGME49_299150	p.Tyr1247Asn	missense_variant	AP2 domain transcription factor AP2III-3
TGME49_299150	p.Val1231Phe	missense_variant	AP2 domain transcription factor AP2III-3
TGME49_299150	p.Val1094Ile	missense_variant	AP2 domain transcription factor AP2III-3
TGME49_299150	p.Val1089Ala	missense_variant	AP2 domain transcription factor AP2III-3
TGME49_299150	p.Pro1020Ser	missense_variant	AP2 domain transcription factor AP2III-3
TGME49_299150	p.Thr780Met	missense_variant	AP2 domain transcription factor AP2III-3
TGME49_299150	p.Met761Ile	missense_variant	AP2 domain transcription factor AP2III-3
TGME49_299150	p.Arg655Pro	missense_variant	AP2 domain transcription factor AP2III-3
TGME49_299150	p.Pro493His	missense_variant	AP2 domain transcription factor AP2III-3
TGME49_299150	p.Val488Ala	missense_variant	AP2 domain transcription factor AP2III-3
TGME49_299150	p.Leu450Arg	missense_variant	AP2 domain transcription factor AP2III-3
TGME49_299150	p.Met392Thr	missense_variant	AP2 domain transcription factor AP2III-3
TGME49_299150	p.Ala306Glu	missense_variant	AP2 domain transcription factor AP2III-3
TGME49_299150	p.Ala283Thr	missense_variant	AP2 domain transcription factor AP2III-3
TGME49_299150	p.Ile126Thr	missense_variant	AP2 domain transcription factor AP2III-3
TGME49_299150	p.Glu111Lys	missense_variant	AP2 domain transcription factor AP2III-3
TGME49_299150	p.Thr78Pro	missense_variant	AP2 domain transcription factor AP2III-3
TGME49_299150	p.Cys49Arg	missense_variant	AP2 domain transcription factor AP2III-3
TGME49_299150	p.Ser41Pro	missense_variant	AP2 domain transcription factor AP2III-3
TGME49_299020	p.Gln885Pro	missense_variant	AP2 domain transcription factor AP2III-4
TGME49_299020	p.Leu746Val	missense_variant	AP2 domain transcription factor AP2III-4
TGME49_318610	p.Thr697Ser	missense_variant	AP2 domain transcription factor AP2IV-3
TGME49_318610	p.Met888Val	missense_variant	AP2 domain transcription factor AP2IV-3
TGME49_318470	p.Thr520Ser	missense_variant	AP2 domain transcription factor AP2IV-4
TGME49_318470	p.Met570Ile	missense_variant	AP2 domain transcription factor AP2IV-4
TGME49_318470	p.Pro1816Thr	missense_variant	AP2 domain transcription factor AP2IV-4
TGME49_318470	p.Phe1873Leu	missense_variant	AP2 domain transcription factor AP2IV-4
TGME49_318470	p.Met1882Thr	missense_variant	AP2 domain transcription factor AP2IV-4
TGME49_318470	p.Leu1923Arg	missense_variant	AP2 domain transcription factor AP2IV-4
TGME49_318470	p.Ala1925Gly	missense_variant	AP2 domain transcription factor AP2IV-4
TGME49_318470	p.Lys1936Thr	missense_variant	AP2 domain transcription factor AP2IV-4
TGME49_318470	p.Gly1941Asp	missense_variant	AP2 domain transcription factor AP2IV-4
TGME49_318470	p.Thr2130Pro	missense_variant	AP2 domain transcription factor AP2IV-4
TGME49_318470	p.Asp2398Glu	missense_variant	AP2 domain transcription factor AP2IV-4
TGME49_211720	p.Arg1814Cys	missense_variant	AP2 domain transcription factor AP2IV-5
TGME49_211720	p.Leu1588Ser	missense_variant	AP2 domain transcription factor AP2IV-5
TGME49_211720	p.Val1361Leu	missense_variant	AP2 domain transcription factor AP2IV-5
TGME49_211720	c.-271C>T	5_prime_UTR_premature_start_codon_gain_variant	AP2 domain transcription factor AP2IV-5
TGME49_211720	c.-1993T>G	5_prime_UTR_premature_start_codon_gain_variant	AP2 domain transcription factor AP2IV-5
TGME49_215340	p.Ser87Tyr	missense_variant	AP2 domain transcription factor AP2X-10
TGME49_215570	p.Glu1178Asp	missense_variant	AP2 domain transcription factor AP2X-11
TGME49_215570	p.Asn1073Thr	missense_variant	AP2 domain transcription factor AP2X-11
TGME49_215570	p.Glu987Asp	missense_variant	AP2 domain transcription factor AP2X-11
TGME49_215570	p.Asn962Thr	missense_variant	AP2 domain transcription factor AP2X-11
TGME49_215570	p.Ala225Val	missense_variant	AP2 domain transcription factor AP2X-11
TGME49_215570	p.Gly87Ser	missense_variant	AP2 domain transcription factor AP2X-11
TGME49_215570	p.Gln71His	missense_variant	AP2 domain transcription factor AP2X-11
TGME49_224230	p.Ser426Ala	missense_variant	AP2 domain transcription factor AP2X-3
TGME49_224230	c.3352-6G>A	splice_region_variant	AP2 domain transcription factor AP2X-3
TGME49_224230	p.Gly1639Val	missense_variant	AP2 domain transcription factor AP2X-3
TGME49_237090	p.Met211Val	missense_variant	AP2 domain transcription factor AP2X-5
TGME49_237090	p.Ala943Thr	missense_variant	AP2 domain transcription factor AP2X-5
TGME49_237090	p.Gly1871Trp	missense_variant	AP2 domain transcription factor AP2X-5
TGME49_237425	p.Gly853Val	missense_variant	AP2 domain transcription factor AP2X-6
TGME49_237425	p.Thr855Pro	missense_variant	AP2 domain transcription factor AP2X-6
TGME49_237425	p.Ser988Ile	missense_variant	AP2 domain transcription factor AP2X-6
TGME49_237425	p.Phe1066Val	missense_variant	AP2 domain transcription factor AP2X-6
TGME49_237425	p.Met1109Thr	missense_variant	AP2 domain transcription factor AP2X-6
TGME49_237425	p.Arg1303Ile	missense_variant	AP2 domain transcription factor AP2X-6
TGME49_237425	p.Pro1724Ser	missense_variant	AP2 domain transcription factor AP2X-6
TGME49_237425	p.Ser1997Thr	missense_variant	AP2 domain transcription factor AP2X-6

TGME49_237425	p.Gln2085Glu	missense_variant	AP2 domain transcription factor AP2X-6
TGME49_237425	p.Ala2272Val	missense_variant	AP2 domain transcription factor AP2X-6
TGME49_237425	p.Thr2382Ala	missense_variant	AP2 domain transcription factor AP2X-6
TGME49_237425	p.Met4244Leu	missense_variant	AP2 domain transcription factor AP2X-6
TGME49_214840	c.-4282G>T	5_prime_UTR_premature_start_codon_gain_variant	AP2 domain transcription factor AP2X-7
TGME49_214960	p.Asp282Glu	missense_variant	AP2 domain transcription factor AP2X-8
TGME49_214960	p.Ala799Thr	missense_variant	AP2 domain transcription factor AP2X-8
TGME49_214960	p.Gly1580Val	missense_variant	AP2 domain transcription factor AP2X-8
TGME49_214960	p.Ile1594Thr	missense_variant	AP2 domain transcription factor AP2X-8
TGME49_214960	p.Val1725Ala	missense_variant	AP2 domain transcription factor AP2X-8
TGME49_214960	p.Ala1752Thr	missense_variant	AP2 domain transcription factor AP2X-8
TGME49_214960	p.Ala2369Thr	missense_variant	AP2 domain transcription factor AP2X-8
TGME49_214960	p.Thr2484Ala	missense_variant	AP2 domain transcription factor AP2X-8
TGME49_214960	p.Thr2841Ala	missense_variant	AP2 domain transcription factor AP2X-8
TGME49_214960	p.Ala2849Pro	missense_variant	AP2 domain transcription factor AP2X-8
TGME49_215150	p.Ala349Thr	missense_variant	AP2 domain transcription factor AP2X-9
TGME49_215150	p.Ser148Asn	missense_variant	AP2 domain transcription factor AP2X-9
TGME49_215150	c.-1208A>G	5_prime_UTR_premature_start_codon_gain_variant	AP2 domain transcription factor AP2X-9
TGME49_218960	p.Ser2017Pro	missense_variant	AP2 domain transcription factor AP2XII-1
TGME49_218960	p.Leu213Ile	missense_variant	AP2 domain transcription factor AP2XII-1
TGME49_218960	p.Gly72Ser	missense_variant	AP2 domain transcription factor AP2XII-1
TGME49_218960	p.Ala28Val	missense_variant	AP2 domain transcription factor AP2XII-1
TGME49_217700	p.Pro15Leu	missense_variant	AP2 domain transcription factor AP2XII-2
TGME49_217700	p.Ala181Thr	missense_variant	AP2 domain transcription factor AP2XII-2
TGME49_217700	p.Ala364Ser	missense_variant	AP2 domain transcription factor AP2XII-2
TGME49_217700	p.Ala579Thr	missense_variant	AP2 domain transcription factor AP2XII-2
TGME49_217700	p.Glu1071Ala	missense_variant	AP2 domain transcription factor AP2XII-2
TGME49_217700	p.Leu1184Ile	missense_variant	AP2 domain transcription factor AP2XII-2
TGME49_246660	c.-406T>G	5_prime_UTR_premature_start_codon_gain_variant	AP2 domain transcription factor AP2XII-3
TGME49_247700	p.Ser3776Ala	missense_variant	AP2 domain transcription factor AP2XII-4
TGME49_247700	p.Ala3767Pro	missense_variant	AP2 domain transcription factor AP2XII-4
TGME49_247700	p.Ala3744Gly	missense_variant	AP2 domain transcription factor AP2XII-4
TGME49_247700	p.Gly2877Ser	missense_variant	AP2 domain transcription factor AP2XII-4
TGME49_247700	p.Leu2450Val	missense_variant	AP2 domain transcription factor AP2XII-4
TGME49_247700	p.Gln2342Pro	missense_variant	AP2 domain transcription factor AP2XII-4
TGME49_247700	p.Ser2271Gly	missense_variant	AP2 domain transcription factor AP2XII-4
TGME49_247700	p.Glu1615Asp	missense_variant	AP2 domain transcription factor AP2XII-4
TGME49_247700	p.Gly1062Ala	missense_variant	AP2 domain transcription factor AP2XII-4
TGME49_247700	p.Pro597Leu	missense_variant	AP2 domain transcription factor AP2XII-4
TGME49_247700	p.Gly159Cys	missense_variant	AP2 domain transcription factor AP2XII-4
TGME49_247730	p.Ala150Gly	missense_variant	AP2 domain transcription factor AP2XII-5
TGME49_247730	p.Val92Leu	missense_variant	AP2 domain transcription factor AP2XII-5
TGME49_249190	p.Asn278Asp	missense_variant	AP2 domain transcription factor AP2XII-6
TGME49_249190	p.Ser221Arg	missense_variant	AP2 domain transcription factor AP2XII-6
TGME49_249190	c.-262C>T	5_prime_UTR_premature_start_codon_gain_variant	AP2 domain transcription factor AP2XII-6
TGME49_251740	p.Ala1602Thr	missense_variant	AP2 domain transcription factor AP2XII-9
TGME49_251740	p.Thr1169Pro	missense_variant	AP2 domain transcription factor AP2XII-9
TGME49_251740	p.Ser1052Thr	missense_variant	AP2 domain transcription factor AP2XII-9
TGME49_251740	p.His926Gln	missense_variant	AP2 domain transcription factor AP2XII-9
TGME49_251740	p.Ala636Val	missense_variant	AP2 domain transcription factor AP2XII-9
TGME49_251740	p.Pro628Ala	missense_variant	AP2 domain transcription factor AP2XII-9
TGME49_251740	p.Ala626Thr	missense_variant	AP2 domain transcription factor AP2XII-9
TGME49_251740	p.Pro601Arg	missense_variant	AP2 domain transcription factor AP2XII-9
TGME49_249650	p.Asp62Asn	missense_variant	apolipoprotein A-I binding protein, putative
TGME49_249650	p.Leu77Arg	missense_variant	apolipoprotein A-I binding protein, putative
TGME49_249650	p.Pro79Leu	missense_variant	apolipoprotein A-I binding protein, putative
TGME49_249650	p.Pro87Ala	missense_variant	apolipoprotein A-I binding protein, putative
TGME49_249650	p.Cys88Phe	missense_variant	apolipoprotein A-I binding protein, putative
TGME49_299990	p.Lys241Arg	missense_variant	archease family protein
TGME49_299990	p.Ser76Pro	missense_variant	archease family protein
TGME49_299990	c.-463A>G	5_prime_UTR_premature_start_codon_gain_variant	archease family protein
TGME49_299990	c.-663C>T	5_prime_UTR_premature_start_codon_gain_variant	archease family protein
TGME49_280700	c.-616T>G	5_prime_UTR_premature_start_codon_gain_variant	arginine decarboxylase
TGME49_210840	p.Val282Ile	missense_variant	arginyl-tRNA synthetase family protein
TGME49_210840	c.543+8A>C	splice_region_variant	arginyl-tRNA synthetase family protein
TGME49_235980	c.-400C>G	5_prime_UTR_premature_start_codon_gain_variant	ARIADNE family protein
TGME49_235980	c.-329C>G	5_prime_UTR_premature_start_codon_gain_variant	ARIADNE family protein
TGME49_235980	c.-251C>G	5_prime_UTR_premature_start_codon_gain_variant	ARIADNE family protein
TGME49_235980	c.-174T>G	5_prime_UTR_premature_start_codon_gain_variant	ARIADNE family protein
TGME49_235980	c.214-3C>T	splice_region_variant	ARIADNE family protein
TGME49_246170	p.Ser974Gly	missense_variant	ARID/BRIGHT DNA binding domain-containing protein
TGME49_246170	p.Gly1340Glu	missense_variant	ARID/BRIGHT DNA binding domain-containing protein
TGME49_246170	p.Gly1425Val	missense_variant	ARID/BRIGHT DNA binding domain-containing protein
TGME49_237600	c.-245T>G	5_prime_UTR_premature_start_codon_gain_variant	Armadillo/beta-catenin family repeat-containing protein
TGME49_307990	p.Leu192Met	missense_variant	arrestin (or s-antigen), n-terminal domain-containing protein
TGME49_307990	p.Met71Val	missense_variant	arrestin (or s-antigen), n-terminal domain-containing protein
TGME49_307990	p.Ser54Arg	missense_variant	arrestin (or s-antigen), n-terminal domain-containing protein
TGME49_248600	p.Leu5Arg	missense_variant	aspartate aminotransferase
TGME49_278750	c.-37T>G	5_prime_UTR_premature_start_codon_gain_variant	AT hook motif domain-containing protein
TGME49_278750	p.Pro155Leu	missense_variant	AT hook motif domain-containing protein
TGME49_215090	c.448-4T>G	splice_region_variant	ATP binding protein, putative
TGME49_249820	c.3855-6C>G	splice_region_variant	ATP-binding cassette sub-family B member 5
TGME49_249820	p.Arg1167His	missense_variant	ATP-binding cassette sub-family B member 5
TGME49_249820	p.Thr148Ala	missense_variant	ATP-binding cassette sub-family B member 5
TGME49_318710	c.655+6G>T	splice_region_variant	ATP-binding cassette sub-family F member 1

TGME49_223840	p.Pro165Leu	missense_variant	ATP-citrate lyase, putative
TGME49_318320	c.-407G>T	5_prime_UTR_premature_start_codon_gain_variant	ATP-dependent Clp endopeptidase, proteolytic subunit ClpP domain-containing protein
TGME49_318320	p.Ser46Pro	missense_variant	ATP-dependent Clp endopeptidase, proteolytic subunit ClpP domain-containing protein
TGME49_318320	p.Lys126Arg	missense_variant	ATP-dependent Clp endopeptidase, proteolytic subunit ClpP domain-containing protein
TGME49_318320	p.Ser140Trp	missense_variant	ATP-dependent Clp endopeptidase, proteolytic subunit ClpP domain-containing protein
TGME49_318320	p.Arg302Ser	missense_variant	ATP-dependent Clp endopeptidase, proteolytic subunit ClpP domain-containing protein
TGME49_318320	c.1160-4T>G	splice_region_variant	ATP-dependent Clp endopeptidase, proteolytic subunit ClpP domain-containing protein
TGME49_210730	c.1657-4C>T	splice_region_variant	ATP-dependent hsl protease ATP-binding subunit hslU, putative
TGME49_300020	p.Ala102Thr	missense_variant	ATP-dependent metallopeptidase HflB subfamily protein
TGME49_300020	c.1879-8T>G	splice_region_variant	ATP-dependent metallopeptidase HflB subfamily protein
TGME49_218610	p.His796Asp	missense_variant	ATPase (DUF699) protein
TGME49_252250	p.Pro1124Thr	missense_variant&splice_region_variant	ATPase, AAA family protein
TGME49_252250	p.Arg637Trp	missense_variant	ATPase, AAA family protein
TGME49_252250	p.Thr180Ala	missense_variant	ATPase, AAA family protein
TGME49_299168	c.1106+8C>A	splice_region_variant	ATPase, AAA family protein
TGME49_299168	p.Phe298Leu	missense_variant	ATPase, AAA family protein
TGME49_299168	p.Ala59Thr	missense_variant	ATPase, AAA family protein
TGME49_299168	p.Met41Ile	missense_variant	ATPase, AAA family protein
TGME49_299168	p.Leu6Pro	missense_variant	ATPase, AAA family protein
TGME49_211050	p.Gln334Arg	missense_variant	ATPase, AAA family protein
TGME49_235170	c.-811C>T	5_prime_UTR_premature_start_codon_gain_variant	ATPase, AAA family protein
TGME49_235170	p.Arg142Pro	missense_variant	ATPase, AAA family protein
TGME49_235170	p.Ala461Pro	missense_variant	ATPase, AAA family protein
TGME49_235610	p.Val1204Gly	missense_variant	ATPase, AAA family protein
TGME49_235610	p.Ser274Leu	missense_variant	ATPase, AAA family protein
TGME49_247390	p.Asp299His	missense_variant	ATPase, AAA family protein
TGME49_247390	p.Glu652Ala	missense_variant	ATPase, AAA family protein
TGME49_247390	c.4367-8T>G	splice_region_variant	ATPase, AAA family protein
TGME49_247390	p.Glu1677Asp	missense_variant	ATPase, AAA family protein
TGME49_247390	p.Arg1683Leu	missense_variant	ATPase, AAA family protein
TGME49_247390	p.Glu1684Asp	missense_variant	ATPase, AAA family protein
TGME49_247390	p.Ala1861Thr	missense_variant	ATPase, AAA family protein
TGME49_247390	p.Leu2456Val	missense_variant	ATPase, AAA family protein
TGME49_247390	p.Arg2524Ser	missense_variant	ATPase, AAA family protein
TGME49_247390	p.Arg2524His	missense_variant	ATPase, AAA family protein
TGME49_247390	p.Gly2767Glu	missense_variant	ATPase, AAA family protein
TGME49_247390	p.Asp2775Glu	missense_variant	ATPase, AAA family protein
TGME49_247390	p.Thr2838Ala	missense_variant	ATPase, AAA family protein
TGME49_247390	p.Ser2944Pro	missense_variant	ATPase, AAA family protein
TGME49_247390	p.Glu2987Gly	missense_variant	ATPase, AAA family protein
TGME49_247390	p.Ser3092Asn	missense_variant	ATPase, AAA family protein
TGME49_247390	p.Arg3131Leu	missense_variant	ATPase, AAA family protein
TGME49_247390	p.Asp3175Gly	missense_variant	ATPase, AAA family protein
TGME49_247390	p.Ala3473Thr	missense_variant	ATPase, AAA family protein
TGME49_297780	c.-1217C>G	5_prime_UTR_premature_start_codon_gain_variant	ATPase/histidine kinase/DNA gyrase B/HSP90 domain-containing protein
TGME49_297780	p.Pro183Leu	missense_variant	ATPase/histidine kinase/DNA gyrase B/HSP90 domain-containing protein
TGME49_297780	p.Pro190Leu	missense_variant	ATPase/histidine kinase/DNA gyrase B/HSP90 domain-containing protein
TGME49_318770	p.Leu1394Val	missense_variant	aurora kinase(incomplete catalytic triad)
TGME49_318770	p.Pro1043Thr	missense_variant	aurora kinase(incomplete catalytic triad)
TGME49_318770	p.Asn475Ser	missense_variant	aurora kinase(incomplete catalytic triad)
TGME49_318770	p.Pro327Ala	missense_variant	aurora kinase(incomplete catalytic triad)
TGME49_318770	p.Pro295Leu	missense_variant	aurora kinase(incomplete catalytic triad)
TGME49_318770	p.Asp286Asn	missense_variant	aurora kinase(incomplete catalytic triad)
TGME49_318770	p.Gln224Arg	missense_variant	aurora kinase(incomplete catalytic triad)
TGME49_318770	p.Gln221Glu	missense_variant	aurora kinase(incomplete catalytic triad)
TGME49_318770	p.Thr215Arg	missense_variant	aurora kinase(incomplete catalytic triad)
TGME49_318770	p.Phe20Ser	missense_variant	aurora kinase(incomplete catalytic triad)
TGME49_299190	p.Glu42Gln	missense_variant	B-box zinc finger domain-containing protein
TGME49_299190	p.Ser279Ala	missense_variant	B-box zinc finger domain-containing protein
TGME49_299190	p.Val499Ile	missense_variant	B-box zinc finger domain-containing protein
TGME49_299190	p.Leu682Ile	missense_variant	B-box zinc finger domain-containing protein
TGME49_299190	p.Leu682His	missense_variant	B-box zinc finger domain-containing protein
TGME49_299190	p.Asp714Asn	missense_variant	B-box zinc finger domain-containing protein
TGME49_299190	p.Asp738Asn	missense_variant	B-box zinc finger domain-containing protein
TGME49_288390	p.Leu253Val	missense_variant	B3GNTL1 protein
TGME49_239930	c.-84C>T	5_prime_UTR_premature_start_codon_gain_variant	beta-catenin family protein 1, putative
TGME49_239930	c.-269C>G	5_prime_UTR_premature_start_codon_gain_variant	beta-catenin family protein 1, putative
TGME49_236990	p.Leu1614Ile	missense_variant	beta-ketoacyl synthase, N-terminal domain-containing protein
TGME49_297420	p.Ile2016Leu	missense_variant	beta-tubulin cofactor D, putative
TGME49_297420	p.Val1913Gly	missense_variant	beta-tubulin cofactor D, putative
TGME49_297420	p.Ala1503Val	missense_variant	beta-tubulin cofactor D, putative
TGME49_297420	p.Asn994Asp	missense_variant	beta-tubulin cofactor D, putative
TGME49_297420	p.Ala666Thr	missense_variant	beta-tubulin cofactor D, putative
TGME49_297420	p.Glu650Lys	missense_variant	beta-tubulin cofactor D, putative
TGME49_297420	p.Glu391Asp	missense_variant	beta-tubulin cofactor D, putative
TGME49_297420	p.Lys345Glu	missense_variant	beta-tubulin cofactor D, putative
TGME49_297420	p.Cys284Trp	missense_variant	beta-tubulin cofactor D, putative
TGME49_297420	p.Thr167Ser	missense_variant	beta-tubulin cofactor D, putative
TGME49_297420	c.-1213T>G	5_prime_UTR_premature_start_codon_gain_variant	beta-tubulin cofactor D, putative
TGME49_297420	c.-2395A>T	5_prime_UTR_premature_start_codon_gain_variant	beta-tubulin cofactor D, putative
TGME49_249180	p.Glu591Asp	missense_variant	bifunctional dihydrofolate reductase-thymidylate synthase
TGME49_239320	c.729-7T>C	splice_region_variant	BolA family protein
TGME49_235880	p.Gly31Ala	missense_variant	brain protein 44 family protein
TGME49_239790	c.-200C>T	5_prime_UTR_premature_start_codon_gain_variant	BRCA1 C Terminus (BRCT) domain-containing protein
TGME49_239790	c.1552+8G>T	splice_region_variant	BRCA1 C Terminus (BRCT) domain-containing protein

TGME49_237480	p.Arg1802Gly	missense_variant	BRCA1 C Terminus (BRCT) domain-containing protein
TGME49_237480	p.Arg1744His	missense_variant	BRCA1 C Terminus (BRCT) domain-containing protein
TGME49_237480	p.Pro1715Leu	missense_variant	BRCA1 C Terminus (BRCT) domain-containing protein
TGME49_237480	p.Ser1627Cys	missense_variant	BRCA1 C Terminus (BRCT) domain-containing protein
TGME49_237480	p.Tyr1181Cys	missense_variant	BRCA1 C Terminus (BRCT) domain-containing protein
TGME49_237480	p.Thr406Ile	missense_variant	BRCA1 C Terminus (BRCT) domain-containing protein
TGME49_237480	p.Pro132Ser	missense_variant	BRCA1 C Terminus (BRCT) domain-containing protein
TGME49_237480	p.Ala128Val	missense_variant	BRCA1 C Terminus (BRCT) domain-containing protein
TGME49_237480	p.Met101Val	missense_variant	BRCA1 C Terminus (BRCT) domain-containing protein
TGME49_237480	p.Val68Ala	missense_variant	BRCA1 C Terminus (BRCT) domain-containing protein
TGME49_237480	p.Val68Leu	missense_variant	BRCA1 C Terminus (BRCT) domain-containing protein
TGME49_222000	p.Thr412Ile	missense_variant	brix domain-containing protein
TGME49_222000	p.Lys196Glu	missense_variant	brix domain-containing protein
TGME49_214240	p.Ala311Glu	missense_variant	bromodomain-containing protein
TGME49_214240	p.Phe1129Val	missense_variant	bromodomain-containing protein
TGME49_214240	p.Ala2472Glu	missense_variant	bromodomain-containing protein
TGME49_236020	p.Thr586Ala	missense_variant	BT1 family protein
TGME49_238240	c.-377A>T	5_prime_UTR_premature_start_codon_gain_variant	bystin protein
TGME49_254750	p.Thr291Ile	missense_variant	c3orf15 protein
TGME49_251570	p.Leu1206Phe	missense_variant	CAAX amino terminal protease family protein
TGME49_251570	p.Leu1027Ile	missense_variant	CAAX amino terminal protease family protein
TGME49_251570	p.Pro511Leu	missense_variant	CAAX amino terminal protease family protein
TGME49_300340	p.Ala146Val	missense_variant	CAF1 family ribonuclease
TGME49_318540	p.Ala101Val	missense_variant	calcium binding egf domain-containing protein
TGME49_318540	p.Ser46Cys	missense_variant	calcium binding egf domain-containing protein
TGME49_254190	c.-1556C>T	5_prime_UTR_premature_start_codon_gain_variant	calcium signaling protein kinase MARK, putative
TGME49_254190	c.-1306G>C	5_prime_UTR_premature_start_codon_gain_variant	calcium signaling protein kinase MARK, putative
TGME49_254190	c.-568C>T	5_prime_UTR_premature_start_codon_gain_variant	calcium signaling protein kinase MARK, putative
TGME49_254190	c.-94T>G	5_prime_UTR_premature_start_codon_gain_variant	calcium signaling protein kinase MARK, putative
TGME49_301440	c.-2483G>T	5_prime_UTR_premature_start_codon_gain_variant	calcium-dependent protein kinase CDPK1
TGME49_301440	c.1018+8G>T	splice_region_variant	calcium-dependent protein kinase CDPK1
TGME49_237890	c.-521G>T	5_prime_UTR_premature_start_codon_gain_variant	calcium-dependent protein kinase CDPK4
TGME49_237890	p.Ala227Val	missense_variant	calcium-dependent protein kinase CDPK4
TGME49_237890	p.Ala337Ser	missense_variant	calcium-dependent protein kinase CDPK4
TGME49_237890	p.Ile399Asn	missense_variant	calcium-dependent protein kinase CDPK4
TGME49_218720	p.Leu89Arg	missense_variant	calcium-dependent protein kinase CDPK6
TGME49_218720	p.Lys308Arg	missense_variant	calcium-dependent protein kinase CDPK6
TGME49_218720	p.Val358Ala	missense_variant	calcium-dependent protein kinase CDPK6
TGME49_218720	p.Asn620Thr	missense_variant	calcium-dependent protein kinase CDPK6
TGME49_218720	p.Cys688Ser	missense_variant	calcium-dependent protein kinase CDPK6
TGME49_218720	p.Ala829Thr	missense_variant	calcium-dependent protein kinase CDPK6
TGME49_246930	p.Ala137Thr	missense_variant	calmodulin CAM1
TGME49_253940	p.Glu551Asp	missense_variant	CAM Kinase family, incomplete catalytic triad
TGME49_253940	p.Ala539Val	missense_variant	CAM Kinase family, incomplete catalytic triad
TGME49_253940	p.Val515Leu	missense_variant	CAM Kinase family, incomplete catalytic triad
TGME49_253940	p.Phe468Leu	missense_variant	CAM Kinase family, incomplete catalytic triad
TGME49_253940	c.-1568C>T	5_prime_UTR_premature_start_codon_gain_variant	CAM Kinase family, incomplete catalytic triad
TGME49_253940	c.-1924C>G	5_prime_UTR_premature_start_codon_gain_variant	CAM Kinase family, incomplete catalytic triad
TGME49_253940	c.-2057C>G	5_prime_UTR_premature_start_codon_gain_variant	CAM Kinase family, incomplete catalytic triad
TGME49_253940	c.-2125C>G	5_prime_UTR_premature_start_codon_gain_variant	CAM Kinase family, incomplete catalytic triad
TGME49_253940	c.-2721G>T	5_prime_UTR_premature_start_codon_gain_variant	CAM Kinase family, incomplete catalytic triad
TGME49_253940	c.-2738G>A	5_prime_UTR_premature_start_codon_gain_variant	CAM Kinase family, incomplete catalytic triad
TGME49_253940	c.-3378C>G	5_prime_UTR_premature_start_codon_gain_variant	CAM Kinase family, incomplete catalytic triad
TGME49_215670	p.Arg1519Thr	missense_variant	cAMP-dependent protein kinase
TGME49_215670	p.Ile1449Thr	missense_variant	cAMP-dependent protein kinase
TGME49_215670	p.Glu1441Gly	missense_variant	cAMP-dependent protein kinase
TGME49_215670	p.Pro1175Leu	missense_variant	cAMP-dependent protein kinase
TGME49_215670	p.Gly900Asp	missense_variant	cAMP-dependent protein kinase
TGME49_215670	p.Ser826Phe	missense_variant	cAMP-dependent protein kinase
TGME49_215670	p.Glu423Gly	missense_variant	cAMP-dependent protein kinase
TGME49_215670	p.Thr372Ser	missense_variant	cAMP-dependent protein kinase
TGME49_215260	c.4122-7C>T	splice_region_variant	carbamoylphosphate synthetase
TGME49_215260	c.3398-6C>T	splice_region_variant	carbamoylphosphate synthetase
TGME49_215260	p.Ser1074*	stop_gained	carbamoylphosphate synthetase
TGME49_215260	c.2401+5C>A	splice_region_variant	carbamoylphosphate synthetase
TGME49_215260	c.1705-4T>G	splice_region_variant	carbamoylphosphate synthetase
TGME49_215260	c.1705-5T>C	splice_region_variant	carbamoylphosphate synthetase
TGME49_215260	c.1138+4A>G	splice_region_variant	carbamoylphosphate synthetase
TGME49_215260	p.Asn208Lys	missense_variant	carbamoylphosphate synthetase
TGME49_215260	p.Arg19Gln	missense_variant	carbamoylphosphate synthetase
TGME49_239080	p.Pro207Leu	missense_variant	carrier superfamily protein
TGME49_239080	p.Asn427Ser	missense_variant	carrier superfamily protein
TGME49_239080	p.Gly489Glu	missense_variant	carrier superfamily protein
TGME49_239080	p.Pro530Leu	missense_variant	carrier superfamily protein
TGME49_218770	p.Arg448Gly	missense_variant	carrier superfamily protein
TGME49_218770	p.Ser418Leu	missense_variant	carrier superfamily protein
TGME49_218770	p.Ala254Thr	missense_variant	carrier superfamily protein
TGME49_218770	p.Ala186Val	missense_variant	carrier superfamily protein
TGME49_218770	p.Ser125Pro	missense_variant	carrier superfamily protein
TGME49_218770	p.Ser98Cys	missense_variant	carrier superfamily protein
TGME49_218770	p.Gly4Glu	missense_variant	carrier superfamily protein
TGME49_248950	p.Ala200Pro	missense_variant	carrier superfamily protein
TGME49_247710	c.-1003T>G	5_prime_UTR_premature_start_codon_gain_variant	casein kinase I
TGME49_247710	c.-846T>G	5_prime_UTR_premature_start_codon_gain_variant	casein kinase I
TGME49_247710	p.Leu446Val	missense_variant	casein kinase I

TGME49_249670	p.Glu271Lys	missense_variant	cathepsin B
TGME49_249670	p.Glu94Gln	missense_variant	cathepsin B
TGME49_224190	c.-156C>G	5_prime_UTR_premature_start_codon_gain_variant	cation-transporting atpase family protein
TGME49_224190	c.-703C>T	5_prime_UTR_premature_start_codon_gain_variant	cation-transporting atpase family protein
TGME49_219260	c.-833A>G	5_prime_UTR_premature_start_codon_gain_variant	cation-transporting ATPase, putative
TGME49_219260	p.Pro216Leu	missense_variant	cation-transporting ATPase, putative
TGME49_219260	p.Ala852Thr	missense_variant	cation-transporting ATPase, putative
TGME49_219260	p.Ser1529Ala	missense_variant	cation-transporting ATPase, putative
TGME49_219260	p.Lys1696Glu	missense_variant	cation-transporting ATPase, putative
TGME49_219260	p.Ala2709Ser	missense_variant	cation-transporting ATPase, putative
TGME49_219260	p.Trp2710Cys	missense_variant	cation-transporting ATPase, putative
TGME49_219260	p.Gly2733Arg	missense_variant	cation-transporting ATPase, putative
TGME49_219260	p.Gln2786Arg	missense_variant	cation-transporting ATPase, putative
TGME49_219260	p.Arg2790Gln	missense_variant	cation-transporting ATPase, putative
TGME49_219260	p.Pro2849Leu	missense_variant	cation-transporting ATPase, putative
TGME49_319658	c.-81C>T	5_prime_UTR_premature_start_codon_gain_variant	CBF/Mak21 family protein
TGME49_319658	p.Ala199Pro	missense_variant	CBF/Mak21 family protein
TGME49_319658	p.Gly216Val	missense_variant	CBF/Mak21 family protein
TGME49_319658	p.Pro409Ala	missense_variant	CBF/Mak21 family protein
TGME49_319658	p.Ser532Thr	missense_variant	CBF/Mak21 family protein
TGME49_319658	p.Ala535Val	missense_variant	CBF/Mak21 family protein
TGME49_211350	c.-1617C>G	5_prime_UTR_premature_start_codon_gain_variant	CBS domain-containing protein
TGME49_211350	p.Lys490Arg	missense_variant	CBS domain-containing protein
TGME49_211350	p.Ser790Ala	missense_variant	CBS domain-containing protein
TGME49_211350	p.Pro793Ser	missense_variant	CBS domain-containing protein
TGME49_211350	p.Asp840Glu	missense_variant	CBS domain-containing protein
TGME49_211350	p.Arg973Leu	missense_variant	CBS domain-containing protein
TGME49_211350	p.Ser1168Thr	missense_variant	CBS domain-containing protein
TGME49_254540	p.Phe962Ile	missense_variant	CDP-alcohol phosphatidyltransferase superfamily protein
TGME49_254540	p.Asn923Ser	missense_variant	CDP-alcohol phosphatidyltransferase superfamily protein
TGME49_254540	p.Ser910Phe	missense_variant	CDP-alcohol phosphatidyltransferase superfamily protein
TGME49_254540	p.His495Asp	missense_variant	CDP-alcohol phosphatidyltransferase superfamily protein
TGME49_254540	p.Ala298Pro	missense_variant	CDP-alcohol phosphatidyltransferase superfamily protein
TGME49_254540	p.Ser248Thr	missense_variant	CDP-alcohol phosphatidyltransferase superfamily protein
TGME49_254540	p.Lys235Asn	missense_variant	CDP-alcohol phosphatidyltransferase superfamily protein
TGME49_254540	p.Leu49Phe	missense_variant	CDP-alcohol phosphatidyltransferase superfamily protein
TGME49_221490	c.-349C>T	5_prime_UTR_premature_start_codon_gain_variant	cell cycle regulator protein
TGME49_249260	c.*1147C>A	splice_region_variant	cell-cycle-associated protein kinase CDK, putative
TGME49_249260	p.Gln1312His	missense_variant	cell-cycle-associated protein kinase CDK, putative
TGME49_249260	p.Asn1187Ser	missense_variant	cell-cycle-associated protein kinase CDK, putative
TGME49_249260	p.Leu286Ile	missense_variant	cell-cycle-associated protein kinase CDK, putative
TGME49_249260	p.Val125Ile	missense_variant	cell-cycle-associated protein kinase CDK, putative
TGME49_249260	p.Pro38Thr	missense_variant	cell-cycle-associated protein kinase CDK, putative
TGME49_224480	c.4441-6C>T	splice_region_variant	cell-cycle-associated protein kinase CLK, putative
TGME49_224480	p.Leu1158Phe	missense_variant	cell-cycle-associated protein kinase CLK, putative
TGME49_224480	p.Ser787Ala	missense_variant	cell-cycle-associated protein kinase CLK, putative
TGME49_224480	p.Arg456His	missense_variant	cell-cycle-associated protein kinase CLK, putative
TGME49_224480	p.Arg311His	missense_variant	cell-cycle-associated protein kinase CLK, putative
TGME49_224480	p.Pro73Leu	missense_variant	cell-cycle-associated protein kinase CLK, putative
TGME49_224480	p.Pro73Ala	missense_variant	cell-cycle-associated protein kinase CLK, putative
TGME49_224480	c.-1475G>T	5_prime_UTR_premature_start_codon_gain_variant	cell-cycle-associated protein kinase CLK, putative
TGME49_253440	c.2502-6G>A	splice_region_variant	cell-cycle-associated protein kinase SRPK, putative
TGME49_253440	c.2634+4G>T	splice_region_variant	cell-cycle-associated protein kinase SRPK, putative
TGME49_253440	c.2634+5G>T	splice_region_variant	cell-cycle-associated protein kinase SRPK, putative
TGME49_253440	p.Ser1312Asn	missense_variant	cell-cycle-associated protein kinase SRPK, putative
TGME49_253440	p.Leu1506Ile	missense_variant	cell-cycle-associated protein kinase SRPK, putative
TGME49_320430	p.His251Arg	missense_variant	cell-cycle-control protein (translation regulation), putative
TGME49_320430	p.Gly304Glu	missense_variant	cell-cycle-control protein (translation regulation), putative
TGME49_320430	p.Pro337His	missense_variant	cell-cycle-control protein (translation regulation), putative
TGME49_320430	c.2021+8A>G	splice_region_variant	cell-cycle-control protein (translation regulation), putative
TGME49_250340	c.286-6T>C	splice_region_variant	centrin 2
TGME49_250340	c.-357C>G	5_prime_UTR_premature_start_codon_gain_variant	centrin 2
TGME49_250340	c.-652A>G	5_prime_UTR_premature_start_codon_gain_variant	centrin 2
TGME49_237490	c.-326T>C	5_prime_UTR_premature_start_codon_gain_variant	centrin family protein
TGME49_221420	p.Pro904Ser	missense_variant	Chromosome-associated kinesin KLP1, putative
TGME49_218210	p.Lys625Arg	missense_variant	Chromosome-associated kinesin KLP1, putative
TGME49_221260	p.Ser15Thr	missense_variant	Class-II DAHP synthetase family protein
TGME49_221260	p.Cys92Gly	missense_variant	Class-II DAHP synthetase family protein
TGME49_221260	c.1134+8C>T	splice_region_variant	Class-II DAHP synthetase family protein
TGME49_275690	c.-383C>A	5_prime_UTR_premature_start_codon_gain_variant	ClpB, putative
TGME49_253580	p.Cys771Arg	missense_variant	CMGC kinase, CDK family
TGME49_253580	p.Ala1208Thr	missense_variant	CMGC kinase, CDK family
TGME49_253580	p.His1213Gln	missense_variant	CMGC kinase, CDK family
TGME49_250850	p.Met2454Val	missense_variant	CMGC kinase, putative
TGME49_250850	p.Ser2353Gly	missense_variant	CMGC kinase, putative
TGME49_250850	p.Ser2352Leu	missense_variant	CMGC kinase, putative
TGME49_250850	p.Cys1952Arg	missense_variant	CMGC kinase, putative
TGME49_250850	p.Arg1712Pro	missense_variant	CMGC kinase, putative
TGME49_250850	p.Ser516Thr	missense_variant	CMGC kinase, putative
TGME49_235020	p.Glu1122Asp	missense_variant	COPI protein, putative
TGME49_235402	p.Pro190Ala	missense_variant	CorA family Mg2+ transporter protein
TGME49_235402	c.1060-6T>C	splice_region_variant	CorA family Mg2+ transporter protein
TGME49_235402	p.Leu648Phe	missense_variant	CorA family Mg2+ transporter protein
TGME49_235402	p.Pro1178Leu	missense_variant	CorA family Mg2+ transporter protein
TGME49_254260	c.-93T>C	5_prime_UTR_premature_start_codon_gain_variant	COX19 cytochrome c oxidase assembly family protein

TGME49_224280	p.Pro1944Ser	missense_variant	CPSF A subunit region protein
TGME49_224280	p.His1937Pro	missense_variant	CPSF A subunit region protein
TGME49_224280	p.Tyr1936Cys	missense_variant	CPSF A subunit region protein
TGME49_224280	p.Gly1933Ser	missense_variant	CPSF A subunit region protein
TGME49_224280	p.Ser1780Asn	missense_variant	CPSF A subunit region protein
TGME49_224280	p.Gly1592Asp	missense_variant	CPSF A subunit region protein
TGME49_224280	p.Glu1584Lys	missense_variant	CPSF A subunit region protein
TGME49_224280	p.Ile856Val	missense_variant	CPSF A subunit region protein
TGME49_224280	p.Leu537Arg	missense_variant	CPSF A subunit region protein
TGME49_224280	p.Leu450Met	missense_variant	CPSF A subunit region protein
TGME49_224280	p.Arg322Cys	missense_variant	CPSF A subunit region protein
TGME49_318500	c.914-6A>T	splice_region_variant	cpw-wpc domain-containing protein
TGME49_318500	p.Pro106Ser	missense_variant	cpw-wpc domain-containing protein
TGME49_247310	p.Thr179Ala	missense_variant	cpw-wpc domain-containing protein
TGME49_254390	p.His436Arg	missense_variant	CRAL/TRIO domain-containing protein
TGME49_254390	c.-2973C>T	5_prime_UTR_premature_start_codon_gain_variant	CRAL/TRIO domain-containing protein
TGME49_246330	p.Ala70Thr	missense_variant	CRAL/TRIO domain-containing protein
TGME49_246330	p.Thr63Ser	missense_variant	CRAL/TRIO domain-containing protein
TGME49_221350	c.-636C>G	5_prime_UTR_premature_start_codon_gain_variant	Ctr copper transporter family protein
TGME49_221350	p.Leu30Met	missense_variant	Ctr copper transporter family protein
TGME49_221350	p.Asp129Ala	missense_variant	Ctr copper transporter family protein
TGME49_221350	p.Pro173Ser	missense_variant	Ctr copper transporter family protein
TGME49_221200	p.Gly2613Glu	missense_variant	CW-type Zinc Finger protein
TGME49_221200	p.Val2214Ile	missense_variant	CW-type Zinc Finger protein
TGME49_221200	p.Val2127Gly	missense_variant	CW-type Zinc Finger protein
TGME49_221200	p.Ala2070Thr	missense_variant	CW-type Zinc Finger protein
TGME49_221200	p.Thr756Ala	missense_variant	CW-type Zinc Finger protein
TGME49_221200	p.Glu506Asp	missense_variant	CW-type Zinc Finger protein
TGME49_235350	p.Leu531Ile	missense_variant	cwfj family protein
TGME49_219070	p.Ser2654Asn	missense_variant	cyclic nucleotide-binding domain-containing protein
TGME49_219070	c.6760+4C>A	splice_region_variant	cyclic nucleotide-binding domain-containing protein
TGME49_219070	p.Asn1731Ser	missense_variant	cyclic nucleotide-binding domain-containing protein
TGME49_219070	p.Thr957Ala	missense_variant	cyclic nucleotide-binding domain-containing protein
TGME49_219070	p.Glu933Lys	missense_variant	cyclic nucleotide-binding domain-containing protein
TGME49_219070	p.Asn932His	missense_variant	cyclic nucleotide-binding domain-containing protein
TGME49_219070	p.Met6Val	missense_variant	cyclic nucleotide-binding domain-containing protein
TGME49_239910	c.-3109T>G	5_prime_UTR_premature_start_codon_gain_variant	cyclin-dependent kinase
TGME49_239910	c.-1282T>C	5_prime_UTR_premature_start_codon_gain_variant	cyclin-dependent kinase
TGME49_239910	p.Gln824His	missense_variant	cyclin-dependent kinase
TGME49_239910	p.Gln999Glu	missense_variant	cyclin-dependent kinase
TGME49_239910	p.Val1103Leu	missense_variant	cyclin-dependent kinase
TGME49_239910	p.Ser1122Gly	missense_variant	cyclin-dependent kinase
TGME49_239910	p.Pro1815Leu	missense_variant	cyclin-dependent kinase
TGME49_239910	p.Val1974Ala	missense_variant	cyclin-dependent kinase
TGME49_239910	p.Gln2287His	missense_variant	cyclin-dependent kinase
TGME49_219832	p.Glu1005Gly	missense_variant	cyclin-dependent kinase regulatory subunit protein
TGME49_219100	p.Ser182Asn	missense_variant	cyclin-dependent kinase regulatory subunit protein
TGME49_300350	c.953-3C>T	splice_region_variant	cysteine desulfurase/selenocysteine lyase family PLP dependent transferase superfamily protein
TGME49_299810	p.Thr898Pro	missense_variant	cysteine-tRNA synthetase (CysRS)
TGME49_299810	p.Lys772Arg	missense_variant	cysteine-tRNA synthetase (CysRS)
TGME49_239630	c.-304T>C	5_prime_UTR_premature_start_codon_gain_variant	cytidine and deoxycytidylate deaminase zinc-binding region domain-containing protein
TGME49_239630	c.-89T>C	5_prime_UTR_premature_start_codon_gain_variant	cytidine and deoxycytidylate deaminase zinc-binding region domain-containing protein
TGME49_239630	p.Leu211His	missense_variant	cytidine and deoxycytidylate deaminase zinc-binding region domain-containing protein
TGME49_239630	p.Gly235Ala	missense_variant	cytidine and deoxycytidylate deaminase zinc-binding region domain-containing protein
TGME49_215750	p.Pro470Ser	missense_variant	cytidine and deoxycytidylate deaminase zinc-binding region domain-containing protein
TGME49_277510	c.863+4G>A	splice_region_variant	cytoplasmic dynein intermediate chain
TGME49_277510	c.-75C>T	5_prime_UTR_premature_start_codon_gain_variant	cytoplasmic dynein intermediate chain
TGME49_277510	c.-722C>T	5_prime_UTR_premature_start_codon_gain_variant	cytoplasmic dynein intermediate chain
TGME49_277510	c.-1053T>C	5_prime_UTR_premature_start_codon_gain_variant	cytoplasmic dynein intermediate chain
TGME49_277510	c.-2373T>A	5_prime_UTR_premature_start_codon_gain_variant	cytoplasmic dynein intermediate chain
TGME49_280730	c.716-7G>A	splice_region_variant	cytosolic fe-s cluster assembling factor nbp35, putative
TGME49_219540	p.Ala1265Val	missense_variant	cytosolic tRNA-Ala synthetase
TGME49_219540	c.3576+8G>A	splice_region_variant	cytosolic tRNA-Ala synthetase
TGME49_219540	p.Leu1142Val	missense_variant&splice_region_variant	cytosolic tRNA-Ala synthetase
TGME49_219540	c.3267+8A>T	splice_region_variant	cytosolic tRNA-Ala synthetase
TGME49_219540	c.3267+6A>G	splice_region_variant	cytosolic tRNA-Ala synthetase
TGME49_219540	p.Arg878Lys	missense_variant	cytosolic tRNA-Ala synthetase
TGME49_219540	p.Val772Leu	missense_variant	cytosolic tRNA-Ala synthetase
TGME49_219540	p.Pro224Leu	missense_variant	cytosolic tRNA-Ala synthetase
TGME49_219540	p.Leu186Phe	missense_variant	cytosolic tRNA-Ala synthetase
TGME49_219540	p.Phe151Ser	missense_variant	cytosolic tRNA-Ala synthetase
TGME49_219540	p.Ser94Ala	missense_variant	cytosolic tRNA-Ala synthetase
TGME49_239820	c.-700C>T	5_prime_UTR_premature_start_codon_gain_variant	D-3-phosphoglycerate dehydrogenase
TGME49_236650	c.-201C>T	5_prime_UTR_premature_start_codon_gain_variant	DEAD (Asp-Glu-Ala-Asp) box polypeptide 17
TGME49_236650	c.-1887C>G	5_prime_UTR_premature_start_codon_gain_variant	DEAD (Asp-Glu-Ala-Asp) box polypeptide 17
TGME49_294350	p.Arg2258Leu	missense_variant	DEAD/DEAH box helicase domain-containing protein
TGME49_221660	c.-185C>G	5_prime_UTR_premature_start_codon_gain_variant	DEAD/DEAH box helicase domain-containing protein
TGME49_221660	p.Ser366Thr	missense_variant	DEAD/DEAH box helicase domain-containing protein
TGME49_221660	p.Pro774Ser	missense_variant	DEAD/DEAH box helicase domain-containing protein
TGME49_222140	p.Arg885Lys	missense_variant	DEAD/DEAH box helicase domain-containing protein
TGME49_222140	p.Ala363Thr	missense_variant	DEAD/DEAH box helicase domain-containing protein
TGME49_222140	p.Glu341Lys	missense_variant	DEAD/DEAH box helicase domain-containing protein
TGME49_222140	p.Pro134His	missense_variant	DEAD/DEAH box helicase domain-containing protein
TGME49_222140	p.Pro44Leu	missense_variant	DEAD/DEAH box helicase domain-containing protein

TGME49_222140	p.His10Asn	missense_variant	DEAD/DEAH box helicase domain-containing protein
TGME49_253090	c.-3075C>T	5_prime_UTR_premature_start_codon_gain_variant	DEAD/DEAH box helicase domain-containing protein
TGME49_253090	c.-3044T>C	5_prime_UTR_premature_start_codon_gain_variant	DEAD/DEAH box helicase domain-containing protein
TGME49_253090	p.Ala213Pro	missense_variant	DEAD/DEAH box helicase domain-containing protein
TGME49_253090	p.Phe260Leu	missense_variant	DEAD/DEAH box helicase domain-containing protein
TGME49_253090	p.Arg269Ile	missense_variant	DEAD/DEAH box helicase domain-containing protein
TGME49_253090	p.Pro287Leu	missense_variant	DEAD/DEAH box helicase domain-containing protein
TGME49_253090	p.Leu443His	missense_variant	DEAD/DEAH box helicase domain-containing protein
TGME49_253090	p.Ser526Ala	missense_variant	DEAD/DEAH box helicase domain-containing protein
TGME49_211400	p.Lys812Glu	missense_variant	DEAD/DEAH box helicase domain-containing protein
TGME49_211400	p.Gly784Arg	missense_variant	DEAD/DEAH box helicase domain-containing protein
TGME49_211400	p.Lys770Asn	missense_variant	DEAD/DEAH box helicase domain-containing protein
TGME49_211400	c.2081-6G>A	splice_region_variant	DEAD/DEAH box helicase domain-containing protein
TGME49_211400	c.2080+6C>A	splice_region_variant	DEAD/DEAH box helicase domain-containing protein
TGME49_211400	p.Ser590Asn	missense_variant	DEAD/DEAH box helicase domain-containing protein
TGME49_211400	p.Ala526Pro	missense_variant	DEAD/DEAH box helicase domain-containing protein
TGME49_211400	p.Thr460Pro	missense_variant	DEAD/DEAH box helicase domain-containing protein
TGME49_211400	p.Asp175Val	missense_variant	DEAD/DEAH box helicase domain-containing protein
TGME49_223440	p.Cys1429Arg	missense_variant	DEAD/DEAH box helicase domain-containing protein
TGME49_223440	p.Ala1251Thr	missense_variant	DEAD/DEAH box helicase domain-containing protein
TGME49_223440	p.Ala1243Gly	missense_variant	DEAD/DEAH box helicase domain-containing protein
TGME49_223440	p.Glu1236Asp	missense_variant	DEAD/DEAH box helicase domain-containing protein
TGME49_223440	p.His682Pro	missense_variant	DEAD/DEAH box helicase domain-containing protein
TGME49_223440	p.Gly528Ser	missense_variant	DEAD/DEAH box helicase domain-containing protein
TGME49_223440	p.Glu513Lys	missense_variant	DEAD/DEAH box helicase domain-containing protein
TGME49_223440	p.Ser217Cys	missense_variant	DEAD/DEAH box helicase domain-containing protein
TGME49_223440	p.Leu214Phe	missense_variant	DEAD/DEAH box helicase domain-containing protein
TGME49_223440	p.Pro151Thr	missense_variant	DEAD/DEAH box helicase domain-containing protein
TGME49_223440	p.Pro38His	missense_variant	DEAD/DEAH box helicase domain-containing protein
TGME49_223440	c.-153T>C	5_prime_UTR_premature_start_codon_gain_variant	DEAD/DEAH box helicase domain-containing protein
TGME49_251480	p.Gly1051Ala	missense_variant	DEAD/DEAH box helicase domain-containing protein
TGME49_251480	p.Ser1046Ala	missense_variant	DEAD/DEAH box helicase domain-containing protein
TGME49_251480	p.Ser459Ala	missense_variant	DEAD/DEAH box helicase domain-containing protein
TGME49_251480	p.Ala186Asp	missense_variant	DEAD/DEAH box helicase domain-containing protein
TGME49_251480	c.-348C>G	5_prime_UTR_premature_start_codon_gain_variant	DEAD/DEAH box helicase domain-containing protein
TGME49_219200	c.-392G>T	5_prime_UTR_premature_start_codon_gain_variant	DEAD/DEAH box RNA helicase family protein
TGME49_219200	c.712-4G>T	splice_region_variant	DEAD/DEAH box RNA helicase family protein
TGME49_219200	p.Ser818Gly	missense_variant	DEAD/DEAH box RNA helicase family protein
TGME49_212410	p.Arg4Cys	missense_variant	dense granule protein GRA11
TGME49_212410	p.Thr99Ser	missense_variant	dense granule protein GRA11
TGME49_212410	p.Thr106Ala	missense_variant	dense granule protein GRA11
TGME49_212410	p.Arg108Lys	missense_variant	dense granule protein GRA11
TGME49_237800	p.Gln70His	missense_variant	dense granule protein GRA11
TGME49_237800	p.Asn74Ser	missense_variant	dense granule protein GRA11
TGME49_237800	p.Val101Met	missense_variant	dense granule protein GRA11
TGME49_237800	p.Val101Ala	missense_variant	dense granule protein GRA11
TGME49_237800	p.Ala182Thr	missense_variant	dense granule protein GRA11
TGME49_237800	p.Leu386Phe	missense_variant	dense granule protein GRA11
TGME49_237800	p.Pro389Ala	missense_variant	dense granule protein GRA11
TGME49_275440	p.Val49Phe	missense_variant	dense granule protein GRA6
TGME49_275440	c.-777A>T	5_prime_UTR_premature_start_codon_gain_variant	dense granule protein GRA6
TGME49_254720	p.Ser33Gly	missense_variant	dense granule protein GRA8
TGME49_254720	p.Asn44Ile	missense_variant	dense granule protein GRA8
TGME49_254720	p.Pro93Leu	missense_variant	dense granule protein GRA8
TGME49_254720	p.Gln116His	missense_variant	dense granule protein GRA8
TGME49_254720	p.Phe255Leu	missense_variant	dense granule protein GRA8
TGME49_222170	p.Ala44Val	missense_variant	dense-granule antigen DG32
TGME49_318750	p.Asn212Lys	missense_variant	deoxyribose-phosphate aldolase
TGME49_294290	p.Pro15Ser	missense_variant	Der1ER1
TGME49_301370	p.Gly265Glu	missense_variant	DHHC zinc finger domain-containing protein
TGME49_301370	p.Ile203Phe	missense_variant	DHHC zinc finger domain-containing protein
TGME49_224310	c.-822T>C	5_prime_UTR_premature_start_codon_gain_variant	DHHC zinc finger domain-containing protein
TGME49_224310	p.Pro52Leu	missense_variant	DHHC zinc finger domain-containing protein
TGME49_224310	p.Leu55Val	missense_variant	DHHC zinc finger domain-containing protein
TGME49_224310	p.Ser236Arg	missense_variant	DHHC zinc finger domain-containing protein
TGME49_224310	p.Ser345Cys	missense_variant	DHHC zinc finger domain-containing protein
TGME49_224290	c.290+8G>A	splice_region_variant	DHHC zinc finger domain-containing protein
TGME49_246650	p.Arg374Ser	missense_variant	DHHC zinc finger domain-containing protein
TGME49_249380	p.Ser43Ala	missense_variant	DHHC zinc finger domain-containing protein
TGME49_250870	p.Leu321Phe	missense_variant	DHHC zinc finger domain-containing protein
TGME49_278850	c.-1281A>G	5_prime_UTR_premature_start_codon_gain_variant	DHHC zinc finger domain-containing protein
TGME49_275600	p.Ser49Asn	missense_variant	diacylglycerol acyltransferase
TGME49_239250	p.Thr1708Met	missense_variant	diacylglycerol kinase, putative
TGME49_239250	p.Met1698Ile	missense_variant	diacylglycerol kinase, putative
TGME49_239250	p.Pro1667Leu	missense_variant	diacylglycerol kinase, putative
TGME49_239250	p.Ile1429Met	missense_variant	diacylglycerol kinase, putative
TGME49_239250	p.Leu1315Pro	missense_variant	diacylglycerol kinase, putative
TGME49_239250	c.3271+4A>G	splice_region_variant	diacylglycerol kinase, putative
TGME49_239250	p.Val454Ile	missense_variant	diacylglycerol kinase, putative
TGME49_239250	p.Ala11Thr	missense_variant	diacylglycerol kinase, putative
TGME49_239250	c.-577T>C	5_prime_UTR_premature_start_codon_gain_variant	diacylglycerol kinase, putative
TGME49_239250	c.-1365C>T	5_prime_UTR_premature_start_codon_gain_variant	diacylglycerol kinase, putative
TGME49_278740	c.-396T>C	5_prime_UTR_premature_start_codon_gain_variant	diaminopimelate decarboxylase
TGME49_278740	c.-1066A>G	5_prime_UTR_premature_start_codon_gain_variant	diaminopimelate decarboxylase
TGME49_278740	c.-1080C>T	5_prime_UTR_premature_start_codon_gain_variant	diaminopimelate decarboxylase

TGME49_219550	p.Ala184Thr	missense_variant	dihydrolipoyllysine-residue succinyltransferase component of oxoglutarate dehydrogenase
TGME49_219550	p.Lys41Glu	missense_variant	dihydrolipoyllysine-residue succinyltransferase component of oxoglutarate dehydrogenase
TGME49_219550	c.-72C>G	5_prime_UTR_premature_start_codon_gain_variant	dihydrolipoyllysine-residue succinyltransferase component of oxoglutarate dehydrogenase
TGME49_210790	p.Ser20Cys	missense_variant	dihydroorotate dehydrogenase reveal, putative
TGME49_280780	p.Ala196Ser	missense_variant	dihydrouridine synthase (dus) protein
TGME49_280780	p.Gly540Asp	missense_variant	dihydrouridine synthase (dus) protein
TGME49_300320	p.Glu429Lys	missense_variant	dimethyladenosine transferase
TGME49_245500	p.Thr306Ala	missense_variant	dipeptidyl peptidase iv (dpp iv) n-terminal region domain-containing protein
TGME49_245500	p.Thr140Ala	missense_variant	dipeptidyl peptidase iv (dpp iv) n-terminal region domain-containing protein
TGME49_224910	c.-371G>A	5_prime_UTR_premature_start_codon_gain_variant	divalent cation tolerance protein, CutA1 family protein
TGME49_224910	c.498+7G>C	splice_region_variant	divalent cation tolerance protein, CutA1 family protein
TGME49_221330	p.Thr1146Met	missense_variant	DNA gyrase/topoisomerase IV, A subunit domain-containing protein
TGME49_221330	p.Ser1042Pro	missense_variant	DNA gyrase/topoisomerase IV, A subunit domain-containing protein
TGME49_221330	p.Asp1021Gly	missense_variant	DNA gyrase/topoisomerase IV, A subunit domain-containing protein
TGME49_221330	p.Lys893Gln	missense_variant	DNA gyrase/topoisomerase IV, A subunit domain-containing protein
TGME49_221330	p.Ala543Thr	missense_variant	DNA gyrase/topoisomerase IV, A subunit domain-containing protein
TGME49_221330	p.Arg263His	missense_variant	DNA gyrase/topoisomerase IV, A subunit domain-containing protein
TGME49_221330	p.Gly247Ala	missense_variant	DNA gyrase/topoisomerase IV, A subunit domain-containing protein
TGME49_221330	p.Ala206Val	missense_variant	DNA gyrase/topoisomerase IV, A subunit domain-containing protein
TGME49_221330	p.Leu194Pro	missense_variant	DNA gyrase/topoisomerase IV, A subunit domain-containing protein
TGME49_236200	p.Glu1485Gly	missense_variant	DNA mismatch repair protein, C-terminal domain-containing protein
TGME49_236200	p.Glu1304Lys	missense_variant	DNA mismatch repair protein, C-terminal domain-containing protein
TGME49_236200	p.Ala1219Glu	missense_variant	DNA mismatch repair protein, C-terminal domain-containing protein
TGME49_236200	p.Asp1110Gly	missense_variant	DNA mismatch repair protein, C-terminal domain-containing protein
TGME49_236200	p.Val1105Gly	missense_variant	DNA mismatch repair protein, C-terminal domain-containing protein
TGME49_236200	p.Ser919Arg	missense_variant	DNA mismatch repair protein, C-terminal domain-containing protein
TGME49_236200	p.Leu696Met	missense_variant	DNA mismatch repair protein, C-terminal domain-containing protein
TGME49_236200	p.Ser154Pro	missense_variant	DNA mismatch repair protein, C-terminal domain-containing protein
TGME49_217910	p.Ser200Gly	missense_variant	DNA polymerase (pol2) superfamily protein
TGME49_280690	p.Arg531Lys	missense_variant	DNA polymerase epsilon subunit B protein
TGME49_319860	p.Ala2972Ser	missense_variant	DNA polymerase family B protein
TGME49_319860	p.Pro2295Thr	missense_variant	DNA polymerase family B protein
TGME49_319860	p.Gly2190Asp	missense_variant	DNA polymerase family B protein
TGME49_319860	c.5325+6G>A	splice_region_variant	DNA polymerase family B protein
TGME49_319860	p.Lys1604Arg	missense_variant	DNA polymerase family B protein
TGME49_319860	p.Asp822Asn	missense_variant	DNA polymerase family B protein
TGME49_237830	p.Arg364Lys	missense_variant	DNA polymerase I domain-containing protein
TGME49_237830	p.Ser1176Thr	missense_variant	DNA polymerase I domain-containing protein
TGME49_237830	p.Gly1355Cys	missense_variant	DNA polymerase I domain-containing protein
TGME49_237830	c.4407+7G>A	splice_region_variant	DNA polymerase I domain-containing protein
TGME49_297840	c.620-6T>G	splice_region_variant	DNA primase, large subunit
TGME49_297840	p.Thr119Ala	missense_variant	DNA primase, large subunit
TGME49_297840	p.Met112Thr	missense_variant	DNA primase, large subunit
TGME49_297840	c.-333C>G	5_prime_UTR_premature_start_codon_gain_variant	DNA primase, large subunit
TGME49_301222	p.Leu15Ile	missense_variant	DNA repair protein Rad4 domain-containing protein
TGME49_301222	p.Glu90Gly	missense_variant	DNA repair protein Rad4 domain-containing protein
TGME49_301222	p.Ser123Pro	missense_variant	DNA repair protein Rad4 domain-containing protein
TGME49_301222	p.Glu346Gln	missense_variant	DNA repair protein Rad4 domain-containing protein
TGME49_301222	p.Gly446Ser	missense_variant	DNA repair protein Rad4 domain-containing protein
TGME49_301222	p.Ser799Gly	missense_variant	DNA repair protein Rad4 domain-containing protein
TGME49_301222	p.Pro1107Ser	missense_variant	DNA repair protein Rad4 domain-containing protein
TGME49_301222	p.Leu1230Pro	missense_variant	DNA repair protein Rad4 domain-containing protein
TGME49_301222	p.Ser1304Tyr	missense_variant	DNA repair protein Rad4 domain-containing protein
TGME49_301222	p.Asn1379Ser	missense_variant	DNA repair protein Rad4 domain-containing protein
TGME49_219700	c.-11T>G	5_prime_UTR_premature_start_codon_gain_variant	DNA replication licensing factor MCM4, putative
TGME49_219700	c.1088-4G>C	splice_region_variant	DNA replication licensing factor MCM4, putative
TGME49_277530	p.Pro77Leu	missense_variant	DNA topoisomerase domain-containing protein
TGME49_277530	p.Lys322Thr	missense_variant	DNA topoisomerase domain-containing protein
TGME49_277530	p.Asp323His	missense_variant	DNA topoisomerase domain-containing protein
TGME49_277530	p.Lys431Thr	missense_variant	DNA topoisomerase domain-containing protein
TGME49_277530	p.Gln432Arg	missense_variant	DNA topoisomerase domain-containing protein
TGME49_277530	p.Ala475Thr	missense_variant	DNA topoisomerase domain-containing protein
TGME49_277530	p.Gln616Lys	missense_variant	DNA topoisomerase domain-containing protein
TGME49_277530	p.Ser997Cys	missense_variant	DNA topoisomerase domain-containing protein
TGME49_277530	p.Glu1077Gly	missense_variant	DNA topoisomerase domain-containing protein
TGME49_277530	p.Glu1082Asp	missense_variant	DNA topoisomerase domain-containing protein
TGME49_234360	p.Leu106Gln	missense_variant	DNA topoisomerase I, putative
TGME49_234360	p.Ala671Glu	missense_variant	DNA topoisomerase I, putative
TGME49_246060	p.Leu9Phe	missense_variant	DNA-dependent RNA polymerase
TGME49_246060	p.Ala343Gly	missense_variant	DNA-dependent RNA polymerase
TGME49_246060	p.His722Asn	missense_variant	DNA-dependent RNA polymerase
TGME49_246060	p.Gln1376His	missense_variant	DNA-dependent RNA polymerase
TGME49_246060	p.Ile1824Met	missense_variant	DNA-dependent RNA polymerase
TGME49_249560	p.Ala270Val	missense_variant	DNA-directed RNA polymerase alpha chain rpoA
TGME49_249560	p.Lys600Glu	missense_variant	DNA-directed RNA polymerase alpha chain rpoA
TGME49_249560	p.Pro619Gln	missense_variant	DNA-directed RNA polymerase alpha chain rpoA
TGME49_249560	p.Arg646Cys	missense_variant	DNA-directed RNA polymerase alpha chain rpoA
TGME49_249560	p.Leu706Pro	missense_variant	DNA-directed RNA polymerase alpha chain rpoA
TGME49_250060	c.-392C>G	5_prime_UTR_premature_start_codon_gain_variant	DNA-directed RNA polymerase I RPA12
TGME49_250060	p.Ala120Gly	missense_variant	DNA-directed RNA polymerase I RPA12
TGME49_250060	p.Asp209Gly	missense_variant	DNA-directed RNA polymerase I RPA12
TGME49_297530	c.1372-8T>C	splice_region_variant	DNA-directed RNA polymerase I RPA2
TGME49_246630	p.Ala361Val	missense_variant	DNA-directed RNA polymerase I RPA43
TGME49_238190	c.-477C>T	5_prime_UTR_premature_start_codon_gain_variant	DNA-directed RNA polymerase II RPB3
TGME49_238190	p.Ala81Pro	missense_variant	DNA-directed RNA polymerase II RPB3

TGME49_238190	c.687+4G>A	splice_region_variant	DNA-directed RNA polymerase II RPB3
TGME49_217580	p.Ser17Ala	missense_variant	DNA-directed RNA polymerase III RPC6
TGME49_254280	p.Arg412Ser	missense_variant	DNA-directed RNA polymerase III RPC9
TGME49_254280	p.Thr410Met	missense_variant	DNA-directed RNA polymerase III RPC9
TGME49_253650	p.Asn489Ser	missense_variant	DnaJ C terminal region domain-containing protein
TGME49_320060	c.-855G>T	5_prime_UTR_premature_start_codon_gain_variant	DnaJ domain-containing protein
TGME49_320060	p.Leu71Ile	missense_variant	DnaJ domain-containing protein
TGME49_320060	p.Ser156Pro	missense_variant	DnaJ domain-containing protein
TGME49_320060	p.Leu421Val	missense_variant	DnaJ domain-containing protein
TGME49_320060	p.Asp599His	missense_variant	DnaJ domain-containing protein
TGME49_320060	c.*884C>T	splice_region_variant	DnaJ domain-containing protein
TGME49_301340	c.751-5C>T	splice_region_variant	DnaJ domain-containing protein
TGME49_301340	p.Ile288Leu	missense_variant	DnaJ domain-containing protein
TGME49_301340	p.Gln651Glu	missense_variant	DnaJ domain-containing protein
TGME49_224670	p.Glu2369Gln	missense_variant	DnaJ domain-containing protein
TGME49_224670	p.Tyr2281Asp	missense_variant	DnaJ domain-containing protein
TGME49_224670	p.Lys2024Arg	missense_variant	DnaJ domain-containing protein
TGME49_224670	p.Gln1339His	missense_variant	DnaJ domain-containing protein
TGME49_224670	p.His991Arg	missense_variant	DnaJ domain-containing protein
TGME49_224670	p.Leu806Met	missense_variant	DnaJ domain-containing protein
TGME49_224670	p.Ala706Val	missense_variant	DnaJ domain-containing protein
TGME49_224670	p.Lys645Asn	missense_variant	DnaJ domain-containing protein
TGME49_224670	p.Phe462Tyr	missense_variant	DnaJ domain-containing protein
TGME49_224670	p.Ala419Ser	missense_variant	DnaJ domain-containing protein
TGME49_224670	p.Met398Thr	missense_variant	DnaJ domain-containing protein
TGME49_224670	c.-71C>G	5_prime_UTR_premature_start_codon_gain_variant	DnaJ domain-containing protein
TGME49_214330	p.Phe369Leu	missense_variant	DnaJ domain-containing protein
TGME49_214530	p.Phe61Cys	missense_variant	DnaJ domain-containing protein
TGME49_214530	p.Arg32Thr	missense_variant	DnaJ domain-containing protein
TGME49_246340	c.-1332G>C	5_prime_UTR_premature_start_codon_gain_variant	DnaJ domain-containing protein
TGME49_246340	c.-501G>T	5_prime_UTR_premature_start_codon_gain_variant	DnaJ domain-containing protein
TGME49_246340	p.Leu30Phe	missense_variant	DnaJ domain-containing protein
TGME49_246340	p.Ser871Thr	missense_variant	DnaJ domain-containing protein
TGME49_219310	c.1463-6G>A	splice_region_variant	DnaK family protein
TGME49_219310	p.Val553Ala	missense_variant	DnaK family protein
TGME49_214230	p.Ala54Pro	missense_variant	Dopey, N-terminal domain-containing protein
TGME49_214230	p.Ser746Pro	missense_variant	Dopey, N-terminal domain-containing protein
TGME49_214230	p.Leu1054Val	missense_variant	Dopey, N-terminal domain-containing protein
TGME49_214230	p.Tyr1180Ser	missense_variant	Dopey, N-terminal domain-containing protein
TGME49_214230	p.Leu1193Phe	missense_variant	Dopey, N-terminal domain-containing protein
TGME49_214230	p.His1486Arg	missense_variant	Dopey, N-terminal domain-containing protein
TGME49_215410	c.-440C>T	5_prime_UTR_premature_start_codon_gain_variant	DPCD family protein
TGME49_215410	p.Lys16Glu	missense_variant	DPCD family protein
TGME49_221500	p.Asp785Glu	missense_variant	dual specificity phosphatase, catalytic domain-containing protein
TGME49_221500	p.Arg671Ser	missense_variant	dual specificity phosphatase, catalytic domain-containing protein
TGME49_221500	p.Lys363Thr	missense_variant	dual specificity phosphatase, catalytic domain-containing protein
TGME49_221500	p.Ser258Arg	missense_variant	dual specificity phosphatase, catalytic domain-containing protein
TGME49_221590	p.Arg103Lys	missense_variant	dual specificity phosphatase, catalytic domain-containing protein
TGME49_221590	p.Pro125Ser	missense_variant	dual specificity phosphatase, catalytic domain-containing protein
TGME49_221590	c.724-8C>T	splice_region_variant	dual specificity phosphatase, catalytic domain-containing protein
TGME49_235890	p.Ser43Asn	missense_variant	dual-specificity protein phosphatase
TGME49_235890	p.Val39Ala	missense_variant	dual-specificity protein phosphatase
TGME49_235890	p.Gly34Cys	missense_variant	dual-specificity protein phosphatase
TGME49_235890	p.Lys21Thr	missense_variant	dual-specificity protein phosphatase
TGME49_235890	c.-641T>C	5_prime_UTR_premature_start_codon_gain_variant	dual-specificity protein phosphatase
TGME49_236530	c.-556T>C	5_prime_UTR_premature_start_codon_gain_variant	DUF298 domain-containing protein
TGME49_211300	p.Val62Leu	missense_variant	DUF74 family protein, putative
TGME49_249840	c.467+8C>T	splice_region_variant	dynein heavy chain 2, putative
TGME49_249840	p.Ala688Thr	missense_variant	dynein heavy chain 2, putative
TGME49_249840	c.2394+8T>A	splice_region_variant	dynein heavy chain 2, putative
TGME49_249840	p.Ala881Thr	missense_variant	dynein heavy chain 2, putative
TGME49_249840	p.Gln1149His	missense_variant	dynein heavy chain 2, putative
TGME49_247560	c.212-6T>C	splice_region_variant	dynein light chain protein, putative
TGME49_235920	p.Ile4341Thr	missense_variant	dynein, axonemal, heavy chain 2 family protein
TGME49_235920	p.Lys4310Thr	missense_variant	dynein, axonemal, heavy chain 2 family protein
TGME49_235920	p.Pro3502Ser	missense_variant	dynein, axonemal, heavy chain 2 family protein
TGME49_235920	p.Asp3133Asn	missense_variant	dynein, axonemal, heavy chain 2 family protein
TGME49_235920	p.Ile3014Val	missense_variant	dynein, axonemal, heavy chain 2 family protein
TGME49_235920	p.Leu2702Phe	missense_variant	dynein, axonemal, heavy chain 2 family protein
TGME49_235920	c.7552-5A>T	splice_region_variant	dynein, axonemal, heavy chain 2 family protein
TGME49_235920	c.6958-3T>C	splice_region_variant	dynein, axonemal, heavy chain 2 family protein
TGME49_235920	p.Asp2159Ala	missense_variant	dynein, axonemal, heavy chain 2 family protein
TGME49_235920	p.His1533Asn	missense_variant	dynein, axonemal, heavy chain 2 family protein
TGME49_219080	c.-119T>C	5_prime_UTR_premature_start_codon_gain_variant	edge expressed protein, putative
TGME49_219080	p.Thr231Asn	missense_variant	edge expressed protein, putative
TGME49_223520	p.Ala51Val	missense_variant	EF hand domain-containing protein
TGME49_223520	p.Leu174Val	missense_variant	EF hand domain-containing protein
TGME49_275790	p.Leu92Val	missense_variant	EGF family domain-containing protein
TGME49_275790	p.Leu233Phe	missense_variant	EGF family domain-containing protein
TGME49_238210	p.Glu303Asp	missense_variant	EGF family domain-containing protein
TGME49_238220	p.Leu54Ile	missense_variant	EGF family domain-containing protein
TGME49_319610	c.-493T>G	5_prime_UTR_premature_start_codon_gain_variant	eIF2 kinase IF2K-D (incomplete catalytic triad)
TGME49_319610	p.Cys87Phe	missense_variant	eIF2 kinase IF2K-D (incomplete catalytic triad)
TGME49_319610	p.Arg220Gln	missense_variant	eIF2 kinase IF2K-D (incomplete catalytic triad)
TGME49_319610	p.Gly304Glu	missense_variant	eIF2 kinase IF2K-D (incomplete catalytic triad)

TGME49_319610	p.Arg400Ser	missense_variant	eIF2 kinase IF2K-D (incomplete catalytic triad)
TGME49_319610	p.Ala533Thr	missense_variant	eIF2 kinase IF2K-D (incomplete catalytic triad)
TGME49_319610	p.Arg575Gly	missense_variant	eIF2 kinase IF2K-D (incomplete catalytic triad)
TGME49_319610	p.Ala902Pro	missense_variant	eIF2 kinase IF2K-D (incomplete catalytic triad)
TGME49_319610	p.Leu1554Arg	missense_variant	eIF2 kinase IF2K-D (incomplete catalytic triad)
TGME49_319610	p.Thr1662Met	missense_variant	eIF2 kinase IF2K-D (incomplete catalytic triad)
TGME49_319610	p.Thr2038Ala	missense_variant	eIF2 kinase IF2K-D (incomplete catalytic triad)
TGME49_319610	p.Ala2165Thr	missense_variant	eIF2 kinase IF2K-D (incomplete catalytic triad)
TGME49_319610	p.Ala2251Thr	missense_variant	eIF2 kinase IF2K-D (incomplete catalytic triad)
TGME49_319610	p.Gly2338Val	missense_variant	eIF2 kinase IF2K-D (incomplete catalytic triad)
TGME49_319610	p.Asn2774Ser	missense_variant	eIF2 kinase IF2K-D (incomplete catalytic triad)
TGME49_207250	p.Val255Ile	missense_variant	electron-transfer-flavoprotein, alpha polypeptide
TGME49_253000	p.Asp418Asn	missense_variant	ELMO/CED-12 family protein
TGME49_218790	c.-1098C>T	5_prime_UTR_premature_start_codon_gain_variant	elongation factor G C-terminus domain-containing protein
TGME49_218790	c.-164C>G	5_prime_UTR_premature_start_codon_gain_variant	elongation factor G C-terminus domain-containing protein
TGME49_218790	p.Arg179Gln	missense_variant	elongation factor G C-terminus domain-containing protein
TGME49_222070	c.-481C>T	5_prime_UTR_premature_start_codon_gain_variant	elongation factor Tu GTP binding domain-containing protein
TGME49_222070	p.His135Arg	missense_variant	elongation factor Tu GTP binding domain-containing protein
TGME49_320150	p.Ala910Thr	missense_variant	elongation factor Tu GTP binding domain-containing protein
TGME49_320150	p.Ala686Pro	missense_variant	elongation factor Tu GTP binding domain-containing protein
TGME49_320150	p.Val531Ala	missense_variant	elongation factor Tu GTP binding domain-containing protein
TGME49_320150	p.Ser530Tyr	missense_variant	elongation factor Tu GTP binding domain-containing protein
TGME49_320150	p.Ser525Ala	missense_variant	elongation factor Tu GTP binding domain-containing protein
TGME49_320150	p.Ala521Gly	missense_variant	elongation factor Tu GTP binding domain-containing protein
TGME49_320150	p.Asn493Asp	missense_variant	elongation factor Tu GTP binding domain-containing protein
TGME49_320150	p.Leu480Pro	missense_variant	elongation factor Tu GTP binding domain-containing protein
TGME49_320150	p.Leu480Phe	missense_variant	elongation factor Tu GTP binding domain-containing protein
TGME49_320150	p.Leu325Phe	missense_variant	elongation factor Tu GTP binding domain-containing protein
TGME49_320150	p.Asp255Glu	missense_variant	elongation factor Tu GTP binding domain-containing protein
TGME49_320150	p.Glu231Val	missense_variant	elongation factor Tu GTP binding domain-containing protein
TGME49_320150	p.Glu231*	stop_gained	elongation factor Tu GTP binding domain-containing protein
TGME49_320150	p.Thr230Ile	missense_variant	elongation factor Tu GTP binding domain-containing protein
TGME49_320150	p.Phe38Leu	missense_variant	elongation factor Tu GTP binding domain-containing protein
TGME49_320150	c.-102G>A	5_prime_UTR_premature_start_codon_gain_variant	elongation factor Tu GTP binding domain-containing protein
TGME49_320150	c.-105G>A	5_prime_UTR_premature_start_codon_gain_variant	elongation factor Tu GTP binding domain-containing protein
TGME49_301380	c.3002+6C>T	splice_region_variant	elongation factor Tu GTP binding domain-containing protein
TGME49_301380	c.1819+7C>T	splice_region_variant	elongation factor Tu GTP binding domain-containing protein
TGME49_301380	p.Gly405Val	missense_variant	elongation factor Tu GTP binding domain-containing protein
TGME49_301380	p.Ser308Asn	missense_variant	elongation factor Tu GTP binding domain-containing protein
TGME49_301380	p.Phe304Cys	missense_variant	elongation factor Tu GTP binding domain-containing protein
TGME49_301380	p.Ser279Ala	missense_variant	elongation factor Tu GTP binding domain-containing protein
TGME49_301380	p.Pro278Thr	missense_variant	elongation factor Tu GTP binding domain-containing protein
TGME49_301380	c.-487A>T	5_prime_UTR_premature_start_codon_gain_variant	elongation factor Tu GTP binding domain-containing protein
TGME49_278550	p.Arg2202Thr	missense_variant&splice_region_variant	elongation factor Tu GTP binding domain-containing protein
TGME49_278550	p.Val1540Ala	missense_variant	elongation factor Tu GTP binding domain-containing protein
TGME49_278550	p.Val1540Leu	missense_variant	elongation factor Tu GTP binding domain-containing protein
TGME49_278550	p.Val448Ala	missense_variant	elongation factor Tu GTP binding domain-containing protein
TGME49_278550	p.Ser366Arg	missense_variant	elongation factor Tu GTP binding domain-containing protein
TGME49_278550	p.Ala251Thr	missense_variant	elongation factor Tu GTP binding domain-containing protein
TGME49_278550	p.Arg168Pro	missense_variant	elongation factor Tu GTP binding domain-containing protein
TGME49_254350	p.Arg18Gly	missense_variant	endo-1,3(4)-beta-glucanase
TGME49_238400	p.Pro925Gln	missense_variant	endonuclease/exonuclease/phosphatase domain-containing protein
TGME49_238400	p.Glu969Lys	missense_variant	endonuclease/exonuclease/phosphatase domain-containing protein
TGME49_238400	p.Gln1837Arg	missense_variant	endonuclease/exonuclease/phosphatase domain-containing protein
TGME49_238400	p.Ser1967Asn	missense_variant	endonuclease/exonuclease/phosphatase domain-containing protein
TGME49_238400	p.His1968Leu	missense_variant	endonuclease/exonuclease/phosphatase domain-containing protein
TGME49_238400	p.Ala2076Val	missense_variant	endonuclease/exonuclease/phosphatase domain-containing protein
TGME49_238400	p.Thr2304Ile	missense_variant	endonuclease/exonuclease/phosphatase domain-containing protein
TGME49_238400	p.Gln2578Glu	missense_variant	endonuclease/exonuclease/phosphatase domain-containing protein
TGME49_238400	p.Ala2628Pro	missense_variant	endonuclease/exonuclease/phosphatase domain-containing protein
TGME49_301216	p.Thr202Lys	missense_variant	endonuclease/exonuclease/phosphatase family protein
TGME49_301216	p.Pro199Ala	missense_variant	endonuclease/exonuclease/phosphatase family protein
TGME49_276910	c.-1772T>C	5_prime_UTR_premature_start_codon_gain_variant	endoplasmic reticulum lumen protein retaining receptor (ERD2) family protein
TGME49_254740	p.Ile62Val	missense_variant	endoribonuclease L-PSP protein
TGME49_251930	c.-50G>A	5_prime_UTR_premature_start_codon_gain_variant	enoyl-acyl carrier reductase ENR
TGME49_224090	c.-348A>G	5_prime_UTR_premature_start_codon_gain_variant	enoyl-CoA hydratase/isomerase family protein
TGME49_224090	p.Gly124Ala	missense_variant	enoyl-CoA hydratase/isomerase family protein
TGME49_224090	c.967+8C>G	splice_region_variant	enoyl-CoA hydratase/isomerase family protein
TGME49_214180	c.-719C>T	5_prime_UTR_premature_start_codon_gain_variant	ENTH domain-containing protein
TGME49_214760	p.Val239Ile	missense_variant	erythronate-4-phosphate dehydrogenase domain-containing protein
TGME49_249350	c.-1082C>G	5_prime_UTR_premature_start_codon_gain_variant	esterase/lipase/thioesterase domain-containing protein
TGME49_235540	c.-198C>T	5_prime_UTR_premature_start_codon_gain_variant	eukaryotic initiation factor-2 beta, putative
TGME49_235970	c.549+7C>A	splice_region_variant	eukaryotic initiation factor-2 gamma, putative
TGME49_235970	c.1290+8A>G	splice_region_variant	eukaryotic initiation factor-2 gamma, putative
TGME49_211230	p.Gly47Arg	missense_variant	eukaryotic initiation factor-2B, alpha subunit, putative
TGME49_211230	p.Glu26Gln	missense_variant	eukaryotic initiation factor-2B, alpha subunit, putative
TGME49_315150	p.Lys109Thr	missense_variant	eukaryotic initiation factor-4E, putative
TGME49_318700	c.-552A>C	5_prime_UTR_premature_start_codon_gain_variant	eukaryotic initiation factor-6, putative
TGME49_317720	c.340+4A>G	splice_region_variant	eukaryotic translation initiation factor 3 subunit 7, putative
TGME49_237020	p.Phe1354Leu	missense_variant	exonuclease
TGME49_237020	c.1319-6C>T	splice_region_variant	exonuclease
TGME49_224860	c.457-7T>C	splice_region_variant	exosome component 2, putative
TGME49_224860	p.Ala16Val	missense_variant	exosome component 2, putative
TGME49_224860	c.-268C>A	5_prime_UTR_premature_start_codon_gain_variant	exosome component 2, putative
TGME49_219290	c.-544T>A	5_prime_UTR_premature_start_codon_gain_variant	F-actin-capping protein subunit beta, putative

TGME49_219290	c.-644C>G	5_prime_UTR_premature_start_codon_gain_variant	F-actin-capping protein subunit beta, putative
TGME49_299230	p.Pro522His	missense_variant	F-box domain-containing protein
TGME49_299230	p.Pro525Ser	missense_variant	F-box domain-containing protein
TGME49_223700	c.3938+7T>C	splice_region_variant	F5/8 type C domain-containing protein
TGME49_223700	p.Met1058Leu	missense_variant	F5/8 type C domain-containing protein
TGME49_223830	p.Thr13Ala	missense_variant	fasciclin domain-containing protein
TGME49_248530	p.Val2172Leu	missense_variant	FATC domain-containing protein
TGME49_248530	c.5149+6G>A	splice_region_variant	FATC domain-containing protein
TGME49_248530	c.4788-4T>C	splice_region_variant	FATC domain-containing protein
TGME49_248530	p.Gly1532Ser	missense_variant	FATC domain-containing protein
TGME49_248530	c.4077+6G>T	splice_region_variant	FATC domain-containing protein
TGME49_248530	p.Ala1286Thr	missense_variant	FATC domain-containing protein
TGME49_248530	p.Asn1278Ser	missense_variant	FATC domain-containing protein
TGME49_248530	p.Gly1273Asp	missense_variant	FATC domain-containing protein
TGME49_248530	p.Ala1250Gly	missense_variant	FATC domain-containing protein
TGME49_248530	p.Ser866Ile	missense_variant	FATC domain-containing protein
TGME49_248530	p.Cys830Trp	missense_variant	FATC domain-containing protein
TGME49_248530	p.Met757Thr	missense_variant	FATC domain-containing protein
TGME49_248530	p.Pro520Ala	missense_variant	FATC domain-containing protein
TGME49_248530	p.Val239Leu	missense_variant	FATC domain-containing protein
TGME49_248530	p.Ala76Pro	missense_variant	FATC domain-containing protein
TGME49_238950	p.Arg176His	missense_variant	fatty acyl-CoA desaturase, putative
TGME49_277010	p.Arg156Pro	missense_variant	Fe-S metabolism associated domain-containing protein
TGME49_210750	p.Glu746Gln	missense_variant	Ferredoxin-fold anticodon binding domain-containing protein
TGME49_210750	p.Arg377Gly	missense_variant	Ferredoxin-fold anticodon binding domain-containing protein
TGME49_236000	p.Ser14Leu	missense_variant	ferredoxin, putative
TGME49_301450	p.Ala58Ser	missense_variant	FG-GAP repeat-containing protein
TGME49_301450	p.Ala663Pro	missense_variant	FG-GAP repeat-containing protein
TGME49_301450	p.Ala1087Glu	missense_variant	FG-GAP repeat-containing protein
TGME49_224040	p.Ala220Thr	missense_variant	flagellar associated protein
TGME49_251620	p.Ala487Glu	missense_variant	flap structure-specific endonuclease 1, putative
TGME49_251620	c.-154A>G	5_prime_UTR_premature_start_codon_gain_variant	flap structure-specific endonuclease 1, putative
TGME49_251620	c.-419T>G	5_prime_UTR_premature_start_codon_gain_variant	flap structure-specific endonuclease 1, putative
TGME49_219630	p.Leu136Val	missense_variant	flavodoxin domain-containing protein
TGME49_219630	p.Ser553Pro	missense_variant	flavodoxin domain-containing protein
TGME49_219630	p.Ala928Ser	missense_variant	flavodoxin domain-containing protein
TGME49_249320	c.2893-3T>C	splice_region_variant	flavodoxin domain-containing protein
TGME49_249320	p.Cys677Tyr	missense_variant	flavodoxin domain-containing protein
TGME49_249320	c.1771-7T>C	splice_region_variant	flavodoxin domain-containing protein
TGME49_249320	p.Val21Ile	missense_variant	flavodoxin domain-containing protein
TGME49_235640	p.Pro100Leu	missense_variant	formyl transferase domain-containing protein
TGME49_235640	p.Arg283His	missense_variant	formyl transferase domain-containing protein
TGME49_235640	p.Leu479His	missense_variant	formyl transferase domain-containing protein
TGME49_235640	p.Asp574Asn	missense_variant	formyl transferase domain-containing protein
TGME49_236040	c.-363T>C	5_prime_UTR_premature_start_codon_gain_variant	fructose-1,6-bisphosphate aldolase
TGME49_307770	p.Thr1108Arg	missense_variant	fumble protein
TGME49_237870	c.-300G>T	5_prime_UTR_premature_start_codon_gain_variant	FYVE zinc finger domain-containing protein
TGME49_237870	c.565-5T>G	splice_region_variant	FYVE zinc finger domain-containing protein
TGME49_237870	p.Ser324Asn	missense_variant	FYVE zinc finger domain-containing protein
TGME49_278040	c.-586T>A	5_prime_UTR_premature_start_codon_gain_variant	G-patch domain-containing protein
TGME49_278040	c.-175G>C	5_prime_UTR_premature_start_codon_gain_variant	G-patch domain-containing protein
TGME49_223940	c.-976T>C	5_prime_UTR_premature_start_codon_gain_variant	GAP45
TGME49_253930	p.Gly18Cys	missense_variant	GCC2 and GCC3 domain-containing protein
TGME49_253930	p.Arg20Trp	missense_variant	GCC2 and GCC3 domain-containing protein
TGME49_253930	p.Ala220Pro	missense_variant	GCC2 and GCC3 domain-containing protein
TGME49_253930	p.Thr578Ala	missense_variant	GCC2 and GCC3 domain-containing protein
TGME49_253930	p.Thr1090Ile	missense_variant	GCC2 and GCC3 domain-containing protein
TGME49_253930	p.Pro1595Ser	missense_variant	GCC2 and GCC3 domain-containing protein
TGME49_253930	c.5362+8T>C	splice_region_variant	GCC2 and GCC3 domain-containing protein
TGME49_253930	c.7554+3T>A	splice_region_variant	GCC2 and GCC3 domain-containing protein
TGME49_278878	p.Gln238His	missense_variant	GDA1/CD39 (nucleoside phosphatase) family protein
TGME49_278878	p.Met155Val	missense_variant	GDA1/CD39 (nucleoside phosphatase) family protein
TGME49_278878	p.Ile96Val	missense_variant	GDA1/CD39 (nucleoside phosphatase) family protein
TGME49_278878	p.Glu52Asp	missense_variant	GDA1/CD39 (nucleoside phosphatase) family protein
TGME49_278878	p.Met1?	start_lost	GDA1/CD39 (nucleoside phosphatase) family protein
TGME49_277720	p.Glu75Gly	missense_variant	GDA1/CD39 (nucleoside phosphatase) family protein
TGME49_214520	p.Ala28Thr	missense_variant	general transcription factor IIH polypeptide 4 GTF2H4
TGME49_217450	p.Leu97Gln	missense_variant	general transcription factor IIH polypeptide 5 GTF2H5
TGME49_217450	p.Ser51Asn	missense_variant	general transcription factor IIH polypeptide 5 GTF2H5
TGME49_212170	p.Arg240Thr	missense_variant	GIY-YIG catalytic domain-containing protein
TGME49_212170	p.Asp291Asn	missense_variant	GIY-YIG catalytic domain-containing protein
TGME49_212170	p.Gly1080Arg	missense_variant	GIY-YIG catalytic domain-containing protein
TGME49_318580	p.Ser365Ala	missense_variant	glucosephosphate-mutase GPM2
TGME49_249390	c.-400C>G	5_prime_UTR_premature_start_codon_gain_variant	glutamate/leucine/phenylalanine/valine dehydrogenase family protein
TGME49_249390	p.Asp744Glu	missense_variant	glutamate/leucine/phenylalanine/valine dehydrogenase family protein
TGME49_217460	p.Pro164Leu	missense_variant	glutaminyl-tRNA synthetase (GlnRS)
TGME49_217460	p.Val127Ile	missense_variant	glutaminyl-tRNA synthetase (GlnRS)
TGME49_238070	c.144+6C>A	splice_region_variant	glutaredoxin domain-containing protein
TGME49_246920	c.-255C>T	5_prime_UTR_premature_start_codon_gain_variant	glutathione reductase
TGME49_249630	p.Met353Leu	missense_variant	glutathione S-transferase, N-terminal domain-containing protein
TGME49_249630	c.-334C>G	5_prime_UTR_premature_start_codon_gain_variant	glutathione S-transferase, N-terminal domain-containing protein
TGME49_237470	c.-1501C>T	5_prime_UTR_premature_start_codon_gain_variant	glycine cleavage T-protein (aminomethyl transferase) domain-containing protein
TGME49_237470	c.-43A>G	5_prime_UTR_premature_start_codon_gain_variant	glycine cleavage T-protein (aminomethyl transferase) domain-containing protein
TGME49_237470	p.Leu172Val	missense_variant	glycine cleavage T-protein (aminomethyl transferase) domain-containing protein
TGME49_237470	p.Ser200Tyr	missense_variant	glycine cleavage T-protein (aminomethyl transferase) domain-containing protein

TGME49_237470	p.Lys314Met	missense_variant	glycine cleavage T-protein (aminomethyl transferase) domain-containing protein
TGME49_237470	p.Ala327Ser	missense_variant	glycine cleavage T-protein (aminomethyl transferase) domain-containing protein
TGME49_237470	p.Gly350Glu	missense_variant	glycine cleavage T-protein (aminomethyl transferase) domain-containing protein
TGME49_237470	p.Pro358Leu	missense_variant	glycine cleavage T-protein (aminomethyl transferase) domain-containing protein
TGME49_237470	p.Gln614Arg	missense_variant	glycine cleavage T-protein (aminomethyl transferase) domain-containing protein
TGME49_222800	p.Pro2047Leu	missense_variant	glycogen synthase, putative
TGME49_222800	p.Thr2036Ser	missense_variant	glycogen synthase, putative
TGME49_222800	p.Arg1858Lys	missense_variant	glycogen synthase, putative
TGME49_222800	p.Cys1838Phe	missense_variant	glycogen synthase, putative
TGME49_222800	p.Pro1793Leu	missense_variant	glycogen synthase, putative
TGME49_222800	p.Leu1631Pro	missense_variant	glycogen synthase, putative
TGME49_222800	p.Thr1533Ser	missense_variant	glycogen synthase, putative
TGME49_222800	p.Asn1423Asp	missense_variant	glycogen synthase, putative
TGME49_222800	c.4219+4G>A	splice_region_variant	glycogen synthase, putative
TGME49_222800	p.Pro204Ala	missense_variant	glycogen synthase, putative
TGME49_222800	p.Asp138Glu	missense_variant	glycogen synthase, putative
TGME49_253030	p.Gln557Glu	missense_variant	glycosyl hydrolase, family 31 protein
TGME49_318730	p.Arg72His	missense_variant	glycosyl transferase
TGME49_318730	c.-487A>G	5_prime_UTR_premature_start_codon_gain_variant	glycosyl transferase
TGME49_207070	p.Arg307Lys	missense_variant	glycosyl transferase, putative
TGME49_207070	p.Leu52Val	missense_variant	glycosyl transferase, putative
TGME49_248400	p.Pro6Ser	missense_variant	glyoxalase I, putative
TGME49_248400	c.-72G>T	5_prime_UTR_premature_start_codon_gain_variant	glyoxalase I, putative
TGME49_253880	p.Ala319Thr	missense_variant	GNS1/SUR4 family protein
TGME49_253880	p.Ala524Val	missense_variant	GNS1/SUR4 family protein
TGME49_275568	p.Gln59His	missense_variant	GPI transamidase subunit PIG-U protein
TGME49_214920	c.-738C>T	5_prime_UTR_premature_start_codon_gain_variant	GPI-anchored wall transfer protein GWT1 , putative
TGME49_214920	c.-315C>G	5_prime_UTR_premature_start_codon_gain_variant	GPI-anchored wall transfer protein GWT1 , putative
TGME49_214920	p.Ala7Thr	missense_variant	GPI-anchored wall transfer protein GWT1 , putative
TGME49_214920	p.Leu22Met	missense_variant	GPI-anchored wall transfer protein GWT1 , putative
TGME49_214920	p.Phe191Ser	missense_variant	GPI-anchored wall transfer protein GWT1 , putative
TGME49_214920	c.1408+6A>G	splice_region_variant	GPI-anchored wall transfer protein GWT1 , putative
TGME49_214920	p.Val735Leu	missense_variant	GPI-anchored wall transfer protein GWT1 , putative
TGME49_308000	c.-894C>T	5_prime_UTR_premature_start_codon_gain_variant	Gpi16 subunit, GPI transamidase component protein
TGME49_308000	c.-105C>G	5_prime_UTR_premature_start_codon_gain_variant	Gpi16 subunit, GPI transamidase component protein
TGME49_308000	c.-98G>A	5_prime_UTR_premature_start_codon_gain_variant	Gpi16 subunit, GPI transamidase component protein
TGME49_308000	p.Glu99Ala	missense_variant	Gpi16 subunit, GPI transamidase component protein
TGME49_308000	p.Val186Leu	missense_variant	Gpi16 subunit, GPI transamidase component protein
TGME49_308000	p.Pro542Leu	missense_variant	Gpi16 subunit, GPI transamidase component protein
TGME49_308000	p.Leu677Phe	missense_variant	Gpi16 subunit, GPI transamidase component protein
TGME49_224600	c.771+3A>G	splice_region_variant	GTP binding protein
TGME49_224600	c.-543G>T	5_prime_UTR_premature_start_codon_gain_variant	GTP binding protein
TGME49_253780	p.Ser294Asn	missense_variant	GTP cyclohydrolase I, putative
TGME49_217760	p.Gly89Ala	missense_variant	GTP-binding protein
TGME49_217760	p.Gly89Ser	missense_variant	GTP-binding protein
TGME49_217760	c.225+7C>G	splice_region_variant	GTP-binding protein
TGME49_211480	p.Ser1175Cys	missense_variant	GTP-binding protein engA, putative
TGME49_211480	c.2830+6C>A	splice_region_variant	GTP-binding protein engA, putative
TGME49_211480	c.2331+6T>A	splice_region_variant	GTP-binding protein engA, putative
TGME49_211480	p.Asn623Ile	missense_variant	GTP-binding protein engA, putative
TGME49_211480	p.Gln405Glu	missense_variant	GTP-binding protein engA, putative
TGME49_211480	p.Arg364Thr	missense_variant	GTP-binding protein engA, putative
TGME49_307980	p.Pro27Ser	missense_variant	GTP-binding protein lepA, putative
TGME49_307980	c.1365+6G>A	splice_region_variant	GTP-binding protein lepA, putative
TGME49_278730	c.-525C>T	5_prime_UTR_premature_start_codon_gain_variant	guanine nucleotide-binding protein, putative
TGME49_278730	p.Glu87Gly	missense_variant	guanine nucleotide-binding protein, putative
TGME49_278730	p.Trp90Arg	missense_variant	guanine nucleotide-binding protein, putative
TGME49_278730	p.Cys616Tyr	missense_variant	guanine nucleotide-binding protein, putative
TGME49_254370	p.Met43Val	missense_variant	guanylyl cyclase
TGME49_254370	p.Met1180Ile	missense_variant	guanylyl cyclase
TGME49_254370	c.9436+5T>G	splice_region_variant	guanylyl cyclase
TGME49_254370	p.Pro3503Ala	missense_variant	guanylyl cyclase
TGME49_254370	p.Ala3543Ser	missense_variant	guanylyl cyclase
TGME49_254370	p.Gly3588Glu	missense_variant	guanylyl cyclase
TGME49_254370	p.Pro3625Gln	missense_variant	guanylyl cyclase
TGME49_224610	p.Thr681Asn	missense_variant	GYF domain-containing protein
TGME49_224610	p.Gly692Asp	missense_variant	GYF domain-containing protein
TGME49_222010	c.1698+8T>C	splice_region_variant	HAD hydrolase, family IIID protein
TGME49_222010	p.Ala369Thr	missense_variant	HAD hydrolase, family IIID protein
TGME49_222010	p.Arg241Ser	missense_variant	HAD hydrolase, family IIID protein
TGME49_222010	c.-427G>C	5_prime_UTR_premature_start_codon_gain_variant	HAD hydrolase, family IIID protein
TGME49_236860	p.Pro1542Ser	missense_variant	haloacid dehalogenase family hydrolase domain-containing protein
TGME49_236860	p.Asn2080His	missense_variant	haloacid dehalogenase family hydrolase domain-containing protein
TGME49_217440	p.Pro92Ser	missense_variant	HCNGP family protein
TGME49_220910	p.Leu1464Phe	missense_variant	HEAT repeat-containing protein
TGME49_220910	p.Lys1063Asn	missense_variant	HEAT repeat-containing protein
TGME49_220910	p.Gly918Arg	missense_variant	HEAT repeat-containing protein
TGME49_220910	p.Ile671Thr	missense_variant	HEAT repeat-containing protein
TGME49_220910	c.-438C>T	5_prime_UTR_premature_start_codon_gain_variant	HEAT repeat-containing protein
TGME49_215040	p.Leu1619Val	missense_variant	HEAT repeat-containing protein
TGME49_215040	c.4055+8T>C	splice_region_variant	HEAT repeat-containing protein
TGME49_215040	p.Val1140Ile	missense_variant	HEAT repeat-containing protein
TGME49_215040	p.Thr358Ala	missense_variant	HEAT repeat-containing protein
TGME49_295710	p.Ile3754Asn	missense_variant	HECT-domain (ubiquitin-transferase) domain-containing protein
TGME49_222430	p.Gly1262Arg	missense_variant	HECT-domain (ubiquitin-transferase) domain-containing protein

TGME49_222430	p.Ala1134Gly	missense_variant	HECT-domain (ubiquitin-transferase) domain-containing protein
TGME49_222430	p.Thr1008Ala	missense_variant	HECT-domain (ubiquitin-transferase) domain-containing protein
TGME49_222430	p.Ser201Asn	missense_variant	HECT-domain (ubiquitin-transferase) domain-containing protein
TGME49_275630	p.Pro763Arg	missense_variant	HECT-domain (ubiquitin-transferase) domain-containing protein
TGME49_275630	p.Pro752Ala	missense_variant	HECT-domain (ubiquitin-transferase) domain-containing protein
TGME49_280660	p.Arg16242Lys	missense_variant	HECT-domain (ubiquitin-transferase) domain-containing protein
TGME49_280660	p.Ser16087Ala	missense_variant	HECT-domain (ubiquitin-transferase) domain-containing protein
TGME49_280660	p.Arg15784His	missense_variant	HECT-domain (ubiquitin-transferase) domain-containing protein
TGME49_280660	p.Asp15708Tyr	missense_variant	HECT-domain (ubiquitin-transferase) domain-containing protein
TGME49_280660	p.Asp15670Gly	missense_variant	HECT-domain (ubiquitin-transferase) domain-containing protein
TGME49_280660	p.Asp15464Gly	missense_variant	HECT-domain (ubiquitin-transferase) domain-containing protein
TGME49_280660	p.Arg15429His	missense_variant	HECT-domain (ubiquitin-transferase) domain-containing protein
TGME49_280660	p.Leu15352Val	missense_variant	HECT-domain (ubiquitin-transferase) domain-containing protein
TGME49_280660	p.Phe15336Leu	missense_variant	HECT-domain (ubiquitin-transferase) domain-containing protein
TGME49_280660	p.Val15300Gly	missense_variant	HECT-domain (ubiquitin-transferase) domain-containing protein
TGME49_280660	p.Ala15198Thr	missense_variant	HECT-domain (ubiquitin-transferase) domain-containing protein
TGME49_280660	p.Phe15171Val	missense_variant	HECT-domain (ubiquitin-transferase) domain-containing protein
TGME49_280660	p.Val15099Gly	missense_variant	HECT-domain (ubiquitin-transferase) domain-containing protein
TGME49_280660	p.Thr15024Met	missense_variant	HECT-domain (ubiquitin-transferase) domain-containing protein
TGME49_280660	p.Arg14947His	missense_variant	HECT-domain (ubiquitin-transferase) domain-containing protein
TGME49_280660	p.Gly14935Glu	missense_variant	HECT-domain (ubiquitin-transferase) domain-containing protein
TGME49_280660	p.Leu14843Val	missense_variant	HECT-domain (ubiquitin-transferase) domain-containing protein
TGME49_280660	p.Ser14769Phe	missense_variant	HECT-domain (ubiquitin-transferase) domain-containing protein
TGME49_280660	p.Ile14642Thr	missense_variant	HECT-domain (ubiquitin-transferase) domain-containing protein
TGME49_280660	p.Cys14641Gly	missense_variant	HECT-domain (ubiquitin-transferase) domain-containing protein
TGME49_280660	p.Glu14634Lys	missense_variant	HECT-domain (ubiquitin-transferase) domain-containing protein
TGME49_280660	p.Val14604Gly	missense_variant	HECT-domain (ubiquitin-transferase) domain-containing protein
TGME49_280660	p.Ala14494Thr	missense_variant	HECT-domain (ubiquitin-transferase) domain-containing protein
TGME49_280660	p.Lys14198Glu	missense_variant	HECT-domain (ubiquitin-transferase) domain-containing protein
TGME49_280660	p.Ala14124Ser	missense_variant	HECT-domain (ubiquitin-transferase) domain-containing protein
TGME49_280660	p.Glu14109Ala	missense_variant	HECT-domain (ubiquitin-transferase) domain-containing protein
TGME49_280660	p.Glu13609Ala	missense_variant	HECT-domain (ubiquitin-transferase) domain-containing protein
TGME49_280660	p.Gly13589Ala	missense_variant	HECT-domain (ubiquitin-transferase) domain-containing protein
TGME49_280660	p.Ser13411Ile	missense_variant	HECT-domain (ubiquitin-transferase) domain-containing protein
TGME49_280660	p.Ser13411Gly	missense_variant	HECT-domain (ubiquitin-transferase) domain-containing protein
TGME49_280660	p.Asp13250Glu	missense_variant	HECT-domain (ubiquitin-transferase) domain-containing protein
TGME49_280660	p.Phe13004Cys	missense_variant	HECT-domain (ubiquitin-transferase) domain-containing protein
TGME49_280660	p.Arg12877Leu	missense_variant	HECT-domain (ubiquitin-transferase) domain-containing protein
TGME49_280660	p.Pro12784Leu	missense_variant	HECT-domain (ubiquitin-transferase) domain-containing protein
TGME49_280660	p.Ser12651Asn	missense_variant	HECT-domain (ubiquitin-transferase) domain-containing protein
TGME49_280660	p.Ala12539Val	missense_variant	HECT-domain (ubiquitin-transferase) domain-containing protein
TGME49_280660	p.Ala12478Ser	missense_variant	HECT-domain (ubiquitin-transferase) domain-containing protein
TGME49_280660	p.His12449Tyr	missense_variant	HECT-domain (ubiquitin-transferase) domain-containing protein
TGME49_280660	p.Pro12370Leu	missense_variant	HECT-domain (ubiquitin-transferase) domain-containing protein
TGME49_280660	p.Gly12355Ala	missense_variant	HECT-domain (ubiquitin-transferase) domain-containing protein
TGME49_280660	p.Arg12219Pro	missense_variant	HECT-domain (ubiquitin-transferase) domain-containing protein
TGME49_280660	p.Ser12083Ala	missense_variant	HECT-domain (ubiquitin-transferase) domain-containing protein
TGME49_280660	p.Leu11889Val	missense_variant	HECT-domain (ubiquitin-transferase) domain-containing protein
TGME49_280660	p.Asp11739Gly	missense_variant	HECT-domain (ubiquitin-transferase) domain-containing protein
TGME49_280660	p.Tyr11702Asp	missense_variant	HECT-domain (ubiquitin-transferase) domain-containing protein
TGME49_280660	p.Glu11604Lys	missense_variant	HECT-domain (ubiquitin-transferase) domain-containing protein
TGME49_280660	p.Asp11499Glu	missense_variant	HECT-domain (ubiquitin-transferase) domain-containing protein
TGME49_280660	p.Ala11321Ser	missense_variant	HECT-domain (ubiquitin-transferase) domain-containing protein
TGME49_280660	p.Leu11235Pro	missense_variant	HECT-domain (ubiquitin-transferase) domain-containing protein
TGME49_280660	p.Pro11023Ser	missense_variant	HECT-domain (ubiquitin-transferase) domain-containing protein
TGME49_280660	p.Val10690Ile	missense_variant	HECT-domain (ubiquitin-transferase) domain-containing protein
TGME49_280660	p.Leu10660Phe	missense_variant	HECT-domain (ubiquitin-transferase) domain-containing protein
TGME49_280660	p.Ser10478Thr	missense_variant	HECT-domain (ubiquitin-transferase) domain-containing protein
TGME49_280660	p.Arg10447Ser	missense_variant	HECT-domain (ubiquitin-transferase) domain-containing protein
TGME49_280660	p.Glu10409Asp	missense_variant	HECT-domain (ubiquitin-transferase) domain-containing protein
TGME49_280660	p.Asp10376Glu	missense_variant	HECT-domain (ubiquitin-transferase) domain-containing protein
TGME49_280660	p.Asn10375Ser	missense_variant	HECT-domain (ubiquitin-transferase) domain-containing protein
TGME49_280660	p.Glu9881Asp	missense_variant	HECT-domain (ubiquitin-transferase) domain-containing protein
TGME49_280660	p.Ala9810Thr	missense_variant	HECT-domain (ubiquitin-transferase) domain-containing protein
TGME49_280660	p.Leu9624Val	missense_variant	HECT-domain (ubiquitin-transferase) domain-containing protein
TGME49_280660	p.Glu9597Lys	missense_variant	HECT-domain (ubiquitin-transferase) domain-containing protein
TGME49_280660	p.Leu9428Val	missense_variant	HECT-domain (ubiquitin-transferase) domain-containing protein
TGME49_280660	p.Asp9255Glu	missense_variant	HECT-domain (ubiquitin-transferase) domain-containing protein
TGME49_280660	p.Asp9081Asn	missense_variant	HECT-domain (ubiquitin-transferase) domain-containing protein
TGME49_280660	p.Ser9075Arg	missense_variant	HECT-domain (ubiquitin-transferase) domain-containing protein
TGME49_280660	p.Arg9061Gly	missense_variant	HECT-domain (ubiquitin-transferase) domain-containing protein
TGME49_280660	p.Leu8619Arg	missense_variant	HECT-domain (ubiquitin-transferase) domain-containing protein
TGME49_280660	p.Gln8599His	missense_variant	HECT-domain (ubiquitin-transferase) domain-containing protein
TGME49_280660	p.Glu8553Val	missense_variant	HECT-domain (ubiquitin-transferase) domain-containing protein
TGME49_280660	p.Glu8512Asp	missense_variant	HECT-domain (ubiquitin-transferase) domain-containing protein
TGME49_280660	p.Arg8510Gly	missense_variant	HECT-domain (ubiquitin-transferase) domain-containing protein
TGME49_280660	p.Arg8462Thr	missense_variant	HECT-domain (ubiquitin-transferase) domain-containing protein
TGME49_280660	p.Gly8319Ser	missense_variant	HECT-domain (ubiquitin-transferase) domain-containing protein
TGME49_280660	p.Val8205Ala	missense_variant	HECT-domain (ubiquitin-transferase) domain-containing protein
TGME49_280660	p.Arg8062Leu	missense_variant	HECT-domain (ubiquitin-transferase) domain-containing protein
TGME49_280660	p.Glu8028Asp	missense_variant	HECT-domain (ubiquitin-transferase) domain-containing protein
TGME49_280660	p.Val7962Ile	missense_variant	HECT-domain (ubiquitin-transferase) domain-containing protein
TGME49_280660	p.Ala7842Ser	missense_variant	HECT-domain (ubiquitin-transferase) domain-containing protein
TGME49_280660	p.Glu7833Ala	missense_variant	HECT-domain (ubiquitin-transferase) domain-containing protein
TGME49_280660	p.Ala7178Thr	missense_variant	HECT-domain (ubiquitin-transferase) domain-containing protein

TGME49_280660	p.Ala7160Thr	missense_variant	HECT-domain (ubiquitin-transferase) domain-containing protein
TGME49_280660	p.Arg7158Pro	missense_variant	HECT-domain (ubiquitin-transferase) domain-containing protein
TGME49_280660	p.Gly7149Ala	missense_variant	HECT-domain (ubiquitin-transferase) domain-containing protein
TGME49_280660	p.Arg6713Lys	missense_variant	HECT-domain (ubiquitin-transferase) domain-containing protein
TGME49_280660	p.Lys6712Glu	missense_variant	HECT-domain (ubiquitin-transferase) domain-containing protein
TGME49_280660	p.Cys6376Phe	missense_variant	HECT-domain (ubiquitin-transferase) domain-containing protein
TGME49_280660	p.Arg6080His	missense_variant	HECT-domain (ubiquitin-transferase) domain-containing protein
TGME49_280660	p.Phe5890Val	missense_variant	HECT-domain (ubiquitin-transferase) domain-containing protein
TGME49_280660	p.Val5844Ala	missense_variant	HECT-domain (ubiquitin-transferase) domain-containing protein
TGME49_280660	p.Ala5729Gly	missense_variant	HECT-domain (ubiquitin-transferase) domain-containing protein
TGME49_280660	p.Ala5680Glu	missense_variant	HECT-domain (ubiquitin-transferase) domain-containing protein
TGME49_280660	p.Asp4836Glu	missense_variant	HECT-domain (ubiquitin-transferase) domain-containing protein
TGME49_280660	p.Gly4807Ala	missense_variant	HECT-domain (ubiquitin-transferase) domain-containing protein
TGME49_280660	p.Ser4627Pro	missense_variant	HECT-domain (ubiquitin-transferase) domain-containing protein
TGME49_280660	p.Glu4624Ala	missense_variant	HECT-domain (ubiquitin-transferase) domain-containing protein
TGME49_280660	p.Gly4494Glu	missense_variant	HECT-domain (ubiquitin-transferase) domain-containing protein
TGME49_280660	p.Arg4490Gln	missense_variant	HECT-domain (ubiquitin-transferase) domain-containing protein
TGME49_280660	p.Phe3790Val	missense_variant	HECT-domain (ubiquitin-transferase) domain-containing protein
TGME49_280660	p.Glu3617Ala	missense_variant	HECT-domain (ubiquitin-transferase) domain-containing protein
TGME49_280660	p.Val3421Ala	missense_variant	HECT-domain (ubiquitin-transferase) domain-containing protein
TGME49_280660	p.Arg3413Cys	missense_variant	HECT-domain (ubiquitin-transferase) domain-containing protein
TGME49_280660	p.Tyr3388Asn	missense_variant	HECT-domain (ubiquitin-transferase) domain-containing protein
TGME49_280660	p.Thr3385Arg	missense_variant	HECT-domain (ubiquitin-transferase) domain-containing protein
TGME49_280660	p.Pro3356Leu	missense_variant	HECT-domain (ubiquitin-transferase) domain-containing protein
TGME49_280660	p.Gln3238Arg	missense_variant	HECT-domain (ubiquitin-transferase) domain-containing protein
TGME49_280660	p.Asp3235Glu	missense_variant	HECT-domain (ubiquitin-transferase) domain-containing protein
TGME49_280660	p.Arg3162Trp	missense_variant	HECT-domain (ubiquitin-transferase) domain-containing protein
TGME49_280660	p.Ala3056Thr	missense_variant	HECT-domain (ubiquitin-transferase) domain-containing protein
TGME49_280660	p.Gly3044Arg	missense_variant	HECT-domain (ubiquitin-transferase) domain-containing protein
TGME49_280660	p.Pro3023Leu	missense_variant	HECT-domain (ubiquitin-transferase) domain-containing protein
TGME49_280660	p.Ala2988Asp	missense_variant	HECT-domain (ubiquitin-transferase) domain-containing protein
TGME49_280660	p.Arg2959Ile	missense_variant	HECT-domain (ubiquitin-transferase) domain-containing protein
TGME49_280660	p.Asn2791Asp	missense_variant	HECT-domain (ubiquitin-transferase) domain-containing protein
TGME49_280660	p.Arg2785Leu	missense_variant	HECT-domain (ubiquitin-transferase) domain-containing protein
TGME49_280660	p.Gly2758Arg	missense_variant	HECT-domain (ubiquitin-transferase) domain-containing protein
TGME49_280660	p.Gly2745Arg	missense_variant	HECT-domain (ubiquitin-transferase) domain-containing protein
TGME49_280660	p.Asn2719Ser	missense_variant	HECT-domain (ubiquitin-transferase) domain-containing protein
TGME49_280660	p.Ser2716Asn	missense_variant	HECT-domain (ubiquitin-transferase) domain-containing protein
TGME49_280660	p.Ala2682Gly	missense_variant	HECT-domain (ubiquitin-transferase) domain-containing protein
TGME49_280660	p.Cys2637Ser	missense_variant	HECT-domain (ubiquitin-transferase) domain-containing protein
TGME49_280660	p.Glu2625Lys	missense_variant	HECT-domain (ubiquitin-transferase) domain-containing protein
TGME49_280660	p.Ala2463Thr	missense_variant	HECT-domain (ubiquitin-transferase) domain-containing protein
TGME49_280660	p.His1757Arg	missense_variant	HECT-domain (ubiquitin-transferase) domain-containing protein
TGME49_280660	p.Thr1692Met	missense_variant	HECT-domain (ubiquitin-transferase) domain-containing protein
TGME49_280660	p.Val1258Met	missense_variant	HECT-domain (ubiquitin-transferase) domain-containing protein
TGME49_280660	p.Leu1134Ile	missense_variant	HECT-domain (ubiquitin-transferase) domain-containing protein
TGME49_280660	p.Thr1083Ser	missense_variant	HECT-domain (ubiquitin-transferase) domain-containing protein
TGME49_280660	p.Ala1067Gly	missense_variant	HECT-domain (ubiquitin-transferase) domain-containing protein
TGME49_280660	p.His1066Arg	missense_variant	HECT-domain (ubiquitin-transferase) domain-containing protein
TGME49_280660	p.Arg686Gln	missense_variant	HECT-domain (ubiquitin-transferase) domain-containing protein
TGME49_280660	p.Phe103Leu	missense_variant	HECT-domain (ubiquitin-transferase) domain-containing protein
TGME49_278940	c.-666C>T	5_prime_UTR_premature_start_codon_gain_variant	HECT-domain (ubiquitin-transferase) domain-containing protein
TGME49_318440	c.2389+6T>G	splice_region_variant	helicase associated domain (ha2) protein
TGME49_318440	p.Ala185Ser	missense_variant	helicase associated domain (ha2) protein
TGME49_318440	p.Thr143Ser	missense_variant	helicase associated domain (ha2) protein
TGME49_318440	p.Leu34Val	missense_variant	helicase associated domain (ha2) protein
TGME49_297925	p.Arg90His	missense_variant	HesB-like domain-containing protein
TGME49_297925	p.Ser223Cys	missense_variant	HesB-like domain-containing protein
TGME49_297925	p.Leu325Val	missense_variant	HesB-like domain-containing protein
TGME49_297925	p.Pro344Leu	missense_variant	HesB-like domain-containing protein
TGME49_297925	p.Ser361Ala	missense_variant	HesB-like domain-containing protein
TGME49_280600	p.Val1221Phe	missense_variant	histidyl-tRNA synthetase (HisRS), putative
TGME49_252420	p.Thr30Met	missense_variant	histone arginine methyltransferase PRMT3
TGME49_252420	p.Ala156Val	missense_variant	histone arginine methyltransferase PRMT3
TGME49_252420	p.Val247Ala	missense_variant	histone arginine methyltransferase PRMT3
TGME49_252420	p.Gln296Arg	missense_variant	histone arginine methyltransferase PRMT3
TGME49_252420	p.Thr298Pro	missense_variant	histone arginine methyltransferase PRMT3
TGME49_252420	p.Leu300Val	missense_variant	histone arginine methyltransferase PRMT3
TGME49_252420	p.Leu300*	stop_gained	histone arginine methyltransferase PRMT3
TGME49_252420	p.Glu313Gln	missense_variant	histone arginine methyltransferase PRMT3
TGME49_252420	p.Glu682Gly	missense_variant	histone arginine methyltransferase PRMT3
TGME49_215560	p.Thr369Ile	missense_variant	histone arginine methyltransferase PRMT5
TGME49_249620	p.Leu576Pro	missense_variant	histone deacetylase HDAC2
TGME49_254555	c.-1566G>T	5_prime_UTR_premature_start_codon_gain_variant	histone lysine acetyltransferase GCN5-A
TGME49_254555	c.-893A>T	5_prime_UTR_premature_start_codon_gain_variant	histone lysine acetyltransferase GCN5-A
TGME49_254555	p.Val71Gly	missense_variant	histone lysine acetyltransferase GCN5-A
TGME49_254555	p.Asp437Tyr	missense_variant	histone lysine acetyltransferase GCN5-A
TGME49_254555	p.Asp453Gly	missense_variant	histone lysine acetyltransferase GCN5-A
TGME49_254555	p.Val483Ile	missense_variant	histone lysine acetyltransferase GCN5-A
TGME49_254555	p.Ala493Ser	missense_variant	histone lysine acetyltransferase GCN5-A
TGME49_254555	p.Gly637Ala	missense_variant	histone lysine acetyltransferase GCN5-A
TGME49_254555	p.Gly671Arg	missense_variant	histone lysine acetyltransferase GCN5-A
TGME49_254555	p.Gly671Glu	missense_variant	histone lysine acetyltransferase GCN5-A
TGME49_254555	p.Ala688Thr	missense_variant	histone lysine acetyltransferase GCN5-A
TGME49_254555	p.Glu689Lys	missense_variant	histone lysine acetyltransferase GCN5-A

TGME49_254555	p.Asn693Lys	missense_variant	histone lysine acetyltransferase GCN5-A
TGME49_207080	c.-1068T>G	5_prime_UTR_premature_start_codon_gain_variant	histone lysine acetyltransferase MYST-B
TGME49_224928	p.Leu248Val	missense_variant	histone lysine demethylase JMJD4
TGME49_224928	p.Asp178Ala	missense_variant	histone lysine demethylase JMJD4
TGME49_224928	p.Ala166Asp	missense_variant	histone lysine demethylase JMJD4
TGME49_224928	p.Glu108Gly	missense_variant	histone lysine demethylase JMJD4
TGME49_224928	p.Gln45Glu	missense_variant	histone lysine demethylase JMJD4
TGME49_224928	p.Ser7Ala	missense_variant	histone lysine demethylase JMJD4
TGME49_224928	p.Lys6Thr	missense_variant	histone lysine demethylase JMJD4
TGME49_224928	c.-77C>T	5_prime_UTR_premature_start_codon_gain_variant	histone lysine demethylase JMJD4
TGME49_212110	p.Ala954Thr	missense_variant	histone lysine demethylase JMJD5
TGME49_212110	p.Pro928Leu	missense_variant	histone lysine demethylase JMJD5
TGME49_212110	p.Pro905Ser	missense_variant	histone lysine demethylase JMJD5
TGME49_212110	p.Thr776Pro	missense_variant	histone lysine demethylase JMJD5
TGME49_212110	p.Ser665Arg	missense_variant	histone lysine demethylase JMJD5
TGME49_212110	p.Gly657Arg	missense_variant	histone lysine demethylase JMJD5
TGME49_212110	p.Lys654Thr	missense_variant	histone lysine demethylase JMJD5
TGME49_212110	p.Cys476Phe	missense_variant	histone lysine demethylase JMJD5
TGME49_212110	p.Ile472Met	missense_variant	histone lysine demethylase JMJD5
TGME49_212110	p.Lys365Gln	missense_variant	histone lysine demethylase JMJD5
TGME49_212110	p.Ser231Tyr	missense_variant	histone lysine demethylase JMJD5
TGME49_212110	p.Ser226Tyr	missense_variant	histone lysine demethylase JMJD5
TGME49_212110	p.Leu97Val	missense_variant	histone lysine demethylase JMJD5
TGME49_212110	c.-294A>G	5_prime_UTR_premature_start_codon_gain_variant	histone lysine demethylase JMJD5
TGME49_211730	p.Pro106Ser	missense_variant	histone lysine methyltransferase SET8
TGME49_211730	p.Glu228Asp	missense_variant	histone lysine methyltransferase SET8
TGME49_211730	p.Ser234Asn	missense_variant	histone lysine methyltransferase SET8
TGME49_211730	p.Asp274Glu	missense_variant	histone lysine methyltransferase SET8
TGME49_211730	p.Asn761Thr	missense_variant	histone lysine methyltransferase SET8
TGME49_211730	p.Gly1421Arg	missense_variant	histone lysine methyltransferase SET8
TGME49_211730	p.Gly1470Arg	missense_variant	histone lysine methyltransferase SET8
TGME49_319662	c.-217G>C	5_prime_UTR_premature_start_codon_gain_variant	histone lysine methyltransferase, SET, putative
TGME49_319662	p.Asn12Thr	missense_variant	histone lysine methyltransferase, SET, putative
TGME49_319662	p.Ser94Leu	missense_variant	histone lysine methyltransferase, SET, putative
TGME49_319662	p.Arg126Gly	missense_variant	histone lysine methyltransferase, SET, putative
TGME49_319662	p.Gln274Leu	missense_variant	histone lysine methyltransferase, SET, putative
TGME49_319662	p.Ser356Pro	missense_variant	histone lysine methyltransferase, SET, putative
TGME49_319662	p.Glu368Gly	missense_variant	histone lysine methyltransferase, SET, putative
TGME49_319662	p.Pro380Leu	missense_variant	histone lysine methyltransferase, SET, putative
TGME49_319662	p.Ser449Pro	missense_variant	histone lysine methyltransferase, SET, putative
TGME49_319662	p.Thr474Ala	missense_variant	histone lysine methyltransferase, SET, putative
TGME49_319662	p.Glu619Gly	missense_variant	histone lysine methyltransferase, SET, putative
TGME49_246910	p.Glu448Gln	missense_variant	histone lysine methyltransferase, SET, putative
TGME49_275420	p.Ser2813Phe	missense_variant	histone lysine-specific demethylase LSD1/BHC110/KDMA1A
TGME49_275420	p.Arg1832His	missense_variant	histone lysine-specific demethylase LSD1/BHC110/KDMA1A
TGME49_275420	p.Asp1337His	missense_variant	histone lysine-specific demethylase LSD1/BHC110/KDMA1A
TGME49_275420	p.Lys1335Glu	missense_variant	histone lysine-specific demethylase LSD1/BHC110/KDMA1A
TGME49_275420	p.Ser637Ile	missense_variant	histone lysine-specific demethylase LSD1/BHC110/KDMA1A
TGME49_275420	p.Asp535Gly	missense_variant	histone lysine-specific demethylase LSD1/BHC110/KDMA1A
TGME49_275420	p.Val338Ala	missense_variant	histone lysine-specific demethylase LSD1/BHC110/KDMA1A
TGME49_275420	p.Pro261Leu	missense_variant	histone lysine-specific demethylase LSD1/BHC110/KDMA1A
TGME49_275420	p.Ser213Pro	missense_variant	histone lysine-specific demethylase LSD1/BHC110/KDMA1A
TGME49_275420	p.Leu91Ile	missense_variant	histone lysine-specific demethylase LSD1/BHC110/KDMA1A
TGME49_252400	p.Thr738Ile	missense_variant	HIT zinc finger protein
TGME49_252400	p.Ala705Val	missense_variant	HIT zinc finger protein
TGME49_252400	p.Ala542Pro	missense_variant	HIT zinc finger protein
TGME49_252400	p.Val312Ile	missense_variant	HIT zinc finger protein
TGME49_252400	p.Ala15Val	missense_variant	HIT zinc finger protein
TGME49_252400	p.Ala15Pro	missense_variant	HIT zinc finger protein
TGME49_217500	c.361-3T>C	splice_region_variant	HMG (high mobility group) box domain-containing protein
TGME49_253070	p.Arg29His	missense_variant	hydrolase, TatD family protein
TGME49_253070	p.Pro168Ala	missense_variant	hydrolase, TatD family protein
TGME49_253070	p.Val185Leu	missense_variant	hydrolase, TatD family protein
TGME49_253070	p.Val185Gly	missense_variant	hydrolase, TatD family protein
TGME49_253070	p.Arg187Gln	missense_variant	hydrolase, TatD family protein
TGME49_253070	p.Val269Phe	missense_variant	hydrolase, TatD family protein
TGME49_253070	c.1598-7T>C	splice_region_variant	hydrolase, TatD family protein
TGME49_253070	p.Glu554Gly	missense_variant	hydrolase, TatD family protein
TGME49_329000	c.2038+1G>T	splice_donor_variant	hypothetical protein
TGME49_329000	p.Thr562Ala	missense_variant	hypothetical protein
TGME49_329000	p.Gly561Val	missense_variant	hypothetical protein
TGME49_329000	p.Asp560Gly	missense_variant	hypothetical protein
TGME49_329000	c.1483+7T>G	splice_region_variant	hypothetical protein
TGME49_217961	p.Ala493Pro	missense_variant	hypothetical protein
TGME49_208400	p.Ala1896Pro	missense_variant	hypothetical protein
TGME49_208400	p.Gly1895Ala	missense_variant	hypothetical protein
TGME49_220880	c.-856C>T	5_prime_UTR_premature_start_codon_gain_variant	hypothetical protein
TGME49_220900	p.Glu56Asp	missense_variant	hypothetical protein
TGME49_220920	c.-359A>G	5_prime_UTR_premature_start_codon_gain_variant	hypothetical protein
TGME49_220920	p.Gly590Ala	missense_variant	hypothetical protein
TGME49_220920	p.Thr949Ala	missense_variant	hypothetical protein
TGME49_220920	p.Ile1064Val	missense_variant	hypothetical protein
TGME49_221180	p.Ala315Thr	missense_variant	hypothetical protein
TGME49_221180	p.Pro239Ser	missense_variant	hypothetical protein
TGME49_221180	p.Val111Gly	missense_variant	hypothetical protein

TGME49_221220	c.1561+4A>G	splice_region_variant	hypothetical protein
TGME49_221220	p.Ser969Pro	missense_variant	hypothetical protein
TGME49_221220	p.Leu1012His	missense_variant	hypothetical protein
TGME49_221220	p.Ala1416Ser	missense_variant	hypothetical protein
TGME49_221220	p.Val1653Ile	missense_variant	hypothetical protein
TGME49_221220	p.Asp1758His	missense_variant	hypothetical protein
TGME49_221230	p.Thr87Ala	missense_variant	hypothetical protein
TGME49_221240	p.Thr86Ile	missense_variant	hypothetical protein
TGME49_221250	c.1094+7G>A	splice_region_variant	hypothetical protein
TGME49_221250	p.Thr471Ser	missense_variant	hypothetical protein
TGME49_221250	p.Val516Ile	missense_variant	hypothetical protein
TGME49_221250	p.His808Asn	missense_variant	hypothetical protein
TGME49_221250	p.Ala818Thr	missense_variant	hypothetical protein
TGME49_221265	p.Gly399Glu	missense_variant	hypothetical protein
TGME49_221265	p.Leu140Ile	missense_variant	hypothetical protein
TGME49_221265	p.Phe97Leu	missense_variant	hypothetical protein
TGME49_221275	p.Gly139Arg	missense_variant	hypothetical protein
TGME49_221275	p.Arg90Leu	missense_variant	hypothetical protein
TGME49_221280	p.Pro113Ser	missense_variant	hypothetical protein
TGME49_221280	p.Ser164Ala	missense_variant	hypothetical protein
TGME49_221280	p.Thr222Arg	missense_variant	hypothetical protein
TGME49_221280	p.Arg262Lys	missense_variant	hypothetical protein
TGME49_221280	p.Lys270Asn	missense_variant	hypothetical protein
TGME49_221280	p.His355Asp	missense_variant	hypothetical protein
TGME49_221280	p.Gly553Val	missense_variant	hypothetical protein
TGME49_221280	p.Gly595Arg	missense_variant	hypothetical protein
TGME49_221280	p.Ile798Thr	missense_variant	hypothetical protein
TGME49_221280	p.Pro848Leu	missense_variant	hypothetical protein
TGME49_221280	p.Arg1322Pro	missense_variant	hypothetical protein
TGME49_221280	p.Leu1409Val	missense_variant	hypothetical protein
TGME49_221280	p.Arg1547Ser	missense_variant	hypothetical protein
TGME49_221280	p.Ala1556Val	missense_variant	hypothetical protein
TGME49_221280	p.Gly1582Arg	missense_variant	hypothetical protein
TGME49_221280	p.Tyr1607Cys	missense_variant	hypothetical protein
TGME49_221280	p.Thr1646Arg	missense_variant	hypothetical protein
TGME49_221280	p.Gly2240Asp	missense_variant	hypothetical protein
TGME49_221280	p.Asp2875Asn	missense_variant	hypothetical protein
TGME49_221280	p.Ala2918Pro	missense_variant	hypothetical protein
TGME49_221280	p.Glu2981Lys	missense_variant	hypothetical protein
TGME49_221280	p.Glu3118Lys	missense_variant	hypothetical protein
TGME49_221280	p.Pro3185Gln	missense_variant	hypothetical protein
TGME49_221280	p.Ser3870Gly	missense_variant	hypothetical protein
TGME49_221360	p.Ile418Val	missense_variant	hypothetical protein
TGME49_221360	c.-82G>C	5_prime_UTR_premature_start_codon_gain_variant	hypothetical protein
TGME49_221360	c.-431C>T	5_prime_UTR_premature_start_codon_gain_variant	hypothetical protein
TGME49_221360	c.-639C>T	5_prime_UTR_premature_start_codon_gain_variant	hypothetical protein
TGME49_221370	c.-974C>T	5_prime_UTR_premature_start_codon_gain_variant	hypothetical protein
TGME49_221370	c.-879C>T	5_prime_UTR_premature_start_codon_gain_variant	hypothetical protein
TGME49_221370	p.Pro628Leu	missense_variant	hypothetical protein
TGME49_221370	p.Thr683Pro	missense_variant	hypothetical protein
TGME49_221370	p.Arg1097Gly	missense_variant	hypothetical protein
TGME49_221370	p.Val1142Ala	missense_variant	hypothetical protein
TGME49_221370	p.Arg1199Cys	missense_variant	hypothetical protein
TGME49_221400	p.Leu1596His	missense_variant	hypothetical protein
TGME49_221400	p.Pro1583Ser	missense_variant	hypothetical protein
TGME49_221400	p.Thr836Pro	missense_variant	hypothetical protein
TGME49_221400	p.Phe824Leu	missense_variant	hypothetical protein
TGME49_221400	p.Glu501Asp	missense_variant	hypothetical protein
TGME49_221400	p.Val16Ala	missense_variant	hypothetical protein
TGME49_221430	p.Ser177Ala	missense_variant	hypothetical protein
TGME49_221430	p.Asp370Glu	missense_variant	hypothetical protein
TGME49_221430	p.Ile602Thr	missense_variant	hypothetical protein
TGME49_221430	p.Arg608Leu	missense_variant	hypothetical protein
TGME49_221455	p.Gln219Lys	missense_variant	hypothetical protein
TGME49_221455	p.Leu233Pro	missense_variant	hypothetical protein
TGME49_221480	p.Met70Ile	missense_variant	hypothetical protein
TGME49_221480	c.-785C>G	5_prime_UTR_premature_start_codon_gain_variant	hypothetical protein
TGME49_221540	c.3083-4G>T	splice_region_variant	hypothetical protein
TGME49_221540	p.Phe934Leu	missense_variant	hypothetical protein
TGME49_221540	p.Gln765Lys	missense_variant	hypothetical protein
TGME49_221540	p.Pro564Ser	missense_variant	hypothetical protein
TGME49_221540	c.-338G>T	5_prime_UTR_premature_start_codon_gain_variant	hypothetical protein
TGME49_221560	p.Asn106Thr	missense_variant	hypothetical protein
TGME49_221585	p.Ser89Leu	missense_variant	hypothetical protein
TGME49_221585	p.Phe80Ser	missense_variant	hypothetical protein
TGME49_221585	p.Pro23Leu	missense_variant	hypothetical protein
TGME49_221600	p.Gln852His	missense_variant	hypothetical protein
TGME49_221600	p.Gly525Glu	missense_variant	hypothetical protein
TGME49_221600	p.Gln501His	missense_variant	hypothetical protein
TGME49_221600	p.Gln501Leu	missense_variant	hypothetical protein
TGME49_221600	p.Gln225Arg	missense_variant	hypothetical protein
TGME49_221600	p.Pro222Gln	missense_variant	hypothetical protein
TGME49_221600	p.Gly61Arg	missense_variant	hypothetical protein
TGME49_221600	p.Ala51Ser	missense_variant	hypothetical protein
TGME49_221600	c.-395T>C	5_prime_UTR_premature_start_codon_gain_variant	hypothetical protein

TGME49_221630	p.Gly1140Arg	missense_variant	hypothetical protein
TGME49_221630	p.Asn1096Tyr	missense_variant	hypothetical protein
TGME49_221630	p.Asn1021Ser	missense_variant	hypothetical protein
TGME49_221630	p.Asp491Ala	missense_variant	hypothetical protein
TGME49_221630	p.Glu386Asp	missense_variant	hypothetical protein
TGME49_221630	c.-1833A>T	5_prime_UTR_premature_start_codon_gain_variant	hypothetical protein
TGME49_221640	p.Arg425Trp	missense_variant	hypothetical protein
TGME49_221640	p.Ala674Ser	missense_variant	hypothetical protein
TGME49_221675	p.Ala1119Val	missense_variant	hypothetical protein
TGME49_221675	p.His143Tyr	missense_variant	hypothetical protein
TGME49_221685	c.-1416C>G	5_prime_UTR_premature_start_codon_gain_variant	hypothetical protein
TGME49_221685	p.His127Tyr	missense_variant	hypothetical protein
TGME49_221690	p.Asp90Gly	missense_variant	hypothetical protein
TGME49_221720	p.Pro3083Leu	missense_variant	hypothetical protein
TGME49_221720	p.Pro2833Ala	missense_variant	hypothetical protein
TGME49_221720	p.Val2609Glu	missense_variant	hypothetical protein
TGME49_221720	p.Leu2599Phe	missense_variant	hypothetical protein
TGME49_221720	p.Leu2578Phe	missense_variant	hypothetical protein
TGME49_221720	p.Leu2171Ser	missense_variant	hypothetical protein
TGME49_221720	p.Ala1569Gly	missense_variant	hypothetical protein
TGME49_221720	p.Asn1408Ser	missense_variant	hypothetical protein
TGME49_221720	p.Gly1259Ala	missense_variant	hypothetical protein
TGME49_221720	p.Ser1087Leu	missense_variant	hypothetical protein
TGME49_221720	p.Pro1029Ser	missense_variant	hypothetical protein
TGME49_221720	p.Arg220*	stop_gained	hypothetical protein
TGME49_221840	c.-179A>T	5_prime_UTR_premature_start_codon_gain_variant	hypothetical protein
TGME49_221870	p.Val42Ile	missense_variant	hypothetical protein
TGME49_221870	p.Ala65Asp	missense_variant	hypothetical protein
TGME49_221870	p.Ala103Thr	missense_variant	hypothetical protein
TGME49_221870	p.Arg181Pro	missense_variant	hypothetical protein
TGME49_221870	p.Arg387Ser	missense_variant	hypothetical protein
TGME49_221870	p.Ala1057Thr	missense_variant	hypothetical protein
TGME49_221880	p.Ser584Pro	missense_variant	hypothetical protein
TGME49_221880	p.Glu328Asp	missense_variant	hypothetical protein
TGME49_221880	p.Arg272Lys	missense_variant	hypothetical protein
TGME49_221880	p.Glu238Gly	missense_variant	hypothetical protein
TGME49_221880	c.166-3C>T	splice_region_variant	hypothetical protein
TGME49_221880	p.Val30Ala	missense_variant	hypothetical protein
TGME49_221880	c.-257T>C	5_prime_UTR_premature_start_codon_gain_variant	hypothetical protein
TGME49_221895	p.His186Gln	missense_variant	hypothetical protein
TGME49_221895	p.Pro44Ser	missense_variant	hypothetical protein
TGME49_221818	p.Tyr225His	missense_variant	hypothetical protein
TGME49_221990	p.Leu376Val	missense_variant	hypothetical protein
TGME49_221990	p.Arg96Met	missense_variant	hypothetical protein
TGME49_221990	p.Thr92Met	missense_variant	hypothetical protein
TGME49_221990	p.Ala38Gly	missense_variant	hypothetical protein
TGME49_222030	p.Ala109Ser	missense_variant	hypothetical protein
TGME49_222030	p.Leu233Phe	missense_variant	hypothetical protein
TGME49_222030	p.Gly248Ser	missense_variant	hypothetical protein
TGME49_222030	p.Ser267Pro	missense_variant	hypothetical protein
TGME49_222030	p.Ala833Thr	missense_variant	hypothetical protein
TGME49_222030	p.Cys1208Tyr	missense_variant	hypothetical protein
TGME49_222050	p.Ser1151Asn	missense_variant	hypothetical protein
TGME49_222050	p.Tyr970Cys	missense_variant	hypothetical protein
TGME49_222050	p.Thr853Met	missense_variant	hypothetical protein
TGME49_222050	p.Ala847Thr	missense_variant	hypothetical protein
TGME49_222050	p.Gly734Arg	missense_variant	hypothetical protein
TGME49_222050	p.Gly352Arg	missense_variant	hypothetical protein
TGME49_222050	p.Pro52Leu	missense_variant	hypothetical protein
TGME49_222050	p.Pro52Ser	missense_variant	hypothetical protein
TGME49_222060	p.Pro854Arg	missense_variant	hypothetical protein
TGME49_222060	p.Ser203Ala	missense_variant	hypothetical protein
TGME49_222060	p.Phe116Val	missense_variant	hypothetical protein
TGME49_222060	p.Pro81Ser	missense_variant	hypothetical protein
TGME49_222080	c.390-3C>T	splice_region_variant	hypothetical protein
TGME49_222120	p.Ala956Val	missense_variant	hypothetical protein
TGME49_222120	p.Ala791Thr	missense_variant	hypothetical protein
TGME49_222120	p.Arg629Cys	missense_variant	hypothetical protein
TGME49_222120	p.Ser473Pro	missense_variant	hypothetical protein
TGME49_222120	p.Ala391Thr	missense_variant	hypothetical protein
TGME49_222120	p.Gly150Ser	missense_variant	hypothetical protein
TGME49_222130	c.-307T>C	5_prime_UTR_premature_start_codon_gain_variant	hypothetical protein
TGME49_222150	p.Ile33Thr	missense_variant	hypothetical protein
TGME49_222155	c.-41G>A	5_prime_UTR_premature_start_codon_gain_variant	hypothetical protein
TGME49_222155	c.-21C>T	5_prime_UTR_premature_start_codon_gain_variant	hypothetical protein
TGME49_222155	p.Arg78His	missense_variant	hypothetical protein
TGME49_222155	p.Lys201Glu	missense_variant	hypothetical protein
TGME49_222180	p.Glu83Asp	missense_variant	hypothetical protein
TGME49_222180	p.Val58Ile	missense_variant	hypothetical protein
TGME49_222192	c.-344A>T	5_prime_UTR_premature_start_codon_gain_variant	hypothetical protein
TGME49_222192	c.547+8A>G	splice_region_variant	hypothetical protein
TGME49_222230	p.Gly1252Asp	missense_variant	hypothetical protein
TGME49_222230	p.Gln1203Glu	missense_variant	hypothetical protein
TGME49_222230	p.Gly435Glu	missense_variant	hypothetical protein
TGME49_222230	p.Asn402Lys	missense_variant	hypothetical protein

TGME49_222230	p.Arg43Lys	missense_variant	hypothetical protein
TGME49_222230	p.Met21Ile	missense_variant	hypothetical protein
TGME49_222240	p.Pro406Ser	missense_variant	hypothetical protein
TGME49_222240	p.Ser633Pro	missense_variant	hypothetical protein
TGME49_222240	p.Met663Arg	missense_variant	hypothetical protein
TGME49_222240	p.Ala877Val	missense_variant	hypothetical protein
TGME49_222245	c.-520G>A	5_prime_UTR_premature_start_codon_gain_variant	hypothetical protein
TGME49_222245	p.Glu99Lys	missense_variant	hypothetical protein
TGME49_222250	p.Glu1327Asp	missense_variant	hypothetical protein
TGME49_222250	p.Arg1309Lys	missense_variant	hypothetical protein
TGME49_222250	p.Val1148Ala	missense_variant	hypothetical protein
TGME49_222250	p.Ser962Tyr	missense_variant	hypothetical protein
TGME49_222250	p.Glu932Gly	missense_variant	hypothetical protein
TGME49_222250	p.Thr846Ala	missense_variant	hypothetical protein
TGME49_222250	p.Ile845Val	missense_variant	hypothetical protein
TGME49_222250	p.Ala835Pro	missense_variant	hypothetical protein
TGME49_222250	p.Thr746Ala	missense_variant	hypothetical protein
TGME49_222250	p.Ile271Val	missense_variant	hypothetical protein
TGME49_222250	p.Ala52Gly	missense_variant	hypothetical protein
TGME49_222270	p.Phe6Ser	missense_variant	hypothetical protein
TGME49_222280	p.Ala64Val	missense_variant	hypothetical protein
TGME49_222300	c.2964-5G>C	splice_region_variant	hypothetical protein
TGME49_222300	p.Leu884Ile	missense_variant	hypothetical protein
TGME49_222300	p.Met564Thr	missense_variant	hypothetical protein
TGME49_222300	p.Ser561Pro	missense_variant	hypothetical protein
TGME49_222300	p.Ser446Thr	missense_variant	hypothetical protein
TGME49_222300	p.Ser446Gly	missense_variant	hypothetical protein
TGME49_222300	p.Ile422Thr	missense_variant	hypothetical protein
TGME49_222300	p.Gly298Asp	missense_variant	hypothetical protein
TGME49_222300	p.His196Arg	missense_variant	hypothetical protein
TGME49_222300	p.Ala122Val	missense_variant	hypothetical protein
TGME49_222305	c.92+4G>A	splice_region_variant	hypothetical protein
TGME49_222310	p.Asn446Ser	missense_variant	hypothetical protein
TGME49_222330	p.Arg1966Lys	missense_variant	hypothetical protein
TGME49_222330	p.Ser1880Pro	missense_variant	hypothetical protein
TGME49_222330	p.His1688Arg	missense_variant	hypothetical protein
TGME49_222330	p.Ser1460Phe	missense_variant	hypothetical protein
TGME49_222330	p.Trp1305Cys	missense_variant	hypothetical protein
TGME49_222330	p.Ser1046Asn	missense_variant	hypothetical protein
TGME49_222330	p.Ser934Asn	missense_variant	hypothetical protein
TGME49_222330	p.Ile252Val	missense_variant	hypothetical protein
TGME49_222330	p.Leu112Phe	missense_variant	hypothetical protein
TGME49_222360	c.-399T>C	5_prime_UTR_premature_start_codon_gain_variant	hypothetical protein
TGME49_222360	p.Ser647Thr	missense_variant	hypothetical protein
TGME49_222390	p.Phe257Tyr	missense_variant	hypothetical protein
TGME49_222390	p.Ser82Thr	missense_variant	hypothetical protein
TGME49_222390	c.-142C>T	5_prime_UTR_premature_start_codon_gain_variant	hypothetical protein
TGME49_222400	p.Asp9Glu	missense_variant	hypothetical protein
TGME49_222420	p.Ser156Pro	missense_variant	hypothetical protein
TGME49_222420	p.Val314Met	missense_variant	hypothetical protein
TGME49_222420	p.Phe460Val	missense_variant	hypothetical protein
TGME49_222440	p.Arg197Leu	missense_variant	hypothetical protein
TGME49_222440	p.Val145Phe	missense_variant	hypothetical protein
TGME49_222700	p.Val128Ile	missense_variant	hypothetical protein
TGME49_222700	p.His357Arg	missense_variant	hypothetical protein
TGME49_222870	c.483+4C>T	splice_region_variant	hypothetical protein
TGME49_222880	p.Asn29Asp	missense_variant	hypothetical protein
TGME49_222880	p.Leu178Val	missense_variant	hypothetical protein
TGME49_222880	p.Glu344Lys	missense_variant	hypothetical protein
TGME49_222880	c.1252-7C>A	splice_region_variant	hypothetical protein
TGME49_222930	p.Pro80Leu	missense_variant	hypothetical protein
TGME49_222935	c.-27G>T	5_prime_UTR_premature_start_codon_gain_variant	hypothetical protein
TGME49_222940	p.Leu18Arg	missense_variant	hypothetical protein
TGME49_222975	p.Gly350Arg	missense_variant	hypothetical protein
TGME49_223070	p.Thr161Ser	missense_variant	hypothetical protein
TGME49_223080	p.Ile2787Val	missense_variant	hypothetical protein
TGME49_223080	p.Thr2534Ser	missense_variant	hypothetical protein
TGME49_223080	p.Gly2380Val	missense_variant	hypothetical protein
TGME49_223080	p.Gln1553Leu	missense_variant	hypothetical protein
TGME49_223080	p.Ser984Asn	missense_variant	hypothetical protein
TGME49_223080	p.Arg444Gly	missense_variant	hypothetical protein
TGME49_223080	p.His181Tyr	missense_variant	hypothetical protein
TGME49_223080	p.Ala140Pro	missense_variant	hypothetical protein
TGME49_223080	p.Gly67Arg	missense_variant	hypothetical protein
TGME49_223080	c.-3585A>G	5_prime_UTR_premature_start_codon_gain_variant	hypothetical protein
TGME49_223095	p.Thr705Ile	missense_variant	hypothetical protein
TGME49_223095	p.Gly633Arg	missense_variant	hypothetical protein
TGME49_223095	p.Ser605Pro	missense_variant	hypothetical protein
TGME49_223095	p.Asp186Asn	missense_variant	hypothetical protein
TGME49_223095	p.Glu120Ala	missense_variant	hypothetical protein
TGME49_223095	p.Glu120*	stop_gained	hypothetical protein
TGME49_223130	p.Gly279Asp	missense_variant	hypothetical protein
TGME49_296950	c.211-6T>C	splice_region_variant	hypothetical protein
TGME49_297090	p.Val19Ala	missense_variant	hypothetical protein
TGME49_297100	p.Thr46Met	missense_variant	hypothetical protein

TGME49_297120	p.Tyr8His	missense_variant	hypothetical protein
TGME49_297120	p.Leu62*	stop_gained	hypothetical protein
TGME49_297130	c.-1488C>T	5_prime_UTR_premature_start_codon_gain_variant	hypothetical protein
TGME49_297160	c.842+8A>C	splice_region_variant	hypothetical protein
TGME49_297180	p.Glu773Ala	missense_variant	hypothetical protein
TGME49_297180	p.Asn621Ser	missense_variant	hypothetical protein
TGME49_297190	p.Glu9Lys	missense_variant	hypothetical protein
TGME49_297210	p.Gly1062Asp	missense_variant	hypothetical protein
TGME49_297210	p.Ser1461Cys	missense_variant	hypothetical protein
TGME49_297210	p.Arg1487Pro	missense_variant	hypothetical protein
TGME49_297210	p.Ser2089Thr	missense_variant	hypothetical protein
TGME49_297210	p.Glu2968Gly	missense_variant	hypothetical protein
TGME49_297210	p.Ser3072Asn	missense_variant	hypothetical protein
TGME49_297210	p.Ala3722Ser	missense_variant	hypothetical protein
TGME49_297210	p.Arg3895Lys	missense_variant	hypothetical protein
TGME49_297270	p.Ser71Ala	missense_variant	hypothetical protein
TGME49_297270	c.1098+8C>T	splice_region_variant	hypothetical protein
TGME49_297280	p.Thr62Met	missense_variant	hypothetical protein
TGME49_297280	p.Arg29His	missense_variant	hypothetical protein
TGME49_297280	c.-2300C>T	5_prime_UTR_premature_start_codon_gain_variant	hypothetical protein
TGME49_297290	p.Lys234Asn	missense_variant	hypothetical protein
TGME49_297310	c.-867A>G	5_prime_UTR_premature_start_codon_gain_variant	hypothetical protein
TGME49_297310	p.Leu179Arg	missense_variant	hypothetical protein
TGME49_297310	p.Ala271Ser	missense_variant	hypothetical protein
TGME49_297320	p.Asp810Ala	missense_variant	hypothetical protein
TGME49_297320	c.-319C>T	5_prime_UTR_premature_start_codon_gain_variant	hypothetical protein
TGME49_297320	c.-337G>A	5_prime_UTR_premature_start_codon_gain_variant	hypothetical protein
TGME49_297320	c.-668C>G	5_prime_UTR_premature_start_codon_gain_variant	hypothetical protein
TGME49_297330	p.Gln3158*	stop_gained	hypothetical protein
TGME49_297330	p.Gly2765Glu	missense_variant	hypothetical protein
TGME49_297330	p.Ala2533Ser	missense_variant	hypothetical protein
TGME49_297330	p.Cys2185Arg	missense_variant	hypothetical protein
TGME49_297330	p.Gly2086Arg	missense_variant	hypothetical protein
TGME49_297330	p.Ser2052Asn	missense_variant	hypothetical protein
TGME49_297330	p.Glu1958Lys	missense_variant	hypothetical protein
TGME49_297330	p.Glu1833Asp	missense_variant	hypothetical protein
TGME49_297330	p.His1715Arg	missense_variant	hypothetical protein
TGME49_297330	p.Gly1013Arg	missense_variant	hypothetical protein
TGME49_297330	p.Asn772Thr	missense_variant	hypothetical protein
TGME49_297330	p.Arg594Gly	missense_variant	hypothetical protein
TGME49_297330	p.Leu519Val	missense_variant	hypothetical protein
TGME49_297330	p.Leu338Arg	missense_variant	hypothetical protein
TGME49_297340	p.Arg8Cys	missense_variant	hypothetical protein
TGME49_297340	p.Ile112Leu	missense_variant	hypothetical protein
TGME49_297340	p.Ser267Leu	missense_variant	hypothetical protein
TGME49_297340	p.Thr606Ser	missense_variant	hypothetical protein
TGME49_297340	p.Ala644Thr	missense_variant	hypothetical protein
TGME49_297340	p.Ala777Thr	missense_variant	hypothetical protein
TGME49_297340	p.Arg1052Cys	missense_variant	hypothetical protein
TGME49_297360	p.Thr962Ala	missense_variant	hypothetical protein
TGME49_297380	p.Gly338Arg	missense_variant	hypothetical protein
TGME49_297380	p.Gln246His	missense_variant	hypothetical protein
TGME49_297380	p.Arg25Gln	missense_variant	hypothetical protein
TGME49_297390	p.Arg244Lys	missense_variant	hypothetical protein
TGME49_297430	p.Ser12Gly	missense_variant	hypothetical protein
TGME49_297440	p.Ile40Thr	missense_variant	hypothetical protein
TGME49_297488	p.Met44Ile	missense_variant	hypothetical protein
TGME49_297492	p.Ala143Thr	missense_variant	hypothetical protein
TGME49_297492	p.Gln29*	stop_gained	hypothetical protein
TGME49_297495	c.346+6C>T	splice_region_variant	hypothetical protein
TGME49_297495	c.346+4A>C	splice_region_variant	hypothetical protein
TGME49_297495	p.Ala102Thr	missense_variant	hypothetical protein
TGME49_297495	p.Asp56Ala	missense_variant	hypothetical protein
TGME49_297505	p.Gly18Ala	missense_variant	hypothetical protein
TGME49_297505	p.Arg24Gln	missense_variant	hypothetical protein
TGME49_297510	c.-866C>G	5_prime_UTR_premature_start_codon_gain_variant	hypothetical protein
TGME49_297510	c.-518C>T	5_prime_UTR_premature_start_codon_gain_variant	hypothetical protein
TGME49_297510	p.Ser94Leu	missense_variant	hypothetical protein
TGME49_297643	p.Gly118Arg	missense_variant	hypothetical protein
TGME49_297647	p.Arg112*	stop_gained	hypothetical protein
TGME49_297647	p.Ile85Thr	missense_variant	hypothetical protein
TGME49_297647	c.-1682T>C	5_prime_UTR_premature_start_codon_gain_variant	hypothetical protein
TGME49_297660	c.-152C>T	5_prime_UTR_premature_start_codon_gain_variant	hypothetical protein
TGME49_297660	p.Thr91Ala	missense_variant	hypothetical protein
TGME49_297660	p.Gly145Glu	missense_variant	hypothetical protein
TGME49_297690	p.Thr1935Ala	missense_variant	hypothetical protein
TGME49_297690	p.Asp1879Gly	missense_variant	hypothetical protein
TGME49_297690	p.Asn1873Ser	missense_variant	hypothetical protein
TGME49_297690	p.Gln1734Arg	missense_variant	hypothetical protein
TGME49_297690	p.Ser1299Leu	missense_variant	hypothetical protein
TGME49_297690	p.Phe1048Leu	missense_variant	hypothetical protein
TGME49_297690	p.Ala826Thr	missense_variant	hypothetical protein
TGME49_297690	p.Ala705Thr	missense_variant	hypothetical protein
TGME49_297690	p.Cys357Tyr	missense_variant	hypothetical protein
TGME49_297700	p.Val74Ala	missense_variant	hypothetical protein

TGME49_297710	c.-273G>C	5_prime_UTR_premature_start_codon_gain_variant	hypothetical protein
TGME49_297710	p.Leu440Pro	missense_variant	hypothetical protein
TGME49_297710	p.Thr498Ala	missense_variant	hypothetical protein
TGME49_297725	c.196+2T>A	splice_donor_variant	hypothetical protein
TGME49_297745	p.Gly3795Ala	missense_variant	hypothetical protein
TGME49_297745	p.Leu3468Phe	missense_variant	hypothetical protein
TGME49_297745	p.Thr3368Ala	missense_variant	hypothetical protein
TGME49_297745	p.Thr3223Ser	missense_variant	hypothetical protein
TGME49_297745	p.Gly3203Val	missense_variant	hypothetical protein
TGME49_297745	p.Leu2409Val	missense_variant	hypothetical protein
TGME49_297745	p.Glu1883Val	missense_variant	hypothetical protein
TGME49_297745	p.Asp967Glu	missense_variant	hypothetical protein
TGME49_297745	p.Ser39Phe	missense_variant	hypothetical protein
TGME49_297760	p.Gly22Arg	missense_variant	hypothetical protein
TGME49_297770	c.-2338C>T	5_prime_UTR_premature_start_codon_gain_variant	hypothetical protein
TGME49_297770	c.-2320G>C	5_prime_UTR_premature_start_codon_gain_variant	hypothetical protein
TGME49_297770	c.-2145C>T	5_prime_UTR_premature_start_codon_gain_variant	hypothetical protein
TGME49_297770	c.-815A>T	5_prime_UTR_premature_start_codon_gain_variant	hypothetical protein
TGME49_297770	p.His98Gln	missense_variant	hypothetical protein
TGME49_297770	p.Ser158Ala	missense_variant	hypothetical protein
TGME49_297770	p.Asp208Asn	missense_variant	hypothetical protein
TGME49_297770	p.Ala245Thr	missense_variant	hypothetical protein
TGME49_297770	p.Pro433Ser	missense_variant	hypothetical protein
TGME49_297790	p.Ser63Ala	missense_variant	hypothetical protein
TGME49_297810	p.Arg83Pro	missense_variant	hypothetical protein
TGME49_297830	p.Gln1285Glu	missense_variant	hypothetical protein
TGME49_297830	p.Glu700Lys	missense_variant	hypothetical protein
TGME49_297830	p.Ser669Gly	missense_variant	hypothetical protein
TGME49_297830	p.Ala588Ser	missense_variant	hypothetical protein
TGME49_297845	p.His59Tyr	missense_variant	hypothetical protein
TGME49_297845	p.Thr189Met	missense_variant	hypothetical protein
TGME49_297870	p.Thr527Lys	missense_variant	hypothetical protein
TGME49_297870	p.Gln459His	missense_variant	hypothetical protein
TGME49_297870	p.Pro208Ser	missense_variant	hypothetical protein
TGME49_297870	p.Ser129Trp	missense_variant	hypothetical protein
TGME49_297890	p.Leu979Val	missense_variant	hypothetical protein
TGME49_297890	p.Pro1009Ser	missense_variant	hypothetical protein
TGME49_297890	p.Pro1009Leu	missense_variant	hypothetical protein
TGME49_297890	p.Ser1288Pro	missense_variant	hypothetical protein
TGME49_297890	p.Arg1293Gly	missense_variant	hypothetical protein
TGME49_297890	p.Glu1477Lys	missense_variant	hypothetical protein
TGME49_297900	p.Lys112Asn	missense_variant	hypothetical protein
TGME49_297900	p.Arg380Ser	missense_variant	hypothetical protein
TGME49_297910	p.Thr211Ile	missense_variant	hypothetical protein
TGME49_297910	p.Asn175Ser	missense_variant	hypothetical protein
TGME49_297920	p.Val23Ala	missense_variant	hypothetical protein
TGME49_297920	p.Thr135Asn	missense_variant	hypothetical protein
TGME49_297920	p.Ser161Thr	missense_variant	hypothetical protein
TGME49_297980	c.-149G>T	5_prime_UTR_premature_start_codon_gain_variant	hypothetical protein
TGME49_298010	p.Gln1977Lys	missense_variant	hypothetical protein
TGME49_298010	c.5672-8T>C	splice_region_variant	hypothetical protein
TGME49_298010	p.Thr235Lys	missense_variant	hypothetical protein
TGME49_298040	p.Val520Ala	missense_variant	hypothetical protein
TGME49_298040	p.Ala140Thr	missense_variant	hypothetical protein
TGME49_298050	c.-190C>G	5_prime_UTR_premature_start_codon_gain_variant	hypothetical protein
TGME49_298050	p.Asn391Ser	missense_variant	hypothetical protein
TGME49_275830	p.Lys351Asn	missense_variant	hypothetical protein
TGME49_275830	p.Ala352Pro	missense_variant	hypothetical protein
TGME49_275830	p.Glu363Gly	missense_variant	hypothetical protein
TGME49_275830	p.Ser911Pro	missense_variant	hypothetical protein
TGME49_275830	p.Pro912Ser	missense_variant	hypothetical protein
TGME49_275830	p.Gly989Asp	missense_variant	hypothetical protein
TGME49_275830	p.Lys1037Glu	missense_variant	hypothetical protein
TGME49_275780	p.Gly655Asp	missense_variant	hypothetical protein
TGME49_275780	p.Ser196Pro	missense_variant	hypothetical protein
TGME49_275780	p.Glu141Asp	missense_variant	hypothetical protein
TGME49_275780	p.Pro73Ser	missense_variant	hypothetical protein
TGME49_275760	c.-661C>T	5_prime_UTR_premature_start_codon_gain_variant	hypothetical protein
TGME49_275760	p.Arg44Leu	missense_variant	hypothetical protein
TGME49_275760	p.Arg101Leu	missense_variant	hypothetical protein
TGME49_275740	p.Gly30Arg	missense_variant	hypothetical protein
TGME49_275740	p.Glu675Val	missense_variant	hypothetical protein
TGME49_275740	p.Leu958Phe	missense_variant	hypothetical protein
TGME49_275740	p.Ala1173Val	missense_variant	hypothetical protein
TGME49_275740	p.Thr1190Pro	missense_variant	hypothetical protein
TGME49_275740	p.His1317Gln	missense_variant	hypothetical protein
TGME49_275740	p.Glu1419Asp	missense_variant	hypothetical protein
TGME49_275710	p.Ser441Pro	missense_variant	hypothetical protein
TGME49_275710	p.Ala188Gly	missense_variant	hypothetical protein
TGME49_275710	p.Arg123Gln	missense_variant	hypothetical protein
TGME49_275700	p.Arg196Leu	missense_variant	hypothetical protein
TGME49_275680	c.-1159A>T	5_prime_UTR_premature_start_codon_gain_variant	hypothetical protein
TGME49_275680	p.Asp195Gly	missense_variant	hypothetical protein
TGME49_275680	p.Gln279Arg	missense_variant	hypothetical protein
TGME49_275640	c.-323G>A	5_prime_UTR_premature_start_codon_gain_variant	hypothetical protein

TGME49_252280	p.Arg1431Ser	missense_variant	hypothetical protein
TGME49_252280	p.Ile1304Lys	missense_variant	hypothetical protein
TGME49_252280	p.Ser1190Ala	missense_variant	hypothetical protein
TGME49_252280	p.Glu674Gln	missense_variant	hypothetical protein
TGME49_252280	p.Gly486Arg	missense_variant	hypothetical protein
TGME49_252280	p.Glu459Ala	missense_variant	hypothetical protein
TGME49_252280	c.-679A>G	5_prime_UTR_premature_start_codon_gain_variant	hypothetical protein
TGME49_252295	c.-666G>T	5_prime_UTR_premature_start_codon_gain_variant	hypothetical protein
TGME49_252295	c.-176C>T	5_prime_UTR_premature_start_codon_gain_variant	hypothetical protein
TGME49_252295	p.Pro7Leu	missense_variant	hypothetical protein
TGME49_252295	p.Arg14Leu	missense_variant	hypothetical protein
TGME49_252295	p.Ala127Pro	missense_variant	hypothetical protein
TGME49_252300	p.Ala281Gly	missense_variant	hypothetical protein
TGME49_252300	p.Leu278Phe	missense_variant	hypothetical protein
TGME49_252300	p.Ala274Val	missense_variant	hypothetical protein
TGME49_252300	p.Thr221Ala	missense_variant	hypothetical protein
TGME49_252300	p.Val205Ala	missense_variant	hypothetical protein
TGME49_252300	p.Asp73Ala	missense_variant	hypothetical protein
TGME49_252300	p.Thr30Pro	missense_variant	hypothetical protein
TGME49_252310	p.Ala78Val	missense_variant	hypothetical protein
TGME49_252310	p.Thr33Asn	missense_variant	hypothetical protein
TGME49_252310	p.Thr17Pro	missense_variant	hypothetical protein
TGME49_252310	c.-139C>T	5_prime_UTR_premature_start_codon_gain_variant	hypothetical protein
TGME49_252310	c.-364A>T	5_prime_UTR_premature_start_codon_gain_variant	hypothetical protein
TGME49_252330	p.Phe303Cys	missense_variant	hypothetical protein
TGME49_252340	p.Thr67Ser	missense_variant	hypothetical protein
TGME49_252340	p.Gly1067Glu	missense_variant	hypothetical protein
TGME49_252340	p.Asp1151Gly	missense_variant	hypothetical protein
TGME49_252350	c.-1236G>T	5_prime_UTR_premature_start_codon_gain_variant	hypothetical protein
TGME49_252380	p.Ser1186Gly	missense_variant	hypothetical protein
TGME49_252380	p.Arg1006Gly	missense_variant	hypothetical protein
TGME49_252380	p.Val888Ala	missense_variant	hypothetical protein
TGME49_252380	p.Lys813Asn	missense_variant	hypothetical protein
TGME49_252380	p.Gln749Lys	missense_variant	hypothetical protein
TGME49_252380	p.Ala699Thr	missense_variant	hypothetical protein
TGME49_252380	c.866+7C>T	splice_region_variant	hypothetical protein
TGME49_252380	c.725-7T>C	splice_region_variant	hypothetical protein
TGME49_252380	p.Arg21Gln	missense_variant	hypothetical protein
TGME49_252385	p.Ter122Serext*?	stop_lost	hypothetical protein
TGME49_252390	c.-1575G>T	5_prime_UTR_premature_start_codon_gain_variant	hypothetical protein
TGME49_252395	p.Arg55Leu	missense_variant	hypothetical protein
TGME49_252395	p.Glu665Ala	missense_variant	hypothetical protein
TGME49_252395	p.Gln729Glu	missense_variant	hypothetical protein
TGME49_252395	p.Met1080Val	missense_variant	hypothetical protein
TGME49_252395	p.Asn1354Ser	missense_variant	hypothetical protein
TGME49_252395	p.Leu1402Pro	missense_variant	hypothetical protein
TGME49_252395	p.Thr1672Ala	missense_variant	hypothetical protein
TGME49_252430	c.-2772G>A	5_prime_UTR_premature_start_codon_gain_variant	hypothetical protein
TGME49_252430	c.-2185C>T	5_prime_UTR_premature_start_codon_gain_variant	hypothetical protein
TGME49_252430	c.-556C>A	5_prime_UTR_premature_start_codon_gain_variant	hypothetical protein
TGME49_252430	c.-51C>G	5_prime_UTR_premature_start_codon_gain_variant	hypothetical protein
TGME49_252430	p.Ala30Val	missense_variant	hypothetical protein
TGME49_252450	p.Glu200Lys	missense_variant	hypothetical protein
TGME49_252450	p.Met210Leu	missense_variant	hypothetical protein
TGME49_252450	p.Met210Ile	missense_variant	hypothetical protein
TGME49_252480	c.-213C>T	5_prime_UTR_premature_start_codon_gain_variant	hypothetical protein
TGME49_252480	p.Glu276Gln	missense_variant	hypothetical protein
TGME49_252510	c.124+5C>G	splice_region_variant	hypothetical protein
TGME49_252630	p.Gly109Cys	missense_variant	hypothetical protein
TGME49_252630	c.-668T>G	5_prime_UTR_premature_start_codon_gain_variant	hypothetical protein
TGME49_252630	c.-846A>T	5_prime_UTR_premature_start_codon_gain_variant	hypothetical protein
TGME49_252870	c.-336C>G	5_prime_UTR_premature_start_codon_gain_variant	hypothetical protein
TGME49_252870	p.Tyr117His	missense_variant	hypothetical protein
TGME49_252870	p.Pro118Arg	missense_variant	hypothetical protein
TGME49_252870	p.Gly286Val	missense_variant	hypothetical protein
TGME49_252870	p.Ala643Thr	missense_variant	hypothetical protein
TGME49_252870	p.Ala760Thr	missense_variant	hypothetical protein
TGME49_252870	p.Ser1200Leu	missense_variant	hypothetical protein
TGME49_252870	p.Ala1229Glu	missense_variant	hypothetical protein
TGME49_252880	p.Val2341Ala	missense_variant	hypothetical protein
TGME49_252880	p.Lys2209Arg	missense_variant	hypothetical protein
TGME49_252880	p.Asn2089Thr	missense_variant	hypothetical protein
TGME49_252880	p.Lys1951Arg	missense_variant	hypothetical protein
TGME49_252880	p.Gly1899Ala	missense_variant	hypothetical protein
TGME49_252880	p.Val1581Leu	missense_variant	hypothetical protein
TGME49_252880	p.Thr1562Met	missense_variant	hypothetical protein
TGME49_252880	p.Ala1461Thr	missense_variant	hypothetical protein
TGME49_252880	p.Met1414Thr	missense_variant	hypothetical protein
TGME49_252880	p.Ala1137Gly	missense_variant	hypothetical protein
TGME49_252880	p.Ala709Ser	missense_variant	hypothetical protein
TGME49_252880	p.Pro408Ser	missense_variant	hypothetical protein
TGME49_252880	p.Ala311Pro	missense_variant	hypothetical protein
TGME49_252890	p.Phe7Leu	missense_variant	hypothetical protein
TGME49_252890	p.Ser21Pro	missense_variant	hypothetical protein
TGME49_252990	p.Ser289Pro	missense_variant	hypothetical protein

TGME49_252990	p.Ser205Cys	missense_variant	hypothetical protein
TGME49_252990	p.Ala162Val	missense_variant	hypothetical protein
TGME49_253020	p.Ala36Thr	missense_variant	hypothetical protein
TGME49_253020	p.Arg3Ser	missense_variant	hypothetical protein
TGME49_253020	c.-572C>T	5_prime_UTR_premature_start_codon_gain_variant	hypothetical protein
TGME49_253020	c.-1431T>C	5_prime_UTR_premature_start_codon_gain_variant	hypothetical protein
TGME49_253020	c.-1530C>T	5_prime_UTR_premature_start_codon_gain_variant	hypothetical protein
TGME49_253050	c.-13C>G	5_prime_UTR_premature_start_codon_gain_variant	hypothetical protein
TGME49_253050	p.Val6Met	missense_variant	hypothetical protein
TGME49_253050	p.Pro136Thr	missense_variant	hypothetical protein
TGME49_253050	p.Leu224Val	missense_variant	hypothetical protein
TGME49_253080	p.Val186Met	missense_variant	hypothetical protein
TGME49_253080	p.Ser52Thr	missense_variant	hypothetical protein
TGME49_253100	p.Ala287Thr	missense_variant	hypothetical protein
TGME49_253140	p.Ile1336Asn	missense_variant	hypothetical protein
TGME49_253140	p.Ala759Glu	missense_variant	hypothetical protein
TGME49_253140	p.Gly620Arg	missense_variant	hypothetical protein
TGME49_253140	p.Gly337Glu	missense_variant	hypothetical protein
TGME49_253140	p.Pro126Ser	missense_variant	hypothetical protein
TGME49_253140	p.Arg85Gln	missense_variant	hypothetical protein
TGME49_253160	p.His66Tyr	missense_variant	hypothetical protein
TGME49_253400	p.Ala204Ser	missense_variant&splice_region_variant	hypothetical protein
TGME49_253400	p.Glu80Gly	missense_variant	hypothetical protein
TGME49_253400	c.-1025C>G	5_prime_UTR_premature_start_codon_gain_variant	hypothetical protein
TGME49_253490	c.-574G>T	5_prime_UTR_premature_start_codon_gain_variant	hypothetical protein
TGME49_253490	p.Ala403Thr	missense_variant	hypothetical protein
TGME49_253490	p.Lys1317Thr	missense_variant	hypothetical protein
TGME49_253500	p.Glu199Asp	missense_variant	hypothetical protein
TGME49_253530	p.Leu119Val	missense_variant	hypothetical protein
TGME49_253540	p.Pro197Ser	missense_variant	hypothetical protein
TGME49_253560	p.Ala1826Thr	missense_variant	hypothetical protein
TGME49_253560	p.Ala1312Thr	missense_variant	hypothetical protein
TGME49_253560	p.Ser549Phe	missense_variant	hypothetical protein
TGME49_253600	p.Ala230Thr	missense_variant	hypothetical protein
TGME49_253600	p.Ser419Phe	missense_variant	hypothetical protein
TGME49_253615	p.Asp2032Val	missense_variant	hypothetical protein
TGME49_253615	p.Gly1269Ser	missense_variant	hypothetical protein
TGME49_253615	p.Ile1140Val	missense_variant	hypothetical protein
TGME49_253615	p.Glu883Lys	missense_variant	hypothetical protein
TGME49_253615	p.Ser655Thr	missense_variant	hypothetical protein
TGME49_253615	p.Asp146Gly	missense_variant	hypothetical protein
TGME49_253640	c.-360G>C	5_prime_UTR_premature_start_codon_gain_variant	hypothetical protein
TGME49_253690	c.-363G>A	5_prime_UTR_premature_start_codon_gain_variant	hypothetical protein
TGME49_253690	p.Ala108Thr	missense_variant	hypothetical protein
TGME49_253710	p.Pro1021Arg	missense_variant	hypothetical protein
TGME49_253710	c.179-8G>C	splice_region_variant	hypothetical protein
TGME49_253740	c.-184G>A	5_prime_UTR_premature_start_codon_gain_variant	hypothetical protein
TGME49_253810	p.Glu1089Asp	missense_variant	hypothetical protein
TGME49_253810	p.Ser34Thr	missense_variant	hypothetical protein
TGME49_253820	c.372+8G>A	splice_region_variant	hypothetical protein
TGME49_253850	p.Thr102Pro	missense_variant	hypothetical protein
TGME49_253850	p.Gln369Lys	missense_variant	hypothetical protein
TGME49_253870	p.Asp4569Asn	missense_variant	hypothetical protein
TGME49_253870	p.Met4237Ile	missense_variant	hypothetical protein
TGME49_253870	p.Pro4212Leu	missense_variant	hypothetical protein
TGME49_253870	p.Ala4163Thr	missense_variant	hypothetical protein
TGME49_253870	p.Ala3728Val	missense_variant	hypothetical protein
TGME49_253870	p.Arg3568Leu	missense_variant	hypothetical protein
TGME49_253870	p.Ala3502Gly	missense_variant	hypothetical protein
TGME49_253870	p.Glu3324Gly	missense_variant	hypothetical protein
TGME49_253870	p.Thr2924Arg	missense_variant	hypothetical protein
TGME49_253870	p.Ala2725Ser	missense_variant	hypothetical protein
TGME49_253870	p.Leu2454Val	missense_variant	hypothetical protein
TGME49_253870	p.Phe2385Leu	missense_variant	hypothetical protein
TGME49_253870	p.Pro2375Ala	missense_variant	hypothetical protein
TGME49_253870	p.Lys2284Arg	missense_variant	hypothetical protein
TGME49_253870	p.Lys2284Glu	missense_variant	hypothetical protein
TGME49_253870	p.Ser2076Ala	missense_variant	hypothetical protein
TGME49_253870	c.6192+5A>G	splice_region_variant	hypothetical protein
TGME49_253870	p.Gly1914Arg	missense_variant	hypothetical protein
TGME49_253870	p.His1607Asn	missense_variant	hypothetical protein
TGME49_253870	p.Ser1543Phe	missense_variant	hypothetical protein
TGME49_253870	p.Cys1502Trp	missense_variant	hypothetical protein
TGME49_253870	p.Ala1200Val	missense_variant	hypothetical protein
TGME49_253870	p.Ala672Thr	missense_variant	hypothetical protein
TGME49_253870	p.Ser542Arg	missense_variant	hypothetical protein
TGME49_253870	p.Ser511Tyr	missense_variant	hypothetical protein
TGME49_253870	p.Ile439Thr	missense_variant	hypothetical protein
TGME49_253870	p.Gln210His	missense_variant	hypothetical protein
TGME49_253870	p.Ala188Val	missense_variant	hypothetical protein
TGME49_253870	p.Thr159Asn	missense_variant	hypothetical protein
TGME49_253910	p.Asp397Glu	missense_variant	hypothetical protein
TGME49_253910	p.Lys222Arg	missense_variant	hypothetical protein
TGME49_253970	p.Leu35Phe	missense_variant	hypothetical protein
TGME49_253970	p.Tyr93His	missense_variant	hypothetical protein

TGME49_253970	p.Pro229Ser	missense_variant	hypothetical protein
TGME49_253970	p.Gln281Arg	missense_variant	hypothetical protein
TGME49_253970	p.Glu349Lys	missense_variant	hypothetical protein
TGME49_253970	p.Cys456Phe	missense_variant	hypothetical protein
TGME49_253970	p.Ala478Ser	missense_variant	hypothetical protein
TGME49_253970	p.Ser669Cys	missense_variant	hypothetical protein
TGME49_253990	c.-142C>T	5_prime_UTR_premature_start_codon_gain_variant	hypothetical protein
TGME49_253990	c.-16G>C	5_prime_UTR_premature_start_codon_gain_variant	hypothetical protein
TGME49_253990	p.Arg146Pro	missense_variant	hypothetical protein
TGME49_253990	p.Ser320Pro	missense_variant	hypothetical protein
TGME49_253990	p.Pro323Ser	missense_variant	hypothetical protein
TGME49_253990	p.Ala367Thr	missense_variant	hypothetical protein
TGME49_253990	p.Arg509Gly	missense_variant	hypothetical protein
TGME49_253990	p.Ala678Pro	missense_variant	hypothetical protein
TGME49_253990	p.Asp801Glu	missense_variant	hypothetical protein
TGME49_253990	p.Pro807Ala	missense_variant	hypothetical protein
TGME49_253990	p.Pro1012His	missense_variant	hypothetical protein
TGME49_253990	p.Ser1020Ala	missense_variant	hypothetical protein
TGME49_253990	p.Ser1381Gly	missense_variant	hypothetical protein
TGME49_253990	p.Glu1402Val	missense_variant	hypothetical protein
TGME49_253990	p.Leu1479Pro	missense_variant	hypothetical protein
TGME49_253990	p.Leu1549Val	missense_variant	hypothetical protein
TGME49_253990	p.Ala1596Val	missense_variant	hypothetical protein
TGME49_253990	p.Ser1902Pro	missense_variant	hypothetical protein
TGME49_253990	p.Ala1971Val	missense_variant	hypothetical protein
TGME49_254000	p.Ala339Ser	missense_variant	hypothetical protein
TGME49_254000	p.Pro39Ala	missense_variant	hypothetical protein
TGME49_254000	c.-950T>G	5_prime_UTR_premature_start_codon_gain_variant	hypothetical protein
TGME49_254000	c.-2714G>C	5_prime_UTR_premature_start_codon_gain_variant	hypothetical protein
TGME49_254070	c.-2236C>T	5_prime_UTR_premature_start_codon_gain_variant	hypothetical protein
TGME49_254070	c.-455C>T	5_prime_UTR_premature_start_codon_gain_variant	hypothetical protein
TGME49_254070	p.Val90Ile	missense_variant	hypothetical protein
TGME49_254070	p.Ala203Val	missense_variant	hypothetical protein
TGME49_254090	p.Arg562His	missense_variant	hypothetical protein
TGME49_254090	p.Phe125Ser	missense_variant	hypothetical protein
TGME49_254135	p.Pro1268Leu	missense_variant	hypothetical protein
TGME49_254135	p.Ser1105Asn	missense_variant	hypothetical protein
TGME49_254135	p.Asn1071Asp	missense_variant	hypothetical protein
TGME49_254135	p.Pro870Gln	missense_variant	hypothetical protein
TGME49_254135	p.Ala763Gly	missense_variant	hypothetical protein
TGME49_254135	p.Gln690Glu	missense_variant	hypothetical protein
TGME49_254135	p.Ser680Arg	missense_variant	hypothetical protein
TGME49_254135	p.Arg617Pro	missense_variant	hypothetical protein
TGME49_254135	p.Ala512Val	missense_variant	hypothetical protein
TGME49_254135	p.Phe141Cys	missense_variant	hypothetical protein
TGME49_254135	p.Arg82Gln	missense_variant	hypothetical protein
TGME49_254135	p.Phe55Ile	missense_variant	hypothetical protein
TGME49_254135	p.Tyr26Phe	missense_variant	hypothetical protein
TGME49_254150	p.Val654Ile	missense_variant	hypothetical protein
TGME49_254150	p.Ile619Ser	missense_variant	hypothetical protein
TGME49_254150	p.Glu603Lys	missense_variant	hypothetical protein
TGME49_254150	p.Gly507Val	missense_variant	hypothetical protein
TGME49_254150	p.Glu482Gln	missense_variant	hypothetical protein
TGME49_254150	p.Gln460Leu	missense_variant	hypothetical protein
TGME49_254150	c.-61A>C	5_prime_UTR_premature_start_codon_gain_variant	hypothetical protein
TGME49_254150	c.-124G>C	5_prime_UTR_premature_start_codon_gain_variant	hypothetical protein
TGME49_254160	c.-1820C>T	5_prime_UTR_premature_start_codon_gain_variant	hypothetical protein
TGME49_254160	c.-678G>C	5_prime_UTR_premature_start_codon_gain_variant	hypothetical protein
TGME49_254160	p.His252Asp	missense_variant	hypothetical protein
TGME49_254160	p.Arg292Gly	missense_variant	hypothetical protein
TGME49_254160	p.Ser314Ala	missense_variant	hypothetical protein
TGME49_254160	p.Pro337Ala	missense_variant	hypothetical protein
TGME49_254160	p.Arg786His	missense_variant	hypothetical protein
TGME49_254160	p.Asp1079Asn	missense_variant	hypothetical protein
TGME49_254160	p.Tyr1354Cys	missense_variant	hypothetical protein
TGME49_254160	p.Asn1371Asp	missense_variant	hypothetical protein
TGME49_254160	p.Leu1372Val	missense_variant	hypothetical protein
TGME49_254160	p.Lys1414Glu	missense_variant	hypothetical protein
TGME49_254160	p.Gly1571Ala	missense_variant	hypothetical protein
TGME49_254160	p.Glu1642Lys	missense_variant	hypothetical protein
TGME49_254160	p.Asp1746Asn	missense_variant	hypothetical protein
TGME49_254160	p.Arg1900Met	missense_variant	hypothetical protein
TGME49_254160	p.Leu1986Val	missense_variant	hypothetical protein
TGME49_254160	p.Gln1988Glu	missense_variant	hypothetical protein
TGME49_254160	p.Gly1989Trp	missense_variant	hypothetical protein
TGME49_254160	p.Val2031Ala	missense_variant	hypothetical protein
TGME49_254160	p.Met2053Thr	missense_variant	hypothetical protein
TGME49_254160	p.Glu2194Gln	missense_variant	hypothetical protein
TGME49_254160	p.Glu2200Gly	missense_variant	hypothetical protein
TGME49_254160	p.Ser2224Pro	missense_variant	hypothetical protein
TGME49_254160	p.Gly2260Glu	missense_variant	hypothetical protein
TGME49_254160	p.Val2279Ile	missense_variant	hypothetical protein
TGME49_254160	p.Cys2353Trp	missense_variant	hypothetical protein
TGME49_254160	p.Glu2634Gln	missense_variant	hypothetical protein
TGME49_254160	p.Cys2665Ser	missense_variant	hypothetical protein

TGME49_254160	p.Phe2725Leu	missense_variant	hypothetical protein
TGME49_254160	p.Ser2738Phe	missense_variant	hypothetical protein
TGME49_254160	p.Cys2980Gly	missense_variant	hypothetical protein
TGME49_254160	p.Glu3295Asp	missense_variant	hypothetical protein
TGME49_254160	c.10024+8C>T	splice_region_variant	hypothetical protein
TGME49_254220	p.Ala484Gly	missense_variant	hypothetical protein
TGME49_254220	p.Pro262Ser	missense_variant	hypothetical protein
TGME49_254230	p.Ala195Val	missense_variant	hypothetical protein
TGME49_254255	p.Thr138Ala	missense_variant	hypothetical protein
TGME49_254255	p.Phe135Ile	missense_variant	hypothetical protein
TGME49_254255	p.Arg79Gln	missense_variant	hypothetical protein
TGME49_254255	p.Leu71Val	missense_variant	hypothetical protein
TGME49_254255	p.Leu36Pro	missense_variant	hypothetical protein
TGME49_254270	c.-146T>G	5_prime_UTR_premature_start_codon_gain_variant	hypothetical protein
TGME49_254270	p.Pro20Arg	missense_variant	hypothetical protein
TGME49_254270	p.Ala32Thr	missense_variant	hypothetical protein
TGME49_254290	p.Gly77Arg	missense_variant	hypothetical protein
TGME49_254290	p.Arg272Trp	missense_variant	hypothetical protein
TGME49_254290	p.Ser597Pro	missense_variant	hypothetical protein
TGME49_254290	p.Val684Ala	missense_variant	hypothetical protein
TGME49_254290	p.His870Pro	missense_variant	hypothetical protein
TGME49_254290	p.Gln956His	missense_variant	hypothetical protein
TGME49_254290	p.Gly1107Asp	missense_variant	hypothetical protein
TGME49_254290	p.Arg1331Gln	missense_variant	hypothetical protein
TGME49_254290	p.Ala1729Ser	missense_variant	hypothetical protein
TGME49_254290	p.Ala1729Gly	missense_variant	hypothetical protein
TGME49_254290	p.Ala1798Val	missense_variant	hypothetical protein
TGME49_254300	p.Gly1548Asp	missense_variant	hypothetical protein
TGME49_254300	p.Leu1737Phe	missense_variant	hypothetical protein
TGME49_254320	p.Ala64Val	missense_variant	hypothetical protein
TGME49_254485	p.Ter119Trpext*?	stop_lost	hypothetical protein
TGME49_254485	p.Pro12Arg	missense_variant	hypothetical protein
TGME49_254500	c.-774G>T	5_prime_UTR_premature_start_codon_gain_variant	hypothetical protein
TGME49_254500	c.-171T>C	5_prime_UTR_premature_start_codon_gain_variant	hypothetical protein
TGME49_254500	c.-51C>G	5_prime_UTR_premature_start_codon_gain_variant	hypothetical protein
TGME49_254500	p.Ser127Ala	missense_variant	hypothetical protein
TGME49_254500	p.Thr233Ala	missense_variant	hypothetical protein
TGME49_254500	p.Ala488Gly	missense_variant	hypothetical protein
TGME49_254530	c.-969C>G	5_prime_UTR_premature_start_codon_gain_variant	hypothetical protein
TGME49_254530	p.Pro19Leu	missense_variant	hypothetical protein
TGME49_254530	p.Ser154Ala	missense_variant	hypothetical protein
TGME49_254530	p.Pro348Arg	missense_variant	hypothetical protein
TGME49_254530	p.Ser460Ala	missense_variant	hypothetical protein
TGME49_254530	p.Ala514Thr	missense_variant	hypothetical protein
TGME49_254530	p.Arg697Leu	missense_variant	hypothetical protein
TGME49_254530	p.Gly847Ala	missense_variant	hypothetical protein
TGME49_254530	p.Ala1226Thr	missense_variant	hypothetical protein
TGME49_254530	p.Phe1404Leu	missense_variant	hypothetical protein
TGME49_254530	p.Ser1485Asn	missense_variant	hypothetical protein
TGME49_254530	c.4590-4T>G	splice_region_variant	hypothetical protein
TGME49_254530	p.Leu1549Met	missense_variant	hypothetical protein
TGME49_254570	p.Ser1890Pro	missense_variant	hypothetical protein
TGME49_254570	c.3719+6A>G	splice_region_variant	hypothetical protein
TGME49_254570	p.Glu587Lys	missense_variant	hypothetical protein
TGME49_254570	c.-93T>G	5_prime_UTR_premature_start_codon_gain_variant	hypothetical protein
TGME49_254810	p.Val1518Ala	missense_variant	hypothetical protein
TGME49_254810	p.Asp1425Glu	missense_variant	hypothetical protein
TGME49_254810	p.Thr1161Arg	missense_variant	hypothetical protein
TGME49_254810	p.Glu950Lys	missense_variant	hypothetical protein
TGME49_254810	p.Thr603Ala	missense_variant	hypothetical protein
TGME49_254820	p.Asn134Tyr	missense_variant	hypothetical protein
TGME49_254830	p.Gly1152Asp	missense_variant	hypothetical protein
TGME49_254830	p.Asp990Tyr	missense_variant	hypothetical protein
TGME49_254830	p.Gln714Glu	missense_variant	hypothetical protein
TGME49_254830	p.Leu686Arg	missense_variant	hypothetical protein
TGME49_254830	p.Cys439Trp	missense_variant	hypothetical protein
TGME49_254830	p.Ser44Phe	missense_variant	hypothetical protein
TGME49_254835	p.Gly336Asp	missense_variant	hypothetical protein
TGME49_254835	p.Ala528Thr	missense_variant	hypothetical protein
TGME49_254855	p.Gln172His	missense_variant	hypothetical protein
TGME49_254860	p.Pro920Gln	missense_variant	hypothetical protein
TGME49_254860	p.Ala673Gly	missense_variant	hypothetical protein
TGME49_254860	p.Gln635His	missense_variant	hypothetical protein
TGME49_254870	p.Glu80Ala	missense_variant	hypothetical protein
TGME49_254910	p.Met448Val	missense_variant	hypothetical protein
TGME49_254910	p.Leu52Ile	missense_variant	hypothetical protein
TGME49_254915	c.-419C>T	5_prime_UTR_premature_start_codon_gain_variant	hypothetical protein
TGME49_254920	p.Glu280Asp	missense_variant	hypothetical protein
TGME49_254920	p.Phe84Val	missense_variant	hypothetical protein
TGME49_254930	c.-200C>T	5_prime_UTR_premature_start_codon_gain_variant	hypothetical protein
TGME49_254930	p.Arg335Met	missense_variant	hypothetical protein
TGME49_254930	p.Ala618Thr	missense_variant	hypothetical protein
TGME49_254930	p.Ser1092Asn	missense_variant	hypothetical protein
TGME49_254960	p.Leu1332Met	missense_variant	hypothetical protein
TGME49_254960	p.Glu1130Lys	missense_variant	hypothetical protein

TGME49_254960	p.Gly938Arg	missense_variant	hypothetical protein
TGME49_299270	p.Ser194Phe	missense_variant	hypothetical protein
TGME49_299260	p.Ser84Pro	missense_variant	hypothetical protein
TGME49_299260	p.Ser148Asn	missense_variant	hypothetical protein
TGME49_299250	p.Thr859Ser	missense_variant	hypothetical protein
TGME49_299250	p.Ser426Pro	missense_variant	hypothetical protein
TGME49_299250	p.Thr74Ser	missense_variant	hypothetical protein
TGME49_299240	p.Thr4Asn	missense_variant	hypothetical protein
TGME49_299240	p.Pro73Ser	missense_variant	hypothetical protein
TGME49_299240	p.Ser195Ile	missense_variant	hypothetical protein
TGME49_299160	c.-675T>G	5_prime_UTR_premature_start_codon_gain_variant	hypothetical protein
TGME49_299160	c.-57T>C	5_prime_UTR_premature_start_codon_gain_variant	hypothetical protein
TGME49_299160	p.Lys94Thr	missense_variant	hypothetical protein
TGME49_299160	c.*755G>A	splice_region_variant	hypothetical protein
TGME49_299130	p.Asp2835Glu	missense_variant	hypothetical protein
TGME49_299130	p.Trp2416Cys	missense_variant	hypothetical protein
TGME49_299130	p.Gly2359Ser	missense_variant	hypothetical protein
TGME49_299130	p.Glu2321Val	missense_variant	hypothetical protein
TGME49_299130	p.Ser2298Ala	missense_variant	hypothetical protein
TGME49_299130	p.Asp2253Ala	missense_variant	hypothetical protein
TGME49_299130	p.Pro2154Thr	missense_variant	hypothetical protein
TGME49_299130	p.Val2125Ala	missense_variant	hypothetical protein
TGME49_299130	p.Ser2117Phe	missense_variant	hypothetical protein
TGME49_299130	p.Leu1820Phe	missense_variant	hypothetical protein
TGME49_299130	p.Gly1771Asp	missense_variant	hypothetical protein
TGME49_299130	p.Gln1769Pro	missense_variant	hypothetical protein
TGME49_299130	p.Gln1769Glu	missense_variant	hypothetical protein
TGME49_299130	p.Arg1685Cys	missense_variant	hypothetical protein
TGME49_299130	p.Phe1546Ile	missense_variant	hypothetical protein
TGME49_299130	p.Asp1346Gly	missense_variant	hypothetical protein
TGME49_299130	p.Arg1340Gly	missense_variant	hypothetical protein
TGME49_299130	p.Lys1130Asn	missense_variant	hypothetical protein
TGME49_299130	p.Asp1129Asn	missense_variant	hypothetical protein
TGME49_299130	p.Gly936Glu	missense_variant	hypothetical protein
TGME49_299130	p.Pro877Thr	missense_variant	hypothetical protein
TGME49_299130	p.Asp810Glu	missense_variant	hypothetical protein
TGME49_299130	p.Ser802Asn	missense_variant	hypothetical protein
TGME49_299130	p.Ser665Phe	missense_variant	hypothetical protein
TGME49_299130	p.Val529Ala	missense_variant	hypothetical protein
TGME49_299130	p.His494Arg	missense_variant	hypothetical protein
TGME49_299130	p.Gly321Asp	missense_variant	hypothetical protein
TGME49_299130	p.Ala80Val	missense_variant	hypothetical protein
TGME49_299120	p.Gly61Asp	missense_variant	hypothetical protein
TGME49_299100	p.Arg2168Gln	missense_variant	hypothetical protein
TGME49_299100	p.Gly1997Val	missense_variant	hypothetical protein
TGME49_299100	p.Leu1534Arg	missense_variant	hypothetical protein
TGME49_299100	p.Val1458Leu	missense_variant	hypothetical protein
TGME49_299100	p.Val882Ala	missense_variant	hypothetical protein
TGME49_299100	p.Val874Gly	missense_variant	hypothetical protein
TGME49_299100	p.Glu652Asp	missense_variant	hypothetical protein
TGME49_299100	p.Leu186Phe	missense_variant	hypothetical protein
TGME49_299100	p.Leu104Pro	missense_variant	hypothetical protein
TGME49_299100	p.Phe103Leu	missense_variant	hypothetical protein
TGME49_299100	p.Cys83Arg	missense_variant	hypothetical protein
TGME49_299100	p.Ala31Thr	missense_variant	hypothetical protein
TGME49_299100	c.-149C>G	5_prime_UTR_premature_start_codon_gain_variant	hypothetical protein
TGME49_298840	p.Gln62*	stop_gained	hypothetical protein
TGME49_320410	p.Arg43Lys	missense_variant	hypothetical protein
TGME49_320290	p.Pro68Leu	missense_variant	hypothetical protein
TGME49_320290	p.Ala104Val	missense_variant	hypothetical protein
TGME49_320280	p.Tyr209Ser	missense_variant	hypothetical protein
TGME49_320270	p.Ser12Phe	missense_variant	hypothetical protein
TGME49_320270	p.Glu201Val	missense_variant	hypothetical protein
TGME49_320270	p.Pro470His	missense_variant	hypothetical protein
TGME49_320270	p.His645Asp	missense_variant	hypothetical protein
TGME49_320260	c.2150+4C>G	splice_region_variant	hypothetical protein
TGME49_320260	p.Ala406Ser	missense_variant	hypothetical protein
TGME49_320160	c.-1318C>G	5_prime_UTR_premature_start_codon_gain_variant	hypothetical protein
TGME49_320160	c.-1064C>T	5_prime_UTR_premature_start_codon_gain_variant	hypothetical protein
TGME49_320160	p.Glu246Ala	missense_variant	hypothetical protein
TGME49_320160	p.Val897Met	missense_variant	hypothetical protein
TGME49_320160	p.Asp898Gly	missense_variant	hypothetical protein
TGME49_320160	p.Gly904Arg	missense_variant	hypothetical protein
TGME49_320160	p.Phe1198Leu	missense_variant	hypothetical protein
TGME49_320160	p.Thr1243Ser	missense_variant	hypothetical protein
TGME49_320160	p.Thr1425Ser	missense_variant	hypothetical protein
TGME49_320160	p.Ile1652Met	missense_variant	hypothetical protein
TGME49_320090	p.Arg133Leu	missense_variant	hypothetical protein
TGME49_320080	p.Ala1327Pro	missense_variant	hypothetical protein
TGME49_320030	p.His108Gln	missense_variant	hypothetical protein
TGME49_320015	c.-576C>A	5_prime_UTR_premature_start_codon_gain_variant	hypothetical protein
TGME49_320015	p.Glu342Gln	missense_variant	hypothetical protein
TGME49_320010	c.2780+5G>A	splice_region_variant	hypothetical protein
TGME49_320010	p.Ala921Val	missense_variant	hypothetical protein
TGME49_320010	p.Ala188Glu	missense_variant	hypothetical protein

TGME49_320010	c.-695G>A	5_prime_UTR_premature_start_codon_gain_variant	hypothetical protein
TGME49_320005	c.-439C>T	5_prime_UTR_premature_start_codon_gain_variant	hypothetical protein
TGME49_320005	p.Ile204Thr	missense_variant	hypothetical protein
TGME49_319992	p.Ala191Val	missense_variant	hypothetical protein
TGME49_319988	p.Leu82Ser	missense_variant	hypothetical protein
TGME49_319988	p.Asp72Val	missense_variant	hypothetical protein
TGME49_319988	p.Met1?	start_lost	hypothetical protein
TGME49_319980	p.Ile221Leu	missense_variant	hypothetical protein
TGME49_319980	p.Asp81Gly	missense_variant	hypothetical protein
TGME49_319940	c.-620C>T	5_prime_UTR_premature_start_codon_gain_variant	hypothetical protein
TGME49_319940	p.Ala152Val	missense_variant	hypothetical protein
TGME49_319935	p.His41Arg	missense_variant	hypothetical protein
TGME49_319930	p.Met812Ile	missense_variant	hypothetical protein
TGME49_319930	p.Glu675Ala	missense_variant	hypothetical protein
TGME49_319930	p.Thr627Ile	missense_variant	hypothetical protein
TGME49_319900	p.Asp2299His	missense_variant	hypothetical protein
TGME49_319900	p.Thr1908Pro	missense_variant	hypothetical protein
TGME49_319900	p.His1143Arg	missense_variant	hypothetical protein
TGME49_319900	p.Gly1019Val	missense_variant	hypothetical protein
TGME49_319900	p.Glu918Asp	missense_variant	hypothetical protein
TGME49_319900	p.Arg907Cys	missense_variant	hypothetical protein
TGME49_319900	p.Gly545Arg	missense_variant	hypothetical protein
TGME49_319900	p.Arg521His	missense_variant	hypothetical protein
TGME49_319900	p.Ser487Pro	missense_variant	hypothetical protein
TGME49_319900	p.Arg445Gln	missense_variant	hypothetical protein
TGME49_319900	p.Arg398Gln	missense_variant	hypothetical protein
TGME49_319900	p.Pro323Thr	missense_variant	hypothetical protein
TGME49_319900	p.Pro289Ala	missense_variant	hypothetical protein
TGME49_319900	p.Thr286Ala	missense_variant	hypothetical protein
TGME49_319900	p.Gly284Arg	missense_variant	hypothetical protein
TGME49_319900	p.Asp278His	missense_variant	hypothetical protein
TGME49_319900	p.Asp68Ala	missense_variant	hypothetical protein
TGME49_319900	p.His60Pro	missense_variant	hypothetical protein
TGME49_319900	c.-57T>G	5_prime_UTR_premature_start_codon_gain_variant	hypothetical protein
TGME49_319900	c.-977C>T	5_prime_UTR_premature_start_codon_gain_variant	hypothetical protein
TGME49_319900	c.-1233T>G	5_prime_UTR_premature_start_codon_gain_variant	hypothetical protein
TGME49_319900	c.-1500C>T	5_prime_UTR_premature_start_codon_gain_variant	hypothetical protein
TGME49_319890	p.Val134Ile	missense_variant	hypothetical protein
TGME49_319720	p.Ser750Arg	missense_variant	hypothetical protein
TGME49_319720	p.Glu517Gly	missense_variant	hypothetical protein
TGME49_319720	p.Gly447Ser	missense_variant	hypothetical protein
TGME49_319720	p.Ala392Thr	missense_variant	hypothetical protein
TGME49_319690	c.274-4C>A	splice_region_variant	hypothetical protein
TGME49_319690	c.239-3C>T	splice_region_variant	hypothetical protein
TGME49_319680	p.Ala19Val	missense_variant	hypothetical protein
TGME49_319680	c.716-5A>C	splice_region_variant	hypothetical protein
TGME49_319680	c.797-3C>G	splice_region_variant	hypothetical protein
TGME49_319670	c.-52T>G	5_prime_UTR_premature_start_codon_gain_variant	hypothetical protein
TGME49_319670	c.-148C>G	5_prime_UTR_premature_start_codon_gain_variant	hypothetical protein
TGME49_319640	p.Ala132Pro	missense_variant	hypothetical protein
TGME49_319640	p.Lys176Asn	missense_variant	hypothetical protein
TGME49_319630	c.-739C>T	5_prime_UTR_premature_start_codon_gain_variant	hypothetical protein
TGME49_319620	c.-1428T>G	5_prime_UTR_premature_start_codon_gain_variant	hypothetical protein
TGME49_319620	p.Thr463Ala	missense_variant	hypothetical protein
TGME49_319620	p.Met475Leu	missense_variant	hypothetical protein
TGME49_319620	p.Ser624Gly	missense_variant	hypothetical protein
TGME49_319590	c.236+6A>G	splice_region_variant	hypothetical protein
TGME49_319590	p.Ala170Gly	missense_variant	hypothetical protein
TGME49_319590	p.Ser533Ala	missense_variant	hypothetical protein
TGME49_319590	p.Ser943Pro	missense_variant	hypothetical protein
TGME49_319590	p.Glu963Gly	missense_variant	hypothetical protein
TGME49_319590	p.Ser1061Ala	missense_variant	hypothetical protein
TGME49_319590	p.Glu1540Asp	missense_variant	hypothetical protein
TGME49_319590	p.Arg1548Gly	missense_variant	hypothetical protein
TGME49_319590	p.Ser1608Asn	missense_variant	hypothetical protein
TGME49_319590	p.Asp1620Asn	missense_variant	hypothetical protein
TGME49_319590	p.Ile1626Phe	missense_variant	hypothetical protein
TGME49_319590	p.Thr1639Met	missense_variant	hypothetical protein
TGME49_319590	p.His1726Gln	missense_variant	hypothetical protein
TGME49_319590	p.Gln2120His	missense_variant	hypothetical protein
TGME49_319580	p.Ser167Pro	missense_variant	hypothetical protein
TGME49_319580	p.His448Gln	missense_variant	hypothetical protein
TGME49_319580	p.Thr812Pro	missense_variant	hypothetical protein
TGME49_319580	p.Arg921His	missense_variant	hypothetical protein
TGME49_319580	p.Glu1368Asp	missense_variant	hypothetical protein
TGME49_319580	p.Ala1585Val	missense_variant	hypothetical protein
TGME49_319580	p.Ala1887Ser	missense_variant	hypothetical protein
TGME49_319580	p.Gly2013Ala	missense_variant	hypothetical protein
TGME49_319540	p.Asn427Ser	missense_variant	hypothetical protein
TGME49_319540	p.Pro356Ser	missense_variant	hypothetical protein
TGME49_319540	c.-786G>A	5_prime_UTR_premature_start_codon_gain_variant	hypothetical protein
TGME49_319520	p.Ala1340Ser	missense_variant	hypothetical protein
TGME49_319520	p.Gln1095His	missense_variant	hypothetical protein
TGME49_319520	p.Gly306Cys	missense_variant	hypothetical protein
TGME49_319510	p.Ser30Arg	missense_variant	hypothetical protein

TGME49_319510	c.-44C>G	5_prime_UTR_premature_start_codon_gain_variant	hypothetical protein
TGME49_319510	c.-306A>G	5_prime_UTR_premature_start_codon_gain_variant	hypothetical protein
TGME49_319510	c.-342C>T	5_prime_UTR_premature_start_codon_gain_variant	hypothetical protein
TGME49_319390	p.Ser812Asn	missense_variant	hypothetical protein
TGME49_319390	p.Lys703Glu	missense_variant	hypothetical protein
TGME49_319390	p.Ala393Thr	missense_variant	hypothetical protein
TGME49_319380	p.Asp115Asn	missense_variant	hypothetical protein
TGME49_319380	p.Phe328Leu	missense_variant	hypothetical protein
TGME49_319380	p.Thr330Pro	missense_variant	hypothetical protein
TGME49_319380	p.Leu370Ser	missense_variant	hypothetical protein
TGME49_319380	p.Asp489Asn	missense_variant	hypothetical protein
TGME49_319380	p.Phe920Val	missense_variant	hypothetical protein
TGME49_319380	p.Pro975Ser	missense_variant	hypothetical protein
TGME49_319380	p.Ala1157Val	missense_variant	hypothetical protein
TGME49_319380	p.Val1220Leu	missense_variant	hypothetical protein
TGME49_319380	p.Ser1226Asn	missense_variant	hypothetical protein
TGME49_319380	p.Arg1354Leu	missense_variant	hypothetical protein
TGME49_319380	p.Ala1386Gly	missense_variant	hypothetical protein
TGME49_319380	p.Phe1450Leu	missense_variant	hypothetical protein
TGME49_319380	p.Gly1467Arg	missense_variant	hypothetical protein
TGME49_319380	p.Glu1601Lys	missense_variant	hypothetical protein
TGME49_319380	p.Gly1726Ser	missense_variant	hypothetical protein
TGME49_319380	p.Arg1734Gln	missense_variant	hypothetical protein
TGME49_319380	p.Glu1808Lys	missense_variant	hypothetical protein
TGME49_319380	p.Pro1964Ser	missense_variant	hypothetical protein
TGME49_319380	p.Asn1975Ile	missense_variant	hypothetical protein
TGME49_319380	p.Val2213Ile	missense_variant	hypothetical protein
TGME49_319380	p.Ser2504Trp	missense_variant	hypothetical protein
TGME49_319380	p.Glu2600Lys	missense_variant	hypothetical protein
TGME49_319370	p.Ala2247Gly	missense_variant	hypothetical protein
TGME49_319370	p.Thr2164Ala	missense_variant	hypothetical protein
TGME49_319370	p.Asp2015Asn	missense_variant	hypothetical protein
TGME49_319370	p.His1214Arg	missense_variant	hypothetical protein
TGME49_319370	p.Arg1092Cys	missense_variant	hypothetical protein
TGME49_319370	p.Gln1049Glu	missense_variant	hypothetical protein
TGME49_319370	p.Ala1042Gly	missense_variant	hypothetical protein
TGME49_319370	p.Arg1003Thr	missense_variant	hypothetical protein
TGME49_319370	p.Pro994Ser	missense_variant	hypothetical protein
TGME49_319370	p.Glu939Gly	missense_variant	hypothetical protein
TGME49_319370	p.Leu917Ser	missense_variant	hypothetical protein
TGME49_319370	p.Arg886Gly	missense_variant	hypothetical protein
TGME49_319370	p.Thr788Ile	missense_variant	hypothetical protein
TGME49_319370	p.Ser760Leu	missense_variant	hypothetical protein
TGME49_319370	p.Glu475Gly	missense_variant	hypothetical protein
TGME49_319370	p.Arg205Cys	missense_variant	hypothetical protein
TGME49_319370	p.Val92Met	missense_variant	hypothetical protein
TGME49_319370	p.Gln14Glu	missense_variant	hypothetical protein
TGME49_319370	c.-444T>C	5_prime_UTR_premature_start_codon_gain_variant	hypothetical protein
TGME49_319340	c.-1378A>G	5_prime_UTR_premature_start_codon_gain_variant	hypothetical protein
TGME49_319340	p.Cys47Arg	missense_variant	hypothetical protein
TGME49_319320	p.Ala802Val	missense_variant	hypothetical protein
TGME49_319312	p.Ser204Asn	missense_variant	hypothetical protein
TGME49_319312	p.Glu1247Asp	missense_variant	hypothetical protein
TGME49_319312	p.Arg1261Gly	missense_variant	hypothetical protein
TGME49_319312	p.Glu1276Lys	missense_variant	hypothetical protein
TGME49_319312	p.Glu1514Val	missense_variant	hypothetical protein
TGME49_319308	c.19+8A>G	splice_region_variant	hypothetical protein
TGME49_319308	c.317-8T>C	splice_region_variant	hypothetical protein
TGME49_319308	p.Glu494Asp	missense_variant	hypothetical protein
TGME49_319308	c.1542+7A>G	splice_region_variant	hypothetical protein
TGME49_319308	c.1543-7T>G	splice_region_variant	hypothetical protein
TGME49_319308	p.Tyr632His	missense_variant	hypothetical protein
TGME49_319308	p.Lys656Asn	missense_variant	hypothetical protein
TGME49_319308	c.2443-6T>C	splice_region_variant	hypothetical protein
TGME49_319308	p.Gln1195Lys	missense_variant	hypothetical protein
TGME49_319308	p.Ile1208Val	missense_variant	hypothetical protein
TGME49_319308	p.Leu1298Val	missense_variant	hypothetical protein
TGME49_319308	p.Asn1324Thr	missense_variant	hypothetical protein
TGME49_319308	p.Thr1642Asn	missense_variant	hypothetical protein
TGME49_319308	p.Arg1834Thr	missense_variant	hypothetical protein
TGME49_319300	p.Asn948Thr	missense_variant	hypothetical protein
TGME49_319300	p.Leu895Phe	missense_variant	hypothetical protein
TGME49_318640	p.Arg677Lys	missense_variant	hypothetical protein
TGME49_318640	p.Gly441Glu	missense_variant	hypothetical protein
TGME49_318550	c.-293C>A	5_prime_UTR_premature_start_codon_gain_variant	hypothetical protein
TGME49_318525	p.Val676Gly	missense_variant	hypothetical protein
TGME49_318525	p.Lys266Asn	missense_variant	hypothetical protein
TGME49_318525	p.Val155Ala	missense_variant	hypothetical protein
TGME49_318400	p.Val1372Ile	missense_variant	hypothetical protein
TGME49_318400	p.Leu779Val	missense_variant	hypothetical protein
TGME49_318400	c.1768-7T>C	splice_region_variant	hypothetical protein
TGME49_318400	p.Gln197Pro	missense_variant	hypothetical protein
TGME49_318400	c.-42A>G	5_prime_UTR_premature_start_codon_gain_variant	hypothetical protein
TGME49_318400	c.-1319C>T	5_prime_UTR_premature_start_codon_gain_variant	hypothetical protein
TGME49_318400	c.-2004C>T	5_prime_UTR_premature_start_codon_gain_variant	hypothetical protein

TGME49_318400	c.-2885C>A	5_prime_UTR_premature_start_codon_gain_variant	hypothetical protein
TGME49_318400	c.-3163C>T	5_prime_UTR_premature_start_codon_gain_variant	hypothetical protein
TGME49_318400	c.-3793A>T	5_prime_UTR_premature_start_codon_gain_variant	hypothetical protein
TGME49_318400	c.-4114C>T	5_prime_UTR_premature_start_codon_gain_variant	hypothetical protein
TGME49_318390	p.Cys57Arg	missense_variant	hypothetical protein
TGME49_318390	p.Gln105Leu	missense_variant	hypothetical protein
TGME49_318390	p.Gly159Asp	missense_variant	hypothetical protein
TGME49_318390	p.Gln190Arg	missense_variant	hypothetical protein
TGME49_318390	p.Glu204Gly	missense_variant	hypothetical protein
TGME49_318390	p.Glu378Asp	missense_variant	hypothetical protein
TGME49_318390	p.Ala928Thr	missense_variant	hypothetical protein
TGME49_318380	p.Ala64Ser	missense_variant	hypothetical protein
TGME49_318380	p.Met129Val	missense_variant	hypothetical protein
TGME49_318380	p.Asp132Tyr	missense_variant	hypothetical protein
TGME49_318380	p.Ile147Met	missense_variant	hypothetical protein
TGME49_318380	p.Asn149Asp	missense_variant	hypothetical protein
TGME49_318380	p.His308Arg	missense_variant	hypothetical protein
TGME49_318370	p.Ser115Phe	missense_variant	hypothetical protein
TGME49_318370	p.Arg102Ser	missense_variant	hypothetical protein
TGME49_318370	p.Ala82Gly	missense_variant	hypothetical protein
TGME49_318370	p.Val66Met	missense_variant	hypothetical protein
TGME49_318370	p.Ala17Ser	missense_variant	hypothetical protein
TGME49_318340	p.Pro53Ala	missense_variant	hypothetical protein
TGME49_318300	p.Thr22Ser	missense_variant	hypothetical protein
TGME49_318300	c.-546G>T	5_prime_UTR_premature_start_codon_gain_variant	hypothetical protein
TGME49_318290	p.Leu457Val	missense_variant	hypothetical protein
TGME49_318220	p.Lys455Arg	missense_variant	hypothetical protein
TGME49_318220	p.Asn188Ser	missense_variant	hypothetical protein
TGME49_318220	p.Arg177Ser	missense_variant	hypothetical protein
TGME49_318220	p.Ile43Thr	missense_variant	hypothetical protein
TGME49_318210	p.Asp1108Gly	missense_variant	hypothetical protein
TGME49_318210	p.Arg1100Gly	missense_variant	hypothetical protein
TGME49_318210	p.Ile624Val	missense_variant	hypothetical protein
TGME49_318210	p.Gly240Glu	missense_variant	hypothetical protein
TGME49_318210	p.Asn211His	missense_variant	hypothetical protein
TGME49_318210	p.Val190Leu	missense_variant	hypothetical protein
TGME49_318210	p.Gly187Ala	missense_variant	hypothetical protein
TGME49_318210	p.Thr178Ser	missense_variant	hypothetical protein
TGME49_318210	p.Asp162Gly	missense_variant	hypothetical protein
TGME49_318210	c.-502G>C	5_prime_UTR_premature_start_codon_gain_variant	hypothetical protein
TGME49_318210	c.-1195C>T	5_prime_UTR_premature_start_codon_gain_variant	hypothetical protein
TGME49_318210	c.-1731A>C	5_prime_UTR_premature_start_codon_gain_variant	hypothetical protein
TGME49_318170	p.Val198Ala	missense_variant	hypothetical protein
TGME49_318170	c.-1460G>C	5_prime_UTR_premature_start_codon_gain_variant	hypothetical protein
TGME49_318120	p.Thr7Met	missense_variant	hypothetical protein
TGME49_211700	p.Tyr2237Asp	missense_variant	hypothetical protein
TGME49_211700	p.Ser1488Asn	missense_variant	hypothetical protein
TGME49_211700	p.Glu1482Gln	missense_variant	hypothetical protein
TGME49_211700	p.Val1461Met	missense_variant	hypothetical protein
TGME49_211700	p.Ala1002Ser	missense_variant	hypothetical protein
TGME49_211700	p.Thr774Arg	missense_variant	hypothetical protein
TGME49_211700	p.Leu114Val	missense_variant	hypothetical protein
TGME49_211695	p.Pro43Ser	missense_variant	hypothetical protein
TGME49_211695	p.Leu72His	missense_variant	hypothetical protein
TGME49_211695	p.Ala284Thr	missense_variant	hypothetical protein
TGME49_211695	p.Ala287Gly	missense_variant	hypothetical protein
TGME49_211695	p.Ala363Val	missense_variant	hypothetical protein
TGME49_211675	p.Met1?	start_lost	hypothetical protein
TGME49_211660	p.Ser192Pro	missense_variant	hypothetical protein
TGME49_211660	p.Gln111Arg	missense_variant	hypothetical protein
TGME49_211650	p.Arg376His	missense_variant	hypothetical protein
TGME49_211650	p.Thr194Ser	missense_variant	hypothetical protein
TGME49_211650	c.-281C>T	5_prime_UTR_premature_start_codon_gain_variant	hypothetical protein
TGME49_211650	c.-487C>T	5_prime_UTR_premature_start_codon_gain_variant	hypothetical protein
TGME49_211650	c.-529C>A	5_prime_UTR_premature_start_codon_gain_variant	hypothetical protein
TGME49_211640	p.Pro2768Ser	missense_variant	hypothetical protein
TGME49_211640	p.Pro2753Leu	missense_variant	hypothetical protein
TGME49_211640	p.Ala2718Ser	missense_variant	hypothetical protein
TGME49_211640	p.Pro2661Leu	missense_variant	hypothetical protein
TGME49_211640	p.Ala2612Gly	missense_variant	hypothetical protein
TGME49_211640	p.Glu2589Gly	missense_variant	hypothetical protein
TGME49_211640	p.Val2431Ala	missense_variant	hypothetical protein
TGME49_211640	p.Leu2276Val	missense_variant	hypothetical protein
TGME49_211640	p.Val2168Ala	missense_variant	hypothetical protein
TGME49_211640	p.Gly1769Glu	missense_variant	hypothetical protein
TGME49_211640	p.Val1647Leu	missense_variant	hypothetical protein
TGME49_211640	p.Thr1617Ala	missense_variant	hypothetical protein
TGME49_211640	p.Arg1151Leu	missense_variant	hypothetical protein
TGME49_211640	p.Met1134Lys	missense_variant	hypothetical protein
TGME49_211640	p.Ile1107Thr	missense_variant	hypothetical protein
TGME49_211640	p.Arg1075Lys	missense_variant	hypothetical protein
TGME49_211640	p.Asn724Ser	missense_variant	hypothetical protein
TGME49_211640	p.Val378Ala	missense_variant	hypothetical protein
TGME49_211640	p.Pro110Leu	missense_variant	hypothetical protein
TGME49_211640	p.Ser67Tyr	missense_variant	hypothetical protein

TGME49_211610	p.Pro756Ser	missense_variant	hypothetical protein
TGME49_211610	p.Arg738His	missense_variant	hypothetical protein
TGME49_211610	p.Ser723Asn	missense_variant	hypothetical protein
TGME49_211610	p.Leu365Phe	missense_variant	hypothetical protein
TGME49_211610	p.Ser57Gly	missense_variant	hypothetical protein
TGME49_211600	c.-176C>T	5_prime_UTR_premature_start_codon_gain_variant	hypothetical protein
TGME49_211600	p.Ile20Leu	missense_variant	hypothetical protein
TGME49_211600	p.Ser121Cys	missense_variant	hypothetical protein
TGME49_211600	p.Thr242Ala	missense_variant	hypothetical protein
TGME49_211600	p.Pro531Leu	missense_variant	hypothetical protein
TGME49_211600	p.Gly674Val	missense_variant	hypothetical protein
TGME49_211600	p.Lys797Asn	missense_variant	hypothetical protein
TGME49_211600	p.Pro861Leu	missense_variant	hypothetical protein
TGME49_211600	p.Ala1024Thr	missense_variant	hypothetical protein
TGME49_211600	p.Ser1181Ala	missense_variant	hypothetical protein
TGME49_211600	p.Pro1184Ala	missense_variant	hypothetical protein
TGME49_211600	p.Gly1323Ser	missense_variant	hypothetical protein
TGME49_211600	p.Ala1404Val	missense_variant	hypothetical protein
TGME49_211600	p.Ser1410Pro	missense_variant	hypothetical protein
TGME49_211600	p.Ala1411Glu	missense_variant	hypothetical protein
TGME49_211600	p.Cys1424Ser	missense_variant	hypothetical protein
TGME49_211600	p.Gln1970His	missense_variant	hypothetical protein
TGME49_211460	p.Met610Val	missense_variant	hypothetical protein
TGME49_211450	p.Asn1465Lys	missense_variant	hypothetical protein
TGME49_211450	p.Gly1013Arg	missense_variant	hypothetical protein
TGME49_211450	p.Thr1006Arg	missense_variant	hypothetical protein
TGME49_211450	p.Thr1006Ala	missense_variant	hypothetical protein
TGME49_211450	p.Ser222Cys	missense_variant	hypothetical protein
TGME49_211450	p.Asp109Asn	missense_variant	hypothetical protein
TGME49_211450	p.Pro51Ser	missense_variant	hypothetical protein
TGME49_211440	c.-678T>G	5_prime_UTR_premature_start_codon_gain_variant	hypothetical protein
TGME49_211440	c.-126T>G	5_prime_UTR_premature_start_codon_gain_variant	hypothetical protein
TGME49_211440	p.Gly52Ala	missense_variant	hypothetical protein
TGME49_211440	p.Ser72Trp	missense_variant	hypothetical protein
TGME49_211440	p.Gly85Asp	missense_variant	hypothetical protein
TGME49_211440	p.Leu203Phe	missense_variant	hypothetical protein
TGME49_211440	p.Gly281Ser	missense_variant	hypothetical protein
TGME49_211440	p.Arg303Lys	missense_variant	hypothetical protein
TGME49_211440	p.Tyr324*	stop_gained	hypothetical protein
TGME49_211440	p.Ser424Pro	missense_variant	hypothetical protein
TGME49_211440	p.Gly655Asp	missense_variant	hypothetical protein
TGME49_211440	p.Ser1041Gly	missense_variant	hypothetical protein
TGME49_211440	p.His1407Arg	missense_variant	hypothetical protein
TGME49_211440	p.Ser1567Thr	missense_variant	hypothetical protein
TGME49_211440	p.Val1590Ala	missense_variant	hypothetical protein
TGME49_211390	p.Leu251Ile	missense_variant	hypothetical protein
TGME49_211380	p.Val74Phe	missense_variant	hypothetical protein
TGME49_211380	p.Asn208Thr	missense_variant	hypothetical protein
TGME49_211340	p.Ala2122Thr	missense_variant	hypothetical protein
TGME49_211340	p.Val2074Leu	missense_variant	hypothetical protein
TGME49_211340	p.Ala2051Thr	missense_variant	hypothetical protein
TGME49_211340	p.Gly2027Val	missense_variant	hypothetical protein
TGME49_211340	p.Lys1999Glu	missense_variant	hypothetical protein
TGME49_211340	p.Val1895Phe	missense_variant	hypothetical protein
TGME49_211340	p.Pro1788Ser	missense_variant	hypothetical protein
TGME49_211340	p.Met1781Val	missense_variant	hypothetical protein
TGME49_211340	p.Thr1762Ala	missense_variant	hypothetical protein
TGME49_211340	p.Gly1655Ser	missense_variant	hypothetical protein
TGME49_211340	p.Arg1546Lys	missense_variant	hypothetical protein
TGME49_211340	p.Ser1536Asn	missense_variant	hypothetical protein
TGME49_211340	p.Leu1489Ile	missense_variant	hypothetical protein
TGME49_211340	p.Ala1317Glu	missense_variant	hypothetical protein
TGME49_211340	p.Tyr1286His	missense_variant	hypothetical protein
TGME49_211340	p.Met1142Ile	missense_variant	hypothetical protein
TGME49_211340	p.Ala1120Pro	missense_variant	hypothetical protein
TGME49_211340	p.Gly902Asp	missense_variant	hypothetical protein
TGME49_211340	p.Ala496Val	missense_variant	hypothetical protein
TGME49_211340	p.Arg243Pro	missense_variant	hypothetical protein
TGME49_211340	p.Glu188Val	missense_variant	hypothetical protein
TGME49_211340	c.-138C>T	5_prime_UTR_premature_start_codon_gain_variant	hypothetical protein
TGME49_211320	p.Pro317Leu	missense_variant	hypothetical protein
TGME49_211310	p.Cys165Ser	missense_variant	hypothetical protein
TGME49_211310	p.Leu181Met	missense_variant	hypothetical protein
TGME49_211310	p.Asp483Asn	missense_variant	hypothetical protein
TGME49_211310	p.Gly486Glu	missense_variant	hypothetical protein
TGME49_211310	p.Ile640Val	missense_variant	hypothetical protein
TGME49_211310	p.Ser655Leu	missense_variant	hypothetical protein
TGME49_211310	p.Ala1167Ser	missense_variant	hypothetical protein
TGME49_211310	p.Arg1244Lys	missense_variant	hypothetical protein
TGME49_211310	p.Glu1590Ala	missense_variant	hypothetical protein
TGME49_211310	p.Met1690Thr	missense_variant	hypothetical protein
TGME49_211310	p.Glu1795Gly	missense_variant	hypothetical protein
TGME49_211310	p.Lys1994Arg	missense_variant	hypothetical protein
TGME49_211310	p.Phe2210Ser	missense_variant	hypothetical protein
TGME49_211280	p.Thr58Ile	missense_variant	hypothetical protein

TGME49_211280	p.Leu60Ile	missense_variant	hypothetical protein
TGME49_211280	p.Glu834Lys	missense_variant	hypothetical protein
TGME49_211280	p.Glu836Asp	missense_variant	hypothetical protein
TGME49_211250	c.80-7G>T	splice_region_variant	hypothetical protein
TGME49_211220	p.Leu727His	missense_variant	hypothetical protein
TGME49_211220	p.Arg671Gly	missense_variant	hypothetical protein
TGME49_211220	p.Lys228Asn	missense_variant	hypothetical protein
TGME49_211220	p.Pro41Leu	missense_variant	hypothetical protein
TGME49_211220	c.-1045C>T	5_prime_UTR_premature_start_codon_gain_variant	hypothetical protein
TGME49_211150	p.Ala2347Thr	missense_variant	hypothetical protein
TGME49_211150	p.Gly2282Arg	missense_variant	hypothetical protein
TGME49_211150	p.Phe2191Tyr	missense_variant	hypothetical protein
TGME49_211150	p.Arg1950Gly	missense_variant	hypothetical protein
TGME49_211150	p.Glu1816Gly	missense_variant	hypothetical protein
TGME49_211150	p.Val1449Met	missense_variant	hypothetical protein
TGME49_211150	p.Ala1371Gly	missense_variant	hypothetical protein
TGME49_211150	p.Ala423Ser	missense_variant	hypothetical protein
TGME49_211080	p.Ser83Ala	missense_variant	hypothetical protein
TGME49_211070	p.Ser117Leu	missense_variant	hypothetical protein
TGME49_211070	p.Val120Ala	missense_variant	hypothetical protein
TGME49_211015	p.Leu149Phe	missense_variant	hypothetical protein
TGME49_211015	p.Pro98Arg	missense_variant	hypothetical protein
TGME49_211015	p.Lys78Glu	missense_variant	hypothetical protein
TGME49_211015	p.Ser4Pro	missense_variant	hypothetical protein
TGME49_211010	p.Asp200Tyr	missense_variant	hypothetical protein
TGME49_211010	p.Glu484Asp	missense_variant	hypothetical protein
TGME49_211010	p.Glu1023Asp	missense_variant	hypothetical protein
TGME49_211010	p.Leu1090Ile	missense_variant	hypothetical protein
TGME49_211010	p.Glu1114Asp	missense_variant	hypothetical protein
TGME49_211010	p.Glu1132Ala	missense_variant	hypothetical protein
TGME49_211010	p.Glu1209Asp	missense_variant	hypothetical protein
TGME49_211010	p.Asn1312Lys	missense_variant	hypothetical protein
TGME49_211010	p.Leu1386Ile	missense_variant	hypothetical protein
TGME49_211010	p.Phe1700Leu	missense_variant	hypothetical protein
TGME49_211010	c.*620T>C	splice_region_variant	hypothetical protein
TGME49_211000	p.Asp442Gly	missense_variant	hypothetical protein
TGME49_211000	p.Leu127Ile	missense_variant	hypothetical protein
TGME49_210990	p.Ser416Phe	missense_variant	hypothetical protein
TGME49_210975	p.Arg34His	missense_variant	hypothetical protein
TGME49_210975	p.Gly35Glu	missense_variant	hypothetical protein
TGME49_210975	p.His103Tyr	missense_variant	hypothetical protein
TGME49_210975	p.Ala172Val	missense_variant	hypothetical protein
TGME49_210975	p.Arg226His	missense_variant	hypothetical protein
TGME49_210975	p.Phe245Cys	missense_variant	hypothetical protein
TGME49_210970	p.Pro7Arg	missense_variant	hypothetical protein
TGME49_210970	p.Gln62Leu	missense_variant	hypothetical protein
TGME49_210820	p.Ser756Arg	missense_variant	hypothetical protein
TGME49_210820	p.Leu340Arg	missense_variant	hypothetical protein
TGME49_210820	p.Pro43Ser	missense_variant	hypothetical protein
TGME49_210820	c.-216C>T	5_prime_UTR_premature_start_codon_gain_variant	hypothetical protein
TGME49_210820	c.-593C>T	5_prime_UTR_premature_start_codon_gain_variant	hypothetical protein
TGME49_210815	p.Thr92Ala	missense_variant	hypothetical protein
TGME49_210810	c.261+6T>C	splice_region_variant	hypothetical protein
TGME49_210783	p.Arg74Gln	missense_variant	hypothetical protein
TGME49_210770	p.Arg35Met	missense_variant	hypothetical protein
TGME49_210700	p.Gly747Glu	missense_variant	hypothetical protein
TGME49_210700	p.Arg1462Cys	missense_variant	hypothetical protein
TGME49_210700	p.Gly1466Cys	missense_variant	hypothetical protein
TGME49_210700	p.Gln1668Glu	missense_variant	hypothetical protein
TGME49_210700	p.Ser1839Pro	missense_variant	hypothetical protein
TGME49_210700	p.Leu1926Met	missense_variant	hypothetical protein
TGME49_210700	p.Glu1934Asp	missense_variant	hypothetical protein
TGME49_210700	p.Ala1981Pro	missense_variant	hypothetical protein
TGME49_210700	p.Arg2087Gly	missense_variant	hypothetical protein
TGME49_210700	p.Glu2110Gly	missense_variant	hypothetical protein
TGME49_210700	p.Val2692Phe	missense_variant	hypothetical protein
TGME49_210700	p.Arg2883Gly	missense_variant	hypothetical protein
TGME49_210700	p.Arg3006His	missense_variant	hypothetical protein
TGME49_210700	p.Pro3123Ser	missense_variant	hypothetical protein
TGME49_210700	p.Pro3531Arg	missense_variant	hypothetical protein
TGME49_210700	p.Asp3656Asn	missense_variant	hypothetical protein
TGME49_210700	p.Lys3682Thr	missense_variant	hypothetical protein
TGME49_210700	p.Thr3995Arg	missense_variant	hypothetical protein
TGME49_210700	p.Ser4345Pro	missense_variant	hypothetical protein
TGME49_210700	p.Val4366Ala	missense_variant	hypothetical protein
TGME49_210700	p.Val4692Ala	missense_variant	hypothetical protein
TGME49_210700	p.Gly5171Ser	missense_variant	hypothetical protein
TGME49_210700	p.Pro5500Leu	missense_variant	hypothetical protein
TGME49_210700	p.Thr6174Ala	missense_variant	hypothetical protein
TGME49_210700	p.Arg6344Cys	missense_variant	hypothetical protein
TGME49_210700	p.Met6451Thr	missense_variant	hypothetical protein
TGME49_210700	p.Gly6932Arg	missense_variant	hypothetical protein
TGME49_210700	p.Arg7346Gln	missense_variant	hypothetical protein
TGME49_210700	p.Ala7574Pro	missense_variant	hypothetical protein
TGME49_210700	p.Thr7988Ser	missense_variant	hypothetical protein

TGME49_301190	p.Asn69Thr	missense_variant	hypothetical protein
TGME49_301200	p.Glu339Lys	missense_variant	hypothetical protein
TGME49_301200	p.Gly319Ser	missense_variant	hypothetical protein
TGME49_301200	p.Leu316Val	missense_variant	hypothetical protein
TGME49_301200	p.His312Asp	missense_variant	hypothetical protein
TGME49_301200	p.Gly305Arg	missense_variant	hypothetical protein
TGME49_301200	p.Leu240Ile	missense_variant	hypothetical protein
TGME49_301200	p.Thr239Ala	missense_variant	hypothetical protein
TGME49_301200	p.Thr219Ala	missense_variant	hypothetical protein
TGME49_301200	p.Ile203Arg	missense_variant	hypothetical protein
TGME49_301200	p.Pro200Ser	missense_variant	hypothetical protein
TGME49_301200	p.Leu196His	missense_variant	hypothetical protein
TGME49_301200	p.Ser195Cys	missense_variant	hypothetical protein
TGME49_301200	c.542-8T>G	splice_region_variant	hypothetical protein
TGME49_301200	c.541+5G>T	splice_region_variant	hypothetical protein
TGME49_301200	p.Pro151Thr	missense_variant	hypothetical protein
TGME49_301200	p.Pro141Thr	missense_variant	hypothetical protein
TGME49_301200	p.Gly135Glu	missense_variant	hypothetical protein
TGME49_301200	p.Ile125Val	missense_variant	hypothetical protein
TGME49_301200	p.His116Gln	missense_variant	hypothetical protein
TGME49_301200	p.Glu101Lys	missense_variant	hypothetical protein
TGME49_301200	p.Arg82Leu	missense_variant	hypothetical protein
TGME49_301200	p.Arg81His	missense_variant	hypothetical protein
TGME49_301200	p.Cys78Gly	missense_variant	hypothetical protein
TGME49_301200	p.Pro46Ala	missense_variant	hypothetical protein
TGME49_301200	p.Tyr41Cys	missense_variant	hypothetical protein
TGME49_301200	p.Asn23Ser	missense_variant	hypothetical protein
TGME49_301200	p.Lys19Thr	missense_variant	hypothetical protein
TGME49_301240	c.-634C>T	5_prime_UTR_premature_start_codon_gain_variant	hypothetical protein
TGME49_301250	p.Met188Val	missense_variant	hypothetical protein
TGME49_301250	p.Asp426Glu	missense_variant	hypothetical protein
TGME49_301280	p.Phe194Leu	missense_variant	hypothetical protein
TGME49_301280	p.Asn150His	missense_variant	hypothetical protein
TGME49_301280	p.Arg136Gly	missense_variant	hypothetical protein
TGME49_301280	p.Lys96Arg	missense_variant	hypothetical protein
TGME49_301280	p.Arg7Gln	missense_variant	hypothetical protein
TGME49_301290	p.Arg591Lys	missense_variant	hypothetical protein
TGME49_301290	p.Arg524Gly	missense_variant	hypothetical protein
TGME49_301290	p.Glu481Gln	missense_variant	hypothetical protein
TGME49_301290	p.Glu298Gly	missense_variant	hypothetical protein
TGME49_301290	p.Ser278Thr	missense_variant	hypothetical protein
TGME49_301290	p.Met186Ile	missense_variant	hypothetical protein
TGME49_301290	p.Gly141Ala	missense_variant	hypothetical protein
TGME49_301290	p.Ser30Pro	missense_variant	hypothetical protein
TGME49_301300	p.Asp25Asn	missense_variant	hypothetical protein
TGME49_301300	p.Ser87Ala	missense_variant	hypothetical protein
TGME49_301300	p.Thr112Ala	missense_variant	hypothetical protein
TGME49_301300	p.Gly180Glu	missense_variant	hypothetical protein
TGME49_301310	p.Asn168Ser	missense_variant	hypothetical protein
TGME49_301310	p.Ser25Pro	missense_variant	hypothetical protein
TGME49_301310	c.-278G>T	5_prime_UTR_premature_start_codon_gain_variant	hypothetical protein
TGME49_301310	c.-596C>T	5_prime_UTR_premature_start_codon_gain_variant	hypothetical protein
TGME49_301310	c.-669C>T	5_prime_UTR_premature_start_codon_gain_variant	hypothetical protein
TGME49_301330	c.-147G>T	5_prime_UTR_premature_start_codon_gain_variant	hypothetical protein
TGME49_301355	p.His26Arg	missense_variant	hypothetical protein
TGME49_301400	p.Glu2060Ala	missense_variant	hypothetical protein
TGME49_301400	p.Gln1828Glu	missense_variant	hypothetical protein
TGME49_301400	p.Asp1616Val	missense_variant	hypothetical protein
TGME49_301400	p.Glu1302Lys	missense_variant	hypothetical protein
TGME49_301400	p.Arg1294Pro	missense_variant	hypothetical protein
TGME49_301400	p.Gln1256Glu	missense_variant	hypothetical protein
TGME49_301400	p.Gly1248Ala	missense_variant	hypothetical protein
TGME49_301400	p.His642Asn	missense_variant	hypothetical protein
TGME49_301400	p.His519Tyr	missense_variant	hypothetical protein
TGME49_301400	c.-2841A>T	5_prime_UTR_premature_start_codon_gain_variant	hypothetical protein
TGME49_301400	c.-3328C>G	5_prime_UTR_premature_start_codon_gain_variant	hypothetical protein
TGME49_301400	c.-3826A>G	5_prime_UTR_premature_start_codon_gain_variant	hypothetical protein
TGME49_301410	p.Asn727Ser	missense_variant	hypothetical protein
TGME49_301415	p.Asn3Ser	missense_variant	hypothetical protein
TGME49_301420	c.-915A>C	5_prime_UTR_premature_start_codon_gain_variant	hypothetical protein
TGME49_301460	c.1422+4A>G	splice_region_variant	hypothetical protein
TGME49_301460	p.Arg348Lys	missense_variant	hypothetical protein
TGME49_301470	p.Val387Leu	missense_variant	hypothetical protein
TGME49_301690	p.Asp54Glu	missense_variant	hypothetical protein
TGME49_301690	p.Lys175Thr	missense_variant	hypothetical protein
TGME49_301690	p.Asn182Lys	missense_variant	hypothetical protein
TGME49_301690	p.Val213Ala	missense_variant	hypothetical protein
TGME49_301690	p.Ile285Met	missense_variant	hypothetical protein
TGME49_301690	p.Asn356Ser	missense_variant	hypothetical protein
TGME49_301690	p.Glu387Gln	missense_variant	hypothetical protein
TGME49_237835	p.Arg83Thr	missense_variant	hypothetical protein
TGME49_237840	p.Ser156Thr	missense_variant	hypothetical protein
TGME49_237840	p.Arg163Thr	missense_variant	hypothetical protein
TGME49_237840	p.Ser325Thr	missense_variant	hypothetical protein
TGME49_237840	p.Ala438Pro	missense_variant	hypothetical protein

TGME49_237840	p.Met1001Ile	missense_variant	hypothetical protein
TGME49_237840	p.Ala1004Pro	missense_variant	hypothetical protein
TGME49_238020	p.Pro697Ser	missense_variant	hypothetical protein
TGME49_238140	c.1307-4G>T	splice_region_variant	hypothetical protein
TGME49_238140	p.Ser217Phe	missense_variant	hypothetical protein
TGME49_238160	p.Leu142Ile	missense_variant	hypothetical protein
TGME49_238165	c.171+8C>A	splice_region_variant	hypothetical protein
TGME49_238170	c.-343C>A	5_prime_UTR_premature_start_codon_gain_variant	hypothetical protein
TGME49_238170	p.Leu186Arg	missense_variant	hypothetical protein
TGME49_238170	p.Ser432Asn	missense_variant	hypothetical protein
TGME49_238170	c.2565+7C>G	splice_region_variant	hypothetical protein
TGME49_238170	p.Ala953Ser	missense_variant	hypothetical protein
TGME49_238170	p.Thr1408Ser	missense_variant	hypothetical protein
TGME49_238260	p.Ala51Ser	missense_variant	hypothetical protein
TGME49_238270	c.-377C>T	5_prime_UTR_premature_start_codon_gain_variant	hypothetical protein
TGME49_238270	c.-653A>T	5_prime_UTR_premature_start_codon_gain_variant	hypothetical protein
TGME49_238390	c.-1578T>G	5_prime_UTR_premature_start_codon_gain_variant	hypothetical protein
TGME49_238390	p.Thr539Ser	missense_variant	hypothetical protein
TGME49_238390	p.Arg570His	missense_variant	hypothetical protein
TGME49_238390	p.Ala577Gly	missense_variant	hypothetical protein
TGME49_238390	p.Arg675Thr	missense_variant	hypothetical protein
TGME49_238390	p.Arg853His	missense_variant	hypothetical protein
TGME49_238390	p.Gly1116Asp	missense_variant	hypothetical protein
TGME49_238390	p.Arg1395His	missense_variant	hypothetical protein
TGME49_238390	p.Pro1625Ala	missense_variant	hypothetical protein
TGME49_238410	c.-407C>T	5_prime_UTR_premature_start_codon_gain_variant	hypothetical protein
TGME49_238410	p.Leu11Ile	missense_variant	hypothetical protein
TGME49_238410	p.Asp20Gly	missense_variant	hypothetical protein
TGME49_238410	p.Ser24Phe	missense_variant	hypothetical protein
TGME49_238410	p.Arg104Lys	missense_variant	hypothetical protein
TGME49_238410	p.Glu212Lys	missense_variant	hypothetical protein
TGME49_238410	p.Gln282Glu	missense_variant	hypothetical protein
TGME49_238410	p.Asp465Val	missense_variant	hypothetical protein
TGME49_238410	p.Glu723Gln	missense_variant	hypothetical protein
TGME49_238410	p.Glu869Gly	missense_variant	hypothetical protein
TGME49_238410	p.Pro912Arg	missense_variant	hypothetical protein
TGME49_238410	p.Pro1119Arg	missense_variant	hypothetical protein
TGME49_238410	p.Glu1239Gly	missense_variant	hypothetical protein
TGME49_238410	p.Arg1331Lys	missense_variant	hypothetical protein
TGME49_238420	c.-1254A>G	5_prime_UTR_premature_start_codon_gain_variant	hypothetical protein
TGME49_238420	p.Val288Ile	missense_variant	hypothetical protein
TGME49_238420	p.Pro706Thr	missense_variant	hypothetical protein
TGME49_238420	p.Pro706Leu	missense_variant	hypothetical protein
TGME49_238420	p.Glu731Gly	missense_variant	hypothetical protein
TGME49_238420	p.Val735Asp	missense_variant	hypothetical protein
TGME49_238420	p.Lys768Asn	missense_variant	hypothetical protein
TGME49_238420	p.Arg824His	missense_variant	hypothetical protein
TGME49_238420	p.Gly1034Ala	missense_variant	hypothetical protein
TGME49_238510	p.Gln698Arg	missense_variant	hypothetical protein
TGME49_238870	c.-1351C>T	5_prime_UTR_premature_start_codon_gain_variant	hypothetical protein
TGME49_238870	p.Ala363Thr	missense_variant	hypothetical protein
TGME49_238880	p.Glu112Lys	missense_variant	hypothetical protein
TGME49_238880	p.Ser1500Arg	missense_variant	hypothetical protein
TGME49_238880	p.Arg1519Met	missense_variant	hypothetical protein
TGME49_238880	p.Ala1713Thr	missense_variant	hypothetical protein
TGME49_238880	p.Asp2215Asn	missense_variant	hypothetical protein
TGME49_238880	p.Ile2596Val	missense_variant	hypothetical protein
TGME49_238880	p.Pro2616Ser	missense_variant	hypothetical protein
TGME49_238880	p.Glu2626Asp	missense_variant	hypothetical protein
TGME49_238880	p.Thr2785Met	missense_variant	hypothetical protein
TGME49_238880	p.Gln2862His	missense_variant	hypothetical protein
TGME49_238880	p.Gly2951Asp	missense_variant	hypothetical protein
TGME49_238880	p.Arg3021Leu	missense_variant	hypothetical protein
TGME49_238880	p.Pro3078Arg	missense_variant	hypothetical protein
TGME49_238895	p.Val1308Gly	missense_variant	hypothetical protein
TGME49_238895	p.Gln29His	missense_variant	hypothetical protein
TGME49_238930	p.Glu1434Gln	missense_variant	hypothetical protein
TGME49_238930	p.Arg844Lys	missense_variant	hypothetical protein
TGME49_238930	p.Thr831Ala	missense_variant	hypothetical protein
TGME49_238930	p.Lys615Asn	missense_variant	hypothetical protein
TGME49_238930	p.Lys486Ile	missense_variant	hypothetical protein
TGME49_238930	p.Gln483His	missense_variant	hypothetical protein
TGME49_238930	p.Pro295Ser	missense_variant	hypothetical protein
TGME49_238930	p.Gly283Arg	missense_variant	hypothetical protein
TGME49_238930	p.Asp282Gly	missense_variant	hypothetical protein
TGME49_238930	p.Leu213Phe	missense_variant	hypothetical protein
TGME49_238970	p.Gln152His	missense_variant	hypothetical protein
TGME49_238970	p.Pro791Leu	missense_variant	hypothetical protein
TGME49_238970	p.Ser852Arg	missense_variant	hypothetical protein
TGME49_238970	p.Ser852Thr	missense_variant	hypothetical protein
TGME49_238970	p.Leu1165Val	missense_variant	hypothetical protein
TGME49_238970	p.Val1169Ala	missense_variant	hypothetical protein
TGME49_238970	p.Glu1213Gln	missense_variant	hypothetical protein
TGME49_238970	p.Ile1287Asn	missense_variant	hypothetical protein
TGME49_238970	p.Gln1303Arg	missense_variant	hypothetical protein

TGME49_238970	p.Asp1401Glu	missense_variant	hypothetical protein
TGME49_238970	p.Lys2037Glu	missense_variant	hypothetical protein
TGME49_238970	p.His2157Tyr	missense_variant	hypothetical protein
TGME49_238970	p.Pro2235Ser	missense_variant	hypothetical protein
TGME49_238970	p.Lys2355Arg	missense_variant	hypothetical protein
TGME49_238970	p.Lys2506Arg	missense_variant	hypothetical protein
TGME49_238980	p.Cys69Tyr	missense_variant	hypothetical protein
TGME49_238980	p.Gln413Glu	missense_variant	hypothetical protein
TGME49_238980	p.Lys844Thr	missense_variant	hypothetical protein
TGME49_238980	p.Glu885Lys	missense_variant	hypothetical protein
TGME49_239010	p.Leu106Gln	missense_variant	hypothetical protein
TGME49_239050	c.-616C>G	5_prime_UTR_premature_start_codon_gain_variant	hypothetical protein
TGME49_239050	p.Gly110Glu	missense_variant	hypothetical protein
TGME49_239050	p.Glu114Gly	missense_variant	hypothetical protein
TGME49_239050	c.915-6G>T	splice_region_variant	hypothetical protein
TGME49_239050	p.Lys587Glu	missense_variant	hypothetical protein
TGME49_239050	p.Gly605Ser	missense_variant	hypothetical protein
TGME49_239070	p.Leu496Val	missense_variant	hypothetical protein
TGME49_239070	p.Leu174Val	missense_variant	hypothetical protein
TGME49_239120	c.-340G>A	5_prime_UTR_premature_start_codon_gain_variant	hypothetical protein
TGME49_239120	p.Pro14Ser	missense_variant	hypothetical protein
TGME49_239270	p.Ala314Ser	missense_variant	hypothetical protein
TGME49_239270	p.Lys276Arg	missense_variant	hypothetical protein
TGME49_239270	p.Asn164Thr	missense_variant	hypothetical protein
TGME49_239270	p.Arg96Lys	missense_variant	hypothetical protein
TGME49_239290	p.Arg1726Thr	missense_variant	hypothetical protein
TGME49_239290	p.Thr1704Pro	missense_variant	hypothetical protein
TGME49_239290	p.Lys1611Arg	missense_variant	hypothetical protein
TGME49_239290	p.Lys1610Glu	missense_variant	hypothetical protein
TGME49_239290	p.Leu1600Pro	missense_variant	hypothetical protein
TGME49_239290	p.His1391Gln	missense_variant	hypothetical protein
TGME49_239290	p.Ala1379Asp	missense_variant	hypothetical protein
TGME49_239290	p.Ala1076Glu	missense_variant	hypothetical protein
TGME49_239290	p.Asn967Ile	missense_variant	hypothetical protein
TGME49_239290	p.Gly393Cys	missense_variant	hypothetical protein
TGME49_239290	p.Gln137Glu	missense_variant	hypothetical protein
TGME49_239300	c.-371C>A	5_prime_UTR_premature_start_codon_gain_variant	hypothetical protein
TGME49_239300	c.1332+6C>T	splice_region_variant	hypothetical protein
TGME49_239340	c.-440T>G	5_prime_UTR_premature_start_codon_gain_variant	hypothetical protein
TGME49_239340	p.Ala274Val	missense_variant	hypothetical protein
TGME49_239340	p.Ser752Arg	missense_variant	hypothetical protein
TGME49_239340	p.Pro785Ser	missense_variant	hypothetical protein
TGME49_239340	p.Ser816Asn	missense_variant	hypothetical protein
TGME49_239340	p.Arg867His	missense_variant	hypothetical protein
TGME49_239340	p.Pro898Leu	missense_variant	hypothetical protein
TGME49_239340	p.Glu902Asp	missense_variant	hypothetical protein
TGME49_239340	p.Ala1027Gly	missense_variant	hypothetical protein
TGME49_239340	p.Ala1226Thr	missense_variant	hypothetical protein
TGME49_239340	p.Pro1500Ala	missense_variant	hypothetical protein
TGME49_239340	p.Met1671Thr	missense_variant	hypothetical protein
TGME49_239365	p.Glu236Gly	missense_variant	hypothetical protein
TGME49_239365	p.Arg52His	missense_variant	hypothetical protein
TGME49_239885	p.Arg5982Thr	missense_variant	hypothetical protein
TGME49_239885	p.Ser5961Leu	missense_variant	hypothetical protein
TGME49_239885	p.Lys5932Thr	missense_variant	hypothetical protein
TGME49_239885	p.Pro5929Arg	missense_variant	hypothetical protein
TGME49_239885	p.Ala5923Thr	missense_variant	hypothetical protein
TGME49_239885	p.His5882Arg	missense_variant	hypothetical protein
TGME49_239885	p.Ala5732Thr	missense_variant	hypothetical protein
TGME49_239885	p.Gln5696Glu	missense_variant	hypothetical protein
TGME49_239885	p.Val5680Ala	missense_variant	hypothetical protein
TGME49_239885	p.Val5680Met	missense_variant	hypothetical protein
TGME49_239885	p.Gly5450Arg	missense_variant	hypothetical protein
TGME49_239885	p.Glu5271Asp	missense_variant	hypothetical protein
TGME49_239885	p.Glu5257Ala	missense_variant	hypothetical protein
TGME49_239885	p.Ser5256Asn	missense_variant	hypothetical protein
TGME49_239885	p.Glu5253Gly	missense_variant	hypothetical protein
TGME49_239885	p.Glu5106Val	missense_variant	hypothetical protein
TGME49_239885	p.Arg5053Gln	missense_variant	hypothetical protein
TGME49_239885	p.Arg4936Pro	missense_variant	hypothetical protein
TGME49_239885	p.Arg4722Gln	missense_variant	hypothetical protein
TGME49_239885	p.Gln4662Arg	missense_variant	hypothetical protein
TGME49_239885	p.Gly4508Ser	missense_variant	hypothetical protein
TGME49_239885	p.Ser4488Gly	missense_variant	hypothetical protein
TGME49_239885	p.Phe4252Tyr	missense_variant	hypothetical protein
TGME49_239885	p.Glu4078Lys	missense_variant	hypothetical protein
TGME49_239885	p.Cys4020Trp	missense_variant	hypothetical protein
TGME49_239885	p.Tyr3850Cys	missense_variant	hypothetical protein
TGME49_239885	p.Val3814Ile	missense_variant	hypothetical protein
TGME49_239885	p.Arg3813Gly	missense_variant	hypothetical protein
TGME49_239885	p.Thr3805Pro	missense_variant	hypothetical protein
TGME49_239885	p.Arg3675Thr	missense_variant	hypothetical protein
TGME49_239885	p.Gly3651Arg	missense_variant	hypothetical protein
TGME49_239885	p.Arg3612Pro	missense_variant	hypothetical protein
TGME49_239885	p.Cys3482Arg	missense_variant	hypothetical protein

TGME49_239885	p.Glu3370*	stop_gained	hypothetical protein
TGME49_239885	p.Arg3096Trp	missense_variant	hypothetical protein
TGME49_239885	p.Glu3035Lys	missense_variant	hypothetical protein
TGME49_239885	p.Gln2948Lys	missense_variant	hypothetical protein
TGME49_239885	p.Thr2652Ser	missense_variant	hypothetical protein
TGME49_239885	p.Thr2608Ser	missense_variant	hypothetical protein
TGME49_239885	p.Pro2516Ser	missense_variant	hypothetical protein
TGME49_239885	p.Cys2227Trp	missense_variant	hypothetical protein
TGME49_239885	p.Pro1916Ala	missense_variant	hypothetical protein
TGME49_239885	p.Glu1830Asp	missense_variant	hypothetical protein
TGME49_239885	p.Asp1820Asn	missense_variant	hypothetical protein
TGME49_239885	p.Glu1681Gln	missense_variant	hypothetical protein
TGME49_239885	p.Gly1620Arg	missense_variant	hypothetical protein
TGME49_239885	p.Glu1617Gly	missense_variant	hypothetical protein
TGME49_239885	p.Ala1569Ser	missense_variant	hypothetical protein
TGME49_239885	p.Thr1336Arg	missense_variant	hypothetical protein
TGME49_239885	p.His1144Pro	missense_variant	hypothetical protein
TGME49_239885	p.Ser1070Ala	missense_variant	hypothetical protein
TGME49_239885	p.Val1030Ala	missense_variant	hypothetical protein
TGME49_239885	p.Arg976Gly	missense_variant	hypothetical protein
TGME49_239885	p.Gly934Arg	missense_variant	hypothetical protein
TGME49_239885	p.Arg484Lys	missense_variant	hypothetical protein
TGME49_239885	p.Val460Leu	missense_variant	hypothetical protein
TGME49_239885	p.Cys357Phe	missense_variant	hypothetical protein
TGME49_239885	p.Thr327Ile	missense_variant	hypothetical protein
TGME49_239885	p.Pro324Arg	missense_variant	hypothetical protein
TGME49_239885	p.Glu206Asp	missense_variant	hypothetical protein
TGME49_239885	p.Lys82Asn	missense_variant	hypothetical protein
TGME49_239885	p.Ala43Ser	missense_variant	hypothetical protein
TGME49_239400	c.-215C>T	5_prime_UTR_premature_start_codon_gain_variant	hypothetical protein
TGME49_239400	c.-38C>T	5_prime_UTR_premature_start_codon_gain_variant	hypothetical protein
TGME49_239400	p.His78Gln	missense_variant	hypothetical protein
TGME49_239400	p.Leu301Met	missense_variant	hypothetical protein
TGME49_239400	p.Glu699Gly	missense_variant	hypothetical protein
TGME49_239400	p.His707Arg	missense_variant	hypothetical protein
TGME49_239400	p.Gly880Glu	missense_variant	hypothetical protein
TGME49_239400	p.Pro1106Arg	missense_variant	hypothetical protein
TGME49_239400	p.Arg1118Ser	missense_variant	hypothetical protein
TGME49_239410	p.Met1377Val	missense_variant	hypothetical protein
TGME49_239410	p.Gly1264Ser	missense_variant	hypothetical protein
TGME49_239410	p.Ser1037Thr	missense_variant	hypothetical protein
TGME49_239410	p.Ala962Thr	missense_variant	hypothetical protein
TGME49_239410	p.Ala942Pro	missense_variant	hypothetical protein
TGME49_239410	p.His923Gln	missense_variant	hypothetical protein
TGME49_239410	p.Ala620Thr	missense_variant	hypothetical protein
TGME49_239410	p.Thr428Ala	missense_variant	hypothetical protein
TGME49_239410	p.Ala254Thr	missense_variant	hypothetical protein
TGME49_239475	p.Ala1861Ser	missense_variant	hypothetical protein
TGME49_239475	p.Arg1741Gln	missense_variant	hypothetical protein
TGME49_239475	p.Ser1679Cys	missense_variant	hypothetical protein
TGME49_239475	p.Pro1492Ala	missense_variant	hypothetical protein
TGME49_239475	p.Asp1074Glu	missense_variant	hypothetical protein
TGME49_239475	p.Ala823Val	missense_variant	hypothetical protein
TGME49_239475	p.His795Asn	missense_variant	hypothetical protein
TGME49_239475	p.Met682Ile	missense_variant	hypothetical protein
TGME49_239475	p.Gly639Ser	missense_variant	hypothetical protein
TGME49_239475	p.Pro559Thr	missense_variant	hypothetical protein
TGME49_239475	c.-794T>G	5_prime_UTR_premature_start_codon_gain_variant	hypothetical protein
TGME49_239510	p.Gln341Glu	missense_variant	hypothetical protein
TGME49_239520	p.Ala274Thr	missense_variant	hypothetical protein
TGME49_239570	p.Leu330Trp	missense_variant	hypothetical protein
TGME49_239580	p.Val403Ala	missense_variant	hypothetical protein
TGME49_239580	p.Asp554His	missense_variant	hypothetical protein
TGME49_239580	p.Pro1027His	missense_variant	hypothetical protein
TGME49_239580	p.Leu1405Phe	missense_variant	hypothetical protein
TGME49_239580	p.Ser1516Cys	missense_variant	hypothetical protein
TGME49_239580	p.Lys2194Thr	missense_variant	hypothetical protein
TGME49_239610	c.-1573T>G	5_prime_UTR_premature_start_codon_gain_variant	hypothetical protein
TGME49_239610	p.Glu282Lys	missense_variant	hypothetical protein
TGME49_239640	p.Lys5Thr	missense_variant	hypothetical protein
TGME49_239640	p.Ile10Val	missense_variant	hypothetical protein
TGME49_239650	p.Arg352Pro	missense_variant	hypothetical protein
TGME49_239650	p.Gln329Glu	missense_variant	hypothetical protein
TGME49_239650	p.Cys303Gly	missense_variant	hypothetical protein
TGME49_239650	p.Val248Ala	missense_variant	hypothetical protein
TGME49_239660	p.Gly131Ser	missense_variant	hypothetical protein
TGME49_239660	p.Thr130Ser	missense_variant	hypothetical protein
TGME49_239670	p.Ala129Thr	missense_variant	hypothetical protein
TGME49_239670	p.Cys252Ser	missense_variant	hypothetical protein
TGME49_239670	p.Gln297Leu	missense_variant	hypothetical protein
TGME49_239670	p.Gln599Glu	missense_variant	hypothetical protein
TGME49_239670	p.Pro665Ser	missense_variant	hypothetical protein
TGME49_239670	p.Phe734Cys	missense_variant	hypothetical protein
TGME49_239670	p.Val740Ala	missense_variant	hypothetical protein
TGME49_239670	p.Asn783Ser	missense_variant	hypothetical protein

TGME49_239670	p.Pro837Ser	missense_variant	hypothetical protein
TGME49_239748	c.-1928T>C	5_prime_UTR_premature_start_codon_gain_variant	hypothetical protein
TGME49_239880	p.Ser1030Tyr	missense_variant	hypothetical protein
TGME49_239880	p.Asp116Asn	missense_variant	hypothetical protein
TGME49_240050	p.Thr319Ser	missense_variant	hypothetical protein
TGME49_240050	p.Glu581Asp	missense_variant	hypothetical protein
TGME49_240060	c.-105C>T	5_prime_UTR_premature_start_codon_gain_variant	hypothetical protein
TGME49_240060	p.Lys682Arg	missense_variant	hypothetical protein
TGME49_240060	p.Pro772Ser	missense_variant	hypothetical protein
TGME49_240080	p.Ser1072Phe	missense_variant	hypothetical protein
TGME49_240080	p.Arg784Gln	missense_variant	hypothetical protein
TGME49_240080	p.Phe310Cys	missense_variant	hypothetical protein
TGME49_240080	p.Gly162Glu	missense_variant	hypothetical protein
TGME49_240080	p.Ser157Arg	missense_variant	hypothetical protein
TGME49_240200	c.-678C>T	5_prime_UTR_premature_start_codon_gain_variant	hypothetical protein
TGME49_240200	p.Ile27Ser	missense_variant	hypothetical protein
TGME49_240200	p.Asp71Ala	missense_variant&splice_region_variant	hypothetical protein
TGME49_240200	p.Ser329Leu	missense_variant	hypothetical protein
TGME49_240220	c.-393C>T	5_prime_UTR_premature_start_codon_gain_variant	hypothetical protein
TGME49_240220	c.-294A>C	5_prime_UTR_premature_start_codon_gain_variant	hypothetical protein
TGME49_240220	p.Asn960Ser	missense_variant	hypothetical protein
TGME49_240220	p.Asp1124Gly	missense_variant	hypothetical protein
TGME49_240220	p.Ala1169Gly	missense_variant	hypothetical protein
TGME49_240220	p.Asn1205Ser	missense_variant	hypothetical protein
TGME49_240220	p.Ala1229Gly	missense_variant	hypothetical protein
TGME49_240220	p.Arg1272Leu	missense_variant	hypothetical protein
TGME49_240220	p.Thr1404Ala	missense_variant	hypothetical protein
TGME49_240230	c.607-6T>C	splice_region_variant	hypothetical protein
TGME49_240230	c.664-6A>C	splice_region_variant	hypothetical protein
TGME49_240230	p.Gln237His	missense_variant	hypothetical protein
TGME49_240243	c.-149C>G	5_prime_UTR_premature_start_codon_gain_variant	hypothetical protein
TGME49_240255	p.Gly100Ala	missense_variant	hypothetical protein
TGME49_240260	p.Arg109Pro	missense_variant	hypothetical protein
TGME49_279550	p.Ala54Ser	missense_variant	hypothetical protein
TGME49_280725	p.Lys183Asn	missense_variant	hypothetical protein
TGME49_280725	p.Gly436Ser	missense_variant	hypothetical protein
TGME49_280720	p.Thr27Ser	missense_variant	hypothetical protein
TGME49_280720	p.Asp207Gly	missense_variant	hypothetical protein
TGME49_280720	p.Ala446Thr	missense_variant	hypothetical protein
TGME49_280680	c.-382A>G	5_prime_UTR_premature_start_codon_gain_variant	hypothetical protein
TGME49_280680	c.-118C>G	5_prime_UTR_premature_start_codon_gain_variant	hypothetical protein
TGME49_280680	p.Glu200Asp	missense_variant	hypothetical protein
TGME49_280605	p.Asn3Ile	missense_variant	hypothetical protein
TGME49_280605	p.Arg475Trp	missense_variant	hypothetical protein
TGME49_280605	p.Lys525Glu	missense_variant	hypothetical protein
TGME49_280605	p.Leu1023Phe	missense_variant	hypothetical protein
TGME49_280590	p.Arg107Ile	missense_variant	hypothetical protein
TGME49_280590	p.Asn396His	missense_variant	hypothetical protein
TGME49_280590	p.Leu468Val	missense_variant	hypothetical protein
TGME49_280590	p.Pro990His	missense_variant	hypothetical protein
TGME49_280590	p.Ala1059Ser	missense_variant	hypothetical protein
TGME49_280590	p.Asp1393Asn	missense_variant	hypothetical protein
TGME49_280590	p.Pro1439Ser	missense_variant	hypothetical protein
TGME49_280590	p.Gly1665Asp	missense_variant	hypothetical protein
TGME49_263990	c.-366T>C	5_prime_UTR_premature_start_codon_gain_variant	hypothetical protein
TGME49_257360	p.Val771Ile	missense_variant	hypothetical protein
TGME49_255930	p.Ala130Glu	missense_variant	hypothetical protein
TGME49_266980	p.Ser183Arg	missense_variant	hypothetical protein
TGME49_288940	p.Ser410Pro	missense_variant	hypothetical protein
TGME49_210210	p.Leu319Ile	missense_variant	hypothetical protein
TGME49_306230	p.Ala364Thr	missense_variant	hypothetical protein
TGME49_228670	p.Thr1314Ala	missense_variant	hypothetical protein
TGME49_224920	p.Thr1635Ala	missense_variant	hypothetical protein
TGME49_224920	p.Glu1303Asp	missense_variant	hypothetical protein
TGME49_224920	p.Ile831Leu	missense_variant	hypothetical protein
TGME49_224920	p.Leu225Val	missense_variant	hypothetical protein
TGME49_224920	p.Asn120Thr	missense_variant	hypothetical protein
TGME49_224920	p.Asn120Asp	missense_variant	hypothetical protein
TGME49_224920	p.Gln95Glu	missense_variant	hypothetical protein
TGME49_224920	c.-278A>G	5_prime_UTR_premature_start_codon_gain_variant	hypothetical protein
TGME49_224890	p.Val704Ile	missense_variant	hypothetical protein
TGME49_224890	p.Ile139Thr	missense_variant	hypothetical protein
TGME49_224890	c.-17C>T	5_prime_UTR_premature_start_codon_gain_variant	hypothetical protein
TGME49_224870	p.Glu442Gln	missense_variant	hypothetical protein
TGME49_224870	p.Pro657Arg	missense_variant	hypothetical protein
TGME49_224870	p.Gly1331Arg	missense_variant	hypothetical protein
TGME49_224870	p.Ala1416Glu	missense_variant	hypothetical protein
TGME49_224870	p.Arg1426Trp	missense_variant	hypothetical protein
TGME49_224870	p.Gly1776Ser	missense_variant	hypothetical protein
TGME49_224870	p.Gly1785Arg	missense_variant	hypothetical protein
TGME49_224870	p.Gly1929Asp	missense_variant	hypothetical protein
TGME49_224870	p.Asp2058Gly	missense_variant	hypothetical protein
TGME49_224870	p.Ile2230Val	missense_variant	hypothetical protein
TGME49_224870	p.Val2285Leu	missense_variant	hypothetical protein
TGME49_224870	p.Glu2359Asp	missense_variant	hypothetical protein

TGME49_224870	p.Ser2401Thr	missense_variant	hypothetical protein
TGME49_224870	p.Met2407Ile	missense_variant	hypothetical protein
TGME49_224870	p.Ser3037Ala	missense_variant	hypothetical protein
TGME49_224870	p.Glu3260Gln	missense_variant	hypothetical protein
TGME49_224870	p.Arg3263Lys	missense_variant	hypothetical protein
TGME49_224870	p.Glu3369Gly	missense_variant	hypothetical protein
TGME49_224870	p.Arg3385Gln	missense_variant	hypothetical protein
TGME49_224870	p.Asp3410Glu	missense_variant	hypothetical protein
TGME49_224870	c.10737+3A>G	splice_region_variant	hypothetical protein
TGME49_224870	p.Pro4622Leu	missense_variant	hypothetical protein
TGME49_224870	p.Ser4879Cys	missense_variant	hypothetical protein
TGME49_224870	p.Ala4965Val	missense_variant	hypothetical protein
TGME49_224870	c.16707-5T>C	splice_region_variant	hypothetical protein
TGME49_224870	p.Ala5809Val	missense_variant	hypothetical protein
TGME49_224870	c.18213-5T>C	splice_region_variant	hypothetical protein
TGME49_224870	p.Pro6769Leu	missense_variant	hypothetical protein
TGME49_224870	p.Ala6945Thr	missense_variant	hypothetical protein
TGME49_224870	p.Glu7348Asp	missense_variant	hypothetical protein
TGME49_224870	p.Gly7409Asp	missense_variant	hypothetical protein
TGME49_224870	p.Ala7516Val	missense_variant	hypothetical protein
TGME49_224870	p.Ala7519Thr	missense_variant	hypothetical protein
TGME49_224870	p.Glu7703Ala	missense_variant	hypothetical protein
TGME49_224870	p.Lys8129Asn	missense_variant	hypothetical protein
TGME49_224870	p.Val8174Phe	missense_variant	hypothetical protein
TGME49_224870	p.Pro8261Leu	missense_variant	hypothetical protein
TGME49_224870	p.Val8566Leu	missense_variant	hypothetical protein
TGME49_224845	c.-976A>C	5_prime_UTR_premature_start_codon_gain_variant	hypothetical protein
TGME49_224845	c.-982G>A	5_prime_UTR_premature_start_codon_gain_variant	hypothetical protein
TGME49_224845	c.-1255T>C	5_prime_UTR_premature_start_codon_gain_variant	hypothetical protein
TGME49_224830	p.Gly453Ala	missense_variant	hypothetical protein
TGME49_224830	p.Tyr122Phe	missense_variant	hypothetical protein
TGME49_224820	p.Ile31Val	missense_variant	hypothetical protein
TGME49_224820	p.Arg337His	missense_variant	hypothetical protein
TGME49_224810	p.Ala1649Thr	missense_variant	hypothetical protein
TGME49_224810	p.Asn1528Thr	missense_variant	hypothetical protein
TGME49_224810	p.Ser1462Gly	missense_variant	hypothetical protein
TGME49_224810	p.Arg1363Cys	missense_variant	hypothetical protein
TGME49_224810	p.Glu894Ala	missense_variant	hypothetical protein
TGME49_224740	c.-529A>T	5_prime_UTR_premature_start_codon_gain_variant	hypothetical protein
TGME49_224730	c.-586C>T	5_prime_UTR_premature_start_codon_gain_variant	hypothetical protein
TGME49_224730	p.Gly247Arg	missense_variant	hypothetical protein
TGME49_224700	p.Pro1165Leu	missense_variant	hypothetical protein
TGME49_224700	p.Pro749Leu	missense_variant	hypothetical protein
TGME49_224700	p.Gly501Ser	missense_variant	hypothetical protein
TGME49_224700	p.Val435Ala	missense_variant	hypothetical protein
TGME49_224700	c.-129C>T	5_prime_UTR_premature_start_codon_gain_variant	hypothetical protein
TGME49_224700	c.-186G>A	5_prime_UTR_premature_start_codon_gain_variant	hypothetical protein
TGME49_224680	p.Ser74Pro	missense_variant	hypothetical protein
TGME49_224680	p.Gly70Ala	missense_variant	hypothetical protein
TGME49_224675	p.Arg105Gly	missense_variant	hypothetical protein
TGME49_224620	c.805+7G>C	splice_region_variant	hypothetical protein
TGME49_224620	c.-857G>T	5_prime_UTR_premature_start_codon_gain_variant	hypothetical protein
TGME49_224575	p.Ile48Phe	missense_variant	hypothetical protein
TGME49_224575	c.-77C>T	5_prime_UTR_premature_start_codon_gain_variant	hypothetical protein
TGME49_224560	p.Lys104Thr	missense_variant	hypothetical protein
TGME49_224560	p.Cys98Gly	missense_variant	hypothetical protein
TGME49_224540	p.Asp4554Asn	missense_variant	hypothetical protein
TGME49_224540	p.Ala2117Val	missense_variant	hypothetical protein
TGME49_224540	p.Gly1578Glu	missense_variant	hypothetical protein
TGME49_224540	p.Glu1577Ala	missense_variant	hypothetical protein
TGME49_224540	p.Glu668Gln	missense_variant	hypothetical protein
TGME49_224540	p.Lys658Thr	missense_variant	hypothetical protein
TGME49_224540	p.Asp294Tyr	missense_variant	hypothetical protein
TGME49_224510	p.Ser1134Leu	missense_variant	hypothetical protein
TGME49_224510	p.Ala464Val	missense_variant	hypothetical protein
TGME49_224510	p.Val397Met	missense_variant	hypothetical protein
TGME49_224510	p.Ser302Leu	missense_variant	hypothetical protein
TGME49_224510	p.Arg140Ser	missense_variant	hypothetical protein
TGME49_224470	p.Arg17Gln	missense_variant	hypothetical protein
TGME49_224340	p.Phe130Leu	missense_variant	hypothetical protein
TGME49_224320	p.Ala516Val	missense_variant	hypothetical protein
TGME49_224320	p.Pro601Ser	missense_variant	hypothetical protein
TGME49_224320	p.Arg703Ser	missense_variant	hypothetical protein
TGME49_224320	p.Gly750Ser	missense_variant	hypothetical protein
TGME49_224320	p.Pro782Leu	missense_variant	hypothetical protein
TGME49_224320	p.Arg1096His	missense_variant	hypothetical protein
TGME49_224320	p.Ser1135Leu	missense_variant	hypothetical protein
TGME49_224320	p.Pro1180Leu	missense_variant	hypothetical protein
TGME49_224320	p.Pro1183Leu	missense_variant	hypothetical protein
TGME49_224320	p.Arg1572Ser	missense_variant	hypothetical protein
TGME49_224320	p.Glu1573Gly	missense_variant	hypothetical protein
TGME49_224320	p.Thr1640Ala	missense_variant	hypothetical protein
TGME49_224320	p.Ser1916Leu	missense_variant	hypothetical protein
TGME49_224320	p.Phe2097Leu	missense_variant	hypothetical protein
TGME49_224320	p.Leu2160Arg	missense_variant	hypothetical protein

TGME49_224320	p.Ser2292Thr	missense_variant	hypothetical protein
TGME49_224275	c.168-4T>G	splice_region_variant	hypothetical protein
TGME49_224270	p.Arg3326His	missense_variant	hypothetical protein
TGME49_224270	p.Leu3214Val	missense_variant	hypothetical protein
TGME49_224270	p.His2895Asn	missense_variant	hypothetical protein
TGME49_224270	p.Val2267Asp	missense_variant	hypothetical protein
TGME49_224270	p.Val2267Ile	missense_variant	hypothetical protein
TGME49_224270	p.Cys1978Tyr	missense_variant	hypothetical protein
TGME49_224270	p.Arg1936Leu	missense_variant	hypothetical protein
TGME49_224270	p.Gln1732Glu	missense_variant	hypothetical protein
TGME49_224270	p.Gly1482Arg	missense_variant	hypothetical protein
TGME49_224270	p.Thr1397Ala	missense_variant	hypothetical protein
TGME49_224270	p.Thr1284Ser	missense_variant	hypothetical protein
TGME49_224270	p.Pro1173Ala	missense_variant	hypothetical protein
TGME49_224270	p.Leu984Pro	missense_variant	hypothetical protein
TGME49_224270	p.Ala876Val	missense_variant	hypothetical protein
TGME49_224270	p.Ala666Thr	missense_variant	hypothetical protein
TGME49_224270	p.Ala571Val	missense_variant	hypothetical protein
TGME49_224270	p.Asp459Asn	missense_variant	hypothetical protein
TGME49_224270	p.Lys234Glu	missense_variant	hypothetical protein
TGME49_224270	p.Glu8Lys	missense_variant	hypothetical protein
TGME49_224210	p.Glu573Asp	missense_variant	hypothetical protein
TGME49_224210	p.Gln346His	missense_variant	hypothetical protein
TGME49_224210	p.Gly269Ala	missense_variant	hypothetical protein
TGME49_224210	p.Asn99Ile	missense_variant	hypothetical protein
TGME49_224180	p.Gln1037His	missense_variant	hypothetical protein
TGME49_224180	p.Glu796Asp	missense_variant	hypothetical protein
TGME49_224180	p.Glu787Lys	missense_variant	hypothetical protein
TGME49_224160	p.Leu254Val	missense_variant	hypothetical protein
TGME49_224160	p.Ser586Leu	missense_variant	hypothetical protein
TGME49_224160	p.Pro1747Ser	missense_variant	hypothetical protein
TGME49_224150	c.-35C>T	5_prime_UTR_premature_start_codon_gain_variant	hypothetical protein
TGME49_224150	p.Thr603Ser	missense_variant	hypothetical protein
TGME49_224150	p.Thr673Ile	missense_variant	hypothetical protein
TGME49_224150	p.Met834Val	missense_variant	hypothetical protein
TGME49_224150	p.Lys1261Arg	missense_variant	hypothetical protein
TGME49_224140	p.Phe1042Leu	missense_variant	hypothetical protein
TGME49_224140	p.Phe799Ile	missense_variant	hypothetical protein
TGME49_224140	p.Ala658Glu	missense_variant	hypothetical protein
TGME49_224140	p.Met582Lys	missense_variant	hypothetical protein
TGME49_224140	p.Arg552Gln	missense_variant	hypothetical protein
TGME49_224140	p.Ala455Gly	missense_variant	hypothetical protein
TGME49_224130	c.-161C>T	5_prime_UTR_premature_start_codon_gain_variant	hypothetical protein
TGME49_224100	p.Ile176Thr	missense_variant	hypothetical protein
TGME49_224070	p.Pro487Gln	missense_variant	hypothetical protein
TGME49_224070	p.Ala576Val	missense_variant	hypothetical protein
TGME49_224070	p.Phe853Leu	missense_variant	hypothetical protein
TGME49_224070	p.Ala939Thr	missense_variant	hypothetical protein
TGME49_224070	p.Ala987Asp	missense_variant	hypothetical protein
TGME49_224030	p.Leu1040Phe	missense_variant	hypothetical protein
TGME49_224030	p.Gly570Arg	missense_variant	hypothetical protein
TGME49_224030	p.Asp462Asn	missense_variant	hypothetical protein
TGME49_224030	p.Lys408Arg	missense_variant	hypothetical protein
TGME49_224030	p.Phe89Ile	missense_variant	hypothetical protein
TGME49_224010	p.Asp302Glu	missense_variant	hypothetical protein
TGME49_224010	p.Asn515Lys	missense_variant	hypothetical protein
TGME49_224000	p.Arg184Gln	missense_variant	hypothetical protein
TGME49_223915	p.Glu85Gln	missense_variant	hypothetical protein
TGME49_223900	p.Gln961Lys	missense_variant	hypothetical protein
TGME49_223900	p.Ala989Thr	missense_variant	hypothetical protein
TGME49_223870	p.Ala28Val	missense_variant	hypothetical protein
TGME49_223870	p.Val259Ala	missense_variant	hypothetical protein
TGME49_223870	p.Gln385Lys	missense_variant	hypothetical protein
TGME49_223870	p.Asp509Gly	missense_variant	hypothetical protein
TGME49_223810	p.Phe601Cys	missense_variant	hypothetical protein
TGME49_223810	p.Arg383Met	missense_variant	hypothetical protein
TGME49_223810	p.Glu261Gln	missense_variant	hypothetical protein
TGME49_223810	p.Arg254Gly	missense_variant	hypothetical protein
TGME49_223810	p.Ser234Tyr	missense_variant	hypothetical protein
TGME49_223810	p.Leu233Trp	missense_variant	hypothetical protein
TGME49_223810	p.Glu224Lys	missense_variant	hypothetical protein
TGME49_223810	c.-792C>T	5_prime_UTR_premature_start_codon_gain_variant	hypothetical protein
TGME49_223800	p.Arg124Thr	missense_variant	hypothetical protein
TGME49_223790	p.Gly842Asp	missense_variant	hypothetical protein
TGME49_223780	c.4146-4C>T	splice_region_variant	hypothetical protein
TGME49_223780	p.Pro525Leu	missense_variant	hypothetical protein
TGME49_223780	p.Pro525Ser	missense_variant	hypothetical protein
TGME49_223780	p.Thr450Ala	missense_variant	hypothetical protein
TGME49_223780	c.-177C>T	5_prime_UTR_premature_start_codon_gain_variant	hypothetical protein
TGME49_223780	c.-252G>A	5_prime_UTR_premature_start_codon_gain_variant	hypothetical protein
TGME49_223780	c.-768A>T	5_prime_UTR_premature_start_codon_gain_variant	hypothetical protein
TGME49_223760	c.4281+6T>C	splice_region_variant	hypothetical protein
TGME49_223760	p.Gly1142Ser	missense_variant	hypothetical protein
TGME49_223760	p.Leu1138Phe	missense_variant	hypothetical protein
TGME49_223760	p.Ala1113Pro	missense_variant	hypothetical protein

TGME49_223760	p.Arg946Pro	missense_variant	hypothetical protein
TGME49_223760	p.Ala774Thr	missense_variant	hypothetical protein
TGME49_223760	p.Asn766Ser	missense_variant	hypothetical protein
TGME49_223760	p.Leu525Pro	missense_variant	hypothetical protein
TGME49_223760	c.-723T>G	5_prime_UTR_premature_start_codon_gain_variant	hypothetical protein
TGME49_223725	p.Arg4186His	missense_variant	hypothetical protein
TGME49_223725	p.Val4067Ala	missense_variant	hypothetical protein
TGME49_223725	p.Phe3233Cys	missense_variant	hypothetical protein
TGME49_223725	p.Lys3083Asn	missense_variant	hypothetical protein
TGME49_223725	p.Ser2992Arg	missense_variant	hypothetical protein
TGME49_223725	p.Glu2979Asp	missense_variant	hypothetical protein
TGME49_223725	p.Val2940Met	missense_variant	hypothetical protein
TGME49_223725	p.Glu2896Ala	missense_variant	hypothetical protein
TGME49_223725	p.Gly2877Asp	missense_variant	hypothetical protein
TGME49_223725	p.Asp2636Asn	missense_variant	hypothetical protein
TGME49_223725	p.Ala2634Thr	missense_variant	hypothetical protein
TGME49_223725	p.Ser2580Phe	missense_variant	hypothetical protein
TGME49_223725	p.Thr2217Ala	missense_variant	hypothetical protein
TGME49_223725	p.His1935Gln	missense_variant	hypothetical protein
TGME49_223725	p.His1888Arg	missense_variant	hypothetical protein
TGME49_223725	p.Leu1707Pro	missense_variant	hypothetical protein
TGME49_223725	p.Glu1294Asp	missense_variant	hypothetical protein
TGME49_223725	p.Ser838Pro	missense_variant	hypothetical protein
TGME49_223725	p.Cys185Tyr	missense_variant	hypothetical protein
TGME49_223690	c.-228G>T	5_prime_UTR_premature_start_codon_gain_variant	hypothetical protein
TGME49_223685	c.-43C>G	5_prime_UTR_premature_start_codon_gain_variant	hypothetical protein
TGME49_223650	p.Ser52Cys	missense_variant	hypothetical protein
TGME49_223640	p.Lys197Gln	missense_variant	hypothetical protein
TGME49_223640	p.Arg244Lys	missense_variant	hypothetical protein
TGME49_223640	p.Arg494Gly	missense_variant	hypothetical protein
TGME49_223640	p.Ser748Asn	missense_variant	hypothetical protein
TGME49_223640	p.Lys793Gln	missense_variant	hypothetical protein
TGME49_223640	p.Glu1021Asp	missense_variant	hypothetical protein
TGME49_223640	p.Leu1071Val	missense_variant	hypothetical protein
TGME49_223640	p.Leu1108Gln	missense_variant	hypothetical protein
TGME49_223640	p.Leu1118Pro	missense_variant	hypothetical protein
TGME49_223640	p.Gln1288His	missense_variant	hypothetical protein
TGME49_223640	p.Ser1429Ala	missense_variant	hypothetical protein
TGME49_223640	p.Ala1579Gly	missense_variant	hypothetical protein
TGME49_223630	p.Asp424Gly	missense_variant	hypothetical protein
TGME49_223610	p.Glu1198Asp	missense_variant	hypothetical protein
TGME49_223610	p.Ala187Pro	missense_variant	hypothetical protein
TGME49_223600	p.Ile586Val	missense_variant	hypothetical protein
TGME49_223600	p.Arg253Gln	missense_variant	hypothetical protein
TGME49_223600	p.Gln236His	missense_variant	hypothetical protein
TGME49_223600	p.Pro86Ser	missense_variant	hypothetical protein
TGME49_223600	p.Val67Ala	missense_variant	hypothetical protein
TGME49_223600	p.Ser37Leu	missense_variant	hypothetical protein
TGME49_223600	c.-721G>A	5_prime_UTR_premature_start_codon_gain_variant	hypothetical protein
TGME49_223600	c.-925C>T	5_prime_UTR_premature_start_codon_gain_variant	hypothetical protein
TGME49_223570	p.Ala633Thr	missense_variant	hypothetical protein
TGME49_223570	p.Val130Leu	missense_variant	hypothetical protein
TGME49_223570	p.Arg21His	missense_variant	hypothetical protein
TGME49_223560	c.-1263C>T	5_prime_UTR_premature_start_codon_gain_variant	hypothetical protein
TGME49_223560	p.Ile254Met	missense_variant	hypothetical protein
TGME49_223550	p.Val107Leu	missense_variant	hypothetical protein
TGME49_223530	p.Asn88Ser	missense_variant	hypothetical protein
TGME49_223510	p.Ala180Val	missense_variant	hypothetical protein
TGME49_223510	c.-231T>C	5_prime_UTR_premature_start_codon_gain_variant	hypothetical protein
TGME49_223510	c.-298G>C	5_prime_UTR_premature_start_codon_gain_variant	hypothetical protein
TGME49_223500	p.Ile169Met	missense_variant	hypothetical protein
TGME49_223500	p.Gly768Ala	missense_variant	hypothetical protein
TGME49_223500	p.Met1011Val	missense_variant	hypothetical protein
TGME49_223490	p.Asp150Gly	missense_variant	hypothetical protein
TGME49_223485	p.Leu15Met	missense_variant	hypothetical protein
TGME49_223485	p.Arg33Leu	missense_variant	hypothetical protein
TGME49_223485	p.Leu114Arg	missense_variant	hypothetical protein
TGME49_223270	p.Arg1309Ser	missense_variant	hypothetical protein
TGME49_223270	c.2888-8C>A	splice_region_variant	hypothetical protein
TGME49_223270	p.Val234Leu	missense_variant	hypothetical protein
TGME49_223270	p.Val91Ala	missense_variant	hypothetical protein
TGME49_223270	p.Ala44Thr	missense_variant	hypothetical protein
TGME49_223258	p.Thr298Ala	missense_variant	hypothetical protein
TGME49_212300	p.Val73Leu	missense_variant	hypothetical protein
TGME49_212300	p.Ile641Val	missense_variant	hypothetical protein
TGME49_212270	p.Ala136Thr	missense_variant	hypothetical protein
TGME49_212220	c.-563C>T	5_prime_UTR_premature_start_codon_gain_variant	hypothetical protein
TGME49_212220	p.Gly15Asp	missense_variant	hypothetical protein
TGME49_212200	p.Gly223Trp	missense_variant	hypothetical protein
TGME49_212150	p.Ile488Leu	missense_variant	hypothetical protein
TGME49_212150	c.1025+6G>C	splice_region_variant	hypothetical protein
TGME49_212140	p.Ala115Gly	missense_variant	hypothetical protein
TGME49_212140	p.Gly793Glu	missense_variant	hypothetical protein
TGME49_212140	p.Lys833Gln	missense_variant	hypothetical protein
TGME49_212140	p.Ser1409Ala	missense_variant	hypothetical protein

TGME49_212140	p.Ala1497Gly	missense_variant	hypothetical protein
TGME49_212140	p.Glu1646Asp	missense_variant	hypothetical protein
TGME49_212140	c.5268+7T>C	splice_region_variant	hypothetical protein
TGME49_212090	p.Val122Ala	missense_variant	hypothetical protein
TGME49_212090	c.-1255C>T	5_prime_UTR_premature_start_codon_gain_variant	hypothetical protein
TGME49_212075	p.Gln238Arg	missense_variant	hypothetical protein
TGME49_212030	p.Phe435Leu	missense_variant	hypothetical protein
TGME49_212030	p.Lys320Arg	missense_variant	hypothetical protein
TGME49_211880	p.His72Pro	missense_variant	hypothetical protein
TGME49_211860	p.Asp262Gly	missense_variant	hypothetical protein
TGME49_234180	c.-489T>G	5_prime_UTR_premature_start_codon_gain_variant	hypothetical protein
TGME49_234180	p.Arg148Gly	missense_variant	hypothetical protein
TGME49_234200	p.His728Asp	missense_variant	hypothetical protein
TGME49_234200	p.Ser706Cys	missense_variant	hypothetical protein
TGME49_234200	p.Lys657Asn	missense_variant	hypothetical protein
TGME49_234200	p.Gln127His	missense_variant	hypothetical protein
TGME49_234200	p.Thr47Met	missense_variant	hypothetical protein
TGME49_234200	c.-827C>G	5_prime_UTR_premature_start_codon_gain_variant	hypothetical protein
TGME49_234220	c.-552C>G	5_prime_UTR_premature_start_codon_gain_variant	hypothetical protein
TGME49_234220	c.-500C>A	5_prime_UTR_premature_start_codon_gain_variant	hypothetical protein
TGME49_234220	p.Asn72Asp	missense_variant	hypothetical protein
TGME49_234220	p.Pro219Ser	missense_variant	hypothetical protein
TGME49_234220	p.Asp604Asn	missense_variant	hypothetical protein
TGME49_234220	p.Leu746Phe	missense_variant	hypothetical protein
TGME49_234220	p.Ser837Thr	missense_variant	hypothetical protein
TGME49_234230	p.Asp3952Glu	missense_variant	hypothetical protein
TGME49_234230	p.Ala3901Thr	missense_variant	hypothetical protein
TGME49_234230	p.Glu3857Asp	missense_variant	hypothetical protein
TGME49_234230	p.Glu3573Asp	missense_variant	hypothetical protein
TGME49_234230	p.Pro3357Arg	missense_variant	hypothetical protein
TGME49_234230	p.Ser3267Asn	missense_variant	hypothetical protein
TGME49_234230	c.8974-7C>T	splice_region_variant	hypothetical protein
TGME49_234230	p.Gly2189Ser	missense_variant	hypothetical protein
TGME49_234230	p.Arg203Gly	missense_variant	hypothetical protein
TGME49_234250	p.Gln2051Lys	missense_variant&splice_region_variant	hypothetical protein
TGME49_234250	c.5927-4C>G	splice_region_variant	hypothetical protein
TGME49_234250	p.Ala1479Thr	missense_variant	hypothetical protein
TGME49_234250	p.Ile969Thr	missense_variant	hypothetical protein
TGME49_234250	p.Pro869Ser	missense_variant	hypothetical protein
TGME49_234250	p.Trp614Leu	missense_variant	hypothetical protein
TGME49_234250	p.Ala357Val	missense_variant	hypothetical protein
TGME49_234250	p.Pro224Leu	missense_variant	hypothetical protein
TGME49_234260	p.Asp105His	missense_variant	hypothetical protein
TGME49_234270	p.Lys134Asn	missense_variant	hypothetical protein
TGME49_234270	p.Thr881Lys	missense_variant	hypothetical protein
TGME49_234270	p.Glu891Asp	missense_variant	hypothetical protein
TGME49_234270	p.Ser902Leu	missense_variant	hypothetical protein
TGME49_234270	p.Arg1575Pro	missense_variant	hypothetical protein
TGME49_234270	p.Gly1769Ser	missense_variant	hypothetical protein
TGME49_234270	p.Thr2209Ser	missense_variant	hypothetical protein
TGME49_234300	p.Arg2561Cys	missense_variant	hypothetical protein
TGME49_234300	p.Ala2286Val	missense_variant	hypothetical protein
TGME49_234300	p.Phe2124Val	missense_variant	hypothetical protein
TGME49_234300	p.Ser1957Thr	missense_variant	hypothetical protein
TGME49_234300	p.Val1952Ile	missense_variant	hypothetical protein
TGME49_234300	p.Ala1568Thr	missense_variant	hypothetical protein
TGME49_234300	p.Val1238Phe	missense_variant	hypothetical protein
TGME49_234300	p.Phe552Leu	missense_variant	hypothetical protein
TGME49_234300	p.Ala422Pro	missense_variant	hypothetical protein
TGME49_234300	p.Ser317Cys	missense_variant	hypothetical protein
TGME49_234300	p.Pro218Ser	missense_variant	hypothetical protein
TGME49_234300	p.Asp211Val	missense_variant	hypothetical protein
TGME49_234300	p.Leu96Phe	missense_variant	hypothetical protein
TGME49_234300	p.Arg66Ser	missense_variant	hypothetical protein
TGME49_234300	c.-1815A>G	5_prime_UTR_premature_start_codon_gain_variant	hypothetical protein
TGME49_234310	c.-874T>G	5_prime_UTR_premature_start_codon_gain_variant	hypothetical protein
TGME49_234340	p.Phe58Cys	missense_variant	hypothetical protein
TGME49_234350	p.Thr693Asn	missense_variant	hypothetical protein
TGME49_234350	p.Gln360His	missense_variant	hypothetical protein
TGME49_234375	c.-416T>C	5_prime_UTR_premature_start_codon_gain_variant	hypothetical protein
TGME49_234430	p.Ser2815Pro	missense_variant	hypothetical protein
TGME49_234430	p.Ala2665Thr	missense_variant	hypothetical protein
TGME49_234430	p.Ser2566Phe	missense_variant	hypothetical protein
TGME49_234430	p.Gly2468Glu	missense_variant	hypothetical protein
TGME49_234430	p.Gln2422Glu	missense_variant	hypothetical protein
TGME49_234430	p.Glu2292Lys	missense_variant	hypothetical protein
TGME49_234430	p.Ser2125Pro	missense_variant	hypothetical protein
TGME49_234430	p.Leu2122Val	missense_variant	hypothetical protein
TGME49_234430	p.Pro1947Leu	missense_variant	hypothetical protein
TGME49_234430	p.Arg1819Cys	missense_variant	hypothetical protein
TGME49_234430	p.Gly1672Arg	missense_variant	hypothetical protein
TGME49_234430	p.Met1641Thr	missense_variant	hypothetical protein
TGME49_234430	p.Leu1478His	missense_variant	hypothetical protein
TGME49_234430	p.Gly1144Asp	missense_variant	hypothetical protein
TGME49_234430	p.Arg1136Thr	missense_variant	hypothetical protein

TGME49_234430	p.Gly1062Val	missense_variant	hypothetical protein
TGME49_234430	p.Ala920Ser	missense_variant	hypothetical protein
TGME49_234430	p.Trp872Gly	missense_variant	hypothetical protein
TGME49_234430	p.Gly869Glu	missense_variant	hypothetical protein
TGME49_234430	p.Ser655Pro	missense_variant	hypothetical protein
TGME49_234430	p.Arg410Gly	missense_variant	hypothetical protein
TGME49_234430	p.Asp172Asn	missense_variant	hypothetical protein
TGME49_234430	c.-171C>G	5_prime_UTR_premature_start_codon_gain_variant	hypothetical protein
TGME49_234430	c.-653T>G	5_prime_UTR_premature_start_codon_gain_variant	hypothetical protein
TGME49_234470	c.-2236G>T	5_prime_UTR_premature_start_codon_gain_variant	hypothetical protein
TGME49_234507	p.Thr47Pro	missense_variant	hypothetical protein
TGME49_234540	p.Val449Phe	missense_variant	hypothetical protein
TGME49_234540	p.Ala216Ser	missense_variant	hypothetical protein
TGME49_234560	c.3511-4T>C	splice_region_variant	hypothetical protein
TGME49_234560	c.3510+7T>C	splice_region_variant	hypothetical protein
TGME49_234560	p.Glu1149Ala	missense_variant	hypothetical protein
TGME49_234560	p.Gly763Glu	missense_variant	hypothetical protein
TGME49_234590	p.Arg231Ile	missense_variant	hypothetical protein
TGME49_234590	p.Glu153Gln	missense_variant	hypothetical protein
TGME49_234590	p.Asn137Asp	missense_variant	hypothetical protein
TGME49_234590	p.Ala93Thr	missense_variant	hypothetical protein
TGME49_234590	p.Lys88Arg	missense_variant	hypothetical protein
TGME49_234590	p.Ala53Val	missense_variant	hypothetical protein
TGME49_234600	p.Gln326Arg	missense_variant	hypothetical protein
TGME49_234640	p.Arg348Thr	missense_variant	hypothetical protein
TGME49_234640	p.Arg368Gln	missense_variant	hypothetical protein
TGME49_234640	p.Ser638Ala	missense_variant	hypothetical protein
TGME49_234640	p.Arg1289Ser	missense_variant	hypothetical protein
TGME49_234640	p.Gly1472Ala	missense_variant	hypothetical protein
TGME49_234980	p.Ser666Asn	missense_variant	hypothetical protein
TGME49_234980	p.Arg49Gly	missense_variant	hypothetical protein
TGME49_234980	c.-1155T>C	5_prime_UTR_premature_start_codon_gain_variant	hypothetical protein
TGME49_234980	c.-2060G>T	5_prime_UTR_premature_start_codon_gain_variant	hypothetical protein
TGME49_234990	p.Gly129Ala	missense_variant	hypothetical protein
TGME49_234990	p.Thr586Ala	missense_variant	hypothetical protein
TGME49_234990	p.Phe681Leu	missense_variant	hypothetical protein
TGME49_234990	p.Asp797Glu	missense_variant	hypothetical protein
TGME49_234990	p.Ala906Thr	missense_variant	hypothetical protein
TGME49_234990	p.Val922Ile	missense_variant	hypothetical protein
TGME49_234990	p.Glu1413*	stop_gained	hypothetical protein
TGME49_235010	p.Gln14His	missense_variant	hypothetical protein
TGME49_235140	p.Ile267Met	missense_variant	hypothetical protein
TGME49_235180	p.Pro449Ala	missense_variant	hypothetical protein
TGME49_235187	p.His268Pro	missense_variant	hypothetical protein
TGME49_235187	c.2008-4C>G	splice_region_variant	hypothetical protein
TGME49_235187	p.Ile1096Met	missense_variant	hypothetical protein
TGME49_235190	p.Thr308Ile	missense_variant	hypothetical protein
TGME49_235340	p.Ala153Thr	missense_variant	hypothetical protein
TGME49_235370	c.-597C>T	5_prime_UTR_premature_start_codon_gain_variant	hypothetical protein
TGME49_235380	p.Pro132Leu	missense_variant	hypothetical protein
TGME49_235420	p.Pro3715Ser	missense_variant	hypothetical protein
TGME49_235420	p.Asn3648Ser	missense_variant	hypothetical protein
TGME49_235420	p.Ala3261Thr	missense_variant	hypothetical protein
TGME49_235420	p.Ala3053Pro	missense_variant	hypothetical protein
TGME49_235420	p.Ala2222Val	missense_variant	hypothetical protein
TGME49_235490	c.1407+8A>G	splice_region_variant	hypothetical protein
TGME49_235490	c.1762+6C>T	splice_region_variant	hypothetical protein
TGME49_235500	p.Asp633Gly	missense_variant	hypothetical protein
TGME49_235500	p.Thr572Ala	missense_variant	hypothetical protein
TGME49_235500	p.Ser551Thr	missense_variant	hypothetical protein
TGME49_235500	p.Asp494Val	missense_variant	hypothetical protein
TGME49_235500	p.Ala190Pro	missense_variant	hypothetical protein
TGME49_235500	p.Phe25Ser	missense_variant	hypothetical protein
TGME49_235500	p.Phe20Leu	missense_variant	hypothetical protein
TGME49_235560	p.Arg494Lys	missense_variant	hypothetical protein
TGME49_235560	c.506+5G>A	splice_region_variant	hypothetical protein
TGME49_235560	p.Lys136Gln	missense_variant	hypothetical protein
TGME49_235560	p.Gln107Pro	missense_variant	hypothetical protein
TGME49_235560	c.-101A>G	5_prime_UTR_premature_start_codon_gain_variant	hypothetical protein
TGME49_235630	p.Phe181Leu	missense_variant	hypothetical protein
TGME49_235635	p.Val75Ala	missense_variant	hypothetical protein
TGME49_235635	p.Ala170Ser	missense_variant	hypothetical protein
TGME49_235660	c.1498-7C>T	splice_region_variant	hypothetical protein
TGME49_235660	p.Phe64Leu	missense_variant	hypothetical protein
TGME49_235660	p.Gln49Arg	missense_variant	hypothetical protein
TGME49_235660	p.Ala22Thr	missense_variant	hypothetical protein
TGME49_235660	p.Ala12Val	missense_variant	hypothetical protein
TGME49_235670	p.Pro477Ser	missense_variant	hypothetical protein
TGME49_235690	c.-1045C>T	5_prime_UTR_premature_start_codon_gain_variant	hypothetical protein
TGME49_235710	p.Leu18Arg	missense_variant	hypothetical protein
TGME49_235720	p.Pro364Gln	missense_variant	hypothetical protein
TGME49_235730	c.-149C>T	5_prime_UTR_premature_start_codon_gain_variant	hypothetical protein
TGME49_235740	p.Val338Phe	missense_variant	hypothetical protein
TGME49_235740	p.Ser513Gly	missense_variant	hypothetical protein
TGME49_235875	c.-46A>G	5_prime_UTR_premature_start_codon_gain_variant	hypothetical protein

TGME49_235875	c.-215C>T	5_prime_UTR_premature_start_codon_gain_variant	hypothetical protein
TGME49_235875	c.-753A>G	5_prime_UTR_premature_start_codon_gain_variant	hypothetical protein
TGME49_235960	p.Val485Ala	missense_variant	hypothetical protein
TGME49_235990	p.Asp363Glu	missense_variant	hypothetical protein
TGME49_235990	c.-26+2T>G	splice_donor_variant	hypothetical protein
TGME49_236060	p.Met448Ile	missense_variant	hypothetical protein
TGME49_236060	p.Thr378Met	missense_variant	hypothetical protein
TGME49_236060	p.Ala231Val	missense_variant	hypothetical protein
TGME49_236060	p.Asp210Gly	missense_variant	hypothetical protein
TGME49_236060	p.Ser90Ala	missense_variant	hypothetical protein
TGME49_236120	p.Val102Phe	missense_variant	hypothetical protein
TGME49_236120	c.-55G>C	5_prime_UTR_premature_start_codon_gain_variant	hypothetical protein
TGME49_236120	c.-118T>G	5_prime_UTR_premature_start_codon_gain_variant	hypothetical protein
TGME49_236140	p.Arg414Ser	missense_variant	hypothetical protein
TGME49_236140	p.Ala485Ser	missense_variant	hypothetical protein
TGME49_236140	p.His733Tyr	missense_variant	hypothetical protein
TGME49_236140	p.Cys736Tyr	missense_variant	hypothetical protein
TGME49_236150	p.Lys2Arg	missense_variant	hypothetical protein
TGME49_236150	p.Leu110Val	missense_variant	hypothetical protein
TGME49_236160	p.Gly648Asp	missense_variant	hypothetical protein
TGME49_236230	p.His163Arg	missense_variant	hypothetical protein
TGME49_236230	p.Glu162Asp	missense_variant	hypothetical protein
TGME49_236230	c.-163C>T	5_prime_UTR_premature_start_codon_gain_variant	hypothetical protein
TGME49_236230	c.-637C>T	5_prime_UTR_premature_start_codon_gain_variant	hypothetical protein
TGME49_236270	c.-720T>G	5_prime_UTR_premature_start_codon_gain_variant	hypothetical protein
TGME49_236270	c.-562C>G	5_prime_UTR_premature_start_codon_gain_variant	hypothetical protein
TGME49_236270	c.-380C>G	5_prime_UTR_premature_start_codon_gain_variant	hypothetical protein
TGME49_236270	c.-376C>G	5_prime_UTR_premature_start_codon_gain_variant	hypothetical protein
TGME49_236270	c.-178C>T	5_prime_UTR_premature_start_codon_gain_variant	hypothetical protein
TGME49_236270	p.Ile349Met	missense_variant	hypothetical protein
TGME49_236270	p.Pro363Ser	missense_variant	hypothetical protein
TGME49_236290	p.Ala272Val	missense_variant	hypothetical protein
TGME49_236290	p.Ser48Phe	missense_variant	hypothetical protein
TGME49_236400	c.*415C>T	splice_region_variant	hypothetical protein
TGME49_236510	p.Thr870Ala	missense_variant	hypothetical protein
TGME49_236510	p.Glu75Lys	missense_variant	hypothetical protein
TGME49_236510	p.Ser74Phe	missense_variant	hypothetical protein
TGME49_236510	p.Met16Val	missense_variant	hypothetical protein
TGME49_236520	c.-1506A>T	5_prime_UTR_premature_start_codon_gain_variant	hypothetical protein
TGME49_236550	c.-392A>T	5_prime_UTR_premature_start_codon_gain_variant	hypothetical protein
TGME49_236560	p.Pro224Ser	missense_variant	hypothetical protein
TGME49_236560	p.His790Arg	missense_variant	hypothetical protein
TGME49_236560	p.Met1061Lys	missense_variant	hypothetical protein
TGME49_236560	p.Thr1463Ile	missense_variant	hypothetical protein
TGME49_236630	p.Val42Ala	missense_variant	hypothetical protein
TGME49_236670	p.Thr313Arg	missense_variant	hypothetical protein
TGME49_236670	p.Ile440Met	missense_variant	hypothetical protein
TGME49_236670	p.Thr580Asn	missense_variant	hypothetical protein
TGME49_236670	p.Val587Met	missense_variant	hypothetical protein
TGME49_236670	p.Asp600Glu	missense_variant	hypothetical protein
TGME49_236670	p.Leu617Met	missense_variant	hypothetical protein
TGME49_236670	p.Val771Ile	missense_variant	hypothetical protein
TGME49_236670	p.Pro905His	missense_variant	hypothetical protein
TGME49_236800	p.Asp89Glu	missense_variant	hypothetical protein
TGME49_236820	p.Thr387Ala	missense_variant	hypothetical protein
TGME49_236820	p.Leu323Pro	missense_variant	hypothetical protein
TGME49_236820	p.Thr219Ile	missense_variant	hypothetical protein
TGME49_236820	p.Ala147Val	missense_variant	hypothetical protein
TGME49_236830	p.Ala38Thr	missense_variant	hypothetical protein
TGME49_236850	p.Ala67Thr	missense_variant	hypothetical protein
TGME49_236850	p.Thr221Asn	missense_variant	hypothetical protein
TGME49_236850	p.Lys613Glu	missense_variant	hypothetical protein
TGME49_236850	p.Lys918Asn	missense_variant	hypothetical protein
TGME49_236850	p.Asp1117Asn	missense_variant	hypothetical protein
TGME49_236850	p.Ser1233Phe	missense_variant	hypothetical protein
TGME49_236850	p.Arg1552Gln	missense_variant	hypothetical protein
TGME49_236850	p.Thr1603Arg	missense_variant	hypothetical protein
TGME49_236850	p.Ala1835Thr	missense_variant	hypothetical protein
TGME49_236850	p.Ser1984Cys	missense_variant	hypothetical protein
TGME49_236850	p.Glu2296Lys	missense_variant	hypothetical protein
TGME49_236850	p.Gly2407Glu	missense_variant	hypothetical protein
TGME49_236850	p.Val2650Ala	missense_variant	hypothetical protein
TGME49_236850	p.Asn2701His	missense_variant	hypothetical protein
TGME49_236870	p.Glu204Gln	missense_variant	hypothetical protein
TGME49_236890	p.Gln203Arg	missense_variant	hypothetical protein
TGME49_236890	p.Pro135Ser	missense_variant	hypothetical protein
TGME49_236890	p.Arg69Gly	missense_variant	hypothetical protein
TGME49_236900	c.405-7C>A	splice_region_variant	hypothetical protein
TGME49_236920	c.-243C>T	5_prime_UTR_premature_start_codon_gain_variant	hypothetical protein
TGME49_236930	p.Ser5640Asn	missense_variant	hypothetical protein
TGME49_236930	p.Cys5506Ser	missense_variant	hypothetical protein
TGME49_236930	p.Thr3184Arg	missense_variant	hypothetical protein
TGME49_236930	p.Thr2215Met	missense_variant	hypothetical protein
TGME49_236930	p.Glu1303Lys	missense_variant	hypothetical protein
TGME49_236930	p.Gly1091Arg	missense_variant	hypothetical protein

TGME49_236930	p.Arg651His	missense_variant	hypothetical protein
TGME49_237015	p.Ile141Val	missense_variant	hypothetical protein
TGME49_237160	c.3352-8T>G	splice_region_variant	hypothetical protein
TGME49_237160	p.Ser971Leu	missense_variant	hypothetical protein
TGME49_237160	p.Pro220Ser	missense_variant	hypothetical protein
TGME49_237160	c.-394T>G	5_prime_UTR_premature_start_codon_gain_variant	hypothetical protein
TGME49_237195	p.Lys388Arg	missense_variant	hypothetical protein
TGME49_237240	c.-138T>G	5_prime_UTR_premature_start_codon_gain_variant	hypothetical protein
TGME49_237240	c.-163A>T	5_prime_UTR_premature_start_codon_gain_variant	hypothetical protein
TGME49_237290	p.Val2017Ile	missense_variant	hypothetical protein
TGME49_237290	p.Gln1470His	missense_variant	hypothetical protein
TGME49_237290	p.Arg957Gln	missense_variant	hypothetical protein
TGME49_237290	p.Ala49Thr	missense_variant	hypothetical protein
TGME49_237290	p.Leu39Pro	missense_variant	hypothetical protein
TGME49_237290	c.-249A>T	5_prime_UTR_premature_start_codon_gain_variant	hypothetical protein
TGME49_237290	c.-629G>A	5_prime_UTR_premature_start_codon_gain_variant	hypothetical protein
TGME49_237450	p.Pro94Ser	missense_variant	hypothetical protein
TGME49_237450	p.Ser42Trp	missense_variant	hypothetical protein
TGME49_237450	p.Asp18Ala	missense_variant	hypothetical protein
TGME49_237460	p.Pro440Thr	missense_variant	hypothetical protein
TGME49_237460	p.Asn325Ser	missense_variant	hypothetical protein
TGME49_237460	p.Cys279Gly	missense_variant	hypothetical protein
TGME49_237460	p.Ser72Phe	missense_variant	hypothetical protein
TGME49_237460	p.His19Tyr	missense_variant	hypothetical protein
TGME49_237460	c.-504T>G	5_prime_UTR_premature_start_codon_gain_variant	hypothetical protein
TGME49_237510	p.His933Pro	missense_variant	hypothetical protein
TGME49_237510	p.Ser929Leu	missense_variant	hypothetical protein
TGME49_237510	p.Glu717Ala	missense_variant	hypothetical protein
TGME49_237520	p.Ser418Asn	missense_variant	hypothetical protein
TGME49_237520	p.Ala364Thr	missense_variant	hypothetical protein
TGME49_237530	p.Arg99Cys	missense_variant	hypothetical protein
TGME49_237530	p.Pro157Gln	missense_variant	hypothetical protein
TGME49_237530	p.Gln402His	missense_variant	hypothetical protein
TGME49_237530	p.Gly962Glu	missense_variant	hypothetical protein
TGME49_237540	p.Lys144Met	missense_variant	hypothetical protein
TGME49_237550	p.Leu1117Ile	missense_variant	hypothetical protein
TGME49_237585	p.Ala1247Thr	missense_variant	hypothetical protein
TGME49_237585	p.Asn830Ser	missense_variant	hypothetical protein
TGME49_214070	p.Val41Ala	missense_variant	hypothetical protein
TGME49_214070	p.Val41Phe	missense_variant	hypothetical protein
TGME49_214115	p.Val33Met	missense_variant	hypothetical protein
TGME49_214120	p.Ala1134Thr	missense_variant	hypothetical protein
TGME49_214130	p.Lys3Glu	missense_variant	hypothetical protein
TGME49_214130	p.Glu127Gln	missense_variant	hypothetical protein
TGME49_214130	p.Val259Met	missense_variant	hypothetical protein
TGME49_214130	p.Met391Val	missense_variant	hypothetical protein
TGME49_214130	p.Pro805Leu	missense_variant	hypothetical protein
TGME49_214130	p.Ser841Ala	missense_variant	hypothetical protein
TGME49_214130	p.Ser1116Ala	missense_variant	hypothetical protein
TGME49_214130	p.Arg1518Lys	missense_variant	hypothetical protein
TGME49_214130	p.Ala1570Thr	missense_variant	hypothetical protein
TGME49_214140	p.Pro511Ala	missense_variant	hypothetical protein
TGME49_214140	p.Ala554Thr	missense_variant	hypothetical protein
TGME49_214140	p.Gly789Cys	missense_variant	hypothetical protein
TGME49_214140	p.Gly1329Ser	missense_variant	hypothetical protein
TGME49_214170	p.Gly578Ala	missense_variant	hypothetical protein
TGME49_214170	p.Ser679Phe	missense_variant	hypothetical protein
TGME49_214170	p.Ser683Phe	missense_variant	hypothetical protein
TGME49_214295	p.Ala42Ser	missense_variant	hypothetical protein
TGME49_214300	p.Met844Thr	missense_variant	hypothetical protein
TGME49_214380	p.Gly100Val	missense_variant	hypothetical protein
TGME49_214430	p.Thr370Ala	missense_variant	hypothetical protein
TGME49_214430	p.Ile436Val	missense_variant	hypothetical protein
TGME49_214510	p.Ser898Arg	missense_variant	hypothetical protein
TGME49_214540	p.Ala184Thr	missense_variant	hypothetical protein
TGME49_214545	p.Arg70Ser	missense_variant	hypothetical protein
TGME49_214545	p.Tyr54Cys	missense_variant	hypothetical protein
TGME49_214560	p.Ala836Val	missense_variant	hypothetical protein
TGME49_214560	p.Val105Leu	missense_variant	hypothetical protein
TGME49_214570	p.Gly400Arg	missense_variant	hypothetical protein
TGME49_214600	p.Thr3878Ala	missense_variant	hypothetical protein
TGME49_214600	p.Ala3644Val	missense_variant	hypothetical protein
TGME49_214600	p.Arg3270Cys	missense_variant	hypothetical protein
TGME49_214600	p.Gly2946Asp	missense_variant	hypothetical protein
TGME49_214600	p.Ser2602Leu	missense_variant	hypothetical protein
TGME49_214600	p.Arg2503His	missense_variant	hypothetical protein
TGME49_214600	p.Pro2457Gln	missense_variant	hypothetical protein
TGME49_214600	p.Ser2445Thr	missense_variant	hypothetical protein
TGME49_214600	p.Ser2360Gly	missense_variant	hypothetical protein
TGME49_214600	p.Gly2265Arg	missense_variant	hypothetical protein
TGME49_214600	p.Arg2050Ser	missense_variant	hypothetical protein
TGME49_214600	p.His1965Tyr	missense_variant	hypothetical protein
TGME49_214600	p.Asn1908Asp	missense_variant	hypothetical protein
TGME49_214600	p.Gly1611Ser	missense_variant	hypothetical protein
TGME49_214600	p.Thr1554Ala	missense_variant	hypothetical protein

TGME49_214600	p.Asp1429Gly	missense_variant	hypothetical protein
TGME49_214600	p.Met1355Val	missense_variant	hypothetical protein
TGME49_214600	p.Phe1296Leu	missense_variant	hypothetical protein
TGME49_214600	p.Val1285Ala	missense_variant	hypothetical protein
TGME49_214600	p.Ser1269Thr	missense_variant	hypothetical protein
TGME49_214600	p.Gly706Asp	missense_variant	hypothetical protein
TGME49_214600	p.Gly446Cys	missense_variant	hypothetical protein
TGME49_214600	p.Ala411Gly	missense_variant	hypothetical protein
TGME49_214600	p.Ala372Ser	missense_variant	hypothetical protein
TGME49_214600	p.Asn353Asp	missense_variant	hypothetical protein
TGME49_214600	p.Arg270Trp	missense_variant	hypothetical protein
TGME49_214600	p.Leu211Val	missense_variant	hypothetical protein
TGME49_214600	c.-382C>G	5_prime_UTR_premature_start_codon_gain_variant	hypothetical protein
TGME49_214610	p.Arg3303Gln	missense_variant	hypothetical protein
TGME49_214610	p.Pro3276Ser	missense_variant	hypothetical protein
TGME49_214610	p.Asp3043His	missense_variant	hypothetical protein
TGME49_214610	p.Glu2660Gln	missense_variant	hypothetical protein
TGME49_214610	p.Asp2655Glu	missense_variant	hypothetical protein
TGME49_214610	p.Leu2581Val	missense_variant	hypothetical protein
TGME49_214610	p.Pro2526Leu	missense_variant	hypothetical protein
TGME49_214610	p.Gly2514Ser	missense_variant	hypothetical protein
TGME49_214610	p.Pro2161Ser	missense_variant	hypothetical protein
TGME49_214610	p.Ser1486Phe	missense_variant	hypothetical protein
TGME49_214610	p.Leu1279Phe	missense_variant	hypothetical protein
TGME49_214610	p.Ser655Pro	missense_variant	hypothetical protein
TGME49_214610	p.Ser649Pro	missense_variant	hypothetical protein
TGME49_214610	p.Tyr137Phe	missense_variant	hypothetical protein
TGME49_214610	c.-90T>G	5_prime_UTR_premature_start_codon_gain_variant	hypothetical protein
TGME49_214630	p.Phe1371Val	missense_variant	hypothetical protein
TGME49_214630	p.Ser1294Pro	missense_variant	hypothetical protein
TGME49_214630	p.Phe1253Ile	missense_variant	hypothetical protein
TGME49_214630	p.Gln986Pro	missense_variant	hypothetical protein
TGME49_214630	p.Arg716Trp	missense_variant	hypothetical protein
TGME49_214630	p.Gly656Asp	missense_variant	hypothetical protein
TGME49_214630	p.Cys618Arg	missense_variant	hypothetical protein
TGME49_214630	p.Ser555Pro	missense_variant	hypothetical protein
TGME49_214630	p.Thr539Ile	missense_variant	hypothetical protein
TGME49_214630	p.Ala45Gly	missense_variant	hypothetical protein
TGME49_214630	c.-711C>T	5_prime_UTR_premature_start_codon_gain_variant	hypothetical protein
TGME49_214740	c.-2092C>G	5_prime_UTR_premature_start_codon_gain_variant	hypothetical protein
TGME49_214740	c.-1751C>T	5_prime_UTR_premature_start_codon_gain_variant	hypothetical protein
TGME49_214740	c.-1417C>T	5_prime_UTR_premature_start_codon_gain_variant	hypothetical protein
TGME49_214740	c.-204C>G	5_prime_UTR_premature_start_codon_gain_variant	hypothetical protein
TGME49_214740	p.Val92Met	missense_variant	hypothetical protein
TGME49_214740	p.Pro504Leu	missense_variant	hypothetical protein
TGME49_214740	p.Ala641Thr	missense_variant	hypothetical protein
TGME49_214740	p.Ser698Thr	missense_variant	hypothetical protein
TGME49_214740	p.Ser934Asn	missense_variant	hypothetical protein
TGME49_214783	p.Ala5Thr	missense_variant	hypothetical protein
TGME49_214800	p.Pro591Ser	missense_variant	hypothetical protein
TGME49_214830	p.Ser21Pro	missense_variant	hypothetical protein
TGME49_214830	p.Gln97Arg	missense_variant	hypothetical protein
TGME49_214830	p.Asp100Asn	missense_variant	hypothetical protein
TGME49_214830	p.Ser138Leu	missense_variant	hypothetical protein
TGME49_214830	p.Pro307Thr	missense_variant	hypothetical protein
TGME49_214830	c.2742+7C>T	splice_region_variant	hypothetical protein
TGME49_214830	c.4456-7T>C	splice_region_variant	hypothetical protein
TGME49_214880	p.Ser559Tyr	missense_variant	hypothetical protein
TGME49_214880	p.Gly556Glu	missense_variant	hypothetical protein
TGME49_214880	p.Ser197Pro	missense_variant	hypothetical protein
TGME49_214880	p.Phe123Val	missense_variant	hypothetical protein
TGME49_214900	p.Leu411Val	missense_variant	hypothetical protein
TGME49_214900	p.Ser521Pro	missense_variant	hypothetical protein
TGME49_214900	p.Ser552Ala	missense_variant	hypothetical protein
TGME49_214900	p.Thr751Ala	missense_variant	hypothetical protein
TGME49_214900	p.Pro876Leu	missense_variant	hypothetical protein
TGME49_214900	p.Gly1730Ala	missense_variant	hypothetical protein
TGME49_214900	p.Arg1776Lys	missense_variant	hypothetical protein
TGME49_214900	p.Thr1796Ala	missense_variant	hypothetical protein
TGME49_214910	p.Thr501Ala	missense_variant	hypothetical protein
TGME49_214910	p.Ala478Val	missense_variant	hypothetical protein
TGME49_214910	p.Leu360Met	missense_variant	hypothetical protein
TGME49_214910	p.Phe10Leu	missense_variant	hypothetical protein
TGME49_214980	p.Ala768Thr	missense_variant	hypothetical protein
TGME49_214990	p.Pro3430Ser	missense_variant	hypothetical protein
TGME49_214990	p.Pro2922Ser	missense_variant	hypothetical protein
TGME49_214990	p.Ala2764Thr	missense_variant	hypothetical protein
TGME49_214990	p.Pro2721Ala	missense_variant	hypothetical protein
TGME49_214990	p.Thr2517Ala	missense_variant	hypothetical protein
TGME49_214990	p.Leu2064Ile	missense_variant	hypothetical protein
TGME49_214990	p.Glu1261Lys	missense_variant	hypothetical protein
TGME49_214990	p.Met1063Ile	missense_variant	hypothetical protein
TGME49_214990	p.Asp1061Glu	missense_variant	hypothetical protein
TGME49_214990	p.Pro1004His	missense_variant	hypothetical protein
TGME49_214990	p.Ser330Ala	missense_variant	hypothetical protein

TGME49_214990	p.Ser322Ala	missense_variant	hypothetical protein
TGME49_214990	p.Leu234Val	missense_variant	hypothetical protein
TGME49_215010	c.-1191C>T	5_prime_UTR_premature_start_codon_gain_variant	hypothetical protein
TGME49_215010	p.Pro56Arg	missense_variant	hypothetical protein
TGME49_215010	p.Ala176Gly	missense_variant	hypothetical protein
TGME49_215010	p.Thr758Met	missense_variant	hypothetical protein
TGME49_215020	p.Ile92Ser	missense_variant	hypothetical protein
TGME49_215020	c.-558G>T	5_prime_UTR_premature_start_codon_gain_variant	hypothetical protein
TGME49_215030	p.Ser202Asn	missense_variant	hypothetical protein
TGME49_215030	p.Ile234Thr	missense_variant	hypothetical protein
TGME49_215030	p.Ala789Val	missense_variant	hypothetical protein
TGME49_215030	p.Ala815Ser	missense_variant	hypothetical protein
TGME49_215030	p.Gly879Arg	missense_variant	hypothetical protein
TGME49_215030	p.Asn1434His	missense_variant	hypothetical protein
TGME49_215030	p.Ala1488Pro	missense_variant	hypothetical protein
TGME49_215050	p.Ile121Met	missense_variant	hypothetical protein
TGME49_215055	p.Arg57Lys	missense_variant	hypothetical protein
TGME49_215080	p.Glu3229Asp	missense_variant	hypothetical protein
TGME49_215080	p.Ser3071Cys	missense_variant	hypothetical protein
TGME49_215080	p.Asp3026Glu	missense_variant	hypothetical protein
TGME49_215080	p.Val2498Ile	missense_variant	hypothetical protein
TGME49_215080	p.Trp1955Cys	missense_variant	hypothetical protein
TGME49_215080	p.Ser1359Ala	missense_variant	hypothetical protein
TGME49_215080	p.Gly1355Asp	missense_variant	hypothetical protein
TGME49_215080	p.Leu1183Val	missense_variant	hypothetical protein
TGME49_215080	p.Ala838Val	missense_variant	hypothetical protein
TGME49_215080	p.Val350Leu	missense_variant	hypothetical protein
TGME49_215080	p.Pro33Arg	missense_variant	hypothetical protein
TGME49_215140	p.Ile65Phe	missense_variant	hypothetical protein
TGME49_215160	c.-2866G>T	5_prime_UTR_premature_start_codon_gain_variant	hypothetical protein
TGME49_215300	p.Glu152Asp	missense_variant	hypothetical protein
TGME49_215320	p.Pro250Ser	missense_variant	hypothetical protein
TGME49_215328	c.1988-7C>T	splice_region_variant	hypothetical protein
TGME49_215328	p.Thr393Lys	missense_variant	hypothetical protein
TGME49_215343	p.Leu566Phe	missense_variant	hypothetical protein
TGME49_215347	p.Leu69Val	missense_variant	hypothetical protein
TGME49_215370	p.Arg122Gly	missense_variant	hypothetical protein
TGME49_215370	p.Ala192Ser	missense_variant	hypothetical protein
TGME49_215370	p.Asn746His	missense_variant	hypothetical protein
TGME49_215380	p.Ser583Thr	missense_variant	hypothetical protein
TGME49_215380	p.Val383Ala	missense_variant	hypothetical protein
TGME49_215380	p.Thr292Ala	missense_variant	hypothetical protein
TGME49_215380	p.Gln171Arg	missense_variant	hypothetical protein
TGME49_215540	p.Thr100Ile	missense_variant	hypothetical protein
TGME49_215550	p.Lys1446Thr	missense_variant	hypothetical protein
TGME49_215550	p.Trp1351Cys	missense_variant	hypothetical protein
TGME49_215550	p.Val1291Glu	missense_variant	hypothetical protein
TGME49_215550	p.Val488Ala	missense_variant	hypothetical protein
TGME49_215550	p.Thr471Ser	missense_variant	hypothetical protein
TGME49_215550	p.Ser274Pro	missense_variant	hypothetical protein
TGME49_215550	p.Thr172Ile	missense_variant	hypothetical protein
TGME49_215580	p.Cys108Arg	missense_variant	hypothetical protein
TGME49_215580	p.Phe24Leu	missense_variant	hypothetical protein
TGME49_215620	c.-251T>G	5_prime_UTR_premature_start_codon_gain_variant	hypothetical protein
TGME49_215620	p.Ser164Leu	missense_variant	hypothetical protein
TGME49_215620	p.Ser174Phe	missense_variant	hypothetical protein
TGME49_215620	p.Arg657Cys	missense_variant	hypothetical protein
TGME49_215620	p.Pro1280Ser	missense_variant	hypothetical protein
TGME49_215620	p.Ala1451Val	missense_variant	hypothetical protein
TGME49_215660	c.-593C>G	5_prime_UTR_premature_start_codon_gain_variant	hypothetical protein
TGME49_215660	c.-581C>T	5_prime_UTR_premature_start_codon_gain_variant	hypothetical protein
TGME49_215660	c.-240C>T	5_prime_UTR_premature_start_codon_gain_variant	hypothetical protein
TGME49_215660	p.Ala168Thr	missense_variant	hypothetical protein
TGME49_215660	p.Asn206Tyr	missense_variant	hypothetical protein
TGME49_215660	p.Ser280Pro	missense_variant	hypothetical protein
TGME49_215660	p.Arg591Thr	missense_variant	hypothetical protein
TGME49_215690	p.Asp246Glu	missense_variant	hypothetical protein
TGME49_215690	p.Ala275Ser	missense_variant	hypothetical protein
TGME49_215690	p.Ser450Gly	missense_variant	hypothetical protein
TGME49_215690	p.Lys645Thr	missense_variant	hypothetical protein
TGME49_215690	p.Asp1426Glu	missense_variant	hypothetical protein
TGME49_215710	c.-29C>G	5_prime_UTR_premature_start_codon_gain_variant	hypothetical protein
TGME49_215710	p.Pro133Gln	missense_variant	hypothetical protein
TGME49_275430	c.-1049C>T	5_prime_UTR_premature_start_codon_gain_variant	hypothetical protein
TGME49_275430	p.Leu770Val	missense_variant	hypothetical protein
TGME49_275430	p.Ser1144Asn	missense_variant	hypothetical protein
TGME49_275430	p.Val1777Ile	missense_variant	hypothetical protein
TGME49_275430	p.Val2160Ala	missense_variant	hypothetical protein
TGME49_275430	p.Arg2384Cys	missense_variant	hypothetical protein
TGME49_275430	p.Ala2552Val	missense_variant	hypothetical protein
TGME49_275430	p.Ser2583Thr	missense_variant	hypothetical protein
TGME49_275450	p.Val811Ala	missense_variant	hypothetical protein
TGME49_275450	p.Thr636Ser	missense_variant	hypothetical protein
TGME49_275450	p.Asn246Ser	missense_variant	hypothetical protein
TGME49_275450	p.Ser132Phe	missense_variant	hypothetical protein

TGME49_275450	p.Ala99Ser	missense_variant	hypothetical protein
TGME49_275490	p.Ser919Arg	missense_variant	hypothetical protein
TGME49_275490	p.His365Arg	missense_variant	hypothetical protein
TGME49_275490	p.Asp87Asn	missense_variant	hypothetical protein
TGME49_207030	c.*250C>T	splice_region_variant	hypothetical protein
TGME49_207040	p.Ala1105Pro	missense_variant	hypothetical protein
TGME49_207050	p.Ile297Leu	missense_variant	hypothetical protein
TGME49_207050	p.Pro239Leu	missense_variant	hypothetical protein
TGME49_207065	p.Leu34Val	missense_variant	hypothetical protein
TGME49_207100	p.Val1148Ile	missense_variant	hypothetical protein
TGME49_207100	p.Pro1021Ser	missense_variant	hypothetical protein
TGME49_207100	p.Val647Ala	missense_variant	hypothetical protein
TGME49_207100	p.Pro414Arg	missense_variant	hypothetical protein
TGME49_207100	p.Asp109Glu	missense_variant	hypothetical protein
TGME49_207110	p.Lys235Glu	missense_variant	hypothetical protein
TGME49_207110	p.Met1?	start_lost	hypothetical protein
TGME49_207170	c.-20C>T	5_prime_UTR_premature_start_codon_gain_variant	hypothetical protein
TGME49_207170	p.Val119Ile	missense_variant	hypothetical protein
TGME49_207200	p.Arg1535Gln	missense_variant	hypothetical protein
TGME49_207200	p.Met1240Val	missense_variant	hypothetical protein
TGME49_207200	p.His969Pro	missense_variant	hypothetical protein
TGME49_207200	p.Ser274Ala	missense_variant	hypothetical protein
TGME49_207230	p.Thr1061Ser	missense_variant	hypothetical protein
TGME49_207230	p.His828Pro	missense_variant	hypothetical protein
TGME49_207240	c.-166A>T	5_prime_UTR_premature_start_codon_gain_variant	hypothetical protein
TGME49_207240	p.Asn77Ser	missense_variant	hypothetical protein
TGME49_311230	p.Arg3096Gln	missense_variant	hypothetical protein
TGME49_312700	p.Ser2569Ala	missense_variant	hypothetical protein
TGME49_300330	p.Val9Leu	missense_variant	hypothetical protein
TGME49_300330	p.Arg228Lys	missense_variant	hypothetical protein
TGME49_300330	p.Gly997Ser	missense_variant	hypothetical protein
TGME49_300330	p.Gly1109Ser	missense_variant	hypothetical protein
TGME49_300270	c.-2222C>G	5_prime_UTR_premature_start_codon_gain_variant	hypothetical protein
TGME49_300270	c.-1995C>G	5_prime_UTR_premature_start_codon_gain_variant	hypothetical protein
TGME49_300270	c.-491C>T	5_prime_UTR_premature_start_codon_gain_variant	hypothetical protein
TGME49_300220	p.Val1689Met	missense_variant	hypothetical protein
TGME49_300220	p.Asn1513Ser	missense_variant	hypothetical protein
TGME49_300220	p.Asn15Ser	missense_variant	hypothetical protein
TGME49_300180	p.Gly94Val	missense_variant	hypothetical protein
TGME49_300180	p.Ser46Ala	missense_variant	hypothetical protein
TGME49_300160	p.Ala159Val	missense_variant	hypothetical protein
TGME49_300160	p.Glu280Ala	missense_variant	hypothetical protein
TGME49_300160	p.Glu353Val	missense_variant	hypothetical protein
TGME49_300110	c.-563C>T	5_prime_UTR_premature_start_codon_gain_variant	hypothetical protein
TGME49_300110	c.-272C>G	5_prime_UTR_premature_start_codon_gain_variant	hypothetical protein
TGME49_300090	p.Asp25His	missense_variant	hypothetical protein
TGME49_300090	p.Pro74Ser	missense_variant	hypothetical protein
TGME49_300090	p.Leu111Phe	missense_variant	hypothetical protein
TGME49_300090	p.Ser113Leu	missense_variant	hypothetical protein
TGME49_300080	c.*24C>T	splice_region_variant	hypothetical protein
TGME49_300080	p.Glu48Ala	missense_variant	hypothetical protein
TGME49_300055	p.Ser165Tyr	missense_variant	hypothetical protein
TGME49_300055	p.Ser153Phe	missense_variant	hypothetical protein
TGME49_300052	p.Val40Gly	missense_variant	hypothetical protein
TGME49_300048	p.Asn272Asp	missense_variant	hypothetical protein
TGME49_300048	p.Phe19Ser	missense_variant	hypothetical protein
TGME49_300030	p.Asp326His	missense_variant	hypothetical protein
TGME49_300030	p.Ala225Ser	missense_variant	hypothetical protein
TGME49_300010	p.Glu279Asp	missense_variant	hypothetical protein
TGME49_300010	p.Ala277Glu	missense_variant	hypothetical protein
TGME49_299995	p.Arg50Thr	missense_variant	hypothetical protein
TGME49_299980	p.Gln156Arg	missense_variant	hypothetical protein
TGME49_299780	p.Pro287Ala	missense_variant	hypothetical protein
TGME49_299670	p.Thr370Ser	missense_variant	hypothetical protein
TGME49_307820	c.74-4C>T	splice_region_variant	hypothetical protein
TGME49_307820	p.Glu36Lys	missense_variant	hypothetical protein
TGME49_307860	p.Ser39Leu	missense_variant	hypothetical protein
TGME49_307860	p.Glu99Asp	missense_variant	hypothetical protein
TGME49_307860	p.Pro1041Ala	missense_variant	hypothetical protein
TGME49_307860	p.Cys1273Tyr	missense_variant	hypothetical protein
TGME49_307860	p.Gly1589Val	missense_variant	hypothetical protein
TGME49_307860	p.Glu2322Asp	missense_variant	hypothetical protein
TGME49_307860	p.Pro2905Ala	missense_variant	hypothetical protein
TGME49_307860	p.Arg2926His	missense_variant	hypothetical protein
TGME49_307860	p.Ala2930Gly	missense_variant	hypothetical protein
TGME49_307860	p.Ile3284Ser	missense_variant	hypothetical protein
TGME49_307860	p.Leu3803Val	missense_variant	hypothetical protein
TGME49_307860	p.Gln3876Glu	missense_variant	hypothetical protein
TGME49_307860	p.Thr3897Met	missense_variant	hypothetical protein
TGME49_308010	c.*549C>A	splice_region_variant	hypothetical protein
TGME49_308010	p.Ala1139Gly	missense_variant	hypothetical protein
TGME49_308010	p.Glu881Gln	missense_variant	hypothetical protein
TGME49_308010	p.Glu839Gln	missense_variant	hypothetical protein
TGME49_308010	p.Lys687Asn	missense_variant	hypothetical protein
TGME49_308010	p.Ala378Ser	missense_variant	hypothetical protein

TGME49_308010	c.-733T>C	5_prime_UTR_premature_start_codon_gain_variant	hypothetical protein
TGME49_308030	p.Ile17Val	missense_variant	hypothetical protein
TGME49_308030	p.Ile813Thr	missense_variant	hypothetical protein
TGME49_308030	c.2637+8T>C	splice_region_variant	hypothetical protein
TGME49_308060	p.Asp89Ala	missense_variant	hypothetical protein
TGME49_308060	p.Phe1483Leu	missense_variant	hypothetical protein
TGME49_308060	p.Gln1829Glu	missense_variant	hypothetical protein
TGME49_308070	p.Ala1212Val	missense_variant	hypothetical protein
TGME49_219810	p.Thr410Ser	missense_variant	hypothetical protein
TGME49_219742	p.Pro27Ser	missense_variant	hypothetical protein
TGME49_219742	p.Gly192Glu	missense_variant	hypothetical protein
TGME49_219738	p.Leu129Arg	missense_variant	hypothetical protein
TGME49_219738	p.Arg318Gln	missense_variant	hypothetical protein
TGME49_219738	p.Asp803Glu	missense_variant	hypothetical protein
TGME49_219738	p.Val817Phe	missense_variant	hypothetical protein
TGME49_219738	p.Thr1478Lys	missense_variant	hypothetical protein
TGME49_219738	p.Ala1650Glu	missense_variant	hypothetical protein
TGME49_219738	p.Pro1682Ser	missense_variant	hypothetical protein
TGME49_219738	p.Thr1756Ala	missense_variant	hypothetical protein
TGME49_219738	p.Lys1874Arg	missense_variant	hypothetical protein
TGME49_219738	p.Leu1877Arg	missense_variant	hypothetical protein
TGME49_219738	p.Val1927Ala	missense_variant	hypothetical protein
TGME49_219738	p.Leu1977Ile	missense_variant	hypothetical protein
TGME49_219738	p.Val2238Ala	missense_variant	hypothetical protein
TGME49_219738	p.Ala2273Thr	missense_variant	hypothetical protein
TGME49_219738	p.Leu2311Ile	missense_variant	hypothetical protein
TGME49_219738	p.Met2484Ile	missense_variant	hypothetical protein
TGME49_219730	c.-604A>G	5_prime_UTR_premature_start_codon_gain_variant	hypothetical protein
TGME49_219730	c.-442T>G	5_prime_UTR_premature_start_codon_gain_variant	hypothetical protein
TGME49_219730	p.Glu111Lys	missense_variant	hypothetical protein
TGME49_219730	p.Glu137Asp	missense_variant	hypothetical protein
TGME49_219730	p.Gly173Arg	missense_variant	hypothetical protein
TGME49_219730	p.Ser286Asn	missense_variant	hypothetical protein
TGME49_219730	p.Ser316Arg	missense_variant	hypothetical protein
TGME49_219710	p.His1323Gln	missense_variant	hypothetical protein
TGME49_219710	p.Gly817Ser	missense_variant	hypothetical protein
TGME49_219678	c.-218A>C	5_prime_UTR_premature_start_codon_gain_variant	hypothetical protein
TGME49_219678	p.Gly390Arg	missense_variant	hypothetical protein
TGME49_219678	p.Ser443Asn	missense_variant	hypothetical protein
TGME49_219660	p.His3840Arg	missense_variant	hypothetical protein
TGME49_219660	p.Ala2912Val	missense_variant	hypothetical protein
TGME49_219660	p.Pro2278Thr	missense_variant	hypothetical protein
TGME49_219660	p.Thr1481Ile	missense_variant	hypothetical protein
TGME49_219660	p.Ala1068Gly	missense_variant	hypothetical protein
TGME49_219640	p.Ala192Val	missense_variant	hypothetical protein
TGME49_219640	p.Gly219Arg	missense_variant	hypothetical protein
TGME49_219640	p.Glu924Asp	missense_variant	hypothetical protein
TGME49_219640	p.Gln1125His	missense_variant	hypothetical protein
TGME49_219640	p.Glu1260Gly	missense_variant	hypothetical protein
TGME49_219640	p.Leu1699Val	missense_variant	hypothetical protein
TGME49_219640	c.*485G>A	splice_region_variant	hypothetical protein
TGME49_219620	p.His247Asp	missense_variant	hypothetical protein
TGME49_219620	p.Ser112Pro	missense_variant	hypothetical protein
TGME49_219620	c.-140C>T	5_prime_UTR_premature_start_codon_gain_variant	hypothetical protein
TGME49_219610	p.Val74Leu	missense_variant	hypothetical protein
TGME49_219610	p.Ala105Val	missense_variant	hypothetical protein
TGME49_219610	c.388-6T>C	splice_region_variant	hypothetical protein
TGME49_219610	p.Arg168Gly	missense_variant	hypothetical protein
TGME49_219610	p.Arg174Gly	missense_variant	hypothetical protein
TGME49_219610	p.Ala203Thr	missense_variant	hypothetical protein
TGME49_219600	c.-773A>C	5_prime_UTR_premature_start_codon_gain_variant	hypothetical protein
TGME49_219600	p.Val92Met	missense_variant	hypothetical protein
TGME49_219600	p.Ala199Gly	missense_variant	hypothetical protein
TGME49_219600	p.Gln419Arg	missense_variant	hypothetical protein
TGME49_219600	p.Ala554Ser	missense_variant	hypothetical protein
TGME49_219600	p.Ser566Leu	missense_variant	hypothetical protein
TGME49_219600	p.Gly771Arg	missense_variant	hypothetical protein
TGME49_219600	p.Ala784Asp	missense_variant	hypothetical protein
TGME49_219570	c.-501G>T	5_prime_UTR_premature_start_codon_gain_variant	hypothetical protein
TGME49_219530	p.Gln216Arg	missense_variant	hypothetical protein
TGME49_219530	p.Arg23Lys	missense_variant	hypothetical protein
TGME49_219530	p.Glu15Lys	missense_variant	hypothetical protein
TGME49_219530	c.-421C>T	5_prime_UTR_premature_start_codon_gain_variant	hypothetical protein
TGME49_219500	c.967-6C>T	splice_region_variant	hypothetical protein
TGME49_219485	p.Asn2327Lys	missense_variant	hypothetical protein
TGME49_219485	p.Ala2016Val	missense_variant	hypothetical protein
TGME49_219485	p.Ser1102Ala	missense_variant	hypothetical protein
TGME49_219485	p.Gly741Ser	missense_variant	hypothetical protein
TGME49_219485	p.Gly543Glu	missense_variant	hypothetical protein
TGME49_219485	p.Asp389Gly	missense_variant	hypothetical protein
TGME49_219485	p.Ala216Ser	missense_variant	hypothetical protein
TGME49_219485	p.Ala211Thr	missense_variant	hypothetical protein
TGME49_219485	p.Leu122Pro	missense_variant	hypothetical protein
TGME49_219485	p.Arg98Ser	missense_variant	hypothetical protein
TGME49_219485	c.-770G>A	5_prime_UTR_premature_start_codon_gain_variant	hypothetical protein

TGME49_219460	p.Pro661Ser	missense_variant	hypothetical protein
TGME49_219460	p.Glu662Gly	missense_variant	hypothetical protein
TGME49_219460	p.Ala773Ser	missense_variant	hypothetical protein
TGME49_219460	p.Thr834Ser	missense_variant	hypothetical protein
TGME49_219440	p.His670Leu	missense_variant	hypothetical protein
TGME49_219222	p.Lys148Arg	missense_variant	hypothetical protein
TGME49_219218	p.Arg1366Gly	missense_variant	hypothetical protein
TGME49_219218	p.Glu1334Gly	missense_variant	hypothetical protein
TGME49_219218	p.Gly986Ala	missense_variant	hypothetical protein
TGME49_219218	c.-1588T>G	5_prime_UTR_premature_start_codon_gain_variant	hypothetical protein
TGME49_219218	c.-2167C>T	5_prime_UTR_premature_start_codon_gain_variant	hypothetical protein
TGME49_219218	c.-3391G>A	5_prime_UTR_premature_start_codon_gain_variant	hypothetical protein
TGME49_219160	c.-186G>T	5_prime_UTR_premature_start_codon_gain_variant	hypothetical protein
TGME49_219160	c.268+6G>T	splice_region_variant	hypothetical protein
TGME49_219110	p.Thr238Ala	missense_variant	hypothetical protein
TGME49_219110	p.Glu186Lys	missense_variant	hypothetical protein
TGME49_219110	p.Ile164Thr	missense_variant	hypothetical protein
TGME49_219110	c.213+6T>C	splice_region_variant	hypothetical protein
TGME49_219110	c.174+3A>G	splice_region_variant	hypothetical protein
TGME49_218950	p.Ala1152Ser	missense_variant	hypothetical protein
TGME49_218950	p.Phe731Ile	missense_variant	hypothetical protein
TGME49_218950	p.Ala678Thr	missense_variant	hypothetical protein
TGME49_218950	c.1905+6G>A	splice_region_variant	hypothetical protein
TGME49_218950	p.Glu551Val	missense_variant	hypothetical protein
TGME49_218950	p.Ser237Phe	missense_variant	hypothetical protein
TGME49_218950	p.Gly207Asp	missense_variant	hypothetical protein
TGME49_218940	p.Gly9Ala	missense_variant	hypothetical protein
TGME49_218940	p.Gln67*	stop_gained	hypothetical protein
TGME49_218940	c.205+8G>A	splice_region_variant	hypothetical protein
TGME49_218950	c.-365G>T	5_prime_UTR_premature_start_codon_gain_variant	hypothetical protein
TGME49_218940	p.Ala138Val	missense_variant	hypothetical protein
TGME49_218910	p.Asp538Asn	missense_variant	hypothetical protein
TGME49_218910	p.Asp491Gly	missense_variant	hypothetical protein
TGME49_218900	p.Ala90Gly	missense_variant	hypothetical protein
TGME49_218900	p.Thr666Ala	missense_variant	hypothetical protein
TGME49_218890	p.Gly651Ser	missense_variant	hypothetical protein
TGME49_218860	p.Gly173Arg	missense_variant	hypothetical protein
TGME49_218830	p.Ser627Phe	missense_variant	hypothetical protein
TGME49_218830	p.Leu76Val	missense_variant	hypothetical protein
TGME49_218800	p.Glu883Asp	missense_variant	hypothetical protein
TGME49_218800	c.2161-5T>C	splice_region_variant	hypothetical protein
TGME49_218800	p.Pro605Ala	missense_variant	hypothetical protein
TGME49_218800	p.Pro389Leu	missense_variant	hypothetical protein
TGME49_218800	p.Arg126Leu	missense_variant	hypothetical protein
TGME49_218800	p.Pro113Ser	missense_variant	hypothetical protein
TGME49_218800	p.Gly107Arg	missense_variant	hypothetical protein
TGME49_218800	p.His5Gln	missense_variant	hypothetical protein
TGME49_218750	p.Asn268Asp	missense_variant	hypothetical protein
TGME49_218750	p.Ala226Thr	missense_variant	hypothetical protein
TGME49_218750	p.Asn130Lys	missense_variant	hypothetical protein
TGME49_218750	c.-1654G>A	5_prime_UTR_premature_start_codon_gain_variant	hypothetical protein
TGME49_218730	p.Gly207Arg	missense_variant	hypothetical protein
TGME49_218500	p.Lys1362Glu	missense_variant	hypothetical protein
TGME49_218500	p.Ser425Asn	missense_variant	hypothetical protein
TGME49_218490	p.Lys1618Gln	missense_variant	hypothetical protein
TGME49_218490	p.Val1509Ile	missense_variant	hypothetical protein
TGME49_218490	c.1297+7G>A	splice_region_variant	hypothetical protein
TGME49_218490	p.Arg404Gln	missense_variant	hypothetical protein
TGME49_218490	p.Ala308Val	missense_variant	hypothetical protein
TGME49_218490	p.Ser105Pro	missense_variant	hypothetical protein
TGME49_218490	p.Arg67Leu	missense_variant	hypothetical protein
TGME49_218460	p.Gly12Val	missense_variant	hypothetical protein
TGME49_218460	p.Gln161His	missense_variant	hypothetical protein
TGME49_218450	p.Met125Val	missense_variant	hypothetical protein
TGME49_218450	p.Ala263Pro	missense_variant	hypothetical protein
TGME49_218450	p.Gly288Asp	missense_variant	hypothetical protein
TGME49_218450	p.Gly567Ser	missense_variant	hypothetical protein
TGME49_218390	p.Ser44Arg	missense_variant	hypothetical protein
TGME49_218390	p.Ile123Met	missense_variant	hypothetical protein
TGME49_218240	p.Glu700Asp	missense_variant	hypothetical protein
TGME49_218240	p.Pro223Ala	missense_variant	hypothetical protein
TGME49_218240	p.Phe205Val	missense_variant	hypothetical protein
TGME49_218215	c.1098-5T>C	splice_region_variant	hypothetical protein
TGME49_218215	p.Ser168Asn	missense_variant	hypothetical protein
TGME49_218188	c.2691+3G>C	splice_region_variant	hypothetical protein
TGME49_218188	p.Ala394Val	missense_variant	hypothetical protein
TGME49_218188	p.Arg355Leu	missense_variant	hypothetical protein
TGME49_218188	p.Ala147Glu	missense_variant	hypothetical protein
TGME49_218188	p.Met8Val	missense_variant	hypothetical protein
TGME49_218188	c.-33C>T	5_prime_UTR_premature_start_codon_gain_variant	hypothetical protein
TGME49_218188	c.-303C>T	5_prime_UTR_premature_start_codon_gain_variant	hypothetical protein
TGME49_218130	p.Pro29Leu	missense_variant	hypothetical protein
TGME49_218130	c.-343C>G	5_prime_UTR_premature_start_codon_gain_variant	hypothetical protein
TGME49_218070	p.Gln1392Arg	missense_variant	hypothetical protein
TGME49_218070	p.Ala1382Val	missense_variant	hypothetical protein

TGME49_218070	p.Phe1275Val	missense_variant	hypothetical protein
TGME49_218070	p.Lys1182Met	missense_variant	hypothetical protein
TGME49_218070	p.Arg1137Ser	missense_variant	hypothetical protein
TGME49_218070	c.1687-5C>T	splice_region_variant	hypothetical protein
TGME49_218070	p.Thr293Ser	missense_variant	hypothetical protein
TGME49_218000	p.Thr3227Ile	missense_variant	hypothetical protein
TGME49_218000	p.Ser3221Leu	missense_variant	hypothetical protein
TGME49_218000	p.Ala3142Val	missense_variant	hypothetical protein
TGME49_218000	p.Asp3041Asn	missense_variant	hypothetical protein
TGME49_218000	p.Glu3028Val	missense_variant	hypothetical protein
TGME49_218000	p.Thr2918Ala	missense_variant	hypothetical protein
TGME49_218000	p.Val2356Met	missense_variant	hypothetical protein
TGME49_218000	p.Phe1834Leu	missense_variant	hypothetical protein
TGME49_218000	p.Ala1736Ser	missense_variant	hypothetical protein
TGME49_218000	p.Ser1732Trp	missense_variant	hypothetical protein
TGME49_218000	p.Arg1691Thr	missense_variant	hypothetical protein
TGME49_218000	p.Arg1691Gly	missense_variant	hypothetical protein
TGME49_218000	p.Glu1460Ala	missense_variant	hypothetical protein
TGME49_218000	p.His1189Pro	missense_variant	hypothetical protein
TGME49_218000	p.Lys1032Arg	missense_variant	hypothetical protein
TGME49_218000	p.Ala878Pro	missense_variant	hypothetical protein
TGME49_218000	p.Ser787Ala	missense_variant	hypothetical protein
TGME49_218000	p.Gln713Pro	missense_variant	hypothetical protein
TGME49_218000	p.Gln708Pro	missense_variant	hypothetical protein
TGME49_218000	p.Gly476Ser	missense_variant	hypothetical protein
TGME49_218000	p.Gly473Ser	missense_variant	hypothetical protein
TGME49_217340	c.-541C>T	5_prime_UTR_premature_start_codon_gain_variant	hypothetical protein
TGME49_217340	c.-454C>G	5_prime_UTR_premature_start_codon_gain_variant	hypothetical protein
TGME49_217340	c.-402C>T	5_prime_UTR_premature_start_codon_gain_variant	hypothetical protein
TGME49_217340	c.-157G>A	5_prime_UTR_premature_start_codon_gain_variant	hypothetical protein
TGME49_217340	c.2109+4A>T	splice_region_variant	hypothetical protein
TGME49_217360	c.-989C>T	5_prime_UTR_premature_start_codon_gain_variant	hypothetical protein
TGME49_217360	p.Ala552Thr	missense_variant	hypothetical protein
TGME49_217360	p.Lys562Arg	missense_variant	hypothetical protein
TGME49_217360	p.Arg1921Trp	missense_variant	hypothetical protein
TGME49_217380	p.Pro482Ser	missense_variant	hypothetical protein
TGME49_217390	p.Glu631Lys	missense_variant	hypothetical protein
TGME49_217390	p.Thr418Ile	missense_variant	hypothetical protein
TGME49_217400	p.Ala418Val	missense_variant	hypothetical protein
TGME49_217410	p.His110Arg	missense_variant	hypothetical protein
TGME49_217470	p.Ala30Pro	missense_variant	hypothetical protein
TGME49_217470	p.Arg210Cys	missense_variant	hypothetical protein
TGME49_217470	p.Asn386Ser	missense_variant	hypothetical protein
TGME49_217470	p.Ala757Thr	missense_variant	hypothetical protein
TGME49_217480	c.-2096T>C	5_prime_UTR_premature_start_codon_gain_variant	hypothetical protein
TGME49_217480	c.-668C>G	5_prime_UTR_premature_start_codon_gain_variant	hypothetical protein
TGME49_217480	c.3148+3A>G	splice_region_variant	hypothetical protein
TGME49_217480	p.Val1463Met	missense_variant	hypothetical protein
TGME49_217490	p.Glu95Ala	missense_variant	hypothetical protein
TGME49_217490	p.Leu179Met	missense_variant	hypothetical protein
TGME49_217490	c.627-6G>A	splice_region_variant	hypothetical protein
TGME49_217520	c.*907C>T	splice_region_variant	hypothetical protein
TGME49_217520	p.Ser44Ala	missense_variant	hypothetical protein
TGME49_217520	c.-781C>T	5_prime_UTR_premature_start_codon_gain_variant	hypothetical protein
TGME49_217520	c.-885A>T	5_prime_UTR_premature_start_codon_gain_variant	hypothetical protein
TGME49_217530	p.Pro44Ser	missense_variant	hypothetical protein
TGME49_217530	p.Arg209Gly	missense_variant	hypothetical protein
TGME49_217530	p.Thr248Ile	missense_variant	hypothetical protein
TGME49_217610	p.Ala691Thr	missense_variant	hypothetical protein
TGME49_217610	p.Gln550Leu	missense_variant	hypothetical protein
TGME49_217620	p.Asn241Ser	missense_variant	hypothetical protein
TGME49_217620	p.Leu230Pro	missense_variant	hypothetical protein
TGME49_217620	p.His109Asn	missense_variant	hypothetical protein
TGME49_217620	p.Ile78Val	missense_variant	hypothetical protein
TGME49_217620	c.-453G>C	5_prime_UTR_premature_start_codon_gain_variant	hypothetical protein
TGME49_217630	c.-1016G>C	5_prime_UTR_premature_start_codon_gain_variant	hypothetical protein
TGME49_217630	p.Arg80Gln	missense_variant	hypothetical protein
TGME49_217630	p.Ala160Thr	missense_variant	hypothetical protein
TGME49_217630	p.Ser464Leu	missense_variant	hypothetical protein
TGME49_217630	p.Asp745Gly	missense_variant	hypothetical protein
TGME49_217630	p.Ser976Gly	missense_variant	hypothetical protein
TGME49_217630	p.His992Tyr	missense_variant	hypothetical protein
TGME49_217650	p.Val483Ile	missense_variant	hypothetical protein
TGME49_217665	c.-194C>T	5_prime_UTR_premature_start_codon_gain_variant	hypothetical protein
TGME49_217680	p.Pro2154Leu	missense_variant	hypothetical protein
TGME49_217680	p.Gln2149His	missense_variant	hypothetical protein
TGME49_217680	p.Leu2130Arg	missense_variant	hypothetical protein
TGME49_217680	p.Met2129Val	missense_variant	hypothetical protein
TGME49_217680	p.Glu1934Lys	missense_variant	hypothetical protein
TGME49_217680	p.Leu1542Val	missense_variant	hypothetical protein
TGME49_217680	p.Leu1116Val	missense_variant	hypothetical protein
TGME49_217680	p.Val593Ala	missense_variant	hypothetical protein
TGME49_217680	p.Val593Leu	missense_variant	hypothetical protein
TGME49_217680	c.1123+3C>T	splice_region_variant	hypothetical protein
TGME49_217680	p.His117Asp	missense_variant	hypothetical protein

TGME49_217680	p.Pro93Leu	missense_variant	hypothetical protein
TGME49_217692	p.Ala1316Gly	missense_variant	hypothetical protein
TGME49_217692	p.Glu789Gly	missense_variant	hypothetical protein
TGME49_217692	p.Leu213Arg	missense_variant	hypothetical protein
TGME49_217688	p.Thr394Met	missense_variant	hypothetical protein
TGME49_217688	p.Pro355Leu	missense_variant	hypothetical protein
TGME49_217688	p.Leu283Val	missense_variant	hypothetical protein
TGME49_217688	c.-1698G>A	5_prime_UTR_premature_start_codon_gain_variant	hypothetical protein
TGME49_217720	c.*442C>A	splice_region_variant	hypothetical protein
TGME49_217724	p.Asn73Lys	missense_variant	hypothetical protein
TGME49_217724	p.Pro74Ser	missense_variant	hypothetical protein
TGME49_217726	p.Thr3Ala	missense_variant	hypothetical protein
TGME49_217726	p.Pro11Leu	missense_variant	hypothetical protein
TGME49_217726	p.Ile31Thr	missense_variant	hypothetical protein
TGME49_217728	p.Pro34Gln	missense_variant	hypothetical protein
TGME49_217730	p.Asp1068Gly	missense_variant	hypothetical protein
TGME49_217730	c.3052+7A>C	splice_region_variant	hypothetical protein
TGME49_217730	p.Arg900Leu	missense_variant	hypothetical protein
TGME49_217730	p.Ala892Pro	missense_variant	hypothetical protein
TGME49_217730	p.Phe748Ser	missense_variant	hypothetical protein
TGME49_217730	p.Arg631His	missense_variant	hypothetical protein
TGME49_217730	p.Arg609Gly	missense_variant	hypothetical protein
TGME49_217730	p.Ser600Pro	missense_variant	hypothetical protein
TGME49_217730	p.Arg272Lys	missense_variant	hypothetical protein
TGME49_217730	c.-413C>T	5_prime_UTR_premature_start_codon_gain_variant	hypothetical protein
TGME49_217750	p.Gly18Asp	missense_variant	hypothetical protein
TGME49_217750	p.Pro111Gln	missense_variant	hypothetical protein
TGME49_217750	p.Cys136Trp	missense_variant	hypothetical protein
TGME49_217750	p.Ile143Thr	missense_variant	hypothetical protein
TGME49_217770	c.-645G>A	5_prime_UTR_premature_start_codon_gain_variant	hypothetical protein
TGME49_217770	c.-397G>T	5_prime_UTR_premature_start_codon_gain_variant	hypothetical protein
TGME49_217770	p.Asp59Asn	missense_variant	hypothetical protein
TGME49_217770	p.Asn161Thr	missense_variant	hypothetical protein
TGME49_217770	p.Gly470Arg	missense_variant	hypothetical protein
TGME49_217770	p.Ser535Pro	missense_variant	hypothetical protein
TGME49_217770	p.Arg695Trp	missense_variant	hypothetical protein
TGME49_217770	p.Phe702Leu	missense_variant	hypothetical protein
TGME49_217770	p.Glu819Gln	missense_variant	hypothetical protein
TGME49_217770	p.Glu900Gln	missense_variant	hypothetical protein
TGME49_217770	p.Asp1114Glu	missense_variant	hypothetical protein
TGME49_217770	p.Val1359Glu	missense_variant	hypothetical protein
TGME49_217800	p.Arg1171His	missense_variant	hypothetical protein
TGME49_217800	p.Arg1048Lys	missense_variant	hypothetical protein
TGME49_217800	p.Arg864Gln	missense_variant	hypothetical protein
TGME49_217800	p.Ala833Thr	missense_variant	hypothetical protein
TGME49_217800	p.Glu758Asp	missense_variant	hypothetical protein
TGME49_217800	p.Pro477Ala	missense_variant	hypothetical protein
TGME49_217800	p.Leu440Val	missense_variant	hypothetical protein
TGME49_217810	p.Ser156Pro	missense_variant	hypothetical protein
TGME49_217830	p.Asp1872Gly	missense_variant	hypothetical protein
TGME49_217830	p.Ser1779Cys	missense_variant	hypothetical protein
TGME49_217830	p.Asp1758Asn	missense_variant	hypothetical protein
TGME49_217830	c.5015-7G>A	splice_region_variant	hypothetical protein
TGME49_217830	p.Ala1114Gly	missense_variant	hypothetical protein
TGME49_217830	p.Thr1079Ala	missense_variant	hypothetical protein
TGME49_217830	p.Ala859Thr	missense_variant	hypothetical protein
TGME49_217830	p.Glu192Asp	missense_variant	hypothetical protein
TGME49_217860	c.-333C>T	5_prime_UTR_premature_start_codon_gain_variant	hypothetical protein
TGME49_217860	c.-139G>T	5_prime_UTR_premature_start_codon_gain_variant	hypothetical protein
TGME49_217860	p.Gly20Ala	missense_variant	hypothetical protein
TGME49_217860	p.Ala36Ser	missense_variant	hypothetical protein
TGME49_217860	p.Ala204Gly	missense_variant	hypothetical protein
TGME49_217860	p.Cys228Arg	missense_variant	hypothetical protein
TGME49_217860	p.Phe330Ser	missense_variant	hypothetical protein
TGME49_217860	p.His461Arg	missense_variant	hypothetical protein
TGME49_217860	p.Thr738Ser	missense_variant	hypothetical protein
TGME49_217860	p.Thr949Ala	missense_variant	hypothetical protein
TGME49_217860	p.Asp1161Asn	missense_variant	hypothetical protein
TGME49_217860	p.Ala1171Thr	missense_variant	hypothetical protein
TGME49_217860	p.Phe1261Leu	missense_variant	hypothetical protein
TGME49_217860	p.Ala1396Pro	missense_variant	hypothetical protein
TGME49_217860	p.Met1594Leu	missense_variant	hypothetical protein
TGME49_217860	p.Ala1731Glu	missense_variant	hypothetical protein
TGME49_217860	p.Ser1789Gly	missense_variant	hypothetical protein
TGME49_217860	p.Glu2096Asp	missense_variant	hypothetical protein
TGME49_217860	p.Ala2304Val	missense_variant	hypothetical protein
TGME49_217860	p.Thr2306Met	missense_variant	hypothetical protein
TGME49_217860	p.Pro2362Gln	missense_variant	hypothetical protein
TGME49_217860	p.Lys2569Asn	missense_variant	hypothetical protein
TGME49_217860	p.Phe2572Leu	missense_variant	hypothetical protein
TGME49_217860	p.Gly2618Asp	missense_variant	hypothetical protein
TGME49_217860	p.Asp2853His	missense_variant	hypothetical protein
TGME49_217900	p.Glu171Asp	missense_variant	hypothetical protein
TGME49_217900	p.Cys66Tyr	missense_variant	hypothetical protein
TGME49_217915	p.Asp26Glu	missense_variant	hypothetical protein

TGME49_217915	p.Trp96Ser	missense_variant	hypothetical protein
TGME49_217915	p.Arg160Trp	missense_variant	hypothetical protein
TGME49_217915	p.Lys216Thr	missense_variant	hypothetical protein
TGME49_217940	p.Thr265Ala	missense_variant	hypothetical protein
TGME49_217940	p.Lys967Asn	missense_variant&splice_region_variant	hypothetical protein
TGME49_245428	p.Leu165Val	missense_variant	hypothetical protein
TGME49_245428	p.Tyr103His	missense_variant	hypothetical protein
TGME49_245432	p.Pro175Ser	missense_variant	hypothetical protein
TGME49_245440	p.Asp59His	missense_variant	hypothetical protein
TGME49_245530	p.Pro608Leu	missense_variant	hypothetical protein
TGME49_245530	p.Pro644Leu	missense_variant	hypothetical protein
TGME49_245530	p.Thr681Ala	missense_variant	hypothetical protein
TGME49_245530	p.Arg1536Lys	missense_variant	hypothetical protein
TGME49_245530	p.Thr1712Ser	missense_variant	hypothetical protein
TGME49_245530	p.Ile2108Val	missense_variant	hypothetical protein
TGME49_245560	c.201-8C>A	splice_region_variant	hypothetical protein
TGME49_245560	p.Thr614Pro	missense_variant	hypothetical protein
TGME49_245560	p.Arg629Gly	missense_variant	hypothetical protein
TGME49_245560	p.Ala895Thr	missense_variant	hypothetical protein
TGME49_245560	p.Thr1506Ile	missense_variant	hypothetical protein
TGME49_245560	p.Ser1582Asn	missense_variant	hypothetical protein
TGME49_245560	p.Ala1675Glu	missense_variant	hypothetical protein
TGME49_245560	p.Gly1726Ser	missense_variant	hypothetical protein
TGME49_245560	p.Phe1905Leu	missense_variant	hypothetical protein
TGME49_245580	p.Ala15Thr	missense_variant	hypothetical protein
TGME49_245580	p.Val18Ile	missense_variant	hypothetical protein
TGME49_245580	p.Pro95Ser	missense_variant	hypothetical protein
TGME49_245580	p.Val104Leu	missense_variant	hypothetical protein
TGME49_245580	p.Ala131Ser	missense_variant	hypothetical protein
TGME49_245580	p.Pro861Leu	missense_variant	hypothetical protein
TGME49_245580	p.Cys1005Tyr	missense_variant	hypothetical protein
TGME49_245580	p.Gln1017Arg	missense_variant	hypothetical protein
TGME49_245600	p.Leu69His	missense_variant	hypothetical protein
TGME49_245600	p.Gly49Ala	missense_variant	hypothetical protein
TGME49_245610	c.1492-8A>G	splice_region_variant	hypothetical protein
TGME49_245610	c.1492-4C>T	splice_region_variant	hypothetical protein
TGME49_245640	p.Met123Leu	missense_variant	hypothetical protein
TGME49_245640	p.Gly77Asp	missense_variant	hypothetical protein
TGME49_245640	p.Val18Leu	missense_variant	hypothetical protein
TGME49_245640	p.Ile6Val	missense_variant	hypothetical protein
TGME49_245640	c.-99C>T	5_prime_UTR_premature_start_codon_gain_variant	hypothetical protein
TGME49_245660	p.Ser240Cys	missense_variant	hypothetical protein
TGME49_245660	c.-39A>T	5_prime_UTR_premature_start_codon_gain_variant	hypothetical protein
TGME49_245746	c.1536+4C>A	splice_region_variant	hypothetical protein
TGME49_245746	c.1536+3A>G	splice_region_variant	hypothetical protein
TGME49_245746	p.Ser85Asn	missense_variant	hypothetical protein
TGME49_245746	p.Pro83Ser	missense_variant	hypothetical protein
TGME49_245746	p.Leu20Val	missense_variant	hypothetical protein
TGME49_245748	c.-2057C>G	5_prime_UTR_premature_start_codon_gain_variant	hypothetical protein
TGME49_245748	c.-1328A>C	5_prime_UTR_premature_start_codon_gain_variant	hypothetical protein
TGME49_245748	p.Val201Ile	missense_variant	hypothetical protein
TGME49_245748	p.Pro204Thr	missense_variant	hypothetical protein
TGME49_245990	p.Gly232Glu	missense_variant	hypothetical protein
TGME49_245990	p.Ala9Val	missense_variant	hypothetical protein
TGME49_245995	p.Ser78*	stop_gained	hypothetical protein
TGME49_246050	p.Pro713Ser	missense_variant	hypothetical protein
TGME49_246050	p.Ser553Pro	missense_variant	hypothetical protein
TGME49_246050	p.Ser552Pro	missense_variant	hypothetical protein
TGME49_246050	p.Ser139Phe	missense_variant	hypothetical protein
TGME49_246090	p.Ala884Val	missense_variant	hypothetical protein
TGME49_246140	p.Leu992Gln	missense_variant	hypothetical protein
TGME49_246140	p.Thr513Ile	missense_variant	hypothetical protein
TGME49_246140	p.Gly451Arg	missense_variant	hypothetical protein
TGME49_246140	p.Ala341Val	missense_variant	hypothetical protein
TGME49_246140	p.Arg143Thr	missense_variant	hypothetical protein
TGME49_246160	p.Ala50Ser	missense_variant	hypothetical protein
TGME49_246160	p.Val165Ile	missense_variant	hypothetical protein
TGME49_246160	p.Gly236Arg	missense_variant	hypothetical protein
TGME49_246160	p.Leu256Met	missense_variant	hypothetical protein
TGME49_246160	p.Val905Met	missense_variant	hypothetical protein
TGME49_246190	p.Asp378His	missense_variant	hypothetical protein
TGME49_246190	p.Glu402Ala	missense_variant	hypothetical protein
TGME49_246190	p.Ala417Asp	missense_variant	hypothetical protein
TGME49_246190	p.Leu425His	missense_variant	hypothetical protein
TGME49_246190	p.Val433Met	missense_variant	hypothetical protein
TGME49_246190	p.Ala581Val	missense_variant	hypothetical protein
TGME49_246190	p.Ser646Thr	missense_variant	hypothetical protein
TGME49_246190	p.Val919Gly	missense_variant	hypothetical protein
TGME49_246190	p.Gly1091Glu	missense_variant	hypothetical protein
TGME49_246190	p.Ser1187Thr	missense_variant	hypothetical protein
TGME49_246190	p.Lys1757Asn	missense_variant	hypothetical protein
TGME49_246190	p.Ser1996Arg	missense_variant	hypothetical protein
TGME49_246190	p.Ala2010Glu	missense_variant	hypothetical protein
TGME49_246190	p.Pro2163Ser	missense_variant	hypothetical protein
TGME49_246190	p.Gly2260Ser	missense_variant	hypothetical protein

TGME49_246190	p.Lys2537Arg	missense_variant	hypothetical protein
TGME49_246190	p.Val2603Ala	missense_variant	hypothetical protein
TGME49_246190	p.Pro2703Ala	missense_variant	hypothetical protein
TGME49_246190	p.Glu3135Lys	missense_variant	hypothetical protein
TGME49_246190	p.Arg3447Pro	missense_variant	hypothetical protein
TGME49_246190	p.Ala3694Val	missense_variant	hypothetical protein
TGME49_246190	p.Met3797Ile	missense_variant	hypothetical protein
TGME49_246220	p.Gln634Lys	missense_variant	hypothetical protein
TGME49_246220	p.Leu244Phe	missense_variant	hypothetical protein
TGME49_246220	p.Leu118Val	missense_variant	hypothetical protein
TGME49_246450	c.94+7A>C	splice_region_variant	hypothetical protein
TGME49_246460	p.Ser79Phe	missense_variant	hypothetical protein
TGME49_246475	c.1708-7A>G	splice_region_variant	hypothetical protein
TGME49_246475	p.Gly404Arg	missense_variant	hypothetical protein
TGME49_246475	p.Val285Phe	missense_variant	hypothetical protein
TGME49_246475	p.Leu249Phe	missense_variant	hypothetical protein
TGME49_246475	p.Ser135Phe	missense_variant	hypothetical protein
TGME49_246475	p.Cys23Tyr	missense_variant	hypothetical protein
TGME49_246490	p.Ala234Val	missense_variant	hypothetical protein
TGME49_246520	p.Ala604Thr	missense_variant	hypothetical protein
TGME49_246520	p.Pro275Ala	missense_variant	hypothetical protein
TGME49_246535	p.Glu107Ala	missense_variant	hypothetical protein
TGME49_246535	c.-16C>T	5_prime_UTR_premature_start_codon_gain_variant	hypothetical protein
TGME49_246555	p.Leu102Pro	missense_variant	hypothetical protein
TGME49_246570	p.Glu11Asp	missense_variant	hypothetical protein
TGME49_246570	c.-321T>G	5_prime_UTR_premature_start_codon_gain_variant	hypothetical protein
TGME49_246570	c.-344C>T	5_prime_UTR_premature_start_codon_gain_variant	hypothetical protein
TGME49_246590	p.Val195Ile	missense_variant	hypothetical protein
TGME49_246590	p.His292Arg	missense_variant	hypothetical protein
TGME49_246610	p.Val534Ile	missense_variant	hypothetical protein
TGME49_246610	p.Asp371Asn	missense_variant	hypothetical protein
TGME49_246610	p.Gly249Glu	missense_variant	hypothetical protein
TGME49_246610	p.Gly175Glu	missense_variant	hypothetical protein
TGME49_246610	p.Glu71Ala	missense_variant	hypothetical protein
TGME49_246610	p.Arg9Leu	missense_variant	hypothetical protein
TGME49_246635	p.Ala83Val	missense_variant	hypothetical protein
TGME49_246635	p.Gly104Ala	missense_variant&splice_region_variant	hypothetical protein
TGME49_246670	c.-668A>G	5_prime_UTR_premature_start_codon_gain_variant	hypothetical protein
TGME49_246670	c.232+6A>G	splice_region_variant	hypothetical protein
TGME49_246710	p.Pro292Ala	missense_variant	hypothetical protein
TGME49_246720	c.-852A>T	5_prime_UTR_premature_start_codon_gain_variant	hypothetical protein
TGME49_246720	p.Ser218Arg	missense_variant	hypothetical protein
TGME49_246740	c.-738C>T	5_prime_UTR_premature_start_codon_gain_variant	hypothetical protein
TGME49_246740	p.Thr177Ala	missense_variant	hypothetical protein
TGME49_246750	p.Thr218Met	missense_variant	hypothetical protein
TGME49_246750	p.Ala101Pro	missense_variant	hypothetical protein
TGME49_246750	c.-95C>T	5_prime_UTR_premature_start_codon_gain_variant	hypothetical protein
TGME49_246750	c.-214C>T	5_prime_UTR_premature_start_codon_gain_variant	hypothetical protein
TGME49_246750	c.-752C>T	5_prime_UTR_premature_start_codon_gain_variant	hypothetical protein
TGME49_246760	p.Glu981Gly	missense_variant	hypothetical protein
TGME49_246760	p.Cys1001Arg	missense_variant	hypothetical protein
TGME49_246760	p.His1107Asp	missense_variant	hypothetical protein
TGME49_246760	p.Arg1259Thr	missense_variant	hypothetical protein
TGME49_246760	p.Ser1383Asn	missense_variant	hypothetical protein
TGME49_246760	p.Arg1645Gly	missense_variant	hypothetical protein
TGME49_246760	p.Asn1728Asp	missense_variant	hypothetical protein
TGME49_246760	p.Asp1913Gly	missense_variant	hypothetical protein
TGME49_246760	p.Phe2474Ser	missense_variant	hypothetical protein
TGME49_246770	c.-786C>T	5_prime_UTR_premature_start_codon_gain_variant	hypothetical protein
TGME49_246780	p.Asp540Asn	missense_variant	hypothetical protein
TGME49_246780	p.Glu505Gln	missense_variant	hypothetical protein
TGME49_246780	p.Ala383Val	missense_variant	hypothetical protein
TGME49_246780	p.Arg162Cys	missense_variant	hypothetical protein
TGME49_246780	c.-459C>T	5_prime_UTR_premature_start_codon_gain_variant	hypothetical protein
TGME49_246780	c.-860C>A	5_prime_UTR_premature_start_codon_gain_variant	hypothetical protein
TGME49_246950	p.Ser341Thr	missense_variant	hypothetical protein
TGME49_246960	p.Gln15His	missense_variant	hypothetical protein
TGME49_246960	p.Pro188Ser	missense_variant	hypothetical protein
TGME49_246960	p.Cys210Gly	missense_variant	hypothetical protein
TGME49_246978	p.Val1152Leu	missense_variant	hypothetical protein
TGME49_246978	p.Leu362Val	missense_variant	hypothetical protein
TGME49_246978	c.-702C>T	5_prime_UTR_premature_start_codon_gain_variant	hypothetical protein
TGME49_246982	p.Arg978Trp	missense_variant	hypothetical protein
TGME49_246982	p.Pro779His	missense_variant	hypothetical protein
TGME49_246982	c.-228A>G	5_prime_UTR_premature_start_codon_gain_variant	hypothetical protein
TGME49_246982	c.-381T>C	5_prime_UTR_premature_start_codon_gain_variant	hypothetical protein
TGME49_246990	p.Thr102Ser	missense_variant	hypothetical protein
TGME49_246995	p.Asp1295Glu	missense_variant	hypothetical protein
TGME49_246995	p.His1064Pro	missense_variant	hypothetical protein
TGME49_246995	p.Gln294His	missense_variant	hypothetical protein
TGME49_247010	p.Ala197Asp	missense_variant	hypothetical protein
TGME49_247015	p.Arg223Gly	missense_variant	hypothetical protein
TGME49_247015	c.-570C>A	5_prime_UTR_premature_start_codon_gain_variant	hypothetical protein
TGME49_247015	c.-720T>G	5_prime_UTR_premature_start_codon_gain_variant	hypothetical protein
TGME49_247025	p.Gly176Glu	missense_variant	hypothetical protein

TGME49_247040	p.Cys602Gly	missense_variant	hypothetical protein
TGME49_247040	p.Gly497Ala	missense_variant	hypothetical protein
TGME49_247040	p.Arg264Gln	missense_variant	hypothetical protein
TGME49_247040	p.Asp245Gly	missense_variant	hypothetical protein
TGME49_247040	p.Ser176Gly	missense_variant	hypothetical protein
TGME49_247040	c.-143C>T	5_prime_UTR_premature_start_codon_gain_variant	hypothetical protein
TGME49_247040	c.-184C>T	5_prime_UTR_premature_start_codon_gain_variant	hypothetical protein
TGME49_247040	c.-543G>T	5_prime_UTR_premature_start_codon_gain_variant	hypothetical protein
TGME49_247050	p.Thr143Ile	missense_variant	hypothetical protein
TGME49_247050	p.Lys417Asn	missense_variant	hypothetical protein
TGME49_247050	p.Ser457Phe	missense_variant	hypothetical protein
TGME49_247050	p.Gly961Glu	missense_variant	hypothetical protein
TGME49_247050	p.Glu1712Asp	missense_variant	hypothetical protein
TGME49_247050	p.Ala1857Thr	missense_variant	hypothetical protein
TGME49_247050	p.Ala1912Thr	missense_variant	hypothetical protein
TGME49_247050	p.Gly2526Arg	missense_variant	hypothetical protein
TGME49_247260	p.Val154Gly	missense_variant	hypothetical protein
TGME49_247260	p.Arg3Leu	missense_variant	hypothetical protein
TGME49_247260	c.-615A>G	5_prime_UTR_premature_start_codon_gain_variant	hypothetical protein
TGME49_247270	p.Ala3Thr	missense_variant	hypothetical protein
TGME49_247270	p.Arg7His	missense_variant	hypothetical protein
TGME49_247270	p.Gly243Arg	missense_variant	hypothetical protein
TGME49_247270	c.750-6G>A	splice_region_variant	hypothetical protein
TGME49_247270	p.Gly469Val	missense_variant	hypothetical protein
TGME49_247270	p.Lys568Asn	missense_variant	hypothetical protein
TGME49_247270	p.Ala572Val	missense_variant	hypothetical protein
TGME49_247270	p.Ser769Ala	missense_variant	hypothetical protein
TGME49_247270	p.Arg914Lys	missense_variant	hypothetical protein
TGME49_247270	p.Asn1031His	missense_variant	hypothetical protein
TGME49_247270	p.Glu1120Lys	missense_variant	hypothetical protein
TGME49_247270	p.Gly1240Ala	missense_variant	hypothetical protein
TGME49_247280	p.Arg2383Thr	missense_variant	hypothetical protein
TGME49_247280	p.Asp2337Gly	missense_variant	hypothetical protein
TGME49_247280	p.Asp2337Asn	missense_variant	hypothetical protein
TGME49_247280	p.Leu2275Ser	missense_variant	hypothetical protein
TGME49_247280	p.Gln2256Pro	missense_variant	hypothetical protein
TGME49_247280	p.Phe2251Leu	missense_variant	hypothetical protein
TGME49_247280	p.Leu2205Val	missense_variant	hypothetical protein
TGME49_247280	p.Ala2069Val	missense_variant	hypothetical protein
TGME49_247280	p.Ala2069Thr	missense_variant	hypothetical protein
TGME49_247280	p.Arg1834Gly	missense_variant	hypothetical protein
TGME49_247280	p.Asp1801Ala	missense_variant	hypothetical protein
TGME49_247280	p.Pro1769Leu	missense_variant	hypothetical protein
TGME49_247280	p.Val1764Ala	missense_variant	hypothetical protein
TGME49_247280	p.Leu1744Ser	missense_variant	hypothetical protein
TGME49_247280	p.His1727Leu	missense_variant	hypothetical protein
TGME49_247280	p.Glu1663Gln	missense_variant	hypothetical protein
TGME49_247280	p.His1618Gln	missense_variant	hypothetical protein
TGME49_247280	p.His1618Arg	missense_variant	hypothetical protein
TGME49_247280	p.Glu951Gln	missense_variant	hypothetical protein
TGME49_247280	p.Ala901Val	missense_variant	hypothetical protein
TGME49_247280	p.Ala517Glu	missense_variant	hypothetical protein
TGME49_247280	p.Val462Leu	missense_variant	hypothetical protein
TGME49_247280	p.Glu355Gly	missense_variant	hypothetical protein
TGME49_247280	p.Glu340Asp	missense_variant	hypothetical protein
TGME49_247280	p.Ala209Asp	missense_variant	hypothetical protein
TGME49_247280	p.Glu35Asp	missense_variant	hypothetical protein
TGME49_247280	c.-480C>T	5_prime_UTR_premature_start_codon_gain_variant	hypothetical protein
TGME49_247290	p.Lys9Thr	missense_variant	hypothetical protein
TGME49_247290	p.Ser54Trp	missense_variant	hypothetical protein
TGME49_247290	p.Pro283Leu	missense_variant	hypothetical protein
TGME49_247290	p.Pro293Ala	missense_variant	hypothetical protein
TGME49_247290	p.Thr302Ala	missense_variant	hypothetical protein
TGME49_247290	p.Arg537Cys	missense_variant	hypothetical protein
TGME49_247290	p.Arg634His	missense_variant	hypothetical protein
TGME49_247290	p.Arg640Ser	missense_variant	hypothetical protein
TGME49_247290	p.Glu851Ala	missense_variant	hypothetical protein
TGME49_247290	p.Arg927Lys	missense_variant	hypothetical protein
TGME49_247290	p.Tyr1083Cys	missense_variant	hypothetical protein
TGME49_247290	p.Leu1320His	missense_variant	hypothetical protein
TGME49_247290	p.Pro1379Leu	missense_variant	hypothetical protein
TGME49_247290	p.Val1602Gly	missense_variant	hypothetical protein
TGME49_247290	p.Val1694Leu	missense_variant	hypothetical protein
TGME49_247290	p.Ala1723Thr	missense_variant	hypothetical protein
TGME49_247290	p.Val1743Ala	missense_variant	hypothetical protein
TGME49_247300	p.Val703Ile	missense_variant	hypothetical protein
TGME49_247300	p.Ala474Glu	missense_variant	hypothetical protein
TGME49_247300	p.Arg48Cys	missense_variant	hypothetical protein
TGME49_247330	p.Ala1410Val	missense_variant	hypothetical protein
TGME49_247330	p.Pro1109Arg	missense_variant	hypothetical protein
TGME49_247330	p.Gln1096Pro	missense_variant	hypothetical protein
TGME49_247330	c.3113+8C>A	splice_region_variant	hypothetical protein
TGME49_247330	c.2951-4G>A	splice_region_variant	hypothetical protein
TGME49_247330	p.Asn818Thr	missense_variant	hypothetical protein
TGME49_247330	p.Leu560Trp	missense_variant	hypothetical protein

TGME49_247340	c.-35C>T	5_prime_UTR_premature_start_codon_gain_variant	hypothetical protein
TGME49_247370	c.175+7C>T	splice_region_variant	hypothetical protein
TGME49_247370	c.1640-5T>C	splice_region_variant	hypothetical protein
TGME49_247380	p.Pro338His	missense_variant	hypothetical protein
TGME49_247380	p.Thr299Pro	missense_variant	hypothetical protein
TGME49_247380	p.Ser139Thr	missense_variant	hypothetical protein
TGME49_247380	p.Ser106Ala	missense_variant	hypothetical protein
TGME49_247380	p.Arg61Leu	missense_variant	hypothetical protein
TGME49_247380	p.Gly36Glu	missense_variant	hypothetical protein
TGME49_247400	c.-565C>G	5_prime_UTR_premature_start_codon_gain_variant	hypothetical protein
TGME49_247400	p.Ile25Thr	missense_variant	hypothetical protein
TGME49_247400	p.Ala98Ser	missense_variant	hypothetical protein
TGME49_247400	p.Asn159Ser	missense_variant	hypothetical protein
TGME49_247400	p.Gly224Ser	missense_variant	hypothetical protein
TGME49_247400	p.Asp275Asn	missense_variant	hypothetical protein
TGME49_247410	c.-441A>G	5_prime_UTR_premature_start_codon_gain_variant	hypothetical protein
TGME49_247420	c.-830A>G	5_prime_UTR_premature_start_codon_gain_variant	hypothetical protein
TGME49_247420	p.Thr68Ala	missense_variant	hypothetical protein
TGME49_247440	p.Pro164Leu	missense_variant	hypothetical protein
TGME49_247440	p.Gly74Asp	missense_variant	hypothetical protein
TGME49_247440	p.Val13Leu	missense_variant	hypothetical protein
TGME49_247450	p.Ala648Pro	missense_variant	hypothetical protein
TGME49_247450	p.Glu1425Asp	missense_variant	hypothetical protein
TGME49_247520	p.Pro224Ser	missense_variant	hypothetical protein
TGME49_247520	p.Pro223Leu	missense_variant	hypothetical protein
TGME49_247520	p.Leu128Val	missense_variant	hypothetical protein
TGME49_247570	p.Pro240Leu	missense_variant	hypothetical protein
TGME49_247600	p.Ser274Trp	missense_variant	hypothetical protein
TGME49_247640	p.Met302Ile	missense_variant	hypothetical protein
TGME49_247640	p.Leu184Pro	missense_variant	hypothetical protein
TGME49_247640	c.-743C>T	5_prime_UTR_premature_start_codon_gain_variant	hypothetical protein
TGME49_247640	c.-1174A>G	5_prime_UTR_premature_start_codon_gain_variant	hypothetical protein
TGME49_247652	p.Ala333Thr	missense_variant	hypothetical protein
TGME49_247652	p.Ala369Thr	missense_variant	hypothetical protein
TGME49_247680	c.*353T>C	splice_region_variant	hypothetical protein
TGME49_247680	p.Val1671Leu	missense_variant	hypothetical protein
TGME49_247680	c.4512+4A>C	splice_region_variant	hypothetical protein
TGME49_247680	p.Leu1235Ile	missense_variant	hypothetical protein
TGME49_247680	p.Tyr584Ser	missense_variant	hypothetical protein
TGME49_247680	p.Pro307Ser	missense_variant	hypothetical protein
TGME49_247680	p.His269Arg	missense_variant	hypothetical protein
TGME49_247680	p.His81Asn	missense_variant	hypothetical protein
TGME49_247720	p.Ser24Pro	missense_variant	hypothetical protein
TGME49_247720	p.Gln3His	missense_variant	hypothetical protein
TGME49_247740	c.-484T>C	5_prime_UTR_premature_start_codon_gain_variant	hypothetical protein
TGME49_247740	c.-282T>G	5_prime_UTR_premature_start_codon_gain_variant	hypothetical protein
TGME49_247740	p.Phe46Ile	missense_variant	hypothetical protein
TGME49_247740	p.Arg140His	missense_variant	hypothetical protein
TGME49_247740	p.Pro359Ser	missense_variant	hypothetical protein
TGME49_247740	c.1598+4C>A	splice_region_variant	hypothetical protein
TGME49_247740	p.Gln674Glu	missense_variant	hypothetical protein
TGME49_247740	p.Thr816Ala	missense_variant	hypothetical protein
TGME49_247750	p.Gln169Arg	missense_variant	hypothetical protein
TGME49_247850	p.Ser18Phe	missense_variant	hypothetical protein
TGME49_247940	p.Pro101Leu	missense_variant	hypothetical protein
TGME49_247940	p.Leu47Met	missense_variant	hypothetical protein
TGME49_247940	p.Val16Ile	missense_variant	hypothetical protein
TGME49_247940	p.Gln10Arg	missense_variant	hypothetical protein
TGME49_247940	c.-33C>T	5_prime_UTR_premature_start_codon_gain_variant	hypothetical protein
TGME49_247940	c.-44C>G	5_prime_UTR_premature_start_codon_gain_variant	hypothetical protein
TGME49_247960	p.Asp294Gly	missense_variant	hypothetical protein
TGME49_247960	p.Asp281Glu	missense_variant	hypothetical protein
TGME49_247960	p.Glu271Gln	missense_variant	hypothetical protein
TGME49_247970	p.His720Arg	missense_variant	hypothetical protein
TGME49_247970	p.Glu801Lys	missense_variant	hypothetical protein
TGME49_247970	p.Val1022Ala	missense_variant	hypothetical protein
TGME49_247970	p.Thr1087Ala	missense_variant	hypothetical protein
TGME49_247970	p.Thr1189Ala	missense_variant	hypothetical protein
TGME49_247970	p.Gln1205*	stop_gained	hypothetical protein
TGME49_248110	c.-807A>G	5_prime_UTR_premature_start_codon_gain_variant	hypothetical protein
TGME49_248110	c.-2657A>T	5_prime_UTR_premature_start_codon_gain_variant	hypothetical protein
TGME49_248120	p.Thr872Pro	missense_variant	hypothetical protein
TGME49_248120	p.Pro841Gln	missense_variant	hypothetical protein
TGME49_248120	p.Ala416Pro	missense_variant	hypothetical protein
TGME49_248120	p.Ala220Gly	missense_variant	hypothetical protein
TGME49_248120	c.-636C>T	5_prime_UTR_premature_start_codon_gain_variant	hypothetical protein
TGME49_248130	c.-383A>G	5_prime_UTR_premature_start_codon_gain_variant	hypothetical protein
TGME49_248130	p.Pro517Leu	missense_variant	hypothetical protein
TGME49_248130	p.Arg828Lys	missense_variant	hypothetical protein
TGME49_248130	p.Lys831Arg	missense_variant	hypothetical protein
TGME49_248140	p.Asp263Gly	missense_variant	hypothetical protein
TGME49_248140	p.Thr178Ala	missense_variant	hypothetical protein
TGME49_248140	p.Val169Met	missense_variant	hypothetical protein
TGME49_248140	p.Pro99Ala	missense_variant	hypothetical protein
TGME49_248150	p.Arg1961Lys	missense_variant	hypothetical protein

TGME49_248150	p.Gln1903His	missense_variant	hypothetical protein
TGME49_248150	c.5307+4A>C	splice_region_variant	hypothetical protein
TGME49_248150	p.Leu1603Val	missense_variant	hypothetical protein
TGME49_248150	p.Thr1536Ala	missense_variant	hypothetical protein
TGME49_248150	p.Ala1052Thr	missense_variant	hypothetical protein
TGME49_248150	p.Glu466Asp	missense_variant	hypothetical protein
TGME49_248150	p.Met426Thr	missense_variant	hypothetical protein
TGME49_248160	p.Ser195Tyr	missense_variant	hypothetical protein
TGME49_248160	p.Ala346Pro	missense_variant	hypothetical protein
TGME49_248160	c.1063-4T>G	splice_region_variant	hypothetical protein
TGME49_248160	p.Thr362Ala	missense_variant	hypothetical protein
TGME49_248160	c.2400+7C>T	splice_region_variant	hypothetical protein
TGME49_248170	p.Ala810Asp	missense_variant	hypothetical protein
TGME49_248170	p.Lys594Glu	missense_variant	hypothetical protein
TGME49_248170	p.Ala359Pro	missense_variant	hypothetical protein
TGME49_248170	p.Lys229Glu	missense_variant	hypothetical protein
TGME49_248170	p.Thr226Pro	missense_variant	hypothetical protein
TGME49_248170	c.-837C>G	5_prime_UTR_premature_start_codon_gain_variant	hypothetical protein
TGME49_248170	c.-2019A>G	5_prime_UTR_premature_start_codon_gain_variant	hypothetical protein
TGME49_248190	p.Ala87Val	missense_variant	hypothetical protein
TGME49_248190	p.Glu272Asp	missense_variant	hypothetical protein
TGME49_248190	p.Arg298Leu	missense_variant	hypothetical protein
TGME49_248190	p.Arg302Leu	missense_variant	hypothetical protein
TGME49_248190	p.Ala519Thr	missense_variant	hypothetical protein
TGME49_248190	p.Glu587Asp	missense_variant	hypothetical protein
TGME49_248190	p.Gln597Pro	missense_variant	hypothetical protein
TGME49_248190	p.Asp673Gly	missense_variant	hypothetical protein
TGME49_248190	c.2858+1G>A	splice_donor_variant	hypothetical protein
TGME49_248210	p.Val53Phe	missense_variant	hypothetical protein
TGME49_248280	p.Ser10Tyr	missense_variant	hypothetical protein
TGME49_248280	p.Gly19Arg	missense_variant	hypothetical protein
TGME49_248280	p.Gln98His	missense_variant	hypothetical protein
TGME49_248300	p.Ala237Thr	missense_variant	hypothetical protein
TGME49_248300	p.Pro213Arg	missense_variant	hypothetical protein
TGME49_248300	p.Leu140Pro	missense_variant	hypothetical protein
TGME49_248350	p.Lys132Gln	missense_variant	hypothetical protein
TGME49_248350	p.Ala148Val	missense_variant	hypothetical protein
TGME49_248350	p.Thr182Pro	missense_variant	hypothetical protein
TGME49_248360	c.*1772C>G	splice_region_variant	hypothetical protein
TGME49_248360	c.358-4G>A	splice_region_variant	hypothetical protein
TGME49_248360	p.His101Pro	missense_variant	hypothetical protein
TGME49_248420	c.-929T>G	5_prime_UTR_premature_start_codon_gain_variant	hypothetical protein
TGME49_248425	p.Arg36Gln	missense_variant	hypothetical protein
TGME49_248425	p.Ala52Gly	missense_variant&splice_region_variant	hypothetical protein
TGME49_248425	p.Arg73His	missense_variant	hypothetical protein
TGME49_248440	p.Gln452His	missense_variant	hypothetical protein
TGME49_248440	p.Ala456Thr	missense_variant	hypothetical protein
TGME49_248440	p.Glu764Lys	missense_variant	hypothetical protein
TGME49_248490	c.-355T>C	5_prime_UTR_premature_start_codon_gain_variant	hypothetical protein
TGME49_248490	c.-712C>T	5_prime_UTR_premature_start_codon_gain_variant	hypothetical protein
TGME49_248500	c.-543C>G	5_prime_UTR_premature_start_codon_gain_variant	hypothetical protein
TGME49_248500	c.-215T>G	5_prime_UTR_premature_start_codon_gain_variant	hypothetical protein
TGME49_248510	p.Ser11758Leu	missense_variant	hypothetical protein
TGME49_248510	p.Glu11596Lys	missense_variant	hypothetical protein
TGME49_248510	p.Thr11325Lys	missense_variant	hypothetical protein
TGME49_248510	p.Leu10927Phe	missense_variant	hypothetical protein
TGME49_248510	p.Asp10755Asn	missense_variant	hypothetical protein
TGME49_248510	p.Arg10609Cys	missense_variant	hypothetical protein
TGME49_248510	p.Gly10435Val	missense_variant	hypothetical protein
TGME49_248510	p.Arg9646Cys	missense_variant	hypothetical protein
TGME49_248510	p.Glu9571Asp	missense_variant	hypothetical protein
TGME49_248510	p.Phe9521Leu	missense_variant	hypothetical protein
TGME49_248510	p.Gln9316Arg	missense_variant	hypothetical protein
TGME49_248510	p.Asn9296Ser	missense_variant	hypothetical protein
TGME49_248510	p.Gln9245His	missense_variant	hypothetical protein
TGME49_248510	p.Val9105Leu	missense_variant	hypothetical protein
TGME49_248510	p.Leu8973Val	missense_variant	hypothetical protein
TGME49_248510	p.Gly8823Cys	missense_variant	hypothetical protein
TGME49_248510	p.Lys8311Glu	missense_variant	hypothetical protein
TGME49_248510	p.Leu8144Phe	missense_variant	hypothetical protein
TGME49_248510	p.Ser7704Asn	missense_variant	hypothetical protein
TGME49_248510	p.Pro7499Ser	missense_variant	hypothetical protein
TGME49_248510	p.Arg7464Pro	missense_variant	hypothetical protein
TGME49_248510	p.Asp7384Asn	missense_variant	hypothetical protein
TGME49_248510	p.Tyr7272His	missense_variant	hypothetical protein
TGME49_248510	c.21504+6T>C	splice_region_variant	hypothetical protein
TGME49_248510	p.Ser7004Arg	missense_variant	hypothetical protein
TGME49_248510	p.Ile6321Leu	missense_variant	hypothetical protein
TGME49_248510	p.His6007Tyr	missense_variant	hypothetical protein
TGME49_248510	p.Asp5997Glu	missense_variant	hypothetical protein
TGME49_248510	p.Leu5925Val	missense_variant	hypothetical protein
TGME49_248510	p.Pro5850Leu	missense_variant	hypothetical protein
TGME49_248510	p.Lys5837Gln	missense_variant	hypothetical protein
TGME49_248510	p.Phe5788Leu	missense_variant	hypothetical protein
TGME49_248510	p.Ser5752Asn	missense_variant	hypothetical protein

TGME49_248510	p.Arg5637Gly	missense_variant	hypothetical protein
TGME49_248510	p.Val5228Ala	missense_variant	hypothetical protein
TGME49_248510	p.Glu4814Asp	missense_variant	hypothetical protein
TGME49_248510	p.Thr4475Arg	missense_variant	hypothetical protein
TGME49_248510	p.Thr4475Ser	missense_variant	hypothetical protein
TGME49_248510	p.Thr4412Ser	missense_variant&splice_region_variant	hypothetical protein
TGME49_248510	p.Ser4384Arg	missense_variant	hypothetical protein
TGME49_248510	p.Asn4275Ser	missense_variant	hypothetical protein
TGME49_248510	p.Thr4040Arg	missense_variant	hypothetical protein
TGME49_248510	p.Glu3918Lys	missense_variant	hypothetical protein
TGME49_248510	p.Ser3705Pro	missense_variant	hypothetical protein
TGME49_248510	p.Ile3645Leu	missense_variant	hypothetical protein
TGME49_248510	p.Ser3630Thr	missense_variant	hypothetical protein
TGME49_248510	p.Arg3527Ser	missense_variant	hypothetical protein
TGME49_248510	p.Glu3515Asp	missense_variant	hypothetical protein
TGME49_248510	p.Asn3485Lys	missense_variant	hypothetical protein
TGME49_248510	p.Gln3484Pro	missense_variant	hypothetical protein
TGME49_248510	p.Gln3312His	missense_variant	hypothetical protein
TGME49_248510	p.Ala3280Gly	missense_variant	hypothetical protein
TGME49_248510	p.Arg3266Gly	missense_variant	hypothetical protein
TGME49_248510	p.Ile3173Leu	missense_variant	hypothetical protein
TGME49_248510	p.Cys3086Phe	missense_variant	hypothetical protein
TGME49_248510	p.Val2889Leu	missense_variant	hypothetical protein
TGME49_248510	p.Arg2839Lys	missense_variant	hypothetical protein
TGME49_248510	p.His2718Asp	missense_variant	hypothetical protein
TGME49_248510	p.Lys2634Thr	missense_variant	hypothetical protein
TGME49_248510	p.Asp2367Gly	missense_variant	hypothetical protein
TGME49_248510	p.Asp2333Gly	missense_variant	hypothetical protein
TGME49_248510	p.Gln2167His	missense_variant	hypothetical protein
TGME49_248510	p.Asp1993Gly	missense_variant	hypothetical protein
TGME49_248510	p.Ser1800Ala	missense_variant	hypothetical protein
TGME49_248510	p.Cys1747Trp	missense_variant	hypothetical protein
TGME49_248510	p.Thr1608Ile	missense_variant	hypothetical protein
TGME49_248510	p.Phe1472Ser	missense_variant	hypothetical protein
TGME49_248510	p.Thr1428Ala	missense_variant	hypothetical protein
TGME49_248510	p.Ser1213Phe	missense_variant	hypothetical protein
TGME49_248510	p.Gly1163Ser	missense_variant	hypothetical protein
TGME49_248510	p.Lys945Arg	missense_variant	hypothetical protein
TGME49_248510	p.Phe778Cys	missense_variant	hypothetical protein
TGME49_248510	p.Lys722Asn	missense_variant	hypothetical protein
TGME49_248510	p.Cys720Ser	missense_variant	hypothetical protein
TGME49_248510	p.Glu525Asp	missense_variant	hypothetical protein
TGME49_248520	p.Leu231Val	missense_variant	hypothetical protein
TGME49_248520	p.Gly207Arg	missense_variant	hypothetical protein
TGME49_248520	p.Gln200His	missense_variant	hypothetical protein
TGME49_248520	p.Pro68Ala	missense_variant	hypothetical protein
TGME49_248520	c.150+6G>A	splice_region_variant	hypothetical protein
TGME49_248540	p.Asn3619His	missense_variant	hypothetical protein
TGME49_248540	p.Ser3540Phe	missense_variant	hypothetical protein
TGME49_248540	p.Ser3316Arg	missense_variant	hypothetical protein
TGME49_248540	p.Val3286Gly	missense_variant	hypothetical protein
TGME49_248540	p.Gly3226Arg	missense_variant	hypothetical protein
TGME49_248540	p.Ser2896Phe	missense_variant	hypothetical protein
TGME49_248540	p.Asn2807Thr	missense_variant	hypothetical protein
TGME49_248540	p.Lys2788Glu	missense_variant	hypothetical protein
TGME49_248540	c.8069-5A>G	splice_region_variant	hypothetical protein
TGME49_248540	p.Glu2684Asp	missense_variant	hypothetical protein
TGME49_248540	p.Arg2319Cys	missense_variant	hypothetical protein
TGME49_248540	p.Trp2088Arg	missense_variant	hypothetical protein
TGME49_248540	p.Ser2064Arg	missense_variant	hypothetical protein
TGME49_248540	p.Asp2008Gly	missense_variant	hypothetical protein
TGME49_248540	p.Ser1998Gly	missense_variant	hypothetical protein
TGME49_248540	p.Asn1978Ser	missense_variant	hypothetical protein
TGME49_248540	p.Pro1818Leu	missense_variant	hypothetical protein
TGME49_248540	p.Ala1650Ser	missense_variant	hypothetical protein
TGME49_248540	p.Leu1484Gln	missense_variant	hypothetical protein
TGME49_248540	p.Glu1475Lys	missense_variant	hypothetical protein
TGME49_248540	p.Ser1274Cys	missense_variant	hypothetical protein
TGME49_248540	p.Leu1204Arg	missense_variant	hypothetical protein
TGME49_248540	p.Ser1181Asn	missense_variant	hypothetical protein
TGME49_248540	p.Val891Leu	missense_variant	hypothetical protein
TGME49_248540	p.Asp797Gly	missense_variant	hypothetical protein
TGME49_248540	p.Asp751Ala	missense_variant	hypothetical protein
TGME49_248540	p.Ser596Ala	missense_variant&splice_region_variant	hypothetical protein
TGME49_248540	p.Gly413Arg	missense_variant	hypothetical protein
TGME49_248540	p.Ala327Gly	missense_variant	hypothetical protein
TGME49_248540	p.Phe203Val	missense_variant	hypothetical protein
TGME49_248540	p.Phe197Val	missense_variant	hypothetical protein
TGME49_248560	p.Arg223Ser	missense_variant	hypothetical protein
TGME49_248560	p.Gly218Arg	missense_variant	hypothetical protein
TGME49_248560	p.Glu176Gln	missense_variant	hypothetical protein
TGME49_248560	p.Lys168Met	missense_variant	hypothetical protein
TGME49_248560	p.Gly144Arg	missense_variant	hypothetical protein
TGME49_248560	p.Ser56Phe	missense_variant	hypothetical protein
TGME49_248560	p.Cys36Tyr	missense_variant	hypothetical protein

TGME49_248570	p.Ala162Val	missense_variant	hypothetical protein
TGME49_248570	p.Glu298Lys	missense_variant	hypothetical protein
TGME49_248570	p.Arg310His	missense_variant	hypothetical protein
TGME49_248570	p.Thr311Ala	missense_variant	hypothetical protein
TGME49_248570	p.Thr312Ala	missense_variant	hypothetical protein
TGME49_248570	p.Arg637Gly	missense_variant	hypothetical protein
TGME49_248570	p.Gln747Glu	missense_variant	hypothetical protein
TGME49_248570	p.Ser750Thr	missense_variant	hypothetical protein
TGME49_248570	p.Ile785Val	missense_variant	hypothetical protein
TGME49_248570	p.Gly793Ala	missense_variant	hypothetical protein
TGME49_248570	p.Glu798Asp	missense_variant	hypothetical protein
TGME49_248570	p.Asp854Gly	missense_variant	hypothetical protein
TGME49_248570	p.Leu1246Phe	missense_variant	hypothetical protein
TGME49_248570	p.Leu1447Val	missense_variant	hypothetical protein
TGME49_248570	p.Ala1688Glu	missense_variant	hypothetical protein
TGME49_248590	p.Gln664Leu	missense_variant	hypothetical protein
TGME49_248610	p.Asp520Gly	missense_variant	hypothetical protein
TGME49_248620	p.Asn32His	missense_variant	hypothetical protein
TGME49_248620	c.-38G>T	5_prime_UTR_premature_start_codon_gain_variant	hypothetical protein
TGME49_248680	p.Asp1201Gly	missense_variant	hypothetical protein
TGME49_248680	p.Ser597Leu	missense_variant	hypothetical protein
TGME49_248680	c.-763C>T	5_prime_UTR_premature_start_codon_gain_variant	hypothetical protein
TGME49_248710	p.Leu170Gln	missense_variant	hypothetical protein
TGME49_248710	p.Ala639Thr	missense_variant	hypothetical protein
TGME49_248720	p.Thr1040Pro	missense_variant	hypothetical protein
TGME49_248720	p.Gly1259Glu	missense_variant	hypothetical protein
TGME49_248720	p.Glu1392Asp	missense_variant	hypothetical protein
TGME49_248720	p.Ser1499Arg	missense_variant	hypothetical protein
TGME49_248720	p.Gly1946Asp	missense_variant	hypothetical protein
TGME49_248720	p.Val1947Gly	missense_variant	hypothetical protein
TGME49_248740	p.Leu786Val	missense_variant	hypothetical protein
TGME49_248740	p.Glu324Ala	missense_variant	hypothetical protein
TGME49_248740	p.Gly322Glu	missense_variant	hypothetical protein
TGME49_248740	p.Val319Glu	missense_variant	hypothetical protein
TGME49_248740	p.Val279Leu	missense_variant	hypothetical protein
TGME49_248740	p.Asn193Ser	missense_variant	hypothetical protein
TGME49_248740	p.Ala24Val	missense_variant	hypothetical protein
TGME49_248740	p.Ala24Thr	missense_variant	hypothetical protein
TGME49_248740	c.-410T>G	5_prime_UTR_premature_start_codon_gain_variant	hypothetical protein
TGME49_248750	c.2380+7G>T	splice_region_variant	hypothetical protein
TGME49_248750	p.Met492Val	missense_variant	hypothetical protein
TGME49_248750	p.Leu386Phe	missense_variant	hypothetical protein
TGME49_248750	p.Val244Ile	missense_variant	hypothetical protein
TGME49_248750	p.Ala187Thr	missense_variant	hypothetical protein
TGME49_248750	p.Leu172Val	missense_variant	hypothetical protein
TGME49_248770	p.Arg281Leu	missense_variant	hypothetical protein
TGME49_248770	p.Ala32Glu	missense_variant	hypothetical protein
TGME49_248790	c.-534G>T	5_prime_UTR_premature_start_codon_gain_variant	hypothetical protein
TGME49_248790	c.-549T>C	5_prime_UTR_premature_start_codon_gain_variant	hypothetical protein
TGME49_248800	p.Gly77Asp	missense_variant	hypothetical protein
TGME49_248800	p.Asn91Lys	missense_variant	hypothetical protein
TGME49_248800	p.Asp93Gly	missense_variant	hypothetical protein
TGME49_248860	p.Ser283Phe	missense_variant	hypothetical protein
TGME49_248860	p.Pro173Ala	missense_variant	hypothetical protein
TGME49_248860	p.Trp93Ser	missense_variant	hypothetical protein
TGME49_248860	p.Cys41Arg	missense_variant	hypothetical protein
TGME49_248860	p.Ile29Ser	missense_variant	hypothetical protein
TGME49_248900	p.Pro562Ser	missense_variant	hypothetical protein
TGME49_248900	p.His302Gln	missense_variant	hypothetical protein
TGME49_248900	p.Pro245His	missense_variant	hypothetical protein
TGME49_248900	p.Asp212Glu	missense_variant	hypothetical protein
TGME49_248940	p.Lys206Gln	missense_variant	hypothetical protein
TGME49_248990	p.Ser56Ala	missense_variant	hypothetical protein
TGME49_248990	p.Gln395Lys	missense_variant	hypothetical protein
TGME49_249010	p.Cys407Arg	missense_variant	hypothetical protein
TGME49_249010	c.-1290C>T	5_prime_UTR_premature_start_codon_gain_variant	hypothetical protein
TGME49_249040	p.Ser184Ala	missense_variant	hypothetical protein
TGME49_249215	p.Asp517Asn	missense_variant	hypothetical protein
TGME49_249215	p.Val1033Glu	missense_variant	hypothetical protein
TGME49_249215	p.Ala1210Pro	missense_variant	hypothetical protein
TGME49_249230	p.Ser820Thr	missense_variant	hypothetical protein
TGME49_249230	p.Ala331Thr	missense_variant	hypothetical protein
TGME49_249280	p.Cys130Arg	missense_variant	hypothetical protein
TGME49_249280	p.Glu113Asp	missense_variant	hypothetical protein
TGME49_249290	p.Glu447Asp	missense_variant	hypothetical protein
TGME49_249290	p.Glu46Asp	missense_variant	hypothetical protein
TGME49_249310	c.733-7C>T	splice_region_variant	hypothetical protein
TGME49_249340	p.Glu913Asp	missense_variant	hypothetical protein
TGME49_249340	p.Val777Leu	missense_variant	hypothetical protein
TGME49_249340	p.Ser672Cys	missense_variant	hypothetical protein
TGME49_249340	p.Val649Gly	missense_variant	hypothetical protein
TGME49_249340	p.Arg224Gly	missense_variant	hypothetical protein
TGME49_249340	p.Val81Glu	missense_variant	hypothetical protein
TGME49_249340	p.Val3Met	missense_variant	hypothetical protein
TGME49_249365	p.Phe3Val	missense_variant	hypothetical protein

TGME49_249400	p.Leu280Ser	missense_variant	hypothetical protein
TGME49_249410	p.Ser300Pro	missense_variant	hypothetical protein
TGME49_249425	p.Gly473Ala	missense_variant	hypothetical protein
TGME49_249425	p.Glu391Asp	missense_variant	hypothetical protein
TGME49_249425	p.Gln385Glu	missense_variant	hypothetical protein
TGME49_249425	p.Ala361Val	missense_variant	hypothetical protein
TGME49_249425	p.Val343Ile	missense_variant	hypothetical protein
TGME49_249425	c.604+2T>C	splice_donor_variant	hypothetical protein
TGME49_249425	p.Leu166Pro	missense_variant	hypothetical protein
TGME49_249425	p.Ser149Asn	missense_variant	hypothetical protein
TGME49_249425	p.Asp137Glu	missense_variant	hypothetical protein
TGME49_249425	p.Ser133Phe	missense_variant	hypothetical protein
TGME49_249425	p.Gln117*	stop_gained	hypothetical protein
TGME49_249440	p.Leu1331Phe	missense_variant	hypothetical protein
TGME49_249440	p.Ala1269Gly	missense_variant	hypothetical protein
TGME49_249440	p.Phe1202Leu	missense_variant	hypothetical protein
TGME49_249440	p.Pro1197Thr	missense_variant	hypothetical protein
TGME49_249440	p.Val860Ile	missense_variant	hypothetical protein
TGME49_249450	p.Thr695Met	missense_variant	hypothetical protein
TGME49_249450	p.Arg519Trp	missense_variant	hypothetical protein
TGME49_249450	p.Arg292Pro	missense_variant	hypothetical protein
TGME49_249450	p.Ala99Thr	missense_variant	hypothetical protein
TGME49_249450	p.Gly15Val	missense_variant	hypothetical protein
TGME49_249490	p.Pro1937Leu	missense_variant	hypothetical protein
TGME49_249490	p.Asp1544Glu	missense_variant	hypothetical protein
TGME49_249490	p.Ala1460Gly	missense_variant	hypothetical protein
TGME49_249490	p.Asp1432Gly	missense_variant	hypothetical protein
TGME49_249490	p.Ala1378Ser	missense_variant	hypothetical protein
TGME49_249490	p.Ala1371Val	missense_variant	hypothetical protein
TGME49_249490	p.Phe1250Val	missense_variant	hypothetical protein
TGME49_249490	p.Ser1226Tyr	missense_variant	hypothetical protein
TGME49_249490	p.Thr1133Ala	missense_variant	hypothetical protein
TGME49_249490	p.Val1026Leu	missense_variant	hypothetical protein
TGME49_249490	p.Pro975Ser	missense_variant	hypothetical protein
TGME49_249490	p.Ser969Arg	missense_variant	hypothetical protein
TGME49_249490	p.Ile804Ser	missense_variant	hypothetical protein
TGME49_249490	p.Ala787Thr	missense_variant	hypothetical protein
TGME49_249490	p.Asp708His	missense_variant	hypothetical protein
TGME49_249490	p.Ala661Glu	missense_variant	hypothetical protein
TGME49_249490	p.Gly510Ser	missense_variant	hypothetical protein
TGME49_249490	p.Lys325Arg	missense_variant	hypothetical protein
TGME49_249490	p.Pro296Leu	missense_variant	hypothetical protein
TGME49_249490	p.Arg254Ser	missense_variant	hypothetical protein
TGME49_249535	p.Pro74Ser	missense_variant	hypothetical protein
TGME49_249535	p.Lys83Glu	missense_variant&splice_region_variant	hypothetical protein
TGME49_249535	p.His85Asp	missense_variant	hypothetical protein
TGME49_249535	p.Ter92Glnext*?	stop_lost&splice_region_variant	hypothetical protein
TGME49_249540	p.Asn1477Thr	missense_variant	hypothetical protein
TGME49_249540	p.Leu1430Val	missense_variant	hypothetical protein
TGME49_249540	p.Gly1366Ala	missense_variant	hypothetical protein
TGME49_249540	p.Ala1246Val	missense_variant	hypothetical protein
TGME49_249540	p.Ala1163Ser	missense_variant	hypothetical protein
TGME49_249540	p.Gly1082Ser	missense_variant	hypothetical protein
TGME49_249540	p.Thr386Ser	missense_variant	hypothetical protein
TGME49_249540	p.Pro257His	missense_variant	hypothetical protein
TGME49_249540	p.Ala46Val	missense_variant	hypothetical protein
TGME49_249550	p.Val649Met	missense_variant	hypothetical protein
TGME49_249550	p.His709Pro	missense_variant	hypothetical protein
TGME49_249550	p.Gly1018Arg	missense_variant	hypothetical protein
TGME49_249550	p.Val1744Met	missense_variant	hypothetical protein
TGME49_249570	p.Leu134Phe	missense_variant	hypothetical protein
TGME49_249570	p.Ala63Thr	missense_variant	hypothetical protein
TGME49_249600	p.Val608Ala	missense_variant	hypothetical protein
TGME49_249600	p.Pro514Leu	missense_variant	hypothetical protein
TGME49_249610	p.Thr1164Ser	missense_variant	hypothetical protein
TGME49_249610	p.Gln1068His	missense_variant	hypothetical protein
TGME49_249610	c.796+4G>T	splice_region_variant	hypothetical protein
TGME49_249665	p.Arg119Gly	missense_variant	hypothetical protein
TGME49_249665	p.Arg61Thr	missense_variant	hypothetical protein
TGME49_249685	p.Pro216Leu	missense_variant	hypothetical protein
TGME49_249690	c.-484C>G	5_prime_UTR_premature_start_codon_gain_variant	hypothetical protein
TGME49_249690	p.Arg13Cys	missense_variant	hypothetical protein
TGME49_249725	c.-673T>G	5_prime_UTR_premature_start_codon_gain_variant	hypothetical protein
TGME49_249725	p.Lys87Glu	missense_variant	hypothetical protein
TGME49_249730	c.2362-3C>T	splice_region_variant	hypothetical protein
TGME49_249730	c.2362-4T>G	splice_region_variant	hypothetical protein
TGME49_249750	p.Lys14Thr	missense_variant	hypothetical protein
TGME49_249780	p.Glu121Asp	missense_variant	hypothetical protein
TGME49_249790	c.-506T>G	5_prime_UTR_premature_start_codon_gain_variant	hypothetical protein
TGME49_249800	p.Thr583Asn	missense_variant	hypothetical protein
TGME49_249800	p.Ala571Ser	missense_variant	hypothetical protein
TGME49_249800	p.Ser431Ala	missense_variant	hypothetical protein
TGME49_249800	p.Leu425Arg	missense_variant	hypothetical protein
TGME49_249800	p.Ala331Thr	missense_variant	hypothetical protein
TGME49_249800	p.Ser285Ala	missense_variant	hypothetical protein

TGME49_249800	p.Glu128Lys	missense_variant	hypothetical protein
TGME49_249800	p.Thr48Ile	missense_variant	hypothetical protein
TGME49_249860	p.Ala416Thr	missense_variant	hypothetical protein
TGME49_249860	p.Ala351Val	missense_variant	hypothetical protein
TGME49_249880	p.Arg84Gln	missense_variant	hypothetical protein
TGME49_249880	p.Ser601Phe	missense_variant	hypothetical protein
TGME49_249880	p.Trp632Cys	missense_variant	hypothetical protein
TGME49_249880	p.Ser770Pro	missense_variant	hypothetical protein
TGME49_249880	p.Ile1377Leu	missense_variant	hypothetical protein
TGME49_249890	p.Ala837Pro	missense_variant	hypothetical protein
TGME49_249890	p.Ala745Thr	missense_variant	hypothetical protein
TGME49_249890	p.Phe611Leu	missense_variant	hypothetical protein
TGME49_249890	p.Phe528Val	missense_variant	hypothetical protein
TGME49_249890	p.Arg278Trp	missense_variant	hypothetical protein
TGME49_249890	p.Ser190Leu	missense_variant	hypothetical protein
TGME49_249890	p.Ser176Phe	missense_variant	hypothetical protein
TGME49_249990	p.Arg727Gly	missense_variant	hypothetical protein
TGME49_249990	p.Thr88Ala	missense_variant	hypothetical protein
TGME49_250000	p.Tyr143His	missense_variant	hypothetical protein
TGME49_250000	p.Trp87*	stop_gained	hypothetical protein
TGME49_250000	p.Gly85Ser	missense_variant	hypothetical protein
TGME49_250030	p.Phe212Leu	missense_variant	hypothetical protein
TGME49_250040	p.Ile32Thr	missense_variant	hypothetical protein
TGME49_250050	p.Leu2070Arg	missense_variant	hypothetical protein
TGME49_250050	p.Ser1992Ala	missense_variant	hypothetical protein
TGME49_250050	p.Gln1990His	missense_variant	hypothetical protein
TGME49_250050	p.Ala1977Thr	missense_variant	hypothetical protein
TGME49_250050	p.Ala1826Gly	missense_variant	hypothetical protein
TGME49_250050	p.Ser1676Pro	missense_variant	hypothetical protein
TGME49_250050	p.Asp1453Val	missense_variant	hypothetical protein
TGME49_250050	p.Pro1291Arg	missense_variant	hypothetical protein
TGME49_250050	p.Gln1018Glu	missense_variant	hypothetical protein
TGME49_250050	c.2859-4T>G	splice_region_variant	hypothetical protein
TGME49_250050	p.Ala764Val	missense_variant	hypothetical protein
TGME49_250050	p.Ala671Thr	missense_variant	hypothetical protein
TGME49_250050	p.Ala543Val	missense_variant	hypothetical protein
TGME49_250050	p.Leu307Ile	missense_variant	hypothetical protein
TGME49_250050	p.Ala107Val	missense_variant	hypothetical protein
TGME49_250090	p.Pro93Ala	missense_variant	hypothetical protein
TGME49_250090	p.Asn235Ser	missense_variant	hypothetical protein
TGME49_250090	p.Glu726Lys	missense_variant	hypothetical protein
TGME49_250090	p.Thr770Ala	missense_variant	hypothetical protein
TGME49_250090	p.Cys787Tyr	missense_variant	hypothetical protein
TGME49_250100	c.22-8A>T	splice_region_variant	hypothetical protein
TGME49_250220	p.Thr50Asn	missense_variant	hypothetical protein
TGME49_250220	p.Pro159Thr	missense_variant&splice_region_variant	hypothetical protein
TGME49_250220	p.Leu289Trp	missense_variant	hypothetical protein
TGME49_250220	p.Glu293Lys	missense_variant	hypothetical protein
TGME49_250220	p.Glu321Ala	missense_variant	hypothetical protein
TGME49_250220	p.Cys352Tyr	missense_variant	hypothetical protein
TGME49_250220	p.Thr354Ser	missense_variant	hypothetical protein
TGME49_250330	p.Ala158Gly	missense_variant	hypothetical protein
TGME49_250670	p.Ala336Glu	missense_variant	hypothetical protein
TGME49_250670	p.Ala97Gly	missense_variant	hypothetical protein
TGME49_250670	p.Ala49Ser	missense_variant	hypothetical protein
TGME49_250700	p.Ala179Val	missense_variant	hypothetical protein
TGME49_250700	p.Gly628Ser	missense_variant	hypothetical protein
TGME49_250720	p.Thr556Lys	missense_variant	hypothetical protein
TGME49_250790	p.Ser125Cys	missense_variant	hypothetical protein
TGME49_250790	p.Pro492Ser	missense_variant	hypothetical protein
TGME49_250790	p.Pro1229Ser	missense_variant	hypothetical protein
TGME49_250820	p.His155Asn	missense_variant	hypothetical protein
TGME49_250820	p.Ile343Thr	missense_variant	hypothetical protein
TGME49_250820	p.His578Gln	missense_variant	hypothetical protein
TGME49_250820	p.Ser639Ala	missense_variant	hypothetical protein
TGME49_250840	c.-357T>G	5_prime_UTR_premature_start_codon_gain_variant	hypothetical protein
TGME49_250840	c.-192T>G	5_prime_UTR_premature_start_codon_gain_variant	hypothetical protein
TGME49_250840	p.Ala138Ser	missense_variant	hypothetical protein
TGME49_250840	c.2331+4G>C	splice_region_variant	hypothetical protein
TGME49_250840	p.Ala802Thr	missense_variant	hypothetical protein
TGME49_250910	c.1254-4A>G	splice_region_variant	hypothetical protein
TGME49_250930	p.Leu34Ile	missense_variant	hypothetical protein
TGME49_250940	p.Pro44Ser	missense_variant	hypothetical protein
TGME49_251400	p.Ala4Val	missense_variant	hypothetical protein
TGME49_251400	p.Ser666Ala	missense_variant	hypothetical protein
TGME49_251430	p.Glu2Gln	missense_variant	hypothetical protein
TGME49_251430	p.Ser104Ile	missense_variant	hypothetical protein
TGME49_251430	p.His476Gln	missense_variant	hypothetical protein
TGME49_251450	p.Ala116Thr	missense_variant	hypothetical protein
TGME49_251450	p.Arg261His	missense_variant	hypothetical protein
TGME49_251450	p.Arg294Lys	missense_variant	hypothetical protein
TGME49_251450	p.Met301Ile	missense_variant	hypothetical protein
TGME49_251450	p.Arg559Gln	missense_variant	hypothetical protein
TGME49_251450	p.Arg1052Gln	missense_variant	hypothetical protein
TGME49_251450	p.Gly1094Val	missense_variant	hypothetical protein

TGME49_251450	p.Leu1144Val	missense_variant	hypothetical protein
TGME49_251450	p.Ala1334Thr	missense_variant	hypothetical protein
TGME49_251450	p.Gly1418Ala	missense_variant	hypothetical protein
TGME49_251450	p.Ala1878Glu	missense_variant	hypothetical protein
TGME49_251450	p.Ser1985Thr	missense_variant	hypothetical protein
TGME49_251450	p.Leu1987Val	missense_variant	hypothetical protein
TGME49_251450	p.Met2258Ile	missense_variant	hypothetical protein
TGME49_251450	p.Arg2270Lys	missense_variant	hypothetical protein
TGME49_251460	p.Ser1008Thr	missense_variant	hypothetical protein
TGME49_251460	p.Val987Gly	missense_variant	hypothetical protein
TGME49_251460	p.Arg954Trp	missense_variant	hypothetical protein
TGME49_251460	p.Ser893Leu	missense_variant	hypothetical protein
TGME49_251460	p.Ile738Val	missense_variant	hypothetical protein
TGME49_251460	p.Pro277Leu	missense_variant	hypothetical protein
TGME49_251460	p.Ala105Thr	missense_variant	hypothetical protein
TGME49_251460	p.His93Tyr	missense_variant	hypothetical protein
TGME49_251460	c.-136C>G	5_prime_UTR_premature_start_codon_gain_variant	hypothetical protein
TGME49_251460	c.-448C>T	5_prime_UTR_premature_start_codon_gain_variant	hypothetical protein
TGME49_251520	p.Ala134Val	missense_variant	hypothetical protein
TGME49_251520	p.Pro48Arg	missense_variant	hypothetical protein
TGME49_251530	p.Leu17Gln	missense_variant	hypothetical protein
TGME49_251560	p.Ser41Asn	missense_variant	hypothetical protein
TGME49_251560	p.Ala71Ser	missense_variant	hypothetical protein
TGME49_251560	p.Ala71Val	missense_variant	hypothetical protein
TGME49_251560	p.Arg169Gln	missense_variant	hypothetical protein
TGME49_251560	p.Glu392Lys	missense_variant	hypothetical protein
TGME49_251560	p.Pro659Ser	missense_variant	hypothetical protein
TGME49_251560	p.Ala750Gly	missense_variant	hypothetical protein
TGME49_251560	p.Ser780Phe	missense_variant	hypothetical protein
TGME49_251560	p.Glu820Lys	missense_variant	hypothetical protein
TGME49_251560	p.Asp852Asn	missense_variant	hypothetical protein
TGME49_251560	p.Arg904Cys	missense_variant	hypothetical protein
TGME49_251560	p.Glu989Lys	missense_variant	hypothetical protein
TGME49_251560	p.Val1027Asp	missense_variant	hypothetical protein
TGME49_251560	p.Asp1285His	missense_variant	hypothetical protein
TGME49_251560	p.Ile1345Thr	missense_variant	hypothetical protein
TGME49_251560	p.Gly1607Val	missense_variant	hypothetical protein
TGME49_251560	p.Ser1656Asn	missense_variant	hypothetical protein
TGME49_251580	p.Val46Leu	missense_variant	hypothetical protein
TGME49_251590	p.Arg729Lys	missense_variant	hypothetical protein
TGME49_251590	p.Arg272Lys	missense_variant	hypothetical protein
TGME49_251590	p.Thr34Arg	missense_variant	hypothetical protein
TGME49_251590	p.Ser20Cys	missense_variant	hypothetical protein
TGME49_251590	p.Phe11Leu	missense_variant	hypothetical protein
TGME49_251610	c.-76C>T	5_prime_UTR_premature_start_codon_gain_variant	hypothetical protein
TGME49_251610	p.Ser176Pro	missense_variant	hypothetical protein
TGME49_251610	p.Ala388Pro	missense_variant	hypothetical protein
TGME49_251610	p.Gly563Arg	missense_variant	hypothetical protein
TGME49_251610	p.Ser712Thr	missense_variant	hypothetical protein
TGME49_251650	p.Val2Ile	missense_variant	hypothetical protein
TGME49_251650	p.Phe3Ile	missense_variant	hypothetical protein
TGME49_251650	p.Val16Ile	missense_variant	hypothetical protein
TGME49_251650	p.Arg134Gln	missense_variant	hypothetical protein
TGME49_251650	p.Lys141*	stop_gained	hypothetical protein
TGME49_251650	c.*71C>T	splice_region_variant	hypothetical protein
TGME49_251660	p.Gln41His	missense_variant	hypothetical protein
TGME49_251660	p.Asp47Gly	missense_variant	hypothetical protein
TGME49_251660	p.Ala57Glu	missense_variant	hypothetical protein
TGME49_251660	p.Ala104Glu	missense_variant	hypothetical protein
TGME49_251665	p.Ser28Arg	missense_variant	hypothetical protein
TGME49_251665	p.Thr37Ile	missense_variant	hypothetical protein
TGME49_251665	p.Ser146Asn	missense_variant	hypothetical protein
TGME49_251665	p.Ile181Thr	missense_variant	hypothetical protein
TGME49_251665	p.Asp184His	missense_variant	hypothetical protein
TGME49_251730	c.-123C>G	5_prime_UTR_premature_start_codon_gain_variant	hypothetical protein
TGME49_251730	p.Asn387Thr	missense_variant	hypothetical protein
TGME49_251730	p.Cys1131Arg	missense_variant	hypothetical protein
TGME49_251730	p.Ala1149Val	missense_variant	hypothetical protein
TGME49_251730	p.Ser1335Thr	missense_variant	hypothetical protein
TGME49_251770	p.Gly1173Asp	missense_variant	hypothetical protein
TGME49_251770	p.Thr665Ala	missense_variant	hypothetical protein
TGME49_251770	p.Leu577Gln	missense_variant	hypothetical protein
TGME49_251770	p.Gly553Asp	missense_variant	hypothetical protein
TGME49_251770	c.235+7T>G	splice_region_variant	hypothetical protein
TGME49_251770	c.-440C>T	5_prime_UTR_premature_start_codon_gain_variant	hypothetical protein
TGME49_251790	c.-1148G>A	5_prime_UTR_premature_start_codon_gain_variant	hypothetical protein
TGME49_251790	c.-887C>T	5_prime_UTR_premature_start_codon_gain_variant	hypothetical protein
TGME49_251790	p.Arg80Gln	missense_variant	hypothetical protein
TGME49_251790	p.Glu203Asp	missense_variant	hypothetical protein
TGME49_251800	p.Thr285Lys	missense_variant	hypothetical protein
TGME49_251800	p.Pro29Ser	missense_variant	hypothetical protein
TGME49_251820	p.Ile287Val	missense_variant	hypothetical protein
TGME49_251820	p.Ala123Val	missense_variant	hypothetical protein
TGME49_251830	p.Val58Ala	missense_variant	hypothetical protein
TGME49_251855	c.-367C>T	5_prime_UTR_premature_start_codon_gain_variant	hypothetical protein

TGME49_251860	p.Gln139Glu	missense_variant	hypothetical protein
TGME49_251890	p.Glu1640Asp	missense_variant	hypothetical protein
TGME49_251890	p.Val1483Met	missense_variant	hypothetical protein
TGME49_251890	p.Cys960Ser	missense_variant	hypothetical protein
TGME49_251890	p.Val642Ile	missense_variant	hypothetical protein
TGME49_251890	c.1902-8T>G	splice_region_variant	hypothetical protein
TGME49_251890	p.Lys558Glu	missense_variant	hypothetical protein
TGME49_251890	p.Glu183Asp	missense_variant	hypothetical protein
TGME49_251890	p.Arg89Trp	missense_variant	hypothetical protein
TGME49_251890	p.Val20Ala	missense_variant	hypothetical protein
TGME49_251890	p.Ala12Val	missense_variant	hypothetical protein
TGME49_251920	p.Gly736Arg	missense_variant	hypothetical protein
TGME49_251920	p.Asn355His	missense_variant	hypothetical protein
TGME49_251920	p.Gly300Ser	missense_variant	hypothetical protein
TGME49_278965	p.Ile273Lys	missense_variant	hypothetical protein
TGME49_278920	p.Gly102Ser	missense_variant	hypothetical protein
TGME49_278920	c.-894C>T	5_prime_UTR_premature_start_codon_gain_variant	hypothetical protein
TGME49_278890	p.Glu136Asp	missense_variant	hypothetical protein
TGME49_278890	p.Pro161Ser	missense_variant	hypothetical protein
TGME49_278890	c.2317-8T>A	splice_region_variant	hypothetical protein
TGME49_278860	p.Thr385Ile	missense_variant	hypothetical protein
TGME49_278860	p.Lys239Gln	missense_variant	hypothetical protein
TGME49_278860	c.128-5G>C	splice_region_variant	hypothetical protein
TGME49_278860	c.128-6C>T	splice_region_variant	hypothetical protein
TGME49_278840	p.Ser221Thr	missense_variant	hypothetical protein
TGME49_278840	p.Tyr300His	missense_variant	hypothetical protein
TGME49_278815	p.Arg7Gln	missense_variant	hypothetical protein
TGME49_278815	p.Leu121Val	missense_variant	hypothetical protein
TGME49_278815	p.Cys142Arg	missense_variant	hypothetical protein
TGME49_278815	p.Arg243Gly	missense_variant	hypothetical protein
TGME49_278815	p.Pro291His	missense_variant	hypothetical protein
TGME49_278815	p.Arg321His	missense_variant	hypothetical protein
TGME49_278815	p.Gly382Asp	missense_variant	hypothetical protein
TGME49_278815	p.Glu646Gln	missense_variant	hypothetical protein
TGME49_278815	p.Val955Ala	missense_variant	hypothetical protein
TGME49_278815	p.Gly1381Arg	missense_variant	hypothetical protein
TGME49_278815	p.Gly1609Arg	missense_variant	hypothetical protein
TGME49_278815	p.Ile1685Leu	missense_variant	hypothetical protein
TGME49_278815	p.Ala1790Glu	missense_variant	hypothetical protein
TGME49_278815	p.Ser1919Leu	missense_variant	hypothetical protein
TGME49_278780	p.Ser47Pro	missense_variant	hypothetical protein
TGME49_278770	p.Arg946His	missense_variant	hypothetical protein
TGME49_278770	c.2578-5T>C	splice_region_variant	hypothetical protein
TGME49_278770	p.Thr812Ile	missense_variant	hypothetical protein
TGME49_278770	p.Asp226Gly	missense_variant	hypothetical protein
TGME49_278770	p.Ser177Pro	missense_variant	hypothetical protein
TGME49_278770	c.-538A>G	5_prime_UTR_premature_start_codon_gain_variant	hypothetical protein
TGME49_278760	p.Pro1073Leu	missense_variant	hypothetical protein
TGME49_278760	p.Arg1041Lys	missense_variant	hypothetical protein
TGME49_278760	p.Trp957Cys	missense_variant	hypothetical protein
TGME49_278760	p.Val691Ile	missense_variant	hypothetical protein
TGME49_278760	p.Asp531Val	missense_variant	hypothetical protein
TGME49_278760	c.-181A>G	5_prime_UTR_premature_start_codon_gain_variant	hypothetical protein
TGME49_278753	p.Gly182Arg	missense_variant	hypothetical protein
TGME49_278720	p.Gln334Leu	missense_variant	hypothetical protein
TGME49_278720	p.Ser83Ala	missense_variant	hypothetical protein
TGME49_278710	p.Arg620His	missense_variant	hypothetical protein
TGME49_278710	p.Ala732Thr	missense_variant	hypothetical protein
TGME49_278710	p.Gly733Asp	missense_variant	hypothetical protein
TGME49_278710	p.Ile741Val	missense_variant	hypothetical protein
TGME49_278680	p.Asn160Ser	missense_variant	hypothetical protein
TGME49_278680	p.Val148Met	missense_variant	hypothetical protein
TGME49_278580	p.Leu247Val	missense_variant	hypothetical protein
TGME49_278540	p.Pro69Thr	missense_variant	hypothetical protein
TGME49_278540	p.Ala9Val	missense_variant	hypothetical protein
TGME49_278522	c.25-4A>T	splice_region_variant	hypothetical protein
TGME49_278522	p.Glu67Val	missense_variant	hypothetical protein
TGME49_278470	p.Pro206Arg	missense_variant	hypothetical protein
TGME49_278470	p.Asp577Gly	missense_variant	hypothetical protein
TGME49_278460	c.-217A>G	5_prime_UTR_premature_start_codon_gain_variant	hypothetical protein
TGME49_278460	c.-330C>T	5_prime_UTR_premature_start_codon_gain_variant	hypothetical protein
TGME49_278460	c.-1166C>A	5_prime_UTR_premature_start_codon_gain_variant	hypothetical protein
TGME49_278250	p.Val89Ala	missense_variant	hypothetical protein
TGME49_278250	p.Arg96Pro	missense_variant	hypothetical protein
TGME49_278250	p.Leu167Ser	missense_variant	hypothetical protein
TGME49_278250	c.2157+3A>T	splice_region_variant	hypothetical protein
TGME49_278205	p.Ala4917Thr	missense_variant	hypothetical protein
TGME49_278205	p.Ser4907Phe	missense_variant	hypothetical protein
TGME49_278205	p.Asn4830Thr	missense_variant	hypothetical protein
TGME49_278205	p.Ala4535Thr	missense_variant	hypothetical protein
TGME49_278205	p.Ile4396Thr	missense_variant	hypothetical protein
TGME49_278205	p.Glu4353Asp	missense_variant	hypothetical protein
TGME49_278205	p.Ser4236Asn	missense_variant	hypothetical protein
TGME49_278205	p.Arg4151Thr	missense_variant	hypothetical protein
TGME49_278205	p.Gln4006His	missense_variant	hypothetical protein

TGME49_278205	p.Leu3977Val	missense_variant	hypothetical protein
TGME49_278205	p.Gly3787Ala	missense_variant	hypothetical protein
TGME49_278205	p.Arg3443Ser	missense_variant	hypothetical protein
TGME49_278205	p.Cys2699Ser	missense_variant	hypothetical protein
TGME49_278205	p.Asp1958Asn	missense_variant	hypothetical protein
TGME49_278205	p.Arg989His	missense_variant	hypothetical protein
TGME49_278180	p.Ala155Ser	missense_variant	hypothetical protein
TGME49_278145	p.Leu304Phe	missense_variant	hypothetical protein
TGME49_278145	p.Lys10Ile	missense_variant	hypothetical protein
TGME49_278130	p.Arg1041His	missense_variant	hypothetical protein
TGME49_278130	p.Glu920Gln	missense_variant	hypothetical protein
TGME49_278130	c.1233-6T>A	splice_region_variant	hypothetical protein
TGME49_278030	p.Arg956Lys	missense_variant	hypothetical protein
TGME49_278030	p.Val1047Met	missense_variant	hypothetical protein
TGME49_278030	p.Thr1133Ala	missense_variant	hypothetical protein
TGME49_278030	p.Arg1231Gly	missense_variant	hypothetical protein
TGME49_278030	p.His1909Gln	missense_variant	hypothetical protein
TGME49_278030	p.Asp1939Glu	missense_variant	hypothetical protein
TGME49_278030	p.Gly1987Glu	missense_variant	hypothetical protein
TGME49_278030	p.Ala2002Ser	missense_variant	hypothetical protein
TGME49_278030	p.Ala2002Val	missense_variant	hypothetical protein
TGME49_278030	p.Gly2079Ser	missense_variant	hypothetical protein
TGME49_278020	p.Arg808Thr	missense_variant	hypothetical protein
TGME49_278020	p.Ala717Glu	missense_variant	hypothetical protein
TGME49_278020	p.Leu572His	missense_variant	hypothetical protein
TGME49_278020	p.Val403Met	missense_variant	hypothetical protein
TGME49_278020	p.Gly257Ser	missense_variant	hypothetical protein
TGME49_278020	p.Ser174Cys	missense_variant	hypothetical protein
TGME49_278020	p.Asp156Gly	missense_variant	hypothetical protein
TGME49_277940	p.Ser2769Ala	missense_variant	hypothetical protein
TGME49_277940	p.Arg2363Ile	missense_variant	hypothetical protein
TGME49_277940	p.Ser2323Pro	missense_variant	hypothetical protein
TGME49_277940	p.Gln2054Lys	missense_variant	hypothetical protein
TGME49_277940	p.Ile1918Phe	missense_variant	hypothetical protein
TGME49_277940	p.Ser1500Pro	missense_variant	hypothetical protein
TGME49_277940	p.Val1218Gly	missense_variant	hypothetical protein
TGME49_277940	p.Phe406Cys	missense_variant	hypothetical protein
TGME49_277930	p.Leu1041Phe	missense_variant	hypothetical protein
TGME49_277930	p.Val968Ile	missense_variant	hypothetical protein
TGME49_277930	p.His956Tyr	missense_variant	hypothetical protein
TGME49_277930	p.Thr335Ile	missense_variant	hypothetical protein
TGME49_277930	p.Lys328Arg	missense_variant	hypothetical protein
TGME49_277930	p.Asp302Gly	missense_variant	hypothetical protein
TGME49_277930	p.Ala295Thr	missense_variant	hypothetical protein
TGME49_277930	p.Leu275Ile	missense_variant	hypothetical protein
TGME49_277930	c.798-3T>C	splice_region_variant	hypothetical protein
TGME49_277920	p.Thr17Arg	missense_variant	hypothetical protein
TGME49_277880	p.Ala185Thr	missense_variant	hypothetical protein
TGME49_277870	c.-1137C>T	5_prime_UTR_premature_start_codon_gain_variant	hypothetical protein
TGME49_277870	p.Glu109Val	missense_variant	hypothetical protein
TGME49_277870	c.*1259A>C	splice_region_variant	hypothetical protein
TGME49_277860	p.Phe118Ile	missense_variant	hypothetical protein
TGME49_277860	p.Gln40Arg	missense_variant	hypothetical protein
TGME49_277860	p.Ser38Ala	missense_variant	hypothetical protein
TGME49_277860	c.-140C>T	5_prime_UTR_premature_start_codon_gain_variant	hypothetical protein
TGME49_277830	p.Ser129Pro	missense_variant	hypothetical protein
TGME49_277830	p.Ala80Ser	missense_variant	hypothetical protein
TGME49_277830	p.Ser35Leu	missense_variant	hypothetical protein
TGME49_277820	p.Leu58Phe	missense_variant	hypothetical protein
TGME49_277820	p.Asp140Glu	missense_variant	hypothetical protein
TGME49_277820	p.Ser197Arg	missense_variant	hypothetical protein
TGME49_277820	p.Lys229Gln	missense_variant	hypothetical protein
TGME49_277820	p.Glu389Lys	missense_variant	hypothetical protein
TGME49_277820	p.Gln607Pro	missense_variant	hypothetical protein
TGME49_277820	p.Ser881Asn	missense_variant	hypothetical protein
TGME49_277820	p.Ala1049Thr	missense_variant	hypothetical protein
TGME49_277820	p.Ala1189Val	missense_variant	hypothetical protein
TGME49_277820	p.Pro1195Ser	missense_variant	hypothetical protein
TGME49_277810	c.-2C>G	5_prime_UTR_premature_start_codon_gain_variant	hypothetical protein
TGME49_277790	p.Glu1353Ala	missense_variant	hypothetical protein
TGME49_277790	p.Glu1338Asp	missense_variant	hypothetical protein
TGME49_277790	p.Ser1228Asn	missense_variant	hypothetical protein
TGME49_277790	p.Asn1222Ser	missense_variant	hypothetical protein
TGME49_277790	p.Pro1092Ala	missense_variant	hypothetical protein
TGME49_277790	p.Ile577Val	missense_variant	hypothetical protein
TGME49_277790	c.-960G>A	5_prime_UTR_premature_start_codon_gain_variant	hypothetical protein
TGME49_277780	p.Asp135Asn	missense_variant	hypothetical protein
TGME49_277770	p.Arg821His	missense_variant	hypothetical protein
TGME49_277770	p.Gly721Ser	missense_variant	hypothetical protein
TGME49_277770	p.Gly372Glu	missense_variant	hypothetical protein
TGME49_277770	p.Arg295Ser	missense_variant	hypothetical protein
TGME49_277770	p.Lys102Arg	missense_variant	hypothetical protein
TGME49_277770	p.Lys102Glu	missense_variant	hypothetical protein
TGME49_277770	c.-13-7T>G	splice_region_variant	hypothetical protein
TGME49_277730	p.Trp3*	stop_gained	hypothetical protein

TGME49_277730	p.Pro75Ser	missense_variant	hypothetical protein
TGME49_277730	p.Lys108Asn	missense_variant	hypothetical protein
TGME49_277730	p.Ser109Phe	missense_variant	hypothetical protein
TGME49_277710	p.Cys130Ser	missense_variant	hypothetical protein
TGME49_277710	p.Pro16Ala	missense_variant	hypothetical protein
TGME49_277710	c.-559C>T	5_prime_UTR_premature_start_codon_gain_variant	hypothetical protein
TGME49_277710	c.-934C>G	5_prime_UTR_premature_start_codon_gain_variant	hypothetical protein
TGME49_277705	p.Leu14Val	missense_variant	hypothetical protein
TGME49_277705	p.Glu31Lys	missense_variant	hypothetical protein
TGME49_277690	c.-96C>G	5_prime_UTR_premature_start_codon_gain_variant	hypothetical protein
TGME49_277560	p.Arg202Lys	missense_variant	hypothetical protein
TGME49_277560	p.Pro491Arg	missense_variant	hypothetical protein
TGME49_277560	p.His586Asp	missense_variant	hypothetical protein
TGME49_277560	p.Pro589Ser	missense_variant	hypothetical protein
TGME49_277560	p.Asn598Asp	missense_variant	hypothetical protein
TGME49_277560	p.Thr629Ala	missense_variant	hypothetical protein
TGME49_277560	p.Ala786Pro	missense_variant	hypothetical protein
TGME49_277560	p.Leu855Pro	missense_variant	hypothetical protein
TGME49_277560	p.Pro949Ser	missense_variant	hypothetical protein
TGME49_277560	p.Gly1137Ala	missense_variant	hypothetical protein
TGME49_277560	p.Ala1359Glu	missense_variant	hypothetical protein
TGME49_277560	p.Glu1651Asp	missense_variant	hypothetical protein
TGME49_277560	p.Lys1692Thr	missense_variant	hypothetical protein
TGME49_277560	p.Arg1701His	missense_variant	hypothetical protein
TGME49_277560	p.Ala1768Val	missense_variant	hypothetical protein
TGME49_277540	p.Ser2023Pro	missense_variant	hypothetical protein
TGME49_277540	p.Asp1996Gly	missense_variant	hypothetical protein
TGME49_277540	p.Gly1934Ala	missense_variant	hypothetical protein
TGME49_277540	p.Thr1928Met	missense_variant	hypothetical protein
TGME49_277540	p.Asp1863Asn	missense_variant	hypothetical protein
TGME49_277540	p.Gly1856Asp	missense_variant	hypothetical protein
TGME49_277540	p.Ala1728Ser	missense_variant	hypothetical protein
TGME49_277540	p.Ser1604Ala	missense_variant	hypothetical protein
TGME49_277540	p.Met1335Lys	missense_variant	hypothetical protein
TGME49_277540	p.Gln1322Arg	missense_variant	hypothetical protein
TGME49_277540	p.Pro1253Ser	missense_variant	hypothetical protein
TGME49_277540	p.Leu487Gln	missense_variant	hypothetical protein
TGME49_277540	p.Arg439Ser	missense_variant	hypothetical protein
TGME49_277540	p.Ala437Thr	missense_variant	hypothetical protein
TGME49_277540	p.Glu316Gln	missense_variant	hypothetical protein
TGME49_277490	p.Leu2264Val	missense_variant	hypothetical protein
TGME49_277490	p.Gly2190Ala	missense_variant	hypothetical protein
TGME49_277490	p.Phe2067Leu	missense_variant	hypothetical protein
TGME49_277490	p.Pro1870Leu	missense_variant	hypothetical protein
TGME49_277490	p.Thr1274Ala	missense_variant	hypothetical protein
TGME49_277490	p.Glu1272Lys	missense_variant	hypothetical protein
TGME49_277490	p.Ser952Arg	missense_variant	hypothetical protein
TGME49_277490	p.Thr121Ala	missense_variant	hypothetical protein
TGME49_277490	p.Ala101Thr	missense_variant	hypothetical protein
TGME49_277490	p.His32Arg	missense_variant	hypothetical protein
TGME49_277260	p.Glu1598Ala	missense_variant	hypothetical protein
TGME49_277260	p.Arg1339Leu	missense_variant	hypothetical protein
TGME49_277260	c.3373-5C>A	splice_region_variant	hypothetical protein
TGME49_277260	p.Ile1118Leu	missense_variant	hypothetical protein
TGME49_277260	p.Val575Ile	missense_variant	hypothetical protein
TGME49_277260	p.Ile320Leu	missense_variant	hypothetical protein
TGME49_277260	p.Ser303Tyr	missense_variant	hypothetical protein
TGME49_277260	p.Thr236Met	missense_variant	hypothetical protein
TGME49_277260	p.Asp233Gly	missense_variant	hypothetical protein
TGME49_277260	c.-2178C>T	5_prime_UTR_premature_start_codon_gain_variant	hypothetical protein
TGME49_277260	c.-3112G>A	5_prime_UTR_premature_start_codon_gain_variant	hypothetical protein
TGME49_277230	p.Ser391Pro	missense_variant	hypothetical protein
TGME49_277220	p.Ala473Ser	missense_variant	hypothetical protein
TGME49_277220	p.Gly395Ser	missense_variant	hypothetical protein
TGME49_277220	p.Val354Ile	missense_variant	hypothetical protein
TGME49_277220	p.Ala273Gly	missense_variant	hypothetical protein
TGME49_277220	p.Arg211His	missense_variant	hypothetical protein
TGME49_277060	p.Gly1480Ala	missense_variant	hypothetical protein
TGME49_277060	p.Cys1406Arg	missense_variant	hypothetical protein
TGME49_277060	p.Arg1228Ser	missense_variant	hypothetical protein
TGME49_277060	p.Ser1037Leu	missense_variant	hypothetical protein
TGME49_277060	c.-338C>T	5_prime_UTR_premature_start_codon_gain_variant	hypothetical protein
TGME49_277020	p.Met91Val	missense_variant	hypothetical protein
TGME49_277020	p.Arg19Gly	missense_variant	hypothetical protein
TGME49_276980	p.Pro56Ser	missense_variant	hypothetical protein
TGME49_276970	p.Ala189Val	missense_variant	hypothetical protein
TGME49_276970	p.Ala351Thr	missense_variant	hypothetical protein
TGME49_276860	p.Ala120Val	missense_variant	hypothetical protein
TGME49_276840	p.Gly133Glu	missense_variant	hypothetical protein
TGME49_214620	p.Ala206Thr	missense_variant	hypoxia- inducible factor prolyl hydroxylase (phd2), putative
TGME49_278975	c.-152C>T	5_prime_UTR_premature_start_codon_gain_variant	ICE family protease (caspase) p20 domain-containing protein
TGME49_319090	p.Gln675Arg	missense_variant	IgA-specific serine endopeptidase
TGME49_319090	p.Asp844Glu	missense_variant	IgA-specific serine endopeptidase
TGME49_222710	p.Ser14Leu	missense_variant	IMP-specific 5'-nucleotidase 1, putative
TGME49_222710	p.Ser40Pro	missense_variant	IMP-specific 5'-nucleotidase 1, putative

TGME49_222710	p.Ala59Ser	missense_variant	IMP-specific 5'-nucleotidase 1, putative
TGME49_251600	p.Val110Ile	missense_variant	IMP4 family U3 small nucleolar ribonucleoprotein (snoRNP), putative
TGME49_234580	p.Asp984Gly	missense_variant	ImpB/MucB/SamB family protein
TGME49_234580	p.Cys979Ser	missense_variant	ImpB/MucB/SamB family protein
TGME49_234580	p.Val825Met	missense_variant	ImpB/MucB/SamB family protein
TGME49_234580	p.Ala106Ser	missense_variant	ImpB/MucB/SamB family protein
TGME49_234580	p.Ile105Thr	missense_variant	ImpB/MucB/SamB family protein
TGME49_222380	c.-3574A>G	5_prime_UTR_premature_start_codon_gain_variant	importin-beta N-terminal domain-containing protein
TGME49_222380	c.-2085T>G	5_prime_UTR_premature_start_codon_gain_variant	importin-beta N-terminal domain-containing protein
TGME49_222380	c.-630C>T	5_prime_UTR_premature_start_codon_gain_variant	importin-beta N-terminal domain-containing protein
TGME49_207180	p.Pro49Ser	missense_variant	indole-3-glycerol phosphate synthase domain-containing protein
TGME49_207180	p.Met65Val	missense_variant	indole-3-glycerol phosphate synthase domain-containing protein
TGME49_207180	p.Thr1049Ser	missense_variant	indole-3-glycerol phosphate synthase domain-containing protein
TGME49_207180	p.Gln1146Arg	missense_variant	indole-3-glycerol phosphate synthase domain-containing protein
TGME49_207180	p.Gln1405Lys	missense_variant	indole-3-glycerol phosphate synthase domain-containing protein
TGME49_238995	p.His2104Tyr	missense_variant	ion channel protein
TGME49_277030	p.His2348Asp	missense_variant	isoleucyl-tRNA synthetase, putative
TGME49_277030	p.Glu2242Gly	missense_variant	isoleucyl-tRNA synthetase, putative
TGME49_277030	p.Thr1977Pro	missense_variant	isoleucyl-tRNA synthetase, putative
TGME49_277030	p.Val1955Met	missense_variant	isoleucyl-tRNA synthetase, putative
TGME49_277030	p.Pro1757Ser	missense_variant	isoleucyl-tRNA synthetase, putative
TGME49_277030	p.Gly1258Val	missense_variant	isoleucyl-tRNA synthetase, putative
TGME49_277030	p.Lys1152Thr	missense_variant	isoleucyl-tRNA synthetase, putative
TGME49_277030	p.Ala887Thr	missense_variant	isoleucyl-tRNA synthetase, putative
TGME49_277030	p.Pro850Arg	missense_variant	isoleucyl-tRNA synthetase, putative
TGME49_277030	p.His794Arg	missense_variant	isoleucyl-tRNA synthetase, putative
TGME49_277030	p.Lys690Arg	missense_variant	isoleucyl-tRNA synthetase, putative
TGME49_277030	c.-992T>G	5_prime_UTR_premature_start_codon_gain_variant	isoleucyl-tRNA synthetase, putative
TGME49_224080	p.Ser142Pro	missense_variant	Kazal-type serine protease inhibitor domain-containing protein
TGME49_224080	p.Leu143Pro	missense_variant	Kazal-type serine protease inhibitor domain-containing protein
TGME49_224080	p.Ala434Gly	missense_variant	Kazal-type serine protease inhibitor domain-containing protein
TGME49_224080	p.Phe601Leu	missense_variant	Kazal-type serine protease inhibitor domain-containing protein
TGME49_224080	p.Leu854Phe	missense_variant	Kazal-type serine protease inhibitor domain-containing protein
TGME49_224080	p.Leu854Arg	missense_variant	Kazal-type serine protease inhibitor domain-containing protein
TGME49_224080	p.Gly906Glu	missense_variant	Kazal-type serine protease inhibitor domain-containing protein
TGME49_224080	p.Lys907Arg	missense_variant	Kazal-type serine protease inhibitor domain-containing protein
TGME49_224080	p.Ala1162Ser	missense_variant	Kazal-type serine protease inhibitor domain-containing protein
TGME49_224080	p.Leu1233Phe	missense_variant	Kazal-type serine protease inhibitor domain-containing protein
TGME49_224080	p.Ala1311Ser	missense_variant	Kazal-type serine protease inhibitor domain-containing protein
TGME49_224080	p.Pro1314Ser	missense_variant	Kazal-type serine protease inhibitor domain-containing protein
TGME49_224080	p.Gly1418Val	missense_variant	Kazal-type serine protease inhibitor domain-containing protein
TGME49_224080	p.Pro1428Leu	missense_variant	Kazal-type serine protease inhibitor domain-containing protein
TGME49_224080	p.Ser1452Phe	missense_variant	Kazal-type serine protease inhibitor domain-containing protein
TGME49_224080	p.Thr1593Ala	missense_variant	Kazal-type serine protease inhibitor domain-containing protein
TGME49_224080	p.Ser1703Ala	missense_variant	Kazal-type serine protease inhibitor domain-containing protein
TGME49_224080	p.Asp1756Glu	missense_variant	Kazal-type serine protease inhibitor domain-containing protein
TGME49_224080	p.Pro1757Arg	missense_variant	Kazal-type serine protease inhibitor domain-containing protein
TGME49_224080	p.Leu1828Pro	missense_variant	Kazal-type serine protease inhibitor domain-containing protein
TGME49_224080	p.Gln1934Glu	missense_variant	Kazal-type serine protease inhibitor domain-containing protein
TGME49_224080	p.Gly1953Asp	missense_variant	Kazal-type serine protease inhibitor domain-containing protein
TGME49_224080	p.Thr1963Lys	missense_variant	Kazal-type serine protease inhibitor domain-containing protein
TGME49_224080	p.Lys1999Arg	missense_variant	Kazal-type serine protease inhibitor domain-containing protein
TGME49_234490	p.Glu875Gly	missense_variant	kelch repeat-containing protein
TGME49_250880	p.Gly403Ala	missense_variant	kinase, pfkB family protein
TGME49_250880	p.Ala367Val	missense_variant	kinase, pfkB family protein
TGME49_297110	p.Asn1259Ser	missense_variant	kinesin motor domain-containing protein
TGME49_297110	p.His1112Arg	missense_variant	kinesin motor domain-containing protein
TGME49_297110	p.Gly377Ala	missense_variant	kinesin motor domain-containing protein
TGME49_297110	c.-162C>T	5_prime_UTR_premature_start_codon_gain_variant	kinesin motor domain-containing protein
TGME49_319710	p.Ala299Ser	missense_variant	kinesin motor domain-containing protein
TGME49_319710	p.Ala360Thr	missense_variant	kinesin motor domain-containing protein
TGME49_319710	p.Thr535Ser	missense_variant	kinesin motor domain-containing protein
TGME49_319710	p.Ala657Thr	missense_variant	kinesin motor domain-containing protein
TGME49_319710	p.Pro812Arg	missense_variant	kinesin motor domain-containing protein
TGME49_319710	p.Ser1016Gly	missense_variant	kinesin motor domain-containing protein
TGME49_319710	p.Ala1047Ser	missense_variant	kinesin motor domain-containing protein
TGME49_319710	p.Ser1081Ala	missense_variant	kinesin motor domain-containing protein
TGME49_224880	p.Val1562Ala	missense_variant	kinesin motor domain-containing protein
TGME49_224880	p.Lys1316Gln	missense_variant	kinesin motor domain-containing protein
TGME49_224880	p.Thr1177Ile	missense_variant	kinesin motor domain-containing protein
TGME49_224880	p.Ile901Val	missense_variant	kinesin motor domain-containing protein
TGME49_224880	p.Asp63Glu	missense_variant	kinesin motor domain-containing protein
TGME49_249020	p.Ala813Val	missense_variant	kinesin motor domain-containing protein
TGME49_249020	p.Glu1109Asp	missense_variant	kinesin motor domain-containing protein
TGME49_249020	p.Gly1213Glu	missense_variant	kinesin motor domain-containing protein
TGME49_211910	p.Trp696Arg	missense_variant	kinesin-related protein 3A, putative
TGME49_211910	p.Val353Ala	missense_variant	kinesin-related protein 3A, putative
TGME49_323000	p.Arg18Leu	missense_variant	KRUF family protein
TGME49_250955	p.Ser143Ala	missense_variant	KRUF family protein
TGME49_252190	p.Gly460Asp	missense_variant	KRUF family protein
TGME49_251170	p.Val215Gly	missense_variant	KRUF family protein
TGME49_251170	p.Glu211Gly	missense_variant	KRUF family protein
TGME49_251180	p.Leu258Val	missense_variant	KRUF family protein
TGME49_251180	p.Asn215His	missense_variant	KRUF family protein
TGME49_251180	c.-2554C>T	5_prime_UTR_premature_start_codon_gain_variant	KRUF family protein

TGME49_251180	c.-3271T>C	5_prime_UTR_premature_start_codon_gain_variant	KRUF family protein
TGME49_222660	c.4792-7T>C	splice_region_variant	leucine rich repeat-containing protein
TGME49_211430	p.Ser25Asn	missense_variant	leucine rich repeat-containing protein
TGME49_211430	p.Arg209Lys	missense_variant	leucine rich repeat-containing protein
TGME49_211430	p.Ser578Gly	missense_variant	leucine rich repeat-containing protein
TGME49_239110	p.Thr209Ile	missense_variant	leucine rich repeat-containing protein
TGME49_248240	p.Glu61Lys	missense_variant	leucine rich repeat-containing protein
TGME49_248240	p.Gly94Asp	missense_variant	leucine rich repeat-containing protein
TGME49_248240	p.Arg168Lys	missense_variant	leucine rich repeat-containing protein
TGME49_248240	p.Pro525Ser	missense_variant	leucine rich repeat-containing protein
TGME49_248240	p.Pro644Ser	missense_variant	leucine rich repeat-containing protein
TGME49_248240	p.Ala785Pro	missense_variant	leucine rich repeat-containing protein
TGME49_248240	p.Val957Leu	missense_variant	leucine rich repeat-containing protein
TGME49_248240	p.Asp1159His	missense_variant	leucine rich repeat-containing protein
TGME49_248240	p.Gln1238Leu	missense_variant	leucine rich repeat-containing protein
TGME49_248970	p.Arg319Gly	missense_variant	leucine rich repeat-containing protein
TGME49_249980	p.Met156Ile	missense_variant	leucine rich repeat-containing protein
TGME49_251940	p.Ala115Thr	missense_variant	leucine rich repeat-containing protein
TGME49_254330	p.Asp1131Tyr	missense_variant	lipase
TGME49_254330	p.Gly930Ser	missense_variant	lipase
TGME49_254330	p.Gly588Ser	missense_variant	lipase
TGME49_254330	p.Val400Ala	missense_variant	lipase
TGME49_277950	p.Asn139Asp	missense_variant	lipase
TGME49_277950	p.Pro287Ser	missense_variant	lipase
TGME49_277950	p.Phe489Val	missense_variant	lipase
TGME49_222200	p.Ser120Thr	missense_variant	LMBR1 family region protein, putative
TGME49_222200	c.773+6C>T	splice_region_variant	LMBR1 family region protein, putative
TGME49_300280	c.64-8G>T	splice_region_variant	LSM domain-containing protein
TGME49_211870	p.Thr64Ile	missense_variant	LSU ribosomal protein L18P, putative
TGME49_211870	c.1369+7A>G	splice_region_variant	LSU ribosomal protein L18P, putative
TGME49_254400	c.-1205C>T	5_prime_UTR_premature_start_codon_gain_variant	LSU ribosomal protein L2P, putative
TGME49_254400	c.-393C>T	5_prime_UTR_premature_start_codon_gain_variant	LSU ribosomal protein L2P, putative
TGME49_254400	c.-321C>G	5_prime_UTR_premature_start_codon_gain_variant	LSU ribosomal protein L2P, putative
TGME49_240250	p.Glu481Asp	missense_variant	macro domain-containing protein
TGME49_240250	p.Ser638Cys	missense_variant	macro domain-containing protein
TGME49_240250	p.Glu771Asp	missense_variant	macro domain-containing protein
TGME49_240250	p.Lys812Arg	missense_variant	macro domain-containing protein
TGME49_315590	p.Arg206Ser	missense_variant	macro domain-containing protein
TGME49_318430	p.Leu103Trp	missense_variant	malate dehydrogenase MDH
TGME49_253120	p.Val597Asp	missense_variant	mandelonitrile lyase, putative
TGME49_249702	p.Ala102Val	missense_variant	MC family transporter, putative
TGME49_249702	c.628-7T>C	splice_region_variant	MC family transporter, putative
TGME49_246030	p.Val147Ala	missense_variant	mediator complex subunit MED17
TGME49_246030	p.Ser265Gly	missense_variant	mediator complex subunit MED17
TGME49_246030	p.Gly653Asp	missense_variant	mediator complex subunit MED17
TGME49_246030	p.Pro783Gln	missense_variant	mediator complex subunit MED17
TGME49_223580	p.Cys79Phe	missense_variant	mediator complex subunit MED4
TGME49_223580	c.747-6G>T	splice_region_variant	mediator complex subunit MED4
TGME49_223580	p.Gln331Arg	missense_variant	mediator complex subunit MED4
TGME49_223580	p.Arg336Cys	missense_variant	mediator complex subunit MED4
TGME49_319550	p.Val24Ile	missense_variant	membrane protein C17G8.08c, putative
TGME49_319550	p.Ile126Val	missense_variant	membrane protein C17G8.08c, putative
TGME49_218740	c.-272A>T	5_prime_UTR_premature_start_codon_gain_variant	membrane protein, putative
TGME49_254080	p.Arg109Ser	missense_variant	metal cation transporter, ZIP family protein
TGME49_211330	p.Glu35Gly	missense_variant	methionine aminopeptidase
TGME49_211330	p.Thr110Ala	missense_variant	methionine aminopeptidase
TGME49_211330	p.Gly216Glu	missense_variant	methionine aminopeptidase
TGME49_211330	p.Gly259Arg	missense_variant	methionine aminopeptidase
TGME49_211330	p.Ala303Ser	missense_variant	methionine aminopeptidase
TGME49_211330	p.Thr647Met	missense_variant	methionine aminopeptidase
TGME49_301000	c.-620C>T	5_prime_UTR_premature_start_codon_gain_variant	methyltransferase
TGME49_247590	p.Gly46Arg	missense_variant	methyltransferase domain-containing protein
TGME49_247590	p.Gly172Val	missense_variant	methyltransferase domain-containing protein
TGME49_247590	p.Ala328Pro	missense_variant	methyltransferase domain-containing protein
TGME49_247590	p.Cys623Trp	missense_variant	methyltransferase domain-containing protein
TGME49_247590	p.Glu662Gly	missense_variant	methyltransferase domain-containing protein
TGME49_247920	p.Thr276Ile	missense_variant	methyltransferase domain-containing protein
TGME49_247920	p.Ala248Thr	missense_variant	methyltransferase domain-containing protein
TGME49_248960	p.Thr50Ile	missense_variant	methyltransferase domain-containing protein
TGME49_217350	c.1900-4T>G	splice_region_variant	methyltransferase MTA70, putative
TGME49_214590	c.-152C>T	5_prime_UTR_premature_start_codon_gain_variant	micro-fibrillar-associated protein 1
TGME49_214590	c.-61C>T	5_prime_UTR_premature_start_codon_gain_variant	micro-fibrillar-associated protein 1
TGME49_250710	p.Val32Ala	missense_variant	microneme protein MIC10
TGME49_247195	p.Asn1022His	missense_variant	microneme protein MIC15
TGME49_319560	c.-1365A>C	5_prime_UTR_premature_start_codon_gain_variant	microneme protein MIC3
TGME49_277080	p.Lys179Asn	missense_variant	microneme protein MIC5
TGME49_218520	c.-2776C>T	5_prime_UTR_premature_start_codon_gain_variant	microneme protein MIC6
TGME49_254940	p.Pro405Ser	missense_variant	MIF4G domain-containing protein
TGME49_254940	p.Gln916Lys	missense_variant	MIF4G domain-containing protein
TGME49_254940	p.Glu2727Gln	missense_variant	MIF4G domain-containing protein
TGME49_254940	p.Ser3093Gly	missense_variant	MIF4G domain-containing protein
TGME49_246040	p.Glu377Ala	missense_variant	MIF4G domain-containing protein
TGME49_246040	c.2397+8G>A	splice_region_variant	MIF4G domain-containing protein
TGME49_246040	p.Gly1040Ser	missense_variant	MIF4G domain-containing protein
TGME49_245470	c.-649C>G	5_prime_UTR_premature_start_codon_gain_variant	mitotic checkpoint protein, BUB3 family protein

TGME49_278640	p.Leu1658Pro	missense_variant	MIZ/SP-RING zinc finger domain-containing protein
TGME49_278640	p.Leu1527Pro	missense_variant	MIZ/SP-RING zinc finger domain-containing protein
TGME49_278640	p.Pro1433Ser	missense_variant	MIZ/SP-RING zinc finger domain-containing protein
TGME49_278640	p.Arg889Pro	missense_variant	MIZ/SP-RING zinc finger domain-containing protein
TGME49_278640	c.-1122C>T	5_prime_UTR_premature_start_codon_gain_variant	MIZ/SP-RING zinc finger domain-containing protein
TGME49_278640	c.-1997A>T	5_prime_UTR_premature_start_codon_gain_variant	MIZ/SP-RING zinc finger domain-containing protein
TGME49_223060	p.Glu23Lys	missense_variant	MORN repeat-containing protein
TGME49_223060	p.Val465Met	missense_variant	MORN repeat-containing protein
TGME49_223060	p.Glu702Lys	missense_variant	MORN repeat-containing protein
TGME49_223060	p.Ser1061Thr	missense_variant	MORN repeat-containing protein
TGME49_223060	p.Thr1162Ile	missense_variant	MORN repeat-containing protein
TGME49_223060	p.Glu1164Val	missense_variant	MORN repeat-containing protein
TGME49_223060	p.Leu1318Phe	missense_variant	MORN repeat-containing protein
TGME49_223060	p.Ala1366Glu	missense_variant	MORN repeat-containing protein
TGME49_223060	p.Thr1424Ala	missense_variant	MORN repeat-containing protein
TGME49_223060	p.Ala1437Val	missense_variant	MORN repeat-containing protein
TGME49_223060	p.Ser1443Ala	missense_variant	MORN repeat-containing protein
TGME49_223060	p.Arg1447Ser	missense_variant	MORN repeat-containing protein
TGME49_223060	p.Ile1516Val	missense_variant	MORN repeat-containing protein
TGME49_223060	p.Ile1516Met	missense_variant	MORN repeat-containing protein
TGME49_223060	p.Ala1570Thr	missense_variant	MORN repeat-containing protein
TGME49_223060	p.Pro1586Arg	missense_variant	MORN repeat-containing protein
TGME49_223060	p.Phe1914Leu	missense_variant	MORN repeat-containing protein
TGME49_223060	c.7534-3T>C	splice_region_variant	MORN repeat-containing protein
TGME49_297150	p.Asp416Gly	missense_variant	MORN repeat-containing protein
TGME49_297150	p.Pro399Ser	missense_variant	MORN repeat-containing protein
TGME49_297150	p.Ser293Gly	missense_variant	MORN repeat-containing protein
TGME49_297150	c.-18C>T	5_prime_UTR_premature_start_codon_gain_variant	MORN repeat-containing protein
TGME49_297150	c.-2015T>C	5_prime_UTR_premature_start_codon_gain_variant	MORN repeat-containing protein
TGME49_297150	c.-2478C>T	5_prime_UTR_premature_start_codon_gain_variant	MORN repeat-containing protein
TGME49_319880	p.Val146Ala	missense_variant	MORN repeat-containing protein
TGME49_319880	p.Arg229Lys	missense_variant	MORN repeat-containing protein
TGME49_319880	p.Arg246Gly	missense_variant	MORN repeat-containing protein
TGME49_319880	p.Gly300Asp	missense_variant	MORN repeat-containing protein
TGME49_319880	p.Val464Leu	missense_variant	MORN repeat-containing protein
TGME49_319880	p.Arg590Cys	missense_variant	MORN repeat-containing protein
TGME49_319880	p.Arg593Ser	missense_variant	MORN repeat-containing protein
TGME49_319880	c.2173+6A>G	splice_region_variant	MORN repeat-containing protein
TGME49_319880	p.Asn894Lys	missense_variant	MORN repeat-containing protein
TGME49_319880	c.3925-8T>C	splice_region_variant	MORN repeat-containing protein
TGME49_319880	p.Pro1503Ser	missense_variant	MORN repeat-containing protein
TGME49_319880	p.Pro1504Ser	missense_variant	MORN repeat-containing protein
TGME49_235515	p.Pro809Ser	missense_variant	MORN repeat-containing protein
TGME49_235515	p.Arg565Gln	missense_variant	MORN repeat-containing protein
TGME49_235515	c.944-6T>G	splice_region_variant	MORN repeat-containing protein
TGME49_235515	p.Val237Ala	missense_variant	MORN repeat-containing protein
TGME49_215510	c.2619+8G>A	splice_region_variant	MraW methylase family protein
TGME49_215510	p.Phe619Val	missense_variant	MraW methylase family protein
TGME49_215510	p.Gly518Ser	missense_variant	MraW methylase family protein
TGME49_215510	p.Cys349Arg	missense_variant	MraW methylase family protein
TGME49_215510	p.Val348Ala	missense_variant	MraW methylase family protein
TGME49_215510	p.Gly127Arg	missense_variant	MraW methylase family protein
TGME49_278060	c.1132-4G>A	splice_region_variant	Mre11 DNA-binding domain-containing protein
TGME49_224650	p.Val541Ala	missense_variant	mRNA capping enzyme, beta chain protein
TGME49_224650	p.Phe19Ile	missense_variant	mRNA capping enzyme, beta chain protein
TGME49_224650	c.-390C>G	5_prime_UTR_premature_start_codon_gain_variant	mRNA capping enzyme, beta chain protein
TGME49_221190	p.Pro111Ala	missense_variant	mrna cleavage factor family protein, putative
TGME49_318590	p.Thr519Lys	missense_variant	MRP family domain-containing protein
TGME49_318590	p.Asp461His	missense_variant	MRP family domain-containing protein
TGME49_318590	p.Gly36Ala	missense_variant	MRP family domain-containing protein
TGME49_300250	p.Pro38Thr	missense_variant	MtN3/saliva family protein
TGME49_300250	p.Phe165Ser	missense_variant	MtN3/saliva family protein
TGME49_300250	p.Val599Gly	missense_variant	MtN3/saliva family protein
TGME49_218840	p.Ala27Thr	missense_variant	mutS domain protein
TGME49_218840	p.Leu73Pro	missense_variant	mutS domain protein
TGME49_218840	p.Gly109Ser	missense_variant	mutS domain protein
TGME49_218840	p.Trp129Arg	missense_variant	mutS domain protein
TGME49_218840	p.Ser201Ala	missense_variant	mutS domain protein
TGME49_218840	p.Pro635Ser	missense_variant	mutS domain protein
TGME49_218840	p.Val726Ile	missense_variant	mutS domain protein
TGME49_218840	p.Pro785Ser	missense_variant	mutS domain protein
TGME49_218840	p.Glu1085Ala	missense_variant	mutS domain protein
TGME49_218840	c.*352T>C	splice_region_variant	mutS domain protein
TGME49_235470	c.-453C>A	5_prime_UTR_premature_start_codon_gain_variant	myosin A
TGME49_239560	p.Val815Met	missense_variant	myosin E
TGME49_278870	c.2934-6G>A	splice_region_variant	myosin F
TGME49_254850	p.Asp23Asn	missense_variant	myosin heavy chain, putative
TGME49_254850	p.Glu47Lys	missense_variant	myosin heavy chain, putative
TGME49_297470	c.-834C>T	5_prime_UTR_premature_start_codon_gain_variant	myosin light chain 2, putative
TGME49_278518	p.Pro830Thr	missense_variant	N-acetylgalactosaminyl transferase
TGME49_318510	c.1110+6C>T	splice_region_variant	N-ethylmaleimide-sensitive fusion protein, putative
TGME49_246080	p.Arg606Cys	missense_variant	NAD dependent epimerase/dehydratase family protein
TGME49_246080	p.Ala466Val	missense_variant	NAD dependent epimerase/dehydratase family protein
TGME49_301210	p.Ser25Arg	missense_variant	NAD(P) transhydrogenase subunit beta, putative
TGME49_301210	p.Thr448Ala	missense_variant	NAD(P) transhydrogenase subunit beta, putative

TGME49_219130	c.1578+7G>A	splice_region_variant	NADPH-glutathione reductase
TGME49_219130	p.Glu165Asp	missense_variant	NADPH-glutathione reductase
TGME49_219130	p.His51Asn	missense_variant	NADPH-glutathione reductase
TGME49_319700	c.89+6T>G	splice_region_variant	NEK kinase
TGME49_319700	p.Tyr289Cys	missense_variant	NEK kinase
TGME49_218400	p.Ala2642Val	missense_variant	NEK kinase
TGME49_218400	p.Thr2494Ala	missense_variant	NEK kinase
TGME49_218400	p.Val2479Ala	missense_variant	NEK kinase
TGME49_218400	p.Pro2205Ser	missense_variant	NEK kinase
TGME49_218400	p.Glu1830Gly	missense_variant	NEK kinase
TGME49_218400	p.Glu1778Gly	missense_variant	NEK kinase
TGME49_218400	p.Ser1731Arg	missense_variant	NEK kinase
TGME49_218400	p.Arg1631Lys	missense_variant	NEK kinase
TGME49_218400	p.Ser1331Pro	missense_variant	NEK kinase
TGME49_218400	p.Thr1158Ala	missense_variant	NEK kinase
TGME49_218400	p.Val410Ala	missense_variant	NEK kinase
TGME49_221922	p.Gln438His	missense_variant	NifU family domain-containing protein
TGME49_221922	p.Glu474Asp	missense_variant	NifU family domain-containing protein
TGME49_218570	p.Ala29Pro	missense_variant	Nin one binding (NOB1) Zn-ribbon family protein
TGME49_218570	p.Pro8Ala	missense_variant	Nin one binding (NOB1) Zn-ribbon family protein
TGME49_218570	c.-190G>T	5_prime_UTR_premature_start_codon_gain_variant	Nin one binding (NOB1) Zn-ribbon family protein
TGME49_249770	p.His148Asp	missense_variant	Nmda1 protein
TGME49_222340	p.Gly165Arg	missense_variant	NOL1/NOP2/sun family protein
TGME49_222340	p.Thr170Ala	missense_variant	NOL1/NOP2/sun family protein
TGME49_222340	p.Ser230Ala	missense_variant	NOL1/NOP2/sun family protein
TGME49_222340	p.Cys341Gly	missense_variant	NOL1/NOP2/sun family protein
TGME49_222340	p.Pro384Ser	missense_variant	NOL1/NOP2/sun family protein
TGME49_222340	p.Leu599Ser	missense_variant	NOL1/NOP2/sun family protein
TGME49_219190	c.-63C>T	5_prime_UTR_premature_start_codon_gain_variant	nuclear movement protein domain containing protein
TGME49_238050	p.Val915Met	missense_variant	nuclease and tudor domain-containing protein
TGME49_300230	c.-146C>G	5_prime_UTR_premature_start_codon_gain_variant	nuclease, putative
TGME49_248677	p.Ala144Thr	missense_variant	nucleic acid binding protein, putative
TGME49_247470	p.Asp240Ala	missense_variant	nucleolar protein, putative
TGME49_248980	c.-290C>G	5_prime_UTR_premature_start_codon_gain_variant	NudC family protein
TGME49_248980	p.Ala110Ser	missense_variant	NudC family protein
TGME49_248980	p.Ser111Pro	missense_variant	NudC family protein
TGME49_248980	p.Val131Ala	missense_variant	NudC family protein
TGME49_278910	p.Arg162Gly	missense_variant	O-acetylserine (thiol) lyase 2, putative
TGME49_210950	p.Ala674Val	missense_variant	oocyst wall protein
TGME49_210950	p.Gly10Asp	missense_variant	oocyst wall protein
TGME49_210950	p.Leu4Arg	missense_variant	oocyst wall protein
TGME49_254050	c.-632T>G	5_prime_UTR_premature_start_codon_gain_variant	optic atrophy 3 protein (opa3) protein
TGME49_254050	c.-817C>T	5_prime_UTR_premature_start_codon_gain_variant	optic atrophy 3 protein (opa3) protein
TGME49_254050	c.-1025A>G	5_prime_UTR_premature_start_codon_gain_variant	optic atrophy 3 protein (opa3) protein
TGME49_302005	p.Gly22Glu	missense_variant	ORF F
TGME49_245570	p.His269Asn	missense_variant	origin recognition complex subunit 2 protein
TGME49_245570	p.Pro205Ser	missense_variant	origin recognition complex subunit 2 protein
TGME49_245570	p.Leu68Arg	missense_variant	origin recognition complex subunit 2 protein
TGME49_277990	p.Val243Ala	missense_variant	OTU family cysteine protease
TGME49_277990	p.Glu118Ala	missense_variant	OTU family cysteine protease
TGME49_277990	p.Glu118Lys	missense_variant	OTU family cysteine protease
TGME49_277990	c.-609G>C	5_prime_UTR_premature_start_codon_gain_variant	OTU family cysteine protease
TGME49_221270	p.Asp104Glu	missense_variant	oxidoreductase, 2OG-Fe(II) oxygenase family protein
TGME49_221270	p.Pro70Leu	missense_variant	oxidoreductase, 2OG-Fe(II) oxygenase family protein
TGME49_253960	p.Glu322Lys	missense_variant	oxidoreductase, short chain dehydrogenase/reductase family protein
TGME49_253960	p.His80Asp	missense_variant&splice_region_variant	oxidoreductase, short chain dehydrogenase/reductase family protein
TGME49_253960	p.Gly20Arg	missense_variant	oxidoreductase, short chain dehydrogenase/reductase family protein
TGME49_318460	c.2088+6G>C	splice_region_variant	P-type ATPase of unknown pump specificity (type V) protein
TGME49_252640	p.Glu487Asp	missense_variant	P-type ATPase PMA1
TGME49_278660	p.Glu1181Gln	missense_variant	P-type ATPase4, putative
TGME49_235183	p.Tyr1387Phe	missense_variant	PAN domain-containing protein
TGME49_235200	p.Ala1921Thr	missense_variant	PAN domain-containing protein
TGME49_249150	p.Lys280Asn	missense_variant	PAN domain-containing protein
TGME49_249150	p.Ile653Met	missense_variant	PAN domain-containing protein
TGME49_249150	p.Glu967Lys	missense_variant	PAN domain-containing protein
TGME49_249150	p.Ile997Thr	missense_variant	PAN domain-containing protein
TGME49_249150	p.Val1030Leu	missense_variant	PAN domain-containing protein
TGME49_235478	p.Ala1517Thr	missense_variant	pantothenate kinase
TGME49_235478	p.Leu1480His	missense_variant	pantothenate kinase
TGME49_235478	p.Glu975Gly	missense_variant	pantothenate kinase
TGME49_247360	c.-276G>T	5_prime_UTR_premature_start_codon_gain_variant	PAP2 superfamily protein
TGME49_253900	p.His59Gln	missense_variant	parasite porphobilinogen synthase PBGS
TGME49_249710	c.-999G>A	5_prime_UTR_premature_start_codon_gain_variant	Parkinson disease 7 domain containing 1 family protein
TGME49_249710	p.His387Arg	missense_variant	Parkinson disease 7 domain containing 1 family protein
TGME49_249710	p.Gly544Arg	missense_variant	Parkinson disease 7 domain containing 1 family protein
TGME49_249710	p.Val577Ala	missense_variant	Parkinson disease 7 domain containing 1 family protein
TGME49_236220	p.Thr337Ala	missense_variant	PCI domain-containing protein
TGME49_254450	p.Ala332Ser	missense_variant	Pelota, putative
TGME49_252210	c.-281C>G	5_prime_UTR_premature_start_codon_gain_variant	pentatricopeptide repeat domain-containing protein
TGME49_250760	p.Thr421Ala	missense_variant	pentatricopeptide repeat domain-containing protein
TGME49_250760	p.Leu449Val	missense_variant	pentatricopeptide repeat domain-containing protein
TGME49_250760	p.Asn584Lys	missense_variant	pentatricopeptide repeat domain-containing protein
TGME49_250760	p.Ser615Tyr	missense_variant	pentatricopeptide repeat domain-containing protein
TGME49_250760	p.Gly917Arg	missense_variant	pentatricopeptide repeat domain-containing protein
TGME49_250760	p.Lys1064Arg	missense_variant	pentatricopeptide repeat domain-containing protein

TGME49_250760	p.Ala1899Val	missense_variant	pentatricopeptide repeat domain-containing protein
TGME49_252440	p.Ile490Ser	missense_variant	peptidase c13 family protein
TGME49_253890	p.Pro909Ser	missense_variant	peptidase M16 inactive domain-containing protein
TGME49_253890	p.Asn829Thr	missense_variant	peptidase M16 inactive domain-containing protein
TGME49_253890	p.Lys818Glu	missense_variant	peptidase M16 inactive domain-containing protein
TGME49_253890	p.Gln749His	missense_variant	peptidase M16 inactive domain-containing protein
TGME49_253890	p.Pro401Ser	missense_variant	peptidase M16 inactive domain-containing protein
TGME49_253890	p.Phe86Leu	missense_variant	peptidase M16 inactive domain-containing protein
TGME49_253890	p.Arg82Leu	missense_variant	peptidase M16 inactive domain-containing protein
TGME49_235680	p.Gly408Arg	missense_variant	peptidase M16 inactive domain-containing protein
TGME49_235680	p.Pro355Ser	missense_variant	peptidase M16 inactive domain-containing protein
TGME49_235680	p.Glu269Asp	missense_variant	peptidase M16 inactive domain-containing protein
TGME49_235680	p.Gly109Glu	missense_variant	peptidase M16 inactive domain-containing protein
TGME49_235680	c.-916A>G	5_prime_UTR_premature_start_codon_gain_variant	peptidase M16 inactive domain-containing protein
TGME49_214490	p.Ser783Phe	missense_variant	peptidase M16 inactive domain-containing protein
TGME49_318275	c.-355T>G	5_prime_UTR_premature_start_codon_gain_variant	peptidyl-prolyl cis-trans isomerase, FKBP-type domain-containing protein
TGME49_238000	p.Asp47Asn	missense_variant	peptidyl-prolyl isomerase
TGME49_219240	p.Met250Leu	missense_variant	Peptidyl-tRNA hydrolase PTH2 domain-containing protein
TGME49_219240	c.-279C>T	5_prime_UTR_premature_start_codon_gain_variant	Peptidyl-tRNA hydrolase PTH2 domain-containing protein
TGME49_219240	c.-1843G>T	5_prime_UTR_premature_start_codon_gain_variant	Peptidyl-tRNA hydrolase PTH2 domain-containing protein
TGME49_211240	c.-182C>T	5_prime_UTR_premature_start_codon_gain_variant	peptidyl-tRNA hydrolase, putative
TGME49_211240	c.29-7C>T	splice_region_variant	peptidyl-tRNA hydrolase, putative
TGME49_248225	c.1111-8G>T	splice_region_variant	PF2 arrest specific protein 8/11, putative
TGME49_248225	c.1111-4G>C	splice_region_variant	PF2 arrest specific protein 8/11, putative
TGME49_248225	p.Glu414Asp	missense_variant	PF2 arrest specific protein 8/11, putative
TGME49_224260	p.Gly5379Asp	missense_variant	PHD-finger domain-containing protein
TGME49_224260	p.Ser4580Gly	missense_variant	PHD-finger domain-containing protein
TGME49_224260	p.Pro4205Leu	missense_variant	PHD-finger domain-containing protein
TGME49_224260	p.Val3748Gly	missense_variant	PHD-finger domain-containing protein
TGME49_224260	p.Val3724Ile	missense_variant	PHD-finger domain-containing protein
TGME49_224260	p.Pro3690Gln	missense_variant	PHD-finger domain-containing protein
TGME49_224260	p.Lys3651Thr	missense_variant	PHD-finger domain-containing protein
TGME49_224260	p.Ala3643Pro	missense_variant	PHD-finger domain-containing protein
TGME49_224260	p.Ser3573Leu	missense_variant	PHD-finger domain-containing protein
TGME49_224260	p.Lys3515Glu	missense_variant	PHD-finger domain-containing protein
TGME49_224260	p.Pro3414Gln	missense_variant	PHD-finger domain-containing protein
TGME49_224260	p.Gly3366Asp	missense_variant	PHD-finger domain-containing protein
TGME49_224260	p.Ala3345Thr	missense_variant	PHD-finger domain-containing protein
TGME49_224260	p.Thr3273Met	missense_variant	PHD-finger domain-containing protein
TGME49_224260	p.Asp2222Asn	missense_variant	PHD-finger domain-containing protein
TGME49_224260	p.Pro2188Ser	missense_variant	PHD-finger domain-containing protein
TGME49_224260	p.Glu2185Gly	missense_variant	PHD-finger domain-containing protein
TGME49_224260	p.Glu2161Asp	missense_variant	PHD-finger domain-containing protein
TGME49_224260	p.Asp1479Gly	missense_variant	PHD-finger domain-containing protein
TGME49_224260	p.Leu1376Arg	missense_variant	PHD-finger domain-containing protein
TGME49_224260	p.Gly1352Asp	missense_variant	PHD-finger domain-containing protein
TGME49_224260	p.Ala615Thr	missense_variant	PHD-finger domain-containing protein
TGME49_224260	p.Ile148Val	missense_variant	PHD-finger domain-containing protein
TGME49_234900	c.-1337C>T	5_prime_UTR_premature_start_codon_gain_variant	PHD-finger domain-containing protein
TGME49_234900	c.-723C>G	5_prime_UTR_premature_start_codon_gain_variant	PHD-finger domain-containing protein
TGME49_234900	p.Glu628Ala	missense_variant	PHD-finger domain-containing protein
TGME49_234900	p.Lys693Arg	missense_variant	PHD-finger domain-containing protein
TGME49_234900	p.Arg1089His	missense_variant	PHD-finger domain-containing protein
TGME49_234900	p.Val1889Gly	missense_variant	PHD-finger domain-containing protein
TGME49_234900	p.Glu2299Asp	missense_variant	PHD-finger domain-containing protein
TGME49_234900	p.Ala2306Val	missense_variant	PHD-finger domain-containing protein
TGME49_234900	p.Leu2327Val	missense_variant	PHD-finger domain-containing protein
TGME49_235550	c.-323G>A	5_prime_UTR_premature_start_codon_gain_variant	PHD-finger domain-containing protein
TGME49_235550	p.Val33Ala	missense_variant	PHD-finger domain-containing protein
TGME49_235550	p.Gly145Ser	missense_variant	PHD-finger domain-containing protein
TGME49_246100	c.-502C>T	5_prime_UTR_premature_start_codon_gain_variant	phosducin, putative
TGME49_246100	c.-107T>G	5_prime_UTR_premature_start_codon_gain_variant	phosducin, putative
TGME49_278990	p.Gln30His	missense_variant	phosphate carrier, putative
TGME49_240210	c.-1023T>C	5_prime_UTR_premature_start_codon_gain_variant	phosphate transporter family protein, putative
TGME49_254365	p.Arg39His	missense_variant	phosphatidate cytidylyltransferase
TGME49_215700	p.Val2201Leu	missense_variant	phosphatidylinositol 3- and 4-kinase
TGME49_215700	p.Val2190Ile	missense_variant	phosphatidylinositol 3- and 4-kinase
TGME49_215700	p.Asn1415Tyr	missense_variant	phosphatidylinositol 3- and 4-kinase
TGME49_215700	p.Val1088Leu	missense_variant	phosphatidylinositol 3- and 4-kinase
TGME49_215700	p.Ser648Pro	missense_variant	phosphatidylinositol 3- and 4-kinase
TGME49_215700	p.Ala548Thr	missense_variant	phosphatidylinositol 3- and 4-kinase
TGME49_215700	p.Ser487Cys	missense_variant	phosphatidylinositol 3- and 4-kinase
TGME49_215700	p.Ser486Trp	missense_variant	phosphatidylinositol 3- and 4-kinase
TGME49_215700	p.Pro69Ser	missense_variant	phosphatidylinositol 3- and 4-kinase
TGME49_299040	c.-273C>T	5_prime_UTR_premature_start_codon_gain_variant	phosphatidylinositol n-acetylglucosaminyltransferase
TGME49_299040	c.-684C>A	5_prime_UTR_premature_start_codon_gain_variant	phosphatidylinositol n-acetylglucosaminyltransferase
TGME49_299040	c.-999T>G	5_prime_UTR_premature_start_codon_gain_variant	phosphatidylinositol n-acetylglucosaminyltransferase
TGME49_245730	p.Ser187Tyr	missense_variant	phosphatidylinositol-4-phosphate 5-Kinase
TGME49_245730	p.Gly195Asp	missense_variant	phosphatidylinositol-4-phosphate 5-Kinase
TGME49_245730	p.Ser695Tyr	missense_variant	phosphatidylinositol-4-phosphate 5-Kinase
TGME49_245730	p.Pro1652Leu	missense_variant	phosphatidylinositol-4-phosphate 5-Kinase
TGME49_245730	c.5786+8T>C	splice_region_variant	phosphatidylinositol-4-phosphate 5-Kinase
TGME49_245730	p.Asp2081Val	missense_variant	phosphatidylinositol-4-phosphate 5-Kinase
TGME49_214280	c.-666C>T	5_prime_UTR_premature_start_codon_gain_variant	phosphoadenosine phosphosulfate reductase family protein
TGME49_214280	p.Ser250Arg	missense_variant	phosphoadenosine phosphosulfate reductase family protein

TGME49_214280	p.Ser488Leu	missense_variant	phosphoadenosine phosphosulfate reductase family protein
TGME49_214280	p.Val504Asp	missense_variant	phosphoadenosine phosphosulfate reductase family protein
TGME49_222020	p.Phe28Val	missense_variant	phosphoglycerate kinase PGKII
TGME49_222020	p.Val426Ile	missense_variant	phosphoglycerate kinase PGKII
TGME49_221460	c.94-6C>G	splice_region_variant	phosphoglycerate mutase family protein
TGME49_318190	c.5461-4G>T	splice_region_variant	phosphoglycerate mutase family protein
TGME49_318190	p.Ala1604Thr	missense_variant	phosphoglycerate mutase family protein
TGME49_318190	p.Ala1389Thr	missense_variant	phosphoglycerate mutase family protein
TGME49_318190	p.Thr1161Ser	missense_variant	phosphoglycerate mutase family protein
TGME49_318190	p.Ser920Pro	missense_variant	phosphoglycerate mutase family protein
TGME49_318190	p.Leu878Val	missense_variant	phosphoglycerate mutase family protein
TGME49_318190	p.Gly701Asp	missense_variant	phosphoglycerate mutase family protein
TGME49_222952	p.Arg101Cys	missense_variant	phosphohistidine phosphatase
TGME49_248830	c.-256C>T	5_prime_UTR_premature_start_codon_gain_variant	phosphoinositide phospholipase PIPLC
TGME49_246530	p.Glu355Gly	missense_variant	phospholipase D active site domain-containing protein
TGME49_246530	p.Ala405Thr	missense_variant	phospholipase D active site domain-containing protein
TGME49_246530	p.Ala593Val	missense_variant	phospholipase D active site domain-containing protein
TGME49_254420	p.Pro5Leu	missense_variant	phospholipase, patatin family protein
TGME49_254420	p.Ala40Ser	missense_variant	phospholipase, patatin family protein
TGME49_254420	p.Asn60Ser	missense_variant	phospholipase, patatin family protein
TGME49_254420	p.Ala108Thr	missense_variant	phospholipase, patatin family protein
TGME49_254420	p.Ser180Asn	missense_variant	phospholipase, patatin family protein
TGME49_254420	p.Asn293Thr	missense_variant	phospholipase, patatin family protein
TGME49_254420	p.Ala372Glu	missense_variant	phospholipase, patatin family protein
TGME49_254420	p.Gly505Cys	missense_variant	phospholipase, patatin family protein
TGME49_254420	p.Lys655Met	missense_variant	phospholipase, patatin family protein
TGME49_254420	p.Leu663Val	missense_variant	phospholipase, patatin family protein
TGME49_212130	p.Pro308Ser	missense_variant	phospholipase, patatin family protein
TGME49_212130	p.Arg436His	missense_variant	phospholipase, patatin family protein
TGME49_245510	c.4699-5G>T	splice_region_variant	phospholipid-translocating P-type ATPase, flippase subfamily protein
TGME49_245510	p.His953Arg	missense_variant	phospholipid-translocating P-type ATPase, flippase subfamily protein
TGME49_245510	p.Asn807Ser	missense_variant	phospholipid-translocating P-type ATPase, flippase subfamily protein
TGME49_245510	p.Asp670Asn	missense_variant	phospholipid-translocating P-type ATPase, flippase subfamily protein
TGME49_247690	c.441+7A>C	splice_region_variant	phospholipid-translocating P-type ATPase, flippase subfamily protein
TGME49_247690	p.Ser290Leu	missense_variant	phospholipid-translocating P-type ATPase, flippase subfamily protein
TGME49_247690	p.Arg561Gln	missense_variant	phospholipid-translocating P-type ATPase, flippase subfamily protein
TGME49_247690	p.Asp597Val	missense_variant	phospholipid-translocating P-type ATPase, flippase subfamily protein
TGME49_247690	p.Ala654Thr	missense_variant	phospholipid-translocating P-type ATPase, flippase subfamily protein
TGME49_247690	p.Lys829Met	missense_variant	phospholipid-translocating P-type ATPase, flippase subfamily protein
TGME49_239710	p.Lys9Gln	missense_variant	phosphomannomutase
TGME49_235000	p.Met284Val	missense_variant	phosphorylase family protein
TGME49_235000	c.-176A>G	5_prime_UTR_premature_start_codon_gain_variant	phosphorylase family protein
TGME49_222900	p.Ala226Val	missense_variant	phosphoserine phosphatase
TGME49_222900	p.Arg113His	missense_variant	phosphoserine phosphatase
TGME49_253750	p.Ala5805Thr	missense_variant	PLU-1 family protein
TGME49_253750	p.Thr5696Asn	missense_variant	PLU-1 family protein
TGME49_253750	p.His5694Gln	missense_variant	PLU-1 family protein
TGME49_253750	p.Ala5318Ser	missense_variant	PLU-1 family protein
TGME49_253750	p.Gly5268Val	missense_variant	PLU-1 family protein
TGME49_253750	p.Leu4900Ser	missense_variant	PLU-1 family protein
TGME49_253750	p.Ala4084Thr	missense_variant	PLU-1 family protein
TGME49_253750	p.Ala4061Thr	missense_variant	PLU-1 family protein
TGME49_253750	p.Val3968Leu	missense_variant	PLU-1 family protein
TGME49_253750	p.Pro3676Leu	missense_variant	PLU-1 family protein
TGME49_253750	p.Asp3286Gly	missense_variant	PLU-1 family protein
TGME49_253750	p.Glu2796Asp	missense_variant	PLU-1 family protein
TGME49_253750	p.Ala1687Val	missense_variant	PLU-1 family protein
TGME49_253750	p.Ser1667Pro	missense_variant	PLU-1 family protein
TGME49_224850	c.-1230C>T	5_prime_UTR_premature_start_codon_gain_variant	polyadenylate binding protein, putative
TGME49_224850	p.Met73Val	missense_variant	polyadenylate binding protein, putative
TGME49_278260	p.Ala1861Glu	missense_variant	polynucleotide adenylyltransferase
TGME49_278260	p.Phe1748Leu	missense_variant	polynucleotide adenylyltransferase
TGME49_278260	p.Pro1412Leu	missense_variant	polynucleotide adenylyltransferase
TGME49_278260	c.1535+5T>G	splice_region_variant	polynucleotide adenylyltransferase
TGME49_278260	p.Gln378Lys	missense_variant	polynucleotide adenylyltransferase
TGME49_278260	p.Gly195Arg	missense_variant	polynucleotide adenylyltransferase
TGME49_224490	p.Arg26His	missense_variant	polyprenyl synthetase superfamily protein
TGME49_224490	p.Lys31Asn	missense_variant	polyprenyl synthetase superfamily protein
TGME49_224490	p.Ala257Ser	missense_variant	polyprenyl synthetase superfamily protein
TGME49_254730	p.His1718Arg	missense_variant	POPLD (NUC188) domain-containing protein
TGME49_254730	p.Gly1711Asp	missense_variant	POPLD (NUC188) domain-containing protein
TGME49_254730	p.Pro1710Ser	missense_variant	POPLD (NUC188) domain-containing protein
TGME49_215100	p.Gly2144Trp	missense_variant	PP-loop family protein
TGME49_215100	p.Gln2113Glu	missense_variant	PP-loop family protein
TGME49_215100	p.Glu2103Lys	missense_variant	PP-loop family protein
TGME49_215100	p.Asp1841Ala	missense_variant	PP-loop family protein
TGME49_215100	p.Pro1548His	missense_variant	PP-loop family protein
TGME49_215100	p.Asp1100Ala	missense_variant	PP-loop family protein
TGME49_215100	p.Arg849Gly	missense_variant	PP-loop family protein
TGME49_215100	p.Gly271Asp	missense_variant	PP-loop family protein
TGME49_318660	p.Ser201Arg	missense_variant	PP2C, putative
TGME49_318660	c.-721C>A	5_prime_UTR_premature_start_codon_gain_variant	PP2C, putative
TGME49_221570	p.Gly311Ala	missense_variant	pre-rRNA-processing protein, putative
TGME49_248370	c.-224C>A	5_prime_UTR_premature_start_codon_gain_variant	prefoldin subunit 6, putative
TGME49_254250	p.Thr397Lys	missense_variant	PRELI family protein

TGME49_299180	c.-281C>G	5_prime_UTR_premature_start_codon_gain_variant	prenylated protein, putative
TGME49_299180	c.-951C>G	5_prime_UTR_premature_start_codon_gain_variant	prenylated protein, putative
TGME49_236010	p.Arg248His	missense_variant	prenylcysteine oxidase
TGME49_236010	p.Ala168Thr	missense_variant	prenylcysteine oxidase
TGME49_221850	c.-761T>C	5_prime_UTR_premature_start_codon_gain_variant	prohibitin family protein, putative
TGME49_278050	c.-246C>G	5_prime_UTR_premature_start_codon_gain_variant	proteasome subunit alpha type 1, putative
TGME49_218920	c.-219C>T	5_prime_UTR_premature_start_codon_gain_variant	proteasome subunit beta type, putative
TGME49_275410	p.Val2515Met	missense_variant	Proteasome/cyclosome repeat-containing protein
TGME49_249270	c.688+3G>A	splice_region_variant	protein disulfide isomerase-related protein (provisional), putative
TGME49_249270	c.1391-6T>C	splice_region_variant	protein disulfide isomerase-related protein (provisional), putative
TGME49_238040	p.Glu875Asp	missense_variant	protein disulfide-isomerase domain-containing protein
TGME49_238040	p.Ser221Leu	missense_variant	protein disulfide-isomerase domain-containing protein
TGME49_218470	p.Gly454Ser	missense_variant	protein disulfide-isomerase, putative
TGME49_253950	p.Ala169Thr	missense_variant	protein fam50a, putative
TGME49_239420	p.Gly1717Ser	missense_variant	protein kinase
TGME49_239420	p.Ala1712Thr	missense_variant	protein kinase
TGME49_239420	p.Val1272Ala	missense_variant	protein kinase
TGME49_239420	p.His969Pro	missense_variant	protein kinase
TGME49_239420	p.Pro801Gln	missense_variant	protein kinase
TGME49_239420	c.1569-4T>C	splice_region_variant	protein kinase
TGME49_239420	c.-4048G>T	5_prime_UTR_premature_start_codon_gain_variant	protein kinase
TGME49_239440	p.Ser220Thr	missense_variant	protein kinase (incomplete catalytic triad)
TGME49_239440	p.Ala480Val	missense_variant	protein kinase (incomplete catalytic triad)
TGME49_239440	p.Arg713Ser	missense_variant	protein kinase (incomplete catalytic triad)
TGME49_239440	p.Ala1054Ser	missense_variant	protein kinase (incomplete catalytic triad)
TGME49_234950	p.Leu65Arg	missense_variant	protein kinase (incomplete catalytic triad)
TGME49_236620	c.664-4C>G	splice_region_variant	protein kinase (incomplete catalytic triad)
TGME49_237860	p.Ser180Phe	missense_variant	protein kinase domain-containing protein
TGME49_237860	p.Ser501Ala	missense_variant	protein kinase domain-containing protein
TGME49_237860	p.Gly957Arg	missense_variant	protein kinase domain-containing protein
TGME49_237860	p.Met1652Ile	missense_variant	protein kinase domain-containing protein
TGME49_237860	p.Asp1674Asn	missense_variant	protein kinase domain-containing protein
TGME49_237860	p.Asp1932His	missense_variant	protein kinase domain-containing protein
TGME49_237860	p.Pro1933Ser	missense_variant	protein kinase domain-containing protein
TGME49_275610	c.-560A>T	5_prime_UTR_premature_start_codon_gain_variant	protein kinase, other
TGME49_278900	p.Glu308Lys	missense_variant	protein kinase, putative
TGME49_246510	p.Val126Leu	missense_variant	protein phosphatase 2a regulatory b subunit (b56 family) protein
TGME49_224240	c.-164C>T	5_prime_UTR_premature_start_codon_gain_variant	protein phosphatase 2C domain-containing protein
TGME49_224240	p.Gln57Arg	missense_variant	protein phosphatase 2C domain-containing protein
TGME49_224240	p.Ala110Pro	missense_variant	protein phosphatase 2C domain-containing protein
TGME49_224240	p.Ile250Met	missense_variant	protein phosphatase 2C domain-containing protein
TGME49_224240	p.His270Gln	missense_variant	protein phosphatase 2C domain-containing protein
TGME49_224240	p.Leu381Phe	missense_variant	protein phosphatase 2C domain-containing protein
TGME49_224240	p.Arg439His	missense_variant	protein phosphatase 2C domain-containing protein
TGME49_224240	p.Ser529Ala	missense_variant	protein phosphatase 2C domain-containing protein
TGME49_224240	p.Ala666Thr	missense_variant	protein phosphatase 2C domain-containing protein
TGME49_224240	p.Ala1092Thr	missense_variant	protein phosphatase 2C domain-containing protein
TGME49_236260	p.Glu207Gln	missense_variant	protein phosphatase 2C domain-containing protein
TGME49_237410	p.Gly1039Arg	missense_variant	protein phosphatase 2C domain-containing protein
TGME49_237410	p.Thr1038Ser	missense_variant	protein phosphatase 2C domain-containing protein
TGME49_237410	p.Arg930Gly	missense_variant	protein phosphatase 2C domain-containing protein
TGME49_237410	p.Gln637Pro	missense_variant	protein phosphatase 2C domain-containing protein
TGME49_237410	p.Leu141Ile	missense_variant	protein phosphatase 2C domain-containing protein
TGME49_237410	p.Ser68Ala	missense_variant	protein phosphatase 2C domain-containing protein
TGME49_237410	c.-1265C>G	5_prime_UTR_premature_start_codon_gain_variant	protein phosphatase 2C domain-containing protein
TGME49_237410	c.-1772C>G	5_prime_UTR_premature_start_codon_gain_variant	protein phosphatase 2C domain-containing protein
TGME49_237410	c.-2526A>G	5_prime_UTR_premature_start_codon_gain_variant	protein phosphatase 2C domain-containing protein
TGME49_237410	c.-3063G>T	5_prime_UTR_premature_start_codon_gain_variant	protein phosphatase 2C domain-containing protein
TGME49_237500	p.Gly168Arg	missense_variant	protein phosphatase 2C domain-containing protein
TGME49_237500	p.Glu230Asp	missense_variant	protein phosphatase 2C domain-containing protein
TGME49_237500	p.Gln232His	missense_variant	protein phosphatase 2C domain-containing protein
TGME49_237500	p.Ala250Val	missense_variant	protein phosphatase 2C domain-containing protein
TGME49_237500	p.Val278Ile	missense_variant	protein phosphatase 2C domain-containing protein
TGME49_237500	p.Asp415Ala	missense_variant	protein phosphatase 2C domain-containing protein
TGME49_237500	p.Ser495Leu	missense_variant	protein phosphatase 2C domain-containing protein
TGME49_237500	p.His738Arg	missense_variant	protein phosphatase 2C domain-containing protein
TGME49_218590	p.Arg429Gln	missense_variant	protein phosphatase 2C domain-containing protein
TGME49_218590	p.Thr244Ile	missense_variant	protein phosphatase 2C domain-containing protein
TGME49_254410	p.Ser384Ala	missense_variant	protein phosphatase 2C, putative
TGME49_254410	p.Arg286Gly	missense_variant	protein phosphatase 2C, putative
TGME49_214110	p.Leu268Phe	missense_variant	protein yipf5, putative
TGME49_297520	p.Ser1499Pro	missense_variant	proteophosphoglycan PPG1
TGME49_297520	c.4132+6C>T	splice_region_variant	proteophosphoglycan PPG1
TGME49_297520	p.Ala1300Ser	missense_variant	proteophosphoglycan PPG1
TGME49_297520	p.Leu814Val	missense_variant	proteophosphoglycan PPG1
TGME49_297520	p.Glu268Asp	missense_variant	proteophosphoglycan PPG1
TGME49_236580	c.390+4C>A	splice_region_variant	Prp31-15.5k-U4 Snrna Complex family protein
TGME49_222110	p.Ala281Ser	missense_variant	PUB domain-containing protein
TGME49_222110	p.Ala281Val	missense_variant	PUB domain-containing protein
TGME49_318350	p.His1578Tyr	missense_variant	Pumilio-family RNA binding repeat-containing protein
TGME49_318350	p.Thr836Ser	missense_variant	Pumilio-family RNA binding repeat-containing protein
TGME49_318350	p.Ala226Val	missense_variant	Pumilio-family RNA binding repeat-containing protein
TGME49_318350	p.Ser225Gly	missense_variant	Pumilio-family RNA binding repeat-containing protein
TGME49_318720	p.Ala502Val	missense_variant	pyridoxal phosphate enzyme, YggS family protein
TGME49_318720	p.Asp474Tyr	missense_variant	pyridoxal phosphate enzyme, YggS family protein

TGME49_318720	p.Ser389Tyr	missense_variant	pyridoxal phosphate enzyme, YggS family protein
TGME49_318720	c.958+7G>T	splice_region_variant	pyridoxal phosphate enzyme, YggS family protein
TGME49_318720	c.958+4A>G	splice_region_variant	pyridoxal phosphate enzyme, YggS family protein
TGME49_318720	c.-511A>G	5_prime_UTR_premature_start_codon_gain_variant	pyridoxal phosphate enzyme, YggS family protein
TGME49_245670	p.Thr564Ser	missense_variant	pyruvate dehydrogenase complex subunit PDH-E1Alpha
TGME49_299070	p.Thr65Ser	missense_variant	pyruvate kinase PyKII
TGME49_299070	p.Ala510Val	missense_variant	pyruvate kinase PyKII
TGME49_299070	p.Arg605Ser	missense_variant	pyruvate kinase PyKII
TGME49_299070	p.Cys764Gly	missense_variant	pyruvate kinase PyKII
TGME49_252465	p.Lys334Thr	missense_variant	radical SAM domain-containing protein
TGME49_252465	p.Leu146His	missense_variant	radical SAM domain-containing protein
TGME49_252465	p.Ser120Thr	missense_variant	radical SAM domain-containing protein
TGME49_219300	p.Leu230Val	missense_variant	ran binding protein
TGME49_222040	c.-548C>T	5_prime_UTR_premature_start_codon_gain_variant	Ran-interacting Mog1 protein
TGME49_222040	c.-277A>T	5_prime_UTR_premature_start_codon_gain_variant	Ran-interacting Mog1 protein
TGME49_222040	c.212+6C>T	splice_region_variant	Ran-interacting Mog1 protein
TGME49_239350	p.Asp2228Glu	missense_variant	RAP domain-containing protein
TGME49_239350	p.Val2226Gly	missense_variant	RAP domain-containing protein
TGME49_239350	p.Gly2218Ser	missense_variant	RAP domain-containing protein
TGME49_239350	p.Asn2150Asp	missense_variant	RAP domain-containing protein
TGME49_239350	p.Glu2093Asp	missense_variant	RAP domain-containing protein
TGME49_239350	p.Ala1989Ser	missense_variant	RAP domain-containing protein
TGME49_239350	p.Ser1966Pro	missense_variant	RAP domain-containing protein
TGME49_239350	p.Asp1456Asn	missense_variant	RAP domain-containing protein
TGME49_239350	p.Ser1090Pro	missense_variant	RAP domain-containing protein
TGME49_239350	p.Leu443Gln	missense_variant	RAP domain-containing protein
TGME49_239350	p.Pro389Ser	missense_variant	RAP domain-containing protein
TGME49_239350	c.944-5C>A	splice_region_variant	RAP domain-containing protein
TGME49_239350	p.Pro255Ser	missense_variant	RAP domain-containing protein
TGME49_239350	p.Asp110Asn	missense_variant	RAP domain-containing protein
TGME49_239350	p.Leu89Phe	missense_variant	RAP domain-containing protein
TGME49_211890	p.Phe5229Ser	missense_variant	RAP domain-containing protein
TGME49_211890	p.Gly5134Val	missense_variant	RAP domain-containing protein
TGME49_211890	p.Ser4966Ile	missense_variant	RAP domain-containing protein
TGME49_211890	p.Arg4466Ile	missense_variant	RAP domain-containing protein
TGME49_211890	p.Val3215Met	missense_variant	RAP domain-containing protein
TGME49_211890	p.Ala2673Thr	missense_variant	RAP domain-containing protein
TGME49_211890	p.Tyr1626His	missense_variant	RAP domain-containing protein
TGME49_211890	p.Trp174Leu	missense_variant	RAP domain-containing protein
TGME49_211890	p.Ser137Asn	missense_variant	RAP domain-containing protein
TGME49_211890	p.Cys52Gly	missense_variant	RAP domain-containing protein
TGME49_237100	p.Arg1412Lys	missense_variant	RAP domain-containing protein
TGME49_249170	p.Val332Ala	missense_variant	Ras family protein
TGME49_249170	c.1027-4A>G	splice_region_variant	Ras family protein
TGME49_249170	p.Thr668Ala	missense_variant	Ras family protein
TGME49_249170	p.Ala680Thr	missense_variant	Ras family protein
TGME49_277840	c.664-5C>T	splice_region_variant	Ras family protein
TGME49_277840	c.-328T>C	5_prime_UTR_premature_start_codon_gain_variant	Ras family protein
TGME49_277840	c.-1488C>T	5_prime_UTR_premature_start_codon_gain_variant	Ras family protein
TGME49_219720	c.-251C>G	5_prime_UTR_premature_start_codon_gain_variant	Ras-related protein Rab-5C, putative
TGME49_247250	p.Glu235Asp	missense_variant	RbAp46
TGME49_247250	p.Glu182Asp	missense_variant	RbAp46
TGME49_247250	p.Arg95Leu	missense_variant	RbAp46
TGME49_247250	p.Ser72Pro	missense_variant	RbAp46
TGME49_297800	p.Pro611Ala	missense_variant	RecF/RecN/SMC N terminal domain-containing protein
TGME49_297800	p.Ala435Thr	missense_variant	RecF/RecN/SMC N terminal domain-containing protein
TGME49_297800	c.-213C>G	5_prime_UTR_premature_start_codon_gain_variant	RecF/RecN/SMC N terminal domain-containing protein
TGME49_297800	c.-221T>C	5_prime_UTR_premature_start_codon_gain_variant	RecF/RecN/SMC N terminal domain-containing protein
TGME49_239700	p.Ala35Thr	missense_variant	regulator of chromosome condensation (RCC1) repeat-containing protein
TGME49_239700	p.Thr361Ala	missense_variant	regulator of chromosome condensation (RCC1) repeat-containing protein
TGME49_239700	p.Leu702Pro	missense_variant	regulator of chromosome condensation (RCC1) repeat-containing protein
TGME49_239700	p.Cys1188Ser	missense_variant	regulator of chromosome condensation (RCC1) repeat-containing protein
TGME49_239700	p.Asp1808Asn	missense_variant	regulator of chromosome condensation (RCC1) repeat-containing protein
TGME49_239700	p.Ala2572Pro	missense_variant	regulator of chromosome condensation (RCC1) repeat-containing protein
TGME49_239700	p.Gly2595Arg	missense_variant	regulator of chromosome condensation (RCC1) repeat-containing protein
TGME49_239700	p.Glu2711Asp	missense_variant	regulator of chromosome condensation (RCC1) repeat-containing protein
TGME49_280770	c.682+3G>A	splice_region_variant	regulator of chromosome condensation (RCC1) repeat-containing protein
TGME49_280770	c.863-6C>G	splice_region_variant	regulator of chromosome condensation (RCC1) repeat-containing protein
TGME49_280770	p.Gly608Asp	missense_variant	regulator of chromosome condensation (RCC1) repeat-containing protein
TGME49_280770	p.Lys861Thr	missense_variant	regulator of chromosome condensation (RCC1) repeat-containing protein
TGME49_280770	p.Ala938Thr	missense_variant	regulator of chromosome condensation (RCC1) repeat-containing protein
TGME49_280770	p.Ala1139Thr	missense_variant	regulator of chromosome condensation (RCC1) repeat-containing protein
TGME49_236250	c.-15C>T	5_prime_UTR_premature_start_codon_gain_variant	regulator of chromosome condensation (RCC1) repeat-containing protein
TGME49_236250	p.Ala82Thr	missense_variant	regulator of chromosome condensation (RCC1) repeat-containing protein
TGME49_236250	p.Val438Leu	missense_variant	regulator of chromosome condensation (RCC1) repeat-containing protein
TGME49_236250	p.Arg699Gln	missense_variant	regulator of chromosome condensation (RCC1) repeat-containing protein
TGME49_236250	p.Pro763Ser	missense_variant	regulator of chromosome condensation (RCC1) repeat-containing protein
TGME49_236250	p.Gly773Ser	missense_variant	regulator of chromosome condensation (RCC1) repeat-containing protein
TGME49_236250	p.Pro836Ala	missense_variant	regulator of chromosome condensation (RCC1) repeat-containing protein
TGME49_248640	c.1351-8T>C	splice_region_variant	regulator of chromosome condensation (RCC1) repeat-containing protein
TGME49_248640	p.Ile1532Val	missense_variant	regulator of chromosome condensation (RCC1) repeat-containing protein
TGME49_249000	p.Ser681*	stop_gained	regulator of chromosome condensation (RCC1) repeat-containing protein
TGME49_249000	p.Ser681Thr	missense_variant	regulator of chromosome condensation (RCC1) repeat-containing protein
TGME49_249000	p.Gly95Ala	missense_variant	regulator of chromosome condensation (RCC1) repeat-containing protein
TGME49_238110	p.Arg75His	missense_variant	replication factor a protein 3 protein

TGME49_214480	p.Pro158Ser	missense_variant	replication factor a protein 3 protein
TGME49_237110	p.Leu98Val	missense_variant	replication factor C subunit 2, putative
TGME49_210960	p.Glu245Gln	missense_variant	replication factor C subunit 4, putative
TGME49_236080	c.-523A>C	5_prime_UTR_premature_start_codon_gain_variant	replication factor-a protein 1 (rpa1) subfamily protein
TGME49_219860	c.-447T>G	5_prime_UTR_premature_start_codon_gain_variant	replication licensing factor, putative
TGME49_245590	p.Phe472Leu	missense_variant	rhomboid protease ROM6
TGME49_308096	p.His350Arg	missense_variant	rhoptry kinase family protein (incomplete catalytic triad)
TGME49_308096	p.Pro313Thr	missense_variant	rhoptry kinase family protein (incomplete catalytic triad)
TGME49_239600	p.Lys545Arg	missense_variant	rhoptry kinase family protein ROP23 (incomplete catalytic triad)
TGME49_252360	p.Gln229Lys	missense_variant	rhoptry kinase family protein ROP24 (incomplete catalytic triad)
TGME49_252360	p.Ser462Leu	missense_variant	rhoptry kinase family protein ROP24 (incomplete catalytic triad)
TGME49_211260	p.Thr191Ala	missense_variant	rhoptry kinase family protein ROP26 (incomplete catalytic triad)
TGME49_240090	p.Phe46Leu	missense_variant	rhoptry kinase family protein ROP34, putative
TGME49_240090	p.Glu506Asp	missense_variant	rhoptry kinase family protein ROP34, putative
TGME49_240090	p.Gln527His	missense_variant	rhoptry kinase family protein ROP34, putative
TGME49_300100	p.Gln378His	missense_variant	rhoptry neck protein RON2
TGME49_300100	p.Asn372Asp	missense_variant	rhoptry neck protein RON2
TGME49_223920	p.Lys1548Arg	missense_variant	rhoptry neck protein RON3
TGME49_297960	p.Asn1196Tyr	missense_variant	rhoptry neck protein RON6
TGME49_297960	p.Ile1181Leu	missense_variant	rhoptry neck protein RON6
TGME49_297960	p.Ala1102Val	missense_variant	rhoptry neck protein RON6
TGME49_297960	p.Ala1072Thr	missense_variant	rhoptry neck protein RON6
TGME49_297960	p.Pro751Ser	missense_variant	rhoptry neck protein RON6
TGME49_211290	p.His317Gln	missense_variant	rhoptry protein ROP15
TGME49_211290	p.Asp238Glu	missense_variant	rhoptry protein ROP15
TGME49_211290	p.Ile220Leu	missense_variant	rhoptry protein ROP15
TGME49_235905	p.Val259Leu	missense_variant	ribonuclease z, putative
TGME49_235905	c.952-8C>T	splice_region_variant	ribonuclease z, putative
TGME49_235905	p.Ser355Pro	missense_variant	ribonuclease z, putative
TGME49_235905	p.Pro357Ser	missense_variant	ribonuclease z, putative
TGME49_235905	p.Thr398Ala	missense_variant	ribonuclease z, putative
TGME49_235905	p.Lys531Arg	missense_variant	ribonuclease z, putative
TGME49_235905	p.Ser897Thr	missense_variant	ribonuclease z, putative
TGME49_235905	c.2932-3T>C	splice_region_variant	ribonuclease z, putative
TGME49_254380	c.418-5T>C	splice_region_variant	ribosomal protein L11, putative
TGME49_253800	p.Gly126Arg	missense_variant	ribosomal protein L15, putative
TGME49_253800	p.Gly189Asp	missense_variant	ribosomal protein L15, putative
TGME49_300613	p.Thr40Ala	missense_variant	ribosomal protein L16, putative
TGME49_214870	p.Val123Met	missense_variant	ribosomal protein L9, N-terminal domain-containing protein
TGME49_214870	p.Gly182Asp	missense_variant	ribosomal protein L9, N-terminal domain-containing protein
TGME49_300000	c.-1922C>T	5_prime_UTR_premature_start_codon_gain_variant	ribosomal protein RPL18
TGME49_300000	c.-1430G>T	5_prime_UTR_premature_start_codon_gain_variant	ribosomal protein RPL18
TGME49_300000	c.-116T>G	5_prime_UTR_premature_start_codon_gain_variant	ribosomal protein RPL18
TGME49_300000	c.467-8G>A	splice_region_variant	ribosomal protein RPL18
TGME49_245680	c.-7G>C	5_prime_UTR_premature_start_codon_gain_variant	ribosomal protein RPL21
TGME49_238010	c.-263T>C	5_prime_UTR_premature_start_codon_gain_variant	ribosomal protein RPL23A
TGME49_250810	c.-1014G>C	5_prime_UTR_premature_start_codon_gain_variant	ribosomal protein RPL35
TGME49_218410	p.Glu229Gly	missense_variant	ribosomal protein RPP0
TGME49_217670	p.Ser165Pro	missense_variant	ribosomal protein RPS6
TGME49_217670	p.Leu390Phe	missense_variant	ribosomal protein RPS6
TGME49_239100	c.*1314G>T	splice_region_variant	ribosomal protein RPS7
TGME49_218850	p.Trp6Cys	missense_variant	ribosomal protein RPS9
TGME49_277700	c.-130C>T	5_prime_UTR_premature_start_codon_gain_variant	ribosomal protein S14 precursor, putative
TGME49_222990	p.Ala17Thr	missense_variant	ribosomal protein S18, putative
TGME49_300606	p.Ile265Arg	missense_variant	ribosomal protein S5, putative
TGME49_248200	c.-667C>T	5_prime_UTR_premature_start_codon_gain_variant	ribosomal RNA (adenine(1779)-N(6)/adenine(1780)-N(6))-dimethyltransferase, putative
TGME49_248200	c.-485C>T	5_prime_UTR_premature_start_codon_gain_variant	ribosomal RNA (adenine(1779)-N(6)/adenine(1780)-N(6))-dimethyltransferase, putative
TGME49_248200	p.Phe106Ser	missense_variant	ribosomal RNA (adenine(1779)-N(6)/adenine(1780)-N(6))-dimethyltransferase, putative
TGME49_221580	p.Glu200Asp	missense_variant	ribosomal RNA large subunit methyltransferase J protein
TGME49_221580	c.3684-6C>G	splice_region_variant	ribosomal RNA large subunit methyltransferase J protein
TGME49_221580	p.Cys1834Gly	missense_variant	ribosomal RNA large subunit methyltransferase J protein
TGME49_221580	p.Glu1960Gly	missense_variant	ribosomal RNA large subunit methyltransferase J protein
TGME49_221580	p.Pro2032Ser	missense_variant	ribosomal RNA large subunit methyltransferase J protein
TGME49_278500	p.Ile6Leu	missense_variant	ribosomal RNA large subunit methyltransferase J protein
TGME49_278500	c.2344+6C>T	splice_region_variant	ribosomal RNA large subunit methyltransferase J protein
TGME49_278500	p.Asp913Gly	missense_variant	ribosomal RNA large subunit methyltransferase J protein
TGME49_278500	p.Gly924Arg	missense_variant	ribosomal RNA large subunit methyltransferase J protein
TGME49_278500	p.Ser938Ala	missense_variant	ribosomal RNA large subunit methyltransferase J protein
TGME49_278500	c.3974-4G>T	splice_region_variant	ribosomal RNA large subunit methyltransferase J protein
TGME49_220940	p.Ala228Val	missense_variant	ribosomal RNA methyltransferase (FtsJ) family protein
TGME49_301390	p.Ser28Pro	missense_variant	ribosome biogenesis protein BOP1, putative
TGME49_239310	c.-394A>G	5_prime_UTR_premature_start_codon_gain_variant	ribulose 5-phosphate isomerase
TGME49_239310	c.-196T>C	5_prime_UTR_premature_start_codon_gain_variant	ribulose 5-phosphate isomerase
TGME49_239310	p.Lys183Glu	missense_variant	ribulose 5-phosphate isomerase
TGME49_210830	p.Ser200Arg	missense_variant	RIO1 family protein
TGME49_210830	p.Ala503Glu	missense_variant	RIO1 family protein
TGME49_210830	p.Gln575Glu	missense_variant	RIO1 family protein
TGME49_210830	p.Arg623Lys	missense_variant	RIO1 family protein
TGME49_210830	p.Glu766Asp	missense_variant	RIO1 family protein
TGME49_254950	p.Cys287Ser	missense_variant	RNA cap guanine-N2 methyltransferase
TGME49_254950	p.Asn310His	missense_variant	RNA cap guanine-N2 methyltransferase
TGME49_254950	p.Arg323Gly	missense_variant	RNA cap guanine-N2 methyltransferase
TGME49_254950	p.Asp445Glu	missense_variant	RNA cap guanine-N2 methyltransferase
TGME49_254950	p.Ala920Val	missense_variant	RNA cap guanine-N2 methyltransferase
TGME49_218580	p.Ala438Ser	missense_variant	RNA methyltransferase, TrmH family protein

TGME49_218580	p.Ile448Val	missense_variant	RNA methyltransferase, TrmH family protein
TGME49_218580	p.Asn1531Lys	missense_variant	RNA methyltransferase, TrmH family protein
TGME49_218580	p.Asn1571Ser	missense_variant	RNA methyltransferase, TrmH family protein
TGME49_218580	p.Cys1921Arg	missense_variant	RNA methyltransferase, TrmH family protein
TGME49_218580	p.Val2116Gly	missense_variant	RNA methyltransferase, TrmH family protein
TGME49_218580	c.7691-8T>G	splice_region_variant	RNA methyltransferase, TrmH family protein
TGME49_218580	c.8409+5C>T	splice_region_variant	RNA methyltransferase, TrmH family protein
TGME49_249680	c.-228A>G	5_prime_UTR_premature_start_codon_gain_variant	RNA polymerase II associated Paf1 complex component PAF1
TGME49_249680	p.Arg238Gly	missense_variant	RNA polymerase II associated Paf1 complex component PAF1
TGME49_299030	p.His210Leu	missense_variant	RNA recognition motif 2 protein
TGME49_254210	c.-560C>G	5_prime_UTR_premature_start_codon_gain_variant	RNA recognition motif-containing protein
TGME49_254210	p.Val205Ile	missense_variant	RNA recognition motif-containing protein
TGME49_254210	p.Ser418Cys	missense_variant	RNA recognition motif-containing protein
TGME49_254210	p.Ser436Tyr	missense_variant	RNA recognition motif-containing protein
TGME49_254210	p.Leu510Val	missense_variant	RNA recognition motif-containing protein
TGME49_254210	p.Gly1003Ser	missense_variant	RNA recognition motif-containing protein
TGME49_254210	p.Gln1030His	missense_variant	RNA recognition motif-containing protein
TGME49_320100	c.-2695C>A	5_prime_UTR_premature_start_codon_gain_variant	RNA recognition motif-containing protein
TGME49_320100	c.-992A>G	5_prime_UTR_premature_start_codon_gain_variant	RNA recognition motif-containing protein
TGME49_320100	c.-380C>T	5_prime_UTR_premature_start_codon_gain_variant	RNA recognition motif-containing protein
TGME49_320100	p.Ala337Thr	missense_variant	RNA recognition motif-containing protein
TGME49_320100	p.Val383Met	missense_variant	RNA recognition motif-containing protein
TGME49_318690	p.Ala839Gly	missense_variant	RNA recognition motif-containing protein
TGME49_240070	c.-956A>T	5_prime_UTR_premature_start_codon_gain_variant	RNA recognition motif-containing protein
TGME49_240070	c.-1685A>T	5_prime_UTR_premature_start_codon_gain_variant	RNA recognition motif-containing protein
TGME49_223855	p.Gln392Glu	missense_variant	RNA recognition motif-containing protein
TGME49_223855	p.His320Asp	missense_variant	RNA recognition motif-containing protein
TGME49_223855	p.Glu164Gly	missense_variant	RNA recognition motif-containing protein
TGME49_223855	p.Ser73Ala	missense_variant	RNA recognition motif-containing protein
TGME49_223855	p.Leu68Phe	missense_variant	RNA recognition motif-containing protein
TGME49_236540	c.-760G>T	5_prime_UTR_premature_start_codon_gain_variant	RNA recognition motif-containing protein
TGME49_215230	c.-168G>T	5_prime_UTR_premature_start_codon_gain_variant	RNA recognition motif-containing protein
TGME49_218600	p.Ala64Thr	missense_variant	RNA recognition motif-containing protein
TGME49_218600	p.Pro595Ser	missense_variant	RNA recognition motif-containing protein
TGME49_218600	p.Arg682Gln	missense_variant	RNA recognition motif-containing protein
TGME49_218600	p.Ser705Thr	missense_variant&splice_region_variant	RNA recognition motif-containing protein
TGME49_218600	p.Arg711Ser	missense_variant	RNA recognition motif-containing protein
TGME49_239480	p.Lys1075Glu	missense_variant&splice_region_variant	RNB family domain-containing protein
TGME49_239480	p.Pro950Ser	missense_variant	RNB family domain-containing protein
TGME49_239480	p.Glu905Ala	missense_variant	RNB family domain-containing protein
TGME49_239480	p.Arg699Gly	missense_variant	RNB family domain-containing protein
TGME49_239480	p.Ile329Val	missense_variant	RNB family domain-containing protein
TGME49_239480	p.Ile129Leu	missense_variant	RNB family domain-containing protein
TGME49_239480	c.-31A>T	5_prime_UTR_premature_start_codon_gain_variant	RNB family domain-containing protein
TGME49_221440	p.Leu1933Arg	missense_variant	RPGR, putative
TGME49_221440	p.Asp1610Tyr	missense_variant	RPGR, putative
TGME49_221440	p.Ser1251Cys	missense_variant	RPGR, putative
TGME49_221440	p.Ser1026Tyr	missense_variant	RPGR, putative
TGME49_221440	p.Arg772His	missense_variant	RPGR, putative
TGME49_221440	p.Gly727Arg	missense_variant	RPGR, putative
TGME49_221440	p.Glu656Asp	missense_variant	RPGR, putative
TGME49_221440	p.Glu646Lys	missense_variant	RPGR, putative
TGME49_221440	p.His401Tyr	missense_variant	RPGR, putative
TGME49_221440	p.Ala360Glu	missense_variant	RPGR, putative
TGME49_221440	p.Ala311Gly	missense_variant	RPGR, putative
TGME49_221440	p.Ile263Val	missense_variant	RPGR, putative
TGME49_221440	p.Arg34Trp	missense_variant	RPGR, putative
TGME49_221440	p.Asp31Glu	missense_variant	RPGR, putative
TGME49_214210	c.-629T>G	5_prime_UTR_premature_start_codon_gain_variant	rRNA pseudouridine synthase
TGME49_319950	c.4-7C>A	splice_region_variant	rRNA-processing protein FCF1, putative
TGME49_319950	c.3+6C>G	splice_region_variant	rRNA-processing protein FCF1, putative
TGME49_319950	c.-419C>T	5_prime_UTR_premature_start_codon_gain_variant	rRNA-processing protein FCF1, putative
TGME49_280760	c.-100C>T	5_prime_UTR_premature_start_codon_gain_variant	RUB1 conjugating enzyme
TGME49_280750	c.-734T>G	5_prime_UTR_premature_start_codon_gain_variant	rudimentary enhancer, putative
TGME49_211670	p.Thr296Ala	missense_variant	S1 RNA binding domain-containing protein
TGME49_217790	p.Ile2101Thr	missense_variant	S1 RNA binding domain-containing protein
TGME49_217790	p.Ala2047Pro	missense_variant	S1 RNA binding domain-containing protein
TGME49_217790	p.Ser2019Pro	missense_variant	S1 RNA binding domain-containing protein
TGME49_217790	p.Glu1928Gly	missense_variant	S1 RNA binding domain-containing protein
TGME49_217790	p.Asp1913Gly	missense_variant	S1 RNA binding domain-containing protein
TGME49_217790	p.Leu1869Pro	missense_variant	S1 RNA binding domain-containing protein
TGME49_217790	p.Val1851Ile	missense_variant	S1 RNA binding domain-containing protein
TGME49_217790	p.Gly1842Arg	missense_variant	S1 RNA binding domain-containing protein
TGME49_217790	p.Ala1837Val	missense_variant	S1 RNA binding domain-containing protein
TGME49_217790	p.Ser1756Phe	missense_variant	S1 RNA binding domain-containing protein
TGME49_217790	p.Ala1749Thr	missense_variant	S1 RNA binding domain-containing protein
TGME49_217790	p.Ile983Val	missense_variant	S1 RNA binding domain-containing protein
TGME49_217790	p.Val957Gly	missense_variant	S1 RNA binding domain-containing protein
TGME49_217790	p.Leu679His	missense_variant	S1 RNA binding domain-containing protein
TGME49_217790	p.Lys408Thr	missense_variant	S1 RNA binding domain-containing protein
TGME49_217790	p.Leu315Pro	missense_variant	S1 RNA binding domain-containing protein
TGME49_217790	p.Thr307Arg	missense_variant	S1 RNA binding domain-containing protein
TGME49_217790	p.Ser280Ala	missense_variant	S1 RNA binding domain-containing protein
TGME49_217790	p.Arg279Pro	missense_variant	S1 RNA binding domain-containing protein
TGME49_217790	p.Pro109Leu	missense_variant	S1 RNA binding domain-containing protein

TGME49_250010	p.Phe709Val	missense_variant	Sad1 / UNC family C-terminal protein
TGME49_250010	p.Thr684Ser	missense_variant	Sad1 / UNC family C-terminal protein
TGME49_250010	p.Ala86Thr	missense_variant	Sad1 / UNC family C-terminal protein
TGME49_250010	p.Thr67Ala	missense_variant	Sad1 / UNC family C-terminal protein
TGME49_250010	c.-329C>T	5_prime_UTR_premature_start_codon_gain_variant	Sad1 / UNC family C-terminal protein
TGME49_207120	p.Lys714Thr	missense_variant	Sad1/UNC family protein
TGME49_207120	p.Ala775Thr	missense_variant	Sad1/UNC family protein
TGME49_207120	p.Ala884Val	missense_variant	Sad1/UNC family protein
TGME49_207120	p.Lys1656Arg	missense_variant	Sad1/UNC family protein
TGME49_207120	p.Arg1805His	missense_variant	Sad1/UNC family protein
TGME49_207120	p.Gly2045Ser	missense_variant	Sad1/UNC family protein
TGME49_207120	p.Pro2074Arg	missense_variant	Sad1/UNC family protein
TGME49_207120	p.Ala2954Thr	missense_variant	Sad1/UNC family protein
TGME49_207120	p.Ala3040Val	missense_variant	Sad1/UNC family protein
TGME49_207120	p.Ala3157Gly	missense_variant	Sad1/UNC family protein
TGME49_207120	p.Gln3181Pro	missense_variant	Sad1/UNC family protein
TGME49_207120	p.Phe3248Val	missense_variant	Sad1/UNC family protein
TGME49_222370	p.Thr671Pro	missense_variant	SAG-related sequence SRS13
TGME49_222370	p.Phe880Leu	missense_variant	SAG-related sequence SRS13
TGME49_320250	p.Asn349Lys	missense_variant	SAG-related sequence SRS15A
TGME49_320250	p.Asn349Thr	missense_variant	SAG-related sequence SRS15A
TGME49_320250	p.Gly343Glu	missense_variant	SAG-related sequence SRS15A
TGME49_320250	p.Pro337Leu	missense_variant	SAG-related sequence SRS15A
TGME49_320250	p.Asp300Asn	missense_variant	SAG-related sequence SRS15A
TGME49_320250	p.Ala263Glu	missense_variant	SAG-related sequence SRS15A
TGME49_320250	p.Lys223Glu	missense_variant	SAG-related sequence SRS15A
TGME49_320250	p.Pro202Ser	missense_variant	SAG-related sequence SRS15A
TGME49_320250	p.Glu201Gln	missense_variant	SAG-related sequence SRS15A
TGME49_320250	p.Val200Gly	missense_variant	SAG-related sequence SRS15A
TGME49_320250	p.Asp196Ala	missense_variant	SAG-related sequence SRS15A
TGME49_320250	p.Arg194Ser	missense_variant	SAG-related sequence SRS15A
TGME49_320250	p.Ile172Met	missense_variant	SAG-related sequence SRS15A
TGME49_320250	p.Thr164Ile	missense_variant	SAG-related sequence SRS15A
TGME49_320250	p.Lys162Asn	missense_variant	SAG-related sequence SRS15A
TGME49_320250	p.Thr161Ala	missense_variant	SAG-related sequence SRS15A
TGME49_320250	p.Asp140Asn	missense_variant	SAG-related sequence SRS15A
TGME49_320250	p.Glu136Asp	missense_variant	SAG-related sequence SRS15A
TGME49_320250	p.Thr135Lys	missense_variant	SAG-related sequence SRS15A
TGME49_320250	p.Asp125Glu	missense_variant	SAG-related sequence SRS15A
TGME49_320240	p.Thr341Asn	missense_variant	SAG-related sequence SRS15B
TGME49_320240	p.His335Gln	missense_variant	SAG-related sequence SRS15B
TGME49_320240	p.Asp283Asn	missense_variant	SAG-related sequence SRS15B
TGME49_320240	p.Ile250Met	missense_variant	SAG-related sequence SRS15B
TGME49_320240	p.Ile250Thr	missense_variant	SAG-related sequence SRS15B
TGME49_320240	p.Arg237Lys	missense_variant	SAG-related sequence SRS15B
TGME49_320240	p.Gln229Arg	missense_variant	SAG-related sequence SRS15B
TGME49_320240	p.Leu187Phe	missense_variant	SAG-related sequence SRS15B
TGME49_320240	p.Thr127Ala	missense_variant	SAG-related sequence SRS15B
TGME49_320240	p.Glu115Asp	missense_variant	SAG-related sequence SRS15B
TGME49_320240	p.Pro112His	missense_variant	SAG-related sequence SRS15B
TGME49_320240	p.Ala105Ser	missense_variant	SAG-related sequence SRS15B
TGME49_320240	p.Glu84Asp	missense_variant	SAG-related sequence SRS15B
TGME49_320240	p.Pro81Ser	missense_variant	SAG-related sequence SRS15B
TGME49_320240	p.Ser73Asn	missense_variant	SAG-related sequence SRS15B
TGME49_320240	p.Glu61Ala	missense_variant	SAG-related sequence SRS15B
TGME49_320240	p.Ala58Val	missense_variant	SAG-related sequence SRS15B
TGME49_320240	p.Phe32Leu	missense_variant	SAG-related sequence SRS15B
TGME49_320240	c.-70T>G	5_prime_UTR_premature_start_codon_gain_variant	SAG-related sequence SRS15B
TGME49_320240	c.-338C>T	5_prime_UTR_premature_start_codon_gain_variant	SAG-related sequence SRS15B
TGME49_320240	c.-385G>T	5_prime_UTR_premature_start_codon_gain_variant	SAG-related sequence SRS15B
TGME49_320240	c.-528C>T	5_prime_UTR_premature_start_codon_gain_variant	SAG-related sequence SRS15B
TGME49_320240	c.-926A>T	5_prime_UTR_premature_start_codon_gain_variant	SAG-related sequence SRS15B
TGME49_320240	c.-951C>T	5_prime_UTR_premature_start_codon_gain_variant	SAG-related sequence SRS15B
TGME49_320230	p.Gly381Ser	missense_variant	SAG-related sequence SRS15C
TGME49_320230	p.Ser344Pro	missense_variant	SAG-related sequence SRS15C
TGME49_320230	p.Asn312Ser	missense_variant	SAG-related sequence SRS15C
TGME49_320230	p.Ile300Met	missense_variant	SAG-related sequence SRS15C
TGME49_320230	p.Asn273Ser	missense_variant	SAG-related sequence SRS15C
TGME49_320230	p.Val252Ile	missense_variant	SAG-related sequence SRS15C
TGME49_320230	p.Glu198Gln	missense_variant	SAG-related sequence SRS15C
TGME49_320230	p.Asn174Ser	missense_variant	SAG-related sequence SRS15C
TGME49_320230	p.Thr166Ala	missense_variant	SAG-related sequence SRS15C
TGME49_320230	p.Glu164Asp	missense_variant	SAG-related sequence SRS15C
TGME49_320230	p.Glu164Gly	missense_variant	SAG-related sequence SRS15C
TGME49_320230	p.Ala144Thr	missense_variant	SAG-related sequence SRS15C
TGME49_320230	p.Gly124Ser	missense_variant	SAG-related sequence SRS15C
TGME49_320230	c.-6T>G	5_prime_UTR_premature_start_codon_gain_variant	SAG-related sequence SRS15C
TGME49_320200	p.Ala334Val	missense_variant	SAG-related sequence SRS16A
TGME49_320190	p.Glu319Ala	missense_variant	SAG-related sequence SRS16B
TGME49_320190	p.Pro304Ser	missense_variant	SAG-related sequence SRS16B
TGME49_320190	p.Ala270Gly	missense_variant	SAG-related sequence SRS16B
TGME49_320180	p.Ala384Thr	missense_variant	SAG-related sequence SRS16C
TGME49_320180	p.Val374Leu	missense_variant	SAG-related sequence SRS16C
TGME49_320180	p.Thr334Ile	missense_variant	SAG-related sequence SRS16C
TGME49_320180	p.Asn239Ile	missense_variant	SAG-related sequence SRS16C

TGME49_320180	p.Lys210Arg	missense_variant	SAG-related sequence SRS16C
TGME49_320180	p.Ala197Thr	missense_variant	SAG-related sequence SRS16C
TGME49_319360	p.Val11Ala	missense_variant	SAG-related sequence SRS17A
TGME49_319350	c.-340A>T	5_prime_UTR_premature_start_codon_gain_variant	SAG-related sequence SRS17B
TGME49_319350	p.Leu164Arg	missense_variant	SAG-related sequence SRS17B
TGME49_319350	p.Ile270Val	missense_variant	SAG-related sequence SRS17B
TGME49_319350	p.Gly309Arg	missense_variant	SAG-related sequence SRS17B
TGME49_319350	p.Val319Ile	missense_variant	SAG-related sequence SRS17B
TGME49_319350	p.Asn346Asp	missense_variant	SAG-related sequence SRS17B
TGME49_301140	p.Lys315Arg	missense_variant	SAG-related sequence SRS19A
TGME49_301140	p.Asp303Asn	missense_variant	SAG-related sequence SRS19A
TGME49_301150	p.Thr372Ala	missense_variant	SAG-related sequence SRS19B
TGME49_301150	p.Lys328Gln	missense_variant	SAG-related sequence SRS19B
TGME49_301150	p.Asn213Asp	missense_variant	SAG-related sequence SRS19B
TGME49_301150	p.Val208Ala	missense_variant	SAG-related sequence SRS19B
TGME49_301150	p.Thr205Ile	missense_variant	SAG-related sequence SRS19B
TGME49_301150	p.Lys202Asn	missense_variant	SAG-related sequence SRS19B
TGME49_301150	p.Lys202Arg	missense_variant	SAG-related sequence SRS19B
TGME49_301150	p.Gln200Lys	missense_variant	SAG-related sequence SRS19B
TGME49_301150	p.Ala184Val	missense_variant	SAG-related sequence SRS19B
TGME49_301150	p.Gly169Glu	missense_variant	SAG-related sequence SRS19B
TGME49_301150	p.Gln166Lys	missense_variant	SAG-related sequence SRS19B
TGME49_301150	p.Gln165His	missense_variant	SAG-related sequence SRS19B
TGME49_301150	p.Glu157Gly	missense_variant	SAG-related sequence SRS19B
TGME49_301150	p.Ser145Pro	missense_variant	SAG-related sequence SRS19B
TGME49_301150	p.Asp132Glu	missense_variant	SAG-related sequence SRS19B
TGME49_301150	p.Arg131Gln	missense_variant	SAG-related sequence SRS19B
TGME49_301150	p.Lys129Arg	missense_variant	SAG-related sequence SRS19B
TGME49_301150	p.Asn122Lys	missense_variant	SAG-related sequence SRS19B
TGME49_301150	p.Gly114Glu	missense_variant	SAG-related sequence SRS19B
TGME49_301150	p.Glu113Lys	missense_variant	SAG-related sequence SRS19B
TGME49_301150	p.Glu64Gln	missense_variant	SAG-related sequence SRS19B
TGME49_301150	p.Gln36His	missense_variant	SAG-related sequence SRS19B
TGME49_301160	p.Leu396Pro	missense_variant	SAG-related sequence SRS19C
TGME49_301160	p.Thr373Ala	missense_variant	SAG-related sequence SRS19C
TGME49_301160	p.Thr370Ala	missense_variant	SAG-related sequence SRS19C
TGME49_301160	p.Ile291Val	missense_variant	SAG-related sequence SRS19C
TGME49_301160	p.Asn210Lys	missense_variant	SAG-related sequence SRS19C
TGME49_301160	p.Asn210Ile	missense_variant	SAG-related sequence SRS19C
TGME49_301160	p.Ala205Glu	missense_variant	SAG-related sequence SRS19C
TGME49_301160	p.Glu201Ala	missense_variant	SAG-related sequence SRS19C
TGME49_301160	p.Asp174Asn	missense_variant	SAG-related sequence SRS19C
TGME49_301160	p.Ile164Leu	missense_variant	SAG-related sequence SRS19C
TGME49_301160	p.Ser140Ala	missense_variant	SAG-related sequence SRS19C
TGME49_301160	p.Lys139Arg	missense_variant	SAG-related sequence SRS19C
TGME49_301160	p.Asp113Gly	missense_variant	SAG-related sequence SRS19C
TGME49_301160	p.Ala78Val	missense_variant	SAG-related sequence SRS19C
TGME49_301160	p.Ser72Thr	missense_variant	SAG-related sequence SRS19C
TGME49_301160	p.Thr70Ile	missense_variant	SAG-related sequence SRS19C
TGME49_301160	p.Gln63Lys	missense_variant	SAG-related sequence SRS19C
TGME49_301160	p.Thr58Ala	missense_variant	SAG-related sequence SRS19C
TGME49_301160	p.Leu57Pro	missense_variant	SAG-related sequence SRS19C
TGME49_301160	p.Gly54Ala	missense_variant	SAG-related sequence SRS19C
TGME49_301160	p.Trp13Gly	missense_variant	SAG-related sequence SRS19C
TGME49_301160	p.Arg8Trp	missense_variant	SAG-related sequence SRS19C
TGME49_301170	p.Leu376Phe	missense_variant	SAG-related sequence SRS19D
TGME49_301170	p.Gly359Ala	missense_variant	SAG-related sequence SRS19D
TGME49_301170	p.Ala339Thr	missense_variant	SAG-related sequence SRS19D
TGME49_301170	p.Asp337Glu	missense_variant	SAG-related sequence SRS19D
TGME49_301170	p.Ala336Val	missense_variant	SAG-related sequence SRS19D
TGME49_301170	p.His335Asp	missense_variant	SAG-related sequence SRS19D
TGME49_301170	p.Glu328Asp	missense_variant	SAG-related sequence SRS19D
TGME49_301170	p.Thr307Lys	missense_variant	SAG-related sequence SRS19D
TGME49_301170	p.Gln300Leu	missense_variant	SAG-related sequence SRS19D
TGME49_301170	p.Gln300Glu	missense_variant	SAG-related sequence SRS19D
TGME49_301170	p.Ser299Asn	missense_variant	SAG-related sequence SRS19D
TGME49_301170	p.Lys289Arg	missense_variant	SAG-related sequence SRS19D
TGME49_301170	p.Pro270Leu	missense_variant	SAG-related sequence SRS19D
TGME49_301170	p.Ala201Asp	missense_variant	SAG-related sequence SRS19D
TGME49_301170	p.Gly197Arg	missense_variant	SAG-related sequence SRS19D
TGME49_301170	p.Lys193Glu	missense_variant	SAG-related sequence SRS19D
TGME49_301170	p.Pro183Thr	missense_variant	SAG-related sequence SRS19D
TGME49_301170	p.Glu160Gly	missense_variant	SAG-related sequence SRS19D
TGME49_301170	p.Glu154Lys	missense_variant	SAG-related sequence SRS19D
TGME49_301170	p.Lys152Asn	missense_variant	SAG-related sequence SRS19D
TGME49_301170	p.Ser143Thr	missense_variant	SAG-related sequence SRS19D
TGME49_301170	p.Glu135Gln	missense_variant	SAG-related sequence SRS19D
TGME49_301170	p.Lys127Arg	missense_variant	SAG-related sequence SRS19D
TGME49_301170	p.Gly122Asp	missense_variant	SAG-related sequence SRS19D
TGME49_301170	p.Arg119Lys	missense_variant	SAG-related sequence SRS19D
TGME49_301170	p.Ser110Asn	missense_variant	SAG-related sequence SRS19D
TGME49_301170	p.Glu109Lys	missense_variant	SAG-related sequence SRS19D
TGME49_301170	p.Val74Ala	missense_variant	SAG-related sequence SRS19D
TGME49_301170	p.Ser73Ala	missense_variant	SAG-related sequence SRS19D
TGME49_301170	p.Thr48Ser	missense_variant	SAG-related sequence SRS19D

TGME49_301170	p.Lys41Thr	missense_variant	SAG-related sequence SRS19D
TGME49_301170	p.Pro39Ser	missense_variant	SAG-related sequence SRS19D
TGME49_301170	p.Glu36Gln	missense_variant	SAG-related sequence SRS19D
TGME49_301170	c.-53C>T	5_prime_UTR_premature_start_codon_gain_variant	SAG-related sequence SRS19D
TGME49_301180	p.Ser333Leu	missense_variant	SAG-related sequence SRS19F
TGME49_301180	p.Gln304Leu	missense_variant	SAG-related sequence SRS19F
TGME49_301180	p.Gln304Glu	missense_variant	SAG-related sequence SRS19F
TGME49_301180	p.Gly303Asp	missense_variant	SAG-related sequence SRS19F
TGME49_301180	p.Lys293Arg	missense_variant	SAG-related sequence SRS19F
TGME49_301180	p.Pro274Leu	missense_variant	SAG-related sequence SRS19F
TGME49_301180	p.Ala265Thr	missense_variant	SAG-related sequence SRS19F
TGME49_301180	p.Met263Val	missense_variant	SAG-related sequence SRS19F
TGME49_301180	p.Ile227Val	missense_variant	SAG-related sequence SRS19F
TGME49_301180	p.Glu223Lys	missense_variant	SAG-related sequence SRS19F
TGME49_301180	p.Glu217Lys	missense_variant	SAG-related sequence SRS19F
TGME49_301180	p.Gln198Leu	missense_variant	SAG-related sequence SRS19F
TGME49_301180	p.Gln198Glu	missense_variant	SAG-related sequence SRS19F
TGME49_301180	p.Thr186Met	missense_variant	SAG-related sequence SRS19F
TGME49_301180	p.Ala173Thr	missense_variant	SAG-related sequence SRS19F
TGME49_301180	p.Asn170Lys	missense_variant	SAG-related sequence SRS19F
TGME49_301180	p.Asn170Asp	missense_variant	SAG-related sequence SRS19F
TGME49_301180	p.Val161Ala	missense_variant	SAG-related sequence SRS19F
TGME49_301180	p.Ala141Gly	missense_variant	SAG-related sequence SRS19F
TGME49_301180	p.Thr139Ser	missense_variant	SAG-related sequence SRS19F
TGME49_301180	p.Ser124Arg	missense_variant	SAG-related sequence SRS19F
TGME49_301180	p.Glu116Gly	missense_variant	SAG-related sequence SRS19F
TGME49_301180	p.Arg113Pro	missense_variant	SAG-related sequence SRS19F
TGME49_301180	p.Gly96Glu	missense_variant	SAG-related sequence SRS19F
TGME49_301180	p.Glu81Gly	missense_variant	SAG-related sequence SRS19F
TGME49_301180	p.Thr69Ala	missense_variant	SAG-related sequence SRS19F
TGME49_301180	p.Gln64Glu	missense_variant	SAG-related sequence SRS19F
TGME49_301180	p.Asp57Asn	missense_variant	SAG-related sequence SRS19F
TGME49_301180	p.Gly54Ser	missense_variant	SAG-related sequence SRS19F
TGME49_301180	p.Gly48Arg	missense_variant	SAG-related sequence SRS19F
TGME49_301180	c.-53G>A	5_prime_UTR_premature_start_codon_gain_variant	SAG-related sequence SRS19F
TGME49_238460	p.Ile88Met	missense_variant	SAG-related sequence SRS22B
TGME49_238460	p.Pro63Thr	missense_variant	SAG-related sequence SRS22B
TGME49_239090	p.Thr252Ala	missense_variant	SAG-related sequence SRS23
TGME49_239090	p.Lys131Arg	missense_variant	SAG-related sequence SRS23
TGME49_224790	p.Gln373His	missense_variant	SAG-related sequence SRS40A
TGME49_224790	p.Thr363Met	missense_variant	SAG-related sequence SRS40A
TGME49_224790	p.Ala305Thr	missense_variant	SAG-related sequence SRS40A
TGME49_224790	p.Glu159Lys	missense_variant	SAG-related sequence SRS40A
TGME49_224790	p.Ser113Asn	missense_variant	SAG-related sequence SRS40A
TGME49_224790	p.Lys80Arg	missense_variant	SAG-related sequence SRS40A
TGME49_224790	p.Lys66Gln	missense_variant	SAG-related sequence SRS40A
TGME49_224790	c.-279C>G	5_prime_UTR_premature_start_codon_gain_variant	SAG-related sequence SRS40A
TGME49_224790	c.-1011A>G	5_prime_UTR_premature_start_codon_gain_variant	SAG-related sequence SRS40A
TGME49_224770	p.Gly199Ala	missense_variant	SAG-related sequence SRS40D
TGME49_224770	p.Ser136Pro	missense_variant	SAG-related sequence SRS40D
TGME49_224770	p.Ala135Asp	missense_variant	SAG-related sequence SRS40D
TGME49_224770	p.Asn132Ser	missense_variant	SAG-related sequence SRS40D
TGME49_224760	p.Met280Ile	missense_variant	SAG-related sequence SRS40E
TGME49_224760	p.Gln176Lys	missense_variant	SAG-related sequence SRS40E
TGME49_224760	p.Asp136Asn	missense_variant	SAG-related sequence SRS40E
TGME49_224760	c.-102T>G	5_prime_UTR_premature_start_codon_gain_variant	SAG-related sequence SRS40E
TGME49_224750	p.Glu267Lys	missense_variant	SAG-related sequence SRS40F
TGME49_224750	p.Asn224Asp	missense_variant	SAG-related sequence SRS40F
TGME49_224750	p.Ser199Ala	missense_variant	SAG-related sequence SRS40F
TGME49_224750	p.Gln196Arg	missense_variant	SAG-related sequence SRS40F
TGME49_224750	p.Ala194Gly	missense_variant	SAG-related sequence SRS40F
TGME49_224750	p.Asn128Lys	missense_variant	SAG-related sequence SRS40F
TGME49_224750	p.Gln60His	missense_variant	SAG-related sequence SRS40F
TGME49_224750	p.Gln56Glu	missense_variant	SAG-related sequence SRS40F
TGME49_224750	p.Gly53Ala	missense_variant	SAG-related sequence SRS40F
TGME49_234930	p.Ala374Thr	missense_variant	SAG-related sequence SRS43
TGME49_234930	p.Asp117Asn	missense_variant	SAG-related sequence SRS43
TGME49_234930	p.Gln104His	missense_variant	SAG-related sequence SRS43
TGME49_234930	p.Ala58Gly	missense_variant	SAG-related sequence SRS43
TGME49_207010	p.Pro119Arg	missense_variant	SAG-related sequence SRS48K
TGME49_207150	p.Asn108Lys	missense_variant	SAG-related sequence SRS49C
TGME49_207150	p.Leu15Val	missense_variant	SAG-related sequence SRS49C
TGME49_207160	p.Ala100Thr	missense_variant	SAG-related sequence SRS49D
TGME49_308020	c.-248T>G	5_prime_UTR_premature_start_codon_gain_variant	SAG-related sequence SRS57
TGME49_308020	c.-447G>T	5_prime_UTR_premature_start_codon_gain_variant	SAG-related sequence SRS57
TGME49_279000	p.Pro69Gln	missense_variant	SAG-related sequence SRS59J
TGME49_279000	c.-279C>T	5_prime_UTR_premature_start_codon_gain_variant	SAG-related sequence SRS59J
TGME49_251962	p.Lys386Gln	missense_variant	SAG-related sequence SRS59K
TGME49_224170	p.Lys160Gln	missense_variant	SAG-related sequence SRS60A
TGME49_224170	p.Ala227Ser	missense_variant	SAG-related sequence SRS60A
TGME49_252320	p.Ser158Arg	missense_variant	Sas10/Utp3/C1D family protein
TGME49_222960	c.-174C>T	5_prime_UTR_premature_start_codon_gain_variant	SCY kinase-related protein (incomplete catalytic triad)
TGME49_222960	p.Ala569Ser	missense_variant	SCY kinase-related protein (incomplete catalytic triad)
TGME49_222960	p.His799Gln	missense_variant	SCY kinase-related protein (incomplete catalytic triad)
TGME49_217780	c.*542G>C	splice_region_variant	Sec20 protein

TGME49_217780	p.Arg805His	missense_variant	Sec20 protein
TGME49_217780	p.Ala555Ser	missense_variant	Sec20 protein
TGME49_217780	p.Leu484Pro	missense_variant	Sec20 protein
TGME49_217780	c.1329+7T>C	splice_region_variant	Sec20 protein
TGME49_217780	p.Ser340Asn	missense_variant	Sec20 protein
TGME49_217780	c.-310A>G	5_prime_UTR_premature_start_codon_gain_variant	Sec20 protein
TGME49_217780	c.-571C>T	5_prime_UTR_premature_start_codon_gain_variant	Sec20 protein
TGME49_217780	c.-1810A>G	5_prime_UTR_premature_start_codon_gain_variant	Sec20 protein
TGME49_217780	c.-2083G>C	5_prime_UTR_premature_start_codon_gain_variant	Sec20 protein
TGME49_235700	p.Ser290Thr	missense_variant	sedoheptulose-1,7-bisphosphatase
TGME49_254490	p.Ala61Val	missense_variant	Sel1 repeat-containing protein
TGME49_254490	p.Arg64Gln	missense_variant	Sel1 repeat-containing protein
TGME49_254490	p.Gly118Arg	missense_variant	Sel1 repeat-containing protein
TGME49_254490	p.His1100Asn	missense_variant	Sel1 repeat-containing protein
TGME49_254490	c.*1565C>T	splice_region_variant	Sel1 repeat-containing protein
TGME49_301430	p.Asp35Gly	missense_variant	septum formation protein maf, putative
TGME49_301430	p.Ala169Glu	missense_variant&splice_region_variant	septum formation protein maf, putative
TGME49_301430	p.Ser234Gly	missense_variant	septum formation protein maf, putative
TGME49_301430	p.Ter391Glnext*?	stop_lost	septum formation protein maf, putative
TGME49_222840	p.Arg68Gly	missense_variant	Ser/Thr phosphatase family protein
TGME49_297650	c.1574-6T>G	splice_region_variant	Ser/Thr phosphatase family protein
TGME49_297650	p.Lys476Asn	missense_variant	Ser/Thr phosphatase family protein
TGME49_297650	c.874+6C>T	splice_region_variant	Ser/Thr phosphatase family protein
TGME49_297650	p.Ser24Pro	missense_variant	Ser/Thr phosphatase family protein
TGME49_297650	c.-157T>C	5_prime_UTR_premature_start_codon_gain_variant	Ser/Thr phosphatase family protein
TGME49_254770	p.Arg363Thr	missense_variant	Ser/Thr phosphatase family protein
TGME49_238230	c.-798A>T	5_prime_UTR_premature_start_codon_gain_variant	Ser/Thr phosphatase family protein
TGME49_238230	c.-479C>T	5_prime_UTR_premature_start_codon_gain_variant	Ser/Thr phosphatase family protein
TGME49_254010	p.Val254Ala	missense_variant	serine carboxypeptidase s28 protein
TGME49_254010	c.1123+4G>T	splice_region_variant	serine carboxypeptidase s28 protein
TGME49_254010	p.Glu504Lys	missense_variant	serine carboxypeptidase s28 protein
TGME49_254010	c.1667-7T>C	splice_region_variant	serine carboxypeptidase s28 protein
TGME49_254010	p.Gly684Arg	missense_variant	serine carboxypeptidase s28 protein
TGME49_254010	p.Ala703Gly	missense_variant	serine carboxypeptidase s28 protein
TGME49_254710	p.Ala656Thr	missense_variant	serine esterase (DUF676) protein
TGME49_254710	c.-695T>G	5_prime_UTR_premature_start_codon_gain_variant	serine esterase (DUF676) protein
TGME49_234190	p.Ala593Val	missense_variant	serine hydroxymethyltransferase 2, putative
TGME49_234190	p.Ile580Val	missense_variant	serine hydroxymethyltransferase 2, putative
TGME49_251850	c.-958C>T	5_prime_UTR_premature_start_codon_gain_variant	serine/threonine protein phosphatase
TGME49_301010	c.-1359C>G	5_prime_UTR_premature_start_codon_gain_variant	serine/threonine protein phosphatase, putative
TGME49_223985	p.Pro1555Arg	missense_variant	serine/threonine specific protein phosphatase
TGME49_223985	p.Leu227Phe	missense_variant	serine/threonine specific protein phosphatase
TGME49_223985	p.Phe215Leu	missense_variant	serine/threonine specific protein phosphatase
TGME49_223985	p.Asn211Ser	missense_variant	serine/threonine specific protein phosphatase
TGME49_223985	p.Ala4Ser	missense_variant	serine/threonine specific protein phosphatase
TGME49_223985	c.-2368A>C	5_prime_UTR_premature_start_codon_gain_variant	serine/threonine specific protein phosphatase
TGME49_224220	c.-501C>T	5_prime_UTR_premature_start_codon_gain_variant	serine/threonine-protein phosphatase PP2A catalytic subunit
TGME49_218880	c.-1095C>T	5_prime_UTR_premature_start_codon_gain_variant	SF-assemblin, putative
TGME49_280740	c.-38C>T	5_prime_UTR_premature_start_codon_gain_variant	signal peptidase
TGME49_214090	p.Leu882Phe	missense_variant	signal peptidase
TGME49_214090	p.Glu1216Asp	missense_variant	signal peptidase
TGME49_214090	p.Asn1356Ser	missense_variant	signal peptidase
TGME49_300060	c.-34C>T	5_prime_UTR_premature_start_codon_gain_variant	signal peptidase subunit protein
TGME49_236130	c.-184T>G	5_prime_UTR_premature_start_codon_gain_variant	signal recognition particle (SRP9) domain-containing protein
TGME49_236130	c.358-5T>C	splice_region_variant	signal recognition particle (SRP9) domain-containing protein
TGME49_236130	c.556+6A>G	splice_region_variant	signal recognition particle (SRP9) domain-containing protein
TGME49_280610	c.-380A>T	5_prime_UTR_premature_start_codon_gain_variant	signal recognition particle receptor alpha subunit
TGME49_280610	c.-223C>T	5_prime_UTR_premature_start_codon_gain_variant	signal recognition particle receptor alpha subunit
TGME49_297940	p.Ser548Thr	missense_variant	single-strand binding protein
TGME49_297940	p.Ala354Thr	missense_variant	single-strand binding protein
TGME49_297940	p.Ser284Pro	missense_variant	single-strand binding protein
TGME49_297940	p.Thr69Ala	missense_variant	single-strand binding protein
TGME49_212260	p.Leu184Arg	missense_variant	Sjogren's syndrome/scleroderma autoantigen 1 (Autoantigen p27) protein
TGME49_251630	p.Leu43Val	missense_variant	slc30a2 protein
TGME49_251630	p.Arg97Ser	missense_variant	slc30a2 protein
TGME49_251630	p.Val173Leu	missense_variant	slc30a2 protein
TGME49_251630	p.Arg290Thr	missense_variant	slc30a2 protein
TGME49_251630	p.Ala308Ser	missense_variant	slc30a2 protein
TGME49_251630	p.Ala308Val	missense_variant	slc30a2 protein
TGME49_251630	p.Gly352Val	missense_variant	slc30a2 protein
TGME49_251630	p.Ser423Asn	missense_variant	slc30a2 protein
TGME49_251630	p.Leu424His	missense_variant	slc30a2 protein
TGME49_214770	c.87+6T>G	splice_region_variant	small GTP binding protein rab1a, putative
TGME49_247610	c.-74T>G	5_prime_UTR_premature_start_codon_gain_variant	small nuclear ribonucleoprotein E, putative
TGME49_248870	p.Ala18Thr	missense_variant	SNARE associated Golgi protein
TGME49_248870	p.Leu36Ile	missense_variant	SNARE associated Golgi protein
TGME49_301350	c.-2312C>T	5_prime_UTR_premature_start_codon_gain_variant	SNARE associated protein
TGME49_301350	c.-965C>T	5_prime_UTR_premature_start_codon_gain_variant	SNARE associated protein
TGME49_301350	p.Pro22Ser	missense_variant	SNARE associated protein
TGME49_301350	p.Pro402Leu	missense_variant	SNARE associated protein
TGME49_301350	p.Val411Ile	missense_variant	SNARE associated protein
TGME49_248310	c.-525G>T	5_prime_UTR_premature_start_codon_gain_variant	SNF7 family protein
TGME49_208420	p.Lys393Asn	missense_variant	Sodium:neurotransmitter symporter family protein
TGME49_299060	c.-18G>T	5_prime_UTR_premature_start_codon_gain_variant	sodium/hydrogen exchanger NHE2
TGME49_299060	p.Glu482Asp	missense_variant	sodium/hydrogen exchanger NHE2

TGME49_222210	c.-186A>G	5_prime_UTR_premature_start_codon_gain_variant	SPFH domain / Band 7 family protein
TGME49_237200	c.-440A>G	5_prime_UTR_premature_start_codon_gain_variant	sphingolipid delta 4 desaturase/c-4 hydroxylase protein des2 family protein
TGME49_319530	c.-395T>G	5_prime_UTR_premature_start_codon_gain_variant	splicing factor SF2
TGME49_224720	p.Ala670Ser	missense_variant	SPOC domain-containing protein
TGME49_224720	p.Thr625Ile	missense_variant	SPOC domain-containing protein
TGME49_224720	p.Glu589Asp	missense_variant	SPOC domain-containing protein
TGME49_224720	p.Ala88Gly	missense_variant	SPOC domain-containing protein
TGME49_246020	p.Ala310Ser	missense_variant	SprT domain-containing protein
TGME49_246020	p.Lys491Thr	missense_variant	SprT domain-containing protein
TGME49_221450	c.-253C>G	5_prime_UTR_premature_start_codon_gain_variant	SPRY domain-containing protein
TGME49_221450	p.Gln109Pro	missense_variant	SPRY domain-containing protein
TGME49_248550	c.2320-6G>A	splice_region_variant	SPX domain-containing protein
TGME49_275802	p.Thr728Ala	missense_variant	SRP72 RNA-binding domain-containing protein
TGME49_275802	p.Met471Val	missense_variant	SRP72 RNA-binding domain-containing protein
TGME49_248780	p.Gly29Val	missense_variant	SRR1 protein
TGME49_248780	p.Thr65Ile	missense_variant	SRR1 protein
TGME49_248780	p.Ala130Pro	missense_variant	SRR1 protein
TGME49_248780	p.Asn458Lys	missense_variant	SRR1 protein
TGME49_248780	p.Glu562Lys	missense_variant	SRR1 protein
TGME49_248780	p.Ala598Val	missense_variant	SRR1 protein
TGME49_248780	p.Trp751Ser	missense_variant	SRR1 protein
TGME49_236660	p.Glu900Lys	missense_variant	START domain-containing protein
TGME49_319970	p.Thr80Ile	missense_variant	subtilisin SUB10
TGME49_319970	c.1288-4T>G	splice_region_variant	subtilisin SUB10
TGME49_235860	p.Tyr189Asp	missense_variant	subtilisin SUB11
TGME49_235860	p.Val220Ala	missense_variant	subtilisin SUB11
TGME49_235860	c.1852+6G>A	splice_region_variant	subtilisin SUB11
TGME49_221830	c.1338+7G>A	splice_region_variant	subtilisin SUB12
TGME49_221830	p.Asn143Ser	missense_variant	subtilisin SUB12
TGME49_200350	p.Leu437Phe	missense_variant	subtilisin SUB3
TGME49_240240	p.Ser54Cys	missense_variant	subtilisin SUB5
TGME49_240240	p.Pro216Leu	missense_variant	subtilisin SUB5
TGME49_240240	p.Pro413Leu	missense_variant	subtilisin SUB5
TGME49_240240	p.Gly715Arg	missense_variant	subtilisin SUB5
TGME49_240240	p.Ala1003Pro	missense_variant	subtilisin SUB5
TGME49_248470	c.1315-4G>A	splice_region_variant	subtilisin SUB6
TGME49_235950	p.Met98Leu	missense_variant	subtilisin SUB8
TGME49_235950	p.Ala115Val	missense_variant	subtilisin SUB8
TGME49_235950	p.Glu554Asp	missense_variant	subtilisin SUB8
TGME49_235950	p.Ser717Thr	missense_variant	subtilisin SUB8
TGME49_251760	c.652-6T>C	splice_region_variant	subunit of proteaseome activator complex, putative
TGME49_251760	c.186+7G>A	splice_region_variant	subunit of proteaseome activator complex, putative
TGME49_215280	c.400+5G>T	splice_region_variant	succinate dehydrogenase [ubiquinone] iron-sulfur protein
TGME49_217850	c.924+8C>T	splice_region_variant	Sucrose-6F-phosphate phosphohydrolase
TGME49_217850	c.717-5T>C	splice_region_variant	Sucrose-6F-phosphate phosphohydrolase
TGME49_246500	p.Thr518Ala	missense_variant	surp module domain-containing protein
TGME49_211270	p.Asn3882Thr	missense_variant	sushi domain (scr repeat) domain-containing protein
TGME49_211270	p.Leu3879Ser	missense_variant	sushi domain (scr repeat) domain-containing protein
TGME49_211270	p.Arg3754Trp	missense_variant	sushi domain (scr repeat) domain-containing protein
TGME49_211270	p.Tyr3620Ser	missense_variant	sushi domain (scr repeat) domain-containing protein
TGME49_211270	p.Pro3558Leu	missense_variant	sushi domain (scr repeat) domain-containing protein
TGME49_211270	p.Pro3368Leu	missense_variant	sushi domain (scr repeat) domain-containing protein
TGME49_211270	p.Ser1243Leu	missense_variant	sushi domain (scr repeat) domain-containing protein
TGME49_211270	p.Ala1161Thr	missense_variant	sushi domain (scr repeat) domain-containing protein
TGME49_211270	p.Gly524Arg	missense_variant	sushi domain (scr repeat) domain-containing protein
TGME49_223480	p.Ser2Phe	missense_variant	sushi domain (scr repeat) domain-containing protein
TGME49_223480	p.Asp16Gly	missense_variant	sushi domain (scr repeat) domain-containing protein
TGME49_223480	p.Ser651Ala	missense_variant	sushi domain (scr repeat) domain-containing protein
TGME49_223480	p.Val1864Ile	missense_variant	sushi domain (scr repeat) domain-containing protein
TGME49_223480	p.Glu2121Gly	missense_variant	sushi domain (scr repeat) domain-containing protein
TGME49_223480	p.Val2315Met	missense_variant	sushi domain (scr repeat) domain-containing protein
TGME49_223480	p.Thr3254Arg	missense_variant	sushi domain (scr repeat) domain-containing protein
TGME49_223480	p.Ser3437Leu	missense_variant	sushi domain (scr repeat) domain-containing protein
TGME49_223480	p.Thr3743Arg	missense_variant	sushi domain (scr repeat) domain-containing protein
TGME49_223480	p.Gln3961His	missense_variant	sushi domain (scr repeat) domain-containing protein
TGME49_320300	c.3384-6T>C	splice_region_variant	SWI2/SNF2 Brahma-like putative
TGME49_320300	p.Glu624Asp	missense_variant	SWI2/SNF2 Brahma-like putative
TGME49_320300	p.Ser615Ile	missense_variant	SWI2/SNF2 Brahma-like putative
TGME49_320300	c.-982C>T	5_prime_UTR_premature_start_codon_gain_variant	SWI2/SNF2 Brahma-like putative
TGME49_320300	c.-1525G>T	5_prime_UTR_premature_start_codon_gain_variant	SWI2/SNF2 Brahma-like putative
TGME49_278440	c.-261C>T	5_prime_UTR_premature_start_codon_gain_variant	SWI2/SNF2 Brahma-like putative
TGME49_278440	p.Gly93Arg	missense_variant	SWI2/SNF2 Brahma-like putative
TGME49_278440	p.Pro863Leu	missense_variant	SWI2/SNF2 Brahma-like putative
TGME49_278440	p.Leu1863Val	missense_variant	SWI2/SNF2 Brahma-like putative
TGME49_278440	c.6219+6C>T	splice_region_variant	SWI2/SNF2 Brahma-like putative
TGME49_278440	c.6370-4A>G	splice_region_variant	SWI2/SNF2 Brahma-like putative
TGME49_278440	p.Pro2255Gln	missense_variant	SWI2/SNF2 Brahma-like putative
TGME49_280800	p.Ser2111Cys	missense_variant	SWI2/SNF2 SRCAP/Ino80
TGME49_280800	c.4658+8A>C	splice_region_variant	SWI2/SNF2 SRCAP/Ino80
TGME49_280800	p.Leu912Pro	missense_variant	SWI2/SNF2 SRCAP/Ino80
TGME49_280800	c.-741C>T	5_prime_UTR_premature_start_codon_gain_variant	SWI2/SNF2 SRCAP/Ino80
TGME49_280800	c.-996A>G	5_prime_UTR_premature_start_codon_gain_variant	SWI2/SNF2 SRCAP/Ino80
TGME49_236970	p.Ala649Thr	missense_variant	SWI2/SNF2-containing PHD finger protein
TGME49_236970	p.Ala2180Ser	missense_variant	SWI2/SNF2-containing PHD finger protein
TGME49_245720	p.Pro810Ser	missense_variant	SWI2/SNF2-containing protein

TGME49_245720	p.Pro810Leu	missense_variant	SWI2/SNF2-containing protein
TGME49_245720	p.Pro1080Gln	missense_variant	SWI2/SNF2-containing protein
TGME49_245720	p.Phe1630Leu	missense_variant	SWI2/SNF2-containing protein
TGME49_245720	p.Leu2236Arg	missense_variant	SWI2/SNF2-containing protein
TGME49_245720	p.Gly2707Asp	missense_variant	SWI2/SNF2-containing protein
TGME49_245720	c.8781+7T>C	splice_region_variant	SWI2/SNF2-containing protein
TGME49_245720	p.Cys3108*	stop_gained	SWI2/SNF2-containing protein
TGME49_277070	c.-2240C>T	5_prime_UTR_premature_start_codon_gain_variant	SWI2/SNF2-containing protein
TGME49_277070	p.Gly164Cys	missense_variant	SWI2/SNF2-containing protein
TGME49_277070	p.Gly892Glu	missense_variant	SWI2/SNF2-containing protein
TGME49_277070	p.Gly902Arg	missense_variant	SWI2/SNF2-containing protein
TGME49_318480	c.-267C>T	5_prime_UTR_premature_start_codon_gain_variant	SWI2/SNF2-containing protein RAD5
TGME49_318480	p.Gly1426Val	missense_variant	SWI2/SNF2-containing protein RAD5
TGME49_248100	c.-2984T>G	5_prime_UTR_premature_start_codon_gain_variant	synaptobrevin protein
TGME49_248100	c.459+6A>G	splice_region_variant	synaptobrevin protein
TGME49_297500	c.1423+6A>C	splice_region_variant	T-complex protein 1 eta subunit
TGME49_297500	c.453-6C>T	splice_region_variant	T-complex protein 1 eta subunit
TGME49_297500	c.-405C>G	5_prime_UTR_premature_start_codon_gain_variant	T-complex protein 1 eta subunit
TGME49_218250	c.964-4A>G	splice_region_variant	TAP42 family protein
TGME49_218250	p.Gly46Cys	missense_variant	TAP42 family protein
TGME49_250680	p.Arg877His	missense_variant	TBC domain-containing kinase (incomplete catalytic triad)
TGME49_250680	p.Met859Val	missense_variant	TBC domain-containing kinase (incomplete catalytic triad)
TGME49_250680	p.Ala768Ser	missense_variant	TBC domain-containing kinase (incomplete catalytic triad)
TGME49_250680	p.Val703Met	missense_variant	TBC domain-containing kinase (incomplete catalytic triad)
TGME49_221710	p.Phe174Ser	missense_variant	TBC domain-containing protein
TGME49_239830	p.Val981Ile	missense_variant	TBC domain-containing protein
TGME49_239830	p.Val1162Ala	missense_variant	TBC domain-containing protein
TGME49_218870	p.Ser38Pro	missense_variant	TBC domain-containing protein
TGME49_217660	p.Val22Leu	missense_variant	Tctex-1 family protein
TGME49_278790	c.-242C>T	5_prime_UTR_premature_start_codon_gain_variant	Tctex2-related light chain
TGME49_278790	p.Leu55Ile	missense_variant	Tctex2-related light chain
TGME49_254840	p.Thr397Ser	missense_variant	tetratricopeptide repeat-containing protein
TGME49_254840	p.Glu357Asp	missense_variant	tetratricopeptide repeat-containing protein
TGME49_254840	c.-446T>G	5_prime_UTR_premature_start_codon_gain_variant	tetratricopeptide repeat-containing protein
TGME49_254840	c.-2032A>G	5_prime_UTR_premature_start_codon_gain_variant	tetratricopeptide repeat-containing protein
TGME49_254840	c.-2937G>A	5_prime_UTR_premature_start_codon_gain_variant	tetratricopeptide repeat-containing protein
TGME49_254840	c.-3201A>G	5_prime_UTR_premature_start_codon_gain_variant	tetratricopeptide repeat-containing protein
TGME49_211370	p.Cys55Ser	missense_variant	tetratricopeptide repeat-containing protein
TGME49_211370	p.Cys55Phe	missense_variant	tetratricopeptide repeat-containing protein
TGME49_211370	p.His360Gln	missense_variant	tetratricopeptide repeat-containing protein
TGME49_211370	p.Ala393Thr	missense_variant	tetratricopeptide repeat-containing protein
TGME49_223710	p.Glu862Asp	missense_variant	tetratricopeptide repeat-containing protein
TGME49_247000	p.Leu4Val	missense_variant	tetratricopeptide repeat-containing protein
TGME49_247000	p.Lys136Asn	missense_variant	tetratricopeptide repeat-containing protein
TGME49_247000	p.Glu653Asp	missense_variant	tetratricopeptide repeat-containing protein
TGME49_247000	p.Gln741Arg	missense_variant	tetratricopeptide repeat-containing protein
TGME49_251410	p.Ala1659Thr	missense_variant	tetratricopeptide repeat-containing protein
TGME49_251410	p.Pro1051Ser	missense_variant	tetratricopeptide repeat-containing protein
TGME49_251410	p.Glu1027Lys	missense_variant	tetratricopeptide repeat-containing protein
TGME49_251410	p.Ala262Ser	missense_variant	tetratricopeptide repeat-containing protein
TGME49_251410	p.Leu63Pro	missense_variant	tetratricopeptide repeat-containing protein
TGME49_251410	c.-10G>T	5_prime_UTR_premature_start_codon_gain_variant	tetratricopeptide repeat-containing protein
TGME49_278630	p.Met134Val	missense_variant	tetratricopeptide repeat-containing protein
TGME49_276960	p.Asn84Lys	missense_variant	tetratricopeptide repeat-containing protein
TGME49_215250	p.Glu140Gly	missense_variant	thiamin pyrophosphokinase, catalytic domain-containing protein
TGME49_215250	p.Pro339Gln	missense_variant	thiamin pyrophosphokinase, catalytic domain-containing protein
TGME49_212100	c.-1633C>T	5_prime_UTR_premature_start_codon_gain_variant	ThiF family protein
TGME49_212100	c.-667C>G	5_prime_UTR_premature_start_codon_gain_variant	ThiF family protein
TGME49_212100	p.Leu32Phe	missense_variant	ThiF family protein
TGME49_212100	p.Gly259Arg	missense_variant	ThiF family protein
TGME49_212100	p.Gln375Glu	missense_variant&splice_region_variant	ThiF family protein
TGME49_212100	p.Ser453Thr	missense_variant	ThiF family protein
TGME49_212100	p.Arg508Cys	missense_variant	ThiF family protein
TGME49_212100	p.Arg611Gln	missense_variant	ThiF family protein
TGME49_212100	p.Lys1036Thr	missense_variant	ThiF family protein
TGME49_212100	p.Val1059Ile	missense_variant	ThiF family protein
TGME49_308050	c.-273G>T	5_prime_UTR_premature_start_codon_gain_variant	thioredoxin domain-containing protein
TGME49_247660	p.Val43Leu	missense_variant	thioredoxin domain-containing protein
TGME49_224060	p.Glu570Asp	missense_variant	thioredoxin, putative
TGME49_300260	p.Ser37Thr	missense_variant	threonyl-tRNA synthetase family protein
TGME49_300260	p.His679Arg	missense_variant	threonyl-tRNA synthetase family protein
TGME49_277910	p.Glu864Asp	missense_variant	thrombospondin type 1 domain-containing protein
TGME49_293368	p.Arg434His	missense_variant	TIP49 C-terminus family protein
TGME49_262390	p.Ala291Val	missense_variant	TLD protein
TGME49_237280	p.Thr932Met	missense_variant	TLD protein
TGME49_237280	p.Thr1473Ala	missense_variant	TLD protein
TGME49_237280	p.Gly1680Val	missense_variant	TLD protein
TGME49_253480	c.-747G>T	5_prime_UTR_premature_start_codon_gain_variant	topoisomerase VIA, putative
TGME49_253480	c.-526G>C	5_prime_UTR_premature_start_codon_gain_variant	topoisomerase VIA, putative
TGME49_253480	c.-213A>G	5_prime_UTR_premature_start_codon_gain_variant	topoisomerase VIA, putative
TGME49_214080	p.Ile115Val	missense_variant	toxofilin
TGME49_278430	p.Gly241Glu	missense_variant	Toxoplasma gondii family A protein
TGME49_278430	p.Gly237Val	missense_variant	Toxoplasma gondii family A protein
TGME49_278430	p.Asn132Ser	missense_variant	Toxoplasma gondii family A protein
TGME49_278430	p.Glu46Lys	missense_variant	Toxoplasma gondii family A protein

TGME49_278420	p.Glu311Gln	missense_variant	Toxoplasma gondii family A protein
TGME49_278420	p.Ile256Val	missense_variant	Toxoplasma gondii family A protein
TGME49_278420	p.Ala252Thr	missense_variant	Toxoplasma gondii family A protein
TGME49_278420	p.Ser86Thr	missense_variant	Toxoplasma gondii family A protein
TGME49_278420	p.His20Arg	missense_variant	Toxoplasma gondii family A protein
TGME49_278410	p.Gln321His	missense_variant	Toxoplasma gondii family A protein
TGME49_278410	p.Ala306Val	missense_variant	Toxoplasma gondii family A protein
TGME49_278410	p.Gln283His	missense_variant	Toxoplasma gondii family A protein
TGME49_278410	p.Ser256Asn	missense_variant	Toxoplasma gondii family A protein
TGME49_278410	p.Leu149Met	missense_variant	Toxoplasma gondii family A protein
TGME49_278410	p.Leu6Val	missense_variant	Toxoplasma gondii family A protein
TGME49_278410	c.-653A>G	5_prime_UTR_premature_start_codon_gain_variant	Toxoplasma gondii family A protein
TGME49_278400	p.His187Asn	missense_variant	Toxoplasma gondii family A protein
TGME49_278400	p.Asp162Glu	missense_variant	Toxoplasma gondii family A protein
TGME49_278400	p.Val62Ala	missense_variant	Toxoplasma gondii family A protein
TGME49_278400	c.-86A>G	5_prime_UTR_premature_start_codon_gain_variant	Toxoplasma gondii family A protein
TGME49_278400	c.-190A>C	5_prime_UTR_premature_start_codon_gain_variant	Toxoplasma gondii family A protein
TGME49_278400	c.-304G>T	5_prime_UTR_premature_start_codon_gain_variant	Toxoplasma gondii family A protein
TGME49_278390	p.Asn288Lys	missense_variant	Toxoplasma gondii family A protein
TGME49_278390	p.Pro218Ala	missense_variant	Toxoplasma gondii family A protein
TGME49_278380	p.Val324Ile	missense_variant	Toxoplasma gondii family A protein
TGME49_278380	p.Gly276Arg	missense_variant	Toxoplasma gondii family A protein
TGME49_278380	c.-86A>G	5_prime_UTR_premature_start_codon_gain_variant	Toxoplasma gondii family A protein
TGME49_278370	c.-399T>C	5_prime_UTR_premature_start_codon_gain_variant	Toxoplasma gondii family A protein
TGME49_278365	c.*852C>T	splice_region_variant	Toxoplasma gondii family A protein
TGME49_278365	p.Ser340Pro	missense_variant	Toxoplasma gondii family A protein
TGME49_278365	p.Gly292Ser	missense_variant	Toxoplasma gondii family A protein
TGME49_278365	p.Gln245His	missense_variant	Toxoplasma gondii family A protein
TGME49_278365	p.Thr233Pro	missense_variant	Toxoplasma gondii family A protein
TGME49_278365	p.Asn215Thr	missense_variant	Toxoplasma gondii family A protein
TGME49_278365	p.Asp205Gly	missense_variant	Toxoplasma gondii family A protein
TGME49_278365	p.Ser187Asn	missense_variant	Toxoplasma gondii family A protein
TGME49_278365	p.Ala39Ser	missense_variant	Toxoplasma gondii family A protein
TGME49_278365	c.-304C>T	5_prime_UTR_premature_start_codon_gain_variant	Toxoplasma gondii family A protein
TGME49_278365	c.-537C>T	5_prime_UTR_premature_start_codon_gain_variant	Toxoplasma gondii family A protein
TGME49_278365	c.-667C>T	5_prime_UTR_premature_start_codon_gain_variant	Toxoplasma gondii family A protein
TGME49_278365	c.-701C>T	5_prime_UTR_premature_start_codon_gain_variant	Toxoplasma gondii family A protein
TGME49_278360	p.Phe612Leu	missense_variant	Toxoplasma gondii family A protein
TGME49_278360	p.Val574Ala	missense_variant	Toxoplasma gondii family A protein
TGME49_278360	p.Ala563Thr	missense_variant	Toxoplasma gondii family A protein
TGME49_278360	p.Ser525Pro	missense_variant	Toxoplasma gondii family A protein
TGME49_278360	p.Met447Leu	missense_variant	Toxoplasma gondii family A protein
TGME49_278360	p.Ser431Ala	missense_variant	Toxoplasma gondii family A protein
TGME49_278360	p.Asn319Thr	missense_variant	Toxoplasma gondii family A protein
TGME49_278360	p.Asn319Asp	missense_variant	Toxoplasma gondii family A protein
TGME49_278360	p.Ser221Thr	missense_variant	Toxoplasma gondii family A protein
TGME49_278360	p.Gly211Ser	missense_variant	Toxoplasma gondii family A protein
TGME49_278360	p.Glu199Asp	missense_variant	Toxoplasma gondii family A protein
TGME49_278360	p.Lys130Thr	missense_variant	Toxoplasma gondii family A protein
TGME49_278360	p.Ser124Arg	missense_variant	Toxoplasma gondii family A protein
TGME49_278360	p.Thr59Ser	missense_variant	Toxoplasma gondii family A protein
TGME49_278360	p.Asp47Glu	missense_variant	Toxoplasma gondii family A protein
TGME49_278360	c.-189C>T	5_prime_UTR_premature_start_codon_gain_variant	Toxoplasma gondii family A protein
TGME49_278360	c.-572C>G	5_prime_UTR_premature_start_codon_gain_variant	Toxoplasma gondii family A protein
TGME49_278350	p.Ala606Val	missense_variant	Toxoplasma gondii family A protein
TGME49_278350	p.Val586Ile	missense_variant	Toxoplasma gondii family A protein
TGME49_278350	p.Glu570Gln	missense_variant	Toxoplasma gondii family A protein
TGME49_278350	p.Gly558Ser	missense_variant	Toxoplasma gondii family A protein
TGME49_278350	p.Ala498Thr	missense_variant	Toxoplasma gondii family A protein
TGME49_278350	p.Ala494Thr	missense_variant	Toxoplasma gondii family A protein
TGME49_278350	p.Asn490Lys	missense_variant	Toxoplasma gondii family A protein
TGME49_278350	p.Ser473Asn	missense_variant	Toxoplasma gondii family A protein
TGME49_278350	p.Pro470Ser	missense_variant	Toxoplasma gondii family A protein
TGME49_278350	p.Lys444Arg	missense_variant	Toxoplasma gondii family A protein
TGME49_278350	p.Glu415Gly	missense_variant	Toxoplasma gondii family A protein
TGME49_278350	p.Lys414Arg	missense_variant	Toxoplasma gondii family A protein
TGME49_278350	p.Glu404Asp	missense_variant	Toxoplasma gondii family A protein
TGME49_278350	p.Glu373Asp	missense_variant	Toxoplasma gondii family A protein
TGME49_278350	p.Ser361Thr	missense_variant	Toxoplasma gondii family A protein
TGME49_278350	p.Glu346Gln	missense_variant	Toxoplasma gondii family A protein
TGME49_278350	p.Thr331Ile	missense_variant	Toxoplasma gondii family A protein
TGME49_278350	p.Asn318Ile	missense_variant	Toxoplasma gondii family A protein
TGME49_278350	p.Asp313Glu	missense_variant	Toxoplasma gondii family A protein
TGME49_278350	p.Ser262Arg	missense_variant	Toxoplasma gondii family A protein
TGME49_278350	p.Ser262Gly	missense_variant	Toxoplasma gondii family A protein
TGME49_278350	p.Pro259Thr	missense_variant	Toxoplasma gondii family A protein
TGME49_278350	p.Ala213Ser	missense_variant	Toxoplasma gondii family A protein
TGME49_278350	p.Val51Ile	missense_variant	Toxoplasma gondii family A protein
TGME49_278350	p.Ile45Thr	missense_variant	Toxoplasma gondii family A protein
TGME49_278350	p.Thr37Ser	missense_variant	Toxoplasma gondii family A protein
TGME49_278340	p.Ser653Pro	missense_variant	Toxoplasma gondii family A protein
TGME49_278340	p.Glu596Asp	missense_variant	Toxoplasma gondii family A protein
TGME49_278340	p.Glu535Asp	missense_variant	Toxoplasma gondii family A protein
TGME49_278340	p.Pro500Leu	missense_variant	Toxoplasma gondii family A protein
TGME49_278340	p.Ala498Asp	missense_variant	Toxoplasma gondii family A protein

TGME49_278340	p.Ala398Val	missense_variant	Toxoplasma gondii family A protein
TGME49_278340	p.His397Arg	missense_variant	Toxoplasma gondii family A protein
TGME49_278340	p.Val275Ile	missense_variant	Toxoplasma gondii family A protein
TGME49_278340	p.Glu260Gly	missense_variant	Toxoplasma gondii family A protein
TGME49_278340	p.Lys258Thr	missense_variant	Toxoplasma gondii family A protein
TGME49_278340	p.Ala256Val	missense_variant	Toxoplasma gondii family A protein
TGME49_278340	p.Asp194Glu	missense_variant	Toxoplasma gondii family A protein
TGME49_278340	p.Gly131Glu	missense_variant	Toxoplasma gondii family A protein
TGME49_278340	p.Ala82Val	missense_variant	Toxoplasma gondii family A protein
TGME49_278340	p.Glu81Asp	missense_variant	Toxoplasma gondii family A protein
TGME49_278340	c.-270G>T	5_prime_UTR_premature_start_codon_gain_variant	Toxoplasma gondii family A protein
TGME49_278330	p.Val237Ala	missense_variant	Toxoplasma gondii family A protein
TGME49_278330	p.Lys213Glu	missense_variant	Toxoplasma gondii family A protein
TGME49_278320	p.Ser81Phe	missense_variant	Toxoplasma gondii family A protein
TGME49_278320	p.Val8Ile	missense_variant	Toxoplasma gondii family A protein
TGME49_278300	p.Arg91Leu	missense_variant	Toxoplasma gondii family A protein
TGME49_278300	p.Arg7Gln	missense_variant	Toxoplasma gondii family A protein
TGME49_278290	p.Ser279Pro	missense_variant	Toxoplasma gondii family A protein
TGME49_278290	p.Ser109Arg	missense_variant	Toxoplasma gondii family A protein
TGME49_278290	p.Gly36Glu	missense_variant	Toxoplasma gondii family A protein
TGME49_278100	c.-1384C>G	5_prime_UTR_premature_start_codon_gain_variant	Toxoplasma gondii family A protein
TGME49_224660	c.-812G>T	5_prime_UTR_premature_start_codon_gain_variant	transcription factor s-ii (tfiis), central domain-containing protein
TGME49_224660	p.Arg1060Gly	missense_variant	transcription factor s-ii (tfiis), central domain-containing protein
TGME49_224660	p.Asp1122Asn	missense_variant	transcription factor s-ii (tfiis), central domain-containing protein
TGME49_224660	p.Val1136Ala	missense_variant	transcription factor s-ii (tfiis), central domain-containing protein
TGME49_318260	p.Thr559Ala	missense_variant	transcription initiation factor TFIID subunit TAF5
TGME49_221670	c.1481-3C>T	splice_region_variant	transcriptional elongation factor FACT140
TGME49_221670	c.-465C>T	5_prime_UTR_premature_start_codon_gain_variant	transcriptional elongation factor FACT140
TGME49_223970	c.-1372C>T	5_prime_UTR_premature_start_codon_gain_variant	translation elongation and release factors (gtpases), putative
TGME49_223970	c.-978G>A	5_prime_UTR_premature_start_codon_gain_variant	translation elongation and release factors (gtpases), putative
TGME49_223970	p.Pro56Leu	missense_variant	translation elongation and release factors (gtpases), putative
TGME49_223970	p.Ser111Pro	missense_variant	translation elongation and release factors (gtpases), putative
TGME49_214270	c.3175-5T>C	splice_region_variant	translation initiation factor IF-2, putative
TGME49_214270	p.Thr493Ser	missense_variant	translation initiation factor IF-2, putative
TGME49_214270	c.102+7G>A	splice_region_variant	translation initiation factor IF-2, putative
TGME49_224235	p.Thr5Arg	missense_variant	translation initiation factor IF-3 protein
TGME49_224235	p.Val19Ala	missense_variant	translation initiation factor IF-3 protein
TGME49_224235	p.Arg61Gly	missense_variant	translation initiation factor IF-3 protein
TGME49_224235	p.His115Arg	missense_variant	translation initiation factor IF-3 protein
TGME49_211410	p.Lys226Ile	missense_variant	translation initiation factor sui1 protein
TGME49_211410	p.Met573Leu	missense_variant	translation initiation factor sui1 protein
TGME49_235130	c.-2456G>T	5_prime_UTR_premature_start_codon_gain_variant	transmembrane protein
TGME49_235130	c.-1368T>C	5_prime_UTR_premature_start_codon_gain_variant	transmembrane protein
TGME49_297245	p.Ala475Val	missense_variant	transporter, major facilitator family protein
TGME49_297245	p.Val457Leu	missense_variant	transporter, major facilitator family protein
TGME49_297245	p.Ala444Thr	missense_variant	transporter, major facilitator family protein
TGME49_297245	p.Pro73His	missense_variant	transporter, major facilitator family protein
TGME49_253130	p.Ile435Val	missense_variant	transporter, major facilitator family protein
TGME49_253130	c.953+8G>C	splice_region_variant	transporter, major facilitator family protein
TGME49_253700	p.Gln931Pro	missense_variant	transporter, major facilitator family protein
TGME49_253700	c.1883-6G>A	splice_region_variant	transporter, major facilitator family protein
TGME49_253700	c.-240T>G	5_prime_UTR_premature_start_codon_gain_variant	transporter, major facilitator family protein
TGME49_319740	p.Ala274Val	missense_variant	transporter, major facilitator family protein
TGME49_319740	p.Asp397Gly	missense_variant	transporter, major facilitator family protein
TGME49_319740	p.Glu403Gly	missense_variant	transporter, major facilitator family protein
TGME49_319740	p.Gln441His	missense_variant	transporter, major facilitator family protein
TGME49_318150	c.-1444A>G	5_prime_UTR_premature_start_codon_gain_variant	transporter, major facilitator family protein
TGME49_248840	p.Gly703Ser	missense_variant	transporter, major facilitator family protein
TGME49_234410	c.-369C>T	5_prime_UTR_premature_start_codon_gain_variant	transporter, small conductance mechanosensitive ion channel (MscS) family protein
TGME49_234410	c.-100A>T	5_prime_UTR_premature_start_codon_gain_variant	transporter, small conductance mechanosensitive ion channel (MscS) family protein
TGME49_234410	p.Ala79Val	missense_variant	transporter, small conductance mechanosensitive ion channel (MscS) family protein
TGME49_234410	p.Ser158Gly	missense_variant	transporter, small conductance mechanosensitive ion channel (MscS) family protein
TGME49_234410	p.Ile244Phe	missense_variant	transporter, small conductance mechanosensitive ion channel (MscS) family protein
TGME49_234410	p.Ala269Thr	missense_variant	transporter, small conductance mechanosensitive ion channel (MscS) family protein
TGME49_234410	p.Val345Ile	missense_variant	transporter, small conductance mechanosensitive ion channel (MscS) family protein
TGME49_234410	p.His1573Tyr	missense_variant	transporter, small conductance mechanosensitive ion channel (MscS) family protein
TGME49_234410	p.His1643Arg	missense_variant	transporter, small conductance mechanosensitive ion channel (MscS) family protein
TGME49_234410	p.Leu1689Ser	missense_variant	transporter, small conductance mechanosensitive ion channel (MscS) family protein
TGME49_234410	p.Thr1997Ala	missense_variant	transporter, small conductance mechanosensitive ion channel (MscS) family protein
TGME49_234410	p.Ser2561Asn	missense_variant	transporter, small conductance mechanosensitive ion channel (MscS) family protein
TGME49_234410	c.8599-5C>T	splice_region_variant	transporter, small conductance mechanosensitive ion channel (MscS) family protein
TGME49_234410	p.Leu3246Phe	missense_variant	transporter, small conductance mechanosensitive ion channel (MscS) family protein
TGME49_219650	c.7783-6C>A	splice_region_variant	transporter, small conductance mechanosensitive ion channel (MscS) family protein
TGME49_219650	p.Asn2107Asp	missense_variant	transporter, small conductance mechanosensitive ion channel (MscS) family protein
TGME49_219650	p.Lys1626Asn	missense_variant	transporter, small conductance mechanosensitive ion channel (MscS) family protein
TGME49_219650	p.Leu1523Val	missense_variant	transporter, small conductance mechanosensitive ion channel (MscS) family protein
TGME49_219650	p.Ser1334Arg	missense_variant	transporter, small conductance mechanosensitive ion channel (MscS) family protein
TGME49_219650	p.Glu1298Gly	missense_variant	transporter, small conductance mechanosensitive ion channel (MscS) family protein
TGME49_219650	p.Glu1275Gln	missense_variant	transporter, small conductance mechanosensitive ion channel (MscS) family protein
TGME49_219650	p.Ser1149Leu	missense_variant	transporter, small conductance mechanosensitive ion channel (MscS) family protein
TGME49_219650	p.Arg1034Thr	missense_variant	transporter, small conductance mechanosensitive ion channel (MscS) family protein
TGME49_219650	p.Ser1022Asn	missense_variant	transporter, small conductance mechanosensitive ion channel (MscS) family protein
TGME49_219650	p.Lys842Asn	missense_variant	transporter, small conductance mechanosensitive ion channel (MscS) family protein
TGME49_219650	p.Asp694Glu	missense_variant	transporter, small conductance mechanosensitive ion channel (MscS) family protein
TGME49_219650	c.1363-1G>T	splice_acceptor_variant	transporter, small conductance mechanosensitive ion channel (MscS) family protein

TGME49_253510	c.-858C>T	5_prime_UTR_premature_start_codon_gain_variant	transporter/permease protein
TGME49_253510	c.-129T>C	5_prime_UTR_premature_start_codon_gain_variant	transporter/permease protein
TGME49_297720	p.Val527Asp	missense_variant	trehalose-phosphatase
TGME49_297720	c.3096+7G>C	splice_region_variant	trehalose-phosphatase
TGME49_250750	p.Gly1063Arg	missense_variant	tRNA (uracil-5-)-methyltransferase
TGME49_250750	p.Asp821Asn	missense_variant	tRNA (uracil-5-)-methyltransferase
TGME49_250750	p.Ser646Pro	missense_variant	tRNA (uracil-5-)-methyltransferase
TGME49_250750	p.Thr573Ala	missense_variant	tRNA (uracil-5-)-methyltransferase
TGME49_250750	p.Gly283Glu	missense_variant	tRNA (uracil-5-)-methyltransferase
TGME49_250750	p.Glu214Gln	missense_variant	tRNA (uracil-5-)-methyltransferase
TGME49_250750	c.102+7A>G	splice_region_variant	tRNA (uracil-5-)-methyltransferase
TGME49_219430	c.-336T>G	5_prime_UTR_premature_start_codon_gain_variant	tRNA ligase class II core domain (G, H, P, S and T) domain-containing protein
TGME49_219430	p.Glu490Gln	missense_variant	tRNA ligase class II core domain (G, H, P, S and T) domain-containing protein
TGME49_219430	p.Asp831Glu	missense_variant	tRNA ligase class II core domain (G, H, P, S and T) domain-containing protein
TGME49_219430	p.Arg941Lys	missense_variant	tRNA ligase class II core domain (G, H, P, S and T) domain-containing protein
TGME49_224200	p.Ala489Ser	missense_variant	tRNA pseudouridine synthase
TGME49_224200	p.Arg403Lys	missense_variant	tRNA pseudouridine synthase
TGME49_224200	p.Gly356Asp	missense_variant	tRNA pseudouridine synthase
TGME49_224200	p.Asp341Asn	missense_variant	tRNA pseudouridine synthase
TGME49_224200	p.Gln258Arg	missense_variant	tRNA pseudouridine synthase
TGME49_224200	p.Thr230Ala	missense_variant	tRNA pseudouridine synthase
TGME49_224200	p.Lys228Thr	missense_variant	tRNA pseudouridine synthase
TGME49_224200	p.Gln48Lys	missense_variant	tRNA pseudouridine synthase
TGME49_224200	c.-92C>A	5_prime_UTR_premature_start_codon_gain_variant	tRNA pseudouridine synthase
TGME49_254110	p.Asn333His	missense_variant	tryptophanyl-tRNA synthetase (TrpRS1)
TGME49_318240	p.Thr1563Arg	missense_variant	Tubulin-tyrosine ligase family protein
TGME49_318240	p.Asp1081Asn	missense_variant	Tubulin-tyrosine ligase family protein
TGME49_253860	p.Thr2004Ala	missense_variant	Tyrosine kinase-like (TKL) protein
TGME49_253860	p.Pro1997Leu	missense_variant	Tyrosine kinase-like (TKL) protein
TGME49_253860	p.Pro1316Leu	missense_variant	Tyrosine kinase-like (TKL) protein
TGME49_253860	p.Glu334Gln	missense_variant	Tyrosine kinase-like (TKL) protein
TGME49_253860	p.Glu284Gly	missense_variant	Tyrosine kinase-like (TKL) protein
TGME49_301270	c.-108G>C	5_prime_UTR_premature_start_codon_gain_variant	Tyrosine kinase-like (TKL) protein
TGME49_301270	p.Phe165Tyr	missense_variant	Tyrosine kinase-like (TKL) protein
TGME49_301270	p.Arg894Met	missense_variant	Tyrosine kinase-like (TKL) protein
TGME49_301270	p.Leu909Met	missense_variant	Tyrosine kinase-like (TKL) protein
TGME49_301270	p.Leu1273Gln	missense_variant	Tyrosine kinase-like (TKL) protein
TGME49_301270	p.Thr1798Ala	missense_variant	Tyrosine kinase-like (TKL) protein
TGME49_301270	p.Ala2290Val	missense_variant	Tyrosine kinase-like (TKL) protein
TGME49_301270	p.Lys3131Gln	missense_variant	Tyrosine kinase-like (TKL) protein
TGME49_301270	p.Gln3554Pro	missense_variant	Tyrosine kinase-like (TKL) protein
TGME49_239130	p.Asp626His	missense_variant	Tyrosine kinase-like (TKL) protein
TGME49_239130	p.Arg703Leu	missense_variant	Tyrosine kinase-like (TKL) protein
TGME49_239130	p.Gly717Ala	missense_variant	Tyrosine kinase-like (TKL) protein
TGME49_239130	p.Arg837Cys	missense_variant	Tyrosine kinase-like (TKL) protein
TGME49_239130	p.Asn1001Ser	missense_variant	Tyrosine kinase-like (TKL) protein
TGME49_239130	p.Val1099Ile	missense_variant	Tyrosine kinase-like (TKL) protein
TGME49_239130	p.Arg1268Gly	missense_variant	Tyrosine kinase-like (TKL) protein
TGME49_234970	p.Ala57Val	missense_variant	Tyrosine kinase-like (TKL) protein
TGME49_234970	p.Gly114Ala	missense_variant	Tyrosine kinase-like (TKL) protein
TGME49_234970	c.1550-7T>C	splice_region_variant	Tyrosine kinase-like (TKL) protein
TGME49_234970	c.1550-4G>A	splice_region_variant	Tyrosine kinase-like (TKL) protein
TGME49_234970	p.Val988Ala	missense_variant	Tyrosine kinase-like (TKL) protein
TGME49_234970	p.Glu1160Asp	missense_variant	Tyrosine kinase-like (TKL) protein
TGME49_234970	c.4621-7T>G	splice_region_variant	Tyrosine kinase-like (TKL) protein
TGME49_234970	p.Pro1744Gln	missense_variant	Tyrosine kinase-like (TKL) protein
TGME49_234970	p.Ala1962Thr	missense_variant	Tyrosine kinase-like (TKL) protein
TGME49_236240	c.-847T>G	5_prime_UTR_premature_start_codon_gain_variant	Tyrosine kinase-like (TKL) protein
TGME49_236240	c.237+6T>C	splice_region_variant	Tyrosine kinase-like (TKL) protein
TGME49_236240	p.Arg182His	missense_variant	Tyrosine kinase-like (TKL) protein
TGME49_236240	p.Cys234Arg	missense_variant	Tyrosine kinase-like (TKL) protein
TGME49_236240	p.Gly722Arg	missense_variant	Tyrosine kinase-like (TKL) protein
TGME49_236240	p.Glu725Gln	missense_variant	Tyrosine kinase-like (TKL) protein
TGME49_236240	p.Leu739Ser	missense_variant	Tyrosine kinase-like (TKL) protein
TGME49_236240	c.*1272G>T	splice_region_variant	Tyrosine kinase-like (TKL) protein
TGME49_221980	p.Asp95Asn	missense_variant	U1 zinc finger protein
TGME49_221980	p.Pro274Ser	missense_variant	U1 zinc finger protein
TGME49_221980	p.Glu490Gly	missense_variant	U1 zinc finger protein
TGME49_234520	c.1389+8T>A	splice_region_variant	U2 snRNP auxilliary factor, large subunit, splicing factor subfamily protein
TGME49_253520	p.Glu675Asp	missense_variant	U3 small nucleolar RNA-associated protein 6 protein
TGME49_253520	p.Ile438Met	missense_variant	U3 small nucleolar RNA-associated protein 6 protein
TGME49_297140	c.-352T>C	5_prime_UTR_premature_start_codon_gain_variant	U6 snRNA-associated sm family protein Lsm2, putative
TGME49_297140	p.Pro7Leu	missense_variant	U6 snRNA-associated sm family protein Lsm2, putative
TGME49_249160	p.Arg723Cys	missense_variant	UAA transporter family protein
TGME49_249160	p.Pro526Arg	missense_variant	UAA transporter family protein
TGME49_249160	p.Thr231Met	missense_variant	UAA transporter family protein
TGME49_249160	p.Lys213Asn	missense_variant	UAA transporter family protein
TGME49_320220	c.1378+6T>C	splice_region_variant	ubiquinol cytochrome c oxidoreductase, putative
TGME49_320220	p.Cys32Tyr	missense_variant	ubiquinol cytochrome c oxidoreductase, putative
TGME49_298030	c.763-5C>G	splice_region_variant	Ubiquinol-cytochrome c chaperone, putative
TGME49_221610	c.-2931C>T	5_prime_UTR_premature_start_codon_gain_variant	ubiquitin carboxyl-terminal hydrolase
TGME49_221610	c.-79A>T	5_prime_UTR_premature_start_codon_gain_variant	ubiquitin carboxyl-terminal hydrolase
TGME49_221610	p.Arg3His	missense_variant	ubiquitin carboxyl-terminal hydrolase
TGME49_221610	p.Glu175Lys	missense_variant	ubiquitin carboxyl-terminal hydrolase
TGME49_221610	p.Val410Ile	missense_variant	ubiquitin carboxyl-terminal hydrolase

TGME49_210781	p.Cys24Ser	missense_variant	ubiquitin carboxyl-terminal hydrolase
TGME49_210781	p.Pro166Ala	missense_variant	ubiquitin carboxyl-terminal hydrolase
TGME49_210781	p.Pro428His	missense_variant	ubiquitin carboxyl-terminal hydrolase
TGME49_210781	p.Ala536Val	missense_variant	ubiquitin carboxyl-terminal hydrolase
TGME49_210781	p.Ala1128Val	missense_variant	ubiquitin carboxyl-terminal hydrolase
TGME49_210781	p.Glu1133Asp	missense_variant	ubiquitin carboxyl-terminal hydrolase
TGME49_210781	p.Ala1500Asp	missense_variant	ubiquitin carboxyl-terminal hydrolase
TGME49_210781	p.Gly3187Ser	missense_variant	ubiquitin carboxyl-terminal hydrolase
TGME49_223450	p.Glu2346Gly	missense_variant	ubiquitin carboxyl-terminal hydrolase
TGME49_223450	p.Asp1615Gly	missense_variant	ubiquitin carboxyl-terminal hydrolase
TGME49_223450	p.Arg1366Ser	missense_variant	ubiquitin carboxyl-terminal hydrolase
TGME49_223450	p.Ala939Val	missense_variant	ubiquitin carboxyl-terminal hydrolase
TGME49_223450	p.Ala939Ser	missense_variant	ubiquitin carboxyl-terminal hydrolase
TGME49_223450	p.Glu862Lys	missense_variant	ubiquitin carboxyl-terminal hydrolase
TGME49_223450	p.Thr756Asn	missense_variant	ubiquitin carboxyl-terminal hydrolase
TGME49_223450	p.Ser254Thr	missense_variant	ubiquitin carboxyl-terminal hydrolase
TGME49_223450	p.Cys186Trp	missense_variant	ubiquitin carboxyl-terminal hydrolase
TGME49_223450	p.Asn161Thr	missense_variant	ubiquitin carboxyl-terminal hydrolase
TGME49_223450	p.Ser113Gly	missense_variant	ubiquitin carboxyl-terminal hydrolase
TGME49_223450	c.-1266C>T	5_prime_UTR_premature_start_codon_gain_variant	ubiquitin carboxyl-terminal hydrolase
TGME49_277895	p.Pro79Leu	missense_variant	ubiquitin carboxyl-terminal hydrolase
TGME49_277895	p.Gly542Arg	missense_variant	ubiquitin carboxyl-terminal hydrolase
TGME49_277895	p.Ser650Ala	missense_variant	ubiquitin carboxyl-terminal hydrolase
TGME49_277895	p.Gly752Glu	missense_variant	ubiquitin carboxyl-terminal hydrolase
TGME49_277895	p.Ala944Thr	missense_variant	ubiquitin carboxyl-terminal hydrolase
TGME49_277895	p.Asp990Tyr	missense_variant	ubiquitin carboxyl-terminal hydrolase
TGME49_277895	p.Gly994Trp	missense_variant	ubiquitin carboxyl-terminal hydrolase
TGME49_277895	p.Lys1002Thr	missense_variant	ubiquitin carboxyl-terminal hydrolase
TGME49_277895	p.Met1005Val	missense_variant	ubiquitin carboxyl-terminal hydrolase
TGME49_277895	p.Ala1341Ser	missense_variant	ubiquitin carboxyl-terminal hydrolase
TGME49_277895	p.Thr1447Met	missense_variant	ubiquitin carboxyl-terminal hydrolase
TGME49_277895	p.Met1582Val	missense_variant	ubiquitin carboxyl-terminal hydrolase
TGME49_277895	p.Lys1663Arg	missense_variant	ubiquitin carboxyl-terminal hydrolase
TGME49_277895	p.Ala1683Thr	missense_variant	ubiquitin carboxyl-terminal hydrolase
TGME49_277895	p.Val2878Ile	missense_variant	ubiquitin carboxyl-terminal hydrolase
TGME49_247240	c.970-5C>T	splice_region_variant	ubiquitin carboxyl-terminal hydrolase, family 1 protein
TGME49_247240	p.Glu340Asp	missense_variant	ubiquitin carboxyl-terminal hydrolase, family 1 protein
TGME49_319870	c.838+5T>G	splice_region_variant	ubiquitin-conjugating enzyme subfamily protein
TGME49_319870	c.517-4G>T	splice_region_variant	ubiquitin-conjugating enzyme subfamily protein
TGME49_251640	c.316-7T>C	splice_region_variant	ubiquitin-conjugating enzyme subfamily protein
TGME49_248460	c.-382G>T	5_prime_UTR_premature_start_codon_gain_variant	ubiquitin, putative
TGME49_248460	p.Glu26Asp	missense_variant	ubiquitin, putative
TGME49_235750	c.727-4T>G	splice_region_variant	ULK kinase
TGME49_214470	p.Pro210Leu	missense_variant	Ulp1 protease family, C-terminal catalytic domain-containing protein
TGME49_214470	p.Pro210Ser	missense_variant	Ulp1 protease family, C-terminal catalytic domain-containing protein
TGME49_251510	p.Ala1673Ser	missense_variant	Ulp1 protease family, C-terminal catalytic domain-containing protein
TGME49_251510	p.Lys1655Met	missense_variant	Ulp1 protease family, C-terminal catalytic domain-containing protein
TGME49_251510	p.Lys1655*	stop_gained	Ulp1 protease family, C-terminal catalytic domain-containing protein
TGME49_251510	p.Ala1653Pro	missense_variant	Ulp1 protease family, C-terminal catalytic domain-containing protein
TGME49_251510	p.Asp1563Gly	missense_variant	Ulp1 protease family, C-terminal catalytic domain-containing protein
TGME49_251510	p.Gly1471Glu	missense_variant	Ulp1 protease family, C-terminal catalytic domain-containing protein
TGME49_251510	p.Leu1222Phe	missense_variant	Ulp1 protease family, C-terminal catalytic domain-containing protein
TGME49_251510	p.Gln1108Glu	missense_variant	Ulp1 protease family, C-terminal catalytic domain-containing protein
TGME49_251510	p.Val1107Glu	missense_variant	Ulp1 protease family, C-terminal catalytic domain-containing protein
TGME49_251510	p.Ala1106Gly	missense_variant	Ulp1 protease family, C-terminal catalytic domain-containing protein
TGME49_251510	p.Ala1069Thr	missense_variant	Ulp1 protease family, C-terminal catalytic domain-containing protein
TGME49_251510	p.Glu1068Asp	missense_variant	Ulp1 protease family, C-terminal catalytic domain-containing protein
TGME49_251510	p.Val1048Ala	missense_variant	Ulp1 protease family, C-terminal catalytic domain-containing protein
TGME49_251510	p.Asn1047Asp	missense_variant	Ulp1 protease family, C-terminal catalytic domain-containing protein
TGME49_251510	p.Ala1021Val	missense_variant	Ulp1 protease family, C-terminal catalytic domain-containing protein
TGME49_251510	p.Glu904Gln	missense_variant	Ulp1 protease family, C-terminal catalytic domain-containing protein
TGME49_251510	p.Pro820Leu	missense_variant	Ulp1 protease family, C-terminal catalytic domain-containing protein
TGME49_251510	p.Arg717Gln	missense_variant	Ulp1 protease family, C-terminal catalytic domain-containing protein
TGME49_251510	p.Asp505Glu	missense_variant	Ulp1 protease family, C-terminal catalytic domain-containing protein
TGME49_251510	p.Lys504Gln	missense_variant	Ulp1 protease family, C-terminal catalytic domain-containing protein
TGME49_251510	p.Ala469Val	missense_variant	Ulp1 protease family, C-terminal catalytic domain-containing protein
TGME49_251510	p.Ala356Ser	missense_variant	Ulp1 protease family, C-terminal catalytic domain-containing protein
TGME49_251510	p.His329Gln	missense_variant	Ulp1 protease family, C-terminal catalytic domain-containing protein
TGME49_251510	p.Ser232Cys	missense_variant	Ulp1 protease family, C-terminal catalytic domain-containing protein
TGME49_251510	p.Pro139Gln	missense_variant	Ulp1 protease family, C-terminal catalytic domain-containing protein
TGME49_277550	p.Gln33His	missense_variant	UvrD/REP helicase domain-containing protein
TGME49_277550	p.Glu217Gly	missense_variant	UvrD/REP helicase domain-containing protein
TGME49_277550	p.Ala256Asp	missense_variant	UvrD/REP helicase domain-containing protein
TGME49_277550	p.Thr324Lys	missense_variant	UvrD/REP helicase domain-containing protein
TGME49_277550	p.Ser472Pro	missense_variant	UvrD/REP helicase domain-containing protein
TGME49_277550	p.Thr962Lys	missense_variant	UvrD/REP helicase domain-containing protein
TGME49_277550	p.Ala979Ser	missense_variant	UvrD/REP helicase domain-containing protein
TGME49_277550	p.Ala1138Val	missense_variant	UvrD/REP helicase domain-containing protein
TGME49_277550	p.Gly1268Arg	missense_variant	UvrD/REP helicase domain-containing protein
TGME49_277550	p.Ile1527Asn	missense_variant	UvrD/REP helicase domain-containing protein
TGME49_277550	p.Glu1677Gln	missense_variant	UvrD/REP helicase domain-containing protein
TGME49_277550	p.Arg1853His	missense_variant	UvrD/REP helicase domain-containing protein
TGME49_277550	p.Asp2002His	missense_variant	UvrD/REP helicase domain-containing protein
TGME49_277550	p.Glu2131Asp	missense_variant	UvrD/REP helicase domain-containing protein
TGME49_277550	p.Glu2187Asp	missense_variant	UvrD/REP helicase domain-containing protein

TGME49_277550	p.Gln2669His	missense_variant	UvrD/REP helicase domain-containing protein
TGME49_277550	p.Ser2739Thr	missense_variant	UvrD/REP helicase domain-containing protein
TGME49_277550	p.Thr2927Ala	missense_variant	UvrD/REP helicase domain-containing protein
TGME49_277550	p.Ala2948Ser	missense_variant	UvrD/REP helicase domain-containing protein
TGME49_277550	p.Tyr2953His	missense_variant	UvrD/REP helicase domain-containing protein
TGME49_277550	p.Ser3126Phe	missense_variant	UvrD/REP helicase domain-containing protein
TGME49_277550	p.Ser3144Phe	missense_variant	UvrD/REP helicase domain-containing protein
TGME49_248670	c.-1802T>C	5_prime_UTR_premature_start_codon_gain_variant	V-type H(+)-translocating pyrophosphatase VP1
TGME49_248670	c.-350G>A	5_prime_UTR_premature_start_codon_gain_variant	V-type H(+)-translocating pyrophosphatase VP1
TGME49_212310	c.244+3G>T	splice_region_variant	vacuolar ATP synthetase
TGME49_297230	p.Ala1016Val	missense_variant	Vps53 family, N-terminal protein
TGME49_247790	p.Thr203Ile	missense_variant	VRR-NUC domain-containing protein
TGME49_247790	p.Glu390Asp	missense_variant	VRR-NUC domain-containing protein
TGME49_247790	p.Ser752Thr	missense_variant	VRR-NUC domain-containing protein
TGME49_247790	p.Val856Ala	missense_variant	VRR-NUC domain-containing protein
TGME49_247790	p.Val1012Leu	missense_variant	VRR-NUC domain-containing protein
TGME49_247790	p.Gly1243Asp	missense_variant	VRR-NUC domain-containing protein
TGME49_247790	p.Asp1668Gly	missense_variant	VRR-NUC domain-containing protein
TGME49_247790	p.Gly1671Arg	missense_variant	VRR-NUC domain-containing protein
TGME49_247790	p.Lys2027Glu	missense_variant	VRR-NUC domain-containing protein
TGME49_247790	p.Arg2054Pro	missense_variant	VRR-NUC domain-containing protein
TGME49_247790	p.Asn2759Ser	missense_variant	VRR-NUC domain-containing protein
TGME49_299080	p.Gln376Glu	missense_variant	VTC domain-containing protein
TGME49_320210	c.-1042C>A	5_prime_UTR_premature_start_codon_gain_variant	WD domain, G-beta repeat domain containing protein
TGME49_221890	p.Ala582Thr	missense_variant	WD domain, G-beta repeat-containing protein
TGME49_221890	c.1443+8G>C	splice_region_variant	WD domain, G-beta repeat-containing protein
TGME49_221890	c.1443+6T>C	splice_region_variant	WD domain, G-beta repeat-containing protein
TGME49_221890	c.1020+4G>A	splice_region_variant	WD domain, G-beta repeat-containing protein
TGME49_221890	c.27+2T>C	splice_donor_variant	WD domain, G-beta repeat-containing protein
TGME49_253060	p.Pro268Ser	missense_variant	WD domain, G-beta repeat-containing protein
TGME49_253060	p.Arg562Pro	missense_variant	WD domain, G-beta repeat-containing protein
TGME49_253060	p.Glu606Gly	missense_variant	WD domain, G-beta repeat-containing protein
TGME49_253060	p.Leu634Val	missense_variant	WD domain, G-beta repeat-containing protein
TGME49_319910	p.Arg388Gln	missense_variant	WD domain, G-beta repeat-containing protein
TGME49_319910	c.232-6G>A	splice_region_variant	WD domain, G-beta repeat-containing protein
TGME49_319910	c.-81C>G	5_prime_UTR_premature_start_codon_gain_variant	WD domain, G-beta repeat-containing protein
TGME49_319570	p.Ser236Thr	missense_variant	WD domain, G-beta repeat-containing protein
TGME49_319570	c.1095-5C>T	splice_region_variant	WD domain, G-beta repeat-containing protein
TGME49_223262	p.Ala96Thr	missense_variant	WD domain, G-beta repeat-containing protein
TGME49_214200	p.Asp1293Glu	missense_variant	WD domain, G-beta repeat-containing protein
TGME49_214200	p.Ser780Pro	missense_variant	WD domain, G-beta repeat-containing protein
TGME49_214200	p.Ser402Arg	missense_variant	WD domain, G-beta repeat-containing protein
TGME49_214200	c.-13G>T	5_prime_UTR_premature_start_codon_gain_variant	WD domain, G-beta repeat-containing protein
TGME49_219450	p.Arg1446Ser	missense_variant	WD domain, G-beta repeat-containing protein
TGME49_219450	p.Val1965Met	missense_variant	WD domain, G-beta repeat-containing protein
TGME49_219450	p.Ser2064Leu	missense_variant	WD domain, G-beta repeat-containing protein
TGME49_219450	p.Pro2095Ser	missense_variant	WD domain, G-beta repeat-containing protein
TGME49_218420	c.1048+8C>G	splice_region_variant	WD domain, G-beta repeat-containing protein
TGME49_218420	p.Thr2Ala	missense_variant	WD domain, G-beta repeat-containing protein
TGME49_245752	p.Thr1591Ile	missense_variant	WD domain, G-beta repeat-containing protein
TGME49_245752	p.Leu1581His	missense_variant	WD domain, G-beta repeat-containing protein
TGME49_245752	p.Ala1359Gly	missense_variant	WD domain, G-beta repeat-containing protein
TGME49_245752	p.Ser1135Ala	missense_variant	WD domain, G-beta repeat-containing protein
TGME49_245752	p.Asp1112Gly	missense_variant	WD domain, G-beta repeat-containing protein
TGME49_245752	p.Gln1100His	missense_variant	WD domain, G-beta repeat-containing protein
TGME49_245752	p.Ile1070Phe	missense_variant	WD domain, G-beta repeat-containing protein
TGME49_245752	p.Ser862Tyr	missense_variant	WD domain, G-beta repeat-containing protein
TGME49_245752	p.Val586Ala	missense_variant	WD domain, G-beta repeat-containing protein
TGME49_248290	p.Asp514His	missense_variant	WD domain, G-beta repeat-containing protein
TGME49_248290	p.Lys708Asn	missense_variant	WD domain, G-beta repeat-containing protein
TGME49_248290	p.Ser1002Gly	missense_variant	WD domain, G-beta repeat-containing protein
TGME49_248290	p.Leu1043Val	missense_variant	WD domain, G-beta repeat-containing protein
TGME49_248290	p.Pro1281Leu	missense_variant	WD domain, G-beta repeat-containing protein
TGME49_248290	p.Ile1336Val	missense_variant	WD domain, G-beta repeat-containing protein
TGME49_248290	p.Arg1392Leu	missense_variant	WD domain, G-beta repeat-containing protein
TGME49_248290	p.Ser1741Thr	missense_variant	WD domain, G-beta repeat-containing protein
TGME49_248290	p.Ser1741Leu	missense_variant	WD domain, G-beta repeat-containing protein
TGME49_248290	p.Pro2145Arg	missense_variant	WD domain, G-beta repeat-containing protein
TGME49_248290	p.Asp2268Asn	missense_variant	WD domain, G-beta repeat-containing protein
TGME49_248290	p.Asn2282His	missense_variant	WD domain, G-beta repeat-containing protein
TGME49_248290	p.Val2393Met	missense_variant	WD domain, G-beta repeat-containing protein
TGME49_248290	p.Thr2404Arg	missense_variant	WD domain, G-beta repeat-containing protein
TGME49_248290	p.Ala2444Thr	missense_variant	WD domain, G-beta repeat-containing protein
TGME49_248290	p.Pro2445Thr	missense_variant	WD domain, G-beta repeat-containing protein
TGME49_248290	p.Leu2495Pro	missense_variant	WD domain, G-beta repeat-containing protein
TGME49_248290	p.Lys2611Met	missense_variant	WD domain, G-beta repeat-containing protein
TGME49_248290	p.Pro2670Leu	missense_variant	WD domain, G-beta repeat-containing protein
TGME49_248290	p.Gly2901Asp	missense_variant	WD domain, G-beta repeat-containing protein
TGME49_248290	p.Ala3080Thr	missense_variant	WD domain, G-beta repeat-containing protein
TGME49_248290	p.Gly3137Arg	missense_variant	WD domain, G-beta repeat-containing protein
TGME49_248290	p.Glu3155Asp	missense_variant	WD domain, G-beta repeat-containing protein
TGME49_249460	p.Ala117Glu	missense_variant	WD domain, G-beta repeat-containing protein
TGME49_249460	p.Gln149Arg	missense_variant	WD domain, G-beta repeat-containing protein
TGME49_249460	p.Thr308Met	missense_variant	WD domain, G-beta repeat-containing protein
TGME49_249460	p.Pro745Leu	missense_variant	WD domain, G-beta repeat-containing protein

TGME49_249460	p.Glu967Gly	missense_variant	WD domain, G-beta repeat-containing protein
TGME49_249460	p.Glu970Gly	missense_variant	WD domain, G-beta repeat-containing protein
TGME49_249460	p.Gly1071Asp	missense_variant	WD domain, G-beta repeat-containing protein
TGME49_249460	p.Ala1123Thr	missense_variant	WD domain, G-beta repeat-containing protein
TGME49_249460	p.Ser1147Phe	missense_variant	WD domain, G-beta repeat-containing protein
TGME49_249460	p.Thr1196Ala	missense_variant	WD domain, G-beta repeat-containing protein
TGME49_249460	p.Thr1196Ile	missense_variant	WD domain, G-beta repeat-containing protein
TGME49_249460	p.Pro1336His	missense_variant	WD domain, G-beta repeat-containing protein
TGME49_278280	c.-744C>T	5_prime_UTR_premature_start_codon_gain_variant	WD domain, G-beta repeat-containing protein
TGME49_234610	c.-2299A>T	5_prime_UTR_premature_start_codon_gain_variant	WD-40 repeat protein
TGME49_234610	c.-657C>T	5_prime_UTR_premature_start_codon_gain_variant	WD-40 repeat protein
TGME49_251670	c.-829C>G	5_prime_UTR_premature_start_codon_gain_variant	werner helicase interacting protein 1, putative
TGME49_251670	p.Gly28Ser	missense_variant	werner helicase interacting protein 1, putative
TGME49_251670	p.Val160Asp	missense_variant	werner helicase interacting protein 1, putative
TGME49_251670	p.Leu200Val	missense_variant	werner helicase interacting protein 1, putative
TGME49_251670	p.Leu200His	missense_variant	werner helicase interacting protein 1, putative
TGME49_251670	p.Cys205Ser	missense_variant	werner helicase interacting protein 1, putative
TGME49_251670	p.Phe211Ser	missense_variant	werner helicase interacting protein 1, putative
TGME49_251670	p.His367Asn	missense_variant	werner helicase interacting protein 1, putative
TGME49_251670	p.Asp386Glu	missense_variant	werner helicase interacting protein 1, putative
TGME49_251670	p.Gly402Asp	missense_variant	werner helicase interacting protein 1, putative
TGME49_251670	p.Lys404Arg	missense_variant	werner helicase interacting protein 1, putative
TGME49_251670	p.Arg466Lys	missense_variant	werner helicase interacting protein 1, putative
TGME49_251670	p.Ala558Thr	missense_variant	werner helicase interacting protein 1, putative
TGME49_251670	c.1909-5T>C	splice_region_variant	werner helicase interacting protein 1, putative
TGME49_251670	p.Ser891Gly	missense_variant	werner helicase interacting protein 1, putative
TGME49_215440	p.Arg509Ile	missense_variant	WWE domain-containing protein
TGME49_215440	p.Ser302Asn	missense_variant	WWE domain-containing protein
TGME49_300650	p.Glu103Lys	missense_variant	ycf24 family protein, putative
TGME49_253170	p.Ser118Tyr	missense_variant	zinc carboxypeptidase, putative
TGME49_253170	c.2854+7T>G	splice_region_variant	zinc carboxypeptidase, putative
TGME49_253170	p.Arg1552Leu	missense_variant	zinc carboxypeptidase, putative
TGME49_253790	p.Ala40Val	missense_variant	zinc finger (CCCH type) motif-containing protein
TGME49_253790	p.Ser1284Leu	missense_variant	zinc finger (CCCH type) motif-containing protein
TGME49_224630	p.Gly1821Asp	missense_variant	zinc finger (CCCH type) motif-containing protein
TGME49_224630	p.Asp1751Val	missense_variant	zinc finger (CCCH type) motif-containing protein
TGME49_224630	p.Ala1712Thr	missense_variant	zinc finger (CCCH type) motif-containing protein
TGME49_224630	p.Val1468Ala	missense_variant	zinc finger (CCCH type) motif-containing protein
TGME49_224630	p.Val1468Leu	missense_variant	zinc finger (CCCH type) motif-containing protein
TGME49_224630	p.Ser1033Pro	missense_variant	zinc finger (CCCH type) motif-containing protein
TGME49_224630	p.Asp657Gly	missense_variant	zinc finger (CCCH type) motif-containing protein
TGME49_224630	p.Thr382Pro	missense_variant	zinc finger (CCCH type) motif-containing protein
TGME49_224630	p.Thr379Met	missense_variant	zinc finger (CCCH type) motif-containing protein
TGME49_224630	p.Gln358Arg	missense_variant	zinc finger (CCCH type) motif-containing protein
TGME49_219120	c.-260A>G	5_prime_UTR_premature_start_codon_gain_variant	zinc finger (CCCH type) motif-containing protein
TGME49_219120	p.Ser389Gly	missense_variant	zinc finger (CCCH type) motif-containing protein
TGME49_219120	p.Glu851Asp	missense_variant	zinc finger (CCCH type) motif-containing protein
TGME49_246200	p.Cys770Ser	missense_variant	zinc finger (CCCH type) motif-containing protein
TGME49_248270	p.Pro325Ser	missense_variant	zinc finger (CCCH type) motif-containing protein
TGME49_248270	p.Ala484Val	missense_variant	zinc finger (CCCH type) motif-containing protein
TGME49_248270	p.Gly803Asp	missense_variant	zinc finger (CCCH type) motif-containing protein
TGME49_250690	p.Pro35Gln	missense_variant	zinc finger (CCCH type) motif-containing protein
TGME49_250690	p.Gly560Ala	missense_variant	zinc finger (CCCH type) motif-containing protein
TGME49_250690	p.Ser1368Arg	missense_variant	zinc finger (CCCH type) motif-containing protein
TGME49_250690	p.Arg1440His	missense_variant	zinc finger (CCCH type) motif-containing protein
TGME49_250690	p.Ala1816Thr	missense_variant	zinc finger (CCCH type) motif-containing protein
TGME49_250690	p.Trp1996Ser	missense_variant	zinc finger (CCCH type) motif-containing protein
TGME49_250690	p.Pro2100Arg	missense_variant	zinc finger (CCCH type) motif-containing protein
TGME49_250690	p.Ser2201Ala	missense_variant	zinc finger (CCCH type) motif-containing protein
TGME49_250690	p.Ser2513Pro	missense_variant	zinc finger (CCCH type) motif-containing protein
TGME49_250690	p.Leu2537Ser	missense_variant	zinc finger (CCCH type) motif-containing protein
TGME49_250690	p.Asp2701Gly	missense_variant	zinc finger (CCCH type) motif-containing protein
TGME49_250690	p.Gly2735Arg	missense_variant	zinc finger (CCCH type) motif-containing protein
TGME49_250690	p.Glu2774Asp	missense_variant	zinc finger (CCCH type) motif-containing protein
TGME49_295658	p.Ala1576Thr	missense_variant	zinc finger in N-recognin protein
TGME49_278800	p.Phe1824Val	missense_variant	zinc finger protein 36 family 3 protein
TGME49_278800	p.Arg1822His	missense_variant	zinc finger protein 36 family 3 protein
TGME49_278800	p.Pro1666Ser	missense_variant	zinc finger protein 36 family 3 protein
TGME49_278800	p.Ile1306Thr	missense_variant	zinc finger protein 36 family 3 protein
TGME49_278800	p.Pro1295Leu	missense_variant	zinc finger protein 36 family 3 protein
TGME49_278800	p.Ala1263Ser	missense_variant	zinc finger protein 36 family 3 protein
TGME49_278800	p.Leu1222Phe	missense_variant	zinc finger protein 36 family 3 protein
TGME49_278800	p.Ala1177Pro	missense_variant	zinc finger protein 36 family 3 protein
TGME49_278800	p.Arg1102Gln	missense_variant	zinc finger protein 36 family 3 protein
TGME49_278800	p.Thr954Lys	missense_variant	zinc finger protein 36 family 3 protein
TGME49_223880	p.Asp1970Asn	missense_variant	zinc finger, C3HC4 type (RING finger) domain-containing protein
TGME49_223880	p.Glu1804Val	missense_variant	zinc finger, C3HC4 type (RING finger) domain-containing protein
TGME49_223880	p.Pro1199Ser	missense_variant	zinc finger, C3HC4 type (RING finger) domain-containing protein
TGME49_223880	p.Ala1151Val	missense_variant	zinc finger, C3HC4 type (RING finger) domain-containing protein
TGME49_223880	p.Glu1097Asp	missense_variant	zinc finger, C3HC4 type (RING finger) domain-containing protein
TGME49_223880	p.Ala570Thr	missense_variant	zinc finger, C3HC4 type (RING finger) domain-containing protein
TGME49_223880	p.Gly547Glu	missense_variant	zinc finger, C3HC4 type (RING finger) domain-containing protein
TGME49_223880	p.Arg427Gln	missense_variant	zinc finger, C3HC4 type (RING finger) domain-containing protein
TGME49_223880	p.Ala193Thr	missense_variant	zinc finger, C3HC4 type (RING finger) domain-containing protein
TGME49_236640	c.-492A>G	5_prime_UTR_premature_start_codon_gain_variant	zinc finger, C3HC4 type (RING finger) domain-containing protein

TGME49_236640	p.Ser274Gly	missense_variant	zinc finger, C3HC4 type (RING finger) domain-containing protein
TGME49_236780	p.Tyr347Phe	missense_variant	zinc finger, C3HC4 type (RING finger) domain-containing protein
TGME49_215640	p.Pro849Arg	missense_variant	zinc finger, C3HC4 type (RING finger) domain-containing protein
TGME49_215640	p.Glu745Asp	missense_variant	zinc finger, C3HC4 type (RING finger) domain-containing protein
TGME49_215640	p.Asn448Ser	missense_variant	zinc finger, C3HC4 type (RING finger) domain-containing protein
TGME49_215640	c.-2021G>A	5_prime_UTR_premature_start_codon_gain_variant	zinc finger, C3HC4 type (RING finger) domain-containing protein
TGME49_247485	p.Ser837Tyr	missense_variant	zinc finger, C3HC4 type (RING finger) domain-containing protein
TGME49_247485	p.Asp858Glu	missense_variant	zinc finger, C3HC4 type (RING finger) domain-containing protein
TGME49_247485	p.Val942Gly	missense_variant	zinc finger, C3HC4 type (RING finger) domain-containing protein
TGME49_248330	p.Cys8Phe	missense_variant&splice_region_variant	zinc finger, C3HC4 type (RING finger) domain-containing protein
TGME49_277740	p.Arg1558Pro	missense_variant	zinc finger, C3HC4 type (RING finger) domain-containing protein
TGME49_277740	p.Glu1519Asp	missense_variant	zinc finger, C3HC4 type (RING finger) domain-containing protein
TGME49_277740	p.Ile1518Met	missense_variant	zinc finger, C3HC4 type (RING finger) domain-containing protein
TGME49_277740	p.Val1447Met	missense_variant	zinc finger, C3HC4 type (RING finger) domain-containing protein
TGME49_277740	p.Ala1315Gly	missense_variant	zinc finger, C3HC4 type (RING finger) domain-containing protein
TGME49_277740	p.Pro1233Ala	missense_variant	zinc finger, C3HC4 type (RING finger) domain-containing protein
TGME49_277740	p.Asn1093Thr	missense_variant	zinc finger, C3HC4 type (RING finger) domain-containing protein
TGME49_277740	p.Ser379Pro	missense_variant	zinc finger, C3HC4 type (RING finger) domain-containing protein
TGME49_277740	p.Thr121Ala	missense_variant	zinc finger, C3HC4 type (RING finger) domain-containing protein
TGME49_277740	c.-232T>C	5_prime_UTR_premature_start_codon_gain_variant	zinc finger, C3HC4 type (RING finger) domain-containing protein
TGME49_250780	p.Val2015Leu	missense_variant	Zn-finger in ubiquitin-hydrolases domain-containing protein
TGME49_250780	p.Leu1648Phe	missense_variant	Zn-finger in ubiquitin-hydrolases domain-containing protein
TGME49_250780	p.Ser1440Asn	missense_variant	Zn-finger in ubiquitin-hydrolases domain-containing protein
TGME49_250780	p.Ala1428Gly	missense_variant	Zn-finger in ubiquitin-hydrolases domain-containing protein
TGME49_250780	p.Thr1281Ala	missense_variant	Zn-finger in ubiquitin-hydrolases domain-containing protein
TGME49_250780	p.Pro1279Ser	missense_variant	Zn-finger in ubiquitin-hydrolases domain-containing protein
TGME49_250780	p.Ala602Thr	missense_variant	Zn-finger in ubiquitin-hydrolases domain-containing protein
TGME49_250780	p.Arg381Gly	missense_variant	Zn-finger in ubiquitin-hydrolases domain-containing protein
TGME49_250780	p.Met375Val	missense_variant	Zn-finger in ubiquitin-hydrolases domain-containing protein
TGME49_250780	p.Pro367Gln	missense_variant	Zn-finger in ubiquitin-hydrolases domain-containing protein
TGME49_253925	p.Cys311*	stop_gained	zonadhesin family protein
TGME49_308040	c.-7C>T	splice_region_variant	ZPR1 zinc finger domain-containing protein
TGME49_308040	c.-487C>T	5_prime_UTR_premature_start_codon_gain_variant	ZPR1 zinc finger domain-containing protein

Supplementary Table S1B: GO Slim (Biological process) analysis of T. gondii genes carrying potential change-of-function mutations in E[illegible] or TgCatBr1 strains compared to reference strain ME49

GO term	GO genes
biological_process (others)	TGME49_207070, TGME49_207080, TGME49_207180, TGME49_210790, TGME49_210800, TGME49_210830, TGME49_211050, TGME49_211090, TGME49_211230, TGME49_211240, TGME49_211390, TGME49_211400, TGME49_211670, TGME49_211720, TGME49_211730, TGME49_211910, TGME49_212100, TGME49_212110, TGME49_212130, TGME49_214260, TGME49_214280, TGME49_214490, TGME49_214840, TGME49_215030, TGME49_215150, TGME49_215260, TGME49_215480, TGME49_215510, TGME49_215560, TGME49_215570, TGME49_215750, TGME49_217350, TGME49_217440, TGME49_217450, TGME49_217700, TGME49_217850, TGME49_218210, TGME49_218250, TGME49_218410, TGME49_218420, TGME49_218470, TGME49_218560, TGME49_218580, TGME49_218590, TGME49_218610, TGME49_218960, TGME49_219070, TGME49_219100, TGME49_219180, TGME49_219200, TGME49_219230, TGME49_219240, TGME49_219250, TGME49_219260, TGME49_219310, TGME49_219550, TGME49_219620, TGME49_219832, TGME49_220940, TGME49_221160, TGME49_221200, TGME49_221230, TGME49_221320, TGME49_221380, TGME49_221420, TGME49_221500, TGME49_221540, TGME49_221580, TGME49_221590, TGME49_221660, TGME49_221890, TGME49_222010, TGME49_222140, TGME49_222160, TGME49_222340, TGME49_222430, TGME49_222840, TGME49_223130, TGME49_223440, TGME49_223690, TGME49_223840, TGME49_223985, TGME49_224060, TGME49_224090, TGME49_224190, TGME49_224220, TGME49_224240, TGME49_224290, TGME49_224310, TGME49_224840, TGME49_224880, TGME49_224890, TGME49_224910, TGME49_224920, TGME49_224928, TGME49_234190, TGME49_234280, TGME49_234490, TGME49_234640, TGME49_235000, TGME49_235020, TGME49_235470, TGME49_235610, TGME49_235680, TGME49_235905, TGME49_235920, TGME49_236040, TGME49_236130, TGME49_236240, TGME49_236260, TGME49_236650, TGME49_236860, TGME49_236980, TGME49_236990, TGME49_237020, TGME49_237090, TGME49_237410, TGME49_237470, TGME49_237500, TGME49_238040, TGME49_238050, TGME49_239020, TGME49_239130, TGME49_239440, TGME49_239480, TGME49_239530, TGME49_239560, TGME49_239630, TGME49_239710, TGME49_239748, TGME49_239820, TGME49_239910, TGME49_245510, TGME49_245670, TGME49_246510, TGME49_246530, TGME49_246650, TGME49_246690, TGME49_246740, TGME49_246910, TGME49_246970, TGME49_247250, TGME49_247360, TGME49_247390, TGME49_247500, TGME49_247590, TGME49_247600, TGME49_247690, TGME49_247700, TGME49_247730, TGME49_247760, TGME49_247790, TGME49_247920, TGME49_248110, TGME49_248200, TGME49_248400, TGME49_248600, TGME49_248670, TGME49_248830, TGME49_248890, TGME49_248960, TGME49_249010, TGME49_249020, TGME49_249180, TGME49_249260, TGME49_249270, TGME49_249380, TGME49_249460, TGME49_249630, TGME49_249810, TGME49_249820, TGME49_249840, TGME49_250750, TGME49_250780, TGME49_250870, TGME49_251480, TGME49_251620, TGME49_251640, TGME49_251740, TGME49_252420, TGME49_252465, TGME49_253030, TGME49_253040, TGME49_253070, TGME49_253090, TGME49_253380, TGME49_253480, TGME49_253890, TGME49_253900, TGME49_253960, TGME49_254280, TGME49_254350, TGME49_254365, TGME49_254410, TGME49_254420, TGME49_254555, TGME49_254770, TGME49_254880, TGME49_254950, TGME49_275420, TGME49_275600, TGME49_275630, TGME49_276910, TGME49_277030, TGME49_277550, TGME49_277720, TGME49_277760, TGME49_278260, TGME49_278280, TGME49_278500, TGME49_278518, TGME49_278640, TGME49_278660, TGME49_278740, TGME49_278750, TGME49_278850, TGME49_278870, TGME49_278878, TGME49_278940, TGME49_280600, TGME49_280610, TGME49_280660, TGME49_280700, TGME49_280750, TGME49_280760, TGME49_280780, TGME49_288390, TGME49_294350, TGME49_295710, TGME49_297110, TGME49_297150, TGME49_297640, TGME49_297650, TGME49_297720, TGME49_299020, TGME49_299070, TGME49_299150, TGME49_299168, TGME49_300120, TGME49_300320, TGME49_300340, TGME49_300350, TGME49_301000, TGME49_301010, TGME49_301120, TGME49_301216, TGME49_301370, TGME49_307980, TGME49_318190, TGME49_318260, TGME49_318430, TGME49_318440, TGME49_318460, TGME49_318510, TGME49_318580, TGME49_318610, TGME49_318660, TGME49_318675, TGME49_318710, TGME49_318730, TGME49_318750, TGME49_319090, TGME49_319570, TGME49_319662, TGME49_319710, TGME49_319870, TGME49_319920
cellular protein modification process	TGME49_207080, TGME49_210830, TGME49_211260, TGME49_211730, TGME49_214170, TGME49_214920, TGME49_215560, TGME49_215670, TGME49_217850, TGME49_218400, TGME49_218590, TGME49_218720, TGME49_219070, TGME49_219460, TGME49_221230, TGME49_221270, TGME49_221500, TGME49_221590, TGME49_221890, TGME49_222010, TGME49_222430, TGME49_222710, TGME49_222900, TGME49_222960, TGME49_223985, TGME49_224240, TGME49_224480, TGME49_234950, TGME49_234970, TGME49_235020, TGME49_235370, TGME49_235700, TGME49_235750, TGME49_235980, TGME49_236240, TGME49_236260, TGME49_236620, TGME49_237410, TGME49_237500, TGME49_237860, TGME49_237890, TGME49_238000, TGME49_239130, TGME49_239420, TGME49_239440, TGME49_239600, TGME49_239620, TGME49_239885, TGME49_239910, TGME49_240090, TGME49_245580, TGME49_247710, TGME49_249260, TGME49_249460, TGME49_249620, TGME49_249680, TGME49_250680, TGME49_250850, TGME49_251850, TGME49_252360, TGME49_252420, TGME49_252440, TGME49_253400, TGME49_253440, TGME49_253580, TGME49_253860, TGME49_253940, TGME49_254190, TGME49_254410, TGME49_254555, TGME49_275568, TGME49_275610, TGME49_275630, TGME49_278280, TGME49_278900, TGME49_278940, TGME49_280660, TGME49_295710, TGME49_297150, TGME49_297650, TGME49_297720, TGME49_299040, TGME49_301270, TGME49_301440, TGME49_308000, TGME49_308096, TGME49_318240, TGME49_318275, TGME49_318660, TGME49_318770, TGME49_319600, TGME49_319610, TGME49_319700, TGME49_320210
biosynthetic process	TGME49_210790, TGME49_210960, TGME49_214480, TGME49_214850, TGME49_214920, TGME49_215250, TGME49_215260, TGME49_215700, TGME49_217580, TGME49_217910, TGME49_218560, TGME49_219460, TGME49_219600, TGME49_219700, TGME49_219860, TGME49_221260, TGME49_221320, TGME49_221922, TGME49_222800, TGME49_222900, TGME49_224490, TGME49_234280, TGME49_234580, TGME49_235170, TGME49_235478, TGME49_235640, TGME49_237110, TGME49_237200, TGME49_237480, TGME49_237830, TGME49_238110, TGME49_238190, TGME49_238950, TGME49_239710, TGME49_239820, TGME49_245570, TGME49_246060, TGME49_246530, TGME49_246630, TGME49_248600, TGME49_249180, TGME49_249560, TGME49_250060, TGME49_250880, TGME49_251620, TGME49_251670, TGME49_252440, TGME49_252640, TGME49_253400, TGME49_253780, TGME49_253880, TGME49_253900, TGME49_254280, TGME49_254370, TGME49_254540, TGME49_275568, TGME49_277540, TGME49_278110, TGME49_278740, TGME49_278910, TGME49_280600, TGME49_280690, TGME49_280730, TGME49_280750, TGME49_297530, TGME49_297720, TGME49_297840, TGME49_297925, TGME49_297940, TGME49_299040, TGME49_300650, TGME49_307770, TGME49_308000, TGME49_319860
phosphorylation	TGME49_210830, TGME49_211260, TGME49_214260, TGME49_215250, TGME49_215670, TGME49_215700, TGME49_218400, TGME49_218720, TGME49_219070, TGME49_219100, TGME49_219832, TGME49_222020, TGME49_222960, TGME49_224480, TGME49_224900, TGME49_234950, TGME49_234970, TGME49_235370, TGME49_235478, TGME49_235750, TGME49_236040, TGME49_236240, TGME49_236620, TGME49_237860, TGME49_237890, TGME49_239130, TGME49_239250, TGME49_239420, TGME49_239440, TGME49_239600, TGME49_239885, TGME49_239910, TGME49_240090, TGME49_245580, TGME49_245670, TGME49_245730, TGME49_247710, TGME49_248530, TGME49_249260, TGME49_250680, TGME49_250850, TGME49_250880, TGME49_252360, TGME49_253440, TGME49_253580, TGME49_253860, TGME49_253940, TGME49_254190, TGME49_275610, TGME49_278900, TGME49_297780, TGME49_299070, TGME49_301270, TGME49_301440, TGME49_307770, TGME49_308096, TGME49_318580, TGME49_318770, TGME49_319308, TGME49_319610, TGME49_319700
translation	TGME49_210750, TGME49_210840, TGME49_211230, TGME49_211410, TGME49_211870, TGME49_214270, TGME49_217460, TGME49_217590, TGME49_217620, TGME49_217670, TGME49_218790, TGME49_218850, TGME49_219430, TGME49_219540, TGME49_221670, TGME49_222070, TGME49_222990, TGME49_223660, TGME49_223970, TGME49_224235, TGME49_235540, TGME49_235970, TGME49_238010, TGME49_239100, TGME49_245680, TGME49_250810, TGME49_250955, TGME49_253800, TGME49_254110, TGME49_254200, TGME49_254380, TGME49_254400, TGME49_277030, TGME49_278550, TGME49_280600, TGME49_299810, TGME49_300000, TGME49_300260, TGME49_300606, TGME49_300613, TGME49_301380, TGME49_307980, TGME49_315150, TGME49_317720, TGME49_318260, TGME49_318700, TGME49_320150
protein metabolic process	TGME49_200350, TGME49_210730, TGME49_210781, TGME49_211330, TGME49_214470, TGME49_215010, TGME49_218920, TGME49_219485, TGME49_221310, TGME49_221610, TGME49_221830, TGME49_223450, TGME49_224080, TGME49_224350, TGME49_224460, TGME49_235200, TGME49_235860, TGME49_235950, TGME49_240240, TGME49_245500, TGME49_245590, TGME49_246800, TGME49_247240, TGME49_248470, TGME49_248710, TGME49_249670, TGME49_251510, TGME49_251570, TGME49_251760, TGME49_252440, TGME49_253170, TGME49_254010, TGME49_275690, TGME49_277500, TGME49_277895, TGME49_277990, TGME49_278050, TGME49_278975, TGME49_280710, TGME49_280740, TGME49_300020, TGME49_318320, TGME49_319970
RNA metabolic process	TGME49_211450, TGME49_214200, TGME49_214210, TGME49_217580, TGME49_218570, TGME49_218580, TGME49_219440, TGME49_219600, TGME49_220940, TGME49_221380, TGME49_222120, TGME49_224200, TGME49_237020, TGME49_238190, TGME49_239480, TGME49_239790, TGME49_246060, TGME49_246500, TGME49_246630, TGME49_246760, TGME49_246970, TGME49_247740, TGME49_248200, TGME49_249560, TGME49_249610, TGME49_250060, TGME49_250750, TGME49_251620, TGME49_252465, TGME49_253530, TGME49_254280, TGME49_254450, TGME49_254730, TGME49_254950, TGME49_297530, TGME49_297840, TGME49_298040, TGME49_300320, TGME49_300340, TGME49_301390
DNA metabolic process	TGME49_210960, TGME49_214480, TGME49_214520, TGME49_215550, TGME49_217450, TGME49_217910, TGME49_218840, TGME49_219700, TGME49_219860, TGME49_221330, TGME49_234360, TGME49_234580, TGME49_235170, TGME49_236200, TGME49_237110, TGME49_237480, TGME49_237830, TGME49_238110, TGME49_245570, TGME49_248160, TGME49_248890, TGME49_251620, TGME49_251670, TGME49_253040, TGME49_253070, TGME49_253480, TGME49_277220, TGME49_277530, TGME49_277540, TGME49_278060, TGME49_280690, TGME49_297780, TGME49_297840, TGME49_297940, TGME49_301222, TGME49_319860

oxidation-reduction process	TGME49_210790, TGME49_214620, TGME49_214760, TGME49_214850, TGME49_215280, TGME49_219130, TGME49_219630, TGME49_221270, TGME49_222160, TGME49_236010, TGME49_237200, TGME49_238070, TGME49_238950, TGME49_239820, TGME49_245670, TGME49_246920, TGME49_247500, TGME49_248990, TGME49_249180, TGME49_249320, TGME49_249390, TGME49_249840, TGME49_251930, TGME49_253120, TGME49_253750, TGME49_253960, TGME49_275420, TGME49_277790, TGME49_280780, TGME49_301210, TGME49_318430, TGME49_320220
cellular nitrogen compound metabolic process	TGME49_210790, TGME49_215250, TGME49_215260, TGME49_215550, TGME49_217350, TGME49_217910, TGME49_224650, TGME49_224900, TGME49_234190, TGME49_234280, TGME49_235000, TGME49_237020, TGME49_238400, TGME49_246920, TGME49_246970, TGME49_249180, TGME49_250880, TGME49_250890, TGME49_251620, TGME49_253780, TGME49_253900, TGME49_254450, TGME49_275830, TGME49_278060, TGME49_280740, TGME49_300060, TGME49_301216, TGME49_319308, TGME49_319860
transmembrane transport	TGME49_212310, TGME49_219260, TGME49_219650, TGME49_221350, TGME49_234410, TGME49_236860, TGME49_239020, TGME49_239080, TGME49_240080, TGME49_240210, TGME49_245510, TGME49_248330, TGME49_248670, TGME49_248840, TGME49_248950, TGME49_249820, TGME49_251630, TGME49_252640, TGME49_253640, TGME49_253700, TGME49_254080, TGME49_278660, TGME49_278800, TGME49_297245, TGME49_299060, TGME49_318150, TGME49_320220
chromosome organization	TGME49_207080, TGME49_211730, TGME49_214520, TGME49_215560, TGME49_219700, TGME49_219860, TGME49_221330, TGME49_221890, TGME49_234360, TGME49_235020, TGME49_248160, TGME49_248290, TGME49_248890, TGME49_249460, TGME49_249620, TGME49_249680, TGME49_252420, TGME49_253040, TGME49_253480, TGME49_254555, TGME49_277530, TGME49_278280, TGME49_293368, TGME49_297150, TGME49_297780, TGME49_297800
transport	TGME49_208420, TGME49_211370, TGME49_212310, TGME49_214770, TGME49_219720, TGME49_221522, TGME49_221940, TGME49_222380, TGME49_235020, TGME49_236020, TGME49_239020, TGME49_239080, TGME49_246330, TGME49_248100, TGME49_248950, TGME49_249170, TGME49_249610, TGME49_249702, TGME49_249820, TGME49_253640, TGME49_275490, TGME49_276910, TGME49_278990, TGME49_280610, TGME49_299060, TGME49_299180
cellular amino acid metabolic process	TGME49_210750, TGME49_210840, TGME49_215260, TGME49_217460, TGME49_219430, TGME49_219540, TGME49_221260, TGME49_222900, TGME49_234190, TGME49_236010, TGME49_237470, TGME49_239820, TGME49_248600, TGME49_248990, TGME49_249180, TGME49_249390, TGME49_253780, TGME49_254110, TGME49_254200, TGME49_277030, TGME49_278740, TGME49_278910, TGME49_280600, TGME49_299810, TGME49_300260
lipid metabolic process	TGME49_212130, TGME49_214850, TGME49_214920, TGME49_215700, TGME49_218560, TGME49_219230, TGME49_221320, TGME49_224490, TGME49_237200, TGME49_238400, TGME49_238950, TGME49_245730, TGME49_246530, TGME49_247500, TGME49_247760, TGME49_248830, TGME49_252440, TGME49_253880, TGME49_254330, TGME49_254540, TGME49_275568, TGME49_277950, TGME49_299040, TGME49_308000
ion transport	TGME49_208420, TGME49_212200, TGME49_212310, TGME49_219260, TGME49_221350, TGME49_224190, TGME49_234540, TGME49_235402, TGME49_235880, TGME49_236860, TGME49_240210, TGME49_245510, TGME49_247690, TGME49_248670, TGME49_251630, TGME49_252640, TGME49_253640, TGME49_254080, TGME49_254370, TGME49_278660, TGME49_299060, TGME49_318460, TGME49_319740, TGME49_320220
response to stress	TGME49_214480, TGME49_214520, TGME49_217450, TGME49_218840, TGME49_234580, TGME49_235170, TGME49_236200, TGME49_237480, TGME49_238110, TGME49_248160, TGME49_248890, TGME49_251620, TGME49_253040, TGME49_253650, TGME49_275690, TGME49_277220, TGME49_277540, TGME49_278060, TGME49_301222, TGME49_319860
catabolic process	TGME49_210781, TGME49_218920, TGME49_221610, TGME49_222020, TGME49_223450, TGME49_236010, TGME49_236040, TGME49_237470, TGME49_245670, TGME49_247240, TGME49_247500, TGME49_248710, TGME49_248830, TGME49_248990, TGME49_254350, TGME49_277500, TGME49_277895, TGME49_278050, TGME49_280710, TGME49_299070
signal transduction	TGME49_214770, TGME49_215700, TGME49_219540, TGME49_219720, TGME49_224840, TGME49_234970, TGME49_236240, TGME49_239130, TGME49_239250, TGME49_246510, TGME49_248830, TGME49_249170, TGME49_249260, TGME49_250850, TGME49_252880, TGME49_254370, TGME49_277790, TGME49_277840, TGME49_318675
small molecule metabolic process	TGME49_210790, TGME49_215260, TGME49_215280, TGME49_218560, TGME49_219550, TGME49_221320, TGME49_234190, TGME49_234280, TGME49_235000, TGME49_238950, TGME49_239630, TGME49_247500, TGME49_247760, TGME49_249180, TGME49_250880, TGME49_253880, TGME49_318430
tRNA metabolic process	TGME49_210750, TGME49_210840, TGME49_215100, TGME49_217460, TGME49_218580, TGME49_219430, TGME49_219540, TGME49_223130, TGME49_253530, TGME49_254110, TGME49_254200, TGME49_254730, TGME49_277030, TGME49_280600, TGME49_280780, TGME49_299810, TGME49_300260
carbohydrate metabolic process	TGME49_222020, TGME49_222800, TGME49_223840, TGME49_235700, TGME49_236040, TGME49_239710, TGME49_245670, TGME49_246690, TGME49_253030, TGME49_253400, TGME49_254880, TGME49_278110, TGME49_297720, TGME49_299070, TGME49_318430, TGME49_318580
nucleotide metabolic process	TGME49_222710, TGME49_224900, TGME49_234280, TGME49_235478, TGME49_239310, TGME49_239620, TGME49_239710, TGME49_249180, TGME49_250880, TGME49_254370, TGME49_280750, TGME49_307770, TGME49_318750
ribosome biogenesis	TGME49_214200, TGME49_218410, TGME49_218570, TGME49_236580, TGME49_239790, TGME49_248200, TGME49_252465, TGME49_298040, TGME49_300320, TGME49_301390, TGME49_318700, TGME49_319658
cofactor metabolic process	TGME49_215250, TGME49_222020, TGME49_234190, TGME49_235478, TGME49_236040, TGME49_239310, TGME49_245670, TGME49_249180, TGME49_253780, TGME49_253900, TGME49_299070, TGME49_307770
transcription, DNA-templated	TGME49_217580, TGME49_219600, TGME49_224660, TGME49_238190, TGME49_246060, TGME49_248290, TGME49_249560, TGME49_249680, TGME49_250060, TGME49_254280, TGME49_297530
vesicle-mediated transport	TGME49_211370, TGME49_221522, TGME49_221940, TGME49_224150, TGME49_235020, TGME49_238210, TGME49_238220, TGME49_246610, TGME49_248100, TGME49_278890, TGME49_299180
homeostatic process	TGME49_218470, TGME49_219130, TGME49_224060, TGME49_238040, TGME49_238070, TGME49_246920, TGME49_247660, TGME49_248160, TGME49_249270, TGME49_277790
membrane organization	TGME49_219260, TGME49_224190, TGME49_236130, TGME49_245510, TGME49_247690, TGME49_254370, TGME49_275802, TGME49_280610
mRNA processing	TGME49_210980, TGME49_219440, TGME49_221190, TGME49_234520, TGME49_238230, TGME49_246760, TGME49_249610, TGME49_297140
protein folding	TGME49_218470, TGME49_238000, TGME49_238040, TGME49_248370, TGME49_249270, TGME49_253650, TGME49_297500, TGME49_318275
nucleobase-containing compound catabolic process	TGME49_239620, TGME49_239630, TGME49_251620, TGME49_253480, TGME49_254450, TGME49_318750
nitrogen compound metabolic process	TGME49_215260, TGME49_235183, TGME49_235187, TGME49_237200, TGME49_238880, TGME49_253930
protein targeting	TGME49_236130, TGME49_247652, TGME49_248330, TGME49_275802, TGME49_280610
cellular component assembly	TGME49_217760, TGME49_221922, TGME49_280730, TGME49_297925, TGME49_300650
cell cycle	TGME49_217760, TGME49_219100, TGME49_219832, TGME49_280750, TGME49_297520
cellular component organization	TGME49_219290, TGME49_247652, TGME49_248330, TGME49_249365, TGME49_250720
sulfur compound metabolic process	TGME49_215250, TGME49_236010, TGME49_246920, TGME49_248990, TGME49_278910
ATP metabolic process	TGME49_222020, TGME49_236040, TGME49_245670, TGME49_252640, TGME49_299070
generation of precursor metabolites and energy	TGME49_222020, TGME49_236040, TGME49_245670, TGME49_299070
protein maturation	TGME49_275690, TGME49_280740, TGME49_300060
cell division	TGME49_217760, TGME49_219100, TGME49_219832
cell differentiation	TGME49_211270, TGME49_223480
cytoskeleton-dependent intracellular transport	TGME49_249365, TGME49_250720
cell motility	TGME49_217650, TGME49_248225
cell projection assembly	TGME49_248970, TGME49_250720
anatomical structure formation involved in morphogenesis	TGME49_248970, TGME49_250720
cell morphogenesis	TGME49_248970, TGME49_250720
mitotic nuclear division	TGME49_246740, TGME49_297800
ribonucleoprotein complex assembly	TGME49_210980, TGME49_318700
cellular respiration	TGME49_215280, TGME49_219550

cell wall organization or biogenesis	TGME49_254350
vacuolar transport	TGME49_248310
mitochondrion organization	TGME49_219540
chromosome segregation	TGME49_297800
autophagy	TGME49_221360

Supplementary Table S2: Differential expression analysis of human genes from HFF hours.

gene_ID	logFC 2h / Uninf	logFC 18h / Uninf	logFC 48h / Uninf	logCPM	PValue	FDR
ENSG00000004846	-0.15	-4.24	-10.36	3.89	1.20E-29	1.71E-25
ENSG00000196616	-0.27	-5.58	-12.98	3.90	2.23E-23	1.58E-19
ENSG00000169607	0.88	4.90	5.69	5.04	4.74E-18	2.24E-14
ENSG00000089685	0.19	2.98	3.78	6.00	4.19E-16	1.48E-12
ENSG00000131747	0.64	4.07	4.31	7.91	9.35E-16	2.65E-12
ENSG00000101057	-0.06	3.84	3.04	4.58	1.44E-15	3.40E-12
ENSG00000148773	0.70	4.04	4.33	7.05	3.73E-15	7.55E-12
ENSG00000170458	-0.94	-3.04	-5.04	6.06	5.77E-15	1.02E-11
ENSG00000138347	1.33	3.91	3.54	4.64	1.26E-14	1.88E-11
ENSG00000138180	0.87	3.79	4.54	5.34	1.33E-14	1.88E-11
ENSG00000078401	1.16	-2.53	-3.32	5.07	2.01E-14	2.59E-11
ENSG00000154839	0.78	4.73	4.72	4.73	2.80E-14	3.12E-11
ENSG00000068489	0.81	3.48	4.36	4.83	2.87E-14	3.12E-11
ENSG00000123358	2.02	-4.40	-4.52	6.00	5.06E-14	5.12E-11
ENSG00000145386	0.51	4.28	4.94	4.95	6.43E-14	5.72E-11
ENSG00000072571	0.67	4.37	4.79	4.82	6.46E-14	5.72E-11
ENSG00000092853	0.39	3.85	3.52	5.08	1.84E-13	1.53E-10
ENSG00000093009	0.12	4.32	3.54	4.22	2.70E-13	2.12E-10
ENSG00000142945	0.27	3.50	4.13	5.12	3.36E-13	2.50E-10
ENSG00000169679	0.24	4.10	4.82	5.29	3.83E-13	2.71E-10
ENSG00000111206	0.25	2.79	3.52	4.71	4.46E-13	3.01E-10
ENSG00000109805	0.10	4.51	4.61	5.71	8.07E-13	5.20E-10
ENSG00000088325	0.04	2.99	3.84	6.72	1.08E-12	6.67E-10
ENSG00000117724	0.46	3.94	4.54	6.34	1.34E-12	7.74E-10
ENSG00000101447	1.24	3.43	4.52	4.51	1.37E-12	7.74E-10
ENSG00000168078	0.72	4.09	4.74	6.00	1.52E-12	8.26E-10
ENSG00000161888	-0.25	3.20	3.54	3.88	1.58E-12	8.30E-10
ENSG00000237649	0.72	3.39	3.39	4.71	1.76E-12	8.88E-10
ENSG00000111665	0.23	3.06	3.69	3.99	2.19E-12	1.07E-09
ENSG00000138778	0.68	3.81	3.89	5.08	2.54E-12	1.16E-09
ENSG00000196611	0.45	4.45	4.97	9.06	3.06E-12	1.36E-09
ENSG00000146670	0.67	3.86	3.37	5.01	3.87E-12	1.60E-09

ENSG00000198901	0.27	2.81	3.41	6.29	5.02E-12	1.92E-09
ENSG00000186871	-1.06	4.11	4.13	3.59	5.78E-12	2.10E-09
ENSG00000090889	0.17	3.53	3.88	5.14	6.33E-12	2.19E-09
ENSG00000112742	-0.24	4.17	4.79	4.95	7.25E-12	2.45E-09
ENSG00000165480	0.58	4.30	4.54	4.24	7.81E-12	2.54E-09
ENSG00000164109	0.51	3.73	4.53	4.86	9.72E-12	3.02E-09
ENSG00000076382	-0.05	3.24	3.58	4.83	9.83E-12	3.02E-09
ENSG00000119508	1.48	-3.24	-4.26	4.71	1.10E-11	3.32E-09
ENSG00000143476	0.29	3.85	3.54	4.88	1.40E-11	4.07E-09
ENSG00000107968	1.88	-1.74	-1.01	4.75	1.41E-11	4.07E-09
ENSG00000198826	0.20	3.21	4.14	5.04	1.69E-11	4.78E-09
ENSG00000013810	-0.03	3.02	3.08	5.36	1.78E-11	4.89E-09
ENSG00000172548	0.61	-4.93	-4.60	4.93	1.79E-11	4.89E-09
ENSG00000137331	2.18	-2.15	-2.31	6.66	2.35E-11	6.27E-09
ENSG00000024526	0.14	4.26	5.66	4.54	2.83E-11	7.41E-09
ENSG00000112984	0.64	3.00	4.41	5.80	3.22E-11	8.17E-09
ENSG00000166592	1.27	-5.02	-3.14	4.10	3.29E-11	8.17E-09
ENSG00000158402	-0.76	3.59	4.21	3.00	3.31E-11	8.17E-09
ENSG00000108342	6.25	2.52	8.82	2.31	3.40E-11	8.17E-09
ENSG00000171241	0.64	3.95	4.06	5.13	3.40E-11	8.17E-09
ENSG00000122952	-0.15	3.44	3.18	5.29	3.62E-11	8.55E-09
ENSG00000163874	2.83	-2.49	-1.21	5.08	4.03E-11	9.36E-09
ENSG00000131153	0.82	3.78	3.05	4.44	5.93E-11	1.35E-08
ENSG00000167900	0.10	2.57	3.19	5.65	6.84E-11	1.54E-08
ENSG00000143228	0.11	4.22	4.72	4.11	7.58E-11	1.68E-08
ENSG00000149968	0.49	2.82	3.64	10.63	8.08E-11	1.76E-08
ENSG00000241644	-0.77	-3.80	-4.78	5.13	9.13E-11	1.96E-08
ENSG00000117399	-0.15	3.12	3.85	5.14	9.67E-11	2.04E-08
ENSG00000175063	1.38	4.46	4.61	4.12	1.09E-10	2.27E-08
ENSG00000134690	-0.42	3.65	3.73	4.66	1.26E-10	2.60E-08
ENSG00000154027	0.45	3.28	2.92	4.65	1.37E-10	2.75E-08
ENSG00000122877	1.33	-6.24	-6.00	5.49	1.38E-10	2.75E-08
ENSG00000183856	-0.17	3.03	3.39	5.00	1.54E-10	3.03E-08
ENSG00000205835	-0.09	-2.62	-7.23	2.31	1.76E-10	3.39E-08
ENSG00000011426	0.40	3.53	4.15	7.42	1.77E-10	3.39E-08
ENSG00000071539	0.62	3.37	3.86	4.28	2.08E-10	3.93E-08
ENSG00000080986	-0.06	3.45	3.76	5.67	2.22E-10	4.13E-08
ENSG00000090339	1.49	-2.27	-1.20	8.66	2.68E-10	4.93E-08
ENSG00000087494	0.30	-3.14	-5.48	3.30	2.74E-10	4.97E-08
ENSG00000118193	0.70	4.18	5.11	4.14	3.16E-10	5.67E-08
ENSG00000179388	1.29	-7.45	-6.06	7.13	3.45E-10	6.11E-08
ENSG00000137193	1.29	-2.09	-1.63	4.80	3.95E-10	6.91E-08

ENSG00000163535	0.27	2.63	3.84	4.85	4.38E-10	7.52E-08
ENSG00000075702	1.30	4.35	4.16	3.17	4.41E-10	7.52E-08
ENSG00000151725	0.29	3.71	3.38	5.04	4.93E-10	8.30E-08
ENSG00000163808	-0.26	3.65	3.89	3.97	5.04E-10	8.40E-08
ENSG00000137310	0.62	3.71	3.13	4.81	5.30E-10	8.64E-08
ENSG00000065328	0.49	4.75	4.19	4.18	5.31E-10	8.64E-08
ENSG00000186185	0.82	4.66	4.16	2.84	5.65E-10	9.09E-08
ENSG00000122966	0.01	3.37	3.77	4.83	5.90E-10	9.38E-08
ENSG00000161800	0.23	3.00	3.46	6.93	7.58E-10	1.18E-07
ENSG00000087586	0.05	3.07	4.40	5.86	7.62E-10	1.18E-07
ENSG00000162063	-0.09	2.39	2.97	4.38	8.65E-10	1.32E-07
ENSG00000182667	-0.13	4.36	4.03	3.30	8.81E-10	1.32E-07
ENSG00000171848	0.58	4.37	3.90	7.13	8.83E-10	1.32E-07
ENSG00000166396	1.78	4.16	6.43	5.24	1.01E-09	1.49E-07
ENSG00000136928	0.35	3.84	1.79	3.36	1.19E-09	1.72E-07
ENSG00000178999	-0.69	3.44	3.23	3.25	1.31E-09	1.87E-07
ENSG00000126787	1.32	4.22	5.01	4.92	1.39E-09	1.97E-07
ENSG00000075218	0.49	3.75	4.04	4.51	1.53E-09	2.14E-07
ENSG00000100297	-0.09	2.60	2.08	5.49	1.66E-09	2.30E-07
ENSG00000138182	0.15	2.83	3.53	5.13	2.50E-09	3.41E-07
ENSG00000094804	0.36	3.73	2.48	5.54	2.65E-09	3.53E-07
ENSG00000113368	0.13	3.46	3.48	4.99	2.79E-09	3.69E-07
ENSG00000170312	-0.09	3.99	4.30	5.83	3.06E-09	3.97E-07
ENSG00000184557	1.82	-0.87	-0.62	6.53	3.67E-09	4.72E-07
ENSG00000134057	0.66	2.53	3.88	6.93	3.70E-09	4.72E-07
ENSG00000104738	-0.02	2.88	2.46	6.87	4.78E-09	5.99E-07
ENSG00000166825	0.16	1.90	2.26	9.82	5.50E-09	6.77E-07
ENSG00000187955	-0.61	-1.71	-4.96	5.26	6.05E-09	7.38E-07
ENSG00000119969	0.17	3.62	2.99	4.43	6.18E-09	7.48E-07
ENSG00000198554	-0.12	2.80	2.38	4.80	7.58E-09	9.01E-07
ENSG00000056558	1.63	-2.76	-2.64	5.09	8.05E-09	9.42E-07
ENSG00000185215	1.70	-1.55	-0.76	6.79	8.54E-09	9.92E-07
ENSG00000260549	1.01	4.18	4.77	3.75	8.63E-09	9.94E-07
ENSG00000166803	0.16	3.47	3.72	5.26	8.95E-09	1.02E-06
ENSG00000166851	0.23	2.92	3.93	4.46	1.09E-08	1.23E-06
ENSG00000163710	-0.97	-2.19	-4.49	4.38	1.22E-08	1.36E-06
ENSG00000163507	0.33	2.95	3.63	5.58	1.31E-08	1.44E-06
ENSG00000105011	0.41	3.22	2.67	4.60	1.41E-08	1.54E-06
ENSG00000137804	0.33	3.32	3.40	5.17	1.52E-08	1.65E-06
ENSG00000124875	2.87	4.34	7.68	5.93	1.55E-08	1.66E-06
ENSG00000144554	-0.26	2.88	2.71	4.31	1.57E-08	1.67E-06
ENSG00000085999	0.07	3.49	2.58	2.99	1.60E-08	1.68E-06

ENSG00000140525	0.02	3.13	3.13	5.74	1.70E-08	1.77E-06
ENSG00000041982	0.99	4.02	3.66	9.33	1.72E-08	1.78E-06
ENSG00000164611	0.00	2.28	3.42	5.80	1.80E-08	1.83E-06
ENSG00000125740	1.12	-6.58	-5.90	7.48	1.87E-08	1.89E-06
ENSG00000112312	0.18	2.89	2.87	5.27	2.25E-08	2.24E-06
ENSG00000184661	0.98	4.37	4.56	4.66	2.56E-08	2.48E-06
ENSG00000117650	1.02	4.19	5.34	3.96	2.56E-08	2.48E-06
ENSG00000157456	0.32	2.54	3.65	4.70	2.58E-08	2.48E-06
ENSG00000103489	0.96	2.97	3.00	4.70	3.29E-08	3.14E-06
ENSG00000187741	-0.37	2.61	2.16	3.90	3.45E-08	3.28E-06
ENSG00000123485	-0.04	3.68	3.36	4.41	3.83E-08	3.60E-06
ENSG00000134222	-0.49	2.04	2.62	4.10	4.00E-08	3.71E-06
ENSG00000242265	0.30	3.02	4.24	3.97	4.03E-08	3.71E-06
ENSG00000198355	1.28	-2.29	-1.39	6.04	4.18E-08	3.74E-06
ENSG00000101412	-0.25	2.76	2.08	4.34	4.19E-08	3.74E-06
ENSG00000146918	0.23	2.81	2.91	5.32	4.21E-08	3.74E-06
ENSG00000113810	0.29	2.03	2.69	6.52	4.58E-08	4.03E-06
ENSG00000166508	-0.12	2.12	2.01	6.90	4.90E-08	4.27E-06
ENSG00000164283	1.64	6.40	7.66	4.91	4.94E-08	4.27E-06
ENSG00000115163	-0.38	3.06	3.71	4.14	5.10E-08	4.36E-06
ENSG00000143333	1.02	-2.95	-4.51	2.70	5.11E-08	4.36E-06
ENSG00000187479	1.79	-4.03	-4.59	2.82	6.10E-08	5.17E-06
ENSG00000132510	1.26	-2.92	-2.40	6.12	6.26E-08	5.27E-06
ENSG00000177084	0.02	2.23	2.19	4.88	6.47E-08	5.42E-06
ENSG00000136982	0.99	4.46	4.19	3.81	6.70E-08	5.58E-06
ENSG00000171320	-0.07	4.76	4.58	3.83	6.92E-08	5.73E-06
ENSG00000169908	1.18	3.57	5.06	3.25	7.48E-08	6.16E-06
ENSG00000156970	1.76	5.01	5.31	4.26	9.19E-08	7.52E-06
ENSG00000165244	-0.04	2.80	2.23	4.59	9.81E-08	7.99E-06
ENSG00000167306	-0.97	-2.67	-4.75	3.55	1.20E-07	9.64E-06
ENSG00000138160	0.05	3.23	3.66	6.05	1.32E-07	1.04E-05
ENSG00000189057	-0.19	3.88	3.37	4.08	1.36E-07	1.07E-05
ENSG00000135451	-0.09	3.31	3.97	3.03	1.54E-07	1.21E-05
ENSG00000276043	0.07	2.69	2.23	5.56	1.73E-07	1.34E-05
ENSG00000167670	0.01	2.41	2.16	4.55	1.85E-07	1.42E-05
ENSG00000156802	0.33	3.25	2.89	6.19	2.01E-07	1.53E-05
ENSG00000051341	0.53	4.21	4.11	3.68	2.05E-07	1.56E-05
ENSG00000100906	1.67	-0.87	-0.23	6.96	2.09E-07	1.58E-05
ENSG00000137831	0.00	2.30	1.72	6.81	2.49E-07	1.83E-05
ENSG00000117632	-0.01	1.81	2.57	8.36	2.64E-07	1.94E-05
ENSG00000162772	1.58	-3.01	-3.59	6.06	2.79E-07	2.04E-05
ENSG00000147883	1.36	-1.47	-2.87	3.72	3.59E-07	2.56E-05

ENSG00000176907	1.20	-1.74	-2.47	7.19	3.63E-07	2.57E-05
ENSG00000107984	-0.16	2.07	1.52	9.28	3.91E-07	2.75E-05
ENSG00000110031	-0.32	1.85	3.30	4.38	4.14E-07	2.90E-05
ENSG00000106462	0.62	3.33	2.81	4.39	4.46E-07	3.11E-05
ENSG00000165304	-0.38	2.37	2.36	5.19	5.32E-07	3.65E-05
ENSG00000163545	1.42	-1.57	-1.39	5.19	5.33E-07	3.65E-05
ENSG00000138166	1.24	-1.95	-2.01	7.12	5.36E-07	3.65E-05
ENSG00000139618	-0.03	3.88	3.86	3.36	5.50E-07	3.73E-05
ENSG00000092470	-0.09	3.01	2.74	4.86	6.05E-07	4.06E-05
ENSG00000073111	-0.05	2.29	1.86	5.84	6.13E-07	4.08E-05
ENSG00000085840	-0.02	4.14	3.64	3.07	6.14E-07	4.08E-05
ENSG00000137033	0.95	4.43	2.76	4.76	6.61E-07	4.35E-05
ENSG00000012048	0.13	2.99	2.48	4.59	6.68E-07	4.38E-05
ENSG00000269289	-0.64	-6.83	-3.25	2.36	6.73E-07	4.39E-05
ENSG00000176890	-0.07	2.76	2.67	4.32	6.88E-07	4.47E-05
ENSG00000137812	0.01	4.03	4.66	4.85	6.97E-07	4.51E-05
ENSG00000183763	-0.01	3.19	2.94	2.63	7.33E-07	4.67E-05
ENSG00000112118	-0.26	2.24	1.67	6.51	7.47E-07	4.74E-05
ENSG00000171223	1.12	-3.19	-2.49	7.19	9.10E-07	5.73E-05
ENSG00000077152	-0.51	2.30	2.47	4.19	9.79E-07	6.12E-05
ENSG00000168685	1.73	3.83	4.66	6.11	1.04E-06	6.40E-05
ENSG00000152253	0.69	3.66	3.56	3.34	1.07E-06	6.57E-05
ENSG00000137563	0.32	1.70	3.07	4.79	1.08E-06	6.57E-05
ENSG00000110427	0.17	3.55	3.72	4.22	1.28E-06	7.66E-05
ENSG00000142910	-1.22	-2.85	-4.45	6.91	1.30E-06	7.74E-05
ENSG00000144655	1.26	-2.21	-1.94	6.04	1.45E-06	8.51E-05
ENSG00000112029	-0.08	3.25	2.94	4.58	1.51E-06	8.77E-05
ENSG00000128805	-0.08	1.32	2.28	4.48	1.57E-06	9.05E-05
ENSG00000079616	-0.33	1.96	2.04	4.91	1.64E-06	9.43E-05
ENSG00000100162	-0.41	3.29	3.25	3.64	2.09E-06	0.0001178
ENSG00000163735	2.40	5.85	9.49	5.08	2.27E-06	0.0001269
ENSG00000167513	1.52	3.71	2.81	3.37	2.29E-06	0.00012764
ENSG00000175505	1.32	-1.44	-2.26	5.43	2.34E-06	0.00012973
ENSG00000121152	-0.49	3.34	3.02	4.15	2.35E-06	0.00012973
ENSG00000146232	1.13	-1.58	-1.06	5.31	3.15E-06	0.00017011
ENSG00000228716	0.45	2.25	2.64	5.36	3.70E-06	0.00019691
ENSG00000130816	0.13	1.85	1.79	6.69	3.94E-06	0.00020825
ENSG00000109674	0.38	3.66	3.88	2.97	4.21E-06	0.00022174
ENSG00000111247	0.50	3.90	3.50	4.10	4.39E-06	0.00023018
ENSG00000120129	1.10	-1.54	-1.58	7.29	4.41E-06	0.00023036
ENSG00000145358	-0.60	2.26	2.59	3.43	4.53E-06	0.00023562
ENSG00000106003	0.71	-4.29	-5.56	3.50	4.66E-06	0.00024056

ENSG00000168389	1.63	-2.35	-1.35	3.57	4.68E-06	0.00024076
ENSG00000165349	-0.66	-2.65	-4.52	3.84	4.69E-06	0.00024076
ENSG00000125347	1.54	-0.69	-0.83	6.70	5.07E-06	0.00025822
ENSG00000168496	-0.02	2.10	1.92	5.00	5.38E-06	0.00027224
ENSG00000116717	1.30	-1.09	-1.49	7.87	5.50E-06	0.00027625
ENSG00000165891	0.23	3.49	3.93	5.21	5.78E-06	0.00028932
ENSG00000170961	2.39	3.39	6.89	4.87	6.14E-06	0.00030618
ENSG00000111602	-0.12	1.96	1.76	5.42	6.69E-06	0.00032984
ENSG00000006210	1.06	-3.23	-2.84	4.06	6.76E-06	0.00033224
ENSG00000139734	0.10	2.06	2.40	5.41	7.12E-06	0.00034859
ENSG00000164087	0.58	2.54	2.68	4.31	7.27E-06	0.00035516
ENSG00000149503	-0.73	1.66	1.91	4.21	7.66E-06	0.00037261
ENSG00000167325	0.03	2.32	2.37	7.03	7.75E-06	0.0003757
ENSG00000102384	0.80	3.28	3.54	4.31	7.81E-06	0.00037725
ENSG00000221829	-0.51	2.01	1.64	4.19	8.89E-06	0.0004238
ENSG00000164104	-1.11	1.50	1.59	6.42	9.41E-06	0.000447
ENSG00000105486	-0.02	2.09	1.95	4.10	1.05E-05	0.00049536
ENSG00000197467	0.33	2.56	2.25	3.51	1.12E-05	0.00052901
ENSG00000118503	2.49	-0.89	0.22	5.89	1.16E-05	0.00054309
ENSG00000164045	1.67	4.63	3.94	3.28	1.21E-05	0.00055907
ENSG00000066279	0.02	3.73	4.53	4.89	1.21E-05	0.00055907
ENSG00000014138	-0.19	2.10	1.81	4.37	1.48E-05	0.00066911
ENSG00000136108	-0.12	2.35	2.88	5.76	1.61E-05	0.00072663
ENSG00000080839	0.05	2.60	2.52	4.01	1.64E-05	0.00073926
ENSG00000241749	0.85	3.93	4.37	2.69	1.84E-05	0.00082371
ENSG00000162267	-0.18	-3.94	-4.98	2.37	1.96E-05	0.00086906
ENSG00000136492	0.38	3.02	2.87	4.01	2.07E-05	0.00090887
ENSG00000101003	-0.03	2.71	2.38	4.15	2.22E-05	0.00095362
ENSG00000142731	0.53	3.67	3.96	3.61	2.23E-05	0.00095647
ENSG00000181938	-0.35	3.06	2.86	2.82	2.25E-05	0.00096057
ENSG00000214357	0.13	2.76	2.58	3.31	2.33E-05	0.00098563
ENSG00000010292	-0.03	1.62	1.99	6.59	2.45E-05	0.00102813
ENSG00000164619	0.94	4.34	3.77	4.29	2.76E-05	0.00113824
ENSG00000125538	1.38	1.67	4.66	3.53	2.97E-05	0.00121042
ENSG00000136244	2.43	-2.45	0.13	3.74	3.14E-05	0.00126962
ENSG00000213853	0.15	1.34	2.16	5.34	3.28E-05	0.00131958
ENSG00000138658	-0.80	2.29	1.96	3.34	3.39E-05	0.0013582
ENSG00000091409	-0.12	2.32	3.30	5.42	3.43E-05	0.00136756
ENSG00000184992	-0.19	2.28	1.67	5.01	3.43E-05	0.00136756
ENSG00000117266	-0.57	-4.50	-1.79	2.58	3.45E-05	0.00137274
ENSG00000104889	0.26	2.12	2.09	4.34	3.82E-05	0.00150689
ENSG00000171033	0.73	3.21	6.54	3.10	3.83E-05	0.00150761

ENSG00000095303	-0.01	1.51	2.14	5.45	4.06E-05	0.00157851
ENSG00000133110	0.90	4.32	5.10	3.11	4.07E-05	0.00157851
ENSG00000109685	-0.27	1.43	1.82	6.57	4.27E-05	0.00165337
ENSG00000166845	-0.56	2.97	3.15	2.95	4.43E-05	0.00170466
ENSG00000128342	1.32	-5.64	-4.48	7.77	4.58E-05	0.00174853
ENSG00000162607	-0.15	2.03	1.87	6.00	4.78E-05	0.00182066
ENSG00000278259	-0.23	1.78	1.40	4.69	4.86E-05	0.001845
ENSG00000114346	-0.30	2.06	2.81	5.04	5.08E-05	0.00192264
ENSG00000109881	-0.13	0.98	2.65	4.17	5.42E-05	0.00203556
ENSG00000000460	-0.74	2.45	2.27	3.62	5.66E-05	0.00212166
ENSG00000101868	-0.19	1.98	1.48	4.81	5.89E-05	0.00219505
ENSG00000176619	-0.05	0.91	1.73	6.45	5.94E-05	0.00220746
ENSG00000180875	-0.33	1.72	1.27	5.05	6.33E-05	0.00232851
ENSG00000186638	0.42	3.33	2.50	2.87	6.33E-05	0.00232851
ENSG00000144354	1.20	2.83	3.70	2.58	6.35E-05	0.00232851
ENSG00000123473	0.57	2.80	2.79	4.29	6.51E-05	0.00238212
ENSG00000121621	1.08	3.32	4.23	3.83	7.02E-05	0.00252246
ENSG00000121211	0.70	3.50	3.00	2.82	7.52E-05	0.00269654
ENSG00000146263	0.09	2.71	2.21	4.21	7.54E-05	0.00269654
ENSG00000171208	0.82	3.06	3.01	3.97	7.94E-05	0.00283269
ENSG00000125885	-0.45	2.30	1.90	4.51	8.31E-05	0.0029218
ENSG00000188486	0.30	1.98	1.79	5.59	8.34E-05	0.0029218
ENSG00000132436	0.45	2.20	2.28	4.39	8.36E-05	0.002924
ENSG00000179750	0.33	2.80	2.94	2.65	8.40E-05	0.00292833
ENSG00000127586	-0.53	2.54	2.57	2.50	8.53E-05	0.00295899
ENSG00000133392	1.31	-0.80	-2.69	2.58	8.60E-05	0.00297585
ENSG00000144810	0.61	1.79	1.93	5.57	8.72E-05	0.00301275
ENSG00000262406	1.10	2.84	2.79	4.10	9.03E-05	0.00309703
ENSG00000185697	0.63	3.47	3.45	4.19	9.43E-05	0.00321848
ENSG00000013573	-0.24	2.45	2.13	2.69	9.76E-05	0.00332359
ENSG00000137309	0.51	1.53	2.25	8.08	0.00010777	0.00362463
ENSG00000153044	0.02	1.77	2.61	3.89	0.00010777	0.00362463
ENSG00000075213	0.37	1.91	0.58	7.00	0.00011153	0.00372443
ENSG00000173207	-0.66	1.39	1.98	4.50	0.00011217	0.00372816
ENSG00000130695	-1.34	1.72	1.43	2.93	0.00011422	0.00378757
ENSG00000176208	1.29	4.01	3.88	2.77	0.00011585	0.00382132
ENSG00000147536	0.15	2.62	2.49	4.30	0.00011605	0.00382132
ENSG00000005513	0.21	-5.82	-2.86	2.47	0.00012015	0.00394715
ENSG00000132646	-0.03	2.19	1.74	7.80	0.00012483	0.00407499
ENSG00000128578	-1.00	2.24	2.24	2.34	0.00012499	0.00407499
ENSG00000159259	-0.03	2.61	1.83	4.03	0.0001346	0.00435099
ENSG00000213551	-0.54	1.67	1.63	3.74	0.0001389	0.00447985

ENSG00000129534	0.32	2.28	2.76	4.11	0.000158	0.00506145
ENSG00000151503	-0.22	1.94	1.80	4.88	0.00015937	0.00509364
ENSG00000183814	-0.47	2.39	2.93	2.52	0.00016926	0.00537278
ENSG00000172061	0.04	1.30	2.10	7.55	0.00017142	0.0054104
ENSG00000178966	-0.45	2.35	2.26	3.24	0.00017463	0.00549449
ENSG00000137807	0.44	2.28	2.42	6.82	0.00017649	0.00553163
ENSG00000136824	-0.01	2.08	2.29	5.22	0.00017659	0.00553163
ENSG00000139354	0.83	3.43	4.41	3.15	0.00018813	0.00585447
ENSG00000120539	0.47	2.29	1.94	5.16	0.00018959	0.0058869
ENSG00000051180	-0.07	2.68	1.99	3.29	0.00019222	0.00595539
ENSG00000123416	0.44	2.01	1.99	7.79	0.00019289	0.0059631
ENSG00000169213	0.41	1.85	1.53	8.35	0.00019528	0.00599776
ENSG00000058804	0.03	1.87	2.15	5.70	0.00020396	0.00618384
ENSG00000123374	-0.10	1.66	1.50	5.75	0.00020762	0.0062681
ENSG00000198056	0.56	3.48	3.31	3.00	0.00021116	0.0063612
ENSG0000111331	-0.20	1.08	1.84	4.44	0.00023091	0.00689771
ENSG00000185361	0.80	0.68	2.86	2.78	0.0002327	0.00692548
ENSG00000122861	0.92	2.38	2.71	7.54	0.0002488	0.00733909
ENSG00000131979	1.36	-1.71	-1.31	3.53	0.00025136	0.00739926
ENSG00000138395	0.15	2.09	2.15	3.14	0.0002822	0.00820469
ENSG00000125148	1.19	2.84	3.25	9.23	0.00029049	0.00839826
ENSG00000144802	2.35	-0.28	0.47	6.03	0.00031992	0.00913259
ENSG00000152457	0.03	1.46	2.22	3.24	0.00032173	0.00914622
ENSG00000251562	0.54	-0.66	2.93	10.88	0.00032298	0.00914622
ENSG00000031691	-0.32	2.00	2.16	4.07	0.00032608	0.00921545
ENSG00000197299	-0.50	3.27	3.03	2.93	0.00032898	0.00926043
ENSG00000057019	-0.08	1.90	2.12	8.40	0.00033064	0.00928876
ENSG00000062822	-0.14	1.94	1.56	3.39	0.00034053	0.00951006
ENSG00000135476	0.53	2.29	2.01	4.14	0.00036896	0.01016365
ENSG00000163814	0.26	2.79	4.19	3.81	0.00037183	0.01021734
ENSG00000101670	1.15	-1.59	-2.77	2.83	0.00037411	0.01021734
ENSG00000138669	0.16	2.16	1.93	3.88	0.00037439	0.01021734
ENSG00000011201	-0.64	3.95	4.88	2.43	0.00037452	0.01021734
ENSG00000006468	-1.14	1.02	2.50	3.03	0.0003775	0.01027875
ENSG00000184445	0.18	2.29	2.20	5.37	0.00038392	0.01043365
ENSG00000198576	1.64	-4.85	-3.67	2.90	0.00039828	0.01080328
ENSG00000119707	0.28	0.07	2.18	8.37	0.0004261	0.01142647
ENSG00000146410	-0.20	2.53	2.86	2.99	0.0004303	0.01151722
ENSG00000157601	-0.47	-1.71	1.61	5.51	0.00045501	0.01213276
ENSG00000077514	-0.28	1.94	1.10	4.58	0.00048338	0.01274534
ENSG00000049541	0.54	2.03	1.61	4.85	0.0004909	0.01289
ENSG00000158050	2.41	-2.86	-2.01	2.64	0.00049125	0.01289

ENSG00000013297	0.15	1.26	1.73	6.66	0.00050076	0.01307254
ENSG00000178878	-0.34	2.06	1.50	3.58	0.00050379	0.01311236
ENSG00000100526	-0.12	2.40	3.38	2.57	0.00051905	0.01348469
ENSG00000159388	1.01	-0.94	-0.93	5.31	0.00058088	0.01479338
ENSG00000166250	0.19	1.69	1.24	8.22	0.00058091	0.01479338
ENSG00000115687	-0.57	2.30	1.63	2.64	0.00058593	0.01489444
ENSG00000123136	0.46	1.27	1.78	5.48	0.00060528	0.01533127
ENSG00000121957	-0.84	0.55	1.70	3.57	0.00065267	0.01629842
ENSG00000133119	0.08	2.91	2.71	4.89	0.00066495	0.01657563
ENSG00000030419	-1.59	1.18	1.54	2.93	0.00066621	0.01657789
ENSG00000166004	-0.42	1.76	1.59	3.41	0.00067209	0.01669504
ENSG00000108691	2.19	-2.42	-0.76	7.18	0.00070236	0.01735547
ENSG00000176974	-0.45	1.19	1.61	3.87	0.00070659	0.01742964
ENSG00000149948	0.97	2.68	3.27	6.54	0.00076471	0.01866826
ENSG00000163739	4.12	0.05	4.15	5.81	0.00076687	0.01868873
ENSG00000123496	0.92	4.12	3.29	4.19	0.00076938	0.0187177
ENSG00000168393	-0.04	1.34	1.46	5.28	0.00078158	0.01894922
ENSG00000170779	0.50	2.28	2.05	3.22	0.00079188	0.01913358
ENSG00000160957	-0.44	2.00	1.31	2.91	0.00079982	0.01925971
ENSG00000072501	-0.16	1.27	1.22	6.48	0.00086195	0.02065044
ENSG00000105738	0.17	1.25	1.70	4.67	0.00091088	0.02169565
ENSG00000023171	0.36	2.31	2.23	2.81	0.00093846	0.02229475
ENSG00000079156	0.05	1.74	1.90	3.51	0.0009721	0.02297813
ENSG00000100578	-0.27	1.19	1.71	4.63	0.00098843	0.02328646
ENSG00000151014	2.03	-0.03	-0.27	3.32	0.00100873	0.02363885
ENSG00000122483	0.54	1.73	2.17	3.47	0.00101006	0.02363885
ENSG00000257219	1.21	2.82	2.13	2.91	0.00101662	0.02367498
ENSG00000097021	0.54	1.73	1.58	5.06	0.00102879	0.02391883
ENSG00000103995	0.05	2.35	1.89	3.19	0.00104914	0.02431229
ENSG00000140534	0.38	2.44	1.72	3.13	0.00106408	0.02461819
ENSG00000149554	-0.21	1.56	1.66	4.89	0.00112177	0.02574252
ENSG00000100714	0.26	1.51	1.34	6.65	0.00116896	0.0266098
ENSG00000156113	0.32	1.82	0.73	5.52	0.00117238	0.02664478
ENSG00000069399	1.07	-1.57	-0.63	5.31	0.00121622	0.02732448
ENSG00000005189	-0.26	1.84	1.82	3.22	0.0012188	0.02732448
ENSG00000157193	0.29	1.84	1.73	3.60	0.00124484	0.02780068
ENSG00000181751	0.15	1.78	1.72	6.26	0.00133375	0.02950718
ENSG00000117155	0.05	1.91	1.97	5.79	0.00135171	0.02985776
ENSG00000118655	0.14	2.30	1.83	3.24	0.00136987	0.0301649
ENSG00000143493	-0.01	1.74	1.50	4.43	0.00142923	0.0313744
ENSG00000101911	0.06	1.90	1.50	4.75	0.00152164	0.03299377
ENSG00000095002	0.28	1.85	1.48	5.84	0.0015538	0.03350728

ENSG00000138376	0.40	2.59	2.69	3.95	0.00155479	0.03350728
ENSG00000205208	-0.77	1.35	1.34	4.62	0.00156651	0.03360631
ENSG00000187173	0.25	2.04	0.77	3.54	0.00161291	0.03434168
ENSG00000120802	0.09	1.82	2.28	6.45	0.0016249	0.03444158
ENSG00000182481	-0.05	1.26	1.65	8.05	0.00163251	0.03445568
ENSG00000148848	0.59	1.59	1.54	7.10	0.00163336	0.03445568
ENSG00000145147	0.39	2.07	2.20	6.44	0.0016555	0.03472635
ENSG00000187257	-0.10	0.27	1.68	3.98	0.00165859	0.03473951
ENSG00000120549	0.09	1.94	2.54	2.47	0.00178047	0.03707305
ENSG00000138092	0.34	2.12	1.70	4.42	0.00180923	0.03756136
ENSG00000050438	-0.12	1.49	2.19	3.07	0.00182254	0.03772718
ENSG00000164171	0.60	2.78	2.60	6.53	0.00188488	0.03867823
ENSG00000095752	1.30	-2.81	-2.40	3.93	0.00191864	0.03925735
ENSG00000172167	-0.54	2.43	2.57	2.64	0.00194844	0.03975208
ENSG00000124207	0.06	1.83	1.90	7.17	0.00202822	0.04108375
ENSG00000177606	1.01	-0.56	-0.58	7.55	0.00215848	0.04316652
ENSG00000145779	1.43	-0.15	-0.02	5.17	0.00221988	0.04414513
ENSG00000143401	0.05	0.98	1.62	5.86	0.00230601	0.04562559
ENSG00000175166	0.18	1.22	1.28	9.12	0.00232428	0.04583077
ENSG00000041353	0.38	2.34	2.66	2.60	0.0023753	0.04658158
ENSG00000171914	-0.17	1.30	0.95	6.09	0.00239954	0.04699186
ENSG00000099250	-0.13	1.32	1.21	8.04	0.00246951	0.04783278
ENSG00000164985	-0.09	0.95	1.38	5.56	0.00253896	0.04891039
ENSG00000163781	-0.01	1.51	1.39	5.51	0.00254399	0.04894069
ENSG00000117748	0.21	1.86	1.16	4.79	0.00257758	0.04938557

cells infected with T. gondii EGS strain for 2, 18 and 48

Product Name
ATP-binding cassette, sub-family B (MDR/TAP), member 5
alcohol dehydrogenase 1B (class I), beta polypeptide
cytoskeleton associated protein 2-like
baculoviral IAP repeat containing 5
topoisomerase (DNA) II alpha 170kDa
v-myb avian myeloblastosis viral oncogene homolog-like 2
marker of proliferation Ki-67
CD14 molecule
myopalladin
centrosomal protein 55kDa
endothelin 1
spindle and kinetochore associated complex subunit 1
proline rich 11
nuclear receptor subfamily 4, group A, member 1
cyclin A2
hyaluronan-mediated motility receptor (RHAMM)
claspin
cell division cycle 45
kinesin family member 2C
BUB1 mitotic checkpoint serine/threonine kinase
forkhead box M1
non-SMC condensin I complex, subunit G
TPX2, microtubule-associated
centromere protein F, 350/400kDa
family with sequence similarity 83, member D
PDZ binding kinase
SPC24, NDC80 kinetochore complex component
kinesin family member C1
cell division cycle associated 3
centromere protein E, 312kDa
matrix metallopeptidase 1
cell division cycle associated 5

protein regulator of cytokinesis 1
excision repair cross-complementation group 6-like
kinesin family member 4A
TTK protein kinase
spindle and kinetochore associated complex subunit 3
MAD2 mitotic arrest deficient-like 1 (yeast)
sperm associated antigen 5
nuclear receptor subfamily 4, group A, member 3
denticleless E3 ubiquitin protein ligase homolog (Drosophila)
mitogen-activated protein kinase kinase kinase 8
Rho GTPase activating protein 11A
transforming, acidic coiled-coil containing protein 3
NIPA-like domain containing 4
immediate early response 3
DEP domain containing 1
kinesin family member 20A
Ras-related associated with diabetes
cell division cycle 25C
colony stimulating factor 3 (granulocyte)
SHC SH2-domain binding protein 1
ZW10 interacting kinetochore protein
zinc finger CCCH-type containing 12A
GINS complex subunit 2 (Psf2 homolog)
thymidine kinase 1, soluble
NUF2, NDC80 kinetochore complex component
matrix metallopeptidase 3
indolethylamine N-methyltransferase
cell division cycle 20
ubiquitin-conjugating enzyme E2C
cell division cycle associated 8
adenylate kinase 5
early growth response 2
IQ motif containing GTPase activating protein 3
geminin coiled-coil domain containing
anillin, actin binding protein
thyroid hormone receptor interactor 13
NDC80 kinetochore complex component
intercellular adhesion molecule 1
parathyroid hormone-like hormone
kinesin family member 14
early growth response 3
Pim-1 proto-oncogene, serine/threonine kinase

shugoshin-like 2 (S. pombe)
WD repeat domain 62
centromere protein U
kinesin family member 15
transcription factor 19
minichromosome maintenance complex component 10
kinesin family member 18B
citron rho-interacting serine/threonine kinase
Rac GTPase activating protein 1
aurora kinase A
cyclin F
neurotrimin
ribonucleotide reductase M2
serpin peptidase inhibitor, clade B (ovalbumin), member 7
gamma-aminobutyric acid (GABA) B receptor, 2
aurora kinase B
discs, large (Drosophila) homolog-associated protein 5
G-2 and S-phase expressed 1
minichromosome maintenance complex component 5
kinesin family member 20B
cell division cycle 6
lamin B1
cyclin-dependent kinase 1
suppressor of cytokine signaling 3
cyclin B1
minichromosome maintenance complex component 4
alanyl (membrane) aminopeptidase
collagen, type XIV, alpha 1
helicase, lymphoid-specific
WD repeat and HMG-box DNA binding protein 1
TNF receptor-associated factor 1
tumor necrosis factor, alpha-induced protein 2
metallothionein 1L (gene/pseudogene)
KIAA0101
polo-like kinase 1
procollagen C-endopeptidase enhancer 2
KIAA1524
anti-silencing function 1B histone chaperone
nucleolar and spindle associated protein 1
chemokine (C-X-C motif) ligand 6
Fanconi anemia, complementation group D2
RAD54-like (S. cerevisiae)

Fanconi anemia, complementation group I
tenascin C
pituitary tumor-transforming 1
FBJ murine osteosarcoma viral oncogene homolog B
geminin, DNA replication inhibitor
cell division cycle associated 2
NIMA-related kinase 2
cyclin B2
xylosyltransferase I
Fanconi anemia, complementation group A
Holliday junction recognition protein
proline/serine-rich coiled-coil 1
paternally expressed 10
Pim-3 proto-oncogene, serine/threonine kinase
E2F transcription factor 1
non-SMC condensin II complex, subunit G2
structural maintenance of chromosomes 4
minichromosome maintenance complex component 7
endothelial cell-specific molecule 1
centromere protein A
regulator of G-protein signaling 16
chromosome 11 open reading frame 96
lysine (K)-specific demethylase 6B
polymerase (DNA directed), epsilon, catalytic subunit
DNA replication and sister chromatid cohesion 1
establishment of sister chromatid cohesion N-acetyltransferase
transmembrane 4 L six family member 1
BUB1 mitotic checkpoint serine/threonine kinase B
zinc finger protein 367
myosin VB
kinesin family member 11
family with sequence similarity 111, member B
trophinin associated protein
ubiquitin-like with PHD and ring finger domains 1
chromatin assembly factor 1, subunit A (p150)
ATPase family, AAA domain containing 2
polymerase (DNA directed), theta
nuclear factor of kappa light polypeptide gene enhancer in B-c
uveal autoantigen with coiled-coil domains and ankyrin repeats
stathmin 1
activating transcription factor 3
cyclin-dependent kinase inhibitor 2B (p15, inhibits CDK4)

chromosome 8 open reading frame 4
dickkopf WNT signaling pathway inhibitor 1
leupaxin
enhancer of zeste 2 polycomb repressive complex 2 subunit
maternal embryonic leucine zipper kinase
NUAK family, SNF1-like kinase, 2
dual specificity phosphatase 5
breast cancer 2, early onset
WD repeat domain 76
minichromosome maintenance complex component 2
origin recognition complex, subunit 1
interleukin 33
breast cancer 1, early onset
CTB-92J24.3
thymidylate synthetase
cancer susceptibility candidate 5
TRAF interacting protein
minichromosome maintenance complex component 3
jun B proto-oncogene
ubiquitin-conjugating enzyme E2T
interleukin 7 receptor
SPC25, NDC80 kinetochore complex component
gamma-glutamyl hydrolase (conjugase, folylpolygammaglutam
KIAA1549-like
tubulointerstitial nephritis antigen-like 1
cysteine-serine-rich nuclear protein 1
F-box protein 5
Rho GTPase activating protein 22
kinesin family member 22
centromere protein M
chemokine (C-X-C motif) ligand 5
chromatin licensing and DNA replication factor 1
cardiotrophin-like cytokine factor 1
non-SMC condensin I complex, subunit H
nuclear factor of kappa light polypeptide gene enhancer in B-c
dihydrofolate reductase
DNA (cytosine-5-)-methyltransferase 1
nei endonuclease VIII-like 3 (E. coli)
RAD51 associated protein 1
dual specificity phosphatase 1
DNA-damage-inducible transcript 4-like
LFNG O-fucosylpeptide 3-beta-N-acetylglucosaminyltransferas

major facilitator superfamily domain containing 2A
solute carrier family 7 (cationic amino acid transporter, y+ syste
interferon regulatory factor 1
flap structure-specific endonuclease 1
growth arrest and DNA-damage-inducible, alpha
E2F transcription factor 7
hyaluronan synthase 2
timeless circadian clock
chemokine (C-X3-C motif) ligand 1
diaphanous-related formin 3
POC1 centriolar protein A
inner centromere protein antigens 135/155kDa
ribonucleotide reductase M1
centromere protein I
Fanconi anemia, complementation group G
high mobility group box 2
ligase I, DNA, ATP-dependent
collagen, type XIII, alpha 1
tumor necrosis factor, alpha-induced protein 3
cell division cycle 25A
asp (abnormal spindle) homolog, microcephaly associated (Dr
polymerase (DNA directed), alpha 2, accessory subunit
cytoskeleton associated protein 2
retinoblastoma-like 1
ribosomal protein SA pseudogene 52
inter-alpha-trypsin inhibitor heavy chain 3
BRCA1 interacting protein C-terminal helicase 1
GINS complex subunit 1 (Psf1 homolog)
polo-like kinase 4
GINS complex subunit 3 (Psf3 homolog)
neuralized E3 ubiquitin protein ligase 1B
non-SMC condensin I complex, subunit D2
BMP binding endothelial regulator
interleukin 1, beta
interleukin 6
epithelial membrane protein 2
zinc finger, GRF-type containing 1
integrin, alpha 6
BRI3 binding protein
cyclin-dependent kinase 18
ribonuclease H2, subunit A
protein kinase (cAMP-dependent, catalytic) inhibitor alpha

prostaglandin-endoperoxide synthase 1 (prostaglandin G/H sy
periostin, osteoblast specific factor
Wolf-Hirschhorn syndrome candidate 1
chromosome 18 open reading frame 54
leukemia inhibitory factor
ubiquitin specific peptidase 1
myosin XIX
epithelial cell transforming 2
coiled-coil domain containing 34
chromosome 1 open reading frame 112
polymerase (DNA directed), alpha 1, catalytic subunit
lamin B2
gremlin 2, DAN family BMP antagonist
kinesin family member 24
cell division cycle associated 7
SCL/TAL1 interrupting locus
kinesin family member 18A
meiotic nuclear divisions 1 homolog (S. cerevisiae)
MMS22-like, DNA repair protein
neuropilin (NRP) and tolloid (TLL)-like 2
minichromosome maintenance complex component 8
H2A histone family, member X
fidgetin-like 1
apolipoprotein B mRNA editing enzyme, catalytic polypeptide-l
CTF18, chromosome transmission fidelity factor 18 homolog (S
myosin, heavy chain 11, smooth muscle
collagen, type VIII, alpha 1
matrix metallopeptidase 12
v-myb avian myeloblastosis viral oncogene homolog-like 1
DEAD/H (Asp-Glu-Ala-Asp/His) box helicase 11
high mobility group AT-hook 1
centromere protein H
sema domain, immunoglobulin domain (Ig), short basic domair
CDC28 protein kinase regulatory subunit 1B
centrosomal protein 85kDa
ATPase family, AAA domain containing 5
GINS complex subunit 4 (Sld5 homolog)
SRY (sex determining region Y)-box 8
proliferating cell nuclear antigen
striatin interacting protein 2
chromatin assembly factor 1, subunit B (p60)
DnaJ (Hsp40) homolog, subfamily C, member 9

MIS18 binding protein 1
non-SMC condensin II complex, subunit D3
lin-9 DREAM MuvB core complex component
leucine rich repeat containing 15
RecQ mediated genome instability 1
kinesin family member 23
structural maintenance of chromosomes 2
growth arrest-specific 2 like 3
microtubule associated serine/threonine kinase-like
RAD51 recombinase
tubulin, alpha 1b
RAB3B, member RAS oncogene family
NDC1 transmembrane nucleoporin
cyclin-dependent kinase 2
primase, DNA, polypeptide 1 (49kDa)
2'-5'-oligoadenylate synthetase 3, 100kDa
tumor necrosis factor, alpha-induced protein 8-like 1
plasminogen activator, urokinase
GTP cyclohydrolase 1
cyclin-dependent kinase 15
metallothionein 2A
nuclear factor of kappa light polypeptide gene enhancer in B-c
DNA cross-link repair 1C
metastasis associated lung adenocarcinoma transcript 1 (non-
centromere protein Q
Bloom syndrome, RecQ helicase-like
discoidin, CUB and LCCL domain containing 2
polymerase (DNA directed), delta 1, catalytic subunit
extra spindle pole bodies homolog 1 (S. cerevisiae)
CUB domain containing protein 1
lipase, endothelial
protein kinase, cGMP-dependent, type II
anosmin 1
ets variant 1
kinetochore associated 1
activity-regulated cytoskeleton-associated protein
RNA binding motif protein 25
mitochondrial fission regulator 2
MX dynamin-like GTPase 1
polymerase (DNA-directed), delta 3, accessory subunit
replication factor C (activator 1) 2, 40kDa
dual specificity phosphatase 2

claudin 11
apolipoprotein L domain containing 1
cyclin-dependent kinase inhibitor 3
BTG family, member 2
CXADR-like membrane protein
PAS domain containing serine/threonine kinase
DEAD (Asp-Glu-Ala-Asp) box polypeptide 39A
G-protein signaling modulator 2
replication factor C (activator 1) 3, 38kDa
IKAROS family zinc finger 2 (Helios)
centrosomal protein 295kDa
chemokine (C-C motif) ligand 2
serine hydroxymethyltransferase 1 (soluble)
high mobility group AT-hook 2
chemokine (C-X-C motif) ligand 1 (melanoma growth stimulatin
interleukin 13 receptor, alpha 2
deoxythymidylate kinase (thymidylate kinase)
cell division cycle associated 4
RecQ protein-like 4
structural maintenance of chromosomes 1A
signal-induced proliferation-associated 1 like 3
GRAM domain containing 1B
oxysterol binding protein-like 6
KIAA0586
CCR4 carbon catabolite repression 4-like (S. cerevisiae)
coiled-coil domain containing 18
RP11-54A9.1
acyl-CoA thioesterase 7
centrosomal protein 152kDa
TOPBP1-interacting checkpoint and replication regulator
checkpoint kinase 1
methylenetetrahydrofolate dehydrogenase (NADP+ dependent
potassium channel, calcium activated large conductance subfa
B-cell CLL/lymphoma 3
Putative RNA exonuclease NEF-sp
low density lipoprotein receptor-related protein 8, apolipoprotei
chromosome 5 open reading frame 30
synovial sarcoma, X breakpoint 2 interacting protein
DNA cross-link repair 1B
integrator complex subunit 7
phosphoribosyl pyrophosphate synthetase 2
mutS homolog 2

BRCA1 associated RING domain 1
chromosome 4 open reading frame 46
late cornified envelope 2A
thymopoietin
karyopherin alpha 2 (RAG cohort 1, importin alpha 1)
ADAM metallopeptidase domain 12
slit homolog 2 (Drosophila)
round spermatid basic protein 1-like
KIAA1217
centromere protein O
solute carrier family 4, sodium bicarbonate cotransporter, mem
integrin, alpha 2 (CD49B, alpha 2 subunit of VLA-2 receptor)
interleukin 11
MDM2 binding protein
CSE1 chromosome segregation 1-like (yeast)
jun proto-oncogene
tumor necrosis factor, alpha-induced protein 8
acidic (leucine-rich) nuclear phosphoprotein 32 family, member
proteasome (prosome, macropain) 26S subunit, non-ATPase,
RAB27B, member RAS oncogene family
talin 2
neuropilin 1
PC4 and SFRS1 interacting protein 1
topoisomerase (DNA) II binding protein 1
replication protein A2, 32kDa

Supplementary Table S3A: Differencial T. gondii gene expressin analysis of human HFF cells infected with T. gondii EGS strain for 2 hours compared to the average T. gondii gene expression of human MM6 and neuronal stem cells infected with either GT1, ME49 or VEG strains of T. gondii for 18 hours.

gene ID	logFC	logCPM	PValue	FDR	Product name
TGME49_462910	13.60	7.86	4.06E-197	3.14E-193	large subunit ribosomal RNA
TGME49_457990	15.26	11.10	1.06E-128	4.09E-125	28S ribosomal RNA
TGME49_200010	11.50	6.04	5.76E-92	1.49E-88	hypothetical protein
TGME49_208370	3.55	9.24	1.07E-58	2.07E-55	myosin heavy chain, putative
TGME49_280570	4.82	8.90	4.21E-58	6.50E-55	SAG-related sequence SRS35A
TGME49_251180	3.93	6.82	2.38E-48	3.07E-45	KRUF family protein
TGME49_275860	3.00	9.69	2.06E-47	2.27E-44	hypothetical protein
TGME49_252430	2.95	8.32	2.45E-44	2.37E-41	hypothetical protein
TGME49_290700	2.81	8.51	1.41E-40	1.21E-37	hypothetical protein
TGME49_205250	3.16	9.76	1.25E-39	9.69E-37	rhoptry protein ROP18
TGME49_321480	6.55	5.82	2.31E-38	1.63E-35	SAG-related sequence SRS12B
TGME49_316250	3.42	9.32	2.77E-37	1.79E-34	hypothetical protein
TGME49_206550	4.76	6.24	5.30E-37	3.15E-34	hypothetical protein
TGME49_293790	4.78	6.58	6.49E-36	3.59E-33	hypothetical protein
TGME49_204050	3.32	11.00	3.38E-34	1.74E-31	subtilisin SUB1
TGME49_226380	2.73	8.78	1.34E-32	6.09E-30	hypothetical protein
TGME49_214980	2.49	8.60	1.34E-32	6.09E-30	hypothetical protein
TGME49_275460	3.28	7.38	1.90E-31	8.17E-29	hypothetical protein
TGME49_212300	2.42	8.95	4.41E-31	1.79E-28	hypothetical protein
TGME49_311100	2.17	9.34	1.02E-30	3.95E-28	zinc finger (CCCH type) motif-containing protein
TGME49_214080	3.25	9.15	6.28E-29	2.31E-26	toxofilin
TGME49_289920	2.39	8.81	1.34E-28	4.72E-26	hypothetical protein
TGME49_312420	2.29	8.62	1.65E-28	5.54E-26	hypothetical protein
TGME49_270700	2.58	7.38	3.21E-28	1.03E-25	hypothetical protein
TGME49_215960	2.54	9.11	7.59E-28	2.35E-25	hypothetical protein
TGME49_249990	2.71	10.11	1.62E-27	4.81E-25	hypothetical protein
TGME49_205680	2.76	8.25	2.41E-27	6.91E-25	hypothetical protein
TGME49_288475	7.24	6.01	2.97E-27	8.20E-25	hypothetical protein
TGME49_287040	2.75	7.28	4.41E-27	1.18E-24	hypothetical protein
TGME49_301250	3.63	14.08	1.40E-26	3.60E-24	hypothetical protein
TGME49_240060	2.32	9.26	1.51E-26	3.76E-24	hypothetical protein
TGME49_208450	2.27	8.91	6.15E-26	1.46E-23	protease inhibitor PI2
TGME49_233450	3.57	8.80	6.22E-26	1.46E-23	SAG-related sequence SRS29A
TGME49_213280	2.23	9.31	7.55E-26	1.72E-23	SAG-related sequence SRS25
TGME49_230830	3.75	6.09	9.18E-26	2.03E-23	ATPase family associated with various cellular activities (AAA) domain-containing protein
TGME49_308020	2.11	8.94	2.67E-25	5.73E-23	SAG-related sequence SRS57
TGME49_207400	2.61	8.87	4.74E-25	9.91E-23	hypothetical protein
TGME49_220380	2.75	6.97	9.11E-25	1.85E-22	hypothetical protein
TGME49_272370	3.11	8.31	2.56E-24	5.08E-22	hypothetical protein
TGME49_320490	2.28	8.94	3.71E-24	7.16E-22	N-acyl-phosphatidylethanolamine-hydrolyzing phospholipase D family protein
TGME49_207210	3.66	7.68	3.80E-24	7.16E-22	hypothetical protein

TGME49_249030	2.30	7.90	4.93E-24	9.08E-22	endonuclease/exonuclease/phosphatase family protein
TGME49_275870	3.61	6.07	6.84E-24	1.23E-21	tubulin/FtsZ family, GTPase domain-containing protein
TGME49_289050	2.92	8.45	1.38E-23	2.42E-21	FIKK kinase, putative
TGME49_214410	2.75	7.87	1.55E-23	2.66E-21	hypothetical protein
TGME49_289600	2.17	8.64	2.63E-23	4.41E-21	heat shock protein HSP29
TGME49_237230	1.97	9.94	3.84E-23	6.31E-21	hypothetical protein
TGME49_313440	3.95	8.63	4.20E-23	6.77E-21	hypothetical protein
TGME49_226540	3.06	7.21	4.37E-23	6.89E-21	protein kinase
TGME49_289630	2.58	7.72	5.63E-23	8.71E-21	microneme protein MIC16
TGME49_206510	2.16	9.01	8.29E-23	1.26E-20	toxolysin TLN4
TGME49_260310	3.51	6.15	1.26E-22	1.87E-20	ATP-binding cassette transporter ABC.B1
TGME49_294200	2.28	10.19	5.00E-22	7.30E-20	glucose-6-phosphate 1-dehydrogenase
TGME49_216820	2.24	8.73	6.17E-22	8.84E-20	transporter, major facilitator family protein
TGME49_321530	1.96	8.88	7.13E-22	1.00E-19	cathepsin CPL
TGME49_252390	2.25	8.81	2.02E-21	2.79E-19	hypothetical protein
TGME49_254460	2.40	7.76	3.88E-21	5.26E-19	hypothetical protein
TGME49_220950	2.53	10.12	5.99E-21	7.84E-19	hypothetical protein
TGME49_217680	1.82	9.52	1.67E-20	2.15E-18	hypothetical protein
TGME49_201785	2.09	9.22	2.15E-20	2.73E-18	hypothetical protein
TGME49_214575	3.21	6.21	2.91E-20	3.63E-18	hypothetical protein
TGME49_283540	2.04	8.69	8.08E-20	9.91E-18	hypothetical protein
TGME49_323110	3.13	6.57	8.71E-20	1.04E-17	hypothetical protein
TGME49_250115	2.68	8.62	8.73E-20	1.04E-17	hypothetical protein
TGME49_243930	1.98	8.91	1.24E-19	1.46E-17	hypothetical protein
TGME49_314970	-5.24	7.00	2.79E-19	3.21E-17	root hair defective 3 gtp-binding protein (rhd3) protein
TGME49_218520	1.83	10.68	3.02E-19	3.43E-17	microneme protein MIC6
TGME49_257380	1.95	8.77	3.11E-19	3.49E-17	hypothetical protein
TGME49_251170	3.55	6.54	1.09E-18	1.20E-16	KRUF family protein
TGME49_276170	2.06	9.19	1.70E-18	1.85E-16	phosphatidylinositol 3- and 4-kinase
TGME49_219120	1.77	8.62	3.72E-18	4.00E-16	zinc finger (CCCH type) motif-containing protein
TGME49_236890	2.15	7.52	3.83E-18	4.05E-16	hypothetical protein
TGME49_277080	1.90	10.94	2.69E-17	2.73E-15	microneme protein MIC5
TGME49_228170	2.17	9.45	2.77E-17	2.78E-15	inner membrane complex protein IMC2A
TGME49_225540	1.96	8.19	3.10E-17	3.07E-15	hypothetical protein
TGME49_233140	-10.27	6.89	5.69E-17	5.50E-15	deoxyuridine 5'-triphosphate nucleotidohydrolase, putative
TGME49_259950	2.49	7.58	6.12E-17	5.84E-15	carbonate dehydratase, eukaryotic-type domain-containing protein
TGME49_214940	1.94	10.92	6.55E-17	6.17E-15	MIC2-associated protein M2AP
TGME49_305980	-10.15	7.01	8.00E-17	7.39E-15	pyruvate dehydrogenase complex subunit PDH-E3I
TGME49_243730	1.73	10.33	8.03E-17	7.39E-15	rhoptry protein ROP9
TGME49_286470	2.65	8.81	8.37E-17	7.61E-15	AGC kinase
TGME49_202020	2.76	8.16	1.12E-16	1.00E-14	DnAK-TPR
TGME49_222130	2.68	7.05	1.44E-16	1.28E-14	hypothetical protein
TGME49_321360	2.18	7.92	1.61E-16	1.41E-14	clustered-asparagine-rich protein
TGME49_206610	-9.64	6.27	1.70E-16	1.46E-14	pyruvate dehydrogenase complex subunit PDH-E2
TGME49_226020	1.83	9.12	1.81E-16	1.54E-14	transporter, major facilitator family protein

TGME49_212900	1.93	7.94	2.67E-16	2.24E-14	hypothetical protein
TGME49_298980	2.32	7.26	2.71E-16	2.25E-14	RNA pseudouridine synthase superfamily protein
TGME49_307820	2.86	6.37	3.92E-16	3.23E-14	hypothetical protein
TGME49_270320	2.09	8.41	4.34E-16	3.49E-14	protein phosphatase 2C domain-containing protein
TGME49_252190	2.83	6.82	4.93E-16	3.93E-14	KRUF family protein
TGME49_246550	1.90	9.67	5.50E-16	4.34E-14	aspartyl protease ASP3
TGME49_289540	1.84	8.25	6.21E-16	4.85E-14	hypothetical protein
TGME49_268860	4.55	5.85	7.81E-16	6.04E-14	enolase 1
TGME49_217740	-6.72	6.50	1.05E-15	7.97E-14	3-ketoacyl-(acyl-carrier-protein) reductase
TGME49_293260	2.21	8.31	1.12E-15	8.38E-14	ATPase/histidine kinase/DNA gyrase B/HSP90 domain-containing protein
TGME49_227100	5.99	4.82	1.76E-15	1.31E-13	hypothetical protein
TGME49_209060	-9.60	6.36	1.93E-15	1.42E-13	thrombospondin type 1 domain-containing protein
TGME49_221320	-4.92	8.67	1.96E-15	1.43E-13	acetyl-CoA carboxylase ACC1
TGME49_313910	1.86	10.58	4.09E-15	2.95E-13	RNA recognition motif 2 protein
TGME49_252070	4.04	5.64	4.74E-15	3.39E-13	KRUF family protein
TGME49_203310	1.77	11.75	5.05E-15	3.55E-13	dense granule protein GRA7
TGME49_232670	1.76	8.87	5.66E-15	3.94E-13	hypothetical protein
TGME49_200360	2.02	9.94	6.55E-15	4.48E-13	hypothetical protein
TGME49_221590	1.92	8.97	6.85E-15	4.60E-13	dual specificity phosphatase, catalytic domain-containing protein
TGME49_277230	3.94	5.13	9.95E-15	6.63E-13	hypothetical protein
TGME49_212270	2.04	9.54	1.41E-14	9.30E-13	hypothetical protein
TGME49_254470	1.94	9.52	1.58E-14	1.04E-12	hypothetical protein
TGME49_232000	2.06	8.50	1.65E-14	1.07E-12	hypothetical protein
TGME49_296340	3.99	5.50	1.72E-14	1.11E-12	hypothetical protein
TGME49_225170	2.77	6.06	2.06E-14	1.32E-12	hypothetical protein
TGME49_297400	1.90	7.67	2.29E-14	1.45E-12	hypothetical protein
TGME49_277260	2.20	10.15	2.44E-14	1.53E-12	hypothetical protein
TGME49_304500	1.63	8.38	3.57E-14	2.23E-12	hypothetical protein
TGME49_215360	2.87	6.36	3.83E-14	2.37E-12	hypothetical protein
TGME49_259020	3.96	7.36	3.89E-14	2.39E-12	bradyzoite antigen BAG1
TGME49_252640	4.18	6.77	4.01E-14	2.44E-12	P-type ATPase PMA1
TGME49_269690	2.27	8.26	4.19E-14	2.53E-12	hypothetical protein
TGME49_253690	2.11	9.24	5.89E-14	3.47E-12	hypothetical protein
TGME49_247220	1.64	8.86	7.02E-14	4.11E-12	nudix -type motif 9 isoform a family protein
TGME49_247360	2.27	7.63	7.20E-14	4.18E-12	PAP2 superfamily protein
TGME49_262400	1.91	8.80	7.37E-14	4.24E-12	lipase
TGME49_254120	1.76	8.69	8.45E-14	4.80E-12	autophagy-related protein 8 atg8, putative
TGME49_280380	1.68	10.07	8.51E-14	4.80E-12	poly(ADP-ribose) glycohydrolase
TGME49_323400	3.25	8.61	1.56E-13	8.76E-12	cytochrome c oxidase subunit iii subfamily protein
TGME49_263550	2.42	7.01	1.69E-13	9.40E-12	39S ribosomal protein L47, mitochondrial precursor, putative
TGME49_287460	3.19	5.08	1.81E-13	1.00E-11	hypothetical protein
TGME49_246130	1.87	8.43	1.89E-13	1.03E-11	serpin (serine proteinase inhibitor) superfamily protein
TGME49_264870	2.11	7.67	1.90E-13	1.03E-11	Sodium:neurotransmitter symporter family protein
TGME49_209100	-9.29	6.06	2.04E-13	1.10E-11	PUB domain-containing protein

TGME49_240090	2.18	8.84	2.44E-13	1.30E-11	rhoptry kinase family protein ROP34, putative
TGME49_304740	1.68	8.68	2.70E-13	1.43E-11	rhoptry kinase family protein ROP35
TGME49_204520	3.03	5.24	2.79E-13	1.47E-11	hypothetical protein
TGME49_201780	1.83	10.52	3.35E-13	1.75E-11	microneme protein MIC2
TGME49_213050	2.09	8.68	3.77E-13	1.94E-11	hypothetical protein
TGME49_208020	3.45	5.27	4.19E-13	2.15E-11	AP2 domain transcription factor AP2Ib-1
TGME49_319640	1.76	8.29	4.31E-13	2.19E-11	hypothetical protein
TGME49_250710	1.75	11.55	4.37E-13	2.21E-11	microneme protein MIC10
TGME49_232410	1.61	9.84	4.43E-13	2.22E-11	PDI family protein
TGME49_253440	1.50	9.12	4.79E-13	2.39E-11	cell-cycle-associated protein kinase SRPK, putative
TGME49_214440	1.78	9.01	6.38E-13	3.16E-11	4'-phosphopantetheinyl transferase superfamily protein
TGME49_313760	1.52	8.19	6.42E-13	3.16E-11	hypothetical protein
TGME49_314430	1.61	9.84	7.51E-13	3.68E-11	serine/threonine specific protein phosphatase
TGME49_291940	-6.49	6.35	8.70E-13	4.23E-11	hypothetical protein
TGME49_260620	1.62	8.18	8.92E-13	4.31E-11	hypothetical protein
TGME49_297650	1.85	8.52	1.04E-12	5.01E-11	Ser/Thr phosphatase family protein
TGME49_225790	2.37	7.65	1.06E-12	5.04E-11	PDI family protein
TGME49_293252	5.73	5.29	1.16E-12	5.52E-11	hypothetical protein
TGME49_231430	-4.50	6.39	1.24E-12	5.83E-11	oligosaccharyl transferase stt3 protein, putative
TGME49_233380	1.51	10.22	1.42E-12	6.56E-11	hypothetical protein
TGME49_314700	2.10	7.49	1.57E-12	7.24E-11	hypothetical protein
TGME49_310130	-6.15	6.00	1.64E-12	7.49E-11	Spc97 / Spc98 family protein
TGME49_291040	2.94	7.28	1.75E-12	7.96E-11	lactate dehydrogenase LDH2
TGME49_315910	2.18	6.01	2.67E-12	1.19E-10	hypothetical protein
TGME49_310520	2.51	5.61	2.70E-12	1.20E-10	3'5'-cyclic nucleotide phosphodiesterase domain-containing protein
TGME49_209140	-5.74	6.69	2.87E-12	1.26E-10	anti-silencing protein, ASF1 family protein
TGME49_220240	1.89	9.13	2.93E-12	1.28E-10	hypothetical protein
TGME49_215895	2.52	6.46	3.10E-12	1.35E-10	AP2 domain-containing protein
TGME49_216680	-5.08	7.09	3.17E-12	1.37E-10	ankyrin repeat-containing protein
TGME49_244530	1.75	8.75	3.36E-12	1.44E-10	hypothetical protein
TGME49_254390	2.10	7.74	3.65E-12	1.56E-10	CRAL/TRIO domain-containing protein
TGME49_286460	4.54	4.91	4.15E-12	1.75E-10	hypothetical protein
TGME49_253560	2.68	6.24	5.04E-12	2.12E-10	hypothetical protein
TGME49_228160	2.08	7.19	6.36E-12	2.64E-10	acid phosphatase
TGME49_294980	2.96	6.15	7.10E-12	2.94E-10	hypothetical protein
TGME49_222020	-4.34	6.30	7.85E-12	3.19E-10	phosphoglycerate kinase PGKII
TGME49_225160	1.72	7.70	8.91E-12	3.59E-10	hypothetical protein
TGME49_276930	1.62	8.24	9.59E-12	3.82E-10	hypothetical protein
TGME49_209985	2.61	6.55	1.25E-11	4.95E-10	cAMP-dependent protein kinase
TGME49_229930	1.87	7.67	1.31E-11	5.17E-10	p25-alpha family protein
TGME49_247530	1.79	8.80	1.65E-11	6.39E-10	hypothetical protein
TGME49_218540	-4.61	6.89	1.81E-11	6.93E-10	peptidase S15, putative
TGME49_309580	-9.07	6.13	1.90E-11	7.25E-10	transporter, major facilitator family protein
TGME49_266310	-5.93	5.84	2.03E-11	7.67E-10	Alpha-1,3-mannosyl-glycoprotein 2-beta-N-acetylglucosaminyltransferase
TGME49_293770	1.91	8.29	2.50E-11	9.38E-10	chitinase-like protein CLP1
TGME49_275640	2.11	7.24	2.52E-11	9.42E-10	hypothetical protein
TGME49_239420	1.44	9.53	2.60E-11	9.66E-10	protein kinase
TGME49_254000	1.79	7.49	3.40E-11	1.26E-09	hypothetical protein

TGME49_305460	2.12	7.84	4.84E-11	1.77E-09	methionine aminopeptidase 2, putative
TGME49_250220	2.58	6.34	5.99E-11	2.19E-09	hypothetical protein
TGME49_323100	1.90	7.08	6.33E-11	2.29E-09	hypothetical protein
TGME49_263560	2.29	7.61	9.68E-11	3.48E-09	hypothetical protein
TGME49_266760	-6.67	6.61	1.06E-10	3.78E-09	isocitrate dehydrogenase
TGME49_210682	3.75	6.03	1.06E-10	3.78E-09	hypothetical protein
TGME49_312160	1.82	8.48	1.41E-10	4.99E-09	hypothetical protein
TGME49_255260	1.67	10.88	1.79E-10	6.28E-09	apical membrane antigen AMA1
TGME49_227390	-8.52	5.36	2.09E-10	7.32E-09	hypothetical protein
TGME49_286000	3.36	5.05	2.18E-10	7.60E-09	hypothetical protein
TGME49_316710	2.21	7.67	2.50E-10	8.64E-09	hypothetical protein
TGME49_208590	1.56	7.67	2.94E-10	1.00E-08	vacuolar ATP synthase subunit 54kD, putative
TGME49_288210	-8.82	5.82	3.15E-10	1.06E-08	PUL domain-containing protein
TGME49_306620	2.37	6.77	3.71E-10	1.24E-08	AP2 domain transcription factor AP2IX-9
TGME49_275420	-4.88	6.20	3.78E-10	1.26E-08	histone lysine-specific demethylase LSD1/BHC110/KDMA1A
TGME49_209900	-5.24	6.34	4.03E-10	1.34E-08	hypothetical protein
TGME49_253470	1.78	7.73	4.10E-10	1.35E-08	alveolin domain containing intermediate filament IMC13
TGME49_288650	1.57	11.92	4.23E-10	1.39E-08	dense granule protein GRA12
TGME49_300055	1.35	8.47	4.43E-10	1.45E-08	hypothetical protein
TGME49_217530	3.83	5.85	4.83E-10	1.57E-08	hypothetical protein
TGME49_255180	-5.14	6.02	5.00E-10	1.62E-08	ubiquitin carboxyl-terminal hydrolase
TGME49_226310	-4.66	7.37	5.48E-10	1.76E-08	zinc finger (CCCH type) motif-containing protein
TGME49_259880	1.86	6.83	6.21E-10	1.98E-08	hypothetical protein
TGME49_212250	1.77	7.91	6.57E-10	2.07E-08	XPG N-terminal domain-containing protein
TGME49_215550	-8.72	5.82	6.92E-10	2.18E-08	hypothetical protein
TGME49_213635	4.34	5.89	7.70E-10	2.41E-08	hypothetical protein
TGME49_288840	2.10	7.53	8.37E-10	2.60E-08	hypothetical protein
TGME49_222948	-8.96	5.77	8.46E-10	2.62E-08	hypothetical protein
TGME49_232750	1.73	7.12	9.19E-10	2.83E-08	23S rRNA (adenine(1618)-N(6))-methyltransferase, putative
TGME49_270260	3.39	4.99	9.31E-10	2.85E-08	hypothetical protein
TGME49_253750	1.53	8.32	9.34E-10	2.85E-08	PLU-1 family protein
TGME49_295420	1.78	8.00	9.83E-10	2.99E-08	hypothetical protein
TGME49_315680	-8.86	5.71	1.07E-09	3.22E-08	vacuolar protein sorting-associated protein vps4, putative
TGME49_321630	1.67	6.76	1.16E-09	3.50E-08	RNA recognition motif-containing protein
TGME49_284010	-8.39	5.23	1.27E-09	3.80E-08	5'-3' exonuclease, N-terminal resolvase family domain-containing protein
TGME49_269310	3.72	4.56	1.30E-09	3.89E-08	hypothetical protein
TGME49_222240	2.10	7.03	1.46E-09	4.29E-08	hypothetical protein
TGME49_217750	-9.01	5.70	1.52E-09	4.44E-08	hypothetical protein
TGME49_288000	2.12	7.50	1.63E-09	4.75E-08	hypothetical protein
TGME49_251540	1.18	9.20	1.76E-09	5.08E-08	dense granule protein GRA9
TGME49_209755	2.81	7.50	1.77E-09	5.08E-08	hypothetical protein
TGME49_219590	1.90	7.26	1.81E-09	5.18E-08	RuvB family 1 protein
TGME49_290190	1.81	6.70	2.02E-09	5.72E-08	hypothetical protein
TGME49_220440	-4.80	5.75	2.40E-09	6.77E-08	cyclin-dependent kinase regulatory subunit protein
TGME49_260540	1.60	7.84	2.78E-09	7.80E-08	alveolin domain containing intermediate filament IMC14

TGME49_220890	1.77	6.85	3.02E-09	8.39E-08	hypothetical protein
TGME49_232160	1.48	7.26	3.26E-09	9.01E-08	zinc finger, C3HC4 type (RING finger) domain-containing protein
TGME49_216500	-8.82	5.73	3.30E-09	9.07E-08	tRNA synthetase, putative
TGME49_261790	-9.05	5.96	3.44E-09	9.38E-08	hypothetical protein
TGME49_245490	1.68	9.25	4.14E-09	1.12E-07	microneme protein MIC8
TGME49_202580	2.18	6.60	4.53E-09	1.21E-07	ATPase, AAA family protein
TGME49_243470	3.58	4.96	4.81E-09	1.28E-07	hypothetical protein
TGME49_244450	3.01	5.42	5.95E-09	1.56E-07	protein phosphatase 2C domain-containing protein
TGME49_249230	2.26	6.44	6.20E-09	1.62E-07	hypothetical protein
TGME49_268010	-8.58	5.46	7.13E-09	1.82E-07	hypothetical protein
TGME49_299030	1.68	7.72	8.06E-09	2.05E-07	RNA recognition motif 2 protein
TGME49_265400	-5.11	6.52	8.57E-09	2.17E-07	hypothetical protein
TGME49_226520	1.96	6.25	8.80E-09	2.22E-07	hypothetical protein
TGME49_293280	2.44	5.66	8.97E-09	2.26E-07	cyclin protein
TGME49_312105	-8.91	5.62	9.61E-09	2.40E-07	hypothetical protein
TGME49_222050	1.45	7.23	9.63E-09	2.40E-07	hypothetical protein
TGME49_274150	-8.45	5.49	9.71E-09	2.41E-07	hypothetical protein
TGME49_254710	1.70	7.63	1.00E-08	2.48E-07	serine esterase (DUF676) protein
TGME49_215210	2.30	6.28	1.11E-08	2.73E-07	hypothetical protein
TGME49_221350	-4.63	5.57	1.12E-08	2.76E-07	Ctr copper transporter family protein
TGME49_221840	2.64	5.63	1.19E-08	2.89E-07	hypothetical protein
TGME49_268225	-9.66	6.16	1.71E-08	4.11E-07	hypothetical protein
TGME49_265460	2.08	6.31	1.91E-08	4.55E-07	hypothetical protein
TGME49_249540	1.55	7.42	1.94E-08	4.60E-07	hypothetical protein
TGME49_266920	1.73	9.58	2.01E-08	4.75E-07	3'5'-cyclic nucleotide phosphodiesterase domain-containing protein
TGME49_268230	-9.26	5.82	2.14E-08	5.02E-07	hypothetical protein
TGME49_226580	1.42	7.95	2.21E-08	5.15E-07	hypothetical protein
TGME49_234530	1.56	7.27	2.22E-08	5.16E-07	hypothetical protein
TGME49_248110	1.59	7.94	2.38E-08	5.49E-07	hypothetical protein
TGME49_202572	-8.42	5.40	2.51E-08	5.74E-07	ribophorin i protein
TGME49_220390	1.60	7.34	2.54E-08	5.79E-07	hypothetical protein
TGME49_315440	1.66	7.45	2.65E-08	5.99E-07	hypothetical protein
TGME49_223750	1.86	6.43	2.76E-08	6.20E-07	hypothetical protein
TGME49_244300	-8.49	5.33	2.80E-08	6.26E-07	hypothetical protein
TGME49_258710	-4.42	6.17	2.88E-08	6.41E-07	T-complex protein 10 C-terminus protein
TGME49_202770	1.16	9.06	4.04E-08	8.73E-07	RNA recognition motif-containing protein
TGME49_232035	-5.45	5.47	4.35E-08	9.37E-07	hypothetical protein
TGME49_254690	1.39	8.49	4.44E-08	9.54E-07	phospholipase/carboxylesterase
TGME49_280370	1.29	9.18	4.55E-08	9.75E-07	hypothetical protein
TGME49_242845	-8.83	5.62	4.59E-08	9.78E-07	hypothetical protein
TGME49_226990	2.15	5.68	4.59E-08	9.78E-07	hypothetical protein
TGME49_207100	-4.76	5.64	4.66E-08	9.89E-07	hypothetical protein
TGME49_219810	1.54	8.73	4.70E-08	9.97E-07	hypothetical protein
TGME49_280522	1.95	7.21	4.79E-08	1.01E-06	hypothetical protein
TGME49_315950	1.24	8.56	5.07E-08	1.06E-06	zinc finger, C3HC4 type (RING finger) domain-containing protein
TGME49_212940	-8.65	5.91	5.19E-08	1.08E-06	hypothetical protein
TGME49_203060	1.66	7.56	5.19E-08	1.08E-06	hypothetical protein
TGME49_269130	-8.01	5.02	5.20E-08	1.08E-06	hypothetical protein
TGME49_258030	-4.43	5.87	5.39E-08	1.12E-06	DNA polymerase

TGME49_224970	1.48	7.01	5.53E-08	1.14E-06	nucleolar protein,nop52 protein
TGME49_221180	1.55	8.39	5.87E-08	1.21E-06	hypothetical protein
TGME49_311230	1.38	9.71	5.90E-08	1.21E-06	hypothetical protein
TGME49_247300	1.29	8.26	6.94E-08	1.41E-06	hypothetical protein
TGME49_319590	-5.10	5.29	7.33E-08	1.48E-06	hypothetical protein
TGME49_240780	2.25	5.15	7.93E-08	1.60E-06	hypothetical protein
TGME49_208310	1.90	6.42	8.01E-08	1.61E-06	hypothetical protein
TGME49_254920	2.66	5.18	8.43E-08	1.69E-06	hypothetical protein
TGME49_221480	1.67	8.37	1.03E-07	2.04E-06	hypothetical protein
TGME49_204530	1.64	11.14	1.11E-07	2.21E-06	microneme protein MIC11
TGME49_213730	1.31	7.71	1.22E-07	2.40E-06	lanthionine synthetase C family protein
TGME49_293270	1.22	7.47	1.25E-07	2.45E-06	hypothetical protein
TGME49_304880	1.59	7.59	1.38E-07	2.66E-06	hypothetical protein
TGME49_252420	2.21	5.41	1.38E-07	2.66E-06	histone arginine methyltransferase PRMT3
TGME49_265790	1.27	8.72	1.49E-07	2.85E-06	hypothetical protein
TGME49_270865	1.35	7.55	1.50E-07	2.86E-06	adenylate cyclase, putative
TGME49_269950	1.18	9.55	1.50E-07	2.86E-06	hypothetical protein
TGME49_245630	1.58	7.26	1.60E-07	3.03E-06	hypothetical protein
TGME49_220330	-7.99	5.03	1.76E-07	3.30E-06	hypothetical protein
TGME49_215160	1.29	8.85	1.85E-07	3.46E-06	hypothetical protein
TGME49_231890	-8.61	5.55	1.94E-07	3.63E-06	beta-ketoacyl-acyl carrier protein synthase III, putative
TGME49_310210	1.97	6.30	1.95E-07	3.63E-06	hypothetical protein
TGME49_225680	-8.69	5.26	1.95E-07	3.63E-06	hypothetical protein
TGME49_242640	1.52	6.95	1.97E-07	3.66E-06	hypothetical protein
TGME49_255690	-8.33	5.25	1.98E-07	3.66E-06	2-C-methyl-D-erythritol 2,4-cyclodiphosphate synthase domain-containing protein
TGME49_253640	1.48	7.00	2.22E-07	4.07E-06	hypothetical protein
TGME49_237500	1.28	7.49	2.29E-07	4.19E-06	protein phosphatase 2C domain-containing protein
TGME49_308040	2.22	6.34	2.31E-07	4.21E-06	ZPR1 zinc finger domain-containing protein
TGME49_211460	1.37	7.65	2.43E-07	4.42E-06	hypothetical protein
TGME49_300048	1.43	7.13	2.47E-07	4.48E-06	hypothetical protein
TGME49_250730	1.91	7.22	2.56E-07	4.64E-06	60S ribosomal protein L7-B, putative
TGME49_306040	1.39	6.98	2.57E-07	4.65E-06	CHY zinc finger protein
TGME49_315100	2.95	5.66	2.74E-07	4.92E-06	hypothetical protein
TGME49_250950	2.85	5.69	2.77E-07	4.95E-06	KRUF family protein
TGME49_202520	1.93	6.95	2.78E-07	4.95E-06	hypothetical protein
TGME49_257080	1.53	7.01	2.90E-07	5.14E-06	3'5'-cyclic nucleotide phosphodiesterase domain-containing protein
TGME49_205180	1.15	8.28	2.93E-07	5.16E-06	RNA recognition motif-containing protein
TGME49_266450	-4.66	5.92	2.94E-07	5.16E-06	lysine decarboxylase family protein
TGME49_315280	-8.97	5.60	2.98E-07	5.21E-06	hypothetical protein
TGME49_330000	2.51	9.57	3.16E-07	5.49E-06	cytochrome b
TGME49_207065	-5.31	5.36	3.17E-07	5.49E-06	hypothetical protein
TGME49_209095	-4.32	6.51	3.57E-07	6.15E-06	hypothetical protein
TGME49_238510	-4.93	5.09	4.01E-07	6.78E-06	hypothetical protein
TGME49_232340	1.32	8.41	4.30E-07	7.23E-06	protein phosphatase 2C domain-containing protein
TGME49_250100	2.76	6.91	4.32E-07	7.25E-06	hypothetical protein
TGME49_228980	-8.09	4.76	4.35E-07	7.27E-06	hypothetical protein
TGME49_321450	1.47	7.51	4.40E-07	7.35E-06	Myb family DNA-binding domain-containing protein

TGME49_260410	-8.05	5.12	4.62E-07	7.67E-06	hypothetical protein
TGME49_225990	-9.17	5.87	4.62E-07	7.67E-06	acyl transferase domain-containing protein
TGME49_205490	1.22	8.17	4.97E-07	8.22E-06	integral membrane protein, putative
TGME49_236800	-7.92	4.83	5.05E-07	8.31E-06	hypothetical protein
TGME49_257350	1.54	8.63	5.29E-07	8.69E-06	eukaryotic translation initiation factor, putative
TGME49_223660	2.80	7.51	5.52E-07	9.01E-06	50S ribosomal protein L4, putative
TGME49_306890	2.18	5.76	5.73E-07	9.28E-06	hypothetical protein
TGME49_230180	1.89	8.98	6.09E-07	9.78E-06	hypothetical protein
TGME49_203280	1.88	5.91	6.12E-07	9.80E-06	hypothetical protein
TGME49_268680	1.39	7.20	6.16E-07	9.84E-06	hypothetical protein
TGME49_223390	1.09	8.03	6.28E-07	9.99E-06	activating signal cointegrator 1 complex subunit 3 family 1 ASCC3L1, putative
TGME49_206450	1.74	8.16	6.43E-07	1.02E-05	autophagy-related cysteine peptidase atg4, putative
TGME49_273970	-7.87	4.79	6.58E-07	1.04E-05	CorA family Mg2+ transporter protein
TGME49_277270	1.32	9.98	6.86E-07	1.08E-05	NTPase II
TGME49_263420	-6.06	6.00	7.40E-07	1.16E-05	ubiquitin-specific protease USP4
TGME49_239710	-8.73	5.40	7.48E-07	1.16E-05	phosphomannomutase
TGME49_286480	2.85	6.43	8.35E-07	1.29E-05	hypothetical protein
TGME49_278230	-8.10	5.09	8.62E-07	1.33E-05	prenyltransferase and squalene oxidase repeat-containing protein
TGME49_207930	1.58	6.29	9.06E-07	1.38E-05	phosphatidylethanolamine-binding protein
TGME49_219820	1.57	8.21	9.16E-07	1.39E-05	polyubiquitin UbC, putative
TGME49_258870	1.39	9.49	9.26E-07	1.41E-05	hypothetical protein
TGME49_243430	2.29	7.80	9.79E-07	1.47E-05	OTU family cysteine protease
TGME49_215910	1.01	9.02	1.08E-06	1.62E-05	hypothetical protein
TGME49_259200	-8.68	5.46	1.12E-06	1.66E-05	Na+/H+ exchanger NHE1
TGME49_225900	1.89	6.25	1.13E-06	1.68E-05	hypothetical protein
TGME49_251400	2.12	5.90	1.15E-06	1.70E-05	hypothetical protein
TGME49_200450	-8.12	5.23	1.18E-06	1.73E-05	hypothetical protein
TGME49_242810	-8.36	5.37	1.26E-06	1.85E-05	hypothetical protein
TGME49_302055	4.99	4.56	1.28E-06	1.87E-05	ribosomal protein RPS12
TGME49_259040	-4.59	5.51	1.37E-06	1.99E-05	hypothetical protein
TGME49_314500	1.57	8.23	1.47E-06	2.12E-05	subtilisin SUB2
TGME49_295430	4.21	5.60	1.47E-06	2.12E-05	hypothetical protein
TGME49_250870	1.45	7.38	1.48E-06	2.13E-05	DHHC zinc finger domain-containing protein
TGME49_287515	-7.54	4.51	1.50E-06	2.15E-05	hypothetical protein
TGME49_260430	2.73	5.07	1.52E-06	2.18E-05	hypothetical protein
TGME49_279350	2.72	6.32	1.61E-06	2.29E-05	hypothetical protein
TGME49_248850	-7.96	5.00	1.61E-06	2.29E-05	methionine aminopeptidase
TGME49_285700	-7.81	4.81	1.66E-06	2.36E-05	ubiquitin fusion degradation protein UFD1AP
TGME49_253950	1.95	5.93	1.67E-06	2.36E-05	protein fam50a, putative
TGME49_318440	1.36	7.85	1.68E-06	2.37E-05	helicase associated domain (ha2) protein
TGME49_200430	-8.05	4.95	1.93E-06	2.70E-05	cytidine and deoxycytidylate deaminase zinc-binding region domain-containing protein
TGME49_283702	-9.11	5.97	2.09E-06	2.90E-05	FATC domain-containing protein
TGME49_262080	-5.29	5.33	2.24E-06	3.06E-05	hypothetical protein
TGME49_293000	1.59	7.26	2.33E-06	3.17E-05	3'5'-cyclic nucleotide phosphodiesterase domain-containing protein
TGME49_261480	1.43	6.59	2.39E-06	3.25E-05	phosphatidyl serine synthase
TGME49_204360	2.15	6.27	2.44E-06	3.31E-05	subtilisin SUB4
TGME49_318170	1.49	6.30	2.44E-06	3.31E-05	hypothetical protein
TGME49_224620	1.86	6.17	2.52E-06	3.40E-05	hypothetical protein

TGME49_301170	-7.88	5.29	2.54E-06	3.42E-05	SAG-related sequence SRS19D
TGME49_225550	1.23	7.56	2.59E-06	3.48E-05	phosphatidylserine decarboxylase
TGME49_213460	1.67	7.26	2.60E-06	3.48E-05	hypothetical protein
TGME49_201710	2.12	5.86	2.66E-06	3.54E-05	WD domain, G-beta repeat-containing protein
TGME49_291890	1.25	11.15	2.69E-06	3.56E-05	microneme protein MIC1
TGME49_253700	1.07	8.39	2.70E-06	3.56E-05	transporter, major facilitator family protein
TGME49_252065	2.45	5.50	2.77E-06	3.65E-05	KRUF family protein
TGME49_278940	1.43	6.90	2.84E-06	3.72E-05	HECT-domain (ubiquitin-transferase) domain-containing protein
TGME49_209770	-7.57	4.59	2.89E-06	3.77E-05	helicase, putative
TGME49_285950	2.05	5.44	2.89E-06	3.77E-05	hypothetical protein
TGME49_217160	2.74	5.01	2.92E-06	3.80E-05	Der1 family protein
TGME49_286010	2.32	6.14	3.31E-06	4.27E-05	hypothetical protein
TGME49_286050	-5.13	5.45	3.44E-06	4.44E-05	hypothetical protein
TGME49_269010	1.22	9.39	3.47E-06	4.46E-05	AP2 domain transcription factor AP2VIII-7
TGME49_248990	1.08	8.50	3.57E-06	4.58E-05	hypothetical protein
TGME49_217410	3.40	4.69	3.58E-06	4.58E-05	hypothetical protein
TGME49_253180	1.69	7.97	3.67E-06	4.68E-05	hypothetical protein
TGME49_232360	1.34	6.77	3.94E-06	5.00E-05	exonuclease
TGME49_301400	1.23	9.68	4.44E-06	5.57E-05	hypothetical protein
TGME49_211330	-7.65	4.51	4.51E-06	5.65E-05	methionine aminopeptidase
TGME49_205265	2.09	6.76	4.52E-06	5.66E-05	transporter, cation channel family protein
TGME49_277490	1.20	7.52	4.78E-06	5.95E-05	hypothetical protein
TGME49_279100	1.47	9.31	4.86E-06	6.03E-05	hypothetical protein
TGME49_216140	1.52	7.81	4.87E-06	6.03E-05	tetratricopeptide repeat-containing protein
TGME49_263190	-7.51	4.66	4.90E-06	6.06E-05	adenylosuccinate lyase, putative
TGME49_230820	1.29	7.86	5.07E-06	6.25E-05	hypothetical protein
TGME49_239748	1.71	6.03	5.17E-06	6.36E-05	hypothetical protein
TGME49_293650	-7.56	4.86	5.21E-06	6.39E-05	hypothetical protein
TGME49_256840	-5.74	5.72	5.23E-06	6.40E-05	hypothetical protein
TGME49_305990	2.30	5.91	5.28E-06	6.45E-05	hypothetical protein
TGME49_251770	1.56	7.36	5.67E-06	6.91E-05	hypothetical protein
TGME49_305930	2.59	5.05	5.68E-06	6.92E-05	hypothetical protein
TGME49_264220	-8.48	5.18	5.70E-06	6.93E-05	hypothetical protein
TGME49_217790	-7.49	4.58	5.93E-06	7.18E-05	S1 RNA binding domain-containing protein
TGME49_310410	-7.42	4.48	5.95E-06	7.18E-05	WD domain, G-beta repeat-containing protein
TGME49_266480	3.02	4.64	6.03E-06	7.26E-05	hypothetical protein
TGME49_247030	1.13	9.66	6.13E-06	7.34E-05	hypothetical protein
TGME49_216810	1.48	7.97	7.32E-06	8.55E-05	5'-nucleotidase, C-terminal domain-containing protein
TGME49_312600	1.16	8.45	7.41E-06	8.60E-05	heat shock protein HSP21
TGME49_254150	2.89	5.42	7.46E-06	8.65E-05	hypothetical protein
TGME49_202730	-8.38	5.13	7.55E-06	8.72E-05	hypothetical protein
TGME49_319580	-8.92	5.58	7.58E-06	8.74E-05	hypothetical protein
TGME49_255980	1.70	6.15	7.84E-06	9.03E-05	hypothetical protein
TGME49_213300	-8.59	5.32	9.11E-06	0.0001037	hypothetical protein
TGME49_242870	-8.59	5.43	9.17E-06	0.0001041	histone lysine methyltransferase, SET, putative
TGME49_270720	-4.81	5.08	9.61E-06	0.0001086	hypothetical protein
TGME49_274130	-5.04	5.18	9.65E-06	0.0001089	TBC domain-containing protein
TGME49_293900	1.41	8.24	1.08E-05	0.0001212	sporozoite protein with an altered thrombospondin repeat SPATR
TGME49_211470	2.22	4.98	1.16E-05	0.0001294	Fcf2 pre-rRNA processing protein
TGME49_301300	-8.04	4.80	1.18E-05	0.0001312	hypothetical protein

TGME49_291600	1.14	7.49	1.20E-05	0.0001324	gamma interferon inducible lysosomal thiol reductase (GILT) protein
TGME49_272380	1.43	8.19	1.22E-05	0.0001348	hypothetical protein
TGME49_242670	-7.47	4.42	1.24E-05	0.0001364	hypothetical protein
TGME49_207040	-7.78	4.80	1.26E-05	0.0001385	hypothetical protein
TGME49_313340	1.38	7.45	1.28E-05	0.0001405	hypothetical protein
TGME49_236620	2.44	6.35	1.29E-05	0.0001409	protein kinase (incomplete catalytic triad)
TGME49_262590	-7.98	5.01	1.33E-05	0.0001449	hypothetical protein
TGME49_226060	1.62	7.34	1.33E-05	0.0001449	transmembrane amino acid transporter protein
TGME49_273050	1.55	6.29	1.40E-05	0.0001504	hypothetical protein
TGME49_308590	-5.00	5.97	1.41E-05	0.0001508	Mov34/MPN/PAD-1 family protein
TGME49_313418	-4.53	5.51	1.43E-05	0.000153	hypothetical protein
TGME49_200590	1.85	5.61	1.44E-05	0.0001532	Toxoplasma gondii family C protein
TGME49_237130	2.07	9.60	1.44E-05	0.0001535	cytochrome b, putative
TGME49_273280	-7.48	4.42	1.46E-05	0.000155	hypothetical protein
TGME49_269700	1.47	8.11	1.50E-05	0.0001581	NLI interacting factor family phosphatase
TGME49_312380	-8.23	4.91	1.50E-05	0.0001581	tetratricopeptide repeat-containing protein
TGME49_297900	1.21	7.44	1.54E-05	0.000162	hypothetical protein
TGME49_213480	2.47	5.98	1.57E-05	0.0001643	hypothetical protein
TGME49_237530	-8.47	5.19	1.63E-05	0.0001696	hypothetical protein
TGME49_200350	2.76	5.91	1.76E-05	0.0001822	subtilisin SUB3
TGME49_254380	1.75	5.84	1.79E-05	0.0001846	ribosomal protein L11, putative
TGME49_202610	1.16	7.70	1.79E-05	0.0001846	protein phosphatase 2C domain-containing protein
TGME49_240240	-7.67	4.43	1.85E-05	0.0001902	subtilisin SUB5
TGME49_255060	2.13	9.20	1.88E-05	0.0001928	cytochrome b(N-terminal)/b6/petB subfamily protein
TGME49_309600	1.03	8.90	1.89E-05	0.0001932	hypothetical protein
TGME49_271840	1.74	4.82	1.91E-05	0.0001953	hypothetical protein
TGME49_320620	-8.06	4.97	2.04E-05	0.0002061	queuine tRNA ribosyl transferase
TGME49_289620	1.53	8.81	2.06E-05	0.0002077	cathepsin CPC1
TGME49_226260	1.49	7.48	2.11E-05	0.0002118	hypothetical protein
TGME49_255190	1.03	8.51	2.11E-05	0.0002118	myosin C
TGME49_218850	-7.60	4.54	2.12E-05	0.0002119	ribosomal protein RPS9
TGME49_277560	-8.40	5.26	2.18E-05	0.0002173	hypothetical protein
TGME49_254555	1.57	6.66	2.29E-05	0.0002265	histone lysine acetyltransferase GCN5-A
TGME49_225510	1.81	7.68	2.31E-05	0.0002283	RAP domain-containing protein
TGME49_229020	-4.43	6.21	2.37E-05	0.0002331	cell-cycle-associated protein kinase CDK, putative
TGME49_221490	1.51	5.89	2.39E-05	0.0002356	cell cycle regulator protein
TGME49_253960	1.91	5.45	2.43E-05	0.0002387	oxidoreductase, short chain dehydrogenase/reductase family protein
TGME49_260520	1.42	7.53	2.50E-05	0.0002444	hypothetical protein
TGME49_288800	1.99	6.45	2.55E-05	0.0002477	endonuclease/exonuclease/phosphatase family protein
TGME49_222370	1.21	7.44	2.55E-05	0.000248	SAG-related sequence SRS13
TGME49_313160	1.41	6.19	2.66E-05	0.000257	hypothetical protein
TGME49_299980	1.35	7.50	2.82E-05	0.000271	hypothetical protein
TGME49_216335	2.42	5.09	2.89E-05	0.0002767	hypothetical protein
TGME49_288820	1.42	7.00	2.92E-05	0.0002787	hypothetical protein
TGME49_244540	1.33	6.72	3.00E-05	0.0002863	mitochondrial carrier superfamily protein
TGME49_310310	-8.00	4.89	3.01E-05	0.0002864	WD domain, G-beta repeat-containing protein
TGME49_227780	1.87	5.75	3.13E-05	0.000297	hypothetical protein

TGME49_234670	-7.39	4.64	3.15E-05	0.0002983	actin-like family protein
TGME49_299240	2.10	5.54	3.19E-05	0.0003012	hypothetical protein
TGME49_244510	-7.66	4.44	3.21E-05	0.000303	AP2 domain transcription factor AP2VI-3
TGME49_253570	1.62	5.91	3.27E-05	0.0003076	hypothetical protein
TGME49_225500	2.09	6.93	3.42E-05	0.0003194	hypothetical protein
TGME49_249698	-8.06	4.89	3.48E-05	0.000325	hypothetical protein
TGME49_239290	-8.14	4.88	3.54E-05	0.0003297	hypothetical protein
TGME49_240960	1.62	6.86	3.90E-05	0.0003564	AIG2 family protein
TGME49_275980	1.61	6.08	3.92E-05	0.0003581	coenzyme q (ubiquinone) biosynthesis protein coq4 protein
TGME49_233220	1.34	7.41	4.02E-05	0.0003655	hypothetical protein
TGME49_259650	-7.71	4.58	4.10E-05	0.0003716	hypothetical protein
TGME49_209820	-5.09	5.26	4.10E-05	0.0003716	syntaxin protein
TGME49_206700	-7.82	4.61	4.12E-05	0.0003722	hypothetical protein
TGME49_286740	3.34	5.35	4.12E-05	0.0003722	microneme-like protein
TGME49_263580	1.79	7.04	4.12E-05	0.0003722	bromodomain-containing protein
TGME49_273310	1.47	6.76	4.20E-05	0.0003779	hypothetical protein
TGME49_205190	1.48	6.13	4.35E-05	0.0003909	hypothetical protein
TGME49_204130	1.11	9.58	4.48E-05	0.0004012	perforin-like protein PLP1
TGME49_203050	1.89	6.32	4.57E-05	0.0004087	AP2 domain transcription factor AP2VIIa-6
TGME49_313680	1.54	7.21	4.61E-05	0.0004112	hypothetical protein
TGME49_201390	1.21	10.94	4.70E-05	0.0004176	hypothetical protein
TGME49_223480	-4.91	5.31	4.74E-05	0.0004205	sushi domain (scr repeat) domain-containing protein
TGME49_245428	2.44	5.32	4.91E-05	0.0004333	hypothetical protein
TGME49_265260	1.36	6.21	5.02E-05	0.0004418	hypothetical protein
TGME49_316220	2.34	4.59	5.15E-05	0.0004508	mediator complex subunit MED6
TGME49_263390	1.87	5.79	5.16E-05	0.0004508	hypothetical protein
TGME49_313710	-8.33	5.22	5.18E-05	0.0004524	hypothetical protein
TGME49_237550	2.55	6.05	5.26E-05	0.0004578	hypothetical protein
TGME49_285710	1.53	6.33	5.29E-05	0.0004599	hypothetical protein
TGME49_208550	1.25	6.92	5.32E-05	0.000461	hypothetical protein
TGME49_233830	-7.61	4.39	5.50E-05	0.0004747	hypothetical protein
TGME49_260150	-7.18	4.60	5.58E-05	0.0004797	tetratricopeptide repeat-containing protein
TGME49_231800	-8.11	4.94	5.77E-05	0.0004945	helicase, putative
TGME49_269420	1.56	7.56	5.94E-05	0.0005049	hypothetical protein
TGME49_262900	-7.84	4.67	6.06E-05	0.0005141	hypothetical protein
TGME49_207980	-4.42	4.52	6.06E-05	0.0005141	PIG-P protein
TGME49_253600	1.73	6.69	6.21E-05	0.000524	hypothetical protein
TGME49_249680	1.66	6.46	6.33E-05	0.0005314	RNA polymerase II associated Paf1 complex component PAF1
TGME49_212735	-7.90	4.76	6.33E-05	0.0005314	hypothetical protein
TGME49_217692	-7.66	4.48	6.66E-05	0.0005563	hypothetical protein
TGME49_253940	1.35	7.11	6.72E-05	0.0005601	CAM Kinase family, incomplete catalytic triad
TGME49_221190	2.14	5.56	6.74E-05	0.0005606	mrna cleavage factor family protein, putative
TGME49_257700	-7.24	4.46	6.74E-05	0.0005606	hypothetical protein
TGME49_218720	1.45	7.06	6.96E-05	0.0005756	calcium-dependent protein kinase CDPK6
TGME49_249840	-5.06	5.01	7.17E-05	0.0005917	dynein heavy chain 2, putative
TGME49_318610	2.42	4.83	7.39E-05	0.0006067	AP2 domain transcription factor AP2IV-3
TGME49_235360	2.14	4.74	7.50E-05	0.0006153	hypothetical protein
TGME49_267775	-7.80	4.74	7.85E-05	0.0006401	hypothetical protein
TGME49_233540	-7.86	4.91	7.96E-05	0.0006485	transporter, major facilitator family protein
TGME49_291590	-7.62	4.47	8.22E-05	0.000668	hypothetical protein

TGME49_286240	1.06	7.19	8.25E-05	0.0006691	kelch repeat protein, putative
TGME49_266930	1.65	5.61	8.26E-05	0.0006691	general transcription factor IIH polypeptide 3 GTF2H3
TGME49_236870	1.67	7.34	8.33E-05	0.0006743	hypothetical protein
TGME49_234230	1.02	7.55	8.46E-05	0.0006829	hypothetical protein
TGME49_225490	1.09	8.16	8.50E-05	0.000685	calcium-dependent protein kinase CDPK2
TGME49_280410	1.07	6.95	8.60E-05	0.0006924	3'5'-cyclic nucleotide phosphodiesterase domain-containing protein
TGME49_258100	-4.59	4.61	8.66E-05	0.0006958	TPR repeat region protein
TGME49_276210	1.11	7.27	8.76E-05	0.0007021	phosphoglycerate mutase family protein
TGME49_253100	1.01	7.65	8.82E-05	0.0007058	hypothetical protein
TGME49_234510	1.21	6.86	9.38E-05	0.0007466	ankyrin repeat-containing protein
TGME49_294690	1.18	7.36	9.59E-05	0.0007604	rhomboid protease ROM5
TGME49_285480	-8.25	5.22	9.79E-05	0.0007752	hypothetical protein
TGME49_227560	1.20	7.06	9.83E-05	0.0007773	IWS1 transcription factor, putative
TGME49_234980	1.44	6.46	0.0001014	0.0007999	hypothetical protein
TGME49_255330	-7.85	4.78	0.000102	0.0008037	hypothetical protein
TGME49_285170	-7.62	4.38	0.0001023	0.0008051	methyltransferase small, putative
TGME49_232760	1.40	6.54	0.0001029	0.0008086	protein phosphatase inhibitor IPP2
TGME49_232630	1.30	8.59	0.0001052	0.0008251	hypothetical protein
TGME49_216700	-7.82	4.74	0.0001072	0.000838	hypothetical protein
TGME49_314250	2.39	5.27	0.00011	0.0008563	bradyzoite rhoptry protein BRP1
TGME49_283550	1.35	7.29	0.0001143	0.0008861	hypothetical protein
TGME49_267580	1.35	7.15	0.0001154	0.000893	cyclin2 related protein
TGME49_203160	1.65	6.12	0.000116	0.0008972	hypothetical protein
TGME49_263490	1.53	5.63	0.0001176	0.0009073	ubiquitin conjugating enzyme E2, putative
TGME49_293780	1.32	7.63	0.0001223	0.0009374	hypothetical protein
TGME49_299250	1.58	5.86	0.0001274	0.0009701	hypothetical protein
TGME49_261075	-6.85	4.62	0.0001279	0.000973	hypothetical protein
TGME49_306510	2.48	4.93	0.0001283	0.0009751	hypothetical protein
TGME49_234460	1.30	6.92	0.0001318	0.0009958	hypothetical protein
TGME49_254770	1.13	6.69	0.0001338	0.0010093	Ser/Thr phosphatase family protein
TGME49_297647	2.86	4.83	0.0001367	0.0010251	hypothetical protein
TGME49_237425	-7.81	4.65	0.0001368	0.0010251	AP2 domain transcription factor AP2X-6
TGME49_207665	-7.87	4.57	0.0001447	0.001076	kinesin motor domain-containing protein
TGME49_205110	-7.19	4.59	0.000151	0.0011191	hypothetical protein
TGME49_246120	1.67	5.90	0.0001523	0.0011275	tetratricopeptide repeat-containing protein
TGME49_245432	1.31	7.40	0.0001524	0.0011275	hypothetical protein
TGME49_268560	1.44	5.68	0.0001527	0.0011288	XPG N-terminal domain-containing protein
TGME49_311450	2.53	4.54	0.0001548	0.0011411	zinc finger, c2h2 type domain-containing protein
TGME49_268000	-5.04	5.01	0.0001649	0.0012098	hypothetical protein
TGME49_305950	1.45	5.97	0.000166	0.0012155	tetratricopeptide repeat-containing protein
TGME49_265170	-7.58	4.53	0.0001675	0.0012239	hypothetical protein
TGME49_220510	1.70	6.25	0.0001763	0.0012802	hypothetical protein
TGME49_246990	1.66	6.02	0.0001774	0.0012841	hypothetical protein
TGME49_310560	-8.01	5.05	0.0001799	0.0012998	hypothetical protein
TGME49_297770	1.71	4.87	0.000181	0.0013069	hypothetical protein
TGME49_206540	1.44	7.28	0.000182	0.001311	hypothetical protein
TGME49_236140	-7.22	4.58	0.000194	0.0013871	hypothetical protein
TGME49_279330	1.71	5.84	0.0002	0.0014193	DEAD/DEAH box helicase family protein
TGME49_254730	1.58	5.56	0.0002009	0.0014238	POPLD (NUC188) domain-containing protein
TGME49_319360	3.27	4.58	0.0002013	0.0014254	SAG-related sequence SRS17A

TGME49_319720	-7.63	4.59	0.000211	0.0014858	hypothetical protein
TGME49_243510	-7.90	4.80	0.0002129	0.0014952	OTU family cysteine protease
TGME49_226850	1.87	6.27	0.0002228	0.0015515	TBC domain-containing protein
TGME49_298830	1.92	5.82	0.0002321	0.0016049	hypothetical protein
TGME49_271800	1.81	6.45	0.0002379	0.0016434	serine esterase (DUF676) protein
TGME49_264120	-4.57	4.61	0.0002383	0.0016452	Myb family DNA-binding domain-containing protein
TGME49_320680	2.33	4.71	0.0002414	0.0016575	AP2 domain transcription factor AP2IV-2
TGME49_207900	1.26	6.53	0.0002483	0.0017003	transcription initiation factor TFIIIB
TGME49_270580	1.61	5.35	0.0002519	0.0017231	HECT-domain (ubiquitin-transferase) domain-containing protein
TGME49_266740	1.97	5.45	0.0002587	0.0017634	RNA recognition motif-containing protein
TGME49_239810	1.52	6.36	0.0002634	0.0017923	hypothetical protein
TGME49_253490	1.36	7.30	0.0002664	0.0018081	hypothetical protein
TGME49_255245	-7.67	4.59	0.0002729	0.0018473	hypothetical protein
TGME49_230170	1.28	7.44	0.0002734	0.001848	hypothetical protein
TGME49_311280	-7.88	4.64	0.0002735	0.001848	hypothetical protein
TGME49_288860	2.12	7.04	0.000277	0.0018705	RuvB family 2 protein
TGME49_239910	1.52	6.52	0.0002868	0.0019315	cyclin-dependent kinase
TGME49_231215	-7.74	4.69	0.0002926	0.0019669	hypothetical protein
TGME49_292055	1.65	6.01	0.0002934	0.0019693	calcium dependent protein kinase CDPK8
TGME49_226100	-7.32	5.21	0.0002934	0.0019693	haloacid dehalogenase family hydrolase domain-containing protein
TGME49_310500	1.41	6.50	0.0002938	0.00197	hypothetical protein
TGME49_237020	1.45	6.35	0.0003042	0.002036	exonuclease
TGME49_205540	1.35	6.25	0.0003117	0.0020754	DEAD/DEAH box helicase domain-containing protein
TGME49_296121	1.29	7.39	0.0003138	0.0020874	hypothetical protein
TGME49_293480	1.64	7.25	0.0003297	0.002186	MoeA N-terminal region (domain I and II) domain-containing protein
TGME49_213060	1.35	6.07	0.0003324	0.002198	WD domain, G-beta repeat-containing protein
TGME49_254720	1.31	9.48	0.0003356	0.0022178	dense granule protein GRA8
TGME49_219230	1.39	6.18	0.0003382	0.0022328	AMP-binding enzyme domain-containing protein
TGME49_280700	1.29	7.39	0.0003547	0.0023257	arginine decarboxylase
TGME49_214580	-7.26	4.92	0.0003677	0.0024029	tetratricopeptide repeat-containing protein
TGME49_289140	1.28	6.57	0.0003793	0.002466	ribosomal protein l22/l43, putative
TGME49_219630	1.02	6.96	0.0004235	0.0027168	flavodoxin domain-containing protein
TGME49_278250	-4.60	4.88	0.0004356	0.0027833	hypothetical protein
TGME49_257370	1.68	5.81	0.000451	0.0028652	hypothetical protein
TGME49_295960	1.20	7.08	0.0004597	0.0029108	hypothetical protein
TGME49_278720	1.34	5.96	0.0004651	0.0029355	hypothetical protein
TGME49_224270	1.17	6.50	0.000479	0.0030157	hypothetical protein
TGME49_284590	1.95	4.79	0.0004797	0.0030176	hypothetical protein
TGME49_267670	2.48	5.25	0.0004851	0.0030468	hypothetical protein
TGME49_245980	2.03	6.49	0.0004929	0.0030899	hypothetical protein
TGME49_275310	1.30	6.47	0.0005006	0.0031312	hypothetical protein
TGME49_250090	1.35	6.21	0.0005286	0.003293	hypothetical protein
TGME49_210360	1.42	6.33	0.0005334	0.0033173	DEAD (Asp-Glu-Ala-Asp) box polypeptide 41 family protein
TGME49_226560	1.30	5.98	0.0005368	0.0033281	zinc finger (CCCH type) motif-containing protein
TGME49_262480	1.43	6.65	0.0005425	0.0033609	dynein light chain roadblock-type 2, putative

TGME49_229390	2.31	4.60	0.0005596	0.0034473	hypothetical protein
TGME49_268650	2.31	4.85	0.0005611	0.0034538	chaperone clpB protein, putative
TGME49_287430	2.69	4.52	0.0006203	0.0037758	hypothetical protein
TGME49_242270	1.05	6.42	0.000631	0.003835	hypothetical protein
TGME49_284598	-7.76	5.11	0.0006433	0.0039067	haloacid dehalogenase family hydrolase domain-containing protein
TGME49_312210	1.46	5.61	0.0006502	0.0039419	hypothetical protein
TGME49_268730	1.55	5.79	0.0006506	0.0039419	glutaredoxin-related protein
TGME49_224470	1.33	6.33	0.0006626	0.0039987	hypothetical protein
TGME49_225290	1.57	6.51	0.0006781	0.0040702	GDA1/CD39 (nucleoside phosphatase) family protein
TGME49_273660	1.35	7.35	0.0006844	0.0041046	AP2 domain transcription factor AP2VIII-3
TGME49_305120	1.08	7.25	0.0006856	0.0041086	transporter, solute:sodium symporter (SSS) family protein
TGME49_266890	1.37	7.32	0.0006963	0.0041571	hypothetical protein
TGME49_269200	1.72	5.82	0.0007085	0.0042132	crooked neck family 1 protein isoform 2, putative
TGME49_309990	2.15	6.26	0.0007109	0.0042181	hypothetical protein
TGME49_220880	2.09	6.09	0.0007208	0.0042666	hypothetical protein
TGME49_271600	2.44	4.53	0.0007244	0.0042801	hypothetical protein
TGME49_248450	1.14	6.38	0.0007251	0.0042801	zinc finger, C3HC4 type (RING finger) domain-containing protein
TGME49_267370	1.09	7.19	0.0007264	0.0042838	kinesin motor domain-containing protein
TGME49_220870	2.20	5.94	0.0007404	0.0043528	hypothetical protein
TGME49_216800	2.07	5.04	0.0007473	0.0043827	flagellar/basal body protein
TGME49_258010	1.27	5.82	0.0007506	0.0043963	calcium signaling protein kinase RAD53, putative
TGME49_202568	1.57	5.12	0.0007655	0.0044632	hypothetical protein
TGME49_257560	2.08	4.98	0.000769	0.0044802	WD domain, G-beta repeat-containing protein
TGME49_247340	2.52	5.26	0.0007743	0.0045009	hypothetical protein
TGME49_258458	1.76	6.62	0.0007771	0.00451	hypothetical protein
TGME49_236790	-4.71	4.76	0.000781	0.0045291	hypothetical protein
TGME49_270800	1.45	6.28	0.0007815	0.0045291	GAF domain-containing protein
TGME49_220360	1.16	6.41	0.0008119	0.0046782	FAD binding domain-containing protein
TGME49_315310	1.22	6.45	0.0008121	0.0046782	hypothetical protein
TGME49_254220	1.88	5.43	0.000826	0.0047511	hypothetical protein
TGME49_280375	2.39	6.55	0.0008464	0.0048472	hypothetical protein
TGME49_202620	1.53	8.40	0.000849	0.0048582	hypothetical protein
TGME49_216770	1.52	6.79	0.0008628	0.0049191	hypothetical protein
TGME49_218910	2.44	5.59	0.0008691	0.0049515	hypothetical protein
TGME49_220470	1.08	7.20	0.0008958	0.0050758	hypothetical protein
TGME49_300020	1.17	6.17	0.0008962	0.0050758	ATP-dependent metallopeptidase HflB subfamily protein
TGME49_278030	1.03	7.66	0.0008997	0.0050884	hypothetical protein
TGME49_206460	1.47	6.32	0.0009063	0.0051145	hypothetical protein
TGME49_255250	-4.38	5.30	0.0009313	0.0052304	tRNA (cytosine(34)-C(5))-methyltransferase, putative
TGME49_251840	1.61	6.41	0.0009318	0.0052304	hypothetical protein
TGME49_306630	1.78	4.95	0.0009338	0.0052352	tRNA methyltransferase complex GCD14 subunit protein
TGME49_246610	1.41	5.44	0.0009473	0.0052952	hypothetical protein
TGME49_282140	1.32	6.74	0.0009524	0.0053203	cwf21 protein
TGME49_300052	1.91	5.47	0.0009744	0.0054273	hypothetical protein

TGME49_234350	2.21	5.05	0.0010008	0.0055463	hypothetical protein
TGME49_281440	1.10	7.93	0.0010033	0.0055533	hypothetical protein
TGME49_222070	1.30	5.57	0.0010049	0.0055533	elongation factor Tu GTP binding domain-containing protein
TGME49_233080	1.43	6.01	0.0010267	0.0056617	G-patch domain-containing protein
TGME49_202025	2.35	4.49	0.0010454	0.0057463	hypothetical protein
TGME49_269075	1.55	5.28	0.00105	0.005757	hypothetical protein
TGME49_232590	1.35	6.37	0.0010641	0.0058304	glutamate-cysteine ligase, catalytic subunit domain-containing protein
TGME49_288670	1.87	5.27	0.0010771	0.005885	hypothetical protein
TGME49_275750	1.05	7.30	0.001086	0.0059294	small nuclear ribonucleoprotein E, putative
TGME49_300990	1.63	5.19	0.0010941	0.0059567	Toxoplasma gondii family C protein
TGME49_257755	-7.01	4.73	0.0011529	0.006207	hypothetical protein
TGME49_320640	1.36	6.35	0.0011582	0.0062224	peptidylprolyl isomerase domain-containing protein
TGME49_248660	1.27	5.66	0.0011606	0.006229	hypothetical protein
TGME49_236000	1.45	5.40	0.001168	0.0062558	ferredoxin, putative
TGME49_208540	1.44	5.77	0.0011868	0.0063276	DEAD/DEAH box helicase domain-containing protein
TGME49_299000	1.23	6.72	0.0011955	0.0063699	hypothetical protein
TGME49_246010	2.01	5.36	0.001205	0.006416	hypothetical protein
TGME49_224160	-4.81	4.96	0.0012116	0.0064467	hypothetical protein
TGME49_289170	-6.97	4.56	0.0012358	0.0065581	adenylate and guanylate cyclase catalytic domain-containing protein
TGME49_221220	2.00	5.26	0.0012437	0.0065903	hypothetical protein
TGME49_273500	-4.79	4.94	0.0012635	0.0066677	O-linked N-acetylglucosamine transferase
TGME49_313495	-4.77	4.87	0.0013052	0.0068716	hypothetical protein
TGME49_217610	2.23	5.48	0.0013137	0.006895	hypothetical protein
TGME49_217640	2.05	6.33	0.0013167	0.0069011	hypothetical protein
TGME49_293580	1.03	7.41	0.0013315	0.0069517	prefoldin subunit protein
TGME49_269705	2.45	4.71	0.0013342	0.0069588	hypothetical protein
TGME49_281910	2.08	6.17	0.0013526	0.0070272	hypothetical protein
TGME49_245730	1.83	5.77	0.001356	0.0070356	phosphatidylinositol-4-phosphate 5-Kinase
TGME49_305090	1.14	6.45	0.0013838	0.0071605	kinase binding protein cgi-121 protein
TGME49_232640	1.51	5.52	0.0013884	0.0071798	RNA 2'-phosphotransferase, Tpt1/KptA family protein
TGME49_211695	1.87	4.79	0.0014198	0.007314	hypothetical protein
TGME49_217010	1.28	6.35	0.0014219	0.0073187	hypothetical protein
TGME49_258720	1.45	6.16	0.001457	0.0074597	Ubiquitin family protein, putative
TGME49_262060	1.09	6.26	0.0014599	0.0074672	hypothetical protein
TGME49_268320	1.47	6.18	0.0015344	0.0077942	hypothetical protein
TGME49_306520	1.18	7.07	0.0015424	0.0078175	tRNA pseudouridine synthase B, putative
TGME49_252510	2.06	6.03	0.001549	0.0078422	hypothetical protein
TGME49_262970	1.30	7.43	0.0015598	0.0078797	hypothetical protein
TGME49_219250	1.11	7.27	0.0015604	0.0078797	acetyltransferase, GNAT family protein
TGME49_313060	1.11	6.71	0.0015857	0.0079863	eukaryotic translation initiation factor 2B, putative
TGME49_314070	1.15	6.34	0.0016108	0.0080919	hypothetical protein
TGME49_255270	1.99	5.09	0.0016658	0.0083381	hypothetical protein
TGME49_206500	1.65	5.25	0.0017224	0.0085633	hypothetical protein
TGME49_271320	1.92	4.81	0.0017953	0.0088574	hypothetical protein
TGME49_212090	1.04	6.73	0.0018001	0.0088584	hypothetical protein
TGME49_294750	1.67	5.16	0.0018479	0.0090646	hypothetical protein

TGME49_228780	2.19	5.54	0.0018801	0.0091996	Toxoplasma gondii family C protein
TGME49_202780	1.14	7.63	0.0019136	0.0093222	rhoptry kinase family protein ROP25
TGME49_222245	1.30	6.79	0.0019395	0.0094302	hypothetical protein
TGME49_277150	-4.49	5.20	0.0019806	0.009594	XPG N-terminal domain-containing protein
TGME49_232955	-5.63	10.84	0.0020171	0.0097282	hypothetical protein
TGME49_217510	1.08	8.80	0.0020573	0.0098961	hypothetical protein
TGME49_313360	1.22	6.29	0.0020583	0.0098961	hypothetical protein
TGME49_234490	1.13	6.17	0.0021327	0.0101776	kelch repeat-containing protein
TGME49_202510	-4.48	5.80	0.0022035	0.010451	multi-pass transmembrane protein
TGME49_219240	1.70	5.69	0.0022309	0.0105679	Peptidyl-tRNA hydrolase PTH2 domain-containing protein
TGME49_229500	1.44	6.14	0.0022366	0.0105823	hypothetical protein
TGME49_259830	2.00	6.41	0.0023268	0.0109286	diacylglycerol kinase catalytic domain-containing protein
TGME49_266940	1.89	6.11	0.002385	0.0111275	DHHC zinc finger domain-containing protein
TGME49_202450	1.98	5.35	0.0025222	0.0116459	hypothetical protein
TGME49_300320	-4.56	4.45	0.002549	0.0117368	dimethyladenosine transferase
TGME49_215440	1.26	6.52	0.0026367	0.0120902	WWE domain-containing protein
TGME49_313110	1.21	5.91	0.002663	0.0121835	hypothetical protein
TGME49_264650	-4.36	4.67	0.0026859	0.012265	phosphoacetylglucosamine mutase
TGME49_204320	1.43	5.13	0.0027171	0.0123856	hypothetical protein
TGME49_211480	1.96	5.52	0.002738	0.0124588	GTP-binding protein engA, putative
TGME49_264030	1.02	6.90	0.0028328	0.0127996	aminotransferase, putative
TGME49_208570	1.07	7.57	0.002848	0.0128461	ubiquitin conjugating enzyme E2, putative
TGME49_231020	1.12	5.82	0.0028915	0.0130263	hypothetical protein
TGME49_215540	-6.53	4.49	0.0029155	0.0130741	hypothetical protein
TGME49_295950	1.53	6.39	0.0029623	0.0132534	KRUF family protein
TGME49_262680	1.70	4.41	0.00305	0.013575	hypothetical protein
TGME49_224990	1.10	5.97	0.0031031	0.0137795	hypothetical protein
TGME49_299780	1.20	8.77	0.0031105	0.0137906	hypothetical protein
TGME49_275755	1.65	5.48	0.003111	0.0137906	hypothetical protein
TGME49_248540	-6.69	4.43	0.0031971	0.0141239	hypothetical protein
TGME49_239795	1.67	6.48	0.0032041	0.0141423	hypothetical protein
TGME49_271935	1.78	6.58	0.0033199	0.0145663	hypothetical protein
TGME49_209680	1.27	5.58	0.0033451	0.0146606	hypothetical protein
TGME49_318880	1.99	4.84	0.0033684	0.0147457	hypothetical protein
TGME49_246160	2.07	4.55	0.003472	0.0151564	hypothetical protein
TGME49_241175	1.49	4.72	0.0035521	0.0154409	hypothetical protein
TGME49_316360	1.18	6.01	0.0035787	0.0155169	hypothetical protein
TGME49_209490	1.16	6.16	0.0037049	0.0159658	hypothetical protein
TGME49_270805	2.37	4.88	0.0037259	0.0160472	hypothetical protein
TGME49_213020	1.82	5.47	0.0038899	0.0166422	hypothetical protein
TGME49_260220	1.24	7.04	0.0039053	0.0166988	folate/biopterin transporter subfamily protein
TGME49_295020	1.62	4.83	0.0040456	0.0172325	Sterol-sensing domain of SREBP cleavage-activation domain-containing protein
TGME49_248830	1.03	6.65	0.0040634	0.0172985	phosphoinositide phospholipase PIPLC
TGME49_258850	1.86	5.34	0.0040876	0.0173676	hypothetical protein
TGME49_262600	1.54	4.89	0.0041972	0.0177509	hypothetical protein
TGME49_264730	1.12	5.74	0.0042381	0.0179043	hypothetical protein
TGME49_260470	1.02	7.48	0.0043474	0.018296	heat shock protein DNAJ pfj4, putative
TGME49_222940	2.36	5.20	0.0046175	0.0192336	hypothetical protein
TGME49_221640	1.26	6.76	0.0047685	0.0197353	hypothetical protein
TGME49_224090	1.20	6.19	0.0049045	0.0202007	enoyl-CoA hydratase/isomerase family protein

TGME49_316200	1.35	6.57	0.0049399	0.0203103	phosphoglycerate mutase family protein
TGME49_215380	1.43	5.48	0.004961	0.0203791	hypothetical protein
TGME49_282170	1.24	7.12	0.0049955	0.0204882	hypothetical protein
TGME49_263220	1.24	5.56	0.0050312	0.0205911	rhoptry kinase family protein ROP21
TGME49_254090	1.04	6.60	0.0050392	0.0206128	hypothetical protein
TGME49_256090	2.08	4.88	0.0051382	0.020929	glycerophosphodiester phosphodiesterase family protein
TGME49_309410	1.73	5.76	0.0051489	0.0209504	AP2 domain transcription factor AP2XI-1
TGME49_219660	2.01	4.74	0.00519	0.0210846	hypothetical protein
TGME49_251450	1.07	6.46	0.0052728	0.021387	hypothetical protein
TGME49_310390	1.13	7.18	0.0053346	0.0215814	hypothetical protein
TGME49_301350	1.65	5.02	0.0054512	0.0219725	SNARE associated protein
TGME49_205200	1.69	6.62	0.0055026	0.0221335	hypothetical protein
TGME49_254950	1.22	5.55	0.0055886	0.0223862	RNA cap guanine-N2 methyltransferase
TGME49_206340	1.06	8.06	0.0056366	0.0225014	hypothetical protein
TGME49_286430	1.30	5.67	0.0056583	0.0225719	hypothetical protein
TGME49_243680	2.16	4.51	0.0056799	0.022623	dihydrodipicolinate reductase
TGME49_250955	1.83	6.73	0.0057673	0.0229003	KRUF family protein
TGME49_251920	1.77	5.21	0.0059116	0.0233206	hypothetical protein
TGME49_277070	1.40	5.66	0.0059636	0.0234868	SWI2/SNF2-containing protein
TGME49_280470	1.48	6.49	0.0059876	0.0235692	AP2 domain transcription factor AP2VIIa-1
TGME49_254890	1.78	5.36	0.0061005	0.0239406	hypothetical protein
TGME49_238970	-5.69	4.46	0.0061053	0.0239472	hypothetical protein
TGME49_254280	2.05	5.22	0.0061742	0.0241397	DNA-directed RNA polymerase III RPC9
TGME49_224890	1.10	7.05	0.006255	0.0244042	hypothetical protein
TGME49_218610	1.22	5.72	0.006279	0.0244358	ATPase (DUF699) protein
TGME49_214960	1.21	6.75	0.00632	0.024528	AP2 domain transcription factor AP2X-8
TGME49_228150	1.65	7.14	0.006329	0.0245505	hypothetical protein
TGME49_242730	1.32	6.07	0.0064318	0.0249242	guanylate kinase family protein
TGME49_233770	2.02	6.44	0.0064581	0.0249707	calcium-translocating P-type ATPase, PMCA-type protein
TGME49_215750	1.71	4.88	0.0064608	0.0249707	cytidine and deoxycytidylate deaminase zinc-binding region domain-containing protein
TGME49_255350	1.52	5.11	0.0064631	0.0249707	ATPase, putative
TGME49_320700	1.20	5.53	0.0065432	0.0252298	AP2 domain transcription factor AP2IV-1
TGME49_225230	1.32	6.39	0.0066615	0.0255712	hypothetical protein
TGME49_278080	1.46	5.97	0.0069742	0.0265257	Toxoplasma gondii family A protein
TGME49_249410	1.81	6.16	0.0070756	0.0268448	hypothetical protein
TGME49_211610	1.65	5.95	0.0070935	0.0268822	hypothetical protein
TGME49_308965	2.09	4.65	0.0071947	0.027199	hypothetical protein
TGME49_270595	1.30	7.13	0.0073229	0.0275492	UBA/TS-N domain-containing protein
TGME49_220250	1.57	6.85	0.0073531	0.0276222	Nucleotide-sensitive chloride conductance regulator (ICln) protein
TGME49_218740	1.93	5.59	0.0074568	0.0279036	membrane protein, putative
TGME49_277090	1.17	6.97	0.0074609	0.0279051	carrier superfamily protein
TGME49_273640	1.29	5.96	0.0077969	0.0290495	hypothetical protein
TGME49_225470	1.11	6.33	0.007813	0.0290825	peptide methionine sulfoxide reductase
TGME49_288370	1.26	5.61	0.0078428	0.0291504	hypothetical protein
TGME49_268840	1.09	5.80	0.0080056	0.0296274	N6-adenosine-methyltransferase, putative
TGME49_270890	1.34	6.23	0.0081428	0.0300343	hypothetical protein
TGME49_200385	1.25	8.25	0.0081973	0.0301921	Myb family DNA-binding domain-containing protein
TGME49_253510	1.14	6.34	0.0085099	0.0312247	transporter/permease protein

TGME49_289550	1.02	6.24	0.0087685	0.0320365	WD domain, G-beta repeat-containing protein
TGME49_297730	1.14	5.99	0.0089858	0.0326913	transcription elongation factor 1, putative
TGME49_232960	1.07	7.02	0.0089926	0.0326922	oxidoreductase, 2OG-Fe(II) oxygenase family protein
TGME49_250940	1.01	6.67	0.0090802	0.0329726	hypothetical protein
TGME49_266880	1.76	5.44	0.0092208	0.033452	dihydrouridine synthase, putative
TGME49_217560	1.68	6.33	0.0092334	0.0334821	DNA-directed RNA polymerase II RPB10
TGME49_226640	2.02	5.09	0.0095497	0.034403	zinc binding protein, putative
TGME49_319090	1.64	4.44	0.0096092	0.0346012	IgA-specific serine endopeptidase
TGME49_304490	1.70	6.34	0.0096709	0.0347208	hypothetical protein
TGME49_261590	1.15	5.50	0.0096739	0.0347208	ankyrin, putative
TGME49_237015	1.03	7.54	0.0097246	0.0348705	hypothetical protein
TGME49_277870	1.58	6.38	0.0098135	0.0351241	hypothetical protein
TGME49_252880	1.02	7.59	0.0098238	0.0351285	hypothetical protein
TGME49_250500	1.99	4.89	0.009862	0.0352073	hypothetical protein
TGME49_222970	1.37	6.07	0.0098725	0.035221	inositol(myo)-1(or 4)-monophosphatase 2, putative
TGME49_202310	1.01	6.02	0.0100534	0.0357182	O-sialoglycoprotein endopeptidase
TGME49_250670	1.76	6.34	0.0100743	0.0357762	hypothetical protein
TGME49_305080	1.40	5.45	0.0101734	0.0360722	hypothetical protein
TGME49_217430	1.15	5.77	0.0102379	0.0362405	protease inhibitor PI1
TGME49_225410	1.29	5.30	0.0103324	0.036508	histone H3 centromeric CENH3
TGME49_227860	1.15	5.38	0.0104556	0.0368927	hypothetical protein
TGME49_261220	1.34	5.51	0.0106235	0.0374	transcription elongation factor SPT4
TGME49_270300	1.06	6.87	0.0106487	0.0374545	hypothetical protein
TGME49_270610	1.31	5.36	0.0108755	0.0380789	hypothetical protein
TGME49_252870	1.25	4.81	0.0114827	0.0397372	hypothetical protein
TGME49_319620	1.30	5.96	0.0117027	0.0404131	hypothetical protein
TGME49_301180	-5.93	4.45	0.0117273	0.0404389	SAG-related sequence SRS19F
TGME49_268980	1.48	8.06	0.0117812	0.0405703	hypothetical protein
TGME49_218950	1.79	5.54	0.0118847	0.0408689	hypothetical protein
TGME49_267970	1.26	5.47	0.0118891	0.0408689	DEAD/DEAH box helicase domain-containing protein
TGME49_285470	1.54	5.82	0.0119801	0.041054	patched family protein
TGME49_240300	1.30	5.42	0.0120055	0.0410915	zinc finger domain, LSD1 subclass domain-containing protein
TGME49_289250	1.70	5.80	0.012204	0.0416001	cyclophilin, putative
TGME49_220208	1.33	5.09	0.012628	0.0427572	hypothetical protein
TGME49_257020	1.02	6.00	0.012728	0.0430072	hypothetical protein
TGME49_230150	1.11	5.95	0.0127475	0.0430377	ChAPs (Chs5p-Arf1p-binding proteins) protein
TGME49_294990	1.13	6.50	0.0131865	0.0442661	hypothetical protein
TGME49_236670	1.20	5.77	0.0133237	0.0446299	hypothetical protein
TGME49_309190	1.06	6.91	0.013401	0.0447917	hypothetical protein
TGME49_312280	1.13	7.06	0.0134487	0.0448901	pre-mRNA-splicing factor ATP-dependent RNA helicase, putative
TGME49_208722	1.76	5.42	0.0134643	0.0448901	hypothetical protein
TGME49_256880	1.13	5.82	0.0140952	0.046648	protein kinase domain-containing protein
TGME49_222850	1.23	7.66	0.0145545	0.0477592	stress responsive a/b barrel domain-containing protein
TGME49_219150	1.76	5.80	0.0148013	0.0484396	zinc finger, zz type domain-containing protein
TGME49_216030	1.67	4.95	0.0149234	0.048692	hypothetical protein
TGME49_222180	1.89	4.90	0.0149785	0.0488398	hypothetical protein
TGME49_227430	1.03	6.42	0.0150796	0.0491278	transmembrane amino acid transporter protein

TGME49_221500	1.65	5.74	0.0151474	0.0492967	dual specificity phosphatase, catalytic domain-containing protein
TGME49_220260	1.31	5.31	0.0154095	0.0499241	hypothetical protein

Supplementary Table S3B: Differencial T. gondii gene expressin analysis of human HFF cells infected with T. gondii EGS strain for 18 hours compared to the average T. gondii gene expression of human MM6 and neuronal stem cells infected with either GT1, ME49 or VEG strains of T. gondii for 18 hours.

gene ID	logFC	logCPM	PValue	FDR	Product name
TGME49_457990	17.05	11.10	2.48E-155	1.91E-151	28S ribosomal RNA
TGME49_462910	12.08	7.86	9.78E-138	3.78E-134	large subunit ribosomal RNA
TGME49_280570	5.30	8.90	3.57E-69	9.20E-66	SAG-related sequence SRS35A
TGME49_205250	3.92	9.76	7.12E-63	1.38E-59	rhoptry protein ROP18
TGME49_200010	9.86	6.04	1.83E-51	2.82E-48	hypothetical protein
TGME49_214080	4.01	9.15	1.40E-44	1.81E-41	toxofilin
TGME49_207210	4.66	7.68	4.55E-39	5.03E-36	hypothetical protein
TGME49_301250	3.87	14.08	4.14E-30	4.00E-27	hypothetical protein
TGME49_252640	6.04	6.77	1.12E-29	9.62E-27	P-type ATPase PMA1
TGME49_293790	4.41	6.58	4.09E-26	3.16E-23	hypothetical protein
TGME49_291040	4.24	7.28	4.73E-26	3.32E-23	lactate dehydrogenase LDH2
TGME49_221840	4.17	5.63	9.11E-22	5.87E-19	hypothetical protein
TGME49_237130	4.00	9.60	3.68E-21	2.19E-18	cytochrome b, putative
TGME49_206550	4.04	6.24	4.05E-21	2.24E-18	hypothetical protein
TGME49_321480	5.25	5.82	2.01E-20	1.03E-17	SAG-related sequence SRS12B
TGME49_202020	3.09	8.16	2.25E-20	1.09E-17	DnAK-TPR
TGME49_209755	4.10	7.50	7.15E-20	3.07E-17	hypothetical protein
TGME49_259020	4.75	7.36	7.15E-20	3.07E-17	bradyzoite antigen BAG1
TGME49_278080	4.04	5.97	3.85E-19	1.57E-16	Toxoplasma gondii family A protein
TGME49_330000	3.95	9.57	1.01E-16	3.90E-14	cytochrome b
TGME49_243470	4.58	4.96	3.70E-14	1.27E-11	hypothetical protein
TGME49_312160	2.15	8.48	3.79E-14	1.27E-11	hypothetical protein
TGME49_209985	2.97	6.55	8.58E-14	2.76E-11	cAMP-dependent protein kinase
TGME49_310970	-4.58	7.27	1.30E-13	4.03E-11	hypothetical protein
TGME49_239530	-6.09	7.04	5.67E-13	1.69E-10	alanine-glyoxylate aminotransferase
TGME49_207160	3.72	7.01	7.98E-13	2.29E-10	SAG-related sequence SRS49D
TGME49_244700	-4.99	7.43	8.56E-13	2.36E-10	NAD(+)/NADH kinase domain-containing protein
TGME49_217740	-9.88	6.50	4.37E-12	1.13E-09	3-ketoacyl-(acyl-carrier-protein) reductase
TGME49_323400	3.56	8.61	9.15E-12	2.21E-09	cytochrome c oxidase subunit iii subfamily protein
TGME49_216140	2.20	7.81	1.61E-11	3.77E-09	tetratricopeptide repeat-containing protein
TGME49_253330	2.37	8.53	7.79E-11	1.77E-08	Rhoptry kinase family protein, truncated (incomplete catalytic triad)
TGME49_249260	-9.86	6.77	9.93E-11	2.16E-08	cell-cycle-associated protein kinase CDK, putative
TGME49_255060	3.16	9.20	1.01E-10	2.16E-08	cytochrome b(N-terminal)/b6/petB subfamily protein
TGME49_252070	3.55	5.64	4.88E-10	9.67E-08	KRUF family protein
TGME49_305460	2.07	7.84	8.23E-10	1.56E-07	methionine aminopeptidase 2, putative
TGME49_313270	-4.80	6.83	8.65E-10	1.56E-07	hypothetical protein
TGME49_315100	3.51	5.66	1.41E-09	2.42E-07	hypothetical protein
TGME49_260430	3.55	5.07	1.64E-09	2.76E-07	hypothetical protein
TGME49_225290	2.64	6.51	2.05E-09	3.38E-07	GDA1/CD39 (nucleoside phosphatase) family protein
TGME49_208730	1.74	8.82	5.87E-09	9.26E-07	microneme protein, putative
TGME49_290970	2.59	7.40	7.39E-09	1.12E-06	8-amino-7-oxononanoate synthase
TGME49_254460	1.68	7.76	8.00E-09	1.19E-06	hypothetical protein
TGME49_312660	-9.00	6.00	9.49E-09	1.38E-06	hypothetical protein
TGME49_209970	-9.27	6.12	1.09E-08	1.56E-06	Spc97 / Spc98 family protein
TGME49_253100	1.49	7.65	1.40E-08	1.93E-06	hypothetical protein
TGME49_243210	-10.07	6.87	2.36E-08	3.06E-06	DUF862 domain-containing protein
TGME49_202025	3.76	4.49	2.37E-08	3.06E-06	hypothetical protein
TGME49_319730	-8.83	5.87	4.34E-08	5.02E-06	YOU2 family C2C2 zinc finger protein
TGME49_310530	-9.05	5.82	4.52E-08	5.14E-06	SNF2 family N-terminal domain-containing protein

TGME49_299990	1.99	5.96	5.79E-08	6.48E-06	archease family protein
TGME49_208740	1.28	9.94	7.09E-08	7.72E-06	microneme protein, putative
TGME49_275870	2.37	6.07	7.43E-08	7.98E-06	tubulin/FtsZ family, GTPase domain-containing protein
TGME49_291810	-4.40	6.35	8.89E-08	9.29E-06	thioredoxin domain-containing protein
TGME49_269438	-9.21	6.22	9.27E-08	9.56E-06	hypothetical protein
TGME49_290700	1.34	8.51	1.06E-07	1.08E-05	hypothetical protein
TGME49_241305	-8.92	6.00	1.07E-07	1.08E-05	hypothetical protein
TGME49_312430	-8.70	5.72	1.29E-07	1.26E-05	hypothetical protein
TGME49_209070	-9.94	6.81	1.86E-07	1.77E-05	hypothetical protein
TGME49_228180	-8.92	5.90	2.09E-07	1.92E-05	cytochrome C oxidase assembly factor COX15, putative
TGME49_286928	-8.89	5.85	2.11E-07	1.92E-05	hypothetical protein
TGME49_318575	-8.67	5.62	2.37E-07	2.13E-05	hypothetical protein
TGME49_293780	1.77	7.63	2.63E-07	2.33E-05	hypothetical protein
TGME49_264670	-8.85	5.75	3.39E-07	2.91E-05	DNA polymerase family B protein
TGME49_229200	-8.72	5.49	3.85E-07	3.27E-05	hypothetical protein
TGME49_268860	3.33	5.85	3.89E-07	3.27E-05	enolase 1
TGME49_268840	-8.64	5.80	4.56E-07	3.75E-05	N6-adenosine-methyltransferase, putative
TGME49_273840	-8.66	5.51	4.88E-07	3.97E-05	brix domain-containing protein
TGME49_298990	1.79	6.52	5.07E-07	4.08E-05	ferredoxin NADP+ oxidoreductase FNR
TGME49_234980	-4.80	6.46	5.13E-07	4.09E-05	hypothetical protein
TGME49_222948	-8.96	5.77	5.72E-07	4.47E-05	hypothetical protein
TGME49_216335	2.91	5.09	6.80E-07	5.21E-05	hypothetical protein
TGME49_312140	-4.37	8.23	7.28E-07	5.52E-05	hypothetical protein
TGME49_232410	1.17	9.84	7.43E-07	5.57E-05	PDI family protein
TGME49_224600	-8.73	5.68	7.90E-07	5.87E-05	GTP binding protein
TGME49_216930	1.78	7.26	8.10E-07	5.92E-05	cholinephosphate cytidylyltransferase
TGME49_314250	3.09	5.27	8.21E-07	5.93E-05	bradyzoite rhoptry protein BRP1
TGME49_220370	-5.05	6.18	8.62E-07	6.12E-05	hypothetical protein
TGME49_272000	-8.53	5.44	1.01E-06	7.00E-05	hypothetical protein
TGME49_271320	2.97	4.81	1.10E-06	7.50E-05	hypothetical protein
TGME49_268225	-9.66	6.16	1.30E-06	8.62E-05	hypothetical protein
TGME49_232035	-8.62	5.47	1.30E-06	8.62E-05	hypothetical protein
TGME49_213060	-4.64	6.07	1.37E-06	8.89E-05	WD domain, G-beta repeat-containing protein
TGME49_232550	1.62	7.77	1.58E-06	9.98E-05	hypothetical protein
TGME49_252420	2.16	5.41	1.78E-06	0.0001113	histone arginine methyltransferase PRMT3
TGME49_256880	-8.61	5.82	1.87E-06	0.0001157	protein kinase domain-containing protein
TGME49_245580	-8.59	5.61	1.93E-06	0.0001184	hypothetical protein
TGME49_312105	-8.91	5.62	2.34E-06	0.0001396	hypothetical protein
TGME49_268230	-9.26	5.82	2.35E-06	0.0001396	hypothetical protein
TGME49_278500	-8.69	5.44	2.43E-06	0.000141	ribosomal RNA large subunit methyltransferase J protein
TGME49_309950	-8.44	5.44	2.53E-06	0.0001457	NLE (NUC135) domain-containing protein
TGME49_254920	2.59	5.18	2.95E-06	0.0001653	hypothetical protein
TGME49_221900	-8.31	5.36	2.97E-06	0.0001654	hypothetical protein
TGME49_311100	1.01	9.34	3.24E-06	0.0001788	zinc finger (CCCH type) motif-containing protein
TGME49_249560	-8.51	5.36	3.28E-06	0.0001795	DNA-directed RNA polymerase alpha chain rpoA
TGME49_264660	1.56	9.56	3.62E-06	0.0001958	SAG-related sequence SRS44
TGME49_238020	-8.56	5.54	3.90E-06	0.0002067	hypothetical protein
TGME49_240970	-8.94	5.78	3.96E-06	0.000208	hypothetical protein
TGME49_275860	1.13	9.69	4.30E-06	0.0002248	hypothetical protein
TGME49_301430	-8.67	5.56	4.48E-06	0.0002308	septum formation protein maf, putative
TGME49_214340	-4.76	6.07	4.78E-06	0.0002448	hypothetical protein
TGME49_230000	-8.74	5.70	5.09E-06	0.0002591	hypothetical protein
TGME49_246060	-8.27	5.10	5.39E-06	0.0002706	DNA-dependent RNA polymerase
TGME49_263710	-5.14	6.15	5.63E-06	0.0002808	acyl-CoA:cholesterol acyltransferase alpha ACAT1-alpha
TGME49_261740	1.22	11.35	6.17E-06	0.0003039	hypothetical protein
TGME49_242845	-8.83	5.62	6.32E-06	0.0003088	hypothetical protein
TGME49_305050	1.88	7.40	6.35E-06	0.0003088	calmodulin, putative
TGME49_246590	-8.29	5.43	6.60E-06	0.0003185	hypothetical protein
TGME49_235460	-8.56	5.57	6.74E-06	0.0003214	hypothetical protein

TGME49_266950	-9.24	6.38	6.89E-06	0.0003267	protein kinase, putative
TGME49_222370	1.35	7.44	7.54E-06	0.0003532	SAG-related sequence SRS13
TGME49_212940	-8.65	5.91	8.71E-06	0.0003984	hypothetical protein
TGME49_219290	-8.61	5.50	8.95E-06	0.0004071	F-actin-capping protein subunit beta, putative
TGME49_203760	-8.87	5.77	9.15E-06	0.0004135	hypothetical protein
TGME49_299030	1.42	7.72	9.70E-06	0.0004361	RNA recognition motif 2 protein
TGME49_246200	-8.46	5.41	9.93E-06	0.0004437	zinc finger (CCCH type) motif-containing protein
TGME49_254390	1.48	7.74	1.02E-05	0.0004509	CRAL/TRIO domain-containing protein
TGME49_277260	1.36	10.15	1.15E-05	0.0005031	hypothetical protein
TGME49_313960	-8.02	5.12	1.20E-05	0.0005226	ribosomal protein L19 protein
TGME49_249480	1.62	6.66	1.22E-05	0.0005261	tetratricopeptide repeat-containing protein
TGME49_254080	1.09	8.07	1.27E-05	0.0005438	metal cation transporter, ZIP family protein
TGME49_230110	-8.60	5.37	1.37E-05	0.000574	hypothetical protein
TGME49_221220	2.68	5.26	1.42E-05	0.000586	hypothetical protein
TGME49_255350	2.40	5.11	1.52E-05	0.0006176	ATPase, putative
TGME49_315280	-8.97	5.60	1.60E-05	0.0006352	hypothetical protein
TGME49_268770	-8.36	5.20	1.65E-05	0.0006458	dual specificity phosphatase, catalytic domain-containing protein
TGME49_240660	-8.25	5.27	1.68E-05	0.0006556	hypothetical protein
TGME49_225680	-8.69	5.26	1.73E-05	0.0006725	hypothetical protein
TGME49_288475	3.36	6.01	1.80E-05	0.0006967	hypothetical protein
TGME49_244160	-8.35	5.43	1.82E-05	0.0006997	transcription initiation factor TFIID complex subunit TAF12
TGME49_250220	1.94	6.34	1.86E-05	0.0007127	hypothetical protein
TGME49_253690	1.30	9.24	1.90E-05	0.0007172	hypothetical protein
TGME49_271145	-8.12	5.32	1.94E-05	0.0007285	hypothetical protein
TGME49_212250	1.34	7.91	1.95E-05	0.0007285	XPG N-terminal domain-containing protein
TGME49_319940	-8.14	5.23	1.98E-05	0.0007356	hypothetical protein
TGME49_208370	1.14	9.24	2.07E-05	0.0007582	myosin heavy chain, putative
TGME49_267970	-8.22	5.47	2.12E-05	0.0007617	DEAD/DEAH box helicase domain-containing protein
TGME49_243298	-8.28	5.17	2.12E-05	0.0007617	ICE family protease (caspase) p20 domain-containing protein
TGME49_297320	-9.10	5.78	2.14E-05	0.0007623	hypothetical protein
TGME49_268170	-8.39	5.04	2.32E-05	0.0008125	hypothetical protein
TGME49_221585	-8.87	5.69	2.32E-05	0.0008125	hypothetical protein
TGME49_313440	1.91	8.63	2.42E-05	0.0008388	hypothetical protein
TGME49_270620	-8.01	5.35	2.51E-05	0.0008656	DEAD/DEAH box helicase domain-containing protein
TGME49_278270	-8.09	5.09	2.75E-05	0.0009382	nucleolar protein, structural component of H/ACA snoRNPs, putative
TGME49_254630	1.35	7.14	3.02E-05	0.0010164	CMGC kinase
TGME49_298060	-8.32	5.13	3.11E-05	0.0010425	Toxoplasma gondii family C protein
TGME49_263550	1.57	7.01	3.36E-05	0.0010978	39S ribosomal protein L47, mitochondrial precursor, putative
TGME49_253510	1.81	6.34	3.37E-05	0.0010978	transporter/permease protein
TGME49_297510	-8.78	5.54	3.50E-05	0.0011286	hypothetical protein
TGME49_239710	-8.73	5.40	3.58E-05	0.0011478	phosphomannomutase
TGME49_285850	-8.30	5.37	3.61E-05	0.0011492	peptidyl-prolyl cis-trans isomerase, FKBP-type domain-containing protein
TGME49_313480	-8.44	5.49	3.63E-05	0.00115	hypothetical protein
TGME49_245980	-8.63	6.49	3.78E-05	0.0011922	hypothetical protein
TGME49_257290	-8.13	5.45	3.97E-05	0.0012425	RNA 2'-phosphotransferase, Tpt1/KptA family protein
TGME49_313200	-8.34	5.22	4.04E-05	0.0012601	leucine rich repeat-containing protein
TGME49_296340	2.61	5.50	4.09E-05	0.0012686	hypothetical protein
TGME49_248660	1.70	5.66	4.23E-05	0.0013077	hypothetical protein
TGME49_252440	2.33	4.64	4.43E-05	0.0013474	peptidase c13 family protein
TGME49_254365	1.24	7.90	4.53E-05	0.0013695	phosphatidate cytidylyltransferase
TGME49_238190	1.55	6.14	4.69E-05	0.0014062	DNA-directed RNA polymerase II RPB3
TGME49_267340	1.84	5.39	4.92E-05	0.0014641	hypothetical protein
TGME49_275640	1.46	7.24	5.25E-05	0.0015491	hypothetical protein
TGME49_262050	1.14	9.45	5.55E-05	0.0016157	rhoptry kinase family protein ROP39

TGME49_204490	-7.99	5.15	5.56E-05	0.0016157	hypothetical protein
TGME49_232510	-8.65	5.31	5.75E-05	0.0016588	hypothetical protein
TGME49_228980	-8.09	4.76	5.83E-05	0.0016767	hypothetical protein
TGME49_283585	-8.28	5.09	5.91E-05	0.0016867	hypothetical protein
TGME49_247610	-8.03	5.13	5.98E-05	0.0016995	small nuclear ribonucleoprotein E, putative
TGME49_240480	-8.00	5.10	6.12E-05	0.0017198	cpw-wpc domain-containing protein
TGME49_244200	1.00	7.80	6.18E-05	0.0017249	2-oxoglutarate dehydrogenase e1 component, mitochondrial precursor, putative
TGME49_227100	3.51	4.82	6.20E-05	0.0017252	hypothetical protein
TGME49_258940	-7.94	5.16	6.69E-05	0.0018535	acylphosphatase family protein
TGME49_317705	-5.13	5.96	6.81E-05	0.0018742	enoyl-CoA hydratase/isomerase family protein
TGME49_280580	1.89	6.00	7.12E-05	0.0019461	SAG-related sequence SRS35B
TGME49_293500	-8.17	5.24	7.37E-05	0.0019841	hypothetical protein
TGME49_227115	-8.32	5.16	7.47E-05	0.0020055	hypothetical protein
TGME49_316090	-7.84	5.01	7.91E-05	0.0021095	RNAse P Rpr2/Rpp21 subunit domain-containing protein
TGME49_253790	1.16	7.52	8.14E-05	0.0021623	zinc finger (CCCH type) motif-containing protein
TGME49_258780	-8.93	5.89	8.17E-05	0.002163	OTU family cysteine protease
TGME49_275780	-7.96	5.09	8.30E-05	0.0021906	hypothetical protein
TGME49_220230	-4.57	5.80	8.44E-05	0.0022116	leucine rich repeat-containing protein
TGME49_255215	-8.40	5.80	8.54E-05	0.002231	hypothetical protein
TGME49_275750	1.29	7.30	9.56E-05	0.0024304	small nuclear ribonucleoprotein E, putative
TGME49_205010	1.08	7.54	9.87E-05	0.0024941	U2 small nuclear ribonucleoprotein family protein, putative
TGME49_254690	1.06	8.49	0.0001114	0.0027754	phospholipase/carboxylesterase
TGME49_279540	-8.17	4.83	0.0001161	0.0028492	hypothetical protein
TGME49_244645	-7.70	5.13	0.0001205	0.0029289	hypothetical protein
TGME49_250900	-7.59	4.48	0.0001245	0.0030174	hypothetical protein
TGME49_301310	-4.46	5.78	0.0001277	0.0030763	hypothetical protein
TGME49_248670	1.02	8.72	0.0001322	0.0031712	V-type H(+)-translocating pyrophosphatase VP1
TGME49_223150	-8.54	5.16	0.0001325	0.0031712	START domain-containing protein
TGME49_230080	-8.52	5.47	0.0001347	0.0032141	DEAD/DEAH box helicase domain-containing protein
TGME49_226700	-7.84	5.05	0.0001373	0.0032667	nuclease, putative
TGME49_204520	2.00	5.24	0.0001395	0.0032683	hypothetical protein
TGME49_235478	-4.89	5.82	0.00014	0.0032683	pantothenate kinase
TGME49_248440	-8.41	5.74	0.0001401	0.0032683	hypothetical protein
TGME49_255930	-8.12	5.09	0.0001401	0.0032683	hypothetical protein
TGME49_287040	1.24	7.28	0.0001405	0.0032683	hypothetical protein
TGME49_226280	-7.80	5.02	0.0001412	0.0032683	ribosomal protein L28, putative
TGME49_221370	-8.41	5.18	0.0001521	0.0034376	hypothetical protein
TGME49_310290	-7.56	4.69	0.0001558	0.0035016	regulator of chromosome condensation (RCC1) repeat-containing protein
TGME49_264752	-7.90	5.01	0.0001629	0.0036514	HEAT repeat-containing protein
TGME49_293260	1.17	8.31	0.000168	0.0037182	ATPase/histidine kinase/DNA gyrase B/HSP90 domain-containing protein
TGME49_232160	1.08	7.26	0.0001683	0.0037182	zinc finger, C3HC4 type (RING finger) domain-containing protein
TGME49_309390	2.01	4.86	0.0001694	0.0037322	hypothetical protein
TGME49_314790	-5.50	6.49	0.0001749	0.0038205	small nuclear ribonucleoprotein G, putative
TGME49_222940	3.09	5.20	0.0001766	0.0038341	hypothetical protein
TGME49_309080	-7.56	4.78	0.0001793	0.0038838	hypothetical protein
TGME49_239087	-8.18	5.02	0.000183	0.0039363	hypothetical protein
TGME49_301170	-7.88	5.29	0.0001838	0.0039363	SAG-related sequence SRS19D
TGME49_269430	-8.29	5.18	0.000189	0.0040361	polyprenyl synthetase superfamily protein
TGME49_289000	-8.54	5.37	0.0001903	0.0040529	hypothetical protein
TGME49_217450	-8.05	5.18	0.0001927	0.0040703	general transcription factor IIH polypeptide 5 GTF2H5
TGME49_277700	-7.85	5.09	0.0001995	0.0041695	ribosomal protein S14 precursor, putative
TGME49_281950	-8.54	5.46	0.0002202	0.0045596	membrane protein, putative
TGME49_254770	1.21	6.69	0.0002224	0.004585	Ser/Thr phosphatase family protein
TGME49_287515	-7.54	4.51	0.000229	0.0046833	hypothetical protein
TGME49_209930	-8.42	5.34	0.0002402	0.0048603	hypothetical protein

TGME49_316470	-8.45	5.70	0.0002452	0.0049238	hypothetical protein
TGME49_323010	-7.77	4.68	0.0002467	0.0049316	hypothetical protein
TGME49_289010	-8.16	5.19	0.0002473	0.0049316	RNA recognition motif-containing protein
TGME49_258100	-7.80	4.61	0.0002475	0.0049316	TPR repeat region protein
TGME49_243760	-8.49	5.41	0.0002614	0.0051252	hypothetical protein
TGME49_297730	1.62	5.99	0.0002632	0.0051392	transcription elongation factor 1, putative
TGME49_263215	-7.63	4.62	0.0002664	0.0051816	hypothetical protein
TGME49_306338	-8.39	5.67	0.0002668	0.0051816	dynein gamma chain, flagellar outer arm, putative
TGME49_254930	1.13	7.23	0.0002743	0.005275	hypothetical protein
TGME49_293860	-4.63	5.67	0.0002743	0.005275	hypothetical protein
TGME49_275755	2.12	5.48	0.0002757	0.0052894	hypothetical protein
TGME49_270580	-7.35	5.35	0.000277	0.0053001	HECT-domain (ubiquitin-transferase) domain-containing protein
TGME49_287460	2.09	5.08	0.0002784	0.0053135	hypothetical protein
TGME49_236990	1.82	5.87	0.000279	0.0053135	beta-ketoacyl synthase, N-terminal domain-containing protein
TGME49_220150	-8.31	5.06	0.0002818	0.0053535	50S ribosomal protein L16, putative
TGME49_236130	-8.58	5.40	0.0002985	0.0055698	signal recognition particle (SRP9) domain-containing protein
TGME49_284170	-7.74	4.84	0.000299	0.0055698	DHHC zinc finger domain-containing protein
TGME49_321170	1.91	5.58	0.0002997	0.0055698	Toxoplasma gondii family C protein
TGME49_218790	-7.73	4.86	0.0003021	0.0055816	elongation factor G C-terminus domain-containing protein
TGME49_320700	1.63	5.53	0.0003025	0.0055816	AP2 domain transcription factor AP2IV-1
TGME49_312380	-8.23	4.91	0.0003286	0.0059913	tetratricopeptide repeat-containing protein
TGME49_310200	-8.08	5.12	0.0003294	0.0059918	hypothetical protein
TGME49_290240	-8.41	5.39	0.0003307	0.0060015	hypothetical protein
TGME49_288290	-8.19	5.36	0.0003358	0.0060369	hypothetical protein
TGME49_244680	-7.56	4.87	0.000339	0.0060546	hypothetical protein
TGME49_293252	3.40	5.29	0.0003391	0.0060546	hypothetical protein
TGME49_301300	-8.04	4.80	0.0003423	0.0060968	hypothetical protein
TGME49_242118	-8.30	5.24	0.0003454	0.006109	myosin-light-chain kinase
TGME49_224200	-7.93	4.82	0.0003462	0.006109	tRNA pseudouridine synthase
TGME49_220480	-7.77	4.68	0.0003469	0.006109	hypothetical protein
TGME49_229290	-7.60	4.45	0.0003503	0.0061405	kelch repeat-containing protein
TGME49_295320	-8.26	5.21	0.0003559	0.006225	embryo sac development arrest EDA7, putative
TGME49_277560	-8.40	5.26	0.0003596	0.0062473	hypothetical protein
TGME49_243265	-7.82	5.00	0.0003621	0.0062483	protamine P1 protein
TGME49_229720	-8.10	5.07	0.0003706	0.0063535	hypothetical protein
TGME49_220530	-8.48	5.32	0.0003768	0.0064445	AP2 domain transcription factor AP2V-1
TGME49_226090	-7.82	4.52	0.0003831	0.0065061	DEAD/DEAH box helicase domain-containing protein
TGME49_262590	-7.98	5.01	0.0003858	0.0065061	hypothetical protein
TGME49_270050	1.95	5.36	0.0003898	0.0065477	hypothetical protein
TGME49_270960	-8.11	5.14	0.0003991	0.0066191	hypothetical protein
TGME49_269830	2.93	4.40	0.0004071	0.0066961	RAP domain-containing protein
TGME49_288845	-8.19	5.04	0.0004106	0.006725	hypothetical protein
TGME49_257160	1.16	7.33	0.0004421	0.0071402	hypothetical protein
TGME49_243615	-7.78	5.09	0.0004421	0.0071402	hypothetical protein
TGME49_264120	-7.79	4.61	0.0004437	0.0071402	Myb family DNA-binding domain-containing protein
TGME49_297460	-8.44	5.17	0.0004442	0.0071402	hypothetical protein
TGME49_312210	1.63	5.61	0.0004548	0.0072948	hypothetical protein
TGME49_254200	1.84	4.96	0.0004618	0.0073459	anticodon binding domain-containing protein
TGME49_248800	-7.78	5.02	0.0004702	0.0074637	hypothetical protein
TGME49_205050	-8.15	5.33	0.0004763	0.0075307	hypothetical protein
TGME49_254135	-7.46	4.78	0.000484	0.0076208	hypothetical protein
TGME49_311790	1.99	5.15	0.0004865	0.007629	hypothetical protein
TGME49_310410	-7.42	4.48	0.0004878	0.007634	WD domain, G-beta repeat-containing protein
TGME49_233838	-7.66	4.41	0.0005113	0.0079212	PET112 family, C terminal region domain-containing protein
TGME49_227270	-8.24	5.34	0.0005143	0.0079514	hypothetical protein
TGME49_254840	-7.96	5.49	0.0005243	0.0080259	tetratricopeptide repeat-containing protein

TGME49_260310	1.66	6.15	0.0005277	0.0080622	ATP-binding cassette transporter ABC.B1
TGME49_288240	-7.71	4.87	0.0005362	0.0081596	hypothetical protein
TGME49_242110	-7.95	5.04	0.0005415	0.0081926	rhoptry kinase family protein ROP38
TGME49_285510	1.03	7.63	0.0005514	0.0082786	hypothetical protein
TGME49_266740	2.00	5.45	0.0006052	0.0089888	RNA recognition motif-containing protein
TGME49_204140	-7.83	4.63	0.0006058	0.0089888	PHD-finger domain-containing protein
TGME49_269940	-7.77	4.83	0.0006204	0.0091226	zinc finger motif, C2HC5-type protein
TGME49_300130	2.35	4.80	0.0006207	0.0091226	apical membrane antigen 1 domain-containing protein
TGME49_201180	-8.19	5.26	0.0006271	0.0091473	hypothetical protein
TGME49_226670	-7.51	4.64	0.000629	0.0091582	hypothetical protein
TGME49_316480	-7.54	4.93	0.0006314	0.0091752	XRN 5'-3' exonuclease N-terminus protein
TGME49_242670	-7.47	4.42	0.0006337	0.0091914	hypothetical protein
TGME49_305120	1.15	7.25	0.0006353	0.0091983	transporter, solute:sodium symporter (SSS) family protein
TGME49_314530	-7.84	4.86	0.0006401	0.0092503	RPAP1 family, C-terminal protein
TGME49_300360	-8.03	5.02	0.0006428	0.009256	ADP/ATP translocase
TGME49_215400	-8.32	5.17	0.0006429	0.009256	RNA recognition motif-containing protein
TGME49_210310	-7.90	4.82	0.0006537	0.0093767	hypothetical protein
TGME49_305990	1.86	5.91	0.00066	0.0094494	hypothetical protein
TGME49_238070	-8.06	4.94	0.0006641	0.0094904	glutaredoxin domain-containing protein
TGME49_241175	1.89	4.72	0.0006705	0.0095462	hypothetical protein
TGME49_209460	-7.93	4.78	0.0006918	0.0098276	hypothetical protein
TGME49_218850	-7.60	4.54	0.0007134	0.0099828	ribosomal protein RPS9
TGME49_202240	-7.95	4.79	0.0007241	0.0100259	RAP domain-containing protein
TGME49_257560	-7.70	4.98	0.0007247	0.0100259	WD domain, G-beta repeat-containing protein
TGME49_301280	-7.38	4.50	0.0007249	0.0100259	hypothetical protein
TGME49_207180	-7.87	4.94	0.0007501	0.0103187	indole-3-glycerol phosphate synthase domain-containing protein
TGME49_224150	-7.63	4.72	0.0007564	0.0103603	hypothetical protein
TGME49_268740	-7.62	4.67	0.0007587	0.0103603	hypothetical protein
TGME49_218740	-8.05	5.59	0.0007587	0.0103603	membrane protein, putative
TGME49_216370	-7.59	4.47	0.0007635	0.0103734	hypothetical protein
TGME49_233450	1.33	8.80	0.0008014	0.0108314	SAG-related sequence SRS29A
TGME49_306620	1.44	6.77	0.0008097	0.010886	AP2 domain transcription factor AP2IX-9
TGME49_246930	1.17	7.67	0.0008149	0.0109371	calmodulin CAM1
TGME49_222210	-4.48	5.45	0.0008199	0.0109478	SPFH domain / Band 7 family protein
TGME49_244510	-7.66	4.44	0.0008256	0.0109713	AP2 domain transcription factor AP2VI-3
TGME49_309610	-7.99	4.83	0.0008342	0.0110431	hypothetical protein
TGME49_232640	1.73	5.52	0.0008409	0.0110989	RNA 2'-phosphotransferase, Tpt1/KptA family protein
TGME49_251590	-4.36	5.78	0.0008427	0.0110989	hypothetical protein
TGME49_316490	-7.84	4.86	0.0008612	0.0112852	hypothetical protein
TGME49_221980	-7.78	4.78	0.0008746	0.0114017	U1 zinc finger protein
TGME49_223530	1.30	6.96	0.0008909	0.0115564	hypothetical protein
TGME49_318380	-8.30	5.02	0.0009013	0.0116721	hypothetical protein
TGME49_246690	-7.90	4.76	0.0009077	0.0117146	alpha amylase, catalytic domain-containing protein
TGME49_247290	-8.11	5.13	0.0009235	0.0118792	hypothetical protein
TGME49_262000	-7.73	4.68	0.000932	0.0119612	AP2 domain transcription factor AP2VIIb-2
TGME49_277770	-8.16	5.05	0.0009329	0.0119612	hypothetical protein
TGME49_285540	-7.78	4.67	0.0009347	0.0119637	DNA-directed DNA polymerase
TGME49_225410	1.74	5.30	0.000943	0.0120107	histone H3 centromeric CENH3
TGME49_232220	-7.38	4.35	0.0009568	0.0121267	SWIB/MDM2 domain-containing protein
TGME49_253470	1.07	7.73	0.0009777	0.0123508	alveolin domain containing intermediate filament IMC13
TGME49_247340	2.56	5.26	0.000994	0.0125358	hypothetical protein
TGME49_258826	-8.03	4.94	0.0010452	0.0130021	hypothetical protein
TGME49_285710	1.37	6.33	0.0010461	0.0130021	hypothetical protein
TGME49_228040	-7.48	4.69	0.0010516	0.0130497	PPIC-type PPIASE domain-containing protein
TGME49_311060	-7.75	4.67	0.001066	0.0131857	metal-dependent phosphohydrolase HD domain-containing protein
TGME49_210478	2.80	4.55	0.0010783	0.0132959	hypothetical protein
TGME49_309000	-7.64	4.58	0.0010817	0.0133159	hypothetical protein

TGME49_305610	-7.34	4.51	0.0011129	0.0136131	hypothetical protein
TGME49_273530	-7.78	4.68	0.0011407	0.0137867	flagellar associated protein
TGME49_246560	1.01	7.73	0.0011416	0.0137867	vacuolar ATP synthase subunit g, putative
TGME49_233830	-7.61	4.39	0.0011434	0.0137867	hypothetical protein
TGME49_249450	-7.65	4.57	0.0011452	0.0137867	hypothetical protein
TGME49_258820	1.08	8.23	0.0011496	0.0137867	hypothetical protein
TGME49_310910	1.39	5.82	0.0011506	0.0137867	WD domain, G-beta repeat-containing protein
TGME49_264070	-7.41	4.80	0.0011582	0.0138388	F5/8 type C domain-containing protein
TGME49_267775	-7.80	4.74	0.0011919	0.0140387	hypothetical protein
TGME49_295020	-7.23	4.83	0.001193	0.0140387	Sterol-sensing domain of SREBP cleavage-activation domain-containing protein
TGME49_217692	-7.66	4.48	0.0012156	0.0142602	hypothetical protein
TGME49_310250	-7.69	4.54	0.0012234	0.01433	hypothetical protein
TGME49_217470	-7.70	4.81	0.0012315	0.0144035	hypothetical protein
TGME49_224170	2.43	6.03	0.0012754	0.0148545	SAG-related sequence SRS60A
TGME49_219640	-7.67	4.83	0.0012758	0.0148545	hypothetical protein
TGME49_271280	-7.40	4.70	0.0013199	0.0151897	60S ribosome subunit biogenesis protein NIP7, putative
TGME49_262810	-7.54	4.44	0.0013218	0.0151897	iron donor protein CyaY protein
TGME49_305455	-7.11	4.39	0.0013293	0.0152251	hypothetical protein
TGME49_241860	-7.70	4.57	0.0013574	0.015348	N-acetylglucosaminyl-phosphatidylinositol biosynthetic protein PigA, family GT4 protein
TGME49_291590	-7.62	4.47	0.0014007	0.0156489	hypothetical protein
TGME49_270940	-7.74	4.64	0.0014076	0.0156971	hypothetical protein
TGME49_232240	-7.27	4.44	0.0014091	0.0156971	hypothetical protein
TGME49_304640	-7.64	4.49	0.0014229	0.0158052	hypothetical protein
TGME49_255160	-7.75	4.70	0.0014402	0.0159512	hypothetical protein
TGME49_299270	1.55	5.46	0.0014858	0.0162702	hypothetical protein
TGME49_226640	2.46	5.09	0.0015181	0.0165532	zinc binding protein, putative
TGME49_273550	-8.07	4.90	0.0015222	0.0165745	hypothetical protein
TGME49_251530	-7.49	4.66	0.0015439	0.0167168	hypothetical protein
TGME49_285170	-7.62	4.38	0.001573	0.0169845	methyltransferase small, putative
TGME49_299070	1.18	6.85	0.0016299	0.017501	pyruvate kinase PyKII
TGME49_205625	-7.77	4.69	0.0016423	0.0175607	hypothetical protein
TGME49_283740	-7.60	4.51	0.0016431	0.0175607	RNA recognition motif-containing protein
TGME49_262630	-7.33	5.08	0.0016507	0.0176017	hypothetical protein
TGME49_268350	-7.61	4.37	0.0016533	0.017606	hypothetical protein
TGME49_218192	-7.59	4.97	0.0016853	0.0178975	hypothetical protein
TGME49_297925	-7.46	4.45	0.0017012	0.0179915	HesB-like domain-containing protein
TGME49_260150	-7.18	4.60	0.0017282	0.0182519	tetratricopeptide repeat-containing protein
TGME49_221880	-8.02	4.94	0.0017456	0.0183656	hypothetical protein
TGME49_215347	-7.36	4.37	0.0017461	0.0183656	hypothetical protein
TGME49_246140	-8.20	5.00	0.0017657	0.0185223	hypothetical protein
TGME49_318490	-7.78	4.67	0.0017976	0.0187339	hypothetical protein
TGME49_241310	-7.57	4.52	0.001798	0.0187339	hypothetical protein
TGME49_230920	-7.70	4.59	0.0018451	0.0190961	adaptor complexes medium subunit family protein
TGME49_242580	-7.80	4.77	0.0018546	0.0191103	iron only hydrogenase large subunit, c-terminal domain-containing protein
TGME49_253530	-7.47	4.49	0.0018864	0.0192994	hypothetical protein
TGME49_314680	-7.00	4.37	0.0018868	0.0192994	hypothetical protein
TGME49_235640	-7.65	4.76	0.0018873	0.0192994	formyl transferase domain-containing protein
TGME49_244140	-7.17	4.55	0.0019218	0.0195495	hypothetical protein
TGME49_243610	-7.67	4.80	0.0019393	0.0196756	C-5 cytosine-specific DNA methylase superfamily protein
TGME49_209530	-7.62	4.54	0.0019444	0.0196756	hypothetical protein
TGME49_269750	-7.63	4.49	0.0019585	0.0197412	CrcB family protein
TGME49_211400	-7.61	4.49	0.0019694	0.0198243	DEAD/DEAH box helicase domain-containing protein
TGME49_222160	1.75	5.27	0.0019933	0.0199876	aldehyde dehydrogenase
TGME49_314295	2.11	5.01	0.0020681	0.0205768	ribosomal l25 family protein
TGME49_254280	2.32	5.22	0.0021148	0.0209724	DNA-directed RNA polymerase III RPC9
TGME49_267670	-7.58	5.25	0.002116	0.0209724	hypothetical protein

TGME49_268340	2.13	4.37	0.0021654	0.0213531	glycosyltransferase family 28 C-terminal domain-containing protein
TGME49_314920	-7.87	4.87	0.0021915	0.0214971	hypothetical protein
TGME49_266870	-7.38	4.48	0.0022165	0.0216755	transporter, major facilitator family protein
TGME49_313273	-7.84	5.06	0.00222	0.0216755	hypothetical protein
TGME49_277050	-7.26	4.58	0.0022267	0.0216807	hypothetical protein
TGME49_291010	-7.55	4.59	0.0023266	0.0224558	hypothetical protein
TGME49_320680	-7.24	4.71	0.002411	0.0229693	AP2 domain transcription factor AP2IV-2
TGME49_204055	-7.60	4.62	0.0024155	0.0229693	hypothetical protein
TGME49_297495	-6.99	4.35	0.0024403	0.0231702	hypothetical protein
TGME49_211695	-7.00	4.79	0.002465	0.0232685	hypothetical protein
TGME49_262510	-7.33	4.36	0.0024853	0.0234028	GTP-binding protein engB, putative
TGME49_223580	-7.81	4.76	0.0025759	0.0239356	mediator complex subunit MED4
TGME49_249930	-7.03	4.51	0.0026156	0.0242125	hypothetical protein
TGME49_286550	1.37	5.61	0.0026182	0.0242125	hypothetical protein
TGME49_261410	-7.58	4.35	0.0026238	0.0242353	protein-tyrosine-phosphatase
TGME49_301160	-7.44	4.71	0.0026314	0.024272	SAG-related sequence SRS19C
TGME49_261030	-7.65	4.56	0.0026341	0.024272	pyridine nucleotide-disulfide oxidoreductase domain-containing protein
TGME49_274120	1.07	6.42	0.0026646	0.0244363	hypothetical protein
TGME49_213635	2.41	5.89	0.0027146	0.0246902	hypothetical protein
TGME49_205110	-7.19	4.59	0.0027214	0.0247224	hypothetical protein
TGME49_323320	-7.24	5.12	0.0027838	0.0250836	hypothetical protein
TGME49_216840	-7.25	4.42	0.0028127	0.0252915	hypothetical protein
TGME49_257980	-7.57	4.79	0.0028134	0.0252915	ribosome recycling factor protein
TGME49_271190	-7.37	4.36	0.0028708	0.0256547	bicoid-interacting protein BIN3
TGME49_236140	-7.22	4.58	0.0030213	0.0265432	hypothetical protein
TGME49_291330	1.08	7.23	0.0030644	0.0268601	RNA recognition motif-containing protein
TGME49_258420	-4.66	5.57	0.0031086	0.0270335	hypothetical protein
TGME49_270630	-7.29	4.51	0.0031264	0.0271271	hypothetical protein
TGME49_262680	-6.86	4.41	0.0031357	0.0271432	hypothetical protein
TGME49_219720	1.15	6.60	0.0031388	0.0271432	Ras-related protein Rab-5C, putative
TGME49_308930	1.34	6.31	0.0032033	0.0275778	50S ribosomal protein L33, putative
TGME49_329100	-7.28	4.45	0.003252	0.0279033	Toxoplasma gondii family C protein
TGME49_244580	-7.43	4.45	0.0032721	0.027944	L1P family of ribosomal protein
TGME49_255870	-7.37	4.35	0.0032754	0.027944	WD domain, G-beta repeat-containing protein
TGME49_216190	-7.43	4.52	0.0032966	0.027944	hypothetical protein
TGME49_254140	-6.57	4.37	0.0033503	0.0281838	DNA-directed RNA polymerase II RPABC4
TGME49_313090	-7.45	4.70	0.0033659	0.0282844	hypothetical protein
TGME49_210770	-7.39	4.36	0.003382	0.0283893	hypothetical protein
TGME49_226100	-7.32	5.21	0.0035371	0.0291995	haloacid dehalogenase family hydrolase domain-containing protein
TGME49_230830	1.42	6.09	0.0035493	0.0292219	ATPase family associated with various cellular activities (AAA) domain-containing protein
TGME49_271290	-7.33	4.59	0.0035583	0.0292343	hypothetical protein
TGME49_273540	-7.60	4.73	0.0036445	0.0298765	phosphatidylserine synthase, putative
TGME49_320580	-7.38	4.40	0.0036687	0.0299814	hypothetical protein
TGME49_268985	-7.07	4.36	0.0037098	0.0301269	hypothetical protein
TGME49_262430	1.19	6.47	0.003721	0.0301858	4-hydroxy-3-methylbut-2-en-1-yl diphosphate synthase
TGME49_239310	1.33	5.65	0.0037359	0.0302431	ribulose 5-phosphate isomerase
TGME49_234510	1.01	6.86	0.0037592	0.0303683	ankyrin repeat-containing protein
TGME49_253680	-7.15	4.57	0.003791	0.0305293	hypothetical protein
TGME49_225360	-7.78	4.57	0.003881	0.0310275	hypothetical protein
TGME49_318880	-7.09	4.84	0.0039113	0.0312375	hypothetical protein
TGME49_244430	-7.77	4.79	0.0039444	0.0312702	pseudouridylate synthase, putative
TGME49_263323	-7.32	4.34	0.0039518	0.0312702	tetratricopeptide repeat protein 11, putative
TGME49_264420	-7.53	5.00	0.0041327	0.032303	lipoprotein, putative
TGME49_275470	-7.33	4.92	0.0041528	0.032332	dense granule protein GRA15
TGME49_220212	-7.25	4.42	0.0043016	0.033095	pseudouridine synthase

TGME49_263430	-7.50	4.44	0.0043566	0.0334132	3-hydroxyisobutyrate dehydrogenase
TGME49_207970	-7.09	4.39	0.0046183	0.0348335	HEAT repeat-containing protein
TGME49_254260	1.66	4.71	0.0047217	0.0354746	COX19 cytochrome c oxidase assembly family protein
TGME49_289900	-7.04	4.43	0.0047322	0.0355193	n-acetyltransferase family protein
TGME49_272370	1.03	8.31	0.0047662	0.0356358	hypothetical protein
TGME49_316610	-7.62	4.67	0.0048161	0.0359392	hypothetical protein
TGME49_271250	-7.50	4.45	0.0049018	0.0364736	hypothetical protein
TGME49_323110	1.23	6.57	0.0049629	0.0368215	hypothetical protein
TGME49_201840	1.04	7.40	0.005109	0.0377967	aspartyl protease ASP1
TGME49_312680	1.54	4.40	0.0051626	0.0380118	60S ribosomal protein L27, putative
TGME49_233930	-7.30	4.47	0.0052027	0.0382336	CAF1 family ribonuclease
TGME49_306455	2.46	4.82	0.005421	0.0393198	hypothetical protein
TGME49_223725	-7.40	4.70	0.0055391	0.0399466	hypothetical protein
TGME49_209490	1.17	6.16	0.0057731	0.0411735	hypothetical protein
TGME49_258900	1.36	5.48	0.0058558	0.0414574	hypothetical protein
TGME49_222155	-6.92	4.39	0.0059128	0.0417839	hypothetical protein
TGME49_290630	1.29	5.88	0.006016	0.0422817	AP2 domain transcription factor AP2IX-7
TGME49_269417	-7.03	4.43	0.0060976	0.042777	hypothetical protein
TGME49_207470	-7.26	4.39	0.0063103	0.043852	hypothetical protein
TGME49_210800	1.23	6.12	0.0063132	0.043852	activator of hsp90 ATPase, putative
TGME49_267580	1.04	7.15	0.0064424	0.0445642	cyclin2 related protein
TGME49_250500	-7.08	4.89	0.0064611	0.0445991	hypothetical protein
TGME49_213460	1.08	7.26	0.0065283	0.0449614	hypothetical protein
TGME49_233130	1.30	5.80	0.0065939	0.0453056	nucleoside transporter protein
TGME49_207070	2.47	4.76	0.006761	0.0460114	glycosyl transferase, putative
TGME49_257740	1.11	6.52	0.0068932	0.0468286	UMP-CMP kinase
TGME49_263410	2.78	4.51	0.0069213	0.0469357	scavenger receptor cysteine-rich domain-containing protein
TGME49_234460	1.01	6.92	0.0071892	0.0482398	hypothetical protein
TGME49_244260	-7.47	4.72	0.0075131	0.0499575	hypothetical protein

Supplementary Table S3C: Differencial T. gondii gene expressin analysis of human HFF cells infected with T. gondii EGS strain for 48 hours compared to the average T. gondii gene expression of human MM6 and neuronal stem cells infected with either GT1, ME49 or VEG strains of T. gondii for 18 hours.

gene ID	logFC	logCPM	PValue	FDR	Product name
TGME49_462910	11.89	7.86	3.02E-142	2.34E-138	large subunit ribosomal RNA
TGME49_457990	14.29	11.10	1.90E-114	7.36E-111	28S ribosomal RNA
TGME49_280570	6.48	8.90	1.67E-109	4.31E-106	SAG-related sequence SRS35A
TGME49_200010	10.81	6.04	1.23E-80	2.39E-77	hypothetical protein
TGME49_205250	4.15	9.76	2.56E-77	3.96E-74	rhoptry protein ROP18
TGME49_207210	4.91	7.68	3.88E-47	5.00E-44	hypothetical protein
TGME49_291040	5.12	7.28	9.57E-44	1.06E-40	lactate dehydrogenase LDH2
TGME49_209755	5.58	7.50	1.56E-40	1.50E-37	hypothetical protein
TGME49_323400	4.59	8.61	2.99E-38	2.57E-35	cytochrome c oxidase subunit iii subfamily protein
TGME49_214080	3.46	9.15	7.52E-34	5.82E-31	toxofilin
TGME49_259020	5.87	7.36	7.52E-32	5.29E-29	bradyzoite antigen BAG1
TGME49_221840	4.38	5.63	1.01E-31	6.52E-29	hypothetical protein
TGME49_237130	4.35	9.60	4.84E-30	2.88E-27	cytochrome b, putative
TGME49_278080	4.54	5.97	3.08E-29	1.70E-26	Toxoplasma gondii family A protein
TGME49_330000	4.62	9.57	1.34E-28	6.89E-26	cytochrome b
TGME49_252640	5.81	6.77	2.04E-28	9.85E-26	P-type ATPase PMA1
TGME49_301250	3.67	14.08	2.90E-27	1.32E-24	hypothetical protein
TGME49_268860	5.65	5.85	1.94E-25	8.31E-23	enolase 1
TGME49_293790	3.95	6.58	2.20E-25	8.94E-23	hypothetical protein
TGME49_202020	3.30	8.16	2.64E-25	1.02E-22	DnAK-TPR
TGME49_252070	4.63	5.64	1.49E-21	5.50E-19	KRUF family protein
TGME49_207160	4.66	7.01	4.42E-21	1.55E-18	SAG-related sequence SRS49D
TGME49_321480	4.55	5.82	2.88E-18	9.25E-16	SAG-related sequence SRS12B
TGME49_260310	3.09	6.15	3.11E-18	9.26E-16	ATP-binding cassette transporter ABC.B1
TGME49_206550	3.33	6.24	4.16E-18	1.19E-15	hypothetical protein
TGME49_243470	4.67	4.96	1.38E-17	3.78E-15	hypothetical protein
TGME49_216140	2.49	7.81	1.87E-16	4.83E-14	tetratricopeptide repeat-containing protein
TGME49_305460	2.50	7.84	4.49E-16	1.12E-13	methionine aminopeptidase 2, putative
TGME49_253100	1.81	7.65	2.78E-15	6.72E-13	hypothetical protein
TGME49_227430	2.76	6.42	5.87E-15	1.37E-12	transmembrane amino acid transporter protein
TGME49_254460	1.98	7.76	7.95E-15	1.81E-12	hypothetical protein
TGME49_287460	3.13	5.08	8.89E-15	1.96E-12	hypothetical protein
TGME49_222370	1.97	7.44	2.20E-14	4.72E-12	SAG-related sequence SRS13
TGME49_225290	3.06	6.51	2.52E-14	5.27E-12	GDA1/CD39 (nucleoside phosphatase) family protein
TGME49_253330	2.64	8.53	3.93E-14	7.99E-12	Rhoptry kinase family protein, truncated (incomplete catalytic triad)
TGME49_255060	3.30	9.20	4.31E-14	8.54E-12	cytochrome b(N-terminal)/b6/petB subfamily protein
TGME49_315100	3.91	5.66	1.56E-13	2.94E-11	hypothetical protein
TGME49_280580	2.66	6.00	4.74E-12	8.72E-10	SAG-related sequence SRS35B
TGME49_268230	-9.26	5.82	2.59E-11	4.66E-09	hypothetical protein
TGME49_254520	1.88	7.59	4.93E-11	8.66E-09	mediator complex subunit MED11
TGME49_254080	1.41	8.07	7.26E-11	1.22E-08	metal cation transporter, ZIP family protein
TGME49_275750	1.80	7.30	6.15E-10	9.70E-08	small nuclear ribonucleoprotein E, putative
TGME49_299030	1.73	7.72	8.40E-10	1.27E-07	RNA recognition motif 2 protein
TGME49_232035	-4.53	5.47	1.04E-09	1.55E-07	hypothetical protein
TGME49_253880	1.45	8.09	1.13E-09	1.65E-07	GNS1/SUR4 family protein
TGME49_253790	1.53	7.52	1.35E-09	1.88E-07	zinc finger (CCCH type) motif-containing protein
TGME49_293252	4.86	5.29	1.58E-09	2.14E-07	hypothetical protein
TGME49_276940	1.41	8.43	1.69E-09	2.25E-07	ribosome associated membrane protein RAMP4, putative

TGME49_293840	2.10	6.76	2.73E-09	3.48E-07	hypothetical protein
TGME49_268225	-7.05	6.16	2.76E-09	3.48E-07	hypothetical protein
TGME49_293780	1.90	7.63	2.79E-09	3.48E-07	hypothetical protein
TGME49_312105	-5.44	5.62	3.68E-09	4.38E-07	hypothetical protein
TGME49_254630	1.64	7.14	4.37E-09	4.97E-07	CMGC kinase
TGME49_271320	3.07	4.81	4.84E-09	5.42E-07	hypothetical protein
TGME49_217750	-4.62	5.70	9.05E-09	9.85E-07	hypothetical protein
TGME49_290970	2.50	7.40	9.77E-09	1.05E-06	8-amino-7-oxononanoate synthase
TGME49_312160	1.57	8.48	2.70E-08	2.78E-06	hypothetical protein
TGME49_298980	1.58	7.26	5.09E-08	4.86E-06	RNA pseudouridine synthase superfamily protein
TGME49_321170	2.39	5.58	6.97E-08	6.49E-06	Toxoplasma gondii family C protein
TGME49_228980	-4.97	4.76	7.79E-08	7.00E-06	hypothetical protein
TGME49_248740	1.82	8.90	8.97E-08	7.97E-06	hypothetical protein
TGME49_296340	2.84	5.50	9.58E-08	8.42E-06	hypothetical protein
TGME49_208590	1.30	7.67	1.04E-07	8.98E-06	vacuolar ATP synthase subunit 54kD, putative
TGME49_254365	1.44	7.90	2.03E-07	1.68E-05	phosphatidate cytidylyltransferase
TGME49_313900	2.08	6.92	2.82E-07	2.29E-05	non-specific serine/threonine protein kinase
TGME49_308020	1.10	8.94	2.90E-07	2.31E-05	SAG-related sequence SRS57
TGME49_254920	2.48	5.18	2.90E-07	2.31E-05	hypothetical protein
TGME49_253470	1.44	7.73	3.11E-07	2.43E-05	alveolin domain containing intermediate filament IMC13
TGME49_209985	1.97	6.55	3.11E-07	2.43E-05	cAMP-dependent protein kinase
TGME49_229990	1.15	8.03	4.48E-07	3.30E-05	T-complex protein 1 subunit alpha, putative
TGME49_268170	-4.39	5.04	5.00E-07	3.63E-05	hypothetical protein
TGME49_315220	1.15	8.41	5.03E-07	3.63E-05	rhoptry protein ROP14
TGME49_270320	1.34	8.41	5.96E-07	4.27E-05	protein phosphatase 2C domain-containing protein
TGME49_263215	-4.51	4.62	6.32E-07	4.43E-05	hypothetical protein
TGME49_232410	1.14	9.84	6.36E-07	4.43E-05	PDI family protein
TGME49_290700	1.15	8.51	6.45E-07	4.45E-05	hypothetical protein
TGME49_310060	1.98	6.19	7.64E-07	5.20E-05	small nuclease
TGME49_264240	-4.81	5.23	8.57E-07	5.76E-05	hypothetical protein
TGME49_288475	3.34	6.01	1.24E-06	8.17E-05	hypothetical protein
TGME49_261740	1.29	11.35	1.37E-06	8.85E-05	hypothetical protein
TGME49_285710	1.72	6.33	1.38E-06	8.85E-05	hypothetical protein
TGME49_258820	1.48	8.23	1.58E-06	9.86E-05	hypothetical protein
TGME49_299200	1.55	7.12	1.61E-06	9.96E-05	Bet3 transport protein, putative
TGME49_247770	1.08	8.12	1.68E-06	0.0001028	hypothetical protein
TGME49_254690	1.20	8.49	1.82E-06	0.0001105	phospholipase/carboxylesterase
TGME49_223900	2.97	5.26	2.28E-06	0.0001333	hypothetical protein
TGME49_276140	1.13	9.71	2.76E-06	0.0001558	ADP ribosylation factor ARF1
TGME49_221220	2.66	5.26	2.87E-06	0.0001606	hypothetical protein
TGME49_268730	1.94	5.79	3.06E-06	0.0001689	glutaredoxin-related protein
TGME49_207830	1.54	6.50	3.66E-06	0.0001964	MORN repeat-containing protein
TGME49_315250	1.54	6.28	4.04E-06	0.0002122	GAMM1 protein, putative
TGME49_275860	1.07	9.69	4.17E-06	0.0002148	hypothetical protein
TGME49_262590	-5.38	5.01	4.59E-06	0.0002334	hypothetical protein
TGME49_232955	-5.63	10.84	4.62E-06	0.0002335	hypothetical protein
TGME49_254400	2.00	5.74	4.71E-06	0.0002366	LSU ribosomal protein L2P, putative
TGME49_252880	1.68	7.59	4.87E-06	0.0002428	hypothetical protein
TGME49_299070	1.47	6.85	4.95E-06	0.0002456	pyruvate kinase PyKII
TGME49_239500	1.30	8.04	5.03E-06	0.0002476	proteasome subunit alpha type, putative
TGME49_314250	2.65	5.27	5.88E-06	0.0002807	bradyzoite rhoptry protein BRP1
TGME49_319550	1.10	7.84	6.05E-06	0.0002869	membrane protein C17G8.08c, putative
TGME49_226100	-7.32	5.21	6.43E-06	0.0002994	haloacid dehalogenase family hydrolase domain-containing protein
TGME49_253000	1.20	7.09	7.72E-06	0.0003509	ELMO/CED-12 family protein
TGME49_254000	1.21	7.49	8.99E-06	0.0003994	hypothetical protein
TGME49_299990	1.46	5.96	1.03E-05	0.0004493	archease family protein
TGME49_260870	2.09	5.93	1.09E-05	0.0004715	zinc finger cdgsh type protein

TGME49_260430	2.41	5.07	1.32E-05	0.0005475	hypothetical protein
TGME49_276210	1.17	7.27	1.35E-05	0.0005564	phosphoglycerate mutase family protein
TGME49_262050	1.18	9.45	1.46E-05	0.0005956	rhoptry kinase family protein ROP39
TGME49_226755	1.30	6.78	1.51E-05	0.0006155	3'5'-cyclic nucleotide phosphodiesterase domain-containing protein
TGME49_218270	1.22	7.71	1.57E-05	0.0006341	hypothetical protein
TGME49_252440	2.11	4.64	1.58E-05	0.0006343	peptidase c13 family protein
TGME49_239490	1.14	8.37	1.66E-05	0.0006524	dehydrogenase E1 component family protein
TGME49_209850	1.51	7.19	1.79E-05	0.0006974	RNA recognition motif-containing protein
TGME49_249780	1.09	7.77	1.81E-05	0.0007034	hypothetical protein
TGME49_254570	1.21	7.56	1.88E-05	0.0007169	hypothetical protein
TGME49_208850	2.67	6.23	1.92E-05	0.0007232	SAG-related sequence SRS11
TGME49_211010	1.79	7.49	2.04E-05	0.0007662	hypothetical protein
TGME49_278020	1.86	5.35	2.26E-05	0.0008314	hypothetical protein
TGME49_225050	1.06	7.72	2.32E-05	0.0008489	adenosylhomocysteinase, putative
TGME49_283790	1.50	6.66	2.37E-05	0.0008592	protein kinase, putative
TGME49_292260	3.28	4.64	2.39E-05	0.000862	SAG-related sequence SRS36B
TGME49_252190	1.56	6.82	2.43E-05	0.0008739	KRUF family protein
TGME49_312600	1.07	8.45	2.45E-05	0.0008739	heat shock protein HSP21
TGME49_202025	2.76	4.49	2.64E-05	0.0009358	hypothetical protein
TGME49_254410	1.30	7.01	2.69E-05	0.0009484	protein phosphatase 2C, putative
TGME49_276220	1.53	6.43	3.01E-05	0.001043	hypothetical protein
TGME49_287040	1.15	7.28	3.25E-05	0.0011012	hypothetical protein
TGME49_310520	1.52	5.61	3.46E-05	0.0011564	3'5'-cyclic nucleotide phosphodiesterase domain-containing protein
TGME49_205050	-5.06	5.33	3.51E-05	0.0011701	hypothetical protein
TGME49_254380	1.62	5.84	3.95E-05	0.0012872	ribosomal protein L11, putative
TGME49_320515	1.15	7.08	4.28E-05	0.0013551	hypothetical protein
TGME49_215960	1.03	9.11	4.90E-05	0.0015346	hypothetical protein
TGME49_217160	2.35	5.01	5.26E-05	0.001639	Der1 family protein
TGME49_246930	1.29	7.67	6.29E-05	0.0018994	calmodulin CAM1
TGME49_300990	1.84	5.19	6.36E-05	0.0019129	Toxoplasma gondii family C protein
TGME49_311230	1.03	9.71	7.26E-05	0.0021121	hypothetical protein
TGME49_207970	-5.26	4.39	7.86E-05	0.0022268	HEAT repeat-containing protein
TGME49_290678	1.70	6.76	8.00E-05	0.0022581	hypothetical protein
TGME49_213820	1.16	7.05	8.85E-05	0.0024611	hypothetical protein
TGME49_258550	1.79	6.28	8.94E-05	0.0024767	SAG-related sequence SRS28
TGME49_236990	1.74	5.87	9.11E-05	0.0025146	beta-ketoacyl synthase, N-terminal domain-containing protein
TGME49_242118	-4.57	5.24	9.47E-05	0.0026059	myosin-light-chain kinase
TGME49_259700	1.14	7.11	9.71E-05	0.0026626	hypothetical protein
TGME49_248450	1.22	6.38	9.93E-05	0.0027125	zinc finger, C3HC4 type (RING finger) domain-containing protein
TGME49_288880	2.14	4.68	0.0001064	0.002865	hypothetical protein
TGME49_260480	1.80	5.57	0.0001079	0.0028857	leucine rich repeat-containing protein
TGME49_311490	2.49	4.65	0.0001089	0.0028937	hypothetical protein
TGME49_285720	1.70	5.25	0.0001097	0.0028941	ATP binding protein, putative
TGME49_213460	1.36	7.26	0.0001097	0.0028941	hypothetical protein
TGME49_288870	1.95	4.96	0.0001199	0.0031013	hypothetical protein
TGME49_289090	2.52	4.72	0.0001218	0.0031391	hypothetical protein
TGME49_254390	1.21	7.74	0.0001226	0.0031499	CRAL/TRIO domain-containing protein
TGME49_285510	1.03	7.63	0.0001291	0.003293	hypothetical protein
TGME49_264660	1.29	9.56	0.0001361	0.0034509	SAG-related sequence SRS44
TGME49_215540	-6.53	4.49	0.0001403	0.0035208	hypothetical protein
TGME49_313440	1.66	8.63	0.0001847	0.0044769	hypothetical protein
TGME49_266740	1.93	5.45	0.000186	0.0044934	RNA recognition motif-containing protein
TGME49_253970	1.91	4.68	0.0001869	0.0045006	hypothetical protein
TGME49_223095	2.42	4.37	0.0002005	0.0047684	hypothetical protein

TGME49_215750	2.18	4.88	0.0002022	0.0047959	cytidine and deoxycytidylate deaminase zinc-binding region domain-containing protein
TGME49_245475	1.73	5.61	0.0002541	0.0057776	hypothetical protein
TGME49_275870	1.43	6.07	0.0002993	0.0066299	tubulin/FtsZ family, GTPase domain-containing protein
TGME49_308930	1.46	6.31	0.0003092	0.0067915	50S ribosomal protein L33, putative
TGME49_270560	1.90	4.63	0.0003175	0.0069545	peptidyl-prolyl cis-trans isomerase family 1
TGME49_232550	1.18	7.77	0.0003311	0.0071908	hypothetical protein
TGME49_257685	1.16	7.60	0.0003497	0.0075106	hypothetical protein
TGME49_232640	1.57	5.52	0.0003847	0.0081151	RNA 2'-phosphotransferase, Tpt1/KptA family protein
TGME49_249480	1.21	6.66	0.0003944	0.0082861	tetratricopeptide repeat-containing protein
TGME49_275770	1.18	6.36	0.0004123	0.0085909	hypothetical protein
TGME49_315260	-4.45	4.43	0.0004197	0.0087225	alanine dehydrogenase
TGME49_307820	1.33	6.37	0.0004367	0.0089557	hypothetical protein
TGME49_208730	1.06	8.82	0.0004414	0.0089816	microneme protein, putative
TGME49_298970	1.05	7.13	0.0004469	0.0090505	LSM3, U6 small nuclear RNA associated isoform 2 family protein
TGME49_277260	1.08	10.15	0.0004764	0.0095409	hypothetical protein
TGME49_257350	1.09	8.63	0.0004881	0.0097499	eukaryotic translation initiation factor, putative
TGME49_270260	2.03	4.99	0.0004964	0.0098485	hypothetical protein
TGME49_251180	1.09	6.82	0.0004991	0.0098485	KRUF family protein
TGME49_309130	2.10	5.09	0.0005346	0.0104098	hypothetical protein
TGME49_306640	1.55	6.29	0.0005476	0.0105818	hypothetical protein
TGME49_214575	1.31	6.21	0.0005808	0.0110176	hypothetical protein
TGME49_252290	1.33	7.47	0.0006241	0.0116834	importin alpha, putative
TGME49_286560	1.58	6.07	0.0006436	0.0119897	U6 snRNA-associated Sm family protein
TGME49_225470	1.32	6.33	0.0007319	0.0132196	peptide methionine sulfoxide reductase
TGME49_299980	1.08	7.50	0.000761	0.0136499	hypothetical protein
TGME49_229500	1.54	6.14	0.000772	0.0137844	hypothetical protein
TGME49_257160	1.02	7.33	0.0008212	0.0143774	hypothetical protein
TGME49_226068	1.08	6.67	0.0008776	0.0150822	DnaJ domain-containing protein
TGME49_233695	1.09	6.72	0.0009198	0.015595	hypothetical protein
TGME49_230100	1.63	5.78	0.0009601	0.0161712	membrane protein, putative
TGME49_219660	2.21	4.74	0.000976	0.0162963	hypothetical protein
TGME49_231000	1.28	6.28	0.001028	0.0169098	START domain-containing protein
TGME49_305990	1.67	5.91	0.0010349	0.0169367	hypothetical protein
TGME49_223610	1.63	6.77	0.0010384	0.0169367	hypothetical protein
TGME49_204520	1.45	5.24	0.0010408	0.01694	hypothetical protein
TGME49_218810	1.22	6.03	0.0010561	0.0170552	histidyl-tRNA synthetase
TGME49_250220	1.36	6.34	0.0011201	0.017698	hypothetical protein
TGME49_276130	1.13	6.83	0.0011217	0.017698	cathepsin CPC2
TGME49_271090	1.87	4.91	0.0011372	0.0178335	hypothetical protein
TGME49_249890	1.73	5.18	0.0011841	0.0184567	hypothetical protein
TGME49_243980	1.48	5.21	0.0012235	0.0189942	hypothetical protein
TGME49_275755	1.72	5.48	0.0012724	0.0196737	hypothetical protein
TGME49_270800	1.35	6.28	0.0013471	0.020501	GAF domain-containing protein
TGME49_216335	1.84	5.09	0.0014676	0.0217776	hypothetical protein
TGME49_238420	1.45	6.38	0.0014765	0.021868	hypothetical protein
TGME49_313040	1.23	5.74	0.0014925	0.0220375	hypothetical protein
TGME49_294040	1.03	7.00	0.0014972	0.0220375	KDEL endoplasmic reticulum proteinretention receptor 2 (KDELR2), putative
TGME49_252065	1.68	5.50	0.0015271	0.0224023	KRUF family protein
TGME49_212960	1.39	6.08	0.0016819	0.0242332	hypothetical protein
TGME49_233100	1.21	7.71	0.0017099	0.0245715	SPFH domain / Band 7 family protein
TGME49_310070	1.00	7.66	0.0017328	0.0248076	methyltransferase, putative
TGME49_312210	1.28	5.61	0.0017411	0.0248808	hypothetical protein
TGME49_262430	1.17	6.47	0.0017995	0.0255261	4-hydroxy-3-methylbut-2-en-1-yl diphosphate synthase
TGME49_238880	1.82	6.36	0.0018575	0.0262045	hypothetical protein
TGME49_309380	1.09	6.75	0.0019237	0.0269424	Nuf2

TGME49_213746	1.98	4.73	0.001955	0.0272813	hypothetical protein
TGME49_202580	1.20	6.60	0.0019867	0.027674	ATPase, AAA family protein
TGME49_308090	1.04	10.08	0.0020052	0.0278317	rhoptry protein ROP5
TGME49_258490	1.73	5.52	0.002038	0.028135	hypothetical protein
TGME49_299080	1.04	6.80	0.0020499	0.0282174	VTC domain-containing protein
TGME49_239610	1.28	5.65	0.0021372	0.0290388	hypothetical protein
TGME49_253730	1.12	7.18	0.0022206	0.0299088	importin-beta N-terminal domain-containing protein
TGME49_253940	1.02	7.11	0.0022363	0.030067	CAM Kinase family, incomplete catalytic triad
TGME49_288820	1.04	7.00	0.0022597	0.0302239	hypothetical protein
TGME49_310220	1.17	6.44	0.0024318	0.0317371	hypothetical protein
TGME49_243480	1.88	5.50	0.0024923	0.0321133	50S ribosomal protein L3, putative
TGME49_253900	1.26	6.78	0.0025305	0.0323792	parasite porphobilinogen synthase PBGS
TGME49_224170	2.22	6.03	0.0026178	0.0332779	SAG-related sequence SRS60A
TGME49_244645	1.38	5.13	0.0026214	0.0332779	hypothetical protein
TGME49_204060	1.41	6.00	0.0026463	0.0335385	SNARE domain-containing protein
TGME49_260150	1.72	4.60	0.0027033	0.034064	tetratricopeptide repeat-containing protein
TGME49_217400	1.19	6.75	0.0027168	0.0340963	hypothetical protein
TGME49_254090	1.06	6.60	0.0028079	0.0348443	hypothetical protein
TGME49_260520	1.01	7.53	0.0028225	0.0348518	hypothetical protein
TGME49_260660	1.25	5.71	0.002919	0.0357065	50S ribosomal protein L23, putative
TGME49_252630	1.05	8.18	0.0029302	0.0357876	hypothetical protein
TGME49_263390	1.37	5.79	0.0029886	0.0364426	hypothetical protein
TGME49_211860	1.05	6.19	0.0030585	0.0371192	hypothetical protein
TGME49_301222	1.27	6.33	0.0033819	0.0398783	DNA repair protein Rad4 domain-containing protein
TGME49_269420	1.15	7.56	0.0033838	0.0398783	hypothetical protein
TGME49_252870	1.32	4.81	0.0036159	0.0416609	hypothetical protein
TGME49_228710	1.91	4.99	0.0037212	0.0424575	hypothetical protein
TGME49_215550	1.25	5.82	0.0037217	0.0424575	hypothetical protein
TGME49_271240	1.25	5.40	0.003736	0.0424628	hypothetical protein
TGME49_218530	1.09	6.69	0.0037938	0.0428714	proteasome-interacting thioredoxin domain-containing protein
TGME49_254890	1.83	5.36	0.0038642	0.0433252	hypothetical protein
TGME49_251450	1.06	6.46	0.0039132	0.0435925	hypothetical protein
TGME49_256100	1.60	5.01	0.0039304	0.0436584	tetratricopeptide repeat-containing protein
TGME49_251770	1.01	7.36	0.003954	0.0438573	hypothetical protein
TGME49_262910	1.72	5.45	0.0039895	0.0441875	NADH-cytochrome b5 reductase 1, putative
TGME49_285470	1.70	5.82	0.0040374	0.0445271	patched family protein
TGME49_279330	1.31	5.84	0.0041227	0.0453376	DEAD/DEAH box helicase family protein
TGME49_255890	1.19	6.04	0.0041367	0.0453629	pyridine nucleotide-disulfide oxidoreductase domain-containing protein
TGME49_253180	1.08	7.97	0.0041855	0.0458333	hypothetical protein
TGME49_231940	1.10	6.46	0.0043071	0.0466856	ThiF family protein
TGME49_222130	1.02	7.05	0.0043649	0.0469335	hypothetical protein
TGME49_253960	1.29	5.45	0.0043774	0.0470025	oxidoreductase, short chain dehydrogenase/reductase family protein
TGME49_251570	2.16	4.62	0.0043857	0.047026	CAAX amino terminal protease family protein
TGME49_262480	1.16	6.65	0.0044027	0.047078	dynein light chain roadblock-type 2, putative
TGME49_233130	1.18	5.80	0.0044241	0.0471764	nucleoside transporter protein
TGME49_251400	1.27	5.90	0.0045372	0.0479293	hypothetical protein
TGME49_310840	1.42	5.00	0.0045639	0.0480052	hypothetical protein
TGME49_261075	1.60	4.62	0.0046303	0.0485408	hypothetical protein
TGME49_313700	1.58	4.85	0.0046337	0.0485408	hypothetical protein
TGME49_262040	1.01	6.73	0.0046568	0.048717	SAC3/GANP family protein
TGME49_294980	1.32	6.15	0.0048281	0.049901	hypothetical protein
TGME49_234300	1.29	6.21	0.0048389	0.0499457	hypothetical protein
TGME49_285810	1.46	5.26	0.0048492	0.0499853	MYND finger domain-containing protein

Supplementary Table S4: Differential gene expression analysis of human MM6 cells infected with T. gondii EGS strain for 18 hours.

gene ID	logFC (EGS/CONT)	logCPM	PValue	FDR	Product Name
ENSG00000137959	8.67	7.38	3.4319E-151	4.5554E-147	interferon-induced protein 44-like
ENSG00000185885	8.20	4.54	5.06787E-75	4.80507E-72	interferon induced transmembrane protein 1
ENSG00000088827	8.08	4.98	9.16289E-79	1.10571E-75	sialic acid binding Ig-like lectin 1, sialoadhesin
ENSG00000111335	7.66	5.56	2.50817E-81	3.69927E-78	2'-5'-oligoadenylate synthetase 2, 69/71kDa
ENSG00000213626	7.25	4.68	5.13744E-73	4.26215E-70	limb bud and heart development
ENSG00000137965	6.74	6.59	1.924E-113	1.277E-109	interferon-induced protein 44
ENSG00000100453	6.72	4.83	4.94419E-75	4.80507E-72	granzyme B (granzyme 2, cytotoxic T-lymphocyte-associated serine esterase 1)
ENSG00000133106	6.54	4.72	1.03262E-68	6.52715E-66	epithelial stromal interaction 1 (breast)
ENSG00000115155	6.51	5.35	2.25682E-85	4.27958E-82	otoferlin
ENSG00000137801	6.42	7.08	9.5633E-110	4.2314E-106	thrombospondin 1
ENSG00000157601	6.39	8.70	9.8017E-94	2.60216E-90	MX dynamin-like GTPase 1
ENSG00000198959	6.36	7.57	3.52821E-83	5.85419E-80	transglutaminase 2
ENSG00000133316	6.27	11.34	3.35032E-11	6.46397E-10	WD repeat domain 74
ENSG00000111331	6.19	5.93	7.16376E-80	9.50918E-77	2'-5'-oligoadenylate synthetase 3, 100kDa
ENSG00000089127	6.14	6.34	7.95904E-86	1.7608E-82	2'-5'-oligoadenylate synthetase 1, 40/46kDa
ENSG00000169871	6.10	8.22	1.0399E-107	3.4508E-104	tripartite motif containing 56
ENSG00000135114	5.99	4.84	3.71637E-52	1.40946E-49	2'-5'-oligoadenylate synthetase-like
ENSG00000072694	5.87	5.86	1.30322E-50	4.55235E-48	Fc fragment of IgG, low affinity IIb, receptor (CD32)
ENSG00000196562	5.78	4.31	2.26174E-45	6.38773E-43	sulfatase 2
ENSG00000189221	5.62	4.22	3.80742E-49	1.26349E-46	monoamine oxidase A
ENSG00000269337	5.61	5.24	2.60947E-69	1.7319E-66	AL591479.1
ENSG00000181195	5.60	4.57	8.21492E-57	4.19403E-54	proenkephalin
ENSG00000225614	5.52	5.10	5.63922E-71	4.15862E-68	zinc finger protein 469
ENSG00000134369	5.24	5.29	2.59166E-16	1.00297E-14	neuron navigator 1
ENSG00000152404	5.23	8.72	5.87336E-70	4.10332E-67	CWF19-like 2, cell cycle control (S. pombe)
ENSG00000142192	5.11	6.36	2.6033E-72	2.03272E-69	amyloid beta (A4) precursor protein
ENSG00000154153	5.00	4.24	7.42069E-42	1.69831E-39	family with sequence similarity 134, member B
ENSG00000134321	4.99	5.26	2.23195E-56	1.09729E-53	radical S-adenosyl methionine domain containing 2
ENSG00000126709	4.96	9.69	5.67393E-60	3.2746E-57	interferon, alpha-inducible protein 6
ENSG00000187608	4.89	7.31	1.44161E-76	1.59466E-73	ISG15 ubiquitin-like modifier
ENSG00000136960	4.79	4.81	4.70732E-46	1.35837E-43	ectonucleotide pyrophosphatase/phosphodiesterase 2
ENSG00000196141	4.74	4.94	1.11687E-51	4.11814E-49	spermatogenesis associated, serine-rich 2-like
ENSG00000136235	4.71	5.73	3.04853E-40	6.5268E-38	glycoprotein (transmembrane) nmb
ENSG00000184557	4.68	4.96	7.10429E-51	2.54871E-48	suppressor of cytokine signaling 3
ENSG00000115414	4.65	8.27	6.83832E-43	1.6504E-40	fibronectin 1
ENSG00000152104	4.50	7.55	4.36243E-55	1.99679E-52	protein tyrosine phosphatase, non-receptor type 14
ENSG00000115977	4.50	7.37	5.22892E-74	4.62724E-71	AP2 associated kinase 1
ENSG00000213918	4.46	4.40	1.60216E-40	3.48641E-38	deoxyribonuclease I
ENSG00000028277	4.45	8.29	2.6818E-66	1.6181E-63	POU class 2 homeobox 2
ENSG00000130821	4.44	6.14	4.7242E-47	1.49307E-44	solute carrier family 6 (neurotransmitter transporter), member 8
ENSG00000184979	4.42	4.53	5.34401E-31	7.23841E-29	ubiquitin specific peptidase 18
ENSG00000203812	4.25	6.19	5.77851E-50	1.96677E-47	histone cluster 2, H2aa3
ENSG00000130589	4.25	6.48	2.07185E-53	8.33386E-51	helicase with zinc finger 2, transcriptional coactivator
ENSG00000181458	4.21	5.34	6.83957E-22	4.90748E-20	transmembrane protein 45A
ENSG00000247095	4.19	4.36	2.67412E-17	1.1646E-15	MIR210 host gene
ENSG00000135931	4.16	5.06	1.61538E-37	2.97813E-35	armadillo repeat containing 9
ENSG00000180509	4.04	5.10	2.07949E-48	6.73248E-46	potassium channel, voltage gated subfamily E regulatory beta subunit 1
ENSG00000038427	4.02	9.79	6.23954E-40	1.29412E-37	versican
ENSG00000125148	3.99	6.60	1.6252E-34	2.50848E-32	metallothionein 2A
ENSG00000109881	3.98	6.66	2.461E-37	4.47498E-35	coiled-coil domain containing 34
ENSG00000114013	3.95	6.20	4.88717E-53	1.90801E-50	CD86 molecule
ENSG00000204642	3.94	5.21	2.3395E-42	5.54545E-40	major histocompatibility complex, class I, F
ENSG00000152457	3.93	7.01	1.73576E-59	9.60017E-57	DNA cross-link repair 1C
ENSG00000203710	3.88	4.99	2.28673E-35	3.74741E-33	complement component (3b/4b) receptor 1 (Knops blood group)
ENSG00000244509	3.86	6.61	2.36519E-55	1.12127E-52	apolipoprotein B mRNA editing enzyme, catalytic polypeptide-like 3C
ENSG00000143190	3.85	8.21	6.10007E-57	3.23889E-54	POU class 2 homeobox 1
ENSG00000185650	3.85	6.65	9.37316E-39	1.8297E-36	ZFP36 ring finger protein-like 1
ENSG00000179029	3.85	5.92	2.13432E-09	2.93889E-08	transmembrane protein 107
ENSG00000182578	3.85	5.30	2.18089E-46	6.57934E-44	colony stimulating factor 1 receptor
ENSG00000187193	3.85	5.92	6.3196E-35	9.86898E-33	metallothionein 1X
ENSG00000110324	3.83	5.19	3.12021E-45	8.61783E-43	interleukin 10 receptor, alpha
ENSG00000261064	3.83	5.00	7.74958E-42	1.74352E-39	RP11-1000B6.3

ENSG00000166750	3.81	5.89	4.33151E-46	1.2777E-43	schlafen family member 5
ENSG00000116741	3.79	4.63	6.73338E-37	1.19172E-34	regulator of G-protein signaling 2
ENSG00000020633	3.79	7.78	5.59344E-45	1.45583E-42	runt-related transcription factor 3
ENSG00000125726	3.77	4.86	1.5561E-13	4.33032E-12	CD70 molecule
ENSG00000243004	3.71	6.37	4.53854E-18	2.17489E-16	AC005062.2
ENSG00000114268	3.70	6.51	2.24485E-17	9.9327E-16	6-phosphofructo-2-kinase/fructose-2,6-biphosphatase 4
ENSG00000185507	3.67	6.68	3.18121E-45	8.61783E-43	interferon regulatory factor 7
ENSG00000153815	3.63	7.67	2.0782E-54	8.89872E-52	c-Maf inducing protein
ENSG00000204103	3.59	5.01	4.10086E-09	5.37894E-08	v-maf avian musculoaponeurotic fibrosarcoma oncogene homolog B
ENSG00000163633	3.58	5.05	2.86118E-37	5.13234E-35	chromosome 4 open reading frame 36
ENSG00000102524	3.57	8.84	2.42796E-31	3.35717E-29	tumor necrosis factor (ligand) superfamily, member 13b
ENSG00000196739	3.57	5.16	1.89033E-32	2.78803E-30	collagen, type XXVII, alpha 1
ENSG00000259112	3.56	5.23	3.4157E-15	1.16555E-13	NDUFC2-KCTD14 readthrough
ENSG00000088826	3.55	5.62	3.79537E-43	9.5056E-41	spermine oxidase
ENSG00000188313	3.54	7.61	1.43311E-54	6.34104E-52	phospholipid scramblase 1
ENSG00000134326	3.53	5.53	5.08311E-39	1.02232E-36	cytidine monophosphate (UMP-CMP) kinase 2, mitochondrial
ENSG00000186918	3.49	7.86	5.20546E-47	1.60691E-44	zinc finger protein 395
ENSG00000158769	3.47	5.05	1.88984E-36	3.25788E-34	F11 receptor
ENSG00000124762	3.47	5.15	1.41362E-29	1.75368E-27	cyclin-dependent kinase inhibitor 1A (p21, Cip1)
ENSG00000049323	3.47	4.94	6.17733E-11	1.12945E-09	latent transforming growth factor beta binding protein 1
ENSG00000138640	3.46	4.58	6.73363E-31	8.93821E-29	family with sequence similarity 13, member A
ENSG00000275565	3.45	4.21	1.18341E-26	1.24671E-24	arachidonate 5-lipoxygenase
ENSG00000213741	3.42	11.26	1.86784E-24	1.6982E-22	ribosomal protein S29
ENSG00000130402	3.42	11.00	6.90837E-22	4.9302E-20	actinin, alpha 4
ENSG00000251474	3.39	5.58	3.90458E-40	8.22688E-38	ribosomal protein L32 pseudogene 3
ENSG00000177410	3.37	8.48	8.40323E-44	2.14509E-41	ZNFX1 antisense RNA 1
ENSG00000138433	3.32	7.26	9.98061E-42	2.20804E-39	corepressor interacting with RBPJ, 1
ENSG00000267855	3.28	4.84	3.4714E-25	3.33908E-23	NADH dehydrogenase (ubiquinone) 1 alpha subcomplex, 7, 14.5kDa
ENSG00000115165	3.28	5.03	2.81061E-26	2.86984E-24	cytohesin 1 interacting protein
ENSG00000109107	3.25	6.63	6.80002E-22	4.90562E-20	aldolase C, fructose-bisphosphate
ENSG00000072682	3.21	4.72	5.10442E-26	5.13304E-24	prolyl 4-hydroxylase, alpha polypeptide II
ENSG00000138496	3.19	6.67	3.1869E-42	7.42155E-40	poly (ADP-ribose) polymerase family, member 9
ENSG00000111674	3.17	6.20	1.12517E-30	1.45005E-28	enolase 2 (gamma, neuronal)
ENSG00000165029	3.17	4.31	1.18757E-25	1.16769E-23	ATP-binding cassette, sub-family A (ABC1), member 1
ENSG00000101745	3.17	7.63	6.23374E-38	1.18209E-35	ankyrin repeat domain 12
ENSG00000259529	3.15	5.99	7.59612E-39	1.50494E-36	Uncharacterized protein
ENSG00000163823	3.15	6.12	2.64465E-17	1.15858E-15	chemokine (C-C motif) receptor 1
ENSG00000160593	3.13	4.62	2.03445E-11	4.0917E-10	adhesion molecule, interacts with CXADR antigen 1
ENSG00000166012	3.10	9.58	4.11026E-06	2.74583E-05	TATA box binding protein (TBP)-associated factor, RNA polymerase I, D, 41kDa
ENSG00000119547	3.06	8.17	6.9064E-20	4.05644E-18	one cut homeobox 2
ENSG00000160932	3.06	8.18	1.77521E-34	2.70852E-32	lymphocyte antigen 6 complex, locus E
ENSG00000187837	3.05	5.94	1.91335E-26	1.99983E-24	histone cluster 1, H1c
ENSG00000186660	3.04	5.15	9.97434E-11	1.78677E-09	ZFP91 zinc finger protein
ENSG00000171858	3.03	10.39	2.16854E-23	1.81039E-21	ribosomal protein S21
ENSG00000169715	3.01	4.84	2.07767E-25	2.01307E-23	metallothionein 1E
ENSG00000138642	3.01	4.40	2.82404E-23	2.34289E-21	HECT and RLD domain containing E3 ubiquitin protein ligase family member 6
ENSG00000197555	3.00	5.60	4.16865E-35	6.66683E-33	signal-induced proliferation-associated 1 like 1
ENSG00000177409	2.97	6.39	1.02535E-20	6.54354E-19	sterile alpha motif domain containing 9-like
ENSG00000178726	2.95	5.92	5.49068E-35	8.67658E-33	thrombomodulin
ENSG00000133059	2.95	5.60	1.02919E-30	1.35263E-28	dual serine/threonine and tyrosine protein kinase
ENSG00000095066	2.94	7.52	3.63011E-39	7.41323E-37	hook microtubule-tethering protein 2
ENSG00000176171	2.92	7.30	6.16488E-26	6.15283E-24	BCL2/adenovirus E1B 19kDa interacting protein 3
ENSG00000170412	2.92	4.31	5.21803E-15	1.75352E-13	G protein-coupled receptor, class C, group 5, member C
ENSG00000141905	2.92	7.96	8.27445E-23	6.46089E-21	nuclear factor I/C (CCAAT-binding transcription factor)
ENSG00000134597	2.91	6.47	6.43172E-36	1.09455E-33	RNA binding motif protein, X-linked 2
ENSG00000181690	2.89	4.61	5.31759E-25	4.97082E-23	pleiomorphic adenoma gene 1
ENSG00000147872	2.89	8.38	1.32703E-24	1.21483E-22	perilipin 2
ENSG00000267121	2.87	4.86	8.88106E-25	8.24386E-23	CTD-2020K17.1
ENSG00000134955	2.87	5.60	2.71121E-31	3.71016E-29	solute carrier family 37 (glucose-6-phosphate transporter), member 2
ENSG00000173821	2.86	8.82	1.06696E-27	1.22094E-25	ring finger protein 213
ENSG00000153006	2.85	7.35	5.68221E-32	8.28854E-30	SREK1-interacting protein 1
ENSG00000184678	2.85	4.35	2.65258E-18	1.3237E-16	histone cluster 2, H2be
ENSG00000203875	2.84	8.95	1.08847E-30	1.4165E-28	small nucleolar RNA host gene 5
ENSG00000131944	2.84	4.99	4.21269E-25	3.99424E-23	Fanconi anemia core complex associated protein 24
ENSG00000122884	2.84	7.94	8.38096E-36	1.40821E-33	prolyl 4-hydroxylase, alpha polypeptide I
ENSG00000055332	2.84	7.50	1.18699E-37	2.21916E-35	eukaryotic translation initiation factor 2-alpha kinase 2
ENSG00000270316	2.82	4.81	7.22721E-23	5.71035E-21	C10orf32-ASMT readthrough (NMD candidate)
ENSG00000197013	2.81	4.83	7.93287E-22	5.60111E-20	zinc finger protein 429
ENSG00000165071	2.79	4.29	1.58313E-19	8.79267E-18	transmembrane protein 71
ENSG00000270882	2.78	4.34	1.18573E-13	3.3417E-12	histone cluster 2, H4a
ENSG00000177954	2.77	10.90	3.79752E-18	1.83972E-16	ribosomal protein S27
ENSG00000146192	2.76	4.32	1.51263E-17	6.87485E-16	FYVE, RhoGEF and PH domain containing 2

ENSG00000067082	2.75	5.81	9.95132E-27	1.06527E-24	Kruppel-like factor 6
ENSG00000143315	2.74	6.36	5.55563E-31	7.44904E-29	phosphatidylinositol glycan anchor biosynthesis, class M
ENSG00000280755	2.73	6.02	1.73321E-30	2.21217E-28	SP110 nuclear body protein
ENSG00000171766	2.73	4.23	3.16777E-15	1.08654E-13	glycine amidinotransferase (L-arginine:glycine amidinotransferase)
ENSG00000132274	2.73	4.63	4.75193E-09	6.15987E-08	tripartite motif containing 22
ENSG00000118515	2.73	5.96	2.76496E-30	3.46246E-28	serum/glucocorticoid regulated kinase 1
ENSG00000140941	2.72	6.79	3.9334E-35	6.36731E-33	microtubule-associated protein 1 light chain 3 beta
ENSG00000162959	2.70	5.00	3.85316E-22	2.81026E-20	mediator of cell motility 1
ENSG00000169403	2.67	4.97	3.34787E-22	2.45523E-20	platelet-activating factor receptor
ENSG00000205413	2.67	6.26	2.1686E-27	2.4395E-25	sterile alpha motif domain containing 9
ENSG00000008952	2.66	8.93	1.25949E-22	9.66384E-21	SEC62 homolog (S. cerevisiae)
ENSG00000088888	2.65	6.26	2.7402E-22	2.055E-20	mitochondrial antiviral signaling protein
ENSG00000139626	2.65	5.88	9.85521E-10	1.4471E-08	integrin, beta 7
ENSG00000100592	2.64	4.98	3.82051E-21	2.53567E-19	dishevelled associated activator of morphogenesis 1
ENSG00000197358	2.62	5.95	2.96864E-14	9.10064E-13	BCL2/adenovirus E1B 19kDa interacting protein 3 pseudogene 1
ENSG00000113140	2.61	6.16	3.06128E-20	1.8555E-18	secreted protein, acidic, cysteine-rich (osteonectin)
ENSG00000104765	2.58	7.05	3.26246E-18	1.60392E-16	BCL2/adenovirus E1B 19kDa interacting protein 3-like
ENSG00000135916	2.55	7.57	1.89517E-30	2.39585E-28	integral membrane protein 2C
ENSG00000171475	2.55	6.50	1.79754E-29	2.18904E-27	WAS/WASL interacting protein family, member 2
ENSG00000274233	2.55	7.57	1.44318E-31	2.04394E-29	chemokine (C-C motif) ligand 5
ENSG00000163565	2.54	7.62	3.21951E-10	5.23082E-09	interferon, gamma-inducible protein 16
ENSG00000155926	2.54	6.61	4.39511E-27	4.86172E-25	Src-like-adaptor
ENSG00000138172	2.53	4.77	2.58814E-16	1.00297E-14	calcium homeostasis modulator 2
ENSG00000171724	2.53	4.70	1.51089E-18	7.74344E-17	vesicle amine transport 1-like
ENSG00000197063	2.53	8.61	2.20633E-26	2.28803E-24	v-maf avian musculoaponeurotic fibrosarcoma oncogene homolog G
ENSG00000171860	2.52	4.58	0.000210197	0.000899468	complement component 3a receptor 1
ENSG00000108771	2.52	4.98	5.27324E-21	3.4652E-19	DEXH (Asp-Glu-X-His) box polypeptide 58
ENSG00000137815	2.52	8.06	2.07772E-27	2.35724E-25	RTF1 homolog, Paf1/RNA polymerase II complex component
ENSG00000103111	2.52	6.86	1.77961E-29	2.18727E-27	MON1 secretory trafficking family member B
ENSG00000217555	2.51	6.03	2.54726E-26	2.62111E-24	chemokine-like factor
ENSG00000189060	2.49	5.87	1.33774E-25	1.30567E-23	H1 histone family, member 0
ENSG00000165233	2.48	5.42	2.10445E-23	1.77927E-21	chromosome 9 open reading frame 89
ENSG00000278550	2.48	5.60	3.50362E-23	2.83579E-21	solute carrier family 43 (amino acid system L transporter), member 2
ENSG00000076053	2.47	4.25	3.4057E-13	9.04146E-12	RNA binding motif protein 7
ENSG00000138646	2.47	5.88	1.92602E-21	1.29776E-19	HECT and RLD domain containing E3 ubiquitin protein ligase 5
ENSG00000234545	2.44	6.57	7.81288E-24	6.86809E-22	family with sequence similarity 133, member B
ENSG00000135318	2.44	6.91	3.39614E-10	5.47092E-09	5'-nucleotidase, ecto (CD73)
ENSG00000141027	2.44	7.95	1.86294E-28	2.22781E-26	nuclear receptor corepressor 1
ENSG00000104081	2.43	5.44	1.24744E-18	6.41804E-17	Bcl2 modifying factor
ENSG00000139289	2.42	5.00	4.29693E-16	1.59323E-14	pleckstrin homology-like domain, family A, member 1
ENSG00000270898	2.42	6.17	2.14543E-23	1.80243E-21	GPR75-ASB3 readthrough
ENSG00000138061	2.42	6.60	7.40997E-24	6.55733E-22	cytochrome P450, family 1, subfamily B, polypeptide 1
ENSG00000007944	2.41	7.38	2.55896E-27	2.85442E-25	myosin regulatory light chain interacting protein
ENSG00000167851	2.41	5.01	1.72592E-06	1.25602E-05	CD300a molecule
ENSG00000173193	2.41	7.13	2.79211E-22	2.08216E-20	poly (ADP-ribose) polymerase family, member 14
ENSG00000107201	2.40	5.42	6.29358E-22	4.56508E-20	DEAD (Asp-Glu-Ala-Asp) box polypeptide 58
ENSG00000000938	2.39	5.81	2.82389E-10	4.63342E-09	FGR proto-oncogene, Src family tyrosine kinase
ENSG00000125676	2.39	9.23	2.13494E-19	1.1759E-17	THO complex 2
ENSG00000123384	2.38	7.53	3.78729E-16	1.41704E-14	low density lipoprotein receptor-related protein 1
ENSG00000132530	2.38	6.80	1.08681E-08	1.31267E-07	XIAP associated factor 1
ENSG00000142089	2.38	6.52	3.63859E-18	1.76918E-16	interferon induced transmembrane protein 3
ENSG00000158373	2.36	5.01	1.24963E-19	7.0887E-18	histone cluster 1, H2bd
ENSG00000111424	2.36	4.79	5.74517E-19	3.0383E-17	vitamin D (1,25- dihydroxyvitamin D3) receptor
ENSG00000188177	2.35	5.44	6.97895E-19	3.66161E-17	zinc finger CCCH-type containing 6
ENSG00000056558	2.34	4.35	6.1894E-16	2.26331E-14	TNF receptor-associated factor 1
ENSG00000196968	2.34	5.62	1.49156E-20	9.42806E-19	fucosyltransferase 11 (alpha (1,3) fucosyltransferase)
ENSG00000139168	2.33	6.09	1.47699E-19	8.2724E-18	zinc finger CCHC-type and RNA binding motif 1
ENSG00000272886	2.33	6.94	6.23793E-27	6.78707E-25	decapping mRNA 1A
ENSG00000258472	2.33	4.63	4.63766E-09	6.02941E-08	Uncharacterized protein
ENSG00000171992	2.33	4.76	2.06583E-18	1.04664E-16	synaptopodin
ENSG00000162599	2.33	6.03	1.68917E-20	1.05268E-18	nuclear factor I/A
ENSG00000143847	2.33	5.36	1.55144E-11	3.16828E-10	protein tyrosine phosphatase, receptor type, f polypeptide (PTPRF), interacting protein (liprin), alpha 4
ENSG00000216490	2.32	7.34	7.46164E-07	5.99208E-06	interferon, gamma-inducible protein 30
ENSG00000147894	2.32	4.77	2.01328E-17	8.96789E-16	chromosome 9 open reading frame 72
ENSG00000116017	2.31	8.25	3.50421E-25	3.3464E-23	AT rich interactive domain 3A (BRIGHT-like)
ENSG00000146247	2.30	7.98	2.25319E-18	1.13722E-16	pleckstrin homology domain interacting protein
ENSG00000069399	2.30	4.83	3.11269E-17	1.34586E-15	B-cell CLL/lymphoma 3
ENSG00000151461	2.30	6.92	5.72901E-24	5.1383E-22	UPF2 regulator of nonsense transcripts homolog (yeast)
ENSG00000145012	2.29	6.65	4.83444E-25	4.55123E-23	LIM domain containing preferred translocation partner in lipoma
ENSG00000237727	2.29	5.40	1.62717E-22	1.24132E-20	allograft inflammatory factor 1
ENSG00000180573	2.28	6.41	3.54825E-23	2.85451E-21	histone cluster 1, H2ac
ENSG00000188825	2.28	4.34	3.70134E-10	5.94094E-09	long intergenic non-protein coding RNA 910

ENSG00000143546	2.28	9.69	1.74238E-14	5.50181E-13	S100 calcium binding protein A8
ENSG00000101384	2.28	4.45	4.46896E-15	1.50561E-13	jagged 1
ENSG00000271605	2.28	6.11	1.97962E-16	7.82066E-15	mast cell immunoglobulin-like receptor 1
ENSG00000127920	2.27	5.96	1.67383E-17	7.53168E-16	guanine nucleotide binding protein (G protein), gamma 11
ENSG00000183486	2.27	8.07	1.28048E-21	8.80681E-20	MX dynamin-like GTPase 2
ENSG00000135185	2.26	4.63	2.47953E-14	7.69003E-13	transmembrane protein 243, mitochondrial
ENSG00000159388	2.26	5.11	3.06067E-10	4.99721E-09	BTG family, member 2
ENSG00000130208	2.26	5.02	2.7503E-06	1.92246E-05	apolipoprotein C-I
ENSG00000172831	2.26	6.34	7.87611E-23	6.18624E-21	carboxylesterase 2
ENSG00000159140	2.25	9.64	1.50421E-14	4.78822E-13	SON DNA binding protein
ENSG00000197323	2.24	6.97	1.07493E-22	8.34423E-21	tripartite motif containing 33
ENSG00000183354	2.22	5.73	1.54327E-20	9.70874E-19	KIAA2026
ENSG00000145592	2.22	10.72	2.42892E-13	6.56651E-12	ribosomal protein L37
ENSG00000156603	2.22	4.85	6.74899E-17	2.82606E-15	mediator complex subunit 19
ENSG00000169439	2.21	7.02	3.16643E-23	2.59452E-21	syndecan 2
ENSG00000165801	2.21	5.45	1.36506E-19	7.67787E-18	Rho guanine nucleotide exchange factor (GEF) 40
ENSG00000139266	2.20	5.68	1.16088E-20	7.37298E-19	membrane-associated ring finger (C3HC4) 9
ENSG00000182899	2.20	10.16	4.25298E-15	1.43649E-13	ribosomal protein L35a
ENSG00000147443	2.20	5.75	6.77891E-14	1.99078E-12	docking protein 2, 56kDa
ENSG00000101966	2.19	6.86	7.18967E-23	5.71035E-21	X-linked inhibitor of apoptosis, E3 ubiquitin protein ligase
ENSG00000168209	2.19	8.96	0.000559037	0.002112944	DNA-damage-inducible transcript 4
ENSG00000126759	2.18	4.38	3.56771E-13	9.43382E-12	complement factor properdin
ENSG00000196199	2.18	7.42	7.73741E-20	4.52451E-18	M-phase phosphoprotein 8
ENSG00000164096	2.18	7.82	8.56931E-22	5.95544E-20	chromosome 4 open reading frame 3
ENSG00000165355	2.17	5.52	5.38844E-18	2.55451E-16	F-box protein 33
ENSG00000166482	2.17	4.28	4.30586E-05	0.000222397	microfibrillar-associated protein 4
ENSG00000188157	2.16	6.17	1.41877E-21	9.685E-20	agrin
ENSG00000204130	2.16	4.33	1.16429E-13	3.28825E-12	RUN and FYVE domain containing 2
ENSG00000178951	2.15	7.21	4.00831E-21	2.64708E-19	zinc finger and BTB domain containing 7A
ENSG00000058272	2.15	6.59	4.82232E-18	2.29432E-16	protein phosphatase 1, regulatory subunit 12A
ENSG00000170458	2.15	7.05	3.21989E-05	0.000171376	CD14 molecule
ENSG00000275342	2.15	5.41	4.49193E-19	2.414E-17	Tyrosine-protein kinase SgK223
ENSG00000205758	2.14	5.30	1.41714E-17	6.48661E-16	crystallin, zeta (quinone reductase)-like 1
ENSG00000099337	2.14	5.53	2.81675E-18	1.39513E-16	potassium channel, two pore domain subfamily K, member 6
ENSG00000160602	2.13	4.82	1.62703E-16	6.50516E-15	NIMA-related kinase 8
ENSG00000174938	2.13	6.26	2.63013E-12	6.01936E-11	seizure related 6 homolog (mouse)-like 2
ENSG00000104419	2.13	8.95	8.64863E-14	2.48489E-12	N-myc downstream regulated 1
ENSG00000229314	2.13	4.73	0.000410386	0.001611198	orosomucoid 1
ENSG00000059804	2.12	6.19	2.0418E-08	2.2949E-07	solute carrier family 2 (facilitated glucose transporter), member 3
ENSG00000139278	2.11	6.81	1.32834E-19	7.50312E-18	GLI pathogenesis-related 1
ENSG00000157350	2.10	5.63	9.87881E-20	5.65221E-18	ST3 beta-galactoside alpha-2,3-sialyltransferase 2
ENSG00000095303	2.10	5.35	3.35118E-18	1.64146E-16	prostaglandin-endoperoxide synthase 1 (prostaglandin G/H synthase and cyclooxygenase)
ENSG00000146278	2.10	6.81	2.88522E-21	1.93426E-19	proline-rich nuclear receptor coactivator 1
ENSG00000119004	2.10	4.41	5.89083E-14	1.74153E-12	cytochrome P450, family 20, subfamily A, polypeptide 1
ENSG00000121281	2.09	6.03	9.98949E-20	5.69101E-18	adenylate cyclase 7
ENSG00000245209	2.08	4.93	7.44557E-14	2.16738E-12	AP001007.1
ENSG00000204054	2.08	7.36	7.27281E-14	2.12174E-12	long intergenic non-protein coding RNA 963
ENSG00000166595	2.08	6.21	2.30798E-20	1.41834E-18	family with sequence similarity 96, member B
ENSG00000160613	2.07	6.92	1.48497E-19	8.28216E-18	proprotein convertase subtilisin/kexin type 7
ENSG00000103248	2.07	4.70	2.30637E-13	6.27352E-12	methenyltetrahydrofolate synthetase domain containing
ENSG00000213853	2.06	5.14	1.222E-09	1.7555E-08	epithelial membrane protein 2
ENSG00000162645	2.06	4.72	2.58513E-12	5.94715E-11	guanylate binding protein 2, interferon-inducible
ENSG00000135218	2.05	4.33	1.48486E-08	1.72744E-07	CD36 molecule (thrombospondin receptor)
ENSG00000120688	2.05	4.76	1.4445E-13	4.0367E-12	WW domain binding protein 4
ENSG00000059728	2.05	7.24	7.2008E-22	5.11141E-20	MAX dimerization protein 1
ENSG00000196227	2.05	6.05	8.81193E-19	4.6051E-17	family with sequence similarity 217, member B
ENSG00000198863	2.05	5.58	4.87028E-17	2.05232E-15	RUN domain containing 1
ENSG00000136826	2.05	5.46	9.03106E-16	3.25756E-14	Kruppel-like factor 4 (gut)
ENSG00000233927	2.04	9.09	1.04874E-16	4.28338E-15	ribosomal protein S28
ENSG00000179085	2.04	5.12	2.15894E-14	6.77489E-13	dolichyl-phosphate mannosyltransferase polypeptide 3
ENSG00000065989	2.04	4.27	1.0279E-11	2.1831E-10	phosphodiesterase 4A, cAMP-specific
ENSG00000129667	2.03	5.32	2.81022E-15	9.7143E-14	rhomboid 5 homolog 2 (Drosophila)
ENSG00000116977	2.03	7.02	8.34955E-20	4.81878E-18	lectin, galactoside-binding, soluble, 8
ENSG00000242711	2.02	5.74	1.56807E-15	5.56541E-14	proteasome (prosome, macropain) subunit, beta type, 9
ENSG00000135637	2.01	6.57	3.91265E-19	2.11124E-17	coiled-coil domain containing 142
ENSG00000186162	2.01	4.57	6.13488E-14	1.80564E-12	cell death-inducing DFFA-like effector c pseudogene
ENSG00000197956	2.00	9.31	1.38626E-10	2.41803E-09	S100 calcium binding protein A6
ENSG00000273686	2.00	12.53	2.38196E-07	2.1306E-06	beta-2-microglobulin
ENSG00000163536	2.00	4.71	3.93621E-11	7.47486E-10	serpin peptidase inhibitor, clade I (neuroserpin), member 1
ENSG00000116667	2.00	4.79	2.80599E-07	2.45691E-06	chromosome 1 open reading frame 21
ENSG00000163221	2.00	4.94	9.84647E-10	1.4471E-08	S100 calcium binding protein A12
ENSG00000187257	1.99	6.98	3.08537E-18	1.5225E-16	round spermatid basic protein 1-like

ENSG00000235309	1.99	6.62	3.87229E-20	2.31535E-18	major histocompatibility complex, class I, J (pseudogene)
ENSG00000163644	1.99	6.12	4.85283E-11	9.02191E-10	protein phosphatase, Mg2+/Mn2+ dependent, 1K
ENSG00000101336	1.98	6.42	2.2226E-14	6.94182E-13	HCK proto-oncogene, Src family tyrosine kinase
ENSG00000130813	1.98	5.89	2.4746E-16	9.66114E-15	chromosome 19 open reading frame 66
ENSG00000110077	1.98	5.75	1.07316E-13	3.0569E-12	membrane-spanning 4-domains, subfamily A, member 6A
ENSG00000140948	1.98	5.18	1.91314E-16	7.58061E-15	zinc finger, CCHC domain containing 14
ENSG00000143333	1.97	4.95	4.20049E-11	7.93135E-10	regulator of G-protein signaling 16
ENSG00000172809	1.97	10.42	4.20872E-11	7.93558E-10	ribosomal protein L38
ENSG00000167207	1.97	4.77	1.59963E-13	4.43289E-12	nucleotide-binding oligomerization domain containing 2
ENSG00000131503	1.97	8.06	2.38191E-19	1.30113E-17	ankyrin repeat and KH domain containing 1
ENSG00000136770	1.97	6.61	2.73335E-17	1.1857E-15	DnaJ (Hsp40) homolog, subfamily C, member 1
ENSG00000167604	1.97	4.62	2.32881E-13	6.3087E-12	nuclear factor of kappa light polypeptide gene enhancer in B-cells inhibitor, delta
ENSG00000170801	1.96	5.82	2.20805E-07	1.98881E-06	HtrA serine peptidase 3
ENSG00000131067	1.96	5.07	5.48874E-15	1.83984E-13	gamma-glutamyltransferase 7
ENSG00000111981	1.95	4.30	9.30055E-08	9.10438E-07	UL16 binding protein 1
ENSG00000196576	1.94	6.58	9.5067E-15	3.09294E-13	plexin B2
ENSG00000085514	1.93	4.43	1.56995E-12	3.73467E-11	paired immunoglobin-like type 2 receptor alpha
ENSG00000177606	1.93	5.47	9.67006E-06	5.89892E-05	jun proto-oncogene
ENSG00000114023	1.93	6.60	6.87937E-18	3.2041E-16	family with sequence similarity 162, member A
ENSG00000197903	1.92	8.22	2.19644E-07	1.98067E-06	histone cluster 1, H2bk
ENSG00000053254	1.92	7.04	3.57446E-17	1.53056E-15	forkhead box N3
ENSG00000106479	1.92	5.22	9.25361E-16	3.32879E-14	zinc finger protein 862
ENSG00000131469	1.92	10.24	2.95992E-11	5.761E-10	ribosomal protein L27
ENSG00000148180	1.92	4.82	3.00682E-13	8.03069E-12	gelsolin
ENSG00000176788	1.91	8.37	2.67593E-17	1.1646E-15	brain abundant, membrane attached signal protein 1
ENSG00000112343	1.91	6.43	1.0637E-16	4.33113E-15	tripartite motif containing 38
ENSG00000234741	1.91	8.84	3.78973E-16	1.41704E-14	growth arrest-specific 5 (non-protein coding)
ENSG00000125657	1.91	4.24	6.47963E-06	4.11731E-05	tumor necrosis factor (ligand) superfamily, member 9
ENSG00000051523	1.90	8.82	7.68454E-15	2.53113E-13	cytochrome b-245, alpha polypeptide
ENSG00000197879	1.90	5.63	1.98778E-16	7.82962E-15	myosin IC
ENSG00000257529	1.89	8.18	4.67886E-17	1.97794E-15	RPL36A-HNRNPH2 readthrough
ENSG00000110628	1.89	6.06	2.31037E-15	8.09177E-14	solute carrier family 22, member 18
ENSG00000130303	1.89	8.59	9.91333E-17	4.07398E-15	bone marrow stromal cell antigen 2
ENSG00000196369	1.88	4.76	1.36605E-11	2.82885E-10	SLIT-ROBO Rho GTPase activating protein 2B
ENSG00000156482	1.88	10.76	3.73863E-10	5.98632E-09	ribosomal protein L30
ENSG00000071082	1.88	10.73	5.01063E-10	7.83404E-09	ribosomal protein L31
ENSG00000089335	1.87	6.62	7.87779E-15	2.58197E-13	zinc finger protein 302
ENSG00000060339	1.87	8.66	4.18576E-12	9.32245E-11	cell division cycle and apoptosis regulator 1
ENSG00000115415	1.87	8.21	1.3818E-15	4.91742E-14	signal transducer and activator of transcription 1, 91kDa
ENSG00000122694	1.87	6.55	6.13971E-12	1.34931E-10	GLI pathogenesis-related 2
ENSG00000090863	1.87	7.58	7.17341E-15	2.37456E-13	golgi glycoprotein 1
ENSG00000115267	1.86	5.69	7.34407E-15	2.425E-13	interferon induced with helicase C domain 1
ENSG00000160445	1.86	5.07	4.15184E-12	9.26245E-11	zyg-11 related, cell cycle regulator
ENSG00000196209	1.86	4.67	1.1279E-11	2.38025E-10	signal-regulatory protein beta 2
ENSG00000073803	1.86	4.59	7.40391E-12	1.6085E-10	mitogen-activated protein kinase kinase kinase 13
ENSG00000125733	1.85	5.61	5.69559E-16	2.08849E-14	thyroid hormone receptor interactor 10
ENSG00000166913	1.85	10.01	1.76161E-11	3.57002E-10	tyrosine 3-monooxygenase/tryptophan 5-monooxygenase activation protein, beta
ENSG00000204164	1.85	5.14	3.51707E-11	6.75623E-10	BMS1P5
ENSG00000114480	1.85	7.68	4.04225E-16	1.50299E-14	glucan (1,4-alpha-), branching enzyme 1
ENSG00000169925	1.84	6.05	5.7144E-15	1.90108E-13	bromodomain containing 3
ENSG00000152256	1.84	4.85	9.94849E-10	1.45918E-08	pyruvate dehydrogenase kinase, isozyme 1
ENSG00000100918	1.84	4.75	1.92494E-07	1.75734E-06	REC8 meiotic recombination protein
ENSG00000130775	1.84	6.36	3.16322E-16	1.20311E-14	thymocyte selection associated family member 2
ENSG00000106404	1.84	5.73	1.53048E-14	4.86018E-13	claudin 15
ENSG00000168461	1.83	6.49	1.42555E-16	5.7516E-15	RAB31, member RAS oncogene family
ENSG00000141506	1.83	4.68	1.37848E-11	2.85014E-10	phosphoinositide-3-kinase, regulatory subunit 5
ENSG00000034713	1.83	5.85	5.68249E-15	1.89521E-13	GABA(A) receptor-associated protein like 2
ENSG00000002549	1.83	7.68	3.22916E-17	1.39169E-15	leucine aminopeptidase 3
ENSG00000120594	1.83	5.16	1.14334E-10	2.02897E-09	plexin domain containing 2
ENSG00000174720	1.83	7.38	1.25035E-15	4.46159E-14	La ribonucleoprotein domain family, member 7
ENSG00000139182	1.82	5.42	1.78507E-15	6.31868E-14	calsyntenin 3
ENSG00000068697	1.82	7.17	6.64549E-16	2.41677E-14	lysosomal protein transmembrane 4 alpha
ENSG00000183401	1.82	5.83	9.79666E-16	3.51462E-14	coiled-coil domain containing 159
ENSG00000069329	1.81	7.33	4.34839E-18	2.09893E-16	VPS35 retromer complex component
ENSG00000121858	1.80	5.53	1.16864E-12	2.85156E-11	tumor necrosis factor (ligand) superfamily, member 10
ENSG00000281490	1.80	4.69	5.00117E-11	9.25878E-10	capicua transcriptional repressor pseudogene 14
ENSG00000066933	1.79	5.32	3.7216E-14	1.12787E-12	myosin IXA
ENSG00000137628	1.79	5.61	2.46984E-10	4.12386E-09	DEAD (Asp-Glu-Ala-Asp) box polypeptide 60
ENSG00000105939	1.79	7.49	4.37179E-14	1.3159E-12	zinc finger CCCH-type, antiviral 1
ENSG00000121310	1.79	6.42	5.55544E-15	1.8575E-13	enoyl CoA hydratase domain containing 2
ENSG00000085117	1.78	8.46	1.60489E-10	2.77386E-09	CD82 molecule
ENSG00000141096	1.78	7.74	1.41381E-10	2.46284E-09	dipeptidase 3

ENSG00000142156	1.78	4.59	7.59678E-05	0.000367492	collagen, type VI, alpha 1
ENSG00000197756	1.77	10.70	2.29111E-09	3.14176E-08	ribosomal protein L37a
ENSG00000160352	1.77	4.87	2.12193E-09	2.92487E-08	zinc finger protein 714
ENSG00000204138	1.76	6.80	4.94596E-16	1.82369E-14	phosphatase and actin regulator 4
ENSG00000136819	1.76	7.98	2.656E-15	9.22926E-14	chromosome 9 open reading frame 78
ENSG00000153029	1.76	6.71	1.3753E-16	5.56578E-15	major histocompatibility complex, class I-related
ENSG00000149187	1.75	8.57	1.74496E-14	5.50181E-13	CUGBP, Elav-like family member 1
ENSG00000166888	1.74	9.40	8.52167E-12	1.83037E-10	signal transducer and activator of transcription 6, interleukin-4 induced
ENSG00000214176	1.74	4.86	1.28094E-11	2.65675E-10	pleckstrin homology domain containing, family M (with RUN domain) member 1 pseudogene
ENSG00000175061	1.74	9.24	3.20332E-11	6.19837E-10	LRRC75A antisense RNA 1
ENSG00000170525	1.73	5.60	4.98846E-13	1.28327E-11	6-phosphofructo-2-kinase/fructose-2,6-biphosphatase 3
ENSG00000121289	1.73	4.29	3.32769E-09	4.43492E-08	centrosomal protein 89kDa
ENSG00000077380	1.73	7.78	8.28611E-16	3.00519E-14	dynein, cytoplasmic 1, intermediate chain 2
ENSG00000133112	1.73	11.92	3.68083E-07	3.14613E-06	tumor protein, translationally-controlled 1
ENSG00000178695	1.72	6.18	2.0075E-12	4.69146E-11	potassium channel tetramerization domain containing 12
ENSG00000131669	1.72	4.72	1.30211E-10	2.28628E-09	ninjurin 1
ENSG00000143771	1.72	7.08	7.83876E-14	2.26692E-12	cornichon family AMPA receptor auxiliary protein 4
ENSG00000158270	1.72	4.83	0.001313284	0.004444808	collectin sub-family member 12
ENSG00000109390	1.72	6.72	2.43069E-15	8.46851E-14	NADH dehydrogenase (ubiquinone) 1, subcomplex unknown, 1, 6kDa
ENSG00000276701	1.71	7.14	2.74652E-16	1.05368E-14	macrophage migration inhibitory factor (glycosylation-inhibiting factor)
ENSG00000105639	1.71	5.03	4.46763E-11	8.36436E-10	Janus kinase 3
ENSG00000185432	1.71	5.83	0.000161856	0.000717113	methyltransferase like 7A
ENSG00000160991	1.70	6.42	4.58282E-11	8.55589E-10	ORAI calcium release-activated calcium modulator 2
ENSG00000128524	1.70	8.25	4.67728E-14	1.40149E-12	ATPase, H+ transporting, lysosomal 14kDa, V1 subunit F
ENSG00000110002	1.70	5.76	0.001049471	0.003656347	von Willebrand factor A domain containing 5A
ENSG00000163171	1.69	8.35	1.01202E-09	1.48274E-08	CDC42 effector protein (Rho GTPase binding) 3
ENSG00000166803	1.69	7.40	3.26262E-10	5.28791E-09	KIAA0101
ENSG00000143891	1.68	5.19	9.87016E-06	6.00442E-05	galactose mutarotase (aldose 1-epimerase)
ENSG00000008988	1.68	10.88	2.35498E-08	2.60935E-07	ribosomal protein S20
ENSG00000162735	1.68	6.25	1.07715E-07	1.04137E-06	peroxisomal biogenesis factor 19
ENSG00000102921	1.68	5.33	4.67031E-13	1.20845E-11	NEDD4 binding protein 1
ENSG00000068489	1.68	5.18	1.73505E-09	2.4449E-08	proline rich 11
ENSG00000212338	1.68	6.37	1.50889E-13	4.20777E-12	Small nucleolar RNA SNORA67
ENSG00000135046	1.68	7.84	1.3403E-14	4.28701E-13	annexin A1
ENSG00000070190	1.68	5.07	8.97573E-11	1.61223E-09	dual adaptor of phosphotyrosine and 3-phosphoinositides
ENSG00000083444	1.67	7.99	7.79041E-13	1.94015E-11	procollagen-lysine, 2-oxoglutarate 5-dioxygenase 1
ENSG00000232388	1.67	5.19	3.86346E-12	8.66276E-11	long intergenic non-protein coding RNA 493
ENSG00000103066	1.66	5.05	1.03162E-10	1.84236E-09	phospholipase A2, group XV
ENSG00000112715	1.66	8.11	0.00024504	0.001030302	vascular endothelial growth factor A
ENSG00000101040	1.66	6.68	1.07265E-08	1.29912E-07	zinc finger, MYND-type containing 8
ENSG0000113811	1.66	5.94	3.13434E-13	8.33773E-12	selenoprotein K
ENSG00000185068	1.65	5.73	1.52122E-12	3.63178E-11	GTF2H5
ENSG00000186468	1.65	10.27	2.00113E-08	2.25493E-07	ribosomal protein S23
ENSG00000164733	1.64	10.13	4.73642E-07	3.96164E-06	cathepsin B
ENSG00000127663	1.64	6.94	4.93494E-14	1.47205E-12	lysine (K)-specific demethylase 4B
ENSG00000223960	1.64	5.10	7.84313E-07	6.26789E-06	AC009948.5
ENSG00000105647	1.64	6.84	1.84931E-06	1.33629E-05	phosphoinositide-3-kinase, regulatory subunit 2 (beta)
ENSG00000162909	1.64	7.13	3.62787E-15	1.23478E-13	calpain 2, (m/II) large subunit
ENSG00000164687	1.63	8.08	0.001207109	0.004126493	fatty acid binding protein 5 (psoriasis-associated)
ENSG00000254635	1.63	5.46	1.21774E-11	2.54156E-10	WAC antisense RNA 1 (head to head)
ENSG00000198918	1.63	9.79	5.19088E-10	8.10632E-09	ribosomal protein L39
ENSG00000174428	1.63	4.94	1.62453E-08	1.86379E-07	GTF2I repeat domain containing 2B
ENSG00000172530	1.63	4.52	4.56431E-09	5.9457E-08	BTG3 associated nuclear protein
ENSG00000137145	1.63	5.40	1.4512E-11	2.98655E-10	DENN/MADD domain containing 4C
ENSG00000100439	1.63	4.42	1.94732E-08	2.19989E-07	abhydrolase domain containing 4
ENSG00000132256	1.63	5.20	8.09043E-12	1.74622E-10	tripartite motif containing 5
ENSG00000265972	1.62	9.04	2.95208E-11	5.75418E-10	thioredoxin interacting protein
ENSG00000245910	1.62	7.28	1.08129E-14	3.49221E-13	small nucleolar RNA host gene 6
ENSG00000067066	1.62	6.31	1.51522E-12	3.62396E-11	SP100 nuclear antigen
ENSG00000166681	1.62	5.40	2.18615E-05	0.000121571	nerve growth factor receptor (TNFRSF16) associated protein 1
ENSG00000117475	1.61	4.73	9.5875E-10	1.41562E-08	basic leucine zipper nuclear factor 1
ENSG00000125691	1.61	10.19	7.22333E-09	8.97777E-08	ribosomal protein L23
ENSG00000168172	1.61	6.79	2.76977E-09	3.7478E-08	hook microtubule-tethering protein 3
ENSG00000010278	1.61	5.58	7.8664E-06	4.90458E-05	CD9 molecule
ENSG00000173214	1.61	5.10	9.70054E-11	1.74007E-09	KIAA1919
ENSG00000126860	1.60	6.12	2.87754E-12	6.54048E-11	ecotropic viral integration site 2A
ENSG00000167419	1.60	4.79	8.5671E-06	5.29421E-05	lactoperoxidase
ENSG00000118263	1.60	4.22	5.18774E-07	4.29583E-06	Kruppel-like factor 7 (ubiquitous)
ENSG00000235823	1.60	4.81	1.3213E-05	7.76746E-05	oligodendrocyte maturation-associated long intergenic non-coding RNA
ENSG00000111859	1.60	5.15	1.01975E-10	1.82429E-09	neural precursor cell expressed, developmentally down-regulated 9
ENSG00000089041	1.60	5.02	8.89452E-06	5.47108E-05	purinergic receptor P2X, ligand gated ion channel, 7
ENSG00000125810	1.60	8.53	6.43459E-12	1.41178E-10	CD93 molecule

ENSG00000113494	1.59	5.09	8.21423E-10	1.23344E-08	prolactin receptor
ENSG00000163993	1.59	6.22	2.84226E-05	0.000153429	S100 calcium binding protein P
ENSG00000124831	1.59	8.28	3.39556E-10	5.47092E-09	leucine rich repeat (in FLII) interacting protein 1
ENSG00000170581	1.59	7.80	1.53299E-09	2.16939E-08	signal transducer and activator of transcription 2, 113kDa
ENSG00000056972	1.59	4.66	4.23071E-09	5.54377E-08	TRAF3 interacting protein 2
ENSG00000218739	1.59	5.02	6.82418E-10	1.0424E-08	CEBPZ opposite strand
ENSG00000204272	1.58	4.66	3.05576E-09	4.09305E-08	RP11-622K12.1
ENSG00000176845	1.58	5.89	7.7017E-12	1.66774E-10	meteorin, glial cell differentiation regulator-like
ENSG00000185880	1.58	4.70	2.08774E-07	1.89294E-06	tripartite motif containing 69
ENSG00000156587	1.58	6.29	1.92096E-10	3.27748E-09	ubiquitin-conjugating enzyme E2L 6
ENSG00000101347	1.58	8.70	3.50219E-10	5.63492E-09	SAM domain and HD domain 1
ENSG00000159792	1.58	4.90	5.93627E-05	0.000295788	protein serine kinase H1
ENSG00000103995	1.58	5.96	1.44143E-12	3.45995E-11	centrosomal protein 152kDa
ENSG00000226710	1.57	7.92	8.17519E-10	1.22896E-08	chromosome 6 open reading frame 48
ENSG00000025708	1.57	6.35	7.04702E-07	5.68939E-06	thymidine phosphorylase
ENSG00000115548	1.57	6.72	5.51853E-13	1.40601E-11	lysine (K)-specific demethylase 3A
ENSG00000070214	1.57	7.91	3.88354E-12	8.68911E-11	solute carrier family 44 (choline transporter), member 1
ENSG00000188243	1.56	7.50	4.37008E-13	1.13979E-11	COMM domain containing 6
ENSG00000132024	1.56	6.19	2.37946E-12	5.49304E-11	coiled-coil and C2 domain containing 1A
ENSG00000168056	1.56	4.78	2.42308E-08	2.67587E-07	latent transforming growth factor beta binding protein 3
ENSG00000125482	1.56	5.35	2.30758E-11	4.60614E-10	transcription termination factor, RNA polymerase I
ENSG00000109099	1.56	4.99	2.27493E-05	0.000125875	peripheral myelin protein 22
ENSG00000130066	1.55	7.25	7.60817E-12	1.65018E-10	spermidine/spermine N1-acetyltransferase 1
ENSG00000171617	1.55	5.75	2.72428E-11	5.32578E-10	ectodermal-neural cortex 1 (with BTB domain)
ENSG00000138814	1.55	7.20	4.37059E-13	1.13979E-11	protein phosphatase 3, catalytic subunit, alpha isozyme
ENSG00000148660	1.54	6.65	1.25862E-12	3.05428E-11	calcium/calmodulin-dependent protein kinase II gamma
ENSG00000168528	1.54	7.29	1.58276E-13	4.39532E-12	serine incorporator 2
ENSG00000138398	1.54	7.68	5.08329E-12	1.12272E-10	peptidylprolyl isomerase G (cyclophilin G)
ENSG00000111596	1.53	6.79	1.73328E-12	4.08659E-11	CCR4-NOT transcription complex, subunit 2
ENSG00000135686	1.53	5.31	3.55848E-11	6.81606E-10	kelch-like family member 36
ENSG00000081189	1.53	5.14	1.19091E-05	7.0984E-05	myocyte enhancer factor 2C
ENSG00000134419	1.53	9.98	1.42405E-08	1.66707E-07	ribosomal protein S15a
ENSG00000251562	1.52	5.57	2.98897E-11	5.80902E-10	metastasis associated lung adenocarcinoma transcript 1 (non-protein coding)
ENSG00000089159	1.52	4.66	7.75149E-09	9.59826E-08	paxillin
ENSG00000141012	1.52	5.57	6.56019E-11	1.19615E-09	galactosamine (N-acetyl)-6-sulfatase
ENSG00000104998	1.52	4.92	1.88374E-08	2.13169E-07	interleukin 27 receptor, alpha
ENSG00000164609	1.52	6.35	1.04999E-11	2.21935E-10	SLU7 splicing factor homolog (S. cerevisiae)
ENSG00000243749	1.51	5.16	5.85617E-05	0.000292446	ZMYM6 neighbor
ENSG00000187243	1.51	4.69	1.2469E-08	1.48044E-07	melanoma antigen family D4B
ENSG00000146425	1.51	5.59	1.30887E-09	1.87422E-08	dynein, light chain, Tctex-type 1
ENSG00000103121	1.51	5.09	2.73624E-09	3.71379E-08	C-x(9)-C motif containing 2
ENSG00000134138	1.50	5.12	5.7074E-09	7.23592E-08	Meis homeobox 2
ENSG00000265148	1.50	4.99	1.37411E-09	1.96129E-08	BZRAP1 antisense RNA 1
ENSG00000165030	1.49	5.54	4.2398E-11	7.98285E-10	nuclear factor, interleukin 3 regulated
ENSG00000121060	1.49	7.70	2.13021E-12	4.95209E-11	tripartite motif containing 25
ENSG00000103018	1.49	6.98	3.8883E-12	8.68911E-11	cytochrome b5 type B (outer mitochondrial membrane)
ENSG00000163840	1.49	6.40	3.75896E-11	7.14848E-10	deltex 3 like, E3 ubiquitin ligase
ENSG00000151366	1.49	8.00	1.8725E-12	4.39144E-11	NADH dehydrogenase (ubiquinone) 1, subcomplex unknown, 2, 14.5kDa
ENSG00000132432	1.49	6.76	8.74474E-12	1.87525E-10	Sec61 gamma subunit
ENSG00000106771	1.49	6.62	1.07673E-08	1.30287E-07	transmembrane protein 245
ENSG00000160691	1.48	6.85	6.6962E-12	1.46434E-10	SHC (Src homology 2 domain containing) transforming protein 1
ENSG00000099977	1.48	5.76	1.82362E-11	3.68444E-10	D-dopachrome tautomerase
ENSG00000181523	1.48	4.91	6.7799E-07	5.50032E-06	N-sulfoglucosamine sulfohydrolase
ENSG00000011422	1.48	6.26	2.75859E-09	3.73788E-08	plasminogen activator, urokinase receptor
ENSG00000100055	1.48	4.89	7.27853E-06	4.57675E-05	cytohesin 4
ENSG00000164125	1.48	4.76	7.94448E-05	0.000381945	family with sequence similarity 198, member B
ENSG00000039523	1.48	5.15	4.75713E-10	7.45526E-09	family with sequence similarity 65, member A
ENSG00000142534	1.48	10.95	1.42843E-06	1.06283E-05	ribosomal protein S11
ENSG00000188021	1.48	5.66	2.72997E-10	4.50717E-09	ubiquilin 2
ENSG00000131446	1.48	7.62	2.94106E-09	3.95137E-08	mannosyl (alpha-1,3-)-glycoprotein beta-1,2-N-acetylglucosaminyltransferase
ENSG00000134825	1.47	7.51	1.54955E-11	3.16828E-10	transmembrane protein 258
ENSG00000106948	1.47	7.26	1.8705E-12	4.39144E-11	AT-hook transcription factor
ENSG00000187098	1.47	4.55	2.77147E-07	2.42829E-06	microphthalmia-associated transcription factor
ENSG00000257923	1.47	6.69	1.80384E-10	3.10157E-09	cut-like homeobox 1
ENSG00000118181	1.47	10.18	1.61044E-07	1.50119E-06	ribosomal protein S25
ENSG00000228474	1.47	7.55	1.83623E-12	4.32166E-11	oligosaccharyltransferase complex subunit 4 (non-catalytic)
ENSG00000104731	1.47	5.67	2.67426E-10	4.42069E-09	kelch domain containing 4
ENSG00000177981	1.47	5.33	1.21684E-09	1.74998E-08	ankyrin repeat and SOCS box containing 8
ENSG00000067057	1.46	6.35	4.32841E-11	8.12664E-10	phosphofructokinase, platelet
ENSG00000118267	1.46	5.74	5.21716E-11	9.63179E-10	ZNF271
ENSG00000143226	1.45	5.81	8.68524E-07	6.86237E-06	Fc fragment of IgG, low affinity IIa, receptor (CD32)
ENSG00000172936	1.45	7.30	1.18129E-11	2.48502E-10	myeloid differentiation primary response 88

ENSG00000069966	1.45	4.99	3.4571E-08	3.71274E-07	guanine nucleotide binding protein (G protein), beta 5
ENSG00000014914	1.45	4.92	3.92386E-07	3.33239E-06	myotubularin related protein 11
ENSG00000078124	1.44	7.01	5.69527E-12	1.25372E-10	alkaline ceramidase 3
ENSG00000132823	1.44	5.52	4.66877E-10	7.34281E-09	oxidative stress responsive serine-rich 1
ENSG00000123360	1.44	5.11	1.88656E-09	2.63879E-08	phosphodiesterase 1B, calmodulin-dependent
ENSG00000027697	1.44	7.56	1.19843E-11	2.51533E-10	interferon gamma receptor 1
ENSG00000119801	1.44	5.23	5.95197E-10	9.19749E-09	yippee-like 5
ENSG00000134107	1.44	7.32	6.31818E-10	9.70688E-09	basic helix-loop-helix family, member e40
ENSG00000104921	1.44	6.17	4.46819E-07	3.75384E-06	Fc fragment of IgE, low affinity II, receptor for (CD23)
ENSG00000028137	1.43	7.49	1.88183E-11	3.79626E-10	tumor necrosis factor receptor superfamily, member 1B
ENSG00000105355	1.43	8.13	2.49273E-11	4.92388E-10	perilipin 3
ENSG00000109475	1.43	9.76	1.18772E-07	1.13997E-06	ribosomal protein L34
ENSG00000255439	1.43	4.87	5.3073E-08	5.48242E-07	Uncharacterized protein
ENSG00000137575	1.43	8.34	7.29796E-11	1.32521E-09	syndecan binding protein (syntenin)
ENSG00000168273	1.43	4.40	9.85757E-08	9.59306E-07	small integral membrane protein 4
ENSG00000086065	1.43	6.77	4.1832E-11	7.90995E-10	charged multivesicular body protein 5
ENSG00000205542	1.43	12.33	4.92893E-05	0.000251351	thymosin beta 4, X-linked
ENSG00000105193	1.42	10.17	4.0735E-07	3.44405E-06	ribosomal protein S16
ENSG00000163220	1.42	10.12	9.80832E-06	5.96954E-05	S100 calcium binding protein A9
ENSG00000168404	1.42	5.65	2.78525E-10	4.58134E-09	mixed lineage kinase domain-like
ENSG00000085719	1.42	4.69	2.33695E-07	2.09458E-06	copine III
ENSG00000157895	1.41	4.80	7.66089E-08	7.698E-07	chromosome 12 open reading frame 43
ENSG00000140199	1.41	4.42	2.02477E-07	1.83961E-06	solute carrier family 12 (potassium/chloride transporter), member 6
ENSG00000137818	1.41	11.23	5.47636E-06	3.5391E-05	ribosomal protein, large, P1
ENSG00000272657	1.41	4.24	4.41232E-07	3.70925E-06	AP000320.7
ENSG00000164587	1.41	10.05	6.23891E-07	5.1089E-06	ribosomal protein S14
ENSG00000131981	1.41	4.83	2.72244E-08	2.97429E-07	lectin, galactoside-binding, soluble, 3
ENSG00000254995	1.41	4.64	9.68052E-08	9.44153E-07	STX16-NPEPL1 readthrough (NMD candidate)
ENSG00000095951	1.41	4.71	8.75641E-08	8.64826E-07	human immunodeficiency virus type I enhancer binding protein 1
ENSG00000133816	1.41	4.79	4.63534E-08	4.83722E-07	microtubule associated monooxygenase, calponin and LIM domain containing 2
ENSG00000144802	1.41	5.29	5.77981E-08	5.92899E-07	nuclear factor of kappa light polypeptide gene enhancer in B-cells inhibitor, zeta
ENSG00000270106	1.40	4.25	6.88552E-07	5.56968E-06	TSNAX-DISC1 readthrough (NMD candidate)
ENSG00000196123	1.40	5.04	1.15959E-08	1.3867E-07	KIAA0895-like
ENSG00000115561	1.40	7.07	1.32254E-10	2.31602E-09	charged multivesicular body protein 3
ENSG00000266714	1.40	7.40	3.4447E-09	4.57249E-08	myosin XVB
ENSG00000101132	1.40	5.13	1.90991E-07	1.74722E-06	prefoldin subunit 4
ENSG00000124370	1.40	4.60	7.85653E-08	7.86482E-07	methylmalonyl CoA epimerase
ENSG00000156052	1.40	8.24	2.84444E-10	4.66137E-09	guanine nucleotide binding protein (G protein), q polypeptide
ENSG00000119950	1.39	5.06	5.95135E-07	4.88548E-06	MAX interactor 1, dimerization protein
ENSG00000163319	1.39	6.60	1.15599E-10	2.04867E-09	mitochondrial ribosomal protein S18C
ENSG00000161970	1.39	9.39	3.72845E-08	3.96884E-07	ribosomal protein L26
ENSG00000197747	1.39	7.52	5.76146E-11	1.05778E-09	S100 calcium binding protein A10
ENSG00000186318	1.39	5.08	4.42517E-08	4.64346E-07	beta-site APP-cleaving enzyme 1
ENSG00000204364	1.39	5.62	4.67759E-05	0.000240102	complement component 2
ENSG00000158006	1.39	4.65	1.75995E-07	1.62572E-06	platelet-activating factor acetylhydrolase 2, 40kDa
ENSG00000088970	1.39	4.64	2.32554E-07	2.08576E-06	kizuna centrosomal protein
ENSG00000114737	1.38	5.90	1.91421E-07	1.74874E-06	cytokine inducible SH2-containing protein
ENSG00000115738	1.38	7.94	1.35169E-10	2.36083E-09	inhibitor of DNA binding 2, dominant negative helix-loop-helix protein
ENSG00000257341	1.38	4.81	6.44822E-05	0.000318429	cysteine-rich protein 1 (intestinal)
ENSG00000160710	1.38	8.79	2.679E-09	3.63982E-08	adenosine deaminase, RNA-specific
ENSG00000142347	1.38	9.51	3.72116E-08	3.96426E-07	myosin IF
ENSG00000121552	1.38	7.86	3.38684E-10	5.46921E-09	cystatin A (stefin A)
ENSG00000198258	1.38	7.90	1.1771E-10	2.0833E-09	ubiquitin-like 5
ENSG00000136816	1.38	5.95	1.43925E-08	1.68174E-07	torsin family 1, member B (torsin B)
ENSG00000243147	1.37	7.86	9.4719E-09	1.15561E-07	mitochondrial ribosomal protein L33
ENSG00000119720	1.37	5.47	1.36231E-08	1.60597E-07	NRDE-2, necessary for RNA interference, domain containing
ENSG00000115866	1.37	8.17	2.23782E-10	3.77445E-09	aspartyl-tRNA synthetase
ENSG00000276276	1.37	4.34	3.02521E-06	2.08931E-05	ADP-ribosylation factor-like 17B
ENSG00000224152	1.36	4.52	6.47212E-07	5.27708E-06	AC009506.1
ENSG00000143553	1.36	5.57	2.78976E-09	3.771E-08	SNAP-associated protein
ENSG00000124172	1.36	8.31	5.63476E-10	8.7583E-09	ATP synthase, H+ transporting, mitochondrial F1 complex, epsilon subunit
ENSG00000105374	1.36	8.50	7.90748E-09	9.78228E-08	natural killer cell granule protein 7
ENSG00000186174	1.36	4.42	2.36264E-06	1.67441E-05	B-cell CLL/lymphoma 9-like
ENSG00000169860	1.36	6.09	1.9709E-09	2.73372E-08	purinergic receptor P2Y, G-protein coupled, 1
ENSG00000159399	1.36	7.72	8.52735E-08	8.44086E-07	hexokinase 2
ENSG00000113810	1.36	9.87	3.93074E-07	3.33611E-06	structural maintenance of chromosomes 4
ENSG00000122026	1.36	11.82	4.86501E-05	0.000248473	ribosomal protein L21
ENSG00000189043	1.36	7.89	5.15498E-09	6.59221E-08	NDUFA4, mitochondrial complex associated
ENSG00000143382	1.36	6.21	8.64235E-10	1.28897E-08	ADAMTS-like 4
ENSG00000167461	1.36	7.08	3.6573E-09	4.83536E-08	RAB8A, member RAS oncogene family
ENSG00000214022	1.36	5.77	0.000239189	0.001009218	replication initiator 1
ENSG00000185722	1.36	4.77	1.23874E-07	1.18442E-06	ankyrin repeat and FYVE domain containing 1

ENSG00000090339	1.35	4.57	2.07624E-07	1.8838E-06	intercellular adhesion molecule 1
ENSG00000166147	1.35	4.92	0.000705924	0.002598818	fibrillin 1
ENSG00000117523	1.35	8.94	9.82113E-09	1.19382E-07	proline-rich coiled-coil 2C
ENSG00000169756	1.35	7.15	2.17529E-10	3.67364E-09	LIM and senescent cell antigen-like domains 1
ENSG00000177917	1.35	6.43	5.2784E-09	6.72413E-08	ADP-ribosylation factor-like 6 interacting protein 6
ENSG00000143545	1.35	6.19	1.33936E-08	1.5824E-07	RAB13, member RAS oncogene family
ENSG00000100097	1.35	9.03	3.83918E-08	4.0769E-07	lectin, galactoside-binding, soluble, 1
ENSG00000237945	1.35	4.72	6.54381E-05	0.000322909	long intergenic non-protein coding RNA 649
ENSG00000111640	1.35	12.00	7.05425E-05	0.000344385	glyceraldehyde-3-phosphate dehydrogenase
ENSG00000186812	1.34	4.50	4.40977E-07	3.70925E-06	zinc finger protein 397
ENSG00000065518	1.34	7.27	1.21414E-10	2.14315E-09	NADH dehydrogenase (ubiquinone) 1 beta subcomplex, 4, 15kDa
ENSG00000168275	1.34	6.21	8.69769E-10	1.29432E-08	cytochrome c oxidase assembly factor 6
ENSG00000125977	1.34	8.33	9.87437E-08	9.59534E-07	eukaryotic translation initiation factor 2, subunit 2 beta, 38kDa
ENSG00000144791	1.34	6.58	6.86255E-10	1.04705E-08	LIM domains containing 1
ENSG00000206573	1.34	5.15	3.1164E-08	3.36866E-07	THUMPD3 antisense RNA 1
ENSG00000240344	1.33	5.48	3.74416E-08	3.97918E-07	peptidylprolyl isomerase (cyclophilin)-like 3
ENSG00000188559	1.33	5.54	5.24876E-07	4.33824E-06	Ral GTPase activating protein, alpha subunit 2 (catalytic)
ENSG00000175482	1.33	4.89	2.50014E-07	2.22134E-06	polymerase (DNA-directed), delta 4, accessory subunit
ENSG00000134970	1.33	4.56	0.000587658	0.002209167	transmembrane emp24 protein transport domain containing 7
ENSG00000131507	1.33	4.98	0.000194188	0.000839424	Nedd4 family interacting protein 1
ENSG00000231500	1.33	11.22	5.25556E-05	0.000265964	ribosomal protein S18
ENSG00000101608	1.33	9.57	2.41426E-07	2.15369E-06	myosin, light chain 12A, regulatory, non-sarcomeric
ENSG00000187210	1.33	5.80	5.07599E-09	6.51002E-08	glucosaminyl (N-acetyl) transferase 1, core 2
ENSG00000180964	1.33	5.35	3.25472E-08	3.50107E-07	transcription elongation factor A (SII)-like 8
ENSG00000118260	1.32	5.59	6.19779E-09	7.82773E-08	cAMP responsive element binding protein 1
ENSG00000105829	1.32	5.21	1.14622E-08	1.37443E-07	Bet1 golgi vesicular membrane trafficking protein
ENSG00000163975	1.32	5.77	2.48526E-05	0.000136151	antigen p97 (melanoma associated) identified by monoclonal antibodies 133.2 and 96.5
ENSG00000169032	1.32	6.60	1.01968E-09	1.49066E-08	mitogen-activated protein kinase kinase 1
ENSG00000196683	1.32	6.95	8.67589E-10	1.29252E-08	translocase of outer mitochondrial membrane 7 homolog (yeast)
ENSG00000070814	1.32	7.23	4.06242E-09	5.33907E-08	Treacher Collins-Franceschetti syndrome 1
ENSG00000111275	1.32	5.64	6.97245E-09	8.68221E-08	aldehyde dehydrogenase 2 family (mitochondrial)
ENSG00000182534	1.32	5.18	6.68565E-05	0.00032893	matrix-remodelling associated 7
ENSG00000214413	1.32	4.36	1.02267E-05	6.19292E-05	BBSome interacting protein 1
ENSG00000136448	1.32	7.84	5.06388E-09	6.50077E-08	N-myristoyltransferase 1
ENSG00000189057	1.31	4.39	9.49188E-07	7.42021E-06	family with sequence similarity 111, member B
ENSG00000150991	1.31	9.80	4.64652E-07	3.8889E-06	ubiquitin C
ENSG00000119878	1.31	4.78	3.32205E-07	2.86902E-06	cysteine-rich PDZ-binding protein
ENSG00000197256	1.31	5.09	5.54479E-08	5.70554E-07	KN motif and ankyrin repeat domains 2
ENSG00000280828	1.31	4.26	1.06199E-05	6.39893E-05	RP11-274B21.1
ENSG00000262418	1.31	8.73	1.35063E-08	1.59362E-07	protein tyrosine phosphatase, receptor type, C
ENSG00000120709	1.31	6.19	3.9269E-09	5.17119E-08	family with sequence similarity 53, member C
ENSG00000106560	1.31	4.51	9.98483E-07	7.7508E-06	GTPase, IMAP family member 2
ENSG00000177738	1.31	4.73	7.46189E-07	5.99208E-06	CTD-2201E18.3
ENSG00000155876	1.30	5.67	8.09836E-09	9.99049E-08	Ras-related GTP binding A
ENSG00000119820	1.30	7.38	3.544E-10	5.69528E-09	Yip1 domain family, member 4
ENSG00000136238	1.30	9.01	5.19893E-08	5.38725E-07	ras-related C3 botulinum toxin substrate 1 (rho family, small GTP binding protein Rac1)
ENSG00000124201	1.30	5.62	2.10455E-07	1.90558E-06	zinc finger, NFX1-type containing 1
ENSG00000124614	1.30	10.03	4.54715E-06	2.99399E-05	ribosomal protein S10
ENSG00000141526	1.30	8.84	6.79043E-07	5.50282E-06	solute carrier family 16 (monocarboxylate transporter), member 3
ENSG00000198604	1.30	8.76	7.06723E-08	7.15018E-07	bromodomain adjacent to zinc finger domain, 1A
ENSG00000104814	1.30	6.15	1.10421E-07	1.06598E-06	mitogen-activated protein kinase kinase kinase 1
ENSG00000110700	1.30	10.18	3.67255E-06	2.49103E-05	ribosomal protein S13
ENSG00000198682	1.30	4.62	7.90713E-07	6.31524E-06	3'-phosphoadenosine 5'-phosphosulfate synthase 2
ENSG00000167850	1.29	4.53	3.22744E-06	2.2186E-05	CD300c molecule
ENSG00000198265	1.29	6.87	1.1495E-09	1.65853E-08	helicase with zinc finger
ENSG00000113108	1.29	5.25	2.14878E-08	2.40091E-07	amyloid beta (A4) precursor protein-binding, family B, member 3
ENSG00000186470	1.28	5.84	5.1613E-07	4.2766E-06	butyrophilin, subfamily 3, member A2
ENSG00000109046	1.28	7.95	1.41196E-07	1.33018E-06	WD repeat and SOCS box containing 1
ENSG00000185808	1.28	4.86	4.64149E-07	3.88714E-06	phosphatidylinositol glycan anchor biosynthesis, class P
ENSG00000159055	1.28	5.58	8.43052E-08	8.35125E-07	MIS18 kinetochore protein A
ENSG00000164054	1.28	8.20	1.42418E-08	1.66707E-07	shisa family member 5
ENSG00000168961	1.28	7.40	1.47406E-09	2.09376E-08	lectin, galactoside-binding, soluble, 9
ENSG00000102265	1.28	7.27	9.03804E-09	1.10674E-07	TIMP metallopeptidase inhibitor 1
ENSG00000198951	1.28	5.27	6.76551E-08	6.86061E-07	N-acetylgalactosaminidase, alpha-
ENSG00000147439	1.27	4.91	2.55031E-06	1.79305E-05	bridging integrator 3
ENSG00000085224	1.27	6.98	1.4378E-07	1.35261E-06	alpha thalassemia/mental retardation syndrome X-linked
ENSG00000124067	1.27	4.34	4.19476E-06	2.79397E-05	solute carrier family 12 (potassium/chloride transporter), member 4
ENSG00000129810	1.27	5.19	2.34508E-06	1.66334E-05	shugoshin-like 1 (S. pombe)
ENSG00000145743	1.27	5.23	8.02404E-06	4.98881E-05	F-box and leucine-rich repeat protein 17
ENSG00000157349	1.27	4.29	2.94922E-05	0.000158686	DEAD (Asp-Glu-Ala-Asp) box polypeptide 19B
ENSG00000281794	1.26	6.44	1.76276E-08	2.00849E-07	long intergenic non-protein coding RNA 969
ENSG00000253230	1.26	5.11	9.27913E-07	7.28208E-06	long intergenic non-protein coding RNA 599

ENSG00000100599	1.26	5.06	2.62895E-07	2.3249E-06	Ras and Rab interactor 3
ENSG00000105372	1.26	10.45	1.75031E-05	9.96721E-05	ribosomal protein S19
ENSG00000068137	1.26	4.60	5.07951E-05	0.00025794	pleckstrin homology domain containing, family H (with MyTH4 domain) member 3
ENSG00000248092	1.26	4.94	4.00432E-06	2.68587E-05	NNT antisense RNA 1
ENSG00000132963	1.26	8.00	2.75962E-09	3.73788E-08	proteasome maturation protein
ENSG00000130164	1.26	7.21	1.13492E-08	1.3621E-07	low density lipoprotein receptor
ENSG00000144034	1.25	5.95	2.02997E-08	2.28468E-07	TP53RK binding protein
ENSG00000102977	1.25	4.51	1.35502E-05	7.93058E-05	adrenocortical dysplasia homolog (mouse)
ENSG00000128016	1.25	7.22	9.04722E-08	8.87604E-07	ZFP36 ring finger protein
ENSG00000117394	1.25	6.09	1.43468E-08	1.67788E-07	solute carrier family 2 (facilitated glucose transporter), member 1
ENSG00000163125	1.25	6.39	1.27153E-08	1.50564E-07	regulation of nuclear pre-mRNA domain containing 2
ENSG00000229117	1.24	10.31	6.46238E-06	4.11225E-05	ribosomal protein L41
ENSG00000169499	1.24	5.67	2.62989E-08	2.88744E-07	pleckstrin homology domain containing, family A (phosphoinositide binding specific) member 2
ENSG00000117614	1.24	6.27	1.75806E-07	1.62522E-06	SYF2 pre-mRNA-splicing factor
ENSG00000082074	1.24	5.22	1.17055E-06	8.88388E-06	FYN binding protein
ENSG00000090013	1.24	5.55	4.14718E-08	4.36902E-07	biliverdin reductase B
ENSG00000135974	1.24	5.74	4.35949E-08	4.58178E-07	chromosome 2 open reading frame 49
ENSG00000173207	1.24	6.64	8.70867E-09	1.0674E-07	CDC28 protein kinase regulatory subunit 1B
ENSG00000132950	1.24	4.24	4.6889E-05	0.000240497	zinc finger, MYM-type 5
ENSG00000205423	1.23	4.29	5.89987E-05	0.000294306	CTD nuclear envelope phosphatase 1 regulatory subunit 1
ENSG00000143353	1.23	4.98	5.04374E-06	3.28189E-05	lysophospholipase-like 1
ENSG00000172725	1.23	7.10	0.00016445	0.000726573	coronin, actin binding protein, 1B
ENSG00000254999	1.23	7.82	7.09567E-08	7.17349E-07	BRICK1, SCAR/WAVE actin-nucleating complex subunit
ENSG00000175727	1.23	7.26	1.02721E-08	1.24636E-07	MLX interacting protein
ENSG00000221983	1.23	10.29	1.74668E-05	9.95082E-05	ubiquitin A-52 residue ribosomal protein fusion product 1
ENSG00000143507	1.22	5.00	1.81943E-06	1.31685E-05	dual specificity phosphatase 10
ENSG00000239382	1.22	4.53	3.86714E-06	2.6057E-05	alkB homolog 6
ENSG00000118985	1.22	4.90	7.05818E-07	5.692E-06	elongation factor, RNA polymerase II, 2
ENSG00000178802	1.22	6.21	2.22789E-08	2.4768E-07	mannose phosphate isomerase
ENSG00000135452	1.22	4.72	2.3776E-06	1.68232E-05	tetraspanin 31
ENSG00000227669	1.22	6.48	5.70432E-08	5.85608E-07	major histocompatibility complex, class I, H (pseudogene)
ENSG00000106617	1.22	4.59	4.64346E-06	3.04417E-05	protein kinase, AMP-activated, gamma 2 non-catalytic subunit
ENSG00000117697	1.22	6.16	2.36814E-08	2.62175E-07	NSL1, MIS12 kinetochore complex component
ENSG00000168255	1.21	4.65	1.0825E-05	6.51362E-05	polymerase (RNA) II (DNA directed) polypeptide J3
ENSG00000166128	1.21	5.95	7.0505E-08	7.1387E-07	RAB8B, member RAS oncogene family
ENSG00000129696	1.21	4.83	0.000343877	0.001380709	TELO2 interacting protein 2
ENSG00000141971	1.21	5.93	9.1813E-08	9.00093E-07	multivesicular body subunit 12A
ENSG00000145247	1.21	4.50	5.79461E-06	3.72844E-05	OCIA domain containing 2
ENSG00000166341	1.21	5.45	1.12522E-07	1.0845E-06	dachsous cadherin-related 1
ENSG00000186010	1.20	6.48	3.73201E-08	3.96945E-07	NADH dehydrogenase (ubiquinone) 1 alpha subcomplex, 13
ENSG00000125971	1.20	6.77	9.31071E-09	1.13699E-07	dynein, light chain, roadblock-type 1
ENSG00000160223	1.20	5.66	4.29593E-06	2.85121E-05	inducible T-cell co-stimulator ligand
ENSG00000165264	1.20	5.82	1.58603E-07	1.48156E-06	NADH dehydrogenase (ubiquinone) 1 beta subcomplex, 6, 17kDa
ENSG00000163131	1.20	7.75	1.68093E-08	1.92185E-07	cathepsin S
ENSG00000158869	1.20	7.44	1.13179E-08	1.35958E-07	Fc fragment of IgE, high affinity I, receptor for; gamma polypeptide
ENSG00000114439	1.20	4.33	0.000182099	0.000795386	bobby sox homolog (Drosophila)
ENSG00000114062	1.20	6.74	6.84118E-07	5.53719E-06	ubiquitin protein ligase E3A
ENSG00000162704	1.19	9.00	6.99194E-07	5.64887E-06	actin related protein 2/3 complex, subunit 5, 16kDa
ENSG00000102316	1.19	6.17	4.62309E-08	4.83204E-07	melanoma antigen family D2
ENSG00000005238	1.19	4.33	1.23359E-05	7.31337E-05	family with sequence similarity 214, member B
ENSG00000149257	1.19	6.79	1.80091E-08	2.04319E-07	serpin peptidase inhibitor, clade H (heat shock protein 47), member 1, (collagen binding protein 1)
ENSG00000084676	1.18	5.02	2.54149E-05	0.000138918	nuclear receptor coactivator 1
ENSG00000124766	1.18	6.67	2.67319E-08	2.93014E-07	SRY (sex determining region Y)-box 4
ENSG00000114391	1.18	9.58	2.70447E-06	1.89442E-05	ribosomal protein L24
ENSG00000159882	1.18	4.46	5.16751E-05	0.000261808	zinc finger protein 230
ENSG00000141428	1.18	4.34	1.52418E-05	8.78888E-05	chromosome 18 open reading frame 21
ENSG00000142687	1.18	6.84	4.0911E-08	4.31336E-07	KIAA0319-like
ENSG00000127540	1.18	6.30	1.60626E-07	1.49835E-06	ubiquinol-cytochrome c reductase, complex III subunit XI
ENSG00000160753	1.18	5.54	6.55096E-07	5.32501E-06	RUN and SH3 domain containing 1
ENSG00000114796	1.17	4.69	7.20889E-06	4.53726E-05	kelch-like family member 24
ENSG00000196154	1.17	8.19	1.14692E-06	8.71951E-06	S100 calcium binding protein A4
ENSG00000177576	1.17	4.70	3.15722E-05	0.00016858	chromosome 18 open reading frame 32
ENSG00000164167	1.17	5.65	2.02229E-06	1.45024E-05	LSM6 homolog, U6 small nuclear RNA and mRNA degradation associated
ENSG00000278461	1.17	5.01	1.0335E-05	6.24997E-05	mitochondrial ribosomal protein S36
ENSG00000096996	1.17	4.38	1.18648E-05	7.08156E-05	interleukin 12 receptor, beta 1
ENSG00000137806	1.16	4.95	2.74348E-06	1.9187E-05	NADH dehydrogenase (ubiquinone) complex I, assembly factor 1
ENSG00000175305	1.16	4.39	0.000164097	0.000725505	cyclin E2
ENSG00000185088	1.16	6.83	2.20846E-07	1.98881E-06	ribosomal protein S27-like
ENSG00000104812	1.16	6.96	1.53691E-07	1.43871E-06	glycogen synthase 1 (muscle)
ENSG00000160049	1.16	7.06	6.16438E-08	6.29915E-07	DNA fragmentation factor, 45kDa, alpha polypeptide
ENSG00000197324	1.16	6.91	3.1561E-05	0.00016858	low density lipoprotein receptor-related protein 10

ENSG00000196628	1.16	7.78	2.38858E-07	2.13365E-06	transcription factor 4
ENSG00000269858	1.16	6.00	0.00020036	0.000861821	egl-9 family hypoxia-inducible factor 2
ENSG00000134248	1.16	6.19	2.26017E-07	2.03125E-06	late endosomal/lysosomal adaptor, MAPK and MTOR activator 5
ENSG00000223745	1.16	4.94	1.48769E-05	8.60839E-05	RP4-717I23.3
ENSG00000158417	1.16	8.72	9.60568E-07	7.49593E-06	eukaryotic translation initiation factor 5B
ENSG00000185950	1.15	8.44	0.000162497	0.000719475	insulin receptor substrate 2
ENSG00000007047	1.15	5.20	8.10374E-07	6.44512E-06	MAP/microtubule affinity-regulating kinase 4
ENSG00000105220	1.15	9.48	7.08514E-06	4.46572E-05	glucose-6-phosphate isomerase
ENSG00000153113	1.15	5.84	2.79195E-06	1.94849E-05	calpastatin
ENSG00000135926	1.15	5.06	4.26335E-06	2.83136E-05	transmembrane BAX inhibitor motif containing 1
ENSG00000145050	1.15	7.72	3.34554E-07	2.88743E-06	mesencephalic astrocyte-derived neurotrophic factor
ENSG00000183648	1.14	6.32	5.34046E-07	4.40853E-06	NADH dehydrogenase (ubiquinone) 1 beta subcomplex, 1, 7kDa
ENSG00000113732	1.14	7.85	7.3031E-06	4.58568E-05	ATPase, H+ transporting, lysosomal 9kDa, V0 subunit e1
ENSG00000124006	1.14	4.82	0.001024095	0.00358204	obscurin-like 1
ENSG00000105856	1.14	4.88	4.78908E-06	3.12846E-05	HMG-box transcription factor 1
ENSG00000196507	1.14	5.25	7.44863E-07	5.99165E-06	transcription elongation factor A (SII)-like 3
ENSG00000187118	1.14	5.83	4.87541E-07	4.0549E-06	C-x(9)-C motif containing 1
ENSG00000166928	1.14	4.39	0.000113191	0.000524068	membrane-spanning 4-domains, subfamily A, member 14
ENSG00000167625	1.14	4.50	0.000128862	0.000586397	zinc finger protein 526
ENSG00000102144	1.13	10.06	5.4357E-05	0.000273412	phosphoglycerate kinase 1
ENSG00000174282	1.13	4.84	1.02185E-05	6.19112E-05	zinc finger and BTB domain containing 4
ENSG00000156535	1.13	5.88	2.04029E-05	0.000114418	CD109 molecule
ENSG00000229252	1.13	9.09	2.91342E-06	2.01947E-05	major histocompatibility complex, class I, E
ENSG00000134954	1.13	6.77	8.99648E-08	8.83932E-07	v-ets avian erythroblastosis virus E26 oncogene homolog 1
ENSG00000275199	1.13	4.56	2.97896E-05	0.000160092	v-akt murine thymoma viral oncogene homolog 3
ENSG00000076650	1.13	4.86	6.22678E-06	3.97568E-05	G patch domain containing 1
ENSG00000117616	1.13	7.82	1.52057E-07	1.42542E-06	arginine/serine-rich protein 1
ENSG00000114544	1.13	5.29	1.65479E-05	9.48434E-05	solute carrier family 41, member 3
ENSG00000075711	1.13	6.37	2.66597E-07	2.35137E-06	discs, large homolog 1 (Drosophila)
ENSG00000005448	1.13	4.64	0.001038847	0.003624088	WD repeat domain 54
ENSG00000088854	1.13	5.19	5.80185E-06	3.73129E-05	chromosome 20 open reading frame 194
ENSG00000265681	1.13	9.74	1.73511E-05	9.9019E-05	ribosomal protein L17
ENSG00000168802	1.12	6.69	7.28295E-06	4.57736E-05	chromosome transmission fidelity factor 8
ENSG00000139684	1.12	6.61	1.19991E-07	1.15001E-06	esterase D
ENSG00000008324	1.12	4.99	6.2086E-06	3.96598E-05	synovial sarcoma translocation gene on chromosome 18-like 2
ENSG00000124098	1.12	4.87	1.25285E-05	7.41762E-05	family with sequence similarity 210, member B
ENSG00000170315	1.12	10.24	0.000118081	0.000542917	ubiquitin B
ENSG00000064393	1.12	7.04	7.41904E-05	0.000360206	homeodomain interacting protein kinase 2
ENSG00000177200	1.12	5.40	2.38576E-06	1.68675E-05	chromodomain helicase DNA binding protein 9
ENSG00000105640	1.12	9.91	3.68773E-05	0.000192922	ribosomal protein L18a
ENSG00000006534	1.12	6.77	4.79049E-07	3.9993E-06	aldehyde dehydrogenase 3 family, member B1
ENSG00000276879	1.12	4.22	9.64528E-05	0.000452888	interferon, alpha-inducible protein 27-like 2
ENSG00000125870	1.11	7.59	8.35172E-08	8.29796E-07	small nuclear ribonucleoprotein polypeptide B
ENSG00000164074	1.11	5.44	1.62464E-06	1.19278E-05	chromosome 4 open reading frame 29
ENSG00000163110	1.11	5.56	4.3795E-06	2.89798E-05	PDZ and LIM domain 5
ENSG00000164088	1.11	5.01	5.84135E-06	3.75487E-05	protein phosphatase, Mg2+/Mn2+ dependent, 1M
ENSG00000125354	1.11	6.88	4.2311E-07	3.56594E-06	septin 6
ENSG00000130311	1.10	6.37	3.7365E-07	3.18853E-06	DET1 and DDB1 associated 1
ENSG00000206503	1.10	11.22	0.000397168	0.001566694	major histocompatibility complex, class I, A
ENSG00000141298	1.10	5.07	0.000811334	0.002933711	slingshot protein phosphatase 2
ENSG00000116205	1.10	4.60	2.83249E-05	0.000152963	transcription elongation factor A (SII) N-terminal and central domain containing 2
ENSG00000237765	1.10	4.57	0.000581606	0.002190762	family with sequence similarity 200, member B
ENSG00000125347	1.09	5.35	0.000762255	0.002780484	interferon regulatory factor 1
ENSG00000107738	1.09	7.11	1.39054E-07	1.31187E-06	chromosome 10 open reading frame 54
ENSG00000038002	1.09	6.05	1.04154E-05	6.29285E-05	aspartylglucosaminidase
ENSG00000153250	1.09	6.73	3.31704E-07	2.86657E-06	RNA binding motif, single stranded interacting protein 1
ENSG00000161203	1.09	7.99	0.000215419	0.000919572	adaptor-related protein complex 2, mu 1 subunit
ENSG00000132694	1.09	5.95	1.01844E-06	7.88265E-06	Rho guanine nucleotide exchange factor (GEF) 11
ENSG00000184752	1.09	5.84	2.26751E-06	1.61302E-05	NADH dehydrogenase (ubiquinone) 1 alpha subcomplex, 12
ENSG00000133246	1.09	5.89	9.27711E-06	5.67486E-05	PML-RARA regulated adaptor molecule 1
ENSG00000125037	1.09	6.40	6.85364E-06	4.32597E-05	ER membrane protein complex subunit 3
ENSG00000241343	1.09	7.22	8.81585E-07	6.9449E-06	ribosomal protein L36a
ENSG00000163960	1.09	6.15	7.73561E-07	6.20063E-06	UBX domain protein 7
ENSG00000108599	1.09	5.10	7.45023E-06	4.66483E-05	A kinase (PRKA) anchor protein 10
ENSG00000150540	1.09	6.52	3.55152E-06	2.42255E-05	histamine N-methyltransferase
ENSG00000105011	1.08	6.88	2.01036E-07	1.82903E-06	anti-silencing function 1B histone chaperone
ENSG00000082996	1.08	5.97	1.58634E-06	1.16724E-05	ring finger protein 13
ENSG00000110492	1.08	5.40	8.97973E-06	5.51582E-05	midkine (neurite growth-promoting factor 2)
ENSG00000105849	1.08	7.27	6.17609E-06	3.94711E-05	TWIST neighbor
ENSG00000160058	1.08	5.76	7.48994E-07	6.00734E-06	BSD domain containing 1
ENSG00000105223	1.08	8.09	3.32092E-06	2.27696E-05	phospholipase D family, member 3
ENSG00000129636	1.08	5.53	9.20264E-06	5.63584E-05	integrin alpha FG-GAP repeat containing 1

ENSG00000034510	1.08	8.62	2.51156E-06	1.76861E-05	thymosin beta 10
ENSG00000143119	1.08	8.28	9.93592E-07	7.72187E-06	CD53 molecule
ENSG00000132581	1.07	5.22	1.04961E-05	6.33299E-05	stromal cell-derived factor 2
ENSG00000105404	1.07	5.11	5.95899E-06	3.82082E-05	Rab acceptor 1 (prenylated)
ENSG00000205220	1.07	6.60	1.4488E-06	1.07679E-05	proteasome (prosome, macropain) subunit, beta type, 10
ENSG00000262349	1.07	5.97	1.38447E-06	1.03302E-05	RP11-620J15.3
ENSG00000143013	1.07	7.78	2.15594E-05	0.000120042	LIM domain only 4
ENSG00000100644	1.07	6.57	1.09486E-06	8.38611E-06	hypoxia inducible factor 1, alpha subunit (basic helix-loop-helix transcription factor)
ENSG00000173486	1.07	6.37	8.79346E-07	6.93549E-06	FK506 binding protein 2, 13kDa
ENSG00000169926	1.07	6.98	8.49178E-07	6.71784E-06	Kruppel-like factor 13
ENSG00000010404	1.07	4.60	3.69335E-05	0.000192943	iduronate 2-sulfatase
ENSG00000153132	1.06	5.17	0.000130251	0.000591905	calmegin
ENSG00000282230	1.06	5.96	2.82724E-06	1.96898E-05	ADAM metallopeptidase domain 9
ENSG00000079739	1.06	6.48	0.000171742	0.000755371	phosphoglucomutase 1
ENSG00000143198	1.06	7.21	2.49439E-07	2.21772E-06	microsomal glutathione S-transferase 3
ENSG00000143458	1.06	5.52	3.537E-06	2.41389E-05	GA binding protein transcription factor, beta subunit 2
ENSG00000077152	1.06	6.00	2.28225E-06	1.62263E-05	ubiquitin-conjugating enzyme E2T
ENSG00000173221	1.06	4.79	0.000359586	0.001432518	glutaredoxin (thioltransferase)
ENSG00000153048	1.06	5.92	1.42349E-05	8.26935E-05	calcium regulated heat stable protein 1, 24kDa
ENSG00000101363	1.06	4.64	3.61028E-05	0.000189269	mannosidase, beta A, lysosomal-like
ENSG00000120727	1.06	7.20	6.30736E-07	5.16177E-06	poly(A) binding protein interacting protein 2
ENSG00000103335	1.05	7.98	2.82494E-06	1.96841E-05	piezo-type mechanosensitive ion channel component 1
ENSG00000112773	1.05	10.06	8.94377E-05	0.000423243	family with sequence similarity 46, member A
ENSG00000121067	1.05	6.81	3.00015E-06	2.0734E-05	speckle-type POZ protein
ENSG00000142002	1.05	6.83	1.81304E-06	1.31295E-05	dipeptidyl-peptidase 9
ENSG00000178567	1.05	6.07	8.82338E-06	5.43488E-05	EPM2A (laforin) interacting protein 1
ENSG00000103671	1.05	5.26	6.5052E-06	4.12961E-05	thyroid hormone receptor interactor 4
ENSG00000247556	1.05	8.35	1.64333E-06	1.2045E-05	OIP5 antisense RNA 1
ENSG00000256235	1.05	5.90	5.05676E-06	3.28714E-05	small integral membrane protein 3
ENSG00000119844	1.05	6.59	4.52704E-06	2.98371E-05	aftiphilin
ENSG00000171320	1.04	4.29	0.000463658	0.001792252	establishment of sister chromatid cohesion N-acetyltransferase 2
ENSG00000119471	1.04	5.64	5.59254E-05	0.000280876	hydroxysteroid dehydrogenase like 2
ENSG00000102910	1.04	5.87	2.59117E-06	1.81985E-05	lon peptidase 2, peroxisomal
ENSG00000106261	1.04	6.83	1.20963E-06	9.14387E-06	zinc finger with KRAB and SCAN domains 1
ENSG00000138085	1.04	6.88	1.41682E-06	1.05478E-05	all-trans retinoic acid-induced differentiation factor
ENSG00000102908	1.04	5.66	0.000117999	0.000542729	nuclear factor of activated T-cells 5, tonicity-responsive
ENSG00000161955	1.04	5.06	1.95196E-05	0.000109929	tumor necrosis factor (ligand) superfamily, member 13
ENSG00000189343	1.04	4.93	0.00026884	0.001115531	ribosomal protein S2 pseudogene 46
ENSG00000148908	1.04	4.43	0.000154398	0.000688342	regulator of G-protein signaling 10
ENSG00000135090	1.04	8.03	1.20847E-06	9.14031E-06	TAO kinase 3
ENSG00000152253	1.04	5.11	9.35682E-05	0.000440747	SPC25, NDC80 kinetochore complex component
ENSG00000165118	1.04	5.38	6.10458E-06	3.90329E-05	chromosome 9 open reading frame 64
ENSG00000167193	1.03	5.12	1.71444E-05	9.80083E-05	v-crk avian sarcoma virus CT10 oncogene homolog
ENSG00000172830	1.03	6.09	2.05762E-06	1.47478E-05	slingshot protein phosphatase 3
ENSG00000166927	1.03	5.48	3.30678E-05	0.000175507	membrane-spanning 4-domains, subfamily A, member 7
ENSG00000123975	1.03	7.30	8.97416E-07	7.06123E-06	CDC28 protein kinase regulatory subunit 2
ENSG00000107819	1.03	6.67	1.14526E-06	8.71183E-06	sideroflexin 3
ENSG00000143369	1.03	5.11	1.36546E-05	7.98115E-05	extracellular matrix protein 1
ENSG00000038219	1.02	6.26	1.84549E-05	0.000104644	biorientation of chromosomes in cell division 1-like 1
ENSG00000127507	1.02	5.54	2.0606E-05	0.000115362	adhesion G protein-coupled receptor E2
ENSG00000113971	1.02	5.66	1.29057E-05	7.60701E-05	nephronophthisis 3 (adolescent)
ENSG00000182179	1.02	6.91	5.00949E-06	3.26281E-05	ubiquitin-like modifier activating enzyme 7
ENSG00000124357	1.02	5.91	9.14909E-06	5.61206E-05	N-acetylglucosamine kinase
ENSG00000114423	1.02	4.59	8.97356E-05	0.000424048	Cbl proto-oncogene B, E3 ubiquitin protein ligase
ENSG00000135441	1.02	5.87	4.57289E-05	0.000235365	biogenesis of lysosomal organelles complex-1, subunit 1
ENSG00000129292	1.02	6.53	5.65805E-06	3.65119E-05	PHD finger protein 20-like 1
ENSG00000024862	1.02	5.51	0.000712073	0.002619024	coiled-coil domain containing 28A
ENSG00000102893	1.02	5.80	4.02337E-06	2.6939E-05	phosphorylase kinase, beta
ENSG00000099860	1.02	4.66	0.000132186	0.000599056	growth arrest and DNA-damage-inducible, beta
ENSG00000124226	1.02	7.05	1.24494E-06	9.3894E-06	ring finger protein 114
ENSG00000133639	1.02	6.63	4.11535E-06	2.74784E-05	B-cell translocation gene 1, anti-proliferative
ENSG00000162222	1.02	5.13	6.83936E-05	0.000335622	tetratricopeptide repeat domain 9C
ENSG00000131408	1.02	6.61	6.41987E-05	0.000317383	nuclear receptor subfamily 1, group H, member 2
ENSG00000117407	1.01	5.35	1.1973E-05	7.12773E-05	artemin
ENSG00000282031	1.01	6.48	0.00018946	0.00082186	transmembrane BAX inhibitor motif containing 4
ENSG00000170145	1.01	5.03	0.001220335	0.004170629	salt-inducible kinase 2
ENSG00000179152	1.01	5.78	7.95948E-06	4.95331E-05	T cell activation inhibitor, mitochondrial
ENSG00000178878	1.01	5.98	9.68612E-06	5.906E-05	apolipoprotein L domain containing 1
ENSG00000122257	1.01	7.35	2.69443E-06	1.88838E-05	retinoblastoma binding protein 6
ENSG00000107798	1.01	7.03	1.94551E-05	0.000109659	lipase A, lysosomal acid, cholesterol esterase
ENSG00000106780	1.01	5.62	2.58505E-05	0.000141093	multiple EGF-like-domains 9
ENSG00000104976	1.01	4.58	0.000404706	0.001590309	small nuclear RNA activating complex, polypeptide 2, 45kDa

ENSG00000175550	1.01	6.17	2.15453E-05	0.000120042	DR1-associated protein 1 (negative cofactor 2 alpha)
ENSG00000162244	1.01	10.29	0.000260847	0.001087124	ribosomal protein L29
ENSG00000161202	1.00	4.28	0.000221531	0.000942804	dishevelled segment polarity protein 3
ENSG00000125629	1.00	4.85	0.000197747	0.000852239	insulin induced gene 2
ENSG00000008513	1.00	7.18	2.84691E-05	0.000153617	ST3 beta-galactoside alpha-2,3-sialyltransferase 1
ENSG00000206190	1.00	4.23	0.002526845	0.00782579	ATPase, class V, type 10A
ENSG00000035141	-1.00	6.01	1.33505E-05	7.82402E-05	family with sequence similarity 136, member A
ENSG00000140403	-1.00	5.65	5.27981E-05	0.000266734	DnaJ (Hsp40) homolog, subfamily A, member 4
ENSG00000107104	-1.00	4.94	8.76854E-05	0.000416137	KN motif and ankyrin repeat domains 1
ENSG00000114867	-1.00	8.27	2.75931E-05	0.000149682	eukaryotic translation initiation factor 4 gamma, 1
ENSG00000132436	-1.00	4.87	6.21906E-05	0.000308605	fidgetin-like 1
ENSG00000108883	-1.00	7.33	3.28058E-06	2.25161E-05	elongation factor Tu GTP binding domain containing 2
ENSG00000102900	-1.00	5.16	8.77487E-05	0.000416289	nucleoporin 93kDa
ENSG00000168936	-1.00	5.23	1.40737E-05	8.19721E-05	transmembrane protein 129, E3 ubiquitin protein ligase
ENSG00000163389	-1.00	5.28	3.20986E-05	0.000171047	protein O-glucosyltransferase 1
ENSG00000213639	-1.01	6.82	1.56499E-06	1.15409E-05	protein phosphatase 1, catalytic subunit, beta isozyme
ENSG00000057663	-1.01	5.75	5.56793E-06	3.59653E-05	autophagy related 5
ENSG00000109736	-1.01	8.12	1.262E-05	7.45714E-05	major facilitator superfamily domain containing 10
ENSG00000025156	-1.01	4.56	9.15211E-05	0.00043141	heat shock transcription factor 2
ENSG00000138018	-1.01	5.58	8.63884E-06	5.33358E-05	ethanolaminephosphotransferase 1
ENSG00000166557	-1.01	6.69	1.999E-05	0.000112293	transmembrane emp24 protein transport domain containing 3
ENSG00000185344	-1.01	5.17	0.000114694	0.000529362	ATPase, H+ transporting, lysosomal V0 subunit a2
ENSG00000160803	-1.01	6.01	8.17204E-06	5.06853E-05	ubiquilin 4
ENSG00000087365	-1.01	7.77	1.88147E-06	1.35732E-05	splicing factor 3b, subunit 2, 145kDa
ENSG00000188971	-1.01	5.12	3.13472E-05	0.000167648	RP11-427H3.3
ENSG00000258984	-1.01	4.56	0.000109747	0.000509363	UBE2F-SCLY readthrough (NMD candidate)
ENSG00000175467	-1.01	6.34	4.53069E-06	2.98464E-05	squamous cell carcinoma antigen recognized by T cells 1
ENSG00000171497	-1.01	5.58	1.28508E-05	7.5814E-05	peptidylprolyl isomerase D
ENSG00000163444	-1.01	6.46	3.17376E-06	2.18282E-05	transmembrane protein 183A
ENSG00000112249	-1.01	5.36	7.51465E-06	4.70073E-05	activating signal cointegrator 1 complex subunit 3
ENSG00000170854	-1.01	4.34	0.000217972	0.000928848	MYC induced nuclear antigen
ENSG00000172053	-1.01	7.62	4.55771E-06	2.9992E-05	glutaminyl-tRNA synthetase
ENSG00000204946	-1.01	4.38	0.000208519	0.000893153	zinc finger family member 783
ENSG00000169045	-1.02	9.42	4.3042E-05	0.000222397	heterogeneous nuclear ribonucleoprotein H1 (H)
ENSG00000198799	-1.02	4.78	0.000115669	0.000533306	leucine-rich repeats and immunoglobulin-like domains 2
ENSG00000105676	-1.02	5.80	1.10672E-05	6.64734E-05	armadillo repeat containing 6
ENSG00000043514	-1.02	4.61	0.000271705	0.001126009	tRNA isopentenyltransferase 1
ENSG00000169689	-1.02	5.82	3.01623E-05	0.000161898	stimulated by retinoic acid 13
ENSG00000230257	-1.02	4.73	0.000400998	0.001579013	nuclear factor, erythroid 4
ENSG00000134186	-1.02	6.74	1.13916E-06	8.6704E-06	pre-mRNA processing factor 38B
ENSG00000164087	-1.02	4.49	0.000109283	0.000507744	POC1 centriolar protein A
ENSG00000161996	-1.02	6.18	3.9295E-05	0.000204309	WD repeat domain 90
ENSG00000132819	-1.02	5.82	7.66378E-05	0.000370463	RNA binding motif protein 38
ENSG00000273562	-1.02	6.91	1.4466E-06	1.07575E-05	nucleosome assembly protein 1-like 4
ENSG00000141522	-1.02	8.98	2.40679E-05	0.000132179	Rho GDP dissociation inhibitor (GDI) alpha
ENSG00000111144	-1.02	6.02	0.000103192	0.000481636	leukotriene A4 hydrolase
ENSG00000111667	-1.02	5.20	1.56188E-05	8.98672E-05	ubiquitin specific peptidase 5 (isopeptidase T)
ENSG00000103148	-1.02	5.02	2.89801E-05	0.000156248	NPR3-like, GATOR1 complex subunit
ENSG00000086289	-1.02	4.25	0.000635816	0.002373401	ependymin related 1
ENSG00000160949	-1.02	6.37	7.36501E-05	0.000357975	tonsoku-like, DNA repair protein
ENSG00000146463	-1.02	6.65	1.57207E-06	1.15756E-05	zinc finger, MYM-type 4
ENSG00000137288	-1.02	5.29	6.46626E-06	4.11276E-05	ubiquinol-cytochrome c reductase complex assembly factor 2
ENSG00000175634	-1.03	5.54	5.26048E-06	3.41122E-05	ribosomal protein S6 kinase, 70kDa, polypeptide 2
ENSG00000095319	-1.03	6.96	2.78122E-05	0.000150686	nucleoporin 188kDa
ENSG00000001497	-1.03	5.91	1.26233E-05	7.45714E-05	LAS1-like, ribosome biogenesis factor
ENSG00000113048	-1.03	5.81	4.10303E-06	2.74238E-05	mitochondrial ribosomal protein S27
ENSG00000106628	-1.03	7.09	9.29145E-07	7.28498E-06	polymerase (DNA directed), delta 2, accessory subunit
ENSG00000165792	-1.03	6.31	5.90435E-06	3.78803E-05	methyltransferase like 17
ENSG00000118900	-1.03	5.92	0.000116511	0.000536817	ubinuclein 1
ENSG00000008441	-1.03	4.38	0.000626907	0.002344104	nuclear factor I/X (CCAAT-binding transcription factor)
ENSG00000162613	-1.03	6.84	8.57722E-07	6.78106E-06	far upstream element (FUSE) binding protein 1
ENSG00000128309	-1.03	4.67	7.52059E-05	0.000364337	mercaptopyruvate sulfurtransferase
ENSG00000166987	-1.04	6.46	5.17989E-05	0.000262234	methyl-CpG binding domain protein 6
ENSG00000168439	-1.04	7.88	9.90955E-07	7.70588E-06	stress-induced phosphoprotein 1
ENSG00000132323	-1.04	5.26	6.69523E-06	4.24213E-05	integrin-linked kinase-associated serine/threonine phosphatase
ENSG00000157193	-1.04	5.90	9.30708E-06	5.69057E-05	low density lipoprotein receptor-related protein 8, apolipoprotein e receptor
ENSG00000089280	-1.04	8.85	5.31887E-06	3.4474E-05	FUS RNA binding protein
ENSG00000186501	-1.04	5.39	1.0219E-05	6.19112E-05	transmembrane protein 222
ENSG00000160131	-1.04	6.52	1.40074E-06	1.04399E-05	VMA21 vacuolar H+-ATPase homolog (S. cerevisiae)
ENSG00000165688	-1.04	6.39	4.60423E-06	3.02407E-05	peptidase (mitochondrial processing) alpha
ENSG00000163349	-1.04	6.18	2.71112E-05	0.000147429	homeodomain interacting protein kinase 1
ENSG00000153574	-1.04	5.49	2.82626E-05	0.00015272	ribose 5-phosphate isomerase A

ENSG00000261739	-1.04	7.00	9.45392E-07	7.39925E-06	golgin A8 family, member S
ENSG00000008853	-1.04	4.30	0.000175732	0.000771129	Rho-related BTB domain containing 2
ENSG00000013306	-1.04	6.19	1.64876E-06	1.20781E-05	solute carrier family 25, member 39
ENSG00000169914	-1.04	4.34	0.000130459	0.000592419	OTU deubiquitinase 3
ENSG00000280987	-1.04	7.95	6.41944E-07	5.2438E-06	matrin 3
ENSG00000007080	-1.04	5.30	6.05157E-06	3.87125E-05	coiled-coil domain containing 124
ENSG00000004455	-1.05	9.49	6.62154E-05	0.000326483	adenylate kinase 2
ENSG00000241945	-1.05	5.95	1.9386E-05	0.000109316	PWP2 periodic tryptophan protein homolog (yeast)
ENSG00000138363	-1.05	6.47	9.47242E-06	5.78622E-05	5-aminoimidazole-4-carboxamide ribonucleotide formyltransferase/IMP cyclohydrolase
ENSG00000167721	-1.05	5.51	7.93094E-06	4.94018E-05	TSR1, 20S rRNA accumulation, homolog (S. cerevisiae)
ENSG00000234495	-1.05	7.47	4.61279E-07	3.86554E-06	tripartite motif containing 27
ENSG00000170085	-1.05	5.21	8.17517E-06	5.06853E-05	SUMO-interacting motifs containing 1
ENSG00000178952	-1.05	7.62	6.52135E-07	5.30744E-06	Tu translation elongation factor, mitochondrial
ENSG00000163875	-1.05	5.42	6.74702E-06	4.27263E-05	MYST/Esa1-associated factor 6
ENSG00000146223	-1.05	7.93	3.93665E-06	2.64851E-05	ribosomal protein L7-like 1
ENSG00000134440	-1.05	7.70	7.20526E-07	5.80004E-06	asparaginyl-tRNA synthetase
ENSG00000110721	-1.05	5.87	2.38839E-06	1.68675E-05	choline kinase alpha
ENSG00000096746	-1.05	7.40	1.37312E-06	1.02571E-05	heterogeneous nuclear ribonucleoprotein H3 (2H9)
ENSG00000198301	-1.05	5.76	2.65825E-06	1.864E-05	SDA1 domain containing 1
ENSG00000171612	-1.05	4.36	0.000470696	0.001815757	solute carrier family 25 (pyrimidine nucleotide carrier), member 33
ENSG00000164024	-1.05	5.65	9.18278E-06	5.62955E-05	methionyl aminopeptidase 1
ENSG00000261221	-1.05	4.43	0.000120218	0.000550836	zinc finger protein 865
ENSG00000189306	-1.05	5.39	3.61454E-06	2.45545E-05	ribosomal RNA processing 7 homolog A
ENSG00000196700	-1.05	5.51	5.05944E-05	0.000257342	zinc finger protein 512B
ENSG00000118640	-1.06	7.88	6.33958E-07	5.18175E-06	vesicle-associated membrane protein 8
ENSG00000276187	-1.06	4.39	0.000136279	0.000615715	ER membrane-associated RNA degradation
ENSG00000071626	-1.06	7.83	4.92541E-07	4.09136E-06	DAZ associated protein 1
ENSG00000211460	-1.06	7.02	3.51824E-07	3.01687E-06	translin
ENSG00000025796	-1.06	7.41	7.6791E-06	4.79908E-05	SEC63 homolog (S. cerevisiae)
ENSG00000115306	-1.06	6.30	1.82158E-06	1.31769E-05	spectrin, beta, non-erythrocytic 1
ENSG00000169750	-1.06	4.93	5.15411E-05	0.000261428	ras-related C3 botulinum toxin substrate 3 (rho family, small GTP binding protein Rac3)
ENSG00000118508	-1.06	6.49	1.40906E-05	8.19985E-05	RAB32, member RAS oncogene family
ENSG00000090376	-1.07	6.89	8.80764E-07	6.94255E-06	interleukin-1 receptor-associated kinase 3
ENSG00000105472	-1.07	8.83	7.79564E-06	4.86504E-05	C-type lectin domain family 11, member A
ENSG00000035115	-1.07	4.86	2.19899E-05	0.00012208	SH3 and SYLF domain containing 1
ENSG00000128050	-1.07	7.73	4.60771E-07	3.86373E-06	phosphoribosylaminoimidazole carboxylase, phosphoribosylaminoimidazole succinocarboxamide synthetase
ENSG00000039123	-1.07	6.24	8.02649E-07	6.39902E-06	superkiller viralicidic activity 2-like 2 (S. cerevisiae)
ENSG00000110075	-1.07	6.63	5.27641E-07	4.35837E-06	protein phosphatase 6, regulatory subunit 3
ENSG00000139437	-1.07	4.82	3.94026E-05	0.000204789	trichoplein, keratin filament binding
ENSG00000103319	-1.07	4.93	2.06234E-05	0.000115411	eukaryotic elongation factor 2 kinase
ENSG00000119707	-1.07	7.27	2.63877E-07	2.33203E-06	RNA binding motif protein 25
ENSG00000169660	-1.07	5.25	9.41957E-06	5.7567E-05	hexosaminidase (glycosyl hydrolase family 20, catalytic domain) containing
ENSG00000086504	-1.07	5.73	1.22364E-06	9.23923E-06	mitochondrial ribosomal protein L28
ENSG00000170606	-1.07	7.10	3.42924E-07	2.95199E-06	heat shock 70kDa protein 4
ENSG00000215440	-1.07	4.53	5.68039E-05	0.000284641	aminopeptidase-like 1
ENSG00000163528	-1.07	4.53	0.002564821	0.007928607	coiled-coil-helix-coiled-coil-helix domain containing 4
ENSG00000136463	-1.07	4.52	8.05663E-05	0.000386637	translational activator of mitochondrially encoded cytochrome c oxidase I
ENSG00000230124	-1.07	4.68	4.19501E-05	0.000217348	acyl-CoA binding domain containing 6
ENSG00000184428	-1.08	6.11	3.34342E-05	0.000177027	topoisomerase (DNA) I, mitochondrial
ENSG00000156261	-1.08	8.48	1.23986E-06	9.35637E-06	chaperonin containing TCP1, subunit 8 (theta)
ENSG00000145191	-1.08	6.44	1.02074E-06	7.89584E-06	eukaryotic translation initiation factor 2B, subunit 5 epsilon, 82kDa
ENSG00000106415	-1.08	4.35	0.000157556	0.0007004	glucocorticoid induced 1
ENSG00000108773	-1.08	6.10	9.5315E-07	7.4468E-06	K(lysine) acetyltransferase 2A
ENSG00000114030	-1.08	6.17	1.95824E-06	1.40735E-05	karyopherin alpha 1 (importin alpha 5)
ENSG00000175471	-1.08	5.88	9.70027E-06	5.91191E-05	multiple C2 domains, transmembrane 1
ENSG00000198160	-1.08	5.65	3.77631E-06	2.55098E-05	mesoderm induction early response 1, transcriptional regulator
ENSG00000135776	-1.08	5.90	2.09407E-06	1.49606E-05	ATP-binding cassette, sub-family B (MDR/TAP), member 10
ENSG00000160221	-1.08	7.48	1.91357E-07	1.74874E-06	chromosome 21 open reading frame 33
ENSG00000005801	-1.08	5.40	3.25519E-06	2.23535E-05	zinc finger protein 195
ENSG00000104522	-1.08	5.75	8.19445E-07	6.50557E-06	tissue specific transplantation antigen P35B
ENSG00000187961	-1.08	4.30	0.000135958	0.000614651	kelch-like family member 17
ENSG00000152234	-1.08	8.95	2.88088E-06	2.00109E-05	ATP synthase, H+ transporting, mitochondrial F1 complex, alpha subunit 1, cardiac muscle
ENSG00000184967	-1.08	4.43	8.10308E-05	0.000388725	nucleolar complex associated 4 homolog
ENSG00000196756	-1.09	5.66	1.05802E-06	8.14628E-06	small nucleolar RNA host gene 17
ENSG00000154174	-1.09	6.68	3.73765E-07	3.18853E-06	translocase of outer mitochondrial membrane 70 homolog A (S. cerevisiae)
ENSG00000109689	-1.09	4.98	8.90585E-06	5.47551E-05	stromal interaction molecule 2
ENSG00000101194	-1.09	6.05	1.15759E-06	8.79057E-06	solute carrier family 17 (vesicular nucleotide transporter), member 9
ENSG00000116489	-1.09	8.58	1.69297E-06	1.23407E-05	capping protein (actin filament) muscle Z-line, alpha 1
ENSG00000274523	-1.09	5.75	2.37754E-06	1.68232E-05	Williams-Beuren syndrome chromosome region 16
ENSG00000100220	-1.09	5.04	6.63807E-05	0.000326831	RNA 2',3'-cyclic phosphate and 5'-OH ligase
ENSG00000277278	-1.09	5.99	6.27432E-06	4.00218E-05	HECT and RLD domain containing E3 ubiquitin protein ligase 2

ENSG00000120685	-1.09	5.09	1.49394E-05	8.637E-05	proline and serine rich 1
ENSG00000130731	-1.09	5.24	3.14945E-06	2.16723E-05	chromosome 16 open reading frame 13
ENSG00000130935	-1.09	6.48	1.76317E-06	1.27892E-05	nucleolar protein 11
ENSG00000162607	-1.09	6.38	1.27664E-06	9.60666E-06	ubiquitin specific peptidase 1
ENSG00000147813	-1.09	6.28	5.22291E-07	4.31956E-06	nicotinate phosphoribosyltransferase
ENSG00000115419	-1.09	6.15	8.71613E-07	6.88268E-06	glutaminase
ENSG00000165699	-1.09	5.07	8.65547E-06	5.34136E-05	tuberous sclerosis 1
ENSG00000100216	-1.09	5.41	3.58377E-06	2.44192E-05	translocase of outer mitochondrial membrane 22 homolog (yeast)
ENSG00000173638	-1.09	7.27	0.000259147	0.001080717	solute carrier family 19 (folate transporter), member 1
ENSG00000110080	-1.09	6.11	4.14835E-06	2.76709E-05	ST3 beta-galactoside alpha-2,3-sialyltransferase 4
ENSG00000099804	-1.09	5.57	7.92181E-06	4.93681E-05	cell division cycle 34
ENSG00000275700	-1.09	5.60	1.37202E-06	1.02546E-05	apoptosis antagonizing transcription factor
ENSG00000134697	-1.09	6.37	6.43806E-07	5.25577E-06	guanine nucleotide binding protein-like 2 (nucleolar)
ENSG00000125912	-1.10	7.07	5.95061E-05	0.000296392	nicalin
ENSG00000004487	-1.10	6.60	3.99303E-06	2.68101E-05	lysine (K)-specific demethylase 1A
ENSG00000115827	-1.10	4.29	6.92354E-05	0.000339001	DDB1 and CUL4 associated factor 17
ENSG00000165733	-1.10	5.86	2.33173E-06	1.65522E-05	BMS1 ribosome biogenesis factor
ENSG00000167969	-1.10	4.43	5.92881E-05	0.000295527	enoyl-CoA delta isomerase 1
ENSG00000075240	-1.10	5.71	9.20484E-06	5.63584E-05	GRAM domain containing 4
ENSG00000164654	-1.10	4.54	4.44044E-05	0.000228992	missing oocyte, meiosis regulator, homolog (Drosophila)
ENSG00000121210	-1.10	4.96	1.00432E-05	6.1041E-05	KIAA0922
ENSG00000124784	-1.10	5.82	1.46444E-06	1.08658E-05	RIO kinase 1
ENSG00000075856	-1.10	4.80	0.000186596	0.000811025	squamous cell carcinoma antigen recognized by T cells 3
ENSG00000151849	-1.10	4.90	1.2569E-05	7.435E-05	centromere protein J
ENSG00000067533	-1.10	4.71	5.0678E-05	0.000257542	ribosomal RNA processing 15 homolog
ENSG00000120694	-1.10	7.21	8.17102E-05	0.000391277	heat shock 105kDa/110kDa protein 1
ENSG00000136682	-1.10	6.46	2.18275E-06	1.55606E-05	COBW domain containing 2
ENSG00000112208	-1.11	5.26	1.65282E-05	9.47714E-05	BCL2-associated athanogene 2
ENSG00000004809	-1.11	4.95	2.19527E-05	0.000121925	solute carrier family 22 (organic cation/carnitine transporter), member 16
ENSG00000164933	-1.11	5.37	1.50226E-06	1.11154E-05	solute carrier family 25 (mitochondrial folate carrier), member 32
ENSG00000118308	-1.11	5.83	0.001967613	0.00630263	lymphoid-restricted membrane protein
ENSG00000071859	-1.11	5.61	1.51465E-06	1.11759E-05	family with sequence similarity 50, member A
ENSG00000116459	-1.11	7.69	2.19135E-07	1.97743E-06	ATP synthase, H+ transporting, mitochondrial Fo complex, subunit B1
ENSG00000116133	-1.11	8.41	1.93093E-06	1.39073E-05	24-dehydrocholesterol reductase
ENSG00000174579	-1.11	5.60	1.28329E-06	9.64577E-06	male-specific lethal 2 homolog (Drosophila)
ENSG00000128059	-1.11	5.25	1.73085E-06	1.25754E-05	phosphoribosyl pyrophosphate amidotransferase
ENSG00000164050	-1.11	4.78	6.33709E-05	0.000313758	plexin B1
ENSG00000100714	-1.11	6.80	2.04892E-07	1.86029E-06	methylenetetrahydrofolate dehydrogenase (NADP+ dependent) 1, methenyltetrahydrofolate cyclohydrolase, formyltetrahydrofolate synthetase
ENSG00000138758	-1.11	6.17	4.10537E-07	3.46659E-06	septin 11
ENSG00000197774	-1.11	4.44	0.000984254	0.00346037	essential meiotic structure-specific endonuclease subunit 2
ENSG00000162384	-1.11	5.02	7.47133E-06	4.67583E-05	chromosome 1 open reading frame 123
ENSG00000103495	-1.11	8.36	3.9661E-06	2.66562E-05	MYC-associated zinc finger protein (purine-binding transcription factor)
ENSG00000024526	-1.12	5.01	0.002134052	0.006762332	DEP domain containing 1
ENSG00000077312	-1.12	6.48	1.48189E-06	1.09771E-05	small nuclear ribonucleoprotein polypeptide A
ENSG00000196547	-1.12	7.00	7.99237E-08	7.97076E-07	mannosidase, alpha, class 2A, member 2
ENSG00000099783	-1.12	8.40	3.64749E-07	3.11964E-06	heterogeneous nuclear ribonucleoprotein M
ENSG00000023734	-1.12	5.92	6.54944E-07	5.32501E-06	serine/threonine kinase receptor associated protein
ENSG00000106554	-1.12	6.69	1.6205E-06	1.1904E-05	coiled-coil-helix-coiled-coil-helix domain containing 3
ENSG00000115761	-1.12	5.12	2.89096E-06	2.00599E-05	nucleolar protein 10
ENSG00000164880	-1.12	6.88	1.14013E-07	1.09747E-06	integrator complex subunit 1
ENSG00000123505	-1.12	7.18	7.47142E-08	7.52471E-07	adenosylmethionine decarboxylase 1
ENSG00000130204	-1.12	6.94	1.62616E-06	1.19324E-05	translocase of outer mitochondrial membrane 40 homolog (yeast)
ENSG00000175376	-1.12	5.21	1.46118E-06	1.08538E-05	eukaryotic translation initiation factor 1A domain containing
ENSG00000079134	-1.12	5.60	8.08145E-07	6.43124E-06	THO complex 1
ENSG00000114770	-1.12	4.42	2.59891E-05	0.000141734	ATP-binding cassette, sub-family C (CFTR/MRP), member 5
ENSG00000157778	-1.12	5.09	4.80116E-06	3.13481E-05	proteasome (prosome, macropain) assembly chaperone 3
ENSG00000141456	-1.13	5.00	4.6023E-06	3.02407E-05	proline, glutamate and leucine rich protein 1
ENSG00000234032	-1.13	6.06	8.28639E-07	6.5707E-06	valyl-tRNA synthetase 2, mitochondrial
ENSG00000149115	-1.13	4.68	0.000135326	0.000612033	tankyrase 1 binding protein 1, 182kDa
ENSG00000006652	-1.13	5.54	1.80656E-05	0.000102655	interferon-related developmental regulator 1
ENSG00000155850	-1.13	4.65	0.000114212	0.000527688	solute carrier family 26 (anion exchanger), member 2
ENSG00000100359	-1.13	5.01	7.69562E-06	4.80714E-05	small G protein signaling modulator 3
ENSG00000187051	-1.13	4.62	5.05999E-05	0.000257342	ribosomal protein S19 binding protein 1
ENSG00000213465	-1.13	6.03	2.23076E-07	2.00754E-06	ADP-ribosylation factor-like 2
ENSG00000085415	-1.13	6.40	8.71968E-06	5.37456E-05	SEH1-like nucleoporin
ENSG00000168259	-1.13	6.15	1.04944E-06	8.10298E-06	DnaJ (Hsp40) homolog, subfamily C, member 7
ENSG00000148296	-1.13	5.84	1.01782E-06	7.88265E-06	surfeit 6
ENSG00000138698	-1.13	5.56	1.25224E-06	9.43525E-06	RAP1, GTP-GDP dissociation stimulator 1
ENSG00000184164	-1.13	5.57	3.00965E-05	0.000161611	cysteine-rich with EGF-like domains 2
ENSG00000176058	-1.13	4.21	0.001204948	0.004122288	taperin
ENSG00000065060	-1.13	5.19	5.18548E-06	3.36752E-05	UHRF1 binding protein 1
ENSG00000100722	-1.13	5.13	1.68084E-06	1.22726E-05	zinc finger CCCH-type containing 14

ENSG00000163257	-1.13	5.06	2.33811E-05	0.000128994	DDB1 and CUL4 associated factor 16
ENSG00000275176	-1.13	6.21	4.25291E-07	3.57978E-06	acetyl-CoA carboxylase alpha
ENSG00000107262	-1.13	6.28	6.82506E-06	4.31203E-05	BCL2-associated athanogene
ENSG00000168906	-1.13	8.90	1.19133E-06	9.02096E-06	methionine adenosyltransferase II, alpha
ENSG00000185104	-1.14	6.31	2.86965E-07	2.50768E-06	Fas (TNFRSF6) associated factor 1
ENSG00000187954	-1.14	5.70	3.82051E-07	3.25504E-06	cysteine/histidine-rich 1
ENSG00000158882	-1.14	5.19	2.48033E-06	1.74848E-05	translocase of outer mitochondrial membrane 40 homolog (yeast)-like
ENSG00000141385	-1.14	5.80	0.000115525	0.000532828	AFG3-like AAA ATPase 2
ENSG00000168944	-1.14	4.62	0.002453145	0.007629579	centrosomal protein 120kDa
ENSG00000119929	-1.14	4.65	2.96893E-05	0.000159617	cutC copper transporter
ENSG00000132953	-1.14	5.76	1.17184E-06	8.88858E-06	exportin 4
ENSG00000116120	-1.14	5.96	2.57899E-07	2.28375E-06	phenylalanyl-tRNA synthetase, beta subunit
ENSG00000180198	-1.14	7.00	8.09196E-08	8.05796E-07	regulator of chromosome condensation 1
ENSG00000127837	-1.14	6.00	3.92373E-07	3.33239E-06	angio-associated, migratory cell protein
ENSG00000138399	-1.14	6.00	1.43235E-07	1.34844E-06	FAST kinase domains 1
ENSG00000130024	-1.14	7.69	0.000112996	0.000523345	PHD finger protein 10
ENSG00000087191	-1.14	7.28	3.67454E-08	3.92089E-07	proteasome (prosome, macropain) 26S subunit, ATPase, 5
ENSG00000116791	-1.14	4.29	8.83294E-05	0.000418744	crystallin, zeta (quinone reductase)
ENSG00000166986	-1.14	7.02	0.000577181	0.002175325	methionyl-tRNA synthetase
ENSG00000112640	-1.14	5.77	1.04996E-06	8.10298E-06	protein phosphatase 2, regulatory subunit B', delta
ENSG00000120948	-1.15	7.72	3.50922E-07	3.01302E-06	TAR DNA binding protein
ENSG00000146701	-1.15	7.75	7.79612E-08	7.81614E-07	malate dehydrogenase 2, NAD (mitochondrial)
ENSG00000130810	-1.15	5.32	9.09596E-06	5.58205E-05	peter pan homolog (Drosophila)
ENSG00000173457	-1.15	6.54	2.46862E-07	2.19628E-06	protein phosphatase 1, regulatory (inhibitor) subunit 14B
ENSG00000182162	-1.15	6.47	4.69972E-06	3.07614E-05	purinergic receptor P2Y, G-protein coupled, 8
ENSG00000008838	-1.15	6.10	5.71719E-07	4.70198E-06	mediator complex subunit 24
ENSG00000100029	-1.15	5.43	7.09328E-07	5.71683E-06	pescadillo ribosomal biogenesis factor 1
ENSG00000162852	-1.15	4.85	2.30858E-05	0.000127472	consortin, connexin sorting protein
ENSG00000169718	-1.15	7.55	1.08834E-06	8.34579E-06	dihydrouridine synthase 1-like
ENSG00000116898	-1.15	5.76	2.06994E-06	1.48201E-05	mitochondrial ribosomal protein S15
ENSG00000083750	-1.15	4.61	0.000537773	0.002039544	Ras-related GTP binding B
ENSG00000145907	-1.15	7.49	2.7187E-07	2.38835E-06	GTPase activating protein (SH3 domain) binding protein 1
ENSG00000163539	-1.15	6.16	1.32819E-07	1.26021E-06	cytoplasmic linker associated protein 2
ENSG00000104164	-1.15	5.50	2.75746E-05	0.000149642	biogenesis of lysosomal organelles complex-1, subunit 6, pallidin
ENSG00000012983	-1.15	4.95	5.45518E-06	3.52885E-05	mitogen-activated protein kinase kinase kinase 5
ENSG00000166411	-1.15	5.95	2.57087E-07	2.27809E-06	isocitrate dehydrogenase 3 (NAD+) alpha
ENSG00000128191	-1.16	5.17	1.68046E-06	1.22726E-05	DGCR8 microprocessor complex subunit
ENSG00000125247	-1.16	5.23	9.69706E-07	7.55836E-06	transmembrane and tetratricopeptide repeat containing 4
ENSG00000138668	-1.16	8.62	3.95177E-07	3.35181E-06	heterogeneous nuclear ribonucleoprotein D (AU-rich element RNA binding protein 1, 37kDa)
ENSG00000177830	-1.16	6.51	8.86552E-08	8.73004E-07	chitinase domain containing 1
ENSG00000129351	-1.16	8.68	3.17226E-07	2.74502E-06	interleukin enhancer binding factor 3, 90kDa
ENSG00000100410	-1.16	4.36	1.88179E-05	0.000106474	PHD finger protein 5A
ENSG00000138442	-1.16	6.22	1.25573E-07	1.19917E-06	WD repeat domain 12
ENSG00000124181	-1.16	4.87	6.89287E-06	4.34867E-05	phospholipase C, gamma 1
ENSG00000105063	-1.16	8.16	1.12765E-06	8.5976E-06	protein phosphatase 6, regulatory subunit 1
ENSG00000084090	-1.16	7.87	1.48387E-07	1.39398E-06	StAR-related lipid transfer (START) domain containing 7
ENSG00000150768	-1.16	6.35	1.67919E-07	1.55762E-06	dihydrolipoamide S-acetyltransferase
ENSG00000162694	-1.16	4.96	3.0006E-06	2.0734E-05	exostosin-like glycosyltransferase 2
ENSG00000037474	-1.16	6.27	1.97637E-07	1.80057E-06	NOP2/Sun RNA methyltransferase family, member 2
ENSG00000197299	-1.16	5.32	4.8062E-07	4.0099E-06	Bloom syndrome, RecQ helicase-like
ENSG00000185883	-1.17	6.75	2.64963E-07	2.34006E-06	ATPase, H+ transporting, lysosomal 16kDa, V0 subunit c
ENSG00000100060	-1.17	6.08	3.70434E-06	2.50619E-05	MFNG O-fucosylpeptide 3-beta-N-acetylglucosaminyltransferase
ENSG00000135624	-1.17	8.17	2.53201E-07	2.24815E-06	chaperonin containing TCP1, subunit 7 (eta)
ENSG00000116560	-1.17	8.79	6.67465E-07	5.42223E-06	splicing factor proline/glutamine-rich
ENSG00000196591	-1.17	7.36	4.52724E-08	4.73932E-07	histone deacetylase 2
ENSG00000135372	-1.17	6.73	2.83546E-08	3.07499E-07	N-acetyltransferase 10 (GCN5-related)
ENSG00000169710	-1.17	9.19	3.44212E-05	0.000181384	fatty acid synthase
ENSG00000103202	-1.17	6.09	1.59477E-06	1.17279E-05	NME/NM23 nucleoside diphosphate kinase 4
ENSG00000183431	-1.17	6.81	2.29643E-08	2.54873E-07	splicing factor 3a, subunit 3, 60kDa
ENSG00000239285	-1.17	4.62	8.83641E-06	5.44038E-05	lymphocyte antigen 6 complex, locus G5B
ENSG00000084073	-1.17	6.00	1.64459E-07	1.52981E-06	zinc metallopeptidase STE24
ENSG00000152117	-1.17	5.41	9.8172E-07	7.63854E-06	AC093838.4
ENSG00000109586	-1.17	6.04	0.000411366	0.001614092	polypeptide N-acetylgalactosaminyltransferase 7
ENSG00000138709	-1.18	4.30	2.05542E-05	0.000115121	La ribonucleoprotein domain family, member 1B
ENSG00000085998	-1.18	4.68	3.69346E-05	0.000192943	protein O-linked mannose N-acetylglucosaminyltransferase 1 (beta 1,2-)
ENSG00000084754	-1.18	8.56	1.61257E-07	1.50213E-06	hydroxyacyl-CoA dehydrogenase/3-ketoacyl-CoA thiolase/enoyl-CoA hydratase (trifunctional protein), alpha subunit
ENSG00000110200	-1.18	4.32	0.000180333	0.00078845	anaphase promoting complex subunit 15
ENSG00000145494	-1.18	5.46	2.28544E-07	2.05257E-06	NADH dehydrogenase (ubiquinone) Fe-S protein 6, 13kDa (NADH-coenzyme Q reductase)
ENSG00000274081	-1.18	6.64	1.29474E-06	9.72631E-06	poly-U binding splicing factor 60KDa
ENSG00000105176	-1.18	5.85	1.06261E-06	8.17214E-06	URI1, prefoldin-like chaperone

ENSG00000115241	-1.18	7.36	2.56572E-08	2.82166E-07	protein phosphatase, Mg2+/Mn2+ dependent, 1G
ENSG00000146731	-1.18	8.17	5.10328E-08	5.2964E-07	chaperonin containing TCP1, subunit 6A (zeta 1)
ENSG00000232045	-1.18	6.64	8.91985E-08	8.77703E-07	euchromatic histone-lysine N-methyltransferase 2
ENSG00000077097	-1.18	8.87	2.73271E-07	2.39908E-06	topoisomerase (DNA) II beta 180kDa
ENSG00000163468	-1.18	8.51	2.67315E-07	2.35614E-06	chaperonin containing TCP1, subunit 3 (gamma)
ENSG00000128626	-1.19	5.14	6.29146E-05	0.000311731	mitochondrial ribosomal protein S12
ENSG00000137449	-1.19	4.24	0.000449669	0.001746314	cytoplasmic polyadenylation element binding protein 2
ENSG00000130764	-1.19	5.93	1.50407E-07	1.41095E-06	leucine rich repeat containing 47
ENSG00000231502	-1.19	5.51	2.41906E-06	1.70619E-05	LSM2 homolog, U6 small nuclear RNA and mRNA degradation associated
ENSG00000141577	-1.19	4.63	4.81954E-06	3.14371E-05	centrosomal protein 131kDa
ENSG00000075239	-1.19	6.24	1.87961E-07	1.72306E-06	acetyl-CoA acetyltransferase 1
ENSG00000104903	-1.19	6.13	1.10333E-06	8.43642E-06	lymphoblastic leukemia associated hematopoiesis regulator 1
ENSG00000163597	-1.19	6.89	1.3652E-08	1.60653E-07	small nucleolar RNA host gene 16
ENSG00000108395	-1.19	4.65	1.02564E-05	6.20809E-05	tripartite motif containing 37
ENSG00000276681	-1.19	8.13	6.61438E-08	6.71762E-07	leukocyte receptor cluster (LRC) member 8
ENSG00000092529	-1.19	4.38	2.79626E-05	0.000151376	calpain 3
ENSG00000187630	-1.19	4.51	1.4134E-05	8.2143E-05	dehydrogenase/reductase (SDR family) member 4 like 2
ENSG00000011485	-1.20	6.14	6.28229E-08	6.40977E-07	protein phosphatase 5, catalytic subunit
ENSG00000185803	-1.20	5.82	8.85408E-06	5.44873E-05	solute carrier family 52 (riboflavin transporter), member 2
ENSG00000204843	-1.20	6.85	1.76106E-08	2.00827E-07	dynactin 1
ENSG00000152990	-1.20	4.76	7.2206E-06	4.54247E-05	adhesion G protein-coupled receptor A3
ENSG00000107581	-1.20	8.41	9.46648E-08	9.25317E-07	eukaryotic translation initiation factor 3, subunit A
ENSG00000235125	-1.20	4.33	5.3495E-05	0.000269792	nuclear factor of kappa light polypeptide gene enhancer in B-cells inhibitor-like 1
ENSG00000239900	-1.20	5.33	1.35582E-07	1.28368E-06	adenylosuccinate lyase
ENSG00000117448	-1.20	6.70	1.12819E-08	1.35648E-07	aldo-keto reductase family 1, member A1 (aldehyde reductase)
ENSG00000224531	-1.21	4.47	1.13865E-05	6.81751E-05	small integral membrane protein 13
ENSG00000091527	-1.21	7.75	7.11929E-08	7.19189E-07	CDV3 homolog (mouse)
ENSG00000196642	-1.21	7.14	3.28447E-07	2.84026E-06	RAB, member RAS oncogene family-like 6
ENSG00000166669	-1.21	4.57	1.7784E-05	0.000101142	activating transcription factor 7 interacting protein 2
ENSG00000155189	-1.21	6.05	1.88607E-07	1.72779E-06	1-acylglycerol-3-phosphate O-acyltransferase 5
ENSG00000140474	-1.21	6.35	2.9833E-08	3.23268E-07	unc-51 like kinase 3
ENSG00000086848	-1.21	4.22	1.19337E-05	7.10987E-05	ALG9, alpha-1,2-mannosyltransferase
ENSG00000100448	-1.21	11.92	0.000287631	0.001181315	cathepsin G
ENSG00000184465	-1.21	5.00	2.29939E-05	0.000127122	WD repeat domain 27
ENSG00000125445	-1.21	6.29	1.49949E-07	1.40766E-06	mitochondrial ribosomal protein S7
ENSG00000170632	-1.21	5.44	2.31614E-06	1.64585E-05	armadillo repeat containing 10
ENSG00000198888	-1.21	10.88	0.000465925	0.001798397	mitochondrially encoded NADH dehydrogenase 1
ENSG00000156876	-1.21	4.48	1.35362E-05	7.92589E-05	SAS-6 centriolar assembly protein
ENSG00000205903	-1.22	4.25	0.000150452	0.000672879	zinc finger protein 316
ENSG00000113360	-1.22	5.57	2.10376E-07	1.90558E-06	drosha, ribonuclease type III
ENSG00000112290	-1.22	5.08	2.85739E-06	1.98581E-05	WAS protein family, member 1
ENSG00000051128	-1.22	6.06	3.99127E-08	4.21816E-07	homer scaffolding protein 3
ENSG00000125630	-1.22	5.62	5.20352E-08	5.38779E-07	polymerase (RNA) I polypeptide B, 128kDa
ENSG00000179304	-1.22	4.94	1.72737E-06	1.25603E-05	family with sequence similarity 156, member B
ENSG00000148840	-1.22	5.69	1.96159E-06	1.40823E-05	peroxisome proliferator-activated receptor gamma, coactivator-related 1
ENSG00000247982	-1.22	7.28	1.40335E-08	1.64559E-07	long intergenic non-protein coding RNA 926
ENSG00000075415	-1.22	7.12	3.94229E-09	5.18631E-08	solute carrier family 25 (mitochondrial carrier; phosphate carrier), member 3
ENSG00000256269	-1.22	5.22	2.8365E-07	2.48198E-06	hydroxymethylbilane synthase
ENSG00000107833	-1.22	5.04	7.45233E-07	5.99165E-06	nucleophosmin/nucleoplasmin 3
ENSG00000167136	-1.22	4.32	1.83108E-05	0.000103915	endonuclease G
ENSG00000109534	-1.22	5.08	3.87393E-07	3.29632E-06	GAR1 homolog, ribonucleoprotein
ENSG00000065809	-1.22	8.00	1.50999E-08	1.75207E-07	family with sequence similarity 107, member B
ENSG00000274811	-1.22	6.65	7.91774E-08	7.90258E-07	CLPTM1-like
ENSG00000163938	-1.22	7.05	1.21177E-08	1.44131E-07	guanine nucleotide binding protein-like 3 (nucleolar)
ENSG00000169682	-1.23	5.55	4.50924E-08	4.7242E-07	spinster homolog 1 (Drosophila)
ENSG00000197451	-1.23	7.50	2.25358E-08	2.50327E-07	heterogeneous nuclear ribonucleoprotein A/B
ENSG00000185420	-1.23	5.30	2.30156E-05	0.000127189	SET and MYND domain containing 3
ENSG00000141858	-1.23	5.17	4.3714E-06	2.89406E-05	sterile alpha motif domain containing 1
ENSG00000085999	-1.23	4.32	8.47648E-06	5.24554E-05	RAD54-like (S. cerevisiae)
ENSG00000281184	-1.23	7.20	1.80658E-09	2.53494E-08	proteasome (prosome, macropain) subunit, beta type, 1
ENSG00000108651	-1.23	5.94	1.32443E-07	1.25755E-06	UTP6, small subunit (SSU) processome component, homolog (yeast)
ENSG00000100726	-1.23	6.24	6.58508E-08	6.69298E-07	telomere maintenance 2
ENSG00000164151	-1.23	4.59	3.42439E-06	2.34306E-05	interactor of little elongation complex ELL subunit 1
ENSG00000164045	-1.24	5.00	1.20934E-05	7.19208E-05	cell division cycle 25A
ENSG00000212907	-1.24	9.34	0.001989879	0.006363203	mitochondrially encoded NADH dehydrogenase 4L
ENSG00000233369	-1.24	4.45	7.3169E-05	0.000355898	general transcription factor IIi, pseudogene 4
ENSG00000182568	-1.24	6.58	1.395E-08	1.63724E-07	SATB homeobox 1
ENSG00000136950	-1.24	6.12	5.01491E-07	4.15789E-06	actin related protein 2/3 complex, subunit 5-like
ENSG00000168101	-1.24	4.93	3.10003E-06	2.13654E-05	nudix (nucleoside diphosphate linked moiety X)-type motif 16-like 1
ENSG00000152193	-1.24	5.35	7.12902E-08	7.19624E-07	ring finger protein 219
ENSG00000270647	-1.24	7.13	9.52467E-08	9.30319E-07	TAF15 RNA polymerase II, TATA box binding protein (TBP)-associated factor, 68kDa
ENSG00000140650	-1.24	4.68	1.25964E-05	7.44784E-05	phosphomannomutase 2

ENSG00000106263	-1.24	8.00	9.6783E-07	7.54816E-06	eukaryotic translation initiation factor 3, subunit B
ENSG00000049449	-1.25	6.24	2.15932E-08	2.40864E-07	reticulocalbin 1, EF-hand calcium binding domain
ENSG00000066044	-1.25	6.87	4.95795E-09	6.40193E-08	ELAV like RNA binding protein 1
ENSG00000165684	-1.25	5.08	2.89156E-07	2.52517E-06	small nuclear RNA activating complex, polypeptide 4, 190kDa
ENSG00000111641	-1.25	5.66	2.15273E-08	2.40331E-07	NOP2 nucleolar protein
ENSG00000143952	-1.25	4.97	5.20368E-07	4.30634E-06	vacuolar protein sorting 54 homolog (S. cerevisiae)
ENSG00000058600	-1.25	5.24	4.01894E-07	3.40225E-06	polymerase (RNA) III (DNA directed) polypeptide E (80kD)
ENSG00000167113	-1.25	4.74	1.41098E-06	1.05103E-05	coenzyme Q4
ENSG00000264364	-1.25	6.33	5.52913E-08	5.69384E-07	dynein, light chain, LC8-type 2
ENSG00000104529	-1.25	7.83	1.16135E-08	1.38756E-07	eukaryotic translation elongation factor 1 delta (guanine nucleotide exchange protein)
ENSG00000165874	-1.25	5.72	8.92377E-07	7.02575E-06	family with sequence similarity 35, member B, pseudogene
ENSG00000165271	-1.26	5.86	2.67456E-05	0.000145679	nucleolar protein 6 (RNA-associated)
ENSG00000161618	-1.26	5.73	2.05845E-08	2.3097E-07	aldehyde dehydrogenase 16 family, member A1
ENSG00000134215	-1.26	4.95	3.5154E-07	3.01638E-06	vav 3 guanine nucleotide exchange factor
ENSG00000124571	-1.26	6.09	2.25934E-07	2.03125E-06	exportin 5
ENSG00000104907	-1.26	5.89	1.1749E-07	1.12974E-06	tRNA methyltransferase 1 homolog (S. cerevisiae)
ENSG00000165097	-1.26	5.26	1.48145E-07	1.39269E-06	lysine (K)-specific demethylase 1B
ENSG00000107949	-1.26	6.28	7.54687E-09	9.36235E-08	BRCA2 and CDKN1A interacting protein
ENSG00000116221	-1.26	6.58	4.19577E-08	4.4167E-07	mitochondrial ribosomal protein L37
ENSG00000141759	-1.26	5.69	3.69231E-06	2.50171E-05	thioredoxin-like 4A
ENSG00000090447	-1.26	4.45	2.33182E-06	1.65522E-05	transcription factor AP-4 (activating enhancer binding protein 4)
ENSG00000036549	-1.26	5.62	1.85204E-08	2.09939E-07	zinc finger, ZZ-type containing 3
ENSG00000213593	-1.27	6.89	1.08957E-08	1.31481E-07	thioredoxin-related transmembrane protein 2
ENSG00000204149	-1.27	5.66	1.63048E-07	1.51774E-06	ArfGAP with GTPase domain, ankyrin repeat and PH domain 6
ENSG00000031003	-1.27	5.05	2.89907E-07	2.53006E-06	family with sequence similarity 13, member B
ENSG00000100522	-1.27	4.48	4.62227E-05	0.000237446	glucosamine-phosphate N-acetyltransferase 1
ENSG00000167702	-1.27	4.77	1.01808E-06	7.88265E-06	kinesin family member C2
ENSG00000095906	-1.27	5.14	4.14613E-06	2.76701E-05	nucleotide binding protein 2
ENSG00000028310	-1.27	5.35	5.36826E-08	5.53677E-07	bromodomain containing 9
ENSG00000243725	-1.27	4.71	1.14959E-06	8.73475E-06	tetratricopeptide repeat domain 4
ENSG00000156697	-1.27	4.50	1.13832E-05	6.81751E-05	UTP14, U3 small nucleolar ribonucleoprotein, homolog A (yeast)
ENSG00000115232	-1.27	7.58	1.76033E-09	2.47528E-08	integrin, alpha 4 (antigen CD49D, alpha 4 subunit of VLA-4 receptor)
ENSG00000087087	-1.27	7.43	1.021E-09	1.49095E-08	serrate, RNA effector molecule
ENSG00000086200	-1.27	4.42	3.88312E-06	2.61382E-05	importin 11
ENSG00000188352	-1.27	4.75	6.5312E-06	4.14413E-05	focadhesin
ENSG00000164818	-1.27	5.36	6.31134E-08	6.42953E-07	dynein, axonemal, assembly factor 5
ENSG00000134748	-1.27	5.83	1.59041E-08	1.83253E-07	pre-mRNA processing factor 38A
ENSG00000138028	-1.27	4.31	6.483E-06	4.11748E-05	cell growth regulator with EF-hand domain 1
ENSG00000255587	-1.28	5.61	1.65964E-08	1.90078E-07	RAB44, member RAS oncogene family
ENSG00000145725	-1.28	6.15	4.34072E-09	5.67113E-08	diphosphoinositol pentakisphosphate kinase 2
ENSG00000198824	-1.28	6.17	4.84261E-09	6.26519E-08	chromosome alignment maintaining phosphoprotein 1
ENSG00000152520	-1.28	10.08	1.6124E-05	9.25757E-05	PAN3 poly(A) specific ribonuclease subunit
ENSG00000206053	-1.28	6.15	8.07649E-09	9.98022E-08	hematological and neurological expressed 1-like
ENSG00000007376	-1.28	5.03	6.96751E-05	0.000340651	RNA pseudouridylate synthase domain containing 1
ENSG00000150401	-1.28	4.86	6.68016E-07	5.42339E-06	DCN1, defective in cullin neddylation 1, domain containing 2
ENSG00000087495	-1.28	5.86	1.23229E-07	1.17934E-06	phosphatase and actin regulator 3
ENSG00000099899	-1.28	5.04	1.32051E-06	9.89751E-06	tRNA methyltransferase 2 homolog A (S. cerevisiae)
ENSG00000161036	-1.28	4.55	8.93609E-06	5.49156E-05	leucine-rich repeats and WD repeat domain containing 1
ENSG00000197006	-1.28	7.26	1.51339E-08	1.75447E-07	methyltransferase like 9
ENSG00000142864	-1.28	9.19	1.12584E-07	1.0845E-06	SERPINE1 mRNA binding protein 1
ENSG00000187796	-1.29	6.35	7.87732E-08	7.8797E-07	caspase recruitment domain family, member 9
ENSG00000169957	-1.29	5.11	3.38843E-07	2.91876E-06	zinc finger protein 768
ENSG00000198648	-1.29	4.68	1.51359E-06	1.11743E-05	serine threonine kinase 39
ENSG00000167700	-1.29	4.42	2.64426E-06	1.85518E-05	major facilitator superfamily domain containing 3
ENSG00000134684	-1.29	7.79	1.69469E-05	9.70043E-05	tyrosyl-tRNA synthetase
ENSG00000007923	-1.29	6.14	4.20164E-06	2.79421E-05	DnaJ (Hsp40) homolog, subfamily C, member 11
ENSG00000183137	-1.29	4.45	3.64452E-05	0.000190762	centrosomal protein 57kDa-like 1
ENSG00000153395	-1.29	6.60	1.59177E-08	1.83253E-07	lysophosphatidylcholine acyltransferase 1
ENSG00000113368	-1.30	7.83	2.20816E-08	2.45693E-07	lamin B1
ENSG00000151247	-1.30	6.27	6.47934E-09	8.14458E-08	eukaryotic translation initiation factor 4E
ENSG00000136379	-1.30	4.76	4.44844E-06	2.93628E-05	abhydrolase domain containing 17C
ENSG00000092820	-1.30	7.41	3.74195E-09	4.94235E-08	ezrin
ENSG00000151835	-1.30	5.93	2.32753E-08	2.58109E-07	sacsin molecular chaperone
ENSG00000048162	-1.30	5.65	2.71437E-08	2.96792E-07	NOP16 nucleolar protein
ENSG00000162402	-1.30	6.03	3.41987E-09	4.54408E-08	ubiquitin specific peptidase 24
ENSG00000086475	-1.30	5.02	6.48258E-07	5.28237E-06	selenophosphate synthetase 1
ENSG00000188976	-1.30	6.78	1.19779E-09	1.72446E-08	NOC2-like nucleolar associated transcriptional repressor
ENSG00000167792	-1.30	6.88	1.0137E-09	1.48355E-08	NADH dehydrogenase (ubiquinone) flavoprotein 1, 51kDa
ENSG00000104884	-1.30	5.96	8.26021E-09	1.01618E-07	excision repair cross-complementation group 2
ENSG00000230903	-1.30	5.11	1.72783E-06	1.25603E-05	RPL9P8
ENSG00000100890	-1.30	5.76	5.63671E-08	5.79115E-07	KIAA0391
ENSG00000188452	-1.31	6.47	2.29983E-09	3.15046E-08	ceramide kinase-like

ENSG00000076248	-1.31	4.82	1.35353E-05	7.92589E-05	uracil DNA glycosylase
ENSG00000165661	-1.31	4.87	2.81813E-06	1.9647E-05	quiescin Q6 sulfhydryl oxidase 2
ENSG00000250067	-1.31	6.60	2.8501E-06	1.9831E-05	YjeF N-terminal domain containing 3
ENSG00000131323	-1.31	5.22	1.31045E-05	7.70941E-05	TNF receptor-associated factor 3
ENSG00000070785	-1.31	4.52	2.38861E-06	1.68675E-05	eukaryotic translation initiation factor 2B, subunit 3 gamma, 58kDa
ENSG00000057757	-1.31	5.37	8.08252E-09	9.98022E-08	PITH (C-terminal proteasome-interacting domain of thioredoxin-like) domain containing 1
ENSG00000135316	-1.31	8.20	2.58565E-09	3.52754E-08	synaptotagmin binding, cytoplasmic RNA interacting protein
ENSG00000122965	-1.31	4.57	6.75304E-06	4.27263E-05	RNA binding motif protein 19
ENSG00000181472	-1.31	4.87	2.72142E-06	1.90528E-05	zinc finger and BTB domain containing 2
ENSG00000125485	-1.31	4.50	3.76663E-05	0.000196457	DEAD (Asp-Glu-Ala-Asp) box polypeptide 31
ENSG00000135596	-1.32	8.02	8.56567E-08	8.47249E-07	microtubule associated monooxygenase, calponin and LIM domain containing 1
ENSG00000135763	-1.32	4.51	1.74427E-05	9.94313E-05	URB2 ribosome biogenesis 2 homolog (S. cerevisiae)
ENSG00000228253	-1.32	9.43	0.000255894	0.001069167	MT-ATP8
ENSG00000139842	-1.32	7.88	1.13566E-09	1.64034E-08	cullin 4A
ENSG00000033011	-1.32	4.84	7.83738E-07	6.26789E-06	ALG1, chitobiosyldiphosphodolichol beta-mannosyltransferase
ENSG00000237550	-1.32	5.14	1.37853E-05	8.03982E-05	RPL9P9
ENSG00000146540	-1.33	4.93	8.36628E-08	8.30621E-07	chromosome 7 open reading frame 50
ENSG00000099901	-1.33	6.32	3.88308E-06	2.61382E-05	RAN binding protein 1
ENSG00000107815	-1.33	4.58	6.62475E-05	0.000326483	chromosome 10 open reading frame 2
ENSG00000075702	-1.33	5.54	7.6867E-09	9.52692E-08	WD repeat domain 62
ENSG00000101220	-1.33	5.25	1.57729E-08	1.8206E-07	chromosome 20 open reading frame 27
ENSG00000112787	-1.33	6.02	1.51146E-06	1.11648E-05	fibrosin-like 1
ENSG00000122566	-1.33	10.26	4.97356E-06	3.24258E-05	heterogeneous nuclear ribonucleoprotein A2/B1
ENSG00000072274	-1.33	8.63	4.84055E-05	0.000247462	transferrin receptor
ENSG00000148300	-1.33	5.52	9.28226E-07	7.28208E-06	REX4 homolog, 3'-5' exonuclease
ENSG00000099949	-1.33	4.38	1.30546E-06	9.80126E-06	leucine-zipper-like transcription regulator 1
ENSG00000174744	-1.33	4.71	4.13445E-07	3.48765E-06	breast cancer metastasis suppressor 1
ENSG00000164163	-1.33	6.63	4.28871E-09	5.6087E-08	ATP-binding cassette, sub-family E (OABP), member 1
ENSG00000110042	-1.33	5.52	0.000759449	0.002775585	deltex 4, E3 ubiquitin ligase
ENSG00000138074	-1.33	6.15	2.86026E-07	2.50113E-06	solute carrier family 5 (sodium/multivitamin and iodide cotransporter), member 6
ENSG00000165724	-1.34	5.14	2.22896E-06	1.58644E-05	zinc finger, MYND-type containing 19
ENSG00000136718	-1.34	5.47	8.80458E-08	8.68291E-07	IMP4, U3 small nucleolar ribonucleoprotein
ENSG00000197498	-1.34	5.70	2.89453E-09	3.89675E-08	ribosome production factor 2 homolog
ENSG00000154277	-1.34	4.81	3.5773E-07	3.06356E-06	ubiquitin carboxyl-terminal esterase L1 (ubiquitin thiolesterase)
ENSG00000172232	-1.34	10.12	4.33128E-06	2.86893E-05	azurocidin 1
ENSG00000177225	-1.34	6.46	7.16908E-10	1.09006E-08	Parkinson disease 7 domain containing 1
ENSG00000125910	-1.34	4.26	5.97515E-05	0.000297502	sphingosine-1-phosphate receptor 4
ENSG00000162408	-1.34	5.08	3.60141E-08	3.85214E-07	nucleolar protein 9
ENSG00000117450	-1.34	8.78	1.15643E-08	1.38417E-07	peroxiredoxin 1
ENSG00000033050	-1.34	6.34	8.9433E-08	8.79359E-07	ATP-binding cassette, sub-family F (GCN20), member 2
ENSG00000142875	-1.34	4.86	1.19744E-05	7.12773E-05	protein kinase, cAMP-dependent, catalytic, beta
ENSG00000133422	-1.34	5.32	6.96673E-09	8.68221E-08	MORC family CW-type zinc finger 2
ENSG00000065978	-1.34	10.01	1.951E-05	0.000109922	Y box binding protein 1
ENSG00000168066	-1.34	8.12	1.99092E-09	2.75574E-08	splicing factor 1
ENSG00000048052	-1.34	4.58	2.30348E-05	0.000127242	histone deacetylase 9
ENSG00000142409	-1.35	5.11	6.43791E-08	6.54842E-07	zinc finger protein 787
ENSG00000148334	-1.35	4.73	8.03587E-06	4.99382E-05	prostaglandin E synthase 2
ENSG00000163291	-1.35	4.98	1.36599E-06	1.02153E-05	progestin and adipoQ receptor family member III
ENSG00000155393	-1.35	4.94	4.72362E-08	4.92161E-07	HEAT repeat containing 3
ENSG00000108106	-1.35	6.82	1.98338E-09	2.74816E-08	ubiquitin-conjugating enzyme E2S
ENSG00000174547	-1.35	5.60	3.05736E-08	3.31022E-07	mitochondrial ribosomal protein L11
ENSG00000144867	-1.35	6.13	1.19345E-07	1.14464E-06	signal recognition particle receptor, B subunit
ENSG00000184207	-1.35	5.29	8.01346E-07	6.39247E-06	phosphoglycolate phosphatase
ENSG00000130638	-1.35	6.06	4.24564E-09	5.55785E-08	ataxin 10
ENSG00000001084	-1.35	4.81	1.77387E-07	1.63744E-06	glutamate-cysteine ligase, catalytic subunit
ENSG00000228875	-1.35	7.28	4.76626E-10	7.46077E-09	casein kinase 2, beta polypeptide
ENSG00000104341	-1.35	6.96	2.14257E-10	3.623E-09	lysosomal protein transmembrane 4 beta
ENSG00000144354	-1.35	6.60	3.57994E-08	3.83536E-07	cell division cycle associated 7
ENSG00000117419	-1.35	4.25	0.000154965	0.000690461	ERI1 exoribonuclease family member 3
ENSG00000184281	-1.35	4.69	2.3944E-07	2.13741E-06	tumor suppressing subtransferable candidate 4
ENSG00000173145	-1.36	4.97	2.66198E-07	2.34941E-06	NOC3-like DNA replication regulator
ENSG00000073060	-1.36	5.52	1.11337E-07	1.07405E-06	scavenger receptor class B, member 1
ENSG00000156502	-1.36	4.99	2.98994E-07	2.60423E-06	suppressor of var1, 3-like 1 (S. cerevisiae)
ENSG00000272325	-1.36	7.00	7.574E-11	1.37159E-09	nudix (nucleoside diphosphate linked moiety X)-type motif 3
ENSG00000096384	-1.36	10.13	1.20581E-06	9.1254E-06	heat shock protein 90kDa alpha (cytosolic), class B member 1
ENSG00000269713	-1.36	4.35	1.10785E-06	8.46613E-06	neuroblastoma breakpoint family, member 9
ENSG00000213339	-1.36	4.73	5.96965E-07	4.89748E-06	queuine tRNA-ribosyltransferase 1
ENSG00000145220	-1.37	5.79	1.23018E-09	1.76534E-08	Ly1 antibody reactive
ENSG00000006744	-1.37	5.54	3.38669E-09	4.50571E-08	elaC ribonuclease Z 2
ENSG00000005844	-1.37	8.01	1.39754E-06	1.04219E-05	integrin, alpha L (antigen CD11A (p180), lymphocyte function-associated antigen 1; alpha polypeptide)
ENSG00000050393	-1.37	4.54	7.74291E-07	6.20274E-06	mitochondrial calcium uniporter regulator 1

ENSG00000165138	-1.37	4.55	2.00774E-07	1.8279E-06	ankyrin repeat and sterile alpha motif domain containing 6
ENSG00000105447	-1.37	5.45	1.74876E-08	1.99596E-07	glutamate-rich WD repeat containing 1
ENSG00000185198	-1.38	5.06	2.77601E-08	3.02039E-07	protease, serine, 57
ENSG00000135002	-1.38	4.73	3.87844E-07	3.29804E-06	riboflavin kinase
ENSG00000068383	-1.38	4.75	8.34015E-08	8.29267E-07	inositol polyphosphate-5-phosphatase, 40kDa
ENSG00000149557	-1.38	7.62	1.55567E-10	2.69231E-09	fasciculation and elongation protein zeta 1 (zygin I)
ENSG00000235569	-1.38	6.19	5.21788E-08	5.39845E-07	mutS homolog 5
ENSG00000124587	-1.38	5.29	3.46353E-09	4.58831E-08	peroxisomal biogenesis factor 6
ENSG00000005379	-1.38	8.45	2.67721E-08	2.93212E-07	benzodiazepine receptor (peripheral) associated protein 1
ENSG00000144381	-1.38	9.02	5.11403E-09	6.54767E-08	heat shock 60kDa protein 1 (chaperonin)
ENSG00000110619	-1.38	6.31	0.001596487	0.005261116	cysteinyl-tRNA synthetase
ENSG00000146433	-1.39	5.74	3.22542E-10	5.23401E-09	transmembrane protein 181
ENSG00000074071	-1.39	6.25	1.37778E-09	1.96441E-08	mitochondrial ribosomal protein S34
ENSG00000124562	-1.39	6.73	2.53834E-10	4.2223E-09	small nuclear ribonucleoprotein polypeptide C
ENSG00000100401	-1.39	6.60	4.32097E-10	6.82817E-09	Ran GTPase activating protein 1
ENSG00000114767	-1.39	5.04	4.19495E-06	2.79397E-05	ribosomal RNA processing 9, small subunit (SSU) processome component, homolog (yeast)
ENSG00000155229	-1.39	5.84	1.91761E-09	2.67658E-08	MMS19 homolog, cytosolic iron-sulfur assembly component
ENSG00000228300	-1.39	5.53	6.12234E-08	6.27068E-07	chromosome 19 open reading frame 24
ENSG00000122025	-1.39	10.59	1.48548E-06	1.09974E-05	fms-related tyrosine kinase 3
ENSG00000140750	-1.39	6.05	2.47433E-10	4.12616E-09	Rho GTPase activating protein 17
ENSG00000047188	-1.39	4.72	4.8449E-07	4.03458E-06	YTH domain containing 2
ENSG00000176022	-1.39	5.00	1.68396E-06	1.22886E-05	UDP-Gal:betaGal beta 1,3-galactosyltransferase polypeptide 6
ENSG00000164164	-1.40	6.02	1.07763E-09	1.56333E-08	OTU deubiquitinase 4
ENSG00000121716	-1.40	6.23	1.61537E-08	1.85648E-07	paired immunoglobin-like type 2 receptor beta
ENSG00000111666	-1.40	4.65	1.17966E-07	1.13305E-06	choline phosphotransferase 1
ENSG00000113013	-1.40	8.02	5.68548E-11	1.04528E-09	heat shock 70kDa protein 9 (mortalin)
ENSG00000136261	-1.40	6.52	1.94283E-09	2.70327E-08	basic leucine zipper and W2 domains 2
ENSG00000106211	-1.40	7.69	4.75576E-10	7.45526E-09	heat shock 27kDa protein 1
ENSG00000160208	-1.40	6.88	2.35408E-11	4.68486E-10	ribosomal RNA processing 1B
ENSG00000204839	-1.40	4.38	4.13557E-07	3.48765E-06	maestro heat-like repeat family member 6
ENSG00000179051	-1.40	8.48	7.54388E-10	1.14312E-08	regulator of chromosome condensation 2
ENSG00000163811	-1.40	6.38	1.53329E-10	2.66399E-09	WD repeat domain 43
ENSG00000077232	-1.40	6.14	2.01064E-09	2.78013E-08	DnaJ (Hsp40) homolog, subfamily C, member 10
ENSG00000163362	-1.41	6.11	2.50341E-10	4.16942E-09	chromosome 1 open reading frame 106
ENSG00000184110	-1.41	6.70	3.85535E-08	4.0908E-07	eukaryotic translation initiation factor 3, subunit C
ENSG00000170144	-1.41	9.24	3.17704E-08	3.42027E-07	heterogeneous nuclear ribonucleoprotein A3
ENSG00000169714	-1.41	8.17	3.16932E-10	5.16191E-09	CCHC-type zinc finger, nucleic acid binding protein
ENSG00000169683	-1.41	5.58	3.50414E-09	4.63749E-08	leucine rich repeat containing 45
ENSG00000188807	-1.41	4.74	2.2661E-05	0.000125576	transmembrane protein 201
ENSG00000181666	-1.41	4.70	2.1854E-07	1.9734E-06	HKR1, GLI-Kruppel zinc finger family member
ENSG00000105281	-1.41	7.80	3.20848E-09	4.28753E-08	solute carrier family 1 (neutral amino acid transporter), member 5
ENSG00000184220	-1.41	4.43	1.74256E-07	1.61415E-06	cms1 ribosomal small subunit homolog (yeast)
ENSG00000148362	-1.41	5.16	2.59427E-08	2.85069E-07	chromosome 9 open reading frame 142
ENSG00000198786	-1.42	10.71	0.000390991	0.001545107	mitochondrially encoded NADH dehydrogenase 5
ENSG00000148824	-1.42	4.91	4.17719E-05	0.000216509	mitochondrial ribosome-associated GTPase 1
ENSG00000152620	-1.42	4.41	1.38401E-07	1.3085E-06	NAD kinase 2, mitochondrial
ENSG00000175857	-1.42	5.23	1.47481E-09	2.09376E-08	GRB2-binding adaptor protein, transmembrane
ENSG00000131778	-1.42	6.41	6.50402E-11	1.18754E-09	chromodomain helicase DNA binding protein 1-like
ENSG00000158715	-1.43	4.37	3.25353E-05	0.000173026	solute carrier family 45, member 3
ENSG00000140691	-1.44	4.98	1.18806E-06	9.00644E-06	armadillo repeat containing 5
ENSG00000068654	-1.44	6.19	9.55243E-10	1.41201E-08	polymerase (RNA) I polypeptide A, 194kDa
ENSG00000181029	-1.44	4.27	4.42154E-06	2.92143E-05	trafficking protein particle complex 5
ENSG00000074582	-1.44	5.00	7.14202E-09	8.88502E-08	BC1 (ubiquinol-cytochrome c reductase) synthesis-like
ENSG00000120053	-1.44	4.50	4.67098E-06	3.05883E-05	glutamic-oxaloacetic transaminase 1, soluble
ENSG00000101255	-1.45	5.97	0.000120793	0.000553088	tribbles pseudokinase 3
ENSG00000108963	-1.45	4.22	3.48672E-07	2.99758E-06	diphthamide biosynthesis 1
ENSG00000198868	-1.45	12.76	0.000469497	0.001811657	MT-ND4
ENSG00000101444	-1.45	6.52	5.37625E-06	3.48289E-05	adenosylhomocysteinase
ENSG00000140743	-1.45	4.56	7.49479E-08	7.54252E-07	cerebellar degeneration-related protein 2, 62kDa
ENSG00000160957	-1.45	6.27	6.01629E-11	1.10304E-09	RecQ protein-like 4
ENSG00000142686	-1.45	5.26	1.36288E-09	1.94735E-08	chromosome 1 open reading frame 216
ENSG00000214753	-1.45	6.58	1.30905E-10	2.29541E-09	heterogeneous nuclear ribonucleoprotein U-like 2
ENSG00000104835	-1.45	4.49	9.5972E-08	9.36715E-07	seryl-tRNA synthetase 2, mitochondrial
ENSG00000167685	-1.45	4.67	4.39938E-08	4.62004E-07	zinc finger protein 444
ENSG00000198945	-1.45	4.76	7.58709E-08	7.62962E-07	l(3)mbt-like 3 (Drosophila)
ENSG00000130713	-1.46	5.54	4.59502E-10	7.24398E-09	exosome component 2
ENSG00000009830	-1.46	4.44	1.02766E-07	9.94976E-07	protein-O-mannosyltransferase 2
ENSG00000149150	-1.46	6.03	3.54419E-11	6.79849E-10	solute carrier family 43 (amino acid system L transporter), member 1
ENSG00000084774	-1.46	6.83	1.7832E-09	2.50478E-08	carbamoyl-phosphate synthetase 2, aspartate transcarbamylase, and dihydroorotase
ENSG00000270629	-1.46	4.87	1.76662E-08	2.00943E-07	neuroblastoma breakpoint family, member 14
ENSG00000205560	-1.46	5.30	7.75795E-08	7.78375E-07	carnitine palmitoyltransferase 1B (muscle)
ENSG00000148843	-1.46	6.36	3.01589E-09	4.04782E-08	programmed cell death 11

ENSG00000178718	-1.46	4.39	4.78226E-06	3.12554E-05	ribonuclease P/MRP 25kDa subunit
ENSG00000130826	-1.46	7.26	1.1267E-12	2.75938E-11	dyskeratosis congenita 1, dyskerin
ENSG00000132361	-1.46	6.33	6.94684E-08	7.03911E-07	clustered mitochondria (cluA/CLU1) homolog
ENSG00000214160	-1.46	5.36	1.8949E-07	1.73469E-06	ALG3, alpha-1,3- mannosyltransferase
ENSG00000128973	-1.47	5.93	4.54498E-10	7.17362E-09	ceroid-lipofuscinosis, neuronal 6, late infantile, variant
ENSG00000214706	-1.47	6.16	3.17182E-08	3.41743E-07	interferon-related developmental regulator 2
ENSG00000137168	-1.47	5.68	3.95955E-11	7.50843E-10	peptidylprolyl isomerase (cyclophilin)-like 1
ENSG00000125257	-1.47	4.93	1.7613E-06	1.27892E-05	ATP-binding cassette, sub-family C (CFTR/MRP), member 4
ENSG00000082516	-1.47	4.58	4.20134E-06	2.79421E-05	gem (nuclear organelle) associated protein 5
ENSG00000130726	-1.47	8.44	2.06677E-10	3.51722E-09	tripartite motif containing 28
ENSG00000123545	-1.48	4.94	6.29185E-08	6.41459E-07	NADH dehydrogenase (ubiquinone) complex I, assembly factor 4
ENSG00000135521	-1.48	6.54	2.30981E-10	3.87616E-09	LTV1 ribosome biogenesis factor
ENSG00000215041	-1.48	4.63	3.70714E-08	3.9525E-07	neuralized E3 ubiquitin protein ligase 4
ENSG00000065150	-1.48	7.46	7.11138E-12	1.55002E-10	importin 5
ENSG00000143643	-1.48	4.70	1.76955E-08	2.01104E-07	tetratricopeptide repeat domain 13
ENSG00000134905	-1.48	6.40	1.04758E-11	2.21935E-10	cysteinyl-tRNA synthetase 2, mitochondrial (putative)
ENSG00000185504	-1.48	6.34	3.56876E-11	6.8259E-10	Fanconi anemia core complex associated protein 100
ENSG00000110090	-1.49	5.93	1.8574E-06	1.34068E-05	carnitine palmitoyltransferase 1A (liver)
ENSG00000196535	-1.49	6.54	4.3739E-09	5.70886E-08	myosin XVIIIA
ENSG00000144136	-1.49	6.77	6.61661E-10	1.01536E-08	solute carrier family 20 (phosphate transporter), member 1
ENSG00000124541	-1.49	5.45	6.90309E-11	1.25523E-09	ribosomal RNA processing 36
ENSG00000075407	-1.49	6.35	1.63039E-06	1.19568E-05	zinc finger protein 37A
ENSG00000241978	-1.49	5.81	8.95214E-10	1.32772E-08	A kinase (PRKA) anchor protein 2
ENSG00000142185	-1.49	7.37	5.21656E-13	1.33419E-11	transient receptor potential cation channel, subfamily M, member 2
ENSG00000273645	-1.50	5.67	3.3876E-09	4.50571E-08	kelch repeat and BTB (POZ) domain containing 11
ENSG00000100219	-1.50	7.50	6.31863E-08	6.43201E-07	X-box binding protein 1
ENSG00000112159	-1.50	6.29	2.22768E-11	4.46006E-10	midasin AAA ATPase 1
ENSG00000178921	-1.50	4.84	2.01904E-07	1.83566E-06	phosphoribosylformylglycinamidine synthase
ENSG00000108179	-1.50	7.16	2.31802E-11	4.62003E-10	peptidylprolyl isomerase F
ENSG00000197150	-1.50	4.28	2.56103E-07	2.27088E-06	ATP-binding cassette, sub-family B (MDR/TAP), member 8
ENSG00000106105	-1.50	7.32	3.90757E-05	0.000203328	glycyl-tRNA synthetase
ENSG00000185238	-1.50	4.47	9.8263E-08	9.56965E-07	protein arginine methyltransferase 3
ENSG00000085511	-1.51	4.85	1.90884E-09	2.66715E-08	mitogen-activated protein kinase kinase kinase 4
ENSG00000163393	-1.51	6.03	4.30866E-11	8.10101E-10	solute carrier family 22, member 15
ENSG00000149136	-1.51	7.74	4.4128E-11	8.27338E-10	structure specific recognition protein 1
ENSG00000112578	-1.51	5.09	7.67573E-10	1.15913E-08	bystin-like
ENSG00000274679	-1.52	4.63	1.11423E-08	1.34213E-07	spermatogenesis and centriole associated 1-like
ENSG00000132305	-1.52	6.79	7.38404E-13	1.84587E-11	inner membrane protein, mitochondrial
ENSG00000116922	-1.52	4.41	1.95712E-07	1.78426E-06	chromosome 1 open reading frame 109
ENSG00000164934	-1.52	6.07	1.4867E-11	3.04544E-10	DDB1 and CUL4 associated factor 13
ENSG00000071655	-1.52	6.98	2.61832E-12	6.00268E-11	methyl-CpG binding domain protein 3
ENSG00000165209	-1.52	5.28	5.80707E-10	9.00503E-09	spermatid perinuclear RNA binding protein
ENSG00000166123	-1.52	5.58	3.27968E-05	0.000174208	glutamic pyruvate transaminase (alanine aminotransferase) 2
ENSG00000167962	-1.53	6.22	1.24519E-11	2.59476E-10	zinc finger protein 598
ENSG00000179271	-1.53	5.45	3.27941E-10	5.30864E-09	growth arrest and DNA-damage-inducible, gamma interacting protein 1
ENSG00000268006	-1.53	4.46	1.77222E-08	2.01236E-07	PTOV1 antisense RNA 1
ENSG00000185760	-1.54	5.03	7.93798E-10	1.19466E-08	potassium channel, voltage gated KQT-like subfamily Q, member 5
ENSG00000230606	-1.54	4.47	1.50544E-05	8.68834E-05	AC159540.1
ENSG00000247077	-1.54	5.57	2.56103E-11	5.04379E-10	phosphoglycerate mutase family member 5
ENSG00000197728	-1.54	6.33	2.65041E-11	5.19668E-10	ribosomal protein S26
ENSG00000140263	-1.54	5.80	1.50244E-10	2.61382E-09	sorbitol dehydrogenase
ENSG00000171262	-1.55	5.17	2.63291E-10	4.3632E-09	family with sequence similarity 98, member B
ENSG00000277804	-1.55	6.09	1.17138E-11	2.46807E-10	proteinase 3
ENSG00000198727	-1.55	10.97	1.08125E-05	6.5091E-05	mitochondrially encoded cytochrome b
ENSG00000109917	-1.55	5.78	4.87869E-12	1.08294E-10	ZPR1 zinc finger
ENSG00000007392	-1.56	5.26	2.6217E-11	5.148E-10	LUC7-like
ENSG00000130725	-1.56	6.41	2.46719E-11	4.8807E-10	ubiquitin-conjugating enzyme E2M
ENSG00000112651	-1.56	4.84	5.45511E-09	6.93594E-08	mitochondrial ribosomal protein L2
ENSG00000166197	-1.56	7.42	3.44869E-11	6.6441E-10	nucleolar and coiled-body phosphoprotein 1
ENSG00000108561	-1.56	6.22	1.85E-10	3.17221E-09	complement component 1, q subcomponent binding protein
ENSG00000103876	-1.56	5.75	1.7296E-11	3.51588E-10	fumarylacetoacetate hydrolase (fumarylacetoacetase)
ENSG00000178605	-1.56	4.52	3.52859E-08	3.78646E-07	GTP binding protein 6 (putative)
ENSG00000162129	-1.56	4.90	5.05991E-09	6.50077E-08	ClpB homolog, mitochondrial AAA ATPase chaperonin
ENSG00000224078	-1.57	4.96	7.83898E-07	6.26789E-06	small nucleolar RNA host gene 14
ENSG00000204316	-1.57	5.84	1.95367E-11	3.93521E-10	mitochondrial ribosomal protein L38
ENSG00000146830	-1.57	6.96	8.37875E-12	1.80551E-10	GRB10 interacting GYF protein 1
ENSG00000255302	-1.57	4.38	2.94773E-07	2.56915E-06	EP300 interacting inhibitor of differentiation 1
ENSG00000226950	-1.57	4.69	6.62072E-09	8.28308E-08	differentiation antagonizing non-protein coding RNA
ENSG00000145703	-1.58	5.06	1.85876E-08	2.10522E-07	IQ motif containing GTPase activating protein 2
ENSG00000244165	-1.58	4.22	3.64822E-08	3.89907E-07	purinergic receptor P2Y, G-protein coupled, 11
ENSG00000132382	-1.59	5.79	2.76691E-10	4.55681E-09	MYB binding protein (P160) 1a
ENSG00000096063	-1.59	6.86	1.30933E-12	3.16576E-11	SRSF protein kinase 1

ENSG00000161547	-1.59	8.56	1.65239E-12	3.90978E-11	serine/arginine-rich splicing factor 2
ENSG00000131828	-1.59	6.06	2.02914E-12	4.73371E-11	pyruvate dehydrogenase (lipoamide) alpha 1
ENSG00000142102	-1.59	4.74	8.94646E-10	1.32772E-08	ATH1, acid trehalase-like 1 (yeast)
ENSG00000196510	-1.59	5.41	2.89169E-12	6.56142E-11	anaphase promoting complex subunit 7
ENSG00000140006	-1.59	4.33	9.55205E-09	1.16432E-07	WD repeat domain 89
ENSG00000165526	-1.60	5.00	7.79643E-10	1.17469E-08	RNA pseudouridylate synthase domain containing 4
ENSG00000184575	-1.60	7.72	5.09164E-13	1.30476E-11	exportin, tRNA
ENSG00000183010	-1.60	6.52	5.82202E-13	1.47766E-11	pyrroline-5-carboxylate reductase 1
ENSG00000117174	-1.60	4.38	1.66986E-08	1.91084E-07	zinc finger, HIT-type containing 6
ENSG00000143093	-1.61	4.92	1.29975E-10	2.28581E-09	striatin interacting protein 1
ENSG00000058804	-1.61	6.03	2.90801E-13	7.79817E-12	NDC1 transmembrane nucleoporin
ENSG00000155846	-1.62	4.35	9.27819E-08	9.0892E-07	peroxisome proliferator-activated receptor gamma, coactivator 1 beta
ENSG00000138095	-1.62	7.52	4.89134E-14	1.46233E-12	leucine-rich pentatricopeptide repeat containing
ENSG00000133265	-1.62	5.01	5.11849E-11	9.46279E-10	HSPA (heat shock 70kDa) binding protein, cytoplasmic cochaperone 1
ENSG00000223705	-1.63	5.64	1.1443E-12	2.79732E-11	NOP2/Sun domain family, member 5 pseudogene 1
ENSG00000126602	-1.63	6.55	4.9289E-11	9.13774E-10	TNF receptor-associated protein 1
ENSG00000123064	-1.64	6.18	5.29988E-13	1.3529E-11	DEAD (Asp-Glu-Ala-Asp) box polypeptide 54
ENSG00000070061	-1.64	5.56	1.08712E-12	2.67229E-11	inhibitor of kappa light polypeptide gene enhancer in B-cells, kinase complex-associated protein
ENSG00000137309	-1.64	8.08	5.25754E-12	1.15928E-10	high mobility group AT-hook 1
ENSG00000197785	-1.64	6.19	1.38926E-08	1.63195E-07	ATPase family, AAA domain containing 3A
ENSG00000106344	-1.65	5.62	6.69346E-13	1.67956E-11	RNA binding motif protein 28
ENSG00000254870	-1.65	5.83	3.45899E-09	4.58688E-08	ATP6V1G2-DDX39B readthrough (NMD candidate)
ENSG00000118513	-1.65	8.85	2.12662E-10	3.60521E-09	v-myb avian myeloblastosis viral oncogene homolog
ENSG00000171163	-1.65	6.31	7.85887E-14	2.2678E-12	zinc finger protein 692
ENSG00000131368	-1.65	5.87	3.92405E-10	6.26056E-09	mitochondrial ribosomal protein S25
ENSG00000184232	-1.66	5.66	5.90065E-10	9.13567E-09	out at first homolog
ENSG00000017483	-1.66	5.46	7.62537E-13	1.90262E-11	solute carrier family 38, member 5
ENSG00000125901	-1.67	4.38	2.66855E-08	2.92747E-07	mitochondrial ribosomal protein S26
ENSG00000140044	-1.67	5.10	0.001895411	0.006096362	Jun dimerization protein 2
ENSG00000184787	-1.67	7.99	7.78092E-15	2.55653E-13	ubiquitin-conjugating enzyme E2G 2
ENSG00000175602	-1.67	5.26	9.67736E-10	1.4273E-08	coiled-coil domain containing 85B
ENSG00000139514	-1.68	7.61	1.67429E-13	4.6301E-12	solute carrier family 7 (cationic amino acid transporter, y+ system), member 1
ENSG00000128272	-1.69	7.88	0.00012075	0.000553083	activating transcription factor 4
ENSG00000272752	-1.70	5.23	3.89464E-10	6.22111E-09	STAG3L5P-PVRIG2P-PILRB readthrough
ENSG00000107937	-1.70	5.65	5.98758E-13	1.51678E-11	GTP binding protein 4
ENSG00000180730	-1.70	5.02	6.49162E-09	8.1523E-08	shisa family member 2
ENSG00000065183	-1.70	5.71	3.83593E-13	1.01229E-11	WD repeat domain 3
ENSG00000177192	-1.70	5.62	4.4186E-13	1.14811E-11	pseudouridylate synthase 1
ENSG00000090273	-1.70	6.65	1.13214E-13	3.20427E-12	nudC nuclear distribution protein
ENSG00000115053	-1.71	9.73	1.30012E-10	2.28581E-09	nucleolin
ENSG00000167635	-1.71	6.05	2.26761E-13	6.19346E-12	zinc finger protein 146
ENSG00000227686	-1.71	7.19	3.99795E-16	1.49069E-14	valyl-tRNA synthetase
ENSG00000115652	-1.72	5.86	1.26388E-07	1.20523E-06	UDP-glucuronate decarboxylase 1
ENSG00000104635	-1.72	5.69	7.10447E-14	2.0772E-12	solute carrier family 39 (zinc transporter), member 14
ENSG00000135972	-1.72	4.48	5.11522E-09	6.54767E-08	mitochondrial ribosomal protein S9
ENSG00000160256	-1.72	4.75	2.06999E-10	3.51819E-09	family with sequence similarity 207, member A
ENSG00000167114	-1.72	4.75	9.46815E-07	7.40603E-06	solute carrier family 27 (fatty acid transporter), member 4
ENSG00000261236	-1.72	6.33	1.55232E-12	3.69938E-11	block of proliferation 1
ENSG00000182168	-1.73	5.91	5.80655E-09	7.35459E-08	unc-5 netrin receptor C
ENSG00000183889	-1.73	5.01	3.81776E-06	2.57766E-05	Protein LOC102724993
ENSG00000171793	-1.73	6.31	7.30486E-09	9.0706E-08	CTP synthase 1
ENSG00000053372	-1.73	6.31	1.22875E-14	3.95885E-13	MRT4 homolog, ribosome maturation factor
ENSG00000140284	-1.73	5.09	1.86316E-09	2.61157E-08	solute carrier family 27 (fatty acid transporter), member 2
ENSG00000080608	-1.73	5.65	5.90508E-10	9.13567E-09	KIAA0020
ENSG00000091127	-1.73	4.55	8.20727E-09	1.01061E-07	pseudouridylate synthase 7 (putative)
ENSG00000136444	-1.74	5.28	4.02615E-13	1.05828E-11	radical S-adenosyl methionine domain containing 1
ENSG00000165689	-1.75	6.15	2.8164E-12	6.42352E-11	serologically defined colon cancer antigen 3
ENSG00000108578	-1.75	5.31	2.92976E-13	7.84065E-12	bleomycin hydrolase
ENSG00000117528	-1.76	5.89	8.05625E-15	2.63396E-13	ATP-binding cassette, sub-family D (ALD), member 3
ENSG00000188933	-1.78	4.44	0.000122213	0.000559012	ubiquitin specific peptidase 32 pseudogene 1
ENSG00000160214	-1.78	6.16	1.10066E-13	3.12852E-12	ribosomal RNA processing 1
ENSG00000110104	-1.78	5.30	1.60795E-11	3.27864E-10	coiled-coil domain containing 86
ENSG00000127586	-1.78	5.42	2.74622E-14	8.45784E-13	chromosome transmission fidelity factor 18
ENSG00000007520	-1.78	4.23	1.68802E-09	2.38116E-08	TSR3, 20S rRNA accumulation, homolog (S. cerevisiae)
ENSG00000125459	-1.79	4.95	5.46042E-11	1.00529E-09	misato 1, mitochondrial distribution and morphology regulator
ENSG00000189007	-1.79	5.50	9.62149E-15	3.12263E-13	adenosine deaminase, tRNA-specific 2
ENSG00000063241	-1.79	4.60	1.73957E-10	2.99884E-09	isochorismatase domain containing 2
ENSG00000179041	-1.80	5.09	9.75803E-13	2.41207E-11	ribosome biogenesis regulator homolog
ENSG00000169230	-1.80	6.60	2.43054E-15	8.46851E-14	PRELI domain containing 1
ENSG00000180900	-1.82	6.70	8.77998E-17	3.61942E-15	scribbled planar cell polarity protein
ENSG00000213066	-1.82	5.04	3.03962E-13	8.10199E-12	FGFR1 oncogene partner
ENSG00000152642	-1.82	4.69	2.46582E-08	2.7208E-07	glycerol-3-phosphate dehydrogenase 1-like

ENSG00000134987	-1.82	6.15	1.27074E-11	2.63972E-10	WD repeat domain 36
ENSG00000167965	-1.82	5.60	8.1276E-09	1.00173E-07	MTOR associated protein, LST8 homolog
ENSG00000115758	-1.82	6.64	1.34883E-12	3.25533E-11	ornithine decarboxylase 1
ENSG00000071994	-1.82	6.23	4.41093E-16	1.63094E-14	programmed cell death 2
ENSG00000100427	-1.82	5.48	5.2312E-10	8.15969E-09	megalencephalic leukoencephalopathy with subcortical cysts 1
ENSG00000182676	-1.83	9.40	1.50295E-12	3.60112E-11	protein phosphatase 1, regulatory subunit 27
ENSG00000253729	-1.83	8.36	2.71232E-16	1.04357E-14	protein kinase, DNA-activated, catalytic polypeptide
ENSG00000106133	-1.84	4.25	8.70001E-11	1.56907E-09	NOP2/Sun domain family, member 5 pseudogene 2
ENSG00000232169	-1.84	7.07	1.68841E-17	7.57162E-16	ATP-binding cassette, sub-family F (GCN20), member 1
ENSG00000068831	-1.84	7.15	1.46968E-16	5.91167E-15	RAS guanyl releasing protein 2 (calcium and DAG-regulated)
ENSG00000168924	-1.84	6.09	5.52022E-16	2.02979E-14	leucine zipper-EF-hand containing transmembrane protein 1
ENSG00000162676	-1.85	7.16	2.76593E-18	1.37509E-16	growth factor independent 1 transcription repressor
ENSG00000196449	-1.85	4.76	2.82703E-12	6.43671E-11	yrdC N(6)-threonylcarbamoyltransferase domain containing
ENSG00000170571	-1.85	6.83	1.70658E-17	7.62731E-16	embigin
ENSG00000004478	-1.85	6.35	5.98416E-14	1.76519E-12	FK506 binding protein 4, 59kDa
ENSG00000141569	-1.86	6.09	1.97254E-13	5.4098E-12	tripartite motif containing 65
ENSG00000007541	-1.86	4.66	1.0468E-10	1.86513E-09	phosphatidylinositol glycan anchor biosynthesis, class Q
ENSG00000188206	-1.86	7.50	2.84997E-13	7.658E-12	HNRNPU antisense RNA 1
ENSG00000123213	-1.87	4.66	1.24863E-11	2.59786E-10	neurolysin (metallopeptidase M3 family)
ENSG00000060982	-1.87	6.01	3.34247E-10	5.40414E-09	branched chain amino-acid transaminase 1, cytosolic
ENSG00000120254	-1.87	5.18	1.23539E-12	3.00892E-11	methylenetetrahydrofolate dehydrogenase (NADP+ dependent) 1-like
ENSG00000196305	-1.89	7.47	3.4994E-17	1.50327E-15	isoleucyl-tRNA synthetase
ENSG00000103253	-1.89	4.38	4.75037E-09	6.15987E-08	hydroxyacylglutathione hydrolase-like
ENSG00000171791	-1.89	5.21	2.12398E-15	7.45866E-14	B-cell CLL/lymphoma 2
ENSG00000105677	-1.90	5.52	1.71774E-16	6.82674E-15	transmembrane protein 147
ENSG00000178105	-1.90	4.81	1.59279E-12	3.78223E-11	DEAD (Asp-Glu-Ala-Asp) box polypeptide 10
ENSG00000132768	-1.91	4.92	5.49441E-10	8.55016E-09	DPH2 homolog
ENSG00000133706	-1.91	7.15	6.50062E-19	3.42418E-17	leucyl-tRNA synthetase
ENSG00000116649	-1.92	6.97	9.08931E-19	4.73143E-17	spermidine synthase
ENSG00000171492	-1.92	6.29	1.67344E-12	3.95255E-11	leucine rich repeat containing 8 family, member D
ENSG00000120438	-1.92	8.18	7.86178E-17	3.27139E-15	t-complex 1
ENSG00000183426	-1.94	6.27	2.25241E-14	7.01841E-13	nuclear pore complex interacting protein family, member A1
ENSG00000172366	-1.95	4.24	6.18571E-08	6.31608E-07	family with sequence similarity 195, member A
ENSG00000183048	-1.96	5.30	3.4332E-16	1.2924E-14	solute carrier family 25 (mitochondrial carrier; dicarboxylate transporter), member 10
ENSG00000144485	-1.97	5.68	5.40617E-14	1.6054E-12	hes family bHLH transcription factor 6
ENSG00000282283	-1.97	7.47	3.24063E-11	6.26144E-10	LPPR3
ENSG00000083097	-1.97	4.93	6.10674E-13	1.54402E-11	dopey family member 1
ENSG00000146457	-1.97	5.02	2.89901E-14	8.90776E-13	Wilms tumor 1 associated protein
ENSG00000132780	-1.98	7.71	3.85763E-20	2.31535E-18	nuclear autoantigenic sperm protein (histone-binding)
ENSG00000198744	-1.98	12.21	1.31581E-06	9.8678E-06	MT-CO3
ENSG00000114631	-1.98	5.23	6.46925E-09	8.13961E-08	podocalyxin-like 2
ENSG00000196497	-1.99	5.92	5.97727E-10	9.22585E-09	importin 4
ENSG00000172179	-2.01	5.85	4.18167E-10	6.61592E-09	prolactin
ENSG00000102241	-2.03	5.94	2.1421E-17	9.5098E-16	HIV-1 Tat specific factor 1
ENSG00000175416	-2.03	4.95	3.08604E-15	1.06124E-13	clathrin, light chain B
ENSG00000070081	-2.03	8.80	1.2584E-17	5.77993E-16	nucleobindin 2
ENSG00000198520	-2.05	6.95	1.46919E-17	6.70173E-16	chromosome 1 open reading frame 228
ENSG00000113575	-2.08	6.51	1.93878E-20	1.20259E-18	protein phosphatase 2, catalytic subunit, alpha isozyme
ENSG00000135069	-2.08	6.80	2.39815E-08	2.65054E-07	phosphoserine aminotransferase 1
ENSG00000124802	-2.09	5.78	3.07361E-19	1.67209E-17	eukaryotic translation elongation factor 1 epsilon 1
ENSG00000099624	-2.09	6.12	6.4182E-18	2.99983E-16	ATP synthase, H+ transporting, mitochondrial F1 complex, delta subunit
ENSG00000087269	-2.09	6.46	6.71609E-20	3.96219E-18	NOP14 nucleolar protein
ENSG00000185163	-2.10	4.99	2.82451E-16	1.07737E-14	DEAD (Asp-Glu-Ala-Asp) box polypeptide 51
ENSG00000221823	-2.10	5.90	6.25141E-16	2.2797E-14	protein phosphatase 3, regulatory subunit B, alpha
ENSG00000155380	-2.11	6.22	7.09988E-21	4.57494E-19	solute carrier family 16 (monocarboxylate transporter), member 1
ENSG00000235173	-2.11	4.92	1.59785E-14	5.06202E-13	HGH1 homolog
ENSG00000198763	-2.11	11.15	5.00774E-09	6.44116E-08	mitochondrially encoded NADH dehydrogenase 2
ENSG00000103266	-2.11	6.11	2.57342E-18	1.28904E-16	STIP1 homology and U-box containing protein 1, E3 ubiquitin protein ligase
ENSG00000282367	-2.12	4.93	3.27219E-14	9.98508E-13	phosphatidylserine synthase 2
ENSG00000116455	-2.12	5.36	5.17386E-17	2.17335E-15	WD repeat domain 77
ENSG00000179862	-2.15	4.52	1.01083E-12	2.48939E-11	Cbp/p300-interacting transactivator, with Glu/Asp-rich carboxy-terminal domain, 4
ENSG00000139675	-2.17	5.35	5.00746E-13	1.28567E-11	heterogeneous nuclear ribonucleoprotein A1-like 2
ENSG00000243716	-2.18	6.51	1.07676E-15	3.85254E-14	nuclear pore complex interacting protein family, member B5
ENSG00000183751	-2.19	5.08	2.29098E-13	6.24444E-12	transducin (beta)-like 3
ENSG00000114446	-2.20	5.56	2.53116E-20	1.54122E-18	intraflagellar transport 57
ENSG00000183605	-2.21	5.00	3.50111E-13	9.2762E-12	sideroflexin 4
ENSG00000103342	-2.26	6.84	2.61855E-16	1.01043E-14	G1 to S phase transition 1
ENSG00000198899	-2.26	11.63	1.49454E-08	1.73718E-07	mitochondrially encoded ATP synthase 6
ENSG00000120738	-2.28	6.61	2.60779E-17	1.14622E-15	early growth response 1
ENSG00000007264	-2.29	4.54	1.2058E-11	2.52458E-10	megakaryocyte-associated tyrosine kinase
ENSG00000226916	-2.33	5.80	4.60489E-19	2.45483E-17	WD repeat domain 46
ENSG00000174669	-2.33	5.15	1.50345E-21	1.0182E-19	solute carrier family 29 (equilibrative nucleoside transporter), member 2

ENSG00000183688	-2.36	7.05	6.09326E-18	2.85802E-16	family with sequence similarity 101, member B
ENSG00000116852	-2.36	4.67	2.12237E-10	3.60261E-09	kinesin family member 21B
ENSG00000074935	-2.39	4.40	8.0411E-06	4.99474E-05	tubulin, epsilon 1
ENSG00000160072	-2.41	5.74	3.36921E-18	1.64423E-16	ATPase family, AAA domain containing 3B
ENSG00000224877	-2.45	4.67	7.9127E-14	2.27838E-12	chromosome 17 open reading frame 89
ENSG00000110660	-2.45	5.14	1.18364E-21	8.18314E-20	solute carrier family 35, member F2
ENSG00000011052	-2.45	5.30	3.35185E-21	2.23581E-19	NME/NM23 nucleoside diphosphate kinase 2
ENSG00000070669	-2.45	5.53	1.58819E-08	1.83159E-07	asparagine synthetase (glutamine-hydrolyzing)
ENSG00000277957	-2.46	6.62	1.19734E-08	1.42696E-07	SENP3-EIF4A1 readthrough (NMD candidate)
ENSG00000106070	-2.46	5.15	5.75921E-20	3.41285E-18	growth factor receptor-bound protein 10
ENSG00000262814	-2.54	5.57	1.7156E-23	1.47875E-21	mitochondrial ribosomal protein L12
ENSG00000175445	-2.60	5.23	3.88344E-05	0.000202264	lipoprotein lipase
ENSG00000160818	-2.67	5.60	5.14005E-28	5.98501E-26	G patch domain containing 4
ENSG00000164284	-2.72	4.53	2.55435E-07	2.26647E-06	GrpE-like 2, mitochondrial (E. coli)
ENSG00000052749	-2.74	5.30	8.42927E-22	5.88896E-20	ribosomal RNA processing 12 homolog
ENSG00000106588	-2.77	5.08	3.70105E-05	0.000193264	proteasome (prosome, macropain) subunit, alpha type, 2
ENSG00000169246	-3.18	6.11	1.17126E-22	9.0391E-21	nuclear pore complex interacting protein family, member B3
ENSG00000169136	-3.29	6.03	2.19389E-12	5.09121E-11	activating transcription factor 5
ENSG00000176076	-3.41	4.80	6.66331E-28	7.6912E-26	potassium channel, voltage gated subfamily E regulatory beta subunit 5
ENSG00000239672	-4.00	6.13	4.18997E-45	1.11235E-42	NME/NM23 nucleoside diphosphate kinase 1
ENSG00000128965	-4.85	4.55	0.000222143	0.000944803	ChaC glutathione-specific gamma-glutamylcyclotransferase 1

Supplementary Table S5A: Differencial expression analysis of T. gondii genes from MM6 cells infected with T. gondii EGS strain for 18 hours copmared to T. gondii gene expression from MM6 cells infected with GT1 strain for 18 hours.

gene ID	logFC (EGS/GT1)	logCPM	PValue	FDR	Product Name
TGME49_232955	8.01	8.96	1.23E-54	9.09E-51	hypothetical protein
TGME49_323400	7.44	9.09	3.98E-42	1.47E-38	cytochrome c oxidase subunit iii subfamily protein
TGME49_322200	7.72	6.25	3.10E-41	7.66E-38	apocytochrome b, putative
TGME49_206550	4.89	5.45	6.10E-28	1.13E-24	hypothetical protein
TGME49_330000	8.23	9.81	2.24E-26	3.32E-23	cytochrome b
TGME49_237130	8.00	9.77	7.17E-24	8.86E-21	cytochrome b, putative
TGME49_255060	6.97	9.39	1.12E-23	1.19E-20	cytochrome b(N-terminal)/b6/petB subfamily protein
TGME49_252065	3.93	5.35	3.54E-16	3.27E-13	KRUF family protein
TGME49_305460	2.60	7.39	2.57E-15	2.12E-12	methionine aminopeptidase 2, putative
TGME49_301222	2.58	6.51	4.94E-15	3.66E-12	DNA repair protein Rad4 domain-containing protein
TGME49_252220	2.24	7.62	4.27E-14	2.87E-11	tetratricopeptide repeat domain containing protein
TGME49_250670	3.64	5.81	5.48E-14	3.38E-11	hypothetical protein
TGME49_302055	8.07	5.01	4.21E-13	2.23E-10	ribosomal protein RPS12
TGME49_279340	2.25	6.65	1.99E-12	9.83E-10	hypothetical protein
TGME49_252070	3.61	4.72	4.39E-12	2.03E-09	KRUF family protein
TGME49_225555	1.93	8.72	7.76E-12	3.19E-09	hypothetical protein
TGME49_275860	2.23	9.13	1.90E-11	7.40E-09	hypothetical protein
TGME49_217530	3.58	4.70	2.75E-11	1.02E-08	hypothetical protein
TGME49_254030	1.93	8.07	2.89E-11	1.02E-08	zinc finger CDGSH-type domain-containing protein
TGME49_253790	2.72	7.19	9.06E-11	3.05E-08	zinc finger (CCCH type) motif-containing protein
TGME49_249230	3.23	6.21	1.99E-10	6.41E-08	hypothetical protein
TGME49_260250	-2.90	6.13	2.34E-10	6.92E-08	cyclin domain protein, cyclin H family protein
TGME49_294400	-3.34	6.97	2.26E-10	6.92E-08	hypothetical protein
TGME49_226310	-2.88	7.53	3.68E-10	1.05E-07	zinc finger (CCCH type) motif-containing protein
TGME49_233925	10.75	11.32	4.53E-10	1.24E-07	hypothetical protein
TGME49_223660	1.91	7.24	1.08E-09	2.87E-07	50S ribosomal protein L4, putative
TGME49_235630	1.82	6.97	1.78E-09	4.47E-07	hypothetical protein
TGME49_286928	-3.71	6.04	1.81E-09	4.47E-07	hypothetical protein
TGME49_294980	4.14	4.97	1.87E-09	4.47E-07	hypothetical protein
TGME49_273870	-2.06	7.40	1.99E-09	4.60E-07	SWI2/SNF2 ISWI-like (AT hook)
TGME49_260430	3.50	4.69	2.30E-09	5.17E-07	hypothetical protein
TGME49_251180	2.60	5.76	4.02E-09	8.76E-07	KRUF family protein
TGME49_301250	7.47	14.41	5.72E-09	1.21E-06	hypothetical protein
TGME49_299030	1.91	7.31	5.88E-09	1.21E-06	RNA recognition motif 2 protein
TGME49_285710	2.51	5.78	7.61E-09	1.48E-06	hypothetical protein
TGME49_290150	-2.45	6.93	7.51E-09	1.48E-06	hypothetical protein
TGME49_260480	2.83	5.50	8.95E-09	1.70E-06	leucine rich repeat-containing protein
TGME49_277270	2.73	9.46	9.50E-09	1.76E-06	NTPase II
TGME49_322000	-2.57	6.91	1.08E-08	1.95E-06	myosin-light-chain kinase
TGME49_258390	2.65	5.51	1.41E-08	2.49E-06	DnaJ protein, putative
TGME49_266740	3.44	5.27	1.53E-08	2.64E-06	RNA recognition motif-containing protein
TGME49_300010	-4.97	5.19	1.84E-08	3.09E-06	hypothetical protein
TGME49_242240	-3.22	5.94	1.94E-08	3.20E-06	rhoptry kinase family protein ROP19A
TGME49_207430	-2.44	6.38	2.87E-08	4.52E-06	ATP-dependent RNA helicase DDX1, putative
TGME49_253900	2.85	5.99	3.14E-08	4.84E-06	parasite porphobilinogen synthase PBGS
TGME49_212940	-8.20	6.33	4.13E-08	6.24E-06	hypothetical protein
TGME49_315885	5.95	9.43	4.84E-08	7.17E-06	glycosyltransferase, putative
TGME49_251170	2.42	5.46	5.30E-08	7.70E-06	KRUF family protein
TGME49_234460	2.14	6.80	5.44E-08	7.75E-06	hypothetical protein
TGME49_259950	2.03	6.67	6.86E-08	9.59E-06	carbonate dehydratase, eukaryotic-type domain-containing
TGME49_213067	1.80	7.89	7.39E-08	1.01E-05	hypothetical protein
TGME49_221320	2.55	9.10	8.61E-08	1.16E-05	acetyl-CoA carboxylase ACC1

TGME49_257350	1.55	8.19	9.06E-08	1.20E-05	eukaryotic translation initiation factor, putative
TGME49_313100	-3.03	5.89	9.88E-08	1.28E-05	signal recognition particle SRP54 protein
TGME49_241155	3.09	5.87	1.02E-07	1.30E-05	hypothetical protein
TGME49_215430	4.78	9.38	1.07E-07	1.35E-05	hypothetical protein
TGME49_200310	1.68	7.75	1.14E-07	1.38E-05	hypothetical protein
TGME49_236270	-3.59	5.33	1.13E-07	1.38E-05	hypothetical protein
TGME49_275640	1.79	6.96	1.54E-07	1.70E-05	hypothetical protein
TGME49_299080	1.66	6.87	1.61E-07	1.75E-05	VTC domain-containing protein
TGME49_232600	2.23	6.53	1.89E-07	2.03E-05	phospholipase, patatin family protein
TGME49_312140	-4.04	8.68	2.22E-07	2.26E-05	hypothetical protein
TGME49_215930	-8.19	4.44	2.21E-07	2.26E-05	mediator complex subunit MED21
TGME49_299000	2.45	6.29	2.83E-07	2.80E-05	hypothetical protein
TGME49_254710	1.72	7.19	2.87E-07	2.80E-05	serine esterase (DUF676) protein
TGME49_247410	1.50	7.58	2.85E-07	2.80E-05	hypothetical protein
TGME49_233130	3.15	5.01	3.29E-07	3.16E-05	nucleoside transporter protein
TGME49_267020	-2.38	7.23	3.35E-07	3.18E-05	hypothetical protein
TGME49_253750	1.73	7.45	3.75E-07	3.47E-05	PLU-1 family protein
TGME49_227810	1.57	8.47	4.61E-07	4.21E-05	rhoptry kinase family protein ROP11 (incomplete catalytic triad)
TGME49_207960	-2.40	6.09	4.69E-07	4.24E-05	hypothetical protein
TGME49_272410	3.57	8.19	4.75E-07	4.24E-05	phosphogluconate dehydrogenase (decarboxylating), NAD
TGME49_215940	-2.46	6.61	5.07E-07	4.47E-05	Acetyl-coenzyme A transporter, putative
TGME49_233870	1.95	6.13	5.52E-07	4.60E-05	hypothetical protein
TGME49_254690	1.57	7.81	5.52E-07	4.60E-05	phospholipase/carboxylesterase
TGME49_217555	1.35	7.97	5.43E-07	4.60E-05	hypothetical protein
TGME49_240700	-2.10	6.20	5.66E-07	4.66E-05	ubiquitin family protein
TGME49_260310	2.97	4.60	7.39E-07	6.02E-05	ATP-binding cassette transporter ABC.B1
TGME49_258720	1.64	6.22	7.89E-07	6.29E-05	Ubiquitin family protein, putative
TGME49_301280	-3.03	5.06	8.45E-07	6.66E-05	hypothetical protein
TGME49_254660	1.60	6.61	8.70E-07	6.72E-05	ankyrin repeat-containing protein
TGME49_247930	-3.26	5.11	8.62E-07	6.72E-05	SNARE domain-containing protein
TGME49_268225	-8.90	6.07	1.02E-06	7.79E-05	hypothetical protein
TGME49_206695	3.11	4.49	1.08E-06	8.09E-05	hypothetical protein
TGME49_231370	2.43	5.17	1.08E-06	8.09E-05	phospholipase, patatin family protein
TGME49_214380	-2.49	5.63	1.12E-06	8.28E-05	hypothetical protein
TGME49_259010	-1.97	6.56	1.17E-06	8.57E-05	vacuolar ATP synthase subunit d, putative
TGME49_306020	2.01	5.49	1.26E-06	9.15E-05	hypothetical protein
TGME49_254080	1.47	7.77	1.28E-06	9.20E-05	metal cation transporter, ZIP family protein
TGME49_240250	-1.53	7.48	1.29E-06	9.20E-05	macro domain-containing protein
TGME49_283790	3.32	5.43	1.41E-06	9.87E-05	protein kinase, putative
TGME49_293820	-1.59	7.28	1.44E-06	9.98E-05	calpain family cysteine protease domain-containing protein
TGME49_258790	-1.72	7.15	1.50E-06	0.00010279	zinc finger, C3HC4 type (RING finger) domain-containing protein
TGME49_220240	1.59	8.76	1.69E-06	0.00011512	hypothetical protein
TGME49_295935	1.50	9.03	1.76E-06	0.00011836	KRUF family protein
TGME49_246490	-1.55	7.85	1.78E-06	0.00011881	hypothetical protein
TGME49_225930	1.29	8.16	1.84E-06	0.00012105	triose-phosphate isomerase TPI-I
TGME49_297495	-7.98	4.96	1.85E-06	0.00012105	hypothetical protein
TGME49_240510	2.77	5.61	2.04E-06	0.00013164	hypothetical protein
TGME49_299210	1.86	8.19	2.04E-06	0.00013164	CTP synthase
TGME49_306895	1.69	7.00	2.06E-06	0.00013186	hypothetical protein
TGME49_321540	-1.74	7.23	2.12E-06	0.00013442	hypothetical protein
TGME49_254770	1.45	6.49	2.16E-06	0.00013461	Ser/Thr phosphatase family protein
TGME49_290580	-2.49	6.26	2.15E-06	0.00013461	ATP-binding cassette G family transporter ABCG89
TGME49_234640	-1.84	6.39	2.31E-06	0.0001414	hypothetical protein
TGME49_208440	1.33	8.08	2.33E-06	0.00014165	hypothetical protein
TGME49_252310	1.63	6.62	2.43E-06	0.00014609	hypothetical protein
TGME49_252360	1.57	9.12	2.47E-06	0.00014754	rhoptry kinase family protein ROP24 (incomplete catalytic triad)
TGME49_254620	1.89	9.88	2.50E-06	0.00014819	ribosomal protein RPL39

TGME49_243410	-2.01	6.18	2.55E-06	0.0001497	tetratricopeptide repeat-containing protein
TGME49_279350	2.28	5.67	2.62E-06	0.00015306	hypothetical protein
TGME49_293280	2.37	5.03	2.66E-06	0.0001542	cyclin protein
TGME49_224220	-1.94	6.62	2.70E-06	0.00015528	serine/threonine-protein phosphatase PP2A catalytic subunit
TGME49_253470	1.39	7.33	2.80E-06	0.00015979	alveolin domain containing intermediate filament IMC13
TGME49_239270	-1.71	7.52	2.98E-06	0.00016849	hypothetical protein
TGME49_203540	1.80	7.63	3.19E-06	0.00017919	RNA binding protein, putative
TGME49_293430	1.38	8.30	3.24E-06	0.00018036	hypothetical protein
TGME49_244370	6.17	11.45	3.35E-06	0.00018549	TDC1, putative
TGME49_219520	-1.51	7.45	3.39E-06	0.00018629	histone arginine methyltransferase PRMT1
TGME49_261750	-1.75	6.89	3.55E-06	0.00019345	rhoptry neck protein RON10
TGME49_242340	1.66	9.48	3.74E-06	0.00020246	ribosomal protein RPS29
TGME49_285190	-1.73	6.63	3.79E-06	0.00020349	zinc finger, C3HC4 type (RING finger) domain-containing protein
TGME49_319540	-2.36	5.83	3.84E-06	0.0002045	hypothetical protein
TGME49_278050	-1.45	8.02	3.95E-06	0.0002089	proteasome subunit alpha type 1, putative
TGME49_247340	2.08	5.11	4.35E-06	0.00022713	hypothetical protein
TGME49_320005	-2.72	5.44	4.33E-06	0.00022713	hypothetical protein
TGME49_305160	3.60	4.53	4.40E-06	0.00022771	histone H2Ba
TGME49_289050	2.13	7.40	4.44E-06	0.00022867	FIKK kinase, putative
TGME49_252290	1.69	6.98	4.53E-06	0.00023125	importin alpha, putative
TGME49_262010	1.74	6.19	4.56E-06	0.00023127	calmodulin CAM2
TGME49_249770	2.68	4.97	5.01E-06	0.00024904	Nmda1 protein
TGME49_228750	2.50	5.68	5.00E-06	0.00024904	TGME49_228750 CAM kinase, RAD family
TGME49_277700	2.31	4.92	5.13E-06	0.00025165	ribosomal protein S14 precursor, putative
TGME49_233500	1.50	7.35	5.10E-06	0.00025165	triose-phosphate isomerase TPI-II
TGME49_311440	1.65	6.20	5.19E-06	0.00025291	SAG-related sequence SRS50
TGME49_308840	1.66	9.63	5.35E-06	0.00025737	SAG-related sequence SRS51
TGME49_277710	-1.81	6.74	5.32E-06	0.00025737	hypothetical protein
TGME49_215390	2.51	4.45	5.47E-06	0.00026036	TIM10 family protein, putative
TGME49_215895	1.71	6.10	5.48E-06	0.00026036	AP2 domain-containing protein
TGME49_260520	1.96	6.90	5.73E-06	0.0002702	hypothetical protein
TGME49_278510	-1.51	8.05	5.99E-06	0.00028086	protein phosphatase 2C domain-containing protein
TGME49_252390	1.30	8.34	6.47E-06	0.00030075	hypothetical protein
TGME49_312310	-2.52	5.48	6.49E-06	0.00030075	ATPase, AAA family protein
TGME49_254050	1.39	6.49	6.79E-06	0.00031229	optic atrophy 3 protein (opa3) protein
TGME49_213280	1.45	9.22	7.06E-06	0.00032101	SAG-related sequence SRS25
TGME49_260210	-2.94	5.25	7.04E-06	0.00032101	DnaJ domain-containing protein
TGME49_246978	-2.87	5.37	8.07E-06	0.00036442	hypothetical protein
TGME49_279450	-7.76	4.57	8.25E-06	0.00037046	adenylosuccinate synthetase, putative
TGME49_233030	-1.36	8.47	8.33E-06	0.00037168	gliding-associated protein GAP70
TGME49_270530	-1.63	6.58	8.57E-06	0.00038009	ubiquitin fusion degradation protein UFD1CY
TGME49_308090	2.51	9.77	8.67E-06	0.00038169	rhoptry protein ROP5
TGME49_205680	1.65	7.62	8.71E-06	0.00038169	hypothetical protein
TGME49_268570	-1.42	7.32	9.02E-06	0.00039091	zinc finger (CCCH type) motif-containing protein
TGME49_263270	-1.53	7.37	9.01E-06	0.00039091	glycerophosphodiester phosphodiesterase family protein
TGME49_262910	2.83	4.81	9.27E-06	0.00039681	NADH-cytochrome b5 reductase 1, putative
TGME49_219832	-2.70	5.42	9.26E-06	0.00039681	cyclin-dependent kinase regulatory subunit protein
TGME49_217010	-1.86	6.08	9.70E-06	0.00041299	hypothetical protein
TGME49_312600	1.17	8.45	1.01E-05	0.00042687	heat shock protein HSP21
TGME49_249860	-1.63	7.01	1.02E-05	0.00043038	hypothetical protein
TGME49_223040	1.52	7.92	1.03E-05	0.00043061	hypothetical protein
TGME49_275320	-1.56	7.96	1.03E-05	0.00043061	penicillin amidase
TGME49_255650	-1.54	7.47	1.07E-05	0.00044089	DHHC zinc finger domain-containing protein
TGME49_242110	-2.88	5.29	1.08E-05	0.00044285	rhoptry kinase family protein ROP38
TGME49_254520	1.33	7.27	1.18E-05	0.0004827	mediator complex subunit MED11

TGME49_266100	1.48	6.81	1.20E-05	0.00048446	rhoptry kinase family protein ROP41
TGME49_247770	1.21	7.98	1.19E-05	0.00048446	hypothetical protein
TGME49_215540	-8.74	4.96	1.21E-05	0.00048615	hypothetical protein
TGME49_254910	2.11	5.42	1.26E-05	0.00050411	hypothetical protein
TGME49_287470	2.41	4.97	1.28E-05	0.00050863	hypothetical protein
TGME49_252250	2.15	5.19	1.34E-05	0.00052787	ATPase, AAA family protein
TGME49_240520	2.21	5.27	1.35E-05	0.00052938	hypothetical protein
TGME49_220400	2.00	10.26	1.43E-05	0.00055589	actin depolymerizing factor ADF
TGME49_262500	-1.75	7.65	1.47E-05	0.0005644	hypothetical protein
TGME49_207630	-3.63	4.99	1.47E-05	0.0005644	peptidyl-tRNA hydrolase domain-containing protein
TGME49_210430	-1.34	7.63	1.53E-05	0.00058311	DnaJ domain-containing protein
TGME49_290600	6.12	12.08	1.54E-05	0.00058622	succinyl-CoA-synthetase alpha SCSA
TGME49_211220	-1.89	5.90	1.55E-05	0.00058636	hypothetical protein
TGME49_286140	-1.99	5.87	1.56E-05	0.00058636	hypothetical protein
TGME49_268730	2.02	5.02	1.57E-05	0.00058932	glutaredoxin-related protein
TGME49_311210	-1.66	6.42	1.65E-05	0.00061299	hypothetical protein
TGME49_284190	1.69	6.11	1.69E-05	0.00062421	pyruvate carboxylase
TGME49_200595	-9.05	4.67	1.71E-05	0.00062899	hypothetical protein
TGME49_271935	1.72	6.37	1.81E-05	0.00066234	hypothetical protein
TGME49_261000	-1.86	6.99	1.88E-05	0.00068266	MutS domain V domain-containing protein
TGME49_280518	-1.51	7.81	1.92E-05	0.00069334	hypothetical protein
TGME49_320110	2.87	8.51	1.94E-05	0.0006969	proliferating cell nuclear antigen PCNA2
TGME49_209755	-1.64	5.73	1.95E-05	0.00069769	hypothetical protein
TGME49_286720	1.48	6.76	2.07E-05	0.00073232	heat shock protein HSP28
TGME49_321360	1.18	7.60	2.09E-05	0.00073655	clustered-asparagine-rich protein
TGME49_202540	-1.37	7.57	2.17E-05	0.00075938	3'5'-cyclic nucleotide phosphodiesterase domain-containing
TGME49_230490	-1.72	6.28	2.22E-05	0.00077392	phosphatidylinositol-4-phosphate 5-kinase
TGME49_297420	-2.07	5.89	2.24E-05	0.0007752	beta-tubulin cofactor D, putative
TGME49_253290	1.20	7.51	2.28E-05	0.00078324	valyl-tRNA synthetase
TGME49_217951	-1.96	6.01	2.28E-05	0.00078324	hypothetical protein
TGME49_259530	-1.67	6.38	2.34E-05	0.00079776	GalNac
TGME49_268620	-3.08	5.03	2.40E-05	0.00081637	blood stage antigen 41-3 precursor, putative
TGME49_223150	-2.63	5.54	2.57E-05	0.00086931	START domain-containing protein
TGME49_223840	1.73	6.63	2.61E-05	0.00088029	ATP-citrate lyase, putative
TGME49_201790	-1.60	6.54	2.64E-05	0.00088159	FHA domain-containing protein
TGME49_237195	-2.18	5.58	2.74E-05	0.00090962	hypothetical protein
TGME49_252270	1.93	5.51	2.80E-05	0.00092612	L1P family of ribosomal protein
TGME49_253990	1.44	6.61	2.82E-05	0.00092612	hypothetical protein
TGME49_293550	-1.38	7.18	2.82E-05	0.00092612	hypothetical protein
TGME49_254610	1.75	5.28	2.97E-05	0.00096083	Tim10/DDP family zinc finger superfamily protein
TGME49_254900	1.33	6.54	2.96E-05	0.00096083	proteasome subunit beta type 2, putative
TGME49_306730	-3.20	5.07	2.97E-05	0.00096083	hypothetical protein
TGME49_248630	-1.60	6.81	3.06E-05	0.00098587	actin-related protein ARP1
TGME49_252500	1.20	7.79	3.08E-05	0.00098914	polo kinase
TGME49_238073	-2.22	5.29	3.12E-05	0.00099647	hypothetical protein
TGME49_227330	1.71	7.01	3.15E-05	0.00100085	hypothetical protein
TGME49_222900	1.37	7.03	3.17E-05	0.00100085	phosphoserine phosphatase
TGME49_205510	-1.20	7.77	3.17E-05	0.00100085	nucleolar protein 5, putative
TGME49_255635	-4.70	5.32	3.20E-05	0.00100344	hypothetical protein
TGME49_204110	-2.91	5.27	3.22E-05	0.00100569	eIF2 kinase IF2K-C
TGME49_259115	-2.00	7.48	3.46E-05	0.00107702	ABC1 family protein
TGME49_321550	1.54	5.79	3.62E-05	0.00112303	hypothetical protein
TGME49_213800	-1.29	8.01	3.67E-05	0.00112788	protein phosphatase 2b regulatory subunit, putative
TGME49_258030	-2.43	5.92	3.67E-05	0.00112788	DNA polymerase
TGME49_252190	2.13	5.74	3.72E-05	0.00113785	KRUF family protein
TGME49_292200	-1.23	7.70	3.80E-05	0.00115745	RNA recognition motif-containing protein

TGME49_247530	1.25	8.79	3.90E-05	0.00117188	hypothetical protein
TGME49_298990	1.50	5.96	4.15E-05	0.00123856	ferredoxin NADP+ oxidoreductase FNR
TGME49_229440	-1.85	6.25	4.19E-05	0.0012467	zinc finger, C3HC4 type (RING finger) domain-containing protein
TGME49_293480	1.93	7.21	4.24E-05	0.00125452	MoeA N-terminal region (domain I and II) domain-containing
TGME49_239410	-1.36	7.88	4.37E-05	0.0012803	hypothetical protein
TGME49_237410	-1.64	6.56	4.38E-05	0.0012803	protein phosphatase 2C domain-containing protein
TGME49_257180	-1.98	5.94	4.47E-05	0.00129844	RecF/RecN/SMC N terminal domain-containing protein
TGME49_217350	-2.50	5.20	4.57E-05	0.00132174	methyltransferase MTA70, putative
TGME49_230140	2.10	5.26	4.66E-05	0.00134276	vacuolar sorting protein 9 (vps9) domain-containing protein
TGME49_319580	-2.55	5.43	4.68E-05	0.001345	hypothetical protein
TGME49_281450	-1.99	5.73	4.92E-05	0.00140719	cell-cycle-associated protein kinase, putative
TGME49_203390	-1.39	7.31	4.97E-05	0.00141688	CRAL/TRIO domain-containing protein
TGME49_295950	1.65	5.58	5.03E-05	0.00142151	KRUF family protein
TGME49_270580	2.21	4.80	5.06E-05	0.00142551	HECT-domain (ubiquitin-transferase) domain-containing protein
TGME49_243465	3.08	4.46	5.16E-05	0.00143746	hypothetical protein
TGME49_253860	1.32	6.69	5.13E-05	0.00143746	Tyrosine kinase-like (TKL) protein
TGME49_251460	-1.84	6.07	5.15E-05	0.00143746	hypothetical protein
TGME49_253100	1.15	7.21	5.22E-05	0.00144494	hypothetical protein
TGME49_222040	-2.70	5.45	5.23E-05	0.00144494	Ran-interacting Mog1 protein
TGME49_289310	-1.53	6.48	5.42E-05	0.00148763	cullin family protein
TGME49_228360	1.39	7.28	5.49E-05	0.00150005	peptidyl-prolyl isomerase FKBP12, putative
TGME49_201260	-1.25	8.89	5.60E-05	0.00151997	sugar transporter ST3
TGME49_228630	-1.29	6.96	5.62E-05	0.00151997	hypothetical protein
TGME49_232960	-1.48	7.01	5.61E-05	0.00151997	oxidoreductase, 2OG-Fe(II) oxygenase family protein
TGME49_262825	2.05	4.65	5.72E-05	0.00154004	peptidase family c50 protein
TGME49_263060	1.64	7.25	5.98E-05	0.00160441	Proteasome/cyclosome repeat-containing protein
TGME49_240950	-3.19	5.11	6.00E-05	0.00160441	hypothetical protein
TGME49_240440	1.45	5.78	6.09E-05	0.0016228	hypothetical protein
TGME49_316270	1.43	6.15	6.13E-05	0.00162808	Rab geranylgeranyl transferase type II beta subunit, putative
TGME49_289720	-1.72	6.89	6.25E-05	0.00164683	hypothetical protein
TGME49_273400	-2.22	5.01	6.25E-05	0.00164683	hypothetical protein
TGME49_288580	2.50	4.44	6.33E-05	0.00165757	RNA methylase, putative
TGME49_287160	-1.25	7.58	6.32E-05	0.00165757	internal kinesin motor domain protein
TGME49_213635	2.50	5.03	6.41E-05	0.00167208	hypothetical protein
TGME49_298610	-1.15	7.94	6.46E-05	0.0016728	GYF domain-containing protein
TGME49_203730	-2.06	5.79	6.45E-05	0.0016728	hypothetical protein
TGME49_313230	2.39	9.56	6.55E-05	0.00169215	eukaryotic initiation factor-2, alpha subunit
TGME49_226755	1.53	6.33	6.75E-05	0.0017362	3'5'-cyclic nucleotide phosphodiesterase domain-containing
TGME49_253600	1.77	6.44	6.97E-05	0.0017858	hypothetical protein
TGME49_254630	1.34	6.41	7.09E-05	0.00180609	CMGC kinase
TGME49_309070	-2.16	5.42	7.10E-05	0.00180609	hypothetical protein
TGME49_233680	-2.80	4.92	7.12E-05	0.00180609	nuclear movement family protein
TGME49_229470	-1.47	6.59	7.39E-05	0.00185511	hypothetical protein
TGME49_207180	2.21	4.68	7.45E-05	0.0018629	indole-3-glycerol phosphate synthase domain-containing protein
TGME49_290740	-2.43	5.25	7.47E-05	0.0018629	hypothetical protein
TGME49_230430	-2.22	5.37	7.55E-05	0.00187748	vesicle-associated membrane protein, putative
TGME49_294060	1.52	6.68	7.60E-05	0.00188327	hypothetical protein
TGME49_300980	2.31	6.38	7.63E-05	0.00188405	hypothetical protein
TGME49_286510	1.70	5.67	7.76E-05	0.00190332	hypothetical protein
TGME49_248130	-2.53	5.04	7.75E-05	0.00190332	hypothetical protein
TGME49_285470	2.15	5.47	7.89E-05	0.00193003	patched family protein
TGME49_283550	1.34	6.70	8.37E-05	0.00202647	hypothetical protein
TGME49_300055	-1.16	8.63	8.45E-05	0.00203886	hypothetical protein
TGME49_297780	-1.71	6.48	8.68E-05	0.00207456	ATPase/histidine kinase/DNA gyrase B/HSP90 domain-containing
TGME49_293040	-1.91	6.54	8.80E-05	0.00208882	hypothetical protein
TGME49_258590	-2.37	5.43	8.99E-05	0.00212727	hypothetical protein
TGME49_215785	2.21	9.54	9.18E-05	0.0021639	rhoptry protein ROP2A
TGME49_214400	1.75	4.87	9.20E-05	0.0021639	hypothetical protein

TGME49_237290	-1.50	6.65	9.23E-05	0.0021639	hypothetical protein
TGME49_268810	-1.75	6.39	9.49E-05	0.0022065	ck2 beta subunit
TGME49_237830	-1.92	5.67	9.50E-05	0.0022065	DNA polymerase I domain-containing protein
TGME49_226380	1.18	8.18	9.53E-05	0.00220694	hypothetical protein
TGME49_227335	2.40	5.11	9.70E-05	0.00222412	hypothetical protein
TGME49_274070	-2.79	5.15	9.69E-05	0.00222412	ThiF family protein
TGME49_215400	-2.20	5.40	9.82E-05	0.00224455	RNA recognition motif-containing protein
TGME49_241300	1.80	6.54	0.00010022	0.00228465	hypothetical protein
TGME49_253140	1.54	5.83	0.0001029	0.0023386	hypothetical protein
TGME49_305990	3.44	4.61	0.00010446	0.00236674	hypothetical protein
TGME49_288400	-1.71	6.11	0.00010768	0.00241755	LETM1 family protein
TGME49_273530	-2.31	5.13	0.00010751	0.00241755	flagellar associated protein
TGME49_277540	2.13	4.45	0.00011213	0.00249299	hypothetical protein
TGME49_220500	-1.33	7.82	0.00011272	0.00249299	UBX domain-containing protein
TGME49_225440	-4.18	4.61	0.00011257	0.00249299	hypothetical protein
TGME49_293840	1.98	5.98	0.00011632	0.00255037	hypothetical protein
TGME49_212090	1.14	6.72	0.00011646	0.00255037	hypothetical protein
TGME49_244230	-2.07	5.98	0.00011621	0.00255037	hypothetical protein
TGME49_258410	1.26	9.04	0.00011772	0.00256533	photosensitized INA-labeled protein PHIL1
TGME49_248160	-2.51	4.95	0.00012128	0.00262737	hypothetical protein
TGME49_261660	1.96	5.03	0.00012207	0.00263668	hypothetical protein
TGME49_241610	1.80	6.29	0.00012295	0.00264546	hypothetical protein
TGME49_249390	-1.14	8.70	0.00012319	0.00264546	glutamate/leucine/phenylalanine/valine dehydrogenase family
TGME49_205330	1.10	7.80	0.00012409	0.0026572	hypothetical protein
TGME49_214970	-1.38	8.09	0.00012606	0.0026916	DNA replication licensing factor, putative
TGME49_291930	3.85	9.99	0.00012661	0.00269557	RNA recognition motif-containing protein
TGME49_309990	1.26	6.25	0.00012806	0.00271854	hypothetical protein
TGME49_210380	-1.30	6.94	0.00012882	0.00272686	hypothetical protein
TGME49_213570	4.50	11.16	0.00012919	0.00272706	hypothetical protein
TGME49_221675	1.12	7.42	0.00013215	0.00278151	hypothetical protein
TGME49_246530	2.25	5.05	0.00013406	0.00281351	phospholipase D active site domain-containing protein
TGME49_289620	1.19	8.43	0.00013443	0.00281351	cathepsin CPC1
TGME49_294890	-1.47	6.50	0.00013489	0.00281516	hypothetical protein
TGME49_202580	2.00	6.20	0.00013583	0.00281888	ATPase, AAA family protein
TGME49_266860	1.40	7.09	0.00013555	0.00281888	BTB/POZ domain-containing protein
TGME49_312270	1.16	8.08	0.00013814	0.00285658	rhoptry protein ROP13
TGME49_266280	-1.44	6.56	0.00013841	0.00285658	HEAT repeat-containing protein
TGME49_218960	1.65	7.78	0.00014012	0.00287581	AP2 domain transcription factor AP2XII-1
TGME49_263860	-2.07	5.11	0.00014061	0.00287785	hypothetical protein
TGME49_310130	-1.62	6.23	0.00014159	0.00288993	Spc97 / Spc98 family protein
TGME49_316255	1.05	7.55	0.00014433	0.00293772	hypothetical protein
TGME49_254720	1.69	9.40	0.00014684	0.00297184	dense granule protein GRA8
TGME49_271930	-1.06	8.48	0.00014721	0.00297184	hypothetical protein
TGME49_225130	-2.67	5.10	0.00014677	0.00297184	hypothetical protein
TGME49_301130	-2.25	5.52	0.00015178	0.00304743	hypothetical protein
TGME49_248400	1.31	6.18	0.0001524	0.00305179	glyoxalase I, putative
TGME49_270830	1.20	7.26	0.00015443	0.00308407	small nuclear ribonucleoprotein
TGME49_221330	-1.48	6.74	0.00015494	0.00308595	DNA gyrase/topoisomerase IV, A subunit domain-containing
TGME49_255510	-1.22	7.27	0.00015579	0.00309455	ankyrin repeat-containing protein
TGME49_253780	1.32	6.63	0.00015745	0.00311917	GTP cyclohydrolase I, putative
TGME49_238240	4.88	11.68	0.0001588	0.00313744	bystin protein
TGME49_208910	-1.58	6.43	0.0001609	0.00317054	hypothetical protein
TGME49_225470	1.44	5.79	0.00016156	0.00317497	peptide methionine sulfoxide reductase
TGME49_310390	1.86	6.89	0.00016648	0.00326315	hypothetical protein
TGME49_279410	1.27	7.09	0.00016722	0.00326892	hypothetical protein
TGME49_270550	1.65	5.47	0.00016962	0.00329848	gamma-glutamyl phosphate reductase, putative
TGME49_247590	1.92	5.01	0.00017076	0.00330331	methyltransferase domain-containing protein

TGME49_262880	1.76	5.55	0.00017042	0.00330331	hypothetical protein
TGME49_293380	-2.19	5.59	0.00017376	0.00335252	histone lysine acetyltransferase HAT1
TGME49_224520	-1.10	7.89	0.00017459	0.00335988	alveolin domain containing intermediate filament IMC8
TGME49_259240	4.14	11.70	0.00017549	0.00336838	ribosomal protein RPS21
TGME49_319530	-1.18	8.07	0.00017696	0.00338776	splicing factor SF2
TGME49_312105	-2.30	6.27	0.00017757	0.00339073	hypothetical protein
TGME49_289940	1.77	5.27	0.00017975	0.00342353	uroporphyrinogen decarboxylase
TGME49_226240	1.50	5.23	0.0001827	0.00347079	bud site selection protein, putative
TGME49_244380	2.38	8.24	0.00018547	0.00350539	cactin
TGME49_283820	-2.80	4.90	0.00018615	0.00350931	glycine cleavage T-protein (aminomethyl transferase) domain-
TGME49_241305	1.59	6.01	0.00018883	0.00353294	hypothetical protein
TGME49_296121	1.39	7.18	0.00018847	0.00353294	hypothetical protein
TGME49_288890	1.67	5.64	0.00019266	0.00358651	hypothetical protein
TGME49_299240	1.61	5.35	0.00019322	0.00358791	hypothetical protein
TGME49_255410	-1.45	6.98	0.00019633	0.00362751	hypothetical protein
TGME49_288000	1.31	7.03	0.0001972	0.00363445	hypothetical protein
TGME49_216020	-1.98	5.94	0.00019919	0.00365885	peptidase family c78 protein
TGME49_220640	-1.66	7.29	0.00020186	0.00369288	hypothetical protein
TGME49_320050	7.35	16.23	0.00021625	0.00392691	ribosomal protein RPL5
TGME49_278800	-1.23	7.30	0.00021783	0.00394595	zinc finger protein 36 family 3 protein
TGME49_298830	2.52	4.79	0.00022148	0.00400224	hypothetical protein
TGME49_253330	1.55	7.81	0.00022257	0.00401228	Rhoptry kinase family protein, truncated (incomplete catalytic
TGME49_265840	-1.35	6.56	0.00022337	0.00401601	hypothetical protein
TGME49_297730	-1.93	5.78	0.00022386	0.00401601	transcription elongation factor 1, putative
TGME49_239490	1.17	8.02	0.00022473	0.00402174	dehydrogenase E1 component family protein
TGME49_226680	-1.19	7.34	0.00023	0.00410621	hypothetical protein
TGME49_260790	-1.74	5.56	0.0002354	0.00418251	RAP domain-containing protein
TGME49_283710	-1.48	6.77	0.00024452	0.00433408	Longevity-assurance protein (LAG1) domain-containing protein
TGME49_266450	1.70	5.91	0.00024531	0.00433776	lysine decarboxylase family protein
TGME49_306890	2.64	4.80	0.00024657	0.00434957	hypothetical protein
TGME49_250100	1.44	6.54	0.00025068	0.00439028	hypothetical protein
TGME49_263710	-1.52	6.20	0.00025304	0.00441129	acyl-CoA:cholesterol acyltransferase alpha ACAT1-alpha
TGME49_236050	-3.84	4.86	0.00025378	0.00441379	fructose-bisphosphate aldolase, putative
TGME49_225250	1.27	5.81	0.00025579	0.00441765	LSU ribosomal protein L14P, putative
TGME49_311920	-1.24	7.15	0.00025537	0.00441765	GRAM domain-containing protein
TGME49_300240	-1.77	5.46	0.00025472	0.00441765	syntaxin 6, n-terminal protein
TGME49_203180	1.79	5.27	0.00025727	0.00443274	leucine rich repeat-containing protein
TGME49_267460	2.01	4.67	0.00026012	0.00445353	AP2 domain transcription factor AP2IX-1
TGME49_209985	1.74	5.16	0.00025946	0.00445353	cAMP-dependent protein kinase
TGME49_295990	1.16	6.85	0.0002603	0.00445353	ubiquitin conjugating enzyme E2, putative
TGME49_252630	1.14	8.01	0.00026088	0.00445353	hypothetical protein
TGME49_209440	1.71	6.17	0.00026233	0.00445811	hypothetical protein
TGME49_228160	1.14	7.00	0.00026273	0.00445811	acid phosphatase
TGME49_221410	-1.40	6.55	0.00026295	0.00445811	actin-like protein ALP4
TGME49_214270	2.82	9.52	0.00026521	0.00447603	translation initiation factor IF-2, putative
TGME49_280700	-1.43	7.01	0.00026734	0.00450165	arginine decarboxylase
TGME49_204055	-2.14	5.00	0.00026814	0.00450486	hypothetical protein
TGME49_290670	1.08	8.78	0.00027077	0.00453881	leucyl aminopeptidase LAP
TGME49_315620	-1.29	6.98	0.00027423	0.0045864	vacuolar ATP synthase subunit C, putative
TGME49_271270	1.10	7.35	0.0002788	0.00465236	hypothetical protein
TGME49_316230	-1.61	5.94	0.0002813	0.00468346	SAC1 phosphoinositide phosphatase, putative
TGME49_252430	1.05	7.44	0.0002845	0.00471556	hypothetical protein
TGME49_308010	-1.98	6.49	0.00028614	0.00473223	hypothetical protein
TGME49_295030	-1.06	8.09	0.00029037	0.00478077	hypothetical protein
TGME49_289650	1.03	8.20	0.00029167	0.00479155	PEP-carboxykinase I
TGME49_233160	-1.12	7.28	0.0002956	0.00484537	hypothetical protein

TGME49_208730	0.96	8.09	0.00029809	0.00487545	microneme protein, putative
TGME49_321410	2.80	8.06	0.00029938	0.0048857	hypothetical protein
TGME49_315300	-1.40	6.20	0.00030653	0.00496954	transcription factor IIB, putative
TGME49_262120	-1.16	7.22	0.00030846	0.00498998	IQ calmodulin-binding motif domain-containing protein
TGME49_289910	1.02	7.75	0.00031333	0.00505327	hypothetical protein
TGME49_231960	-1.88	6.35	0.00031374	0.00505327	omega secalin, putative
TGME49_212210	1.36	6.79	0.00032217	0.00516652	hypothetical protein
TGME49_214100	-1.36	7.77	0.00032199	0.00516652	hypothetical protein
TGME49_314790	-1.76	6.88	0.00032407	0.00518586	small nuclear ribonucleoprotein G, putative
TGME49_293260	1.75	7.14	0.00032615	0.00520779	ATPase/histidine kinase/DNA gyrase B/HSP90 domain-containing
TGME49_269710	-1.40	6.75	0.00033646	0.00534941	hypothetical protein
TGME49_211440	-1.48	6.23	0.00033624	0.00534941	hypothetical protein
TGME49_251500	-1.24	7.59	0.00034002	0.00539438	eukaryotic initiation factor-3, subunit 3, putative
TGME49_254365	1.08	7.70	0.0003419	0.00540118	phosphatidate cytidylyltransferase
TGME49_246330	-1.24	6.92	0.00034164	0.00540118	CRAL/TRIO domain-containing protein
TGME49_214090	1.76	7.04	0.00034265	0.00540148	signal peptidase
TGME49_288040	-1.30	6.46	0.00034627	0.00544692	hypothetical protein
TGME49_262935	2.22	4.84	0.00034817	0.00544949	hypothetical protein
TGME49_239710	-1.71	5.95	0.00034791	0.00544949	phosphomannomutase
TGME49_267420	0.99	8.24	0.00035022	0.00546268	mago nashi family protein 2, putative
TGME49_304520	-1.27	6.73	0.00035162	0.00547301	hypothetical protein
TGME49_202770	-1.12	8.91	0.00035311	0.00548467	RNA recognition motif-containing protein
TGME49_226710	0.97	8.58	0.0003614	0.00554371	hypothetical protein
TGME49_321630	-1.29	6.33	0.00036105	0.00554371	RNA recognition motif-containing protein
TGME49_207060	-1.46	8.77	0.00035959	0.00554371	ribonucleoside-diphosphate reductase small subunit
TGME49_300285	-1.55	5.96	0.00036094	0.00554371	hypothetical protein
TGME49_235740	-1.65	5.82	0.00035943	0.00554371	hypothetical protein
TGME49_249180	0.99	8.01	0.00036305	0.00555746	bifunctional dihydrofolate reductase-thymidylate synthase
TGME49_258490	-1.76	5.25	0.00036996	0.00565161	hypothetical protein
TGME49_222952	2.17	5.22	0.00038641	0.00587864	phosphohistidine phosphatase
TGME49_285230	-1.63	6.69	0.00038622	0.00587864	PRP38 family protein
TGME49_290310	-2.41	6.02	0.00038864	0.00590048	hypothetical protein
TGME49_223600	-1.87	5.52	0.00039524	0.00598841	hypothetical protein
TGME49_213820	1.24	6.73	0.00039811	0.00601956	hypothetical protein
TGME49_204270	-1.57	6.40	0.00040307	0.00608218	hypothetical protein
TGME49_225050	1.08	7.65	0.00040524	0.00610251	adenosylhomocysteinase, putative
TGME49_263480	1.83	5.37	0.00040763	0.00610615	sodium/hydrogen exchanger 3 protein
TGME49_265450	1.53	7.11	0.00040787	0.00610615	hexokinase
TGME49_275630	1.12	7.37	0.00040796	0.00610615	HECT-domain (ubiquitin-transferase) domain-containing protein
TGME49_237160	-1.53	5.78	0.00041754	0.00623699	hypothetical protein
TGME49_262780	-1.49	6.39	0.00041856	0.0062396	FHA domain-containing protein
TGME49_244130	1.91	5.94	0.00042033	0.00624092	hypothetical protein
TGME49_265650	-1.60	5.56	0.00041967	0.00624092	protein phosphatase 2C domain-containing protein
TGME49_242640	1.08	6.51	0.0004232	0.00627094	hypothetical protein
TGME49_208590	-1.26	7.09	0.00042612	0.00630161	vacuolar ATP synthase subunit 54kD, putative
TGME49_305340	-1.03	8.17	0.00044081	0.00648002	corepressor complex CRC230
TGME49_217780	1.70	5.16	0.00044333	0.00650423	Sec20 protein
TGME49_299250	1.42	5.36	0.00045182	0.00661569	hypothetical protein
TGME49_314070	1.15	6.15	0.000459	0.00670762	hypothetical protein
TGME49_258740	-1.51	6.23	0.00046744	0.0068174	eukaryotic initiation factor-2A, putative
TGME49_269425	-1.35	7.07	0.00047062	0.00685029	hypothetical protein
TGME49_231140	3.19	11.22	0.00047452	0.00687061	ribosomal protein RPS25
TGME49_305950	1.63	5.38	0.00047425	0.00687061	tetratricopeptide repeat-containing protein
TGME49_285170	-3.11	4.61	0.00047572	0.00687061	methyltransferase small, putative
TGME49_245460	4.21	12.61	0.00047767	0.00688535	ribosomal protein RPS8
TGME49_320620	1.73	5.12	0.0004796	0.00689979	queuine tRNA ribosyl transferase
TGME49_309580	-1.39	6.11	0.00048374	0.00694583	transporter, major facilitator family protein
TGME49_254950	1.60	5.04	0.00048672	0.00697508	RNA cap guanine-N2 methyltransferase

TGME49_305080	2.30	4.81	0.00049099	0.00702269	hypothetical protein
TGME49_275990	1.24	6.60	0.00049527	0.00706887	hypothetical protein
TGME49_268380	2.64	6.24	0.00052	0.00739476	RNA recognition motif-containing protein
TGME49_300060	1.24	6.85	0.00052358	0.00743136	signal peptidase subunit protein
TGME49_246080	-1.16	6.89	0.00052813	0.00748169	NAD dependent epimerase/dehydratase family protein
TGME49_275430	-1.69	5.84	0.00053265	0.00753131	hypothetical protein
TGME49_231100	1.16	6.73	0.00053481	0.00754744	hypothetical protein
TGME49_237110	-1.34	6.44	0.00053862	0.00758672	replication factor C subunit 2, putative
TGME49_289680	1.16	7.08	0.00054313	0.00763581	Ras-related protein Rab11
TGME49_249350	-1.19	7.35	0.00054694	0.00764583	esterase/lipase/thioesterase domain-containing protein
TGME49_248900	-1.98	5.41	0.00054513	0.00764583	hypothetical protein
TGME49_253950	1.35	5.56	0.00054826	0.00764878	protein fam50a, putative
TGME49_224060	0.99	7.95	0.00055025	0.00764878	thioredoxin, putative
TGME49_216375	-2.42	4.74	0.00055157	0.00765273	hypothetical protein
TGME49_318370	2.04	5.00	0.00055391	0.00767091	hypothetical protein
TGME49_218830	-2.05	5.10	0.00057705	0.00794673	hypothetical protein
TGME49_215010	-1.06	7.02	0.0005828	0.00799626	hypothetical protein
TGME49_217360	-1.96	5.84	0.00058393	0.00799686	hypothetical protein
TGME49_310360	-1.15	7.38	0.00059373	0.00810117	hypothetical protein
TGME49_218730	-1.39	6.81	0.00059296	0.00810117	hypothetical protein
TGME49_209945	2.28	4.49	0.0006056	0.00823288	hypothetical protein
TGME49_272010	-1.63	6.93	0.0006128	0.00831541	Gar1 protein RNA binding region protein
TGME49_308060	1.48	6.55	0.00061415	0.00831855	hypothetical protein
TGME49_260580	1.56	6.09	0.00061937	0.00837392	hypothetical protein
TGME49_265440	-1.26	6.82	0.00062989	0.00846661	hypothetical protein
TGME49_235980	-1.64	5.63	0.0006308	0.00846661	ARIADNE family protein
TGME49_214170	-1.70	5.90	0.0006354	0.00851296	hypothetical protein
TGME49_309400	-1.56	6.01	0.0006375	0.00852568	RecF/RecN/SMC N terminal domain-containing protein
TGME49_270140	-1.57	5.92	0.0006411	0.00855835	splicing factor DIM1, putative
TGME49_211250	-1.10	7.63	0.00064403	0.00856661	hypothetical protein
TGME49_312260	-1.42	5.95	0.00064316	0.00856661	hypothetical protein
TGME49_321560	-1.35	6.59	0.00064707	0.00859167	zinc knuckle domain-containing protein
TGME49_321640	-1.69	6.04	0.00065086	0.00862658	cell division protein CDC48AP
TGME49_268170	-1.89	5.31	0.00065339	0.00864453	hypothetical protein
TGME49_254915	3.02	4.55	0.00066328	0.0087442	hypothetical protein
TGME49_255290	-2.99	4.89	0.00066673	0.00877412	hypothetical protein
TGME49_222370	1.40	6.71	0.00067193	0.00882679	SAG-related sequence SRS13
TGME49_215347	-2.65	4.97	0.00067394	0.00883758	hypothetical protein
TGME49_286480	2.29	5.39	0.0006761	0.008839	hypothetical protein
TGME49_217220	-1.22	6.81	0.00067716	0.008839	zinc finger, C3HC4 type (RING finger) domain-containing protein
TGME49_240060	1.23	8.72	0.00068223	0.00888332	hypothetical protein
TGME49_268200	1.63	7.01	0.00069309	0.00898336	RNA recognition motif-containing protein
TGME49_272640	-1.40	6.48	0.00069355	0.00898336	eukaryotic initiation factor-2B, epsilon subunit, putative
TGME49_321570	1.11	6.61	0.00069503	0.00898689	beta-hydroxyacyl-acyl carrier protein dehydratase (FABZ)
TGME49_216040	-2.01	6.31	0.00070024	0.00903844	30S ribosomal protein S15, putative
TGME49_232050	-1.13	7.68	0.00070722	0.00909681	DnaJ domain-containing protein
TGME49_228150	1.93	6.33	0.0007139	0.00915104	hypothetical protein
TGME49_250955	1.98	5.63	0.00071938	0.00920537	KRUF family protein
TGME49_228320	-3.53	4.54	0.00072295	0.00923509	hypothetical protein
TGME49_264820	1.40	6.10	0.00072852	0.00929024	RbAp48
TGME49_232130	-1.00	8.66	0.00073791	0.00939371	hypothetical protein
TGME49_290300	1.03	7.98	0.00073961	0.00939929	hypothetical protein
TGME49_255340	-1.52	5.95	0.00074343	0.00943164	tetratricopeptide repeat-containing protein
TGME49_207230	1.10	6.18	0.00074805	0.00945712	hypothetical protein
TGME49_240740	-1.49	5.71	0.00074927	0.00945712	Sec1 family protein
TGME49_228300	-1.88	5.32	0.00074914	0.00945712	CCDC25 protein
TGME49_215040	-1.26	6.56	0.00076411	0.00962803	HEAT repeat-containing protein

TGME49_313590	-1.70	5.92	0.00076667	0.00964385	hypothetical protein
TGME49_294690	-1.04	7.30	0.00076906	0.00965761	rhomboid protease ROM5
TGME49_254730	1.74	4.90	0.00077141	0.00967069	POPLD (NUC188) domain-containing protein
TGME49_289150	-1.30	6.48	0.00077623	0.00971466	hypothetical protein
TGME49_300048	1.09	6.93	0.00078522	0.00981058	hypothetical protein
TGME49_281570	-1.64	5.86	0.00079109	0.00985069	hypothetical protein
TGME49_290840	-1.24	6.05	0.0008008	0.00995486	serine protease
TGME49_313180	-0.96	8.51	0.00081961	0.01017171	cell-cycle-associated protein kinase PRP4, putative
TGME49_289320	-1.06	7.44	0.00082927	0.01025716	casein kinase I, putative
TGME49_294730	-1.44	6.07	0.00082918	0.01025716	hypothetical protein
TGME49_251640	-1.48	5.81	0.00083166	0.01026965	ubiquitin-conjugating enzyme subfamily protein
TGME49_266990	-1.05	7.64	0.00083493	0.01029278	beta-COP
TGME49_288360	1.42	9.40	0.0008403	0.0103247	tryptophanyl-tRNA synthetase (TrpRS2)
TGME49_263610	-1.62	6.26	0.00084002	0.0103247	hypothetical protein
TGME49_280560	-1.19	6.83	0.00085399	0.01047554	selenide, water dikinase
TGME49_251790	1.23	6.10	0.00085793	0.01050644	hypothetical protein
TGME49_318360	-1.34	6.53	0.00086386	0.01054417	hypothetical protein
TGME49_266900	1.15	8.02	0.00086671	0.01056157	cyclin, N-terminal domain-containing protein
TGME49_225800	1.18	6.01	0.00086896	0.01057164	iron-sulfur assembly ATPase
TGME49_261850	2.02	5.09	0.00087126	0.01057681	helicase, putative
TGME49_288840	1.13	7.00	0.00087734	0.01057681	hypothetical protein
TGME49_253000	1.04	6.88	0.00087673	0.01057681	ELMO/CED-12 family protein
TGME49_318320	-1.94	5.30	0.00087448	0.01057681	ATP-dependent Clp endopeptidase, proteolytic subunit ClpP
TGME49_201200	-2.28	4.87	0.00087795	0.01057681	zinc finger (CCCH type) motif-containing protein
TGME49_288700	-1.75	5.73	0.00088954	0.01069907	RecF/RecN/SMC N terminal domain-containing protein
TGME49_262430	1.49	5.77	0.00090469	0.01085163	4-hydroxy-3-methylbut-2-en-1-yl diphosphate synthase
TGME49_244260	-2.77	4.83	0.00090516	0.01085163	hypothetical protein
TGME49_254930	0.99	7.07	0.00091218	0.01090913	hypothetical protein
TGME49_214210	-1.23	8.20	0.0009129	0.01090913	rRNA pseudouridine synthase
TGME49_223950	1.07	6.29	0.00092139	0.01099293	hypothetical protein
TGME49_202250	1.21	5.90	0.00092719	0.01104432	hypothetical protein
TGME49_270650	1.57	5.21	0.00093035	0.01105672	deoxyribose-phosphate aldolase
TGME49_260610	-2.51	4.95	0.00093122	0.01105672	methyltransferase
TGME49_215710	-1.39	6.00	0.00094546	0.01119001	hypothetical protein
TGME49_318570	2.26	5.58	0.00094726	0.01119334	SFT2 family protein
TGME49_202650	-1.59	5.60	0.00095246	0.01123692	hypothetical protein
TGME49_226830	-1.17	8.44	0.00097006	0.01138546	DnaK family protein
TGME49_312380	-1.95	5.23	0.00097797	0.01141062	tetratricopeptide repeat-containing protein
TGME49_218200	-2.13	5.22	0.00097766	0.01141062	UDP-sugar pyrophospharylase
TGME49_260390	-1.48	5.62	0.00098353	0.01143952	hypothetical protein
TGME49_267450	-1.51	6.10	0.00098351	0.01143952	alpha-tubulin suppressor protein
TGME49_319930	-1.46	6.12	0.00099318	0.01153365	hypothetical protein
TGME49_253370	1.14	7.36	0.00099554	0.01154296	hypothetical protein
TGME49_298840	1.20	6.46	0.00101017	0.01169428	hypothetical protein
TGME49_235490	-1.11	6.76	0.00102453	0.01184208	hypothetical protein
TGME49_300290	-1.47	5.92	0.00103474	0.01194136	SNARE domain-containing protein
TGME49_311310	-1.09	8.09	0.00103868	0.01196827	protein phosphatase 2B catalytic subunit, calcineurin family
TGME49_225560	-1.13	8.81	0.0010552	0.0121397	hypothetical protein
TGME49_269430	-1.63	5.52	0.00105913	0.01216606	polyprenyl synthetase superfamily protein
TGME49_267620	1.08	6.83	0.0010627	0.0121882	multi-pass transmembrane protein
TGME49_268580	-1.25	6.39	0.00107221	0.01225929	hypothetical protein
TGME49_253560	1.29	5.72	0.00107853	0.01231252	hypothetical protein
TGME49_319308	-1.95	5.73	0.00108228	0.01233634	hypothetical protein
TGME49_252230	1.47	6.21	0.00108922	0.01239642	hypothetical protein

TGME49_282170	1.22	6.39	0.00109738	0.01241297	hypothetical protein
TGME49_294820	1.18	7.52	0.00109703	0.01241297	type I fatty acid synthase, putative
TGME49_265390	-1.40	6.56	0.00109428	0.01241297	hypothetical protein
TGME49_233760	-1.53	6.40	0.00109293	0.01241297	hypothetical protein
TGME49_309280	-1.70	5.10	0.0011053	0.01246828	zinc finger, C3HC4 type (RING finger) domain-containing protein
TGME49_229250	1.53	9.83	0.00110783	0.01247313	ribosomal protein RPL28
TGME49_313830	-1.30	6.30	0.00110943	0.01247313	AARP2CN (NUC121) domain-containing protein
TGME49_235020	-0.99	7.55	0.00111319	0.01249519	COPI protein, putative
TGME49_269420	2.18	6.35	0.00113009	0.01264782	hypothetical protein
TGME49_211020	-1.69	6.55	0.00113601	0.01267574	RNA recognition motif-containing protein
TGME49_252260	1.73	4.57	0.00114403	0.01274607	hypothetical protein
TGME49_254110	1.55	5.41	0.00114632	0.01274743	tryptophanyl-tRNA synthetase (TrpRS1)
TGME49_264840	-2.19	4.93	0.0011476	0.01274743	ATP-dependent DNA helicase, RecQ family protein
TGME49_263085	-1.79	5.01	0.00114944	0.01274879	hypothetical protein
TGME49_204420	-1.23	5.87	0.00115475	0.01278859	oocyst wall protein OWP1
TGME49_214180	-0.96	7.70	0.00116403	0.01287208	ENTH domain-containing protein
TGME49_222920	1.22	6.27	0.00117192	0.01292891	mbp-1 interacting protein-2a family protein
TGME49_212130	-1.33	6.44	0.00117307	0.01292891	phospholipase, patatin family protein
TGME49_316140	-1.10	6.99	0.00117961	0.01296697	hypothetical protein
TGME49_278770	-1.28	7.00	0.00118238	0.01297811	hypothetical protein
TGME49_270930	-1.14	6.53	0.00118592	0.01299771	hypothetical protein
TGME49_220940	1.41	5.59	0.00124934	0.01365246	ribosomal RNA methyltransferase (FtsJ) family protein
TGME49_253730	1.23	6.80	0.00125547	0.01367906	importin-beta N-terminal domain-containing protein
TGME49_225060	-1.19	6.75	0.00125546	0.01367906	nucleoredoxin family protein
TGME49_234360	-1.04	6.88	0.00128091	0.01389496	DNA topoisomerase I, putative
TGME49_249950	-1.55	5.77	0.00127981	0.01389496	Mak16 protein
TGME49_214530	-1.85	6.70	0.00127734	0.01389496	DnaJ domain-containing protein
TGME49_267580	2.00	5.90	0.00130161	0.01409886	cyclin2 related protein
TGME49_204880	1.39	5.85	0.00130853	0.01412612	hypothetical protein
TGME49_311510	0.88	8.03	0.00130801	0.01412612	eIF2 kinase IF2K-B
TGME49_216920	-1.58	5.80	0.00130985	0.01412612	mediator complex subunit MED8
TGME49_310910	1.39	5.43	0.00132952	0.01425532	WD domain, G-beta repeat-containing protein
TGME49_231210	-1.13	7.07	0.00132694	0.01425532	sarcalumenin/eps15 family protein
TGME49_314660	-1.21	6.93	0.00132826	0.01425532	TPRX1 protein
TGME49_224730	-1.53	5.88	0.00133151	0.01425603	hypothetical protein
TGME49_301216	-2.44	4.54	0.00133865	0.01429114	endonuclease/exonuclease/phosphatase family protein
TGME49_244600	-1.52	5.95	0.0013467	0.01435642	hypothetical protein
TGME49_212220	0.94	7.85	0.00136052	0.01448288	hypothetical protein
TGME49_292110	1.45	6.61	0.00138528	0.01470267	formate/nitrite transporter protein
TGME49_249620	-1.31	6.08	0.00138712	0.01470267	histone deacetylase HDAC2
TGME49_207680	-1.13	7.36	0.00138918	0.0147035	suppressor of kinetochore protein 1, putative
TGME49_289710	1.56	6.31	0.00139221	0.01471456	AP2 domain transcription factor AP2IX-5
TGME49_312050	-0.96	7.51	0.00140219	0.0147824	small GTPase Rab2, putative
TGME49_220290	-1.77	5.53	0.00142665	0.01501422	hypothetical protein
TGME49_313350	-1.35	5.79	0.00142881	0.0150157	hypothetical protein
TGME49_261540	1.31	5.45	0.00144	0.01511188	DNA-directed RNA polymerase I RPAC2
TGME49_262980	-1.74	5.57	0.0014427	0.0151188	hypothetical protein
TGME49_229350	-1.31	6.25	0.00145963	0.01527456	HEAT repeat-containing protein
TGME49_299780	1.00	8.50	0.00147029	0.01532119	hypothetical protein
TGME49_268900	-1.44	5.76	0.00146848	0.01532119	dense granular protein GRA10
TGME49_280710	-1.25	8.04	0.00147353	0.01533344	20S proteasome subunit beta 7, putative
TGME49_237260	-1.08	6.71	0.00148539	0.01543517	Coiled-coil domain containing 124 family protein
TGME49_263595	-1.57	5.60	0.00149897	0.01553266	RNA-binding protein
TGME49_246800	-1.24	6.38	0.00150353	0.01555818	acylaminoacyl-peptidase, putative
TGME49_205700	1.48	10.39	0.0015088	0.01559093	cyclophilin precursor
TGME49_268230	-2.84	5.64	0.00151721	0.01563428	hypothetical protein
TGME49_212800	-1.49	6.59	0.00152503	0.015693	hypothetical protein

TGME49_273320	-0.86	8.00	0.00153692	0.01579341	hypothetical protein
TGME49_231230	-2.42	4.52	0.00155329	0.01589543	hypothetical protein
TGME49_214580	-3.68	4.76	0.00155806	0.01592232	tetratricopeptide repeat-containing protein
TGME49_209095	-1.13	6.83	0.00156533	0.01597459	hypothetical protein
TGME49_259600	1.52	5.22	0.00160114	0.01631753	hypothetical protein
TGME49_208820	1.15	5.90	0.00161946	0.0164156	1-deoxy-D-xylulose-5-phosphate synthase
TGME49_224980	-1.23	6.80	0.00162714	0.01646863	hypothetical protein
TGME49_235610	-1.70	5.16	0.0016293	0.01646863	ATPase, AAA family protein
TGME49_294790	0.91	8.88	0.00163592	0.01651299	hypothetical protein
TGME49_248610	1.33	5.56	0.00163968	0.01652846	hypothetical protein
TGME49_292610	1.36	5.59	0.00164799	0.01658962	Toxoplasma gondii family C protein
TGME49_253310	1.22	5.37	0.00166864	0.01677471	hypothetical protein
TGME49_275870	1.41	5.25	0.00169095	0.01697595	tubulin/FtsZ family, GTPase domain-containing protein
TGME49_292950	1.10	7.04	0.00170228	0.01704351	hypothetical protein
TGME49_315100	2.60	4.60	0.00170643	0.01706199	hypothetical protein
TGME49_200360	1.25	9.81	0.00172505	0.01722174	hypothetical protein
TGME49_207900	-1.23	6.22	0.00172706	0.01722174	transcription initiation factor TFIIIB
TGME49_314695	-1.50	6.57	0.0017351	0.01727869	hypothetical protein
TGME49_230705	1.26	6.59	0.00173754	0.01727979	hypothetical protein
TGME49_297510	-1.70	5.94	0.00174138	0.01729396	hypothetical protein
TGME49_228720	-1.65	5.08	0.00175019	0.01733578	hypothetical protein
TGME49_226100	-2.58	5.42	0.00175709	0.01738084	haloacid dehalogenase family hydrolase domain-containing
TGME49_270160	-1.39	5.77	0.00176194	0.0174056	hypothetical protein
TGME49_278830	-0.99	8.64	0.00177988	0.01755946	glucose-6-phosphate 1-dehydrogenase
TGME49_249550	-1.45	5.67	0.00178513	0.01758779	hypothetical protein
TGME49_271030	1.84	6.63	0.00182048	0.01786483	AP2 domain transcription factor AP2VIII-6
TGME49_246050	-2.03	5.13	0.00182376	0.01787335	hypothetical protein
TGME49_314700	1.42	6.55	0.00183723	0.01795781	hypothetical protein
TGME49_254800	1.27	5.96	0.0018369	0.01795781	hypothetical protein
TGME49_207065	1.19	5.52	0.00185127	0.01804748	hypothetical protein
TGME49_208740	1.29	9.78	0.00186231	0.0181312	microneme protein, putative
TGME49_253820	0.94	8.35	0.00186765	0.01815939	hypothetical protein
TGME49_267660	-1.04	6.87	0.00188778	0.01830701	hypothetical protein
TGME49_267750	-1.15	6.69	0.0019001	0.01840238	hypothetical protein
TGME49_224310	-1.28	6.08	0.00190472	0.01842307	DHHC zinc finger domain-containing protein
TGME49_223910	-1.14	7.08	0.00191217	0.01844697	acyltransferase domain-containing protein
TGME49_258980	-1.74	5.28	0.0019158	0.01845798	hypothetical protein
TGME49_309010	-1.67	4.97	0.00193965	0.01863924	elongation factor P, putative
TGME49_238170	1.47	5.73	0.00196147	0.0188002	hypothetical protein
TGME49_275450	0.85	7.53	0.00196078	0.0188002	hypothetical protein
TGME49_253650	0.84	8.06	0.00198352	0.01896245	DnaJ C terminal region domain-containing protein
TGME49_315250	1.10	5.99	0.00199665	0.0190531	GAMM1 protein, putative
TGME49_221230	-1.20	6.64	0.00199815	0.0190531	hypothetical protein
TGME49_304670	-1.33	6.35	0.00200227	0.01906791	leucine rich repeat-containing protein
TGME49_284660	1.30	5.36	0.0020125	0.01909169	mitochondrial ribosomal protein s6-2, putative
TGME49_249380	-1.03	6.91	0.00200743	0.01909169	DHHC zinc finger domain-containing protein
TGME49_311905	-2.42	4.73	0.00201007	0.01909169	hypothetical protein
TGME49_263840	-1.19	6.12	0.00202438	0.01915536	hypothetical protein
TGME49_263785	-2.30	4.86	0.00202384	0.01915536	phosphatidate cytidylyltransferase
TGME49_272030	-1.20	6.10	0.002036	0.01919176	kelch repeat-containing protein
TGME49_268690	1.67	4.84	0.00208062	0.01951306	hypothetical protein
TGME49_244870	-1.26	5.84	0.00208811	0.0195585	quinone oxidoreductase, putative
TGME49_214320	1.13	9.30	0.00209948	0.01964026	facilitative glucose transporter GT1
TGME49_229140	1.35	6.54	0.00211017	0.01971532	MaoC family domain-containing protein
TGME49_255240	-1.71	5.60	0.0021158	0.01974306	hypothetical protein
TGME49_247690	-1.07	7.31	0.00213482	0.01989545	phospholipid-translocating P-type ATPase, flippase subfamily

TGME49_214750	-1.02	6.75	0.00215339	0.01994802	hypothetical protein
TGME49_263510	-1.86	5.94	0.00214682	0.01994802	Spc97 / Spc98 family protein
TGME49_308050	-1.10	6.70	0.00215877	0.01996799	thioredoxin domain-containing protein
TGME49_321170	1.39	4.77	0.00217103	0.02000644	Toxoplasma gondii family C protein
TGME49_222245	-1.08	6.95	0.00216899	0.02000644	hypothetical protein
TGME49_258070	-1.18	6.52	0.00219516	0.02017857	hypothetical protein
TGME49_289140	0.96	6.35	0.00220571	0.0202504	ribosomal protein l22/l43, putative
TGME49_247460	0.85	8.25	0.00222221	0.02035148	proliferating cell nuclear antigen PCNA1
TGME49_251530	2.11	4.57	0.0022663	0.02070408	hypothetical protein
TGME49_226270	1.09	5.99	0.00226603	0.02070408	hypothetical protein
TGME49_204350	2.00	5.81	0.00227743	0.02078011	hypothetical protein
TGME49_228070	1.56	4.89	0.00228269	0.02080255	hypothetical protein
TGME49_280375	2.33	5.70	0.00233779	0.02127848	hypothetical protein
TGME49_203080	1.05	6.45	0.00236947	0.02154035	RNA recognition motif-containing protein
TGME49_301890	-1.68	6.19	0.0023948	0.02171733	Toxoplasma gondii family B protein
TGME49_235540	1.00	8.28	0.00242332	0.02192997	eukaryotic initiation factor-2 beta, putative
TGME49_306260	1.02	6.14	0.00243955	0.02202621	hypothetical protein
TGME49_299180	-1.97	5.70	0.00244372	0.02202621	prenylated protein, putative
TGME49_294310	1.42	4.82	0.0024595	0.02214151	hypothetical protein
TGME49_261670	1.33	5.19	0.00247502	0.02222932	ribonuclease H1/H2 small subunit protein
TGME49_208830	1.17	8.09	0.00248697	0.02230742	hypothetical protein
TGME49_245540	-1.08	6.98	0.0025046	0.02243842	hypothetical protein
TGME49_216880	3.06	12.29	0.00255082	0.02278298	guanine nucleotide-binding protein
TGME49_253615	1.30	5.78	0.00255111	0.02278298	hypothetical protein
TGME49_295070	1.17	5.71	0.00255681	0.02278298	helicase associated domain (ha2) protein
TGME49_228110	-1.31	6.14	0.00255843	0.02278298	hypothetical protein
TGME49_285490	-1.46	5.79	0.00255613	0.02278298	helix-hairpin-helix motif domain-containing protein
TGME49_276930	0.90	8.13	0.0025666	0.02282335	hypothetical protein
TGME49_214780	-1.54	5.82	0.00256913	0.02282335	hydrolase, NUDIX family protein
TGME49_224480	-1.12	6.40	0.00258737	0.02295789	cell-cycle-associated protein kinase CLK, putative
TGME49_202280	-1.90	5.61	0.00260292	0.02306827	WD domain, G-beta repeat-containing protein
TGME49_210255	0.92	6.96	0.00262003	0.02319214	hypothetical protein
TGME49_265410	1.53	5.34	0.00262775	0.02323269	G-protein beta WD-40 repeat containing protein
TGME49_245500	1.22	5.63	0.00263293	0.02325078	dipeptidyl peptidase iv (dpp iv) n-terminal region domain-
TGME49_245480	2.22	5.06	0.00264901	0.0233649	hypothetical protein
TGME49_202980	-1.12	6.64	0.00267091	0.02353002	hypothetical protein
TGME49_209210	-1.10	6.93	0.00267911	0.02357424	hypothetical protein
TGME49_319710	-1.31	6.20	0.00268688	0.02361461	kinesin motor domain-containing protein
TGME49_220160	2.33	4.59	0.00272524	0.02387523	WD domain-containing protein
TGME49_263080	0.90	7.13	0.0027262	0.02387523	hypothetical protein
TGME49_306470	-1.14	6.37	0.00274211	0.02398614	isoprenylcysteine carboxyl methyltransferase (icmt) family
TGME49_313277	-2.03	4.54	0.0027478	0.02400765	hypothetical protein
TGME49_285540	-1.59	4.92	0.00278664	0.02431824	DNA-directed DNA polymerase
TGME49_201220	-1.50	5.67	0.00279131	0.02433034	zinc finger protein
TGME49_293190	-1.01	7.22	0.00279837	0.02436324	endonuclease/exonuclease/phosphatase family protein
TGME49_319500	-1.04	7.26	0.00281846	0.02450936	hypothetical protein
TGME49_238110	-1.50	6.11	0.00283115	0.02453564	replication factor a protein 3 protein
TGME49_246760	-2.21	4.72	0.00283104	0.02453564	hypothetical protein
TGME49_313600	-1.33	6.04	0.00286526	0.02477092	DDHD domain-containing protein
TGME49_265520	-0.89	7.43	0.00288969	0.02495307	hypothetical protein
TGME49_203350	1.48	5.16	0.00291196	0.02508687	hypothetical protein
TGME49_215060	-1.03	7.21	0.00290964	0.02508687	small GTP-binding protein sar1, putative
TGME49_211860	1.10	6.12	0.00296059	0.02537743	hypothetical protein
TGME49_312940	0.97	8.38	0.00296091	0.02537743	hypothetical protein
TGME49_246970	-1.45	5.30	0.00295268	0.02537743	3'-5' exonuclease domain-containing protein
TGME49_320000	-1.41	5.47	0.00299175	0.02556616	SCY kinase (incomplete catalytic triad)
TGME49_294930	-1.08	7.12	0.00301822	0.02575003	leucine rich repeat-containing protein

TGME49_219720	1.24	6.23	0.00303944	0.02588418	Ras-related protein Rab-5C, putative
TGME49_254460	1.01	7.06	0.0030588	0.0260191	hypothetical protein
TGME49_266070	1.33	10.10	0.00307714	0.02611669	ribosomal protein RPL31
TGME49_305140	1.08	6.47	0.00307899	0.02611669	phospholipase, patatin family protein
TGME49_293790	1.08	5.45	0.00308085	0.02611669	hypothetical protein
TGME49_257595	-1.23	5.98	0.00309458	0.02620314	hypothetical protein
TGME49_254120	0.96	8.64	0.00312777	0.0264206	autophagy-related protein 8 atg8, putative
TGME49_221390	-1.24	6.10	0.00313096	0.0264206	hypothetical protein
TGME49_293220	1.44	5.11	0.00317283	0.02674343	DHHC zinc finger domain-containing protein
TGME49_311890	-0.94	7.52	0.00323902	0.0272419	hypothetical protein
TGME49_278740	-1.25	5.53	0.00323932	0.0272419	diaminopimelate decarboxylase
TGME49_253880	0.81	7.79	0.00324602	0.02726731	GNS1/SUR4 family protein
TGME49_213910	-1.27	5.99	0.00326551	0.02736896	hypothetical protein
TGME49_304680	-1.43	5.66	0.00326373	0.02736896	ubiquitin family protein
TGME49_275810	1.45	10.26	0.0032805	0.02741037	ribosomal protein RPS10
TGME49_221510	-1.15	8.07	0.00327742	0.02741037	hypothetical protein
TGME49_228370	-1.26	5.97	0.00328525	0.02741037	hypothetical protein
TGME49_315590	-1.38	5.72	0.00328197	0.02741037	macro domain-containing protein
TGME49_263290	1.46	5.21	0.00329622	0.02747094	rhomboid protease ROM2
TGME49_290270	-1.20	6.19	0.00330298	0.0274964	SPRY domain-containing protein
TGME49_202240	-2.36	4.63	0.00330818	0.02750879	RAP domain-containing protein
TGME49_248370	1.41	5.44	0.00333287	0.02768302	prefoldin subunit 6, putative
TGME49_290720	-0.83	7.77	0.00333803	0.02769484	vacuolar proton translocating ATPase subunit, putative
TGME49_263660	-2.33	4.59	0.0033557	0.02781031	hypothetical protein
TGME49_213710	-1.28	6.17	0.00338044	0.02798397	WD domain, G-beta repeat-containing protein
TGME49_283585	-1.30	5.67	0.00342378	0.02831113	hypothetical protein
TGME49_237480	-1.55	5.26	0.00343441	0.02836739	BRCA1 C Terminus (BRCT) domain-containing protein
TGME49_220530	-1.64	5.46	0.00346311	0.02850911	AP2 domain transcription factor AP2V-1
TGME49_263720	-1.87	5.02	0.00347647	0.02858735	HMG (high mobility group) box domain-containing protein
TGME49_249240	1.13	9.83	0.00349874	0.02870671	calmodulin, putative
TGME49_225680	-1.74	5.41	0.00349521	0.02870671	hypothetical protein
TGME49_201400	-1.41	5.98	0.00351383	0.02876679	Sin3-associated polypeptide SAP18
TGME49_306410	-1.76	5.32	0.00352326	0.02881217	hypothetical protein
TGME49_249480	1.07	5.88	0.00353917	0.02891037	tetratricopeptide repeat-containing protein
TGME49_216260	-0.98	7.05	0.0035732	0.02914173	eukaryotic initiation factor-2B, gamma subunit, putative
TGME49_289180	-1.21	5.71	0.00358233	0.0291608	thioredoxin family redox-active protein, putative
TGME49_252640	1.57	4.57	0.003606	0.02923072	P-type ATPase PMA1
TGME49_290950	-0.86	7.68	0.00360004	0.02923072	clathrin heavy chain, putative
TGME49_262700	-1.61	5.53	0.00360512	0.02923072	tetratricopeptide repeat-containing protein
TGME49_269075	1.68	4.72	0.00363291	0.02941663	hypothetical protein
TGME49_253380	0.83	8.39	0.0036537	0.02952049	AP2 domain transcription factor AP2III-2
TGME49_258105	-2.15	4.46	0.00366285	0.02956213	hypothetical protein
TGME49_237230	1.11	9.68	0.00367339	0.0296003	hypothetical protein
TGME49_202300	-1.29	5.80	0.00367557	0.0296003	inosine triphosphate pyrophosphatase, putative
TGME49_215990	-1.57	5.28	0.00370626	0.02981504	helicase, putative
TGME49_314840	-1.19	6.09	0.00371979	0.02989146	ubiquitin carboxyl-terminal hydrolase
TGME49_253440	0.81	8.62	0.00375668	0.03013232	cell-cycle-associated protein kinase SRPK, putative
TGME49_213950	-1.11	6.02	0.00376325	0.0301426	hypothetical protein
TGME49_308040	-1.22	5.84	0.00376848	0.0301519	ZPR1 zinc finger domain-containing protein
TGME49_207970	-3.06	4.61	0.00379992	0.03033791	HEAT repeat-containing protein
TGME49_208850	1.56	5.42	0.00384551	0.03048944	SAG-related sequence SRS11
TGME49_210408	1.42	10.56	0.00383279	0.03048944	HMG (high mobility group) box domain-containing protein
TGME49_254820	0.97	7.23	0.00383891	0.03048944	hypothetical protein
TGME49_293680	-1.30	6.69	0.0038477	0.03048944	hypothetical protein
TGME49_291690	-1.33	5.50	0.00384417	0.03048944	hypothetical protein
TGME49_260830	-2.20	5.12	0.00383052	0.03048944	hypothetical protein
TGME49_295420	-0.94	7.67	0.00386814	0.03061866	hypothetical protein

TGME49_226320	-1.05	6.35	0.00387296	0.03062405	hypothetical protein
TGME49_245730	2.35	4.70	0.00391347	0.03087848	phosphatidylinositol-4-phosphate 5-Kinase
TGME49_262730	0.88	8.05	0.00391304	0.03087848	rhoptry protein ROP16
TGME49_312210	1.76	4.65	0.0039584	0.03119975	hypothetical protein
TGME49_240600	0.97	7.27	0.00404162	0.03178807	chaperonin cpn60, putative
TGME49_301460	1.63	4.68	0.00405212	0.03183682	hypothetical protein
TGME49_297650	-1.02	8.05	0.00406762	0.0318744	Ser/Thr phosphatase family protein
TGME49_259720	-1.19	6.13	0.0040698	0.0318744	hypothetical protein
TGME49_305860	-0.87	7.96	0.00407955	0.03191695	calcium-dependent protein kinase CDPK3
TGME49_208580	-1.19	6.18	0.00412095	0.03220684	DNA ligase 1, putative
TGME49_291350	-1.66	5.13	0.00413899	0.03231379	hypothetical protein
TGME49_226080	-1.60	5.21	0.00416693	0.03249532	polyA polymerase
TGME49_215250	-1.94	5.14	0.0042011	0.0326953	thiamin pyrophosphokinase, catalytic domain-containing protein
TGME49_319860	-1.01	6.84	0.00421515	0.03273593	DNA polymerase family B protein
TGME49_216750	-1.10	6.58	0.00421486	0.03273593	Paf1/RNA polymerase II complex component LEO1
TGME49_202510	1.65	5.53	0.00423183	0.03279744	multi-pass transmembrane protein
TGME49_261720	-0.98	7.53	0.00425096	0.03284191	metal cation transporter, ZIP family protein
TGME49_262860	-1.51	5.63	0.00424704	0.03284191	ADP-ribosylation factor family protein 1, putative
TGME49_207990	0.97	6.24	0.00425883	0.03286844	ribosomal RNA large subunit methyltransferase J protein
TGME49_320650	-1.52	4.91	0.00427597	0.03293206	ankyrin repeat-containing protein
TGME49_221160	1.68	5.19	0.00428297	0.03295174	acetyltransferase, GNAT family protein
TGME49_309090	-1.20	5.89	0.00431084	0.03313175	hypothetical protein
TGME49_237550	1.76	5.18	0.00432132	0.03314026	hypothetical protein
TGME49_244680	1.39	4.89	0.00432537	0.03314026	hypothetical protein
TGME49_231870	-1.74	5.00	0.00432282	0.03314026	tetratricopeptide repeat-containing protein
TGME49_308020	-0.87	8.65	0.00433427	0.03315037	SAG-related sequence SRS57
TGME49_251470	-1.01	7.05	0.00433563	0.03315037	hypothetical protein
TGME49_253960	1.10	5.35	0.00434544	0.03315694	oxidoreductase, short chain dehydrogenase/reductase family
TGME49_232750	0.95	6.52	0.00434431	0.03315694	23S rRNA (adenine(1618)-N(6))-methyltransferase, putative
TGME49_254000	0.92	7.14	0.00437175	0.03332338	hypothetical protein
TGME49_237190	1.43	5.53	0.00438821	0.0333459	hypothetical protein
TGME49_269730	-0.92	7.62	0.00438473	0.0333459	myosin-light-chain kinase
TGME49_202430	-1.36	5.89	0.00439497	0.03336302	hypothetical protein
TGME49_225080	1.55	10.82	0.00443559	0.0336369	ribosomal protein RPS18
TGME49_231200	1.47	5.30	0.00444267	0.03365616	hypothetical protein
TGME49_262690	2.92	12.26	0.00446774	0.03381154	ribosomal protein RPL27
TGME49_272910	0.85	7.65	0.00450539	0.03406163	T-complex protein 1 delta subunit
TGME49_311625	-1.37	5.89	0.00451377	0.03409024	WD domain, G-beta repeat-containing protein
TGME49_231180	-2.31	5.84	0.00452938	0.03417327	hypothetical protein
TGME49_259570	-1.51	5.08	0.00456137	0.03437961	CMP/dCMP deaminase, zinc-binding, putative
TGME49_212310	0.98	9.06	0.00457683	0.03446109	vacuolar ATP synthetase
TGME49_237280	-0.92	7.18	0.00458256	0.03446922	TLD protein
TGME49_276220	0.97	6.04	0.00460593	0.03460987	hypothetical protein
TGME49_268680	0.90	6.98	0.00469509	0.03524408	hypothetical protein
TGME49_275420	-1.14	6.14	0.0047073	0.0353	histone lysine-specific demethylase LSD1/BHC110/KDMA1A
TGME49_235140	-0.88	7.38	0.00471836	0.03534715	hypothetical protein
TGME49_219750	0.84	8.70	0.00474225	0.03543795	cytochrome c, putative
TGME49_227830	-1.02	6.68	0.00474483	0.03543795	mitochondrial inner membrane translocase TIM44, putative
TGME49_318650	0.85	8.08	0.00475372	0.03546859	transhydrogenase
TGME49_291620	-0.92	6.76	0.00477651	0.03556698	hypothetical protein
TGME49_227570	0.90	6.79	0.00480145	0.03569856	transmembrane amino acid transporter protein
TGME49_316660	-1.10	6.57	0.00482512	0.03578513	cullin family protein
TGME49_207665	-2.56	4.58	0.00482377	0.03578513	kinesin motor domain-containing protein
TGME49_207120	-1.46	5.34	0.0048485	0.0359225	Sad1/UNC family protein
TGME49_273595	1.50	5.44	0.00491084	0.03631182	hypothetical protein
TGME49_210360	1.17	5.56	0.0049735	0.03670188	DEAD (Asp-Glu-Ala-Asp) box polypeptide 41 family protein
TGME49_311160	-0.88	7.00	0.00497017	0.03670188	PWI domain-containing protein

TGME49_286580	0.92	7.96	0.00498245	0.03673134	hypothetical protein
TGME49_280770	1.02	6.58	0.00501624	0.03692538	regulator of chromosome condensation (RCC1) repeat-containing
TGME49_217820	-0.96	7.80	0.00502373	0.03692538	PCI domain-containing protein
TGME49_226020	0.95	8.77	0.00502921	0.03692906	transporter, major facilitator family protein
TGME49_216230	-0.92	6.77	0.0050387	0.03696209	hypothetical protein
TGME49_253410	0.98	6.01	0.00505681	0.03701443	hypothetical protein
TGME49_255660	-1.12	6.20	0.00506082	0.03701443	EF hand domain-containing protein
TGME49_270120	-1.15	6.25	0.00505196	0.03701443	thioredoxin-like protein TLP1
TGME49_205130	-0.83	7.87	0.00507881	0.03710937	hypothetical protein
TGME49_249410	1.07	5.80	0.00509453	0.03715099	hypothetical protein
TGME49_209090	-1.16	6.23	0.00513049	0.03737641	proteasome maturation factor ump1 protein
TGME49_277760	-0.91	7.01	0.00515085	0.0374879	adenylosuccinate lyase, putative
TGME49_211630	-0.80	8.48	0.00516997	0.03759012	hypothetical protein
TGME49_310950	-0.80	7.88	0.00521663	0.0378922	AP2 domain transcription factor AP2XI-3
TGME49_225240	0.91	6.65	0.00524457	0.03805783	50S ribosomal protein L13, putative
TGME49_213010	-1.19	7.01	0.00525518	0.03809746	hypothetical protein
TGME49_204060	-1.35	5.49	0.00526991	0.03812967	SNARE domain-containing protein
TGME49_227780	1.46	5.00	0.00529441	0.03826956	hypothetical protein
TGME49_238230	0.96	5.98	0.00530521	0.03831024	Ser/Thr phosphatase family protein
TGME49_211030	-0.85	8.08	0.0053339	0.03847989	hypothetical protein
TGME49_228330	-1.61	5.62	0.00535503	0.03859479	NLI interacting factor family phosphatase
TGME49_310802	-1.21	5.73	0.0053644	0.03862471	CRAL/TRIO domain-containing protein
TGME49_219590	-1.13	6.71	0.00543186	0.0389799	RuvB family 1 protein
TGME49_319600	-1.15	5.74	0.00543276	0.0389799	alpha-tubulin N-acetyltransferase, putative
TGME49_280410	-0.91	6.63	0.00546897	0.03912811	3'5'-cyclic nucleotide phosphodiesterase domain-containing
TGME49_301170	-6.07	5.07	0.00547128	0.03912811	SAG-related sequence SRS19D
TGME49_235560	1.73	5.20	0.00549438	0.03925542	hypothetical protein
TGME49_280750	-0.98	6.42	0.00551256	0.03934736	rudimentary enhancer, putative
TGME49_254380	-1.33	5.32	0.00551878	0.03935383	ribosomal protein L11, putative
TGME49_252280	1.08	5.98	0.00552617	0.03936864	hypothetical protein
TGME49_230850	-0.86	7.40	0.00553609	0.03940143	hypothetical protein
TGME49_248690	-1.36	5.60	0.00555775	0.03947977	hypothetical protein
TGME49_278205	-0.80	7.72	0.00558797	0.0396048	hypothetical protein
TGME49_215640	-0.99	6.80	0.00559139	0.0396048	zinc finger, C3HC4 type (RING finger) domain-containing protein
TGME49_202390	-0.93	6.90	0.00564633	0.03995576	S15 sporozoite-expressed protein
TGME49_257685	0.90	7.00	0.00566822	0.04002038	hypothetical protein
TGME49_228010	-1.60	5.14	0.00566406	0.04002038	hypothetical protein
TGME49_278060	-1.66	5.81	0.00567707	0.04002038	Mre11 DNA-binding domain-containing protein
TGME49_313200	-1.67	5.25	0.00575654	0.04054204	leucine rich repeat-containing protein
TGME49_257300	-0.97	7.02	0.00577241	0.04060588	hypothetical protein
TGME49_219190	-1.06	6.13	0.00577657	0.04060588	nuclear movement protein domain containing protein
TGME49_246178	-1.17	6.54	0.00585277	0.04102479	hypothetical protein
TGME49_248870	-0.98	6.24	0.00587648	0.041152	SNARE associated Golgi protein
TGME49_213930	-1.48	5.46	0.00589416	0.04123683	3' exoribonuclease family, domain 1 domain-containing protein
TGME49_287515	-1.69	4.85	0.00590333	0.04126206	hypothetical protein
TGME49_297720	-1.02	7.42	0.0059096	0.04126695	trehalose-phosphatase
TGME49_251730	-1.16	5.85	0.00592861	0.04135002	hypothetical protein
TGME49_205200	0.92	6.26	0.00594215	0.04137519	hypothetical protein
TGME49_213388	-1.46	5.26	0.00595302	0.04137519	hypothetical protein
TGME49_254480	1.45	5.50	0.00602032	0.04173191	WD domain, G-beta repeat-containing protein
TGME49_238180	-0.95	7.28	0.00604521	0.04181978	26s proteasome regulatory complex subunit, putative
TGME49_220470	-1.04	6.96	0.00605177	0.04182611	hypothetical protein
TGME49_218740	1.54	5.51	0.00608702	0.04199139	membrane protein, putative
TGME49_222110	-1.24	6.57	0.00611209	0.04212511	PUB domain-containing protein
TGME49_236250	-1.28	5.31	0.00612661	0.04216101	regulator of chromosome condensation (RCC1) repeat-containing protein
TGME49_310230	-1.14	6.60	0.00615478	0.04230128	hypothetical protein
TGME49_256060	-1.00	6.61	0.00619041	0.04250674	nucleosome assembly protein (nap) protein

TGME49_205250	0.96	9.50	0.00626427	0.04297404	rhoptry protein ROP18
TGME49_240570	1.00	6.81	0.00632295	0.04333648	hypothetical protein
TGME49_312160	0.89	7.86	0.00633698	0.04339253	hypothetical protein
TGME49_315280	-1.30	5.80	0.00638054	0.04365043	hypothetical protein
TGME49_256760	-0.93	8.64	0.00638833	0.04366342	pyruvate kinase PyK1
TGME49_217400	1.51	6.47	0.00639882	0.04369481	hypothetical protein
TGME49_254470	0.87	9.17	0.00641415	0.04371889	hypothetical protein
TGME49_217770	0.82	6.54	0.00641184	0.04371889	hypothetical protein
TGME49_252210	1.25	10.38	0.00644226	0.04384183	pentatricopeptide repeat domain-containing protein
TGME49_306610	-1.56	4.93	0.00644402	0.04384183	3' exoribonuclease family, domain 1 domain-containing protein
TGME49_308093	0.79	8.24	0.00646022	0.04391174	rhoptry kinase family protein (incomplete catalytic triad)
TGME49_261460	-1.01	7.09	0.00649329	0.04393498	transcriptional elongation factor FACT80
TGME49_240910	-1.09	6.41	0.00649062	0.04393498	hypothetical protein
TGME49_294180	-1.26	5.80	0.00648307	0.04393498	tRNA -methyltransferase family protein
TGME49_234900	-0.85	7.30	0.00651263	0.0439855	PHD-finger domain-containing protein
TGME49_214470	-1.19	5.86	0.00650985	0.0439855	Ulp1 protease family, C-terminal catalytic domain-containing
TGME49_224070	-1.28	5.80	0.0065297	0.04406061	hypothetical protein
TGME49_221670	-0.81	7.54	0.00655712	0.04416518	transcriptional elongation factor FACT140
TGME49_242010	1.26	5.35	0.00659851	0.04440361	glucose inhibited division protein A subfamily protein
TGME49_254090	1.09	6.12	0.00663691	0.04462146	hypothetical protein
TGME49_216910	-1.01	6.32	0.00666042	0.04473893	hypothetical protein
TGME49_269438	1.09	6.27	0.00671232	0.04504672	hypothetical protein
TGME49_279430	1.00	6.42	0.0068092	0.04555988	cwf18 pre-mRNA splicing factor protein
TGME49_275650	1.13	7.60	0.00688341	0.04590387	hypothetical protein
TGME49_225170	-1.22	5.59	0.00696876	0.04643124	hypothetical protein
TGME49_286790	-1.12	6.11	0.00700071	0.04651861	nuclear factor NF2
TGME49_295960	-1.27	6.45	0.00699541	0.04651861	hypothetical protein
TGME49_233430	1.41	4.98	0.00701699	0.04652501	hypothetical protein
TGME49_253070	1.36	4.92	0.00702051	0.04652501	hydrolase, TatD family protein
TGME49_249900	1.17	10.08	0.00701439	0.04652501	adenine nucleotide translocator, putative
TGME49_273140	-1.37	5.60	0.00703216	0.04656058	radical SAM methylthiotransferase, MiaB/RimO family protein
TGME49_231480	0.94	6.53	0.00710621	0.04700887	GCN1, putative
TGME49_321650	-0.82	7.42	0.00714291	0.04720409	hypothetical protein
TGME49_319740	-1.41	5.14	0.00714847	0.04720409	transporter, major facilitator family protein
TGME49_201880	0.73	8.04	0.00717594	0.04734334	hypothetical protein
TGME49_204040	1.61	4.96	0.00720955	0.04752273	hypothetical protein
TGME49_266750	0.85	7.27	0.00728625	0.04790049	transporter/permease protein, putative
TGME49_227150	0.87	7.65	0.00739765	0.04846634	glutaredoxin, putative
TGME49_291120	-1.11	5.77	0.00739849	0.04846634	trafficking protein mon1 subfamily protein
TGME49_285950	-1.50	5.04	0.00750799	0.04909684	hypothetical protein
TGME49_239680	0.97	6.25	0.00752842	0.04918703	hypothetical protein
TGME49_213790	-1.09	6.02	0.0075429	0.04923821	hypothetical protein
TGME49_318690	1.29	4.49	0.00756471	0.04933711	RNA recognition motif-containing protein
TGME49_254290	1.08	5.58	0.00762164	0.04966466	hypothetical protein

Supplementary Table S5B: Differencial expression analysis of T. gondii genes from MM6 cells infected with T. gondii EGS strain for 18 hours copmared to T. gondii gene expression from MM6 cells infected with ME49 strain for 18 hours.

gene ID	logFC (EGS/ME49)	logCPM	PValue	FDR	Product Name
TGME49_232955	8.68	8.96	2.61E-67	1.94E-63	hypothetical protein
TGME49_322200	8.34	6.25	2.98E-58	1.10E-54	apocytochrome b, putative
TGME49_323400	8.28	9.09	9.62E-52	2.38E-48	cytochrome c oxidase subunit iii subfamily protein
TGME49_280570	5.19	7.23	1.69E-38	3.13E-35	SAG-related sequence SRS35A
TGME49_259115	-5.11	7.48	3.58E-37	5.31E-34	ABC1 family protein
TGME49_293790	5.43	5.45	6.30E-32	7.79E-29	hypothetical protein
TGME49_330000	9.02	9.81	6.60E-31	6.11E-28	cytochrome b
TGME49_291040	5.96	6.32	6.45E-31	6.11E-28	lactate dehydrogenase LDH2
TGME49_212940	-10.97	6.33	6.99E-30	5.75E-27	hypothetical protein
TGME49_255060	7.47	9.39	2.43E-27	1.80E-24	cytochrome b(N-terminal)/b6/petB subfamily protein
TGME49_237130	8.26	9.77	1.06E-26	7.12E-24	cytochrome b, putative
TGME49_267020	-4.42	7.23	2.86E-25	1.76E-22	hypothetical protein
TGME49_259020	5.33	6.32	5.88E-24	3.11E-21	bradyzoite antigen BAG1
TGME49_216140	3.66	7.04	5.67E-24	3.11E-21	tetratricopeptide repeat-containing protein
TGME49_299010	4.46	6.15	2.26E-23	1.12E-20	hypothetical protein
TGME49_202970	-5.95	7.16	5.94E-23	2.75E-20	hypothetical protein
TGME49_252065	4.28	5.35	8.59E-23	3.74E-20	KRUF family protein
TGME49_264240	-10.43	5.73	7.44E-22	3.06E-19	hypothetical protein
TGME49_301222	2.86	6.51	2.80E-21	1.09E-18	DNA repair protein Rad4 domain-containing protein
TGME49_206550	2.98	5.45	1.91E-20	7.07E-18	hypothetical protein
TGME49_312905	-3.09	7.33	4.54E-20	1.60E-17	hypothetical protein
TGME49_244700	-2.91	7.42	9.89E-20	3.33E-17	NAD(+)/NADH kinase domain-containing protein
TGME49_302055	10.62	5.01	6.21E-19	2.00E-16	ribosomal protein RPS12
TGME49_223660	2.65	7.24	1.73E-18	5.35E-16	50S ribosomal protein L4, putative
TGME49_305460	2.65	7.39	8.61E-18	2.55E-15	methionine aminopeptidase 2, putative
TGME49_253330	3.85	7.81	1.37E-17	3.91E-15	Rhoptry kinase family protein, truncated (incomplete catalytic triad)
TGME49_252070	3.75	4.72	3.21E-16	8.49E-14	KRUF family protein
TGME49_252640	5.22	4.57	6.72E-16	1.68E-13	P-type ATPase PMA1
TGME49_279340	2.37	6.65	6.79E-16	1.68E-13	hypothetical protein
TGME49_226310	-3.64	7.53	8.93E-16	2.13E-13	zinc finger (CCCH type) motif-containing protein
TGME49_217530	3.78	4.70	1.28E-15	2.96E-13	hypothetical protein
TGME49_280400	-3.11	7.79	3.42E-15	7.67E-13	hypothetical protein
TGME49_290580	-3.63	6.26	5.02E-15	1.09E-12	ATP-binding cassette G family transporter ABCG89
TGME49_301170	-9.48	5.07	9.03E-15	1.91E-12	SAG-related sequence SRS19D
TGME49_209755	3.95	5.73	2.22E-14	4.58E-12	hypothetical protein
TGME49_251180	3.13	5.76	4.01E-14	8.03E-12	KRUF family protein
TGME49_233000	-2.52	6.91	4.92E-14	9.35E-12	KOW motif domain-containing protein
TGME49_266950	-2.61	6.64	4.81E-14	9.35E-12	protein kinase, putative
TGME49_254030	2.07	8.07	1.85E-13	3.34E-11	zinc finger CDGSH-type domain-containing protein
TGME49_234460	2.80	6.80	2.13E-13	3.76E-11	hypothetical protein
TGME49_263510	-3.66	5.94	3.51E-13	6.05E-11	Spc97 / Spc98 family protein
TGME49_213010	-2.88	7.01	3.94E-13	6.64E-11	hypothetical protein
TGME49_213280	2.31	9.22	1.38E-12	2.23E-10	SAG-related sequence SRS25
TGME49_203720	2.05	7.96	1.38E-12	2.23E-10	vitamin k epoxide reductase family protein
TGME49_235680	-2.63	6.97	1.54E-12	2.39E-10	peptidase M16 inactive domain-containing protein
TGME49_231960	-3.35	6.35	1.55E-12	2.39E-10	omega secalin, putative

TGME49_289350	-3.12	6.16	1.70E-12	2.57E-10	ATP-binding cassette G family transporter ABCG84
TGME49_275860	2.29	9.13	2.46E-12	3.64E-10	hypothetical protein
TGME49_307770	-2.38	6.88	2.85E-12	4.14E-10	fumble protein
TGME49_312600	1.81	8.45	4.28E-12	6.10E-10	heat shock protein HSP21
TGME49_207210	3.26	6.03	7.41E-12	1.04E-09	hypothetical protein
TGME49_299030	2.12	7.31	8.13E-12	1.12E-09	RNA recognition motif 2 protein
TGME49_311460	-2.95	6.39	8.29E-12	1.12E-09	hypothetical protein
TGME49_209940	-2.26	6.74	1.27E-11	1.68E-09	transporter/permease protein
TGME49_208730	1.76	8.09	1.35E-11	1.75E-09	microneme protein, putative
TGME49_225555	1.82	8.72	1.93E-11	2.47E-09	hypothetical protein
TGME49_247530	1.99	8.79	5.08E-11	6.38E-09	hypothetical protein
TGME49_239270	-2.30	7.52	7.09E-11	8.76E-09	hypothetical protein
TGME49_297780	-2.60	6.48	9.04E-11	1.10E-08	ATPase/histidine kinase/DNA gyrase B/HSP90 domain-containing protein
TGME49_297495	-9.14	4.96	1.04E-10	1.24E-08	hypothetical protein
TGME49_283540	1.98	8.44	1.17E-10	1.35E-08	hypothetical protein
TGME49_277270	3.04	9.46	1.52E-10	1.73E-08	NTPase II
TGME49_210210	-9.04	4.47	1.81E-10	2.03E-08	hypothetical protein
TGME49_213255	-2.66	5.87	1.88E-10	2.08E-08	hypothetical protein
TGME49_301250	8.53	14.41	2.03E-10	2.21E-08	hypothetical protein
TGME49_264260	-4.82	5.63	2.16E-10	2.32E-08	hypothetical protein
TGME49_315100	5.72	4.60	2.38E-10	2.52E-08	hypothetical protein
TGME49_321360	1.69	7.60	2.52E-10	2.63E-08	clustered-asparagine-rich protein
TGME49_280490	-1.99	7.44	4.45E-10	4.58E-08	U-box domain-containing protein
TGME49_299080	1.84	6.87	4.77E-10	4.84E-08	VTC domain-containing protein
TGME49_258720	1.93	6.22	5.33E-10	5.34E-08	Ubiquitin family protein, putative
TGME49_279350	2.79	5.67	5.92E-10	5.77E-08	hypothetical protein
TGME49_213067	1.98	7.89	5.99E-10	5.77E-08	hypothetical protein
TGME49_295950	2.39	5.58	6.25E-10	5.93E-08	KRUF family protein
TGME49_233925	10.32	11.32	7.49E-10	6.85E-08	hypothetical protein
TGME49_221840	3.66	4.58	7.45E-10	6.85E-08	hypothetical protein
TGME49_301890	-3.19	6.19	7.31E-10	6.85E-08	Toxoplasma gondii family B protein
TGME49_220240	2.02	8.76	8.56E-10	7.74E-08	hypothetical protein
TGME49_306688	-3.91	4.99	1.12E-09	9.74E-08	hypothetical protein
TGME49_238140	-2.18	6.40	1.15E-09	9.92E-08	hypothetical protein
TGME49_263820	-2.10	6.58	1.28E-09	1.09E-07	DEAD/DEAH box helicase domain-containing protein
TGME49_226380	1.80	8.18	1.42E-09	1.19E-07	hypothetical protein
TGME49_240220	-2.27	6.67	1.87E-09	1.56E-07	hypothetical protein
TGME49_216680	-2.19	7.25	2.37E-09	1.95E-07	ankyrin repeat-containing protein
TGME49_240060	2.18	8.72	2.40E-09	1.96E-07	hypothetical protein
TGME49_258790	-2.03	7.15	3.28E-09	2.64E-07	zinc finger, C3HC4 type (RING finger) domain-containing protein
TGME49_214410	1.67	7.48	3.34E-09	2.66E-07	hypothetical protein
TGME49_253690	1.66	8.81	3.96E-09	3.12E-07	hypothetical protein
TGME49_287470	2.95	4.97	4.41E-09	3.41E-07	hypothetical protein
TGME49_268225	-9.99	6.07	4.47E-09	3.41E-07	hypothetical protein
TGME49_233760	-2.58	6.40	4.85E-09	3.67E-07	hypothetical protein
TGME49_312140	-4.67	8.68	4.95E-09	3.71E-07	hypothetical protein
TGME49_254770	1.66	6.49	5.22E-09	3.87E-07	Ser/Thr phosphatase family protein
TGME49_260250	-2.61	6.13	6.11E-09	4.48E-07	cyclin domain protein, cyclin H family protein
TGME49_251170	2.24	5.46	7.33E-09	5.32E-07	KRUF family protein
TGME49_306338	-2.76	5.70	1.24E-08	8.80E-07	dynein gamma chain, flagellar outer arm, putative

TGME49_228160	1.73	7.00	1.34E-08	9.37E-07	acid phosphatase
TGME49_244260	-3.96	4.83	1.56E-08	1.06E-06	hypothetical protein
TGME49_278060	-3.05	5.81	2.28E-08	1.54E-06	Mre11 DNA-binding domain-containing protein
TGME49_297840	-1.92	7.30	2.44E-08	1.62E-06	DNA primase, large subunit
TGME49_202280	-3.15	5.61	2.45E-08	1.62E-06	WD domain, G-beta repeat-containing protein
TGME49_271935	2.09	6.37	2.98E-08	1.95E-06	hypothetical protein
TGME49_204050	2.64	10.28	3.06E-08	1.99E-06	subtilisin SUB1
TGME49_254050	1.59	6.49	3.19E-08	2.05E-06	optic atrophy 3 protein (opa3) protein
TGME49_298060	-2.83	5.46	3.31E-08	2.11E-06	Toxoplasma gondii family C protein
TGME49_202020	2.74	7.60	3.42E-08	2.17E-06	DnAK-TPR
TGME49_275460	2.53	6.68	3.68E-08	2.31E-06	hypothetical protein
TGME49_200310	1.64	7.75	3.99E-08	2.48E-06	hypothetical protein
TGME49_240880	-2.58	5.67	4.05E-08	2.50E-06	hypothetical protein
TGME49_315885	5.94	9.43	4.09E-08	2.51E-06	glycosyltransferase, putative
TGME49_253600	2.31	6.44	4.19E-08	2.54E-06	hypothetical protein
TGME49_205210	-5.32	4.86	4.66E-08	2.80E-06	hypothetical protein
TGME49_253960	2.02	5.35	4.87E-08	2.91E-06	oxidoreductase, short chain dehydrogenase/reductase family protein
TGME49_227810	1.65	8.47	5.11E-08	3.01E-06	rhoptry kinase family protein ROP11 (incomplete catalytic triad)
TGME49_273870	-1.83	7.40	5.10E-08	3.01E-06	SWI2/SNF2 ISWI-like (AT hook)
TGME49_251540	1.64	9.00	5.52E-08	3.22E-06	dense granule protein GRA9
TGME49_260430	2.69	4.69	5.60E-08	3.22E-06	hypothetical protein
TGME49_322000	-2.38	6.91	5.75E-08	3.28E-06	myosin-light-chain kinase
TGME49_236670	2.12	5.44	6.01E-08	3.40E-06	hypothetical protein
TGME49_254120	1.77	8.64	6.13E-08	3.44E-06	autophagy-related protein 8 atg8, putative
TGME49_254520	1.57	7.27	6.74E-08	3.73E-06	mediator complex subunit MED11
TGME49_208440	1.46	8.08	7.24E-08	3.94E-06	hypothetical protein
TGME49_316470	-2.66	5.82	7.20E-08	3.94E-06	hypothetical protein
TGME49_221675	1.51	7.42	7.90E-08	4.27E-06	hypothetical protein
TGME49_217555	1.38	7.97	8.00E-08	4.29E-06	hypothetical protein
TGME49_217020	-2.26	6.14	8.56E-08	4.56E-06	ATPase, AFG1 family protein
TGME49_300048	1.65	6.93	9.20E-08	4.87E-06	hypothetical protein
TGME49_218750	-2.82	5.38	9.62E-08	5.05E-06	hypothetical protein
TGME49_312520	-2.75	5.63	1.05E-07	5.49E-06	tRNA dimethylallyltransferase
TGME49_253470	1.48	7.33	1.14E-07	5.91E-06	alveolin domain containing intermediate filament IMC13
TGME49_234530	1.69	7.13	1.63E-07	8.35E-06	hypothetical protein
TGME49_233870	1.82	6.13	1.75E-07	8.87E-06	hypothetical protein
TGME49_267420	1.43	8.24	1.76E-07	8.88E-06	mago nashi family protein 2, putative
TGME49_290150	-2.18	6.93	1.92E-07	9.60E-06	hypothetical protein
TGME49_316250	1.44	8.64	1.94E-07	9.67E-06	hypothetical protein
TGME49_205680	1.88	7.62	1.98E-07	9.79E-06	hypothetical protein
TGME49_215430	4.60	9.38	2.10E-07	1.03E-05	hypothetical protein
TGME49_254800	2.05	5.96	2.21E-07	1.08E-05	hypothetical protein
TGME49_275640	1.61	6.96	2.31E-07	1.11E-05	hypothetical protein
TGME49_261750	-1.87	6.89	2.30E-07	1.11E-05	rhoptry neck protein RON10
TGME49_253750	1.65	7.45	2.61E-07	1.25E-05	PLU-1 family protein
TGME49_254080	1.48	7.77	2.93E-07	1.38E-05	metal cation transporter, ZIP family protein
TGME49_208740	2.17	9.78	2.98E-07	1.40E-05	microneme protein, putative
TGME49_274070	-3.35	5.15	3.28E-07	1.53E-05	ThiF family protein
TGME49_257350	1.40	8.19	3.63E-07	1.67E-05	eukaryotic translation initiation factor, putative

TGME49_315130	1.51	6.70	3.70E-07	1.69E-05	L-isoaspartyl protein carboxyl methyltransferase family protein
TGME49_240650	-1.59	7.64	4.05E-07	1.84E-05	coatomer protein complex, subunit alpha, putative
TGME49_272410	3.54	8.19	4.08E-07	1.84E-05	phosphogluconate dehydrogenase (decarboxylating), NAD binding domain-containing protein
TGME49_319540	-2.49	5.83	4.12E-07	1.85E-05	hypothetical protein
TGME49_221320	2.37	9.10	4.42E-07	1.97E-05	acetyl-CoA carboxylase ACC1
TGME49_260480	2.05	5.50	5.22E-07	2.27E-05	leucine rich repeat-containing protein
TGME49_203730	-2.40	5.79	5.21E-07	2.27E-05	hypothetical protein
TGME49_297910	-3.38	5.31	5.23E-07	2.27E-05	hypothetical protein
TGME49_286928	-3.10	6.04	5.56E-07	2.39E-05	hypothetical protein
TGME49_236270	-3.34	5.33	5.83E-07	2.50E-05	hypothetical protein
TGME49_207960	-2.30	6.09	5.90E-07	2.51E-05	hypothetical protein
TGME49_205330	1.39	7.80	6.04E-07	2.56E-05	hypothetical protein
TGME49_262050	2.31	9.30	6.18E-07	2.60E-05	rhoptry kinase family protein ROP39
TGME49_239250	-1.47	6.95	6.25E-07	2.62E-05	diacylglycerol kinase, putative
TGME49_255290	-3.89	4.89	6.29E-07	2.62E-05	hypothetical protein
TGME49_312270	1.48	8.08	6.35E-07	2.63E-05	rhoptry protein ROP13
TGME49_268860	4.31	4.79	6.40E-07	2.63E-05	enolase 1
TGME49_252500	1.38	7.79	6.52E-07	2.67E-05	polo kinase
TGME49_300010	-4.45	5.19	7.72E-07	3.14E-05	hypothetical protein
TGME49_267460	2.48	4.67	8.98E-07	3.62E-05	AP2 domain transcription factor AP2IX-1
TGME49_320005	-2.78	5.44	9.38E-07	3.76E-05	hypothetical protein
TGME49_290300	1.44	7.98	9.56E-07	3.79E-05	hypothetical protein
TGME49_240840	-2.01	5.91	9.54E-07	3.79E-05	histone lysine demethylase JmjC NO66
TGME49_285190	-1.76	6.63	9.68E-07	3.81E-05	zinc finger, C3HC4 type (RING finger) domain-containing protein
TGME49_279450	-8.01	4.57	9.93E-07	3.89E-05	adenylosuccinate synthetase, putative
TGME49_306895	1.61	7.00	1.01E-06	3.93E-05	hypothetical protein
TGME49_202780	1.32	7.70	1.01E-06	3.93E-05	rhoptry kinase family protein ROP25
TGME49_225130	-3.12	5.10	1.07E-06	4.13E-05	hypothetical protein
TGME49_262590	-3.61	5.21	1.09E-06	4.17E-05	hypothetical protein
TGME49_244370	6.57	11.45	1.16E-06	4.41E-05	TDC1, putative
TGME49_276930	1.44	8.13	1.16E-06	4.42E-05	hypothetical protein
TGME49_260520	1.94	6.90	1.17E-06	4.43E-05	hypothetical protein
TGME49_201400	-2.11	5.98	1.24E-06	4.64E-05	Sin3-associated polypeptide SAP18
TGME49_305860	-1.42	7.96	1.26E-06	4.67E-05	calcium-dependent protein kinase CDPK3
TGME49_312330	1.73	7.92	1.27E-06	4.68E-05	hypothetical protein
TGME49_301160	-3.93	4.57	1.27E-06	4.68E-05	SAG-related sequence SRS19C
TGME49_215940	-2.34	6.61	1.29E-06	4.70E-05	Acetyl-coenzyme A transporter, putative
TGME49_293740	1.33	8.81	1.36E-06	4.92E-05	hypothetical protein
TGME49_211440	-1.86	6.23	1.38E-06	4.96E-05	hypothetical protein
TGME49_244120	-2.11	5.76	1.38E-06	4.96E-05	hypothetical protein
TGME49_226100	-3.82	5.42	1.38E-06	4.96E-05	haloacid dehalogenase family hydrolase domain-containing protein
TGME49_253290	1.29	7.51	1.45E-06	5.15E-05	valyl-tRNA synthetase
TGME49_254660	1.43	6.61	1.48E-06	5.24E-05	ankyrin repeat-containing protein
TGME49_252190	2.25	5.74	1.51E-06	5.27E-05	KRUF family protein
TGME49_288860	-1.61	6.48	1.51E-06	5.27E-05	RuvB family 2 protein
TGME49_292975	-1.81	5.91	1.56E-06	5.40E-05	hypothetical protein
TGME49_254930	1.37	7.07	1.57E-06	5.41E-05	hypothetical protein
TGME49_294400	-2.54	6.97	1.66E-06	5.70E-05	hypothetical protein

TGME49_253180	2.53	8.08	1.70E-06	5.79E-05	hypothetical protein
TGME49_215930	-7.79	4.44	1.78E-06	6.04E-05	mediator complex subunit MED21
TGME49_275430	-2.15	5.84	1.79E-06	6.05E-05	hypothetical protein
TGME49_214580	-5.07	4.76	1.80E-06	6.07E-05	tetratricopeptide repeat-containing protein
TGME49_254365	1.40	7.70	1.85E-06	6.20E-05	phosphatidate cytidylyltransferase
TGME49_211220	-1.98	5.90	1.97E-06	6.57E-05	hypothetical protein
TGME49_297492	-4.59	4.52	2.01E-06	6.68E-05	hypothetical protein
TGME49_264840	-2.86	4.93	2.08E-06	6.87E-05	ATP-dependent DNA helicase, RecQ family protein
TGME49_253100	1.26	7.21	2.26E-06	7.45E-05	hypothetical protein
TGME49_235630	1.28	6.97	2.28E-06	7.49E-05	hypothetical protein
TGME49_252430	1.30	7.44	2.41E-06	7.80E-05	hypothetical protein
TGME49_258030	-2.70	5.92	2.50E-06	8.06E-05	DNA polymerase
TGME49_299210	1.80	8.19	2.52E-06	8.09E-05	CTP synthase
TGME49_277710	-1.81	6.74	2.70E-06	8.61E-05	hypothetical protein
TGME49_254710	1.44	7.19	2.74E-06	8.70E-05	serine esterase (DUF676) protein
TGME49_277510	-1.70	6.50	3.26E-06	0.00010203	cytoplasmic dynein intermediate chain
TGME49_247410	1.28	7.58	3.38E-06	0.00010527	hypothetical protein
TGME49_293430	1.33	8.30	3.40E-06	0.00010537	hypothetical protein
TGME49_246200	-2.81	5.02	3.55E-06	0.00010953	zinc finger (CCCH type) motif-containing protein
TGME49_262730	1.38	8.05	3.58E-06	0.00011004	rhoptry protein ROP16
TGME49_259010	-1.82	6.56	3.62E-06	0.00011068	vacuolar ATP synthase subunit d, putative
TGME49_258230	2.52	5.43	3.74E-06	0.00011398	rhoptry kinase family protein ROP20
TGME49_278830	-1.44	8.64	3.80E-06	0.00011543	glucose-6-phosphate 1-dehydrogenase
TGME49_213710	-1.86	6.17	3.96E-06	0.00011953	WD domain, G-beta repeat-containing protein
TGME49_217420	-2.47	5.56	3.98E-06	0.00011953	hypothetical protein
TGME49_254720	2.04	9.40	4.29E-06	0.00012772	dense granule protein GRA8
TGME49_275870	1.90	5.25	4.59E-06	0.00013607	tubulin/FtsZ family, GTPase domain-containing protein
TGME49_316255	1.20	7.55	4.81E-06	0.00014192	hypothetical protein
TGME49_263785	-3.00	4.86	4.87E-06	0.00014322	phosphatidate cytidylyltransferase
TGME49_215700	-1.84	6.21	4.91E-06	0.00014372	phosphatidylinositol 3- and 4-kinase
TGME49_240250	-1.40	7.48	5.17E-06	0.0001496	macro domain-containing protein
TGME49_319580	-2.72	5.43	5.17E-06	0.0001496	hypothetical protein
TGME49_246978	-2.84	5.37	5.28E-06	0.00015214	hypothetical protein
TGME49_204270	-1.92	6.40	5.86E-06	0.00016831	hypothetical protein
TGME49_262780	-1.80	6.39	6.30E-06	0.00017958	FHA domain-containing protein
TGME49_219832	-2.64	5.42	6.46E-06	0.0001826	cyclin-dependent kinase regulatory subunit protein
TGME49_226755	1.60	6.33	7.15E-06	0.00019991	3'5'-cyclic nucleotide phosphodiesterase domain-containing protein
TGME49_217951	-2.00	6.01	7.13E-06	0.00019991	hypothetical protein
TGME49_298830	2.69	4.79	7.21E-06	0.00020085	hypothetical protein
TGME49_309990	1.37	6.25	7.36E-06	0.00020346	hypothetical protein
TGME49_257180	-2.08	5.94	7.34E-06	0.00020346	RecF/RecN/SMC N terminal domain-containing protein
TGME49_295935	1.37	9.03	7.39E-06	0.00020354	KRUF family protein
TGME49_222040	-2.83	5.45	7.45E-06	0.00020453	Ran-interacting Mog1 protein
TGME49_312310	-2.41	5.48	7.49E-06	0.00020476	ATPase, AAA family protein
TGME49_225290	2.97	4.81	7.85E-06	0.00021394	GDA1/CD39 (nucleoside phosphatase) family protein
TGME49_307650	-1.81	6.17	7.96E-06	0.00021589	uracil-DNA glycosylase
TGME49_263730	-1.66	6.51	8.06E-06	0.00021807	FAD-dependent glycerol-3-phosphate dehydrogenase
TGME49_223040	1.48	7.92	8.44E-06	0.00022586	hypothetical protein

TGME49_263100	-2.14	5.60	8.41E-06	0.00022586	hypothetical protein
TGME49_313100	-2.51	5.89	8.43E-06	0.00022586	signal recognition particle SRP54 protein
TGME49_290600	6.38	12.08	8.52E-06	0.00022711	succinyl-CoA-synthetase alpha SCSA
TGME49_225930	1.14	8.16	8.68E-06	0.0002304	triose-phosphate isomerase TPI-I
TGME49_248990	1.22	8.48	9.31E-06	0.00024537	hypothetical protein
TGME49_269690	1.16	7.76	9.44E-06	0.00024808	hypothetical protein
TGME49_280560	-1.50	6.83	9.51E-06	0.00024893	selenide, water dikinase
TGME49_260210	-2.82	5.25	9.98E-06	0.00025842	DnaJ domain-containing protein
TGME49_213635	2.45	5.03	1.01E-05	0.00026178	hypothetical protein
TGME49_310970	-1.43	7.06	1.03E-05	0.00026406	hypothetical protein
TGME49_233680	-2.92	4.92	1.10E-05	0.00028088	nuclear movement family protein
TGME49_278510	-1.43	8.05	1.10E-05	0.0002816	protein phosphatase 2C domain-containing protein
TGME49_323100	1.64	6.90	1.22E-05	0.00030956	hypothetical protein
TGME49_242240	-2.54	5.94	1.29E-05	0.00032624	rhoptry kinase family protein ROP19A
TGME49_316710	1.24	7.47	1.30E-05	0.00032698	hypothetical protein
TGME49_229440	-1.88	6.25	1.32E-05	0.00033145	zinc finger, C3HC4 type (RING finger) domain-containing protein
TGME49_215540	-8.70	4.96	1.36E-05	0.00034055	hypothetical protein
TGME49_223910	-1.53	7.08	1.40E-05	0.00034744	acyltransferase domain-containing protein
TGME49_247930	-2.87	5.11	1.39E-05	0.00034744	SNARE domain-containing protein
TGME49_290700	1.27	7.87	1.46E-05	0.0003627	hypothetical protein
TGME49_239810	1.60	5.96	1.52E-05	0.00037498	hypothetical protein
TGME49_292110	1.84	6.61	1.56E-05	0.00038434	formate/nitrite transporter protein
TGME49_214400	1.71	4.87	1.69E-05	0.00041128	hypothetical protein
TGME49_259530	-1.62	6.38	1.73E-05	0.0004191	GalNac
TGME49_288400	-1.81	6.11	1.74E-05	0.00042041	LETM1 family protein
TGME49_243410	-1.79	6.18	1.78E-05	0.00042769	tetratricopeptide repeat-containing protein
TGME49_305160	3.04	4.53	1.80E-05	0.00043138	histone H2Ba
TGME49_266100	1.33	6.81	1.85E-05	0.00044017	rhoptry kinase family protein ROP41
TGME49_224220	-1.73	6.62	1.92E-05	0.00045509	serine/threonine-protein phosphatase PP2A catalytic subunit
TGME49_263710	-1.68	6.20	1.94E-05	0.00045757	acyl-CoA:cholesterol acyltransferase alpha ACAT1-alpha
TGME49_214170	-2.00	5.90	1.94E-05	0.00045757	hypothetical protein
TGME49_321640	-2.03	6.04	1.96E-05	0.00046214	cell division protein CDC48AP
TGME49_269180	-1.20	7.79	2.05E-05	0.00048165	MIF4G domain-containing protein
TGME49_276220	1.35	6.04	2.14E-05	0.00050091	hypothetical protein
TGME49_253440	1.17	8.62	2.16E-05	0.00050272	cell-cycle-associated protein kinase SRPK, putative
TGME49_219520	-1.34	7.45	2.17E-05	0.00050379	histone arginine methyltransferase PRMT1
TGME49_296121	1.48	7.18	2.27E-05	0.00052443	hypothetical protein
TGME49_255510	-1.31	7.27	2.35E-05	0.00054113	ankyrin repeat-containing protein
TGME49_253140	1.52	5.83	2.44E-05	0.00055545	hypothetical protein
TGME49_316270	1.40	6.15	2.44E-05	0.00055545	Rab geranylgeranyl transferase type II beta subunit, putative
TGME49_207430	-1.84	6.38	2.44E-05	0.00055545	ATP-dependent RNA helicase DDX1, putative
TGME49_315280	-1.86	5.80	2.48E-05	0.00056212	hypothetical protein
TGME49_212090	1.15	6.72	2.53E-05	0.00057222	hypothetical protein
TGME49_285870	1.32	9.13	2.63E-05	0.0005917	SAG-related sequence SRS20A
TGME49_268230	-3.64	5.64	2.67E-05	0.00059853	hypothetical protein
TGME49_289540	1.20	7.57	2.69E-05	0.00060174	hypothetical protein
TGME49_314070	1.29	6.15	2.76E-05	0.00061523	hypothetical protein
TGME49_246130	1.27	7.99	2.78E-05	0.00061523	serpin (serine proteinase inhibitor) superfamily protein

TGME49_255340	-1.77	5.95	2.78E-05	0.00061523	tetratricopeptide repeat-containing protein
TGME49_263740	-1.88	5.41	2.77E-05	0.00061523	ABC transporter transmembrane region domain-containing protein
TGME49_233130	2.06	5.01	2.88E-05	0.0006334	nucleoside transporter protein
TGME49_253860	1.25	6.69	2.88E-05	0.0006334	Tyrosine kinase-like (TKL) protein
TGME49_260190	-1.57	6.16	2.94E-05	0.00064486	microneme protein MIC13
TGME49_254690	1.25	7.81	3.09E-05	0.00067562	phospholipase/carboxylesterase
TGME49_240700	-1.71	6.20	3.18E-05	0.00069247	ubiquitin family protein
TGME49_203540	1.51	7.63	3.19E-05	0.00069365	RNA binding protein, putative
TGME49_276920	-1.63	6.74	3.24E-05	0.00070203	protein phosphatase 2C domain-containing protein
TGME49_253700	1.05	8.07	3.34E-05	0.00072151	transporter, major facilitator family protein
TGME49_254150	1.91	5.06	3.48E-05	0.00074456	hypothetical protein
TGME49_237015	1.37	7.25	3.49E-05	0.00074582	hypothetical protein
TGME49_258070	-1.50	6.52	3.51E-05	0.00074672	hypothetical protein
TGME49_264870	1.39	7.17	3.56E-05	0.00075299	Sodium:neurotransmitter symporter family protein
TGME49_321540	-1.49	7.23	3.55E-05	0.00075299	hypothetical protein
TGME49_208820	1.40	5.90	3.58E-05	0.00075454	1-deoxy-D-xylulose-5-phosphate synthase
TGME49_226580	1.12	7.53	3.60E-05	0.00075454	hypothetical protein
TGME49_292610	1.62	5.59	3.73E-05	0.00078154	Toxoplasma gondii family C protein
TGME49_213570	4.95	11.16	3.76E-05	0.00078515	hypothetical protein
TGME49_202620	1.18	8.17	3.81E-05	0.00079086	hypothetical protein
TGME49_213680	-2.26	5.20	3.81E-05	0.00079086	MmgE/PrpD family protein
TGME49_245530	3.38	5.81	3.83E-05	0.00079126	hypothetical protein
TGME49_296015	1.22	6.47	3.83E-05	0.00079126	hypothetical protein
TGME49_247770	1.09	7.98	3.90E-05	0.00080249	hypothetical protein
TGME49_272600	-1.29	6.43	4.03E-05	0.0008268	adaptin c-terminal domain-containing protein
TGME49_284660	1.58	5.36	4.09E-05	0.00083268	mitochondrial ribosomal protein s6-2, putative
TGME49_232600	1.57	6.53	4.09E-05	0.00083268	phospholipase, patatin family protein
TGME49_228320	-3.92	4.54	4.07E-05	0.00083268	hypothetical protein
TGME49_253000	1.22	6.88	4.16E-05	0.00084394	ELMO/CED-12 family protein
TGME49_285830	-1.60	5.93	4.17E-05	0.00084394	hypothetical protein
TGME49_304680	-1.82	5.66	4.31E-05	0.00087025	ubiquitin family protein
TGME49_226260	1.23	7.27	4.35E-05	0.00087489	hypothetical protein
TGME49_313230	2.44	9.56	4.39E-05	0.00088135	eukaryotic initiation factor-2, alpha subunit
TGME49_210430	-1.23	7.63	4.57E-05	0.00091561	DnaJ domain-containing protein
TGME49_310210	1.73	5.89	4.63E-05	0.00092286	hypothetical protein
TGME49_255960	-1.75	6.00	4.63E-05	0.00092286	hypothetical protein
TGME49_286480	2.55	5.39	4.70E-05	0.00093441	hypothetical protein
TGME49_240580	-1.94	5.54	4.80E-05	0.00095103	hypothetical protein
TGME49_299240	1.62	5.35	4.84E-05	0.00095571	hypothetical protein
TGME49_297180	-1.25	8.34	4.88E-05	0.00096169	hypothetical protein
TGME49_254000	1.26	7.14	5.12E-05	0.00100273	hypothetical protein
TGME49_311660	-1.65	5.95	5.21E-05	0.00101937	histone lysine methyltransferase, SET, putative
TGME49_240950	-3.14	5.11	5.27E-05	0.00102775	hypothetical protein
TGME49_249770	1.95	4.97	5.30E-05	0.00103013	Nmda1 protein
TGME49_238240	5.33	11.68	5.32E-05	0.00103241	bystin protein
TGME49_200360	1.62	9.81	5.48E-05	0.00105807	hypothetical protein
TGME49_253490	1.38	6.93	5.48E-05	0.00105807	hypothetical protein
TGME49_257910	4.40	5.13	5.72E-05	0.00110166	hypothetical protein
TGME49_261000	-1.73	6.99	5.75E-05	0.00110431	MutS domain V domain-containing protein
TGME49_217770	1.14	6.54	5.80E-05	0.00110691	hypothetical protein
TGME49_207630	-3.31	4.99	5.80E-05	0.00110691	peptidyl-tRNA hydrolase domain-containing protein
TGME49_269930	-2.18	6.65	5.99E-05	0.00114015	calcium binding egf domain-containing protein

TGME49_266990	-1.22	7.64	6.00E-05	0.00114068	beta-COP
TGME49_234640	-1.52	6.39	6.03E-05	0.0011418	hypothetical protein
TGME49_270830	1.18	7.26	6.44E-05	0.00121316	small nuclear ribonucleoprotein
TGME49_249180	1.05	8.01	6.61E-05	0.00123684	bifunctional dihydrofolate reductase-thymidylate synthase
TGME49_201380	1.04	8.56	6.67E-05	0.00124462	chorismate synthase, putative
TGME49_253170	1.97	6.90	6.82E-05	0.00126914	zinc carboxypeptidase, putative
TGME49_307570	-1.64	6.39	6.85E-05	0.00127289	glycerol-3-phosphate dehydrogenase (gpdh), putative
TGME49_310100	-2.01	5.38	7.17E-05	0.00132208	mannosyltransferase (pig-m) protein
TGME49_275450	1.04	7.53	7.33E-05	0.00134483	hypothetical protein
TGME49_249620	-1.53	6.08	7.73E-05	0.00140355	histone deacetylase HDAC2
TGME49_280375	2.90	5.70	7.79E-05	0.00140723	hypothetical protein
TGME49_208910	-1.60	6.43	7.77E-05	0.00140723	hypothetical protein
TGME49_289050	1.75	7.40	8.16E-05	0.00147089	FIKK kinase, putative
TGME49_249860	-1.42	7.01	8.26E-05	0.00148335	hypothetical protein
TGME49_299060	1.37	6.85	8.31E-05	0.00148654	sodium/hydrogen exchanger NHE2
TGME49_242640	1.10	6.51	8.35E-05	0.00148733	hypothetical protein
TGME49_269710	-1.47	6.75	8.34E-05	0.00148733	hypothetical protein
TGME49_203290	1.61	8.29	8.49E-05	0.00150776	hypothetical protein
TGME49_312160	1.26	7.86	8.61E-05	0.0015259	hypothetical protein
TGME49_214530	-2.24	6.70	8.74E-05	0.00154499	DnaJ domain-containing protein
TGME49_226680	-1.23	7.34	8.89E-05	0.00156828	hypothetical protein
TGME49_247250	-1.28	6.92	9.00E-05	0.00158135	RbAp46
TGME49_264780	-1.66	5.70	9.06E-05	0.00158626	UTP-glucose-1-phosphate uridylyltransferase subfamily protein
TGME49_214380	-1.99	5.63	9.11E-05	0.00159144	hypothetical protein
TGME49_277540	1.89	4.45	9.22E-05	0.00160702	hypothetical protein
TGME49_267750	-1.38	6.69	9.24E-05	0.00160702	hypothetical protein
TGME49_214090	1.81	7.04	9.43E-05	0.00163393	signal peptidase
TGME49_293380	-2.21	5.59	9.44E-05	0.00163393	histone lysine acetyltransferase HAT1
TGME49_287160	-1.19	7.58	9.53E-05	0.00164537	internal kinesin motor domain protein
TGME49_241155	1.88	5.87	9.76E-05	0.00167846	hypothetical protein
TGME49_288840	1.26	7.00	9.76E-05	0.00167846	hypothetical protein
TGME49_237230	1.49	9.68	9.95E-05	0.00170485	hypothetical protein
TGME49_268790	1.04	8.26	9.96E-05	0.00170485	hypothetical protein
TGME49_253650	1.02	8.06	0.00010196	0.00174052	DnaJ C terminal region domain-containing protein
TGME49_268620	-2.80	5.03	0.00010302	0.00175472	blood stage antigen 41-3 precursor, putative
TGME49_217360	-2.15	5.84	0.00010341	0.00175727	hypothetical protein
TGME49_298840	1.36	6.46	0.0001044	0.00177003	hypothetical protein
TGME49_217350	-2.31	5.20	0.0001057	0.00177991	methyltransferase MTA70, putative
TGME49_275990	1.29	6.60	0.00010839	0.00180531	hypothetical protein
TGME49_259700	1.16	7.16	0.00010835	0.00180531	hypothetical protein
TGME49_207230	1.16	6.18	0.00010773	0.00180531	hypothetical protein
TGME49_271930	-1.06	8.48	0.00010864	0.00180531	hypothetical protein
TGME49_205150	-1.81	5.18	0.00010837	0.00180531	ACR, YagE family COG1723 domain-containing protein
TGME49_223150	-2.38	5.54	0.00010867	0.00180531	START domain-containing protein
TGME49_294930	-1.37	7.12	0.00011075	0.00183576	leucine rich repeat-containing protein
TGME49_260310	1.84	4.60	0.00011361	0.00187882	ATP-binding cassette transporter ABC.B1
TGME49_275320	-1.34	7.96	0.00011405	0.0018819	penicillin amidase
TGME49_248830	-1.43	6.41	0.00011478	0.00188945	phosphoinositide phospholipase PIPLC
TGME49_253370	1.27	7.36	0.00011576	0.00189739	hypothetical protein

TGME49_283710	-1.50	6.77	0.00011619	0.00189739	Longevity-assurance protein (LAG1) domain-containing protein
TGME49_254580	1.29	7.13	0.00011748	0.001913	UDP-galactose transporter family protein
TGME49_321560	-1.47	6.59	0.00011777	0.00191356	zinc knuckle domain-containing protein
TGME49_278270	-2.06	5.13	0.00012045	0.00194848	nucleolar protein, structural component of H/ACA snoRNPs, putative
TGME49_264660	1.41	8.88	0.00012145	0.00196038	SAG-related sequence SRS44
TGME49_229650	-1.58	6.31	0.00012329	0.00198585	josephin protein
TGME49_210380	-1.26	6.94	0.00012436	0.00199871	hypothetical protein
TGME49_235740	-1.69	5.82	0.00012526	0.00200877	hypothetical protein
TGME49_225440	-4.09	4.61	0.00012692	0.00203106	hypothetical protein
TGME49_263080	1.10	7.13	0.0001306	0.00208087	hypothetical protein
TGME49_320110	2.51	8.51	0.00013476	0.00213799	proliferating cell nuclear antigen PCNA2
TGME49_228370	-1.53	5.97	0.00013468	0.00213799	hypothetical protein
TGME49_214540	1.16	6.96	0.000143	0.00225424	hypothetical protein
TGME49_251500	-1.28	7.59	0.0001428	0.00225424	eukaryotic initiation factor-3, subunit 3, putative
TGME49_273030	-1.68	5.86	0.00014367	0.00225997	phosphoglycerate mutase family protein
TGME49_311780	-1.47	6.01	0.0001462	0.00229487	Zn-containing alcohol dehydrogenase
TGME49_215360	1.53	5.92	0.00014656	0.00229568	hypothetical protein
TGME49_225240	1.15	6.65	0.00014707	0.00229887	50S ribosomal protein L13, putative
TGME49_262970	1.54	7.43	0.00015	0.00233965	hypothetical protein
TGME49_293820	-1.22	7.28	0.0001509	0.00234738	calpain family cysteine protease domain-containing protein
TGME49_289720	-1.60	6.89	0.00015113	0.00234738	hypothetical protein
TGME49_295990	1.11	6.85	0.00015382	0.00238426	ubiquitin conjugating enzyme E2, putative
TGME49_206695	1.95	4.49	0.00016032	0.00247464	hypothetical protein
TGME49_311440	1.24	6.20	0.00016394	0.00251799	SAG-related sequence SRS50
TGME49_202980	-1.34	6.64	0.00016458	0.00251932	hypothetical protein
TGME49_205250	1.32	9.50	0.00016665	0.00254576	rhoptry protein ROP18
TGME49_226020	1.25	8.77	0.00016756	0.00255441	transporter, major facilitator family protein
TGME49_278800	-1.21	7.30	0.00016884	0.00256872	zinc finger protein 36 family 3 protein
TGME49_262500	-1.50	7.65	0.00017147	0.00260332	hypothetical protein
TGME49_306260	1.17	6.14	0.00017508	0.00264731	hypothetical protein
TGME49_318320	-2.05	5.30	0.00017586	0.00265368	ATP-dependent Clp endopeptidase, proteolytic subunit ClpP domain-containing protein
TGME49_255240	-1.95	5.60	0.00017657	0.00265893	hypothetical protein
TGME49_220400	1.70	10.26	0.00017926	0.00269402	actin depolymerizing factor ADF
TGME49_241140	-1.88	5.27	0.0001812	0.00271213	DEAD/DEAH box helicase domain-containing protein
TGME49_219610	1.77	5.75	0.00018223	0.00272213	hypothetical protein
TGME49_306470	-1.34	6.37	0.00018271	0.0027237	isoprenylcysteine carboxyl methyltransferase (icmt) family protein
TGME49_217010	-1.53	6.08	0.00018638	0.00277281	hypothetical protein
TGME49_277260	2.11	9.98	0.00018803	0.00279175	hypothetical protein
TGME49_231180	-2.97	5.84	0.00018927	0.00280466	hypothetical protein
TGME49_254610	1.37	5.28	0.00019092	0.00281784	Tim10/DDP family zinc finger superfamily protein
TGME49_315910	1.44	5.62	0.00019569	0.00287684	hypothetical protein
TGME49_201260	-1.14	8.89	0.0001957	0.00287684	sugar transporter ST3
TGME49_283820	-2.68	4.90	0.00020268	0.00296774	glycine cleavage T-protein (aminomethyl transferase) domain-containing protein
TGME49_211030	-1.10	8.08	0.00020475	0.00298627	hypothetical protein
TGME49_260830	-2.72	5.12	0.00020594	0.00299762	hypothetical protein
TGME49_240430	-2.18	4.90	0.00020659	0.00300129	glyoxalase family protein

TGME49_275755	2.33	5.33	0.00020703	0.00300178	hypothetical protein
TGME49_310130	-1.52	6.23	0.00020806	0.00301072	Spc97 / Spc98 family protein
TGME49_240440	1.19	5.78	0.00020901	0.00301866	hypothetical protein
TGME49_258390	1.39	5.51	0.0002127	0.00305427	DnaJ protein, putative
TGME49_307580	-1.23	6.83	0.00021354	0.00305427	CBS domain-containing protein
TGME49_215040	-1.33	6.56	0.00021334	0.00305427	HEAT repeat-containing protein
TGME49_216040	-2.16	6.31	0.00021273	0.00305427	30S ribosomal protein S15, putative
TGME49_215390	1.69	4.45	0.00021478	0.00306603	TIM10 family protein, putative
TGME49_310360	-1.20	7.38	0.00021601	0.00307772	hypothetical protein
TGME49_291930	3.68	9.99	0.00021864	0.00310928	RNA recognition motif-containing protein
TGME49_257360	1.87	5.87	0.00022073	0.00312699	hypothetical protein
TGME49_202830	1.10	9.13	0.00022118	0.00312739	Elicitor-responsive protein, putative
TGME49_263720	-2.18	5.02	0.00022205	0.00313362	HMG (high mobility group) box domain-containing protein
TGME49_248900	-2.04	5.41	0.00022272	0.00313709	hypothetical protein
TGME49_311210	-1.38	6.42	0.00022784	0.00320318	hypothetical protein
TGME49_258940	1.50	5.29	0.00023364	0.00327231	acylphosphatase family protein
TGME49_297420	-1.75	5.89	0.0002366	0.00330753	beta-tubulin cofactor D, putative
TGME49_220940	1.51	5.59	0.00024171	0.00336642	ribosomal RNA methyltransferase (FtsJ) family protein
TGME49_279100	1.08	9.07	0.00024172	0.00336642	hypothetical protein
TGME49_254880	1.03	8.02	0.00024401	0.00339181	Alpha-galactosidase
TGME49_253615	1.46	5.78	0.00024777	0.00341981	hypothetical protein
TGME49_285710	1.31	5.78	0.00024654	0.00341981	hypothetical protein
TGME49_271430	-2.03	5.19	0.00024712	0.00341981	hypothetical protein
TGME49_252630	1.10	8.01	0.00025155	0.00345142	hypothetical protein
TGME49_298610	-1.02	7.94	0.00025138	0.00345142	GYF domain-containing protein
TGME49_202650	-1.66	5.60	0.00025401	0.00347274	hypothetical protein
TGME49_301280	-2.29	5.06	0.00025405	0.00347274	hypothetical protein
TGME49_244380	2.30	8.24	0.0002549	0.003478	cactin
TGME49_309070	-1.93	5.42	0.00025634	0.00348477	hypothetical protein
TGME49_268570	-1.14	7.32	0.00025918	0.00351065	zinc finger (CCCH type) motif-containing protein
TGME49_220530	-1.91	5.46	0.00025947	0.00351065	AP2 domain transcription factor AP2V-1
TGME49_257290	-1.92	5.15	0.00025966	0.00351065	RNA 2'-phosphotransferase, Tpt1/KptA family protein
TGME49_253710	1.14	7.77	0.00026178	0.00353284	hypothetical protein
TGME49_245980	2.76	5.83	0.00026623	0.00358635	hypothetical protein
TGME49_253900	1.57	5.99	0.00027101	0.00363758	parasite porphobilinogen synthase PBGS
TGME49_227390	-1.67	5.40	0.00027099	0.00363758	hypothetical protein
TGME49_321410	2.79	8.06	0.00027185	0.00364221	hypothetical protein
TGME49_272650	-1.50	6.03	0.00027238	0.00364278	3'5'-cyclic nucleotide phosphodiesterase domain-containing protein
TGME49_290970	2.67	6.33	0.00028983	0.00384145	8-amino-7-oxononanoate synthase
TGME49_218830	-2.05	5.10	0.00028966	0.00384145	hypothetical protein
TGME49_237290	-1.34	6.65	0.0002916	0.00385804	hypothetical protein
TGME49_306410	-2.03	5.32	0.00029279	0.00385987	hypothetical protein
TGME49_227430	-1.65	5.71	0.0002961	0.00389663	transmembrane amino acid transporter protein
TGME49_258670	-1.66	5.14	0.00029873	0.00392422	hypothetical protein
TGME49_208010	1.18	6.38	0.00030131	0.00395111	hypothetical protein
TGME49_224830	-2.46	4.54	0.00030261	0.0039612	hypothetical protein
TGME49_293500	-2.44	5.07	0.00030727	0.00401513	hypothetical protein
TGME49_227570	1.08	6.79	0.00030857	0.00402499	transmembrane amino acid transporter protein
TGME49_320050	7.07	16.23	0.00031559	0.00409026	ribosomal protein RPL5

TGME49_217640	1.39	6.15	0.00031515	0.00409026	hypothetical protein
TGME49_253780	1.16	6.63	0.00031578	0.00409026	GTP cyclohydrolase I, putative
TGME49_214970	-1.28	8.09	0.00031639	0.00409098	DNA replication licensing factor, putative
TGME49_256060	-1.24	6.61	0.00031722	0.00409186	nucleosome assembly protein (nap) protein
TGME49_286140	-1.63	5.87	0.00032595	0.00419266	hypothetical protein
TGME49_253820	1.05	8.35	0.00033729	0.00433105	hypothetical protein
TGME49_289650	0.98	8.20	0.00034007	0.00435909	PEP-carboxykinase I
TGME49_246530	1.85	5.05	0.00034283	0.00438696	phospholipase D active site domain-containing protein
TGME49_248160	-2.29	4.95	0.00034393	0.00439345	hypothetical protein
TGME49_245480	2.41	5.06	0.00034499	0.00439932	hypothetical protein
TGME49_262010	1.18	6.19	0.00035281	0.00449132	calmodulin CAM2
TGME49_295400	-2.03	5.29	0.00035919	0.00456473	hypothetical protein
TGME49_267590	-1.55	5.96	0.00036778	0.00465789	hypothetical protein
TGME49_259240	3.87	11.70	0.0003732	0.0047185	ribosomal protein RPS21
TGME49_251730	-1.40	5.85	0.00037483	0.00473105	hypothetical protein
TGME49_218200	-2.16	5.22	0.00037597	0.00473731	UDP-sugar pyrophospharylase
TGME49_251530	2.18	4.57	0.00038103	0.00478487	hypothetical protein
TGME49_225680	-2.00	5.41	0.00039855	0.00497119	hypothetical protein
TGME49_289340	1.81	5.26	0.00040109	0.00499445	hypothetical protein
TGME49_323110	1.76	5.68	0.00040292	0.00500874	hypothetical protein
TGME49_280580	1.79	5.41	0.00040369	0.00500994	SAG-related sequence SRS35B
TGME49_214270	2.72	9.52	0.00040485	0.00501594	translation initiation factor IF-2, putative
TGME49_228630	-1.09	6.96	0.00041207	0.00506598	hypothetical protein
TGME49_209210	-1.25	6.93	0.00041234	0.00506598	hypothetical protein
TGME49_288700	-1.76	5.73	0.00041191	0.00506598	RecF/RecN/SMC N terminal domain-containing protein
TGME49_248400	1.09	6.18	0.00041542	0.0050873	glyoxalase I, putative
TGME49_251520	1.15	5.78	0.0004188	0.00512022	hypothetical protein
TGME49_247360	0.99	7.30	0.00042302	0.00515238	PAP2 superfamily protein
TGME49_216020	-1.83	5.94	0.00042351	0.00515238	peptidase family c78 protein
TGME49_244040	-1.83	5.58	0.00044241	0.0053735	HEAT repeat-containing protein
TGME49_295472	-1.50	6.42	0.00044644	0.00540307	C2 domain-containing protein
TGME49_318480	-1.61	5.98	0.00044602	0.00540307	SWI2/SNF2-containing protein RAD5
TGME49_244230	-1.85	5.98	0.00044703	0.00540307	hypothetical protein
TGME49_254630	1.08	6.41	0.00044998	0.00542985	CMGC kinase
TGME49_224540	-1.65	5.44	0.00045125	0.00543632	hypothetical protein
TGME49_263860	-1.85	5.11	0.00045363	0.00545612	hypothetical protein
TGME49_220290	-1.84	5.53	0.00045466	0.00545961	hypothetical protein
TGME49_258590	-2.06	5.43	0.00046042	0.0055198	hypothetical protein
TGME49_312210	1.89	4.65	0.00046326	0.00554495	hypothetical protein
TGME49_254915	2.78	4.55	0.0004714	0.0056254	hypothetical protein
TGME49_246050	-2.18	5.13	0.0004715	0.0056254	hypothetical protein
TGME49_239710	-1.61	5.95	0.00047234	0.00562632	phosphomannomutase
TGME49_237830	-1.66	5.67	0.00047386	0.00563538	DNA polymerase I domain-containing protein
TGME49_200320	-1.13	7.51	0.00047618	0.00565326	hypoxanthine-xanthine-guanine phosphoribosyl transferase HXGPRT
TGME49_300030	-1.19	6.63	0.00047689	0.00565326	hypothetical protein
TGME49_203790	1.02	8.37	0.00048396	0.00572027	hypothetical protein
TGME49_206610	-1.41	6.14	0.00048409	0.00572027	pyruvate dehydrogenase complex subunit PDH-E2
TGME49_209050	-1.38	6.89	0.00048677	0.00574284	Tyrosine kinase-like (TKL) protein
TGME49_236890	1.02	7.34	0.00049118	0.00578556	hypothetical protein
TGME49_313910	1.57	10.01	0.00049959	0.00587529	RNA recognition motif 2 protein

TGME49_254390	1.58	7.06	0.00050075	0.0058796	CRAL/TRIO domain-containing protein
TGME49_277490	1.05	7.17	0.00050586	0.00592088	hypothetical protein
TGME49_261022	-2.98	7.41	0.0005053	0.00592088	dynein heavy chain family protein
TGME49_273950	-1.88	5.27	0.00051067	0.00596775	replication factor C subunit 5, putative
TGME49_224060	0.94	7.95	0.00051455	0.00598479	thioredoxin, putative
TGME49_242570	-1.04	7.55	0.00051418	0.00598479	hypothetical protein
TGME49_261720	-1.16	7.53	0.00051304	0.00598479	metal cation transporter, ZIP family protein
TGME49_207065	1.20	5.52	0.00051894	0.00600469	hypothetical protein
TGME49_207830	1.05	6.06	0.00051865	0.00600469	MORN repeat-containing protein
TGME49_203390	-1.16	7.31	0.00051776	0.00600469	CRAL/TRIO domain-containing protein
TGME49_221390	-1.37	6.10	0.0005195	0.00600469	hypothetical protein
TGME49_249550	-1.52	5.67	0.00053136	0.00610449	hypothetical protein
TGME49_212100	-1.56	5.33	0.00053143	0.00610449	ThiF family protein
TGME49_216375	-2.35	4.74	0.00053084	0.00610449	hypothetical protein
TGME49_253950	1.22	5.56	0.00053665	0.0061454	protein fam50a, putative
TGME49_290670	0.99	8.78	0.00053847	0.00615662	leucyl aminopeptidase LAP
TGME49_217780	1.47	5.16	0.00054126	0.006179	Sec20 protein
TGME49_290740	-2.09	5.25	0.00054624	0.00620723	hypothetical protein
TGME49_308010	-1.86	6.49	0.00054969	0.0062368	hypothetical protein
TGME49_253940	1.04	6.85	0.00055157	0.00624856	CAM Kinase family, incomplete catalytic triad
TGME49_294730	-1.42	6.07	0.00055332	0.00625884	hypothetical protein
TGME49_215710	-1.38	6.00	0.00055445	0.00626204	hypothetical protein
TGME49_230430	-1.88	5.37	0.00056273	0.00634589	vesicle-associated membrane protein, putative
TGME49_224170	1.76	4.86	0.00056589	0.00637183	SAG-related sequence SRS60A
TGME49_220910	-1.47	6.95	0.0005682	0.00638819	HEAT repeat-containing protein
TGME49_246330	-1.15	6.92	0.00056962	0.0063944	CRAL/TRIO domain-containing protein
TGME49_233030	-1.03	8.47	0.00058262	0.00652056	gliding-associated protein GAP70
TGME49_289310	-1.26	6.48	0.00058673	0.00654682	cullin family protein
TGME49_235980	-1.58	5.63	0.00058866	0.00655846	ARIADNE family protein
TGME49_297720	-1.24	7.42	0.00059117	0.00657655	trehalose-phosphatase
TGME49_311920	-1.12	7.15	0.00059573	0.00661734	GRAM domain-containing protein
TGME49_297730	-1.75	5.78	0.00059895	0.00664309	transcription elongation factor 1, putative
TGME49_249990	1.43	9.14	0.00060552	0.00670593	hypothetical protein
TGME49_224020	1.06	6.40	0.00061361	0.00678538	hypothetical protein
TGME49_254950	1.38	5.04	0.00061786	0.00681209	RNA cap guanine-N2 methyltransferase
TGME49_262825	1.47	4.65	0.00062213	0.00682602	peptidase family c50 protein
TGME49_239410	-1.12	7.88	0.00062358	0.00682602	hypothetical protein
TGME49_293470	-1.12	7.24	0.00062105	0.00682602	hypothetical protein
TGME49_208040	-1.50	5.94	0.00062308	0.00682602	aldo-keto reductase
TGME49_212300	0.96	8.44	0.00063163	0.00689214	hypothetical protein
TGME49_286180	-2.39	4.55	0.00063137	0.00689214	tRNA ligases class I (M) protein
TGME49_299015	1.90	5.22	0.00063513	0.00691973	hypothetical protein
TGME49_312380	-1.92	5.23	0.00063603	0.00691973	tetratricopeptide repeat-containing protein
TGME49_269420	2.15	6.35	0.00064069	0.00696024	hypothetical protein
TGME49_237860	-2.24	4.89	0.00064292	0.00697421	protein kinase domain-containing protein
TGME49_306020	1.21	5.49	0.00064776	0.00701154	hypothetical protein
TGME49_213730	0.99	7.46	0.00064825	0.00701154	lanthionine synthetase C family protein
TGME49_239490	1.04	8.02	0.00065182	0.00702957	dehydrogenase E1 component family protein
TGME49_244670	0.95	7.10	0.00065302	0.00703235	hypothetical protein
TGME49_225480	-1.79	5.80	0.00065672	0.00706193	hypothetical protein
TGME49_264970	-1.82	5.65	0.00066279	0.00711679	hypothetical protein
TGME49_253360	0.97	7.20	0.0006654	0.00713448	hypothetical protein
TGME49_260610	-2.50	4.95	0.00066699	0.00714123	methyltransferase

TGME49_232780	-1.39	6.35	0.00066938	0.00715652	hypothetical protein
TGME49_305990	2.44	4.61	0.00067037	0.00715669	hypothetical protein
TGME49_202580	1.59	6.20	0.00068139	0.00724304	ATPase, AAA family protein
TGME49_315250	1.11	5.99	0.00068027	0.00724304	GAMM1 protein, putative
TGME49_220610	-1.34	6.23	0.00068103	0.00724304	protein phosphatase 2C domain-containing protein
TGME49_254460	1.12	7.06	0.00068717	0.00728632	hypothetical protein
TGME49_320610	-1.67	5.23	0.00068743	0.00728632	hypothetical protein
TGME49_208370	1.17	7.87	0.00069257	0.00728871	myosin heavy chain, putative
TGME49_234180	1.08	7.18	0.00069215	0.00728871	hypothetical protein
TGME49_293550	-1.09	7.18	0.00069176	0.00728871	hypothetical protein
TGME49_235610	-1.73	5.16	0.00069063	0.00728871	ATPase, AAA family protein
TGME49_240740	-1.43	5.71	0.00070131	0.00737021	Sec1 family protein
TGME49_293040	-1.63	6.54	0.00070282	0.00737567	hypothetical protein
TGME49_262600	-2.01	4.55	0.00070808	0.00742029	hypothetical protein
TGME49_321550	1.11	5.79	0.00071173	0.00744802	hypothetical protein
TGME49_210408	1.67	10.56	0.00072462	0.00753998	HMG (high mobility group) box domain-containing protein
TGME49_277700	1.39	4.92	0.00072482	0.00753998	ribosomal protein S14 precursor, putative
TGME49_285170	-2.94	4.61	0.0007256	0.00753998	methyltransferase small, putative
TGME49_209090	-1.32	6.23	0.00073556	0.00763277	proteasome maturation factor ump1 protein
TGME49_205200	1.04	6.26	0.00073947	0.00766253	hypothetical protein
TGME49_232960	-1.21	7.01	0.00074891	0.00774959	oxidoreductase, 2OG-Fe(II) oxygenase family protein
TGME49_207160	1.90	5.07	0.0007634	0.00786841	SAG-related sequence SRS49D
TGME49_253310	1.17	5.37	0.00076358	0.00786841	hypothetical protein
TGME49_305340	-0.97	8.17	0.0007628	0.00786841	corepressor complex CRC230
TGME49_308060	1.36	6.55	0.00076739	0.00788569	hypothetical protein
TGME49_311905	-2.49	4.73	0.00076666	0.00788569	hypothetical protein
TGME49_206640	-1.11	6.71	0.00077106	0.00791244	hypothetical protein
TGME49_286510	1.27	5.67	0.00077336	0.00792508	hypothetical protein
TGME49_301216	-2.44	4.54	0.00077868	0.00796859	endonuclease/exonuclease/phosphatase family protein
TGME49_283550	1.04	6.70	0.0007963	0.00811693	hypothetical protein
TGME49_293680	-1.47	6.69	0.00079647	0.00811693	hypothetical protein
TGME49_214220	1.16	9.62	0.00079924	0.00813402	hypothetical protein
TGME49_286440	1.01	6.38	0.00080114	0.00814214	malic enzyme
TGME49_260790	-1.54	5.56	0.00080772	0.00818658	RAP domain-containing protein
TGME49_255635	-3.83	5.32	0.00080688	0.00818658	hypothetical protein
TGME49_294890	-1.25	6.50	0.00081081	0.00820665	hypothetical protein
TGME49_258410	1.07	9.04	0.00083772	0.00844447	photosensitized INA-labeled protein PHIL1
TGME49_294200	1.35	9.66	0.00084177	0.00847377	glucose-6-phosphate 1-dehydrogenase
TGME49_313830	-1.28	6.30	0.00084914	0.00852557	AARP2CN (NUC121) domain-containing protein
TGME49_268810	-1.47	6.39	0.00084922	0.00852557	ck2 beta subunit
TGME49_223600	-1.69	5.52	0.00085042	0.00852602	hypothetical protein
TGME49_203080	1.07	6.45	0.00085363	0.00853513	RNA recognition motif-containing protein
TGME49_278050	-1.03	8.02	0.00085675	0.00855481	proteasome subunit alpha type 1, putative
TGME49_320430	-1.26	6.00	0.00088294	0.00879262	cell-cycle-control protein (translation regulation), putative
TGME49_284010	-1.68	5.11	0.00088993	0.00885031	5'-3' exonuclease, N-terminal resolvase family domain-containing protein
TGME49_316230	-1.42	5.94	0.00090056	0.00894403	SAC1 phosphoinositide phosphatase, putative
TGME49_293330	1.37	5.12	0.00090322	0.00895842	hypothetical protein
TGME49_243430	1.04	7.30	0.00091738	0.00908673	OTU family cysteine protease

TGME49_255660	-1.26	6.20	0.00092147	0.00911501	EF hand domain-containing protein
TGME49_241130	-2.00	4.51	0.00092372	0.0091251	hypothetical protein
TGME49_315620	-1.14	6.98	0.00093803	0.00924181	vacuolar ATP synthase subunit C, putative
TGME49_261400	-0.97	7.67	0.00094728	0.00932062	hypothetical protein
TGME49_278770	-1.27	7.00	0.00094942	0.00932922	hypothetical protein
TGME49_224210	-1.35	5.83	0.00095096	0.009332	hypothetical protein
TGME49_209970	-1.38	6.05	0.00095587	0.00936781	Spc97 / Spc98 family protein
TGME49_234900	-0.99	7.30	0.00096935	0.00947094	PHD-finger domain-containing protein
TGME49_213300	-1.59	5.24	0.00097023	0.00947094	hypothetical protein
TGME49_297940	-1.28	7.54	0.00098247	0.0095778	single-strand binding protein
TGME49_238110	-1.61	6.11	0.0009905	0.00964341	replication factor a protein 3 protein
TGME49_311310	-1.08	8.09	0.00099693	0.00969328	protein phosphatase 2B catalytic subunit, calcineurin family phosphatse superfamily protein
TGME49_253990	1.02	6.61	0.00100433	0.00972686	hypothetical protein
TGME49_237110	-1.22	6.44	0.00100263	0.00972686	replication factor C subunit 2, putative
TGME49_262980	-1.73	5.57	0.00100373	0.00972686	hypothetical protein
TGME49_250100	1.23	6.54	0.00100861	0.00974839	hypothetical protein
TGME49_311770	-1.18	6.85	0.00100918	0.00974839	hypothetical protein
TGME49_318760	1.10	6.50	0.00101542	0.00979586	hypothetical protein
TGME49_262700	-1.71	5.53	0.00101735	0.0097972	tetratricopeptide repeat-containing protein
TGME49_231870	-1.89	5.00	0.00101979	0.00979974	tetratricopeptide repeat-containing protein
TGME49_245475	1.52	5.44	0.00103691	0.00995141	hypothetical protein
TGME49_299250	1.19	5.36	0.00104767	0.01002863	hypothetical protein
TGME49_260620	0.84	8.02	0.00106431	0.01014859	hypothetical protein
TGME49_253880	0.86	7.79	0.00109769	0.01044003	GNS1/SUR4 family protein
TGME49_270510	-1.37	9.30	0.00110234	0.01045741	asparaginyl-tRNA synthetase (NOB+tRNA synthase)
TGME49_227780	1.55	5.00	0.00110586	0.01047738	hypothetical protein
TGME49_272640	-1.31	6.48	0.00114752	0.01083051	eukaryotic initiation factor-2B, epsilon subunit, putative
TGME49_239740	1.16	9.48	0.00115435	0.01086729	dense granule protein GRA14
TGME49_308950	1.04	8.04	0.00115316	0.01086729	histidine acid phosphatase superfamily protein
TGME49_254270	1.10	5.62	0.00115896	0.01089686	hypothetical protein
TGME49_271270	0.92	7.35	0.00116086	0.01090093	hypothetical protein
TGME49_288040	-1.13	6.46	0.00117831	0.01103674	hypothetical protein
TGME49_306190	0.96	9.06	0.00118114	0.01104931	hypothetical protein
TGME49_262860	-1.63	5.63	0.00118524	0.01107372	ADP-ribosylation factor family protein 1, putative
TGME49_225990	-1.34	5.72	0.0011889	0.01109393	acyl transferase domain-containing protein
TGME49_263660	-2.43	4.59	0.00119822	0.01116681	hypothetical protein
TGME49_258740	-1.35	6.23	0.00120544	0.01121994	eukaryotic initiation factor-2A, putative
TGME49_252360	1.05	9.12	0.00121013	0.01124416	rhoptry kinase family protein ROP24 (incomplete catalytic triad)
TGME49_247485	-1.40	5.90	0.00121107	0.01124416	zinc finger, C3HC4 type (RING finger) domain-containing protein
TGME49_215785	1.79	9.54	0.00121831	0.0112831	rhoptry protein ROP2A
TGME49_254470	1.02	9.17	0.00121725	0.0112831	hypothetical protein
TGME49_318570	1.98	5.58	0.00123734	0.01143076	SFT2 family protein
TGME49_225790	1.61	7.29	0.00124598	0.0114802	PDI family protein
TGME49_230705	1.20	6.59	0.00124771	0.0114802	hypothetical protein
TGME49_265440	-1.16	6.82	0.00124889	0.0114802	hypothetical protein
TGME49_226520	1.14	6.01	0.00125087	0.01148413	hypothetical protein
TGME49_228300	-1.74	5.32	0.00127947	0.01171766	CCDC25 protein
TGME49_304750	-1.89	4.72	0.00127835	0.01171766	zinc finger, C3HC4 type (RING finger) domain-containing protein

TGME49_285470	1.63	5.47	0.00128831	0.01178402	patched family protein
TGME49_248130	-2.03	5.04	0.00129028	0.01178755	hypothetical protein
TGME49_295015	1.45	5.35	0.00129605	0.01179016	patched family protein
TGME49_201840	1.27	7.09	0.00129543	0.01179016	aspartyl protease ASP1
TGME49_272380	0.85	8.00	0.00130012	0.01179016	hypothetical protein
TGME49_270930	-1.08	6.53	0.00129811	0.01179016	hypothetical protein
TGME49_212800	-1.48	6.59	0.00129997	0.01179016	hypothetical protein
TGME49_210370	0.86	8.26	0.0013018	0.01179097	hypothetical protein
TGME49_313600	-1.36	6.04	0.00130427	0.01179895	DDHD domain-containing protein
TGME49_237195	-1.66	5.58	0.00131048	0.01182627	hypothetical protein
TGME49_222100	1.01	6.62	0.00131438	0.01183537	hypothetical protein
TGME49_291150	-1.79	5.30	0.00131469	0.01183537	hypothetical protein
TGME49_246490	-1.02	7.85	0.00133295	0.01198525	hypothetical protein
TGME49_292200	-0.93	7.70	0.001336	0.0119876	RNA recognition motif-containing protein
TGME49_315600	-1.37	5.85	0.00133645	0.0119876	MCM2/3/5 family protein
TGME49_214600	-1.21	6.07	0.00133916	0.01199741	hypothetical protein
TGME49_205265	1.64	6.35	0.00135967	0.01216642	transporter, cation channel family protein
TGME49_218920	0.86	7.67	0.0013621	0.01217347	proteasome subunit beta type, putative
TGME49_270530	-1.14	6.58	0.00139367	0.01244061	ubiquitin fusion degradation protein UFD1CY
TGME49_208580	-1.26	6.18	0.00140175	0.01248359	DNA ligase 1, putative
TGME49_201780	1.52	10.10	0.00140892	0.01253144	microneme protein MIC2
TGME49_254070	0.92	7.76	0.00141251	0.0125483	hypothetical protein
TGME49_273815	-1.49	5.38	0.00142162	0.01259169	hypothetical protein
TGME49_214320	1.16	9.30	0.00143812	0.0127148	facilitative glucose transporter GT1
TGME49_212270	1.15	9.35	0.00146576	0.01292836	hypothetical protein
TGME49_253640	0.95	6.96	0.0014647	0.01292836	hypothetical protein
TGME49_249240	1.22	9.83	0.00147131	0.01293111	calmodulin, putative
TGME49_316260	0.99	8.32	0.0014702	0.01293111	hypothetical protein
TGME49_311890	-0.98	7.52	0.00146916	0.01293111	hypothetical protein
TGME49_316430	-1.74	6.04	0.00148264	0.01299983	target of rapamycin (TOR), putative
TGME49_285230	-1.43	6.69	0.00149909	0.01312855	PRP38 family protein
TGME49_204060	-1.44	5.49	0.00154469	0.01351189	SNARE domain-containing protein
TGME49_255410	-1.21	6.98	0.00155176	0.01354675	hypothetical protein
TGME49_279410	0.99	7.09	0.00155805	0.01357884	hypothetical protein
TGME49_217520	0.98	8.97	0.00155967	0.01357884	hypothetical protein
TGME49_258050	-1.98	4.70	0.00156623	0.01359356	actin like protein ALP2a
TGME49_244600	-1.44	5.95	0.00157538	0.01363548	hypothetical protein
TGME49_222920	1.08	6.27	0.00159645	0.01379494	mbp-1 interacting protein-2a family protein
TGME49_268730	1.22	5.02	0.00160544	0.01384713	glutaredoxin-related protein
TGME49_315580	-0.97	7.08	0.00161199	0.01387136	hypothetical protein
TGME49_204110	-2.23	5.27	0.00161041	0.01387136	eIF2 kinase IF2K-C
TGME49_320190	-1.69	5.09	0.00161597	0.01388948	SAG-related sequence SRS16B
TGME49_266280	-1.16	6.56	0.00162976	0.01395939	HEAT repeat-containing protein
TGME49_280518	-1.09	7.81	0.00163751	0.0140096	hypothetical protein
TGME49_257800	-1.81	4.81	0.00167302	0.01429688	polynucleotide adenylyltransferase
TGME49_295658	-1.27	5.67	0.00167941	0.01433498	zinc finger in N-recognin protein
TGME49_216920	-1.49	5.80	0.00175009	0.0148357	mediator complex subunit MED8
TGME49_220950	1.49	9.51	0.00179107	0.01507957	hypothetical protein
TGME49_312105	-1.90	6.27	0.00178639	0.01507957	hypothetical protein
TGME49_254510	1.21	5.21	0.00179706	0.01511289	ankyrin repeat-containing protein
TGME49_261660	1.38	5.03	0.0018078	0.01516075	hypothetical protein
TGME49_242030	-1.58	5.29	0.0018089	0.01516075	hypothetical protein
TGME49_221410	-1.15	6.55	0.00181213	0.01517073	actin-like protein ALP4

TGME49_228010	-1.70	5.14	0.00182004	0.01521969	hypothetical protein
TGME49_275420	-1.19	6.14	0.00184023	0.01537121	histone lysine-specific demethylase LSD1/BHC110/KDMA1A
TGME49_312420	0.88	8.00	0.00184727	0.01541267	hypothetical protein
TGME49_281450	-1.50	5.73	0.00186418	0.01551879	cell-cycle-associated protein kinase, putative
TGME49_286790	-1.22	6.11	0.00190561	0.01582812	nuclear factor NF2
TGME49_202250	1.03	5.90	0.00191449	0.01588407	hypothetical protein
TGME49_245460	3.64	12.61	0.00192745	0.0159737	ribosomal protein RPS8
TGME49_208420	1.49	6.62	0.00194296	0.01599753	Sodium:neurotransmitter symporter family protein
TGME49_288360	1.28	9.40	0.0019414	0.01599753	tryptophanyl-tRNA synthetase (TrpRS2)
TGME49_313970	-2.25	5.18	0.00193704	0.01599753	Phytanoyl-CoA dioxygenase (PhyH) superfamily protein
TGME49_200595	-7.22	4.67	0.00194328	0.01599753	hypothetical protein
TGME49_262120	-0.97	7.22	0.00194766	0.01601575	IQ calmodulin-binding motif domain-containing protein
TGME49_203050	1.29	5.92	0.00197416	0.01621567	AP2 domain transcription factor AP2VIIa-6
TGME49_318650	0.90	8.08	0.0019926	0.01633013	transhydrogenase
TGME49_254900	0.89	6.54	0.0019947	0.01633013	proteasome subunit beta type 2, putative
TGME49_298070	-2.10	5.47	0.00200947	0.01641477	hypothetical protein
TGME49_318690	1.35	4.49	0.00201297	0.01641586	RNA recognition motif-containing protein
TGME49_202540	-0.98	7.57	0.00201404	0.01641586	3'5'-cyclic nucleotide phosphodiesterase domain-containing protein
TGME49_265390	-1.28	6.56	0.00201892	0.01643757	hypothetical protein
TGME49_311625	-1.42	5.89	0.00202961	0.01648836	WD domain, G-beta repeat-containing protein
TGME49_216510	-1.35	5.83	0.00203782	0.01651883	thioredoxin, putative
TGME49_313180	-0.87	8.51	0.00204285	0.01652345	cell-cycle-associated protein kinase PRP4, putative
TGME49_231370	1.26	5.17	0.00204928	0.01653933	phospholipase, patatin family protein
TGME49_226320	-1.07	6.35	0.00207093	0.01669591	hypothetical protein
TGME49_258826	-1.97	4.89	0.00207953	0.01673609	hypothetical protein
TGME49_290160	-1.03	7.33	0.00209174	0.01680875	sortilin, putative
TGME49_217400	-1.37	6.47	0.00210943	0.01691428	hypothetical protein
TGME49_250220	1.23	5.89	0.00211561	0.0169455	hypothetical protein
TGME49_270330	1.36	5.83	0.00213638	0.01709171	cell-cycle-associated protein kinase, putative
TGME49_304955	0.93	9.11	0.00213848	0.01709171	serine/threonine specific protein phosphatase
TGME49_236870	1.69	6.81	0.00214253	0.01709332	hypothetical protein
TGME49_301290	-1.43	5.30	0.0021433	0.01709332	hypothetical protein
TGME49_266690	-0.95	7.30	0.00214805	0.01711278	hypothetical protein
TGME49_251460	-1.36	6.07	0.00218151	0.01734208	hypothetical protein
TGME49_289140	0.87	6.35	0.00218544	0.01735469	ribosomal protein l22/l43, putative
TGME49_213480	2.09	5.16	0.00219359	0.01740079	hypothetical protein
TGME49_272370	1.06	7.49	0.002202	0.01744875	hypothetical protein
TGME49_214980	0.80	7.97	0.0022083	0.01746224	hypothetical protein
TGME49_279420	1.17	7.69	0.00222931	0.01760871	hypothetical protein
TGME49_266900	1.04	8.02	0.00223609	0.01764343	cyclin, N-terminal domain-containing protein
TGME49_255400	1.35	5.36	0.00224216	0.01767252	hypothetical protein
TGME49_318290	-1.31	5.58	0.00226138	0.01778613	hypothetical protein
TGME49_314790	-1.48	6.88	0.00226088	0.01778613	small nuclear ribonucleoprotein G, putative
TGME49_259990	-1.01	6.56	0.00231232	0.01814827	SAC3/GANP family protein
TGME49_220230	-1.27	5.76	0.00232082	0.01819574	leucine rich repeat-containing protein
TGME49_201710	1.23	5.63	0.00232775	0.01821152	WD domain, G-beta repeat-containing protein
TGME49_319740	-1.51	5.14	0.00232644	0.01821152	transporter, major facilitator family protein
TGME49_310530	-1.33	5.63	0.00236731	0.01845985	SNF2 family N-terminal domain-containing protein

TGME49_293260	1.38	7.14	0.00241078	0.01871944	ATPase/histidine kinase/DNA gyrase B/HSP90 domain-containing protein
TGME49_212310	1.04	9.06	0.00240864	0.01871944	vacuolar ATP synthetase
TGME49_215640	-1.04	6.80	0.00241288	0.01871944	zinc finger, C3HC4 type (RING finger) domain-containing protein
TGME49_225560	-1.03	8.81	0.00242612	0.01880242	hypothetical protein
TGME49_289910	0.80	7.75	0.00251343	0.01943843	hypothetical protein
TGME49_226240	1.06	5.23	0.00251961	0.01946591	bud site selection protein, putative
TGME49_313590	-1.47	5.92	0.00254953	0.01967654	hypothetical protein
TGME49_201790	-1.12	6.54	0.0025599	0.01973602	FHA domain-containing protein
TGME49_285290	1.07	6.54	0.00257695	0.01984681	hypothetical protein
TGME49_215250	-1.94	5.14	0.00259271	0.01994742	thiamin pyrophosphokinase, catalytic domain-containing protein
TGME49_232550	0.93	7.35	0.0025986	0.019972	hypothetical protein
TGME49_278530	0.76	8.32	0.00261031	0.02002046	multiprotein bridging factor type 1 family transcriptional co-activator, putative
TGME49_230490	-1.20	6.28	0.00261663	0.02004819	phosphatidylinositol-4-phosphate 5-kinase
TGME49_210682	1.85	5.24	0.00263159	0.02010047	hypothetical protein
TGME49_294790	0.85	8.88	0.00265473	0.02025634	hypothetical protein
TGME49_218930	3.26	4.53	0.00267821	0.02039348	BTB/POZ domain-containing protein
TGME49_319530	-0.93	8.07	0.00267744	0.02039348	splicing factor SF2
TGME49_247330	-0.87	6.97	0.00269904	0.02053102	hypothetical protein
TGME49_299070	0.88	6.57	0.0027028	0.0205385	pyruvate kinase PyKII
TGME49_206680	1.12	5.62	0.00270764	0.02055419	hypothetical protein
TGME49_240590	0.94	7.11	0.00272396	0.02065695	DNA-directed RNA polymerase II RPB5
TGME49_263060	1.13	7.25	0.00273752	0.02072879	Proteasome/cyclosome repeat-containing protein
TGME49_319930	-1.27	6.12	0.00275175	0.02078259	hypothetical protein
TGME49_214810	1.18	5.74	0.00276124	0.02083305	hypothetical protein
TGME49_281570	-1.42	5.86	0.00277174	0.02089097	hypothetical protein
TGME49_292950	0.99	7.04	0.00279433	0.02103984	hypothetical protein
TGME49_208590	-1.04	7.09	0.00281248	0.021155	vacuolar ATP synthase subunit 54kD, putative
TGME49_273530	-1.77	5.13	0.00287007	0.02156625	flagellar associated protein
TGME49_227330	1.13	7.01	0.00288459	0.02163852	hypothetical protein
TGME49_200450	-1.67	4.90	0.00288553	0.02163852	hypothetical protein
TGME49_253130	0.85	7.00	0.00290401	0.02175511	transporter, major facilitator family protein
TGME49_249670	0.96	7.83	0.00291232	0.0217577	cathepsin B
TGME49_235020	-0.87	7.55	0.00290943	0.0217577	COPI protein, putative
TGME49_216750	-1.10	6.58	0.00291316	0.0217577	Paf1/RNA polymerase II complex component LEO1
TGME49_250955	1.57	5.63	0.0029175	0.02176816	KRUF family protein
TGME49_229930	0.93	7.64	0.00293469	0.02183045	p25-alpha family protein
TGME49_320000	-1.35	5.47	0.00293217	0.02183045	SCY kinase (incomplete catalytic triad)
TGME49_313350	-1.21	5.79	0.00294013	0.021849	hypothetical protein
TGME49_293190	-0.97	7.22	0.00294532	0.0218656	endonuclease/exonuclease/phosphatase family protein
TGME49_240510	1.44	5.61	0.00295511	0.02188292	hypothetical protein
TGME49_259720	-1.18	6.13	0.00295651	0.02188292	hypothetical protein
TGME49_273140	-1.45	5.60	0.00295141	0.02188292	radical SAM methylthiotransferase, MiaB/RimO family protein
TGME49_231120	1.02	5.98	0.00296934	0.02195595	ribosomal protein S11, putative
TGME49_221190	1.32	5.16	0.00298089	0.02201936	mrna cleavage factor family protein, putative
TGME49_273380	1.10	5.22	0.00300883	0.0222036	ion channel protein
TGME49_218610	-1.45	5.39	0.0030137	0.02221744	ATPase (DUF699) protein
TGME49_244570	-1.74	5.07	0.00303224	0.02233187	hypothetical protein

TGME49_221590	0.87	8.63	0.00307314	0.02261063	dual specificity phosphatase, catalytic domain-containing protein
TGME49_263840	-1.10	6.12	0.00309654	0.0227376	hypothetical protein
TGME49_233380	1.13	9.74	0.00310502	0.02277731	hypothetical protein
TGME49_269430	-1.43	5.52	0.00311242	0.02280903	polyprenyl synthetase superfamily protein
TGME49_319570	-0.96	6.68	0.00311705	0.0228204	WD domain, G-beta repeat-containing protein
TGME49_268380	2.05	6.24	0.0031238	0.02284724	RNA recognition motif-containing protein
TGME49_310390	1.34	6.89	0.00314802	0.02293382	hypothetical protein
TGME49_231100	0.90	6.73	0.00314681	0.02293382	hypothetical protein
TGME49_269940	-1.72	4.83	0.00314198	0.02293382	zinc finger motif, C2HC5-type protein
TGME49_300060	1.00	6.85	0.00317782	0.02310549	signal peptidase subunit protein
TGME49_285490	-1.37	5.79	0.0031751	0.02310549	helix-hairpin-helix motif domain-containing protein
TGME49_202300	-1.24	5.80	0.00318859	0.02311572	inosine triphosphate pyrophosphatase, putative
TGME49_258490	-1.42	5.25	0.00318455	0.02311572	hypothetical protein
TGME49_201200	-1.97	4.87	0.00318636	0.02311572	zinc finger (CCCH type) motif-containing protein
TGME49_289600	0.79	8.48	0.00320105	0.02318333	heat shock protein HSP29
TGME49_248630	-1.11	6.81	0.00320464	0.0231867	actin-related protein ARP1
TGME49_221440	-1.36	5.23	0.00320944	0.02319876	RPGR, putative
TGME49_242380	-0.94	7.53	0.00323036	0.02328185	fatty acid elongase
TGME49_250690	-0.83	8.66	0.00323667	0.02330465	zinc finger (CCCH type) motif-containing protein
TGME49_244530	1.03	8.45	0.00324136	0.02331577	hypothetical protein
TGME49_315720	-1.23	5.60	0.00325853	0.02339387	Smg-4/UPF3 family protein
TGME49_313385	1.23	5.33	0.00326791	0.02343846	hypothetical protein
TGME49_224070	-1.32	5.80	0.00327493	0.02346611	hypothetical protein
TGME49_293900	1.04	7.90	0.00329082	0.02354945	sporozoite protein with an altered thrombospondin repeat SPATR
TGME49_247370	-1.52	5.12	0.00329292	0.02354945	hypothetical protein
TGME49_263610	-1.39	6.26	0.00329962	0.02357461	hypothetical protein
TGME49_216490	-1.65	5.01	0.00331811	0.02368392	hypothetical protein
TGME49_308580	-1.44	5.77	0.00332805	0.02373196	Lon protease family protein
TGME49_202240	-2.25	4.63	0.00334989	0.02386478	RAP domain-containing protein
TGME49_246140	-1.42	5.11	0.00336282	0.02393388	hypothetical protein
TGME49_294980	1.48	4.97	0.00337634	0.024007	hypothetical protein
TGME49_268220	4.47	4.65	0.00338535	0.024048	hypothetical protein
TGME49_316700	1.40	4.86	0.00341195	0.0242137	uridine kinase
TGME49_277840	0.86	7.26	0.00341752	0.02423002	Ras family protein
TGME49_214490	-1.37	5.48	0.00342106	0.02423193	peptidase M16 inactive domain-containing protein
TGME49_233450	1.51	7.79	0.00342751	0.02425029	SAG-related sequence SRS29A
TGME49_229470	-1.06	6.59	0.00345759	0.0244207	hypothetical protein
TGME49_246080	-0.95	6.89	0.00346916	0.02447902	NAD dependent epimerase/dehydratase family protein
TGME49_227580	-1.02	7.72	0.0034788	0.02452369	transmembrane amino acid transporter protein
TGME49_253320	1.18	5.24	0.00354009	0.02493208	hypothetical protein
TGME49_265790	0.83	8.49	0.00356856	0.02508488	hypothetical protein
TGME49_246120	0.98	5.80	0.003572	0.02508527	tetratricopeptide repeat-containing protein
TGME49_218570	0.92	6.13	0.00357882	0.02510935	Nin one binding (NOB1) Zn-ribbon family protein
TGME49_291690	-1.27	5.50	0.00360916	0.02527435	hypothetical protein
TGME49_237160	-1.22	5.78	0.00361693	0.02530484	hypothetical protein
TGME49_318525	-1.15	6.53	0.00363645	0.02541738	hypothetical protein
TGME49_297510	-1.54	5.94	0.00366466	0.02559046	hypothetical protein
TGME49_268170	-1.58	5.31	0.00369459	0.025758	hypothetical protein
TGME49_319900	-1.12	6.85	0.00370348	0.02578859	hypothetical protein
TGME49_267580	1.62	5.90	0.00375445	0.026119	cyclin2 related protein

TGME49_281910	1.21	5.48	0.00377925	0.02626688	hypothetical protein
TGME49_297230	-1.25	6.27	0.00380929	0.02640288	Vps53 family, N-terminal protein
TGME49_213800	-0.89	8.01	0.00382702	0.02648638	protein phosphatase 2b regulatory subunit, putative
TGME49_313200	-1.66	5.25	0.00383409	0.02648638	leucine rich repeat-containing protein
TGME49_278815	-0.92	6.99	0.00384281	0.02650964	hypothetical protein
TGME49_225470	0.98	5.79	0.00386848	0.02661238	peptide methionine sulfoxide reductase
TGME49_215895	0.96	6.10	0.0038721	0.02661262	AP2 domain-containing protein
TGME49_306310	-0.96	6.30	0.00390059	0.02678359	RecF/RecN/SMC N terminal domain-containing protein
TGME49_255900	0.75	8.26	0.00392374	0.0268728	Bax inhibitor-1, putative
TGME49_218600	-1.24	5.47	0.00392447	0.0268728	RNA recognition motif-containing protein
TGME49_309380	-1.21	6.32	0.00393995	0.0269539	Nuf2
TGME49_200430	-1.64	5.09	0.00396273	0.02705976	cytidine and deoxycytidylate deaminase zinc-binding region domain-containing protein
TGME49_237410	-1.14	6.56	0.00397944	0.02714887	protein phosphatase 2C domain-containing protein
TGME49_210360	1.08	5.56	0.00400217	0.02719745	DEAD (Asp-Glu-Ala-Asp) box polypeptide 41 family protein
TGME49_216435	-1.06	6.22	0.00399763	0.02719745	hypothetical protein
TGME49_289180	-1.13	5.71	0.00400264	0.02719745	thioredoxin family redox-active protein, putative
TGME49_301380	-1.44	5.62	0.00400492	0.02719745	elongation factor Tu GTP binding domain-containing protein
TGME49_213900	-0.88	7.16	0.00402708	0.02732295	regulator of chromosome condensation RCC1
TGME49_308075	-0.99	6.78	0.00404149	0.0273956	hypothetical protein
TGME49_207900	-1.09	6.22	0.00407533	0.02757452	transcription initiation factor TFIIIB
TGME49_268210	-1.35	5.24	0.00408008	0.02758146	AGC kinase
TGME49_268980	0.81	8.08	0.00409304	0.02764388	hypothetical protein
TGME49_266070	1.28	10.10	0.00413331	0.02789043	ribosomal protein RPL31
TGME49_201220	-1.39	5.67	0.00414379	0.02793572	zinc finger protein
TGME49_205700	1.33	10.39	0.00417889	0.02812119	cyclophilin precursor
TGME49_224480	-1.03	6.40	0.00417786	0.02812119	cell-cycle-associated protein kinase CLK, putative
TGME49_204055	-1.67	5.00	0.00419787	0.02822325	hypothetical protein
TGME49_223540	-1.08	6.49	0.00426374	0.02861246	importin-beta N-terminal domain-containing protein
TGME49_319710	-1.20	6.20	0.00426735	0.02861246	kinesin motor domain-containing protein
TGME49_228980	-1.55	4.98	0.00427457	0.02863501	hypothetical protein
TGME49_267450	-1.25	6.10	0.00431001	0.02884628	alpha-tubulin suppressor protein
TGME49_235398	-1.20	5.54	0.00432251	0.02887783	hypothetical protein
TGME49_209930	-1.40	5.64	0.00431873	0.02887783	hypothetical protein
TGME49_236010	0.76	8.24	0.00433761	0.0289265	prenylcysteine oxidase
TGME49_236630	0.79	7.95	0.00435576	0.02902143	hypothetical protein
TGME49_218520	1.39	10.64	0.00437683	0.02910944	microneme protein MIC6
TGME49_275610	-1.03	8.30	0.00437665	0.02910944	protein kinase, other
TGME49_301130	-1.69	5.52	0.00438957	0.02916799	hypothetical protein
TGME49_225800	0.91	6.01	0.0044123	0.02922495	iron-sulfur assembly ATPase
TGME49_278450	0.78	7.85	0.00441311	0.02922495	hypothetical protein
TGME49_222120	-1.52	5.28	0.00440753	0.02922495	hypothetical protein
TGME49_235540	0.92	8.28	0.00441878	0.02922549	eukaryotic initiation factor-2 beta, putative
TGME49_263595	-1.37	5.60	0.00442189	0.02922549	RNA-binding protein
TGME49_225250	0.88	5.81	0.00446629	0.02937812	LSU ribosomal protein L14P, putative
TGME49_235880	0.82	7.65	0.00445284	0.02937812	brain protein 44 family protein
TGME49_265840	-1.01	6.56	0.00446877	0.02937812	hypothetical protein
TGME49_213460	-1.05	6.67	0.00446337	0.02937812	hypothetical protein

TGME49_291120	-1.11	5.77	0.00446403	0.02937812	trafficking protein mon1 subfamily protein
TGME49_246760	-2.02	4.72	0.00445435	0.02937812	hypothetical protein
TGME49_213790	-1.09	6.02	0.00447285	0.02937888	hypothetical protein
TGME49_253300	1.61	4.67	0.00448013	0.02940063	hypothetical protein
TGME49_215400	-1.61	5.40	0.00448924	0.02943429	RNA recognition motif-containing protein
TGME49_250950	1.30	5.22	0.00451669	0.029562	KRUF family protein
TGME49_262910	1.44	4.81	0.00458498	0.02995004	NADH-cytochrome b5 reductase 1, putative
TGME49_224310	-1.12	6.08	0.00458593	0.02995004	DHHC zinc finger domain-containing protein
TGME49_313480	-2.19	4.81	0.00458811	0.02995004	hypothetical protein
TGME49_285840	-1.13	5.72	0.00461352	0.03008943	RAP domain-containing protein
TGME49_257530	1.55	11.03	0.00462602	0.03014443	transporter, major facilitator family protein
TGME49_232630	0.73	8.16	0.00463952	0.03019404	hypothetical protein
TGME49_232050	-0.93	7.68	0.00464179	0.03019404	DnaJ domain-containing protein
TGME49_285510	0.78	7.41	0.00464631	0.03019693	hypothetical protein
TGME49_269410	1.18	5.26	0.00465321	0.03020407	hypothetical protein
TGME49_272030	-1.05	6.10	0.00465556	0.03020407	kelch repeat-containing protein
TGME49_231950	0.82	7.69	0.00466209	0.03021998	hypothetical protein
TGME49_254620	1.10	9.88	0.00469908	0.03040654	ribosomal protein RPL39
TGME49_210255	0.81	6.96	0.00472189	0.030479	hypothetical protein
TGME49_205670	0.79	7.31	0.00472262	0.030479	SF-assemblin/beta giardin protein
TGME49_248570	-1.51	5.00	0.00471588	0.030479	hypothetical protein
TGME49_320650	-1.43	4.91	0.00473721	0.03054653	ankyrin repeat-containing protein
TGME49_248690	-1.32	5.60	0.00474817	0.03059059	hypothetical protein
TGME49_221580	-0.96	6.88	0.00480193	0.03088322	ribosomal RNA large subunit methyltransferase J protein
TGME49_312260	-1.13	5.95	0.0047986	0.03088322	hypothetical protein
TGME49_249010	0.83	7.09	0.00484612	0.03111343	hypothetical protein
TGME49_237840	-1.28	5.50	0.00486648	0.03119008	hypothetical protein
TGME49_239087	-1.40	5.24	0.00491434	0.03146963	hypothetical protein
TGME49_253740	-1.12	5.77	0.00492475	0.03150903	hypothetical protein
TGME49_270140	-1.27	5.92	0.00494228	0.0315186	splicing factor DIM1, putative
TGME49_211330	-1.87	4.59	0.00493663	0.0315186	methionine aminopeptidase
TGME49_233838	-1.99	4.53	0.00493635	0.0315186	PET112 family, C terminal region domain-containing protein
TGME49_261460	-1.01	7.09	0.00499236	0.03177696	transcriptional elongation factor FACT80
TGME49_226270	0.91	5.99	0.00499846	0.03178847	hypothetical protein
TGME49_246970	-1.31	5.30	0.00508832	0.03233221	3'-5' exonuclease domain-containing protein
TGME49_237500	0.84	7.29	0.0050997	0.03237677	protein phosphatase 2C domain-containing protein
TGME49_268900	-1.22	5.76	0.00515323	0.03268858	dense granular protein GRA10
TGME49_310230	-1.13	6.60	0.00516003	0.0326969	hypothetical protein
TGME49_244870	-1.10	5.84	0.00517768	0.03275955	quinone oxidoreductase, putative
TGME49_298990	0.89	5.96	0.00522945	0.03300253	ferredoxin NADP+ oxidoreductase FNR
TGME49_299270	1.01	5.30	0.00524668	0.03308314	hypothetical protein
TGME49_226830	-0.98	8.44	0.00526712	0.03316934	DnaK family protein
TGME49_268580	-1.03	6.39	0.00527379	0.03316934	hypothetical protein
TGME49_253560	0.99	5.72	0.0052825	0.03319594	hypothetical protein
TGME49_229340	-1.63	4.98	0.00531199	0.03335302	hypothetical protein
TGME49_258180	-1.49	4.83	0.00531657	0.03335351	hypothetical protein
TGME49_244220	-1.97	4.51	0.00533309	0.03342879	hypothetical protein
TGME49_276170	0.73	8.59	0.00534184	0.03345537	phosphatidylinositol 3- and 4-kinase
TGME49_202420	1.43	5.50	0.00536435	0.03355536	hypothetical protein
TGME49_235490	-0.90	6.76	0.00536686	0.03355536	hypothetical protein
TGME49_208030	1.24	10.29	0.00538211	0.03362228	microneme protein MIC4

TGME49_312500	-0.98	6.61	0.00538903	0.03363719	hypothetical protein
TGME49_273770	0.86	7.75	0.00546748	0.03399171	hypothetical protein
TGME49_242260	-1.23	5.84	0.00546877	0.03399171	hypothetical protein
TGME49_245428	1.44	5.35	0.00547464	0.03399965	hypothetical protein
TGME49_213388	-1.41	5.26	0.00550065	0.03413259	hypothetical protein
TGME49_311250	-1.11	5.97	0.0055207	0.03421125	hypothetical protein
TGME49_273400	-1.55	5.01	0.00552718	0.03421125	hypothetical protein
TGME49_215990	-1.45	5.28	0.00555031	0.03432574	helicase, putative
TGME49_217820	-0.93	7.80	0.0055764	0.03443246	PCI domain-containing protein
TGME49_294060	0.97	6.68	0.00558967	0.03447324	hypothetical protein
TGME49_318580	-0.96	6.23	0.00559568	0.03447324	glucosephosphate-mutase GPM2
TGME49_219070	-0.99	6.86	0.00561056	0.03452542	cyclic nucleotide-binding domain-containing protein
TGME49_213600	1.73	4.46	0.00561528	0.03452582	hypothetical protein
TGME49_206600	-1.24	5.46	0.00562796	0.03457509	sigma-70, region 3 protein
TGME49_266850	1.02	5.79	0.0056452	0.0346414	3-demethylubiquinone-9 3-O-methyltransferase
TGME49_239600	1.33	5.83	0.00567069	0.03472246	rhoptry kinase family protein ROP23 (incomplete catalytic triad)
TGME49_315590	-1.24	5.72	0.00566621	0.03472246	macro domain-containing protein
TGME49_211410	-1.26	6.19	0.00572769	0.03504251	translation initiation factor sui1 protein
TGME49_219820	1.02	7.81	0.00574664	0.03510048	polyubiquitin UbC, putative
TGME49_216760	-1.38	5.08	0.00577326	0.03523399	RNA pseudouridine synthase superfamily protein
TGME49_221200	0.86	6.67	0.0057946	0.03533513	CW-type Zinc Finger protein
TGME49_206415	-0.99	6.32	0.00580537	0.03537169	myosin K
TGME49_213820	0.89	6.73	0.00581663	0.03541119	hypothetical protein
TGME49_232640	1.17	5.17	0.00585027	0.03549933	RNA 2'-phosphotransferase, Tpt1/KptA family protein
TGME49_286720	0.86	6.76	0.00584587	0.03549933	heat shock protein HSP28
TGME49_243340	-1.43	5.11	0.00584731	0.03549933	atypical MEK-related kinase (incomplete catalytic triad)
TGME49_270450	-1.71	4.65	0.00584977	0.03549933	MCM2/3/5 family protein
TGME49_254290	1.01	5.58	0.00589265	0.03572722	hypothetical protein
TGME49_236240	-0.96	6.17	0.00594119	0.03597696	Tyrosine kinase-like (TKL) protein
TGME49_247680	-1.68	5.02	0.00594356	0.03597696	hypothetical protein
TGME49_258105	-1.98	4.46	0.00595484	0.03601586	hypothetical protein
TGME49_313760	0.81	7.74	0.0059722	0.0360877	hypothetical protein
TGME49_246800	-1.04	6.38	0.00598084	0.0360877	acylaminoacyl-peptidase, putative
TGME49_262740	-1.64	4.58	0.00598133	0.0360877	hypothetical protein
TGME49_237550	1.49	5.18	0.00602042	0.03622681	hypothetical protein
TGME49_298970	0.88	6.90	0.00602323	0.03622681	LSM3, U6 small nuclear RNA associated isoform 2 family protein
TGME49_313120	-1.89	4.63	0.00610841	0.03670498	DNA-directed RNA polymerase, alpha subunit
TGME49_229490	-0.97	6.52	0.00612318	0.03676386	tetratricopeptide repeat-containing protein
TGME49_219170	-0.87	6.82	0.00613032	0.03677695	hypothetical protein
TGME49_238170	1.16	5.73	0.00618323	0.03703438	hypothetical protein
TGME49_201880	0.72	8.04	0.006205	0.03713475	hypothetical protein
TGME49_246090	-1.11	6.69	0.00622272	0.03715077	hypothetical protein
TGME49_278540	0.93	7.28	0.00626267	0.03732916	hypothetical protein
TGME49_236050	-2.92	4.86	0.00630852	0.03757223	fructose-bisphosphate aldolase, putative
TGME49_307610	-0.87	6.49	0.00633675	0.03771	elongation factor TS, putative
TGME49_249810	-1.68	4.81	0.00644532	0.03820271	activating signal cointegrator 1 complex subunit 3, putative
TGME49_299980	0.75	7.27	0.00647818	0.03833615	hypothetical protein
TGME49_223590	0.69	8.22	0.00647488	0.03833615	proteasome subunit

TGME49_213910	-1.12	5.99	0.00651287	0.03851068	hypothetical protein
TGME49_289620	0.81	8.43	0.00654068	0.03861344	cathepsin CPC1
TGME49_277770	-1.42	5.35	0.00656808	0.03874434	hypothetical protein
TGME49_305950	1.09	5.38	0.0065792	0.03877906	tetratricopeptide repeat-containing protein
TGME49_215530	0.97	9.69	0.00658805	0.03880034	hypothetical protein
TGME49_231140	2.39	11.22	0.00662415	0.03898197	ribosomal protein RPS25
TGME49_238230	0.85	5.98	0.00663918	0.03902328	Ser/Thr phosphatase family protein
TGME49_230410	-1.11	8.15	0.00666705	0.03907925	peroxiredoxin PRX3
TGME49_229620	-1.21	5.36	0.00665985	0.03907925	hypothetical protein
TGME49_315820	-1.15	5.93	0.00669693	0.03922335	hypothetical protein
TGME49_304520	-0.94	6.73	0.00671794	0.03927838	hypothetical protein
TGME49_309580	-1.05	6.11	0.00671543	0.03927838	transporter, major facilitator family protein
TGME49_280710	-1.05	8.04	0.00672223	0.03927838	20S proteasome subunit beta 7, putative
TGME49_227335	1.44	5.11	0.00673195	0.03928573	hypothetical protein
TGME49_209020	-1.85	4.48	0.00673409	0.03928573	hypothetical protein
TGME49_216650	0.72	8.30	0.00678833	0.0395399	S15 sporozoite-expressed protein
TGME49_207420	0.81	6.43	0.00680087	0.03958182	hypothetical protein
TGME49_305120	1.05	6.89	0.0068113	0.03961138	transporter, solute:sodium symporter (SSS) family protein
TGME49_235920	-1.00	6.30	0.00681993	0.03963047	dynein, axonemal, heavy chain 2 family protein
TGME49_228360	0.88	7.28	0.0068272	0.03964163	peptidyl-prolyl isomerase FKBP12, putative
TGME49_220330	-1.48	4.86	0.00685146	0.03972024	hypothetical protein
TGME49_259630	0.85	8.88	0.00689029	0.03991412	hypothetical protein
TGME49_227060	-1.03	5.79	0.00692767	0.04006798	hypothetical protein
TGME49_223410	0.85	7.90	0.00693929	0.0401039	eukaryotic initiation factor-4E, putative
TGME49_321650	-0.79	7.42	0.00697864	0.04029986	hypothetical protein
TGME49_271030	1.47	6.63	0.00700483	0.0403555	AP2 domain transcription factor AP2VIII-6
TGME49_298050	-1.10	5.70	0.007	0.0403555	hypothetical protein
TGME49_221330	-1.02	6.74	0.00708536	0.04069414	DNA gyrase/topoisomerase IV, A subunit domain-containing protein
TGME49_220510	1.22	6.04	0.00710918	0.04079931	hypothetical protein
TGME49_233830	-1.85	4.60	0.00713276	0.04090294	hypothetical protein
TGME49_310470	0.80	8.64	0.00718156	0.04115093	cytochrome C oxidase subunit IIb, putative
TGME49_233480	1.85	12.19	0.00724072	0.04145786	SAG-related sequence SRS29C
TGME49_312670	-1.43	4.82	0.00727682	0.0416324	hypothetical protein
TGME49_258480	-0.96	6.60	0.00739188	0.04225807	hypothetical protein
TGME49_212130	-1.06	6.44	0.00743415	0.04246695	phospholipase, patatin family protein
TGME49_289680	0.82	7.08	0.00746259	0.04254956	Ras-related protein Rab11
TGME49_212200	-1.40	5.65	0.00748047	0.04260013	hypothetical protein
TGME49_214840	0.81	9.20	0.00752872	0.0427763	AP2 domain transcription factor AP2X-7
TGME49_214150	-1.32	6.36	0.00762878	0.0432453	mitochondrial inner membrane translocase subunit TIM17, putative
TGME49_214780	-1.33	5.82	0.00763779	0.04326329	hydrolase, NUDIX family protein
TGME49_207800	0.92	5.84	0.00767059	0.04336372	hypothetical protein
TGME49_224520	-0.76	7.89	0.00766751	0.04336372	alveolin domain containing intermediate filament IMC8
TGME49_259950	0.86	6.67	0.00770039	0.04346	carbonate dehydratase, eukaryotic-type domain-containing protein
TGME49_203700	0.94	6.22	0.00776428	0.04371242	SFT2 family protein
TGME49_209570	0.97	5.47	0.0077864	0.04377124	suppressor of mitotic defects protein
TGME49_309090	-1.08	5.89	0.00778654	0.04377124	hypothetical protein
TGME49_225160	0.81	7.50	0.00781852	0.0438844	hypothetical protein
TGME49_293760	0.71	7.69	0.0078136	0.0438844	EF hand domain-containing protein

TGME49_270770	-0.99	6.32	0.00787269	0.04415279	PWI domain-containing protein
TGME49_228110	-1.12	6.14	0.00787825	0.04415279	hypothetical protein
TGME49_265650	-1.18	5.56	0.0079293	0.0444053	protein phosphatase 2C domain-containing protein
TGME49_288580	1.40	4.44	0.00794945	0.04444417	RNA methylase, putative
TGME49_268280	-0.76	7.44	0.00794844	0.04444417	'chromo' (CHRromatin Organization MOdifier) domain-containing protein
TGME49_310870	-1.03	6.08	0.00796024	0.04444417	integral membrane protein, putative
TGME49_320480	-0.83	7.01	0.008016	0.04465454	Rab11b
TGME49_241150	-1.02	6.51	0.00800406	0.04465454	hypothetical protein
TGME49_218260	1.31	10.76	0.00805397	0.04466455	histone H3.3
TGME49_240500	0.99	5.76	0.00804494	0.04466455	hypothetical protein
TGME49_295410	-0.98	6.10	0.00805265	0.04466455	transcription initiation factor TFIID complex subunit TAF6
TGME49_315660	-1.25	5.51	0.00802696	0.04466455	hypothetical protein
TGME49_290330	-1.39	5.28	0.00803448	0.04466455	chloride transporter, chloride channel (ClC) family protein
TGME49_269130	-1.63	4.97	0.00804621	0.04466455	hypothetical protein
TGME49_310730	-0.94	6.24	0.00806559	0.04469554	hypothetical protein
TGME49_293180	0.77	8.77	0.0080825	0.04471785	NADP-specific glutamate dehydrogenase
TGME49_297710	-1.42	6.65	0.00808788	0.04471785	hypothetical protein
TGME49_272400	-1.45	5.54	0.0080773	0.04471785	casein kinase ii regulatory subunit protein
TGME49_232390	-1.67	5.02	0.00809376	0.04471785	18S rRNA biogenesis protein RCL1 protein
TGME49_267680	-1.03	6.31	0.00811196	0.04478053	microneme protein MIC12
TGME49_249560	-1.17	5.43	0.00811719	0.04478053	DNA-directed RNA polymerase alpha chain rpoA
TGME49_253540	1.00	5.61	0.00815873	0.04495053	hypothetical protein
TGME49_220460	-1.73	4.82	0.00816014	0.04495053	SNF7 family protein
TGME49_216460	0.89	6.86	0.00817029	0.04497301	hypothetical protein
TGME49_235500	-1.48	5.80	0.00820078	0.04510734	hypothetical protein
TGME49_253730	0.96	6.80	0.00822704	0.04520078	importin-beta N-terminal domain-containing protein
TGME49_294220	0.93	6.10	0.00822997	0.04520078	hypothetical protein
TGME49_232130	-0.77	8.66	0.00823656	0.04520345	hypothetical protein
TGME49_209080	2.34	4.87	0.00826631	0.04533313	transport protein particle (trapp) component, bet3 protein
TGME49_252390	0.73	8.34	0.00835529	0.04571849	hypothetical protein
TGME49_251640	-1.13	5.81	0.00836126	0.04571849	ubiquitin-conjugating enzyme subfamily protein
TGME49_209200	0.83	7.04	0.00837174	0.04574205	hypothetical protein
TGME49_321170	1.06	4.77	0.00844552	0.04611117	Toxoplasma gondii family C protein
TGME49_225060	-0.95	6.75	0.00846566	0.04615312	nucleoredoxin family protein
TGME49_306610	-1.45	4.93	0.00846097	0.04615312	3' exoribonuclease family, domain 1 domain-containing protein
TGME49_265410	1.20	5.34	0.00851115	0.04633291	G-protein beta WD-40 repeat containing protein
TGME49_312950	0.82	6.41	0.00850989	0.04633291	hypothetical protein
TGME49_295125	1.05	10.03	0.00853138	0.04640896	rhoptry protein ROP4
TGME49_220350	-1.05	6.07	0.0086097	0.0467663	tRNA ligases class II (D, K and N) domain-containing protein
TGME49_289780	0.98	5.49	0.00863965	0.04689465	ATP-dependent hsl protease ATP-binding subunit hslU, putative
TGME49_289110	-1.75	4.62	0.0086863	0.04707886	hypothetical protein
TGME49_223530	1.04	6.77	0.00870489	0.04711066	hypothetical protein
TGME49_263130	0.88	7.88	0.00873015	0.04721291	citrate synthase, putative
TGME49_295050	-0.97	6.08	0.00874009	0.0472322	tRNA ligase class II core domain (G, H, P, S and T) domain-containing protein

TGME49_224350	-0.82	8.27	0.00884632	0.04777143	aminopeptidase N, putative
TGME49_314660	-0.96	6.93	0.00892555	0.04812925	TPRX1 protein
TGME49_230220	-0.92	6.18	0.00895119	0.04819724	hypothetical protein
TGME49_231750	-1.19	5.72	0.00901438	0.04850221	hypothetical protein
TGME49_291630	-1.59	5.63	0.00907342	0.04874905	hypothetical protein
TGME49_270690	-0.96	6.29	0.00910179	0.04886605	arginyl-tRNA synthetase
TGME49_282000	1.40	6.18	0.00913145	0.04888363	hypothetical protein
TGME49_254090	0.96	6.12	0.009119	0.04888363	hypothetical protein
TGME49_236030	-1.72	4.52	0.00912723	0.04888363	hypothetical protein
TGME49_247960	1.49	5.74	0.00914671	0.04892996	hypothetical protein
TGME49_260670	-1.26	5.75	0.0091724	0.04903199	centrin, putative
TGME49_283860	-1.48	5.12	0.00918716	0.0490401	leucine rich repeat-containing protein
TGME49_272010	-1.23	6.93	0.00929363	0.04957271	Gar1 protein RNA binding region protein
TGME49_301340	-0.84	6.38	0.00931391	0.04964516	DnaJ domain-containing protein
TGME49_254280	1.24	4.76	0.00935207	0.04977693	DNA-directed RNA polymerase III RPC9

Supplementary Table S5C: Differencial expression analysis of T. gondii genes from MM6 cells infected with T. gondii EGS strain for 18 hours copmared to T. gondii gene expression from MM6 cells infected with VEG strain for 18 hours.

gene ID	logFC (EGS/VEG)	logCPM	PValue	FDR	Product Name
TGME49_205250	7.91	9.50	5.79E-55	4.29E-51	rhoptry protein ROP18
TGME49_322200	8.72	6.25	1.82E-51	6.75E-48	apocytochrome b, putative
TGME49_232955	6.65	8.96	9.66E-50	2.39E-46	hypothetical protein
TGME49_323400	8.19	9.09	1.19E-48	2.21E-45	cytochrome c oxidase subunit iii subfamily protein
TGME49_214080	7.75	8.54	2.19E-44	3.25E-41	toxofilin
TGME49_206550	6.27	5.45	1.07E-40	1.32E-37	hypothetical protein
TGME49_280570	5.07	7.23	1.14E-32	1.20E-29	SAG-related sequence SRS35A
TGME49_330000	7.63	9.81	3.49E-25	3.23E-22	cytochrome b
TGME49_252070	6.63	4.72	4.88E-25	4.02E-22	KRUF family protein
TGME49_255060	7.02	9.39	8.13E-25	6.02E-22	cytochrome b(N-terminal)/b6/petB subfamily protein
TGME49_259020	6.15	6.32	4.67E-24	3.15E-21	bradyzoite antigen BAG1
TGME49_237130	7.60	9.77	6.84E-24	4.22E-21	cytochrome b, putative
TGME49_293790	4.52	5.45	4.38E-23	2.49E-20	hypothetical protein
TGME49_291040	4.68	6.32	3.93E-21	2.08E-18	lactate dehydrogenase LDH2
TGME49_313440	4.65	7.70	1.15E-19	5.66E-17	hypothetical protein
TGME49_301222	2.73	6.51	2.67E-18	1.23E-15	DNA repair protein Rad4 domain-containing protein
TGME49_247340	4.66	5.11	8.22E-18	3.58E-15	hypothetical protein
TGME49_322000	-3.55	6.91	2.48E-17	1.02E-14	myosin-light-chain kinase
TGME49_252065	3.49	5.35	3.68E-16	1.43E-13	KRUF family protein
TGME49_207210	4.66	6.03	6.49E-16	2.41E-13	hypothetical protein
TGME49_302055	8.51	5.01	1.97E-15	6.94E-13	ribosomal protein RPS12
TGME49_223660	2.39	7.24	5.55E-15	1.87E-12	50S ribosomal protein L4, putative
TGME49_252640	5.49	4.57	8.52E-15	2.74E-12	P-type ATPase PMA1
TGME49_254030	2.13	8.07	1.09E-13	3.36E-11	zinc finger CDGSH-type domain-containing protein
TGME49_294400	-3.73	6.97	6.11E-13	1.81E-10	hypothetical protein
TGME49_260430	4.09	4.69	7.33E-13	2.09E-10	hypothetical protein
TGME49_209755	3.84	5.73	5.11E-12	1.40E-09	hypothetical protein
TGME49_242240	-3.68	5.94	1.20E-11	3.06E-09	rhoptry kinase family protein ROP19A
TGME49_245432	2.70	7.24	1.77E-11	4.36E-09	hypothetical protein
TGME49_275860	2.17	9.13	3.12E-11	7.46E-09	hypothetical protein
TGME49_235630	1.89	6.97	4.58E-11	1.06E-08	hypothetical protein
TGME49_286928	-3.90	6.04	4.91E-11	1.10E-08	hypothetical protein
TGME49_236670	2.81	5.44	6.62E-11	1.44E-08	hypothetical protein
TGME49_315802	-9.71	4.80	7.76E-11	1.64E-08	hypothetical protein
TGME49_221840	4.30	4.58	1.34E-10	2.76E-08	hypothetical protein
TGME49_270580	3.82	4.80	2.67E-10	5.34E-08	HECT-domain (ubiquitin-transferase) domain-containing protein
TGME49_252390	1.81	8.34	3.10E-10	6.04E-08	hypothetical protein
TGME49_305460	1.90	7.39	4.47E-10	8.42E-08	methionine aminopeptidase 2, putative
TGME49_253690	1.79	8.81	4.54E-10	8.42E-08	hypothetical protein
TGME49_233925	10.59	11.32	4.81E-10	8.69E-08	hypothetical protein
TGME49_280580	3.89	5.41	5.79E-10	1.02E-07	SAG-related sequence SRS35B
TGME49_258720	1.99	6.22	8.20E-10	1.41E-07	Ubiquitin family protein, putative
TGME49_301250	8.01	14.41	1.05E-09	1.76E-07	hypothetical protein
TGME49_213280	1.97	9.22	1.14E-09	1.88E-07	SAG-related sequence SRS25
TGME49_207960	-2.73	6.09	1.51E-09	2.43E-07	hypothetical protein
TGME49_217951	4.96	6.01	1.70E-09	2.63E-07	hypothetical protein

TGME49_308093	1.75	8.24	2.96E-09	4.48E-07	rhoptry kinase family protein (incomplete catalytic triad)
TGME49_275870	2.70	5.25	3.50E-09	5.19E-07	tubulin/FtsZ family, GTPase domain-containing protein
TGME49_228160	1.84	7.00	3.84E-09	5.48E-07	acid phosphatase
TGME49_242110	-3.56	5.29	4.89E-09	6.84E-07	rhoptry kinase family protein ROP38
TGME49_225555	1.58	8.72	5.93E-09	7.99E-07	hypothetical protein
TGME49_209985	2.85	5.16	6.12E-09	8.09E-07	cAMP-dependent protein kinase
TGME49_251180	2.36	5.76	6.26E-09	8.14E-07	KRUF family protein
TGME49_273870	-1.97	7.40	6.39E-09	8.17E-07	SWI2/SNF2 ISWI-like (AT hook)
TGME49_299030	1.79	7.31	8.88E-09	1.10E-06	RNA recognition motif 2 protein
TGME49_320180	4.38	4.76	9.27E-09	1.13E-06	SAG-related sequence SRS16C
TGME49_208440	1.59	8.08	1.09E-08	1.30E-06	hypothetical protein
TGME49_320190	7.35	5.09	1.13E-08	1.32E-06	SAG-related sequence SRS16B
TGME49_321360	1.55	7.60	1.14E-08	1.32E-06	clustered-asparagine-rich protein
TGME49_254770	1.67	6.49	1.16E-08	1.33E-06	Ser/Thr phosphatase family protein
TGME49_252220	1.54	7.62	1.23E-08	1.38E-06	tetratricopeptide repeat domain containing protein
TGME49_247530	1.67	8.79	2.88E-08	3.14E-06	hypothetical protein
TGME49_315885	6.02	9.43	3.27E-08	3.52E-06	glycosyltransferase, putative
TGME49_299080	1.66	6.87	3.84E-08	4.07E-06	VTC domain-containing protein
TGME49_252500	1.53	7.79	5.66E-08	5.91E-06	polo kinase
TGME49_319308	-3.01	5.73	7.18E-08	7.39E-06	hypothetical protein
TGME49_252360	1.78	9.12	7.87E-08	7.98E-06	rhoptry kinase family protein ROP24 (incomplete catalytic triad)
TGME49_217530	2.37	4.70	9.17E-08	9.06E-06	hypothetical protein
TGME49_202020	2.69	7.60	9.42E-08	9.18E-06	DnAK-TPR
TGME49_313100	-2.94	5.89	1.19E-07	1.14E-05	signal recognition particle SRP54 protein
TGME49_254840	-2.21	5.76	1.27E-07	1.21E-05	tetratricopeptide repeat-containing protein
TGME49_222040	-3.28	5.45	1.30E-07	1.22E-05	Ran-interacting Mog1 protein
TGME49_271935	2.02	6.37	1.43E-07	1.32E-05	hypothetical protein
TGME49_272410	3.71	8.19	1.56E-07	1.42E-05	phosphogluconate dehydrogenase (decarboxylating), NAD binding domain-containing protein
TGME49_275640	1.67	6.96	1.57E-07	1.42E-05	hypothetical protein
TGME49_255635	-5.53	5.32	1.99E-07	1.78E-05	hypothetical protein
TGME49_252430	1.46	7.44	2.60E-07	2.29E-05	hypothetical protein
TGME49_241880	-1.64	7.93	2.70E-07	2.33E-05	3'5'-cyclic nucleotide phosphodiesterase domain-containing protein
TGME49_290580	-2.61	6.26	2.70E-07	2.33E-05	ATP-binding cassette G family transporter ABCG89
TGME49_279340	1.48	6.65	2.77E-07	2.36E-05	hypothetical protein
TGME49_200310	1.55	7.75	2.89E-07	2.43E-05	hypothetical protein
TGME49_221220	3.98	4.72	3.11E-07	2.56E-05	hypothetical protein
TGME49_308840	1.87	9.63	3.20E-07	2.61E-05	SAG-related sequence SRS51
TGME49_254000	1.63	7.14	3.59E-07	2.89E-05	hypothetical protein
TGME49_229440	-2.19	6.25	3.95E-07	3.15E-05	zinc finger, C3HC4 type (RING finger) domain-containing protein
TGME49_215430	4.46	9.38	4.19E-07	3.24E-05	hypothetical protein
TGME49_268225	-9.10	6.07	4.19E-07	3.24E-05	hypothetical protein
TGME49_254120	1.66	8.64	4.33E-07	3.31E-05	autophagy-related protein 8 atg8, putative
TGME49_203730	-2.44	5.79	4.70E-07	3.55E-05	hypothetical protein
TGME49_251170	1.98	5.46	5.08E-07	3.80E-05	KRUF family protein
TGME49_218192	-2.95	4.89	5.39E-07	3.99E-05	hypothetical protein
TGME49_275755	3.42	5.33	5.97E-07	4.38E-05	hypothetical protein

TGME49_278510	-1.62	8.05	7.53E-07	5.47E-05	protein phosphatase 2C domain-containing protein
TGME49_300010	-4.50	5.19	7.85E-07	5.64E-05	hypothetical protein
TGME49_254520	1.45	7.27	8.55E-07	6.04E-05	mediator complex subunit MED11
TGME49_297647	3.99	4.57	9.96E-07	6.96E-05	hypothetical protein
TGME49_215390	2.46	4.45	1.04E-06	7.21E-05	TIM10 family protein, putative
TGME49_247410	1.36	7.58	1.30E-06	8.95E-05	hypothetical protein
TGME49_207430	-2.11	6.38	1.35E-06	9.20E-05	ATP-dependent RNA helicase DDX1, putative
TGME49_253960	1.85	5.35	1.47E-06	9.89E-05	oxidoreductase, short chain dehydrogenase/reductase family protein
TGME49_309990	1.54	6.25	1.54E-06	0.00010286	hypothetical protein
TGME49_262730	1.45	8.05	1.64E-06	0.00010819	rhoptry protein ROP16
TGME49_245428	2.67	5.35	1.95E-06	0.0001267	hypothetical protein
TGME49_312600	1.23	8.45	1.95E-06	0.0001267	heat shock protein HSP21
TGME49_253750	1.53	7.45	2.19E-06	0.00013993	PLU-1 family protein
TGME49_255240	-2.42	5.60	2.18E-06	0.00013993	hypothetical protein
TGME49_244370	6.32	11.45	2.22E-06	0.0001407	TDC1, putative
TGME49_242260	-2.03	5.84	2.25E-06	0.00014136	hypothetical protein
TGME49_266100	1.51	6.81	2.28E-06	0.00014226	rhoptry kinase family protein ROP41
TGME49_266900	1.65	8.02	2.37E-06	0.0001465	cyclin, N-terminal domain-containing protein
TGME49_257750	-2.35	7.84	2.41E-06	0.00014762	homocysteine s-methyltransferase domain-containing protein
TGME49_212250	1.38	7.45	2.45E-06	0.00014882	XPG N-terminal domain-containing protein
TGME49_227430	2.88	5.71	2.63E-06	0.00015834	transmembrane amino acid transporter protein
TGME49_280700	-1.78	7.01	3.09E-06	0.00018327	arginine decarboxylase
TGME49_319350	-1.82	9.13	3.07E-06	0.00018327	SAG-related sequence SRS17B
TGME49_225130	-3.05	5.10	3.16E-06	0.00018604	hypothetical protein
TGME49_218200	-2.74	5.22	3.28E-06	0.00019129	UDP-sugar pyrophospharylase
TGME49_316270	1.61	6.15	3.35E-06	0.00019227	Rab geranylgeranyl transferase type II beta subunit, putative
TGME49_239270	-1.67	7.52	3.44E-06	0.00019593	hypothetical protein
TGME49_244670	1.34	7.10	3.77E-06	0.00021331	hypothetical protein
TGME49_285190	-1.68	6.63	4.06E-06	0.00022813	zinc finger, C3HC4 type (RING finger) domain-containing protein
TGME49_226310	-2.13	7.53	4.20E-06	0.00023417	zinc finger (CCCH type) motif-containing protein
TGME49_246978	-2.90	5.37	4.27E-06	0.00023601	hypothetical protein
TGME49_308090	2.58	9.77	4.38E-06	0.00024025	rhoptry protein ROP5
TGME49_312270	1.37	8.08	4.52E-06	0.00024613	rhoptry protein ROP13
TGME49_255510	-1.43	7.27	4.69E-06	0.00025363	ankyrin repeat-containing protein
TGME49_217220	-1.57	6.81	5.16E-06	0.0002751	zinc finger, C3HC4 type (RING finger) domain-containing protein
TGME49_226380	1.34	8.18	5.64E-06	0.00029866	hypothetical protein
TGME49_208850	2.52	5.42	6.06E-06	0.00031474	SAG-related sequence SRS11
TGME49_279100	1.35	9.07	6.21E-06	0.00031519	hypothetical protein
TGME49_201400	-2.01	5.98	6.21E-06	0.00031519	Sin3-associated polypeptide SAP18
TGME49_293500	-2.99	5.07	6.18E-06	0.00031519	hypothetical protein
TGME49_268860	4.11	4.79	6.59E-06	0.00033238	enolase 1
TGME49_298840	1.64	6.46	6.81E-06	0.00034108	hypothetical protein
TGME49_320110	3.01	8.51	7.85E-06	0.00038531	proliferating cell nuclear antigen PCNA2
TGME49_220240	1.45	8.76	7.98E-06	0.00038886	hypothetical protein
TGME49_268230	-3.86	5.64	8.12E-06	0.00039334	hypothetical protein
TGME49_240250	-1.39	7.48	8.21E-06	0.00039478	macro domain-containing protein
TGME49_262860	-2.20	5.63	8.26E-06	0.00039478	ADP-ribosylation factor family protein 1, putative
TGME49_254050	1.30	6.49	8.53E-06	0.00040365	optic atrophy 3 protein (opa3) protein

TGME49_261750	-1.64	6.89	8.91E-06	0.00041783	rhoptry neck protein RON10
TGME49_212940	-7.44	6.33	9.02E-06	0.00042023	hypothetical protein
TGME49_293480	2.09	7.21	9.35E-06	0.00043025	MoeA N-terminal region (domain I and II) domain-containing protein
TGME49_259530	-1.69	6.38	9.33E-06	0.00043025	GalNac
TGME49_279450	-7.66	4.57	9.51E-06	0.00043496	adenylosuccinate synthetase, putative
TGME49_231180	-3.51	5.84	9.58E-06	0.00043523	hypothetical protein
TGME49_290600	6.31	12.08	9.88E-06	0.00044657	succinyl-CoA-synthetase alpha SCSA
TGME49_250100	1.71	6.54	1.09E-05	0.00049006	hypothetical protein
TGME49_288700	-2.16	5.73	1.16E-05	0.00051614	RecF/RecN/SMC N terminal domain-containing protein
TGME49_276920	-1.73	6.74	1.19E-05	0.00052752	protein phosphatase 2C domain-containing protein
TGME49_258590	-2.53	5.43	1.29E-05	0.00056831	hypothetical protein
TGME49_270650	2.02	5.21	1.34E-05	0.00058835	deoxyribose-phosphate aldolase
TGME49_275450	1.16	7.53	1.41E-05	0.00061313	hypothetical protein
TGME49_219520	-1.38	7.45	1.48E-05	0.00063501	histone arginine methyltransferase PRMT1
TGME49_243298	-2.67	4.90	1.57E-05	0.00066937	ICE family protease (caspase) p20 domain-containing protein
TGME49_262050	1.98	9.30	1.60E-05	0.00067189	rhoptry kinase family protein ROP39
TGME49_269180	-1.22	7.79	1.61E-05	0.00067189	MIF4G domain-containing protein
TGME49_247930	-2.90	5.11	1.61E-05	0.00067189	SNARE domain-containing protein
TGME49_228320	-4.10	4.54	1.59E-05	0.00067189	hypothetical protein
TGME49_223480	-2.62	4.98	1.64E-05	0.00067846	sushi domain (scr repeat) domain-containing protein
TGME49_312380	-2.37	5.23	1.66E-05	0.00068166	tetratricopeptide repeat-containing protein
TGME49_213680	-2.37	5.20	1.70E-05	0.00069717	MmgE/PrpD family protein
TGME49_249860	-1.57	7.01	1.76E-05	0.00071444	hypothetical protein
TGME49_275320	-1.50	7.96	1.79E-05	0.00072583	penicillin amidase
TGME49_277270	1.95	9.46	1.87E-05	0.00075196	NTPase II
TGME49_220940	1.84	5.59	2.11E-05	0.0008438	ribosomal RNA methyltransferase (FtsJ) family protein
TGME49_277260	2.43	9.98	2.32E-05	0.0009244	hypothetical protein
TGME49_226240	1.60	5.23	2.33E-05	0.0009245	bud site selection protein, putative
TGME49_253100	1.15	7.21	2.39E-05	0.0009431	hypothetical protein
TGME49_319580	-2.59	5.43	2.45E-05	0.00096193	hypothetical protein
TGME49_224170	2.34	4.86	2.56E-05	0.00099256	SAG-related sequence SRS60A
TGME49_214540	1.33	6.96	2.58E-05	0.00099437	hypothetical protein
TGME49_310060	1.91	5.85	2.60E-05	0.00099944	small nuclease
TGME49_209090	-1.65	6.23	2.64E-05	0.00100892	proteasome maturation factor ump1 protein
TGME49_253000	1.28	6.88	2.73E-05	0.00103665	ELMO/CED-12 family protein
TGME49_313870	-1.40	6.70	2.74E-05	0.00103665	zinc finger, C3HC4 type (RING finger) domain-containing protein
TGME49_254800	1.68	5.96	2.77E-05	0.00104312	hypothetical protein
TGME49_253650	1.12	8.06	2.92E-05	0.00108559	DnaJ C terminal region domain-containing protein
TGME49_207630	-3.45	4.99	2.93E-05	0.00108559	peptidyl-tRNA hydrolase domain-containing protein
TGME49_203720	1.19	7.96	3.05E-05	0.00112546	vitamin k epoxide reductase family protein
TGME49_306895	1.38	7.00	3.13E-05	0.00114773	hypothetical protein
TGME49_253440	1.15	8.62	3.17E-05	0.00115682	cell-cycle-associated protein kinase SRPK, putative
TGME49_220460	-2.59	4.82	3.31E-05	0.00120346	SNF7 family protein
TGME49_254660	1.25	6.61	3.33E-05	0.00120421	ankyrin repeat-containing protein
TGME49_270510	-1.76	9.30	3.39E-05	0.00122099	asparaginyl-tRNA synthetase (NOB+tRNA synthase)
TGME49_254930	1.20	7.07	3.46E-05	0.00123954	hypothetical protein

TGME49_236990	2.33	5.52	3.56E-05	0.00126967	beta-ketoacyl synthase, N-terminal domain-containing protein
TGME49_201260	-1.27	8.89	3.73E-05	0.00131483	sugar transporter ST3
TGME49_259010	-1.66	6.56	3.77E-05	0.00132209	vacuolar ATP synthase subunit d, putative
TGME49_223020	-1.62	6.52	3.86E-05	0.00134304	coproporphyrinogen III oxidase
TGME49_237830	-1.95	5.67	3.85E-05	0.00134304	DNA polymerase I domain-containing protein
TGME49_205680	1.48	7.62	3.90E-05	0.0013509	hypothetical protein
TGME49_277710	-1.61	6.74	3.99E-05	0.00136779	hypothetical protein
TGME49_263510	-2.31	5.94	3.98E-05	0.00136779	Spc97 / Spc98 family protein
TGME49_315590	-1.80	5.72	4.01E-05	0.00136872	macro domain-containing protein
TGME49_234530	1.33	7.13	4.12E-05	0.00139486	hypothetical protein
TGME49_212090	1.15	6.72	4.14E-05	0.00139486	hypothetical protein
TGME49_313230	2.45	9.56	4.32E-05	0.00143499	eukaryotic initiation factor-2, alpha subunit
TGME49_274070	-2.85	5.15	4.35E-05	0.00143775	ThiF family protein
TGME49_208730	1.05	8.09	4.50E-05	0.00146959	microneme protein, putative
TGME49_232640	1.87	5.17	4.66E-05	0.00150626	RNA 2'-phosphotransferase, Tpt1/KptA family protein
TGME49_200360	1.64	9.81	4.64E-05	0.00150626	hypothetical protein
TGME49_323320	-2.94	5.08	5.02E-05	0.00160876	hypothetical protein
TGME49_213570	4.84	11.16	5.08E-05	0.00162125	hypothetical protein
TGME49_244120	-1.84	5.76	5.12E-05	0.00162825	hypothetical protein
TGME49_311210	-1.53	6.42	5.18E-05	0.00164069	hypothetical protein
TGME49_213820	1.35	6.73	5.23E-05	0.0016485	hypothetical protein
TGME49_252280	1.54	5.98	5.26E-05	0.00164914	hypothetical protein
TGME49_253290	1.10	7.51	5.28E-05	0.00164914	valyl-tRNA synthetase
TGME49_305340	-1.17	8.17	5.55E-05	0.00172919	corepressor complex CRC230
TGME49_312310	-2.24	5.48	5.60E-05	0.00173537	ATPase, AAA family protein
TGME49_320005	-2.39	5.44	6.07E-05	0.0018665	hypothetical protein
TGME49_208580	-1.58	6.18	6.28E-05	0.00190634	DNA ligase 1, putative
TGME49_318525	-1.59	6.53	6.26E-05	0.00190634	hypothetical protein
TGME49_215940	-1.97	6.61	6.38E-05	0.00192266	Acetyl-coenzyme A transporter, putative
TGME49_219832	-2.42	5.42	6.38E-05	0.00192266	cyclin-dependent kinase regulatory subunit protein
TGME49_238240	5.25	11.68	6.46E-05	0.0019387	bystin protein
TGME49_230490	-1.58	6.28	6.68E-05	0.00199534	phosphatidylinositol-4-phosphate 5-kinase
TGME49_299240	1.64	5.35	6.71E-05	0.00199771	hypothetical protein
TGME49_233030	-1.20	8.47	6.90E-05	0.00204475	gliding-associated protein GAP70
TGME49_253140	1.47	5.83	7.19E-05	0.00212168	hypothetical protein
TGME49_313200	-2.23	5.25	7.37E-05	0.00216801	leucine rich repeat-containing protein
TGME49_257180	-1.89	5.94	7.50E-05	0.00219527	RecF/RecN/SMC N terminal domain-containing protein
TGME49_248160	-2.51	4.95	7.77E-05	0.00225775	hypothetical protein
TGME49_321170	1.75	4.77	7.83E-05	0.00226195	Toxoplasma gondii family C protein
TGME49_214410	1.11	7.48	8.00E-05	0.00229749	hypothetical protein
TGME49_200320	-1.28	7.51	8.16E-05	0.00233334	hypoxanthine-xanthine-guanine phosphoribosyl transferase HXGPRT
TGME49_260250	-1.90	6.13	8.19E-05	0.00233492	cyclin domain protein, cyclin H family protein
TGME49_254620	1.56	9.88	8.23E-05	0.00233767	ribosomal protein RPL39
TGME49_269710	-1.49	6.75	8.60E-05	0.00243087	hypothetical protein
TGME49_262780	-1.60	6.39	8.66E-05	0.00244032	FHA domain-containing protein
TGME49_293430	1.13	8.30	8.79E-05	0.00246332	hypothetical protein
TGME49_283820	-2.83	4.90	8.81E-05	0.00246332	glycine cleavage T-protein (aminomethyl transferase) domain-containing protein
TGME49_236270	-2.80	5.33	8.94E-05	0.00249078	hypothetical protein

TGME49_301170	-7.08	5.07	9.02E-05	0.00250402	SAG-related sequence SRS19D
TGME49_246800	-1.47	6.38	9.06E-05	0.00250536	acylaminoacyl-peptidase, putative
TGME49_254080	1.13	7.77	9.22E-05	0.00254059	metal cation transporter, ZIP family protein
TGME49_263610	-1.84	6.26	9.60E-05	0.00262369	hypothetical protein
TGME49_228360	1.31	7.28	9.85E-05	0.00268293	peptidyl-prolyl isomerase FKBP12, putative
TGME49_290300	1.15	7.98	0.00010516	0.00285405	hypothetical protein
TGME49_240700	-1.63	6.20	0.00010791	0.00291796	ubiquitin family protein
TGME49_311440	1.31	6.20	0.00010845	0.00292181	SAG-related sequence SRS50
TGME49_288400	-1.67	6.11	0.00011037	0.00295201	LETM1 family protein
TGME49_297230	-1.67	6.27	0.00011013	0.00295201	Vps53 family, N-terminal protein
TGME49_249550	-1.70	5.67	0.00011136	0.00296793	hypothetical protein
TGME49_258070	-1.42	6.52	0.0001122	0.00297944	hypothetical protein
TGME49_228370	-1.57	5.97	0.00011557	0.00305802	hypothetical protein
TGME49_229640	-1.65	6.18	0.00011697	0.00308412	hypothetical protein
TGME49_246200	-2.46	5.02	0.00011924	0.00313277	zinc finger (CCCH type) motif-containing protein
TGME49_214270	2.98	9.52	0.00012409	0.00320121	translation initiation factor IF-2, putative
TGME49_230830	2.06	5.05	0.00012444	0.00320121	ATPase family associated with various cellular activities (AAA) domain-containing protein
TGME49_253180	2.00	8.08	0.00012363	0.00320121	hypothetical protein
TGME49_266990	-1.18	7.64	0.00012372	0.00320121	beta-COP
TGME49_316230	-1.65	5.94	0.0001242	0.00320121	SAC1 phosphoinositide phosphatase, putative
TGME49_291930	3.85	9.99	0.0001262	0.00323535	RNA recognition motif-containing protein
TGME49_253330	1.57	7.81	0.00013494	0.00338915	Rhoptry kinase family protein, truncated (incomplete catalytic triad)
TGME49_278830	-1.19	8.64	0.00013481	0.00338915	glucose-6-phosphate 1-dehydrogenase
TGME49_233680	-2.65	4.92	0.00013451	0.00338915	nuclear movement family protein
TGME49_257350	1.05	8.19	0.00013608	0.00340617	eukaryotic translation initiation factor, putative
TGME49_268620	-2.80	5.03	0.00013716	0.00342171	blood stage antigen 41-3 precursor, putative
TGME49_202780	1.04	7.70	0.00014093	0.00350376	rhoptry kinase family protein ROP25
TGME49_296015	1.16	6.47	0.00014142	0.00350418	hypothetical protein
TGME49_306410	-2.16	5.32	0.00014402	0.00355684	hypothetical protein
TGME49_258790	-1.35	7.15	0.00014733	0.00362656	zinc finger, C3HC4 type (RING finger) domain-containing protein
TGME49_304680	-1.73	5.66	0.00014843	0.0036313	ubiquitin family protein
TGME49_263720	-2.27	5.02	0.00014851	0.0036313	HMG (high mobility group) box domain-containing protein
TGME49_247680	-2.26	5.02	0.00014949	0.00364344	hypothetical protein
TGME49_260210	-2.52	5.25	0.00015119	0.0036606	DnaJ domain-containing protein
TGME49_227150	1.22	7.65	0.00015509	0.00371856	glutaredoxin, putative
TGME49_229470	-1.37	6.59	0.00015476	0.00371856	hypothetical protein
TGME49_214170	-1.82	5.90	0.00015463	0.00371856	hypothetical protein
TGME49_310130	-1.58	6.23	0.00015747	0.00376363	Spc97 / Spc98 family protein
TGME49_252260	1.92	4.57	0.00015824	0.00376985	hypothetical protein
TGME49_220910	-1.62	6.95	0.00015963	0.00379064	HEAT repeat-containing protein
TGME49_223150	-2.36	5.54	0.00016026	0.00379359	START domain-containing protein
TGME49_285710	1.39	5.78	0.00016365	0.00386136	hypothetical protein
TGME49_217360	-2.11	5.84	0.0001689	0.00397272	hypothetical protein
TGME49_267070	1.12	7.77	0.00017227	0.00403898	aquaporin 2
TGME49_204270	-1.63	6.40	0.00017545	0.00410063	hypothetical protein
TGME49_240430	-2.23	4.90	0.00017918	0.00416166	glyoxalase family protein
TGME49_314250	2.07	4.49	0.0001811	0.00419307	bradyzoite rhoptry protein BRP1
TGME49_254270	1.33	5.62	0.00018265	0.00421575	hypothetical protein
TGME49_230430	-2.06	5.37	0.00018328	0.00421706	vesicle-associated membrane protein, putative

TGME49_247360	1.07	7.30	0.0001861	0.00426873	PAP2 superfamily protein
TGME49_208910	-1.53	6.43	0.0001868	0.00427166	hypothetical protein
TGME49_201710	1.59	5.63	0.00019086	0.00435097	WD domain, G-beta repeat-containing protein
TGME49_321410	2.86	8.06	0.00020402	0.00462083	hypothetical protein
TGME49_263785	-2.59	4.86	0.00020457	0.00462083	phosphatidate cytidylyltransferase
TGME49_214575	1.71	5.04	0.00021055	0.00474163	hypothetical protein
TGME49_313180	-1.05	8.51	0.00021188	0.004757	cell-cycle-associated protein kinase PRP4, putative
TGME49_217350	-2.26	5.20	0.00021405	0.00479115	methyltransferase MTA70, putative
TGME49_313385	1.64	5.33	0.00021608	0.00482205	hypothetical protein
TGME49_230080	-1.72	5.34	0.00021823	0.00484095	DEAD/DEAH box helicase domain-containing protein
TGME49_259240	4.06	11.70	0.00022027	0.00485719	ribosomal protein RPS21
TGME49_275990	1.26	6.60	0.00022018	0.00485719	hypothetical protein
TGME49_319312	-2.01	5.18	0.00022662	0.00496749	hypothetical protein
TGME49_222920	1.31	6.27	0.00023378	0.00509433	mbp-1 interacting protein-2a family protein
TGME49_253820	1.08	8.35	0.00023979	0.00514958	hypothetical protein
TGME49_207880	-1.59	6.29	0.00023847	0.00514958	hypothetical protein
TGME49_231960	-1.88	6.35	0.00023921	0.00514958	omega secalin, putative
TGME49_232060	1.73	5.60	0.00024263	0.00519545	hypothetical protein
TGME49_282190	-1.15	7.00	0.00024573	0.00524339	hydrolase, NUDIX family protein
TGME49_215930	-6.94	4.44	0.00024628	0.00524339	mediator complex subunit MED21
TGME49_305160	2.59	4.53	0.00024984	0.00530208	histone H2Ba
TGME49_283540	1.10	8.44	0.00025047	0.00530208	hypothetical protein
TGME49_232270	-1.53	5.61	0.00025197	0.00531867	histidine acid phosphatase superfamily protein
TGME49_233300	-1.71	5.26	0.00025802	0.00543094	RhoGAP domain-containing protein
TGME49_253470	1.02	7.33	0.00026302	0.00552036	alveolin domain containing intermediate filament IMC13
TGME49_298970	1.21	6.90	0.00026645	0.00556087	LSM3, U6 small nuclear RNA associated isoform 2 family protein
TGME49_216140	1.19	7.04	0.00026615	0.00556087	tetratricopeptide repeat-containing protein
TGME49_232960	-1.32	7.01	0.00027333	0.00568857	oxidoreductase, 2OG-Fe(II) oxygenase family protein
TGME49_311905	-2.69	4.73	0.0002766	0.00574043	hypothetical protein
TGME49_318650	1.08	8.08	0.00028096	0.00579849	transhydrogenase
TGME49_254365	1.07	7.70	0.00028074	0.00579849	phosphatidate cytidylyltransferase
TGME49_314070	1.14	6.15	0.00028688	0.00590407	hypothetical protein
TGME49_205330	1.01	7.80	0.00028841	0.00590648	hypothetical protein
TGME49_304670	-1.51	6.35	0.00028938	0.00590648	leucine rich repeat-containing protein
TGME49_242118	-3.74	4.95	0.00028901	0.00590648	myosin-light-chain kinase
TGME49_254460	1.22	7.06	0.00029138	0.00593077	hypothetical protein
TGME49_293190	-1.18	7.22	0.0002937	0.00596178	endonuclease/exonuclease/phosphatase family protein
TGME49_253170	1.79	6.90	0.00029676	0.00600733	zinc carboxypeptidase, putative
TGME49_316250	1.00	8.64	0.00029892	0.00603455	hypothetical protein
TGME49_207160	2.16	5.07	0.00030009	0.00603463	SAG-related sequence SRS49D
TGME49_251500	-1.23	7.59	0.00030055	0.00603463	eukaryotic initiation factor-3, subunit 3, putative
TGME49_213710	-1.50	6.17	0.0003018	0.00603489	WD domain, G-beta repeat-containing protein
TGME49_313970	-2.61	5.18	0.00030301	0.00603489	Phytanoyl-CoA dioxygenase (PhyH) superfamily protein
TGME49_295950	1.36	5.58	0.00030397	0.0060378	KRUF family protein
TGME49_221320	1.65	9.10	0.0003076	0.00609352	acetyl-CoA carboxylase ACC1
TGME49_320050	7.08	16.23	0.00031077	0.00613997	ribosomal protein RPL5
TGME49_297730	-1.86	5.78	0.00031384	0.00618415	transcription elongation factor 1, putative

TGME49_205200	1.15	6.26	0.00032129	0.00631415	hypothetical protein
TGME49_271930	-0.99	8.48	0.00033394	0.0065282	hypothetical protein
TGME49_293740	0.99	8.81	0.00033811	0.0065922	hypothetical protein
TGME49_306020	1.32	5.49	0.00034053	0.0066061	hypothetical protein
TGME49_289140	1.06	6.35	0.0003415	0.0066061	ribosomal protein l22/l43, putative
TGME49_263710	-1.45	6.20	0.00034145	0.0066061	acyl-CoA:cholesterol acyltransferase alpha ACAT1-alpha
TGME49_202280	-2.17	5.61	0.00034579	0.00667178	WD domain, G-beta repeat-containing protein
TGME49_275430	-1.69	5.84	0.00035795	0.00688843	hypothetical protein
TGME49_202240	-2.68	4.63	0.00036471	0.0069642	RAP domain-containing protein
TGME49_206430	-1.19	6.71	0.00036692	0.00698479	formin FRM1
TGME49_216020	-1.87	5.94	0.00036767	0.00698479	peptidase family c78 protein
TGME49_297780	-1.53	6.48	0.00036885	0.00698931	ATPase/histidine kinase/DNA gyrase B/HSP90 domain-containing protein
TGME49_227580	-1.25	7.72	0.00037195	0.0070301	transmembrane amino acid transporter protein
TGME49_249730	-1.78	5.40	0.00037465	0.00706299	hypothetical protein
TGME49_263080	1.04	7.13	0.00037761	0.00709871	hypothetical protein
TGME49_313480	-2.71	4.81	0.00037846	0.00709871	hypothetical protein
TGME49_264870	1.20	7.17	0.00038614	0.00722461	Sodium:neurotransmitter symporter family protein
TGME49_243580	-1.07	7.00	0.00038764	0.00723421	Hit family protein involved in cell-cycle regulation, putative
TGME49_240650	-1.13	7.64	0.00039034	0.00724817	coatomer protein complex, subunit alpha, putative
TGME49_295935	1.08	9.03	0.00039705	0.00735441	KRUF family protein
TGME49_311920	-1.17	7.15	0.00040272	0.00742228	GRAM domain-containing protein
TGME49_239600	1.75	5.83	0.00042169	0.00773345	rhoptry kinase family protein ROP23 (incomplete catalytic triad)
TGME49_216510	-1.55	5.83	0.00042557	0.00778529	thioredoxin, putative
TGME49_253490	1.22	6.93	0.00042778	0.00780639	hypothetical protein
TGME49_257340	-2.29	4.72	0.00043817	0.00797647	Ras family protein
TGME49_216920	-1.69	5.80	0.00044017	0.00799311	mediator complex subunit MED8
TGME49_276930	1.04	8.13	0.00044148	0.00799745	hypothetical protein
TGME49_270595	2.39	6.82	0.00044406	0.00802454	UBA/TS-N domain-containing protein
TGME49_289650	0.97	8.20	0.0004498	0.00808867	PEP-carboxykinase I
TGME49_253600	1.45	6.44	0.00045447	0.00815295	hypothetical protein
TGME49_312560	-1.26	6.38	0.00047041	0.0083982	hypothetical protein
TGME49_221580	-1.20	6.88	0.00047959	0.00852098	ribosomal RNA large subunit methyltransferase J protein
TGME49_267420	0.95	8.24	0.00049023	0.00867479	mago nashi family protein 2, putative
TGME49_270530	-1.26	6.58	0.00049227	0.00867479	ubiquitin fusion degradation protein UFD1CY
TGME49_251460	-1.56	6.07	0.00049293	0.00867479	hypothetical protein
TGME49_217555	0.89	7.97	0.00049656	0.00871801	hypothetical protein
TGME49_277920	1.19	7.07	0.00050086	0.00877276	hypothetical protein
TGME49_244380	2.19	8.24	0.00050211	0.00877391	cactin
TGME49_311250	-1.39	5.97	0.00053395	0.00928651	hypothetical protein
TGME49_315750	-1.33	7.11	0.00053527	0.00928759	hypothetical protein
TGME49_214970	-1.23	8.09	0.00054158	0.00937513	DNA replication licensing factor, putative
TGME49_254150	1.62	5.06	0.00054947	0.00948962	hypothetical protein
TGME49_257120	-1.63	6.07	0.00055897	0.00963119	sugar transporter ST1
TGME49_262630	2.15	4.84	0.00056298	0.00967783	hypothetical protein
TGME49_210380	-1.15	6.94	0.00056806	0.00974136	hypothetical protein
TGME49_214400	1.40	4.87	0.0005728	0.00977856	hypothetical protein
TGME49_268220	5.79	4.65	0.00059543	0.01011826	hypothetical protein
TGME49_209050	-1.37	6.89	0.00060153	0.0101984	Tyrosine kinase-like (TKL) protein

TGME49_221170	-1.28	6.84	0.00060944	0.01026237	CAAX metallo endopeptidase
TGME49_253860	1.04	6.69	0.00061773	0.01031911	Tyrosine kinase-like (TKL) protein
TGME49_255650	-1.18	7.47	0.00061854	0.01031911	DHHC zinc finger domain-containing protein
TGME49_269290	-1.82	5.13	0.00062668	0.01041055	hypothetical protein
TGME49_310730	-1.22	6.24	0.00063057	0.01045163	hypothetical protein
TGME49_268810	-1.52	6.39	0.00064029	0.01056542	ck2 beta subunit
TGME49_318320	-1.92	5.30	0.00063975	0.01056542	ATP-dependent Clp endopeptidase, proteolytic subunit ClpP domain-containing protein
TGME49_264970	-1.84	5.65	0.00066745	0.0109406	hypothetical protein
TGME49_284660	1.34	5.36	0.00066934	0.01094734	mitochondrial ribosomal protein s6-2, putative
TGME49_254710	1.05	7.19	0.00067379	0.01099578	serine esterase (DUF676) protein
TGME49_231140	3.09	11.22	0.00067728	0.01102846	ribosomal protein RPS25
TGME49_245460	4.06	12.61	0.00068283	0.01108366	ribosomal protein RPS8
TGME49_213050	1.31	8.33	0.00068366	0.01108366	hypothetical protein
TGME49_229690	-1.13	6.42	0.00070208	0.01135747	autophagy-related protein 7 atg7, putative
TGME49_219610	1.62	5.75	0.00070979	0.01138331	hypothetical protein
TGME49_205700	1.58	10.39	0.00070824	0.01138331	cyclophilin precursor
TGME49_318750	1.30	6.14	0.00070629	0.01138331	deoxyribose-phosphate aldolase
TGME49_252290	1.16	6.98	0.00070982	0.01138331	importin alpha, putative
TGME49_232130	-0.98	8.66	0.00075497	0.01202923	hypothetical protein
TGME49_297420	-1.64	5.89	0.00075485	0.01202923	beta-tubulin cofactor D, putative
TGME49_246330	-1.14	6.92	0.00078269	0.01236448	CRAL/TRIO domain-containing protein
TGME49_295472	-1.45	6.42	0.0007853	0.01237927	C2 domain-containing protein
TGME49_310460	-1.11	6.61	0.00079421	0.0124667	Rab6
TGME49_290160	-1.13	7.33	0.00080711	0.0126424	sortilin, putative
TGME49_288245	1.31	6.23	0.00083168	0.01292563	hypothetical protein
TGME49_308950	1.08	8.04	0.000835	0.01292563	histidine acid phosphatase superfamily protein
TGME49_295990	1.00	6.85	0.00082928	0.01292563	ubiquitin conjugating enzyme E2, putative
TGME49_210255	0.99	6.96	0.00083223	0.01292563	hypothetical protein
TGME49_310360	-1.10	7.38	0.00083566	0.01292563	hypothetical protein
TGME49_240710	-1.19	6.28	0.00083307	0.01292563	RNA recognition motif-containing protein
TGME49_242030	-1.71	5.29	0.00084056	0.01297445	hypothetical protein
TGME49_285170	-2.95	4.61	0.00086138	0.01324058	methyltransferase small, putative
TGME49_314970	-1.05	7.01	0.00087225	0.01337999	root hair defective 3 gtp-binding protein (rhd3) protein
TGME49_224020	1.06	6.40	0.00087744	0.01340404	hypothetical protein
TGME49_204400	-0.97	8.61	0.00089028	0.01352629	ATPase synthase subunit alpha, putative
TGME49_321560	-1.29	6.59	0.00089092	0.01352629	zinc knuckle domain-containing protein
TGME49_308580	-1.64	5.77	0.0008882	0.01352629	Lon protease family protein
TGME49_211440	-1.34	6.23	0.00089381	0.0135424	hypothetical protein
TGME49_243730	1.29	9.93	0.0009079	0.01369992	rhoptry protein ROP9
TGME49_253310	1.20	5.37	0.00091216	0.01373615	hypothetical protein
TGME49_237290	-1.25	6.65	0.00091464	0.0137456	hypothetical protein
TGME49_298030	-1.96	4.94	0.000944	0.0141581	Ubiquinol-cytochrome c chaperone, putative
TGME49_216810	1.13	7.66	0.00095327	0.01426829	5'-nucleotidase, C-terminal domain-containing protein
TGME49_254720	1.44	9.40	0.00096501	0.01438714	dense granule protein GRA8
TGME49_238040	-1.31	6.05	0.00096704	0.01438714	protein disulfide-isomerase domain-containing protein
TGME49_235740	-1.50	5.82	0.00096556	0.01438714	hypothetical protein
TGME49_266850	1.27	5.79	0.00097381	0.0144589	3-demethylubiquinone-9 3-O-methyltransferase
TGME49_290150	-1.42	6.93	0.00098669	0.01462071	hypothetical protein
TGME49_314780	-1.05	7.21	0.00100056	0.01476718	myosin G

TGME49_306350	1.14	6.54	0.00102208	0.01505484	variable surface lipoprotein
TGME49_258090	-1.11	6.96	0.00103856	0.0152673	hypothetical protein
TGME49_248570	-1.74	5.00	0.00106235	0.01555529	hypothetical protein
TGME49_201880	0.87	8.04	0.00107156	0.0156529	hypothetical protein
TGME49_225560	-1.11	8.81	0.00107325	0.0156529	hypothetical protein
TGME49_211860	1.16	6.12	0.00109557	0.01591585	hypothetical protein
TGME49_240450	1.47	5.06	0.00112395	0.01623578	Maf family protein
TGME49_294790	0.93	8.88	0.00112502	0.01623578	hypothetical protein
TGME49_294730	-1.37	6.07	0.00113817	0.01633648	hypothetical protein
TGME49_242870	-1.50	5.64	0.00113996	0.01633648	histone lysine methyltransferase, SET, putative
TGME49_298980	1.08	6.84	0.00115131	0.01643551	RNA pseudouridine synthase superfamily protein
TGME49_224220	-1.35	6.62	0.00115842	0.01650532	serine/threonine-protein phosphatase PP2A catalytic subunit
TGME49_297320	-1.30	5.89	0.0011648	0.01656427	hypothetical protein
TGME49_310300	-1.53	5.25	0.00120233	0.01705872	hypothetical protein
TGME49_225440	-3.61	4.61	0.00120417	0.01705872	hypothetical protein
TGME49_318690	1.49	4.49	0.00120743	0.01707098	RNA recognition motif-containing protein
TGME49_274160	-1.05	6.52	0.00120965	0.01707098	hypothetical protein
TGME49_316660	-1.20	6.57	0.00124589	0.0175157	cullin family protein
TGME49_220400	1.45	10.26	0.00125938	0.01764355	actin depolymerizing factor ADF
TGME49_290040	0.97	7.91	0.00126616	0.01769992	macrophage migration inhibitory factor, putative
TGME49_316430	-1.77	6.04	0.00130408	0.01816149	target of rapamycin (TOR), putative
TGME49_311625	-1.49	5.89	0.00131879	0.01833197	WD domain, G-beta repeat-containing protein
TGME49_240950	-2.61	5.11	0.00132731	0.01838143	hypothetical protein
TGME49_223950	0.97	6.29	0.00134077	0.01853201	hypothetical protein
TGME49_321540	-1.18	7.23	0.00134319	0.01853201	hypothetical protein
TGME49_307570	-1.35	6.39	0.00134959	0.01858571	glycerol-3-phosphate dehydrogenase (gpdh), putative
TGME49_295400	-1.87	5.29	0.00136174	0.01871825	hypothetical protein
TGME49_252310	1.02	6.62	0.00137291	0.01883681	hypothetical protein
TGME49_225250	1.03	5.81	0.00137573	0.01883815	LSU ribosomal protein L14P, putative
TGME49_280560	-1.12	6.83	0.00138008	0.01883815	selenide, water dikinase
TGME49_215250	-2.07	5.14	0.00140106	0.01904667	thiamin pyrophosphokinase, catalytic domain-containing protein
TGME49_329800	-1.24	6.42	0.00140429	0.01905571	hypothetical protein
TGME49_218530	-1.09	6.53	0.00141492	0.01916474	proteasome-interacting thioredoxin domain-containing protein
TGME49_255215	1.78	5.63	0.00145107	0.01952607	hypothetical protein
TGME49_203600	-1.16	9.05	0.00145213	0.01952607	hypothetical protein
TGME49_258410	1.02	9.04	0.00147514	0.01979951	photosensitized INA-labeled protein PHIL1
TGME49_297492	-3.43	4.52	0.00149451	0.01998702	hypothetical protein
TGME49_270010	-1.50	5.45	0.00149954	0.02001819	hypothetical protein
TGME49_254070	0.93	7.76	0.0015086	0.02009092	hypothetical protein
TGME49_234640	-1.24	6.39	0.00151312	0.02009092	hypothetical protein
TGME49_258740	-1.35	6.23	0.00151045	0.02009092	eukaryotic initiation factor-2A, putative
TGME49_253900	1.38	5.99	0.00151839	0.02012475	parasite porphobilinogen synthase PBGS
TGME49_226755	1.13	6.33	0.0015725	0.02080468	3'5'-cyclic nucleotide phosphodiesterase domain-containing protein
TGME49_231870	-1.86	5.00	0.00158699	0.02095908	tetratricopeptide repeat-containing protein
TGME49_264760	-1.12	6.27	0.00159164	0.02098306	Oxysterol-binding protein
TGME49_315300	-1.20	6.20	0.00160345	0.02108525	transcription factor IIB, putative
TGME49_244040	-1.68	5.58	0.00160509	0.02108525	HEAT repeat-containing protein
TGME49_217010	-1.33	6.08	0.00162105	0.0212573	hypothetical protein

TGME49_297880	-1.54	10.22	0.00163174	0.02135967	dense granule protein DG32
TGME49_262070	-0.99	7.01	0.00163574	0.0213742	ribosomal rna assembly protein mis3, putative
TGME49_271760	-1.67	5.44	0.00167307	0.02182356	seryl-tRNA synthetase (SerRS2)
TGME49_260310	1.52	4.60	0.00168182	0.02184941	ATP-binding cassette transporter ABC.B1
TGME49_286440	0.97	6.38	0.00167841	0.02184941	malic enzyme
TGME49_224540	-1.52	5.44	0.0016839	0.02184941	hypothetical protein
TGME49_216770	2.12	6.83	0.00170286	0.02201831	hypothetical protein
TGME49_243410	-1.36	6.18	0.00171962	0.02219625	tetratricopeptide repeat-containing protein
TGME49_315130	0.93	6.70	0.00174785	0.02249924	L-isoaspartyl protein carboxyl methyltransferase family protein
TGME49_313590	-1.54	5.92	0.00178161	0.02287684	hypothetical protein
TGME49_253730	1.16	6.80	0.00178625	0.02288993	importin-beta N-terminal domain-containing protein
TGME49_297720	-1.14	7.42	0.00178881	0.02288993	trehalose-phosphatase
TGME49_306730	-2.45	5.07	0.00180396	0.02304404	hypothetical protein
TGME49_316260	0.98	8.32	0.00182694	0.02329744	hypothetical protein
TGME49_235515	1.91	4.58	0.00183264	0.0233186	MORN repeat-containing protein
TGME49_306440	-1.34	5.97	0.0018349	0.0233186	hypothetical protein
TGME49_248130	-2.01	5.04	0.00186308	0.02359582	hypothetical protein
TGME49_240910	-1.21	6.41	0.00186806	0.02361847	hypothetical protein
TGME49_306280	0.99	6.62	0.00190188	0.02400522	mediator complex subunit MED7
TGME49_259860	-1.24	5.70	0.00191143	0.02408468	hypothetical protein
TGME49_224810	-1.98	4.85	0.00192209	0.02417439	hypothetical protein
TGME49_209020	-2.11	4.48	0.00192508	0.02417439	hypothetical protein
TGME49_275460	1.38	6.68	0.00195144	0.02427203	hypothetical protein
TGME49_278770	-1.20	7.00	0.0019513	0.02427203	hypothetical protein
TGME49_251620	-1.24	6.29	0.00195086	0.02427203	flap structure-specific endonuclease 1, putative
TGME49_209970	-1.33	6.05	0.00194648	0.02427203	Spc97 / Spc98 family protein
TGME49_262710	0.96	8.75	0.0019608	0.02429365	Ctr copper transporter family protein
TGME49_218830	-1.83	5.10	0.00195896	0.02429365	hypothetical protein
TGME49_234180	1.00	7.18	0.00201506	0.02467699	hypothetical protein
TGME49_216730	-0.88	7.83	0.00200971	0.02467699	MCM2/3/5 family protein
TGME49_291940	-1.03	6.46	0.00200642	0.02467699	hypothetical protein
TGME49_267680	-1.21	6.31	0.0020017	0.02467699	microneme protein MIC12
TGME49_255960	-1.38	6.00	0.00201361	0.02467699	hypothetical protein
TGME49_222120	-1.66	5.28	0.00199738	0.02467699	hypothetical protein
TGME49_263660	-2.37	4.59	0.00201448	0.02467699	hypothetical protein
TGME49_220530	-1.67	5.46	0.00203992	0.02489908	AP2 domain transcription factor AP2V-1
TGME49_271430	-1.77	5.19	0.00204409	0.02490897	hypothetical protein
TGME49_248750	-1.52	5.03	0.00208653	0.02536982	hypothetical protein
TGME49_319540	-1.62	5.83	0.00210067	0.02547273	hypothetical protein
TGME49_229790	-1.43	5.59	0.00210596	0.02549522	hypothetical protein
TGME49_240440	1.01	5.78	0.002127	0.02570794	hypothetical protein
TGME49_267590	-1.36	5.96	0.00215429	0.02599528	hypothetical protein
TGME49_252190	1.41	5.74	0.00216231	0.02600739	KRUF family protein
TGME49_283550	0.97	6.70	0.00216115	0.02600739	hypothetical protein
TGME49_208590	-1.08	7.09	0.00217409	0.02608973	vacuolar ATP synthase subunit 54kD, putative
TGME49_288040	-1.09	6.46	0.0021762	0.02608973	hypothetical protein
TGME49_293820	-1.01	7.28	0.00218433	0.02612197	calpain family cysteine protease domain-containing protein
TGME49_228300	-1.69	5.32	0.00218594	0.02612197	CCDC25 protein
TGME49_237110	-1.16	6.44	0.00220922	0.02635771	replication factor C subunit 2, putative
TGME49_228980	-1.68	4.98	0.0022169	0.02640676	hypothetical protein

TGME49_237230	1.16	9.68	0.00222637	0.02647699	hypothetical protein
TGME49_225930	0.78	8.16	0.00223061	0.02648492	triose-phosphate isomerase TPI-I
TGME49_239010	1.54	6.91	0.0022347	0.02649108	hypothetical protein
TGME49_247690	-1.05	7.31	0.00224321	0.0265494	phospholipid-translocating P-type ATPase, flippase subfamily protein
TGME49_309070	-1.67	5.42	0.00224943	0.02658059	hypothetical protein
TGME49_214810	1.24	5.74	0.00226596	0.02673327	hypothetical protein
TGME49_275650	1.27	7.60	0.00228085	0.02678282	hypothetical protein
TGME49_235920	-1.14	6.30	0.00227959	0.02678282	dynein, axonemal, heavy chain 2 family protein
TGME49_229650	-1.29	6.31	0.00228462	0.02678282	josephin protein
TGME49_215400	-1.74	5.40	0.00228185	0.02678282	RNA recognition motif-containing protein
TGME49_253880	0.81	7.79	0.00228968	0.02679972	GNS1/SUR4 family protein
TGME49_221330	-1.17	6.74	0.00230189	0.02690021	DNA gyrase/topoisomerase IV, A subunit domain-containing protein
TGME49_207065	1.08	5.52	0.00234689	0.02733981	hypothetical protein
TGME49_223690	1.13	5.90	0.00235945	0.02739997	hypothetical protein
TGME49_222400	-0.85	7.23	0.00235804	0.02739997	hypothetical protein
TGME49_222380	-0.86	7.82	0.00237312	0.02747252	importin-beta N-terminal domain-containing protein
TGME49_252250	1.30	5.19	0.002391	0.02763637	ATPase, AAA family protein
TGME49_247760	-1.29	6.62	0.00239737	0.02766684	AMP-binding enzyme domain-containing protein
TGME49_203170	-1.11	7.15	0.00245339	0.02822542	OB-fold nucleic acid binding domain-containing protein
TGME49_200450	-1.73	4.90	0.00246852	0.02835542	hypothetical protein
TGME49_208830	1.16	8.09	0.00247261	0.02835843	hypothetical protein
TGME49_229250	1.42	9.83	0.00247961	0.02839475	ribosomal protein RPL28
TGME49_259115	-1.48	7.48	0.00253517	0.02898622	ABC1 family protein
TGME49_283860	-1.72	5.12	0.00253936	0.02898941	leucine rich repeat-containing protein
TGME49_220950	1.44	9.51	0.00254747	0.02903724	hypothetical protein
TGME49_318430	-0.91	9.00	0.00256332	0.02917301	malate dehydrogenase MDH
TGME49_210430	-0.93	7.63	0.00256966	0.02920031	DnaJ domain-containing protein
TGME49_238140	-1.15	6.40	0.00259458	0.02943833	hypothetical protein
TGME49_214750	-0.97	6.75	0.00261043	0.02957291	hypothetical protein
TGME49_271300	1.38	5.25	0.00261811	0.02961461	DNA-directed RNA polymerase II RPB7
TGME49_202430	-1.38	5.89	0.00262765	0.02967718	hypothetical protein
TGME49_274150	-1.72	5.23	0.00263899	0.02975988	hypothetical protein
TGME49_253740	-1.22	5.77	0.00265046	0.02976296	hypothetical protein
TGME49_278060	-1.76	5.81	0.00264946	0.02976296	Mre11 DNA-binding domain-containing protein
TGME49_223490	1.15	5.93	0.00266602	0.0298377	hypothetical protein
TGME49_283710	-1.20	6.77	0.00266498	0.0298377	Longevity-assurance protein (LAG1) domain-containing protein
TGME49_271960	0.83	7.96	0.00267028	0.02984032	hypothetical protein
TGME49_288360	-1.24	9.40	0.00267705	0.02987085	tryptophanyl-tRNA synthetase (TrpRS2)
TGME49_252630	0.91	8.01	0.00269007	0.02989191	hypothetical protein
TGME49_232450	-1.21	5.88	0.00269507	0.02989191	SWI2/SNF2-containing protein RAD54
TGME49_313600	-1.30	6.04	0.00269455	0.02989191	DDHD domain-containing protein
TGME49_216760	-1.52	5.08	0.0026884	0.02989191	RNA pseudouridine synthase superfamily protein
TGME49_262690	3.11	12.26	0.00270269	0.02993045	ribosomal protein RPL27
TGME49_273950	-1.68	5.27	0.00270698	0.02993045	replication factor C subunit 5, putative
TGME49_293380	-1.76	5.59	0.00271067	0.02993045	histone lysine acetyltransferase HAT1
TGME49_254470	0.95	9.17	0.00274606	0.03023113	hypothetical protein
TGME49_306470	-1.10	6.37	0.00275085	0.03023892	isoprenylcysteine carboxyl methyltransferase (icmt) family protein

TGME49_239560	-1.46	4.92	0.0027634	0.03033185	myosin E
TGME49_286140	-1.40	5.87	0.00278686	0.03050933	hypothetical protein
TGME49_259190	-1.93	4.76	0.0027878	0.03050933	hypothetical protein
TGME49_212130	-1.20	6.44	0.00279207	0.03051101	phospholipase, patatin family protein
TGME49_217600	-0.98	7.07	0.00280103	0.03056387	calcium-dependent protein kinase CDPK9
TGME49_225680	-1.74	5.41	0.00280824	0.03059743	hypothetical protein
TGME49_262700	-1.59	5.53	0.00283059	0.03079563	tetratricopeptide repeat-containing protein
TGME49_217670	-1.35	5.22	0.00285644	0.03103136	ribosomal protein RPS6
TGME49_250880	-0.81	7.84	0.00286191	0.03104525	kinase, pfkB family protein
TGME49_305900	1.13	5.80	0.00289532	0.03136177	hypothetical protein
TGME49_249810	-1.84	4.81	0.00291714	0.03155195	activating signal cointegrator 1 complex subunit 3, putative
TGME49_250955	1.59	5.63	0.00294645	0.03173004	KRUF family protein
TGME49_213030	0.78	7.92	0.00294252	0.03173004	hypothetical protein
TGME49_290940	-0.95	6.90	0.00297498	0.03194443	EMP/nonaspanin domain family protein
TGME49_231120	1.05	5.98	0.00304048	0.0325534	ribosomal protein S11, putative
TGME49_240890	-0.94	6.90	0.0031242	0.03330533	6-phosphofructokinase
TGME49_285830	-1.21	5.93	0.00313617	0.03338312	hypothetical protein
TGME49_251640	-1.29	5.81	0.00314051	0.03338312	ubiquitin-conjugating enzyme subfamily protein
TGME49_241140	-1.55	5.27	0.00314558	0.03338917	DEAD/DEAH box helicase domain-containing protein
TGME49_253360	0.86	7.20	0.00315205	0.03340025	hypothetical protein
TGME49_235490	-0.97	6.76	0.00315565	0.03340025	hypothetical protein
TGME49_300060	1.01	6.85	0.00320896	0.03380203	signal peptidase subunit protein
TGME49_236890	0.88	7.34	0.00321185	0.03380203	hypothetical protein
TGME49_314660	-1.09	6.93	0.00320399	0.03380203	TPRX1 protein
TGME49_323110	1.47	5.68	0.00323952	0.03399665	hypothetical protein
TGME49_297220	-1.93	4.43	0.00325665	0.0340868	AMP-binding enzyme domain-containing protein
TGME49_247370	-1.54	5.12	0.00327287	0.03420125	hypothetical protein
TGME49_207230	0.90	6.18	0.00329626	0.03432208	hypothetical protein
TGME49_289310	-1.11	6.48	0.003295	0.03432208	cullin family protein
TGME49_255290	-2.61	4.89	0.00329833	0.03432208	hypothetical protein
TGME49_261720	-0.99	7.53	0.00331755	0.03437722	metal cation transporter, ZIP family protein
TGME49_207400	0.91	8.64	0.00332596	0.03441622	hypothetical protein
TGME49_315620	-1.02	6.98	0.00336347	0.03470158	vacuolar ATP synthase subunit C, putative
TGME49_314460	-1.55	4.82	0.00336758	0.03470158	hypothetical protein
TGME49_217400	-1.32	6.47	0.00337393	0.0347187	hypothetical protein
TGME49_216630	-1.53	5.26	0.00338134	0.03474664	trigger factor protein, putative
TGME49_297150	-1.26	6.89	0.00339792	0.03478956	MORN repeat-containing protein
TGME49_266070	1.31	10.10	0.00348183	0.03548403	ribosomal protein RPL31
TGME49_281480	1.05	6.08	0.00347312	0.03548403	WD domain, G-beta repeat-containing protein
TGME49_215370	1.03	5.74	0.00347762	0.03548403	hypothetical protein
TGME49_253380	0.81	8.39	0.00349771	0.03554415	AP2 domain transcription factor AP2III-2
TGME49_265790	0.83	8.49	0.00350049	0.03554415	hypothetical protein
TGME49_214980	0.77	7.97	0.00350212	0.03554415	hypothetical protein
TGME49_293780	1.38	7.20	0.00351938	0.0356704	hypothetical protein
TGME49_258980	-1.61	5.28	0.00352847	0.03571369	hypothetical protein
TGME49_309752	0.75	7.99	0.00354434	0.03577345	succinate-Coenzyme A ligase, beta subunit, putative
TGME49_267750	-1.06	6.69	0.00354886	0.03577345	hypothetical protein
TGME49_248950	-1.47	5.20	0.00354502	0.03577345	carrier superfamily protein
TGME49_223040	0.97	7.92	0.0035851	0.03600901	hypothetical protein
TGME49_233500	0.89	7.35	0.00359167	0.03600901	triose-phosphate isomerase TPI-II
TGME49_221410	-1.09	6.55	0.00358995	0.03600901	actin-like protein ALP4

TGME49_255340	-1.29	5.95	0.00358429	0.03600901	tetratricopeptide repeat-containing protein
TGME49_209610	1.07	6.29	0.00360856	0.03608071	oocyst wall protein OWP2
TGME49_310560	-1.83	4.51	0.00360533	0.03608071	hypothetical protein
TGME49_274010	-1.62	5.91	0.00363788	0.03622723	hypothetical protein
TGME49_301216	-2.21	4.54	0.00363216	0.03622723	endonuclease/exonuclease/phosphatase family protein
TGME49_221630	-1.23	5.96	0.00366511	0.03644936	hypothetical protein
TGME49_300130	1.58	4.55	0.00370174	0.03656821	apical membrane antigen 1 domain-containing protein
TGME49_243690	0.80	8.47	0.00370032	0.03656821	hypothetical protein
TGME49_270690	-1.09	6.29	0.00370157	0.03656821	arginyl-tRNA synthetase
TGME49_221230	-1.10	6.64	0.0036863	0.03656821	hypothetical protein
TGME49_320610	-1.49	5.23	0.0036962	0.03656821	hypothetical protein
TGME49_268000	-1.45	4.96	0.0037554	0.03699963	hypothetical protein
TGME49_319590	1.20	5.31	0.00377928	0.03708934	hypothetical protein
TGME49_237015	0.96	7.25	0.00378203	0.03708934	hypothetical protein
TGME49_253990	0.91	6.61	0.00378452	0.03708934	hypothetical protein
TGME49_267350	0.91	7.87	0.0037823	0.03708934	LSM domain-containing protein
TGME49_220140	1.47	5.24	0.0038786	0.0378112	EF hand domain-containing protein
TGME49_215895	0.98	6.10	0.00393147	0.03827633	AP2 domain-containing protein
TGME49_262980	-1.55	5.57	0.0039972	0.03886516	hypothetical protein
TGME49_225080	1.56	10.82	0.00402099	0.03899412	ribosomal protein RPS18
TGME49_264240	-6.12	5.73	0.00403307	0.03906017	hypothetical protein
TGME49_270930	-0.99	6.53	0.00405721	0.03924269	hypothetical protein
TGME49_235905	-1.99	4.68	0.00407918	0.03940371	ribonuclease z, putative
TGME49_249300	-0.75	8.21	0.00409636	0.03951817	hypothetical protein
TGME49_277510	-1.09	6.50	0.00411673	0.0395601	cytoplasmic dynein intermediate chain
TGME49_314540	-1.32	5.49	0.00411413	0.0395601	hypothetical protein
TGME49_250670	1.06	5.81	0.00414224	0.03970224	hypothetical protein
TGME49_321630	-1.02	6.33	0.00413875	0.03970224	RNA recognition motif-containing protein
TGME49_272400	-1.58	5.54	0.00415335	0.03975731	casein kinase ii regulatory subunit protein
TGME49_225050	0.85	7.65	0.00417614	0.03992387	adenosylhomocysteinase, putative
TGME49_313410	-0.90	7.05	0.00419219	0.04002568	proteasome 26S regulatory subunit
TGME49_235020	-0.85	7.55	0.00420065	0.04004776	COPI protein, putative
TGME49_313350	-1.19	5.79	0.00420531	0.04004776	hypothetical protein
TGME49_216880	2.88	12.29	0.00423968	0.04011723	guanine nucleotide-binding protein
TGME49_299780	0.89	8.50	0.00423535	0.04011723	hypothetical protein
TGME49_202300	-1.23	5.80	0.00423217	0.04011723	inosine triphosphate pyrophosphatase, putative
TGME49_249240	1.10	9.83	0.00427828	0.04043088	calmodulin, putative
TGME49_306460	-1.21	5.74	0.00433229	0.04088908	bromodomain-containing protein
TGME49_225160	0.88	7.50	0.00436589	0.0411538	hypothetical protein
TGME49_311140	-1.58	4.90	0.00438284	0.0412611	hypothetical protein
TGME49_237010	-0.94	6.66	0.00438876	0.04126437	hypothetical protein
TGME49_221470	-0.79	8.34	0.0044	0.04131759	hypothetical protein
TGME49_307605	1.08	7.13	0.00440566	0.04131838	hypothetical protein
TGME49_233870	0.98	6.13	0.00441248	0.04133	hypothetical protein
TGME49_254606	1.31	5.91	0.00442462	0.04139146	hypothetical protein
TGME49_307830	-0.80	8.19	0.00443043	0.04139351	hypothetical protein
TGME49_299250	1.06	5.36	0.0044593	0.04155841	hypothetical protein
TGME49_286510	1.10	5.67	0.00449005	0.04174003	hypothetical protein
TGME49_217420	-1.59	5.56	0.00448522	0.04174003	hypothetical protein
TGME49_235140	-0.86	7.38	0.004526	0.04196891	hypothetical protein
TGME49_230705	1.07	6.59	0.0045905	0.04246069	hypothetical protein

TGME49_212310	0.97	9.06	0.00466016	0.04305123	vacuolar ATP synthetase
TGME49_267450	-1.26	6.10	0.00468534	0.04316842	alpha-tubulin suppressor protein
TGME49_297510	-1.52	5.94	0.00468683	0.04316842	hypothetical protein
TGME49_245710	-0.95	7.02	0.00470822	0.04327944	phosphatidylinositol-4-phosphate 5-kinase, putative
TGME49_306330	-1.00	6.56	0.00474588	0.04351763	phospholipase
TGME49_237410	-1.13	6.56	0.00476231	0.04361424	protein phosphatase 2C domain-containing protein
TGME49_246490	-0.91	7.85	0.00481666	0.04405756	hypothetical protein
TGME49_254915	2.20	4.55	0.00483664	0.04413833	hypothetical protein
TGME49_300240	-1.37	5.46	0.00485013	0.04420006	syntaxin 6, n-terminal protein
TGME49_279350	1.20	5.67	0.00489168	0.04452392	hypothetical protein
TGME49_268980	0.80	8.08	0.00491346	0.04461638	hypothetical protein
TGME49_228690	-1.37	5.58	0.00491388	0.04461638	phosphatidylinositol 3- and 4-kinase
TGME49_235700	1.00	6.49	0.00492712	0.04467732	sedoheptulose-1,7-bisphosphatase
TGME49_318510	-1.07	6.14	0.00493266	0.04467732	N-ethylmaleimide-sensitive fusion protein, putative
TGME49_300048	0.86	6.93	0.0049408	0.04469642	hypothetical protein
TGME49_260610	-2.15	4.95	0.00495461	0.04476674	methyltransferase
TGME49_297980	-1.38	5.06	0.00498763	0.04501013	hypothetical protein
TGME49_222960	-1.28	5.86	0.00501344	0.04518803	SCY kinase-related protein (incomplete catalytic triad)
TGME49_219860	-0.82	8.22	0.00502004	0.04519252	replication licensing factor, putative
TGME49_239480	-1.11	5.81	0.00504541	0.04536581	RNB family domain-containing protein
TGME49_273060	1.06	6.18	0.00508309	0.04548382	ribosomal protein S17, putative
TGME49_262500	-1.13	7.65	0.00508259	0.04548382	hypothetical protein
TGME49_257800	-1.66	4.81	0.00508197	0.04548382	polynucleotide adenylyltransferase
TGME49_289640	1.30	5.01	0.00512052	0.04576347	hypothetical protein
TGME49_202980	-1.02	6.64	0.00513971	0.04582441	hypothetical protein
TGME49_218920	0.76	7.67	0.00515033	0.04586395	proteasome subunit beta type, putative
TGME49_217520	0.87	8.97	0.00517759	0.04599613	hypothetical protein
TGME49_276120	1.32	5.87	0.00525844	0.04665401	histone lysine methyltransferase, SET, putative
TGME49_259550	-0.82	7.30	0.00526424	0.04665401	dihydropteroate synthase
TGME49_213790	-1.10	6.02	0.0052952	0.04687234	hypothetical protein
TGME49_209270	-1.44	4.93	0.00531883	0.0469434	hypothetical protein
TGME49_248900	-1.61	5.41	0.00532224	0.0469434	hypothetical protein
TGME49_249740	1.12	6.88	0.00533459	0.04696674	translation machinery associated tma7 protein
TGME49_232320	-1.70	5.09	0.00533756	0.04696674	hypothetical protein
TGME49_238880	1.11	5.65	0.00541453	0.04751466	hypothetical protein
TGME49_293470	-0.93	7.24	0.00543599	0.04760668	hypothetical protein
TGME49_275810	1.36	10.26	0.00546111	0.04770687	ribosomal protein RPS10
TGME49_313160	-1.11	5.70	0.00546675	0.04770687	hypothetical protein
TGME49_278270	-1.58	5.13	0.0054613	0.04770687	nucleolar protein, structural component of H/ACA snoRNPs, putative
TGME49_210370	0.74	8.26	0.00549718	0.04775374	hypothetical protein
TGME49_310930	-0.95	6.60	0.00548474	0.04775374	hypothetical protein
TGME49_320588	-1.36	5.72	0.00548903	0.04775374	glycosyl hydrolases family 35 protein
TGME49_255330	-1.84	4.71	0.0054979	0.04775374	hypothetical protein
TGME49_254480	1.42	5.50	0.00550458	0.04775576	WD domain, G-beta repeat-containing protein
TGME49_254510	1.11	5.21	0.00554367	0.04803865	ankyrin repeat-containing protein
TGME49_240060	0.98	8.72	0.00560032	0.0483599	hypothetical protein
TGME49_297360	-1.00	6.40	0.00567058	0.04885269	hypothetical protein
TGME49_272650	-1.17	6.03	0.00566645	0.04885269	3'5'-cyclic nucleotide phosphodiesterase domain-containing protein
TGME49_203790	0.81	8.37	0.0057149	0.04912029	hypothetical protein
TGME49_218910	1.76	5.41	0.00576249	0.04941465	hypothetical protein

TGME49_209070	0.99	7.02	0.0057572	0.04941465	hypothetical protein
TGME49_233220	-0.83	7.14	0.00577695	0.04943922	hypothetical protein
TGME49_267670	-2.57	4.48	0.0057787	0.04943922	hypothetical protein
TGME49_312360	-1.69	4.61	0.00583116	0.04977316	hypothetical protein
TGME49_242340	0.96	9.48	0.00586215	0.04992261	ribosomal protein RPS29
TGME49_249840	-1.64	5.05	0.00585842	0.04992261	dynein heavy chain 2, putative
TGME49_259700	0.83	7.16	0.00588237	0.04992285	hypothetical protein
TGME49_311890	-0.86	7.52	0.00587494	0.04992285	hypothetical protein
TGME49_215710	-1.14	6.00	0.00588239	0.04992285	hypothetical protein

Supplementary Table S6: Differential gene expression analysis of human neuronal stem cells infected with T. gondii EGS strain for 18 hours.

4.5743113

gene ID	logFC (EGS/CONT)	logCPM	PValue	FDR	Product Name
ENSG00000263006	7.14	4.90	2.53265E-14	6.41653E-12	Rho-associated, coiled-coil containing protein kinase 1 pseudogene 1
ENSG00000133316	6.33	9.48	6.75197E-14	1.50418E-11	WD repeat domain 74
ENSG00000240184	5.51	5.43	1.75437E-56	2.26683E-52	protocadherin gamma subfamily C, 3
ENSG00000099139	4.94	5.51	1.82707E-13	3.68868E-11	proprotein convertase subtilisin/kexin type 5
ENSG00000230453	4.79	5.24	1.68679E-30	4.35901E-27	ankyrin repeat domain 18B
ENSG00000136244	4.71	4.80	4.08203E-36	1.75813E-32	interleukin 6
ENSG00000159251	4.02	4.89	2.28142E-09	1.53745E-07	actin, alpha, cardiac muscle 1
ENSG00000169871	3.97	7.27	1.29031E-42	8.33604E-39	tripartite motif containing 56
ENSG00000109881	3.78	4.72	3.04449E-18	1.78808E-15	coiled-coil domain containing 34
ENSG00000028277	3.74	6.92	5.70037E-34	1.84136E-30	POU class 2 homeobox 2
ENSG00000126709	3.67	8.46	7.25477E-15	2.28631E-12	interferon, alpha-inducible protein 6
ENSG00000152404	3.59	5.31	8.24867E-27	1.52259E-23	CWF19-like 2, cell cycle control (S. pombe)
ENSG00000124212	3.55	5.53	6.15055E-09	3.41231E-07	prostaglandin I2 (prostacyclin) synthase
ENSG00000188483	3.43	5.16	1.09136E-23	1.41014E-20	immediate early response 5-like
ENSG00000204054	3.38	4.82	2.60028E-20	2.09989E-17	long intergenic non-protein coding RNA 963
ENSG00000138061	3.33	5.34	1.09205E-10	1.00788E-08	cytochrome P450, family 1, subfamily B, polypeptide 1
ENSG00000100292	3.33	4.81	1.93971E-13	3.85584E-11	heme oxygenase 1
ENSG00000115380	3.23	4.87	2.09094E-06	5.20485E-05	EGF containing fibulin-like extracellular matrix protein 1
ENSG00000218739	3.17	6.17	3.43121E-26	4.92608E-23	CEBPZ opposite strand
ENSG00000069011	3.14	5.72	1.79761E-08	8.41553E-07	paired-like homeodomain 1
ENSG00000178726	3.11	5.12	1.36376E-08	6.59969E-07	thrombomodulin
ENSG00000143190	2.95	5.78	2.2668E-27	4.88156E-24	POU class 2 homeobox 1
ENSG00000243004	2.92	4.68	2.43906E-17	1.26061E-14	AC005062.2
ENSG00000130513	2.72	5.23	3.46191E-15	1.24254E-12	growth differentiation factor 15
ENSG00000115541	2.68	7.01	1.06876E-22	1.25142E-19	heat shock 10kDa protein 1
ENSG00000136235	2.66	4.58	3.32999E-06	7.50792E-05	glycoprotein (transmembrane) nmb
ENSG00000100448	2.60	5.48	1.93401E-05	0.000335878	cathepsin G
ENSG00000173486	2.60	7.29	1.16222E-22	1.25142E-19	FK506 binding protein 2, 13kDa
ENSG00000267855	2.60	4.84	3.19036E-07	1.04361E-05	NADH dehydrogenase (ubiquinone) 1 alpha subcomplex, 7, 14.5kDa
ENSG00000115461	2.53	8.14	1.02558E-14	3.01172E-12	insulin-like growth factor binding protein 5
ENSG00000102802	2.53	5.54	1.14623E-09	8.37475E-08	mesenteric estrogen-dependent adipogenesis
ENSG00000144034	2.52	4.91	4.59017E-15	1.56078E-12	TP53RK binding protein
ENSG00000139343	2.52	6.03	1.58387E-21	1.57424E-18	small nuclear ribonucleoprotein polypeptide F
ENSG00000197182	2.49	5.03	1.48687E-13	3.09869E-11	MIRLET7B host gene
ENSG00000163319	2.47	5.84	1.15392E-20	9.93988E-18	mitochondrial ribosomal protein S18C
ENSG00000104368	2.46	9.53	7.94152E-08	3.10947E-06	plasminogen activator, tissue
ENSG00000203812	2.44	5.70	4.6968E-19	3.03437E-16	histone cluster 2, H2aa3
ENSG00000239927	2.43	4.91	1.48393E-15	5.81025E-13	ribonuclease P/MRP 21kDa subunit
ENSG00000173221	2.42	5.66	6.2073E-16	2.58724E-13	glutaredoxin (thioltransferase)
ENSG00000179085	2.39	4.78	5.57222E-14	1.30907E-11	dolichyl-phosphate mannosyltransferase polypeptide 3
ENSG00000151366	2.39	7.77	2.76189E-18	1.69935E-15	NADH dehydrogenase (ubiquinone) 1, subcomplex unknown, 2, 14.5kDa
ENSG00000111057	2.38	8.03	5.83129E-14	1.34546E-11	keratin 18, type I
ENSG00000145708	2.36	5.32	3.70415E-06	8.25196E-05	corticotropin releasing hormone binding protein
ENSG00000081870	2.36	5.24	3.46944E-14	8.6209E-12	heat shock protein family B (small), member 11
ENSG00000115977	2.36	5.44	1.30383E-11	1.59898E-09	AP2 associated kinase 1
ENSG00000175768	2.36	6.22	1.07324E-15	4.33354E-13	translocase of outer mitochondrial membrane 5 homolog (yeast)
ENSG00000153006	2.34	5.92	3.50343E-19	2.38252E-16	SREK1-interacting protein 1
ENSG00000173207	2.30	4.79	2.67472E-13	5.00871E-11	CDC28 protein kinase regulatory subunit 1B
ENSG00000185068	2.29	6.68	1.88907E-19	1.43581E-16	GTF2H5
ENSG00000134597	2.28	5.17	1.44807E-13	3.06731E-11	RNA binding motif protein, X-linked 2
ENSG00000182004	2.26	6.40	2.07231E-19	1.48757E-16	small nuclear ribonucleoprotein polypeptide E
ENSG00000234545	2.26	5.13	7.38553E-09	3.9271E-07	family with sequence similarity 133, member B
ENSG00000148671	2.22	5.56	6.97624E-09	3.74025E-07	adipogenesis regulatory factor
ENSG00000041982	2.22	7.23	5.37792E-13	9.39029E-11	tenascin C
ENSG00000070814	2.22	6.51	1.13334E-16	5.0496E-14	Treacher Collins-Franceschetti syndrome 1
ENSG00000075826	2.22	5.84	2.73292E-08	1.21347E-06	SEC31 homolog B, COPII coating complex component
ENSG00000166803	2.21	5.26	3.84448E-12	5.32615E-10	KIAA0101
ENSG00000101745	2.19	6.32	8.45176E-15	2.60012E-12	ankyrin repeat domain 12

ENSG00000138433	2.18	6.13	3.45603E-17	1.71751E-14	corepressor interacting with RBPJ, 1
ENSG00000244509	2.18	7.30	7.49569E-11	7.39327E-09	apolipoprotein B mRNA editing enzyme, catalytic polypeptide-like 3C
ENSG00000166012	2.17	7.41	3.61373E-07	1.16732E-05	TATA box binding protein (TBP)-associated factor, RNA polymerase I, D, 41kDa
ENSG00000008952	2.15	8.30	4.18534E-12	5.6925E-10	SEC62 homolog (S. cerevisiae)
ENSG00000243147	2.14	6.61	3.21975E-18	1.8088E-15	mitochondrial ribosomal protein L33
ENSG00000158417	2.14	8.45	2.00635E-13	3.92788E-11	eukaryotic translation initiation factor 5B
ENSG00000141905	2.13	7.64	9.456E-15	2.84142E-12	nuclear factor I/C (CCAAT-binding transcription factor)
ENSG00000106591	2.13	6.17	6.22481E-18	3.35128E-15	mitochondrial ribosomal protein L32
ENSG00000109099	2.11	5.71	3.43432E-13	6.24998E-11	peripheral myelin protein 22
ENSG00000187608	2.10	7.90	6.00322E-12	7.83511E-10	ISG15 ubiquitin-like modifier
ENSG00000143977	2.10	6.46	1.76529E-15	6.51694E-13	small nuclear ribonucleoprotein polypeptide G
ENSG00000132432	2.10	8.14	1.50969E-14	4.15037E-12	Sec61 gamma subunit
ENSG00000164182	2.09	5.83	2.11812E-13	4.08482E-11	NADH dehydrogenase (ubiquinone) complex I, assembly factor 2
ENSG00000132963	2.08	8.33	1.78505E-13	3.66105E-11	proteasome maturation protein
ENSG00000164171	2.08	5.01	1.16153E-08	5.83975E-07	integrin, alpha 2 (CD49B, alpha 2 subunit of VLA-2 receptor)
ENSG00000120688	2.06	5.13	1.84963E-10	1.59327E-08	WW domain binding protein 4
ENSG00000134825	2.06	6.57	1.2865E-16	5.54095E-14	transmembrane protein 258
ENSG00000008394	2.05	7.13	2.65745E-13	5.00871E-11	microsomal glutathione S-transferase 1
ENSG00000152518	2.03	4.78	2.47716E-09	1.64141E-07	ZFP36 ring finger protein-like 2
ENSG00000135637	2.03	6.15	6.44284E-15	2.0812E-12	coiled-coil domain containing 142
ENSG00000213741	2.02	9.15	1.59012E-10	1.40726E-08	ribosomal protein S29
ENSG00000184752	2.00	6.80	5.92046E-15	1.96149E-12	NADH dehydrogenase (ubiquinone) 1 alpha subcomplex, 12
ENSG00000262771	1.98	7.09	2.20075E-14	5.80324E-12	single-stranded DNA binding protein 1, mitochondrial
ENSG00000142396	1.98	4.87	9.76173E-11	9.27437E-09	endogenous retrovirus group K3, member 1
ENSG00000116717	1.98	7.83	1.13936E-13	2.45362E-11	growth arrest and DNA-damage-inducible, alpha
ENSG00000162385	1.98	4.86	2.28458E-09	1.53745E-07	mago homolog, exon junction complex core component
ENSG00000177954	1.98	9.03	4.09699E-10	3.30857E-08	ribosomal protein S27
ENSG00000108691	1.98	5.70	9.46627E-09	4.85371E-07	chemokine (C-C motif) ligand 2
ENSG00000203875	1.98	5.84	6.68499E-14	1.50418E-11	small nucleolar RNA host gene 5
ENSG00000143771	1.96	6.37	4.38785E-15	1.53231E-12	cornichon family AMPA receptor auxiliary protein 4
ENSG00000128524	1.96	7.68	6.9337E-12	8.95904E-10	ATPase, H+ transporting, lysosomal 14kDa, V1 subunit F
ENSG00000198959	1.96	6.02	7.08791E-05	0.000985822	transglutaminase 2
ENSG00000235385	1.95	5.17	0.000854403	0.007272559	GS1-600G8.5
ENSG00000179958	1.94	5.38	5.7602E-11	6.00222E-09	dCTP pyrophosphatase 1
ENSG00000168003	1.94	7.50	1.54161E-12	2.42916E-10	solute carrier family 3 (amino acid transporter heavy chain), member 2
ENSG00000171858	1.93	8.67	9.30779E-11	8.91949E-09	ribosomal protein S21
ENSG00000198258	1.92	7.95	1.79808E-12	2.79916E-10	ubiquitin-like 5
ENSG00000173436	1.91	7.44	9.98563E-14	2.18685E-11	mitochondrial inner membrane organizing system 1
ENSG00000197323	1.91	6.34	1.13085E-10	1.03629E-08	tripartite motif containing 33
ENSG00000163156	1.91	5.61	4.69074E-12	6.1846E-10	sodium channel modifier 1
ENSG00000143256	1.91	6.23	1.30676E-14	3.67057E-12	prefoldin subunit 2
ENSG00000112769	1.91	4.93	0.000259545	0.002781089	laminin, alpha 4
ENSG00000113811	1.90	6.10	1.92631E-14	5.18538E-12	selenoprotein K
ENSG00000082515	1.89	5.00	6.37986E-10	5.02648E-08	mitochondrial ribosomal protein L22
ENSG00000109534	1.88	4.86	7.78797E-10	6.02565E-08	GAR1 homolog, ribonucleoprotein
ENSG00000127922	1.88	6.78	5.09137E-14	1.21825E-11	split hand/foot malformation (ectrodactyly) type 1
ENSG00000198211	1.88	6.41	4.37852E-13	7.85762E-11	tubulin, beta 3 class III
ENSG00000090263	1.87	5.60	6.4075E-11	6.57074E-09	mitochondrial ribosomal protein S33
ENSG00000217930	1.87	4.71	1.82383E-09	1.26698E-07	presequence translocase-associated motor 16 homolog (S. cerevisiae)
ENSG00000123975	1.87	5.41	3.94632E-09	2.39672E-07	CDC28 protein kinase regulatory subunit 2
ENSG00000104825	1.86	5.77	2.52837E-10	2.13524E-08	nuclear factor of kappa light polypeptide gene enhancer in B-cells inhibitor, beta
ENSG00000229833	1.86	5.16	1.33695E-08	6.53829E-07	PET100 homolog
ENSG00000123353	1.86	6.46	1.14166E-14	3.27809E-12	ORMDL sphingolipid biosynthesis regulator 2
ENSG00000167779	1.86	5.99	4.83586E-11	5.12165E-09	insulin-like growth factor binding protein 6
ENSG00000147862	1.86	5.11	1.05798E-07	3.99713E-06	nuclear factor I/B
ENSG00000177426	1.85	5.15	1.31109E-10	1.18465E-08	TGFB-induced factor homeobox 1
ENSG00000080823	1.85	4.85	1.08361E-06	2.99815E-05	MOK protein kinase
ENSG00000137815	1.82	7.04	2.79064E-12	4.00643E-10	RTF1 homolog, Paf1/RNA polymerase II complex component
ENSG00000186010	1.82	7.16	1.13991E-09	8.37475E-08	NADH dehydrogenase (ubiquinone) 1 alpha subcomplex, 13
ENSG00000127540	1.82	6.49	1.29054E-12	2.08438E-10	ubiquinol-cytochrome c reductase, complex III subunit XI
ENSG00000214022	1.82	4.60	4.66084E-08	1.97451E-06	replication initiator 1
ENSG00000154723	1.81	7.31	8.27462E-13	1.35337E-10	ATP synthase, H+ transporting, mitochondrial Fo complex, subunit F6
ENSG00000103121	1.81	5.41	6.64039E-11	6.75595E-09	C-x(9)-C motif containing 2
ENSG00000130402	1.80	11.77	6.71276E-06	0.000137894	actinin, alpha 4
ENSG00000125676	1.80	6.79	1.44811E-09	1.03376E-07	THO complex 2
ENSG00000111832	1.79	6.85	4.34468E-10	3.48681E-08	RWD domain containing 1
ENSG00000176731	1.78	5.48	6.76664E-11	6.8306E-09	chromosome 8 open reading frame 59
ENSG00000198498	1.78	4.83	2.68296E-08	1.19874E-06	translation machinery associated 16 homolog (S. cerevisiae)
ENSG00000135931	1.76	6.11	1.85386E-12	2.81808E-10	armadillo repeat containing 9

ENSG00000185088	1.76	8.31	9.6579E-10	7.29764E-08	ribosomal protein S27-like
ENSG00000174917	1.75	5.99	3.30511E-10	2.72238E-08	chromosome 19 open reading frame 70
ENSG00000131469	1.75	10.34	9.73424E-07	2.74022E-05	ribosomal protein L27
ENSG00000177410	1.74	6.33	4.80787E-13	8.50992E-11	ZNFX1 antisense RNA 1
ENSG00000175063	1.74	4.66	2.28524E-06	5.55707E-05	ubiquitin-conjugating enzyme E2C
ENSG00000174720	1.74	6.08	1.36113E-11	1.64366E-09	La ribonucleoprotein domain family, member 7
ENSG00000154582	1.74	7.10	1.31175E-11	1.59898E-09	transcription elongation factor B (SIII), polypeptide 1 (15kDa, elongin C)
ENSG00000134248	1.73	7.03	1.79954E-11	2.15295E-09	late endosomal/lysosomal adaptor, MAPK and MTOR activator 5
ENSG00000171303	1.73	8.28	3.7008E-09	2.31005E-07	potassium channel, two pore domain subfamily K, member 3
ENSG00000111331	1.73	4.59	0.000392631	0.003867238	2'-5'-oligoadenylate synthetase 3, 100kDa
ENSG00000171246	1.72	6.48	1.02597E-08	5.20688E-07	neuronal pentraxin I
ENSG00000105185	1.72	6.86	4.43008E-12	5.90114E-10	programmed cell death 5
ENSG00000101966	1.71	6.67	1.42765E-10	1.28102E-08	X-linked inhibitor of apoptosis, E3 ubiquitin protein ligase
ENSG00000165233	1.71	5.76	1.88955E-10	1.61688E-08	chromosome 9 open reading frame 89
ENSG00000148688	1.71	4.95	6.0209E-09	3.36779E-07	ribonuclease P/MRP 30kDa subunit
ENSG00000130770	1.71	7.44	2.65618E-11	3.03721E-09	ATPase inhibitory factor 1
ENSG00000139168	1.71	6.01	1.7991E-10	1.56014E-08	zinc finger CCHC-type and RNA binding motif 1
ENSG00000137965	1.71	5.35	1.18221E-06	3.23631E-05	interferon-induced protein 44
ENSG00000161011	1.70	8.65	5.82501E-06	0.000122183	sequestosome 1
ENSG00000107984	1.69	5.45	9.79136E-08	3.75413E-06	dickkopf WNT signaling pathway inhibitor 1
ENSG00000146066	1.69	5.96	2.79636E-11	3.16945E-09	HIG1 hypoxia inducible domain family, member 2A
ENSG00000214413	1.69	4.67	7.30259E-08	2.92126E-06	BBSome interacting protein 1
ENSG00000165389	1.68	5.31	1.34601E-08	6.53829E-07	serine palmitoyltransferase, small subunit A
ENSG00000203805	1.67	4.64	1.69456E-05	0.000299117	phosphatidic acid phosphatase type 2 domain containing 1A
ENSG00000226710	1.67	7.61	1.73928E-10	1.51846E-08	chromosome 6 open reading frame 48
ENSG00000162772	1.66	6.18	1.19409E-08	5.96566E-07	activating transcription factor 3
ENSG00000163827	1.66	5.27	6.74184E-07	2.04968E-05	leucine rich repeat containing 2
ENSG00000213853	1.66	5.85	3.81847E-10	3.10305E-08	epithelial membrane protein 2
ENSG00000133169	1.65	6.68	2.77491E-07	9.27325E-06	brain expressed, X-linked 1
ENSG00000060339	1.65	6.99	4.88523E-09	2.8562E-07	cell division cycle and apoptosis regulator 1
ENSG00000114784	1.65	5.31	5.75171E-09	3.23121E-07	eukaryotic translation initiation factor 1B
ENSG00000196352	1.65	4.73	1.00164E-05	0.000191452	CD55 molecule, decay accelerating factor for complement (Cromer blood group)
ENSG00000182899	1.64	9.43	9.86414E-07	2.77075E-05	ribosomal protein L35a
ENSG00000125977	1.64	8.01	9.36117E-08	3.61062E-06	eukaryotic translation initiation factor 2, subunit 2 beta, 38kDa
ENSG00000147669	1.64	6.18	3.77151E-11	4.1298E-09	polymerase (RNA) II (DNA directed) polypeptide K, 7.0kDa
ENSG00000100902	1.64	7.37	4.15264E-09	2.49564E-07	proteasome (prosome, macropain) subunit, alpha type, 6
ENSG00000105825	1.63	6.85	8.19816E-09	4.25415E-07	tissue factor pathway inhibitor 2
ENSG00000185112	1.63	5.63	1.88039E-06	4.7454E-05	family with sequence similarity 43, member A
ENSG00000214046	1.62	6.25	2.64304E-11	3.03721E-09	small integral membrane protein 7
ENSG00000125743	1.62	7.85	3.66803E-09	2.30071E-07	small nuclear ribonucleoprotein D2 polypeptide 16.5kDa
ENSG00000119705	1.61	6.13	2.88845E-10	2.40785E-08	SRA stem-loop interacting RNA binding protein
ENSG00000148690	1.61	5.04	2.57854E-07	8.67639E-06	fragile site, folic acid type, rare, fra(10)(q23.3) or fra(10)(q24.2) candidate 1
ENSG00000145354	1.61	5.65	9.15833E-10	6.96087E-08	CDGSH iron sulfur domain 2
ENSG00000104763	1.61	6.91	4.3423E-07	1.36361E-05	N-acylsphingosine amidohydrolase (acid ceramidase) 1
ENSG00000160799	1.61	4.81	2.37558E-07	8.09891E-06	coiled-coil domain containing 12
ENSG00000227051	1.61	7.03	1.0404E-10	9.7413E-09	chromosome 14 open reading frame 132
ENSG00000171453	1.61	4.81	1.10626E-07	4.15523E-06	polymerase (RNA) I polypeptide C, 30kDa
ENSG00000147535	1.61	4.60	6.53507E-07	1.99621E-05	phosphatidic acid phosphatase type 2 domain containing 1B
ENSG00000136897	1.60	4.96	7.68263E-08	3.04501E-06	mitochondrial ribosomal protein L50
ENSG00000196199	1.60	6.07	2.03952E-07	7.14164E-06	M-phase phosphoprotein 8
ENSG00000130332	1.60	5.55	1.30667E-09	9.39187E-08	LSM7 homolog, U6 small nuclear RNA and mRNA degradation associated
ENSG00000099337	1.60	6.33	1.34349E-08	6.53829E-07	potassium channel, two pore domain subfamily K, member 6
ENSG00000173113	1.60	7.11	1.65293E-09	1.16708E-07	tRNA methyltransferase 11-2 homolog (S. cerevisiae)
ENSG00000273397	1.59	6.55	7.88431E-11	7.71766E-09	NADH dehydrogenase (ubiquinone) 1 alpha subcomplex, 6, 14kDa
ENSG00000254999	1.59	8.28	8.12302E-09	4.24929E-07	BRICK1, SCAR/WAVE actin-nucleating complex subunit
ENSG00000282031	1.59	5.50	1.81872E-07	6.45594E-06	transmembrane BAX inhibitor motif containing 4
ENSG00000119878	1.58	4.96	9.89984E-08	3.78449E-06	cysteine-rich PDZ-binding protein
ENSG00000151461	1.58	6.21	6.63255E-09	3.616E-07	UPF2 regulator of nonsense transcripts homolog (yeast)
ENSG00000143158	1.57	6.87	2.44593E-09	1.62906E-07	mitochondrial pyruvate carrier 2
ENSG00000166347	1.57	5.13	8.64979E-08	3.34622E-06	cytochrome b5 type A (microsomal)
ENSG00000161980	1.57	4.65	2.06926E-06	5.18157E-05	polymerase (RNA) III (DNA directed) polypeptide K, 12.3 kDa
ENSG00000270898	1.57	4.57	1.25034E-06	3.40118E-05	GPR75-ASB3 readthrough
ENSG00000167863	1.57	7.60	1.93148E-09	1.32748E-07	ATP synthase, H+ transporting, mitochondrial Fo complex, subunit d
ENSG00000101361	1.57	7.35	7.79114E-09	4.12579E-07	NOP56 ribonucleoprotein
ENSG00000156603	1.56	4.97	3.1018E-07	1.02241E-05	mediator complex subunit 19
ENSG00000120963	1.56	5.45	5.18961E-09	2.98497E-07	zinc finger protein 706
ENSG00000112312	1.56	4.60	8.13621E-07	2.37309E-05	geminin, DNA replication inhibitor
ENSG00000146425	1.56	6.70	5.29409E-09	3.01343E-07	dynein, light chain, Tctex-type 1
ENSG00000145050	1.56	6.70	2.21771E-09	1.50816E-07	mesencephalic astrocyte-derived neurotrophic factor

ENSG00000060762	1.56	5.08	6.02293E-08	2.48633E-06	mitochondrial pyruvate carrier 1
ENSG00000065518	1.55	6.64	2.69608E-10	2.26208E-08	NADH dehydrogenase (ubiquinone) 1 beta subcomplex, 4, 15kDa
ENSG00000189403	1.55	8.14	6.20538E-08	2.53077E-06	high mobility group box 1
ENSG00000185043	1.55	6.91	3.3079E-10	2.72238E-08	calcium and integrin binding 1 (calmyrin)
ENSG00000136819	1.55	6.45	2.21489E-09	1.50816E-07	chromosome 9 open reading frame 78
ENSG00000172809	1.54	8.62	1.04129E-07	3.95074E-06	ribosomal protein L38
ENSG00000197063	1.54	6.70	3.86894E-07	1.23828E-05	v-maf avian musculoaponeurotic fibrosarcoma oncogene homolog G
ENSG00000133398	1.54	5.59	3.95095E-09	2.39672E-07	mediator complex subunit 10
ENSG00000104332	1.54	6.50	8.37926E-05	0.001133701	secreted frizzled-related protein 1
ENSG00000186577	1.54	4.96	1.0152E-06	2.82986E-05	chromosome 6 open reading frame 1
ENSG00000176953	1.54	5.90	2.41587E-07	8.19304E-06	nuclear factor of activated T-cells, cytoplasmic, calcineurin-dependent 2 interacting protein
ENSG00000273686	1.53	11.26	0.000165199	0.001967399	beta-2-microglobulin
ENSG00000163170	1.53	4.98	2.77745E-07	9.27325E-06	bolA family member 3
ENSG00000135185	1.53	4.60	1.50642E-06	3.91639E-05	transmembrane protein 243, mitochondrial
ENSG00000180992	1.53	6.19	4.1957E-09	2.50331E-07	mitochondrial ribosomal protein L14
ENSG00000245910	1.53	5.99	1.06339E-09	7.89658E-08	small nucleolar RNA host gene 6
ENSG00000120875	1.52	7.94	7.80707E-08	3.07546E-06	dual specificity phosphatase 4
ENSG00000116473	1.52	5.84	2.57786E-09	1.69079E-07	RAP1A, member of RAS oncogene family
ENSG00000163125	1.52	5.71	5.19789E-09	2.98497E-07	regulation of nuclear pre-mRNA domain containing 2
ENSG00000078124	1.52	4.97	4.34801E-07	1.36361E-05	alkaline ceramidase 3
ENSG00000205981	1.52	4.90	2.30276E-07	7.91328E-06	DnaJ (Hsp40) homolog, subfamily C, member 19
ENSG00000275724	1.51	6.05	1.88752E-09	1.30421E-07	NADH dehydrogenase (Ubiquinone) 1 alpha subcomplex, 3, 9kDa, isoform CRA_e; NDUFA3 protein; cDNA FLJ76508, highly similar to Homo sapiens NADH dehydrogenase (ubiquinone) 1 alpha subcomplex, 3, 9kDa (NDUFA3), mRNA
ENSG00000172336	1.51	5.14	1.36457E-07	5.08116E-06	POP7 homolog, ribonuclease P/MRP subunit
ENSG00000139233	1.51	5.93	3.73244E-09	2.3186E-07	LLP homolog, long-term synaptic facilitation (Aplysia)
ENSG00000164258	1.50	6.59	3.80182E-09	2.3392E-07	NADH dehydrogenase (ubiquinone) Fe-S protein 4, 18kDa (NADH-coenzyme Q reductase)
ENSG00000197956	1.50	12.06	9.17083E-05	0.001209145	S100 calcium binding protein A6
ENSG00000145247	1.50	5.61	6.69716E-09	3.63588E-07	OCIA domain containing 2
ENSG00000117614	1.50	5.68	6.29487E-09	3.4759E-07	SYF2 pre-mRNA-splicing factor
ENSG00000141837	1.50	5.33	0.000106454	0.001370012	calcium channel, voltage-dependent, P/Q type, alpha 1A subunit
ENSG00000278461	1.50	5.31	2.43928E-07	8.25076E-06	mitochondrial ribosomal protein S36
ENSG00000120533	1.49	6.43	1.04853E-09	7.83126E-08	enhancer of yellow 2 homolog (Drosophila)
ENSG00000113732	1.49	8.88	1.6765E-07	5.98398E-06	ATPase, H+ transporting, lysosomal 9kDa, V0 subunit e1
ENSG00000100142	1.48	5.92	6.41E-09	3.52441E-07	polymerase (RNA) II (DNA directed) polypeptide F
ENSG00000180573	1.48	6.06	4.68083E-09	2.74914E-07	histone cluster 1, H2ac
ENSG00000122641	1.48	9.21	6.87604E-06	0.000140801	inhibin, beta A
ENSG00000197070	1.48	4.77	5.35754E-06	0.00011367	arrestin domain containing 1
ENSG00000125356	1.47	7.18	8.16959E-09	4.25415E-07	NADH dehydrogenase (ubiquinone) 1 alpha subcomplex, 1, 7.5kDa
ENSG00000255717	1.47	5.20	3.01077E-05	0.000482821	small nucleolar RNA host gene 1
ENSG00000124172	1.47	8.64	1.83656E-07	6.50141E-06	ATP synthase, H+ transporting, mitochondrial F1 complex, epsilon subunit
ENSG00000189043	1.46	7.19	1.67374E-07	5.98398E-06	NDUFA4, mitochondrial complex associated
ENSG00000183648	1.45	6.24	1.58763E-09	1.12713E-07	NADH dehydrogenase (ubiquinone) 1 beta subcomplex, 1, 7kDa
ENSG00000197903	1.45	6.71	2.19042E-08	1.00009E-06	histone cluster 1, H2bk
ENSG00000225921	1.45	6.60	1.47373E-08	7.07882E-07	nucleolar protein 7, 27kDa
ENSG00000107833	1.45	4.68	2.77287E-05	0.00044954	nucleophosmin/nucleoplasmin 3
ENSG00000171735	1.45	5.35	1.50469E-07	5.50767E-06	calmodulin binding transcription activator 1
ENSG00000111596	1.45	5.98	1.02759E-08	5.20688E-07	CCR4-NOT transcription complex, subunit 2
ENSG00000125971	1.45	7.61	2.36315E-08	1.06763E-06	dynein, light chain, roadblock-type 1
ENSG00000175550	1.44	8.77	1.31175E-06	3.51641E-05	DR1-associated protein 1 (negative cofactor 2 alpha)
ENSG00000124882	1.44	6.09	2.06346E-07	7.18651E-06	epiregulin
ENSG00000156482	1.43	9.33	1.12744E-05	0.000209005	ribosomal protein L30
ENSG00000136770	1.43	5.12	7.39728E-07	2.19725E-05	DnaJ (Hsp40) homolog, subfamily C, member 1
ENSG00000175606	1.43	4.81	2.15615E-06	5.33708E-05	transmembrane protein 70
ENSG00000167772	1.43	5.75	2.59032E-05	0.00042821	angiopoietin-like 4
ENSG00000124357	1.42	7.43	1.31952E-08	6.4827E-07	N-acetylglucosamine kinase
ENSG00000164114	1.42	4.68	1.48149E-05	0.000265865	microtubule-associated protein 9
ENSG00000130311	1.42	7.36	1.8368E-08	8.56798E-07	DET1 and DDB1 associated 1
ENSG00000100836	1.42	6.99	2.25074E-08	1.02042E-06	poly(A) binding protein, nuclear 1
ENSG00000055044	1.42	6.43	1.8711E-06	4.7405E-05	NOP58 ribonucleoprotein
ENSG00000235162	1.42	7.14	2.46479E-08	1.10967E-06	chromosome 12 open reading frame 75
ENSG00000221983	1.41	9.51	6.45217E-06	0.000133603	ubiquitin A-52 residue ribosomal protein fusion product 1
ENSG00000138814	1.40	5.62	9.67261E-06	0.00018543	protein phosphatase 3, catalytic subunit, alpha isozyme
ENSG00000176788	1.39	8.32	2.18697E-06	5.38245E-05	brain abundant, membrane attached signal protein 1
ENSG00000138398	1.39	6.40	6.20891E-08	2.53077E-06	peptidylprolyl isomerase G (cyclophilin G)
ENSG00000169715	1.39	7.76	6.87469E-07	2.08028E-05	metallothionein 1E
ENSG00000163463	1.39	6.03	1.36637E-06	3.64019E-05	keratinocyte associated protein 2

ENSG00000170088	1.38	5.27	7.37427E-07	2.19546E-05	transmembrane protein 192
ENSG00000178127	1.38	6.81	4.69544E-08	1.98267E-06	NADH dehydrogenase (ubiquinone) flavoprotein 2, 24kDa
ENSG00000130066	1.38	7.81	8.02644E-06	0.00015882	spermidine/spermine N1-acetyltransferase 1
ENSG00000106803	1.38	7.33	1.40435E-07	5.19931E-06	Sec61 beta subunit
ENSG00000178896	1.38	4.95	3.04018E-06	6.98879E-05	exosome component 4
ENSG00000136888	1.37	7.20	3.87171E-07	1.23828E-05	ATPase, H+ transporting, lysosomal 13kDa, V1 subunit G1
ENSG00000067082	1.37	8.47	2.34374E-06	5.67107E-05	Kruppel-like factor 6
ENSG00000071462	1.37	6.54	9.40378E-09	4.84088E-07	Williams Beuren syndrome chromosome region 22
ENSG00000183726	1.37	7.75	1.10229E-07	4.15238E-06	transmembrane protein 50A
ENSG00000111237	1.37	6.77	1.85823E-08	8.60582E-07	VPS29 retromer complex component
ENSG00000164405	1.37	7.28	7.60617E-08	3.02398E-06	ubiquinol-cytochrome c reductase, complex III subunit VII, 9.5kDa
ENSG00000109390	1.37	6.30	3.68001E-08	1.59028E-06	NADH dehydrogenase (ubiquinone) 1, subcomplex unknown, 1, 6kDa
ENSG00000115363	1.37	5.80	3.61323E-07	1.16732E-05	eva-1 homolog A (C. elegans)
ENSG00000100889	1.37	4.70	8.24528E-05	0.001118228	phosphoenolpyruvate carboxykinase 2 (mitochondrial)
ENSG00000228253	1.36	10.31	4.12574E-05	0.000638427	MT-ATP8
ENSG00000104823	1.35	5.73	6.59116E-07	2.00859E-05	enoyl CoA hydratase 1, peroxisomal
ENSG00000100028	1.35	6.80	2.89933E-08	1.27423E-06	small nuclear ribonucleoprotein D3 polypeptide 18kDa
ENSG00000112667	1.35	4.70	2.02396E-05	0.000348688	2'-deoxynucleoside 5'-phosphate N-hydrolase 1
ENSG00000188486	1.34	5.68	2.62756E-05	0.000431943	H2A histone family, member X
ENSG00000232388	1.34	4.74	1.48557E-05	0.000266228	long intergenic non-protein coding RNA 493
ENSG00000184557	1.34	4.96	0.000193287	0.002229875	suppressor of cytokine signaling 3
ENSG00000146386	1.34	4.79	3.65544E-05	0.000573203	ABRA C-terminal like
ENSG00000131586	1.34	6.61	6.16268E-08	2.52787E-06	MRPL20
ENSG00000171813	1.34	4.60	7.46108E-05	0.001029407	PWWP domain containing 2B
ENSG00000111231	1.33	4.79	1.15767E-05	0.000213996	GPN-loop GTPase 3
ENSG00000136213	1.33	4.89	5.5654E-06	0.000117118	carbohydrate (chondroitin 4) sulfotransferase 12
ENSG00000105849	1.33	5.21	0.000499462	0.004707179	TWIST neighbor
ENSG00000228474	1.33	8.35	1.78934E-06	4.54226E-05	oligosaccharyltransferase complex subunit 4 (non-catalytic)
ENSG00000125870	1.33	6.10	2.1506E-07	7.46986E-06	small nuclear ribonucleoprotein polypeptide B
ENSG00000171204	1.32	5.34	2.62428E-06	6.19506E-05	transmembrane protein 126B
ENSG00000175727	1.32	6.01	1.59706E-06	4.13539E-05	MLX interacting protein
ENSG00000138175	1.32	5.95	8.0701E-07	2.36448E-05	ADP-ribosylation factor-like 3
ENSG00000134049	1.32	5.98	1.17202E-06	3.21522E-05	immediate early response 3 interacting protein 1
ENSG00000171848	1.32	5.94	1.75559E-05	0.000308626	ribonucleotide reductase M2
ENSG00000105518	1.31	4.86	3.22292E-05	0.000512849	transmembrane protein 205
ENSG00000172465	1.31	4.95	5.79338E-06	0.000121717	transcription elongation factor A (SII)-like 1
ENSG00000108010	1.31	6.67	1.52827E-07	5.5476E-06	glutaredoxin 3
ENSG00000272835	1.31	4.74	2.25643E-05	0.000383119	single-pass membrane protein with aspartate-rich tail 1
ENSG00000162520	1.31	5.98	1.84558E-05	0.000322688	syncoilin, intermediate filament protein
ENSG00000136810	1.31	8.25	1.89512E-06	4.77327E-05	thioredoxin
ENSG00000206408	1.31	4.76	2.64993E-05	0.000434514	G patch domain and ankyrin repeats 1
ENSG00000118267	1.30	5.70	4.10488E-07	1.30317E-05	ZNF271
ENSG00000165502	1.30	7.82	1.00408E-06	2.80817E-05	ribosomal protein L36a-like
ENSG00000111364	1.30	4.85	3.23588E-05	0.000514278	DEAD (Asp-Glu-Ala-Asp) box polypeptide 55
ENSG00000197019	1.30	5.04	5.92697E-06	0.00012412	SERTA domain containing 1
ENSG00000167747	1.30	5.19	0.000163773	0.001961138	chromosome 19 open reading frame 48
ENSG00000173915	1.30	7.15	1.66721E-07	5.98398E-06	up-regulated during skeletal muscle growth 5 homolog (mouse)
ENSG00000095066	1.30	5.04	9.42263E-05	0.001236381	hook microtubule-tethering protein 2
ENSG00000143106	1.30	7.52	1.30185E-06	3.50441E-05	proteasome (prosome, macropain) subunit, alpha type, 5
ENSG00000172977	1.29	5.27	4.7277E-05	0.000711965	K(lysine) acetyltransferase 5
ENSG00000105829	1.29	5.29	8.36333E-06	0.000163731	Bet1 golgi vesicular membrane trafficking protein
ENSG00000108175	1.29	5.67	0.000150716	0.001826828	zinc finger, MIZ-type containing 1
ENSG00000101132	1.29	4.65	4.40512E-05	0.000672001	prefoldin subunit 4
ENSG00000172586	1.29	5.15	1.27998E-05	0.000234257	coiled-coil-helix-coiled-coil-helix domain containing 1
ENSG00000165782	1.28	5.34	3.05345E-06	6.99532E-05	transmembrane protein 55B
ENSG00000196507	1.28	6.74	1.4794E-07	5.43048E-06	transcription elongation factor A (SII)-like 3
ENSG00000070010	1.28	6.72	2.16854E-07	7.4919E-06	ubiquitin fusion degradation 1 like (yeast)
ENSG00000150551	1.28	7.45	1.68744E-06	4.32607E-05	LY6/PLAUR domain containing 1
ENSG00000162300	1.28	5.48	6.90155E-06	0.000140877	zinc finger protein-like 1
ENSG00000090266	1.28	7.35	3.63626E-07	1.17118E-05	NADH dehydrogenase (ubiquinone) 1 beta subcomplex, 2, 8kDa
ENSG00000005075	1.28	6.09	2.72536E-07	9.14658E-06	polymerase (RNA) II (DNA directed) polypeptide J, 13.3kDa
ENSG00000125691	1.27	9.99	0.000159684	0.001921116	ribosomal protein L23
ENSG00000160049	1.27	6.48	9.47734E-07	2.67373E-05	DNA fragmentation factor, 45kDa, alpha polypeptide
ENSG00000183527	1.27	5.40	3.33531E-06	7.50792E-05	proteasome (prosome, macropain) assembly chaperone 1
ENSG00000168653	1.27	7.99	2.52091E-06	6.01722E-05	NADH dehydrogenase (ubiquinone) Fe-S protein 5, 15kDa (NADH-coenzyme Q reductase)
ENSG00000137806	1.27	4.77	5.08802E-05	0.000754405	NADH dehydrogenase (ubiquinone) complex I, assembly factor 1
ENSG00000134107	1.27	5.69	1.44715E-06	3.80827E-05	basic helix-loop-helix family, member e40
ENSG00000170791	1.27	5.59	1.72838E-06	4.42226E-05	coiled-coil-helix-coiled-coil-helix domain containing 7

ENSG00000101182	1.26	8.09	6.39302E-06	0.000132591	proteasome (prosome, macropain) subunit, alpha type, 7
ENSG00000115350	1.26	5.74	1.16838E-06	3.21204E-05	polymerase (DNA-directed), epsilon 4, accessory subunit
ENSG00000171202	1.26	4.97	2.51222E-05	0.000418305	transmembrane protein 126A
ENSG00000008988	1.26	9.54	7.86267E-05	0.001076203	ribosomal protein S20
ENSG00000148834	1.25	7.25	2.24529E-06	5.48014E-05	glutathione S-transferase omega 1
ENSG00000164032	1.25	8.16	1.18195E-05	0.000217861	H2A histone family, member Z
ENSG00000261740	1.25	5.47	1.03892E-05	0.000195968	Uncharacterized protein
ENSG00000143387	1.25	4.89	0.001087326	0.008863936	cathepsin K
ENSG00000117697	1.25	4.76	4.82438E-05	0.000723153	NSL1, MIS12 kinetochore complex component
ENSG00000178951	1.24	6.07	2.20409E-06	5.41426E-05	zinc finger and BTB domain containing 7A
ENSG00000102007	1.24	7.20	8.37147E-07	2.42919E-05	proteolipid protein 2 (colonic epithelium-enriched)
ENSG00000078668	1.23	6.93	7.95208E-07	2.35123E-05	voltage-dependent anion channel 3
ENSG00000173163	1.23	5.22	2.02379E-05	0.000348688	copper metabolism (Murr1) domain containing 1
ENSG00000106355	1.23	4.60	0.000111593	0.00143045	LSM5 homolog, U6 small nuclear RNA and mRNA degradation associated
ENSG00000213551	1.23	4.97	0.00020179	0.00229241	DnaJ (Hsp40) homolog, subfamily C, member 9
ENSG00000183291	1.23	7.82	4.12106E-06	9.04044E-05	15 kDa selenoprotein
ENSG00000143198	1.23	6.65	1.61147E-06	4.16435E-05	microsomal glutathione S-transferase 3
ENSG00000237765	1.23	5.17	2.36172E-05	0.0003989	family with sequence similarity 200, member B
ENSG00000144681	1.22	4.73	0.000259792	0.002781089	SH3 and cysteine rich domain
ENSG00000135919	1.22	4.98	0.000341627	0.003464802	serpin peptidase inhibitor, clade E (nexin, plasminogen activator inhibitor type 1), member 2
ENSG00000090581	1.22	5.81	2.73335E-05	0.000444247	N-acetylglucosamine-1-phosphate transferase, gamma subunit
ENSG00000163814	1.22	6.85	8.65794E-05	0.001158066	CUB domain containing protein 1
ENSG00000135047	1.22	6.10	1.47941E-05	0.000265861	cathepsin L
ENSG00000213024	1.22	5.69	2.79642E-06	6.48593E-05	nucleoporin 62kDa
ENSG00000068697	1.21	8.97	2.73833E-05	0.000444498	lysosomal protein transmembrane 4 alpha
ENSG00000154719	1.21	4.65	0.000144246	0.001776738	mitochondrial ribosomal protein L39
ENSG00000148908	1.21	5.11	0.000691896	0.006152784	regulator of G-protein signaling 10
ENSG00000125733	1.21	6.05	5.3926E-06	0.000114226	thyroid hormone receptor interactor 10
ENSG00000078140	1.21	7.00	1.90267E-06	4.78296E-05	ubiquitin-conjugating enzyme E2K
ENSG00000150779	1.21	6.15	3.31394E-06	7.4908E-05	translocase of inner mitochondrial membrane 8 homolog B (yeast)
ENSG00000155115	1.21	6.25	6.987E-06	0.000142172	general transcription factor IIIC, polypeptide 6, alpha 35kDa
ENSG00000102103	1.21	6.07	1.29592E-06	3.50304E-05	polyglutamine binding protein 1
ENSG00000216490	1.20	6.13	2.04341E-05	0.000350637	interferon, gamma-inducible protein 30
ENSG00000206283	1.20	5.49	7.74813E-06	0.000154287	prefoldin subunit 6
ENSG00000179041	1.20	4.85	0.000148133	0.001806586	ribosome biogenesis regulator homolog
ENSG00000103496	1.20	5.63	4.34966E-06	9.49357E-05	syntaxin 4
ENSG00000165996	1.19	6.33	1.29474E-06	3.50304E-05	3-hydroxyacyl-CoA dehydratase 1
ENSG00000187514	1.19	8.96	0.000219463	0.002444548	prothymosin, alpha
ENSG00000104131	1.19	6.88	2.67552E-06	6.26274E-05	eukaryotic translation initiation factor 3, subunit J
ENSG00000088888	1.19	6.67	2.64671E-06	6.21784E-05	mitochondrial antiviral signaling protein
ENSG00000133997	1.19	4.62	0.000133782	0.001663715	mediator complex subunit 6
ENSG00000144136	1.19	7.34	2.12643E-06	5.27363E-05	solute carrier family 20 (phosphate transporter), member 1
ENSG00000131876	1.19	5.99	1.05997E-05	0.000199357	small nuclear ribonucleoprotein polypeptide A'
ENSG00000256591	1.19	4.85	9.73087E-05	0.00127214	Uncharacterized protein
ENSG00000107404	1.19	6.76	1.48643E-06	3.8859E-05	dishevelled segment polarity protein 1
ENSG00000103152	1.19	6.66	1.28132E-06	3.47813E-05	N-methylpurine DNA glycosylase
ENSG00000120656	1.19	5.02	9.79199E-05	0.001275426	TAF12 RNA polymerase II, TATA box binding protein (TBP)-associated factor, 20kDa
ENSG00000136003	1.18	6.52	2.48407E-06	5.95486E-05	iron-sulfur cluster assembly enzyme
ENSG00000273841	1.18	6.64	6.95854E-07	2.09583E-05	TAF9 RNA polymerase II, TATA box binding protein (TBP)-associated factor, 32kDa
ENSG00000111775	1.18	7.99	1.89255E-05	0.000329563	cytochrome c oxidase subunit VIa polypeptide 1
ENSG00000241343	1.18	6.26	0.000974002	0.008108941	ribosomal protein L36a
ENSG00000163644	1.18	5.22	2.50426E-05	0.000417516	protein phosphatase, Mg2+/Mn2+ dependent, 1K
ENSG00000182154	1.18	6.23	1.13518E-06	3.12742E-05	mitochondrial ribosomal protein L41
ENSG00000090674	1.18	5.04	0.00013437	0.001667809	mucolipin 1
ENSG00000147684	1.17	6.54	3.5051E-06	7.84911E-05	NADH dehydrogenase (ubiquinone) 1 beta subcomplex, 9, 22kDa
ENSG00000092020	1.17	4.86	0.000121496	0.001534552	protein phosphatase 2, regulatory subunit B'', gamma
ENSG00000140307	1.17	6.16	1.46234E-06	3.84042E-05	general transcription factor IIA, 2, 12kDa
ENSG00000161281	1.17	6.93	4.25299E-06	9.31404E-05	cytochrome c oxidase subunit VIIa polypeptide 1 (muscle)
ENSG00000176340	1.17	7.74	1.19709E-05	0.000220336	cytochrome c oxidase subunit VIIIA (ubiquitous)
ENSG00000155034	1.16	5.05	4.99489E-05	0.000742682	F-box and leucine-rich repeat protein 18
ENSG00000278259	1.16	5.64	1.63151E-05	0.000289173	myosin XIX
ENSG00000131732	1.16	4.68	0.00015431	0.001866585	zinc finger, CCHC domain containing 9
ENSG00000204138	1.16	5.85	5.28934E-06	0.000112407	phosphatase and actin regulator 4
ENSG00000131462	1.16	6.86	7.57797E-06	0.000151806	tubulin, gamma 1
ENSG00000228907	1.15	5.98	4.07971E-06	8.98022E-05	ring finger protein 5, E3 ubiquitin protein ligase
ENSG00000196683	1.15	7.33	5.41225E-06	0.000114454	translocase of outer mitochondrial membrane 7 homolog (yeast)
ENSG00000143553	1.15	5.52	1.38515E-05	0.000250666	SNAP-associated protein

ENSG00000213626	1.15	8.82	0.001004649	0.008315866	limb bud and heart development
ENSG00000171621	1.15	4.60	0.000266322	0.002839233	splA/ryanodine receptor domain and SOCS box containing 1
ENSG00000126653	1.15	5.55	1.52934E-05	0.000273314	nuclear speckle splicing regulatory protein 1
ENSG00000177889	1.15	7.07	1.00871E-05	0.000192235	ubiquitin-conjugating enzyme E2N
ENSG00000132646	1.15	6.18	4.34857E-06	9.49357E-05	proliferating cell nuclear antigen
ENSG00000159199	1.15	6.35	2.7477E-06	6.40849E-05	ATP synthase, H+ transporting, mitochondrial Fo complex, subunit C1 (subunit 9)
ENSG00000128708	1.14	5.60	0.000127738	0.001597781	histone acetyltransferase 1
ENSG00000162961	1.14	6.57	1.44075E-06	3.79917E-05	dpy-30 histone methyltransferase complex regulatory subunit
ENSG00000275342	1.14	5.03	0.00027106	0.002875508	Tyrosine-protein kinase SgK223
ENSG00000157895	1.14	4.75	0.000198937	0.002276766	chromosome 12 open reading frame 43
ENSG00000118503	1.14	4.72	0.000241954	0.002644908	tumor necrosis factor, alpha-induced protein 3
ENSG00000169062	1.14	6.03	0.000372337	0.003710115	UPF3 regulator of nonsense transcripts homolog A (yeast)
ENSG00000105379	1.14	6.66	3.42799E-06	7.68976E-05	electron-transfer-flavoprotein, beta polypeptide
ENSG00000275145	1.14	5.52	3.34141E-05	0.000527159	FSHD region gene 1
ENSG00000163281	1.14	4.92	0.000163396	0.001960298	glucosamine-6-phosphate deaminase 2
ENSG00000143314	1.14	5.62	5.45907E-05	0.000801553	mitochondrial ribosomal protein L24
ENSG00000127184	1.14	8.06	3.14242E-05	0.000502515	cytochrome c oxidase subunit VIIc
ENSG00000165264	1.14	5.82	2.68743E-05	0.000440104	NADH dehydrogenase (ubiquinone) 1 beta subcomplex, 6, 17kDa
ENSG00000162222	1.13	4.77	0.000237785	0.002610377	tetratricopeptide repeat domain 9C
ENSG00000277359	1.13	9.35	0.000803225	0.006914374	40S ribosomal protein S9; Ribosomal protein S9, isoform CRA_c
ENSG00000105755	1.13	5.42	0.000163836	0.001961138	ethylmalonic encephalopathy 1
ENSG00000168734	1.13	5.76	0.001111501	0.009004207	protein kinase (cAMP-dependent, catalytic) inhibitor gamma
ENSG00000136238	1.13	8.83	0.000186826	0.002176719	ras-related C3 botulinum toxin substrate 1 (rho family, small GTP binding protein Rac1)
ENSG00000105364	1.12	6.24	1.8631E-05	0.000325094	mitochondrial ribosomal protein L4
ENSG00000159079	1.12	5.16	0.000146129	0.001793095	chromosome 21 open reading frame 59
ENSG00000105202	1.12	6.14	5.37223E-05	0.000791501	fibrillarin
ENSG00000136942	1.12	9.13	0.00039462	0.003883389	ribosomal protein L35
ENSG00000125835	1.12	7.58	2.02967E-05	0.000349206	small nuclear ribonucleoprotein polypeptides B and B1
ENSG00000104522	1.12	6.75	8.66759E-06	0.000168666	tissue specific transplantation antigen P35B
ENSG00000198840	1.12	8.66	0.000137289	0.001700783	mitochondrially encoded NADH dehydrogenase 3
ENSG00000185222	1.11	7.09	1.00523E-05	0.000191855	WW domain binding protein 5
ENSG00000177606	1.11	7.22	1.1013E-05	0.000205338	jun proto-oncogene
ENSG00000147533	1.11	5.96	7.74956E-06	0.000154287	golgin A7
ENSG00000100632	1.11	6.86	1.30715E-05	0.000238554	enhancer of rudimentary homolog (Drosophila)
ENSG00000132581	1.11	6.16	7.18836E-06	0.000145353	stromal cell-derived factor 2
ENSG00000094804	1.11	4.63	0.000717447	0.006313032	cell division cycle 6
ENSG00000104915	1.11	5.77	2.0201E-05	0.000348688	syntaxin 10
ENSG00000188643	1.11	8.21	4.38503E-05	0.000671315	S100 calcium binding protein A16
ENSG00000130766	1.11	5.06	0.000125293	0.001573281	sestrin 2
ENSG00000113068	1.11	6.05	8.40208E-05	0.001134412	prefoldin subunit 1
ENSG00000173141	1.10	5.25	0.000213433	0.002385614	mitochondrial ribosomal protein L57
ENSG00000054148	1.10	6.98	3.41858E-05	0.00053802	phosphohistidine phosphatase 1
ENSG00000147996	1.10	4.68	0.001116688	0.009034893	COBW domain containing 5
ENSG00000168172	1.10	6.45	1.01115E-05	0.000192417	hook microtubule-tethering protein 3
ENSG00000083457	1.10	4.68	0.00079959	0.006892262	integrin, alpha E (antigen CD103, human mucosal lymphocyte antigen 1; alpha polypeptide)
ENSG00000117280	1.10	5.15	0.000169601	0.00201232	RAB29, member RAS oncogene family
ENSG00000157613	1.10	5.48	3.01179E-05	0.000482821	cAMP responsive element binding protein 3-like 1
ENSG00000174021	1.10	6.24	6.70503E-06	0.000137894	guanine nucleotide binding protein (G protein), gamma 5
ENSG00000133641	1.10	4.66	0.000619792	0.005623833	chromosome 12 open reading frame 29
ENSG00000118181	1.10	8.99	0.000290765	0.003044546	ribosomal protein S25
ENSG00000186918	1.09	5.01	0.001228219	0.009706312	zinc finger protein 395
ENSG00000101843	1.09	6.10	7.08196E-06	0.000143877	proteasome (prosome, macropain) 26S subunit, non-ATPase, 10
ENSG00000138785	1.09	5.08	0.000194547	0.002239756	integrator complex subunit 12
ENSG00000008513	1.09	5.73	6.49076E-05	0.000920605	ST3 beta-galactoside alpha-2,3-sialyltransferase 1
ENSG00000122026	1.09	8.72	0.000288144	0.003031048	ribosomal protein L21
ENSG00000123374	1.09	5.23	0.000146336	0.001793549	cyclin-dependent kinase 2
ENSG00000175334	1.09	6.91	6.92523E-05	0.000968992	barrier to autointegration factor 1
ENSG00000099977	1.09	5.97	8.71092E-05	0.001160797	D-dopachrome tautomerase
ENSG00000104979	1.09	6.71	1.32011E-05	0.00024058	chromosome 19 open reading frame 53
ENSG00000070423	1.09	5.87	0.000265249	0.002830125	ring finger protein 126
ENSG00000147586	1.08	4.80	0.00041039	0.003986952	mitochondrial ribosomal protein S28
ENSG00000186283	1.08	4.92	0.000304117	0.003146784	torsin family 3, member A
ENSG00000162783	1.08	5.98	5.55531E-05	0.00081342	immediate early response 5
ENSG00000123136	1.08	5.75	2.37969E-05	0.000400364	DEAD (Asp-Glu-Ala-Asp) box polypeptide 39A
ENSG00000162407	1.08	6.06	0.000100798	0.001310274	phosphatidic acid phosphatase type 2B
ENSG00000160201	1.08	6.42	1.33191E-05	0.000242388	U2 small nuclear RNA auxiliary factor 1
ENSG00000257727	1.08	6.38	5.01542E-06	0.00010747	canopy FGF signaling regulator 2

ENSG00000136868	1.08	5.36	0.000165858	0.001973347	solute carrier family 31 (copper transporter), member 1
ENSG00000138495	1.08	5.09	0.000476244	0.004518019	COX17 cytochrome c oxidase copper chaperone
ENSG00000166595	1.08	6.13	8.71429E-05	0.001160797	family with sequence similarity 96, member B
ENSG00000100442	1.07	6.31	1.02885E-05	0.000194637	FK506 binding protein 3, 25kDa
ENSG00000182117	1.07	7.32	5.52683E-05	0.000810581	NOP10 ribonucleoprotein
ENSG00000185022	1.07	5.74	0.000237692	0.002610377	v-maf avian musculoaponeurotic fibrosarcoma oncogene homolog F
ENSG00000156467	1.07	8.00	0.000106354	0.001370012	ubiquinol-cytochrome c reductase binding protein
ENSG00000048162	1.07	4.99	0.000398735	0.003911959	NOP16 nucleolar protein
ENSG00000115446	1.07	4.76	0.001168549	0.009340217	unc-50 homolog (C. elegans)
ENSG00000171421	1.06	5.15	0.000146443	0.001793549	mitochondrial ribosomal protein L36
ENSG00000188612	1.06	7.41	4.39299E-05	0.000671737	small ubiquitin-like modifier 2
ENSG00000157881	1.06	5.04	0.000289145	0.003031048	pantothenate kinase 4
ENSG00000131495	1.06	5.74	7.75759E-05	0.001065205	NADH dehydrogenase (ubiquinone) 1 alpha subcomplex, 2, 8kDa
ENSG00000152684	1.06	5.01	0.001168882	0.009340217	pelota homolog (Drosophila)
ENSG00000118363	1.06	6.54	4.38345E-05	0.000671315	signal peptidase complex subunit 2
ENSG00000113460	1.05	4.59	0.001013165	0.00836492	BRX1, biogenesis of ribosomes
ENSG00000167641	1.05	8.47	0.000158218	0.001907033	protein phosphatase 1, regulatory (inhibitor) subunit 14A
ENSG00000124226	1.05	6.52	5.0277E-05	0.0007467	ring finger protein 114
ENSG00000213619	1.05	6.22	3.55191E-05	0.000557645	NADH dehydrogenase (ubiquinone) Fe-S protein 3, 30kDa (NADH-coenzyme Q reductase)
ENSG00000101363	1.05	6.20	0.000101824	0.001318308	mannosidase, beta A, lysosomal-like
ENSG00000189060	1.05	7.32	4.32109E-05	0.000664677	H1 histone family, member 0
ENSG00000128463	1.05	6.59	1.09516E-05	0.000204772	ER membrane protein complex subunit 4
ENSG00000010165	1.05	5.21	0.000242532	0.002648991	methyltransferase like 13
ENSG00000173465	1.05	5.92	2.59544E-05	0.000428298	Sjogren syndrome/scleroderma autoantigen 1
ENSG00000117395	1.04	7.05	4.38069E-05	0.000671315	EBNA1 binding protein 2
ENSG00000160888	1.04	6.25	6.71789E-05	0.000946585	immediate early response 2
ENSG00000141560	1.04	5.20	0.000601949	0.005499985	fructosamine 3 kinase related protein
ENSG00000160991	1.04	6.67	0.000173074	0.002045658	ORAI calcium release-activated calcium modulator 2
ENSG00000185651	1.04	7.53	4.77656E-05	0.000717078	ubiquitin-conjugating enzyme E2L 3
ENSG00000115539	1.03	5.22	0.000255675	0.002750687	phosducin-like 3
ENSG00000122952	1.03	4.95	0.001198924	0.009538977	ZW10 interacting kinetochore protein
ENSG00000128609	1.03	5.63	9.29134E-05	0.001223786	NADH dehydrogenase (ubiquinone) 1 alpha subcomplex, 5
ENSG00000167283	1.02	7.21	0.000143356	0.001767464	ATP synthase, H+ transporting, mitochondrial Fo complex, subunit G
ENSG00000112715	1.02	6.90	9.05287E-05	0.001197259	vascular endothelial growth factor A
ENSG00000118292	1.02	5.31	0.000336838	0.003424302	chromosome 1 open reading frame 54
ENSG00000237403	1.02	6.15	6.63855E-05	0.00093745	protein phosphatase 1, regulatory (inhibitor) subunit 11
ENSG00000183520	1.02	5.37	0.000299869	0.003110299	UTP11-like, U3 small nucleolar ribonucleoprotein (yeast)
ENSG00000116750	1.02	5.00	0.001277117	0.009988883	ubiquitin carboxyl-terminal hydrolase L5
ENSG00000146574	1.02	5.99	5.41239E-05	0.000796509	CCZ1 homolog B, vacuolar protein trafficking and biogenesis associated
ENSG00000183011	1.01	5.44	0.000224601	0.002488911	N(alpha)-acetyltransferase 38, NatC auxiliary subunit
ENSG00000232112	1.01	7.08	9.6769E-05	0.001266821	translation machinery associated 7 homolog (S. cerevisiae)
ENSG00000112096	1.01	6.79	0.000357509	0.003580908	superoxide dismutase 2, mitochondrial

Supplementary Table S7A: Differencial expression analysis of T. gondii genes from neuronal stem cells infected with T. gondii EGS strain for 18 hours copmared to T. gondii gene expression from neuronal stem cells infected with GT1 strain for 18 hours.

gene ID	logFC (EGS/GT1)	logCPM	PValue	FDR	Product Name
TGME49_233925	6.94	7.83	7.51E-96	5.55E-92	hypothetical protein
TGME49_237130	7.84	10.26	2.61E-88	9.64E-85	cytochrome b, putative
TGME49_320050	4.19	13.43	2.62E-77	6.45E-74	ribosomal protein RPL5
TGME49_322200	12.41	6.70	8.07E-75	1.49E-71	apocytochrome b, putative
TGME49_330000	8.50	10.23	4.67E-72	6.91E-69	cytochrome b
TGME49_290600	3.14	9.11	1.20E-63	1.47E-60	succinyl-CoA-synthetase alpha SCSA
TGME49_323400	7.83	9.06	2.63E-61	2.78E-58	cytochrome c oxidase subunit iii subfamily protein
TGME49_322800	12.26	6.48	2.09E-53	1.93E-50	hypothetical protein
TGME49_301250	7.82	14.41	3.45E-49	2.83E-46	hypothetical protein
TGME49_255060	7.95	9.95	2.43E-47	1.79E-44	cytochrome b(N-terminal)/b6/petB subfamily prot
TGME49_321360	3.31	8.21	6.33E-47	4.26E-44	clustered-asparagine-rich protein
TGME49_232955	11.47	12.33	5.85E-42	3.61E-39	hypothetical protein
TGME49_302055	11.93	5.97	1.32E-39	7.52E-37	ribosomal protein RPS12
TGME49_251180	4.23	6.80	2.16E-37	1.14E-34	KRUF family protein
TGME49_262690	1.96	10.98	4.59E-36	2.26E-33	ribosomal protein RPL27
TGME49_261240	7.55	5.02	1.21E-33	5.61E-31	histone H3
TGME49_238240	2.08	8.97	1.49E-31	6.48E-29	bystin protein
TGME49_250710	2.10	11.27	5.46E-31	2.24E-28	microneme protein MIC10
TGME49_244370	3.57	8.80	6.24E-29	2.43E-26	TDC1, putative
TGME49_213280	2.15	8.91	2.56E-28	9.47E-26	SAG-related sequence SRS25
TGME49_218520	1.84	10.33	1.27E-27	4.46E-25	microneme protein MIC6
TGME49_217530	4.13	6.35	1.80E-27	6.05E-25	hypothetical protein
TGME49_211695	4.77	5.62	7.56E-26	2.43E-23	hypothetical protein
TGME49_287040	3.80	7.04	8.71E-26	2.68E-23	hypothetical protein
TGME49_277230	3.85	5.63	1.20E-25	3.54E-23	hypothetical protein
TGME49_311230	1.78	9.99	2.24E-25	6.36E-23	hypothetical protein
TGME49_237230	1.90	9.81	4.15E-24	1.14E-21	hypothetical protein
TGME49_223660	3.08	7.34	4.92E-24	1.30E-21	50S ribosomal protein L4, putative
TGME49_293790	4.56	6.72	2.61E-23	6.65E-21	hypothetical protein
TGME49_293170	-5.98	7.60	3.54E-23	8.73E-21	hypothetical protein
TGME49_205680	3.65	8.42	4.09E-23	9.75E-21	hypothetical protein
TGME49_271935	2.89	6.69	4.96E-22	1.14E-19	hypothetical protein
TGME49_254710	2.29	7.81	5.10E-22	1.14E-19	serine esterase (DUF676) protein
TGME49_275860	2.82	9.46	7.11E-22	1.55E-19	hypothetical protein
TGME49_253690	1.70	8.98	6.35E-21	1.34E-18	hypothetical protein
TGME49_221840	4.60	4.71	7.31E-21	1.46E-18	hypothetical protein
TGME49_251400	3.32	6.13	7.29E-21	1.46E-18	hypothetical protein
TGME49_240060	2.01	9.24	8.06E-21	1.57E-18	hypothetical protein
TGME49_224760	6.80	4.85	1.53E-20	2.90E-18	SAG-related sequence SRS40E
TGME49_236910	-4.99	7.47	2.39E-20	4.41E-18	U2 snRNP auxiliary factor, putative
TGME49_243615	4.37	5.68	2.85E-20	5.14E-18	hypothetical protein
TGME49_225790	2.95	7.33	7.51E-20	1.32E-17	PDI family protein
TGME49_277080	1.51	10.55	1.16E-19	2.00E-17	microneme protein MIC5
TGME49_211030	-3.57	8.15	1.31E-19	2.21E-17	hypothetical protein

TGME49_259260	-2.69	9.01	1.40E-19	2.31E-17	membrane protein FtsH1
TGME49_261720	-5.75	7.82	1.64E-19	2.63E-17	metal cation transporter, ZIP family protein
TGME49_229010	-2.20	9.17	3.22E-19	5.06E-17	rhoptry neck protein RON4
TGME49_273320	2.17	7.86	5.07E-19	7.80E-17	hypothetical protein
TGME49_233450	1.96	8.00	1.80E-18	2.72E-16	SAG-related sequence SRS29A
TGME49_239790	-10.72	7.09	1.85E-18	2.74E-16	BRCA1 C Terminus (BRCT) domain-containing prot
TGME49_275640	2.47	6.94	3.72E-18	5.40E-16	hypothetical protein
TGME49_290020	2.52	6.64	1.61E-17	2.29E-15	cyclin dependent kinase binding protein
TGME49_287170	-10.73	7.07	1.91E-17	2.67E-15	hypothetical protein
TGME49_269330	-10.62	7.07	2.34E-17	3.20E-15	hypothetical protein
TGME49_208370	1.60	8.84	3.37E-17	4.53E-15	myosin heavy chain, putative
TGME49_275870	4.13	5.59	4.81E-17	6.35E-15	tubulin/FtsZ family, GTPase domain-containing pr
TGME49_260430	5.02	4.67	7.97E-17	1.03E-14	hypothetical protein
TGME49_270700	2.17	7.08	1.44E-16	1.84E-14	hypothetical protein
TGME49_312140	-3.17	8.29	2.50E-16	3.13E-14	hypothetical protein
TGME49_228160	1.95	7.19	2.70E-16	3.33E-14	acid phosphatase
TGME49_214980	1.71	8.41	2.76E-16	3.35E-14	hypothetical protein
TGME49_201170	-2.54	8.67	3.01E-16	3.59E-14	hypothetical protein
TGME49_301222	3.12	6.56	3.44E-16	4.03E-14	DNA repair protein Rad4 domain-containing prote
TGME49_252430	1.96	8.12	3.96E-16	4.57E-14	hypothetical protein
TGME49_290700	2.40	7.93	4.29E-16	4.88E-14	hypothetical protein
TGME49_226310	-4.12	7.74	5.29E-16	5.93E-14	zinc finger (CCCH type) motif-containing protein
TGME49_251540	1.84	9.03	6.26E-16	6.91E-14	dense granule protein GRA9
TGME49_293570	-3.09	7.85	6.50E-16	7.07E-14	translocation protein sec62, putative
TGME49_290200	-2.13	8.88	1.37E-15	1.47E-13	NAD/NADP octopine/nopaline dehydrogenase, alp
TGME49_261520	-5.37	7.04	1.59E-15	1.68E-13	nucleolar GTP-binding protein 1, putative
TGME49_306670	-3.53	7.92	1.64E-15	1.71E-13	hypothetical protein
TGME49_249990	2.26	10.24	1.73E-15	1.77E-13	hypothetical protein
TGME49_208450	1.90	8.60	2.16E-15	2.15E-13	protease inhibitor PI2
TGME49_214220	1.64	9.74	2.14E-15	2.15E-13	hypothetical protein
TGME49_315885	2.12	7.08	2.42E-15	2.39E-13	glycosyltransferase, putative
TGME49_254620	1.38	9.19	2.76E-15	2.69E-13	ribosomal protein RPL39
TGME49_219520	-2.77	7.68	3.35E-15	3.22E-13	histone arginine methyltransferase PRMT1
TGME49_269310	4.41	4.71	4.15E-15	3.94E-13	hypothetical protein
TGME49_239440	-10.43	6.88	4.35E-15	4.07E-13	protein kinase (incomplete catalytic triad)
TGME49_254120	2.06	8.30	6.90E-15	6.38E-13	autophagy-related protein 8 atg8, putative
TGME49_214350	-3.27	7.39	7.25E-15	6.62E-13	GTP-binding protein, putative
TGME49_206550	5.36	6.05	9.88E-15	8.91E-13	hypothetical protein
TGME49_245980	4.18	7.53	1.10E-14	9.81E-13	hypothetical protein
TGME49_222380	-2.54	8.13	1.33E-14	1.17E-12	importin-beta N-terminal domain-containing prote
TGME49_314500	-2.83	8.36	1.74E-14	1.51E-12	subtilisin SUB2
TGME49_266050	1.90	7.09	1.93E-14	1.66E-12	hypothetical protein
TGME49_223930	1.66	8.26	2.05E-14	1.74E-12	RNA recognition motif-containing protein
TGME49_319500	-10.40	7.06	2.67E-14	2.21E-12	hypothetical protein
TGME49_217820	-2.95	7.71	2.86E-14	2.35E-12	PCI domain-containing protein
TGME49_298620	-5.25	6.83	3.21E-14	2.61E-12	hypothetical protein
TGME49_265190	-4.42	7.20	3.62E-14	2.91E-12	Ulp1 protease family, C-terminal catalytic domain-
TGME49_245460	1.32	10.31	3.84E-14	3.05E-12	ribosomal protein RPS8
TGME49_315620	-5.25	7.07	4.12E-14	3.24E-12	vacuolar ATP synthase subunit C, putative
TGME49_282070	-2.08	8.54	4.34E-14	3.38E-12	hypothetical protein
TGME49_300220	-3.34	7.60	4.42E-14	3.40E-12	hypothetical protein
TGME49_240650	-2.32	8.44	4.99E-14	3.80E-12	coatomer protein complex, subunit alpha, putativ
TGME49_292920	-2.40	7.96	7.17E-14	5.41E-12	heat shock protein 75, putative
TGME49_260500	-2.03	9.04	7.31E-14	5.46E-12	COPI associated protein, putative

TGME49_277000	-4.45	7.32	8.80E-14	6.51E-12	transport protein Sec24, putative
TGME49_305160	3.42	4.99	9.45E-14	6.92E-12	histone H2Ba
TGME49_278870	-1.83	8.87	1.20E-13	8.71E-12	myosin F
TGME49_212880	-2.79	7.91	1.43E-13	1.03E-11	surface antigen repeat-containing protein
TGME49_220930	-3.07	7.43	1.45E-13	1.03E-11	hypothetical protein
TGME49_250340	-2.65	7.79	1.48E-13	1.04E-11	centrin 2
TGME49_287460	3.46	4.88	1.79E-13	1.25E-11	hypothetical protein
TGME49_209050	-10.21	6.78	1.93E-13	1.34E-11	Tyrosine kinase-like (TKL) protein
TGME49_261022	-2.70	7.63	2.24E-13	1.53E-11	dynein heavy chain family protein
TGME49_253170	1.86	7.37	2.27E-13	1.54E-11	zinc carboxypeptidase, putative
TGME49_238895	-3.36	7.20	2.62E-13	1.76E-11	hypothetical protein
TGME49_259240	1.55	9.56	2.68E-13	1.79E-11	ribosomal protein RPS21
TGME49_200300	6.27	4.83	2.79E-13	1.84E-11	hypothetical protein
TGME49_239260	6.69	4.79	4.13E-13	2.68E-11	histone H4
TGME49_228690	-5.20	7.16	4.14E-13	2.68E-11	phosphatidylinositol 3- and 4-kinase
TGME49_321530	1.55	8.75	4.34E-13	2.74E-11	cathepsin CPL
TGME49_244280	-1.60	10.21	4.32E-13	2.74E-11	hypothetical protein
TGME49_247530	3.32	8.95	4.50E-13	2.82E-11	hypothetical protein
TGME49_263870	-5.18	6.97	4.63E-13	2.88E-11	glutamate-tRNA ligase
TGME49_270360	-5.16	7.03	5.71E-13	3.52E-11	hypothetical protein
TGME49_257910	4.73	5.98	6.05E-13	3.69E-11	hypothetical protein
TGME49_251740	-3.48	7.43	6.35E-13	3.85E-11	AP2 domain transcription factor AP2XII-9
TGME49_240280	-2.57	7.78	7.03E-13	4.23E-11	S1/P1nuclease
TGME49_210300	-5.16	6.98	7.30E-13	4.35E-11	hypothetical protein
TGME49_249350	-10.14	6.75	7.38E-13	4.36E-11	esterase/lipase/thioesterase domain-containing p
TGME49_242290	-2.55	7.99	7.94E-13	4.66E-11	proteasome subunit alpha1, putative
TGME49_286120	-2.44	8.02	8.41E-13	4.89E-11	prolyl endopeptidase
TGME49_244880	-4.25	6.91	9.16E-13	5.29E-11	DNA-directed RNA polymerase I RPA1
TGME49_294820	2.77	7.93	1.41E-12	8.05E-11	type I fatty acid synthase, putative
TGME49_289710	-2.76	7.42	1.80E-12	1.03E-10	AP2 domain transcription factor AP2IX-5
TGME49_261070	-4.20	6.54	1.92E-12	1.08E-10	apicoplast triosephosphate translocator APT1
TGME49_294640	-1.70	8.84	1.96E-12	1.10E-10	ribonucleoside-diphosphate reductase large chain
TGME49_290270	1.95	6.58	2.00E-12	1.11E-10	SPRY domain-containing protein
TGME49_202980	-2.87	7.20	2.75E-12	1.52E-10	hypothetical protein
TGME49_259010	-5.04	6.71	3.11E-12	1.70E-10	vacuolar ATP synthase subunit d, putative
TGME49_237210	-5.01	6.72	3.34E-12	1.82E-10	Tyrosine kinase-like (TKL) protein
TGME49_228280	2.80	5.71	3.57E-12	1.92E-10	hypothetical protein
TGME49_259550	-2.88	7.66	3.97E-12	2.13E-10	dihydropteroate synthase
TGME49_231410	-2.46	8.15	4.07E-12	2.16E-10	hypothetical protein
TGME49_272370	1.86	7.74	4.13E-12	2.18E-10	hypothetical protein
TGME49_294610	-1.60	9.34	4.20E-12	2.20E-10	histone lysine methyltransferase, SET, putative
TGME49_241830	2.46	5.87	4.31E-12	2.24E-10	peptidyl-prolyl cis-trans isomerase
TGME49_260800	2.39	6.32	4.45E-12	2.30E-10	hypothetical protein
TGME49_232650	2.05	6.75	4.59E-12	2.36E-10	hypothetical protein
TGME49_202420	2.21	6.26	4.77E-12	2.43E-10	hypothetical protein
TGME49_316280	-5.04	6.90	4.91E-12	2.48E-10	transporter, major facilitator family protein
TGME49_214410	2.79	7.77	4.99E-12	2.49E-10	hypothetical protein
TGME49_232560	-3.04	6.98	4.99E-12	2.49E-10	hypothetical protein
TGME49_253750	2.24	8.98	5.39E-12	2.68E-10	PLU-1 family protein
TGME49_214970	-1.94	8.91	5.84E-12	2.88E-10	DNA replication licensing factor, putative
TGME49_301270	1.53	8.03	6.56E-12	3.21E-10	Tyrosine kinase-like (TKL) protein
TGME49_274060	-2.24	7.69	6.75E-12	3.26E-10	2-oxoglutarate/malate translocase OMT
TGME49_291890	1.28	11.01	7.02E-12	3.37E-10	microneme protein MIC1
TGME49_293480	2.74	7.15	7.74E-12	3.69E-10	MoeA N-terminal region (domain I and II) domain-

TGME49_246330	-5.04	7.00	8.95E-12	4.24E-10	CRAL/TRIO domain-containing protein
TGME49_208530	-3.18	7.28	9.18E-12	4.32E-10	nicotinate phosphoribosyltransferase
TGME49_313870	2.00	7.32	9.48E-12	4.43E-10	zinc finger, C3HC4 type (RING finger) domain-cont
TGME49_255650	-10.23	7.05	1.17E-11	5.43E-10	DHHC zinc finger domain-containing protein
TGME49_262380	-3.40	7.00	1.28E-11	5.93E-10	elongation factor Tu, putative
TGME49_207460	1.59	7.42	1.33E-11	6.11E-10	Rab5B protein
TGME49_221470	-2.08	8.22	1.34E-11	6.12E-10	hypothetical protein
TGME49_254080	1.41	7.96	1.60E-11	7.26E-10	metal cation transporter, ZIP family protein
TGME49_321650	-2.67	7.47	1.62E-11	7.29E-10	hypothetical protein
TGME49_267670	4.04	5.56	1.67E-11	7.43E-10	hypothetical protein
TGME49_260440	1.42	9.39	1.67E-11	7.43E-10	nuclear factor NF3
TGME49_294770	-4.96	6.84	1.71E-11	7.57E-10	Armadillo/beta-catenin family repeat-containing p
TGME49_218530	-9.93	6.40	1.72E-11	7.57E-10	proteasome-interacting thioredoxin domain-conta
TGME49_254720	1.30	9.52	1.86E-11	8.15E-10	dense granule protein GRA8
TGME49_316600	1.58	8.03	1.89E-11	8.22E-10	hypothetical protein
TGME49_219700	-1.98	8.41	2.12E-11	9.16E-10	DNA replication licensing factor MCM4, putative
TGME49_305590	-4.08	6.99	2.32E-11	9.98E-10	ABC transporter transmembrane region domain-co
TGME49_213410	-2.26	7.73	2.49E-11	1.06E-09	small nuclear ribonucleoprotein f (snrnp-f), putativ
TGME49_323310	4.18	5.74	2.81E-11	1.19E-09	hypothetical protein
TGME49_250820	-2.77	7.50	2.95E-11	1.25E-09	hypothetical protein
TGME49_297530	-9.90	6.26	3.16E-11	1.33E-09	DNA-directed RNA polymerase I RPA2
TGME49_299780	1.88	9.06	3.20E-11	1.34E-09	hypothetical protein
TGME49_226960	-1.42	9.46	3.48E-11	1.45E-09	phosphofructokinase PFKII
TGME49_228190	-2.01	7.95	3.52E-11	1.45E-09	eukaryotic initiation factor-3, subunit 5, putative
TGME49_207680	-4.07	7.02	3.51E-11	1.45E-09	suppressor of kinetochore protein 1, putative
TGME49_310750	-2.08	8.35	3.65E-11	1.48E-09	emp24/gp25L/p24 family protein
TGME49_218880	-2.55	7.48	3.63E-11	1.48E-09	SF-assemblin, putative
TGME49_253570	2.47	6.14	3.81E-11	1.54E-09	hypothetical protein
TGME49_235130	-3.68	7.38	3.93E-11	1.58E-09	transmembrane protein
TGME49_220250	1.74	6.94	4.10E-11	1.64E-09	Nucleotide-sensitive chloride conductance regulat
TGME49_243430	2.37	7.87	4.82E-11	1.92E-09	OTU family cysteine protease
TGME49_217700	-9.90	6.41	4.92E-11	1.94E-09	AP2 domain transcription factor AP2XII-2
TGME49_254470	1.47	9.18	4.99E-11	1.96E-09	hypothetical protein
TGME49_211150	-4.87	6.82	5.59E-11	2.19E-09	hypothetical protein
TGME49_203300	2.63	7.42	5.75E-11	2.24E-09	hypothetical protein
TGME49_268980	1.73	7.97	6.47E-11	2.50E-09	hypothetical protein
TGME49_278510	-2.53	7.68	6.71E-11	2.58E-09	protein phosphatase 2C domain-containing protei
TGME49_227650	-3.07	6.80	7.10E-11	2.72E-09	microtubule-associated protein RP/EB family, puta
TGME49_318310	-2.15	7.63	7.31E-11	2.79E-09	transketolase
TGME49_239420	1.07	9.63	7.50E-11	2.84E-09	protein kinase
TGME49_232500	-9.85	6.44	7.64E-11	2.88E-09	hypothetical protein
TGME49_273050	2.18	6.54	7.89E-11	2.96E-09	hypothetical protein
TGME49_228660	-3.57	6.95	7.94E-11	2.96E-09	Sec7 domain-containing protein
TGME49_250030	2.80	5.24	8.89E-11	3.30E-09	hypothetical protein
TGME49_239410	-2.31	7.90	9.04E-11	3.34E-09	hypothetical protein
TGME49_228100	-2.62	7.17	9.27E-11	3.41E-09	hypothetical protein
TGME49_204530	1.26	11.00	9.48E-11	3.47E-09	microneme protein MIC11
TGME49_206610	-3.63	6.76	9.95E-11	3.62E-09	pyruvate dehydrogenase complex subunit PDH-E2
TGME49_306060	-1.39	9.70	1.05E-10	3.79E-09	rhoptry neck protein RON8
TGME49_231430	-9.83	6.39	1.05E-10	3.80E-09	oligosaccharyl transferase stt3 protein, putative
TGME49_201860	1.08	10.42	1.22E-10	4.38E-09	hypothetical protein
TGME49_223050	-1.44	9.44	1.35E-10	4.83E-09	ribosomal protein RPS20
TGME49_216730	-1.68	8.59	1.36E-10	4.83E-09	MCM2/3/5 family protein
TGME49_216820	1.42	8.57	1.44E-10	5.10E-09	transporter, major facilitator family protein

TGME49_249540	1.55	7.36	1.49E-10	5.25E-09	hypothetical protein
TGME49_305180	-9.79	6.43	1.50E-10	5.25E-09	Na+/H+ exchanger NHE3
TGME49_200360	2.53	9.74	1.56E-10	5.43E-09	hypothetical protein
TGME49_255410	-4.81	6.62	1.66E-10	5.71E-09	hypothetical protein
TGME49_316660	-9.79	6.50	1.66E-10	5.71E-09	cullin family protein
TGME49_210682	5.42	5.90	1.73E-10	5.93E-09	hypothetical protein
TGME49_286450	1.08	12.69	1.86E-10	6.33E-09	dense granule protein GRA5
TGME49_227810	1.36	8.87	1.94E-10	6.55E-09	rhoptry kinase family protein ROP11 (incomplete c
TGME49_278800	-2.27	7.62	1.94E-10	6.55E-09	zinc finger protein 36 family 3 protein
TGME49_268225	-5.13	6.81	2.13E-10	7.16E-09	hypothetical protein
TGME49_205580	1.68	7.01	2.25E-10	7.54E-09	nuclear factor NF4
TGME49_247460	-1.69	8.51	2.27E-10	7.55E-09	proliferating cell nuclear antigen PCNA1
TGME49_255180	-9.77	6.36	2.35E-10	7.78E-09	ubiquitin carboxyl-terminal hydrolase
TGME49_296010	-1.92	7.91	2.57E-10	8.49E-09	phosphatidylinositol 3- and 4-kinase
TGME49_293660	-3.96	6.63	2.77E-10	9.11E-09	hypothetical protein
TGME49_239540	-9.77	6.42	2.89E-10	9.46E-09	LEM3 (ligand-effect modulator 3) family / CDC50 f
TGME49_294630	-2.17	7.75	2.91E-10	9.47E-09	hypothetical protein
TGME49_207640	-2.14	7.49	2.97E-10	9.64E-09	isoleucyl-tRNA synthetase family protein
TGME49_318770	-4.78	6.44	3.12E-10	1.01E-08	aurora kinase(incomplete catalytic triad)
TGME49_314410	-2.66	7.22	3.47E-10	1.12E-08	aquarius, putative
TGME49_257360	2.37	6.17	3.79E-10	1.21E-08	hypothetical protein
TGME49_250840	-9.73	6.18	3.79E-10	1.21E-08	hypothetical protein
TGME49_293190	-2.94	7.38	4.04E-10	1.28E-08	endonuclease/exonuclease/phosphatase family pr
TGME49_253860	1.48	7.25	4.37E-10	1.38E-08	Tyrosine kinase-like (TKL) protein
TGME49_278050	-2.35	7.70	4.84E-10	1.52E-08	proteasome subunit alpha type 1, putative
TGME49_280490	-2.79	7.89	4.84E-10	1.52E-08	U-box domain-containing protein
TGME49_246580	-3.90	6.71	5.19E-10	1.62E-08	hypothetical protein
TGME49_232280	-9.84	6.42	5.28E-10	1.64E-08	hypothetical protein
TGME49_266070	1.16	9.66	6.00E-10	1.86E-08	ribosomal protein RPL31
TGME49_271820	-9.72	5.98	6.33E-10	1.95E-08	lipoyltransferase and lipoate-protein ligase subfan
TGME49_321690	-3.97	6.68	6.62E-10	2.03E-08	hypothetical protein
TGME49_227952	-2.77	6.97	7.03E-10	2.14E-08	14-3-3 superfamily protein
TGME49_293410	2.50	5.32	7.24E-10	2.19E-08	hypothetical protein
TGME49_212900	1.48	8.11	7.31E-10	2.20E-08	hypothetical protein
TGME49_313230	2.03	9.82	7.87E-10	2.36E-08	eukaryotic initiation factor-2, alpha subunit
TGME49_250330	-9.69	6.25	8.03E-10	2.39E-08	hypothetical protein
TGME49_294870	1.07	9.50	8.62E-10	2.56E-08	universal stress family protein
TGME49_315530	-4.70	6.48	8.69E-10	2.57E-08	hypothetical protein
TGME49_267500	1.21	8.83	8.74E-10	2.57E-08	hypothetical protein
TGME49_205010	-2.30	7.49	8.77E-10	2.57E-08	U2 small nuclear ribonucleoprotein family protein
TGME49_229020	-9.67	6.41	8.84E-10	2.58E-08	cell-cycle-associated protein kinase CDK, putative
TGME49_206690	-2.61	7.05	8.92E-10	2.60E-08	glideosome-associated protein with multiple-mem
TGME49_268230	-9.94	6.51	9.69E-10	2.80E-08	hypothetical protein
TGME49_319370	-9.68	6.12	9.73E-10	2.80E-08	hypothetical protein
TGME49_293710	-2.10	7.43	1.01E-09	2.91E-08	Zn-finger in Ran binding protein and others domai
TGME49_294690	1.74	7.53	1.05E-09	3.00E-08	rhomboid protease ROM5
TGME49_245510	-2.43	7.52	1.14E-09	3.24E-08	phospholipid-translocating P-type ATPase, flippase
TGME49_265390	-9.66	6.16	1.16E-09	3.29E-08	hypothetical protein
TGME49_213920	-3.43	6.79	1.17E-09	3.30E-08	hypothetical protein
TGME49_247360	2.42	7.48	1.18E-09	3.32E-08	PAP2 superfamily protein
TGME49_264830	-4.67	6.49	1.20E-09	3.35E-08	hypothetical protein
TGME49_202310	2.07	6.20	1.23E-09	3.43E-08	O-sialoglycoprotein endopeptidase
TGME49_234510	1.59	6.89	1.28E-09	3.54E-08	ankyrin repeat-containing protein
TGME49_290170	-2.49	7.34	1.27E-09	3.54E-08	kelch repeat domain containing/Serine/threonine

TGME49_278940	1.78	6.96	1.35E-09	3.70E-08	HECT-domain (ubiquitin-transferase) domain-cont
TGME49_277720	-2.34	7.54	1.34E-09	3.70E-08	GDA1/CD39 (nucleoside phosphatase) family prot
TGME49_265110	-9.66	6.03	1.35E-09	3.70E-08	ribosome biogenesis protein, putative
TGME49_310030	-2.04	7.62	1.39E-09	3.80E-08	cyclase-associated protein, putative
TGME49_315100	4.95	4.83	1.43E-09	3.87E-08	hypothetical protein
TGME49_254520	1.40	7.43	1.47E-09	3.99E-08	mediator complex subunit MED11
TGME49_212170	-9.63	5.94	1.48E-09	3.99E-08	GIY-YIG catalytic domain-containing protein
TGME49_265870	-3.46	6.97	1.60E-09	4.32E-08	pantoate-beta-alanine ligase
TGME49_213010	-4.86	7.34	1.65E-09	4.43E-08	hypothetical protein
TGME49_286460	3.16	5.00	1.66E-09	4.43E-08	hypothetical protein
TGME49_254410	1.95	6.78	1.77E-09	4.70E-08	protein phosphatase 2C, putative
TGME49_230410	-1.96	8.00	1.80E-09	4.78E-08	peroxiredoxin PRX3
TGME49_208020	2.18	6.12	1.88E-09	4.97E-08	AP2 domain transcription factor AP2Ib-1
TGME49_313290	1.71	6.91	1.89E-09	4.97E-08	MORN repeat-containing protein
TGME49_309990	4.23	6.06	1.92E-09	5.04E-08	hypothetical protein
TGME49_315780	-3.14	6.99	1.96E-09	5.13E-08	myosin regulatory light chain, putative
TGME49_289370	1.70	7.66	1.99E-09	5.17E-08	hypothetical protein
TGME49_224880	-3.47	7.04	2.09E-09	5.39E-08	kinesin motor domain-containing protein
TGME49_240870	-2.71	7.10	2.12E-09	5.46E-08	beta adaptin protein, putative
TGME49_277940	-3.85	6.79	2.12E-09	5.46E-08	hypothetical protein
TGME49_252880	-2.57	7.29	2.13E-09	5.46E-08	hypothetical protein
TGME49_254050	1.64	6.83	2.15E-09	5.48E-08	optic atrophy 3 protein (opa3) protein
TGME49_239590	-4.66	6.37	2.17E-09	5.52E-08	WD domain, G-beta repeat-containing protein
TGME49_237550	2.81	6.56	2.37E-09	5.97E-08	hypothetical protein
TGME49_258150	-1.92	7.78	2.36E-09	5.97E-08	proteasome subunit alpha type 7, putative
TGME49_297745	-4.27	6.99	2.61E-09	6.51E-08	hypothetical protein
TGME49_239270	-2.16	7.56	2.83E-09	7.05E-08	hypothetical protein
TGME49_281400	-3.21	6.55	2.89E-09	7.17E-08	phosphofructokinase domain-containing protein
TGME49_318660	1.26	8.55	2.95E-09	7.28E-08	PP2C, putative
TGME49_305060	1.48	7.66	3.02E-09	7.43E-08	CAP-Gly domain-containing protein
TGME49_262970	1.65	7.12	3.05E-09	7.48E-08	hypothetical protein
TGME49_229690	-9.57	6.55	3.40E-09	8.34E-08	autophagy-related protein 7 atg7, putative
TGME49_214575	2.05	5.96	3.50E-09	8.52E-08	hypothetical protein
TGME49_229470	-4.65	6.35	3.51E-09	8.52E-08	hypothetical protein
TGME49_251440	-9.71	5.79	3.51E-09	8.52E-08	troponin c, isotype gamma, putative
TGME49_226705	-9.69	6.39	3.75E-09	9.06E-08	hypothetical protein
TGME49_246490	-2.25	7.46	3.81E-09	9.17E-08	hypothetical protein
TGME49_220880	1.93	6.18	3.90E-09	9.36E-08	hypothetical protein
TGME49_225580	-9.55	5.94	3.96E-09	9.47E-08	proteasome (prosome, macropain) 26S subunit, n
TGME49_209060	-3.78	6.66	4.01E-09	9.56E-08	thrombospondin type 1 domain-containing protei
TGME49_265790	1.15	8.70	4.03E-09	9.58E-08	hypothetical protein
TGME49_215590	-1.91	8.31	4.13E-09	9.80E-08	flavoprotein subunit of succinate dehydrogenase
TGME49_247960	2.06	6.05	4.26E-09	1.01E-07	hypothetical protein
TGME49_214140	1.26	8.62	4.41E-09	1.04E-07	hypothetical protein
TGME49_218560	-1.88	7.74	4.44E-09	1.04E-07	acetyl-coA carboxylase ACC2
TGME49_214230	1.64	6.85	4.48E-09	1.05E-07	Dopey, N-terminal domain-containing protein
TGME49_254370	-1.66	8.18	4.56E-09	1.06E-07	guanylyl cyclase
TGME49_260180	-1.99	7.68	4.57E-09	1.06E-07	hypothetical protein
TGME49_203830	-3.79	6.38	4.78E-09	1.11E-07	FHA domain-containing protein
TGME49_265500	-9.53	6.01	5.20E-09	1.20E-07	chloride transporter, chloride channel (ClC) family
TGME49_219730	1.55	7.57	5.27E-09	1.21E-07	hypothetical protein
TGME49_272010	-4.60	6.12	5.43E-09	1.25E-07	Gar1 protein RNA binding region protein
TGME49_254220	2.33	5.45	5.46E-09	1.25E-07	hypothetical protein
TGME49_237890	-4.58	6.17	5.51E-09	1.26E-07	calcium-dependent protein kinase CDPK4

TGME49_218362	-2.67	6.89	5.53E-09	1.26E-07	zinc finger protein ZFP1
TGME49_209440	-2.15	7.38	5.72E-09	1.30E-07	hypothetical protein
TGME49_232710	-1.08	10.10	5.82E-09	1.32E-07	ribosomal protein RPS3A
TGME49_222860	-1.58	8.81	5.86E-09	1.32E-07	eukaryotic translation initiation factor, putative
TGME49_274190	-4.63	6.59	5.89E-09	1.32E-07	eukaryotic initiation factor 2B epsilon subunit, put
TGME49_248260	2.20	6.49	5.96E-09	1.34E-07	hypothetical protein
TGME49_254030	2.15	8.18	6.03E-09	1.35E-07	zinc finger CDGSH-type domain-containing protein
TGME49_295080	-9.53	5.79	6.08E-09	1.35E-07	hypothetical protein
TGME49_267620	-3.12	6.97	6.12E-09	1.36E-07	multi-pass transmembrane protein
TGME49_246570	2.72	5.60	6.28E-09	1.39E-07	hypothetical protein
TGME49_231150	2.64	5.08	6.30E-09	1.39E-07	hypothetical protein
TGME49_211250	-3.07	6.81	6.32E-09	1.39E-07	hypothetical protein
TGME49_231950	1.86	7.62	6.39E-09	1.40E-07	hypothetical protein
TGME49_289300	-2.17	7.33	6.46E-09	1.41E-07	methionyl-tRNA synthetase
TGME49_205360	-2.51	7.08	6.48E-09	1.41E-07	hypothetical protein
TGME49_251170	2.93	6.41	6.62E-09	1.44E-07	KRUF family protein
TGME49_276970	-3.33	6.73	7.01E-09	1.52E-07	hypothetical protein
TGME49_269660	-2.64	6.75	7.03E-09	1.52E-07	TFIIH basal transcription factor complex helicase X
TGME49_252390	1.43	8.54	7.33E-09	1.58E-07	hypothetical protein
TGME49_266750	-1.78	7.68	7.40E-09	1.59E-07	transporter/permease protein, putative
TGME49_221675	1.34	7.82	7.59E-09	1.62E-07	hypothetical protein
TGME49_266990	-1.79	7.81	7.59E-09	1.62E-07	beta-COP
TGME49_312110	-2.50	7.05	7.92E-09	1.68E-07	apicoplast-associated thioredoxin family protein A
TGME49_268176	-2.82	7.14	8.33E-09	1.76E-07	hypothetical protein
TGME49_237170	-3.09	6.80	8.42E-09	1.78E-07	hypothetical protein
TGME49_238100	-2.33	7.30	8.48E-09	1.79E-07	transmembrane protein
TGME49_254730	1.89	6.04	8.67E-09	1.82E-07	POPLD (NUC188) domain-containing protein
TGME49_235020	-1.87	7.85	8.68E-09	1.82E-07	COPI protein, putative
TGME49_262150	-9.48	5.98	8.71E-09	1.82E-07	kelch repeat and K+ channel tetramerisation doma
TGME49_295990	1.50	7.35	8.78E-09	1.83E-07	ubiquitin conjugating enzyme E2, putative
TGME49_249530	-1.80	7.70	8.84E-09	1.84E-07	exportin 1, putative
TGME49_319880	-4.52	6.31	8.87E-09	1.84E-07	MORN repeat-containing protein
TGME49_283540	1.64	8.26	8.96E-09	1.85E-07	hypothetical protein
TGME49_229420	-9.48	6.04	9.16E-09	1.89E-07	cytochrome c, putative
TGME49_310460	-2.85	7.01	9.41E-09	1.93E-07	Rab6
TGME49_204520	2.74	4.97	9.45E-09	1.94E-07	hypothetical protein
TGME49_222710	-2.82	6.78	9.73E-09	1.99E-07	IMP-specific 5'-nucleotidase 1, putative
TGME49_213660	-1.51	9.72	1.02E-08	2.08E-07	zinc finger (CCCH type) motif-containing protein
TGME49_202470	-9.48	5.84	1.06E-08	2.15E-07	rRNA metabolism protein, SBDS family protein
TGME49_290980	-2.82	6.72	1.08E-08	2.18E-07	glycine C-acetyltransferase, putative
TGME49_263550	1.66	6.57	1.16E-08	2.35E-07	39S ribosomal protein L47, mitochondrial precurso
TGME49_219080	-9.48	5.98	1.19E-08	2.39E-07	edge expressed protein, putative
TGME49_293740	1.80	8.89	1.22E-08	2.45E-07	hypothetical protein
TGME49_263490	2.11	5.71	1.23E-08	2.46E-07	ubiquitin conjugating enzyme E2, putative
TGME49_219810	1.87	8.82	1.24E-08	2.46E-07	hypothetical protein
TGME49_205040	-1.79	7.70	1.24E-08	2.46E-07	PGAP1 family protein
TGME49_270770	-4.56	6.36	1.23E-08	2.46E-07	PWI domain-containing protein
TGME49_213067	1.34	7.78	1.28E-08	2.54E-07	hypothetical protein
TGME49_237015	1.45	7.91	1.30E-08	2.57E-07	hypothetical protein
TGME49_280730	-9.46	5.83	1.31E-08	2.58E-07	cytosolic fe-s cluster assembling factor nbp35, put
TGME49_227420	-9.48	5.89	1.48E-08	2.91E-07	4-hydroxy-3-methylbut-2-enyl diphosphate reduct
TGME49_310220	-4.57	6.48	1.49E-08	2.93E-07	hypothetical protein
TGME49_318400	-2.00	7.52	1.51E-08	2.95E-07	hypothetical protein
TGME49_257520	1.53	7.16	1.59E-08	3.10E-07	synaptobrevin protein

TGME49_226220	-2.77	6.89	1.65E-08	3.20E-07	alveolin domain containing intermediate filament
TGME49_244910	-3.71	6.82	1.65E-08	3.20E-07	MIZ/SP-RING zinc finger domain-containing protei
TGME49_309380	-3.25	6.60	1.69E-08	3.27E-07	Nuf2
TGME49_274110	-9.43	5.68	1.70E-08	3.28E-07	glycoprotease family protein
TGME49_225250	2.04	5.93	1.71E-08	3.30E-07	LSU ribosomal protein L14P, putative
TGME49_223010	-3.26	6.45	1.75E-08	3.35E-07	hypothetical protein
TGME49_278720	-9.47	5.75	1.81E-08	3.47E-07	hypothetical protein
TGME49_320130	1.97	5.97	1.85E-08	3.53E-07	hypothetical protein
TGME49_267690	-4.49	6.16	1.97E-08	3.76E-07	hypothetical protein
TGME49_222930	-2.77	6.77	1.99E-08	3.78E-07	hypothetical protein
TGME49_297800	-3.02	6.62	1.99E-08	3.78E-07	RecF/RecN/SMC N terminal domain-containing pr
TGME49_203730	-9.41	5.89	2.03E-08	3.83E-07	hypothetical protein
TGME49_226910	-4.49	6.44	2.06E-08	3.88E-07	Amylo-alpha-1,6-glucosidase
TGME49_266410	1.84	6.23	2.10E-08	3.95E-07	hypothetical protein
TGME49_292130	-1.14	9.66	2.22E-08	4.17E-07	ribosomal protein RPL13A
TGME49_289730	-2.34	7.06	2.23E-08	4.17E-07	Pep3/Vps18/deep orange family protein
TGME49_201870	-2.79	6.58	2.36E-08	4.40E-07	tetratricopeptide repeat-containing protein
TGME49_216020	-9.41	6.06	2.36E-08	4.40E-07	peptidase family c78 protein
TGME49_313445	2.13	5.72	2.39E-08	4.44E-07	hypothetical protein
TGME49_225990	-3.24	6.32	2.42E-08	4.49E-07	acyl transferase domain-containing protein
TGME49_240860	-3.69	6.54	2.45E-08	4.53E-07	acyltransferase domain-containing protein
TGME49_285140	-9.42	6.26	2.55E-08	4.70E-07	hypothetical protein
TGME49_319580	-9.39	5.99	2.67E-08	4.91E-07	hypothetical protein
TGME49_247580	-9.41	5.86	2.78E-08	5.10E-07	glutaredoxin domain-containing protein
TGME49_253470	1.68	7.35	2.98E-08	5.45E-07	alveolin domain containing intermediate filament
TGME49_246100	-2.64	6.79	2.99E-08	5.45E-07	phosducin, putative
TGME49_270930	-3.02	6.90	3.05E-08	5.56E-07	hypothetical protein
TGME49_241240	1.03	9.79	3.27E-08	5.94E-07	hypothetical protein
TGME49_294940	1.69	6.95	3.42E-08	6.16E-07	hypothetical protein
TGME49_226500	-4.54	6.63	3.42E-08	6.16E-07	hypothetical protein
TGME49_226430	-1.44	8.72	3.45E-08	6.20E-07	reticulon protein
TGME49_299250	2.72	6.36	3.55E-08	6.36E-07	hypothetical protein
TGME49_288820	1.59	6.83	3.59E-08	6.43E-07	hypothetical protein
TGME49_247760	-2.95	6.83	3.67E-08	6.55E-07	AMP-binding enzyme domain-containing protein
TGME49_311720	-1.01	11.50	3.74E-08	6.66E-07	chaperonin protein BiP
TGME49_306660	1.31	7.88	3.78E-08	6.71E-07	RNA pseudouridine synthase superfamily protein
TGME49_254000	1.44	7.06	3.80E-08	6.73E-07	hypothetical protein
TGME49_233140	-2.54	7.17	3.90E-08	6.90E-07	deoxyuridine 5'-triphosphate nucleotidohydrolase
TGME49_280400	-9.41	7.58	4.12E-08	7.26E-07	hypothetical protein
TGME49_290160	-1.66	8.18	4.31E-08	7.59E-07	sortilin, putative
TGME49_271470	-4.41	6.12	4.36E-08	7.66E-07	hypothetical protein
TGME49_214960	1.60	7.09	4.38E-08	7.68E-07	AP2 domain transcription factor AP2X-8
TGME49_202920	-9.36	6.07	4.50E-08	7.87E-07	p-aminobenzoic acid synthase
TGME49_309590	1.01	12.11	4.52E-08	7.89E-07	rhoptry protein ROP1
TGME49_270270	-3.68	6.69	4.64E-08	8.07E-07	hypothetical protein
TGME49_271810	-1.62	7.84	4.80E-08	8.33E-07	lanp, putative
TGME49_286260	-9.33	5.83	4.86E-08	8.41E-07	tetratricopeptide repeat-containing protein
TGME49_216770	-4.44	5.97	4.88E-08	8.43E-07	hypothetical protein
TGME49_205750	-2.90	6.42	4.99E-08	8.60E-07	histone deacetylase complex subunit Sin3
TGME49_201400	-4.43	6.24	5.03E-08	8.66E-07	Sin3-associated polypeptide SAP18
TGME49_273905	-4.39	6.09	5.08E-08	8.71E-07	hypothetical protein
TGME49_249360	-2.40	6.90	5.17E-08	8.84E-07	RED family protein
TGME49_310450	-2.42	7.13	5.17E-08	8.84E-07	myosin heavy chain, putative
TGME49_248860	2.36	5.75	5.24E-08	8.93E-07	hypothetical protein

TGME49_222400	1.21	7.58	5.36E-08	9.12E-07	hypothetical protein
TGME49_313880	-9.36	5.91	5.40E-08	9.16E-07	nuclear protein-like family protein
TGME49_247220	1.32	8.50	5.45E-08	9.21E-07	nudix -type motif 9 isoform a family protein
TGME49_246930	-2.21	7.26	5.45E-08	9.21E-07	calmodulin CAM1
TGME49_259710	-2.95	7.07	5.48E-08	9.24E-07	protein kinase
TGME49_313380	-1.15	9.56	5.74E-08	9.64E-07	hypothetical protein
TGME49_214200	-9.38	5.94	5.75E-08	9.64E-07	WD domain, G-beta repeat-containing protein
TGME49_243930	1.38	8.92	5.88E-08	9.84E-07	hypothetical protein
TGME49_215420	-9.31	5.72	6.08E-08	1.02E-06	SNARE protein
TGME49_311680	-1.89	7.31	6.15E-08	1.02E-06	FUN14 family protein
TGME49_259200	-4.39	6.05	6.21E-08	1.03E-06	Na+/H+ exchanger NHE1
TGME49_268580	-4.42	6.06	6.21E-08	1.03E-06	hypothetical protein
TGME49_269450	2.62	5.12	6.39E-08	1.06E-06	hypothetical protein
TGME49_266450	1.76	6.38	6.57E-08	1.08E-06	lysine decarboxylase family protein
TGME49_288360	-1.46	10.08	6.63E-08	1.09E-06	tryptophanyl-tRNA synthetase (TrpRS2)
TGME49_261480	1.77	6.87	6.82E-08	1.12E-06	phosphatidyl serine synthase
TGME49_210245	-9.40	5.61	6.91E-08	1.13E-06	hypothetical protein
TGME49_288400	-9.31	6.03	6.97E-08	1.14E-06	LETM1 family protein
TGME49_226810	-3.20	6.80	7.05E-08	1.15E-06	histone lysine methyltransferase SET1
TGME49_239800	-2.88	6.90	7.08E-08	1.15E-06	hypothetical protein
TGME49_270550	-9.37	5.78	7.28E-08	1.18E-06	gamma-glutamyl phosphate reductase, putative
TGME49_249250	1.28	10.07	7.46E-08	1.20E-06	ribosomal protein RPL35A
TGME49_207880	-9.44	6.72	7.50E-08	1.21E-06	hypothetical protein
TGME49_269920	-1.46	8.62	7.76E-08	1.24E-06	phosphatidylserine decarboxylase
TGME49_278815	-3.62	6.59	8.03E-08	1.29E-06	hypothetical protein
TGME49_207690	-4.37	6.36	8.14E-08	1.30E-06	programmed cell death 5 protein
TGME49_203720	2.55	8.00	8.47E-08	1.35E-06	vitamin k epoxide reductase family protein
TGME49_237500	1.34	7.44	8.57E-08	1.36E-06	protein phosphatase 2C domain-containing protei
TGME49_320090	-3.11	6.48	9.07E-08	1.44E-06	hypothetical protein
TGME49_286630	-1.68	7.93	9.41E-08	1.49E-06	redoxin domain-containing protein
TGME49_257380	1.16	8.41	9.45E-08	1.49E-06	hypothetical protein
TGME49_225050	-1.78	7.46	9.59E-08	1.51E-06	adenosylhomocysteinase, putative
TGME49_305980	-2.30	7.06	9.96E-08	1.57E-06	pyruvate dehydrogenase complex subunit PDH-E3
TGME49_289600	1.41	7.82	1.01E-07	1.59E-06	heat shock protein HSP29
TGME49_223760	-9.25	6.03	1.07E-07	1.67E-06	hypothetical protein
TGME49_289050	1.24	8.43	1.09E-07	1.70E-06	FIKK kinase, putative
TGME49_213060	1.66	6.59	1.13E-07	1.76E-06	WD domain, G-beta repeat-containing protein
TGME49_269690	1.26	8.07	1.15E-07	1.79E-06	hypothetical protein
TGME49_228750	-3.59	6.46	1.16E-07	1.80E-06	TGME49_228750 CAM kinase, RAD family
TGME49_315250	-4.35	6.00	1.18E-07	1.83E-06	GAMM1 protein, putative
TGME49_320490	1.85	8.78	1.19E-07	1.84E-06	N-acyl-phosphatidylethanolamine-hydrolyzing ph
TGME49_312300	-1.87	7.41	1.20E-07	1.85E-06	Sec7 domain-containing protein
TGME49_260670	-9.26	5.92	1.23E-07	1.89E-06	centrin, putative
TGME49_273815	-9.34	5.51	1.25E-07	1.92E-06	hypothetical protein
TGME49_299200	1.77	7.09	1.25E-07	1.92E-06	Bet3 transport protein, putative
TGME49_236250	2.11	5.67	1.27E-07	1.95E-06	regulator of chromosome condensation (RCC1) re
TGME49_204310	-1.40	8.50	1.32E-07	2.02E-06	hypothetical protein
TGME49_292160	1.77	6.10	1.37E-07	2.09E-06	hypothetical protein
TGME49_234980	1.96	6.57	1.39E-07	2.11E-06	hypothetical protein
TGME49_215390	2.45	4.82	1.42E-07	2.15E-06	TIM10 family protein, putative
TGME49_291940	-4.30	6.39	1.43E-07	2.16E-06	hypothetical protein
TGME49_234390	-9.29	5.43	1.43E-07	2.16E-06	hypothetical protein
TGME49_289580	-9.23	6.04	1.47E-07	2.22E-06	strictosidine synthase subfamily protein
TGME49_205510	-1.74	7.70	1.48E-07	2.23E-06	nucleolar protein 5, putative

TGME49_224540	-9.38	6.05	1.48E-07	2.23E-06	hypothetical protein
TGME49_284190	-3.70	6.62	1.50E-07	2.25E-06	pyruvate carboxylase
TGME49_227060	-9.25	5.72	1.51E-07	2.26E-06	hypothetical protein
TGME49_221720	-4.62	6.44	1.53E-07	2.28E-06	hypothetical protein
TGME49_238200	1.96	6.96	1.61E-07	2.40E-06	alpha/beta hydrolase fold domain-containing prot
TGME49_304460	-1.70	7.52	1.63E-07	2.43E-06	zinc finger, C3HC4 type (RING finger) domain-cont
TGME49_205540	-9.24	5.64	1.66E-07	2.46E-06	DEAD/DEAH box helicase domain-containing prote
TGME49_269650	-1.97	7.00	1.67E-07	2.47E-06	FFD and TFG box motifs protein
TGME49_320588	-9.23	6.31	1.68E-07	2.48E-06	glycosyl hydrolases family 35 protein
TGME49_320640	-9.26	6.10	1.77E-07	2.60E-06	peptidylprolyl isomerase domain-containing prote
TGME49_265010	-9.20	5.64	1.82E-07	2.67E-06	glutamate 5-kinase domain-containing protein
TGME49_314540	-9.20	5.67	1.84E-07	2.70E-06	hypothetical protein
TGME49_204420	3.59	6.08	1.90E-07	2.78E-06	oocyst wall protein OWP1
TGME49_263630	1.20	7.63	1.91E-07	2.78E-06	hypothetical protein
TGME49_288945	-2.45	6.28	1.92E-07	2.80E-06	hypothetical protein
TGME49_240570	-2.67	6.80	1.95E-07	2.83E-06	hypothetical protein
TGME49_249590	-1.74	7.72	2.01E-07	2.91E-06	proteasome subunit alpha type 5-2, putative
TGME49_216430	-1.75	7.55	2.05E-07	2.96E-06	TBC domain-containing protein
TGME49_282000	1.79	6.72	2.08E-07	3.00E-06	hypothetical protein
TGME49_242055	-9.20	5.77	2.08E-07	3.00E-06	DEAD/DEAH box helicase domain-containing prote
TGME49_318210	-9.22	5.53	2.10E-07	3.02E-06	hypothetical protein
TGME49_246970	-9.20	5.55	2.12E-07	3.05E-06	3'-5' exonuclease domain-containing protein
TGME49_280770	-1.52	7.52	2.19E-07	3.14E-06	regulator of chromosome condensation (RCC1) re
TGME49_319910	1.82	5.94	2.20E-07	3.14E-06	WD domain, G-beta repeat-containing protein
TGME49_222960	-9.20	5.95	2.22E-07	3.16E-06	SCY kinase-related protein (incomplete catalytic tr
TGME49_239740	1.01	9.34	2.30E-07	3.27E-06	dense granule protein GRA14
TGME49_310930	-3.09	6.48	2.31E-07	3.28E-06	hypothetical protein
TGME49_278630	-9.17	5.70	2.37E-07	3.35E-06	tetratricopeptide repeat-containing protein
TGME49_220950	1.20	9.89	2.39E-07	3.38E-06	hypothetical protein
TGME49_225745	-9.20	6.00	2.40E-07	3.38E-06	hypothetical protein
TGME49_207100	-9.17	5.81	2.41E-07	3.40E-06	hypothetical protein
TGME49_271460	2.14	6.76	2.42E-07	3.40E-06	protein c14orf29, putative
TGME49_239752	-1.50	8.30	2.44E-07	3.42E-06	hypothetical protein
TGME49_297870	-2.35	6.90	2.50E-07	3.50E-06	hypothetical protein
TGME49_226590	1.50	9.66	2.58E-07	3.59E-06	cytochrome C oxidase subunit IIa, putative
TGME49_236890	1.44	7.15	2.58E-07	3.59E-06	hypothetical protein
TGME49_241890	-9.17	5.67	2.58E-07	3.59E-06	hypothetical protein
TGME49_228770	-4.30	5.98	2.59E-07	3.59E-06	hypothetical protein
TGME49_222210	-9.17	5.46	2.59E-07	3.59E-06	SPFH domain / Band 7 family protein
TGME49_271060	-4.26	5.87	2.64E-07	3.66E-06	Sec1 family protein
TGME49_253180	2.52	7.48	2.73E-07	3.76E-06	hypothetical protein
TGME49_203740	-2.48	6.73	2.77E-07	3.81E-06	hypothetical protein
TGME49_205280	1.30	7.65	2.78E-07	3.82E-06	hypothetical protein
TGME49_313400	-2.19	7.11	2.91E-07	3.98E-06	DnaJ domain-containing protein
TGME49_201680	-1.13	8.83	2.94E-07	4.02E-06	eukaryotic initiation factor-3 subunit 10, putative
TGME49_311070	-3.53	6.42	2.96E-07	4.04E-06	hypothetical protein
TGME49_205200	1.37	6.83	2.97E-07	4.04E-06	hypothetical protein
TGME49_250670	1.80	6.89	3.02E-07	4.11E-06	hypothetical protein
TGME49_212270	2.01	9.40	3.03E-07	4.11E-06	hypothetical protein
TGME49_219860	-1.38	8.73	3.05E-07	4.13E-06	replication licensing factor, putative
TGME49_248490	2.15	6.17	3.09E-07	4.18E-06	hypothetical protein
TGME49_245730	-9.14	5.76	3.16E-07	4.25E-06	phosphatidylinositol-4-phosphate 5-Kinase
TGME49_203560	-9.21	5.94	3.16E-07	4.25E-06	hypothetical protein
TGME49_282150	-9.14	5.86	3.30E-07	4.43E-06	hypothetical protein

TGME49_239020	-9.22	6.19	3.32E-07	4.45E-06	ABC transporter transmembrane region domain-c
TGME49_306440	-9.14	6.04	3.36E-07	4.49E-06	hypothetical protein
TGME49_305860	-1.98	7.93	3.47E-07	4.64E-06	calcium-dependent protein kinase CDPK3
TGME49_216070	-9.14	5.74	3.48E-07	4.64E-06	hypothetical protein
TGME49_209730	-9.14	5.93	3.48E-07	4.64E-06	hypothetical protein
TGME49_259090	1.54	6.78	3.49E-07	4.64E-06	ubiquitin-conjugating enzyme subfamily protein
TGME49_209280	-4.24	6.12	3.50E-07	4.64E-06	hypothetical protein
TGME49_248530	-9.14	5.80	3.54E-07	4.69E-06	FATC domain-containing protein
TGME49_258470	1.18	11.23	3.55E-07	4.70E-06	hypothetical protein
TGME49_249650	-9.14	5.91	3.69E-07	4.87E-06	apolipoprotein A-I binding protein, putative
TGME49_278975	-2.28	6.87	3.70E-07	4.88E-06	ICE family protease (caspase) p20 domain-contain
TGME49_204360	2.57	6.03	3.72E-07	4.89E-06	subtilisin SUB4
TGME49_202572	-9.14	5.64	3.76E-07	4.94E-06	ribophorin i protein
TGME49_210230	-9.18	5.90	3.78E-07	4.95E-06	hypothetical protein
TGME49_262040	-2.58	6.75	3.82E-07	4.99E-06	SAC3/GANP family protein
TGME49_223855	2.71	6.35	3.94E-07	5.14E-06	RNA recognition motif-containing protein
TGME49_240520	-9.14	5.59	3.97E-07	5.17E-06	hypothetical protein
TGME49_221350	-4.21	5.68	3.99E-07	5.18E-06	Ctr copper transporter family protein
TGME49_278700	-3.46	5.93	4.07E-07	5.27E-06	hypothetical protein
TGME49_280390	-2.58	6.88	4.07E-07	5.28E-06	HEAT repeat-containing protein
TGME49_294270	-9.11	5.61	4.12E-07	5.32E-06	histone arginine methyltransferase PRMT4/CARM
TGME49_249470	-9.11	5.77	4.12E-07	5.32E-06	rhoptry kinase family protein, truncated (incomple
TGME49_225800	-3.47	6.11	4.25E-07	5.48E-06	iron-sulfur assembly ATPase
TGME49_282220	1.49	6.58	4.33E-07	5.56E-06	AP2 domain transcription factor AP2VIIa-9
TGME49_264600	-2.40	6.95	4.38E-07	5.62E-06	hypothetical protein
TGME49_297400	1.36	7.21	4.40E-07	5.64E-06	hypothetical protein
TGME49_224960	1.35	7.07	4.43E-07	5.67E-06	hypothetical protein
TGME49_251410	-2.32	6.67	4.44E-07	5.67E-06	tetratricopeptide repeat-containing protein
TGME49_254870	1.36	6.87	4.56E-07	5.81E-06	hypothetical protein
TGME49_200320	-2.28	7.39	4.67E-07	5.95E-06	hypoxanthine-xanthine-guanine phosphoribosyl tr
TGME49_294550	-1.62	8.00	4.80E-07	6.09E-06	dynein heavy chain
TGME49_318880	2.77	5.55	5.09E-07	6.44E-06	hypothetical protein
TGME49_305240	-3.04	6.60	5.13E-07	6.47E-06	XPA binding protein 2 family protein
TGME49_223800	-4.23	5.96	5.30E-07	6.67E-06	hypothetical protein
TGME49_286790	-4.19	6.16	5.33E-07	6.70E-06	nuclear factor NF2
TGME49_316200	1.65	6.43	5.48E-07	6.87E-06	phosphoglycerate mutase family protein
TGME49_266730	-9.08	5.67	5.53E-07	6.93E-06	leucyl-tRNA synthetase (LeuRS2)
TGME49_257700	2.48	5.46	5.88E-07	7.36E-06	hypothetical protein
TGME49_234460	1.47	6.73	5.90E-07	7.37E-06	hypothetical protein
TGME49_236670	1.93	6.27	6.00E-07	7.47E-06	hypothetical protein
TGME49_270140	-9.11	5.48	6.00E-07	7.47E-06	splicing factor DIM1, putative
TGME49_268760	-1.32	8.36	6.03E-07	7.50E-06	hypothetical protein
TGME49_306960	-2.52	6.60	6.24E-07	7.75E-06	phenylalanine--tRNA ligase, beta subunit protein
TGME49_272770	-4.19	5.80	6.37E-07	7.89E-06	hypothetical protein
TGME49_314920	2.44	5.56	6.50E-07	8.03E-06	hypothetical protein
TGME49_251500	-1.41	8.09	6.52E-07	8.03E-06	eukaryotic initiation factor-3, subunit 3, putative
TGME49_244500	-1.77	7.47	6.51E-07	8.03E-06	Tubulin-tyrosine ligase family protein
TGME49_295658	-3.40	6.32	6.59E-07	8.11E-06	zinc finger in N-recognin protein
TGME49_294990	-9.08	6.22	6.61E-07	8.12E-06	hypothetical protein
TGME49_249460	-9.08	5.36	6.67E-07	8.16E-06	WD domain, G-beta repeat-containing protein
TGME49_314700	1.38	7.77	6.72E-07	8.21E-06	hypothetical protein
TGME49_236860	-4.25	6.13	6.84E-07	8.34E-06	haloacid dehalogenase family hydrolase domain-c
TGME49_291020	-2.13	6.79	6.96E-07	8.48E-06	myosin head (motor domain) domain-containing p
TGME49_289700	-3.00	6.24	7.03E-07	8.55E-06	hypothetical protein

TGME49_200350	2.37	5.77	7.11E-07	8.63E-06	subtilisin SUB3
TGME49_257350	1.19	8.60	7.33E-07	8.89E-06	eukaryotic translation initiation factor, putative
TGME49_301380	-4.21	6.01	7.50E-07	9.07E-06	elongation factor Tu GTP binding domain-containi
TGME49_310140	-9.05	5.76	7.74E-07	9.35E-06	hypothetical protein
TGME49_213325	-9.08	5.93	7.95E-07	9.59E-06	TBC domain-containing protein
TGME49_221270	-1.90	6.87	8.03E-07	9.67E-06	oxidoreductase, 2OG-Fe(II) oxygenase family prote
TGME49_227030	-2.15	6.87	8.22E-07	9.88E-06	hypothetical protein
TGME49_215040	-2.38	6.64	8.30E-07	9.96E-06	HEAT repeat-containing protein
TGME49_299970	-1.47	8.14	8.33E-07	9.98E-06	tetratricopeptide repeat-containing protein
TGME49_274010	-4.16	6.06	8.35E-07	9.99E-06	hypothetical protein
TGME49_305470	-9.05	5.79	8.83E-07	1.06E-05	hypothetical protein
TGME49_231910	-1.73	7.36	9.13E-07	1.09E-05	ATP synthase F1 gamma subunit
TGME49_313830	-2.96	6.34	9.16E-07	1.09E-05	AARP2CN (NUC121) domain-containing protein
TGME49_228730	-9.05	5.41	9.49E-07	1.13E-05	hypothetical protein
TGME49_257050	-9.02	5.79	9.52E-07	1.13E-05	3-methyl-2-oxobutanoate hydroxymethyltransfera
TGME49_220430	-9.02	5.67	9.60E-07	1.14E-05	hypothetical protein
TGME49_240880	2.26	5.88	9.73E-07	1.15E-05	hypothetical protein
TGME49_291680	-1.53	7.77	9.90E-07	1.17E-05	Sec23/Sec24 trunk domain-containing protein
TGME49_235398	-9.02	5.77	9.91E-07	1.17E-05	hypothetical protein
TGME49_271270	-1.73	7.33	9.98E-07	1.17E-05	hypothetical protein
TGME49_248450	-2.96	6.26	1.00E-06	1.17E-05	zinc finger, C3HC4 type (RING finger) domain-cont
TGME49_225480	-4.14	5.99	1.03E-06	1.20E-05	hypothetical protein
TGME49_287270	-2.97	6.15	1.06E-06	1.24E-05	hypothetical protein
TGME49_215570	-3.39	6.18	1.07E-06	1.24E-05	AP2 domain transcription factor AP2X-11
TGME49_279340	1.67	6.26	1.08E-06	1.25E-05	hypothetical protein
TGME49_257490	-3.40	6.62	1.07E-06	1.25E-05	prefoldin subunit superfamily protein
TGME49_260390	-9.02	5.56	1.08E-06	1.25E-05	hypothetical protein
TGME49_272410	1.37	6.75	1.09E-06	1.27E-05	phosphogluconate dehydrogenase (decarboxylatin
TGME49_210760	-9.02	5.47	1.11E-06	1.28E-05	glutamine amidotransferase-related, putative
TGME49_264080	-1.51	8.18	1.11E-06	1.28E-05	acyl carrier protein ACP
TGME49_269970	1.29	6.91	1.13E-06	1.31E-05	hypothetical protein
TGME49_264130	1.98	5.62	1.14E-06	1.31E-05	hypothetical protein
TGME49_313780	-1.63	7.72	1.14E-06	1.31E-05	hypothetical protein
TGME49_214750	1.30	7.32	1.14E-06	1.31E-05	hypothetical protein
TGME49_253090	1.71	5.86	1.15E-06	1.32E-05	DEAD/DEAH box helicase domain-containing prote
TGME49_306460	-9.02	5.67	1.17E-06	1.34E-05	bromodomain-containing protein
TGME49_313020	-3.36	6.61	1.17E-06	1.34E-05	STAS domain-containing protein
TGME49_218740	3.24	5.57	1.18E-06	1.34E-05	membrane protein, putative
TGME49_291050	-1.84	7.12	1.18E-06	1.34E-05	histone kinase SNF1, putative
TGME49_219270	-1.01	10.13	1.19E-06	1.35E-05	multi-pass transmembrane protein
TGME49_310530	-4.14	6.15	1.21E-06	1.37E-05	SNF2 family N-terminal domain-containing protein
TGME49_227948	-1.31	8.32	1.21E-06	1.37E-05	peptidase M16 inactive domain-containing protein
TGME49_306520	1.91	7.15	1.22E-06	1.37E-05	tRNA pseudouridine synthase B, putative
TGME49_282040	-4.17	5.99	1.22E-06	1.37E-05	hypothetical protein
TGME49_225770	-8.99	5.71	1.26E-06	1.42E-05	Tyrosine kinase-like (TKL) protein
TGME49_205460	-8.99	5.47	1.28E-06	1.44E-05	AN1 family Zinc finger domain-containing protein
TGME49_214580	-4.14	5.36	1.29E-06	1.45E-05	tetratricopeptide repeat-containing protein
TGME49_268890	-1.88	7.12	1.32E-06	1.48E-05	citrate synthase I
TGME49_253600	2.37	6.68	1.33E-06	1.49E-05	hypothetical protein
TGME49_289750	-1.06	9.50	1.34E-06	1.49E-05	ribosomal-ubiquitin protein RPL40
TGME49_306310	-2.51	6.64	1.34E-06	1.49E-05	RecF/RecN/SMC N terminal domain-containing pro
TGME49_290970	-1.58	7.23	1.35E-06	1.50E-05	8-amino-7-oxononanoate synthase
TGME49_320450	-3.41	6.15	1.35E-06	1.50E-05	ribosome biogenesis regulatory protein (rrs1) prot
TGME49_243210	1.30	7.22	1.36E-06	1.51E-05	DUF862 domain-containing protein

TGME49_243250	-1.48	7.91	1.42E-06	1.58E-05	myosin H
TGME49_264220	-8.99	5.51	1.43E-06	1.58E-05	hypothetical protein
TGME49_234490	-2.94	6.15	1.44E-06	1.59E-05	kelch repeat-containing protein
TGME49_213400	-2.70	6.41	1.45E-06	1.60E-05	zinc finger (CCCH type) motif-containing protein
TGME49_273370	-3.09	6.35	1.46E-06	1.61E-05	coatomer gamma 2-subunit protein, putative
TGME49_205000	-8.99	5.59	1.47E-06	1.62E-05	phosphoglycerate mutase family protein
TGME49_215430	1.95	7.91	1.50E-06	1.64E-05	hypothetical protein
TGME49_306600	-8.99	5.73	1.52E-06	1.67E-05	RNA recognition motif-containing protein
TGME49_216380	-2.76	6.35	1.55E-06	1.70E-05	phospholipid-translocating P-type ATPase, flippase
TGME49_213600	-8.99	5.17	1.56E-06	1.70E-05	hypothetical protein
TGME49_203350	-9.03	5.69	1.60E-06	1.74E-05	hypothetical protein
TGME49_312622	-2.19	7.05	1.65E-06	1.80E-05	DUF803 domain-containing protein
TGME49_261750	-2.50	6.81	1.68E-06	1.83E-05	rhoptry neck protein RON10
TGME49_307820	1.88	5.87	1.70E-06	1.84E-05	hypothetical protein
TGME49_215460	-1.44	9.58	1.72E-06	1.86E-05	ribosomal protein RPS24
TGME49_221500	-8.98	5.41	1.73E-06	1.87E-05	dual specificity phosphatase, catalytic domain-con
TGME49_319900	-2.01	7.18	1.73E-06	1.87E-05	hypothetical protein
TGME49_206600	-8.95	5.40	1.75E-06	1.89E-05	sigma-70, region 3 protein
TGME49_213635	4.91	5.25	1.77E-06	1.90E-05	hypothetical protein
TGME49_294400	-2.02	7.26	1.77E-06	1.90E-05	hypothetical protein
TGME49_262060	-3.32	5.97	1.79E-06	1.91E-05	hypothetical protein
TGME49_306895	1.23	7.35	1.80E-06	1.92E-05	hypothetical protein
TGME49_245670	-1.30	7.70	1.80E-06	1.92E-05	pyruvate dehydrogenase complex subunit PDH-E1
TGME49_214370	-8.95	5.49	1.80E-06	1.92E-05	hypothetical protein
TGME49_316350	-8.98	5.45	1.85E-06	1.96E-05	hypothetical protein
TGME49_297790	-2.45	6.27	1.85E-06	1.96E-05	hypothetical protein
TGME49_213620	2.19	5.49	1.88E-06	1.99E-05	ABC1 family protein
TGME49_277685	-9.05	5.11	1.89E-06	2.00E-05	hypothetical protein
TGME49_217740	-2.10	6.97	1.91E-06	2.02E-05	3-ketoacyl-(acyl-carrier-protein) reductase
TGME49_288290	2.38	5.66	1.94E-06	2.04E-05	hypothetical protein
TGME49_280800	-1.79	7.39	1.94E-06	2.04E-05	SWI2/SNF2 SRCAP/Ino80
TGME49_306620	2.54	6.62	1.95E-06	2.04E-05	AP2 domain transcription factor AP2IX-9
TGME49_278850	-1.35	7.92	1.95E-06	2.04E-05	DHHC zinc finger domain-containing protein
TGME49_292235	-8.95	5.58	1.97E-06	2.06E-05	hypothetical protein
TGME49_271780	-2.90	6.36	2.01E-06	2.10E-05	Filamin/ABP280 repeat-containing protein
TGME49_309110	1.53	6.61	2.03E-06	2.12E-05	tRNA methyl transferase
TGME49_219485	1.57	6.92	2.13E-06	2.22E-05	hypothetical protein
TGME49_299000	1.46	6.82	2.17E-06	2.25E-05	hypothetical protein
TGME49_267330	-2.42	6.42	2.20E-06	2.28E-05	fumarate hydratase
TGME49_284660	-8.95	5.27	2.21E-06	2.28E-05	mitochondrial ribosomal protein s6-2, putative
TGME49_205340	1.16	9.84	2.26E-06	2.34E-05	ribosomal protein RPS12
TGME49_266470	-8.92	5.57	2.27E-06	2.35E-05	hypothetical protein
TGME49_227450	1.21	7.22	2.31E-06	2.38E-05	hydrolase, NUDIX family protein
TGME49_286650	-8.92	5.60	2.32E-06	2.39E-05	hypothetical protein
TGME49_290720	-1.63	7.52	2.32E-06	2.39E-05	vacuolar proton translocating ATPase subunit, put
TGME49_278950	2.09	5.34	2.34E-06	2.40E-05	LSM domain-containing protein
TGME49_239340	-8.92	5.64	2.34E-06	2.40E-05	hypothetical protein
TGME49_262930	-8.92	5.80	2.35E-06	2.40E-05	hypothetical protein
TGME49_239780	-3.35	6.36	2.38E-06	2.44E-05	hypothetical protein
TGME49_309950	-8.92	5.31	2.38E-06	2.44E-05	NLE (NUC135) domain-containing protein
TGME49_285180	-1.64	7.32	2.40E-06	2.44E-05	hypothetical protein
TGME49_255250	-8.92	5.45	2.49E-06	2.53E-05	tRNA (cytosine(34)-C(5))-methyltransferase, putat
TGME49_250100	2.61	6.89	2.55E-06	2.59E-05	hypothetical protein
TGME49_231120	1.56	6.29	2.55E-06	2.59E-05	ribosomal protein S11, putative

TGME49_235630	1.16	7.24	2.56E-06	2.59E-05	hypothetical protein
TGME49_235740	-4.04	5.69	2.59E-06	2.61E-05	hypothetical protein
TGME49_222192	-8.92	5.28	2.63E-06	2.65E-05	hypothetical protein
TGME49_256880	-8.92	5.54	2.63E-06	2.65E-05	protein kinase domain-containing protein
TGME49_257720	2.71	5.36	2.66E-06	2.67E-05	proton ATPase, putative
TGME49_245580	2.05	6.12	2.78E-06	2.78E-05	hypothetical protein
TGME49_233500	1.39	8.04	2.80E-06	2.80E-05	triose-phosphate isomerase TPI-II
TGME49_214820	1.55	6.75	2.84E-06	2.84E-05	G-patch domain-containing protein
TGME49_288340	-8.89	5.69	2.87E-06	2.86E-05	UBX domain-containing protein
TGME49_254230	2.06	5.65	3.00E-06	2.99E-05	hypothetical protein
TGME49_285970	-8.88	5.58	3.01E-06	3.00E-05	30S ribosomal protein S5, putative
TGME49_222020	-2.64	6.35	3.07E-06	3.05E-05	phosphoglycerate kinase PGKII
TGME49_267340	2.10	5.19	3.10E-06	3.08E-05	hypothetical protein
TGME49_293440	-1.65	7.35	3.12E-06	3.09E-05	hypothetical protein
TGME49_266890	-1.81	6.92	3.17E-06	3.14E-05	hypothetical protein
TGME49_263540	2.06	5.09	3.20E-06	3.17E-05	hypothetical protein
TGME49_217688	-8.88	5.67	3.26E-06	3.22E-05	hypothetical protein
TGME49_293860	-8.88	5.58	3.30E-06	3.25E-05	hypothetical protein
TGME49_229380	-8.88	5.38	3.32E-06	3.27E-05	hypothetical protein
TGME49_265220	-4.07	5.88	3.33E-06	3.27E-05	co-chaperone GrpE protein
TGME49_209860	-8.89	5.15	3.37E-06	3.31E-05	SRP40, C-terminal domain-containing protein
TGME49_204280	1.22	7.67	3.43E-06	3.36E-05	cell-cycle-associated protein kinase DYRK, putative
TGME49_216335	2.20	5.08	3.57E-06	3.49E-05	hypothetical protein
TGME49_316680	1.23	7.77	3.60E-06	3.50E-05	RNA pseudouridine synthase superfamily protein
TGME49_251680	-1.35	8.93	3.59E-06	3.50E-05	histamine-releasing factor, putative
TGME49_306930	-1.41	7.80	3.59E-06	3.50E-05	proteasome subunit beta type 7 precursor, putativ
TGME49_266400	-8.92	5.57	3.58E-06	3.50E-05	hypothetical protein
TGME49_246460	-4.03	5.93	3.65E-06	3.55E-05	hypothetical protein
TGME49_290900	-8.89	5.86	3.67E-06	3.56E-05	hydrolase, NUDIX family protein
TGME49_221180	1.10	8.38	3.67E-06	3.56E-05	hypothetical protein
TGME49_270620	2.33	6.19	3.80E-06	3.68E-05	DEAD/DEAH box helicase domain-containing prote
TGME49_320740	-1.78	7.64	3.82E-06	3.69E-05	hypothetical protein
TGME49_202580	1.81	6.39	3.84E-06	3.71E-05	ATPase, AAA family protein
TGME49_260620	1.67	8.06	3.85E-06	3.71E-05	hypothetical protein
TGME49_226600	-2.24	6.43	4.02E-06	3.86E-05	syntaxin 5, putative
TGME49_304700	-3.99	5.84	4.03E-06	3.87E-05	hypothetical protein
TGME49_310860	-2.59	6.37	4.04E-06	3.87E-05	U5 snRNP-specific protein
TGME49_221590	1.01	8.78	4.12E-06	3.94E-05	dual specificity phosphatase, catalytic domain-con
TGME49_217730	2.08	5.67	4.14E-06	3.95E-05	hypothetical protein
TGME49_268170	-8.95	5.25	4.15E-06	3.96E-05	hypothetical protein
TGME49_213900	1.20	7.70	4.17E-06	3.97E-05	regulator of chromosome condensation RCC1
TGME49_230580	-8.85	5.21	4.21E-06	4.00E-05	hypothetical protein
TGME49_304710	-1.30	7.77	4.25E-06	4.03E-05	eukaryotic peptide chain release factor, putative
TGME49_253000	1.29	6.99	4.26E-06	4.04E-05	ELMO/CED-12 family protein
TGME49_223450	-1.94	6.86	4.29E-06	4.06E-05	ubiquitin carboxyl-terminal hydrolase
TGME49_291980	-1.59	7.41	4.32E-06	4.08E-05	HECT-domain (ubiquitin-transferase) domain-cont
TGME49_212960	-8.89	5.30	4.31E-06	4.08E-05	hypothetical protein
TGME49_253100	1.20	7.16	4.38E-06	4.13E-05	hypothetical protein
TGME49_264730	-8.85	5.36	4.40E-06	4.15E-05	hypothetical protein
TGME49_244530	1.01	8.53	4.47E-06	4.20E-05	hypothetical protein
TGME49_231980	-8.85	5.84	4.49E-06	4.22E-05	hypothetical protein
TGME49_215260	-1.63	7.22	4.51E-06	4.23E-05	carbamoylphosphate synthetase
TGME49_253640	1.34	6.73	4.62E-06	4.33E-05	hypothetical protein
TGME49_268880	-3.23	6.09	4.68E-06	4.38E-05	hypothetical protein

TGME49_292375	3.37	5.88	4.71E-06	4.40E-05	KRUF family protein
TGME49_311870	-2.39	6.63	4.75E-06	4.43E-05	WD domain, G-beta repeat-containing protein
TGME49_253360	1.27	7.00	4.78E-06	4.46E-05	hypothetical protein
TGME49_289150	1.52	6.22	4.79E-06	4.46E-05	hypothetical protein
TGME49_267600	-2.83	6.33	4.83E-06	4.49E-05	FHA domain-containing protein
TGME49_207665	-8.89	4.98	4.87E-06	4.52E-05	kinesin motor domain-containing protein
TGME49_308880	-8.85	5.26	4.96E-06	4.61E-05	ImpB/MucB/SamB family protein
TGME49_263500	-4.01	5.93	5.05E-06	4.68E-05	vacuolar protein sorting-associated protein 26, pu
TGME49_219770	-8.91	5.44	5.07E-06	4.69E-05	30S ribosomal protein S12, putative
TGME49_310100	-8.85	5.39	5.10E-06	4.72E-05	mannosyltransferase (pig-m) protein
TGME49_211040	-1.31	8.12	5.27E-06	4.86E-05	Sec61beta family protein
TGME49_214830	-3.98	5.63	5.29E-06	4.87E-05	hypothetical protein
TGME49_285210	-8.90	5.26	5.29E-06	4.87E-05	hypothetical protein
TGME49_313330	-8.99	5.76	5.35E-06	4.91E-05	rhoptry kinase family protein ROP27
TGME49_271760	-8.81	5.72	5.45E-06	4.99E-05	seryl-tRNA synthetase (SerRS2)
TGME49_309870	-8.81	5.78	5.52E-06	5.05E-05	hypothetical protein
TGME49_207480	-2.36	6.41	5.58E-06	5.10E-05	GCC2 and GCC3 domain-containing protein
TGME49_294290	-2.03	7.07	5.63E-06	5.14E-05	Der1ER1
TGME49_313760	1.01	8.20	5.66E-06	5.16E-05	hypothetical protein
TGME49_264690	-8.81	5.40	5.76E-06	5.24E-05	cyclin 4, putative
TGME49_272390	-8.82	5.13	5.77E-06	5.24E-05	hypothetical protein
TGME49_230190	-1.99	6.88	5.92E-06	5.38E-05	hypothetical protein
TGME49_209700	-3.96	5.90	5.94E-06	5.39E-05	hypothetical protein
TGME49_242800	-1.99	6.66	6.07E-06	5.50E-05	ribosome biogenesis protein NSA2, putative
TGME49_252380	1.97	6.44	6.12E-06	5.54E-05	hypothetical protein
TGME49_249610	-2.36	6.80	6.12E-06	5.54E-05	hypothetical protein
TGME49_257480	-2.96	6.03	6.26E-06	5.65E-05	NADP-dependent succinate-semialdehyde dehydr
TGME49_259950	2.29	7.95	6.29E-06	5.67E-05	carbonate dehydratase, eukaryotic-type domain-c
TGME49_250950	2.30	5.35	6.33E-06	5.70E-05	KRUF family protein
TGME49_305090	1.60	6.45	6.37E-06	5.73E-05	kinase binding protein cgi-121 protein
TGME49_264780	-8.81	5.52	6.41E-06	5.75E-05	UTP-glucose-1-phosphate uridylyltransferase subf
TGME49_231030	-3.96	5.64	6.52E-06	5.84E-05	hypothetical protein
TGME49_221600	-8.81	5.26	6.53E-06	5.85E-05	hypothetical protein
TGME49_221200	1.50	7.36	6.60E-06	5.90E-05	CW-type Zinc Finger protein
TGME49_310130	-8.78	6.05	6.63E-06	5.91E-05	Spc97 / Spc98 family protein
TGME49_271200	-1.77	6.92	6.66E-06	5.93E-05	AP2 domain transcription factor AP2VIII-5
TGME49_217770	1.40	7.05	6.69E-06	5.96E-05	hypothetical protein
TGME49_202120	-1.56	7.31	6.76E-06	6.00E-05	hypothetical protein
TGME49_289100	-2.15	6.51	6.90E-06	6.12E-05	hypothetical protein
TGME49_261400	-1.51	7.86	7.15E-06	6.34E-05	hypothetical protein
TGME49_320550	-2.12	6.64	7.19E-06	6.36E-05	hypothetical protein
TGME49_231215	-8.78	5.41	7.19E-06	6.36E-05	hypothetical protein
TGME49_205380	-1.48	7.80	7.24E-06	6.39E-05	fructose-bisphospatase I
TGME49_220100	-2.45	6.73	7.23E-06	6.39E-05	phosphoribosylpyrophosphate synthetase
TGME49_250690	-1.08	8.88	7.33E-06	6.46E-05	zinc finger (CCCH type) motif-containing protein
TGME49_318330	-8.77	5.85	7.34E-06	6.46E-05	histone lysine acetyltransferase MYST-A
TGME49_227930	1.20	7.02	7.47E-06	6.56E-05	hypothetical protein
TGME49_321440	-1.30	7.84	7.54E-06	6.60E-05	SWI2/SNF2 ISWI-like SANT
TGME49_202950	-1.45	7.30	7.55E-06	6.60E-05	hypothetical protein
TGME49_300290	-8.80	5.41	7.65E-06	6.67E-05	SNARE domain-containing protein
TGME49_249030	1.25	7.46	7.66E-06	6.67E-05	endonuclease/exonuclease/phosphatase family pr
TGME49_280380	1.22	9.89	7.82E-06	6.80E-05	poly(ADP-ribose) glycohydrolase
TGME49_311460	-2.88	6.70	7.84E-06	6.81E-05	hypothetical protein
TGME49_261620	1.56	5.83	7.91E-06	6.87E-05	hypothetical protein

TGME49_219070	1.18	7.52	8.17E-06	7.07E-05	cyclic nucleotide-binding domain-containing prote
TGME49_310010	-1.27	8.26	8.17E-06	7.07E-05	rhoptry neck protein RON1
TGME49_237830	-8.77	5.84	8.17E-06	7.07E-05	DNA polymerase I domain-containing protein
TGME49_251800	1.68	5.67	8.21E-06	7.09E-05	hypothetical protein
TGME49_213790	-2.78	6.32	8.24E-06	7.11E-05	hypothetical protein
TGME49_219310	-1.41	8.62	8.35E-06	7.19E-05	DnaK family protein
TGME49_240300	-8.80	5.18	8.38E-06	7.21E-05	zinc finger domain, LSD1 subclass domain-contain
TGME49_298830	1.64	6.26	8.58E-06	7.38E-05	hypothetical protein
TGME49_236570	-1.26	7.93	8.59E-06	7.38E-05	lysine decarboxylase family protein
TGME49_289570	1.04	7.68	8.74E-06	7.50E-05	phosphatidylinositol transfer protein
TGME49_266970	-3.21	5.89	8.80E-06	7.53E-05	hypothetical protein
TGME49_231130	-8.74	5.70	8.82E-06	7.54E-05	hypothetical protein
TGME49_252340	1.57	7.56	8.93E-06	7.62E-05	hypothetical protein
TGME49_240540	-2.36	6.41	8.95E-06	7.63E-05	hypothetical protein
TGME49_209820	-8.77	5.29	9.04E-06	7.69E-05	syntaxin protein
TGME49_318410	-1.38	7.53	9.12E-06	7.75E-05	TCP-1 chaperonin, putative
TGME49_246800	-1.79	6.81	9.20E-06	7.81E-05	acylaminoacyl-peptidase, putative
TGME49_319640	1.06	8.46	9.25E-06	7.84E-05	hypothetical protein
TGME49_263040	-1.10	9.58	9.30E-06	7.87E-05	ribosomal protein RPS16
TGME49_297420	-3.92	5.82	9.32E-06	7.88E-05	beta-tubulin cofactor D, putative
TGME49_264840	-8.74	5.33	9.59E-06	8.09E-05	ATP-dependent DNA helicase, RecQ family protein
TGME49_237000	-3.90	5.99	9.62E-06	8.11E-05	polyphosphoinositide binding protein, putative
TGME49_237530	-8.74	5.45	9.68E-06	8.15E-05	hypothetical protein
TGME49_280410	-1.95	6.69	9.72E-06	8.18E-05	3'5'-cyclic nucleotide phosphodiesterase domain-c
TGME49_290940	-1.57	7.21	9.87E-06	8.29E-05	EMP/nonaspanin domain family protein
TGME49_250115	1.14	7.97	9.96E-06	8.34E-05	hypothetical protein
TGME49_286750	-1.46	7.53	9.98E-06	8.34E-05	MA3 domain-containing protein
TGME49_270800	-3.97	6.03	9.98E-06	8.34E-05	GAF domain-containing protein
TGME49_223690	-8.74	5.06	9.94E-06	8.34E-05	hypothetical protein
TGME49_253320	-8.74	4.96	9.99E-06	8.34E-05	hypothetical protein
TGME49_217020	-3.37	6.25	1.02E-05	8.48E-05	ATPase, AFG1 family protein
TGME49_306470	-2.49	6.39	1.02E-05	8.50E-05	isoprenylcysteine carboxyl methyltransferase (icm
TGME49_314280	2.69	5.65	1.04E-05	8.61E-05	AAR2 protein
TGME49_225690	-1.29	8.01	1.04E-05	8.61E-05	hypothetical protein
TGME49_292950	-1.21	7.64	1.05E-05	8.67E-05	hypothetical protein
TGME49_236550	-1.37	7.92	1.05E-05	8.67E-05	hypothetical protein
TGME49_226260	1.11	7.26	1.08E-05	8.92E-05	hypothetical protein
TGME49_224600	-3.90	5.60	1.09E-05	9.03E-05	GTP binding protein
TGME49_216410	-8.82	5.36	1.09E-05	9.04E-05	hypothetical protein
TGME49_224310	-8.76	5.58	1.10E-05	9.04E-05	DHHC zinc finger domain-containing protein
TGME49_275755	2.14	4.98	1.10E-05	9.08E-05	hypothetical protein
TGME49_312330	-1.16	7.74	1.11E-05	9.10E-05	hypothetical protein
TGME49_219820	-1.23	7.72	1.12E-05	9.19E-05	polyubiquitin UbC, putative
TGME49_231180	1.62	6.18	1.14E-05	9.36E-05	hypothetical protein
TGME49_261970	1.26	7.59	1.15E-05	9.41E-05	hypothetical protein
TGME49_212140	-4.01	6.11	1.15E-05	9.41E-05	hypothetical protein
TGME49_314730	-3.90	5.86	1.15E-05	9.42E-05	ALG6, ALG8 glycosyltransferase family protein
TGME49_297810	-1.39	7.88	1.16E-05	9.45E-05	hypothetical protein
TGME49_238950	-1.54	9.10	1.17E-05	9.54E-05	fatty acyl-CoA desaturase, putative
TGME49_294980	2.12	6.35	1.18E-05	9.63E-05	hypothetical protein
TGME49_292975	-3.16	6.05	1.19E-05	9.66E-05	hypothetical protein
TGME49_312860	-3.18	5.92	1.20E-05	9.81E-05	hypothetical protein
TGME49_268600	-3.13	5.84	1.21E-05	9.83E-05	DNA polymerase epsilon subunit B protein
TGME49_264860	-3.88	5.69	1.21E-05	9.84E-05	zinc finger, C3HC4 type (RING finger) domain-cont

TGME49_270900	-3.25	6.04	1.23E-05	9.98E-05	ATPase, AAA family protein
TGME49_269670	-3.14	5.91	1.23E-05	1.00E-04	hypothetical protein
TGME49_219800	-1.19	8.18	1.23E-05	0.000100012	vacuolar ATP synthase subunit b, putative
TGME49_235490	-1.71	7.03	1.25E-05	0.000100719	hypothetical protein
TGME49_312650	2.16	6.55	1.25E-05	0.00010075	hypothetical protein
TGME49_244720	-3.87	5.51	1.25E-05	0.00010075	hypothetical protein
TGME49_233410	1.71	5.64	1.25E-05	0.00010096	Sof1 family domain-containing protein
TGME49_208200	-3.90	5.78	1.27E-05	0.000102014	PHD-finger domain-containing protein
TGME49_205558	-1.08	8.48	1.29E-05	0.000103284	NAC domain-containing protein
TGME49_256770	-1.33	7.59	1.28E-05	0.000103284	eukaryotic translation initiation factor 4A, isoform
TGME49_225160	1.17	7.43	1.29E-05	0.000103493	hypothetical protein
TGME49_216120	-2.30	6.42	1.29E-05	0.000103645	hypothetical protein
TGME49_272270	-8.70	4.79	1.29E-05	0.000103645	radical SAM domain-containing protein
TGME49_264140	-1.79	7.26	1.30E-05	0.000104062	hypothetical protein
TGME49_207770	-1.45	7.77	1.34E-05	0.000107114	PCI domain-containing protein
TGME49_202280	-3.93	5.77	1.35E-05	0.000107471	WD domain, G-beta repeat-containing protein
TGME49_310850	-8.75	5.31	1.37E-05	0.000109333	MYND finger domain-containing protein
TGME49_223780	-1.90	6.75	1.38E-05	0.000109799	hypothetical protein
TGME49_275350	-2.29	6.21	1.38E-05	0.000109805	TBC domain-containing protein
TGME49_270580	-8.69	5.66	1.39E-05	0.000109868	HECT-domain (ubiquitin-transferase) domain-cont
TGME49_253850	2.01	5.06	1.40E-05	0.000110606	hypothetical protein
TGME49_252310	-2.02	6.41	1.40E-05	0.000110736	hypothetical protein
TGME49_289310	-2.17	6.67	1.42E-05	0.000112488	cullin family protein
TGME49_222060	-2.57	5.98	1.43E-05	0.000113006	hypothetical protein
TGME49_310230	-8.70	5.63	1.44E-05	0.000113172	hypothetical protein
TGME49_306530	-8.70	5.01	1.46E-05	0.000114861	hypothetical protein
TGME49_276930	1.21	7.83	1.46E-05	0.000114975	hypothetical protein
TGME49_259520	-8.69	5.47	1.47E-05	0.0001151	hypothetical protein
TGME49_210390	-8.74	5.21	1.46E-05	0.0001151	WD domain, G-beta repeat-containing protein
TGME49_246230	-8.73	5.20	1.47E-05	0.000115201	hypothetical protein
TGME49_307010	2.11	6.89	1.48E-05	0.000116312	histone lysine demethylase JMJC1/KDM5D/JARID1
TGME49_258670	-8.78	5.62	1.51E-05	0.000117933	hypothetical protein
TGME49_294830	-2.83	6.45	1.51E-05	0.00011794	methyltransferase domain-containing protein
TGME49_315600	-8.73	5.50	1.54E-05	0.000119998	MCM2/3/5 family protein
TGME49_315860	-2.13	6.65	1.60E-05	0.000124734	EF hand domain-containing protein
TGME49_260320	-2.72	6.27	1.61E-05	0.000124902	Noc2p family protein
TGME49_214770	-1.22	7.90	1.68E-05	0.000130724	small GTP binding protein rab1a, putative
TGME49_294705	-8.71	5.03	1.68E-05	0.000130724	hypothetical protein
TGME49_211630	1.02	8.47	1.69E-05	0.000131182	hypothetical protein
TGME49_255890	-3.11	5.76	1.69E-05	0.000131182	pyridine nucleotide-disulfide oxidoreductase dom
TGME49_292140	1.08	7.59	1.70E-05	0.000131776	NIMA-related protein kinase NIMA1
TGME49_202620	1.02	8.38	1.71E-05	0.000131776	hypothetical protein
TGME49_290580	-3.84	6.04	1.71E-05	0.000132033	ATP-binding cassette G family transporter ABCG8
TGME49_223150	-8.66	5.15	1.72E-05	0.000132172	START domain-containing protein
TGME49_319312	2.20	5.71	1.73E-05	0.000132784	hypothetical protein
TGME49_243580	-2.65	7.20	1.73E-05	0.000132847	Hit family protein involved in cell-cycle regulation,
TGME49_201900	-3.84	5.71	1.74E-05	0.000133059	hypothetical protein
TGME49_243960	-1.04	8.61	1.77E-05	0.00013561	nuclear transport factor 2 (ntf2) domain-containin
TGME49_255700	-1.94	7.07	1.85E-05	0.000141114	hypothetical protein
TGME49_207180	-8.69	5.29	1.87E-05	0.000142833	indole-3-glycerol phosphate synthase domain-con
TGME49_292330	1.20	7.38	1.89E-05	0.000143721	hypothetical protein
TGME49_208970	-1.83	6.92	1.89E-05	0.000143777	RNA recognition motif-containing protein
TGME49_264090	-3.12	6.14	1.89E-05	0.000143777	hypothetical protein
TGME49_224020	1.43	6.33	1.90E-05	0.000144672	hypothetical protein

TGME49_293380	-8.69	5.33	1.91E-05	0.000144672	histone lysine acetyltransferase HAT1
TGME49_216390	-3.08	5.89	1.92E-05	0.000145243	RNA methyltransferase, TrmH family protein
TGME49_249410	-2.51	6.04	1.92E-05	0.00014565	hypothetical protein
TGME49_258230	-3.05	5.91	1.93E-05	0.00014565	rhoptry kinase family protein ROP20
TGME49_229370	-3.81	5.92	1.93E-05	0.00014565	AP2 domain transcription factor AP2VIII-1
TGME49_278440	-1.90	6.87	1.94E-05	0.000146332	SWI2/SNF2 Brahma-like putative
TGME49_278930	-2.03	6.40	1.94E-05	0.000146332	Tubulin-tyrosine ligase family protein
TGME49_313270	-1.48	7.49	1.94E-05	0.000146449	hypothetical protein
TGME49_203358	-3.84	5.46	1.97E-05	0.000147909	hypothetical protein
TGME49_252190	2.27	6.80	1.98E-05	0.000148732	KRUF family protein
TGME49_291140	-1.15	8.46	1.98E-05	0.00014894	CCR4-Not complex component, Not1 protein
TGME49_251885	-8.67	5.07	1.99E-05	0.000149224	hypothetical protein
TGME49_296015	1.63	6.18	2.00E-05	0.00014945	hypothetical protein
TGME49_310910	-8.67	5.61	2.00E-05	0.00014945	WD domain, G-beta repeat-containing protein
TGME49_249790	-3.81	5.87	2.02E-05	0.000150697	hypothetical protein
TGME49_269150	-8.65	5.14	2.02E-05	0.000151215	DHHC zinc finger domain-containing protein
TGME49_300250	1.02	7.59	2.08E-05	0.000154394	MtN3/saliva family protein
TGME49_216630	-8.62	5.35	2.08E-05	0.000154462	trigger factor protein, putative
TGME49_207800	-8.62	5.28	2.09E-05	0.00015503	hypothetical protein
TGME49_218240	-1.16	8.12	2.09E-05	0.000155188	hypothetical protein
TGME49_227320	-8.71	5.44	2.10E-05	0.00015546	hypothetical protein
TGME49_222290	-3.81	5.85	2.13E-05	0.000157795	LSM domain-containing protein
TGME49_225060	-2.43	6.46	2.16E-05	0.000159519	nucleoredoxin family protein
TGME49_221295	-3.81	5.92	2.16E-05	0.000159519	hypothetical protein
TGME49_285950	-8.68	5.02	2.17E-05	0.000159663	hypothetical protein
TGME49_245590	2.04	5.36	2.17E-05	0.000159691	rhomboid protease ROM6
TGME49_228130	1.71	6.56	2.18E-05	0.000160344	hypothetical protein
TGME49_251850	-1.68	7.02	2.20E-05	0.000161559	serine/threonine protein phosphatase
TGME49_215660	-8.62	5.03	2.20E-05	0.000161559	hypothetical protein
TGME49_216170	-3.96	5.73	2.22E-05	0.000162772	SufS subfamily cysteine desulfurase
TGME49_229490	-2.69	6.21	2.24E-05	0.000164289	tetratricopeptide repeat-containing protein
TGME49_268900	-8.61	5.16	2.28E-05	0.00016648	dense granular protein GRA10
TGME49_308060	-2.51	6.56	2.34E-05	0.000170857	hypothetical protein
TGME49_305930	2.60	4.79	2.35E-05	0.000171253	hypothetical protein
TGME49_293500	2.16	5.56	2.35E-05	0.000171458	hypothetical protein
TGME49_310270	-3.88	5.89	2.36E-05	0.00017172	hypothetical protein
TGME49_277910	1.20	6.76	2.40E-05	0.000174709	thrombospondin type 1 domain-containing protei
TGME49_289670	2.63	4.94	2.43E-05	0.000176644	DNA repair metallo-beta-lactamase
TGME49_229500	-3.78	5.70	2.49E-05	0.000180675	hypothetical protein
TGME49_212840	-8.67	5.13	2.55E-05	0.000184771	HIT zinc finger protein
TGME49_294812	-3.81	5.19	2.57E-05	0.000185762	RNA recognition motif-containing protein
TGME49_227010	-8.72	5.24	2.58E-05	0.00018657	rhoptry kinase family protein ROP30
TGME49_310670	-1.23	7.93	2.58E-05	0.000186599	glycogen phosphorylase 1, putative
TGME49_231480	-1.54	7.27	2.59E-05	0.000186611	GCN1, putative
TGME49_230950	-8.61	5.62	2.61E-05	0.000187953	hypothetical protein
TGME49_243298	-8.61	5.50	2.61E-05	0.000187953	ICE family protease (caspase) p20 domain-contain
TGME49_234190	-1.19	8.32	2.65E-05	0.000190321	serine hydroxymethyltransferase 2, putative
TGME49_203060	1.16	7.36	2.66E-05	0.000191007	hypothetical protein
TGME49_295420	1.41	7.87	2.68E-05	0.00019235	hypothetical protein
TGME49_233340	-2.45	6.12	2.68E-05	0.00019235	hypothetical protein
TGME49_210980	1.49	6.94	2.72E-05	0.000194768	alternative splicing type 3 and, putative
TGME49_270050	1.87	5.58	2.73E-05	0.000195219	hypothetical protein
TGME49_226900	1.66	6.03	2.74E-05	0.000195277	hypothetical protein
TGME49_293730	-1.72	7.07	2.74E-05	0.000195277	DHHC zinc finger domain-containing protein

TGME49_270090	-3.06	6.03	2.74E-05	0.000195277	hypothetical protein
TGME49_318575	-8.61	5.22	2.74E-05	0.000195277	hypothetical protein
TGME49_222940	3.27	5.07	2.77E-05	0.000196882	hypothetical protein
TGME49_316900	-1.89	6.55	2.77E-05	0.000196909	Sas10 C-terminal domain-containing protein
TGME49_239720	-8.57	5.49	2.78E-05	0.000197474	50S ribosomal protein l24, putative
TGME49_299900	-8.57	5.26	2.79E-05	0.000197679	hypothetical protein
TGME49_204380	-1.66	6.95	2.80E-05	0.000198042	hypothetical protein
TGME49_263270	-1.32	7.15	2.85E-05	0.000200948	glycerophosphodiester phosphodiesterase family
TGME49_290890	-2.41	6.43	2.84E-05	0.000200948	carbonyl reductase 1, putative
TGME49_310570	-8.66	5.49	2.86E-05	0.000201889	hypothetical protein
TGME49_217870	1.24	6.94	2.87E-05	0.000202167	DHHC zinc finger domain-containing protein
TGME49_306910	-2.21	6.36	2.87E-05	0.000202167	hypothetical protein
TGME49_290740	-8.60	4.96	2.87E-05	0.000202167	hypothetical protein
TGME49_309100	2.42	5.47	2.93E-05	0.000205546	hypothetical protein
TGME49_223640	-8.67	5.20	2.97E-05	0.000208381	hypothetical protein
TGME49_310300	-3.06	5.65	3.00E-05	0.00021024	hypothetical protein
TGME49_291030	-3.82	5.60	3.04E-05	0.000212625	zinc finger, C3HC4 type (RING finger) domain-cont
TGME49_244510	-8.57	4.91	3.04E-05	0.000212859	AP2 domain transcription factor AP2VI-3
TGME49_310590	1.88	5.32	3.07E-05	0.000214098	hypothetical protein
TGME49_262980	-8.57	5.23	3.12E-05	0.000217676	hypothetical protein
TGME49_249950	-3.06	5.46	3.15E-05	0.000219437	Mak16 protein
TGME49_219250	1.08	7.49	3.17E-05	0.000220177	acetyltransferase, GNAT family protein
TGME49_275310	-3.06	5.96	3.18E-05	0.000220694	hypothetical protein
TGME49_285780	-8.61	5.31	3.18E-05	0.000220708	hypothetical protein
TGME49_268430	-3.80	5.49	3.19E-05	0.0002214	hypothetical protein
TGME49_300020	-3.75	5.75	3.22E-05	0.000222696	ATP-dependent metallopeptidase HflB subfamily p
TGME49_313350	-3.75	5.75	3.22E-05	0.000222696	hypothetical protein
TGME49_229290	-8.59	4.73	3.24E-05	0.000224035	kelch repeat-containing protein
TGME49_258480	-1.88	6.81	3.27E-05	0.000225612	hypothetical protein
TGME49_310710	-3.75	5.64	3.34E-05	0.000229945	small ribosomal subunit Rsm22 protein
TGME49_230000	1.49	6.52	3.34E-05	0.00022996	hypothetical protein
TGME49_295015	1.87	5.56	3.35E-05	0.000230524	patched family protein
TGME49_272640	-2.08	6.34	3.36E-05	0.000230713	eukaryotic initiation factor-2B, epsilon subunit, pu
TGME49_208820	-3.81	5.82	3.36E-05	0.000230733	1-deoxy-D-xylulose-5-phosphate synthase
TGME49_222920	-2.21	6.07	3.38E-05	0.000231666	mbp-1 interacting protein-2a family protein
TGME49_232160	1.18	6.85	3.38E-05	0.000231731	zinc finger, C3HC4 type (RING finger) domain-cont
TGME49_245485	1.89	6.56	3.39E-05	0.000232185	microneme protein MIC9
TGME49_243490	-8.57	5.07	3.39E-05	0.000232185	BCS1 family isoform 9, putative
TGME49_249560	-8.58	5.35	3.43E-05	0.000234728	DNA-directed RNA polymerase alpha chain rpoA
TGME49_289290	-1.79	7.07	3.44E-05	0.000234926	hypothetical protein
TGME49_244000	-8.57	5.35	3.45E-05	0.000235471	DEAD/DEAH box helicase domain-containing prote
TGME49_285990	-3.82	5.64	3.47E-05	0.000236443	Filamin/ABP280 repeat-containing protein
TGME49_260580	-2.07	6.60	3.53E-05	0.000240346	hypothetical protein
TGME49_296340	3.34	5.35	3.54E-05	0.000241088	hypothetical protein
TGME49_256030	-1.17	7.94	3.58E-05	0.000243376	hypothetical protein
TGME49_263410	6.14	5.00	3.62E-05	0.000245753	scavenger receptor cysteine-rich domain-containi
TGME49_249698	-8.60	4.98	3.62E-05	0.000245873	hypothetical protein
TGME49_255740	-3.07	5.77	3.64E-05	0.000246494	hypothetical protein
TGME49_235960	-8.56	4.72	3.64E-05	0.000246494	hypothetical protein
TGME49_248570	-3.80	5.72	3.65E-05	0.00024679	hypothetical protein
TGME49_268220	9.62	5.12	3.67E-05	0.000247768	hypothetical protein
TGME49_221440	-8.58	5.45	3.68E-05	0.000248083	RPGR, putative
TGME49_259850	-8.53	5.08	3.71E-05	0.000250141	hypothetical protein
TGME49_240370	6.91	4.77	3.75E-05	0.00025251	Toxoplasma gondii family E protein

TGME49_248150	-3.75	5.73	3.76E-05	0.000252712	hypothetical protein
TGME49_243340	-8.53	5.12	3.76E-05	0.000252712	atypical MEK-related kinase (incomplete catalytic
TGME49_209130	-8.53	5.70	3.77E-05	0.00025311	regulator of chromosome condensation (RCC1) re
TGME49_239830	-1.38	8.16	3.79E-05	0.000254401	TBC domain-containing protein
TGME49_221585	-8.57	5.10	3.82E-05	0.000255639	hypothetical protein
TGME49_242870	-8.53	5.35	3.82E-05	0.000255919	histone lysine methyltransferase, SET, putative
TGME49_227115	-8.53	5.08	3.83E-05	0.000256078	hypothetical protein
TGME49_304900	-1.22	7.73	3.88E-05	0.000258867	hypothetical protein
TGME49_269130	-8.53	4.83	3.93E-05	0.000262362	hypothetical protein
TGME49_228490	-2.63	6.14	3.94E-05	0.000262479	hypothetical protein
TGME49_261650	-1.52	7.04	3.94E-05	0.000262778	hypothetical protein
TGME49_304470	-3.00	5.83	3.95E-05	0.000263008	oxidoreductase, putative
TGME49_283790	-1.57	7.20	3.97E-05	0.000263656	protein kinase, putative
TGME49_280720	-8.53	5.01	3.99E-05	0.000265021	hypothetical protein
TGME49_212860	1.22	6.72	4.03E-05	0.000267184	hypothetical protein
TGME49_240460	-3.00	5.64	4.03E-05	0.000267184	AP2 domain transcription factor AP2VI-1
TGME49_279430	-2.66	6.25	4.16E-05	0.000274951	cwf18 pre-mRNA splicing factor protein
TGME49_281510	-3.01	6.07	4.16E-05	0.000274951	ribonuclease H1 large subunit, putative
TGME49_213460	2.47	7.06	4.21E-05	0.000277641	hypothetical protein
TGME49_239560	-8.60	5.38	4.23E-05	0.000278662	myosin E
TGME49_285260	2.21	4.95	4.24E-05	0.000278976	hypothetical protein
TGME49_313360	1.44	6.20	4.32E-05	0.000284277	hypothetical protein
TGME49_247300	1.01	8.35	4.36E-05	0.000286054	hypothetical protein
TGME49_256840	-3.72	5.96	4.36E-05	0.000286054	hypothetical protein
TGME49_259530	-3.72	5.96	4.36E-05	0.000286054	GalNac
TGME49_311500	-8.58	5.49	4.37E-05	0.000286758	ThiF family protein
TGME49_251840	1.29	6.35	4.39E-05	0.000287671	hypothetical protein
TGME49_250220	2.17	5.45	4.42E-05	0.000288498	hypothetical protein
TGME49_216130	-1.94	6.73	4.41E-05	0.000288498	ubiquitin conjugating enzyme E2, putative
TGME49_278130	-2.14	6.84	4.41E-05	0.000288498	hypothetical protein
TGME49_293460	-3.75	5.22	4.42E-05	0.000288514	ATP-dependent DNA ligase domain-containing pro
TGME49_308990	-3.72	5.30	4.44E-05	0.000289666	transporter, solute:sodium symporter (SSS) family
TGME49_248790	2.18	5.02	4.52E-05	0.00029452	hypothetical protein
TGME49_227140	2.65	4.81	4.54E-05	0.000295421	hypothetical protein
TGME49_250830	-1.53	7.32	4.54E-05	0.000295421	SAC3/GANP family protein
TGME49_305560	-3.72	5.81	4.63E-05	0.000300682	Vps51/Vps67 protein
TGME49_300048	1.07	7.17	4.64E-05	0.000301136	hypothetical protein
TGME49_263290	2.97	6.06	4.72E-05	0.000305325	rhomboid protease ROM2
TGME49_286560	-8.57	5.40	4.78E-05	0.000308454	U6 snRNA-associated Sm family protein
TGME49_276900	-3.75	5.41	4.82E-05	0.000310935	hypothetical protein
TGME49_286800	-8.52	5.19	4.83E-05	0.00031096	hypothetical protein
TGME49_274130	-8.48	5.27	4.87E-05	0.000313035	TBC domain-containing protein
TGME49_206695	1.84	5.29	4.88E-05	0.000313552	hypothetical protein
TGME49_227350	-3.09	5.75	4.94E-05	0.000316756	hypothetical protein
TGME49_209270	-8.57	5.06	4.98E-05	0.000319508	hypothetical protein
TGME49_221710	-2.60	5.92	5.01E-05	0.000321026	TBC domain-containing protein
TGME49_202750	-8.48	5.03	5.04E-05	0.000322949	3' exoribonuclease family, domain 1 domain-conta
TGME49_320440	-2.35	6.19	5.07E-05	0.000324308	hypothetical protein
TGME49_262550	-8.48	4.87	5.08E-05	0.000324455	hypothetical protein
TGME49_268960	-2.98	6.09	5.10E-05	0.000325133	5'-AMP-activated protein kinase subunit beta-1 fa
TGME49_218358	-8.52	4.99	5.10E-05	0.000325133	zinc knuckle domain-containing protein
TGME49_262650	-3.77	5.56	5.11E-05	0.000325832	WD domain, G-beta repeat-containing protein
TGME49_202010	-2.51	6.47	5.12E-05	0.000326292	hypothetical protein
TGME49_319308	-2.63	6.63	5.13E-05	0.000326522	hypothetical protein

TGME49_273920	-2.61	6.00	5.15E-05	0.00032768	aldose reductase, putative
TGME49_249740	1.50	6.54	5.21E-05	0.00033084	translation machinery associated tma7 protein
TGME49_309130	-8.48	5.04	5.21E-05	0.000330892	hypothetical protein
TGME49_297900	1.40	7.58	5.23E-05	0.000331261	hypothetical protein
TGME49_312960	1.26	6.29	5.23E-05	0.000331261	hypothetical protein
TGME49_278240	-8.75	4.82	5.30E-05	0.000335237	Zn-finger in Ran binding protein and others domai
TGME49_295060	-8.49	4.82	5.32E-05	0.000336309	exonuclease
TGME49_248870	1.36	6.40	5.33E-05	0.000336464	SNARE associated Golgi protein
TGME49_266390	-8.48	4.86	5.36E-05	0.000338104	DNA mismatch repair protein, C-terminal domain-
TGME49_248140	3.21	5.08	5.38E-05	0.000339395	hypothetical protein
TGME49_270230	-8.48	5.30	5.47E-05	0.000344822	hypothetical protein
TGME49_248160	-8.48	5.47	5.50E-05	0.00034589	hypothetical protein
TGME49_219660	3.07	5.14	5.54E-05	0.000348307	hypothetical protein
TGME49_279540	-8.54	4.99	5.61E-05	0.00035209	hypothetical protein
TGME49_221280	-1.56	6.77	5.70E-05	0.000357319	hypothetical protein
TGME49_318260	-3.69	5.56	5.71E-05	0.00035808	transcription initiation factor TFIID subunit TAF5
TGME49_247700	1.31	8.30	5.82E-05	0.00036455	AP2 domain transcription factor AP2XII-4
TGME49_253410	2.32	6.01	5.86E-05	0.000366857	hypothetical protein
TGME49_250680	-2.58	5.77	5.89E-05	0.00036808	TBC domain-containing kinase (incomplete catalyt
TGME49_254390	1.06	7.35	5.94E-05	0.000370653	CRAL/TRIO domain-containing protein
TGME49_220175	2.95	4.77	6.09E-05	0.000378775	hypothetical protein
TGME49_227910	-1.23	7.80	6.11E-05	0.000379848	hypothetical protein
TGME49_203760	1.36	6.32	6.18E-05	0.000383467	hypothetical protein
TGME49_297245	-1.46	7.00	6.26E-05	0.000387577	transporter, major facilitator family protein
TGME49_271625	-8.49	4.93	6.28E-05	0.000388329	serine--tRNA ligase
TGME49_205300	1.32	6.67	6.31E-05	0.000390271	hypothetical protein
TGME49_226030	-1.07	8.61	6.33E-05	0.000391223	AGC kinase
TGME49_309890	-3.00	5.90	6.34E-05	0.000391465	hypothetical protein
TGME49_260510	-2.95	5.73	6.37E-05	0.000393006	ubiquitin thioesterase otubain-like family protein
TGME49_300285	-8.57	5.23	6.38E-05	0.000393238	hypothetical protein
TGME49_229930	1.35	6.97	6.39E-05	0.00039366	p25-alpha family protein
TGME49_248990	1.18	8.43	6.43E-05	0.000395267	hypothetical protein
TGME49_209240	1.56	5.74	6.46E-05	0.00039696	RNA methyltransferase
TGME49_289890	-3.06	5.61	6.51E-05	0.000399319	hypothetical protein
TGME49_235478	-3.79	5.93	6.54E-05	0.000400825	pantothenate kinase
TGME49_277840	1.13	7.24	6.57E-05	0.000402074	Ras family protein
TGME49_270940	-8.50	4.93	6.59E-05	0.00040332	hypothetical protein
TGME49_224580	-1.86	6.71	6.61E-05	0.000403557	RNA recognition motif-containing protein
TGME49_242118	-8.44	5.88	6.61E-05	0.000403557	myosin-light-chain kinase
TGME49_253510	2.42	6.34	6.64E-05	0.000405041	transporter/permease protein
TGME49_220450	-8.43	4.82	6.75E-05	0.000410851	ribonuclease HI protein
TGME49_262030	-8.43	4.93	6.76E-05	0.000411377	ALG6, ALG8 glycosyltransferase family protein
TGME49_208500	-2.35	6.45	6.79E-05	0.000412097	protein phosphatase 2C domain-containing protei
TGME49_243530	-8.44	4.75	6.79E-05	0.000412097	pentatricopeptide repeat domain-containing prot
TGME49_316300	-8.44	5.17	6.79E-05	0.000412097	ATP-dependent DNA helicase, RecQ family protei
TGME49_227310	-3.69	5.53	6.85E-05	0.000414737	hypothetical protein
TGME49_230590	-8.44	5.22	6.85E-05	0.000414737	chitobiosyldiphosphodolichol beta-mannosyltrans
TGME49_309580	-2.95	5.70	6.87E-05	0.000415668	transporter, major facilitator family protein
TGME49_213420	-8.43	4.75	6.95E-05	0.00042012	RAP domain-containing protein
TGME49_270720	-8.55	5.34	6.97E-05	0.000420827	hypothetical protein
TGME49_264870	1.73	7.51	6.97E-05	0.000420863	Sodium:neurotransmitter symporter family protei
TGME49_268210	-8.89	5.75	6.99E-05	0.000421609	AGC kinase
TGME49_202180	-1.70	6.37	7.04E-05	0.000423957	hypothetical protein
TGME49_318525	-1.80	6.78	7.09E-05	0.000426681	hypothetical protein

TGME49_260160	3.22	4.70	7.11E-05	0.000427524	hypothetical protein
TGME49_233220	1.38	7.46	7.11E-05	0.00042759	hypothetical protein
TGME49_285490	-8.43	5.57	7.19E-05	0.000431905	helix-hairpin-helix motif domain-containing protei
TGME49_268590	1.07	7.11	7.27E-05	0.000435831	rhomboid protease ROM4
TGME49_259210	-8.43	5.17	7.27E-05	0.000435831	jmjC domain-containing protein C2orf60
TGME49_215210	2.40	6.31	7.29E-05	0.000436607	hypothetical protein
TGME49_234670	1.96	5.29	7.32E-05	0.00043773	actin-like family protein
TGME49_263000	1.16	7.22	7.34E-05	0.000438624	Beige/BEACH domain-containing protein
TGME49_229780	-3.65	5.86	7.39E-05	0.000441005	GHMP kinase, N-terminal domain-containing prot
TGME49_316250	1.68	8.50	7.41E-05	0.000441917	hypothetical protein
TGME49_261510	3.43	5.09	7.43E-05	0.000442624	hypothetical protein
TGME49_289180	-8.44	5.18	7.65E-05	0.000454693	thioredoxin family redox-active protein, putative
TGME49_213240	-8.45	5.32	7.65E-05	0.000454693	hypothetical protein
TGME49_278470	-3.77	5.56	7.68E-05	0.000456049	hypothetical protein
TGME49_254910	-2.58	6.12	7.71E-05	0.000457372	hypothetical protein
TGME49_309250	-1.73	6.68	7.92E-05	0.000469158	hypothetical protein
TGME49_278920	-2.97	5.87	8.00E-05	0.00047305	hypothetical protein
TGME49_224660	1.13	6.78	8.02E-05	0.000473976	transcription factor s-ii (tfiis), central domain-cont
TGME49_254090	-1.68	6.52	8.03E-05	0.000474166	hypothetical protein
TGME49_285650	-1.50	6.83	8.07E-05	0.000476358	hypothetical protein
TGME49_285840	1.67	6.34	8.17E-05	0.00048191	RAP domain-containing protein
TGME49_206480	1.17	7.77	8.26E-05	0.000486414	hypothetical protein
TGME49_232020	-1.94	6.60	8.28E-05	0.000487694	hypothetical protein
TGME49_271870	-8.39	5.32	8.35E-05	0.000491295	zinc carboxypeptidase superfamily protein
TGME49_317705	-2.02	6.25	8.45E-05	0.000496497	enoyl-CoA hydratase/isomerase family protein
TGME49_271350	-8.39	5.41	8.62E-05	0.000506431	bifunctional protein FolC subfamily protein
TGME49_204050	1.54	10.48	8.65E-05	0.00050764	subtilisin SUB1
TGME49_231600	-2.33	6.14	8.66E-05	0.000507988	HEAT repeat-containing protein
TGME49_203450	-2.15	6.40	8.75E-05	0.000511962	DUF3228 domain-containing protein
TGME49_272680	-8.39	5.08	8.86E-05	0.000517399	hypothetical protein
TGME49_224150	-8.39	4.83	8.87E-05	0.000517579	hypothetical protein
TGME49_223510	-8.39	5.27	8.92E-05	0.000520224	hypothetical protein
TGME49_311110	-3.62	5.89	9.07E-05	0.00052844	Ubiquitin-fold modifier 1 precursor family protein,
TGME49_226970	1.16	10.38	9.24E-05	0.000537274	ribosomal protein RPS11
TGME49_264660	-1.28	9.56	9.26E-05	0.000538039	SAG-related sequence SRS44
TGME49_263330	-8.48	4.96	9.28E-05	0.000538575	Adaptin ear-binding coat-associated protein 2 (NE
TGME49_254290	1.41	6.05	9.30E-05	0.000539215	hypothetical protein
TGME49_267560	2.04	5.89	9.49E-05	0.000548852	folate-binding protein YgfZ protein
TGME49_228320	-8.39	4.69	9.49E-05	0.000548852	hypothetical protein
TGME49_246060	-8.46	5.11	9.49E-05	0.000548852	DNA-dependent RNA polymerase
TGME49_284010	-3.62	5.48	9.53E-05	0.000550411	5'-3' exonuclease, N-terminal resolvase family dor
TGME49_270750	-2.18	6.29	9.71E-05	0.000559844	hypothetical protein
TGME49_283850	-1.69	8.26	9.81E-05	0.000564943	peptidyl-prolyl cis-trans isomerase
TGME49_209460	-8.39	4.82	9.86E-05	0.000567484	hypothetical protein
TGME49_240960	2.32	7.06	9.90E-05	0.000568497	AIG2 family protein
TGME49_234520	-2.31	5.92	9.91E-05	0.000568497	U2 snRNP auxilliary factor, large subunit, splicing f
TGME49_216850	-8.38	4.72	9.91E-05	0.000568497	hypothetical protein
TGME49_261000	-1.53	7.03	0.00010027	0.000574724	MutS domain V domain-containing protein
TGME49_284170	-8.39	4.98	0.000100406	0.000575057	DHHC zinc finger domain-containing protein
TGME49_293840	1.34	6.85	0.000101199	0.000578306	hypothetical protein
TGME49_221370	-3.61	5.30	0.000101207	0.000578306	hypothetical protein
TGME49_228340	-8.39	5.00	0.000101063	0.000578306	hypothetical protein
TGME49_275670	-1.17	8.05	0.000101348	0.000578661	alveolin domain containing intermediate filament
TGME49_318480	-2.29	6.16	0.000101773	0.00058064	SWI2/SNF2-containing protein RAD5

TGME49_246030	-8.38	5.02	0.000102365	0.000583566	mediator complex subunit MED17
TGME49_268870	-1.28	7.46	0.000103269	0.000588265	tetratricopeptide repeat-containing protein
TGME49_281990	1.60	5.47	0.000103385	0.000588326	Nicotinamidase
TGME49_264880	-2.29	6.07	0.000103439	0.000588326	NEDD8-activating enzyme E1 catalytic subunit
TGME49_288800	3.01	6.83	0.000104709	0.0005946	endonuclease/exonuclease/phosphatase family pr
TGME49_246190	-2.53	6.02	0.000104783	0.0005946	hypothetical protein
TGME49_312500	-1.52	7.05	0.000105089	0.000595878	hypothetical protein
TGME49_208340	-1.20	7.28	0.000105784	0.000599363	hypothetical protein
TGME49_262950	1.01	7.72	0.000105983	0.000600027	hypothetical protein
TGME49_220870	1.62	5.64	0.000106121	0.000600349	hypothetical protein
TGME49_221610	-2.13	6.25	0.000106373	0.000601316	ubiquitin carboxyl-terminal hydrolase
TGME49_211600	-2.28	6.15	0.000108381	0.000612201	hypothetical protein
TGME49_268720	-2.91	5.82	0.000110293	0.000622046	Hrf1 family protein
TGME49_312190	-1.68	6.85	0.000110587	0.00062323	hypothetical protein
TGME49_300270	1.12	7.18	0.00011175	0.000629309	hypothetical protein
TGME49_248740	1.35	8.77	0.000112872	0.000634659	hypothetical protein
TGME49_275990	1.20	6.58	0.000113295	0.000636551	hypothetical protein
TGME49_217050	1.16	7.26	0.000113549	0.000637498	ADA2-A transcriptional co-activator SAGA compon
TGME49_221690	-3.61	5.62	0.000113739	0.000638079	hypothetical protein
TGME49_224950	-1.17	7.33	0.000114213	0.000640249	calcium-dependent protein kinase CDPK5
TGME49_321590	-1.34	7.17	0.000115294	0.000645334	hypothetical protein
TGME49_316230	-2.26	6.16	0.000115997	0.000648773	SAC1 phosphoinositide phosphatase, putative
TGME49_248640	-1.33	7.36	0.000116543	0.000651088	regulator of chromosome condensation (RCC1) re
TGME49_243290	-2.10	6.21	0.000116586	0.000651088	hypothetical protein
TGME49_225470	1.25	6.28	0.000116941	0.000652578	peptide methionine sulfoxide reductase
TGME49_275980	-3.58	5.59	0.000117486	0.000655124	coenzyme q (ubiquinone) biosynthesis protein coq
TGME49_286140	-2.10	5.93	0.00011759	0.000655206	hypothetical protein
TGME49_294390	-8.33	5.02	0.000117733	0.000655511	myosin light chain MLC4, putative
TGME49_237480	-3.58	5.71	0.000118364	0.000658148	BRCA1 C Terminus (BRCT) domain-containing prot
TGME49_310520	-3.61	5.14	0.000118506	0.000658328	3'5'-cyclic nucleotide phosphodiesterase domain-c
TGME49_244670	-1.56	6.91	0.000119222	0.000660816	hypothetical protein
TGME49_233860	-8.40	4.93	0.000119143	0.000660816	DALR anticodon binding domain-containing protei
TGME49_307020	-1.89	6.25	0.000119891	0.000663526	hypothetical protein
TGME49_239620	-3.58	5.74	0.000120509	0.000666448	5'-nucleotidase, C-terminal domain-containing pro
TGME49_310360	-1.39	7.41	0.000120662	0.000666799	hypothetical protein
TGME49_278960	-1.75	6.55	0.000121697	0.000672012	hypothetical protein
TGME49_312340	-8.33	5.02	0.000121787	0.000672012	hypothetical protein
TGME49_209960	-1.70	6.95	0.000123358	0.000680168	glycosyltransferase
TGME49_310380	-8.34	5.10	0.000123768	0.000681924	brix domain containing protein
TGME49_318590	-8.37	4.79	0.000125169	0.000689127	MRP family domain-containing protein
TGME49_201120	-1.68	6.58	0.000125797	0.00069207	ELMO/CED-12 family protein
TGME49_203840	1.45	5.77	0.000125931	0.000692295	DEAD/DEAH box helicase domain-containing prote
TGME49_264960	-8.34	5.17	0.000127115	0.000698284	hypothetical protein
TGME49_218820	-1.40	8.78	0.00012754	0.000700094	alba 2
TGME49_239080	-8.34	4.71	0.000130261	0.000712918	carrier superfamily protein
TGME49_239710	-8.33	5.20	0.000130738	0.000715	phosphomannomutase
TGME49_244110	-1.27	8.05	0.000131107	0.000716485	nucleosome assembly protein (nap) protein
TGME49_258680	-2.07	6.21	0.000132451	0.000723296	TATA-box binding protein TBP2
TGME49_227390	-2.88	5.44	0.000134374	0.000732717	hypothetical protein
TGME49_240660	-8.35	4.90	0.000135116	0.000736215	hypothetical protein
TGME49_290340	-2.30	6.17	0.000135936	0.000740139	HEAT repeat-containing protein
TGME49_259830	1.56	6.46	0.000136716	0.000743292	diacylglycerol kinase catalytic domain-containing p
TGME49_261600	1.04	7.25	0.00013694	0.000743963	creatinase domain-containing protein
TGME49_306330	-1.93	6.75	0.000139289	0.000755617	phospholipase

TGME49_224900	-1.16	7.72	0.000140568	0.000761992	adenylate kinase, putative
TGME49_246530	-3.69	5.27	0.000141163	0.00076466	phospholipase D active site domain-containing pro
TGME49_230010	-1.93	6.26	0.000141695	0.000766979	hypothetical protein
TGME49_203970	-2.86	5.54	0.000141975	0.00076764	dolichyl-diphosphooligosaccharide--protein glycos
TGME49_283810	-3.59	5.42	0.000142025	0.00076764	hypothetical protein
TGME49_297890	1.20	6.67	0.000144451	0.000779614	hypothetical protein
TGME49_300260	-1.04	8.27	0.000144948	0.000781727	threonyl-tRNA synthetase family protein
TGME49_255940	1.68	5.25	0.000147011	0.000792274	hypothetical protein
TGME49_294720	-2.56	5.83	0.000148705	0.00080082	hypothetical protein
TGME49_260650	-3.58	5.40	0.000150313	0.000808302	glycosyltransferase, group 2 family protein
TGME49_257290	-8.36	5.89	0.000150497	0.000808595	RNA 2'-phosphotransferase, Tpt1/KptA family prot
TGME49_316570	1.04	7.16	0.000151569	0.000813281	hypothetical protein
TGME49_238170	1.36	6.42	0.000152903	0.00081925	hypothetical protein
TGME49_224235	-8.28	5.14	0.00015421	0.000825654	translation initiation factor IF-3 protein
TGME49_224230	2.04	5.03	0.000156979	0.000839872	AP2 domain transcription factor AP2X-3
TGME49_242260	1.48	7.17	0.000157792	0.000842339	hypothetical protein
TGME49_299050	1.17	10.43	0.000157613	0.000842339	ribosomal protein RPL17
TGME49_315500	-1.60	6.66	0.000157896	0.000842339	hypothetical protein
TGME49_206390	-8.28	4.99	0.000157715	0.000842339	hypothetical protein
TGME49_248880	-1.20	7.90	0.000158406	0.000844105	GTPase RAB7
TGME49_261710	-1.64	6.65	0.000158455	0.000844105	ankyrin repeat-containing protein
TGME49_294430	-3.54	5.21	0.000160072	0.000852106	hypothetical protein
TGME49_252070	1.65	5.12	0.00016075	0.0008551	KRUF family protein
TGME49_230500	-3.54	5.36	0.000160992	0.000855771	hypothetical protein
TGME49_293330	-8.28	5.03	0.000161712	0.000858573	hypothetical protein
TGME49_217350	-8.29	5.39	0.000162054	0.00085956	methyltransferase MTA70, putative
TGME49_216500	-3.54	5.77	0.000162523	0.000861432	tRNA synthetase, putative
TGME49_212725	-2.49	5.73	0.000163716	0.000866509	hypothetical protein
TGME49_238000	-3.58	5.17	0.000164669	0.000870932	peptidyl-prolyl isomerase
TGME49_236800	-8.29	5.06	0.000164972	0.000871908	hypothetical protein
TGME49_215550	-2.55	5.78	0.000165366	0.000873367	hypothetical protein
TGME49_294050	1.60	7.02	0.000165739	0.000874715	hypothetical protein
TGME49_258180	-8.34	4.82	0.000165943	0.000875165	hypothetical protein
TGME49_246160	2.25	4.93	0.000166186	0.000875824	hypothetical protein
TGME49_288260	-1.61	6.74	0.000166641	0.000877594	hypothetical protein
TGME49_254380	-8.29	5.12	0.000168407	0.000886265	ribosomal protein L11, putative
TGME49_221950	-1.47	6.81	0.000169746	0.000892675	splicesome-associated protein, putative
TGME49_257370	2.21	5.96	0.00017024	0.000894636	hypothetical protein
TGME49_205160	1.94	5.28	0.000170978	0.000897878	hypothetical protein
TGME49_307980	2.27	5.22	0.000171369	0.000899294	GTP-binding protein lepA, putative
TGME49_216870	-2.22	5.83	0.00017196	0.000901753	DNA excision repair helicase
TGME49_235140	1.74	7.26	0.000172893	0.000906003	hypothetical protein
TGME49_216930	-1.60	6.83	0.000173184	0.000906883	cholinephosphate cytidylyltransferase
TGME49_313385	1.96	5.03	0.000174126	0.000909972	hypothetical protein
TGME49_264650	-8.32	4.90	0.000175397	0.000915236	phosphoacetylglucosamine mutase
TGME49_232780	-2.50	6.30	0.000175714	0.000916241	hypothetical protein
TGME49_297150	-1.30	7.18	0.000176262	0.000918449	MORN repeat-containing protein
TGME49_306030	-1.53	7.04	0.000178266	0.000926753	glutathione s-transferase, n-terminal domain cont
TGME49_216920	-2.52	5.62	0.000178203	0.000926753	mediator complex subunit MED8
TGME49_313418	-8.32	5.19	0.000178357	0.000926753	hypothetical protein
TGME49_226330	-8.28	4.87	0.00017944	0.00093173	hypothetical protein
TGME49_233890	-2.25	6.03	0.00017967	0.000932266	hypothetical protein
TGME49_312905	1.94	7.21	0.000180105	0.000933341	hypothetical protein
TGME49_294380	-2.83	5.40	0.000180129	0.000933341	PP-loop domain-containing protein

TGME49_273030	-2.83	5.76	0.00018094	0.00093623	phosphoglycerate mutase family protein
TGME49_311090	-1.28	7.32	0.00018231	0.000942656	ubiquitin carboxyl-terminal hydrolase
TGME49_285980	1.02	8.53	0.000185571	0.00095818	glucosephosphate-mutase GPM1
TGME49_271110	-3.54	5.21	0.000186922	0.000964481	hypothetical protein
TGME49_309800	-1.53	6.85	0.000190219	0.000980124	RNA recognition motif-containing protein
TGME49_264720	-3.51	5.63	0.000193637	0.000995656	hypothetical protein
TGME49_312430	1.36	6.02	0.000194116	0.000996737	hypothetical protein
TGME49_277070	1.47	5.84	0.000194575	0.000998398	SWI2/SNF2-containing protein
TGME49_271860	-8.31	4.88	0.000194995	0.000999857	tRNA (Uracil-5-)-methyltransferase
TGME49_226740	1.27	7.97	0.000195664	0.001002593	zinc finger, C3HC4 type (RING finger) domain-cont
TGME49_281570	-3.51	5.67	0.000197541	0.00101151	hypothetical protein
TGME49_203180	-3.51	5.86	0.000198349	0.001014944	leucine rich repeat-containing protein
TGME49_306410	-3.51	5.79	0.000198997	0.001017553	hypothetical protein
TGME49_221580	-1.28	7.41	0.00019962	0.001020035	ribosomal RNA large subunit methyltransferase J p
TGME49_294740	-1.91	6.49	0.000200004	0.001021292	hypothetical protein
TGME49_257340	-8.23	5.20	0.000200215	0.001021663	Ras family protein
TGME49_305850	-1.04	7.71	0.000200638	0.001023113	RNA recognition motif-containing protein
TGME49_211090	-1.51	6.64	0.000201073	0.001023923	aminotransferase, class V superfamily protein
TGME49_266100	1.08	6.94	0.000202356	0.001029744	rhoptry kinase family protein ROP41
TGME49_306560	-2.91	5.99	0.000202761	0.001030161	hypothetical protein
TGME49_217750	-3.51	5.56	0.000202708	0.001030161	hypothetical protein
TGME49_301130	-8.23	5.01	0.000202856	0.001030161	hypothetical protein
TGME49_226560	-3.51	5.49	0.000203341	0.001031565	zinc finger (CCCH type) motif-containing protein
TGME49_306210	-8.34	5.06	0.000203411	0.001031565	RNA polymerase II accessory factor CDC73
TGME49_202630	-1.21	7.37	0.00020417	0.001034706	ATP-dependent metallopeptidase HflB subfamily p
TGME49_289190	-8.23	4.82	0.000205057	0.001038489	tetratricopeptide repeat-containing protein
TGME49_313140	1.42	8.23	0.000205206	0.00103853	isocitrate dehydrogenase
TGME49_213115	-8.23	4.95	0.000206075	0.001042218	hypothetical protein
TGME49_282140	1.28	6.45	0.000206701	0.001044084	cwf21 protein
TGME49_264040	-1.11	7.86	0.000209065	0.001053015	hypothetical protein
TGME49_208780	-2.45	6.22	0.000209372	0.001053845	ubiquitin-conjugating enzyme subfamily protein
TGME49_272670	-8.23	4.99	0.000210683	0.00105972	peptidase family M3 protein
TGME49_238180	-1.10	7.48	0.00021103	0.001060024	26s proteasome regulatory complex subunit, puta
TGME49_233490	-8.29	5.10	0.000213832	0.001073369	hypothetical protein
TGME49_233790	-8.22	5.24	0.000214028	0.001073625	serine/threonine protein kinase AktR, putative
TGME49_293280	1.31	5.89	0.00021457	0.001075615	cyclin protein
TGME49_286230	1.29	6.35	0.00021533	0.001078691	hypothetical protein
TGME49_310320	-3.51	5.64	0.000215528	0.001078951	calreticulin family protein
TGME49_252870	1.95	4.88	0.000215908	0.001080126	hypothetical protein
TGME49_237290	-1.40	7.15	0.000217042	0.00108433	hypothetical protein
TGME49_268620	-8.23	4.67	0.000217371	0.001085239	blood stage antigen 41-3 precursor, putative
TGME49_221640	2.25	6.98	0.000218155	0.001088417	hypothetical protein
TGME49_213880	2.50	4.99	0.000218743	0.001089881	hypothetical protein
TGME49_309160	-2.03	6.33	0.000219447	0.001092656	IgA-specific metalloendopeptidase
TGME49_260450	-8.23	5.10	0.000220112	0.001095226	DEAD/DEAH box helicase domain-containing prote
TGME49_248850	-8.24	5.24	0.000220826	0.00109804	methionine aminopeptidase
TGME49_254800	1.72	5.35	0.000221776	0.001102025	hypothetical protein
TGME49_300380	-2.80	5.70	0.000221983	0.001102313	endoplasmic reticulum oxidoreductin, putative
TGME49_276170	1.39	9.19	0.000223263	0.001107799	phosphatidylinositol 3- and 4-kinase
TGME49_307040	-1.48	6.89	0.000223388	0.001107799	shikimate dehydrogenase substrate binding doma
TGME49_265850	1.90	6.27	0.000227169	0.001125798	hypothetical protein
TGME49_269290	-2.43	6.26	0.00022976	0.001137872	hypothetical protein
TGME49_318610	1.60	5.24	0.00023033	0.001139933	AP2 domain transcription factor AP2IV-3
TGME49_235460	1.27	6.18	0.000231354	0.001144233	hypothetical protein

TGME49_311250	-2.87	6.06	0.000233011	0.001150889	hypothetical protein
TGME49_202680	-1.26	8.26	0.000240708	0.001187324	peptidase M16, alpha subunit, putative
TGME49_234220	-1.87	6.36	0.000241926	0.001192533	hypothetical protein
TGME49_215920	1.79	5.98	0.000242237	0.001193271	hypothetical protein
TGME49_244470	-1.14	7.80	0.000243628	0.001199323	hypothetical protein
TGME49_244660	-3.51	5.30	0.000244869	0.001204634	hypothetical protein
TGME49_252320	-8.22	5.00	0.000246395	0.001211332	Sas10/Utp3/C1D family protein
TGME49_270060	-2.22	5.79	0.000247757	0.001217218	hypothetical protein
TGME49_262500	-1.42	7.01	0.000248574	0.001220423	hypothetical protein
TGME49_315700	-2.80	5.81	0.000249277	0.001222249	hypothetical protein
TGME49_316540	-1.15	7.70	0.000251845	0.001234024	IMC sub-compartment protein ISP3
TGME49_214870	1.67	5.32	0.00025297	0.001238716	ribosomal protein L9, N-terminal domain-containi
TGME49_258030	-1.82	6.12	0.000254148	0.001243658	DNA polymerase
TGME49_253700	1.04	8.40	0.000258836	0.001263254	transporter, major facilitator family protein
TGME49_251670	-3.47	5.58	0.000259152	0.001263964	werner helicase interacting protein 1, putative
TGME49_313860	1.80	7.16	0.00026019	0.001268193	regulator of chromosome condensation (RCC1) re
TGME49_249840	-8.17	5.34	0.000260988	0.00127124	dynein heavy chain 2, putative
TGME49_272320	1.53	5.67	0.000261804	0.001274376	DHHC zinc finger domain-containing protein
TGME49_218270	-1.01	7.57	0.000262711	0.00127795	hypothetical protein
TGME49_263610	-1.59	6.91	0.000265056	0.00128851	hypothetical protein
TGME49_202460	-1.77	6.49	0.000265873	0.001291631	diacylglycerol kinase accessory domain (presumed
TGME49_271000	-2.27	5.98	0.000267343	0.001297923	hypothetical protein
TGME49_321300	-3.47	5.37	0.000268055	0.001299958	autophagy-related 12 variant 1, putative
TGME49_234950	1.64	6.24	0.000268681	0.001300969	protein kinase (incomplete catalytic triad)
TGME49_220920	-8.17	4.78	0.000268652	0.001300969	hypothetical protein
TGME49_235560	-8.18	5.26	0.000269339	0.001302482	hypothetical protein
TGME49_257595	-2.88	5.61	0.000269797	0.001303464	hypothetical protein
TGME49_305600	-8.17	4.82	0.000269895	0.001303464	hypothetical protein
TGME49_223672	1.66	5.57	0.00027023	0.001304231	3'(2'),5'-bisphosphate nucleotidase
TGME49_229340	-8.17	4.88	0.000270579	0.001305065	hypothetical protein
TGME49_247380	-8.17	5.36	0.000272948	0.001315628	hypothetical protein
TGME49_239610	-3.47	5.48	0.000273559	0.001317716	hypothetical protein
TGME49_273850	-2.77	5.77	0.000274089	0.001318614	hypothetical protein
TGME49_312380	-8.17	4.96	0.000274102	0.001318614	tetratricopeptide repeat-containing protein
TGME49_230710	-3.53	5.18	0.00027871	0.00133991	cell division protein CDC48
TGME49_316360	-3.53	5.40	0.000279845	0.001344494	hypothetical protein
TGME49_229940	-2.40	5.72	0.000280603	0.001346387	cyclophilin, putative
TGME49_242850	-2.40	5.93	0.000280864	0.001346763	hypothetical protein
TGME49_216680	-2.15	7.27	0.000283288	0.001354872	ankyrin repeat-containing protein
TGME49_289000	-2.77	5.79	0.000283136	0.001354872	hypothetical protein
TGME49_228720	-8.17	4.72	0.000283009	0.001354872	hypothetical protein
TGME49_252360	1.02	9.00	0.000284139	0.001356029	rhoptry kinase family protein ROP24 (incomplete
TGME49_312618	-1.89	5.90	0.000284264	0.001356029	hypothetical protein
TGME49_321610	-8.17	4.89	0.000284101	0.001356029	hypothetical protein
TGME49_235750	-8.17	5.02	0.000284723	0.001357344	ULK kinase
TGME49_216970	-1.15	7.94	0.000286589	0.001365362	coronin, putative
TGME49_260370	-1.99	6.27	0.000287321	0.001367967	AtPH1 family protein
TGME49_315690	-3.47	5.51	0.000288086	0.001370724	DnaJ domain-containing protein
TGME49_270610	1.55	5.44	0.000291374	0.00138353	hypothetical protein
TGME49_238110	-2.21	6.29	0.000291525	0.00138353	replication factor a protein 3 protein
TGME49_248890	-2.40	6.03	0.000293937	0.001394078	actin-like protein ALP3b
TGME49_235680	-1.67	7.23	0.000294212	0.001394488	peptidase M16 inactive domain-containing protei
TGME49_319940	-8.17	4.88	0.000295351	0.00139899	hypothetical protein
TGME49_312310	-2.81	5.42	0.000296476	0.001403422	ATPase, AAA family protein

TGME49_239630	-3.49	5.29	0.000300242	0.001420337	cytidine and deoxycytidylate deaminase zinc-bindi
TGME49_272475	-8.17	5.24	0.000301262	0.001424251	protein kinase domain-containing protein
TGME49_212090	1.11	6.61	0.000302007	0.001425951	hypothetical protein
TGME49_275330	-3.46	5.64	0.000301969	0.001425951	ribosomal protein RPL29
TGME49_201230	-2.76	5.61	0.000308872	0.001456503	kinesin motor domain-containing protein
TGME49_252220	-1.36	7.73	0.000311799	0.001469372	tetratricopeptide repeat domain containing protei
TGME49_207940	-3.47	5.31	0.000314362	0.001480507	ribosomal protein S9, putative
TGME49_318460	-1.58	6.53	0.000318007	0.001495768	P-type ATPase of unknown pump specificity (type
TGME49_204270	-2.21	6.05	0.000319175	0.001500306	hypothetical protein
TGME49_263590	-2.42	5.74	0.000319465	0.001500713	hypothetical protein
TGME49_309220	-1.37	7.36	0.000321722	0.001510356	GTPase activating protein for Arf protein
TGME49_210360	1.75	6.68	0.000322762	0.001514277	DEAD (Asp-Glu-Ala-Asp) box polypeptide 41 family
TGME49_310350	-1.78	6.39	0.000323431	0.001516453	PGAP1 family protein
TGME49_300990	1.85	5.28	0.000324463	0.001520329	Toxoplasma gondii family C protein
TGME49_263530	-1.60	6.47	0.000325395	0.00152373	chaperonin , putative
TGME49_265400	-1.62	6.69	0.000327456	0.001532412	hypothetical protein
TGME49_213670	-1.46	6.71	0.000330405	0.001545233	hypothetical protein
TGME49_238230	-2.77	5.73	0.000331045	0.001546283	Ser/Thr phosphatase family protein
TGME49_267390	-1.57	6.54	0.00033138	0.001546859	DNA-directed RNA polymerase I RPAC1
TGME49_300060	-1.10	7.18	0.000332331	0.00155032	signal peptidase subunit protein
TGME49_208590	-1.53	7.00	0.000336002	0.001566454	vacuolar ATP synthase subunit 54kD, putative
TGME49_232360	-1.60	6.79	0.000336842	0.001567408	exonuclease
TGME49_288570	-2.74	5.83	0.000336736	0.001567408	hypothetical protein
TGME49_239130	1.45	5.73	0.000338538	0.001574309	Tyrosine kinase-like (TKL) protein
TGME49_318750	1.28	5.79	0.000339957	0.001578919	deoxyribose-phosphate aldolase
TGME49_226660	-1.30	7.06	0.00033981	0.001578919	hypothetical protein
TGME49_247330	-1.75	6.92	0.000342685	0.001589592	hypothetical protein
TGME49_269140	-1.79	6.33	0.000342489	0.001589592	transport protein particle component, Bet3 domai
TGME49_219460	-2.74	5.68	0.000350061	0.001621774	hypothetical protein
TGME49_270160	-8.11	4.78	0.000354903	0.001642149	hypothetical protein
TGME49_311280	-8.12	4.70	0.000356701	0.001648405	hypothetical protein
TGME49_234610	-1.55	6.61	0.000360883	0.001663623	WD-40 repeat protein
TGME49_310810	-1.83	6.36	0.000360894	0.001663623	apyrase
TGME49_214800	-8.11	4.74	0.000369446	0.001700926	hypothetical protein
TGME49_301180	2.01	5.49	0.000369888	0.001701901	SAG-related sequence SRS19F
TGME49_234440	-3.44	5.42	0.00037356	0.001715593	aminotransferase, putative
TGME49_240830	-8.11	4.70	0.000375009	0.001721178	hydrolase, alpha/beta fold family protein
TGME49_236280	2.04	4.85	0.000376246	0.001724463	hypothetical protein
TGME49_252510	1.49	5.83	0.000376425	0.001724463	hypothetical protein
TGME49_297510	-8.12	5.32	0.000375998	0.001724463	hypothetical protein
TGME49_270650	1.77	5.24	0.000381123	0.001743825	deoxyribose-phosphate aldolase
TGME49_224330	-1.28	6.99	0.000386136	0.001763492	proteasome activator pa28 beta subunit protein
TGME49_310560	-3.42	5.55	0.000385929	0.001763492	hypothetical protein
TGME49_297720	-1.07	7.54	0.000387048	0.001766562	trehalose-phosphatase
TGME49_307810	-1.21	7.09	0.000388971	0.001774247	hypothetical protein
TGME49_258920	-8.13	4.81	0.000390777	0.001781382	hypothetical protein
TGME49_205570	-2.73	5.58	0.000391146	0.001781966	hypothetical protein
TGME49_255170	-3.43	5.01	0.000391958	0.001784271	hypothetical protein
TGME49_255690	-2.74	5.59	0.000393935	0.001791363	2-C-methyl-D-erythritol 2,4-cyclodiphosphate synt
TGME49_227430	-1.72	6.17	0.000394558	0.001793093	transmembrane amino acid transporter protein
TGME49_257150	-2.73	5.49	0.000395383	0.001795737	NOT2 / NOT3 / NOT5 family protein
TGME49_226840	-2.74	5.41	0.000395662	0.001795904	hypothetical protein
TGME49_267120	-2.74	5.47	0.000397422	0.00180256	hypothetical protein
TGME49_248130	-8.13	4.76	0.00040149	0.001818039	hypothetical protein

TGME49_253300	2.29	4.94	0.000402124	0.001818442	hypothetical protein
TGME49_306290	-1.87	6.40	0.000402349	0.001818442	DNA-directed RNA polymerase III RPC1
TGME49_297460	-8.12	5.02	0.000402327	0.001818442	hypothetical protein
TGME49_204140	-8.17	4.77	0.000404667	0.001827799	PHD-finger domain-containing protein
TGME49_209080	1.29	6.24	0.000405496	0.001830427	transport protein particle (trapp) component, bet3
TGME49_273840	-2.40	5.63	0.000406807	0.001835224	brix domain-containing protein
TGME49_209930	-8.12	5.30	0.000412854	0.001856839	hypothetical protein
TGME49_308580	-2.37	5.95	0.000413169	0.001857127	Lon protease family protein
TGME49_313240	-2.78	5.84	0.000413784	0.00185876	ethylene-responsive RNA helicase, putative
TGME49_264440	-1.83	6.16	0.000425412	0.001906385	signal recognition particle receptor beta subunit p
TGME49_231890	-1.86	6.02	0.000425621	0.001906385	beta-ketoacyl-acyl carrier protein synthase III, puta
TGME49_215290	-3.39	5.79	0.000429861	0.001923965	saccharopine dehydrogenase domain-containing p
TGME49_299110	-1.95	6.49	0.000434013	0.001941376	cleft lip and palate transmembrane protein 1 (clpt
TGME49_223060	-1.76	6.32	0.000434715	0.001943339	MORN repeat-containing protein
TGME49_314840	-1.63	6.47	0.000435466	0.001944345	ubiquitin carboxyl-terminal hydrolase
TGME49_311510	-1.44	8.65	0.000436566	0.001948078	eIF2 kinase IF2K-B
TGME49_304760	1.05	7.95	0.000440147	0.001962875	RNA recognition motif-containing protein
TGME49_221490	-2.70	5.40	0.000446759	0.00199116	cell cycle regulator protein
TGME49_297780	-2.34	6.07	0.00045559	0.002027997	ATPase/histidine kinase/DNA gyrase B/HSP90 dom
TGME49_257710	-3.39	5.56	0.000455847	0.002027997	actin-like protein ALP 5
TGME49_225680	-2.70	5.66	0.000459688	0.002043858	hypothetical protein
TGME49_227600	-1.12	9.30	0.000460659	0.002046775	ribosomal protein RPL34
TGME49_224350	-1.37	8.79	0.000460898	0.002046775	aminopeptidase N, putative
TGME49_233820	-8.05	4.93	0.000461811	0.0020496	DNA polymerase epsilon subunit B protein
TGME49_255390	-3.39	5.42	0.000465031	0.002062652	HEAT repeat-containing protein
TGME49_217360	-2.70	5.95	0.000466158	0.002066411	hypothetical protein
TGME49_273350	1.51	5.36	0.000469482	0.002079899	molybdopterin converting factor, subunit 2 protei
TGME49_230430	-8.05	5.34	0.000470515	0.002083225	vesicle-associated membrane protein, putative
TGME49_244230	-8.06	5.32	0.000471535	0.002085244	hypothetical protein
TGME49_242435	-3.39	5.43	0.000472142	0.002086683	hypothetical protein
TGME49_312280	-1.39	6.89	0.000473532	0.002091574	pre-mRNA-splicing factor ATP-dependent RNA hel
TGME49_295450	-1.22	7.30	0.000476546	0.002102763	sjoegren syndrome nuclear autoantigen 1 family p
TGME49_212150	-8.06	5.30	0.000476634	0.002102763	hypothetical protein
TGME49_251890	-3.39	5.68	0.000477605	0.002104772	hypothetical protein
TGME49_318290	-8.05	5.09	0.000477962	0.002104857	hypothetical protein
TGME49_233090	-8.06	4.99	0.000479082	0.002107276	XPG N-terminal domain-containing protein
TGME49_320015	-8.05	4.68	0.000488741	0.002148483	hypothetical protein
TGME49_218250	-2.13	6.10	0.00048922	0.002149311	TAP42 family protein
TGME49_203420	1.97	5.22	0.000490063	0.002151739	4'-phosphopantetheinyl transferase domain-conta
TGME49_221360	-2.13	5.89	0.000490766	0.002153547	hypothetical protein
TGME49_219140	-1.48	6.80	0.000491443	0.002155237	EF-1 guanine nucleotide exchange domain-contair
TGME49_312840	-1.82	6.43	0.000492557	0.002158843	hypothetical protein
TGME49_221830	1.87	5.06	0.00049696	0.00217556	subtilisin SUB12
TGME49_285470	1.99	5.36	0.000500375	0.002187919	patched family protein
TGME49_270450	-8.05	4.74	0.000505018	0.002206175	MCM2/3/5 family protein
TGME49_224190	-1.56	6.40	0.000507577	0.002214175	cation-transporting atpase family protein
TGME49_223070	-1.12	7.72	0.000516222	0.002247908	hypothetical protein
TGME49_313150	2.10	6.73	0.000517113	0.002250462	DUF89/Fructose bisphosphatase
TGME49_226940	-3.41	5.08	0.00051879	0.002255104	ubiquitin carboxyl-terminal hydrolase
TGME49_291090	-8.07	5.22	0.000519452	0.002256658	SWI2/SNF2-containing protein
TGME49_297210	1.08	6.93	0.000521305	0.002263376	hypothetical protein
TGME49_217880	-8.05	4.80	0.000522203	0.002264993	RNA-binding protein Nova-1, putative
TGME49_290180	2.38	5.13	0.000526683	0.002281367	AP2 domain transcription factor AP2IX-6
TGME49_267140	-8.08	4.84	0.000527864	0.002285146	SAG-related sequence SRS38B

TGME49_230520	-1.22	6.96	0.000532586	0.002304238	cyclophilin 1, putative
TGME49_291010	2.02	5.14	0.000536255	0.002317545	hypothetical protein
TGME49_301350	-8.10	4.95	0.000536289	0.002317545	SNARE associated protein
TGME49_285520	-3.38	5.20	0.000538386	0.002325248	RNA cap guanine-N2 methyltransferase
TGME49_249820	-2.67	5.81	0.000541859	0.002338882	ATP-binding cassette sub-family B member 5
TGME49_208730	-1.15	8.92	0.000543215	0.002343366	microneme protein, putative
TGME49_314000	1.22	6.33	0.000544045	0.002345579	peptide methionine sulfoxide reductase msrB, put
TGME49_216760	-8.06	4.80	0.000546123	0.002353165	RNA pseudouridine synthase superfamily protein
TGME49_314970	-1.21	7.24	0.000548489	0.002361987	root hair defective 3 gtp-binding protein (rhd3) pr
TGME49_258850	-8.04	4.82	0.000550175	0.002367867	hypothetical protein
TGME49_220110	1.72	5.05	0.000550997	0.002370026	hypothetical protein
TGME49_321660	-3.42	4.88	0.000555026	0.002385964	mannosyltransferase, putative
TGME49_319950	-1.79	5.79	0.000556686	0.002390524	rRNA-processing protein FCF1, putative
TGME49_244610	-3.34	5.46	0.000556733	0.002390524	zinc finger, C3HC4 type (RING finger) domain-cont
TGME49_288840	1.74	7.58	0.000559293	0.002400123	hypothetical protein
TGME49_205625	-3.38	5.09	0.000561014	0.002404718	hypothetical protein
TGME49_219850	-1.44	8.47	0.000568308	0.00243316	prolyl-tRNA synthetase (ProRS)
TGME49_281910	1.12	6.18	0.000569217	0.002435641	hypothetical protein
TGME49_226540	1.29	6.38	0.000569598	0.002435862	protein kinase
TGME49_233310	-3.35	5.31	0.000569959	0.002435997	peptidase D, putative
TGME49_268280	-1.09	7.38	0.000572146	0.002443934	'chromo' (CHRromatin Organization MOdifier) dor
TGME49_231200	-2.31	6.19	0.000574134	0.002445462	hypothetical protein
TGME49_253020	-2.34	6.02	0.000574064	0.002445462	hypothetical protein
TGME49_297140	-2.67	5.78	0.00057345	0.002445462	U6 snRNA-associated sm family protein Lsm2, puta
TGME49_318300	-2.67	5.50	0.000572951	0.002445462	hypothetical protein
TGME49_309050	-8.03	4.88	0.000574158	0.002445462	hypothetical protein
TGME49_264030	-1.62	6.63	0.000575264	0.002448764	aminotransferase, putative
TGME49_203620	-3.35	5.21	0.000588604	0.002501231	hypothetical protein
TGME49_222840	1.50	8.70	0.000591496	0.002512073	Ser/Thr phosphatase family protein
TGME49_236780	-3.35	5.28	0.000594156	0.002521924	zinc finger, C3HC4 type (RING finger) domain-cont
TGME49_228230	1.08	9.12	0.000596173	0.00252903	hypothetical protein
TGME49_243410	-3.35	5.61	0.000599365	0.002541113	tetratricopeptide repeat-containing protein
TGME49_219300	1.39	5.66	0.000601019	0.002546669	ran binding protein
TGME49_313160	-2.67	5.73	0.000605155	0.002561257	hypothetical protein
TGME49_270150	-2.67	5.78	0.000605047	0.002561257	hypothetical protein
TGME49_253490	1.69	7.37	0.000606529	0.002565605	hypothetical protein
TGME49_300280	-2.36	5.77	0.000609087	0.002574951	LSM domain-containing protein
TGME49_206605	1.58	5.59	0.000614547	0.002596547	hypothetical protein
TGME49_204060	-2.67	5.60	0.000619393	0.00261553	SNARE domain-containing protein
TGME49_295850	-1.16	7.27	0.000621787	0.002624139	cyclic nucleotide-binding domain-containing prote
TGME49_278550	-1.98	6.00	0.000622749	0.002626701	elongation factor Tu GTP binding domain-containi
TGME49_215490	-1.27	8.43	0.000624165	0.002631172	transporter, major facilitator family protein
TGME49_200330	-1.65	6.65	0.000626292	0.002638635	hypothetical protein
TGME49_239320	-7.99	4.74	0.000628142	0.002643417	BolA family protein
TGME49_275780	-7.99	4.85	0.000627979	0.002643417	hypothetical protein
TGME49_202080	-7.99	4.81	0.000629479	0.002644792	hypothetical protein
TGME49_203010	-1.15	7.22	0.000630523	0.002647408	aurora kinase
TGME49_216240	-1.77	6.30	0.000632197	0.002651426	hypothetical protein
TGME49_315590	-2.07	5.93	0.000632809	0.002652489	macro domain-containing protein
TGME49_228030	1.87	4.77	0.000634017	0.002655314	hypothetical protein
TGME49_297760	-7.99	5.12	0.000634561	0.002655314	hypothetical protein
TGME49_255160	-7.99	5.35	0.000635328	0.002657024	hypothetical protein
TGME49_260420	-1.77	6.17	0.000636048	0.00265853	HEC/Ndc80p family protein
TGME49_235420	-1.16	7.24	0.000638889	0.002668897	hypothetical protein

TGME49_268910	1.28	5.89	0.000639558	0.002670181	signal peptidase I protein
TGME49_215070	1.03	6.64	0.000643134	0.002683596	ferodoxin FD
TGME49_228980	-8.00	5.06	0.000647855	0.002701769	hypothetical protein
TGME49_268750	-3.35	5.37	0.000648414	0.00270258	peptidyl-prolyl cis-trans isomerase E, putative
TGME49_224810	-3.54	5.52	0.000665758	0.002771742	hypothetical protein
TGME49_264420	4.88	4.97	0.000667359	0.002776843	lipoprotein, putative
TGME49_232960	-1.60	6.61	0.000678323	0.002819292	oxidoreductase, 2OG-Fe(II) oxygenase family prote
TGME49_218370	-2.06	5.98	0.000684813	0.002842158	hypothetical protein
TGME49_294020	-7.99	5.25	0.000684978	0.002842158	zinc finger, C3HC4 type (RING finger) domain-cont
TGME49_321550	1.09	6.54	0.000689113	0.002857714	hypothetical protein
TGME49_249020	-2.07	6.04	0.000691183	0.002863819	kinesin motor domain-containing protein
TGME49_250500	-3.37	5.36	0.000691941	0.002864619	hypothetical protein
TGME49_226090	-3.34	5.05	0.000693128	0.002867929	DEAD/DEAH box helicase domain-containing prote
TGME49_234300	1.28	6.14	0.000695626	0.002873627	hypothetical protein
TGME49_218800	-8.01	4.77	0.000695671	0.002873627	hypothetical protein
TGME49_305920	-3.30	5.44	0.000698405	0.002883308	endonuclease III family 1 protein
TGME49_211470	1.56	5.17	0.000699421	0.002885892	Fcf2 pre-rRNA processing protein
TGME49_299015	-8.24	4.85	0.000702539	0.002897142	hypothetical protein
TGME49_213445	-1.70	7.01	0.000703053	0.002897644	hypothetical protein
TGME49_265380	-2.07	5.88	0.000704737	0.002901491	tetratricopeptide repeat (TPR)-/ U-box domain-co
TGME49_319570	-1.68	6.69	0.000711871	0.002925834	WD domain, G-beta repeat-containing protein
TGME49_213590	-1.24	6.96	0.000713157	0.002929457	hypothetical protein
TGME49_289910	-1.33	7.86	0.000716668	0.002940647	hypothetical protein
TGME49_215520	-1.24	6.78	0.000719164	0.002947614	hypothetical protein
TGME49_257170	-3.30	5.13	0.000725515	0.002970354	hypothetical protein
TGME49_228630	-1.22	7.14	0.000731057	0.002989025	hypothetical protein
TGME49_290640	-1.34	7.98	0.000731288	0.002989025	DNA mismatch repair protein MSH6-1, putative
TGME49_238410	-2.63	5.77	0.000731094	0.002989025	hypothetical protein
TGME49_215060	-1.26	7.25	0.000732477	0.002990575	small GTP-binding protein sar1, putative
TGME49_320460	-3.30	5.29	0.000732298	0.002990575	ABC transporter transmembrane region domain-co
TGME49_254200	-7.98	4.78	0.000735589	0.00299713	anticodon binding domain-containing protein
TGME49_203160	1.32	6.00	0.000736895	0.003000332	hypothetical protein
TGME49_247680	1.68	5.57	0.000751814	0.003056028	hypothetical protein
TGME49_282130	-2.28	5.72	0.000752302	0.003056332	hypothetical protein
TGME49_311150	-3.31	5.60	0.000754659	0.003062541	hypothetical protein
TGME49_222910	-1.50	6.45	0.000758058	0.003074646	phosphoglycerate mutase
TGME49_285272	-7.99	4.73	0.000759518	0.003077908	hypothetical protein
TGME49_281900	-1.14	7.26	0.000762017	0.003083936	SET domain containing lysine methyltransferase K
TGME49_288330	-3.30	5.36	0.000761907	0.003083936	histone lysine methyltransferase, SET, putative
TGME49_247930	-7.98	4.68	0.000766677	0.003101098	SNARE domain-containing protein
TGME49_226280	1.72	5.02	0.000771463	0.003117967	ribosomal protein L28, putative
TGME49_216750	1.04	6.92	0.000771691	0.003117967	Paf1/RNA polymerase II complex component LEO1
TGME49_271930	-1.16	8.59	0.000772394	0.003119103	hypothetical protein
TGME49_209210	-1.63	6.84	0.000778608	0.003142481	hypothetical protein
TGME49_301400	1.16	9.99	0.000784662	0.003165189	hypothetical protein
TGME49_312520	-3.33	5.37	0.000789269	0.003182037	tRNA dimethylallyltransferase
TGME49_305870	-2.64	5.28	0.000794487	0.003196101	DAD family protein
TGME49_226270	-2.66	5.82	0.00079401	0.003196101	hypothetical protein
TGME49_278230	-3.30	5.10	0.000795126	0.003196933	prenyltransferase and squalene oxidase repeat-co
TGME49_243200	-1.25	9.17	0.000797132	0.003201516	hypothetical protein
TGME49_289720	-1.65	6.27	0.000796708	0.003201516	hypothetical protein
TGME49_222240	-1.28	6.92	0.000798757	0.003204564	hypothetical protein
TGME49_294250	-1.87	6.17	0.000798599	0.003204564	WD domain, G-beta repeat-containing protein
TGME49_311625	-2.04	5.92	0.00080497	0.003227737	WD domain, G-beta repeat-containing protein

TGME49_323110	1.18	6.09	0.000807006	0.003234148	hypothetical protein
TGME49_278160	-3.34	5.32	0.000815731	0.003262041	vesicle transport v-snare protein
TGME49_305540	-1.56	6.49	0.000823147	0.00328992	hypothetical protein
TGME49_236630	-1.03	7.56	0.000827843	0.0033069	hypothetical protein
TGME49_285810	-7.92	4.79	0.000829788	0.00331288	MYND finger domain-containing protein
TGME49_290310	-2.04	5.87	0.000833073	0.003323778	hypothetical protein
TGME49_232680	1.24	6.24	0.000835118	0.003326972	hypothetical protein
TGME49_291590	-7.92	4.86	0.000836947	0.003332466	hypothetical protein
TGME49_295040	-1.04	7.94	0.000839069	0.003339114	HEAT repeat-containing protein
TGME49_251710	1.67	5.03	0.000841468	0.003345061	hypothetical protein
TGME49_278250	1.78	5.20	0.000844481	0.003353431	hypothetical protein
TGME49_270190	-2.73	5.63	0.000844109	0.003353431	protein phosphatase 2C domain-containing protei
TGME49_246130	1.18	8.21	0.000869336	0.00344715	serpin (serine proteinase inhibitor) superfamily pr
TGME49_211230	-1.40	6.73	0.00086948	0.00344715	eukaryotic initiation factor-2B, alpha subunit, puta
TGME49_258450	-1.18	6.92	0.000870351	0.003448755	hypothetical protein
TGME49_249890	1.63	5.11	0.000872453	0.003453383	hypothetical protein
TGME49_211640	1.08	6.52	0.000872266	0.003453383	hypothetical protein
TGME49_284540	-1.11	7.47	0.000873037	0.003453844	ATP synthase F1, delta subunit protein
TGME49_232750	-1.19	6.83	0.000878354	0.003473022	23S rRNA (adenine(1618)-N(6))-methyltransferase
TGME49_254660	1.09	6.94	0.000881476	0.003483503	ankyrin repeat-containing protein
TGME49_311220	-1.93	6.31	0.000886255	0.003500516	hypothetical protein
TGME49_216510	-3.26	5.85	0.000899896	0.003541155	thioredoxin, putative
TGME49_212940	-7.92	5.88	0.000899693	0.003541155	hypothetical protein
TGME49_201150	-1.56	6.72	0.000908296	0.003568159	heavy metal translocating P-type ATPase subfamily
TGME49_263440	-2.25	5.39	0.000908689	0.003568159	hypothetical protein
TGME49_260830	-2.63	5.42	0.000922566	0.003618806	hypothetical protein
TGME49_255960	-1.46	6.58	0.000930274	0.003643246	hypothetical protein
TGME49_210778	-1.73	6.36	0.000931389	0.003645678	hemimethylated DNA binding domain-containing
TGME49_313960	-3.31	5.10	0.000942469	0.003685148	ribosomal protein L19 protein
TGME49_208440	1.08	7.83	0.000949168	0.003707423	hypothetical protein
TGME49_267530	1.84	4.71	0.000949852	0.003708135	hypothetical protein
TGME49_246990	-2.60	5.31	0.000952628	0.003715048	hypothetical protein
TGME49_214380	1.51	5.41	0.00095736	0.003731533	hypothetical protein
TGME49_312660	-2.01	5.79	0.000964458	0.00375524	hypothetical protein
TGME49_269320	-7.92	5.04	0.000968371	0.003768493	hypothetical protein
TGME49_232000	1.02	8.49	0.000972524	0.003780674	hypothetical protein
TGME49_232970	-3.26	5.07	0.000975977	0.003790113	hypothetical protein
TGME49_281980	-1.28	6.99	0.000977942	0.003795751	phosphatidate cytidylyltransferase
TGME49_227050	-3.26	5.12	0.000978597	0.003796301	ATPase/histidine kinase/DNA gyrase B/HSP90 dom
TGME49_320005	-3.26	5.20	0.000980665	0.003802329	hypothetical protein
TGME49_297230	-1.72	6.39	0.00098414	0.003813799	Vps53 family, N-terminal protein
TGME49_243790	-1.85	5.96	0.00099251	0.003838191	SAG-related sequence SRS33
TGME49_269720	1.99	6.13	0.000998243	0.003856781	hypothetical protein
TGME49_207230	-1.74	5.74	0.000999732	0.00385812	hypothetical protein
TGME49_239700	-2.60	5.88	0.000999751	0.00385812	regulator of chromosome condensation (RCC1) re
TGME49_219170	-1.55	6.59	0.001000477	0.003858909	hypothetical protein
TGME49_228170	1.28	9.27	0.001001007	0.003858938	inner membrane complex protein IMC2A
TGME49_266810	-1.31	6.87	0.00100985	0.003886952	hypothetical protein
TGME49_305020	1.21	6.21	0.001011258	0.003890346	hypothetical protein
TGME49_309770	1.00	6.78	0.001021348	0.003923165	hypothetical protein
TGME49_271800	-1.50	6.27	0.001022442	0.003923165	serine esterase (DUF676) protein
TGME49_316240	-1.72	6.03	0.001021553	0.003923165	hypothetical protein
TGME49_202430	1.31	6.35	0.001032085	0.003954008	hypothetical protein
TGME49_214280	-7.97	4.76	0.001038254	0.003975582	phosphoadenosine phosphosulfate reductase fam

TGME49_289640	1.47	5.64	0.001042997	0.003991679	hypothetical protein
TGME49_288900	1.61	5.12	0.001050152	0.004014904	Yos1 family protein
TGME49_270595	-1.69	6.90	0.00105096	0.004015369	UBA/TS-N domain-containing protein
TGME49_203290	1.35	8.25	0.001052316	0.004016944	hypothetical protein
TGME49_283800	-1.89	6.25	0.001060654	0.004046686	hypothetical protein
TGME49_248550	-3.34	5.45	0.001063573	0.004055729	SPX domain-containing protein
TGME49_228060	-3.35	5.12	0.0010668	0.004065938	hypothetical protein
TGME49_205050	2.54	6.18	0.001075985	0.004098834	hypothetical protein
TGME49_211680	-1.20	11.04	0.001092618	0.004155769	protein disulfide isomerase
TGME49_275750	-1.65	6.27	0.001094296	0.004160014	small nuclear ribonucleoprotein E, putative
TGME49_208050	-2.27	5.98	0.001099128	0.004176234	ABC transporter, putative
TGME49_240900	1.01	6.85	0.001100381	0.004178847	AP2 domain transcription factor AP2VI-2
TGME49_204550	-2.60	5.62	0.001111712	0.004213222	hypothetical protein
TGME49_288620	1.02	6.86	0.001112521	0.004214131	Erv1 / Alr family protein
TGME49_209880	-1.09	7.37	0.001113752	0.004216633	glutamic acid-rcih protein, putative
TGME49_204410	-1.86	6.09	0.001115211	0.004219994	endonuclease/exonuclease/phosphatase family pr
TGME49_254900	-1.23	6.78	0.001118635	0.004230788	proteasome subunit beta type 2, putative
TGME49_219550	-1.08	7.18	0.001130178	0.004270074	dihydrolipoyllysine-residue succinyltransferase co
TGME49_233000	-1.28	7.06	0.001144426	0.004312889	KOW motif domain-containing protein
TGME49_202640	-7.85	4.76	0.001143603	0.004312889	RNA pseudouridine synthase superfamily protein
TGME49_220208	-7.86	4.78	0.001142739	0.004312889	hypothetical protein
TGME49_306950	-7.97	4.67	0.001143948	0.004312889	RAP domain-containing protein
TGME49_309390	-7.85	4.72	0.001153544	0.004336946	hypothetical protein
TGME49_277550	-7.86	4.87	0.001153743	0.004336946	UvrD/REP helicase domain-containing protein
TGME49_246780	-7.85	4.74	0.001158712	0.004351204	hypothetical protein
TGME49_299150	-7.85	5.15	0.001161849	0.004360766	AP2 domain transcription factor AP2III-3
TGME49_321640	-1.74	6.09	0.001163604	0.004365138	cell division protein CDC48AP
TGME49_298050	-1.82	5.70	0.001184036	0.004437284	hypothetical protein
TGME49_286740	2.43	5.13	0.001188454	0.004449332	microneme-like protein
TGME49_276990	-3.21	5.48	0.001192299	0.004461468	cytochrome b5 family heme/steroid binding doma
TGME49_232320	-7.85	4.97	0.001205034	0.004500138	hypothetical protein
TGME49_216530	-7.86	4.90	0.001205068	0.004500138	ribosome recycling factor protein
TGME49_288210	1.38	6.24	0.001214925	0.004534657	PUL domain-containing protein
TGME49_215380	-3.24	5.25	0.001229741	0.004580707	hypothetical protein
TGME49_281675	1.77	4.86	0.001233826	0.00458946	protein kinase, putative
TGME49_209740	1.57	5.20	0.001234503	0.00458946	hypothetical protein
TGME49_243910	1.61	6.24	0.001242161	0.004615346	Cof family hydrolase subfamily protein
TGME49_239250	1.05	7.39	0.001243774	0.004619019	diacylglycerol kinase, putative
TGME49_235402	-7.86	4.70	0.001252615	0.004649517	CorA family Mg2+ transporter protein
TGME49_269710	-1.12	7.22	0.001260956	0.004671097	hypothetical protein
TGME49_290960	-1.31	6.72	0.001260428	0.004671097	pyruvate phosphate dikinase, pep/pyruvate bindir
TGME49_247610	1.57	5.08	0.001262378	0.004674023	small nuclear ribonucleoprotein E, putative
TGME49_228000	1.14	6.36	0.00126429	0.00467876	splicing factor 3A subunit 2, putative
TGME49_321170	2.39	5.28	0.00126929	0.004694914	Toxoplasma gondii family C protein
TGME49_214190	1.61	5.29	0.001275498	0.004715515	SAG-related sequence SRS46
TGME49_310730	-1.38	6.50	0.001279295	0.00472247	hypothetical protein
TGME49_323100	1.22	6.67	0.001297135	0.004785935	hypothetical protein
TGME49_288045	-3.25	5.31	0.001301384	0.004799218	hypothetical protein
TGME49_224620	1.22	5.92	0.00130666	0.004815647	hypothetical protein
TGME49_297360	-1.55	6.33	0.001307141	0.004815647	hypothetical protein
TGME49_280590	-3.28	4.77	0.001310937	0.004827224	hypothetical protein
TGME49_242110	-7.85	4.87	0.001327888	0.004882348	rhoptry kinase family protein ROP38
TGME49_268000	-2.56	5.44	0.001333211	0.004897051	hypothetical protein
TGME49_263130	1.16	8.10	0.001336879	0.004908086	citrate synthase, putative

TGME49_284050	-1.53	6.47	0.001365252	0.005007277	DEAD/DEAH box helicase domain-containing prote
TGME49_275568	-1.53	6.27	0.001368067	0.005015116	GPI transamidase subunit PIG-U protein
TGME49_203390	-1.21	7.14	0.00137194	0.005026822	CRAL/TRIO domain-containing protein
TGME49_260310	1.50	5.29	0.001385808	0.005075118	ATP-binding cassette transporter ABC.B1
TGME49_244130	-1.50	6.36	0.001419492	0.005183074	hypothetical protein
TGME49_286640	-3.20	4.82	0.00143133	0.005223719	GTPase
TGME49_218960	-1.52	8.73	0.00143904	0.005246676	AP2 domain transcription factor AP2XII-1
TGME49_273500	-2.28	5.42	0.001449593	0.005279947	O-linked N-acetylglucosamine transferase
TGME49_210380	-1.33	6.73	0.001453893	0.005290398	hypothetical protein
TGME49_266110	1.48	5.29	0.001457129	0.005299562	DEAD/DEAH box helicase domain-containing prote
TGME49_235700	-1.43	6.40	0.001458855	0.005303232	sedoheptulose-1,7-bisphosphatase
TGME49_204400	-1.08	8.95	0.001461224	0.005309234	ATPase synthase subunit alpha, putative
TGME49_298020	-1.25	6.88	0.001464292	0.005317767	DEAD-family helicase
TGME49_262120	-1.20	7.00	0.001467338	0.005326214	IQ calmodulin-binding motif domain-containing pr
TGME49_310802	1.17	5.93	0.001474044	0.005342936	CRAL/TRIO domain-containing protein
TGME49_233680	-7.78	5.02	0.001474113	0.005342936	nuclear movement family protein
TGME49_223125	-1.80	6.12	0.001476835	0.005350181	ubiquitin family protein
TGME49_241840	1.44	6.62	0.001489043	0.005377601	hypothetical protein
TGME49_269250	-1.11	7.17	0.001488295	0.005377601	Mov34/MPN/PAD-1 family protein
TGME49_211410	-3.17	5.74	0.001487967	0.005377601	translation initiation factor sui1 protein
TGME49_301300	-7.78	4.71	0.001486325	0.005377601	hypothetical protein
TGME49_314460	-7.78	4.78	0.001488758	0.005377601	hypothetical protein
TGME49_278770	-1.17	7.22	0.001499134	0.005404483	hypothetical protein
TGME49_307800	1.62	6.04	0.001502457	0.005413824	GDA1/CD39 (nucleoside phosphatase) family prot
TGME49_218280	-1.01	7.67	0.001510625	0.005438002	eukaryotic porin, putative
TGME49_259000	-7.78	4.76	0.001510638	0.005438002	hypothetical protein
TGME49_237110	-1.57	6.30	0.001518802	0.005462073	replication factor C subunit 2, putative
TGME49_261940	-7.78	4.88	0.001521181	0.00546797	hydrolase, alpha/beta fold family protein
TGME49_263230	-1.19	6.78	0.001527089	0.005486539	hypothetical protein
TGME49_223560	-2.58	5.14	0.001538308	0.0055188	hypothetical protein
TGME49_263210	-7.78	5.06	0.001543755	0.005532972	ubiquitin interaction motif domain-containing pro
TGME49_259650	-3.21	4.78	0.001545643	0.005537056	hypothetical protein
TGME49_252490	-1.96	5.95	0.001569464	0.00561423	vacuolar protein sorting 29, putative
TGME49_261780	1.11	8.77	0.00157895	0.00563998	microneme protein MIC7
TGME49_254650	1.46	5.56	0.001586504	0.005663464	zinc finger protein
TGME49_256090	-7.77	4.73	0.001587057	0.005663464	glycerophosphodiester phosphodiesterase family
TGME49_228460	-1.69	6.12	0.001594839	0.005688488	hypothetical protein
TGME49_300130	1.76	4.71	0.001616039	0.005753006	apical membrane antigen 1 domain-containing pro
TGME49_271430	-3.16	5.38	0.00162016	0.005763851	hypothetical protein
TGME49_253800	-3.20	5.03	0.001620645	0.005763851	ribosomal protein L15, putative
TGME49_230150	1.31	6.16	0.001659664	0.005885638	ChAPs (Chs5p-Arf1p-binding proteins) protein
TGME49_277870	-1.80	6.10	0.001662373	0.005886775	hypothetical protein
TGME49_288930	-1.97	6.16	0.001661836	0.005886775	hypothetical protein
TGME49_222410	-1.36	6.72	0.001673509	0.005923373	hypothetical protein
TGME49_210960	-1.52	6.30	0.001676596	0.00593146	replication factor C subunit 4, putative
TGME49_225300	-1.58	6.43	0.001681366	0.00594549	hypothetical protein
TGME49_207160	-1.95	5.87	0.001691343	0.005977912	SAG-related sequence SRS49D
TGME49_248445	-1.12	6.86	0.001697388	0.005990687	hypothetical protein
TGME49_236140	1.78	4.81	0.001713369	0.006038442	hypothetical protein
TGME49_290840	-1.41	6.35	0.001722913	0.006069185	serine protease
TGME49_230860	-1.96	6.09	0.00173639	0.006113747	hypothetical protein
TGME49_230050	1.49	4.96	0.00176377	0.006202329	50S ribosomal protein L3, putative
TGME49_248400	-1.77	6.09	0.001764065	0.006202329	glyoxalase I, putative
TGME49_254940	1.01	9.51	0.001770536	0.00622033	MIF4G domain-containing protein

TGME49_269800	-2.16	5.58	0.001774158	0.006226508	glutamine-dependent NAD(+) synthetase protein,
TGME49_205720	-7.77	4.68	0.001774311	0.006226508	Adenosine/AMP deaminase domain-containing pr
TGME49_221905	-1.03	7.08	0.001780143	0.00624401	hypothetical protein
TGME49_311380	-3.43	5.26	0.001788531	0.00627046	hypothetical protein
TGME49_218955	1.43	5.19	0.001796632	0.006292892	hypothetical protein
TGME49_225910	1.16	5.95	0.001809118	0.006333627	hypothetical protein
TGME49_283830	1.91	5.33	0.001811521	0.00633818	type I inorganic pyrophosphatase PPase
TGME49_213370	1.08	6.46	0.001812133	0.00633818	formin FRM3
TGME49_202230	-1.55	5.96	0.001815013	0.006342252	histone deacetylase HDAC5
TGME49_217560	1.08	6.35	0.001824468	0.006369272	DNA-directed RNA polymerase II RPB10
TGME49_257755	1.96	5.37	0.001829507	0.006383849	hypothetical protein
TGME49_266320	1.01	6.57	0.001850071	0.006452559	hypothetical protein
TGME49_309070	1.76	5.61	0.001853603	0.00646183	hypothetical protein
TGME49_234470	-1.77	5.77	0.001859246	0.006477202	hypothetical protein
TGME49_258380	-2.16	5.55	0.001859765	0.006477202	elongation factor p (ef-p) kow family domain-cont
TGME49_289510	1.23	5.65	0.001862925	0.006483085	hypothetical protein
TGME49_202350	-1.77	5.81	0.001863207	0.006483085	50S ribosomal protein L21, putative
TGME49_253540	1.43	5.28	0.00188764	0.006564679	hypothetical protein
TGME49_215030	-1.93	5.85	0.001888433	0.006564679	hypothetical protein
TGME49_212800	-1.42	6.53	0.001889685	0.006565945	hypothetical protein
TGME49_248630	-1.49	6.51	0.00189086	0.006566941	actin-related protein ARP1
TGME49_264450	-1.93	5.87	0.001907195	0.006620565	DNA topoisomerase III beta-1, putative
TGME49_217450	1.47	4.96	0.001925071	0.006679482	general transcription factor IIH polypeptide 5 GTF
TGME49_213890	-7.79	5.03	0.001940931	0.006731354	Myb family DNA-binding domain-containing prote
TGME49_293060	-1.68	6.24	0.001948743	0.006752112	SPRY domain-containing protein
TGME49_276190	-2.20	5.61	0.001969981	0.006816117	hypothetical protein
TGME49_286180	-7.70	4.90	0.001971246	0.006817302	tRNA ligases class I (M) protein
TGME49_239300	-1.55	6.35	0.001978754	0.00683368	hypothetical protein
TGME49_273595	-1.66	6.00	0.001984978	0.006851972	hypothetical protein
TGME49_278170	-2.17	5.42	0.001993541	0.006878322	hypothetical protein
TGME49_294360	-1.32	6.51	0.002004324	0.00690586	ubiquitin specific protease 39 isoform 2, putative
TGME49_309920	-3.12	5.24	0.002009513	0.006920513	hypothetical protein
TGME49_311740	-2.16	5.52	0.002012726	0.006925126	hypothetical protein
TGME49_288710	1.43	5.08	0.00201376	0.00692546	hypothetical protein
TGME49_230830	1.48	5.63	0.002026905	0.006957722	ATPase family associated with various cellular acti
TGME49_265070	-2.50	5.58	0.002026691	0.006957722	hypothetical protein
TGME49_227860	-7.70	4.75	0.002025411	0.006957722	hypothetical protein
TGME49_248110	1.24	7.91	0.002035391	0.006980371	hypothetical protein
TGME49_297690	1.18	6.06	0.002046978	0.007013933	hypothetical protein
TGME49_209950	-1.12	9.03	0.002054176	0.007035007	thioredoxin, putative
TGME49_244570	-7.71	4.81	0.002057023	0.007036941	hypothetical protein
TGME49_270760	-7.72	4.79	0.002056775	0.007036941	asparagine synthase
TGME49_209120	1.31	6.06	0.002059735	0.007040998	hypothetical protein
TGME49_309880	-1.33	6.92	0.002063303	0.007049935	hypothetical protein
TGME49_265460	1.09	6.01	0.002070054	0.007066471	hypothetical protein
TGME49_263505	-3.14	5.15	0.002072431	0.007071322	hypothetical protein
TGME49_294060	-1.05	7.47	0.002088546	0.007123019	hypothetical protein
TGME49_249870	-1.27	6.54	0.002091526	0.00712661	hypothetical protein
TGME49_271240	-2.48	5.16	0.002112405	0.007194437	hypothetical protein
TGME49_271990	1.28	6.11	0.002122379	0.007221755	hypothetical protein
TGME49_242810	-3.14	5.22	0.002121873	0.007221755	hypothetical protein
TGME49_280450	-7.69	4.79	0.002130667	0.00724662	hypothetical protein
TGME49_288860	-2.73	6.98	0.002141373	0.007272994	RuvB family 2 protein
TGME49_214990	-1.75	5.69	0.002146899	0.007285073	hypothetical protein

TGME49_221390	-1.75	5.94	0.002157904	0.007315701	hypothetical protein
TGME49_305800	-1.75	5.93	0.002168015	0.007343246	6-pyruvoyl tetrahydrobiopterin synthase
TGME49_294730	-1.50	6.29	0.002181819	0.007386616	hypothetical protein
TGME49_263480	-1.46	6.10	0.002182983	0.007387174	sodium/hydrogen exchanger 3 protein
TGME49_201800	-1.16	7.11	0.002198943	0.007430982	hypothetical protein
TGME49_269770	-2.48	5.57	0.00220892	0.007461286	WD domain, G-beta repeat-containing protein
TGME49_213040	-1.54	6.26	0.002222466	0.007503613	hypothetical protein
TGME49_308090	1.09	10.07	0.002230279	0.007526556	rhoptry protein ROP5
TGME49_235880	1.02	7.78	0.002232383	0.00753022	brain protein 44 family protein
TGME49_258970	1.01	6.74	0.002245838	0.007568699	hypothetical protein
TGME49_286270	-1.54	6.58	0.002260753	0.007615493	hypothetical protein
TGME49_263190	-3.12	4.74	0.002274312	0.007654193	adenylosuccinate lyase, putative
TGME49_227300	1.82	4.84	0.002296158	0.007720688	hypothetical protein
TGME49_304750	-2.13	5.37	0.002317787	0.007789872	zinc finger, C3HC4 type (RING finger) domain-cont
TGME49_232630	-1.10	8.33	0.002325238	0.007809889	hypothetical protein
TGME49_301170	-7.68	5.92	0.002346722	0.007869235	SAG-related sequence SRS19D
TGME49_220440	-1.93	5.93	0.002368574	0.007935313	cyclin-dependent kinase regulatory subunit protei
TGME49_265650	-3.11	5.20	0.002411592	0.008064817	protein phosphatase 2C domain-containing protei
TGME49_234420	-1.37	6.17	0.002427592	0.008107323	ATPase, AAA family protein
TGME49_214400	-2.45	5.11	0.002426676	0.008107323	hypothetical protein
TGME49_272730	1.81	4.78	0.002438223	0.008134155	hypothetical protein
TGME49_245710	-1.15	6.93	0.002439462	0.008134155	phosphatidylinositol-4-phosphate 5-kinase, putati
TGME49_208722	-3.11	5.29	0.002440026	0.008134155	hypothetical protein
TGME49_217760	1.21	5.84	0.002453705	0.008168706	GTP-binding protein
TGME49_231930	-3.15	4.73	0.0024809	0.008251811	hypothetical protein
TGME49_214530	-1.60	6.38	0.002492353	0.008286176	DnaJ domain-containing protein
TGME49_225110	1.03	6.39	0.002495409	0.008292609	AP2 domain transcription factor AP2X-2
TGME49_216580	-1.64	6.03	0.002505245	0.008321555	hypothetical protein
TGME49_248370	1.14	5.93	0.002513546	0.008345379	prefoldin subunit 6, putative
TGME49_259600	-1.75	5.75	0.002527706	0.008388627	hypothetical protein
TGME49_254610	1.77	5.99	0.002531818	0.008398503	Tim10/DDP family zinc finger superfamily protein
TGME49_223480	-7.62	5.77	0.002579972	0.008542907	sushi domain (scr repeat) domain-containing prote
TGME49_319720	-3.13	5.17	0.002584781	0.008555002	hypothetical protein
TGME49_258930	-2.46	4.82	0.002586214	0.008555914	peptidylprolyl isomerase
TGME49_316520	-1.37	6.34	0.002587949	0.008557824	1,4-alpha-glucan-branching enzyme
TGME49_209170	-1.18	6.83	0.002600201	0.008582986	hypothetical protein
TGME49_329800	-1.37	6.84	0.002616519	0.008629146	hypothetical protein
TGME49_270370	-2.18	5.67	0.002617893	0.008629826	clathrin assembly protein AP19, putative
TGME49_218230	-1.72	5.66	0.002627376	0.008657226	histone lysine methyltransferase, SET, putative
TGME49_220330	-3.14	5.33	0.00262972	0.00866109	hypothetical protein
TGME49_212930	1.81	5.60	0.002630913	0.008661163	NifU family domain-containing protein
TGME49_291690	-3.07	5.09	0.00263256	0.008662727	hypothetical protein
TGME49_200430	-7.63	4.89	0.00265129	0.00872048	cytidine and deoxycytidylate deaminase zinc-bindi
TGME49_208070	-1.14	6.77	0.002662095	0.008742327	inositol polyphosphate kinase
TGME49_301310	1.75	6.11	0.002667365	0.008753884	hypothetical protein
TGME49_266800	-1.61	6.24	0.002682046	0.008790357	integral membrane protein, putative
TGME49_224670	-1.87	5.88	0.002690307	0.008813528	DnaJ domain-containing protein
TGME49_245650	1.82	4.74	0.002712499	0.008870508	hypothetical protein
TGME49_294190	-3.07	5.22	0.002717812	0.008883953	enoyl-CoA hydratase/isomerase family protein
TGME49_225920	-2.09	5.83	0.002719652	0.008886039	hypothetical protein
TGME49_232210	-7.62	5.03	0.002756624	0.008994916	hypothetical protein
TGME49_247670	-2.15	5.41	0.002796323	0.009108375	ribulose-phosphate 3 epimerase family protein
TGME49_263730	-1.72	6.40	0.002803162	0.009122616	FAD-dependent glycerol-3-phosphate dehydrogen
TGME49_232270	-7.62	5.79	0.002803032	0.009122616	histidine acid phosphatase superfamily protein

TGME49_315670	-1.18	6.74	0.002808028	0.009125733	HEAT repeat-containing protein
TGME49_262000	-3.11	4.73	0.002809267	0.009125733	AP2 domain transcription factor AP2VIIb-2
TGME49_273550	-3.06	5.26	0.002848761	0.009238482	hypothetical protein
TGME49_249600	-7.61	4.74	0.002848736	0.009238482	hypothetical protein
TGME49_232530	-2.09	5.46	0.002857213	0.00926183	hypothetical protein
TGME49_309780	-1.87	5.56	0.002858714	0.009262633	hypothetical protein
TGME49_286010	1.27	5.83	0.002893716	0.009367837	hypothetical protein
TGME49_218830	1.45	5.33	0.002926849	0.009458531	hypothetical protein
TGME49_281450	-2.45	5.45	0.002938536	0.009479953	cell-cycle-associated protein kinase, putative
TGME49_222160	-3.06	4.97	0.002934804	0.009479953	aldehyde dehydrogenase
TGME49_233540	-3.16	5.15	0.002938015	0.009479953	transporter, major facilitator family protein
TGME49_252400	2.27	5.14	0.002944274	0.009489956	HIT zinc finger protein
TGME49_221170	-1.08	6.99	0.002943143	0.009489956	CAAX metallo endopeptidase
TGME49_238940	1.86	4.73	0.002946499	0.00949299	GDP mannose 4,6-dehydratase, putative
TGME49_224490	-3.09	5.05	0.002955621	0.009518232	polyprenyl synthetase superfamily protein
TGME49_287980	-1.59	6.10	0.002964318	0.009542086	FHA domain-containing protein
TGME49_251920	1.40	5.05	0.002976395	0.009576789	hypothetical protein
TGME49_211650	-1.30	6.23	0.002993362	0.009627193	hypothetical protein
TGME49_251620	1.03	6.69	0.003010078	0.009676747	flap structure-specific endonuclease 1, putative
TGME49_221922	-3.06	5.27	0.003044027	0.009777383	NifU family domain-containing protein
TGME49_272550	-1.17	6.75	0.003048418	0.009787236	hypothetical protein
TGME49_271440	-2.41	5.43	0.003054959	0.009803979	NPL4 family protein
TGME49_272710	1.08	7.14	0.00307878	0.009876139	AP2 domain transcription factor AP2VIII-4
TGME49_297330	1.28	5.60	0.003162558	0.01013906	hypothetical protein
TGME49_288460	-1.07	7.02	0.003165874	0.01014232	hypothetical protein
TGME49_293310	1.04	6.76	0.00319676	0.0102324	ribosomal protein L20, putative
TGME49_258650	-2.41	5.11	0.003207778	0.010258783	protoheme ferro-lyase, putative
TGME49_252270	1.74	6.10	0.003216579	0.010278042	L1P family of ribosomal protein
TGME49_314030	1.88	5.75	0.003261487	0.010399068	hypothetical protein
TGME49_206300	-2.41	5.15	0.003260272	0.010399068	hypothetical protein
TGME49_315360	-1.30	6.61	0.003281684	0.010454448	hypothetical protein
TGME49_258990	-2.40	5.52	0.003283765	0.010456571	bromodomain-containing protein
TGME49_249380	1.18	7.53	0.003321855	0.010573308	DHHC zinc finger domain-containing protein
TGME49_257990	-1.24	6.77	0.003327257	0.010585946	heat shock protein 101, putative
TGME49_269075	-7.64	5.19	0.003335476	0.010607532	hypothetical protein
TGME49_202730	-2.08	5.45	0.003362422	0.010678497	hypothetical protein
TGME49_246978	-3.01	5.07	0.003365774	0.010680914	hypothetical protein
TGME49_312220	-3.02	5.27	0.003384403	0.010735426	mitochondrial inner membrane translocase subun
TGME49_250090	1.08	6.15	0.003398472	0.01077543	hypothetical protein
TGME49_221460	-1.18	6.68	0.003440236	0.010889172	phosphoglycerate mutase family protein
TGME49_243390	-2.40	5.53	0.003448703	0.0109113	hypothetical protein
TGME49_272720	-1.85	5.62	0.003467454	0.010965935	methyltransferase domain-containing protein
TGME49_288010	1.77	5.10	0.003481067	0.011004278	hypothetical protein
TGME49_246040	-1.56	6.20	0.003483395	0.011006934	MIF4G domain-containing protein
TGME49_240710	-1.49	6.51	0.003505916	0.01106206	RNA recognition motif-containing protein
TGME49_274000	-2.06	6.01	0.003506826	0.01106206	hypothetical protein
TGME49_240800	-1.41	6.86	0.00351998	0.011084638	MORN repeat-containing protein
TGME49_264820	1.31	6.38	0.003525905	0.011093846	RbAp48
TGME49_243350	1.47	6.80	0.003529872	0.011098977	gamma-glutamyl hydrolase
TGME49_276130	1.13	6.47	0.003536712	0.011104329	cathepsin CPC2
TGME49_246690	-2.05	5.40	0.003538247	0.011104329	alpha amylase, catalytic domain-containing protei
TGME49_308940	-7.53	4.89	0.00353605	0.011104329	hypothetical protein
TGME49_209140	-1.16	6.82	0.003543448	0.011115932	anti-silencing protein, ASF1 family protein
TGME49_313300	1.14	5.75	0.003571872	0.011200347	YL1 nuclear protein C-terminal domain-containing

TGME49_263150	-7.53	4.96	0.00365051	0.011417882	tetratricopeptide repeat-containing protein
TGME49_227640	-1.39	6.07	0.003669526	0.011467658	hypothetical protein
TGME49_231940	-1.43	6.36	0.003704096	0.011570801	ThiF family protein
TGME49_300010	-7.54	4.88	0.003716226	0.011589109	hypothetical protein
TGME49_249850	-1.57	10.25	0.003739433	0.01165165	GAP40 protein
TGME49_266930	1.35	5.42	0.003750203	0.011677001	general transcription factor IIH polypeptide 3 GTF
TGME49_253820	1.02	8.07	0.003823493	0.011873543	hypothetical protein
TGME49_229720	-2.36	5.39	0.003826714	0.011878559	hypothetical protein
TGME49_270330	1.28	6.26	0.003835786	0.011886758	cell-cycle-associated protein kinase, putative
TGME49_312360	-3.00	4.90	0.003878168	0.012013061	hypothetical protein
TGME49_311260	-1.07	6.78	0.003889335	0.012042606	myosin light chain MLC1, putative
TGME49_277090	2.01	7.62	0.003900406	0.012071831	carrier superfamily protein
TGME49_277010	-7.53	4.90	0.003903771	0.012077189	Fe-S metabolism associated domain-containing pr
TGME49_260840	-3.00	4.88	0.003914153	0.012104244	hypothetical protein
TGME49_236580	-1.27	7.51	0.003966693	0.012256467	Prp31-15.5k-U4 Snrna Complex family protein
TGME49_256920	1.51	6.24	0.004090557	0.012618098	phosphatidylinositol-4-phosphate 5-Kinase
TGME49_241170	-1.12	6.88	0.004119042	0.012684796	hypothetical protein
TGME49_248200	1.30	6.31	0.004124083	0.012695035	ribosomal RNA (adenine(1779)-N(6)/adenine(178
TGME49_226420	-1.21	6.96	0.004164352	0.012813658	peptidase family M3 protein
TGME49_221870	1.26	7.14	0.004180046	0.012856597	hypothetical protein
TGME49_202480	-2.96	5.11	0.00418808	0.012875953	hypothetical protein
TGME49_228380	3.13	5.03	0.004217236	0.012954817	hypothetical protein
TGME49_305190	1.02	6.71	0.004233208	0.012987694	CorA family Mg2+ transporter protein
TGME49_213870	-1.14	6.92	0.004231693	0.012987694	UBA/TS-N domain-containing protein
TGME49_311750	1.96	5.91	0.004237632	0.012990485	mago binding protein
TGME49_220160	-2.96	5.04	0.00423701	0.012990485	WD domain-containing protein
TGME49_235610	-2.96	5.08	0.004252331	0.013030143	ATPase, AAA family protein
TGME49_280522	-1.16	6.97	0.004263687	0.013059529	hypothetical protein
TGME49_244860	1.40	5.15	0.00426761	0.013066132	hypothetical protein
TGME49_315640	1.32	5.20	0.004294912	0.013144281	lipoyl(octanoyl) transferase
TGME49_294860	-1.54	6.13	0.004297176	0.013145768	hypothetical protein
TGME49_215480	-1.81	5.60	0.004330572	0.013242451	Adenosine/AMP deaminase domain-containing pr
TGME49_285880	1.58	4.94	0.004359429	0.013319676	hypothetical protein
TGME49_260150	1.60	4.92	0.004395175	0.013417806	tetratricopeptide repeat-containing protein
TGME49_314530	-3.00	5.09	0.004431562	0.013517727	RPAP1 family, C-terminal protein
TGME49_277820	-2.36	5.23	0.004433629	0.013518455	hypothetical protein
TGME49_310660	-2.96	5.16	0.004439235	0.013529967	Dullard family phosphatase domain-containing pr
TGME49_250060	1.45	5.19	0.004442399	0.013534033	DNA-directed RNA polymerase I RPA12
TGME49_306020	-1.44	6.20	0.004449178	0.013549102	hypothetical protein
TGME49_319710	-1.38	6.22	0.004454468	0.013559629	kinesin motor domain-containing protein
TGME49_205240	-1.13	6.70	0.004457099	0.013562053	cleft lip and palate transmembrane protein 1 (clpt
TGME49_203710	-1.14	8.63	0.004475509	0.013595692	AP2 domain transcription factor AP2VIIa-4
TGME49_285700	-2.99	4.70	0.004475298	0.013595692	ubiquitin fusion degradation protein UFD1AP
TGME49_306510	1.91	5.11	0.004478603	0.013599505	hypothetical protein
TGME49_204100	-1.27	6.50	0.004481417	0.013602461	eIF2 kinase IF2K-C
TGME49_271170	-2.95	5.26	0.004485651	0.013609727	dolichol kinase
TGME49_244140	-3.04	4.78	0.004499581	0.013646392	hypothetical protein
TGME49_319930	-1.25	6.40	0.004570028	0.013831676	hypothetical protein
TGME49_277490	1.04	7.63	0.004577985	0.013850091	hypothetical protein
TGME49_219750	1.10	8.48	0.004619953	0.013965629	cytochrome c, putative
TGME49_315190	-1.06	6.83	0.004618999	0.013965629	CAM kinase, SNF1 family
TGME49_226060	1.15	6.99	0.004639471	0.0140189	transmembrane amino acid transporter protein
TGME49_311390	1.35	5.11	0.004647562	0.014037612	tRNA (guanine(9)-N(1))-methyltransferase
TGME49_235905	-2.32	5.09	0.004689275	0.014140496	ribonuclease z, putative

TGME49_319320	-1.38	6.45	0.004696964	0.014157908	hypothetical protein
TGME49_270670	1.17	5.21	0.004716237	0.014204422	hypothetical protein
TGME49_240430	1.18	5.61	0.004724828	0.014218715	glyoxalase family protein
TGME49_315920	-2.01	5.33	0.004724417	0.014218715	DNA-directed RNA polymerase II RPB11A
TGME49_310210	1.03	6.10	0.004735919	0.014246291	hypothetical protein
TGME49_203050	1.92	6.44	0.004781122	0.014358903	AP2 domain transcription factor AP2VIIa-6
TGME49_237195	1.68	4.95	0.004808671	0.014412368	hypothetical protein
TGME49_320690	-1.64	5.76	0.004820425	0.014441742	gamma-soluble NSF attachment protein, putative
TGME49_309940	-1.54	5.91	0.004832276	0.014465526	phospholipase D active site domain-containing pro
TGME49_246178	-1.78	5.82	0.004843127	0.01449214	hypothetical protein
TGME49_212100	-7.44	4.92	0.004856505	0.014504076	ThiF family protein
TGME49_203480	-2.97	5.11	0.004873471	0.014547616	hypothetical protein
TGME49_295460	1.07	6.00	0.004893183	0.014588789	Got1 family protein
TGME49_291350	-2.95	5.02	0.004953377	0.014756355	hypothetical protein
TGME49_240090	1.51	8.58	0.004957372	0.014761299	rhoptry kinase family protein ROP34, putative
TGME49_257960	-2.32	5.69	0.00495903	0.014761299	GDP-D-mannose pyrophosphorylase
TGME49_315910	1.03	5.86	0.004987574	0.014840292	hypothetical protein
TGME49_221518	-1.84	5.23	0.004993453	0.014851806	hypothetical protein
TGME49_242320	-1.23	6.85	0.005192466	0.015374979	B-box zinc finger domain-containing protein
TGME49_295980	-1.65	5.98	0.005194306	0.015374979	hypothetical protein
TGME49_215940	-1.29	6.63	0.005211276	0.015419038	Acetyl-coenzyme A transporter, putative
TGME49_249270	-1.19	9.01	0.005218543	0.015434364	protein disulfide isomerase-related protein (provis
TGME49_266280	-1.08	6.59	0.005266194	0.015569067	HEAT repeat-containing protein
TGME49_252065	1.65	5.06	0.005268968	0.015571042	KRUF family protein
TGME49_271010	1.80	5.05	0.005329856	0.015725839	hypothetical protein
TGME49_226400	-1.41	6.16	0.005347966	0.015772979	lipoic acid synthase LIPA
TGME49_314890	-2.90	5.41	0.005350293	0.01577355	ThiF family protein
TGME49_222120	-2.90	5.09	0.005436011	0.016007116	hypothetical protein
TGME49_294750	-2.90	4.76	0.005441281	0.016016253	hypothetical protein
TGME49_251510	1.45	6.13	0.005452083	0.016040002	Ulp1 protease family, C-terminal catalytic domain-
TGME49_313690	-1.22	6.61	0.005483667	0.016115355	Sel1 repeat-containing protein
TGME49_267700	-2.92	5.07	0.005502309	0.016163715	hypothetical protein
TGME49_248340	-1.04	9.21	0.005516242	0.01619821	GTP-binding nuclear protein ran/tc4
TGME49_227260	-2.31	5.33	0.005605407	0.016440451	RIO1 family protein
TGME49_255300	-1.33	6.62	0.005633216	0.016508917	hypothetical protein
TGME49_259080	1.89	6.29	0.005653595	0.016555519	hypothetical protein
TGME49_229030	-1.17	6.70	0.005717908	0.016730594	hypothetical protein
TGME49_233120	-1.37	6.11	0.005748021	0.016798761	AP2 domain transcription factor AP2VIII-2
TGME49_298980	-1.21	6.23	0.005799085	0.016941301	RNA pseudouridine synthase superfamily protein
TGME49_275770	-1.32	6.31	0.005826632	0.017015053	hypothetical protein
TGME49_267400	-1.04	9.30	0.005844023	0.017059103	ribosomal protein RPL32
TGME49_286130	1.59	4.91	0.005857133	0.017090625	hypothetical protein
TGME49_316480	1.69	5.01	0.00587959	0.017142623	XRN 5'-3' exonuclease N-terminus protein
TGME49_208390	1.29	6.10	0.005894407	0.017176051	hypothetical protein
TGME49_259720	1.14	5.94	0.005895702	0.017176051	hypothetical protein
TGME49_225420	-2.28	5.08	0.005936323	0.017280776	histidine triad domain-containing protein
TGME49_281420	-2.28	5.07	0.005950246	0.01731449	histone deacetylase HDAC1
TGME49_270530	-2.02	5.98	0.005986492	0.017413109	ubiquitin fusion degradation protein UFD1CY
TGME49_246630	-1.49	5.95	0.005989617	0.017415346	DNA-directed RNA polymerase I RPA43
TGME49_253960	1.52	4.75	0.005996147	0.017427481	oxidoreductase, short chain dehydrogenase/reduc
TGME49_274150	-2.90	5.45	0.00599854	0.017427584	hypothetical protein
TGME49_246010	2.82	5.31	0.006027986	0.017506255	hypothetical protein
TGME49_297170	1.44	5.17	0.006039088	0.017531613	50S ribosomal protein L17, putative
TGME49_290225	-2.90	5.04	0.006096369	0.017684013	hypothetical protein

TGME49_279390	-1.25	9.03	0.006108944	0.017707707	proliferation-associated protein 2G4, putative
TGME49_231040	-2.91	4.79	0.006111463	0.017707707	3' exoribonuclease family, domain 1 domain-conta
TGME49_263470	-1.17	6.31	0.006259682	0.018093858	ubiquitin carboxyl-terminal hydrolase UCHL3
TGME49_242625	-1.29	6.49	0.006266223	0.018105687	ATPase family associated with various cellular acti
TGME49_267040	-2.28	5.35	0.006294781	0.018181099	hypothetical protein
TGME49_254606	1.64	5.92	0.006314744	0.018231634	hypothetical protein
TGME49_270975	-1.68	5.71	0.006361291	0.018344535	hypothetical protein
TGME49_284598	-7.35	5.10	0.006360845	0.018344535	haloacid dehalogenase family hydrolase domain-c
TGME49_269705	1.40	5.10	0.006424869	0.01846371	hypothetical protein
TGME49_299030	1.22	7.26	0.006426229	0.01846371	RNA recognition motif 2 protein
TGME49_206415	-1.19	6.70	0.006419892	0.01846371	myosin K
TGME49_237840	-1.61	5.52	0.006424122	0.01846371	hypothetical protein
TGME49_307260	1.81	5.67	0.006449425	0.018483351	Toxoplasma gondii family C protein
TGME49_226040	-1.13	6.54	0.006437648	0.018483351	EF hand domain-containing protein
TGME49_286190	-1.20	6.28	0.006447529	0.018483351	hypothetical protein
TGME49_222870	-1.34	6.27	0.006447988	0.018483351	hypothetical protein
TGME49_277540	-7.34	4.89	0.006442971	0.018483351	hypothetical protein
TGME49_254510	1.59	4.84	0.006483861	0.018560461	ankyrin repeat-containing protein
TGME49_245770	2.70	6.52	0.00655166	0.01873335	hypothetical protein
TGME49_305820	-1.48	5.90	0.006574794	0.018777145	SGS domain-containing protein
TGME49_305620	-1.52	5.96	0.006573046	0.018777145	hypothetical protein
TGME49_278890	-1.97	5.41	0.006588917	0.018802953	hypothetical protein
TGME49_246050	-7.42	4.73	0.006644524	0.018932412	hypothetical protein
TGME49_260270	-1.44	6.12	0.006667192	0.018982372	HEAT repeat-containing protein
TGME49_213020	1.58	5.21	0.006721444	0.019114753	hypothetical protein
TGME49_220530	1.68	5.63	0.00677703	0.019245085	AP2 domain transcription factor AP2V-1
TGME49_209190	-1.16	6.40	0.006812193	0.019320811	ABC transporter transmembrane region domain-co
TGME49_277850	-1.88	5.67	0.006830183	0.019356984	trypsin domain-containing protein
TGME49_294350	-1.06	7.97	0.006855302	0.019398431	DEAD/DEAH box helicase domain-containing prote
TGME49_202520	1.16	6.53	0.006859895	0.019404002	hypothetical protein
TGME49_277710	-1.12	6.64	0.006878235	0.019448439	hypothetical protein
TGME49_206700	-2.89	4.86	0.00692846	0.019575481	hypothetical protein
TGME49_238070	-2.84	5.04	0.006931724	0.019577223	glutaredoxin domain-containing protein
TGME49_242830	-1.39	6.33	0.006961309	0.01964577	XRN 5'-3' exonuclease N-terminus protein
TGME49_217780	1.28	5.45	0.006980491	0.019692388	Sec20 protein
TGME49_306195	2.13	5.14	0.007016845	0.019787396	hypothetical protein
TGME49_221410	-1.07	6.65	0.007036028	0.019833928	actin-like protein ALP4
TGME49_310330	-1.95	5.47	0.007135299	0.020083136	hypothetical protein
TGME49_244630	1.49	4.89	0.007151709	0.020121665	hypothetical protein
TGME49_215450	2.17	6.55	0.007189314	0.020191292	aquaporin 1
TGME49_261430	-1.58	5.96	0.00719011	0.020191292	hypothetical protein
TGME49_232090	-1.71	5.75	0.007223667	0.020277826	3-hydroxyacyl-CoA dehydrogenase, NAD binding d
TGME49_204080	-1.18	6.87	0.007239746	0.02031525	histidine acid phosphatase superfamily protein
TGME49_299060	-1.06	6.76	0.007246921	0.020327667	sodium/hydrogen exchanger NHE2
TGME49_312210	-2.23	5.52	0.00725993	0.020355948	hypothetical protein
TGME49_228070	1.21	5.71	0.007298921	0.020417148	hypothetical protein
TGME49_229160	-2.87	4.89	0.007300912	0.020417148	DHHC zinc finger domain-containing protein
TGME49_318690	1.15	5.00	0.007311581	0.020439255	RNA recognition motif-containing protein
TGME49_301010	-1.14	6.37	0.007322727	0.020462677	serine/threonine protein phosphatase, putative
TGME49_263710	-1.18	6.32	0.007402921	0.020658419	acyl-CoA:cholesterol acyltransferase alpha ACAT1-
TGME49_212735	-2.27	5.29	0.00740395	0.020658419	hypothetical protein
TGME49_221850	1.41	5.16	0.00744717	0.020771171	prohibitin family protein, putative
TGME49_307860	1.05	8.14	0.007468781	0.020815742	hypothetical protein
TGME49_227900	1.49	6.24	0.00752516	0.020957076	AP2 domain transcription factor AP2X-1

TGME49_285930	-1.79	5.78	0.007534925	0.02097637	hypothetical protein
TGME49_268730	1.15	5.50	0.007540834	0.020977022	glutaredoxin-related protein
TGME49_229790	-1.71	5.71	0.007539598	0.020977022	hypothetical protein
TGME49_244010	-1.91	5.45	0.007593517	0.021107695	hypothetical protein
TGME49_272000	-1.91	5.52	0.007634084	0.021204515	hypothetical protein
TGME49_201770	1.67	6.50	0.007722169	0.021425035	cullin 3, putative
TGME49_228780	1.20	5.46	0.007889613	0.021840434	Toxoplasma gondii family C protein
TGME49_309400	-1.94	5.88	0.007894631	0.021846145	RecF/RecN/SMC N terminal domain-containing pr
TGME49_320210	-1.21	7.39	0.007957575	0.02198741	WD domain, G-beta repeat domain containing pro
TGME49_292110	1.19	6.32	0.007995712	0.022076287	formate/nitrite transporter protein
TGME49_234900	-1.02	7.62	0.008117015	0.022361105	PHD-finger domain-containing protein
TGME49_313660	1.36	4.89	0.008126172	0.022377994	hypothetical protein
TGME49_299190	1.15	5.76	0.008196861	0.022547467	B-box zinc finger domain-containing protein
TGME49_258080	-1.59	5.91	0.008195652	0.022547467	hypothetical protein
TGME49_258100	-2.85	4.89	0.008194033	0.022547467	TPR repeat region protein
TGME49_315420	1.07	5.70	0.008251517	0.022655668	hypothetical protein
TGME49_213690	-1.55	5.66	0.008258984	0.022667754	ring box protein 1 family protein
TGME49_204350	-1.55	5.92	0.008279676	0.022716113	hypothetical protein
TGME49_272290	-1.21	6.44	0.008321669	0.022814394	pyruvate dehydrogenase complex subunit PD-HE1
TGME49_245475	1.21	5.26	0.008330712	0.022830721	hypothetical protein
TGME49_268680	-1.07	6.75	0.008336753	0.022838812	hypothetical protein
TGME49_219530	-2.84	5.05	0.008353668	0.022876674	hypothetical protein
TGME49_240910	-1.36	6.32	0.008371633	0.022917384	hypothetical protein
TGME49_232380	-1.49	5.75	0.008378666	0.022928148	WD domain, G-beta repeat-containing protein
TGME49_226390	2.45	5.45	0.00844277	0.023069418	hypothetical protein
TGME49_225190	2.72	4.76	0.008501383	0.023195286	hypothetical protein
TGME49_298010	-1.23	7.06	0.008523356	0.023246659	hypothetical protein
TGME49_230230	1.31	5.70	0.008527711	0.023249962	hypothetical protein
TGME49_202030	-1.05	6.98	0.008587412	0.023395478	hypothetical protein
TGME49_259700	-1.20	6.48	0.008607103	0.023423231	hypothetical protein
TGME49_217000	-1.43	5.87	0.008666248	0.023558177	hypothetical protein
TGME49_243590	-1.72	5.49	0.008789546	0.023867024	endonuclease/exonuclease/phosphatase family pr
TGME49_223040	1.07	7.86	0.008833916	0.023969899	hypothetical protein
TGME49_208040	-1.68	5.83	0.008865366	0.024037594	aldo-keto reductase
TGME49_297840	-1.11	7.20	0.008875609	0.024056545	DNA primase, large subunit
TGME49_255340	-1.12	6.51	0.008888285	0.024082073	tetratricopeptide repeat-containing protein
TGME49_314550	-2.19	4.92	0.008947437	0.024215721	hypothetical protein
TGME49_263840	-1.68	5.67	0.008956697	0.024224736	hypothetical protein
TGME49_316180	-1.68	5.67	0.008969618	0.024231405	hypothetical protein
TGME49_313280	1.19	5.59	0.008980941	0.024253132	WD domain, G-beta repeat-containing protein
TGME49_242845	-2.79	5.19	0.00907222	0.024472817	hypothetical protein
TGME49_200440	1.38	5.08	0.009079813	0.024484367	hypothetical protein
TGME49_249702	-1.82	5.88	0.009100128	0.024530202	MC family transporter, putative
TGME49_221450	1.22	5.49	0.009109888	0.024547563	SPRY domain-containing protein
TGME49_251480	1.55	5.55	0.009195741	0.024736842	DEAD/DEAH box helicase domain-containing prote
TGME49_263360	-1.68	5.69	0.009196859	0.024736842	WD domain, G-beta repeat-containing protein
TGME49_238870	1.12	5.83	0.009229991	0.024807908	hypothetical protein
TGME49_214470	-1.87	5.61	0.009336135	0.025020435	Ulp1 protease family, C-terminal catalytic domain-
TGME49_310770	-1.24	6.43	0.009371398	0.025096746	hypothetical protein
TGME49_264890	-2.19	5.24	0.009466518	0.025305653	hypothetical protein
TGME49_224890	1.01	7.05	0.009472836	0.025313389	hypothetical protein
TGME49_255195	1.40	5.32	0.009641181	0.025716773	hypothetical protein
TGME49_218200	-2.18	5.33	0.009770481	0.026024114	UDP-sugar pyrophospharylase
TGME49_288990	-2.23	5.24	0.009782534	0.026046833	hypothetical protein

TGME49_205730	-2.18	5.43	0.009804006	0.026085218	hypothetical protein
TGME49_214500	1.19	6.17	0.009891797	0.026290421	ankyrin repeat-containing protein
TGME49_243240	1.56	4.74	0.00994001	0.026380631	WD domain, G-beta repeat-containing protein
TGME49_259230	-2.18	5.32	0.009939472	0.026380631	site-specific recombinase, phage integrase family
TGME49_267480	1.00	5.89	0.009969824	0.026412353	tRNA (guanine(26)-N(2))-dimethyltransferase, put
TGME49_320610	-2.21	5.08	0.00996891	0.026412353	hypothetical protein
TGME49_235450	1.19	7.07	0.010035678	0.026577294	ubiquitin-conjugating enzyme subfamily protein
TGME49_255440	1.22	5.19	0.010113642	0.026755026	hypothetical protein
TGME49_207950	-1.54	5.76	0.010120883	0.026755026	hypothetical protein
TGME49_219832	-1.70	5.29	0.010117726	0.026755026	cyclin-dependent kinase regulatory subunit protei
TGME49_295590	1.30	4.96	0.010160712	0.026841124	hypothetical protein
TGME49_308930	-1.40	5.90	0.010216844	0.026970133	50S ribosomal protein L33, putative
TGME49_226620	-1.66	5.71	0.010265571	0.02708909	hypothetical protein
TGME49_202020	1.11	6.95	0.01029751	0.027153989	DnAK-TPR
TGME49_286510	-1.72	5.50	0.010433282	0.027482609	hypothetical protein
TGME49_319740	-1.53	5.49	0.010466296	0.027559754	transporter, major facilitator family protein
TGME49_304720	-1.57	5.94	0.010498759	0.027635395	hypothetical protein
TGME49_209090	-1.19	6.52	0.010539849	0.027733681	proteasome maturation factor ump1 protein
TGME49_203280	-2.18	5.13	0.010567959	0.027797755	hypothetical protein
TGME49_313570	-1.16	6.48	0.010598647	0.027858656	regulator of chromosome condensation (RCC1) re
TGME49_301120	-1.24	6.22	0.010641462	0.027951322	acetyl-CoA acetyltransferase
TGME49_311160	-1.15	6.69	0.010706357	0.02809184	PWI domain-containing protein
TGME49_238040	-1.13	6.79	0.010766104	0.028228572	protein disulfide-isomerase domain-containing pro
TGME49_262450	-1.10	7.29	0.010823229	0.02835824	hypothetical protein
TGME49_290630	1.03	5.90	0.010912421	0.028551466	AP2 domain transcription factor AP2IX-7
TGME49_221630	-2.14	5.92	0.011007276	0.028779279	hypothetical protein
TGME49_215360	1.25	5.74	0.01105408	0.028871024	hypothetical protein
TGME49_273540	-1.52	5.44	0.011100693	0.028981828	phosphatidylserine synthase, putative
TGME49_207720	-1.30	6.17	0.011302938	0.029468943	hypothetical protein
TGME49_207960	-1.51	5.90	0.011300615	0.029468943	hypothetical protein
TGME49_288370	1.17	5.46	0.011372396	0.029578707	hypothetical protein
TGME49_219710	1.08	7.39	0.011382895	0.029579072	hypothetical protein
TGME49_200375	-2.17	5.05	0.011398527	0.029582559	hypothetical protein
TGME49_292610	-7.12	4.84	0.011407192	0.029594659	Toxoplasma gondii family C protein
TGME49_224980	-1.09	6.48	0.011440005	0.029658976	hypothetical protein
TGME49_249690	-2.78	5.20	0.011476285	0.029742604	hypothetical protein
TGME49_273970	-2.72	5.02	0.011490563	0.029769175	CorA family Mg2+ transporter protein
TGME49_265470	1.29	5.36	0.011555967	0.029907181	hypothetical protein
TGME49_240730	-2.72	5.10	0.011562294	0.029913087	hypothetical protein
TGME49_293220	1.96	5.72	0.011570744	0.029924478	DHHC zinc finger domain-containing protein
TGME49_305260	-2.72	4.78	0.011603792	0.029980809	hypothetical protein
TGME49_202800	-7.14	4.80	0.011634067	0.030046206	cytochrome c oxidase assembly protein COX11, pu
TGME49_266680	-1.66	5.29	0.011736173	0.03027818	hypothetical protein
TGME49_245530	2.61	5.75	0.011793449	0.03038354	hypothetical protein
TGME49_278080	1.37	5.22	0.011810456	0.030416759	Toxoplasma gondii family A protein
TGME49_208430	1.29	5.23	0.011839749	0.030477926	serine proteinase inhibitor PI-2, putative
TGME49_307575	-2.73	5.16	0.011900452	0.030616543	hypothetical protein
TGME49_320670	-1.11	6.65	0.011948971	0.030709312	vacuolar protein sorting 16, putative
TGME49_270350	-1.10	6.42	0.01202879	0.030882248	hypothetical protein
TGME49_255670	-2.72	4.91	0.012035093	0.030887704	methyltransferase domain-containing protein
TGME49_266380	-7.12	5.23	0.012050041	0.030915336	hypothetical protein
TGME49_213000	2.03	5.32	0.012054657	0.030916453	replication factor C, subunit 5, putative
TGME49_240080	1.26	6.39	0.012073633	0.030951305	hypothetical protein
TGME49_244100	-1.12	6.45	0.012097686	0.030983821	snoRNA binding domain-containing protein

TGME49_272570	1.42	5.28	0.012144645	0.031091084	dihydrouridine synthase (dus) protein
TGME49_247590	-1.83	5.36	0.012170949	0.031139098	methyltransferase domain-containing protein
TGME49_293050	-2.14	5.54	0.012287068	0.031403589	sybindin family protein
TGME49_214130	-1.12	6.27	0.012397747	0.031653999	hypothetical protein
TGME49_248750	2.15	4.92	0.012440017	0.031745781	hypothetical protein
TGME49_316750	-2.72	4.89	0.01246778	0.03178854	DEAD/DEAH box helicase domain-containing prote
TGME49_276100	-1.66	5.52	0.012540736	0.031952515	hypothetical protein
TGME49_227820	-1.22	6.10	0.012573018	0.032023733	hypothetical protein
TGME49_295430	-1.65	4.79	0.012590034	0.032044996	hypothetical protein
TGME49_232060	-1.54	5.26	0.012606393	0.032064558	hypothetical protein
TGME49_266860	1.15	7.22	0.012622581	0.032087915	BTB/POZ domain-containing protein
TGME49_258625	-7.11	4.74	0.012906674	0.032693371	peptidyl-prolyl cis-trans isomerase, FKBP-type dom
TGME49_276910	-1.07	6.62	0.012932715	0.032748115	endoplasmic reticulum lumen protein retaining re
TGME49_223900	1.30	5.15	0.012952415	0.032786771	hypothetical protein
TGME49_244040	-1.40	5.69	0.012957955	0.032789569	HEAT repeat-containing protein
TGME49_216220	-1.06	6.84	0.013004524	0.032884901	AP2 domain transcription factor AP2XI-5
TGME49_248660	-1.64	5.28	0.013156166	0.033234265	hypothetical protein
TGME49_292960	1.35	7.78	0.013161095	0.033235362	hypothetical protein
TGME49_211480	-1.61	5.45	0.01331665	0.033570852	GTP-binding protein engA, putative
TGME49_207065	-1.61	5.59	0.013329532	0.033591873	hypothetical protein
TGME49_321600	-1.04	6.53	0.013452198	0.033843331	hypothetical protein
TGME49_203362	-2.71	4.97	0.013496237	0.033919502	hypothetical protein
TGME49_293700	-1.29	6.01	0.013606678	0.034172121	WD domain, G-beta repeat-containing protein
TGME49_218580	-1.61	5.45	0.013703087	0.034357621	RNA methyltransferase, TrmH family protein
TGME49_202190	1.08	6.78	0.013839081	0.034675082	hypothetical protein
TGME49_213388	1.10	5.45	0.013894508	0.0347858	hypothetical protein
TGME49_263510	-1.16	6.36	0.013897384	0.0347858	Spc97 / Spc98 family protein
TGME49_314720	1.09	6.05	0.013952925	0.034886577	Sedlin, N-terminal region protein, putative
TGME49_219540	-1.52	8.71	0.013982548	0.03491623	cytosolic tRNA-Ala synthetase
TGME49_269700	1.37	8.27	0.014017164	0.03498004	NLI interacting factor family phosphatase
TGME49_322000	-1.27	7.25	0.014062331	0.035056264	myosin-light-chain kinase
TGME49_216055	-1.16	6.25	0.014092995	0.035120864	hypothetical protein
TGME49_257980	2.55	4.93	0.014111342	0.035154737	ribosome recycling factor protein
TGME49_224090	-1.61	5.72	0.014259996	0.035501147	enoyl-CoA hydratase/isomerase family protein
TGME49_305030	-1.46	5.98	0.014363607	0.035703617	kinase, pfkB family protein
TGME49_307605	-1.00	6.90	0.014391774	0.035756982	hypothetical protein
TGME49_315710	1.43	5.62	0.014454335	0.035888298	hypothetical protein
TGME49_239310	-2.12	5.36	0.014465016	0.035902763	ribulose 5-phosphate isomerase
TGME49_215250	-1.79	5.32	0.014531413	0.036055459	thiamin pyrophosphokinase, catalytic domain-con
TGME49_237160	-1.32	6.04	0.014589863	0.036177748	hypothetical protein
TGME49_218810	-1.29	5.77	0.014608908	0.036211287	histidyl-tRNA synthetase
TGME49_216960	1.10	5.62	0.014730382	0.036463491	elongation factor Tu GTP binding domain-containi
TGME49_238080	1.27	5.19	0.014877493	0.036753819	hypothetical protein
TGME49_293600	1.05	5.75	0.015014351	0.037067148	ribosomal protein RPL27
TGME49_289615	1.86	5.40	0.015061041	0.037157605	hypothetical protein
TGME49_267650	-1.48	5.66	0.015139284	0.037304051	hypothetical protein
TGME49_297270	-2.08	5.28	0.015153785	0.037324146	hypothetical protein
TGME49_239087	-1.78	4.96	0.01524815	0.037514094	hypothetical protein
TGME49_246730	1.03	6.92	0.015300021	0.03760916	hypothetical protein
TGME49_243600	-1.04	6.49	0.015379806	0.037780161	acetyltransferase, GNAT family protein
TGME49_293680	-1.28	6.24	0.015399909	0.037780327	hypothetical protein
TGME49_264210	1.02	5.57	0.015530199	0.038035869	hypothetical protein
TGME49_249300	-1.63	7.71	0.015596485	0.038148236	hypothetical protein
TGME49_249310	-2.65	5.20	0.015592686	0.038148236	hypothetical protein

TGME49_210478	2.28	4.78	0.01561074	0.038157294	hypothetical protein
TGME49_267855	-1.02	6.58	0.015673399	0.038285138	hypothetical protein
TGME49_255430	-1.48	5.29	0.015731868	0.038415268	Rad9 protein
TGME49_234280	-1.08	6.09	0.015846164	0.038668823	AMP deaminase
TGME49_238140	-1.15	6.57	0.01587879	0.038724094	hypothetical protein
TGME49_203750	-1.78	5.36	0.015946314	0.038861912	hypothetical protein
TGME49_246182	-1.29	5.76	0.01600518	0.038992522	hypothetical protein
TGME49_254480	1.43	5.93	0.016087915	0.039141494	WD domain, G-beta repeat-containing protein
TGME49_262990	-2.67	4.94	0.016103385	0.039141494	hypothetical protein
TGME49_269417	-2.65	4.68	0.016199548	0.039323526	hypothetical protein
TGME49_258010	-1.62	5.57	0.016229707	0.039383806	calcium signaling protein kinase RAD53, putative
TGME49_211330	-2.18	4.75	0.01635566	0.03957707	methionine aminopeptidase
TGME49_219510	1.51	6.27	0.016970665	0.040876721	GTP binding protein 7 isoform 2 family protein, pu
TGME49_288560	-1.21	6.49	0.017105288	0.041143949	hypothetical protein
TGME49_248800	2.35	5.16	0.017118414	0.04116213	hypothetical protein
TGME49_289950	-1.08	6.53	0.017359306	0.041619556	hypothetical protein
TGME49_316150	-1.32	5.95	0.017390711	0.041681335	ULK kinase
TGME49_218192	-6.99	5.36	0.017435721	0.041775671	hypothetical protein
TGME49_232520	-1.24	5.75	0.017498202	0.041898222	brix domain-containing protein
TGME49_237100	1.27	5.16	0.017611009	0.042113826	RAP domain-containing protein
TGME49_235660	-1.32	5.76	0.017611029	0.042113826	hypothetical protein
TGME49_311040	-1.12	5.92	0.017777343	0.042429204	hypothetical protein
TGME49_283900	-1.45	5.67	0.017816825	0.042509714	ATPase, AAA family protein
TGME49_226520	-1.12	5.80	0.01787066	0.04261066	hypothetical protein
TGME49_297980	1.38	4.86	0.017879897	0.042618943	hypothetical protein
TGME49_287480	1.04	8.12	0.017985954	0.042844119	hypothetical protein
TGME49_204150	1.32	4.92	0.018014206	0.042897598	hypothetical protein
TGME49_264752	-2.63	5.23	0.0180583	0.042988753	HEAT repeat-containing protein
TGME49_203950	-1.04	6.39	0.018072145	0.043007866	Myb family DNA-binding domain-containing prote
TGME49_220150	-1.74	5.32	0.01808925	0.043026464	50S ribosomal protein L16, putative
TGME49_258050	-2.03	5.14	0.018091954	0.043026464	actin like protein ALP2a
TGME49_273885	-2.04	5.11	0.018097417	0.043026464	hypothetical protein
TGME49_246000	1.33	4.76	0.018195902	0.043232808	large subunit ribosomal protein IMG2
TGME49_210310	-1.74	5.16	0.018338528	0.043519936	hypothetical protein
TGME49_312820	-1.43	5.76	0.018390518	0.043623458	hypothetical protein
TGME49_240480	-2.70	4.71	0.018401618	0.043623458	cpw-wpc domain-containing protein
TGME49_295090	1.07	7.42	0.01845644	0.043711377	hypothetical protein
TGME49_255310	-1.27	5.94	0.018472932	0.043736426	zinc finger (CCCH type) motif-containing protein
TGME49_257070	-2.03	5.02	0.018498038	0.043767838	hypothetical protein
TGME49_293490	1.72	4.86	0.018583042	0.043954897	hypothetical protein
TGME49_251590	1.17	5.99	0.018723141	0.044257961	hypothetical protein
TGME49_273380	1.42	6.00	0.018745094	0.044295693	ion channel protein
TGME49_309960	-1.03	7.38	0.018824934	0.044455945	hypothetical protein
TGME49_262933	-1.61	5.41	0.018899804	0.04460426	hypothetical protein
TGME49_209530	1.26	4.95	0.018955962	0.04470792	hypothetical protein
TGME49_262420	1.18	6.13	0.018953105	0.04470792	AP2 domain transcription factor APVIIb-1/ADA2-B
TGME49_271370	-1.82	4.88	0.019136351	0.045047494	hypothetical protein
TGME49_268640	2.40	5.29	0.019226693	0.045202597	BING4CT (NUC141) domain-containing protein
TGME49_212290	-1.10	9.79	0.019277152	0.045306821	ribosomal protein RPS19
TGME49_260640	-1.54	5.79	0.019294055	0.045332139	autophagy protein apg9 protein
TGME49_306890	-1.30	5.84	0.019335575	0.04541526	hypothetical protein
TGME49_233770	2.55	6.27	0.019374595	0.04549246	calcium-translocating P-type ATPase, PMCA-type p
TGME49_310500	-1.12	6.13	0.019494577	0.045759651	hypothetical protein
TGME49_313090	-1.74	5.25	0.019519824	0.045804374	hypothetical protein

TGME49_278020	-2.58	4.94	0.019695415	0.046157813	hypothetical protein
TGME49_254420	2.12	5.55	0.019797946	0.046368708	phospholipase, patatin family protein
TGME49_292035	1.30	4.87	0.019917707	0.046590169	hypothetical protein
TGME49_219130	-1.39	5.89	0.020144442	0.047060982	NADPH-glutathione reductase
TGME49_261960	1.21	5.17	0.020191326	0.047140721	hypothetical protein
TGME49_262880	-1.15	6.33	0.020334332	0.047459611	hypothetical protein
TGME49_288245	-1.00	6.04	0.020440839	0.047693141	hypothetical protein
TGME49_219100	-1.39	5.69	0.020464923	0.047734272	cyclin-dependent kinase regulatory subunit protei
TGME49_240700	-1.29	5.96	0.020515819	0.047837895	ubiquitin family protein
TGME49_316770	-1.39	5.68	0.020707553	0.048221442	undecaprenyl diphosphate synthase
TGME49_226350	1.16	5.21	0.020820091	0.048454122	hypothetical protein
TGME49_289790	-2.58	4.93	0.02083252	0.048454122	hypothetical protein
TGME49_213752	-1.54	5.38	0.02094416	0.048622016	herpesviridae ul52/ul70 dna primase
TGME49_220460	-6.86	4.98	0.020978103	0.048670251	SNF7 family protein
TGME49_247280	-1.70	5.61	0.021027356	0.048738642	hypothetical protein
TGME49_278030	1.09	7.76	0.021067718	0.048786316	hypothetical protein
TGME49_211020	-1.39	5.85	0.021092568	0.048813286	RNA recognition motif-containing protein
TGME49_255270	1.98	4.98	0.021137791	0.048902636	hypothetical protein
TGME49_226080	1.13	5.42	0.021152026	0.048904968	polyA polymerase
TGME49_313630	2.69	7.24	0.021318974	0.049221514	hypothetical protein
TGME49_229200	-1.40	5.54	0.02133536	0.049236471	hypothetical protein
TGME49_242880	-1.70	5.25	0.021374461	0.049311316	flavoprotein
TGME49_286610	1.41	4.91	0.021463872	0.049481188	ribosomal protein RPS14, putative
TGME49_285660	-1.55	5.85	0.021555536	0.049620683	DEAD/DEAH box helicase domain-containing prote
TGME49_263560	1.13	7.28	0.021583075	0.049660367	hypothetical protein
TGME49_257060	-1.70	5.49	0.021586208	0.049660367	translation initiation factor sui1 protein

Supplementary Table S7B: Differencial expression analysis of T. gondii genes from neuronal stem cells infected with T. gondii EGS strain for 18 hours copmared to T. gondii gene expression from neuronal stem cells infected with ME49 strain for 18 hours.

gene ID	logFC (EGS/ME49)	logCPM	PValue	FDR	Product Name
TGME49_233925	7.05	7.83	2.01E-150	1.49E-146	hypothetical protein
TGME49_322200	10.27	6.70	4.84E-126	1.79E-122	apocytochrome b, putative
TGME49_237130	8.63	10.26	1.14E-111	2.80E-108	cytochrome b, putative
TGME49_330000	9.84	10.23	1.22E-93	2.25E-90	cytochrome b
TGME49_323400	10.51	9.06	1.61E-90	2.38E-87	cytochrome c oxidase subunit iii subfamily protein
TGME49_322800	12.26	6.48	3.45E-88	4.25E-85	hypothetical protein
TGME49_290600	3.32	9.11	5.24E-86	5.54E-83	succinyl-CoA-synthetase alpha SCSA
TGME49_320050	4.45	13.43	8.97E-86	8.29E-83	ribosomal protein RPL5
TGME49_302055	11.93	5.97	4.97E-64	4.08E-61	ribosomal protein RPS12
TGME49_238240	2.80	8.97	3.64E-61	2.69E-58	bystin protein
TGME49_261240	8.23	5.02	3.40E-57	2.29E-54	histone H3
TGME49_255060	8.34	9.95	1.10E-55	6.77E-53	cytochrome b(N-terminal)/b6/petB subfamily protein
TGME49_321360	3.10	8.21	2.77E-54	1.57E-51	clustered-asparagine-rich protein
TGME49_301250	8.17	14.41	2.18E-52	1.15E-49	hypothetical protein
TGME49_232955	11.71	12.33	4.40E-46	2.17E-43	hypothetical protein
TGME49_251180	3.68	6.80	2.34E-45	1.08E-42	KRUF family protein
TGME49_277230	4.50	5.63	4.12E-43	1.79E-40	hypothetical protein
TGME49_280400	-12.29	7.58	1.47E-42	6.05E-40	hypothetical protein
TGME49_217530	4.39	6.35	1.13E-40	4.38E-38	hypothetical protein
TGME49_293790	6.17	6.72	3.29E-40	1.22E-37	hypothetical protein
TGME49_262690	1.87	10.98	5.15E-35	1.81E-32	ribosomal protein RPL27
TGME49_218520	1.97	10.33	5.12E-34	1.72E-31	microneme protein MIC6
TGME49_244370	3.61	8.80	1.82E-32	5.85E-30	TDC1, putative
TGME49_213280	2.05	8.91	4.06E-31	1.25E-28	SAG-related sequence SRS25
TGME49_211695	3.87	5.62	8.07E-31	2.39E-28	hypothetical protein
TGME49_221840	4.42	4.71	1.26E-29	3.59E-27	hypothetical protein
TGME49_277260	2.05	9.53	1.78E-29	4.88E-27	hypothetical protein
TGME49_253690	1.87	8.98	1.02E-28	2.70E-26	hypothetical protein
TGME49_273320	2.45	7.86	5.79E-28	1.48E-25	hypothetical protein
TGME49_271935	2.66	6.69	1.45E-26	3.57E-24	hypothetical protein
TGME49_315885	2.57	7.08	1.22E-25	2.92E-23	glycosyltransferase, putative
TGME49_202970	-12.46	7.43	1.42E-25	3.28E-23	hypothetical protein
TGME49_211030	-3.84	8.15	2.17E-25	4.86E-23	hypothetical protein
TGME49_223660	2.72	7.34	9.71E-25	2.11E-22	50S ribosomal protein L4, putative
TGME49_208020	3.67	6.12	4.87E-24	1.03E-21	AP2 domain transcription factor AP2Ib-1
TGME49_213010	-6.45	7.34	5.99E-24	1.23E-21	hypothetical protein
TGME49_250710	1.75	11.27	2.16E-23	4.31E-21	microneme protein MIC10
TGME49_208370	1.75	8.84	3.17E-23	6.16E-21	myosin heavy chain, putative
TGME49_251400	2.83	6.13	4.84E-23	9.18E-21	hypothetical protein
TGME49_293170	-5.85	7.60	5.00E-23	9.24E-21	hypothetical protein
TGME49_290020	2.52	6.64	9.34E-23	1.68E-20	cyclin dependent kinase binding protein
TGME49_237230	1.74	9.81	9.91E-23	1.75E-20	hypothetical protein
TGME49_207460	2.16	7.42	1.08E-22	1.86E-20	Rab5B protein
TGME49_254710	2.02	7.81	1.28E-22	2.14E-20	serine esterase (DUF676) protein
TGME49_266050	2.21	7.09	1.40E-22	2.30E-20	hypothetical protein
TGME49_311230	1.55	9.99	5.69E-22	9.14E-20	hypothetical protein

TGME49_228160	2.03	7.19	6.82E-22	1.07E-19	acid phosphatase
TGME49_280490	-3.83	7.89	1.12E-21	1.72E-19	U-box domain-containing protein
TGME49_252220	-2.92	7.73	3.09E-21	4.47E-19	tetratricopeptide repeat domain containing protein
TGME49_312140	-3.53	8.29	3.23E-21	4.59E-19	hypothetical protein
TGME49_214980	1.82	8.41	3.39E-21	4.73E-19	hypothetical protein
TGME49_213570	1.61	8.76	5.04E-21	6.90E-19	hypothetical protein
TGME49_290270	2.38	6.58	9.30E-21	1.25E-18	SPRY domain-containing protein
TGME49_251540	2.03	9.03	9.59E-21	1.27E-18	dense granule protein GRA9
TGME49_214220	1.87	9.74	1.07E-20	1.36E-18	hypothetical protein
TGME49_261720	-5.72	7.82	1.06E-20	1.36E-18	metal cation transporter, ZIP family protein
TGME49_305160	3.64	4.99	2.64E-20	3.31E-18	histone H2Ba
TGME49_314500	-3.17	8.36	4.40E-20	5.42E-18	subtilisin SUB2
TGME49_243615	3.28	5.68	5.89E-20	7.13E-18	hypothetical protein
TGME49_254120	2.28	8.30	6.71E-20	8.01E-18	autophagy-related protein 8 atg8, putative
TGME49_229010	-2.17	9.17	9.07E-20	1.06E-17	rhoptry neck protein RON4
TGME49_208740	1.73	9.88	1.02E-19	1.18E-17	microneme protein, putative
TGME49_205680	2.99	8.42	4.43E-19	5.04E-17	hypothetical protein
TGME49_301222	3.03	6.56	4.62E-19	5.18E-17	DNA repair protein Rad4 domain-containing protein
TGME49_235130	-4.32	7.38	7.20E-19	7.94E-17	transmembrane protein
TGME49_251740	-3.84	7.43	9.53E-19	1.04E-16	AP2 domain transcription factor AP2XII-9
TGME49_260430	3.90	4.67	1.09E-18	1.17E-16	hypothetical protein
TGME49_200300	6.44	4.83	1.90E-18	2.00E-16	hypothetical protein
TGME49_275860	2.43	9.46	2.52E-18	2.63E-16	hypothetical protein
TGME49_319500	-10.71	7.06	2.59E-18	2.66E-16	hypothetical protein
TGME49_287460	3.42	4.88	2.89E-18	2.92E-16	hypothetical protein
TGME49_201170	-2.61	8.67	3.06E-18	3.06E-16	hypothetical protein
TGME49_226310	-4.25	7.74	4.51E-18	4.44E-16	zinc finger (CCCH type) motif-containing protein
TGME49_254080	1.64	7.96	1.97E-17	1.92E-15	metal cation transporter, ZIP family protein
TGME49_257910	4.29	5.98	2.83E-17	2.72E-15	hypothetical protein
TGME49_232650	2.25	6.75	3.41E-17	3.19E-15	hypothetical protein
TGME49_275640	2.00	6.94	3.82E-17	3.48E-15	hypothetical protein
TGME49_239790	-10.48	7.09	3.78E-17	3.48E-15	BRCA1 C Terminus (BRCT) domain-containing protein
TGME49_287170	-10.57	7.07	5.50E-17	4.96E-15	hypothetical protein
TGME49_240060	1.66	9.24	5.60E-17	4.99E-15	hypothetical protein
TGME49_224760	3.36	4.85	5.87E-17	5.16E-15	SAG-related sequence SRS40E
TGME49_249540	1.86	7.36	6.15E-17	5.35E-15	hypothetical protein
TGME49_253430	1.25	10.15	7.59E-17	6.53E-15	asparagine synthetase, putative
TGME49_249990	2.33	10.24	8.58E-17	7.29E-15	hypothetical protein
TGME49_269310	3.54	4.71	1.17E-16	9.72E-15	hypothetical protein
TGME49_287040	2.26	7.04	1.16E-16	9.72E-15	hypothetical protein
TGME49_269330	-10.42	7.07	1.28E-16	1.05E-14	hypothetical protein
TGME49_293570	-3.03	7.85	1.46E-16	1.18E-14	translocation protein sec62, putative
TGME49_318660	1.66	8.55	1.91E-16	1.53E-14	PP2C, putative
TGME49_216680	-4.00	7.27	2.01E-16	1.60E-14	ankyrin repeat-containing protein
TGME49_253570	2.76	6.14	2.48E-16	1.95E-14	hypothetical protein
TGME49_228690	-5.43	7.16	2.78E-16	2.16E-14	phosphatidylinositol 3- and 4-kinase
TGME49_290700	2.22	7.93	2.82E-16	2.17E-14	hypothetical protein
TGME49_223930	1.62	8.26	3.30E-16	2.49E-14	RNA recognition motif-containing protein
TGME49_259260	-2.40	9.01	3.64E-16	2.72E-14	membrane protein FtsH1
TGME49_261022	-2.86	7.63	3.83E-16	2.83E-14	dynein heavy chain family protein
TGME49_301890	-3.97	6.15	3.91E-16	2.86E-14	Toxoplasma gondii family B protein
TGME49_265190	-4.49	7.20	3.99E-16	2.89E-14	Ulp1 protease family, C-terminal catalytic domain-co
TGME49_306670	-3.50	7.92	4.67E-16	3.35E-14	hypothetical protein
TGME49_270595	-3.32	6.90	5.10E-16	3.63E-14	UBA/TS-N domain-containing protein
TGME49_236910	-4.53	7.47	5.55E-16	3.91E-14	U2 snRNP auxiliary factor, putative

TGME49_293410	2.96	5.32	5.89E-16	4.11E-14	hypothetical protein
TGME49_212880	-2.89	7.91	6.47E-16	4.47E-14	surface antigen repeat-containing protein
TGME49_222380	-2.55	8.13	6.97E-16	4.77E-14	importin-beta N-terminal domain-containing protein
TGME49_202420	2.25	6.26	9.21E-16	6.25E-14	hypothetical protein
TGME49_239410	-2.63	7.90	1.09E-15	7.35E-14	hypothetical protein
TGME49_236040	1.22	9.85	1.21E-15	8.07E-14	fructose-1,6-bisphosphate aldolase
TGME49_211150	-5.27	6.82	1.25E-15	8.28E-14	hypothetical protein
TGME49_240650	-2.36	8.44	1.45E-15	9.48E-14	coatomer protein complex, subunit alpha, putative
TGME49_234510	1.90	6.89	2.27E-15	1.48E-13	ankyrin repeat-containing protein
TGME49_224880	-4.06	7.04	2.41E-15	1.55E-13	kinesin motor domain-containing protein
TGME49_220250	1.86	6.94	2.74E-15	1.75E-13	Nucleotide-sensitive chloride conductance regulator
TGME49_246330	-5.33	7.00	3.23E-15	2.04E-13	CRAL/TRIO domain-containing protein
TGME49_248260	2.78	6.49	3.28E-15	2.06E-13	hypothetical protein
TGME49_222400	1.64	7.58	3.34E-15	2.08E-13	hypothetical protein
TGME49_261520	-5.22	7.04	3.38E-15	2.08E-13	nucleolar GTP-binding protein 1, putative
TGME49_234460	2.22	6.73	3.49E-15	2.12E-13	hypothetical protein
TGME49_267020	-4.00	6.64	3.50E-15	2.12E-13	hypothetical protein
TGME49_270700	1.73	7.08	3.61E-15	2.17E-13	hypothetical protein
TGME49_301170	-10.53	5.92	4.23E-15	2.52E-13	SAG-related sequence SRS19D
TGME49_210300	-5.29	6.98	4.63E-15	2.74E-13	hypothetical protein
TGME49_239440	-10.30	6.88	4.77E-15	2.80E-13	protein kinase (incomplete catalytic triad)
TGME49_212940	-10.38	5.88	4.93E-15	2.87E-13	hypothetical protein
TGME49_209050	-10.26	6.78	6.12E-15	3.53E-13	Tyrosine kinase-like (TKL) protein
TGME49_252430	1.68	8.12	1.01E-14	5.80E-13	hypothetical protein
TGME49_321650	-2.84	7.47	1.02E-14	5.80E-13	hypothetical protein
TGME49_235680	-2.96	7.23	1.10E-14	6.22E-13	peptidase M16 inactive domain-containing protein
TGME49_278800	-2.53	7.62	1.24E-14	6.94E-13	zinc finger protein 36 family 3 protein
TGME49_204050	3.19	10.48	1.42E-14	7.86E-13	subtilisin SUB1
TGME49_254520	1.63	7.43	1.54E-14	8.41E-13	mediator complex subunit MED11
TGME49_233450	1.48	8.00	1.67E-14	9.08E-13	SAG-related sequence SRS29A
TGME49_254730	2.31	6.04	1.80E-14	9.69E-13	POPLD (NUC188) domain-containing protein
TGME49_313230	2.54	9.82	2.17E-14	1.16E-12	eukaryotic initiation factor-2, alpha subunit
TGME49_283540	2.10	8.26	2.40E-14	1.27E-12	hypothetical protein
TGME49_202310	2.33	6.20	2.73E-14	1.43E-12	O-sialoglycoprotein endopeptidase
TGME49_278510	-2.72	7.68	2.89E-14	1.50E-12	protein phosphatase 2C domain-containing protein
TGME49_277000	-4.39	7.32	3.12E-14	1.61E-12	transport protein Sec24, putative
TGME49_214410	2.96	7.77	3.25E-14	1.67E-12	hypothetical protein
TGME49_282070	-2.02	8.54	3.69E-14	1.88E-12	hypothetical protein
TGME49_221470	-2.20	8.22	3.74E-14	1.90E-12	hypothetical protein
TGME49_250820	-2.93	7.50	3.85E-14	1.93E-12	hypothetical protein
TGME49_270360	-5.18	7.03	3.86E-14	1.93E-12	hypothetical protein
TGME49_305590	-4.24	6.99	6.34E-14	3.14E-12	ABC transporter transmembrane region domain-cont
TGME49_315620	-5.11	7.07	6.47E-14	3.19E-12	vacuolar ATP synthase subunit C, putative
TGME49_260500	-1.98	9.04	7.38E-14	3.61E-12	COPI associated protein, putative
TGME49_259550	-2.93	7.66	7.63E-14	3.71E-12	dihydropteroate synthase
TGME49_231150	3.03	5.08	9.41E-14	4.55E-12	hypothetical protein
TGME49_239020	-10.28	6.19	9.61E-14	4.62E-12	ABC transporter transmembrane region domain-cont
TGME49_298620	-5.08	6.83	1.05E-13	5.00E-12	hypothetical protein
TGME49_216820	1.54	8.57	1.08E-13	5.12E-12	transporter, major facilitator family protein
TGME49_221590	1.58	8.78	1.18E-13	5.58E-12	dual specificity phosphatase, catalytic domain-contai
TGME49_300220	-3.20	7.60	1.55E-13	7.22E-12	hypothetical protein
TGME49_249350	-10.09	6.75	1.76E-13	8.12E-12	esterase/lipase/thioesterase domain-containing prot
TGME49_255180	-10.08	6.36	1.80E-13	8.26E-12	ubiquitin carboxyl-terminal hydrolase
TGME49_216140	2.28	7.52	1.96E-13	8.96E-12	tetratricopeptide repeat-containing protein
TGME49_294690	1.96	7.53	2.04E-13	9.25E-12	rhomboid protease ROM5

TGME49_244530	1.56	8.53	2.06E-13	9.27E-12	hypothetical protein
TGME49_295990	1.76	7.35	2.15E-13	9.61E-12	ubiquitin conjugating enzyme E2, putative
TGME49_220880	2.15	6.18	2.30E-13	1.03E-11	hypothetical protein
TGME49_254050	1.81	6.83	2.54E-13	1.13E-11	optic atrophy 3 protein (opa3) protein
TGME49_295440	2.62	5.97	2.56E-13	1.13E-11	hypothetical protein
TGME49_210408	1.13	10.69	2.93E-13	1.28E-11	HMG (high mobility group) box domain-containing pr
TGME49_237015	1.75	7.91	3.36E-13	1.46E-11	hypothetical protein
TGME49_250030	2.66	5.24	3.73E-13	1.61E-11	hypothetical protein
TGME49_291890	1.33	11.01	3.85E-13	1.65E-11	microneme protein MIC1
TGME49_203300	2.78	7.42	3.93E-13	1.67E-11	hypothetical protein
TGME49_213255	-5.17	5.85	3.92E-13	1.67E-11	hypothetical protein
TGME49_321530	1.44	8.75	4.04E-13	1.71E-11	cathepsin CPL
TGME49_320588	-10.06	6.31	4.09E-13	1.72E-11	glycosyl hydrolases family 35 protein
TGME49_253170	1.61	7.37	4.28E-13	1.79E-11	zinc carboxypeptidase, putative
TGME49_270580	-10.08	5.66	4.87E-13	2.02E-11	HECT-domain (ubiquitin-transferase) domain-contain
TGME49_316280	-5.04	6.90	4.98E-13	2.06E-11	transporter, major facilitator family protein
TGME49_288860	-5.22	6.98	5.57E-13	2.29E-11	RuvB family 2 protein
TGME49_286470	1.35	8.36	6.04E-13	2.47E-11	AGC kinase
TGME49_241830	2.13	5.87	6.26E-13	2.54E-11	peptidyl-prolyl cis-trans isomerase
TGME49_214230	1.80	6.85	7.03E-13	2.83E-11	Dopey, N-terminal domain-containing protein
TGME49_263870	-5.03	6.97	7.64E-13	3.06E-11	glutamate-tRNA ligase
TGME49_294610	-1.61	9.34	8.25E-13	3.28E-11	histone lysine methyltransferase, SET, putative
TGME49_239420	1.11	9.63	8.72E-13	3.45E-11	protein kinase
TGME49_228280	2.37	5.71	1.08E-12	4.26E-11	hypothetical protein
TGME49_247530	3.16	8.95	1.19E-12	4.65E-11	hypothetical protein
TGME49_269840	1.41	7.95	1.23E-12	4.79E-11	proteasome regulatory subunit
TGME49_212900	1.59	8.11	1.44E-12	5.56E-11	hypothetical protein
TGME49_285140	-10.00	6.26	1.48E-12	5.69E-11	hypothetical protein
TGME49_294770	-4.97	6.84	1.52E-12	5.81E-11	Armadillo/beta-catenin family repeat-containing prot
TGME49_229020	-9.96	6.41	1.62E-12	6.16E-11	cell-cycle-associated protein kinase CDK, putative
TGME49_257290	-10.23	5.89	1.64E-12	6.21E-11	RNA 2'-phosphotransferase, Tpt1/KptA family protein
TGME49_255650	-10.24	7.05	2.27E-12	8.57E-11	DHHC zinc finger domain-containing protein
TGME49_244910	-4.10	6.82	2.30E-12	8.65E-11	MIZ/SP-RING zinc finger domain-containing protein
TGME49_237210	-4.90	6.72	2.54E-12	9.49E-11	Tyrosine kinase-like (TKL) protein
TGME49_213920	-3.63	6.79	2.62E-12	9.73E-11	hypothetical protein
TGME49_252880	-2.76	7.29	2.76E-12	1.02E-10	hypothetical protein
TGME49_269970	1.73	6.91	3.07E-12	1.13E-10	hypothetical protein
TGME49_263550	1.82	6.57	3.12E-12	1.14E-10	39S ribosomal protein L47, mitochondrial precursor,
TGME49_266070	1.25	9.66	3.38E-12	1.23E-10	ribosomal protein RPL31
TGME49_275755	3.25	4.98	3.68E-12	1.34E-10	hypothetical protein
TGME49_230410	-2.13	8.00	3.74E-12	1.35E-10	peroxiredoxin PRX3
TGME49_254470	1.48	9.18	3.77E-12	1.35E-10	hypothetical protein
TGME49_263490	2.29	5.71	3.81E-12	1.36E-10	ubiquitin conjugating enzyme E2, putative
TGME49_220950	1.59	9.89	4.00E-12	1.42E-10	hypothetical protein
TGME49_289710	-2.62	7.42	4.09E-12	1.45E-10	AP2 domain transcription factor AP2IX-5
TGME49_242090	1.04	9.97	4.62E-12	1.63E-10	zinc finger (CCCH type) motif-containing protein
TGME49_244880	-4.05	6.91	4.64E-12	1.63E-10	DNA-directed RNA polymerase I RPA1
TGME49_209440	-2.34	7.38	4.73E-12	1.65E-10	hypothetical protein
TGME49_205265	2.34	6.35	4.80E-12	1.67E-10	transporter, cation channel family protein
TGME49_245510	-2.58	7.52	4.93E-12	1.70E-10	phospholipid-translocating P-type ATPase, flippase su
TGME49_214350	-2.91	7.39	4.95E-12	1.70E-10	GTP-binding protein, putative
TGME49_228660	-3.59	6.95	5.00E-12	1.71E-10	Sec7 domain-containing protein
TGME49_226910	-4.89	6.44	5.61E-12	1.91E-10	Amylo-alpha-1,6-glucosidase
TGME49_200350	3.12	5.77	6.43E-12	2.17E-10	subtilisin SUB3
TGME49_278940	1.80	6.96	6.44E-12	2.17E-10	HECT-domain (ubiquitin-transferase) domain-contain

TGME49_316660	-9.87	6.50	6.74E-12	2.27E-10	cullin family protein
TGME49_305180	-9.86	6.43	6.84E-12	2.29E-10	Na+/H+ exchanger NHE3
TGME49_275870	2.54	5.59	6.91E-12	2.30E-10	tubulin/FtsZ family, GTPase domain-containing prote
TGME49_294990	-9.90	6.22	7.15E-12	2.37E-10	hypothetical protein
TGME49_217520	1.29	8.89	7.39E-12	2.43E-10	hypothetical protein
TGME49_201390	1.07	10.65	7.93E-12	2.59E-10	hypothetical protein
TGME49_236670	2.50	6.27	8.29E-12	2.70E-10	hypothetical protein
TGME49_242290	-2.37	7.99	8.81E-12	2.86E-10	proteasome subunit alpha1, putative
TGME49_310450	-2.75	7.13	9.13E-12	2.95E-10	myosin heavy chain, putative
TGME49_221675	1.45	7.82	9.64E-12	3.10E-10	hypothetical protein
TGME49_260440	1.37	9.39	1.07E-11	3.43E-10	nuclear factor NF3
TGME49_247330	-2.74	6.92	1.15E-11	3.65E-10	hypothetical protein
TGME49_290170	-2.60	7.34	1.17E-11	3.71E-10	kelch repeat domain containing/Serine/threonine pro
TGME49_239800	-3.25	6.90	1.19E-11	3.75E-10	hypothetical protein
TGME49_239540	-9.86	6.42	1.21E-11	3.81E-10	LEM3 (ligand-effect modulator 3) family / CDC50 fam
TGME49_286120	-2.26	8.02	1.23E-11	3.84E-10	prolyl endopeptidase
TGME49_266410	1.99	6.23	1.23E-11	3.85E-10	hypothetical protein
TGME49_254220	2.34	5.45	1.31E-11	4.05E-10	hypothetical protein
TGME49_277940	-4.02	6.79	1.30E-11	4.05E-10	hypothetical protein
TGME49_204530	1.30	11.00	1.47E-11	4.54E-10	microneme protein MIC11
TGME49_298830	2.37	6.26	1.52E-11	4.66E-10	hypothetical protein
TGME49_259240	1.36	9.56	1.57E-11	4.81E-10	ribosomal protein RPS21
TGME49_269690	1.49	8.07	1.80E-11	5.47E-10	hypothetical protein
TGME49_253860	1.41	7.25	1.92E-11	5.83E-10	Tyrosine kinase-like (TKL) protein
TGME49_265790	1.22	8.70	1.98E-11	5.96E-10	hypothetical protein
TGME49_201860	1.09	10.42	2.12E-11	6.38E-10	hypothetical protein
TGME49_213410	-2.18	7.73	2.15E-11	6.44E-10	small nuclear ribonucleoprotein f (snrnp-f), putative
TGME49_278870	-1.62	8.87	2.17E-11	6.47E-10	myosin F
TGME49_204520	2.76	4.97	2.29E-11	6.80E-10	hypothetical protein
TGME49_227810	1.34	8.87	2.35E-11	6.94E-10	rhoptry kinase family protein ROP11 (incomplete cata
TGME49_257700	3.12	5.46	2.41E-11	7.09E-10	hypothetical protein
TGME49_257520	1.64	7.16	2.56E-11	7.50E-10	synaptobrevin protein
TGME49_201785	1.34	8.40	2.91E-11	8.51E-10	hypothetical protein
TGME49_208530	-3.01	7.28	3.11E-11	9.02E-10	nicotinate phosphoribosyltransferase
TGME49_232500	-9.78	6.44	3.16E-11	9.13E-10	hypothetical protein
TGME49_294550	-1.96	8.00	3.19E-11	9.17E-10	dynein heavy chain
TGME49_319880	-4.75	6.31	3.54E-11	1.02E-09	MORN repeat-containing protein
TGME49_260180	-2.09	7.68	3.67E-11	1.05E-09	hypothetical protein
TGME49_297940	-2.13	7.42	3.76E-11	1.07E-09	single-strand binding protein
TGME49_217700	-9.79	6.41	4.07E-11	1.15E-09	AP2 domain transcription factor AP2XII-2
TGME49_253090	2.14	5.86	4.21E-11	1.19E-09	DEAD/DEAH box helicase domain-containing protein
TGME49_286460	2.96	5.00	4.32E-11	1.21E-09	hypothetical protein
TGME49_247960	2.01	6.05	4.36E-11	1.22E-09	hypothetical protein
TGME49_203720	3.14	8.00	4.41E-11	1.23E-09	vitamin k epoxide reductase family protein
TGME49_205170	2.05	8.72	4.45E-11	1.24E-09	hypothetical protein
TGME49_265390	-9.76	6.16	4.57E-11	1.27E-09	hypothetical protein
TGME49_292130	-1.30	9.66	4.75E-11	1.31E-09	ribosomal protein RPL13A
TGME49_207680	-3.93	7.02	4.77E-11	1.31E-09	suppressor of kinetochore protein 1, putative
TGME49_225790	1.70	7.33	4.80E-11	1.31E-09	PDI family protein
TGME49_214140	1.33	8.62	4.93E-11	1.33E-09	hypothetical protein
TGME49_238895	-3.03	7.20	4.92E-11	1.33E-09	hypothetical protein
TGME49_231430	-9.75	6.39	4.92E-11	1.33E-09	oligosaccharyl transferase stt3 protein, putative
TGME49_310030	-2.08	7.62	5.18E-11	1.39E-09	cyclase-associated protein, putative
TGME49_226500	-4.91	6.63	5.42E-11	1.45E-09	hypothetical protein
TGME49_278815	-3.98	6.59	5.80E-11	1.54E-09	hypothetical protein

TGME49_310750	-1.99	8.35	5.91E-11	1.57E-09	emp24/gp25L/p24 family protein
TGME49_313870	1.67	7.32	5.97E-11	1.58E-09	zinc finger, C3HC4 type (RING finger) domain-contain
TGME49_217420	-4.93	6.00	6.27E-11	1.65E-09	hypothetical protein
TGME49_294630	-2.14	7.75	6.38E-11	1.67E-09	hypothetical protein
TGME49_251800	2.31	5.67	7.19E-11	1.88E-09	hypothetical protein
TGME49_250330	-9.74	6.25	7.21E-11	1.88E-09	hypothetical protein
TGME49_217820	-2.54	7.71	7.27E-11	1.89E-09	PCI domain-containing protein
TGME49_289580	-9.72	6.04	7.36E-11	1.90E-09	strictosidine synthase subfamily protein
TGME49_226810	-3.51	6.80	7.46E-11	1.92E-09	histone lysine methyltransferase SET1
TGME49_313290	1.62	6.91	7.66E-11	1.97E-09	MORN repeat-containing protein
TGME49_245460	1.09	10.31	7.70E-11	1.97E-09	ribosomal protein RPS8
TGME49_316600	1.38	8.03	8.44E-11	2.15E-09	hypothetical protein
TGME49_255410	-4.73	6.62	8.48E-11	2.15E-09	hypothetical protein
TGME49_261620	2.13	5.83	8.73E-11	2.20E-09	hypothetical protein
TGME49_275610	-1.70	8.61	8.95E-11	2.25E-09	protein kinase, other
TGME49_261480	1.95	6.87	9.98E-11	2.50E-09	phosphatidyl serine synthase
TGME49_229690	-9.70	6.55	1.16E-10	2.90E-09	autophagy-related protein 7 atg7, putative
TGME49_310220	-4.80	6.48	1.18E-10	2.93E-09	hypothetical protein
TGME49_208450	1.40	8.60	1.22E-10	3.02E-09	protease inhibitor PI2
TGME49_220930	-2.68	7.43	1.40E-10	3.45E-09	hypothetical protein
TGME49_209060	-3.86	6.66	1.46E-10	3.61E-09	thrombospondin type 1 domain-containing protein
TGME49_280390	-2.94	6.88	1.47E-10	3.62E-09	HEAT repeat-containing protein
TGME49_202980	-2.59	7.20	1.55E-10	3.80E-09	hypothetical protein
TGME49_232280	-9.83	6.42	1.56E-10	3.80E-09	hypothetical protein
TGME49_265870	-3.49	6.97	1.57E-10	3.81E-09	pantoate-beta-alanine ligase
TGME49_278050	-2.32	7.70	1.59E-10	3.86E-09	proteasome subunit alpha type 1, putative
TGME49_295360	1.14	9.30	1.72E-10	4.14E-09	hypothetical protein
TGME49_219520	-2.28	7.68	1.73E-10	4.18E-09	histone arginine methyltransferase PRMT1
TGME49_284190	-4.12	6.62	1.75E-10	4.20E-09	pyruvate carboxylase
TGME49_261400	-2.00	7.86	1.90E-10	4.54E-09	hypothetical protein
TGME49_215390	2.61	4.82	1.97E-10	4.70E-09	TIM10 family protein, putative
TGME49_318770	-4.68	6.44	2.09E-10	4.97E-09	aurora kinase(incomplete catalytic triad)
TGME49_315780	-3.15	6.99	2.23E-10	5.28E-09	myosin regulatory light chain, putative
TGME49_257490	-3.88	6.62	2.34E-10	5.54E-09	prefoldin subunit superfamily protein
TGME49_301270	1.26	8.03	2.37E-10	5.58E-09	Tyrosine kinase-like (TKL) protein
TGME49_247360	2.39	7.48	2.58E-10	6.05E-09	PAP2 superfamily protein
TGME49_231120	1.93	6.29	2.64E-10	6.18E-09	ribosomal protein S11, putative
TGME49_282000	1.99	6.72	2.68E-10	6.26E-09	hypothetical protein
TGME49_258470	1.46	11.23	2.73E-10	6.36E-09	hypothetical protein
TGME49_240280	-2.24	7.78	2.88E-10	6.63E-09	S1/P1nuclease
TGME49_268176	-2.90	7.14	2.88E-10	6.63E-09	hypothetical protein
TGME49_275630	-2.31	7.61	3.13E-10	7.19E-09	HECT-domain (ubiquitin-transferase) domain-contain
TGME49_311460	-3.71	6.70	3.19E-10	7.29E-09	hypothetical protein
TGME49_225745	-9.66	6.00	3.27E-10	7.47E-09	hypothetical protein
TGME49_292160	1.89	6.10	3.43E-10	7.78E-09	hypothetical protein
TGME49_270930	-3.19	6.90	3.48E-10	7.87E-09	hypothetical protein
TGME49_263630	1.32	7.63	3.51E-10	7.92E-09	hypothetical protein
TGME49_320130	1.91	5.97	3.63E-10	8.16E-09	hypothetical protein
TGME49_264830	-4.61	6.49	3.84E-10	8.61E-09	hypothetical protein
TGME49_291940	-4.60	6.39	3.92E-10	8.74E-09	hypothetical protein
TGME49_259115	-3.62	6.99	4.03E-10	8.96E-09	ABC1 family protein
TGME49_221830	3.37	5.06	4.07E-10	8.98E-09	subtilisin SUB12
TGME49_250840	-9.60	6.18	4.07E-10	8.98E-09	hypothetical protein
TGME49_276970	-3.38	6.73	4.19E-10	9.22E-09	hypothetical protein
TGME49_250340	-2.25	7.79	4.21E-10	9.24E-09	centrin 2

TGME49_219730	1.51	7.57	4.25E-10	9.29E-09	hypothetical protein
TGME49_306060	-1.32	9.70	4.26E-10	9.29E-09	rhoptry neck protein RON8
TGME49_294640	-1.47	8.84	4.39E-10	9.55E-09	ribonucleoside-diphosphate reductase large chain
TGME49_316470	-3.66	6.01	4.42E-10	9.59E-09	hypothetical protein
TGME49_293740	1.93	8.89	4.56E-10	9.83E-09	hypothetical protein
TGME49_253750	1.94	8.98	4.68E-10	1.01E-08	PLU-1 family protein
TGME49_284598	-9.64	5.10	4.87E-10	1.04E-08	haloacid dehalogenase family hydrolase domain-cont
TGME49_297910	-4.84	5.66	5.14E-10	1.10E-08	hypothetical protein
TGME49_293190	-2.84	7.38	5.30E-10	1.13E-08	endonuclease/exonuclease/phosphatase family prote
TGME49_315530	-4.61	6.48	5.51E-10	1.17E-08	hypothetical protein
TGME49_210245	-9.73	5.61	5.72E-10	1.21E-08	hypothetical protein
TGME49_226705	-9.73	6.39	6.08E-10	1.28E-08	hypothetical protein
TGME49_205010	-2.22	7.49	6.29E-10	1.32E-08	U2 small nuclear ribonucleoprotein family protein, pu
TGME49_224540	-9.79	6.05	6.35E-10	1.33E-08	hypothetical protein
TGME49_268980	1.49	7.97	6.62E-10	1.39E-08	hypothetical protein
TGME49_294400	-2.37	7.26	6.67E-10	1.39E-08	hypothetical protein
TGME49_314410	-2.52	7.22	6.69E-10	1.39E-08	aquarius, putative
TGME49_243430	2.08	7.87	7.21E-10	1.49E-08	OTU family cysteine protease
TGME49_218530	-9.55	6.40	7.22E-10	1.49E-08	proteasome-interacting thioredoxin domain-containi
TGME49_200320	-2.54	7.39	7.57E-10	1.56E-08	hypoxanthine-xanthine-guanine phosphoribosyl trans
TGME49_214960	1.61	7.09	7.95E-10	1.64E-08	AP2 domain transcription factor AP2X-8
TGME49_258670	-9.80	5.62	8.08E-10	1.66E-08	hypothetical protein
TGME49_290580	-4.58	6.04	8.15E-10	1.67E-08	ATP-binding cassette G family transporter ABCG89
TGME49_305060	1.38	7.66	8.23E-10	1.68E-08	CAP-Gly domain-containing protein
TGME49_260800	1.71	6.32	8.25E-10	1.68E-08	hypothetical protein
TGME49_262380	-3.08	7.00	8.57E-10	1.74E-08	elongation factor Tu, putative
TGME49_253470	1.71	7.35	8.73E-10	1.76E-08	alveolin domain containing intermediate filament IM(
TGME49_244500	-2.01	7.47	8.74E-10	1.76E-08	Tubulin-tyrosine ligase family protein
TGME49_236860	-4.69	6.13	8.72E-10	1.76E-08	haloacid dehalogenase family hydrolase domain-cont
TGME49_297745	-4.28	6.99	9.01E-10	1.81E-08	hypothetical protein
TGME49_254410	1.74	6.78	9.08E-10	1.82E-08	protein phosphatase 2C, putative
TGME49_273050	1.75	6.54	9.29E-10	1.86E-08	hypothetical protein
TGME49_217400	-3.18	6.55	9.49E-10	1.89E-08	hypothetical protein
TGME49_203830	-3.78	6.38	9.64E-10	1.92E-08	FHA domain-containing protein
TGME49_214970	-1.69	8.91	1.00E-09	1.99E-08	DNA replication licensing factor, putative
TGME49_254030	2.17	8.18	1.01E-09	2.00E-08	zinc finger CDGSH-type domain-containing protein
TGME49_290200	-1.63	8.88	1.02E-09	2.01E-08	NAD/NADP octopine/nopaline dehydrogenase, alpha
TGME49_205580	1.39	7.01	1.03E-09	2.02E-08	nuclear factor NF4
TGME49_261750	-2.87	6.81	1.07E-09	2.10E-08	rhoptry neck protein RON10
TGME49_294870	1.00	9.50	1.13E-09	2.21E-08	universal stress family protein
TGME49_202920	-9.54	6.07	1.21E-09	2.35E-08	p-aminobenzoic acid synthase
TGME49_299780	1.64	9.06	1.29E-09	2.52E-08	hypothetical protein
TGME49_246930	-2.30	7.26	1.30E-09	2.52E-08	calmodulin CAM1
TGME49_231410	-2.14	8.15	1.33E-09	2.58E-08	hypothetical protein
TGME49_210230	-9.59	5.90	1.35E-09	2.60E-08	hypothetical protein
TGME49_213730	1.28	7.74	1.48E-09	2.85E-08	lanthionine synthetase C family protein
TGME49_259710	-3.08	7.07	1.49E-09	2.86E-08	protein kinase
TGME49_272410	1.54	6.75	1.53E-09	2.92E-08	phosphogluconate dehydrogenase (decarboxylating),
TGME49_305860	-2.23	7.93	1.53E-09	2.92E-08	calcium-dependent protein kinase CDPK3
TGME49_237550	2.61	6.56	1.57E-09	2.99E-08	hypothetical protein
TGME49_271820	-9.52	5.98	1.58E-09	3.00E-08	lipoyltransferase and lipoate-protein ligase subfamily
TGME49_320640	-9.58	6.10	1.59E-09	3.01E-08	peptidylprolyl isomerase domain-containing protein
TGME49_306338	-2.72	5.98	1.60E-09	3.02E-08	dynein gamma chain, flagellar outer arm, putative
TGME49_226590	1.73	9.66	1.64E-09	3.09E-08	cytochrome C oxidase subunit IIa, putative
TGME49_277720	-2.24	7.54	1.68E-09	3.15E-08	GDA1/CD39 (nucleoside phosphatase) family protein

TGME49_279340	1.85	6.26	1.72E-09	3.23E-08	hypothetical protein
TGME49_291040	4.27	6.75	1.76E-09	3.29E-08	lactate dehydrogenase LDH2
TGME49_288400	-9.50	6.03	1.76E-09	3.29E-08	LETM1 family protein
TGME49_245980	2.90	7.53	1.87E-09	3.48E-08	hypothetical protein
TGME49_289150	1.83	6.22	1.88E-09	3.49E-08	hypothetical protein
TGME49_286450	1.00	12.69	1.95E-09	3.61E-08	dense granule protein GRA5
TGME49_299250	2.76	6.36	2.07E-09	3.82E-08	hypothetical protein
TGME49_296010	-1.76	7.91	2.10E-09	3.87E-08	phosphatidylinositol 3- and 4-kinase
TGME49_200360	2.31	9.74	2.16E-09	3.96E-08	hypothetical protein
TGME49_235630	1.36	7.24	2.16E-09	3.96E-08	hypothetical protein
TGME49_274190	-4.60	6.59	2.21E-09	4.04E-08	eukaryotic initiation factor 2B epsilon subunit, putativ
TGME49_216430	-1.88	7.55	2.43E-09	4.43E-08	TBC domain-containing protein
TGME49_249230	-4.63	6.14	2.44E-09	4.45E-08	hypothetical protein
TGME49_264140	-2.24	7.26	2.46E-09	4.46E-08	hypothetical protein
TGME49_214200	-9.55	5.94	2.50E-09	4.53E-08	WD domain, G-beta repeat-containing protein
TGME49_246580	-3.68	6.71	2.62E-09	4.74E-08	hypothetical protein
TGME49_239260	3.42	4.79	2.81E-09	5.07E-08	histone H4
TGME49_310130	-9.44	6.05	2.85E-09	5.12E-08	Spc97 / Spc98 family protein
TGME49_268230	-9.74	6.51	2.85E-09	5.12E-08	hypothetical protein
TGME49_304740	1.11	8.67	2.93E-09	5.25E-08	rhoptry kinase family protein ROP35
TGME49_290720	-1.86	7.52	3.12E-09	5.57E-08	vacuolar proton translocating ATPase subunit, putativ
TGME49_305470	-9.47	5.79	3.17E-09	5.65E-08	hypothetical protein
TGME49_206550	3.26	6.05	3.20E-09	5.69E-08	hypothetical protein
TGME49_306660	1.30	7.88	3.21E-09	5.69E-08	RNA pseudouridine synthase superfamily protein
TGME49_261070	-3.67	6.54	3.31E-09	5.86E-08	apicoplast triosephosphate translocator APT1
TGME49_217020	-4.05	6.25	3.37E-09	5.94E-08	ATPase, AFG1 family protein
TGME49_237890	-4.49	6.17	3.37E-09	5.94E-08	calcium-dependent protein kinase CDPK4
TGME49_270270	-3.76	6.69	3.44E-09	6.04E-08	hypothetical protein
TGME49_312110	-2.43	7.05	3.54E-09	6.21E-08	apicoplast-associated thioredoxin family protein Atrx
TGME49_226260	1.38	7.26	3.59E-09	6.28E-08	hypothetical protein
TGME49_318525	-2.33	6.78	3.62E-09	6.31E-08	hypothetical protein
TGME49_297530	-9.43	6.26	3.71E-09	6.45E-08	DNA-directed RNA polymerase I RPA2
TGME49_294830	-3.42	6.45	3.94E-09	6.84E-08	methyltransferase domain-containing protein
TGME49_312960	1.72	6.29	4.19E-09	7.25E-08	hypothetical protein
TGME49_214575	1.73	5.96	4.25E-09	7.33E-08	hypothetical protein
TGME49_247300	1.39	8.35	4.25E-09	7.33E-08	hypothetical protein
TGME49_248990	1.70	8.43	4.37E-09	7.52E-08	hypothetical protein
TGME49_257350	1.35	8.60	4.52E-09	7.74E-08	eukaryotic translation initiation factor, putative
TGME49_289570	1.27	7.68	4.53E-09	7.74E-08	phosphatidylinositol transfer protein
TGME49_319370	-9.43	6.12	4.53E-09	7.74E-08	hypothetical protein
TGME49_285180	-1.87	7.32	4.61E-09	7.84E-08	hypothetical protein
TGME49_259010	-4.48	6.71	4.61E-09	7.84E-08	vacuolar ATP synthase subunit d, putative
TGME49_268225	-4.79	6.81	4.71E-09	7.98E-08	hypothetical protein
TGME49_293660	-3.66	6.63	4.75E-09	8.03E-08	hypothetical protein
TGME49_219080	-9.43	5.98	4.79E-09	8.06E-08	edge expressed protein, putative
TGME49_206690	-2.42	7.05	4.83E-09	8.12E-08	glideosome-associated protein with multiple-membra
TGME49_263540	2.32	5.09	5.03E-09	8.43E-08	hypothetical protein
TGME49_318310	-1.89	7.63	5.17E-09	8.65E-08	transketolase
TGME49_231950	1.71	7.62	5.25E-09	8.76E-08	hypothetical protein
TGME49_240860	-3.68	6.54	5.37E-09	8.93E-08	acyltransferase domain-containing protein
TGME49_286260	-9.40	5.83	5.38E-09	8.93E-08	tetratricopeptide repeat-containing protein
TGME49_257360	1.80	6.17	5.44E-09	9.03E-08	hypothetical protein
TGME49_289540	1.20	8.17	5.49E-09	9.06E-08	hypothetical protein
TGME49_319580	-9.39	5.99	5.49E-09	9.06E-08	hypothetical protein
TGME49_217550	1.24	7.73	5.61E-09	9.22E-08	hypothetical protein

TGME49_254370	-1.59	8.18	5.74E-09	9.41E-08	guanylyl cyclase
TGME49_276930	1.54	7.83	6.00E-09	9.79E-08	hypothetical protein
TGME49_265500	-9.38	6.01	6.04E-09	9.84E-08	chloride transporter, chloride channel (ClC) family pro
TGME49_292920	-1.88	7.96	6.32E-09	1.03E-07	heat shock protein 75, putative
TGME49_228750	-3.68	6.46	6.33E-09	1.03E-07	TGME49_228750 CAM kinase, RAD family
TGME49_202580	2.05	6.39	6.53E-09	1.06E-07	ATPase, AAA family protein
TGME49_227930	1.43	7.02	6.53E-09	1.06E-07	hypothetical protein
TGME49_318330	-9.40	5.85	6.62E-09	1.07E-07	histone lysine acetyltransferase MYST-A
TGME49_233410	2.08	5.64	6.66E-09	1.07E-07	Sof1 family domain-containing protein
TGME49_255700	-2.36	7.07	6.65E-09	1.07E-07	hypothetical protein
TGME49_240870	-2.54	7.10	6.99E-09	1.12E-07	beta adaptin protein, putative
TGME49_313020	-3.63	6.61	7.02E-09	1.12E-07	STAS domain-containing protein
TGME49_218362	-2.55	6.89	7.05E-09	1.12E-07	zinc finger protein ZFP1
TGME49_282220	1.53	6.58	7.18E-09	1.14E-07	AP2 domain transcription factor AP2VIIa-9
TGME49_207880	-9.56	6.72	7.36E-09	1.17E-07	hypothetical protein
TGME49_233000	-1.97	7.06	7.39E-09	1.17E-07	KOW motif domain-containing protein
TGME49_222960	-9.40	5.95	7.59E-09	1.20E-07	SCY kinase-related protein (incomplete catalytic triad
TGME49_320740	-2.10	7.64	7.90E-09	1.25E-07	hypothetical protein
TGME49_247460	-1.50	8.51	8.01E-09	1.26E-07	proliferating cell nuclear antigen PCNA1
TGME49_267690	-4.44	6.16	8.04E-09	1.26E-07	hypothetical protein
TGME49_203180	-4.41	5.86	8.14E-09	1.27E-07	leucine rich repeat-containing protein
TGME49_305240	-3.22	6.60	8.22E-09	1.28E-07	XPA binding protein 2 family protein
TGME49_207100	-9.35	5.81	8.51E-09	1.32E-07	hypothetical protein
TGME49_295658	-3.61	6.32	8.57E-09	1.33E-07	zinc finger in N-recognin protein
TGME49_206610	-3.26	6.76	8.63E-09	1.34E-07	pyruvate dehydrogenase complex subunit PDH-E2
TGME49_297890	1.72	6.67	8.70E-09	1.34E-07	hypothetical protein
TGME49_311070	-3.69	6.42	8.69E-09	1.34E-07	hypothetical protein
TGME49_213325	-9.41	5.93	8.77E-09	1.35E-07	TBC domain-containing protein
TGME49_263730	-2.70	6.40	9.59E-09	1.47E-07	FAD-dependent glycerol-3-phosphate dehydrogenase
TGME49_270620	2.77	6.19	9.74E-09	1.49E-07	DEAD/DEAH box helicase domain-containing protein
TGME49_202020	2.49	6.95	1.01E-08	1.55E-07	DnAK-TPR
TGME49_294940	1.57	6.95	1.06E-08	1.62E-07	hypothetical protein
TGME49_270770	-4.45	6.36	1.06E-08	1.62E-07	PWI domain-containing protein
TGME49_223760	-9.33	6.03	1.12E-08	1.71E-07	hypothetical protein
TGME49_248820	-9.38	4.70	1.20E-08	1.82E-07	hypothetical protein
TGME49_261970	1.55	7.59	1.22E-08	1.84E-07	hypothetical protein
TGME49_209280	-4.39	6.12	1.22E-08	1.84E-07	hypothetical protein
TGME49_202470	-9.34	5.84	1.23E-08	1.86E-07	rRNA metabolism protein, SBDS family protein
TGME49_222930	-2.69	6.77	1.35E-08	2.03E-07	hypothetical protein
TGME49_300048	1.38	7.17	1.39E-08	2.09E-07	hypothetical protein
TGME49_305090	1.86	6.45	1.43E-08	2.13E-07	kinase binding protein cgi-121 protein
TGME49_225250	1.74	5.93	1.42E-08	2.13E-07	LSU ribosomal protein L14P, putative
TGME49_321690	-3.66	6.68	1.42E-08	2.13E-07	hypothetical protein
TGME49_245730	-9.31	5.76	1.43E-08	2.13E-07	phosphatidylinositol-4-phosphate 5-Kinase
TGME49_223050	-1.25	9.44	1.49E-08	2.21E-07	ribosomal protein RPS20
TGME49_240570	-2.74	6.80	1.51E-08	2.23E-07	hypothetical protein
TGME49_319910	1.73	5.94	1.53E-08	2.25E-07	WD domain, G-beta repeat-containing protein
TGME49_271470	-4.36	6.12	1.53E-08	2.26E-07	hypothetical protein
TGME49_213067	1.18	7.78	1.54E-08	2.26E-07	hypothetical protein
TGME49_250690	-1.30	8.88	1.56E-08	2.29E-07	zinc finger (CCCH type) motif-containing protein
TGME49_293440	-1.84	7.35	1.70E-08	2.49E-07	hypothetical protein
TGME49_244700	-2.01	7.88	1.74E-08	2.53E-07	NAD(+)/NADH kinase domain-containing protein
TGME49_211470	2.49	5.17	1.76E-08	2.56E-07	Fcf2 pre-rRNA processing protein
TGME49_310530	-4.38	6.15	1.80E-08	2.62E-07	SNF2 family N-terminal domain-containing protein
TGME49_225770	-9.28	5.71	1.90E-08	2.75E-07	Tyrosine kinase-like (TKL) protein

TGME49_218560	-1.73	7.74	1.94E-08	2.80E-07	acetyl-coA carboxylase ACC2
TGME49_293710	-1.88	7.43	1.97E-08	2.84E-07	Zn-finger in Ran binding protein and others domain-c
TGME49_203560	-9.37	5.94	2.03E-08	2.92E-07	hypothetical protein
TGME49_216020	-9.29	6.06	2.04E-08	2.94E-07	peptidase family c78 protein
TGME49_296340	4.60	5.35	2.11E-08	3.02E-07	hypothetical protein
TGME49_212170	-9.28	5.94	2.11E-08	3.02E-07	GIY-YIG catalytic domain-containing protein
TGME49_265110	-9.30	6.03	2.13E-08	3.05E-07	ribosome biogenesis protein, putative
TGME49_311100	1.22	9.23	2.14E-08	3.06E-07	zinc finger (CCCH type) motif-containing protein
TGME49_224960	1.34	7.07	2.20E-08	3.14E-07	hypothetical protein
TGME49_264080	-1.65	8.18	2.24E-08	3.19E-07	acyl carrier protein ACP
TGME49_215590	-1.78	8.31	2.26E-08	3.20E-07	flavoprotein subunit of succinate dehydrogenase
TGME49_306310	-2.68	6.64	2.26E-08	3.20E-07	RecF/RecN/SMC N terminal domain-containing prote
TGME49_254720	1.02	9.52	2.28E-08	3.22E-07	dense granule protein GRA8
TGME49_313380	-1.15	9.56	2.31E-08	3.26E-07	hypothetical protein
TGME49_274060	-1.82	7.69	2.54E-08	3.58E-07	2-oxoglutarate/malate translocase OMT
TGME49_205360	-2.34	7.08	2.56E-08	3.60E-07	hypothetical protein
TGME49_235398	-9.26	5.77	2.62E-08	3.67E-07	hypothetical protein
TGME49_307820	1.95	5.87	2.67E-08	3.73E-07	hypothetical protein
TGME49_218750	-3.67	5.30	2.67E-08	3.73E-07	hypothetical protein
TGME49_236540	1.04	8.98	2.72E-08	3.79E-07	RNA recognition motif-containing protein
TGME49_271780	-3.11	6.36	2.72E-08	3.79E-07	Filamin/ABP280 repeat-containing protein
TGME49_225580	-9.25	5.94	2.72E-08	3.79E-07	proteasome (prosome, macropain) 26S subunit, non-
TGME49_251890	-4.36	5.68	2.83E-08	3.93E-07	hypothetical protein
TGME49_267500	1.01	8.83	2.96E-08	4.10E-07	hypothetical protein
TGME49_292975	-3.56	6.05	3.14E-08	4.34E-07	hypothetical protein
TGME49_290160	-1.61	8.18	3.20E-08	4.41E-07	sortilin, putative
TGME49_242055	-9.26	5.77	3.24E-08	4.46E-07	DEAD/DEAH box helicase domain-containing protein
TGME49_320440	-2.81	6.19	3.24E-08	4.46E-07	hypothetical protein
TGME49_253930	-2.02	6.95	3.27E-08	4.49E-07	GCC2 and GCC3 domain-containing protein
TGME49_315100	3.08	4.83	3.35E-08	4.59E-07	hypothetical protein
TGME49_232780	-3.16	6.30	3.55E-08	4.86E-07	hypothetical protein
TGME49_216770	-4.35	5.97	3.57E-08	4.87E-07	hypothetical protein
TGME49_229420	-9.23	6.04	3.66E-08	4.98E-07	cytochrome c, putative
TGME49_221720	-4.67	6.44	3.68E-08	4.99E-07	hypothetical protein
TGME49_262150	-9.22	5.98	3.76E-08	5.09E-07	kelch repeat and K+ channel tetramerisation domain
TGME49_220430	-9.22	5.67	3.86E-08	5.21E-07	hypothetical protein
TGME49_247580	-9.25	5.86	3.91E-08	5.27E-07	glutaredoxin domain-containing protein
TGME49_268880	-3.50	6.09	3.92E-08	5.27E-07	hypothetical protein
TGME49_225480	-4.30	5.99	3.96E-08	5.31E-07	hypothetical protein
TGME49_280730	-9.23	5.83	3.96E-08	5.31E-07	cytosolic fe-s cluster assembling factor nbp35, putati
TGME49_239590	-4.32	6.37	3.99E-08	5.34E-07	WD domain, G-beta repeat-containing protein
TGME49_202010	-3.08	6.47	4.03E-08	5.39E-07	hypothetical protein
TGME49_246570	2.11	5.60	4.06E-08	5.42E-07	hypothetical protein
TGME49_202280	-4.40	5.77	4.08E-08	5.44E-07	WD domain, G-beta repeat-containing protein
TGME49_267600	-3.08	6.33	4.12E-08	5.48E-07	FHA domain-containing protein
TGME49_307770	-2.20	6.60	4.19E-08	5.55E-07	fumble protein
TGME49_203730	-9.21	5.89	4.18E-08	5.55E-07	hypothetical protein
TGME49_207690	-4.30	6.36	4.26E-08	5.62E-07	programmed cell death 5 protein
TGME49_254000	1.26	7.06	4.32E-08	5.70E-07	hypothetical protein
TGME49_309380	-3.07	6.60	4.42E-08	5.81E-07	Nuf2
TGME49_229470	-4.34	6.35	4.48E-08	5.88E-07	hypothetical protein
TGME49_290900	-9.25	5.86	4.52E-08	5.93E-07	hydrolase, NUDIX family protein
TGME49_209730	-9.22	5.93	4.58E-08	5.99E-07	hypothetical protein
TGME49_223855	2.76	6.35	4.60E-08	6.01E-07	RNA recognition motif-containing protein
TGME49_266730	-9.20	5.67	4.64E-08	6.05E-07	leucyl-tRNA synthetase (LeuRS2)

TGME49_211250	-2.84	6.81	4.93E-08	6.39E-07	hypothetical protein
TGME49_285260	2.73	4.95	5.03E-08	6.51E-07	hypothetical protein
TGME49_234980	1.83	6.57	5.23E-08	6.76E-07	hypothetical protein
TGME49_319900	-2.15	7.18	5.48E-08	7.07E-07	hypothetical protein
TGME49_244280	-1.19	10.21	5.50E-08	7.07E-07	hypothetical protein
TGME49_227952	-2.43	6.97	5.50E-08	7.07E-07	14-3-3 superfamily protein
TGME49_249610	-2.58	6.80	5.52E-08	7.09E-07	hypothetical protein
TGME49_205200	1.28	6.83	5.56E-08	7.12E-07	hypothetical protein
TGME49_264600	-2.43	6.95	5.58E-08	7.12E-07	hypothetical protein
TGME49_237830	-9.23	5.84	5.57E-08	7.12E-07	DNA polymerase I domain-containing protein
TGME49_213790	-3.06	6.32	5.88E-08	7.49E-07	hypothetical protein
TGME49_254690	1.06	8.47	6.05E-08	7.69E-07	phospholipase/carboxylesterase
TGME49_217770	1.55	7.05	6.18E-08	7.82E-07	hypothetical protein
TGME49_257380	1.09	8.41	6.18E-08	7.82E-07	hypothetical protein
TGME49_215570	-3.51	6.18	6.19E-08	7.82E-07	AP2 domain transcription factor AP2X-11
TGME49_235560	-9.20	5.26	6.32E-08	7.97E-07	hypothetical protein
TGME49_246490	-2.01	7.46	6.53E-08	8.21E-07	hypothetical protein
TGME49_305980	-2.23	7.06	6.52E-08	8.21E-07	pyruvate dehydrogenase complex subunit PDH-E3I
TGME49_306440	-9.17	6.04	6.65E-08	8.35E-07	hypothetical protein
TGME49_214820	1.64	6.75	6.75E-08	8.46E-07	G-patch domain-containing protein
TGME49_258150	-1.69	7.78	6.92E-08	8.66E-07	proteasome subunit alpha type 7, putative
TGME49_295080	-9.17	5.79	7.06E-08	8.82E-07	hypothetical protein
TGME49_226220	-2.58	6.89	7.21E-08	8.99E-07	alveolin domain containing intermediate filament IM
TGME49_226430	-1.36	8.72	7.36E-08	9.16E-07	reticulon protein
TGME49_239780	-3.53	6.36	7.43E-08	9.23E-07	hypothetical protein
TGME49_243930	1.31	8.92	7.74E-08	9.59E-07	hypothetical protein
TGME49_226960	-1.13	9.46	7.74E-08	9.59E-07	phosphofructokinase PFKII
TGME49_315600	-9.28	5.50	7.83E-08	9.68E-07	MCM2/3/5 family protein
TGME49_310910	-9.25	5.61	8.02E-08	9.91E-07	WD domain, G-beta repeat-containing protein
TGME49_294970	1.33	8.63	8.27E-08	1.02E-06	hypothetical protein
TGME49_201150	-2.17	6.72	8.44E-08	1.04E-06	heavy metal translocating P-type ATPase subfamily p
TGME49_245560	1.10	7.95	8.62E-08	1.06E-06	hypothetical protein
TGME49_212140	-4.43	6.11	9.04E-08	1.11E-06	hypothetical protein
TGME49_211630	1.20	8.47	9.20E-08	1.12E-06	hypothetical protein
TGME49_264090	-3.49	6.14	9.19E-08	1.12E-06	hypothetical protein
TGME49_293900	1.09	8.11	9.25E-08	1.13E-06	sporozoite protein with an altered thrombospondin r
TGME49_282150	-9.14	5.86	9.28E-08	1.13E-06	hypothetical protein
TGME49_223800	-4.26	5.96	9.32E-08	1.13E-06	hypothetical protein
TGME49_259200	-4.23	6.05	9.58E-08	1.16E-06	Na+/H+ exchanger NHE1
TGME49_204310	-1.36	8.50	9.98E-08	1.21E-06	hypothetical protein
TGME49_288820	1.34	6.83	1.01E-07	1.22E-06	hypothetical protein
TGME49_260620	1.87	8.06	1.08E-07	1.31E-06	hypothetical protein
TGME49_310230	-9.18	5.63	1.09E-07	1.32E-06	hypothetical protein
TGME49_318650	1.15	8.31	1.10E-07	1.32E-06	transhydrogenase
TGME49_312622	-2.27	7.05	1.11E-07	1.33E-06	DUF803 domain-containing protein
TGME49_231130	-9.12	5.70	1.11E-07	1.33E-06	hypothetical protein
TGME49_251170	2.39	6.41	1.13E-07	1.35E-06	KRUF family protein
TGME49_282040	-4.28	5.99	1.13E-07	1.35E-06	hypothetical protein
TGME49_278630	-9.11	5.70	1.20E-07	1.43E-06	tetratricopeptide repeat-containing protein
TGME49_234440	-4.26	5.42	1.22E-07	1.45E-06	aminotransferase, putative
TGME49_278720	-9.16	5.75	1.26E-07	1.50E-06	hypothetical protein
TGME49_219700	-1.55	8.41	1.27E-07	1.51E-06	DNA replication licensing factor MCM4, putative
TGME49_288010	3.19	5.10	1.28E-07	1.51E-06	hypothetical protein
TGME49_280800	-1.86	7.39	1.29E-07	1.52E-06	SWI2/SNF2 SRCAP/Ino80
TGME49_201400	-4.23	6.24	1.30E-07	1.53E-06	Sin3-associated polypeptide SAP18

TGME49_227420	-9.15	5.89	1.30E-07	1.54E-06	4-hydroxy-3-methylbut-2-enyl diphosphate reductase
TGME49_239720	-9.11	5.49	1.34E-07	1.57E-06	50S ribosomal protein l24, putative
TGME49_239890	1.15	7.77	1.41E-07	1.65E-06	SCP family extracellular subfamily protein
TGME49_209130	-9.11	5.70	1.41E-07	1.66E-06	regulator of chromosome condensation (RCC1) repea
TGME49_209930	-9.20	5.30	1.42E-07	1.66E-06	hypothetical protein
TGME49_230950	-9.15	5.62	1.43E-07	1.67E-06	hypothetical protein
TGME49_313445	1.67	5.72	1.45E-07	1.69E-06	hypothetical protein
TGME49_205340	1.26	9.84	1.47E-07	1.71E-06	ribosomal protein RPS12
TGME49_253360	1.32	7.00	1.49E-07	1.73E-06	hypothetical protein
TGME49_204280	1.27	7.67	1.54E-07	1.78E-06	cell-cycle-associated protein kinase DYRK, putative
TGME49_231980	-9.12	5.84	1.54E-07	1.78E-06	hypothetical protein
TGME49_254870	1.24	6.87	1.57E-07	1.81E-06	hypothetical protein
TGME49_216070	-9.09	5.74	1.57E-07	1.81E-06	hypothetical protein
TGME49_232270	-9.16	5.79	1.58E-07	1.81E-06	histidine acid phosphatase superfamily protein
TGME49_249530	-1.59	7.70	1.61E-07	1.85E-06	exportin 1, putative
TGME49_278975	-2.23	6.87	1.68E-07	1.92E-06	ICE family protease (caspase) p20 domain-containing
TGME49_293730	-1.95	7.07	1.74E-07	1.99E-06	DHHC zinc finger domain-containing protein
TGME49_217410	-9.14	4.73	1.75E-07	2.00E-06	hypothetical protein
TGME49_297780	-2.98	6.07	1.76E-07	2.01E-06	ATPase/histidine kinase/DNA gyrase B/HSP90 domain
TGME49_269930	-1.97	6.51	1.79E-07	2.04E-06	calcium binding egf domain-containing protein
TGME49_212100	-9.12	4.92	1.79E-07	2.04E-06	ThiF family protein
TGME49_258480	-2.15	6.81	1.81E-07	2.05E-06	hypothetical protein
TGME49_249650	-9.09	5.91	1.81E-07	2.06E-06	apolipoprotein A-I binding protein, putative
TGME49_243580	-2.99	7.20	1.84E-07	2.08E-06	Hit family protein involved in cell-cycle regulation, pu
TGME49_310010	-1.42	8.26	1.84E-07	2.08E-06	rhoptry neck protein RON1
TGME49_289730	-2.12	7.06	1.85E-07	2.09E-06	Pep3/Vps18/deep orange family protein
TGME49_297840	-2.00	7.20	1.86E-07	2.09E-06	DNA primase, large subunit
TGME49_286270	-2.25	6.58	1.85E-07	2.09E-06	hypothetical protein
TGME49_227650	-2.52	6.80	1.86E-07	2.09E-06	microtubule-associated protein RP/EB family, putativ
TGME49_203760	1.63	6.32	1.90E-07	2.13E-06	hypothetical protein
TGME49_205280	1.20	7.65	1.92E-07	2.15E-06	hypothetical protein
TGME49_249360	-2.22	6.90	1.94E-07	2.17E-06	RED family protein
TGME49_309220	-1.77	7.36	2.15E-07	2.40E-06	GTPase activating protein for Arf protein
TGME49_285870	1.51	9.32	2.18E-07	2.42E-06	SAG-related sequence SRS20A
TGME49_205540	-9.08	5.64	2.18E-07	2.42E-06	DEAD/DEAH box helicase domain-containing protein
TGME49_211410	-4.13	5.74	2.24E-07	2.49E-06	translation initiation factor sui1 protein
TGME49_225690	-1.43	8.01	2.27E-07	2.51E-06	hypothetical protein
TGME49_260670	-9.06	5.92	2.27E-07	2.51E-06	centrin, putative
TGME49_264840	-9.06	5.33	2.31E-07	2.55E-06	ATP-dependent DNA helicase, RecQ family protein
TGME49_221440	-9.14	5.45	2.32E-07	2.56E-06	RPGR, putative
TGME49_252070	2.10	5.12	2.38E-07	2.62E-06	KRUF family protein
TGME49_312300	-1.75	7.41	2.39E-07	2.63E-06	Sec7 domain-containing protein
TGME49_264780	-9.08	5.52	2.40E-07	2.64E-06	UTP-glucose-1-phosphate uridylyltransferase subfam
TGME49_227060	-9.07	5.72	2.44E-07	2.68E-06	hypothetical protein
TGME49_262930	-9.04	5.80	2.52E-07	2.76E-06	hypothetical protein
TGME49_311870	-2.50	6.63	2.53E-07	2.77E-06	WD domain, G-beta repeat-containing protein
TGME49_286790	-4.13	6.16	2.59E-07	2.83E-06	nuclear factor NF2
TGME49_218880	-2.02	7.48	2.67E-07	2.91E-06	SF-assemblin, putative
TGME49_226580	1.19	7.99	2.68E-07	2.91E-06	hypothetical protein
TGME49_294270	-9.02	5.61	2.68E-07	2.91E-06	histone arginine methyltransferase PRMT4/CARM1
TGME49_270670	2.08	5.21	2.70E-07	2.93E-06	hypothetical protein
TGME49_278850	-1.38	7.92	2.74E-07	2.96E-06	DHHC zinc finger domain-containing protein
TGME49_228190	-1.56	7.95	2.79E-07	3.01E-06	eukaryotic initiation factor-3, subunit 5, putative
TGME49_285970	-9.02	5.58	2.88E-07	3.10E-06	30S ribosomal protein S5, putative
TGME49_301380	-4.18	6.01	2.88E-07	3.11E-06	elongation factor Tu GTP binding domain-containing

TGME49_209210	-2.20	6.84	2.90E-07	3.12E-06	hypothetical protein
TGME49_281910	1.57	6.18	2.94E-07	3.16E-06	hypothetical protein
TGME49_309870	-9.03	5.78	2.98E-07	3.19E-06	hypothetical protein
TGME49_306600	-9.05	5.73	2.98E-07	3.19E-06	RNA recognition motif-containing protein
TGME49_311680	-1.73	7.31	3.00E-07	3.21E-06	FUN14 family protein
TGME49_247760	-2.70	6.83	3.08E-07	3.29E-06	AMP-binding enzyme domain-containing protein
TGME49_292235	-9.04	5.58	3.10E-07	3.31E-06	hypothetical protein
TGME49_314920	2.19	5.56	3.12E-07	3.32E-06	hypothetical protein
TGME49_281510	-3.35	6.07	3.15E-07	3.35E-06	ribonuclease H1 large subunit, putative
TGME49_238950	-1.75	9.10	3.17E-07	3.37E-06	fatty acyl-CoA desaturase, putative
TGME49_248530	-9.02	5.80	3.21E-07	3.40E-06	FATC domain-containing protein
TGME49_253020	-2.98	6.02	3.28E-07	3.48E-06	hypothetical protein
TGME49_260320	-2.93	6.27	3.36E-07	3.56E-06	Noc2p family protein
TGME49_238100	-2.04	7.30	3.47E-07	3.66E-06	transmembrane protein
TGME49_286650	-9.00	5.60	3.49E-07	3.68E-06	hypothetical protein
TGME49_285490	-9.04	5.57	3.53E-07	3.72E-06	helix-hairpin-helix motif domain-containing protein
TGME49_318750	1.70	5.79	3.54E-07	3.73E-06	deoxyribose-phosphate aldolase
TGME49_297800	-2.72	6.62	3.59E-07	3.78E-06	RecF/RecN/SMC N terminal domain-containing prote
TGME49_240800	-2.11	6.86	3.69E-07	3.87E-06	MORN repeat-containing protein
TGME49_290980	-2.49	6.72	3.71E-07	3.89E-06	glycine C-acetyltransferase, putative
TGME49_310460	-2.51	7.01	3.86E-07	4.03E-06	Rab6
TGME49_237000	-4.10	5.99	3.87E-07	4.04E-06	polyphosphoinositide binding protein, putative
TGME49_296015	1.78	6.18	3.88E-07	4.04E-06	hypothetical protein
TGME49_209940	-2.39	6.24	3.89E-07	4.04E-06	transporter/permease protein
TGME49_314540	-8.99	5.67	3.91E-07	4.06E-06	hypothetical protein
TGME49_297900	1.69	7.58	3.93E-07	4.07E-06	hypothetical protein
TGME49_249590	-1.64	7.72	3.95E-07	4.10E-06	proteasome subunit alpha type 5-2, putative
TGME49_273030	-3.34	5.76	3.99E-07	4.13E-06	phosphoglycerate mutase family protein
TGME49_312430	1.71	6.02	4.18E-07	4.31E-06	hypothetical protein
TGME49_208500	-2.69	6.45	4.18E-07	4.31E-06	protein phosphatase 2C domain-containing protein
TGME49_311390	2.33	5.11	4.26E-07	4.39E-06	tRNA (guanine(9)-N(1))-methyltransferase
TGME49_274010	-4.10	6.06	4.29E-07	4.41E-06	hypothetical protein
TGME49_221580	-1.57	7.41	4.36E-07	4.48E-06	ribosomal RNA large subunit methyltransferase J pro
TGME49_306520	1.87	7.15	4.38E-07	4.49E-06	tRNA pseudouridine synthase B, putative
TGME49_246460	-4.13	5.93	4.44E-07	4.54E-06	hypothetical protein
TGME49_240520	-9.00	5.59	4.44E-07	4.54E-06	hypothetical protein
TGME49_205380	-1.55	7.80	4.48E-07	4.57E-06	fructose-bisphospatase I
TGME49_271760	-8.98	5.72	4.57E-07	4.66E-06	seryl-tRNA synthetase (SerRS2)
TGME49_222710	-2.47	6.78	4.63E-07	4.72E-06	IMP-specific 5'-nucleotidase 1, putative
TGME49_205040	-1.55	7.70	4.69E-07	4.77E-06	PGAP1 family protein
TGME49_270550	-9.05	5.78	4.78E-07	4.86E-06	gamma-glutamyl phosphate reductase, putative
TGME49_280500	-4.11	4.70	4.83E-07	4.90E-06	inorganic anion transporter, sulfate permease (SulP)
TGME49_217688	-8.98	5.67	4.84E-07	4.90E-06	hypothetical protein
TGME49_262950	1.23	7.72	4.87E-07	4.93E-06	hypothetical protein
TGME49_283790	-1.79	7.20	4.89E-07	4.94E-06	protein kinase, putative
TGME49_221295	-4.08	5.92	4.90E-07	4.94E-06	hypothetical protein
TGME49_310140	-8.97	5.76	5.03E-07	5.07E-06	hypothetical protein
TGME49_289300	-1.85	7.33	5.09E-07	5.12E-06	methionyl-tRNA synthetase
TGME49_270800	-4.18	6.03	5.50E-07	5.52E-06	GAF domain-containing protein
TGME49_266400	-9.03	5.57	5.57E-07	5.58E-06	hypothetical protein
TGME49_249740	1.72	6.54	5.85E-07	5.85E-06	translation machinery associated tma7 protein
TGME49_292140	1.16	7.59	5.94E-07	5.93E-06	NIMA-related protein kinase NIMA1
TGME49_316200	1.42	6.43	5.97E-07	5.95E-06	phosphoglycerate mutase family protein
TGME49_249470	-8.94	5.77	6.00E-07	5.97E-06	rhoptry kinase family protein, truncated (incomplete
TGME49_209985	-2.18	6.45	6.04E-07	6.00E-06	cAMP-dependent protein kinase

TGME49_265010	-8.94	5.64	6.06E-07	6.01E-06	glutamate 5-kinase domain-containing protein
TGME49_251440	-9.06	5.79	6.07E-07	6.01E-06	troponin c, isotype gamma, putative
TGME49_221630	-3.28	5.92	6.09E-07	6.03E-06	hypothetical protein
TGME49_201780	1.32	10.53	6.10E-07	6.03E-06	microneme protein MIC2
TGME49_249840	-8.94	5.34	6.28E-07	6.20E-06	dynein heavy chain 2, putative
TGME49_214100	1.37	7.24	6.42E-07	6.33E-06	hypothetical protein
TGME49_223450	-1.97	6.86	6.43E-07	6.33E-06	ubiquitin carboxyl-terminal hydrolase
TGME49_224020	1.50	6.33	6.49E-07	6.38E-06	hypothetical protein
TGME49_206600	-8.94	5.40	6.49E-07	6.38E-06	sigma-70, region 3 protein
TGME49_269290	-2.87	6.26	6.51E-07	6.38E-06	hypothetical protein
TGME49_206480	1.40	7.77	6.54E-07	6.40E-06	hypothetical protein
TGME49_269450	1.94	5.12	6.67E-07	6.52E-06	hypothetical protein
TGME49_304700	-4.04	5.84	6.71E-07	6.55E-06	hypothetical protein
TGME49_315860	-2.27	6.65	6.76E-07	6.59E-06	EF hand domain-containing protein
TGME49_323310	2.03	5.74	6.84E-07	6.66E-06	hypothetical protein
TGME49_203350	-9.01	5.69	6.96E-07	6.77E-06	hypothetical protein
TGME49_206695	2.04	5.29	6.97E-07	6.77E-06	hypothetical protein
TGME49_293760	1.11	8.17	7.16E-07	6.93E-06	EF hand domain-containing protein
TGME49_237530	-8.94	5.45	7.16E-07	6.93E-06	hypothetical protein
TGME49_298070	-9.23	5.01	7.16E-07	6.93E-06	hypothetical protein
TGME49_274110	-8.93	5.68	7.26E-07	7.02E-06	glycoprotease family protein
TGME49_247220	1.11	8.50	7.38E-07	7.11E-06	nudix -type motif 9 isoform a family protein
TGME49_231480	-1.68	7.27	7.54E-07	7.26E-06	GCN1, putative
TGME49_250220	2.42	5.45	7.61E-07	7.32E-06	hypothetical protein
TGME49_205000	-8.94	5.59	7.63E-07	7.32E-06	phosphoglycerate mutase family protein
TGME49_243298	-8.97	5.50	7.63E-07	7.32E-06	ICE family protease (caspase) p20 domain-containing
TGME49_288360	-1.31	10.08	7.68E-07	7.34E-06	tryptophanyl-tRNA synthetase (TrpRS2)
TGME49_202572	-8.93	5.64	7.68E-07	7.34E-06	ribophorin i protein
TGME49_207480	-2.40	6.41	7.69E-07	7.35E-06	GCC2 and GCC3 domain-containing protein
TGME49_293860	-8.93	5.58	7.71E-07	7.36E-06	hypothetical protein
TGME49_204420	3.11	6.08	7.78E-07	7.41E-06	oocyst wall protein OWP1
TGME49_288340	-8.91	5.69	7.85E-07	7.47E-06	UBX domain-containing protein
TGME49_220870	1.88	5.64	8.16E-07	7.76E-06	hypothetical protein
TGME49_291680	-1.47	7.77	8.20E-07	7.78E-06	Sec23/Sec24 trunk domain-containing protein
TGME49_275330	-4.05	5.64	8.25E-07	7.82E-06	ribosomal protein RPL29
TGME49_319312	2.19	5.71	8.39E-07	7.91E-06	hypothetical protein
TGME49_254230	1.91	5.65	8.40E-07	7.91E-06	hypothetical protein
TGME49_269010	1.25	9.54	8.40E-07	7.91E-06	AP2 domain transcription factor AP2VIII-7
TGME49_313880	-8.94	5.91	8.39E-07	7.91E-06	nuclear protein-like family protein
TGME49_259090	1.29	6.78	8.42E-07	7.91E-06	ubiquitin-conjugating enzyme subfamily protein
TGME49_241890	-8.91	5.67	8.42E-07	7.91E-06	hypothetical protein
TGME49_256880	-8.93	5.54	8.45E-07	7.93E-06	protein kinase domain-containing protein
TGME49_306460	-8.92	5.67	8.62E-07	8.06E-06	bromodomain-containing protein
TGME49_251840	1.40	6.35	9.01E-07	8.41E-06	hypothetical protein
TGME49_213060	1.34	6.59	9.01E-07	8.41E-06	WD domain, G-beta repeat-containing protein
TGME49_244230	-8.91	5.32	9.02E-07	8.41E-06	hypothetical protein
TGME49_252500	1.20	7.74	9.22E-07	8.57E-06	polo kinase
TGME49_239700	-3.30	5.88	9.28E-07	8.62E-06	regulator of chromosome condensation (RCC1) repea
TGME49_247380	-8.91	5.36	9.47E-07	8.78E-06	hypothetical protein
TGME49_208970	-1.94	6.92	9.52E-07	8.82E-06	RNA recognition motif-containing protein
TGME49_299210	1.03	8.29	9.55E-07	8.83E-06	CTP synthase
TGME49_229370	-4.00	5.92	9.63E-07	8.90E-06	AP2 domain transcription factor AP2VIII-1
TGME49_319640	1.11	8.46	9.77E-07	9.02E-06	hypothetical protein
TGME49_316250	2.06	8.50	9.83E-07	9.05E-06	hypothetical protein
TGME49_264130	1.68	5.62	1.00E-06	9.22E-06	hypothetical protein

TGME49_278950	1.84	5.34	1.01E-06	9.23E-06	LSM domain-containing protein
TGME49_214580	-4.05	5.36	1.03E-06	9.38E-06	tetratricopeptide repeat-containing protein
TGME49_248860	1.77	5.75	1.04E-06	9.50E-06	hypothetical protein
TGME49_307570	-1.29	7.41	1.04E-06	9.50E-06	glycerol-3-phosphate dehydrogenase (gpdh), putative
TGME49_279430	-2.90	6.25	1.04E-06	9.50E-06	cwf18 pre-mRNA splicing factor protein
TGME49_213400	-2.62	6.41	1.05E-06	9.51E-06	zinc finger (CCCH type) motif-containing protein
TGME49_269670	-3.25	5.91	1.05E-06	9.51E-06	hypothetical protein
TGME49_215420	-8.88	5.72	1.05E-06	9.51E-06	SNARE protein
TGME49_260580	-2.25	6.60	1.06E-06	9.57E-06	hypothetical protein
TGME49_268960	-3.24	6.09	1.06E-06	9.58E-06	5'-AMP-activated protein kinase subunit beta-1 famil
TGME49_248490	1.75	6.17	1.06E-06	9.58E-06	hypothetical protein
TGME49_249560	-8.94	5.35	1.06E-06	9.58E-06	DNA-directed RNA polymerase alpha chain rpoA
TGME49_271060	-4.00	5.87	1.07E-06	9.63E-06	Sec1 family protein
TGME49_222860	-1.30	8.81	1.09E-06	9.85E-06	eukaryotic translation initiation factor, putative
TGME49_289750	-1.04	9.50	1.11E-06	9.98E-06	ribosomal-ubiquitin protein RPL40
TGME49_311500	-8.99	5.49	1.17E-06	1.05E-05	ThiF family protein
TGME49_249250	1.13	10.07	1.17E-06	1.05E-05	ribosomal protein RPL35A
TGME49_260390	-8.88	5.56	1.18E-06	1.06E-05	hypothetical protein
TGME49_297510	-8.90	5.32	1.18E-06	1.06E-05	hypothetical protein
TGME49_278440	-2.00	6.87	1.19E-06	1.06E-05	SWI2/SNF2 Brahma-like putative
TGME49_259020	5.30	5.55	1.21E-06	1.08E-05	bradyzoite antigen BAG1
TGME49_234950	2.07	6.24	1.22E-06	1.09E-05	protein kinase (incomplete catalytic triad)
TGME49_263000	1.31	7.22	1.22E-06	1.09E-05	Beige/BEACH domain-containing protein
TGME49_215430	1.89	7.91	1.24E-06	1.10E-05	hypothetical protein
TGME49_257050	-8.85	5.79	1.24E-06	1.10E-05	3-methyl-2-oxobutanoate hydroxymethyltransferase
TGME49_223010	-2.84	6.45	1.28E-06	1.13E-05	hypothetical protein
TGME49_297400	1.14	7.21	1.28E-06	1.14E-05	hypothetical protein
TGME49_311250	-3.38	6.06	1.28E-06	1.14E-05	hypothetical protein
TGME49_235020	-1.55	7.85	1.29E-06	1.14E-05	COPI protein, putative
TGME49_255160	-8.88	5.35	1.30E-06	1.15E-05	hypothetical protein
TGME49_299200	1.45	7.09	1.30E-06	1.15E-05	Bet3 transport protein, putative
TGME49_245485	2.07	6.56	1.31E-06	1.16E-05	microneme protein MIC9
TGME49_248370	1.74	5.93	1.33E-06	1.17E-05	prefoldin subunit 6, putative
TGME49_289050	1.02	8.43	1.36E-06	1.20E-05	FIKK kinase, putative
TGME49_253640	1.24	6.73	1.38E-06	1.21E-05	hypothetical protein
TGME49_253000	1.21	6.99	1.39E-06	1.22E-05	ELMO/CED-12 family protein
TGME49_269660	-2.22	6.75	1.40E-06	1.22E-05	TFIIH basal transcription factor complex helicase XPB
TGME49_318690	2.02	5.00	1.43E-06	1.25E-05	RNA recognition motif-containing protein
TGME49_281400	-2.67	6.55	1.43E-06	1.25E-05	phosphofructokinase domain-containing protein
TGME49_265220	-4.04	5.88	1.43E-06	1.25E-05	co-chaperone GrpE protein
TGME49_318210	-8.87	5.53	1.46E-06	1.27E-05	hypothetical protein
TGME49_225800	-3.24	6.11	1.50E-06	1.30E-05	iron-sulfur assembly ATPase
TGME49_224310	-8.90	5.58	1.51E-06	1.31E-05	DHHC zinc finger domain-containing protein
TGME49_244000	-8.89	5.35	1.55E-06	1.34E-05	DEAD/DEAH box helicase domain-containing protein
TGME49_291050	-1.74	7.12	1.56E-06	1.35E-05	histone kinase SNF1, putative
TGME49_246970	-8.84	5.55	1.56E-06	1.35E-05	3'-5' exonuclease domain-containing protein
TGME49_320490	1.62	8.78	1.58E-06	1.36E-05	N-acyl-phosphatidylethanolamine-hydrolyzing phosp
TGME49_209700	-3.97	5.90	1.59E-06	1.36E-05	hypothetical protein
TGME49_297420	-4.00	5.82	1.58E-06	1.36E-05	beta-tubulin cofactor D, putative
TGME49_286750	-1.50	7.53	1.60E-06	1.37E-05	MA3 domain-containing protein
TGME49_221600	-8.86	5.26	1.60E-06	1.37E-05	hypothetical protein
TGME49_310802	1.67	5.93	1.62E-06	1.39E-05	CRAL/TRIO domain-containing protein
TGME49_313330	-9.05	5.76	1.63E-06	1.39E-05	rhoptry kinase family protein ROP27
TGME49_266450	1.31	6.38	1.67E-06	1.43E-05	lysine decarboxylase family protein
TGME49_215040	-2.23	6.64	1.68E-06	1.43E-05	HEAT repeat-containing protein

TGME49_210980	1.58	6.94	1.69E-06	1.44E-05	alternative splicing type 3 and, putative
TGME49_216510	-3.95	5.85	1.71E-06	1.46E-05	thioredoxin, putative
TGME49_306410	-3.96	5.79	1.75E-06	1.49E-05	hypothetical protein
TGME49_214750	1.14	7.32	1.77E-06	1.50E-05	hypothetical protein
TGME49_239270	-1.73	7.56	1.77E-06	1.50E-05	hypothetical protein
TGME49_311110	-3.95	5.89	1.79E-06	1.51E-05	Ubiquitin-fold modifier 1 precursor family protein, pu
TGME49_297870	-2.13	6.90	1.80E-06	1.52E-05	hypothetical protein
TGME49_316300	-8.83	5.17	1.85E-06	1.56E-05	ATP-dependent DNA helicase, RecQ family protein
TGME49_266470	-8.81	5.57	1.85E-06	1.56E-05	hypothetical protein
TGME49_310270	-4.08	5.89	1.88E-06	1.58E-05	hypothetical protein
TGME49_301160	-4.31	5.24	1.88E-06	1.58E-05	SAG-related sequence SRS19C
TGME49_234490	-2.81	6.15	1.94E-06	1.63E-05	kelch repeat-containing protein
TGME49_268210	-9.53	5.75	1.94E-06	1.63E-05	AGC kinase
TGME49_280370	1.17	9.15	1.98E-06	1.66E-05	hypothetical protein
TGME49_240370	8.07	4.77	1.99E-06	1.66E-05	Toxoplasma gondii family E protein
TGME49_314730	-3.97	5.86	2.00E-06	1.67E-05	ALG6, ALG8 glycosyltransferase family protein
TGME49_219810	1.50	8.82	2.02E-06	1.68E-05	hypothetical protein
TGME49_232560	-2.22	6.98	2.04E-06	1.70E-05	hypothetical protein
TGME49_313830	-2.78	6.34	2.08E-06	1.73E-05	AARP2CN (NUC121) domain-containing protein
TGME49_227600	-1.49	9.30	2.12E-06	1.75E-05	ribosomal protein RPL34
TGME49_216730	-1.23	8.59	2.15E-06	1.77E-05	MCM2/3/5 family protein
TGME49_281570	-3.93	5.67	2.18E-06	1.80E-05	hypothetical protein
TGME49_291090	-8.87	5.22	2.18E-06	1.80E-05	SWI2/SNF2-containing protein
TGME49_231200	-2.78	6.19	2.21E-06	1.82E-05	hypothetical protein
TGME49_207640	-1.63	7.49	2.22E-06	1.83E-05	isoleucyl-tRNA synthetase family protein
TGME49_239340	-8.79	5.64	2.27E-06	1.86E-05	hypothetical protein
TGME49_250950	2.04	5.35	2.30E-06	1.89E-05	KRUF family protein
TGME49_266990	-1.44	7.81	2.34E-06	1.92E-05	beta-COP
TGME49_259530	-3.93	5.96	2.37E-06	1.95E-05	GalNac
TGME49_312520	-4.02	5.37	2.49E-06	2.04E-05	tRNA dimethylallyltransferase
TGME49_202120	-1.54	7.31	2.50E-06	2.04E-05	hypothetical protein
TGME49_310930	-2.79	6.48	2.50E-06	2.04E-05	hypothetical protein
TGME49_288530	1.42	6.22	2.55E-06	2.07E-05	NOL1/NOP2/sun family protein
TGME49_320450	-3.24	6.15	2.57E-06	2.09E-05	ribosome biogenesis regulatory protein (rrs1) protein
TGME49_254910	-2.81	6.12	2.58E-06	2.10E-05	hypothetical protein
TGME49_248160	-8.80	5.47	2.58E-06	2.10E-05	hypothetical protein
TGME49_264720	-3.91	5.63	2.61E-06	2.12E-05	hypothetical protein
TGME49_270230	-8.80	5.30	2.62E-06	2.12E-05	hypothetical protein
TGME49_260450	-8.80	5.10	2.63E-06	2.13E-05	DEAD/DEAH box helicase domain-containing protein
TGME49_225050	-1.53	7.46	2.68E-06	2.17E-05	adenosylhomocysteinase, putative
TGME49_235478	-4.09	5.93	2.69E-06	2.17E-05	pantothenate kinase
TGME49_246100	-2.24	6.79	2.71E-06	2.18E-05	phosducin, putative
TGME49_224235	-8.78	5.14	2.75E-06	2.21E-05	translation initiation factor IF-3 protein
TGME49_213240	-8.83	5.32	2.75E-06	2.21E-05	hypothetical protein
TGME49_305120	1.18	7.12	2.76E-06	2.22E-05	transporter, solute:sodium symporter (SSS) family pro
TGME49_221180	1.04	8.38	2.79E-06	2.24E-05	hypothetical protein
TGME49_228730	-8.79	5.41	2.81E-06	2.25E-05	hypothetical protein
TGME49_313780	-1.52	7.72	2.85E-06	2.28E-05	hypothetical protein
TGME49_264730	-8.77	5.36	2.88E-06	2.30E-05	hypothetical protein
TGME49_264240	-9.04	4.81	2.92E-06	2.33E-05	hypothetical protein
TGME49_219770	-8.87	5.44	2.95E-06	2.36E-05	30S ribosomal protein S12, putative
TGME49_270090	-3.16	6.03	2.96E-06	2.36E-05	hypothetical protein
TGME49_270610	1.83	5.44	2.97E-06	2.36E-05	hypothetical protein
TGME49_284010	-3.91	5.48	3.03E-06	2.41E-05	5'-3' exonuclease, N-terminal resolvase family domai
TGME49_205700	1.01	10.37	3.08E-06	2.44E-05	cyclophilin precursor

TGME49_315250	-3.92	6.00	3.08E-06	2.44E-05	GAMM1 protein, putative
TGME49_315910	1.65	5.86	3.09E-06	2.45E-05	hypothetical protein
TGME49_205460	-8.74	5.47	3.12E-06	2.47E-05	AN1 family Zinc finger domain-containing protein
TGME49_272010	-3.93	6.12	3.14E-06	2.48E-05	Gar1 protein RNA binding region protein
TGME49_318290	-8.76	5.09	3.14E-06	2.48E-05	hypothetical protein
TGME49_229630	1.02	8.70	3.17E-06	2.50E-05	eIF2 kinase IF2K-A (incomplete catalytic triad)
TGME49_278830	-1.14	8.52	3.19E-06	2.50E-05	glucose-6-phosphate 1-dehydrogenase
TGME49_204380	-1.72	6.95	3.18E-06	2.50E-05	hypothetical protein
TGME49_230190	-1.94	6.88	3.19E-06	2.50E-05	hypothetical protein
TGME49_238140	-1.89	6.57	3.22E-06	2.52E-05	hypothetical protein
TGME49_306330	-2.15	6.75	3.27E-06	2.56E-05	phospholipase
TGME49_223640	-8.89	5.20	3.27E-06	2.56E-05	hypothetical protein
TGME49_212270	1.79	9.40	3.29E-06	2.57E-05	hypothetical protein
TGME49_222020	-2.53	6.35	3.32E-06	2.60E-05	phosphoglycerate kinase PGKII
TGME49_270140	-8.77	5.48	3.33E-06	2.60E-05	splicing factor DIM1, putative
TGME49_256840	-3.89	5.96	3.35E-06	2.61E-05	hypothetical protein
TGME49_237500	1.01	7.44	3.37E-06	2.62E-05	protein phosphatase 2C domain-containing protein
TGME49_313350	-3.88	5.75	3.37E-06	2.62E-05	hypothetical protein
TGME49_225990	-2.75	6.32	3.40E-06	2.64E-05	acyl transferase domain-containing protein
TGME49_249790	-3.89	5.87	3.42E-06	2.66E-05	hypothetical protein
TGME49_208590	-1.79	7.00	3.61E-06	2.80E-05	vacuolar ATP synthase subunit 54kD, putative
TGME49_306470	-2.47	6.39	3.63E-06	2.81E-05	isoprenylcysteine carboxyl methyltransferase (icmt) f
TGME49_272475	-8.78	5.24	3.67E-06	2.84E-05	protein kinase domain-containing protein
TGME49_270050	1.81	5.58	3.68E-06	2.84E-05	hypothetical protein
TGME49_291980	-1.53	7.41	3.68E-06	2.84E-05	HECT-domain (ubiquitin-transferase) domain-contain
TGME49_275990	1.31	6.58	3.80E-06	2.92E-05	hypothetical protein
TGME49_212150	-8.74	5.30	3.81E-06	2.93E-05	hypothetical protein
TGME49_267340	1.75	5.19	3.82E-06	2.93E-05	hypothetical protein
TGME49_214870	1.93	5.32	3.84E-06	2.94E-05	ribosomal protein L9, N-terminal domain-containing
TGME49_299900	-8.72	5.26	3.85E-06	2.95E-05	hypothetical protein
TGME49_286180	-8.73	4.90	3.85E-06	2.95E-05	tRNA ligases class I (M) protein
TGME49_227450	1.05	7.22	3.88E-06	2.96E-05	hydrolase, NUDIX family protein
TGME49_281990	1.70	5.47	3.93E-06	3.00E-05	Nicotinamidase
TGME49_318880	2.22	5.55	4.00E-06	3.05E-05	hypothetical protein
TGME49_300270	1.23	7.18	3.99E-06	3.05E-05	hypothetical protein
TGME49_273905	-3.86	6.09	4.00E-06	3.05E-05	hypothetical protein
TGME49_313270	-1.51	7.49	4.02E-06	3.06E-05	hypothetical protein
TGME49_253850	1.84	5.06	4.04E-06	3.07E-05	hypothetical protein
TGME49_294290	-1.98	7.07	4.12E-06	3.12E-05	Der1ER1
TGME49_310860	-2.48	6.37	4.12E-06	3.12E-05	U5 snRNP-specific protein
TGME49_207180	-8.79	5.29	4.12E-06	3.12E-05	indole-3-glycerol phosphate synthase domain-contai
TGME49_273370	-2.91	6.35	4.14E-06	3.13E-05	coatomer gamma 2-subunit protein, putative
TGME49_220330	-4.05	5.33	4.19E-06	3.17E-05	hypothetical protein
TGME49_316350	-8.75	5.45	4.20E-06	3.17E-05	hypothetical protein
TGME49_269075	-8.96	5.19	4.20E-06	3.17E-05	hypothetical protein
TGME49_253510	2.70	6.34	4.20E-06	3.17E-05	transporter/permease protein
TGME49_309890	-3.19	5.90	4.21E-06	3.17E-05	hypothetical protein
TGME49_216390	-3.12	5.89	4.23E-06	3.18E-05	RNA methyltransferase, TrmH family protein
TGME49_216500	-3.88	5.77	4.23E-06	3.18E-05	tRNA synthetase, putative
TGME49_318480	-2.48	6.16	4.29E-06	3.21E-05	SWI2/SNF2-containing protein RAD5
TGME49_207210	2.74	6.73	4.31E-06	3.23E-05	hypothetical protein
TGME49_261600	1.15	7.25	4.31E-06	3.23E-05	creatinase domain-containing protein
TGME49_300020	-3.85	5.75	4.33E-06	3.24E-05	ATP-dependent metallopeptidase HflB subfamily prot
TGME49_210760	-8.72	5.47	4.34E-06	3.24E-05	glutamine amidotransferase-related, putative
TGME49_232360	-1.87	6.79	4.38E-06	3.27E-05	exonuclease

TGME49_251710	2.12	5.03	4.42E-06	3.29E-05	hypothetical protein
TGME49_223510	-8.72	5.27	4.51E-06	3.36E-05	hypothetical protein
TGME49_310100	-8.73	5.39	4.65E-06	3.45E-05	mannosyltransferase (pig-m) protein
TGME49_219170	-1.93	6.59	4.65E-06	3.46E-05	hypothetical protein
TGME49_228490	-2.73	6.14	4.71E-06	3.49E-05	hypothetical protein
TGME49_267620	-2.52	6.97	4.71E-06	3.49E-05	multi-pass transmembrane protein
TGME49_286630	-1.41	7.93	4.77E-06	3.53E-05	redoxin domain-containing protein
TGME49_243250	-1.35	7.91	4.78E-06	3.53E-05	myosin H
TGME49_295960	1.65	7.35	4.82E-06	3.56E-05	hypothetical protein
TGME49_294020	-8.74	5.25	4.84E-06	3.57E-05	zinc finger, C3HC4 type (RING finger) domain-contain
TGME49_255250	-8.70	5.45	4.87E-06	3.58E-05	tRNA (cytosine(34)-C(5))-methyltransferase, putative
TGME49_259520	-8.72	5.47	4.88E-06	3.59E-05	hypothetical protein
TGME49_232160	1.17	6.85	5.01E-06	3.68E-05	zinc finger, C3HC4 type (RING finger) domain-contain
TGME49_270900	-3.24	6.04	5.02E-06	3.68E-05	ATPase, AAA family protein
TGME49_295420	1.47	7.87	5.05E-06	3.71E-05	hypothetical protein
TGME49_304460	-1.44	7.52	5.21E-06	3.82E-05	zinc finger, C3HC4 type (RING finger) domain-contain
TGME49_237480	-3.84	5.71	5.27E-06	3.85E-05	BRCA1 C Terminus (BRCT) domain-containing protein
TGME49_215290	-3.84	5.79	5.28E-06	3.86E-05	saccharopine dehydrogenase domain-containing prot
TGME49_289310	-2.16	6.67	5.39E-06	3.94E-05	cullin family protein
TGME49_225060	-2.45	6.46	5.40E-06	3.94E-05	nucleoredoxin family protein
TGME49_297720	-1.26	7.54	5.41E-06	3.94E-05	trehalose-phosphatase
TGME49_256770	-1.30	7.59	5.48E-06	3.98E-05	eukaryotic translation initiation factor 4A, isoform 3,
TGME49_263500	-3.88	5.93	5.50E-06	4.00E-05	vacuolar protein sorting-associated protein 26, putati
TGME49_268580	-3.87	6.06	5.52E-06	4.01E-05	hypothetical protein
TGME49_214370	-8.68	5.49	5.55E-06	4.02E-05	hypothetical protein
TGME49_231215	-8.68	5.41	5.55E-06	4.02E-05	hypothetical protein
TGME49_309110	1.29	6.61	5.58E-06	4.04E-05	tRNA methyl transferase
TGME49_208780	-2.72	6.22	5.61E-06	4.06E-05	ubiquitin-conjugating enzyme subfamily protein
TGME49_222192	-8.69	5.28	5.73E-06	4.14E-05	hypothetical protein
TGME49_214830	-3.85	5.63	5.80E-06	4.19E-05	hypothetical protein
TGME49_226280	2.12	5.02	5.91E-06	4.26E-05	ribosomal protein L28, putative
TGME49_255940	1.79	5.25	5.97E-06	4.30E-05	hypothetical protein
TGME49_285780	-8.75	5.31	5.99E-06	4.31E-05	hypothetical protein
TGME49_248450	-2.70	6.26	6.04E-06	4.34E-05	zinc finger, C3HC4 type (RING finger) domain-contain
TGME49_245590	1.89	5.36	6.07E-06	4.35E-05	rhomboid protease ROM6
TGME49_237290	-1.58	7.15	6.08E-06	4.35E-05	hypothetical protein
TGME49_229500	-3.82	5.70	6.08E-06	4.35E-05	hypothetical protein
TGME49_229490	-2.71	6.21	6.10E-06	4.37E-05	tetratricopeptide repeat-containing protein
TGME49_314000	1.47	6.33	6.12E-06	4.38E-05	peptide methionine sulfoxide reductase msrB, putati
TGME49_228100	-1.89	7.17	6.14E-06	4.38E-05	hypothetical protein
TGME49_271270	-1.54	7.33	6.15E-06	4.38E-05	hypothetical protein
TGME49_301440	-1.05	9.35	6.16E-06	4.38E-05	calcium-dependent protein kinase CDPK1
TGME49_306030	-1.69	7.04	6.17E-06	4.39E-05	glutathione s-transferase, n-terminal domain contain
TGME49_262040	-2.27	6.75	6.19E-06	4.40E-05	SAC3/GANP family protein
TGME49_208200	-3.86	5.78	6.21E-06	4.41E-05	PHD-finger domain-containing protein
TGME49_288290	1.85	5.66	6.34E-06	4.50E-05	hypothetical protein
TGME49_236250	1.47	5.67	6.34E-06	4.50E-05	regulator of chromosome condensation (RCC1) repea
TGME49_203840	1.53	5.77	6.35E-06	4.50E-05	DEAD/DEAH box helicase domain-containing protein
TGME49_201900	-3.84	5.71	6.38E-06	4.52E-05	hypothetical protein
TGME49_316680	1.10	7.77	6.43E-06	4.54E-05	RNA pseudouridine synthase superfamily protein
TGME49_229780	-3.83	5.86	6.43E-06	4.54E-05	GHMP kinase, N-terminal domain-containing protein
TGME49_305560	-3.83	5.81	6.47E-06	4.57E-05	Vps51/Vps67 protein
TGME49_272390	-8.67	5.13	6.48E-06	4.57E-05	hypothetical protein
TGME49_268890	-1.69	7.12	6.59E-06	4.64E-05	citrate synthase I
TGME49_274130	-8.65	5.27	6.68E-06	4.70E-05	TBC domain-containing protein

TGME49_225000	-1.48	7.35	6.71E-06	4.72E-05	hypothetical protein
TGME49_271430	-3.83	5.38	6.75E-06	4.74E-05	hypothetical protein
TGME49_268590	1.10	7.11	6.82E-06	4.78E-05	rhomboid protease ROM4
TGME49_248790	2.11	5.02	7.03E-06	4.92E-05	hypothetical protein
TGME49_228130	1.67	6.56	7.12E-06	4.97E-05	hypothetical protein
TGME49_242118	-8.65	5.88	7.22E-06	5.04E-05	myosin-light-chain kinase
TGME49_295030	1.07	8.10	7.26E-06	5.06E-05	hypothetical protein
TGME49_239620	-3.81	5.74	7.37E-06	5.13E-05	5'-nucleotidase, C-terminal domain-containing protei
TGME49_248570	-3.88	5.72	7.38E-06	5.14E-05	hypothetical protein
TGME49_260190	-1.70	6.56	7.42E-06	5.16E-05	microneme protein MIC13
TGME49_202620	1.00	8.38	7.49E-06	5.20E-05	hypothetical protein
TGME49_259210	-8.66	5.17	7.52E-06	5.22E-05	jmjC domain-containing protein C2orf60
TGME49_246190	-2.68	6.02	7.62E-06	5.28E-05	hypothetical protein
TGME49_216410	-8.77	5.36	7.67E-06	5.31E-05	hypothetical protein
TGME49_262050	1.21	9.15	7.80E-06	5.39E-05	rhoptry kinase family protein ROP39
TGME49_205750	-2.41	6.42	7.96E-06	5.49E-05	histone deacetylase complex subunit Sin3
TGME49_272770	-3.81	5.80	8.05E-06	5.55E-05	hypothetical protein
TGME49_294740	-2.10	6.49	8.09E-06	5.58E-05	hypothetical protein
TGME49_307610	-2.05	6.31	8.15E-06	5.61E-05	elongation factor TS, putative
TGME49_260830	-3.12	5.42	8.25E-06	5.68E-05	hypothetical protein
TGME49_268900	-8.63	5.16	8.41E-06	5.78E-05	dense granular protein GRA10
TGME49_254290	1.44	6.05	8.52E-06	5.85E-05	hypothetical protein
TGME49_244110	-1.41	8.05	8.61E-06	5.91E-05	nucleosome assembly protein (nap) protein
TGME49_220100	-2.35	6.73	8.64E-06	5.92E-05	phosphoribosylpyrophosphate synthetase
TGME49_260370	-2.21	6.27	8.83E-06	6.04E-05	AtPH1 family protein
TGME49_233140	-2.06	7.17	8.88E-06	6.07E-05	deoxyuridine 5'-triphosphate nucleotidohydrolase, pu
TGME49_252320	-8.67	5.00	8.89E-06	6.07E-05	Sas10/Utp3/C1D family protein
TGME49_247610	2.01	5.08	9.01E-06	6.14E-05	small nuclear ribonucleoprotein E, putative
TGME49_225470	1.29	6.28	9.00E-06	6.14E-05	peptide methionine sulfoxide reductase
TGME49_264740	1.01	7.94	9.02E-06	6.14E-05	hypothetical protein
TGME49_264220	-8.61	5.51	9.15E-06	6.22E-05	hypothetical protein
TGME49_289640	1.86	5.64	9.50E-06	6.45E-05	hypothetical protein
TGME49_289180	-8.64	5.18	9.51E-06	6.45E-05	thioredoxin family redox-active protein, putative
TGME49_318400	-1.56	7.52	9.55E-06	6.47E-05	hypothetical protein
TGME49_269705	2.18	5.10	9.65E-06	6.53E-05	hypothetical protein
TGME49_221500	-8.62	5.41	9.65E-06	6.53E-05	dual specificity phosphatase, catalytic domain-contai
TGME49_228770	-3.81	5.98	9.68E-06	6.54E-05	hypothetical protein
TGME49_229380	-8.60	5.38	9.80E-06	6.61E-05	hypothetical protein
TGME49_217450	1.95	4.96	9.85E-06	6.63E-05	general transcription factor IIH polypeptide 5 GTF2H5
TGME49_234220	-2.06	6.36	9.86E-06	6.64E-05	hypothetical protein
TGME49_235490	-1.64	7.03	9.88E-06	6.64E-05	hypothetical protein
TGME49_323110	1.44	6.09	9.92E-06	6.66E-05	hypothetical protein
TGME49_211040	-1.22	8.12	9.94E-06	6.66E-05	Sec61beta family protein
TGME49_273350	1.74	5.36	1.01E-05	6.77E-05	molybdopterin converting factor, subunit 2 protein
TGME49_277685	-8.69	5.11	1.01E-05	6.77E-05	hypothetical protein
TGME49_267420	1.12	8.10	1.02E-05	6.78E-05	mago nashi family protein 2, putative
TGME49_263750	-2.21	5.91	1.02E-05	6.78E-05	hypothetical protein
TGME49_275310	-3.07	5.96	1.02E-05	6.78E-05	hypothetical protein
TGME49_222290	-3.78	5.85	1.02E-05	6.78E-05	LSM domain-containing protein
TGME49_313400	-1.86	7.11	1.02E-05	6.79E-05	DnaJ domain-containing protein
TGME49_298840	1.42	6.20	1.04E-05	6.92E-05	hypothetical protein
TGME49_233790	-8.60	5.24	1.05E-05	6.97E-05	serine/threonine protein kinase AktR, putative
TGME49_320090	-2.61	6.48	1.05E-05	7.00E-05	hypothetical protein
TGME49_236570	-1.19	7.93	1.06E-05	7.00E-05	lysine decarboxylase family protein
TGME49_289000	-3.04	5.79	1.07E-05	7.05E-05	hypothetical protein

TGME49_211600	-2.40	6.15	1.08E-05	7.11E-05	hypothetical protein
TGME49_203450	-2.25	6.40	1.08E-05	7.12E-05	DUF3228 domain-containing protein
TGME49_264860	-3.77	5.69	1.08E-05	7.15E-05	zinc finger, C3HC4 type (RING finger) domain-contain
TGME49_217740	-1.89	6.97	1.09E-05	7.16E-05	3-ketoacyl-(acyl-carrier-protein) reductase
TGME49_269920	-1.18	8.62	1.09E-05	7.17E-05	phosphatidylserine decarboxylase
TGME49_205510	-1.43	7.70	1.09E-05	7.17E-05	nucleolar protein 5, putative
TGME49_224620	1.54	5.92	1.11E-05	7.31E-05	hypothetical protein
TGME49_262030	-8.59	4.93	1.11E-05	7.31E-05	ALG6, ALG8 glycosyltransferase family protein
TGME49_212840	-8.68	5.13	1.12E-05	7.32E-05	HIT zinc finger protein
TGME49_209240	1.50	5.74	1.13E-05	7.37E-05	RNA methyltransferase
TGME49_263610	-1.78	6.91	1.13E-05	7.39E-05	hypothetical protein
TGME49_224660	1.12	6.78	1.15E-05	7.52E-05	transcription factor s-ii (tfiis), central domain-contain
TGME49_227320	-8.68	5.44	1.17E-05	7.60E-05	hypothetical protein
TGME49_314070	1.40	6.32	1.17E-05	7.61E-05	hypothetical protein
TGME49_247250	-1.57	6.96	1.18E-05	7.68E-05	RbAp46
TGME49_286560	-8.68	5.40	1.18E-05	7.68E-05	U6 snRNA-associated Sm family protein
TGME49_216380	-2.49	6.35	1.21E-05	7.82E-05	phospholipid-translocating P-type ATPase, flippase su
TGME49_278700	-3.04	5.93	1.21E-05	7.82E-05	hypothetical protein
TGME49_259830	1.66	6.46	1.22E-05	7.90E-05	diacylglycerol kinase catalytic domain-containing pro
TGME49_216120	-2.20	6.42	1.24E-05	8.03E-05	hypothetical protein
TGME49_307575	-3.79	5.16	1.28E-05	8.26E-05	hypothetical protein
TGME49_245650	2.51	4.74	1.29E-05	8.34E-05	hypothetical protein
TGME49_249460	-8.57	5.36	1.29E-05	8.34E-05	WD domain, G-beta repeat-containing protein
TGME49_301350	-8.70	4.95	1.29E-05	8.34E-05	SNARE associated protein
TGME49_270720	-8.74	5.34	1.29E-05	8.34E-05	hypothetical protein
TGME49_230580	-8.56	5.21	1.31E-05	8.44E-05	hypothetical protein
TGME49_306300	-1.64	6.39	1.32E-05	8.48E-05	hypothetical protein
TGME49_243210	1.03	7.22	1.33E-05	8.56E-05	DUF862 domain-containing protein
TGME49_263210	-8.59	5.06	1.33E-05	8.56E-05	ubiquitin interaction motif domain-containing protei
TGME49_257340	-8.55	5.20	1.38E-05	8.83E-05	Ras family protein
TGME49_285980	1.15	8.53	1.38E-05	8.85E-05	glucosephosphate-mutase GPM1
TGME49_300055	1.21	7.81	1.39E-05	8.92E-05	hypothetical protein
TGME49_264690	-8.55	5.40	1.43E-05	9.13E-05	cyclin 4, putative
TGME49_244670	-1.63	6.91	1.43E-05	9.13E-05	hypothetical protein
TGME49_228200	1.13	7.28	1.45E-05	9.26E-05	vacuolar (h+)-atpase g subunit protein
TGME49_234390	-8.59	5.43	1.45E-05	9.26E-05	hypothetical protein
TGME49_294050	1.76	7.02	1.47E-05	9.37E-05	hypothetical protein
TGME49_244610	-3.73	5.46	1.48E-05	9.39E-05	zinc finger, C3HC4 type (RING finger) domain-contain
TGME49_229720	-3.02	5.39	1.48E-05	9.41E-05	hypothetical protein
TGME49_203060	1.09	7.36	1.49E-05	9.44E-05	hypothetical protein
TGME49_269650	-1.61	7.00	1.49E-05	9.47E-05	FFD and TFG box motifs protein
TGME49_270750	-2.27	6.29	1.50E-05	9.54E-05	hypothetical protein
TGME49_309950	-8.54	5.31	1.52E-05	9.64E-05	NLE (NUC135) domain-containing protein
TGME49_280410	-1.82	6.69	1.53E-05	9.68E-05	3'5'-cyclic nucleotide phosphodiesterase domain-con
TGME49_263100	-2.77	5.96	1.53E-05	9.69E-05	hypothetical protein
TGME49_275670	-1.25	8.05	1.54E-05	9.76E-05	alveolin domain containing intermediate filament IM
TGME49_312500	-1.57	7.05	1.56E-05	9.85E-05	hypothetical protein
TGME49_288930	-2.41	6.16	1.56E-05	9.86E-05	hypothetical protein
TGME49_202460	-1.92	6.49	1.58E-05	9.93E-05	diacylglycerol kinase accessory domain (presumed) d
TGME49_287270	-2.62	6.15	1.58E-05	9.93E-05	hypothetical protein
TGME49_236890	1.05	7.15	1.61E-05	0.000101415	hypothetical protein
TGME49_216130	-1.93	6.73	1.62E-05	0.000101752	ubiquitin conjugating enzyme E2, putative
TGME49_300380	-2.99	5.70	1.62E-05	0.000101844	endoplasmic reticulum oxidoreductin, putative
TGME49_272680	-8.53	5.08	1.63E-05	0.00010193	hypothetical protein
TGME49_223150	-8.53	5.15	1.65E-05	0.000103079	START domain-containing protein

TGME49_295850	-1.35	7.27	1.65E-05	0.000103369	cyclic nucleotide-binding domain-containing protein
TGME49_221690	-3.74	5.62	1.69E-05	0.000105698	hypothetical protein
TGME49_227350	-3.12	5.75	1.70E-05	0.000106013	hypothetical protein
TGME49_219460	-2.98	5.68	1.71E-05	0.000106427	hypothetical protein
TGME49_240540	-2.20	6.41	1.73E-05	0.000107996	hypothetical protein
TGME49_304470	-2.98	5.83	1.75E-05	0.000109031	oxidoreductase, putative
TGME49_297810	-1.31	7.88	1.79E-05	0.000111477	hypothetical protein
TGME49_213660	-1.12	9.72	1.80E-05	0.000111697	zinc finger (CCCH type) motif-containing protein
TGME49_253180	1.98	7.48	1.82E-05	0.000112467	hypothetical protein
TGME49_233500	1.19	8.04	1.82E-05	0.000112467	triose-phosphate isomerase TPI-II
TGME49_218910	2.04	5.24	1.82E-05	0.000112684	hypothetical protein
TGME49_217350	-8.53	5.39	1.82E-05	0.000112765	methyltransferase MTA70, putative
TGME49_213620	1.68	5.49	1.83E-05	0.00011278	ABC1 family protein
TGME49_264650	-8.61	4.90	1.83E-05	0.000112935	phosphoacetylglucosamine mutase
TGME49_221610	-2.20	6.25	1.84E-05	0.000113422	ubiquitin carboxyl-terminal hydrolase
TGME49_249820	-2.97	5.81	1.86E-05	0.000114634	ATP-binding cassette sub-family B member 5
TGME49_273815	-8.57	5.51	1.86E-05	0.000114773	hypothetical protein
TGME49_209820	-8.54	5.29	1.89E-05	0.000116158	syntaxin protein
TGME49_220450	-8.51	4.82	1.89E-05	0.000116228	ribonuclease HI protein
TGME49_261430	-2.17	5.96	1.90E-05	0.000116801	hypothetical protein
TGME49_329800	-1.74	6.84	1.91E-05	0.000117486	hypothetical protein
TGME49_222210	-8.50	5.46	1.92E-05	0.000117928	SPFH domain / Band 7 family protein
TGME49_280450	-8.54	4.79	1.94E-05	0.000118561	hypothetical protein
TGME49_307260	2.82	5.67	1.96E-05	0.000119856	Toxoplasma gondii family C protein
TGME49_215775	-1.20	10.26	1.97E-05	0.000120105	rhoptry protein ROP8
TGME49_306960	-2.15	6.60	2.01E-05	0.000122538	phenylalanine--tRNA ligase, beta subunit protein
TGME49_297140	-2.97	5.78	2.01E-05	0.000122538	U6 snRNA-associated sm family protein Lsm2, putativ
TGME49_233680	-8.49	5.02	2.01E-05	0.000122538	nuclear movement family protein
TGME49_271350	-8.50	5.41	2.02E-05	0.000122538	bifunctional protein FolC subfamily protein
TGME49_316570	1.05	7.16	2.02E-05	0.000122555	hypothetical protein
TGME49_251670	-3.69	5.58	2.06E-05	0.0001252	werner helicase interacting protein 1, putative
TGME49_311220	-2.25	6.31	2.07E-05	0.000125654	hypothetical protein
TGME49_261000	-1.56	7.03	2.10E-05	0.000126964	MutS domain V domain-containing protein
TGME49_310570	-8.60	5.49	2.10E-05	0.000126964	hypothetical protein
TGME49_254800	1.78	5.35	2.11E-05	0.00012753	hypothetical protein
TGME49_213880	2.67	4.99	2.14E-05	0.000129303	hypothetical protein
TGME49_246160	2.31	4.93	2.14E-05	0.00012945	hypothetical protein
TGME49_230350	-1.13	8.27	2.15E-05	0.000129988	hypothetical protein
TGME49_226420	-1.59	6.96	2.17E-05	0.000130942	peptidase family M3 protein
TGME49_219485	1.26	6.92	2.17E-05	0.000131063	hypothetical protein
TGME49_257945	2.51	4.77	2.21E-05	0.000133157	3'5'-cyclic nucleotide phosphodiesterase domain-con
TGME49_212930	2.47	5.60	2.24E-05	0.00013449	NifU family domain-containing protein
TGME49_271870	-8.47	5.32	2.24E-05	0.00013483	zinc carboxypeptidase superfamily protein
TGME49_224600	-3.70	5.60	2.27E-05	0.00013634	GTP binding protein
TGME49_243340	-8.47	5.12	2.36E-05	0.000141014	atypical MEK-related kinase (incomplete catalytic tria
TGME49_227010	-8.64	5.24	2.35E-05	0.000141014	rhoptry kinase family protein ROP30
TGME49_248870	1.25	6.40	2.36E-05	0.000141145	SNARE associated Golgi protein
TGME49_205300	1.24	6.67	2.38E-05	0.000142361	hypothetical protein
TGME49_299150	-8.50	5.15	2.39E-05	0.000142798	AP2 domain transcription factor AP2III-3
TGME49_310710	-3.67	5.64	2.41E-05	0.000143662	small ribosomal subunit Rsm22 protein
TGME49_310850	-8.54	5.31	2.41E-05	0.000143662	MYND finger domain-containing protein
TGME49_288800	3.24	6.83	2.43E-05	0.000144519	endonuclease/exonuclease/phosphatase family prote
TGME49_309130	-8.47	5.04	2.47E-05	0.00014694	hypothetical protein
TGME49_202030	-1.49	6.98	2.50E-05	0.000148406	hypothetical protein
TGME49_239560	-8.57	5.38	2.53E-05	0.000150281	myosin E

TGME49_310320	-3.68	5.64	2.55E-05	0.000150713	calreticulin family protein
TGME49_269320	-8.53	5.04	2.55E-05	0.000150713	hypothetical protein
TGME49_308880	-8.48	5.26	2.56E-05	0.000151358	ImpB/MucB/SamB family protein
TGME49_300290	-8.49	5.41	2.57E-05	0.000151842	SNARE domain-containing protein
TGME49_217360	-2.95	5.95	2.58E-05	0.000152247	hypothetical protein
TGME49_233760	-2.63	5.69	2.59E-05	0.000152684	hypothetical protein
TGME49_242800	-1.79	6.66	2.61E-05	0.000153773	ribosome biogenesis protein NSA2, putative
TGME49_265460	1.37	6.01	2.63E-05	0.000154666	hypothetical protein
TGME49_244510	-8.46	4.91	2.65E-05	0.000155823	AP2 domain transcription factor AP2VI-3
TGME49_228340	-8.49	5.00	2.67E-05	0.000157303	hypothetical protein
TGME49_281980	-1.49	6.99	2.79E-05	0.000163455	phosphatidate cytidylyltransferase
TGME49_227430	-1.89	6.17	2.79E-05	0.000163455	transmembrane amino acid transporter protein
TGME49_262980	-8.46	5.23	2.82E-05	0.000164979	hypothetical protein
TGME49_310670	-1.17	7.93	2.87E-05	0.000167675	glycogen phosphorylase 1, putative
TGME49_310560	-3.68	5.55	2.87E-05	0.000167675	hypothetical protein
TGME49_212860	1.11	6.72	2.89E-05	0.000168332	hypothetical protein
TGME49_264960	-8.46	5.17	2.89E-05	0.000168332	hypothetical protein
TGME49_309100	2.16	5.47	2.93E-05	0.000170048	hypothetical protein
TGME49_254606	2.48	5.92	2.94E-05	0.000170522	hypothetical protein
TGME49_233490	-8.54	5.10	2.97E-05	0.000172251	hypothetical protein
TGME49_219310	-1.29	8.62	2.97E-05	0.000172366	DnaK family protein
TGME49_219738	-1.76	6.44	3.00E-05	0.000173918	hypothetical protein
TGME49_285210	-8.51	5.26	3.01E-05	0.000173918	hypothetical protein
TGME49_223900	2.09	5.15	3.04E-05	0.000175457	hypothetical protein
TGME49_312530	1.09	6.83	3.05E-05	0.000176189	splicing factor, CC1 family protein
TGME49_268720	-2.96	5.82	3.06E-05	0.000176775	Hrf1 family protein
TGME49_234670	1.80	5.29	3.08E-05	0.000177639	actin-like family protein
TGME49_266750	-1.29	7.68	3.09E-05	0.000178333	transporter/permease protein, putative
TGME49_216170	-3.81	5.73	3.13E-05	0.000180303	SufS subfamily cysteine desulfurase
TGME49_242870	-8.42	5.35	3.16E-05	0.000181587	histone lysine methyltransferase, SET, putative
TGME49_231030	-3.65	5.64	3.16E-05	0.00018177	hypothetical protein
TGME49_240300	-8.47	5.18	3.20E-05	0.000183758	zinc finger domain, LSD1 subclass domain-containing
TGME49_272640	-1.99	6.34	3.20E-05	0.000183775	eukaryotic initiation factor-2B, epsilon subunit, putat
TGME49_221350	-3.63	5.68	3.22E-05	0.00018443	Ctr copper transporter family protein
TGME49_217750	-3.64	5.56	3.27E-05	0.000187373	hypothetical protein
TGME49_320550	-1.90	6.64	3.27E-05	0.000187504	hypothetical protein
TGME49_294190	-3.66	5.22	3.33E-05	0.000190148	enoyl-CoA hydratase/isomerase family protein
TGME49_293380	-8.48	5.33	3.33E-05	0.000190469	histone lysine acetyltransferase HAT1
TGME49_223570	1.23	7.66	3.35E-05	0.000191443	hypothetical protein
TGME49_321610	-8.43	4.89	3.36E-05	0.000191566	hypothetical protein
TGME49_226740	1.36	7.97	3.39E-05	0.00019296	zinc finger, C3HC4 type (RING finger) domain-contain
TGME49_265400	-1.73	6.69	3.39E-05	0.00019296	hypothetical protein
TGME49_314890	-3.63	5.41	3.39E-05	0.00019296	ThiF family protein
TGME49_235420	-1.30	7.24	3.40E-05	0.000193504	hypothetical protein
TGME49_321440	-1.15	7.84	3.41E-05	0.000193602	SWI2/SNF2 ISWI-like SANT
TGME49_250100	2.16	6.89	3.44E-05	0.000195198	hypothetical protein
TGME49_215210	2.39	6.31	3.46E-05	0.000195936	hypothetical protein
TGME49_235460	1.27	6.18	3.46E-05	0.000195936	hypothetical protein
TGME49_213890	-8.58	5.03	3.45E-05	0.000195936	Myb family DNA-binding domain-containing protein
TGME49_254380	-8.44	5.12	3.48E-05	0.000197086	ribosomal protein L11, putative
TGME49_214530	-2.02	6.38	3.53E-05	0.000199604	DnaJ domain-containing protein
TGME49_232020	-1.93	6.60	3.58E-05	0.000202099	hypothetical protein
TGME49_251680	-1.19	8.93	3.61E-05	0.000203821	histamine-releasing factor, putative
TGME49_290890	-2.29	6.43	3.64E-05	0.000204997	carbonyl reductase 1, putative
TGME49_204270	-2.35	6.05	3.64E-05	0.000204997	hypothetical protein

TGME49_246780	-8.42	4.74	3.64E-05	0.000204997	hypothetical protein
TGME49_230590	-8.41	5.22	3.67E-05	0.000206385	chitobiosyldiphosphodolichol beta-mannosyltransfera
TGME49_288710	1.75	5.08	3.67E-05	0.000206582	hypothetical protein
TGME49_210778	-1.95	6.36	3.68E-05	0.000206695	hemimethylated DNA binding domain-containing pro
TGME49_220460	-8.40	4.98	3.74E-05	0.000209642	SNF7 family protein
TGME49_215360	1.97	5.74	3.75E-05	0.000210466	hypothetical protein
TGME49_271460	1.54	6.76	3.81E-05	0.000213142	protein c14orf29, putative
TGME49_227030	-1.78	6.87	3.81E-05	0.000213142	hypothetical protein
TGME49_297230	-1.95	6.39	3.84E-05	0.000214396	Vps53 family, N-terminal protein
TGME49_262060	-2.90	5.97	3.85E-05	0.000214759	hypothetical protein
TGME49_246230	-8.45	5.20	3.87E-05	0.000216064	hypothetical protein
TGME49_297245	-1.42	7.00	3.99E-05	0.00022207	transporter, major facilitator family protein
TGME49_231910	-1.42	7.36	3.99E-05	0.00022207	ATP synthase F1 gamma subunit
TGME49_202950	-1.27	7.30	3.99E-05	0.000222131	hypothetical protein
TGME49_276170	1.53	9.19	4.02E-05	0.000223262	phosphatidylinositol 3- and 4-kinase
TGME49_225920	-2.52	5.83	4.09E-05	0.0002272	hypothetical protein
TGME49_321300	-3.61	5.37	4.16E-05	0.00023074	autophagy-related 12 variant 1, putative
TGME49_283850	-1.75	8.26	4.19E-05	0.000232242	peptidyl-prolyl cis-trans isomerase
TGME49_307605	-1.52	6.90	4.21E-05	0.000232885	hypothetical protein
TGME49_281900	-1.27	7.26	4.28E-05	0.000236494	SET domain containing lysine methyltransferase KMT
TGME49_312190	-1.66	6.85	4.29E-05	0.00023702	hypothetical protein
TGME49_206390	-8.38	4.99	4.29E-05	0.00023702	hypothetical protein
TGME49_248150	-3.61	5.73	4.31E-05	0.000237787	hypothetical protein
TGME49_277910	1.02	6.76	4.31E-05	0.000237802	thrombospondin type 1 domain-containing protein
TGME49_255390	-3.61	5.42	4.35E-05	0.000239606	HEAT repeat-containing protein
TGME49_227948	-1.07	8.32	4.36E-05	0.000239936	peptidase M16 inactive domain-containing protein
TGME49_230430	-8.37	5.34	4.38E-05	0.000240753	vesicle-associated membrane protein, putative
TGME49_238110	-2.31	6.29	4.42E-05	0.000242712	replication factor a protein 3 protein
TGME49_201870	-2.12	6.58	4.42E-05	0.00024272	tetratricopeptide repeat-containing protein
TGME49_319320	-1.77	6.45	4.45E-05	0.000244524	hypothetical protein
TGME49_239710	-8.40	5.20	4.47E-05	0.000244981	phosphomannomutase
TGME49_285810	-8.37	4.79	4.48E-05	0.000245354	MYND finger domain-containing protein
TGME49_257755	2.33	5.37	4.48E-05	0.000245515	hypothetical protein
TGME49_221710	-2.51	5.92	4.52E-05	0.000247134	TBC domain-containing protein
TGME49_212960	-8.41	5.30	4.52E-05	0.000247134	hypothetical protein
TGME49_275980	-3.59	5.59	4.53E-05	0.000247734	coenzyme q (ubiquinone) biosynthesis protein coq4 p
TGME49_311150	-3.61	5.60	4.55E-05	0.000248243	hypothetical protein
TGME49_252870	1.89	4.88	4.69E-05	0.000255365	hypothetical protein
TGME49_312860	-2.92	5.92	4.74E-05	0.000257627	hypothetical protein
TGME49_233860	-8.46	4.93	4.74E-05	0.000257627	DALR anticodon binding domain-containing protein
TGME49_286800	-8.40	5.19	4.75E-05	0.00025781	hypothetical protein
TGME49_300285	-8.51	5.23	4.78E-05	0.000259691	hypothetical protein
TGME49_269150	-8.38	5.14	4.85E-05	0.000262797	DHHC zinc finger domain-containing protein
TGME49_243490	-8.38	5.07	4.87E-05	0.00026392	BCS1 family isoform 9, putative
TGME49_310380	-8.38	5.10	4.88E-05	0.000264349	brix domain containing protein
TGME49_260650	-3.62	5.40	5.04E-05	0.000272486	glycosyltransferase, group 2 family protein
TGME49_215660	-8.35	5.03	5.05E-05	0.000272902	hypothetical protein
TGME49_261510	3.26	5.09	5.09E-05	0.000275083	hypothetical protein
TGME49_275780	-8.35	4.85	5.20E-05	0.000280618	hypothetical protein
TGME49_221200	1.24	7.36	5.23E-05	0.000281877	CW-type Zinc Finger protein
TGME49_308580	-2.50	5.95	5.27E-05	0.000283992	Lon protease family protein
TGME49_209960	-1.69	6.95	5.28E-05	0.000284174	glycosyltransferase
TGME49_306560	-2.99	5.99	5.28E-05	0.000284288	hypothetical protein
TGME49_216930	-1.62	6.83	5.32E-05	0.000286318	cholinephosphate cytidylyltransferase
TGME49_301300	-8.35	4.71	5.36E-05	0.000288024	hypothetical protein

TGME49_210682	2.68	5.90	5.36E-05	0.000288032	hypothetical protein
TGME49_225290	2.09	6.40	5.38E-05	0.000288897	GDA1/CD39 (nucleoside phosphatase) family protein
TGME49_238940	2.31	4.73	5.40E-05	0.000289356	GDP mannose 4,6-dehydratase, putative
TGME49_284050	-1.75	6.47	5.40E-05	0.000289356	DEAD/DEAH box helicase domain-containing protein
TGME49_291590	-8.34	4.86	5.48E-05	0.000293612	hypothetical protein
TGME49_217880	-8.37	4.80	5.59E-05	0.000298702	RNA-binding protein Nova-1, putative
TGME49_245670	-1.06	7.70	5.60E-05	0.00029932	pyruvate dehydrogenase complex subunit PDH-E1Alp
TGME49_315470	-1.22	7.68	5.61E-05	0.000299668	hypothetical protein
TGME49_249380	1.58	7.53	5.64E-05	0.000300711	DHHC zinc finger domain-containing protein
TGME49_215920	1.80	5.98	5.64E-05	0.000300725	hypothetical protein
TGME49_289700	-2.49	6.24	5.68E-05	0.000302477	hypothetical protein
TGME49_286090	1.21	9.78	5.72E-05	0.000303647	translation initiation factor SUI1, putative
TGME49_239752	-1.15	8.30	5.72E-05	0.000303647	hypothetical protein
TGME49_293330	-8.34	5.03	5.71E-05	0.000303647	hypothetical protein
TGME49_234190	-1.10	8.32	5.74E-05	0.000304459	serine hydroxymethyltransferase 2, putative
TGME49_284780	-1.22	7.47	5.76E-05	0.000305516	hypothetical protein
TGME49_261940	-8.35	4.88	5.78E-05	0.000306191	hydrolase, alpha/beta fold family protein
TGME49_246030	-8.35	5.02	5.80E-05	0.00030704	mediator complex subunit MED17
TGME49_314280	2.21	5.65	5.81E-05	0.000307384	AAR2 protein
TGME49_239610	-3.57	5.48	5.82E-05	0.000307456	hypothetical protein
TGME49_251500	-1.11	8.09	5.82E-05	0.000307629	eukaryotic initiation factor-3, subunit 3, putative
TGME49_276900	-3.60	5.41	5.91E-05	0.000311672	hypothetical protein
TGME49_289190	-8.32	4.82	5.93E-05	0.000312565	tetratricopeptide repeat-containing protein
TGME49_308810	-1.22	8.08	5.94E-05	0.000312657	rhoptry neck protein RON9
TGME49_268170	-8.41	5.25	5.93E-05	0.000312657	hypothetical protein
TGME49_259950	1.96	7.95	5.96E-05	0.000313905	carbonate dehydratase, eukaryotic-type domain-cont
TGME49_306620	2.00	6.62	5.98E-05	0.00031422	AP2 domain transcription factor AP2IX-9
TGME49_235402	-8.39	4.70	5.98E-05	0.00031422	CorA family Mg2+ transporter protein
TGME49_230050	1.74	4.96	6.08E-05	0.000319503	50S ribosomal protein L3, putative
TGME49_227115	-8.32	5.08	6.12E-05	0.000321395	hypothetical protein
TGME49_272670	-8.33	4.99	6.15E-05	0.000322525	peptidase family M3 protein
TGME49_248850	-8.35	5.24	6.17E-05	0.000323067	methionine aminopeptidase
TGME49_227140	2.29	4.81	6.21E-05	0.000325185	hypothetical protein
TGME49_266100	1.05	6.94	6.29E-05	0.000328846	rhoptry kinase family protein ROP41
TGME49_264190	-2.85	5.48	6.28E-05	0.000328846	hypothetical protein
TGME49_277540	-8.33	4.89	6.31E-05	0.000329725	hypothetical protein
TGME49_225300	-1.82	6.43	6.37E-05	0.000332843	hypothetical protein
TGME49_208050	-2.54	5.98	6.41E-05	0.000334354	ABC transporter, putative
TGME49_202800	-8.37	4.80	6.41E-05	0.000334354	cytochrome c oxidase assembly protein COX11, putat
TGME49_219260	-1.30	6.99	6.49E-05	0.000337969	cation-transporting ATPase, putative
TGME49_293780	1.19	7.14	6.50E-05	0.000338302	hypothetical protein
TGME49_280570	2.08	6.81	6.59E-05	0.000342679	SAG-related sequence SRS35A
TGME49_225680	-2.84	5.66	6.60E-05	0.000342834	hypothetical protein
TGME49_251850	-1.52	7.02	6.60E-05	0.000342922	serine/threonine protein phosphatase
TGME49_252310	-1.80	6.41	6.65E-05	0.000345113	hypothetical protein
TGME49_284660	-8.32	5.27	6.66E-05	0.00034551	mitochondrial ribosomal protein s6-2, putative
TGME49_261570	-1.05	10.08	6.67E-05	0.000345631	ribosomal protein RPL7A
TGME49_257710	-3.55	5.56	6.68E-05	0.000345935	actin-like protein ALP 5
TGME49_219800	-1.05	8.18	6.71E-05	0.000347039	vacuolar ATP synthase subunit b, putative
TGME49_200430	-8.32	4.89	6.82E-05	0.000352499	cytidine and deoxycytidylate deaminase zinc-binding
TGME49_206320	-1.23	6.96	6.88E-05	0.000355408	hypothetical protein
TGME49_207770	-1.29	7.77	6.88E-05	0.000355408	PCI domain-containing protein
TGME49_236550	-1.20	7.92	6.92E-05	0.000356461	hypothetical protein
TGME49_314840	-1.71	6.47	6.92E-05	0.000356461	ubiquitin carboxyl-terminal hydrolase
TGME49_267330	-2.03	6.42	6.92E-05	0.000356461	fumarate hydratase

TGME49_313240	-2.92	5.84	6.95E-05	0.000357614	ethylene-responsive RNA helicase, putative
TGME49_235750	-8.31	5.02	7.00E-05	0.000359762	ULK kinase
TGME49_254660	1.21	6.94	7.02E-05	0.000360594	ankyrin repeat-containing protein
TGME49_202750	-8.29	5.03	7.08E-05	0.000363064	3' exoribonuclease family, domain 1 domain-containi
TGME49_318575	-8.32	5.22	7.12E-05	0.000364863	hypothetical protein
TGME49_226270	-2.89	5.82	7.13E-05	0.000365325	hypothetical protein
TGME49_255400	1.34	6.07	7.14E-05	0.000365517	hypothetical protein
TGME49_226330	-8.33	4.87	7.28E-05	0.00037228	hypothetical protein
TGME49_228230	1.22	9.12	7.36E-05	0.000375886	hypothetical protein
TGME49_278960	-1.71	6.55	7.38E-05	0.000376986	hypothetical protein
TGME49_271930	-1.34	8.59	7.42E-05	0.000378509	hypothetical protein
TGME49_308940	-8.29	4.89	7.46E-05	0.000380578	hypothetical protein
TGME49_238410	-2.83	5.77	7.49E-05	0.000381523	hypothetical protein
TGME49_254200	-8.32	4.78	7.50E-05	0.000381523	anticodon binding domain-containing protein
TGME49_271860	-8.37	4.88	7.55E-05	0.000383689	tRNA (Uracil-5-)-methyltransferase
TGME49_259850	-8.28	5.08	7.58E-05	0.000384961	hypothetical protein
TGME49_252640	3.47	4.69	7.62E-05	0.000386462	P-type ATPase PMA1
TGME49_268910	1.34	5.89	7.69E-05	0.000389803	signal peptidase I protein
TGME49_271810	-1.16	7.84	7.70E-05	0.000389803	lanp, putative
TGME49_213600	-8.29	5.17	7.71E-05	0.000390266	hypothetical protein
TGME49_315720	-2.06	5.51	7.74E-05	0.000391307	Smg-4/UPF3 family protein
TGME49_244570	-8.31	4.81	7.76E-05	0.000392025	hypothetical protein
TGME49_217870	1.03	6.94	7.78E-05	0.000392699	DHHC zinc finger domain-containing protein
TGME49_278080	2.04	5.22	7.80E-05	0.000393159	Toxoplasma gondii family A protein
TGME49_230860	-2.24	6.09	7.82E-05	0.000394065	hypothetical protein
TGME49_312380	-8.28	4.96	7.91E-05	0.000398104	tetratricopeptide repeat-containing protein
TGME49_223690	-8.28	5.06	7.93E-05	0.000398664	hypothetical protein
TGME49_220208	-8.29	4.78	8.02E-05	0.000403076	hypothetical protein
TGME49_242435	-3.54	5.43	8.07E-05	0.000404514	hypothetical protein
TGME49_223125	-2.03	6.12	8.36E-05	0.000418611	ubiquitin family protein
TGME49_271170	-3.53	5.26	8.38E-05	0.000419155	dolichol kinase
TGME49_257990	-1.50	6.77	8.39E-05	0.000419675	heat shock protein 101, putative
TGME49_290180	2.52	5.13	8.40E-05	0.00041983	AP2 domain transcription factor AP2IX-6
TGME49_204080	-1.55	6.87	8.41E-05	0.000419845	histidine acid phosphatase superfamily protein
TGME49_236800	-8.28	5.06	8.42E-05	0.000419845	hypothetical protein
TGME49_311380	-3.97	5.26	8.43E-05	0.000420191	hypothetical protein
TGME49_291030	-3.57	5.60	8.46E-05	0.000421618	zinc finger, C3HC4 type (RING finger) domain-contain
TGME49_320460	-3.51	5.29	8.58E-05	0.000427364	ABC transporter transmembrane region domain-cont
TGME49_249020	-2.21	6.04	8.60E-05	0.000427694	kinesin motor domain-containing protein
TGME49_290640	-1.52	7.98	8.62E-05	0.000428872	DNA mismatch repair protein MSH6-1, putative
TGME49_289140	1.35	6.44	8.67E-05	0.000430703	ribosomal protein l22/l43, putative
TGME49_285990	-3.59	5.64	8.69E-05	0.000431633	Filamin/ABP280 repeat-containing protein
TGME49_305920	-3.50	5.44	8.76E-05	0.000434045	endonuclease III family 1 protein
TGME49_263150	-8.28	4.96	8.77E-05	0.00043454	tetratricopeptide repeat-containing protein
TGME49_309400	-2.52	5.88	8.80E-05	0.000435286	RecF/RecN/SMC N terminal domain-containing prote
TGME49_220175	2.52	4.77	8.83E-05	0.0004366	hypothetical protein
TGME49_291140	-1.02	8.46	9.09E-05	0.000448868	CCR4-Not complex component, Not1 protein
TGME49_315640	1.67	5.20	9.26E-05	0.000456237	lipoyl(octanoyl) transferase
TGME49_278240	-8.54	4.82	9.30E-05	0.000458329	Zn-finger in Ran binding protein and others domain-c
TGME49_299000	1.03	6.82	9.35E-05	0.000459924	hypothetical protein
TGME49_320750	1.34	5.85	9.36E-05	0.000460024	hypothetical protein
TGME49_311090	-1.25	7.32	9.46E-05	0.000464603	ubiquitin carboxyl-terminal hydrolase
TGME49_203740	-1.93	6.73	9.46E-05	0.000464603	hypothetical protein
TGME49_227860	-8.26	4.75	9.53E-05	0.00046706	hypothetical protein
TGME49_209270	-8.33	5.06	9.63E-05	0.000472074	hypothetical protein

TGME49_270760	-8.29	4.79	9.70E-05	0.000474886	asparagine synthase
TGME49_264880	-2.19	6.07	9.83E-05	0.000479671	NEDD8-activating enzyme E1 catalytic subunit
TGME49_250500	-3.58	5.36	9.81E-05	0.000479671	hypothetical protein
TGME49_277550	-8.26	4.87	9.83E-05	0.000479671	UvrD/REP helicase domain-containing protein
TGME49_218358	-8.28	4.99	9.82E-05	0.000479671	zinc knuckle domain-containing protein
TGME49_263410	5.23	5.00	9.85E-05	0.000479905	scavenger receptor cysteine-rich domain-containing p
TGME49_315690	-3.51	5.51	9.85E-05	0.000479905	DnaJ domain-containing protein
TGME49_217050	1.06	7.26	9.89E-05	0.000481948	ADA2-A transcriptional co-activator SAGA component
TGME49_252190	1.94	6.80	9.95E-05	0.000484259	KRUF family protein
TGME49_257960	-2.82	5.69	0.000101939	0.000494901	GDP-D-mannose pyrophosphorylase
TGME49_318260	-3.49	5.56	0.000101875	0.000494901	transcription initiation factor TFIID subunit TAF5
TGME49_232640	1.84	5.04	0.000102293	0.000495667	RNA 2'-phosphotransferase, Tpt1/KptA family protein
TGME49_215460	-1.16	9.58	0.00010228	0.000495667	ribosomal protein RPS24
TGME49_243530	-8.23	4.75	0.000102724	0.00049741	pentatricopeptide repeat domain-containing protein
TGME49_309580	-2.79	5.70	0.000103372	0.000500219	transporter, major facilitator family protein
TGME49_216970	-1.17	7.94	0.000104524	0.000505463	coronin, putative
TGME49_285650	-1.41	6.83	0.000104719	0.000505965	hypothetical protein
TGME49_255690	-2.81	5.59	0.000105115	0.000507322	2-C-methyl-D-erythritol 2,4-cyclodiphosphate synthas
TGME49_251920	1.67	5.05	0.000105892	0.00051051	hypothetical protein
TGME49_216630	-8.21	5.35	0.000105913	0.00051051	trigger factor protein, putative
TGME49_267530	1.93	4.71	0.000106363	0.000512344	hypothetical protein
TGME49_243200	-1.43	9.17	0.000106575	0.000513033	hypothetical protein
TGME49_262650	-3.55	5.56	0.000106867	0.000514104	WD domain, G-beta repeat-containing protein
TGME49_222160	-3.52	4.97	0.000107537	0.00051699	aldehyde dehydrogenase
TGME49_273850	-2.77	5.77	0.000107683	0.000517353	hypothetical protein
TGME49_289290	-1.62	7.07	0.000109026	0.000523467	hypothetical protein
TGME49_273920	-2.42	6.00	0.000109239	0.000524149	aldose reductase, putative
TGME49_270160	-8.21	4.78	0.000111063	0.000532209	hypothetical protein
TGME49_310360	-1.33	7.41	0.000111759	0.0005352	hypothetical protein
TGME49_218820	-1.39	8.78	0.000112155	0.000536749	alba 2
TGME49_252065	2.13	5.06	0.00011281	0.000539532	KRUF family protein
TGME49_270150	-2.79	5.78	0.000113211	0.000541098	hypothetical protein
TGME49_222840	1.67	8.70	0.000114672	0.000546466	Ser/Thr phosphatase family protein
TGME49_253700	1.05	8.40	0.000114703	0.000546466	transporter, major facilitator family protein
TGME49_262550	-8.21	4.87	0.000114599	0.000546466	hypothetical protein
TGME49_306290	-1.91	6.40	0.000116284	0.00055364	DNA-directed RNA polymerase III RPC1
TGME49_221370	-3.47	5.30	0.000116882	0.000556129	hypothetical protein
TGME49_224580	-1.73	6.71	0.000117388	0.000558178	RNA recognition motif-containing protein
TGME49_310810	-1.85	6.36	0.000118252	0.000561923	apyrase
TGME49_268600	-2.76	5.84	0.000118448	0.000562493	DNA polymerase epsilon subunit B protein
TGME49_232210	-8.23	5.03	0.00011874	0.00056352	hypothetical protein
TGME49_253600	1.71	6.68	0.000121159	0.000574263	hypothetical protein
TGME49_232000	1.15	8.49	0.000121821	0.000576768	hypothetical protein
TGME49_307650	-1.55	6.35	0.000121844	0.000576768	uracil-DNA glycosylase
TGME49_225910	1.30	5.95	0.000122615	0.000579669	hypothetical protein
TGME49_284170	-8.22	4.98	0.000122611	0.000579669	DHHC zinc finger domain-containing protein
TGME49_226600	-1.86	6.43	0.000123325	0.00058217	syntaxin 5, putative
TGME49_293050	-2.81	5.54	0.000123378	0.00058217	sybindin family protein
TGME49_299010	2.76	6.43	0.00012422	0.000585393	hypothetical protein
TGME49_274150	-3.51	5.45	0.000124212	0.000585393	hypothetical protein
TGME49_266890	-1.47	6.92	0.000124398	0.000585861	hypothetical protein
TGME49_209860	-8.20	5.15	0.000125041	0.000588512	SRP40, C-terminal domain-containing protein
TGME49_260310	1.61	5.29	0.000125894	0.000592152	ATP-binding cassette transporter ABC.B1
TGME49_219300	1.38	5.66	0.000126022	0.000592375	ran binding protein
TGME49_210390	-8.25	5.21	0.000127707	0.000599917	WD domain, G-beta repeat-containing protein

TGME49_288330	-3.47	5.36	0.000128379	0.000602689	histone lysine methyltransferase, SET, putative
TGME49_310520	-3.49	5.14	0.000129323	0.000606736	3'5'-cyclic nucleotide phosphodiesterase domain-con
TGME49_306530	-8.20	5.01	0.00012983	0.000608728	hypothetical protein
TGME49_252280	-1.75	6.04	0.000130094	0.000609578	hypothetical protein
TGME49_204410	-2.01	6.09	0.000131211	0.000614422	endonuclease/exonuclease/phosphatase family prote
TGME49_226560	-3.45	5.49	0.000132301	0.000619056	zinc finger (CCCH type) motif-containing protein
TGME49_240830	-8.19	4.70	0.000132368	0.000619056	hydrolase, alpha/beta fold family protein
TGME49_274000	-2.41	6.01	0.000133318	0.000623107	hypothetical protein
TGME49_228980	-8.20	5.06	0.000133436	0.000623263	hypothetical protein
TGME49_294390	-8.18	5.02	0.000133929	0.00062517	myosin light chain MLC4, putative
TGME49_309990	2.22	6.06	0.000134534	0.000627597	hypothetical protein
TGME49_215030	-2.16	5.85	0.000134706	0.000628007	hypothetical protein
TGME49_288045	-3.53	5.31	0.000134804	0.000628068	hypothetical protein
TGME49_219140	-1.50	6.80	0.000135651	0.000631612	EF-1 guanine nucleotide exchange domain-containing
TGME49_319940	-8.19	4.88	0.000138046	0.000641959	hypothetical protein
TGME49_252510	1.41	5.83	0.00013843	0.000643338	hypothetical protein
TGME49_297690	1.33	6.06	0.000139501	0.000647381	hypothetical protein
TGME49_204060	-2.77	5.60	0.000139945	0.00064875	SNARE domain-containing protein
TGME49_224670	-2.14	5.88	0.000140898	0.000652756	DnaJ domain-containing protein
TGME49_255740	-2.80	5.77	0.00014254	0.000659535	hypothetical protein
TGME49_240220	-1.35	6.59	0.000144499	0.000667765	hypothetical protein
TGME49_295060	-8.17	4.82	0.000147726	0.000681827	exonuclease
TGME49_256030	-1.04	7.94	0.000148348	0.000684272	hypothetical protein
TGME49_205730	-2.76	5.43	0.00014974	0.000690259	hypothetical protein
TGME49_266970	-2.77	5.89	0.000150616	0.000693864	hypothetical protein
TGME49_276910	-1.45	6.62	0.00015166	0.000697806	endoplasmic reticulum lumen protein retaining recep
TGME49_295015	1.43	5.56	0.000153221	0.000704113	patched family protein
TGME49_260640	-2.13	5.79	0.00015354	0.000705139	autophagy protein apg9 protein
TGME49_263290	2.59	6.06	0.000154087	0.000707213	rhomboid protease ROM2
TGME49_209880	-1.19	7.37	0.000154218	0.000707375	glutamic acid-rcih protein, putative
TGME49_300010	-8.20	4.88	0.000154655	0.000708729	hypothetical protein
TGME49_221585	-8.19	5.10	0.000156864	0.000718173	hypothetical protein
TGME49_315700	-2.75	5.81	0.000157396	0.000720166	hypothetical protein
TGME49_205570	-2.75	5.58	0.000157787	0.000721508	hypothetical protein
TGME49_318410	-1.14	7.53	0.000159588	0.000729293	TCP-1 chaperonin, putative
TGME49_312340	-8.15	5.02	0.000161737	0.000738198	hypothetical protein
TGME49_218800	-8.20	4.77	0.000161923	0.000738594	hypothetical protein
TGME49_223070	-1.16	7.72	0.000162405	0.000740332	hypothetical protein
TGME49_211720	-1.07	9.35	0.000162875	0.00074195	AP2 domain transcription factor AP2IV-5
TGME49_308060	-2.21	6.56	0.00016296	0.00074195	hypothetical protein
TGME49_253615	1.10	6.39	0.000163206	0.000742614	hypothetical protein
TGME49_246020	-1.71	6.19	0.000163607	0.000743979	SprT domain-containing protein
TGME49_240660	-8.19	4.90	0.000164021	0.000745402	hypothetical protein
TGME49_307800	1.81	6.04	0.000164356	0.000746008	GDA1/CD39 (nucleoside phosphatase) family protein
TGME49_306210	-8.28	5.06	0.000164311	0.000746008	RNA polymerase II accessory factor CDC73
TGME49_300130	1.92	4.71	0.000164851	0.000747074	apical membrane antigen 1 domain-containing protei
TGME49_297460	-8.18	5.02	0.00016486	0.000747074	hypothetical protein
TGME49_202630	-1.16	7.37	0.000165049	0.000747318	ATP-dependent metallopeptidase HflB subfamily prot
TGME49_271625	-8.19	4.93	0.000165433	0.000748599	serine--tRNA ligase
TGME49_277070	1.30	5.84	0.000166584	0.000753345	SWI2/SNF2-containing protein
TGME49_253330	2.26	8.28	0.000167159	0.000755485	Rhoptry kinase family protein, truncated (incomplete
TGME49_230500	-3.42	5.36	0.000168053	0.000758887	hypothetical protein
TGME49_255195	1.92	5.32	0.000168383	0.000759161	hypothetical protein
TGME49_217560	1.18	6.35	0.000168356	0.000759161	DNA-directed RNA polymerase II RPB10
TGME49_201120	-1.57	6.58	0.000168821	0.000760474	ELMO/CED-12 family protein

TGME49_227310	-3.45	5.53	0.00016888	0.000760474	hypothetical protein
TGME49_264870	1.57	7.51	0.000170107	0.000765532	Sodium:neurotransmitter symporter family protein
TGME49_221922	-3.47	5.27	0.000170294	0.000765909	NifU family domain-containing protein
TGME49_204140	-8.24	4.77	0.000170401	0.000765926	PHD-finger domain-containing protein
TGME49_285950	-8.19	5.02	0.00017134	0.000769208	hypothetical protein
TGME49_305540	-1.62	6.49	0.000172601	0.000774399	hypothetical protein
TGME49_253490	1.80	7.37	0.000172952	0.000775504	hypothetical protein
TGME49_249890	1.64	5.11	0.000173295	0.000776102	hypothetical protein
TGME49_264030	-1.66	6.63	0.00017325	0.000776102	aminotransferase, putative
TGME49_253960	1.92	4.75	0.000174526	0.000781143	oxidoreductase, short chain dehydrogenase/reductas
TGME49_214900	1.01	7.26	0.000175195	0.00078366	hypothetical protein
TGME49_248830	-1.37	6.55	0.000176126	0.000787351	phosphoinositide phospholipase PIPLC
TGME49_291020	-1.63	6.79	0.000177058	0.000791037	myosin head (motor domain) domain-containing prot
TGME49_268430	-3.45	5.49	0.000177838	0.000794041	hypothetical protein
TGME49_313300	1.35	5.75	0.0001784	0.000795592	YL1 nuclear protein C-terminal domain-containing pr
TGME49_249698	-8.18	4.98	0.000178391	0.000795592	hypothetical protein
TGME49_205690	1.01	6.66	0.000178764	0.000796735	hypothetical protein
TGME49_202640	-8.13	4.76	0.000179844	0.000800583	RNA pseudouridine synthase superfamily protein
TGME49_248640	-1.23	7.36	0.000182701	0.00081281	regulator of chromosome condensation (RCC1) repea
TGME49_267400	-1.40	9.30	0.000184596	0.000819762	ribosomal protein RPL32
TGME49_207800	-8.11	5.28	0.000186334	0.000826981	hypothetical protein
TGME49_243390	-2.76	5.53	0.000186642	0.000827851	hypothetical protein
TGME49_223780	-1.60	6.75	0.000186784	0.000827988	hypothetical protein
TGME49_248550	-3.55	5.45	0.000188667	0.000835833	SPX domain-containing protein
TGME49_270330	1.56	6.26	0.000189073	0.000837127	cell-cycle-associated protein kinase, putative
TGME49_290740	-8.14	4.96	0.00018989	0.000840244	hypothetical protein
TGME49_260160	2.71	4.70	0.000190652	0.000843111	hypothetical protein
TGME49_275350	-1.94	6.21	0.000193044	0.000852159	TBC domain-containing protein
TGME49_301130	-8.11	5.01	0.000195477	0.000862386	hypothetical protein
TGME49_260510	-2.70	5.73	0.000196945	0.000867827	ubiquitin thioesterase otubain-like family protein
TGME49_318170	1.25	6.11	0.000198628	0.000874722	hypothetical protein
TGME49_314530	-3.48	5.09	0.000199674	0.000878279	RPAP1 family, C-terminal protein
TGME49_309160	-1.94	6.33	0.000202064	0.000888267	IgA-specific metalloendopeptidase
TGME49_239310	-2.79	5.36	0.00020232	0.000888862	ribulose 5-phosphate isomerase
TGME49_312840	-1.83	6.43	0.000203043	0.000891507	hypothetical protein
TGME49_203290	1.50	8.25	0.000205658	0.000901918	hypothetical protein
TGME49_214800	-8.10	4.74	0.000205613	0.000901918	hypothetical protein
TGME49_320015	-8.10	4.68	0.00020868	0.000913547	hypothetical protein
TGME49_258850	-8.13	4.82	0.000208901	0.000913972	hypothetical protein
TGME49_253410	1.99	6.01	0.000209335	0.00091533	hypothetical protein
TGME49_258180	-8.18	4.82	0.000209515	0.000915574	hypothetical protein
TGME49_223040	1.49	7.86	0.000211333	0.000922975	hypothetical protein
TGME49_204350	-1.93	5.92	0.000212281	0.000926022	hypothetical protein
TGME49_321640	-1.84	6.09	0.000213356	0.000930163	cell division protein CDC48AP
TGME49_265650	-3.43	5.20	0.000214331	0.000933862	protein phosphatase 2C domain-containing protein
TGME49_210360	1.72	6.68	0.000214731	0.000935054	DEAD (Asp-Glu-Ala-Asp) box polypeptide 41 family pr
TGME49_259000	-8.10	4.76	0.000215278	0.000936885	hypothetical protein
TGME49_233090	-8.10	4.99	0.000215767	0.00093846	XPG N-terminal domain-containing protein
TGME49_243410	-3.39	5.61	0.00021662	0.000941615	tetratricopeptide repeat-containing protein
TGME49_290940	-1.28	7.21	0.000220608	0.000958386	EMP/nonaspanin domain family protein
TGME49_248400	-1.92	6.09	0.000221861	0.000963263	glyoxalase I, putative
TGME49_246130	1.26	8.21	0.000222375	0.000964932	serpin (serine proteinase inhibitor) superfamily prote
TGME49_283800	-1.99	6.25	0.000223495	0.000968654	hypothetical protein
TGME49_244470	-1.10	7.80	0.000223748	0.000969181	hypothetical protein
TGME49_269770	-2.73	5.57	0.000225293	0.000974733	WD domain, G-beta repeat-containing protein

TGME49_308990	-3.37	5.30	0.000228323	0.000986687	transporter, solute:sodium symporter (SSS) family pr
TGME49_293310	1.21	6.76	0.000228603	0.00098732	ribosomal protein L20, putative
TGME49_237110	-1.68	6.30	0.000231998	0.001000811	replication factor C subunit 2, putative
TGME49_284645	-2.12	5.66	0.000232785	0.001003037	hypothetical protein
TGME49_218790	-3.40	4.93	0.000232722	0.001003037	elongation factor G C-terminus domain-containing pr
TGME49_293770	1.29	8.02	0.000233202	0.00100425	chitinase-like protein CLP1
TGME49_309920	-3.37	5.24	0.000236152	0.001015177	hypothetical protein
TGME49_202080	-8.07	4.81	0.000237399	0.001019945	hypothetical protein
TGME49_265850	1.76	6.27	0.000238209	0.00102283	hypothetical protein
TGME49_270940	-8.13	4.93	0.000238856	0.00102442	hypothetical protein
TGME49_285272	-8.13	4.73	0.000238781	0.00102442	hypothetical protein
TGME49_307010	1.66	6.89	0.000242059	0.001035755	histone lysine demethylase JMJC1/KDM5D/JARID1D
TGME49_316230	-2.08	6.16	0.000241977	0.001035755	SAC1 phosphoinositide phosphatase, putative
TGME49_306020	-1.68	6.20	0.000242619	0.001037551	hypothetical protein
TGME49_247700	1.14	8.30	0.000244314	0.001044161	AP2 domain transcription factor AP2XII-4
TGME49_278920	-2.72	5.87	0.000244447	0.001044161	hypothetical protein
TGME49_263330	-8.17	4.96	0.000245096	0.001046326	Adaptin ear-binding coat-associated protein 2 (NECAI
TGME49_255890	-2.68	5.76	0.000250528	0.001067669	pyridine nucleotide-disulfide oxidoreductase domain-
TGME49_305600	-8.06	4.82	0.000251371	0.001070644	hypothetical protein
TGME49_255300	-1.61	6.62	0.000254255	0.001082306	hypothetical protein
TGME49_297760	-8.05	5.12	0.000255197	0.001085686	hypothetical protein
TGME49_208440	1.14	7.83	0.00025672	0.001091541	hypothetical protein
TGME49_253320	-8.05	4.96	0.000257058	0.001092348	hypothetical protein
TGME49_239080	-8.07	4.71	0.000257715	0.001094513	carrier superfamily protein
TGME49_248130	-8.09	4.76	0.000263052	0.001115897	hypothetical protein
TGME49_315420	1.37	5.70	0.000263712	0.001118054	hypothetical protein
TGME49_288570	-2.66	5.83	0.000264138	0.001119219	hypothetical protein
TGME49_253540	1.51	5.28	0.000265724	0.001125294	hypothetical protein
TGME49_268750	-3.38	5.37	0.000268453	0.001136199	peptidyl-prolyl cis-trans isomerase E, putative
TGME49_310590	1.36	5.32	0.000269039	0.001138029	hypothetical protein
TGME49_227050	-3.36	5.12	0.000269927	0.001141134	ATPase/histidine kinase/DNA gyrase B/HSP90 domair
TGME49_249410	-2.13	6.04	0.00027129	0.001144928	hypothetical protein
TGME49_311625	-2.07	5.92	0.000272424	0.001148404	WD domain, G-beta repeat-containing protein
TGME49_226900	1.23	6.03	0.000273704	0.001153144	hypothetical protein
TGME49_220920	-8.04	4.78	0.000275661	0.001160728	hypothetical protein
TGME49_309250	-1.54	6.68	0.000277868	0.001168086	hypothetical protein
TGME49_217760	1.31	5.84	0.000279889	0.001175185	GTP-binding protein
TGME49_218250	-2.10	6.10	0.000282413	0.001183764	TAP42 family protein
TGME49_223480	-8.02	5.77	0.000287088	0.001201316	sushi domain (scr repeat) domain-containing protein
TGME49_214520	1.30	5.70	0.000287489	0.001202317	general transcription factor IIH polypeptide 4 GTF2H4
TGME49_215380	-3.38	5.25	0.000287714	0.001202578	hypothetical protein
TGME49_263820	-1.71	6.28	0.00028878	0.001206351	DEAD/DEAH box helicase domain-containing protein
TGME49_251410	-1.71	6.67	0.000288955	0.001206401	tetratricopeptide repeat-containing protein
TGME49_313360	1.10	6.20	0.000291851	0.001216428	hypothetical protein
TGME49_313140	1.34	8.23	0.000292036	0.001216517	isocitrate dehydrogenase
TGME49_204550	-2.70	5.62	0.000292342	0.001217103	hypothetical protein
TGME49_223560	-2.75	5.14	0.000292682	0.001217834	hypothetical protein
TGME49_306390	-1.05	7.63	0.000293013	0.001217841	hypothetical protein
TGME49_319720	-3.43	5.17	0.000293002	0.001217841	hypothetical protein
TGME49_321550	1.04	6.54	0.00029391	0.001220884	hypothetical protein
TGME49_216240	-1.76	6.30	0.000294279	0.001221728	hypothetical protein
TGME49_286010	1.40	5.83	0.000298452	0.001236971	hypothetical protein
TGME49_270450	-8.04	4.74	0.000298361	0.001236971	MCM2/3/5 family protein
TGME49_316150	-1.76	5.95	0.000299076	0.00123886	ULK kinase
TGME49_263510	-1.51	6.36	0.000303754	0.001254726	Spc97 / Spc98 family protein

TGME49_257480	-2.42	6.03	0.000305006	0.001259191	NADP-dependent succinate-semialdehyde dehydroge
TGME49_277220	1.44	5.47	0.000306953	0.001266522	hypothetical protein
TGME49_232960	-1.60	6.61	0.00030841	0.001271115	oxidoreductase, 2OG-Fe(II) oxygenase family protein
TGME49_264752	-3.41	5.23	0.000308766	0.001271873	HEAT repeat-containing protein
TGME49_227910	-1.06	7.80	0.000309643	0.00127407	hypothetical protein
TGME49_309050	-8.05	4.88	0.000311184	0.001279696	hypothetical protein
TGME49_239600	-2.30	5.29	0.000313382	0.001287304	rhoptry kinase family protein ROP23 (incomplete cata
TGME49_311080	-1.92	5.91	0.000315733	0.00129624	transporter, cation channel family protein
TGME49_276990	-3.32	5.48	0.000316891	0.001300272	cytochrome b5 family heme/steroid binding domain-
TGME49_232320	-8.04	4.97	0.000318507	0.001305454	hypothetical protein
TGME49_288620	1.03	6.86	0.00032026	0.001311914	Erv1 / Alr family protein
TGME49_316360	-3.41	5.40	0.000321474	0.001316156	hypothetical protein
TGME49_267700	-3.35	5.07	0.000323394	0.001321558	hypothetical protein
TGME49_240710	-1.70	6.51	0.00032431	0.001323455	RNA recognition motif-containing protein
TGME49_289100	-1.72	6.51	0.000325341	0.001326844	hypothetical protein
TGME49_246060	-8.09	5.11	0.000325846	0.001328173	DNA-dependent RNA polymerase
TGME49_313418	-8.06	5.19	0.000326504	0.001330123	hypothetical protein
TGME49_290310	-2.05	5.87	0.000328579	0.001337251	hypothetical protein
TGME49_277490	1.26	7.63	0.000329892	0.001341708	hypothetical protein
TGME49_226090	-3.35	5.05	0.000330111	0.00134186	DEAD/DEAH box helicase domain-containing protein
TGME49_262500	-1.33	7.01	0.000331723	0.001347672	hypothetical protein
TGME49_271770	-2.65	5.22	0.000334604	0.001357883	hypothetical protein
TGME49_257370	1.96	5.96	0.000335058	0.001358979	hypothetical protein
TGME49_219130	-1.87	5.89	0.000335375	0.001359088	NADPH-glutathione reductase
TGME49_220110	1.56	5.05	0.000339559	0.001373669	hypothetical protein
TGME49_278160	-3.38	5.32	0.000339609	0.001373669	vesicle transport v-snare protein
TGME49_285660	-2.12	5.85	0.000340956	0.001378364	DEAD/DEAH box helicase domain-containing protein
TGME49_214280	-8.13	4.76	0.000341237	0.001378745	phosphoadenosine phosphosulfate reductase family
TGME49_248110	1.41	7.91	0.000341878	0.001380583	hypothetical protein
TGME49_268620	-8.00	4.67	0.000345112	0.001390603	blood stage antigen 41-3 precursor, putative
TGME49_271110	-3.33	5.21	0.000345474	0.001391303	hypothetical protein
TGME49_273550	-3.34	5.26	0.000345853	0.001392074	hypothetical protein
TGME49_226060	1.40	6.99	0.000346473	0.001393809	transmembrane amino acid transporter protein
TGME49_266390	-8.00	4.86	0.000347987	0.001399139	DNA mismatch repair protein, C-terminal domain-cor
TGME49_271000	-2.15	5.98	0.000350905	0.001409336	hypothetical protein
TGME49_208820	-3.36	5.82	0.000353282	0.001418114	1-deoxy-D-xylulose-5-phosphate synthase
TGME49_213635	2.19	5.25	0.000355085	0.001424577	hypothetical protein
TGME49_221260	1.13	6.11	0.000362301	0.001451955	Class-II DAHP synthetase family protein
TGME49_235740	-3.29	5.69	0.000362721	0.001452851	hypothetical protein
TGME49_318460	-1.48	6.53	0.00036748	0.001470317	P-type ATPase of unknown pump specificity (type V)
TGME49_258990	-2.65	5.52	0.000370568	0.001480271	bromodomain-containing protein
TGME49_209610	1.15	6.54	0.000373886	0.001491887	oocyst wall protein OWP2
TGME49_280720	-7.98	5.01	0.000374269	0.001491887	hypothetical protein
TGME49_209460	-8.00	4.82	0.000378222	0.001506776	hypothetical protein
TGME49_288560	-1.61	6.49	0.0003798	0.001512246	hypothetical protein
TGME49_311780	-1.84	5.75	0.000380176	0.00151293	Zn-containing alcohol dehydrogenase
TGME49_234900	-1.30	7.62	0.000382204	0.001520183	PHD-finger domain-containing protein
TGME49_270530	-2.36	5.98	0.000384911	0.001527662	ubiquitin fusion degradation protein UFD1CY
TGME49_236280	1.77	4.85	0.000389479	0.001543305	hypothetical protein
TGME49_226660	-1.22	7.06	0.000396487	0.001568654	hypothetical protein
TGME49_205720	-8.02	4.68	0.000396513	0.001568654	Adenosine/AMP deaminase domain-containing prote
TGME49_211230	-1.40	6.73	0.00039843	0.001575398	eukaryotic initiation factor-2B, alpha subunit, putativ
TGME49_201230	-2.62	5.61	0.000398736	0.001575764	kinesin motor domain-containing protein
TGME49_246178	-2.02	5.82	0.000401739	0.001586782	hypothetical protein
TGME49_285840	1.36	6.34	0.000405937	0.001602508	RAP domain-containing protein

TGME49_248740	1.20	8.77	0.000406937	0.001605598	hypothetical protein
TGME49_269885	-1.06	8.06	0.000411891	0.001623412	rhoptry metalloprotease toxolysin TLN1
TGME49_294720	-2.34	5.83	0.000412977	0.001626825	hypothetical protein
TGME49_277010	-8.00	4.90	0.000416444	0.001639608	Fe-S metabolism associated domain-containing prote
TGME49_207665	-7.99	4.98	0.000419803	0.001651077	kinesin motor domain-containing protein
TGME49_230080	-2.25	5.55	0.000421646	0.001656563	DEAD/DEAH box helicase domain-containing protein
TGME49_320480	-1.14	6.98	0.000422399	0.00165864	Rab11b
TGME49_289670	1.85	4.94	0.000422861	0.00165869	DNA repair metallo-beta-lactamase
TGME49_268730	1.39	5.50	0.000423229	0.001659255	glutaredoxin-related protein
TGME49_315760	-1.17	6.89	0.000434231	0.001699317	AP2 domain transcription factor AP2XI-4
TGME49_319570	-1.66	6.69	0.000434437	0.001699317	WD domain, G-beta repeat-containing protein
TGME49_233310	-3.28	5.31	0.000434597	0.001699317	peptidase D, putative
TGME49_244130	-1.54	6.36	0.00043879	0.0017139	hypothetical protein
TGME49_218370	-2.02	5.98	0.000438722	0.0017139	hypothetical protein
TGME49_240960	2.00	7.06	0.000445619	0.001739654	AIG2 family protein
TGME49_306910	-1.84	6.36	0.000447866	0.001747505	hypothetical protein
TGME49_231380	1.20	6.20	0.000451836	0.001762067	DNA-directed RNA polymerase II RPB4
TGME49_209120	1.35	6.06	0.000453681	0.001766589	hypothetical protein
TGME49_295610	-1.14	7.21	0.000453713	0.001766589	histone lysine methyltransferase, SET, putative
TGME49_256090	-7.95	4.73	0.000454164	0.001767413	glycerophosphodiester phosphodiesterase family pro
TGME49_238230	-2.62	5.73	0.000455303	0.001770917	Ser/Thr phosphatase family protein
TGME49_312905	-1.52	7.21	0.000456525	0.001774734	hypothetical protein
TGME49_222100	-1.23	6.78	0.000457355	0.001775525	hypothetical protein
TGME49_310300	-2.61	5.65	0.000456973	0.001775525	hypothetical protein
TGME49_208722	-3.32	5.29	0.000460704	0.001787221	hypothetical protein
TGME49_228720	-7.93	4.72	0.000462857	0.001793693	hypothetical protein
TGME49_233340	-2.04	6.12	0.000463244	0.001794251	hypothetical protein
TGME49_226970	1.03	10.38	0.000466296	0.001804183	ribosomal protein RPS11
TGME49_270190	-2.74	5.63	0.000466811	0.001805231	protein phosphatase 2C domain-containing protein
TGME49_251885	-7.96	5.07	0.000469397	0.001813334	hypothetical protein
TGME49_291010	1.83	5.14	0.000469794	0.001813335	hypothetical protein
TGME49_278470	-3.38	5.56	0.000469888	0.001813335	hypothetical protein
TGME49_203970	-2.59	5.54	0.000470781	0.001815834	dolichyl-diphosphooligosaccharide--protein glycosyltr
TGME49_229340	-7.92	4.88	0.000473982	0.001826275	hypothetical protein
TGME49_280770	-1.02	7.52	0.000478593	0.001843082	regulator of chromosome condensation (RCC1) repea
TGME49_269130	-7.92	4.83	0.000479109	0.001844107	hypothetical protein
TGME49_261710	-1.46	6.65	0.000482086	0.001854221	ankyrin repeat-containing protein
TGME49_264210	1.37	5.57	0.000484852	0.001859437	hypothetical protein
TGME49_299970	-1.03	8.14	0.000484468	0.001859437	tetratricopeptide repeat-containing protein
TGME49_212735	-2.65	5.29	0.000484711	0.001859437	hypothetical protein
TGME49_318590	-7.96	4.79	0.000489192	0.001875109	MRP family domain-containing protein
TGME49_221280	-1.31	6.77	0.000491565	0.001882254	hypothetical protein
TGME49_246978	-3.26	5.07	0.000493292	0.001887887	hypothetical protein
TGME49_294250	-1.84	6.17	0.000495626	0.001895842	WD domain, G-beta repeat-containing protein
TGME49_294705	-7.95	5.03	0.0004988	0.001906996	hypothetical protein
TGME49_225110	1.07	6.39	0.000502278	0.001917318	AP2 domain transcription factor AP2X-2
TGME49_215260	-1.22	7.22	0.000503956	0.00192273	carbamoylphosphate synthetase
TGME49_293060	-1.75	6.24	0.000504876	0.001924254	SPRY domain-containing protein
TGME49_245475	1.46	5.26	0.000506978	0.001930275	hypothetical protein
TGME49_203358	-3.26	5.46	0.00050674	0.001930275	hypothetical protein
TGME49_313860	1.64	7.16	0.000509615	0.001939316	regulator of chromosome condensation (RCC1) repea
TGME49_281675	1.68	4.86	0.000513199	0.001950169	protein kinase, putative
TGME49_207830	1.11	6.32	0.000513258	0.001950169	MORN repeat-containing protein
TGME49_268870	-1.11	7.46	0.000516042	0.00195836	tetratricopeptide repeat-containing protein
TGME49_294860	-1.71	6.13	0.000516208	0.00195836	hypothetical protein

TGME49_244720	-3.24	5.51	0.000520078	0.001972029	hypothetical protein
TGME49_258680	-1.83	6.21	0.00052291	0.001978708	TATA-box binding protein TBP2
TGME49_272270	-7.90	4.79	0.000522859	0.001978708	radical SAM domain-containing protein
TGME49_255960	-1.45	6.58	0.00052516	0.001986206	hypothetical protein
TGME49_312280	-1.31	6.89	0.000525856	0.001987821	pre-mRNA-splicing factor ATP-dependent RNA helicas
TGME49_263130	1.21	8.10	0.000528821	0.001996986	citrate synthase, putative
TGME49_212725	-2.24	5.73	0.000533916	0.0020152	hypothetical protein
TGME49_267560	1.64	5.89	0.000541805	0.002041847	folate-binding protein YgfZ protein
TGME49_202680	-1.16	8.26	0.000546695	0.002055041	peptidase M16, alpha subunit, putative
TGME49_220440	-2.04	5.93	0.000547511	0.002057061	cyclin-dependent kinase regulatory subunit protein
TGME49_207940	-3.27	5.31	0.000551324	0.002070336	ribosomal protein S9, putative
TGME49_246040	-1.69	6.20	0.000553393	0.002077049	MIF4G domain-containing protein
TGME49_251480	1.95	5.55	0.000554925	0.002081741	DEAD/DEAH box helicase domain-containing protein
TGME49_203620	-3.25	5.21	0.000564518	0.002111425	hypothetical protein
TGME49_222120	-3.23	5.09	0.00056585	0.002115215	hypothetical protein
TGME49_257170	-3.23	5.13	0.00056717	0.002119077	hypothetical protein
TGME49_220160	-3.23	5.04	0.000569553	0.002126908	WD domain-containing protein
TGME49_243790	-1.82	5.96	0.000571675	0.002133752	SAG-related sequence SRS33
TGME49_293840	1.05	6.85	0.00057796	0.002155036	hypothetical protein
TGME49_216760	-7.92	4.80	0.000578718	0.002156774	RNA pseudouridine synthase superfamily protein
TGME49_214940	1.07	10.53	0.000588519	0.00218889	MIC2-associated protein M2AP
TGME49_213420	-7.88	4.75	0.00059018	0.002192861	RAP domain-containing protein
TGME49_266930	1.45	5.42	0.000591526	0.002196757	general transcription factor IIH polypeptide 3 GTF2H3
TGME49_236780	-3.23	5.28	0.00059879	0.002221502	zinc finger, C3HC4 type (RING finger) domain-contain
TGME49_224810	-3.46	5.52	0.000600175	0.002225525	hypothetical protein
TGME49_232980	1.64	5.06	0.000602656	0.002232486	hypothetical protein
TGME49_271200	-1.34	6.92	0.000607768	0.002250295	AP2 domain transcription factor AP2VIII-5
TGME49_317705	-1.73	6.25	0.000613563	0.002270614	enoyl-CoA hydratase/isomerase family protein
TGME49_242810	-3.25	5.22	0.000615878	0.00227804	hypothetical protein
TGME49_262880	-1.50	6.33	0.000619047	0.002288615	hypothetical protein
TGME49_214470	-2.20	5.61	0.000619804	0.002290269	Ulp1 protease family, C-terminal catalytic domain-co
TGME49_240890	-1.06	7.45	0.000622544	0.002299244	6-phosphofructokinase
TGME49_225310	-1.18	6.93	0.00063062	0.00232675	ARF1-directed GTPase-activating protein, putative
TGME49_320100	-1.06	8.20	0.000631208	0.002327758	RNA recognition motif-containing protein
TGME49_272320	1.24	5.67	0.000631908	0.002328014	DHHC zinc finger domain-containing protein
TGME49_262390	-1.58	6.20	0.000633316	0.002332039	TLD protein
TGME49_299050	1.05	10.43	0.000634762	0.002333879	ribosomal protein RPL17
TGME49_311280	-7.86	4.70	0.000640184	0.002352645	hypothetical protein
TGME49_218955	1.39	5.19	0.000646753	0.002375605	hypothetical protein
TGME49_205625	-3.24	5.09	0.000651867	0.00239201	hypothetical protein
TGME49_289510	1.20	5.65	0.000652817	0.002394191	hypothetical protein
TGME49_312220	-3.23	5.27	0.000653108	0.002394191	mitochondrial inner membrane translocase subunit T
TGME49_278130	-1.77	6.84	0.000654797	0.002399193	hypothetical protein
TGME49_291690	-3.21	5.09	0.000657489	0.002407862	hypothetical protein
TGME49_239300	-1.58	6.35	0.000663374	0.002427012	hypothetical protein
TGME49_202410	-1.62	6.11	0.000664821	0.002431102	hypothetical protein
TGME49_307040	-1.31	6.89	0.000665746	0.002432078	shikimate dehydrogenase substrate binding domain-c
TGME49_273970	-3.20	5.02	0.00066834	0.002439202	CorA family Mg2+ transporter protein
TGME49_262120	-1.20	7.00	0.000669564	0.002442405	IQ calmodulin-binding motif domain-containing prote
TGME49_281450	-2.60	5.45	0.000675588	0.00246195	cell-cycle-associated protein kinase, putative
TGME49_227390	-2.55	5.44	0.000682111	0.002480983	hypothetical protein
TGME49_229290	-7.86	4.73	0.000682833	0.002482234	kelch repeat-containing protein
TGME49_249600	-7.86	4.74	0.000683793	0.002484503	hypothetical protein
TGME49_258920	-7.87	4.81	0.000686305	0.002491182	hypothetical protein
TGME49_314970	-1.13	7.24	0.000688199	0.002496832	root hair defective 3 gtp-binding protein (rhd3) prote

TGME49_294980	1.48	6.35	0.000691272	0.002505521	hypothetical protein
TGME49_246730	1.38	6.92	0.000692853	0.002510022	hypothetical protein
TGME49_281650	-1.66	5.97	0.000696638	0.002521264	hypothetical protein
TGME49_224150	-7.83	4.83	0.00070555	0.002548528	hypothetical protein
TGME49_250830	-1.24	7.32	0.000706257	0.002549837	SAC3/GANP family protein
TGME49_203480	-3.25	5.11	0.000707316	0.002552411	hypothetical protein
TGME49_315590	-1.95	5.93	0.000722613	0.002602534	macro domain-containing protein
TGME49_235700	-1.43	6.40	0.000723689	0.00260514	sedoheptulose-1,7-bisphosphatase
TGME49_299110	-1.81	6.49	0.000730789	0.002629418	cleft lip and palate transmembrane protein 1 (clptm1
TGME49_233820	-7.82	4.93	0.000735721	0.002644589	DNA polymerase epsilon subunit B protein
TGME49_258350	1.36	5.57	0.000737184	0.002646856	hypothetical protein
TGME49_250670	1.04	6.89	0.000737109	0.002646856	hypothetical protein
TGME49_224490	-3.25	5.05	0.000739876	0.002654362	polyprenyl synthetase superfamily protein
TGME49_263360	-1.96	5.69	0.000742252	0.002660305	WD domain, G-beta repeat-containing protein
TGME49_311890	-1.06	7.36	0.000760976	0.002723453	hypothetical protein
TGME49_306540	1.22	5.76	0.00076783	0.002745326	phosphotransferase enzyme family protein
TGME49_309190	1.05	6.72	0.00077761	0.002777608	hypothetical protein
TGME49_265380	-1.96	5.88	0.000782457	0.002792223	tetratricopeptide repeat (TPR)-/ U-box domain-conta
TGME49_246800	-1.35	6.81	0.000784268	0.002795987	acylaminoacyl-peptidase, putative
TGME49_320005	-3.18	5.20	0.000785528	0.00279913	hypothetical protein
TGME49_260520	1.24	7.70	0.000790282	0.002813359	hypothetical protein
TGME49_280522	-1.27	6.97	0.000791553	0.002816527	hypothetical protein
TGME49_223668	1.55	5.19	0.00079477	0.002825254	LYAR-type C2HC zinc finger protein
TGME49_221360	-1.98	5.89	0.000795472	0.002826391	hypothetical protein
TGME49_216530	-7.84	4.90	0.000796335	0.002828098	ribosome recycling factor protein
TGME49_255170	-3.19	5.01	0.000798551	0.002834608	hypothetical protein
TGME49_263230	-1.17	6.78	0.000800476	0.002840078	hypothetical protein
TGME49_223060	-1.63	6.32	0.000803502	0.00284906	MORN repeat-containing protein
TGME49_247280	-2.16	5.61	0.000803779	0.00284906	hypothetical protein
TGME49_221390	-1.78	5.94	0.000811317	0.002874402	hypothetical protein
TGME49_258625	-7.88	4.74	0.000828007	0.002930724	peptidyl-prolyl cis-trans isomerase, FKBP-type domai
TGME49_238170	1.05	6.42	0.000832116	0.002940685	hypothetical protein
TGME49_254090	-1.39	6.52	0.000837632	0.002956302	hypothetical protein
TGME49_235880	1.06	7.78	0.000838122	0.002956618	brain protein 44 family protein
TGME49_313385	1.47	5.03	0.00084495	0.002975639	hypothetical protein
TGME49_236510	1.02	6.37	0.000845526	0.002975639	hypothetical protein
TGME49_268280	-1.00	7.38	0.000845403	0.002975639	'chromo' (CHRromatin Organization MOdifier) domai
TGME49_240460	-2.50	5.64	0.000849596	0.00298854	AP2 domain transcription factor AP2VI-1
TGME49_301400	1.14	9.99	0.000853494	0.003000824	hypothetical protein
TGME49_229940	-2.15	5.72	0.000863227	0.003032162	cyclophilin, putative
TGME49_294430	-3.16	5.21	0.000866608	0.003042594	hypothetical protein
TGME49_228460	-1.67	6.12	0.000868545	0.003047945	hypothetical protein
TGME49_228030	1.56	4.77	0.000874647	0.003064994	hypothetical protein
TGME49_243290	-1.78	6.21	0.000876991	0.003071752	hypothetical protein
TGME49_244860	1.46	5.15	0.000878866	0.003076863	hypothetical protein
TGME49_241180	-1.11	7.10	0.000880476	0.00308104	hypothetical protein
TGME49_266810	-1.25	6.87	0.000882692	0.003087333	hypothetical protein
TGME49_278020	-3.17	4.94	0.00088375	0.003089573	hypothetical protein
TGME49_222240	-1.20	6.92	0.000896539	0.003132802	hypothetical protein
TGME49_323100	1.12	6.67	0.000901253	0.003147787	hypothetical protein
TGME49_241170	-1.21	6.88	0.000903766	0.003155072	hypothetical protein
TGME49_228630	-1.14	7.14	0.000909206	0.00317107	hypothetical protein
TGME49_263505	-3.18	5.15	0.000911989	0.003176281	hypothetical protein
TGME49_266800	-1.65	6.24	0.000915992	0.003188723	integral membrane protein, putative
TGME49_318300	-2.49	5.50	0.000919571	0.003198169	hypothetical protein

TGME49_290340	-1.97	6.17	0.000922204	0.003205817	HEAT repeat-containing protein
TGME49_236070	1.16	6.93	0.000923455	0.003208659	pyrroline-5-carboxylate reductase
TGME49_265070	-2.53	5.58	0.00092928	0.003227381	hypothetical protein
TGME49_246050	-7.93	4.73	0.000930272	0.003229309	hypothetical protein
TGME49_264450	-1.93	5.87	0.00093149	0.00323202	DNA topoisomerase III beta-1, putative
TGME49_206415	-1.34	6.70	0.000932235	0.003233088	myosin K
TGME49_213040	-1.55	6.26	0.000945713	0.003272158	hypothetical protein
TGME49_233890	-1.95	6.03	0.0009512	0.003289605	hypothetical protein
TGME49_306250	1.53	5.14	0.000953516	0.003296072	hypothetical protein
TGME49_203420	1.65	5.22	0.000970031	0.003345507	4'-phosphopantetheinyl transferase domain-containi
TGME49_212920	1.27	5.72	0.000970079	0.003345507	hypothetical protein
TGME49_229450	-1.79	5.88	0.000974445	0.003355868	hypothetical protein
TGME49_301410	-1.28	6.31	0.000980016	0.003373481	hypothetical protein
TGME49_224230	1.50	5.03	0.000986009	0.003392531	AP2 domain transcription factor AP2X-3
TGME49_227270	2.27	5.63	0.000990104	0.003403455	hypothetical protein
TGME49_288260	-1.37	6.74	0.000990815	0.003404315	hypothetical protein
TGME49_202430	1.19	6.35	0.000999051	0.003424781	hypothetical protein
TGME49_232970	-3.14	5.07	0.001006415	0.003445106	hypothetical protein
TGME49_222410	-1.35	6.72	0.001009748	0.003451722	hypothetical protein
TGME49_308070	-1.96	5.86	0.001012108	0.003458191	hypothetical protein
TGME49_214380	1.31	5.41	0.001013236	0.003460447	hypothetical protein
TGME49_242830	-1.55	6.33	0.001023856	0.003493491	XRN 5'-3' exonuclease N-terminus protein
TGME49_290225	-3.16	5.04	0.001036149	0.003530548	hypothetical protein
TGME49_233220	1.04	7.46	0.001041585	0.003547434	hypothetical protein
TGME49_209070	1.13	7.33	0.001059074	0.003595406	hypothetical protein
TGME49_208390	1.44	6.10	0.001060084	0.003597183	hypothetical protein
TGME49_316900	-1.47	6.55	0.00106591	0.003613635	Sas10 C-terminal domain-containing protein
TGME49_255290	-2.21	5.03	0.001086532	0.003678488	hypothetical protein
TGME49_262170	-2.13	5.41	0.001090326	0.003687955	hypothetical protein
TGME49_315190	-1.13	6.83	0.001091307	0.003689585	CAM kinase, SNF1 family
TGME49_290840	-1.37	6.35	0.00109663	0.003704195	serine protease
TGME49_240580	-1.63	5.66	0.00110104	0.003715697	hypothetical protein
TGME49_215060	-1.16	7.25	0.001105346	0.003728083	small GTP-binding protein sar1, putative
TGME49_260420	-1.62	6.17	0.001105719	0.003728083	HEC/Ndc80p family protein
TGME49_235610	-3.11	5.08	0.001110449	0.003742324	ATPase, AAA family protein
TGME49_251790	1.04	6.44	0.001111705	0.003744849	hypothetical protein
TGME49_230710	-3.17	5.18	0.001141148	0.003835293	cell division protein CDC48
TGME49_273595	-1.64	6.00	0.001147888	0.003853207	hypothetical protein
TGME49_293460	-3.13	5.22	0.001158216	0.00388207	ATP-dependent DNA ligase domain-containing protei
TGME49_282130	-2.12	5.72	0.001160632	0.003888407	hypothetical protein
TGME49_242790	-1.35	6.69	0.001172291	0.003922137	trichohyalin, putative
TGME49_321170	2.26	5.28	0.001173545	0.00392278	Toxoplasma gondii family C protein
TGME49_250680	-2.11	5.77	0.001173205	0.00392278	TBC domain-containing kinase (incomplete catalytic t
TGME49_312480	1.13	6.37	0.001174695	0.003924851	uracil phosphoribosyltransferase FUR1, putative
TGME49_288840	1.59	7.58	0.001180129	0.003941227	hypothetical protein
TGME49_248890	-2.11	6.03	0.001186319	0.003960109	actin-like protein ALP3b
TGME49_284540	-1.03	7.47	0.001197828	0.003989521	ATP synthase F1, delta subunit protein
TGME49_289790	-3.16	4.93	0.001201115	0.00399867	hypothetical protein
TGME49_243600	-1.26	6.49	0.001209361	0.0040225	acetyltransferase, GNAT family protein
TGME49_226320	1.14	6.83	0.001209928	0.004022577	hypothetical protein
TGME49_253800	-3.15	5.03	0.001211862	0.004026502	ribosomal protein L15, putative
TGME49_306950	-7.84	4.67	0.001212743	0.004026502	RAP domain-containing protein
TGME49_205240	-1.20	6.70	0.001216801	0.004036352	cleft lip and palate transmembrane protein 1 (clptm1
TGME49_252380	1.24	6.44	0.001228891	0.004072801	hypothetical protein
TGME49_257595	-2.54	5.61	0.001237609	0.004099855	hypothetical protein

TGME49_305930	1.51	4.79	0.001242253	0.0041134	hypothetical protein
TGME49_233540	-3.25	5.15	0.001256715	0.004159424	transporter, major facilitator family protein
TGME49_231600	-1.91	6.14	0.001259945	0.004168247	HEAT repeat-containing protein
TGME49_223130	1.41	5.12	0.00127197	0.004200513	hypothetical protein
TGME49_272550	-1.18	6.75	0.00127945	0.004223328	hypothetical protein
TGME49_228320	-7.69	4.69	0.001306667	0.004301645	hypothetical protein
TGME49_272520	-1.18	6.87	0.001308754	0.004306515	hypothetical protein
TGME49_241840	1.36	6.62	0.00131873	0.004335566	hypothetical protein
TGME49_228060	-3.21	5.12	0.001328345	0.004365237	hypothetical protein
TGME49_290000	-7.76	4.83	0.001339387	0.004397615	hypothetical protein
TGME49_321660	-3.15	4.88	0.001342488	0.004403886	mannosyltransferase, putative
TGME49_297150	-1.07	7.18	0.001347251	0.004415591	MORN repeat-containing protein
TGME49_298020	-1.19	6.88	0.00134853	0.004417825	DEAD-family helicase
TGME49_310660	-3.08	5.16	0.001349436	0.004418836	Dullard family phosphatase domain-containing protei
TGME49_269140	-1.56	6.33	0.001354822	0.00443451	transport protein particle component, Bet3 domain-c
TGME49_244660	-3.10	5.30	0.001366157	0.004467655	hypothetical protein
TGME49_213370	1.00	6.46	0.001392928	0.004539139	formin FRM3
TGME49_202480	-3.07	5.11	0.001392845	0.004539139	hypothetical protein
TGME49_203160	1.09	6.00	0.001394462	0.004542138	hypothetical protein
TGME49_239630	-3.10	5.29	0.001395176	0.004542463	cytidine and deoxycytidylate deaminase zinc-binding
TGME49_221640	1.86	6.98	0.001404418	0.004568529	hypothetical protein
TGME49_264970	-1.26	6.43	0.001421485	0.004619984	hypothetical protein
TGME49_287980	-1.59	6.10	0.001422644	0.004621719	FHA domain-containing protein
TGME49_266110	1.30	5.29	0.001432093	0.004646291	DEAD/DEAH box helicase domain-containing protein
TGME49_242260	1.15	7.17	0.001438798	0.004665996	hypothetical protein
TGME49_226940	-3.11	5.08	0.001441592	0.004673008	ubiquitin carboxyl-terminal hydrolase
TGME49_244290	-1.20	6.46	0.001443459	0.004674962	adapter-related protein
TGME49_247930	-7.69	4.68	0.001457825	0.004717355	SNARE domain-containing protein
TGME49_242845	-3.08	5.19	0.00146826	0.004749042	hypothetical protein
TGME49_278550	-1.78	6.00	0.001473871	0.004763025	elongation factor Tu GTP binding domain-containing
TGME49_240910	-1.50	6.32	0.001482301	0.004788174	hypothetical protein
TGME49_304720	-1.79	5.94	0.001485336	0.004795883	hypothetical protein
TGME49_273580	-2.08	5.41	0.001494164	0.004818075	hypothetical protein
TGME49_244250	-1.02	8.20	0.001498192	0.004824753	hypothetical protein
TGME49_265410	1.14	5.83	0.001499693	0.004827482	G-protein beta WD-40 repeat containing protein
TGME49_242850	-2.07	5.93	0.001528254	0.004913005	hypothetical protein
TGME49_203390	-1.15	7.14	0.001532491	0.004924483	CRAL/TRIO domain-containing protein
TGME49_222910	-1.34	6.45	0.001537448	0.004936336	phosphoglycerate mutase
TGME49_311160	-1.32	6.69	0.001553567	0.004983546	PWI domain-containing protein
TGME49_304750	-2.09	5.37	0.001555668	0.004988122	zinc finger, C3HC4 type (RING finger) domain-contain
TGME49_261960	1.52	5.17	0.001558889	0.00499352	hypothetical protein
TGME49_272000	-2.08	5.52	0.001559378	0.00499352	hypothetical protein
TGME49_304490	1.05	6.16	0.00156322	0.005003657	hypothetical protein
TGME49_205160	1.40	5.28	0.00156623	0.005011122	hypothetical protein
TGME49_271440	-2.42	5.43	0.001574823	0.005036436	NPL4 family protein
TGME49_216850	-7.64	4.72	0.00158408	0.005061661	hypothetical protein
TGME49_309390	-7.64	4.72	0.001591623	0.005083568	hypothetical protein
TGME49_294730	-1.46	6.29	0.001595766	0.0050924	hypothetical protein
TGME49_260840	-3.08	4.88	0.001598871	0.005100108	hypothetical protein
TGME49_209755	2.88	6.70	0.001612446	0.005136221	hypothetical protein
TGME49_208040	-1.85	5.83	0.001612971	0.005136221	aldo-keto reductase
TGME49_203010	-1.01	7.22	0.001622828	0.005165385	aurora kinase
TGME49_267120	-2.41	5.47	0.001645615	0.005226667	hypothetical protein
TGME49_226400	-1.48	6.16	0.001648766	0.005234425	lipoic acid synthase LIPA
TGME49_316760	1.37	5.65	0.001650794	0.005238614	hypothetical protein

TGME49_202180	-1.32	6.37	0.00166002	0.005265633	hypothetical protein
TGME49_318730	-1.21	6.68	0.001668419	0.005287737	glycosyl transferase
TGME49_277090	2.17	7.62	0.001673708	0.005299957	carrier superfamily protein
TGME49_261780	1.08	8.77	0.001674539	0.005300319	microneme protein MIC7
TGME49_259230	-2.42	5.32	0.001682337	0.005322722	site-specific recombinase, phage integrase family pro
TGME49_278230	-3.04	5.10	0.001704914	0.005384936	prenyltransferase and squalene oxidase repeat-conta
TGME49_258700	-1.09	6.90	0.001713632	0.005407851	transporter, major facilitator family protein
TGME49_253300	1.79	4.94	0.001715594	0.005411734	hypothetical protein
TGME49_294060	-1.03	7.47	0.00173956	0.005473321	hypothetical protein
TGME49_309070	1.63	5.61	0.001748473	0.005499025	hypothetical protein
TGME49_277820	-2.43	5.23	0.00175831	0.005525263	hypothetical protein
TGME49_200330	-1.47	6.65	0.001761611	0.005533284	hypothetical protein
TGME49_280580	1.19	5.84	0.00177146	0.005557807	SAG-related sequence SRS35B
TGME49_293680	-1.52	6.24	0.001771673	0.005557807	hypothetical protein
TGME49_240090	1.67	8.58	0.001775567	0.00556766	rhoptry kinase family protein ROP34, putative
TGME49_319930	-1.28	6.40	0.001796637	0.005630258	hypothetical protein
TGME49_254610	1.73	5.99	0.001807063	0.005656827	Tim10/DDP family zinc finger superfamily protein
TGME49_264440	-1.58	6.16	0.001811324	0.005667765	signal recognition particle receptor beta subunit prot
TGME49_290960	-1.20	6.72	0.001812469	0.005668949	pyruvate phosphate dikinase, pep/pyruvate binding d
TGME49_313570	-1.31	6.48	0.001820391	0.005691321	regulator of chromosome condensation (RCC1) repea
TGME49_285520	-3.05	5.20	0.001822395	0.005695177	RNA cap guanine-N2 methyltransferase
TGME49_218740	1.73	5.57	0.0018238	0.005697158	membrane protein, putative
TGME49_212800	-1.35	6.53	0.0018246	0.005697251	hypothetical protein
TGME49_267590	-1.24	6.55	0.001827119	0.005702709	hypothetical protein
TGME49_286740	2.15	5.13	0.001830383	0.005708078	microneme-like protein
TGME49_266690	-1.12	6.96	0.001831337	0.005708646	hypothetical protein
TGME49_233520	-1.13	6.77	0.001835481	0.005719151	ATP-dependent RNA helicase
TGME49_239130	1.07	5.73	0.001838744	0.005726906	Tyrosine kinase-like (TKL) protein
TGME49_306195	2.36	5.14	0.001851645	0.005762232	hypothetical protein
TGME49_221490	-2.37	5.40	0.001857848	0.005774245	cell cycle regulator protein
TGME49_309610	-3.07	4.89	0.001869204	0.005802224	hypothetical protein
TGME49_237870	-3.04	4.91	0.001870443	0.005803631	FYVE zinc finger domain-containing protein
TGME49_209740	1.31	5.20	0.001890301	0.00585555	hypothetical protein
TGME49_239320	-7.58	4.74	0.00189447	0.005865876	BolA family protein
TGME49_246530	-3.12	5.27	0.001899478	0.005878921	phospholipase D active site domain-containing protei
TGME49_283810	-3.03	5.42	0.00192236	0.005944763	hypothetical protein
TGME49_244412	-2.38	5.00	0.001933536	0.005976689	hypothetical protein
TGME49_244010	-2.04	5.45	0.001941224	0.005995575	hypothetical protein
TGME49_310350	-1.49	6.39	0.001967086	0.006067849	PGAP1 family protein
TGME49_314460	-7.58	4.78	0.001969983	0.006074252	hypothetical protein
TGME49_307860	1.18	8.14	0.001971506	0.006076223	hypothetical protein
TGME49_277870	-1.69	6.10	0.00197256	0.006076223	hypothetical protein
TGME49_209140	-1.15	6.82	0.001983154	0.0060996	anti-silencing protein, ASF1 family protein
TGME49_254915	-2.51	5.07	0.001999464	0.006142101	hypothetical protein
TGME49_314030	1.86	5.75	0.002008902	0.006163413	hypothetical protein
TGME49_230520	-1.04	6.96	0.002011672	0.006164236	cyclophilin 1, putative
TGME49_272720	-1.84	5.62	0.002011581	0.006164236	methyltransferase domain-containing protein
TGME49_257150	-2.37	5.49	0.002016941	0.006177821	NOT2 / NOT3 / NOT5 family protein
TGME49_289890	-2.45	5.61	0.002022454	0.006192143	hypothetical protein
TGME49_283830	1.73	5.33	0.002047368	0.006260643	type I inorganic pyrophosphatase PPase
TGME49_270060	-1.85	5.79	0.002048995	0.006263028	hypothetical protein
TGME49_278930	-1.48	6.40	0.002050701	0.006265654	Tubulin-tyrosine ligase family protein
TGME49_245580	1.12	6.12	0.002061239	0.006292651	hypothetical protein
TGME49_255310	-1.51	5.94	0.002068868	0.006313336	zinc finger (CCCH type) motif-containing protein
TGME49_300180	-3.00	4.77	0.002073791	0.006325747	hypothetical protein

TGME49_267140	-7.60	4.84	0.00208234	0.006343974	SAG-related sequence SRS38B
TGME49_280750	1.06	6.37	0.002087367	0.00635667	rudimentary enhancer, putative
TGME49_202190	1.29	6.78	0.002109783	0.006414366	hypothetical protein
TGME49_211680	-1.12	11.04	0.002114952	0.006422159	protein disulfide isomerase
TGME49_224190	-1.33	6.40	0.002121425	0.006436526	cation-transporting atpase family protein
TGME49_222920	-1.66	6.07	0.002127063	0.006450986	mbp-1 interacting protein-2a family protein
TGME49_202520	1.23	6.53	0.002144585	0.006499309	hypothetical protein
TGME49_240730	-2.99	5.10	0.002157251	0.0065345	hypothetical protein
TGME49_262990	-3.05	4.94	0.002160856	0.006542738	hypothetical protein
TGME49_222245	1.08	6.07	0.002170221	0.006565717	hypothetical protein
TGME49_267040	-2.37	5.35	0.002182672	0.006597988	hypothetical protein
TGME49_293350	-1.37	6.08	0.002230107	0.006727625	mitochondrial carrier superfamily protein
TGME49_232530	-2.03	5.46	0.002248241	0.006779566	hypothetical protein
TGME49_248630	-1.39	6.51	0.002265214	0.00682518	actin-related protein ARP1
TGME49_216335	1.13	5.08	0.002271722	0.006842	hypothetical protein
TGME49_297170	1.43	5.17	0.002276924	0.006854876	50S ribosomal protein L17, putative
TGME49_203362	-3.02	4.97	0.00229675	0.006900517	hypothetical protein
TGME49_261650	-1.11	7.04	0.002304217	0.00691452	hypothetical protein
TGME49_310330	-2.05	5.47	0.002308983	0.006923205	hypothetical protein
TGME49_275568	-1.39	6.27	0.002317697	0.006946516	GPI transamidase subunit PIG-U protein
TGME49_213870	-1.13	6.92	0.002352435	0.007036369	UBA/TS-N domain-containing protein
TGME49_291350	-3.01	5.02	0.002369053	0.007075324	hypothetical protein
TGME49_213115	-7.52	4.95	0.002369286	0.007075324	hypothetical protein
TGME49_261990	-1.08	6.45	0.002388426	0.007129601	zinc finger, C3HC4 type (RING finger) domain-contain
TGME49_297270	-2.35	5.28	0.002393735	0.007142564	hypothetical protein
TGME49_309800	-1.21	6.85	0.002407222	0.00717991	RNA recognition motif-containing protein
TGME49_312650	1.35	6.55	0.002433021	0.007248089	hypothetical protein
TGME49_278260	-2.99	4.79	0.00244108	0.007266243	polynucleotide adenylyltransferase
TGME49_231100	-1.07	6.82	0.002446415	0.00727635	hypothetical protein
TGME49_238000	-3.00	5.17	0.002446444	0.00727635	peptidyl-prolyl isomerase
TGME49_225330	-1.14	6.63	0.002459416	0.00730659	hypothetical protein
TGME49_220300	-1.53	5.78	0.002459576	0.00730659	ribosomal protein L15 protein
TGME49_305820	-1.53	5.90	0.002472192	0.007339811	SGS domain-containing protein
TGME49_229160	-3.02	4.89	0.002473737	0.007339811	DHHC zinc finger domain-containing protein
TGME49_264890	-2.35	5.24	0.002502475	0.007407245	hypothetical protein
TGME49_216870	-1.78	5.83	0.002511365	0.007430585	DNA excision repair helicase
TGME49_313160	-2.33	5.73	0.002544107	0.007518435	hypothetical protein
TGME49_232760	1.05	6.48	0.002556018	0.007547603	protein phosphatase inhibitor IPP2
TGME49_258380	-2.01	5.55	0.002572926	0.007585414	elongation factor p (ef-p) kow family domain-contain
TGME49_309010	1.77	5.04	0.002604122	0.007674323	elongation factor P, putative
TGME49_295950	1.48	6.77	0.002609418	0.007683805	KRUF family protein
TGME49_201800	-1.08	7.11	0.002615892	0.007699803	hypothetical protein
TGME49_253820	1.03	8.07	0.002619986	0.007708784	hypothetical protein
TGME49_267760	-1.67	5.83	0.002625807	0.007719767	hypothetical protein
TGME49_269417	-2.99	4.68	0.002624837	0.007719767	hypothetical protein
TGME49_251510	1.46	6.13	0.002635119	0.007744066	Ulp1 protease family, C-terminal catalytic domain-co
TGME49_295090	1.32	7.42	0.002677128	0.007855033	hypothetical protein
TGME49_216220	-1.21	6.84	0.002697224	0.007907721	AP2 domain transcription factor AP2XI-5
TGME49_289720	-1.42	6.27	0.00271392	0.007944073	hypothetical protein
TGME49_249320	-1.99	5.46	0.00271724	0.007950643	flavodoxin domain-containing protein
TGME49_320670	-1.24	6.65	0.002719138	0.007953047	vacuolar protein sorting 16, putative
TGME49_244630	1.48	4.89	0.002735487	0.007988228	hypothetical protein
TGME49_269700	1.67	8.27	0.002738627	0.007994239	NLI interacting factor family phosphatase
TGME49_240430	1.11	5.61	0.002759083	0.008047597	glyoxalase family protein
TGME49_318610	1.10	5.24	0.002763445	0.008053966	AP2 domain transcription factor AP2IV-3

TGME49_219510	1.85	6.27	0.002771776	0.00806553	GTP binding protein 7 isoform 2 family protein, putat
TGME49_295590	1.36	4.96	0.002785514	0.008096603	hypothetical protein
TGME49_313790	1.11	6.66	0.002798156	0.008125506	hypothetical protein
TGME49_313690	-1.22	6.61	0.002798981	0.008125506	Sel1 repeat-containing protein
TGME49_288900	1.26	5.12	0.002800457	0.008126541	Yos1 family protein
TGME49_285930	-1.89	5.78	0.002806217	0.008136929	hypothetical protein
TGME49_217730	1.06	5.67	0.00283264	0.008203894	hypothetical protein
TGME49_272570	1.57	5.28	0.002859161	0.008270514	dihydrouridine synthase (dus) protein
TGME49_319710	-1.36	6.22	0.002863547	0.008277195	kinesin motor domain-containing protein
TGME49_316240	-1.51	6.03	0.002872813	0.008297492	hypothetical protein
TGME49_261490	-1.03	6.83	0.002897467	0.008365431	hypothetical protein
TGME49_315360	-1.25	6.61	0.00290427	0.0083818	hypothetical protein
TGME49_275420	-1.37	6.42	0.002909488	0.008393585	histone lysine-specific demethylase LSD1/BHC110/KD
TGME49_262860	-2.30	5.32	0.002937342	0.008463257	ADP-ribosylation factor family protein 1, putative
TGME49_221990	-1.01	6.74	0.002960188	0.008516587	hypothetical protein
TGME49_223600	-1.99	5.45	0.002959758	0.008516587	hypothetical protein
TGME49_252490	-1.77	5.95	0.002981333	0.008563937	vacuolar protein sorting 29, putative
TGME49_244140	-3.04	4.78	0.002982437	0.008563937	hypothetical protein
TGME49_206605	1.20	5.59	0.003018304	0.008648146	hypothetical protein
TGME49_315500	-1.24	6.66	0.003016557	0.008648146	hypothetical protein
TGME49_210380	-1.18	6.73	0.003042051	0.008711434	hypothetical protein
TGME49_224330	-1.03	6.99	0.00305893	0.008750782	proteasome activator pa28 beta subunit protein
TGME49_262130	-1.41	6.15	0.003059342	0.008750782	hypothetical protein
TGME49_204880	-1.82	5.83	0.003075733	0.008790865	hypothetical protein
TGME49_255440	1.26	5.19	0.003097836	0.008840369	hypothetical protein
TGME49_221950	-1.13	6.81	0.00310505	0.008857538	splicesome-associated protein, putative
TGME49_320720	-2.34	5.10	0.003112788	0.008876187	hypothetical protein
TGME49_235960	-7.45	4.72	0.003179475	0.009048898	hypothetical protein
TGME49_223270	1.19	5.48	0.003180822	0.009049249	hypothetical protein
TGME49_221870	1.23	7.14	0.003205543	0.009109065	hypothetical protein
TGME49_203710	-1.16	8.63	0.003220576	0.009148267	AP2 domain transcription factor AP2VIIa-4
TGME49_288460	-1.02	7.02	0.003252131	0.009230808	hypothetical protein
TGME49_206700	-2.96	4.86	0.003266051	0.009266761	hypothetical protein
TGME49_288945	-1.50	6.28	0.003274442	0.009287007	hypothetical protein
TGME49_236840	-1.11	6.61	0.003276046	0.009287994	zinc finger (C-x8-C-x5-C-x3-H)-2, putative
TGME49_240950	-3.00	4.75	0.003290916	0.009323001	hypothetical protein
TGME49_279370	-1.05	6.55	0.003300887	0.009347668	SNARE associated Golgi protein
TGME49_312210	-2.28	5.52	0.003302935	0.009349884	hypothetical protein
TGME49_316750	-2.93	4.89	0.003319323	0.00939268	DEAD/DEAH box helicase domain-containing protein
TGME49_219100	-1.60	5.69	0.003342686	0.009455173	cyclin-dependent kinase regulatory subunit protein
TGME49_247640	1.04	5.99	0.003357632	0.009490187	hypothetical protein
TGME49_281420	-2.28	5.07	0.003360672	0.009491525	histone deacetylase HDAC1
TGME49_258100	-2.95	4.89	0.003418186	0.009650275	TPR repeat region protein
TGME49_269720	1.63	6.13	0.003425176	0.009664313	hypothetical protein
TGME49_276120	-2.46	4.94	0.003455137	0.009736008	histone lysine methyltransferase, SET, putative
TGME49_258080	-1.64	5.91	0.003472012	0.009776108	hypothetical protein
TGME49_299060	-1.08	6.76	0.003478994	0.009788311	sodium/hydrogen exchanger NHE2
TGME49_305870	-2.29	5.28	0.003481306	0.00979109	DAD family protein
TGME49_311740	-1.98	5.52	0.003507963	0.00986231	hypothetical protein
TGME49_313960	-2.93	5.10	0.00356258	0.009996856	ribosomal protein L19 protein
TGME49_231920	1.38	6.14	0.003598604	0.010086459	oxidoreductase, short chain dehydrogenase/reductas
TGME49_200250	-1.00	6.44	0.003609349	0.010112742	microneme protein MIC17A
TGME49_278780	-1.37	6.27	0.003631009	0.010169576	hypothetical protein
TGME49_275770	-1.31	6.31	0.003646872	0.010210138	hypothetical protein
TGME49_285830	-1.04	6.53	0.003652299	0.01022146	hypothetical protein

TGME49_215480	-1.74	5.60	0.003670492	0.010262531	Adenosine/AMP deaminase domain-containing prote
TGME49_264460	-2.02	5.15	0.003672526	0.010262531	DNA-directed RNA polymerase III RPC5
TGME49_305230	-2.30	4.82	0.003678592	0.010271718	hypothetical protein
TGME49_208990	1.21	5.29	0.003710934	0.01035139	hypothetical protein
TGME49_252400	2.06	5.14	0.003743208	0.010428514	HIT zinc finger protein
TGME49_295980	-1.62	5.98	0.003748378	0.010435056	hypothetical protein
TGME49_321600	-1.13	6.53	0.003804482	0.010575316	hypothetical protein
TGME49_307980	1.51	5.22	0.003850312	0.010689588	GTP-binding protein lepA, putative
TGME49_297360	-1.34	6.33	0.003860322	0.01070237	hypothetical protein
TGME49_288210	1.11	6.24	0.003872061	0.010730891	PUL domain-containing protein
TGME49_285680	1.13	5.89	0.003890662	0.010778403	dihydrolipoamide acyltransferase, putative
TGME49_214120	-2.91	4.78	0.003929782	0.010874555	hypothetical protein
TGME49_257060	-1.93	5.49	0.003949423	0.010924815	translation initiation factor sui1 protein
TGME49_213445	-1.43	7.01	0.003959912	0.010938937	hypothetical protein
TGME49_249450	-2.89	4.73	0.003959983	0.010938937	hypothetical protein
TGME49_213000	2.22	5.32	0.00397583	0.01097323	replication factor C, subunit 5, putative
TGME49_271240	-2.26	5.16	0.003985288	0.010995231	hypothetical protein
TGME49_260270	-1.44	6.12	0.00401493	0.011064626	HEAT repeat-containing protein
TGME49_255245	1.40	4.95	0.004056443	0.01116239	hypothetical protein
TGME49_306510	1.82	5.11	0.004069493	0.011185812	hypothetical protein
TGME49_252250	-1.61	5.07	0.004067014	0.011185812	ATPase, AAA family protein
TGME49_202730	-1.96	5.45	0.004068963	0.011185812	hypothetical protein
TGME49_269800	-1.93	5.58	0.004082836	0.011218316	glutamine-dependent NAD(+) synthetase protein, put
TGME49_230920	-2.86	4.80	0.004092128	0.011235497	adaptor complexes medium subunit family protein
TGME49_214630	-1.37	5.89	0.004179161	0.011446729	hypothetical protein
TGME49_231815	-1.98	5.33	0.004179898	0.011446729	hypothetical protein
TGME49_226040	-1.12	6.54	0.004184638	0.011446929	EF hand domain-containing protein
TGME49_300280	-1.96	5.77	0.004265226	0.011635056	LSM domain-containing protein
TGME49_298010	-1.28	7.06	0.004270243	0.011642394	hypothetical protein
TGME49_293700	-1.37	6.01	0.004276299	0.011650316	WD domain, G-beta repeat-containing protein
TGME49_301010	-1.13	6.37	0.004278004	0.011650668	serine/threonine protein phosphatase, putative
TGME49_203050	1.88	6.44	0.004296369	0.011687768	AP2 domain transcription factor AP2VIIa-6
TGME49_270370	-2.01	5.67	0.004323828	0.01175814	clathrin assembly protein AP19, putative
TGME49_278890	-1.95	5.41	0.004331526	0.011767317	hypothetical protein
TGME49_294750	-2.84	4.76	0.004329655	0.011767317	hypothetical protein
TGME49_229030	-1.13	6.70	0.004352671	0.011819189	hypothetical protein
TGME49_313660	1.32	4.89	0.0043673	0.011842859	hypothetical protein
TGME49_315980	-1.92	5.45	0.00437057	0.011846038	EREBP-4 family protein
TGME49_242625	-1.28	6.49	0.004380585	0.011860142	ATPase family associated with various cellular activiti
TGME49_287970	-1.02	7.38	0.004398939	0.011905475	hypothetical protein
TGME49_307020	-1.40	6.25	0.004411595	0.011930993	hypothetical protein
TGME49_227260	-2.26	5.33	0.004434004	0.011983425	RIO1 family protein
TGME49_263590	-1.94	5.74	0.004438824	0.011987095	hypothetical protein
TGME49_215550	-1.97	5.78	0.004490268	0.012103916	hypothetical protein
TGME49_224350	-1.10	8.79	0.004528888	0.012190242	aminopeptidase N, putative
TGME49_268000	-2.24	5.44	0.004528223	0.012190242	hypothetical protein
TGME49_224090	-1.72	5.72	0.004601123	0.012362174	enoyl-CoA hydratase/isomerase family protein
TGME49_246990	-2.22	5.31	0.004660605	0.012490219	hypothetical protein
TGME49_286640	-2.84	4.82	0.004723798	0.012632102	GTPase
TGME49_214170	-1.58	5.69	0.004771098	0.01273556	hypothetical protein
TGME49_270170	-1.56	5.71	0.00480516	0.012812604	hypothetical protein
TGME49_270650	1.20	5.24	0.004832209	0.01286617	deoxyribose-phosphate aldolase
TGME49_249850	-1.52	10.25	0.004899821	0.013027427	GAP40 protein
TGME49_218192	-7.38	5.36	0.004931956	0.013099018	hypothetical protein
TGME49_251590	1.31	5.99	0.004942692	0.013113118	hypothetical protein

TGME49_244150	-1.93	5.09	0.004942302	0.013113118	glycerate kinase
TGME49_201380	1.15	9.02	0.004950057	0.013123241	chorismate synthase, putative
TGME49_311510	-1.14	8.65	0.004967435	0.013164592	eIF2 kinase IF2K-B
TGME49_208360	1.14	5.38	0.005011909	0.01326344	hypothetical protein
TGME49_255670	-2.82	4.91	0.005058391	0.013376875	methyltransferase domain-containing protein
TGME49_259600	-1.56	5.75	0.005100183	0.013477754	hypothetical protein
TGME49_249690	-2.89	5.20	0.005125259	0.013529512	hypothetical protein
TGME49_249310	-2.82	5.20	0.005157622	0.01360523	hypothetical protein
TGME49_285720	-2.21	4.96	0.005175359	0.013642283	ATP binding protein, putative
TGME49_313150	1.58	6.73	0.005185547	0.013659401	DUF89/Fructose bisphosphatase
TGME49_230010	-1.43	6.26	0.005208305	0.013697478	hypothetical protein
TGME49_305800	-1.54	5.93	0.005207028	0.013697478	6-pyruvoyl tetrahydrobiopterin synthase
TGME49_277710	-1.08	6.64	0.005214466	0.013701407	hypothetical protein
TGME49_297330	1.06	5.60	0.005272747	0.013838266	hypothetical protein
TGME49_294380	-2.19	5.40	0.00529015	0.013865782	PP-loop domain-containing protein
TGME49_249160	-2.22	4.70	0.005302216	0.013892483	UAA transporter family protein
TGME49_258650	-2.21	5.11	0.005313041	0.013910986	protoheme ferro-lyase, putative
TGME49_263530	-1.22	6.47	0.005341449	0.013980415	chaperonin , putative
TGME49_221270	-1.11	6.87	0.005360909	0.014016464	oxidoreductase, 2OG-Fe(II) oxygenase family protein
TGME49_249950	-2.20	5.46	0.00535967	0.014016464	Mak16 protein
TGME49_267440	1.14	5.72	0.005371409	0.014038953	RING zinc finger protein
TGME49_260480	-2.84	4.87	0.005390978	0.014080145	leucine rich repeat-containing protein
TGME49_226840	-2.20	5.41	0.005408163	0.014110076	hypothetical protein
TGME49_312360	-2.81	4.90	0.005436462	0.014168911	hypothetical protein
TGME49_292035	1.40	4.87	0.005501565	0.014308327	hypothetical protein
TGME49_228780	1.11	5.46	0.005510525	0.014326589	Toxoplasma gondii family C protein
TGME49_227440	1.02	5.77	0.005513505	0.014329298	WD domain, G-beta repeat-containing protein
TGME49_202350	-1.53	5.81	0.005565556	0.014444269	50S ribosomal protein L21, putative
TGME49_286030	-1.08	6.65	0.005570172	0.014451175	hypothetical protein
TGME49_219530	-2.83	5.05	0.00560999	0.01453408	hypothetical protein
TGME49_213670	-1.10	6.71	0.005644578	0.014608333	hypothetical protein
TGME49_262000	-2.84	4.73	0.005677375	0.014682935	AP2 domain transcription factor AP2VIIb-2
TGME49_285700	-2.83	4.70	0.005763416	0.01488169	ubiquitin fusion degradation protein UFD1AP
TGME49_278110	-1.59	5.83	0.005783631	0.014921203	1,3-beta-glucan synthase component protein
TGME49_280590	-2.85	4.77	0.0057965	0.014942835	hypothetical protein
TGME49_259080	1.81	6.29	0.005823562	0.014992832	hypothetical protein
TGME49_203750	-1.89	5.36	0.005821952	0.014992832	hypothetical protein
TGME49_240480	-2.92	4.71	0.005830265	0.015001769	cpw-wpc domain-containing protein
TGME49_305010	-1.73	5.59	0.005833232	0.015002058	pre-mRNA branch site protein p14, putative
TGME49_278250	1.24	5.20	0.005857553	0.015059371	hypothetical protein
TGME49_230890	-1.40	5.65	0.005908812	0.015175324	PHD-finger domain-containing protein
TGME49_319308	-1.88	6.63	0.006031999	0.015470206	hypothetical protein
TGME49_252230	-1.16	6.25	0.006046211	0.015495904	hypothetical protein
TGME49_222300	-1.18	6.16	0.006066599	0.01554277	hypothetical protein
TGME49_234560	-1.19	6.18	0.006137311	0.015694963	hypothetical protein
TGME49_309780	-1.66	5.56	0.006138738	0.015694963	hypothetical protein
TGME49_258940	1.34	5.05	0.006148779	0.015709769	acylphosphatase family protein
TGME49_285880	1.31	4.94	0.006147064	0.015709769	hypothetical protein
TGME49_239690	1.21	5.52	0.006153682	0.015711438	hypothetical protein
TGME49_216580	-1.43	6.03	0.006152237	0.015711438	hypothetical protein
TGME49_228170	1.04	9.27	0.006210194	0.015812044	inner membrane complex protein IMC2A
TGME49_276190	-1.91	5.61	0.006221052	0.015828789	hypothetical protein
TGME49_305030	-1.51	5.98	0.00624691	0.015878189	kinase, pfkB family protein
TGME49_259650	-2.79	4.78	0.006316068	0.016037433	hypothetical protein
TGME49_289950	-1.16	6.53	0.006402046	0.016233445	hypothetical protein

TGME49_306890	-1.42	5.84	0.006427923	0.016276735	hypothetical protein
TGME49_206300	-2.18	5.15	0.006434809	0.016287333	hypothetical protein
TGME49_255340	-1.09	6.51	0.006455532	0.016329868	tetratricopeptide repeat-containing protein
TGME49_242320	-1.15	6.85	0.00648574	0.016400671	B-box zinc finger domain-containing protein
TGME49_218200	-2.17	5.33	0.006555763	0.016566408	UDP-sugar pyrophospharylase
TGME49_309880	-1.14	6.92	0.006626278	0.016704637	hypothetical protein
TGME49_232090	-1.63	5.75	0.006669645	0.016796784	3-hydroxyacyl-CoA dehydrogenase, NAD binding dom
TGME49_218960	-1.28	8.73	0.006675015	0.016804584	AP2 domain transcription factor AP2XII-1
TGME49_225120	1.05	9.47	0.006717548	0.016894405	hypothetical protein
TGME49_314710	-1.22	6.29	0.006773238	0.017028672	carrier superfamily protein
TGME49_288990	-2.21	5.24	0.006847481	0.017197783	hypothetical protein
TGME49_209870	-1.84	5.26	0.006998783	0.017536089	HAD hydrolase, family IIA protein
TGME49_278060	-1.62	5.50	0.007009885	0.017557957	Mre11 DNA-binding domain-containing protein
TGME49_238070	-2.73	5.04	0.007014219	0.017562864	glutaredoxin domain-containing protein
TGME49_231890	-1.41	6.02	0.007048828	0.017637575	beta-ketoacyl-acyl carrier protein synthase III, putativ
TGME49_275740	-2.83	4.73	0.007105599	0.017761596	hypothetical protein
TGME49_246690	-1.83	5.40	0.007189647	0.017941361	alpha amylase, catalytic domain-containing protein
TGME49_304650	-1.23	5.99	0.00722676	0.018021808	histidine acid phosphatase superfamily protein
TGME49_284040	-1.33	6.15	0.007249734	0.018073006	hypothetical protein
TGME49_313970	-1.87	5.45	0.007370295	0.018354989	Phytanoyl-CoA dioxygenase (PhyH) superfamily prote
TGME49_298060	-2.00	5.15	0.00737964	0.018372073	Toxoplasma gondii family C protein
TGME49_263560	1.29	7.28	0.007449424	0.018514635	hypothetical protein
TGME49_249190	1.34	5.13	0.007476296	0.018568939	AP2 domain transcription factor AP2XII-6
TGME49_218580	-1.63	5.45	0.007480997	0.018574376	RNA methyltransferase, TrmH family protein
TGME49_277895	-1.39	5.67	0.007497319	0.018608653	ubiquitin carboxyl-terminal hydrolase
TGME49_236130	1.74	6.02	0.007581343	0.018791988	signal recognition particle (SRP9) domain-containing
TGME49_273310	-1.30	6.16	0.007584417	0.018791988	hypothetical protein
TGME49_226620	-1.65	5.71	0.007600105	0.01881955	hypothetical protein
TGME49_320610	-2.18	5.08	0.007617022	0.018855126	hypothetical protein
TGME49_250060	1.17	5.19	0.007642705	0.01891237	DNA-directed RNA polymerase I RPA12
TGME49_234610	-1.14	6.61	0.007649903	0.018917518	WD-40 repeat protein
TGME49_211610	1.00	5.73	0.007726515	0.019087822	hypothetical protein
TGME49_312310	-2.15	5.42	0.00773769	0.019109044	ATPase, AAA family protein
TGME49_305620	-1.42	5.96	0.007792918	0.019226172	hypothetical protein
TGME49_292610	-7.13	4.84	0.00780307	0.019244795	Toxoplasma gondii family C protein
TGME49_215080	-1.29	5.65	0.007807697	0.019248637	hypothetical protein
TGME49_266380	-7.15	5.23	0.007934623	0.019523661	hypothetical protein
TGME49_275750	-1.31	6.27	0.008033354	0.019753449	small nuclear ribonucleoprotein E, putative
TGME49_318320	-2.16	5.00	0.0080557	0.01980181	ATP-dependent Clp endopeptidase, proteolytic subur
TGME49_229790	-1.61	5.71	0.008122005	0.019938282	hypothetical protein
TGME49_310730	-1.10	6.50	0.008146735	0.019985721	hypothetical protein
TGME49_203030	1.10	7.98	0.008183783	0.020063293	N-methyl-D-aspartate receptor-associated protein
TGME49_234370	-2.22	5.10	0.008197832	0.020091074	SAG-related sequence SRS42
TGME49_240490	-2.20	4.97	0.0082745	0.020245418	hypothetical protein
TGME49_222940	1.75	5.07	0.008444826	0.020588801	hypothetical protein
TGME49_289910	-1.02	7.86	0.008484847	0.020670116	hypothetical protein
TGME49_235905	-2.10	5.09	0.008487216	0.020670116	ribonuclease z, putative
TGME49_279540	-7.14	4.99	0.008560928	0.020801676	hypothetical protein
TGME49_230990	1.32	5.02	0.008637767	0.020947081	hypothetical protein
TGME49_204040	-2.11	4.98	0.008632303	0.020947081	hypothetical protein
TGME49_297790	-1.46	6.27	0.00876696	0.021218626	hypothetical protein
TGME49_258030	-1.31	6.12	0.008771069	0.021221623	DNA polymerase
TGME49_211020	-1.46	5.85	0.008801384	0.021288006	RNA recognition motif-containing protein
TGME49_214500	1.12	6.17	0.00881498	0.021293028	ankyrin repeat-containing protein
TGME49_268010	-1.26	5.82	0.008839642	0.021345627	hypothetical protein

TGME49_308030	-1.38	5.86	0.008856965	0.021373499	hypothetical protein
TGME49_263580	1.49	6.93	0.008874242	0.021404473	bromodomain-containing protein
TGME49_215940	-1.15	6.63	0.00887559	0.021404473	Acetyl-coenzyme A transporter, putative
TGME49_220150	-1.79	5.32	0.008896105	0.021446952	50S ribosomal protein L16, putative
TGME49_226980	1.29	7.50	0.009041628	0.021755898	hypothetical protein
TGME49_268560	-1.60	5.40	0.009041909	0.021755898	XPG N-terminal domain-containing protein
TGME49_207720	-1.26	6.17	0.009153016	0.021980319	hypothetical protein
TGME49_293490	1.78	4.86	0.009298033	0.022286452	hypothetical protein
TGME49_218610	-1.64	5.51	0.009298581	0.022286452	ATPase (DUF699) protein
TGME49_265080	-1.01	6.57	0.009310431	0.022300397	Tubulin-tyrosine ligase family protein
TGME49_301450	-1.00	6.33	0.009389306	0.022452952	FG-GAP repeat-containing protein
TGME49_285470	1.28	5.36	0.00948134	0.022658379	patched family protein
TGME49_233300	-2.07	5.35	0.009501348	0.0226842	RhoGAP domain-containing protein
TGME49_318720	1.15	6.14	0.00957527	0.0228312	pyridoxal phosphate enzyme, YggS family protein
TGME49_320470	1.90	5.10	0.009593188	0.022866549	hypothetical protein
TGME49_289010	-1.78	5.19	0.009677922	0.023033076	RNA recognition motif-containing protein
TGME49_231040	-2.69	4.79	0.009678627	0.023033076	3' exoribonuclease family, domain 1 domain-containi
TGME49_225108	-1.44	5.44	0.00978209	0.023256841	SNF7 family protein
TGME49_257780	-1.44	5.65	0.009788924	0.023265608	hypothetical protein
TGME49_238040	-1.07	6.79	0.009855636	0.023416636	protein disulfide-isomerase domain-containing prote
TGME49_209090	-1.13	6.52	0.009912401	0.023543943	proteasome maturation factor ump1 protein
TGME49_232730	1.04	6.03	0.010040255	0.023824662	acyl-CoA:diacylglycerol acyltransferase 1-related enzy
TGME49_216920	-1.80	5.62	0.010091085	0.023929918	mediator complex subunit MED8
TGME49_300330	1.01	5.61	0.010124859	0.023994618	hypothetical protein
TGME49_316520	-1.13	6.34	0.010138203	0.024018544	1,4-alpha-glucan-branching enzyme
TGME49_294812	-2.65	5.19	0.010158995	0.024060093	RNA recognition motif-containing protein
TGME49_215250	-1.77	5.32	0.010213845	0.02417451	thiamin pyrophosphokinase, catalytic domain-contair
TGME49_243240	1.37	4.74	0.010225547	0.024187357	WD domain, G-beta repeat-containing protein
TGME49_216960	1.04	5.62	0.010231948	0.024194123	elongation factor Tu GTP binding domain-containing
TGME49_214400	-2.07	5.11	0.010353244	0.024457471	hypothetical protein
TGME49_294902	-1.58	5.09	0.01047504	0.024696658	hypothetical protein
TGME49_264200	-1.76	5.19	0.01050678	0.024756893	hypothetical protein
TGME49_258050	-2.05	5.14	0.010595698	0.024942562	actin like protein ALP2a
TGME49_210478	-1.44	4.78	0.010600783	0.02494659	hypothetical protein
TGME49_245435	1.27	5.14	0.010792786	0.025350018	hypothetical protein
TGME49_310770	-1.15	6.43	0.010804695	0.025361877	hypothetical protein
TGME49_263710	-1.06	6.32	0.010834779	0.025416356	acyl-CoA:cholesterol acyltransferase alpha ACAT1-alp
TGME49_301460	1.13	5.61	0.01094793	0.025665503	hypothetical protein
TGME49_206500	-2.71	4.78	0.010996729	0.02574725	hypothetical protein
TGME49_262450	-1.06	7.29	0.011092563	0.02591419	hypothetical protein
TGME49_247590	-1.75	5.36	0.011247444	0.026259425	methyltransferase domain-containing protein
TGME49_240450	1.35	5.84	0.011276774	0.026319591	Maf family protein
TGME49_215450	1.99	6.55	0.011385053	0.026555547	aquaporin 1
TGME49_239580	-7.04	4.82	0.011452633	0.026671109	hypothetical protein
TGME49_217500	2.01	7.03	0.011496637	0.026765154	HMG (high mobility group) box domain-containing pr
TGME49_257500	1.30	5.05	0.011632817	0.027056637	hypothetical protein
TGME49_288640	1.19	5.01	0.011658328	0.027107445	radical SAM domain-containing protein
TGME49_208430	1.13	5.23	0.011724461	0.027218418	serine proteinase inhibitor PI-2, putative
TGME49_210960	-1.20	6.30	0.011771802	0.027319744	replication factor C subunit 4, putative
TGME49_231930	-2.67	4.73	0.011869558	0.027537971	hypothetical protein
TGME49_286510	-1.62	5.50	0.011902661	0.027572741	hypothetical protein
TGME49_220260	1.22	5.44	0.011920663	0.027587287	hypothetical protein
TGME49_247240	-1.21	6.78	0.011916601	0.027587287	ubiquitin carboxyl-terminal hydrolase, family 1 protei
TGME49_242240	-1.15	6.05	0.012127112	0.02801942	rhoptry kinase family protein ROP19A
TGME49_227920	-1.40	5.69	0.012240514	0.028265571	hypothetical protein

TGME49_225420	-2.02	5.08	0.012252115	0.028279725	histidine triad domain-containing protein
TGME49_256910	1.28	5.85	0.012294318	0.028363242	hypothetical protein
TGME49_219850	-1.03	8.47	0.012404611	0.028590928	prolyl-tRNA synthetase (ProRS)
TGME49_248200	1.03	6.31	0.012409435	0.028593134	ribosomal RNA (adenine(1779)-N(6)/adenine(1780)-N
TGME49_238130	-1.23	5.86	0.012494858	0.028772027	hypothetical protein
TGME49_273840	-1.75	5.63	0.012535682	0.028848066	brix domain-containing protein
TGME49_207950	-1.42	5.76	0.01255318	0.028879345	hypothetical protein
TGME49_243310	1.37	6.29	0.012561479	0.028889448	hypothetical protein
TGME49_237840	-1.42	5.52	0.012565937	0.028890716	hypothetical protein
TGME49_256920	1.22	6.24	0.012607514	0.02896829	phosphatidylinositol-4-phosphate 5-Kinase
TGME49_258550	-1.36	5.48	0.012731707	0.029217331	SAG-related sequence SRS28
TGME49_247370	-1.40	5.45	0.012757125	0.029266578	hypothetical protein
TGME49_234520	-1.54	5.92	0.01279096	0.029335098	U2 snRNP auxilliary factor, large subunit, splicing fact
TGME49_257070	-2.02	5.02	0.012830107	0.029406637	hypothetical protein
TGME49_263190	-2.61	4.74	0.01282891	0.029406637	adenylosuccinate lyase, putative
TGME49_213020	1.30	5.21	0.013134745	0.030030398	hypothetical protein
TGME49_295680	-1.29	5.74	0.013130854	0.030030398	periodic tryptophan protein PWP2, putative
TGME49_269760	1.16	5.00	0.013195531	0.030150727	'chromo' (CHRromatin Organization MOdifier) domai
TGME49_289130	-2.02	4.92	0.013193432	0.030150727	hypothetical protein
TGME49_255320	1.18	5.48	0.013286924	0.030309164	mRNA turnover 4 (MRT4) family protein
TGME49_231625	-1.76	4.86	0.013294168	0.030309164	hypothetical protein
TGME49_299030	1.06	7.26	0.013318972	0.030348376	RNA recognition motif 2 protein
TGME49_206470	1.11	8.87	0.013364878	0.030424849	pyruvate dehydrogenase complex subunit PDH-E3II
TGME49_228010	-1.53	5.18	0.013394635	0.030472265	hypothetical protein
TGME49_273500	-1.78	5.42	0.013398071	0.030472265	O-linked N-acetylglucosamine transferase
TGME49_263440	-1.71	5.39	0.013435268	0.030538078	hypothetical protein
TGME49_235660	-1.29	5.76	0.013603297	0.030891516	hypothetical protein
TGME49_305260	-2.57	4.78	0.013614842	0.030898753	hypothetical protein
TGME49_312660	-1.51	5.79	0.013756988	0.031163961	hypothetical protein
TGME49_256820	-1.52	5.47	0.013775466	0.031177164	zinc finger (CCCH type) motif-containing protein
TGME49_269630	1.13	5.06	0.01378414	0.03118725	hypothetical protein
TGME49_243750	2.11	5.73	0.013841599	0.031298099	tetratricopeptide repeat-containing protein
TGME49_236990	1.16	5.51	0.01390239	0.031384699	beta-ketoacyl synthase, N-terminal domain-containin
TGME49_218000	-1.71	5.14	0.013976744	0.03150733	hypothetical protein
TGME49_309940	-1.30	5.91	0.014182457	0.031941847	phospholipase D active site domain-containing protei
TGME49_222870	-1.16	6.27	0.014214822	0.031975781	hypothetical protein
TGME49_305460	1.60	6.98	0.0142746	0.032072385	methionine aminopeptidase 2, putative
TGME49_217951	-1.28	5.22	0.014398835	0.032330698	hypothetical protein
TGME49_272290	-1.07	6.44	0.014411893	0.032340376	pyruvate dehydrogenase complex subunit PD-HE1Bet
TGME49_267970	-1.22	5.73	0.014442853	0.032400017	DEAD/DEAH box helicase domain-containing protein
TGME49_215100	-1.38	5.36	0.014452878	0.032404534	PP-loop family protein
TGME49_219218	-1.31	5.44	0.014679816	0.032851865	hypothetical protein
TGME49_237160	-1.25	6.04	0.014767743	0.033028642	hypothetical protein
TGME49_230230	1.08	5.70	0.014875292	0.033208306	hypothetical protein
TGME49_216440	2.43	5.49	0.015080716	0.033610654	OTU family cysteine protease
TGME49_238510	-1.71	5.29	0.015403267	0.034212002	hypothetical protein
TGME49_233120	-1.16	6.11	0.015740653	0.034929889	AP2 domain transcription factor AP2VIII-2
TGME49_275840	-1.96	4.89	0.015759305	0.034960787	protein phosphatase 2C domain-containing protein
TGME49_220530	1.37	5.63	0.015769093	0.034972008	AP2 domain transcription factor AP2V-1
TGME49_267460	-1.97	4.87	0.015841671	0.035101384	AP2 domain transcription factor AP2IX-1
TGME49_298050	-1.36	5.70	0.015860673	0.035132959	hypothetical protein
TGME49_222430	-1.27	5.65	0.015896904	0.035202668	HECT-domain (ubiquitin-transferase) domain-contain
TGME49_211730	-1.55	7.28	0.015915165	0.035222008	histone lysine methyltransferase SET8
TGME49_214740	-1.09	6.15	0.015936971	0.035259715	hypothetical protein
TGME49_214990	-1.36	5.69	0.015964027	0.03529845	hypothetical protein

TGME49_254510	1.19	4.84	0.016188266	0.035730162	ankyrin repeat-containing protein
TGME49_234470	-1.36	5.77	0.016266731	0.035881924	hypothetical protein
TGME49_253070	-2.03	4.88	0.016276663	0.035886353	hydrolase, TatD family protein
TGME49_289340	1.80	5.08	0.016297785	0.035907576	hypothetical protein
TGME49_233245	-1.96	4.98	0.016374925	0.036052168	hypothetical protein
TGME49_207065	-1.48	5.59	0.016570285	0.036431961	hypothetical protein
TGME49_203280	-1.98	5.13	0.016633489	0.036549188	hypothetical protein
TGME49_220480	-2.53	4.69	0.016631977	0.036549188	hypothetical protein
TGME49_297080	-1.48	5.34	0.016739682	0.036771601	pyridoxal kinase
TGME49_288700	-1.11	6.08	0.016836162	0.036972553	RecF/RecN/SMC N terminal domain-containing prote
TGME49_278170	-1.69	5.42	0.016889681	0.037079067	hypothetical protein
TGME49_271610	-1.05	5.94	0.016932639	0.037162343	pyrroline-5-carboxylate reductase
TGME49_226240	1.13	5.14	0.0169814	0.037258301	bud site selection protein, putative
TGME49_231280	1.09	5.24	0.017039769	0.03736419	WD domain, G-beta repeat-containing protein
TGME49_262933	-1.56	5.41	0.017091721	0.037433705	hypothetical protein
TGME49_264420	2.95	4.97	0.017151343	0.037553162	lipoprotein, putative
TGME49_286140	-1.35	5.93	0.017185876	0.037617633	hypothetical protein
TGME49_316770	-1.34	5.68	0.017322708	0.037883498	undecaprenyl diphosphate synthase
TGME49_267450	-1.01	6.26	0.017419385	0.038039339	alpha-tubulin suppressor protein
TGME49_279390	-1.07	9.03	0.017465636	0.038117152	proliferation-associated protein 2G4, putative
TGME49_308930	-1.24	5.90	0.017546926	0.038283261	50S ribosomal protein L33, putative
TGME49_268240	1.24	4.85	0.01757414	0.038308723	hypothetical protein
TGME49_227820	-1.10	6.10	0.017566566	0.038308723	hypothetical protein
TGME49_228110	-1.24	5.67	0.017589269	0.038330402	hypothetical protein
TGME49_226068	-1.02	6.39	0.017645615	0.038430539	DnaJ domain-containing protein
TGME49_217900	-1.14	6.15	0.017644452	0.038430539	hypothetical protein
TGME49_257390	-1.97	4.86	0.017794235	0.038720003	ribosome biogenesis GTP-binding protein YsxC protei
TGME49_248770	-1.51	5.27	0.017824789	0.038775077	hypothetical protein
TGME49_236140	1.10	4.81	0.017846907	0.038800362	hypothetical protein
TGME49_258010	-1.52	5.57	0.017895722	0.038895052	calcium signaling protein kinase RAD53, putative
TGME49_216700	-2.50	4.75	0.017948096	0.038985964	hypothetical protein
TGME49_273130	1.43	6.10	0.018595829	0.040251042	SAG-related sequence SRS30A
TGME49_231940	-1.13	6.36	0.018593713	0.040251042	ThiF family protein
TGME49_273720	-1.15	5.78	0.018739513	0.040514607	hypothetical protein
TGME49_231220	-1.93	4.89	0.018766528	0.040561154	hypothetical protein
TGME49_218840	-1.23	5.72	0.01884948	0.040716639	mutS domain protein
TGME49_283585	1.23	5.01	0.018909831	0.040811235	hypothetical protein
TGME49_249770	1.47	5.99	0.018916986	0.040814763	Nmda1 protein
TGME49_229350	-1.24	6.02	0.018997985	0.040953673	HEAT repeat-containing protein
TGME49_291120	-1.23	5.68	0.019026072	0.041002267	trafficking protein mon1 subfamily protein
TGME49_239070	-1.47	5.50	0.019146791	0.041226375	hypothetical protein
TGME49_287220	1.78	6.36	0.01924184	0.041394869	hypothetical protein
TGME49_258826	-1.74	5.07	0.019241358	0.041394869	hypothetical protein
TGME49_267650	-1.36	5.66	0.019319793	0.041524904	hypothetical protein
TGME49_268290	-2.00	4.85	0.019443757	0.041744234	DEAD/DEAH box helicase domain-containing protein
TGME49_226390	2.08	5.45	0.019469676	0.041785621	hypothetical protein
TGME49_243910	1.05	6.24	0.01955587	0.041911914	Cof family hydrolase subfamily protein
TGME49_219230	-1.11	6.00	0.019882144	0.0425126	AMP-binding enzyme domain-containing protein
TGME49_255660	-1.14	6.24	0.020127349	0.042962361	EF hand domain-containing protein
TGME49_299015	-6.90	4.85	0.020226484	0.04312417	hypothetical protein
TGME49_242110	-6.77	4.87	0.020318873	0.04329618	rhoptry kinase family protein ROP38
TGME49_232310	-1.63	5.22	0.020405921	0.043444105	endonuclease/exonuclease/phosphatase family prote
TGME49_277680	-1.13	6.15	0.020490312	0.043611216	hypothetical protein
TGME49_270070	2.20	4.92	0.020583888	0.043772583	synaptobrevin family protein
TGME49_267390	-1.02	6.54	0.020623539	0.04383169	DNA-directed RNA polymerase I RPAC1

TGME49_240700	-1.21	5.96	0.020779174	0.044137091	ubiquitin family protein
TGME49_210440	1.04	6.10	0.020874685	0.044263672	polynucleotide adenylyltransferase
TGME49_217000	-1.21	5.87	0.020883682	0.044270052	hypothetical protein
TGME49_228380	2.35	5.03	0.021125391	0.044679959	hypothetical protein
TGME49_242880	-1.61	5.25	0.021184832	0.044792866	flavoprotein
TGME49_270300	1.54	6.74	0.021243675	0.044891608	hypothetical protein
TGME49_232590	1.28	6.36	0.021383923	0.045162161	glutamate-cysteine ligase, catalytic subunit domain-c
TGME49_229200	-1.32	5.54	0.021566084	0.045481924	hypothetical protein
TGME49_230140	-1.13	5.82	0.021712654	0.045699791	vacuolar sorting protein 9 (vps9) domain-containing p
TGME49_294420	-1.89	4.78	0.021734708	0.04572018	programmed cell death protein 2, c-terminal domain-
TGME49_249550	-1.14	5.76	0.021819057	0.045845801	hypothetical protein
TGME49_319600	-1.17	5.53	0.021853669	0.045905122	alpha-tubulin N-acetyltransferase, putative
TGME49_262700	1.19	6.44	0.021882961	0.045942243	tetratricopeptide repeat-containing protein
TGME49_306590	1.35	6.35	0.021986471	0.046129273	hypothetical protein
TGME49_205550	-1.43	5.44	0.022182214	0.046476421	AGC kinase
TGME49_225190	2.20	4.76	0.022626583	0.047300241	hypothetical protein
TGME49_217340	-1.15	5.87	0.022864507	0.047703207	hypothetical protein
TGME49_200460	1.18	6.56	0.02304527	0.048053221	hypothetical protein
TGME49_264670	-1.01	6.15	0.023163067	0.048230841	DNA polymerase family B protein
TGME49_316490	1.24	5.08	0.023199568	0.048279653	hypothetical protein
TGME49_313530	-1.06	5.98	0.023314296	0.048463847	transmembrane protein 167, putative
TGME49_245530	2.09	5.75	0.023328531	0.048479809	hypothetical protein
TGME49_231870	-1.60	5.18	0.023339488	0.04848895	tetratricopeptide repeat-containing protein
TGME49_208850	-1.13	5.79	0.0234156	0.048606106	SAG-related sequence SRS11
TGME49_314390	-1.19	5.75	0.023441938	0.048647121	hypothetical protein
TGME49_213690	-1.28	5.66	0.024089887	0.049865796	ring box protein 1 family protein
TGME49_257980	2.23	4.93	0.024102311	0.049870321	ribosome recycling factor protein
TGME49_320690	-1.29	5.76	0.024112307	0.049870321	gamma-soluble NSF attachment protein, putative

Supplementary Table S7C: Differencial expression analysis of T. gondii genes from neuronal stem cells infected with T. gondii EGS strain for 18 hours copmared to T. gondii gene expression from neuronal stem cells infected with VEG strain for 18 hours.

gene ID	logFC (EGS/VEG)	logCPM	PValue	FDR	Product Name
TGME49_233925	9.00	7.83	1.77E-211	1.31E-207	hypothetical protein
TGME49_322200	12.41	6.70	2.90E-166	1.07E-162	apocytochrome b, putative
TGME49_237130	10.14	10.26	1.50E-137	3.70E-134	cytochrome b, putative
TGME49_322800	12.26	6.48	5.08E-113	9.40E-110	hypothetical protein
TGME49_330000	10.97	10.23	6.79E-110	1.00E-106	cytochrome b
TGME49_205250	11.29	9.21	5.03E-106	6.20E-103	rhoptry protein ROP18
TGME49_323400	10.92	9.06	1.40E-104	1.47E-101	cytochrome c oxidase subunit iii subfamily protein
TGME49_320050	4.35	13.43	5.09E-83	4.70E-80	ribosomal protein RPL5
TGME49_302055	11.93	5.97	3.07E-81	2.52E-78	ribosomal protein RPS12
TGME49_255060	10.42	9.95	4.80E-72	3.55E-69	cytochrome b(N-terminal)/b6/petB subfamily protein
TGME49_293790	8.48	6.72	3.82E-60	2.57E-57	hypothetical protein
TGME49_238240	2.64	8.97	1.06E-58	6.55E-56	bystin protein
TGME49_261240	5.94	5.02	7.31E-58	4.16E-55	histone H3
TGME49_214080	6.66	8.38	3.20E-57	1.69E-54	toxofilin
TGME49_290600	2.50	9.11	1.80E-56	8.88E-54	succinyl-CoA-synthetase alpha SCSA
TGME49_232955	13.87	12.33	9.24E-55	4.27E-52	hypothetical protein
TGME49_321360	2.94	8.21	2.13E-53	9.27E-51	clustered-asparagine-rich protein
TGME49_301250	8.14	14.41	2.88E-52	1.18E-49	hypothetical protein
TGME49_288360	-3.81	10.08	3.39E-50	1.32E-47	tryptophanyl-tRNA synthetase (TrpRS2)
TGME49_221840	6.21	4.71	5.34E-47	1.98E-44	hypothetical protein
TGME49_217530	4.18	6.35	4.73E-43	1.66E-40	hypothetical protein
TGME49_277230	3.76	5.63	7.71E-40	2.59E-37	hypothetical protein
TGME49_252070	10.19	5.12	5.10E-39	1.64E-36	KRUF family protein
TGME49_262690	1.88	10.98	5.67E-36	1.75E-33	ribosomal protein RPL27
TGME49_261720	-6.53	7.82	2.19E-35	6.46E-33	metal cation transporter, ZIP family protein
TGME49_223660	3.20	7.34	2.18E-34	6.19E-32	50S ribosomal protein L4, putative
TGME49_208020	4.25	6.12	7.27E-33	1.99E-30	AP2 domain transcription factor AP2Ib-1
TGME49_251180	2.68	6.80	2.56E-31	6.75E-29	KRUF family protein
TGME49_228160	2.44	7.19	5.22E-31	1.33E-28	acid phosphatase
TGME49_244370	3.44	8.80	7.15E-31	1.76E-28	TDC1, putative
TGME49_269310	5.30	4.71	1.18E-30	2.72E-28	hypothetical protein
TGME49_315885	2.61	7.08	2.99E-28	6.50E-26	glycosyltransferase, putative
TGME49_260430	4.88	4.67	4.52E-28	9.54E-26	hypothetical protein
TGME49_206550	7.43	6.05	1.71E-27	3.42E-25	hypothetical protein
TGME49_251400	3.02	6.13	1.78E-27	3.46E-25	hypothetical protein
TGME49_275870	4.24	5.59	4.42E-27	8.38E-25	tubulin/FtsZ family, GTPase domain-containing protein
TGME49_266050	2.37	7.09	4.54E-27	8.39E-25	hypothetical protein
TGME49_211695	3.16	5.62	4.06E-26	7.31E-24	hypothetical protein
TGME49_301222	3.48	6.56	3.10E-25	5.47E-23	DNA repair protein Rad4 domain-containing protein
TGME49_293780	3.35	7.14	3.20E-25	5.50E-23	hypothetical protein
TGME49_280570	6.96	6.81	3.82E-25	6.42E-23	SAG-related sequence SRS35A
TGME49_278080	7.20	5.22	4.04E-25	6.63E-23	Toxoplasma gondii family A protein
TGME49_286460	5.01	5.00	8.77E-25	1.41E-22	hypothetical protein
TGME49_277260	1.81	9.53	2.43E-24	3.83E-22	hypothetical protein
TGME49_229010	-2.37	9.17	4.59E-24	7.07E-22	rhoptry neck protein RON4
TGME49_252430	2.15	8.12	2.73E-23	4.12E-21	hypothetical protein
TGME49_208740	1.85	9.88	8.37E-23	1.24E-20	microneme protein, putative
TGME49_314500	-3.32	8.36	1.18E-22	1.71E-20	subtilisin SUB2

TGME49_244280	-2.12	10.21	1.34E-22	1.90E-20	hypothetical protein
TGME49_243615	3.29	5.68	4.78E-22	6.67E-20	hypothetical protein
TGME49_253170	2.14	7.37	9.51E-22	1.30E-19	zinc carboxypeptidase, putative
TGME49_202020	4.47	6.95	1.11E-21	1.49E-19	DnAK-TPR
TGME49_291040	8.36	6.75	2.65E-21	3.40E-19	lactate dehydrogenase LDH2
TGME49_275640	2.21	6.94	2.67E-21	3.40E-19	hypothetical protein
TGME49_201170	-2.77	8.67	3.76E-21	4.72E-19	hypothetical protein
TGME49_290020	2.27	6.64	6.27E-21	7.48E-19	cyclin dependent kinase binding protein
TGME49_217820	-3.27	7.71	8.28E-21	9.72E-19	PCI domain-containing protein
TGME49_207210	6.79	6.73	1.08E-20	1.24E-18	hypothetical protein
TGME49_293170	-5.65	7.60	1.65E-20	1.87E-18	hypothetical protein
TGME49_208370	1.58	8.84	3.60E-20	4.03E-18	myosin heavy chain, putative
TGME49_319350	-1.78	9.36	6.67E-20	7.36E-18	SAG-related sequence SRS17B
TGME49_259550	-3.33	7.66	1.22E-19	1.31E-17	dihydropteroate synthase
TGME49_290270	2.17	6.58	1.33E-19	1.41E-17	SPRY domain-containing protein
TGME49_259260	-2.61	9.01	2.65E-19	2.76E-17	membrane protein FtsH1
TGME49_306670	-3.72	7.92	5.90E-19	6.06E-17	hypothetical protein
TGME49_269330	-10.60	7.07	7.00E-19	7.09E-17	hypothetical protein
TGME49_211030	-3.41	8.15	7.59E-19	7.58E-17	hypothetical protein
TGME49_234510	2.08	6.89	1.04E-18	1.02E-16	ankyrin repeat-containing protein
TGME49_277000	-4.77	7.32	1.76E-18	1.67E-16	transport protein Sec24, putative
TGME49_226100	6.20	5.74	1.79E-18	1.68E-16	haloacid dehalogenase family hydrolase domain-contain
TGME49_200320	-3.25	7.39	1.87E-18	1.73E-16	hypoxanthine-xanthine-guanine phosphoribosyl transfer
TGME49_293480	3.17	7.15	2.46E-18	2.24E-16	MoeA N-terminal region (domain I and II) domain-contai
TGME49_293570	-3.13	7.85	2.63E-18	2.37E-16	translocation protein sec62, putative
TGME49_223930	1.70	8.26	2.95E-18	2.63E-16	RNA recognition motif-containing protein
TGME49_230830	4.39	5.63	3.64E-18	3.20E-16	ATPase family associated with various cellular activities (
TGME49_310750	-2.52	8.35	4.48E-18	3.89E-16	emp24/gp25L/p24 family protein
TGME49_280580	4.14	5.84	4.70E-18	4.02E-16	SAG-related sequence SRS35B
TGME49_273320	1.83	7.86	4.73E-18	4.02E-16	hypothetical protein
TGME49_207880	-10.92	6.72	6.95E-18	5.84E-16	hypothetical protein
TGME49_207680	-4.57	7.02	7.69E-18	6.39E-16	suppressor of kinetochore protein 1, putative
TGME49_239790	-10.51	7.09	8.32E-18	6.83E-16	BRCA1 C Terminus (BRCT) domain-containing protein
TGME49_320190	8.02	6.30	8.59E-18	6.98E-16	SAG-related sequence SRS16B
TGME49_287460	3.08	4.88	1.34E-17	1.08E-15	hypothetical protein
TGME49_254080	1.59	7.96	1.69E-17	1.34E-15	metal cation transporter, ZIP family protein
TGME49_278510	-2.92	7.68	1.71E-17	1.34E-15	protein phosphatase 2C domain-containing protein
TGME49_315620	-5.42	7.07	1.79E-17	1.39E-15	vacuolar ATP synthase subunit C, putative
TGME49_255650	-10.91	7.05	2.03E-17	1.56E-15	DHHC zinc finger domain-containing protein
TGME49_254730	2.50	6.04	2.34E-17	1.79E-15	POPLD (NUC188) domain-containing protein
TGME49_224760	3.19	4.85	2.44E-17	1.84E-15	SAG-related sequence SRS40E
TGME49_305160	2.96	4.99	3.34E-17	2.50E-15	histone H2Ba
TGME49_236910	-4.60	7.47	3.76E-17	2.78E-15	U2 snRNP auxiliary factor, putative
TGME49_270510	-1.78	9.75	4.00E-17	2.93E-15	asparaginyl-tRNA synthetase (NOB+tRNA synthase)
TGME49_213570	1.39	8.76	4.17E-17	3.00E-15	hypothetical protein
TGME49_260500	-2.19	9.04	4.15E-17	3.00E-15	COPI associated protein, putative
TGME49_238950	-2.83	9.10	4.96E-17	3.53E-15	fatty acyl-CoA desaturase, putative
TGME49_240650	-2.44	8.44	5.83E-17	4.11E-15	coatomer protein complex, subunit alpha, putative
TGME49_236670	3.04	6.27	6.14E-17	4.24E-15	hypothetical protein
TGME49_319308	-4.12	6.63	6.20E-17	4.24E-15	hypothetical protein
TGME49_229690	-10.43	6.55	6.19E-17	4.24E-15	autophagy-related protein 7 atg7, putative
TGME49_214410	3.26	7.77	6.74E-17	4.57E-15	hypothetical protein
TGME49_214575	2.55	5.96	8.67E-17	5.83E-15	hypothetical protein
TGME49_245432	2.68	7.36	1.07E-16	7.11E-15	hypothetical protein
TGME49_228690	-5.44	7.16	1.08E-16	7.11E-15	phosphatidylinositol 3- and 4-kinase
TGME49_271935	1.85	6.69	1.17E-16	7.67E-15	hypothetical protein
TGME49_251740	-3.65	7.43	1.41E-16	9.11E-15	AP2 domain transcription factor AP2XII-9

TGME49_222380	-2.57	8.13	1.48E-16	9.49E-15	importin-beta N-terminal domain-containing protein
TGME49_249350	-10.39	6.75	1.79E-16	1.14E-14	esterase/lipase/thioesterase domain-containing protein
TGME49_293280	2.97	5.89	1.90E-16	1.20E-14	cyclin protein
TGME49_289170	4.85	5.24	1.93E-16	1.21E-14	adenylate and guanylate cyclase catalytic domain-contai
TGME49_204520	3.39	4.97	2.42E-16	1.51E-14	hypothetical protein
TGME49_250030	2.94	5.24	2.45E-16	1.51E-14	hypothetical protein
TGME49_215390	3.43	4.82	2.85E-16	1.74E-14	TIM10 family protein, putative
TGME49_319500	-10.47	7.06	3.76E-16	2.28E-14	hypothetical protein
TGME49_275860	2.23	9.46	4.97E-16	2.97E-14	hypothetical protein
TGME49_207460	1.67	7.42	4.98E-16	2.97E-14	Rab5B protein
TGME49_232650	2.06	6.75	5.37E-16	3.18E-14	hypothetical protein
TGME49_220250	1.84	6.94	5.73E-16	3.36E-14	Nucleotide-sensitive chloride conductance regulator (ICl
TGME49_239440	-10.36	6.88	6.76E-16	3.94E-14	protein kinase (incomplete catalytic triad)
TGME49_241830	2.35	5.87	6.82E-16	3.94E-14	peptidyl-prolyl cis-trans isomerase
TGME49_205200	1.97	6.83	7.16E-16	4.10E-14	hypothetical protein
TGME49_270360	-5.33	7.03	7.70E-16	4.38E-14	hypothetical protein
TGME49_249540	1.71	7.36	9.75E-16	5.50E-14	hypothetical protein
TGME49_254120	1.94	8.30	1.20E-15	6.74E-14	autophagy-related protein 8 atg8, putative
TGME49_216335	3.61	5.08	1.36E-15	7.53E-14	hypothetical protein
TGME49_242290	-2.68	7.99	1.42E-15	7.76E-14	proteasome subunit alpha1, putative
TGME49_246490	-2.68	7.46	1.45E-15	7.87E-14	hypothetical protein
TGME49_239410	-2.59	7.90	1.48E-15	8.01E-14	hypothetical protein
TGME49_253860	1.66	7.25	1.92E-15	1.03E-13	Tyrosine kinase-like (TKL) protein
TGME49_318610	3.56	5.24	1.96E-15	1.04E-13	AP2 domain transcription factor AP2IV-3
TGME49_306660	1.73	7.88	2.28E-15	1.20E-13	RNA pseudouridine synthase superfamily protein
TGME49_243580	-4.10	7.20	2.45E-15	1.28E-13	Hit family protein involved in cell-cycle regulation, putat
TGME49_200350	3.55	5.77	2.51E-15	1.31E-13	subtilisin SUB3
TGME49_212880	-2.83	7.91	2.65E-15	1.37E-13	surface antigen repeat-containing protein
TGME49_205380	-2.23	7.80	3.26E-15	1.67E-13	fructose-bisphospatase I
TGME49_208530	-3.36	7.28	3.68E-15	1.87E-13	nicotinate phosphoribosyltransferase
TGME49_265190	-4.37	7.20	3.93E-15	1.99E-13	Ulp1 protease family, C-terminal catalytic domain-contai
TGME49_209050	-10.25	6.78	4.09E-15	2.06E-13	Tyrosine kinase-like (TKL) protein
TGME49_219520	-2.64	7.68	4.42E-15	2.19E-13	histone arginine methyltransferase PRMT1
TGME49_235130	-3.97	7.38	4.42E-15	2.19E-13	transmembrane protein
TGME49_212250	2.42	7.68	4.76E-15	2.34E-13	XPG N-terminal domain-containing protein
TGME49_228280	2.53	5.71	4.96E-15	2.43E-13	hypothetical protein
TGME49_240880	3.30	5.88	5.02E-15	2.44E-13	hypothetical protein
TGME49_311230	1.23	9.99	5.06E-15	2.44E-13	hypothetical protein
TGME49_212900	1.72	8.11	6.29E-15	3.02E-13	hypothetical protein
TGME49_254710	1.52	7.81	6.35E-15	3.03E-13	serine esterase (DUF676) protein
TGME49_203300	2.96	7.42	6.72E-15	3.18E-13	hypothetical protein
TGME49_293410	2.64	5.32	6.99E-15	3.29E-13	hypothetical protein
TGME49_218520	1.22	10.33	9.07E-15	4.22E-13	microneme protein MIC6
TGME49_263870	-5.22	6.97	9.81E-15	4.53E-13	glutamate-tRNA ligase
TGME49_253690	1.24	8.98	1.04E-14	4.71E-13	hypothetical protein
TGME49_261520	-5.14	7.04	1.03E-14	4.71E-13	nucleolar GTP-binding protein 1, putative
TGME49_287170	-10.29	7.07	1.04E-14	4.71E-13	hypothetical protein
TGME49_250820	-2.94	7.50	1.25E-14	5.64E-13	hypothetical protein
TGME49_259710	-3.61	7.07	1.34E-14	6.02E-13	protein kinase
TGME49_316280	-5.19	6.90	1.37E-14	6.11E-13	transporter, major facilitator family protein
TGME49_270700	1.62	7.08	1.40E-14	6.19E-13	hypothetical protein
TGME49_290200	-2.00	8.88	1.50E-14	6.55E-13	NAD/NADP octopine/nopaline dehydrogenase, alpha-he
TGME49_258150	-2.22	7.78	1.49E-14	6.55E-13	proteasome subunit alpha type 7, putative
TGME49_278830	-1.79	8.52	1.66E-14	7.21E-13	glucose-6-phosphate 1-dehydrogenase
TGME49_216140	2.32	7.52	1.67E-14	7.21E-13	tetratricopeptide repeat-containing protein
TGME49_226960	-1.58	9.46	1.72E-14	7.41E-13	phosphofructokinase PFKII
TGME49_223480	-10.14	5.77	1.95E-14	8.33E-13	sushi domain (scr repeat) domain-containing protein

TGME49_255210	3.10	6.02	2.00E-14	8.50E-13	ATPase, AAA family protein
TGME49_254050	1.82	6.83	2.29E-14	9.66E-13	optic atrophy 3 protein (opa3) protein
TGME49_306060	-1.59	9.70	2.37E-14	9.91E-13	rhoptry neck protein RON8
TGME49_300220	-3.26	7.60	2.36E-14	9.91E-13	hypothetical protein
TGME49_278870	-1.81	8.87	2.40E-14	9.99E-13	myosin F
TGME49_235630	1.72	7.24	2.90E-14	1.19E-12	hypothetical protein
TGME49_294770	-5.13	6.84	2.97E-14	1.22E-12	Armadillo/beta-catenin family repeat-containing protein
TGME49_236990	4.01	5.51	3.16E-14	1.28E-12	beta-ketoacyl synthase, N-terminal domain-containing p
TGME49_250340	-2.60	7.79	3.18E-14	1.29E-12	centrin 2
TGME49_316660	-10.13	6.50	3.37E-14	1.36E-12	cullin family protein
TGME49_218910	3.84	5.24	3.78E-14	1.51E-12	hypothetical protein
TGME49_205010	-2.55	7.49	4.43E-14	1.76E-12	U2 small nuclear ribonucleoprotein family protein, putat
TGME49_259010	-5.10	6.71	4.64E-14	1.83E-12	vacuolar ATP synthase subunit d, putative
TGME49_214980	1.39	8.41	4.70E-14	1.85E-12	hypothetical protein
TGME49_294630	-2.37	7.75	4.78E-14	1.87E-12	hypothetical protein
TGME49_245510	-2.73	7.52	5.06E-14	1.97E-12	phospholipid-translocating P-type ATPase, flippase subfa
TGME49_268176	-3.25	7.14	5.09E-14	1.97E-12	hypothetical protein
TGME49_305590	-4.21	6.99	5.63E-14	2.16E-12	ABC transporter transmembrane region domain-contain
TGME49_246330	-5.17	7.00	5.63E-14	2.16E-12	CRAL/TRIO domain-containing protein
TGME49_202420	1.97	6.26	5.77E-14	2.20E-12	hypothetical protein
TGME49_291680	-2.05	7.77	6.62E-14	2.51E-12	Sec23/Sec24 trunk domain-containing protein
TGME49_313020	-4.21	6.61	6.95E-14	2.62E-12	STAS domain-containing protein
TGME49_270670	3.19	5.21	7.01E-14	2.63E-12	hypothetical protein
TGME49_315320	-1.69	9.24	8.33E-14	3.11E-12	SAG-related sequence SRS52A
TGME49_293190	-3.24	7.38	8.74E-14	3.25E-12	endonuclease/exonuclease/phosphatase family protein
TGME49_304740	1.38	8.67	9.35E-14	3.46E-12	rhoptry kinase family protein ROP35
TGME49_231150	2.83	5.08	1.02E-13	3.76E-12	hypothetical protein
TGME49_275755	3.34	4.98	1.08E-13	3.95E-12	hypothetical protein
TGME49_254620	1.17	9.19	1.14E-13	4.15E-12	ribosomal protein RPL39
TGME49_221470	-2.15	8.22	1.17E-13	4.24E-12	hypothetical protein
TGME49_213620	3.04	5.49	1.52E-13	5.47E-12	ABC1 family protein
TGME49_265870	-3.80	6.97	2.07E-13	7.40E-12	pantoate-beta-alanine ligase
TGME49_318750	2.56	5.79	2.09E-13	7.42E-12	deoxyribose-phosphate aldolase
TGME49_233450	1.37	8.00	2.39E-13	8.47E-12	SAG-related sequence SRS29A
TGME49_210300	-5.07	6.98	2.59E-13	9.11E-12	hypothetical protein
TGME49_218530	-10.01	6.40	2.71E-13	9.50E-12	proteasome-interacting thioredoxin domain-containing p
TGME49_272370	1.71	7.74	2.76E-13	9.62E-12	hypothetical protein
TGME49_253750	2.28	8.98	2.85E-13	9.86E-12	PLU-1 family protein
TGME49_231410	-2.50	8.15	2.85E-13	9.86E-12	hypothetical protein
TGME49_223050	-1.58	9.44	3.12E-13	1.07E-11	ribosomal protein RPS20
TGME49_306620	3.84	6.62	3.70E-13	1.25E-11	AP2 domain transcription factor AP2IX-9
TGME49_282220	1.93	6.58	3.70E-13	1.25E-11	AP2 domain transcription factor AP2VIIa-9
TGME49_237230	1.26	9.81	3.68E-13	1.25E-11	hypothetical protein
TGME49_245980	3.58	7.53	3.72E-13	1.26E-11	hypothetical protein
TGME49_294550	-2.09	8.00	4.67E-13	1.56E-11	dynein heavy chain
TGME49_247460	-1.81	8.51	7.16E-13	2.39E-11	proliferating cell nuclear antigen PCNA1
TGME49_292920	-2.22	7.96	7.23E-13	2.40E-11	heat shock protein 75, putative
TGME49_286120	-2.35	8.02	7.79E-13	2.57E-11	prolyl endopeptidase
TGME49_242118	-9.98	5.88	8.02E-13	2.64E-11	myosin-light-chain kinase
TGME49_252880	-2.78	7.29	8.38E-13	2.74E-11	hypothetical protein
TGME49_246580	-4.08	6.71	9.38E-13	3.06E-11	hypothetical protein
TGME49_240280	-2.45	7.78	1.02E-12	3.31E-11	S1/P1nuclease
TGME49_238100	-2.61	7.30	1.07E-12	3.42E-11	transmembrane protein
TGME49_244910	-4.11	6.82	1.07E-12	3.42E-11	MIZ/SP-RING zinc finger domain-containing protein
TGME49_311720	-1.30	11.50	1.09E-12	3.48E-11	chaperonin protein BiP
TGME49_257700	3.22	5.46	1.22E-12	3.86E-11	hypothetical protein
TGME49_305180	-9.93	6.43	1.28E-12	4.04E-11	Na+/H+ exchanger NHE3

TGME49_254870	1.70	6.87	1.39E-12	4.38E-11	hypothetical protein
TGME49_214350	-2.94	7.39	1.40E-12	4.38E-11	GTP-binding protein, putative
TGME49_244880	-4.08	6.91	1.47E-12	4.58E-11	DNA-directed RNA polymerase I RPA1
TGME49_253790	1.73	7.54	1.51E-12	4.68E-11	zinc finger (CCCH type) motif-containing protein
TGME49_247760	-3.35	6.83	1.52E-12	4.68E-11	AMP-binding enzyme domain-containing protein
TGME49_232500	-9.93	6.44	1.52E-12	4.68E-11	hypothetical protein
TGME49_250670	2.24	6.89	1.70E-12	5.19E-11	hypothetical protein
TGME49_221675	1.46	7.82	2.00E-12	6.07E-11	hypothetical protein
TGME49_214220	1.37	9.74	2.12E-12	6.41E-11	hypothetical protein
TGME49_253570	2.19	6.14	2.26E-12	6.82E-11	hypothetical protein
TGME49_257945	4.42	4.77	2.33E-12	7.01E-11	3'5'-cyclic nucleotide phosphodiesterase domain-contai
TGME49_282070	-1.87	8.54	2.41E-12	7.22E-11	hypothetical protein
TGME49_253090	2.21	5.86	2.46E-12	7.34E-11	DEAD/DEAH box helicase domain-containing protein
TGME49_263550	1.76	6.57	2.56E-12	7.60E-11	39S ribosomal protein L47, mitochondrial precursor, put
TGME49_219485	2.11	6.92	2.61E-12	7.73E-11	hypothetical protein
TGME49_240870	-2.88	7.10	2.67E-12	7.88E-11	beta adaptin protein, putative
TGME49_220930	-2.83	7.43	2.69E-12	7.90E-11	hypothetical protein
TGME49_320180	5.44	5.15	2.94E-12	8.60E-11	SAG-related sequence SRS16C
TGME49_229020	-9.88	6.41	3.34E-12	9.73E-11	cell-cycle-associated protein kinase CDK, putative
TGME49_214970	-1.90	8.91	3.41E-12	9.87E-11	DNA replication licensing factor, putative
TGME49_313660	3.66	4.89	3.47E-12	1.00E-10	hypothetical protein
TGME49_245460	1.16	10.31	3.50E-12	1.01E-10	ribosomal protein RPS8
TGME49_218880	-2.54	7.48	3.62E-12	1.04E-10	SF-assemblin, putative
TGME49_310460	-3.13	7.01	3.80E-12	1.09E-10	Rab6
TGME49_213060	1.91	6.59	4.02E-12	1.14E-10	WD domain, G-beta repeat-containing protein
TGME49_239540	-9.89	6.42	4.75E-12	1.34E-10	LEM3 (ligand-effect modulator 3) family / CDC50 family
TGME49_213280	1.16	8.91	4.85E-12	1.36E-10	SAG-related sequence SRS25
TGME49_274190	-4.96	6.59	4.87E-12	1.36E-10	eukaryotic initiation factor 2B epsilon subunit, putative
TGME49_315780	-3.31	6.99	4.97E-12	1.38E-10	myosin regulatory light chain, putative
TGME49_212860	1.90	6.72	5.17E-12	1.43E-10	hypothetical protein
TGME49_220950	1.57	9.89	5.31E-12	1.47E-10	hypothetical protein
TGME49_312622	-2.72	7.05	5.65E-12	1.55E-10	DUF803 domain-containing protein
TGME49_295990	1.59	7.35	5.95E-12	1.63E-10	ubiquitin conjugating enzyme E2, putative
TGME49_277940	-4.03	6.79	6.06E-12	1.65E-10	hypothetical protein
TGME49_260180	-2.13	7.68	6.49E-12	1.76E-10	hypothetical protein
TGME49_306440	-9.84	6.04	6.83E-12	1.85E-10	hypothetical protein
TGME49_231430	-9.84	6.39	7.22E-12	1.95E-10	oligosaccharyl transferase stt3 protein, putative
TGME49_310130	-9.82	6.05	7.42E-12	2.00E-10	Spc97 / Spc98 family protein
TGME49_217700	-9.87	6.41	7.59E-12	2.03E-10	AP2 domain transcription factor AP2XII-2
TGME49_266990	-1.94	7.81	7.97E-12	2.13E-10	beta-COP
TGME49_219700	-1.94	8.41	8.34E-12	2.21E-10	DNA replication licensing factor MCM4, putative
TGME49_226705	-10.02	6.39	9.21E-12	2.43E-10	hypothetical protein
TGME49_211150	-4.82	6.82	9.63E-12	2.53E-10	hypothetical protein
TGME49_301270	1.33	8.03	9.96E-12	2.61E-10	Tyrosine kinase-like (TKL) protein
TGME49_228660	-3.53	6.95	1.01E-11	2.63E-10	Sec7 domain-containing protein
TGME49_296010	-1.93	7.91	1.08E-11	2.81E-10	phosphatidylinositol 3- and 4-kinase
TGME49_237210	-4.79	6.72	1.10E-11	2.84E-10	Tyrosine kinase-like (TKL) protein
TGME49_270270	-4.08	6.69	1.11E-11	2.88E-10	hypothetical protein
TGME49_220880	1.87	6.18	1.15E-11	2.96E-10	hypothetical protein
TGME49_214140	1.35	8.62	1.15E-11	2.96E-10	hypothetical protein
TGME49_314410	-2.67	7.22	1.18E-11	3.02E-10	aquarius, putative
TGME49_320130	2.02	5.97	1.25E-11	3.19E-10	hypothetical protein
TGME49_254220	2.22	5.45	1.35E-11	3.43E-10	hypothetical protein
TGME49_274060	-2.10	7.69	1.42E-11	3.60E-10	2-oxoglutarate/malate translocase OMT
TGME49_321650	-2.55	7.47	1.45E-11	3.66E-10	hypothetical protein
TGME49_218560	-2.00	7.74	1.50E-11	3.76E-10	acetyl-coA carboxylase ACC2
TGME49_292160	1.97	6.10	1.53E-11	3.82E-10	hypothetical protein

TGME49_255410	-4.79	6.62	1.63E-11	4.06E-10	hypothetical protein
TGME49_263490	2.10	5.71	1.72E-11	4.27E-10	ubiquitin conjugating enzyme E2, putative
TGME49_268230	-10.16	6.51	1.72E-11	4.27E-10	hypothetical protein
TGME49_207640	-2.13	7.49	1.73E-11	4.28E-10	isoleucyl-tRNA synthetase family protein
TGME49_290700	1.76	7.93	1.80E-11	4.43E-10	hypothetical protein
TGME49_289710	-2.53	7.42	1.95E-11	4.80E-10	AP2 domain transcription factor AP2IX-5
TGME49_279100	1.23	9.39	2.04E-11	4.97E-10	hypothetical protein
TGME49_205680	2.15	8.42	2.24E-11	5.45E-10	hypothetical protein
TGME49_298620	-4.77	6.83	2.35E-11	5.70E-10	hypothetical protein
TGME49_203600	-1.53	9.21	2.38E-11	5.75E-10	hypothetical protein
TGME49_294640	-1.55	8.84	2.46E-11	5.92E-10	ribonucleoside-diphosphate reductase large chain
TGME49_257490	-3.98	6.62	2.51E-11	6.00E-10	prefoldin subunit superfamily protein
TGME49_305090	2.15	6.45	2.64E-11	6.29E-10	kinase binding protein cgi-121 protein
TGME49_257530	-2.22	11.60	2.85E-11	6.73E-10	transporter, major facilitator family protein
TGME49_292130	-1.31	9.66	2.89E-11	6.80E-10	ribosomal protein RPL13A
TGME49_288820	1.67	6.83	2.93E-11	6.88E-10	hypothetical protein
TGME49_245560	1.35	7.95	3.13E-11	7.31E-10	hypothetical protein
TGME49_230410	-2.03	8.00	3.13E-11	7.31E-10	peroxiredoxin PRX3
TGME49_226310	-3.47	7.74	3.38E-11	7.83E-10	zinc finger (CCCH type) motif-containing protein
TGME49_318310	-2.07	7.63	3.61E-11	8.33E-10	transketolase
TGME49_318660	1.29	8.55	3.80E-11	8.75E-10	PP2C, putative
TGME49_273050	1.84	6.54	3.90E-11	8.96E-10	hypothetical protein
TGME49_320588	-9.75	6.31	4.05E-11	9.27E-10	glycosyl hydrolases family 35 protein
TGME49_294990	-9.75	6.22	4.42E-11	1.01E-09	hypothetical protein
TGME49_306330	-2.75	6.75	4.47E-11	1.02E-09	phospholipase
TGME49_257350	1.50	8.60	4.52E-11	1.03E-09	eukaryotic translation initiation factor, putative
TGME49_232280	-9.89	6.42	4.64E-11	1.05E-09	hypothetical protein
TGME49_207690	-4.73	6.36	4.69E-11	1.06E-09	programmed cell death 5 protein
TGME49_217951	8.04	5.22	4.79E-11	1.07E-09	hypothetical protein
TGME49_228190	-1.90	7.95	4.79E-11	1.07E-09	eukaryotic initiation factor-3, subunit 5, putative
TGME49_250710	1.13	11.27	4.86E-11	1.09E-09	microneme protein MIC10
TGME49_308810	-1.91	8.08	4.99E-11	1.11E-09	rhoptry neck protein RON9
TGME49_249610	-2.92	6.80	5.31E-11	1.18E-09	hypothetical protein
TGME49_290170	-2.51	7.34	5.48E-11	1.21E-09	kelch repeat domain containing/Serine/threonine protei
TGME49_272410	1.63	6.75	6.64E-11	1.47E-09	phosphogluconate dehydrogenase (decarboxylating), NA
TGME49_215590	-2.03	8.31	7.01E-11	1.54E-09	flavoprotein subunit of succinate dehydrogenase
TGME49_297880	-2.17	10.28	7.27E-11	1.60E-09	dense granule protein DG32
TGME49_297530	-9.68	6.26	7.67E-11	1.68E-09	DNA-directed RNA polymerase I RPA2
TGME49_251800	2.19	5.67	7.70E-11	1.68E-09	hypothetical protein
TGME49_313445	2.08	5.72	7.85E-11	1.71E-09	hypothetical protein
TGME49_286470	1.18	8.36	7.89E-11	1.71E-09	AGC kinase
TGME49_239800	-3.12	6.90	8.56E-11	1.85E-09	hypothetical protein
TGME49_249230	-4.83	6.14	9.03E-11	1.95E-09	hypothetical protein
TGME49_264830	-4.67	6.49	9.12E-11	1.96E-09	hypothetical protein
TGME49_221830	3.34	5.06	9.30E-11	1.99E-09	subtilisin SUB12
TGME49_291940	-4.65	6.39	9.84E-11	2.10E-09	hypothetical protein
TGME49_226810	-3.47	6.80	1.02E-10	2.17E-09	histone lysine methyltransferase SET1
TGME49_321690	-3.95	6.68	1.05E-10	2.23E-09	hypothetical protein
TGME49_305860	-2.36	7.93	1.08E-10	2.28E-09	calcium-dependent protein kinase CDPK3
TGME49_217890	-1.40	8.67	1.11E-10	2.34E-09	alkyl hydroperoxide reductase/ Thiol specific antioxidant
TGME49_294400	-2.42	7.26	1.12E-10	2.36E-09	hypothetical protein
TGME49_237015	1.50	7.91	1.24E-10	2.59E-09	hypothetical protein
TGME49_232270	-9.76	5.79	1.29E-10	2.69E-09	histidine acid phosphatase superfamily protein
TGME49_205360	-2.57	7.08	1.44E-10	3.00E-09	hypothetical protein
TGME49_225250	1.93	5.93	1.48E-10	3.08E-09	LSU ribosomal protein L14P, putative
TGME49_226220	-2.89	6.89	1.52E-10	3.14E-09	alveolin domain containing intermediate filament IMC9
TGME49_226500	-4.82	6.63	1.58E-10	3.26E-09	hypothetical protein

TGME49_223760	-9.63	6.03	1.61E-10	3.32E-09	hypothetical protein
TGME49_206695	2.63	5.29	1.72E-10	3.53E-09	hypothetical protein
TGME49_216970	-1.81	7.94	1.73E-10	3.54E-09	coronin, putative
TGME49_209060	-3.82	6.66	1.76E-10	3.60E-09	thrombospondin type 1 domain-containing protein
TGME49_294690	1.64	7.53	1.77E-10	3.60E-09	rhomboid protease ROM5
TGME49_261970	1.71	7.59	1.89E-10	3.83E-09	hypothetical protein
TGME49_309110	1.83	6.61	2.03E-10	4.10E-09	tRNA methyl transferase
TGME49_268225	-5.03	6.81	2.09E-10	4.19E-09	hypothetical protein
TGME49_313230	2.07	9.82	2.19E-10	4.38E-09	eukaryotic initiation factor-2, alpha subunit
TGME49_216730	-1.60	8.59	2.22E-10	4.43E-09	MCM2/3/5 family protein
TGME49_292140	1.46	7.59	2.27E-10	4.51E-09	NIMA-related protein kinase NIMA1
TGME49_319910	1.90	5.94	2.39E-10	4.73E-09	WD domain, G-beta repeat-containing protein
TGME49_217520	1.16	8.89	2.57E-10	5.06E-09	hypothetical protein
TGME49_216020	-9.61	6.06	2.57E-10	5.06E-09	peptidase family c78 protein
TGME49_293660	-3.81	6.63	2.59E-10	5.07E-09	hypothetical protein
TGME49_301180	3.30	5.49	2.92E-10	5.71E-09	SAG-related sequence SRS19F
TGME49_318690	2.72	5.00	2.96E-10	5.77E-09	RNA recognition motif-containing protein
TGME49_262380	-3.11	7.00	3.07E-10	5.97E-09	elongation factor Tu, putative
TGME49_315530	-4.61	6.48	3.46E-10	6.71E-09	hypothetical protein
TGME49_227910	-1.72	7.80	3.54E-10	6.86E-09	hypothetical protein
TGME49_204310	-1.56	8.50	3.62E-10	6.99E-09	hypothetical protein
TGME49_261620	1.94	5.83	3.84E-10	7.40E-09	hypothetical protein
TGME49_222940	4.65	5.07	3.92E-10	7.53E-09	hypothetical protein
TGME49_260800	1.69	6.32	3.94E-10	7.55E-09	hypothetical protein
TGME49_249650	-9.59	5.91	4.02E-10	7.68E-09	apolipoprotein A-I binding protein, putative
TGME49_254520	1.24	7.43	4.06E-10	7.74E-09	mediator complex subunit MED11
TGME49_246930	-2.33	7.26	4.24E-10	8.06E-09	calmodulin CAM1
TGME49_211250	-3.09	6.81	4.35E-10	8.25E-09	hypothetical protein
TGME49_264600	-2.67	6.95	4.38E-10	8.29E-09	hypothetical protein
TGME49_263540	2.39	5.09	4.50E-10	8.49E-09	hypothetical protein
TGME49_250330	-9.57	6.25	4.59E-10	8.64E-09	hypothetical protein
TGME49_236570	-1.58	7.93	5.20E-10	9.74E-09	lysine decarboxylase family protein
TGME49_205580	1.37	7.01	5.31E-10	9.92E-09	nuclear factor NF4
TGME49_229420	-9.54	6.04	5.34E-10	9.95E-09	cytochrome c, putative
TGME49_271460	2.34	6.76	5.54E-10	1.03E-08	protein c14orf29, putative
TGME49_251540	1.30	9.03	5.82E-10	1.07E-08	dense granule protein GRA9
TGME49_299250	2.79	6.36	5.93E-10	1.09E-08	hypothetical protein
TGME49_277720	-2.27	7.54	5.98E-10	1.10E-08	GDA1/CD39 (nucleoside phosphatase) family protein
TGME49_249990	1.69	10.24	6.22E-10	1.14E-08	hypothetical protein
TGME49_290970	2.51	7.23	6.37E-10	1.16E-08	8-amino-7-oxononanoate synthase
TGME49_260670	-9.55	5.92	6.50E-10	1.18E-08	centrin, putative
TGME49_289750	-1.30	9.50	7.01E-10	1.27E-08	ribosomal-ubiquitin protein RPL40
TGME49_280400	-9.61	7.58	7.55E-10	1.37E-08	hypothetical protein
TGME49_209730	-9.53	5.93	7.64E-10	1.38E-08	hypothetical protein
TGME49_294610	-1.38	9.34	8.05E-10	1.45E-08	histone lysine methyltransferase, SET, putative
TGME49_237560	-1.20	9.13	8.33E-10	1.50E-08	iron-sulfur cluster protein ISCU
TGME49_272730	3.75	4.78	8.62E-10	1.54E-08	hypothetical protein
TGME49_202310	1.71	6.20	8.85E-10	1.58E-08	O-sialoglycoprotein endopeptidase
TGME49_202920	-9.53	6.07	9.01E-10	1.60E-08	p-aminobenzoic acid synthase
TGME49_313380	-1.25	9.56	9.12E-10	1.62E-08	hypothetical protein
TGME49_251500	-1.61	8.09	9.42E-10	1.67E-08	eukaryotic initiation factor-3, subunit 3, putative
TGME49_270620	2.90	6.19	9.48E-10	1.67E-08	DEAD/DEAH box helicase domain-containing protein
TGME49_313880	-9.55	5.91	9.56E-10	1.68E-08	nuclear protein-like family protein
TGME49_278050	-2.22	7.70	1.04E-09	1.82E-08	proteasome subunit alpha type 1, putative
TGME49_238895	-2.84	7.20	1.04E-09	1.82E-08	hypothetical protein
TGME49_260440	1.21	9.39	1.07E-09	1.87E-08	nuclear factor NF3
TGME49_211040	-1.61	8.12	1.12E-09	1.95E-08	Sec61beta family protein

TGME49_254000	1.37	7.06	1.12E-09	1.95E-08	hypothetical protein
TGME49_255700	-2.42	7.07	1.13E-09	1.96E-08	hypothetical protein
TGME49_257050	-9.48	5.79	1.16E-09	2.01E-08	3-methyl-2-oxobutanoate hydroxymethyltransferase
TGME49_320640	-9.58	6.10	1.17E-09	2.03E-08	peptidylprolyl isomerase domain-containing protein
TGME49_288400	-9.50	6.03	1.19E-09	2.06E-08	LETM1 family protein
TGME49_253850	2.47	5.06	1.21E-09	2.08E-08	hypothetical protein
TGME49_236250	2.02	5.67	1.22E-09	2.09E-08	regulator of chromosome condensation (RCC1) repeat-c
TGME49_239590	-4.54	6.37	1.25E-09	2.13E-08	WD domain, G-beta repeat-containing protein
TGME49_250840	-9.48	6.18	1.26E-09	2.15E-08	hypothetical protein
TGME49_261480	1.76	6.87	1.33E-09	2.26E-08	phosphatidyl serine synthase
TGME49_290720	-1.87	7.52	1.39E-09	2.35E-08	vacuolar proton translocating ATPase subunit, putative
TGME49_306390	-1.62	7.63	1.50E-09	2.53E-08	hypothetical protein
TGME49_286790	-4.49	6.16	1.53E-09	2.57E-08	nuclear factor NF2
TGME49_270930	-3.08	6.90	1.57E-09	2.63E-08	hypothetical protein
TGME49_201800	-1.91	7.11	1.57E-09	2.63E-08	hypothetical protein
TGME49_309870	-9.48	5.78	1.58E-09	2.63E-08	hypothetical protein
TGME49_308060	-3.13	6.56	1.60E-09	2.67E-08	hypothetical protein
TGME49_249530	-1.77	7.70	1.63E-09	2.71E-08	exportin 1, putative
TGME49_285140	-9.49	6.26	1.65E-09	2.73E-08	hypothetical protein
TGME49_255940	2.44	5.25	1.66E-09	2.75E-08	hypothetical protein
TGME49_262930	-9.47	5.80	1.68E-09	2.78E-08	hypothetical protein
TGME49_255180	-9.46	6.36	1.72E-09	2.83E-08	ubiquitin carboxyl-terminal hydrolase
TGME49_243250	-1.70	7.91	1.89E-09	3.10E-08	myosin H
TGME49_213410	-1.97	7.73	1.89E-09	3.10E-08	small nuclear ribonucleoprotein f (snrnp-f), putative
TGME49_231980	-9.49	5.84	2.00E-09	3.27E-08	hypothetical protein
TGME49_320490	2.04	8.78	2.03E-09	3.30E-08	N-acyl-phosphatidylethanolamine-hydrolyzing phospholi
TGME49_309220	-1.98	7.36	2.15E-09	3.49E-08	GTPase activating protein for Arf protein
TGME49_307830	-1.52	8.45	2.22E-09	3.59E-08	hypothetical protein
TGME49_213050	2.16	8.44	2.26E-09	3.65E-08	hypothetical protein
TGME49_256840	-4.47	5.96	2.27E-09	3.67E-08	hypothetical protein
TGME49_262150	-9.42	5.98	2.33E-09	3.73E-08	kelch repeat and K+ channel tetramerisation domain cor
TGME49_224880	-3.34	7.04	2.38E-09	3.81E-08	kinesin motor domain-containing protein
TGME49_270770	-4.53	6.36	2.40E-09	3.84E-08	PWI domain-containing protein
TGME49_232640	2.93	5.04	2.43E-09	3.87E-08	RNA 2'-phosphotransferase, Tpt1/KptA family protein
TGME49_224960	1.40	7.07	2.44E-09	3.88E-08	hypothetical protein
TGME49_289300	-2.10	7.33	2.49E-09	3.95E-08	methionyl-tRNA synthetase
TGME49_237830	-9.48	5.84	2.50E-09	3.95E-08	DNA polymerase I domain-containing protein
TGME49_285260	2.87	4.95	2.96E-09	4.65E-08	hypothetical protein
TGME49_262040	-2.75	6.75	2.96E-09	4.65E-08	SAC3/GANP family protein
TGME49_310220	-4.55	6.48	3.01E-09	4.72E-08	hypothetical protein
TGME49_278815	-3.71	6.59	3.06E-09	4.79E-08	hypothetical protein
TGME49_319370	-9.43	6.12	3.11E-09	4.85E-08	hypothetical protein
TGME49_219270	-1.21	10.13	3.12E-09	4.86E-08	multi-pass transmembrane protein
TGME49_249590	-1.85	7.72	3.16E-09	4.91E-08	proteasome subunit alpha type 5-2, putative
TGME49_247360	2.19	7.48	3.25E-09	5.03E-08	PAP2 superfamily protein
TGME49_309380	-3.21	6.60	3.28E-09	5.07E-08	Nuf2
TGME49_300130	3.11	4.71	3.30E-09	5.09E-08	apical membrane antigen 1 domain-containing protein
TGME49_305240	-3.24	6.60	3.33E-09	5.12E-08	XPA binding protein 2 family protein
TGME49_312960	1.67	6.29	3.40E-09	5.22E-08	hypothetical protein
TGME49_209755	6.35	6.70	3.62E-09	5.55E-08	hypothetical protein
TGME49_213325	-9.45	5.93	3.64E-09	5.56E-08	TBC domain-containing protein
TGME49_266380	-9.53	5.23	3.72E-09	5.67E-08	hypothetical protein
TGME49_225290	3.12	6.40	3.88E-09	5.90E-08	GDA1/CD39 (nucleoside phosphatase) family protein
TGME49_237550	2.49	6.56	3.89E-09	5.90E-08	hypothetical protein
TGME49_310030	-1.89	7.62	3.95E-09	5.99E-08	cyclase-associated protein, putative
TGME49_219860	-1.53	8.73	3.98E-09	6.01E-08	replication licensing factor, putative
TGME49_240860	-3.67	6.54	3.99E-09	6.01E-08	acyltransferase domain-containing protein

TGME49_234980	1.94	6.57	4.00E-09	6.03E-08	hypothetical protein
TGME49_201400	-4.47	6.24	4.03E-09	6.05E-08	Sin3-associated polypeptide SAP18
TGME49_290900	-9.44	5.86	4.12E-09	6.19E-08	hydrolase, NUDIX family protein
TGME49_251840	1.67	6.35	4.20E-09	6.29E-08	hypothetical protein
TGME49_310930	-3.22	6.48	4.23E-09	6.32E-08	hypothetical protein
TGME49_308950	1.10	8.60	4.25E-09	6.33E-08	histidine acid phosphatase superfamily protein
TGME49_318330	-9.40	5.85	4.28E-09	6.37E-08	histone lysine acetyltransferase MYST-A
TGME49_224460	-2.12	11.30	4.55E-09	6.75E-08	aminopeptidase n, putative
TGME49_297810	-1.71	7.88	4.78E-09	7.08E-08	hypothetical protein
TGME49_313440	4.64	8.07	5.04E-09	7.45E-08	hypothetical protein
TGME49_213010	-4.64	7.34	5.15E-09	7.60E-08	hypothetical protein
TGME49_290160	-1.68	8.18	5.44E-09	8.02E-08	sortilin, putative
TGME49_254840	-3.32	5.67	5.49E-09	8.07E-08	tetratricopeptide repeat-containing protein
TGME49_282150	-9.37	5.86	5.53E-09	8.11E-08	hypothetical protein
TGME49_271760	-9.37	5.72	5.62E-09	8.22E-08	seryl-tRNA synthetase (SerRS2)
TGME49_229470	-4.47	6.35	5.70E-09	8.33E-08	hypothetical protein
TGME49_205040	-1.73	7.70	5.72E-09	8.35E-08	PGAP1 family protein
TGME49_253470	1.58	7.35	5.75E-09	8.37E-08	alveolin domain containing intermediate filament IMC13
TGME49_319580	-9.35	5.99	6.23E-09	9.05E-08	hypothetical protein
TGME49_208590	-2.13	7.00	6.32E-09	9.17E-08	vacuolar ATP synthase subunit 54kD, putative
TGME49_250100	3.11	6.89	6.48E-09	9.35E-08	hypothetical protein
TGME49_231120	1.68	6.29	6.49E-09	9.35E-08	ribosomal protein S11, putative
TGME49_276970	-3.19	6.73	6.87E-09	9.86E-08	hypothetical protein
TGME49_249470	-9.34	5.77	6.87E-09	9.86E-08	rhoptry kinase family protein, truncated (incomplete cat
TGME49_209130	-9.35	5.70	7.08E-09	1.01E-07	regulator of chromosome condensation (RCC1) repeat-c
TGME49_265500	-9.34	6.01	7.16E-09	1.02E-07	chloride transporter, chloride channel (ClC) family protei
TGME49_252640	5.57	4.69	7.29E-09	1.04E-07	P-type ATPase PMA1
TGME49_289580	-9.34	6.04	7.29E-09	1.04E-07	strictosidine synthase subfamily protein
TGME49_200300	3.36	4.83	7.36E-09	1.05E-07	hypothetical protein
TGME49_218192	-9.70	5.36	7.44E-09	1.06E-07	hypothetical protein
TGME49_329800	-2.18	6.84	7.61E-09	1.08E-07	hypothetical protein
TGME49_222960	-9.37	5.95	7.61E-09	1.08E-07	SCY kinase-related protein (incomplete catalytic triad)
TGME49_277910	1.46	6.76	7.78E-09	1.10E-07	thrombospondin type 1 domain-containing protein
TGME49_273905	-4.37	6.09	8.09E-09	1.14E-07	hypothetical protein
TGME49_259530	-4.38	5.96	8.08E-09	1.14E-07	GalNac
TGME49_219070	1.38	7.52	8.53E-09	1.20E-07	cyclic nucleotide-binding domain-containing protein
TGME49_297745	-4.07	6.99	8.84E-09	1.24E-07	hypothetical protein
TGME49_289730	-2.27	7.06	8.90E-09	1.24E-07	Pep3/Vps18/deep orange family protein
TGME49_220100	-2.84	6.73	9.22E-09	1.29E-07	phosphoribosylpyrophosphate synthetase
TGME49_310140	-9.32	5.76	9.75E-09	1.35E-07	hypothetical protein
TGME49_288340	-9.30	5.69	1.03E-08	1.43E-07	UBX domain-containing protein
TGME49_203560	-9.40	5.94	1.06E-08	1.46E-07	hypothetical protein
TGME49_234190	-1.51	8.32	1.07E-08	1.47E-07	serine hydroxymethyltransferase 2, putative
TGME49_269970	1.32	6.91	1.08E-08	1.48E-07	hypothetical protein
TGME49_280390	-2.68	6.88	1.19E-08	1.64E-07	HEAT repeat-containing protein
TGME49_223855	2.84	6.35	1.21E-08	1.66E-07	RNA recognition motif-containing protein
TGME49_227952	-2.49	6.97	1.23E-08	1.69E-07	14-3-3 superfamily protein
TGME49_248530	-9.31	5.80	1.25E-08	1.70E-07	FATC domain-containing protein
TGME49_306930	-1.60	7.80	1.25E-08	1.71E-07	proteasome subunit beta type 7 precursor, putative
TGME49_254370	-1.54	8.18	1.27E-08	1.73E-07	guanylyl cyclase
TGME49_320630	-1.44	7.78	1.29E-08	1.76E-07	phosphotransferase enzyme family protein
TGME49_287040	1.45	7.04	1.30E-08	1.76E-07	hypothetical protein
TGME49_267340	2.16	5.19	1.39E-08	1.88E-07	hypothetical protein
TGME49_290890	-2.85	6.43	1.39E-08	1.88E-07	carbonyl reductase 1, putative
TGME49_262630	3.44	5.14	1.41E-08	1.90E-07	hypothetical protein
TGME49_239260	3.08	4.79	1.43E-08	1.92E-07	histone H4
TGME49_280490	-2.50	7.89	1.47E-08	1.97E-07	U-box domain-containing protein

TGME49_267500	1.02	8.83	1.50E-08	2.01E-07	hypothetical protein
TGME49_265110	-9.30	6.03	1.54E-08	2.05E-07	ribosome biogenesis protein, putative
TGME49_226420	-1.98	6.96	1.57E-08	2.10E-07	peptidase family M3 protein
TGME49_310360	-1.82	7.41	1.59E-08	2.12E-07	hypothetical protein
TGME49_219730	1.33	7.57	1.61E-08	2.15E-07	hypothetical protein
TGME49_209960	-2.20	6.95	1.63E-08	2.17E-07	glycosyltransferase
TGME49_219080	-9.29	5.98	1.64E-08	2.18E-07	edge expressed protein, putative
TGME49_217400	-2.97	6.55	1.67E-08	2.21E-07	hypothetical protein
TGME49_266410	1.53	6.23	1.69E-08	2.22E-07	hypothetical protein
TGME49_278940	1.40	6.96	1.71E-08	2.25E-07	HECT-domain (ubiquitin-transferase) domain-containing
TGME49_319640	1.26	8.46	1.73E-08	2.26E-07	hypothetical protein
TGME49_257750	-2.12	8.17	1.74E-08	2.28E-07	homocysteine s-methyltransferase domain-containing pr
TGME49_227450	1.27	7.22	1.75E-08	2.29E-07	hydrolase, NUDIX family protein
TGME49_318770	-4.35	6.44	1.76E-08	2.30E-07	aurora kinase(incomplete catalytic triad)
TGME49_225060	-2.83	6.46	1.78E-08	2.31E-07	nucleoredoxin family protein
TGME49_293730	-2.04	7.07	1.88E-08	2.44E-07	DHHC zinc finger domain-containing protein
TGME49_235020	-1.75	7.85	1.89E-08	2.45E-07	COPI protein, putative
TGME49_206610	-3.18	6.76	1.91E-08	2.47E-07	pyruvate dehydrogenase complex subunit PDH-E2
TGME49_225580	-9.25	5.94	1.91E-08	2.48E-07	proteasome (prosome, macropain) 26S subunit, non-ATI
TGME49_312110	-2.32	7.05	1.94E-08	2.50E-07	apicoplast-associated thioredoxin family protein Atrx1
TGME49_269450	2.15	5.12	1.95E-08	2.51E-07	hypothetical protein
TGME49_226910	-4.34	6.44	2.04E-08	2.62E-07	Amylo-alpha-1,6-glucosidase
TGME49_263630	1.13	7.63	2.06E-08	2.64E-07	hypothetical protein
TGME49_216510	-4.30	5.85	2.12E-08	2.71E-07	thioredoxin, putative
TGME49_243430	1.86	7.87	2.13E-08	2.71E-07	OTU family cysteine protease
TGME49_269920	-1.47	8.62	2.14E-08	2.72E-07	phosphatidylserine decarboxylase
TGME49_232710	-1.01	10.10	2.14E-08	2.73E-07	ribosomal protein RPS3A
TGME49_312190	-2.11	6.85	2.16E-08	2.74E-07	hypothetical protein
TGME49_239340	-9.25	5.64	2.16E-08	2.74E-07	hypothetical protein
TGME49_222400	1.08	7.58	2.17E-08	2.75E-07	hypothetical protein
TGME49_222710	-2.64	6.78	2.18E-08	2.75E-07	IMP-specific 5'-nucleotidase 1, putative
TGME49_247530	2.40	8.95	2.20E-08	2.78E-07	hypothetical protein
TGME49_233140	-2.48	7.17	2.25E-08	2.82E-07	deoxyuridine 5'-triphosphate nucleotidohydrolase, putat
TGME49_203730	-9.23	5.89	2.38E-08	2.99E-07	hypothetical protein
TGME49_205510	-1.74	7.70	2.47E-08	3.09E-07	nucleolar protein 5, putative
TGME49_231130	-9.23	5.70	2.49E-08	3.11E-07	hypothetical protein
TGME49_310450	-2.34	7.13	2.74E-08	3.41E-07	myosin heavy chain, putative
TGME49_261750	-2.65	6.81	2.87E-08	3.58E-07	rhoptry neck protein RON10
TGME49_265390	-9.23	6.16	2.89E-08	3.59E-07	hypothetical protein
TGME49_239780	-3.58	6.36	2.99E-08	3.70E-07	hypothetical protein
TGME49_211470	2.32	5.17	3.03E-08	3.74E-07	Fcf2 pre-rRNA processing protein
TGME49_322000	-2.58	7.25	3.20E-08	3.96E-07	myosin-light-chain kinase
TGME49_206690	-2.30	7.05	3.25E-08	4.00E-07	glideosome-associated protein with multiple-membrane
TGME49_227930	1.31	7.02	3.29E-08	4.04E-07	hypothetical protein
TGME49_240800	-2.22	6.86	3.33E-08	4.09E-07	MORN repeat-containing protein
TGME49_203760	1.68	6.32	3.53E-08	4.32E-07	hypothetical protein
TGME49_227420	-9.24	5.89	3.59E-08	4.38E-07	4-hydroxy-3-methylbut-2-enyl diphosphate reductase
TGME49_298830	1.81	6.26	3.64E-08	4.44E-07	hypothetical protein
TGME49_217688	-9.21	5.67	3.68E-08	4.46E-07	hypothetical protein
TGME49_207160	4.41	5.87	3.74E-08	4.53E-07	SAG-related sequence SRS49D
TGME49_280800	-1.91	7.39	3.75E-08	4.54E-07	SWI2/SNF2 SRCAP/Ino80
TGME49_294940	1.46	6.95	3.80E-08	4.59E-07	hypothetical protein
TGME49_228750	-3.53	6.46	3.97E-08	4.78E-07	TGME49_228750 CAM kinase, RAD family
TGME49_306960	-2.56	6.60	4.06E-08	4.90E-07	phenylalanine--tRNA ligase, beta subunit protein
TGME49_306600	-9.22	5.73	4.16E-08	5.00E-07	RNA recognition motif-containing protein
TGME49_267620	-2.90	6.97	4.17E-08	5.00E-07	multi-pass transmembrane protein
TGME49_323320	-3.55	5.30	4.22E-08	5.06E-07	hypothetical protein

TGME49_237000	-4.26	5.99	4.23E-08	5.07E-07	polyphosphoinositide binding protein, putative
TGME49_269290	-3.04	6.26	4.37E-08	5.22E-07	hypothetical protein
TGME49_251170	2.43	6.41	4.38E-08	5.23E-07	KRUF family protein
TGME49_315750	-1.69	7.00	4.43E-08	5.28E-07	hypothetical protein
TGME49_271270	-1.79	7.33	4.56E-08	5.43E-07	hypothetical protein
TGME49_212170	-9.18	5.94	4.64E-08	5.51E-07	GIY-YIG catalytic domain-containing protein
TGME49_259020	6.07	5.55	4.68E-08	5.53E-07	bradyzoite antigen BAG1
TGME49_216130	-2.29	6.73	4.67E-08	5.53E-07	ubiquitin conjugating enzyme E2, putative
TGME49_274010	-4.26	6.06	4.78E-08	5.64E-07	hypothetical protein
TGME49_225745	-9.21	6.00	4.80E-08	5.65E-07	hypothetical protein
TGME49_311110	-4.24	5.89	4.84E-08	5.69E-07	Ubiquitin-fold modifier 1 precursor family protein, putat
TGME49_202980	-2.28	7.20	5.07E-08	5.95E-07	hypothetical protein
TGME49_230190	-2.17	6.88	5.10E-08	5.97E-07	hypothetical protein
TGME49_201680	-1.14	8.83	5.14E-08	6.00E-07	eukaryotic initiation factor-3 subunit 10, putative
TGME49_306460	-9.19	5.67	5.14E-08	6.00E-07	bromodomain-containing protein
TGME49_203840	1.84	5.77	5.19E-08	6.06E-07	DEAD/DEAH box helicase domain-containing protein
TGME49_311090	-1.64	7.32	5.24E-08	6.10E-07	ubiquitin carboxyl-terminal hydrolase
TGME49_286630	-1.63	7.93	5.28E-08	6.13E-07	redoxin domain-containing protein
TGME49_258580	-1.04	9.75	5.33E-08	6.18E-07	rhoptry protein ROP17
TGME49_215420	-9.16	5.72	5.37E-08	6.22E-07	SNARE protein
TGME49_223010	-3.05	6.45	5.58E-08	6.46E-07	hypothetical protein
TGME49_264080	-1.60	8.18	5.60E-08	6.47E-07	acyl carrier protein ACP
TGME49_288530	1.62	6.22	5.75E-08	6.63E-07	NOL1/NOP2/sun family protein
TGME49_253880	1.18	8.10	5.83E-08	6.72E-07	GNS1/SUR4 family protein
TGME49_230430	-9.16	5.34	5.89E-08	6.77E-07	vesicle-associated membrane protein, putative
TGME49_273350	2.12	5.36	6.01E-08	6.90E-07	molybdopterin converting factor, subunit 2 protein
TGME49_309990	3.23	6.06	6.07E-08	6.96E-07	hypothetical protein
TGME49_310530	-4.26	6.15	6.08E-08	6.96E-07	SNF2 family N-terminal domain-containing protein
TGME49_227380	1.17	9.57	6.15E-08	7.03E-07	hypothetical protein
TGME49_278800	-1.89	7.62	6.18E-08	7.05E-07	zinc finger protein 36 family 3 protein
TGME49_207370	1.01	8.45	6.34E-08	7.22E-07	hypothetical protein
TGME49_201820	1.51	6.72	6.41E-08	7.28E-07	hypothetical protein
TGME49_229780	-4.23	5.86	6.41E-08	7.28E-07	GHMP kinase, N-terminal domain-containing protein
TGME49_204280	1.28	7.67	6.43E-08	7.29E-07	cell-cycle-associated protein kinase DYRK, putative
TGME49_229480	-1.02	9.72	6.84E-08	7.73E-07	calcium binding protein precursor, putative
TGME49_231160	-1.01	9.22	6.93E-08	7.81E-07	hypothetical protein
TGME49_213920	-3.03	6.79	6.95E-08	7.82E-07	hypothetical protein
TGME49_227948	-1.37	8.32	7.28E-08	8.19E-07	peptidase M16 inactive domain-containing protein
TGME49_269010	1.36	9.54	7.31E-08	8.21E-07	AP2 domain transcription factor AP2VIII-7
TGME49_268580	-4.26	6.06	7.44E-08	8.33E-07	hypothetical protein
TGME49_208820	-4.32	5.82	7.51E-08	8.40E-07	1-deoxy-D-xylulose-5-phosphate synthase
TGME49_218820	-1.92	8.78	7.57E-08	8.46E-07	alba 2
TGME49_269980	-1.07	9.36	7.81E-08	8.69E-07	preprotein translocase Sec61, putative
TGME49_207620	-1.28	7.92	8.01E-08	8.88E-07	pyridine nucleotide-disulfide oxidoreductase domain-co
TGME49_297870	-2.31	6.90	8.02E-08	8.88E-07	hypothetical protein
TGME49_240570	-2.61	6.80	8.01E-08	8.88E-07	hypothetical protein
TGME49_247580	-9.15	5.86	8.01E-08	8.88E-07	glutaredoxin domain-containing protein
TGME49_214230	1.24	6.85	8.31E-08	9.18E-07	Dopey, N-terminal domain-containing protein
TGME49_229370	-4.19	5.92	8.35E-08	9.21E-07	AP2 domain transcription factor AP2VIII-1
TGME49_206605	2.24	5.59	8.42E-08	9.28E-07	hypothetical protein
TGME49_306510	3.54	5.11	8.47E-08	9.32E-07	hypothetical protein
TGME49_311070	-3.50	6.42	8.52E-08	9.36E-07	hypothetical protein
TGME49_241880	-1.35	8.19	9.23E-08	1.01E-06	3'5'-cyclic nucleotide phosphodiesterase domain-contai
TGME49_254030	1.86	8.18	9.25E-08	1.01E-06	zinc finger CDGSH-type domain-containing protein
TGME49_313780	-1.69	7.72	9.27E-08	1.01E-06	hypothetical protein
TGME49_207770	-1.66	7.77	9.31E-08	1.02E-06	PCI domain-containing protein
TGME49_249360	-2.24	6.90	9.61E-08	1.05E-06	RED family protein

TGME49_294290	-2.21	7.07	9.64E-08	1.05E-06	Der1ER1
TGME49_232560	-2.40	6.98	9.64E-08	1.05E-06	hypothetical protein
TGME49_278975	-2.23	6.87	9.81E-08	1.07E-06	ICE family protease (caspase) p20 domain-containing pro
TGME49_269420	1.21	7.98	1.01E-07	1.10E-06	hypothetical protein
TGME49_216070	-9.10	5.74	1.04E-07	1.13E-06	hypothetical protein
TGME49_271350	-9.10	5.41	1.12E-07	1.20E-06	bifunctional protein FolC subfamily protein
TGME49_226030	-1.35	8.61	1.15E-07	1.24E-06	AGC kinase
TGME49_231910	-1.74	7.36	1.15E-07	1.24E-06	ATP synthase F1 gamma subunit
TGME49_256770	-1.47	7.59	1.16E-07	1.25E-06	eukaryotic translation initiation factor 4A, isoform 3, put
TGME49_266470	-9.09	5.57	1.16E-07	1.25E-06	hypothetical protein
TGME49_208780	-3.00	6.22	1.18E-07	1.27E-06	ubiquitin-conjugating enzyme subfamily protein
TGME49_272010	-4.20	6.12	1.23E-07	1.31E-06	Gar1 protein RNA binding region protein
TGME49_307820	1.77	5.87	1.24E-07	1.32E-06	hypothetical protein
TGME49_236550	-1.54	7.92	1.24E-07	1.32E-06	hypothetical protein
TGME49_203350	-9.18	5.69	1.25E-07	1.32E-06	hypothetical protein
TGME49_248860	1.86	5.75	1.28E-07	1.35E-06	hypothetical protein
TGME49_232160	1.33	6.85	1.28E-07	1.35E-06	zinc finger, C3HC4 type (RING finger) domain-containing
TGME49_215290	-4.16	5.79	1.33E-07	1.40E-06	saccharopine dehydrogenase domain-containing protein
TGME49_215460	-1.56	9.58	1.33E-07	1.40E-06	ribosomal protein RPS24
TGME49_314920	2.18	5.56	1.38E-07	1.45E-06	hypothetical protein
TGME49_262640	-1.53	7.82	1.39E-07	1.46E-06	Cg8 family protein
TGME49_268960	-3.38	6.09	1.39E-07	1.46E-06	5'-AMP-activated protein kinase subunit beta-1 family pr
TGME49_271010	3.30	5.05	1.42E-07	1.48E-06	hypothetical protein
TGME49_244500	-1.75	7.47	1.42E-07	1.48E-06	Tubulin-tyrosine ligase family protein
TGME49_268890	-1.90	7.12	1.42E-07	1.48E-06	citrate synthase I
TGME49_235398	-9.07	5.77	1.42E-07	1.48E-06	hypothetical protein
TGME49_311390	2.33	5.11	1.43E-07	1.49E-06	tRNA (guanine(9)-N(1))-methyltransferase
TGME49_313330	-9.34	5.76	1.44E-07	1.50E-06	rhoptry kinase family protein ROP27
TGME49_313830	-2.96	6.34	1.45E-07	1.50E-06	AARP2CN (NUC121) domain-containing protein
TGME49_221295	-4.16	5.92	1.47E-07	1.53E-06	hypothetical protein
TGME49_306030	-1.89	7.04	1.49E-07	1.54E-06	glutathione s-transferase, n-terminal domain containing
TGME49_227030	-2.14	6.87	1.52E-07	1.56E-06	hypothetical protein
TGME49_249790	-4.15	5.87	1.51E-07	1.56E-06	hypothetical protein
TGME49_270550	-9.15	5.78	1.53E-07	1.57E-06	gamma-glutamyl phosphate reductase, putative
TGME49_254470	1.10	9.18	1.55E-07	1.59E-06	hypothetical protein
TGME49_231200	-2.96	6.19	1.58E-07	1.62E-06	hypothetical protein
TGME49_251680	-1.49	8.93	1.66E-07	1.70E-06	histamine-releasing factor, putative
TGME49_216630	-9.05	5.35	1.66E-07	1.70E-06	trigger factor protein, putative
TGME49_227140	3.00	4.81	1.68E-07	1.72E-06	hypothetical protein
TGME49_289310	-2.38	6.67	1.73E-07	1.76E-06	cullin family protein
TGME49_296015	1.78	6.18	1.76E-07	1.79E-06	hypothetical protein
TGME49_247960	1.46	6.05	1.76E-07	1.79E-06	hypothetical protein
TGME49_314700	1.31	7.77	1.80E-07	1.82E-06	hypothetical protein
TGME49_263500	-4.19	5.93	1.80E-07	1.82E-06	vacuolar protein sorting-associated protein 26, putative
TGME49_253440	1.08	9.25	1.81E-07	1.83E-06	cell-cycle-associated protein kinase SRPK, putative
TGME49_238970	-9.70	5.17	1.88E-07	1.90E-06	hypothetical protein
TGME49_306470	-2.66	6.39	1.88E-07	1.90E-06	isoprenylcysteine carboxyl methyltransferase (icmt) fam
TGME49_226430	-1.31	8.72	1.89E-07	1.90E-06	reticulon protein
TGME49_230950	-9.09	5.62	1.93E-07	1.94E-06	hypothetical protein
TGME49_209700	-4.13	5.90	1.96E-07	1.97E-06	hypothetical protein
TGME49_285490	-9.07	5.57	1.97E-07	1.97E-06	helix-hairpin-helix motif domain-containing protein
TGME49_241890	-9.04	5.67	1.98E-07	1.99E-06	hypothetical protein
TGME49_217350	-9.07	5.39	2.09E-07	2.09E-06	methyltransferase MTA70, putative
TGME49_259240	1.03	9.56	2.12E-07	2.11E-06	ribosomal protein RPS21
TGME49_254290	1.66	6.05	2.18E-07	2.17E-06	hypothetical protein
TGME49_235490	-1.85	7.03	2.19E-07	2.17E-06	hypothetical protein
TGME49_314540	-9.02	5.67	2.19E-07	2.17E-06	hypothetical protein

TGME49_305980	-2.14	7.06	2.23E-07	2.21E-06	pyruvate dehydrogenase complex subunit PDH-E3I
TGME49_214900	1.39	7.26	2.25E-07	2.23E-06	hypothetical protein
TGME49_248890	-2.93	6.03	2.26E-07	2.23E-06	actin-like protein ALP3b
TGME49_295658	-3.34	6.32	2.31E-07	2.28E-06	zinc finger in N-recognin protein
TGME49_270800	-4.23	6.03	2.34E-07	2.31E-06	GAF domain-containing protein
TGME49_266750	-1.53	7.68	2.38E-07	2.34E-06	transporter/permease protein, putative
TGME49_217360	-3.36	5.95	2.39E-07	2.35E-06	hypothetical protein
TGME49_293860	-9.03	5.58	2.40E-07	2.36E-06	hypothetical protein
TGME49_242870	-9.02	5.35	2.43E-07	2.38E-06	histone lysine methyltransferase, SET, putative
TGME49_286650	-9.01	5.60	2.56E-07	2.50E-06	hypothetical protein
TGME49_293740	1.56	8.89	2.62E-07	2.55E-06	hypothetical protein
TGME49_248260	1.66	6.49	2.65E-07	2.58E-06	hypothetical protein
TGME49_250220	2.46	5.45	2.66E-07	2.58E-06	hypothetical protein
TGME49_207100	-8.99	5.81	2.75E-07	2.67E-06	hypothetical protein
TGME49_278440	-2.07	6.87	2.77E-07	2.68E-06	SWI2/SNF2 Brahma-like putative
TGME49_222290	-4.11	5.85	2.78E-07	2.68E-06	LSM domain-containing protein
TGME49_248160	-9.03	5.47	2.79E-07	2.69E-06	hypothetical protein
TGME49_210980	1.67	6.94	2.81E-07	2.71E-06	alternative splicing type 3 and, putative
TGME49_227060	-9.03	5.72	2.82E-07	2.71E-06	hypothetical protein
TGME49_203740	-2.36	6.73	2.88E-07	2.76E-06	hypothetical protein
TGME49_313400	-2.09	7.11	2.95E-07	2.83E-06	DnaJ domain-containing protein
TGME49_291020	-2.06	6.79	3.06E-07	2.93E-06	myosin head (motor domain) domain-containing protein
TGME49_229630	1.10	8.70	3.16E-07	3.01E-06	eIF2 kinase IF2K-A (incomplete catalytic triad)
TGME49_224310	-9.06	5.58	3.16E-07	3.01E-06	DHHC zinc finger domain-containing protein
TGME49_228770	-4.13	5.98	3.23E-07	3.07E-06	hypothetical protein
TGME49_218362	-2.30	6.89	3.23E-07	3.07E-06	zinc finger protein ZFP1
TGME49_214200	-9.04	5.94	3.25E-07	3.09E-06	WD domain, G-beta repeat-containing protein
TGME49_310670	-1.38	7.93	3.32E-07	3.15E-06	glycogen phosphorylase 1, putative
TGME49_210478	5.25	4.78	3.33E-07	3.16E-06	hypothetical protein
TGME49_225770	-8.97	5.71	3.44E-07	3.25E-06	Tyrosine kinase-like (TKL) protein
TGME49_208500	-2.68	6.45	3.47E-07	3.28E-06	protein phosphatase 2C domain-containing protein
TGME49_313290	1.19	6.91	3.49E-07	3.28E-06	MORN repeat-containing protein
TGME49_244110	-1.59	8.05	3.58E-07	3.37E-06	nucleosome assembly protein (nap) protein
TGME49_312300	-1.71	7.41	3.59E-07	3.37E-06	Sec7 domain-containing protein
TGME49_209280	-4.08	6.12	3.64E-07	3.41E-06	hypothetical protein
TGME49_311870	-2.45	6.63	3.67E-07	3.44E-06	WD domain, G-beta repeat-containing protein
TGME49_301440	-1.17	9.35	3.78E-07	3.54E-06	calcium-dependent protein kinase CDPK1
TGME49_259520	-9.00	5.47	3.78E-07	3.54E-06	hypothetical protein
TGME49_271870	-8.96	5.32	3.80E-07	3.54E-06	zinc carboxypeptidase superfamily protein
TGME49_310230	-9.01	5.63	3.80E-07	3.54E-06	hypothetical protein
TGME49_275990	1.41	6.58	3.86E-07	3.59E-06	hypothetical protein
TGME49_278630	-8.95	5.70	3.88E-07	3.60E-06	tetratricopeptide repeat-containing protein
TGME49_245730	-8.95	5.76	3.92E-07	3.64E-06	phosphatidylinositol-4-phosphate 5-Kinase
TGME49_210230	-9.02	5.90	3.96E-07	3.67E-06	hypothetical protein
TGME49_238040	-1.90	6.79	3.97E-07	3.68E-06	protein disulfide-isomerase domain-containing protein
TGME49_213870	-1.72	6.92	4.02E-07	3.71E-06	UBA/TS-N domain-containing protein
TGME49_282190	-1.51	7.07	4.05E-07	3.73E-06	hydrolase, NUDIX family protein
TGME49_252870	2.35	4.88	4.06E-07	3.74E-06	hypothetical protein
TGME49_315250	-4.09	6.00	4.16E-07	3.82E-06	GAMM1 protein, putative
TGME49_221720	-4.40	6.44	4.16E-07	3.82E-06	hypothetical protein
TGME49_310570	-9.12	5.49	4.17E-07	3.82E-06	hypothetical protein
TGME49_284190	-3.49	6.62	4.26E-07	3.90E-06	pyruvate carboxylase
TGME49_238110	-2.69	6.29	4.28E-07	3.91E-06	replication factor a protein 3 protein
TGME49_264220	-8.96	5.51	4.31E-07	3.94E-06	hypothetical protein
TGME49_219800	-1.29	8.18	4.35E-07	3.97E-06	vacuolar ATP synthase subunit b, putative
TGME49_240370	8.58	4.77	4.47E-07	4.08E-06	Toxoplasma gondii family E protein
TGME49_254230	1.89	5.65	4.55E-07	4.14E-06	hypothetical protein

TGME49_224540	-9.10	6.05	4.81E-07	4.38E-06	hypothetical protein
TGME49_202572	-8.95	5.64	4.89E-07	4.44E-06	ribophorin i protein
TGME49_289640	2.07	5.64	4.93E-07	4.47E-06	hypothetical protein
TGME49_207800	-8.93	5.28	5.06E-07	4.58E-06	hypothetical protein
TGME49_246160	2.70	4.93	5.13E-07	4.64E-06	hypothetical protein
TGME49_266450	1.34	6.38	5.14E-07	4.64E-06	lysine decarboxylase family protein
TGME49_245485	2.12	6.56	5.18E-07	4.68E-06	microneme protein MIC9
TGME49_300290	-8.98	5.41	5.27E-07	4.75E-06	SNARE domain-containing protein
TGME49_274000	-2.90	6.01	5.43E-07	4.87E-06	hypothetical protein
TGME49_208970	-1.95	6.92	5.43E-07	4.88E-06	RNA recognition motif-containing protein
TGME49_200360	1.90	9.74	5.54E-07	4.96E-06	hypothetical protein
TGME49_305560	-4.05	5.81	5.54E-07	4.96E-06	Vps51/Vps67 protein
TGME49_222860	-1.33	8.81	5.60E-07	5.00E-06	eukaryotic translation initiation factor, putative
TGME49_216500	-4.05	5.77	5.61E-07	5.01E-06	tRNA synthetase, putative
TGME49_205000	-8.94	5.59	5.64E-07	5.03E-06	phosphoglycerate mutase family protein
TGME49_290660	-1.01	9.43	6.14E-07	5.46E-06	RNA recognition motif-containing protein
TGME49_214370	-8.91	5.49	6.25E-07	5.54E-06	hypothetical protein
TGME49_239580	-9.11	4.82	6.37E-07	5.65E-06	hypothetical protein
TGME49_263000	1.31	7.22	6.46E-07	5.72E-06	Beige/BEACH domain-containing protein
TGME49_292610	-8.91	4.84	6.47E-07	5.72E-06	Toxoplasma gondii family C protein
TGME49_293440	-1.64	7.35	6.49E-07	5.74E-06	hypothetical protein
TGME49_305470	-8.93	5.79	6.52E-07	5.75E-06	hypothetical protein
TGME49_243410	-4.05	5.61	6.71E-07	5.90E-06	tetratricopeptide repeat-containing protein
TGME49_227580	-1.59	7.61	6.73E-07	5.92E-06	transmembrane amino acid transporter protein
TGME49_252390	1.12	8.54	6.81E-07	5.98E-06	hypothetical protein
TGME49_285970	-8.90	5.58	6.84E-07	6.00E-06	30S ribosomal protein S5, putative
TGME49_280730	-8.90	5.83	6.86E-07	6.01E-06	cytosolic fe-s cluster assembling factor nbp35, putative
TGME49_297760	-8.91	5.12	6.88E-07	6.02E-06	hypothetical protein
TGME49_202120	-1.59	7.31	6.89E-07	6.02E-06	hypothetical protein
TGME49_306910	-2.37	6.36	6.91E-07	6.03E-06	hypothetical protein
TGME49_212140	-4.21	6.11	6.92E-07	6.04E-06	hypothetical protein
TGME49_242055	-8.91	5.77	6.97E-07	6.07E-06	DEAD/DEAH box helicase domain-containing protein
TGME49_239620	-4.01	5.74	7.27E-07	6.32E-06	5'-nucleotidase, C-terminal domain-containing protein
TGME49_270090	-3.26	6.03	7.38E-07	6.40E-06	hypothetical protein
TGME49_286260	-8.89	5.83	7.43E-07	6.44E-06	tetratricopeptide repeat-containing protein
TGME49_217420	-4.14	6.00	7.54E-07	6.53E-06	hypothetical protein
TGME49_265010	-8.88	5.64	7.76E-07	6.71E-06	glutamate 5-kinase domain-containing protein
TGME49_221630	-3.23	5.92	8.10E-07	6.99E-06	hypothetical protein
TGME49_288570	-3.23	5.83	8.09E-07	6.99E-06	hypothetical protein
TGME49_236630	-1.36	7.56	8.15E-07	7.02E-06	hypothetical protein
TGME49_319880	-4.00	6.31	8.23E-07	7.08E-06	MORN repeat-containing protein
TGME49_249250	1.14	10.07	8.36E-07	7.19E-06	ribosomal protein RPL35A
TGME49_306410	-4.00	5.79	8.55E-07	7.34E-06	hypothetical protein
TGME49_220430	-8.87	5.67	8.56E-07	7.34E-06	hypothetical protein
TGME49_285510	-1.44	7.24	8.62E-07	7.38E-06	hypothetical protein
TGME49_314970	-1.54	7.24	8.73E-07	7.47E-06	root hair defective 3 gtp-binding protein (rhd3) protein
TGME49_256880	-8.89	5.54	8.78E-07	7.50E-06	protein kinase domain-containing protein
TGME49_224020	1.43	6.33	8.79E-07	7.51E-06	hypothetical protein
TGME49_266100	1.28	6.94	8.87E-07	7.56E-06	rhoptry kinase family protein ROP41
TGME49_295080	-8.87	5.79	8.99E-07	7.66E-06	hypothetical protein
TGME49_301400	1.69	9.99	9.09E-07	7.73E-06	hypothetical protein
TGME49_268910	1.66	5.89	9.11E-07	7.73E-06	signal peptidase I protein
TGME49_267670	1.90	5.56	9.16E-07	7.77E-06	hypothetical protein
TGME49_242850	-2.81	5.93	9.30E-07	7.88E-06	hypothetical protein
TGME49_216430	-1.58	7.55	9.54E-07	8.07E-06	TBC domain-containing protein
TGME49_271470	-3.99	6.12	9.54E-07	8.07E-06	hypothetical protein
TGME49_214520	1.79	5.70	9.56E-07	8.07E-06	general transcription factor IIH polypeptide 4 GTF2H4

TGME49_246100	-2.29	6.79	9.57E-07	8.07E-06	phosducin, putative
TGME49_214820	1.43	6.75	9.70E-07	8.17E-06	G-patch domain-containing protein
TGME49_249180	-1.22	8.49	9.73E-07	8.19E-06	bifunctional dihydrofolate reductase-thymidylate syntha
TGME49_266730	-8.85	5.67	9.83E-07	8.25E-06	leucyl-tRNA synthetase (LeuRS2)
TGME49_209090	-1.90	6.52	9.88E-07	8.28E-06	proteasome maturation factor ump1 protein
TGME49_217450	2.11	4.96	9.97E-07	8.35E-06	general transcription factor IIH polypeptide 5 GTF2H5
TGME49_271820	-8.87	5.98	1.02E-06	8.55E-06	lipoyltransferase and lipoate-protein ligase subfamily pro
TGME49_254660	1.48	6.94	1.03E-06	8.57E-06	ankyrin repeat-containing protein
TGME49_255160	-8.87	5.35	1.03E-06	8.57E-06	hypothetical protein
TGME49_268790	1.03	8.56	1.03E-06	8.57E-06	hypothetical protein
TGME49_289700	-2.82	6.24	1.07E-06	8.93E-06	hypothetical protein
TGME49_315860	-2.21	6.65	1.08E-06	8.94E-06	EF hand domain-containing protein
TGME49_229490	-2.83	6.21	1.08E-06	8.94E-06	tetratricopeptide repeat-containing protein
TGME49_248850	-8.91	5.24	1.09E-06	9.08E-06	methionine aminopeptidase
TGME49_214870	1.97	5.32	1.10E-06	9.09E-06	ribosomal protein L9, N-terminal domain-containing pro
TGME49_309930	1.37	8.33	1.10E-06	9.09E-06	melibiase subfamily protein
TGME49_225050	-1.56	7.46	1.13E-06	9.31E-06	adenosylhomocysteinase, putative
TGME49_310590	1.85	5.32	1.13E-06	9.34E-06	hypothetical protein
TGME49_260390	-8.85	5.56	1.14E-06	9.40E-06	hypothetical protein
TGME49_306895	1.09	7.35	1.15E-06	9.43E-06	hypothetical protein
TGME49_308010	1.53	6.29	1.15E-06	9.47E-06	hypothetical protein
TGME49_294270	-8.83	5.61	1.16E-06	9.51E-06	histone arginine methyltransferase PRMT4/CARM1
TGME49_271810	-1.39	7.84	1.17E-06	9.56E-06	lanp, putative
TGME49_219250	1.12	7.49	1.17E-06	9.58E-06	acetyltransferase, GNAT family protein
TGME49_250830	-1.69	7.32	1.17E-06	9.58E-06	SAC3/GANP family protein
TGME49_243298	-8.88	5.50	1.17E-06	9.58E-06	ICE family protease (caspase) p20 domain-containing pro
TGME49_232780	-2.87	6.30	1.17E-06	9.58E-06	hypothetical protein
TGME49_217770	1.34	7.05	1.18E-06	9.65E-06	hypothetical protein
TGME49_314400	-1.11	8.35	1.19E-06	9.71E-06	pyruvate dehydrogenase E1 component, beta subunit, p
TGME49_293710	-1.65	7.43	1.19E-06	9.71E-06	Zn-finger in Ran binding protein and others domain-cont
TGME49_221580	-1.50	7.41	1.22E-06	9.93E-06	ribosomal RNA large subunit methyltransferase J protein
TGME49_286928	-2.53	5.69	1.23E-06	9.96E-06	hypothetical protein
TGME49_230350	-1.27	8.27	1.25E-06	1.01E-05	hypothetical protein
TGME49_292235	-8.85	5.58	1.25E-06	1.02E-05	hypothetical protein
TGME49_278950	1.74	5.34	1.28E-06	1.03E-05	LSM domain-containing protein
TGME49_260260	-1.04	9.53	1.28E-06	1.03E-05	ribosomal protein RPP1
TGME49_228100	-1.98	7.17	1.28E-06	1.03E-05	hypothetical protein
TGME49_239020	-8.90	6.19	1.29E-06	1.04E-05	ABC transporter transmembrane region domain-contain
TGME49_270610	1.83	5.44	1.30E-06	1.05E-05	hypothetical protein
TGME49_312905	2.29	7.21	1.31E-06	1.05E-05	hypothetical protein
TGME49_257520	1.11	7.16	1.31E-06	1.05E-05	synaptobrevin protein
TGME49_237480	-3.95	5.71	1.31E-06	1.05E-05	BRCA1 C Terminus (BRCT) domain-containing protein
TGME49_235740	-3.95	5.69	1.35E-06	1.08E-05	hypothetical protein
TGME49_264690	-8.84	5.40	1.36E-06	1.09E-05	cyclin 4, putative
TGME49_312500	-1.71	7.05	1.37E-06	1.09E-05	hypothetical protein
TGME49_248450	-2.80	6.26	1.40E-06	1.12E-05	zinc finger, C3HC4 type (RING finger) domain-containing
TGME49_239752	-1.35	8.30	1.43E-06	1.14E-05	hypothetical protein
TGME49_248150	-3.97	5.73	1.46E-06	1.17E-05	hypothetical protein
TGME49_251920	2.09	5.05	1.47E-06	1.17E-05	hypothetical protein
TGME49_235478	-4.14	5.93	1.47E-06	1.17E-05	pantothenate kinase
TGME49_295440	1.48	5.97	1.47E-06	1.17E-05	hypothetical protein
TGME49_268980	1.13	7.97	1.49E-06	1.18E-05	hypothetical protein
TGME49_214290	-1.17	7.82	1.51E-06	1.20E-05	DJ-1 family protein
TGME49_227650	-2.35	6.80	1.53E-06	1.22E-05	microtubule-associated protein RP/EB family, putative
TGME49_239560	-8.96	5.38	1.53E-06	1.22E-05	myosin E
TGME49_253000	1.17	6.99	1.55E-06	1.23E-05	ELMO/CED-12 family protein
TGME49_318880	2.26	5.55	1.56E-06	1.23E-05	hypothetical protein

TGME49_314730	-3.97	5.86	1.56E-06	1.23E-05	ALG6, ALG8 glycosyltransferase family protein
TGME49_310910	-8.88	5.61	1.56E-06	1.23E-05	WD domain, G-beta repeat-containing protein
TGME49_242110	-8.92	4.87	1.56E-06	1.23E-05	rhoptry kinase family protein ROP38
TGME49_227320	-8.94	5.44	1.56E-06	1.23E-05	hypothetical protein
TGME49_222210	-8.80	5.46	1.57E-06	1.23E-05	SPFH domain / Band 7 family protein
TGME49_260320	-2.79	6.27	1.57E-06	1.23E-05	Noc2p family protein
TGME49_274110	-8.80	5.68	1.58E-06	1.24E-05	glycoprotease family protein
TGME49_278720	-8.84	5.75	1.62E-06	1.27E-05	hypothetical protein
TGME49_286560	-8.93	5.40	1.63E-06	1.27E-05	U6 snRNA-associated Sm family protein
TGME49_300020	-3.92	5.75	1.64E-06	1.28E-05	ATP-dependent metallopeptidase HflB subfamily protein
TGME49_268760	-1.21	8.36	1.65E-06	1.29E-05	hypothetical protein
TGME49_241180	-1.49	7.10	1.69E-06	1.32E-05	hypothetical protein
TGME49_282000	1.43	6.72	1.70E-06	1.33E-05	hypothetical protein
TGME49_313180	-1.09	8.40	1.73E-06	1.34E-05	cell-cycle-associated protein kinase PRP4, putative
TGME49_286740	3.41	5.13	1.73E-06	1.34E-05	microneme-like protein
TGME49_276990	-3.93	5.48	1.73E-06	1.34E-05	cytochrome b5 family heme/steroid binding domain-con
TGME49_246460	-3.97	5.93	1.73E-06	1.34E-05	hypothetical protein
TGME49_202470	-8.80	5.84	1.73E-06	1.34E-05	rRNA metabolism protein, SBDS family protein
TGME49_213900	1.12	7.70	1.74E-06	1.34E-05	regulator of chromosome condensation RCC1
TGME49_313160	-3.20	5.73	1.74E-06	1.35E-05	hypothetical protein
TGME49_281990	1.70	5.47	1.76E-06	1.36E-05	Nicotinamidase
TGME49_289290	-1.92	7.07	1.78E-06	1.37E-05	hypothetical protein
TGME49_313350	-3.92	5.75	1.78E-06	1.37E-05	hypothetical protein
TGME49_215210	2.76	6.31	1.79E-06	1.38E-05	hypothetical protein
TGME49_225480	-3.93	5.99	1.79E-06	1.38E-05	hypothetical protein
TGME49_224660	1.20	6.78	1.81E-06	1.39E-05	transcription factor s-ii (tfiis), central domain-containing
TGME49_213790	-2.78	6.32	1.82E-06	1.39E-05	hypothetical protein
TGME49_243960	-1.09	8.61	1.83E-06	1.40E-05	nuclear transport factor 2 (ntf2) domain-containing prot
TGME49_308020	-1.24	7.95	1.87E-06	1.43E-05	SAG-related sequence SRS57
TGME49_233030	-1.11	8.26	1.87E-06	1.43E-05	gliding-associated protein GAP70
TGME49_236890	1.14	7.15	1.88E-06	1.43E-05	hypothetical protein
TGME49_240520	-8.80	5.59	1.88E-06	1.43E-05	hypothetical protein
TGME49_267330	-2.30	6.42	1.91E-06	1.45E-05	fumarate hydratase
TGME49_225470	1.35	6.28	1.93E-06	1.46E-05	peptide methionine sulfoxide reductase
TGME49_264030	-1.99	6.63	1.93E-06	1.46E-05	aminotransferase, putative
TGME49_247330	-2.07	6.92	1.93E-06	1.46E-05	hypothetical protein
TGME49_212150	-8.79	5.30	1.96E-06	1.48E-05	hypothetical protein
TGME49_216620	-1.15	8.10	1.97E-06	1.49E-05	EF hand domain-containing protein
TGME49_248880	-1.41	7.90	1.98E-06	1.49E-05	GTPase RAB7
TGME49_297800	-2.56	6.62	2.02E-06	1.52E-05	RecF/RecN/SMC N terminal domain-containing protein
TGME49_205460	-8.77	5.47	2.07E-06	1.55E-05	AN1 family Zinc finger domain-containing protein
TGME49_318410	-1.37	7.53	2.11E-06	1.58E-05	TCP-1 chaperonin, putative
TGME49_251850	-1.73	7.02	2.11E-06	1.58E-05	serine/threonine protein phosphatase
TGME49_247380	-8.78	5.36	2.12E-06	1.58E-05	hypothetical protein
TGME49_252500	1.13	7.74	2.13E-06	1.59E-05	polo kinase
TGME49_231480	-1.60	7.27	2.20E-06	1.64E-05	GCN1, putative
TGME49_219140	-1.77	6.80	2.21E-06	1.65E-05	EF-1 guanine nucleotide exchange domain-containing pr
TGME49_264090	-3.20	6.14	2.22E-06	1.65E-05	hypothetical protein
TGME49_311500	-8.88	5.49	2.22E-06	1.65E-05	ThiF family protein
TGME49_209260	-1.17	8.33	2.24E-06	1.66E-05	cytochrome c oxidase subunit, putative
TGME49_248640	-1.50	7.36	2.24E-06	1.66E-05	regulator of chromosome condensation (RCC1) repeat-c
TGME49_269660	-2.16	6.75	2.29E-06	1.70E-05	TFIIH basal transcription factor complex helicase XPB sub
TGME49_261070	-3.15	6.54	2.29E-06	1.70E-05	apicoplast triosephosphate translocator APT1
TGME49_278850	-1.26	7.92	2.32E-06	1.71E-05	DHHC zinc finger domain-containing protein
TGME49_311150	-3.92	5.60	2.33E-06	1.72E-05	hypothetical protein
TGME49_313418	-8.87	5.19	2.36E-06	1.74E-05	hypothetical protein
TGME49_202680	-1.56	8.26	2.38E-06	1.75E-05	peptidase M16, alpha subunit, putative

TGME49_272320	1.74	5.67	2.40E-06	1.77E-05	DHHC zinc finger domain-containing protein
TGME49_252380	1.82	6.44	2.44E-06	1.79E-05	hypothetical protein
TGME49_285840	1.80	6.34	2.45E-06	1.80E-05	RAP domain-containing protein
TGME49_271780	-2.75	6.36	2.45E-06	1.80E-05	Filamin/ABP280 repeat-containing protein
TGME49_233790	-8.77	5.24	2.47E-06	1.81E-05	serine/threonine protein kinase AktR, putative
TGME49_222930	-2.32	6.77	2.48E-06	1.81E-05	hypothetical protein
TGME49_320450	-3.22	6.15	2.53E-06	1.84E-05	ribosome biogenesis regulatory protein (rrs1) protein
TGME49_259200	-3.90	6.05	2.53E-06	1.84E-05	Na+/H+ exchanger NHE1
TGME49_290580	-3.90	6.04	2.54E-06	1.85E-05	ATP-binding cassette G family transporter ABCG89
TGME49_282040	-3.95	5.99	2.58E-06	1.87E-05	hypothetical protein
TGME49_231215	-8.75	5.41	2.58E-06	1.88E-05	hypothetical protein
TGME49_220110	2.05	5.05	2.60E-06	1.89E-05	hypothetical protein
TGME49_202630	-1.38	7.37	2.62E-06	1.90E-05	ATP-dependent metallopeptidase HflB subfamily protein
TGME49_237530	-8.75	5.45	2.62E-06	1.90E-05	hypothetical protein
TGME49_293380	-8.83	5.33	2.64E-06	1.91E-05	histone lysine acetyltransferase HAT1
TGME49_306560	-3.30	5.99	2.65E-06	1.92E-05	hypothetical protein
TGME49_290980	-2.32	6.72	2.74E-06	1.98E-05	glycine C-acetyltransferase, putative
TGME49_269705	2.25	5.10	2.76E-06	1.99E-05	hypothetical protein
TGME49_316230	-2.47	6.16	2.77E-06	2.00E-05	SAC1 phosphoinositide phosphatase, putative
TGME49_311100	1.01	9.23	2.79E-06	2.01E-05	zinc finger (CCCH type) motif-containing protein
TGME49_278920	-3.20	5.87	2.81E-06	2.02E-05	hypothetical protein
TGME49_290000	-8.91	4.83	2.82E-06	2.02E-05	hypothetical protein
TGME49_267070	1.24	7.29	2.84E-06	2.04E-05	aquaporin 2
TGME49_262500	-1.65	7.01	2.86E-06	2.04E-05	hypothetical protein
TGME49_209440	-1.70	7.38	2.86E-06	2.04E-05	hypothetical protein
TGME49_260580	-2.15	6.60	2.86E-06	2.04E-05	hypothetical protein
TGME49_267600	-2.73	6.33	2.86E-06	2.04E-05	FHA domain-containing protein
TGME49_310860	-2.48	6.37	2.90E-06	2.07E-05	U5 snRNP-specific protein
TGME49_211410	-3.86	5.74	2.93E-06	2.09E-05	translation initiation factor sui1 protein
TGME49_267680	-1.27	7.49	3.03E-06	2.16E-05	microneme protein MIC12
TGME49_229380	-8.73	5.38	3.05E-06	2.17E-05	hypothetical protein
TGME49_244230	-8.73	5.32	3.11E-06	2.21E-05	hypothetical protein
TGME49_301380	-3.92	6.01	3.11E-06	2.21E-05	elongation factor Tu GTP binding domain-containing pro
TGME49_266400	-8.79	5.57	3.12E-06	2.21E-05	hypothetical protein
TGME49_224900	-1.32	7.72	3.15E-06	2.23E-05	adenylate kinase, putative
TGME49_255250	-8.73	5.45	3.15E-06	2.23E-05	tRNA (cytosine(34)-C(5))-methyltransferase, putative
TGME49_202030	-1.60	6.98	3.19E-06	2.25E-05	hypothetical protein
TGME49_220460	-8.72	4.98	3.18E-06	2.25E-05	SNF7 family protein
TGME49_271790	1.67	6.36	3.21E-06	2.26E-05	hypothetical protein
TGME49_281910	1.35	6.18	3.22E-06	2.27E-05	hypothetical protein
TGME49_242790	-1.81	6.69	3.22E-06	2.27E-05	trichohyalin, putative
TGME49_310270	-3.99	5.89	3.22E-06	2.27E-05	hypothetical protein
TGME49_203290	1.89	8.25	3.25E-06	2.28E-05	hypothetical protein
TGME49_239720	-8.71	5.49	3.26E-06	2.28E-05	50S ribosomal protein l24, putative
TGME49_249840	-8.71	5.34	3.28E-06	2.30E-05	dynein heavy chain 2, putative
TGME49_320740	-1.72	7.64	3.30E-06	2.31E-05	hypothetical protein
TGME49_309160	-2.28	6.33	3.32E-06	2.32E-05	IgA-specific metalloendopeptidase
TGME49_310320	-3.88	5.64	3.38E-06	2.36E-05	calreticulin family protein
TGME49_299150	-8.75	5.15	3.38E-06	2.36E-05	AP2 domain transcription factor AP2III-3
TGME49_270530	-2.87	5.98	3.42E-06	2.38E-05	ubiquitin fusion degradation protein UFD1CY
TGME49_230590	-8.73	5.22	3.43E-06	2.39E-05	chitobiosyldiphosphodolichol beta-mannosyltransferase
TGME49_211680	-1.71	11.04	3.45E-06	2.40E-05	protein disulfide isomerase
TGME49_292330	1.16	7.38	3.46E-06	2.40E-05	hypothetical protein
TGME49_246970	-8.71	5.55	3.46E-06	2.40E-05	3'-5' exonuclease domain-containing protein
TGME49_297510	-8.74	5.32	3.48E-06	2.41E-05	hypothetical protein
TGME49_210760	-8.72	5.47	3.49E-06	2.41E-05	glutamine amidotransferase-related, putative
TGME49_310010	-1.26	8.26	3.58E-06	2.48E-05	rhoptry neck protein RON1

TGME49_229030	-1.68	6.70	3.76E-06	2.59E-05	hypothetical protein
TGME49_228130	1.68	6.56	3.77E-06	2.60E-05	hypothetical protein
TGME49_281510	-3.11	6.07	3.77E-06	2.60E-05	ribonuclease H1 large subunit, putative
TGME49_219660	3.10	5.14	3.81E-06	2.63E-05	hypothetical protein
TGME49_232210	-8.74	5.03	3.85E-06	2.65E-05	hypothetical protein
TGME49_264780	-8.72	5.52	3.87E-06	2.66E-05	UTP-glucose-1-phosphate uridylyltransferase subfamily p
TGME49_315700	-3.13	5.81	3.89E-06	2.67E-05	hypothetical protein
TGME49_208730	1.55	8.92	3.91E-06	2.68E-05	microneme protein, putative
TGME49_247550	-1.04	8.65	3.91E-06	2.68E-05	heat shock protein HSP60
TGME49_253100	1.03	7.16	3.92E-06	2.68E-05	hypothetical protein
TGME49_319900	-1.86	7.18	3.94E-06	2.69E-05	hypothetical protein
TGME49_266810	-1.62	6.87	3.98E-06	2.72E-05	hypothetical protein
TGME49_270650	2.01	5.24	4.09E-06	2.79E-05	deoxyribose-phosphate aldolase
TGME49_239710	-8.73	5.20	4.11E-06	2.80E-05	phosphomannomutase
TGME49_304700	-3.83	5.84	4.12E-06	2.81E-05	hypothetical protein
TGME49_221500	-8.71	5.41	4.15E-06	2.83E-05	dual specificity phosphatase, catalytic domain-containing
TGME49_215610	-1.93	6.26	4.18E-06	2.84E-05	hypothetical protein
TGME49_246800	-1.73	6.81	4.25E-06	2.89E-05	acylaminoacyl-peptidase, putative
TGME49_211640	1.34	6.52	4.26E-06	2.89E-05	hypothetical protein
TGME49_312840	-2.14	6.43	4.27E-06	2.89E-05	hypothetical protein
TGME49_208200	-3.87	5.78	4.36E-06	2.95E-05	PHD-finger domain-containing protein
TGME49_231600	-2.49	6.14	4.41E-06	2.98E-05	HEAT repeat-containing protein
TGME49_279430	-2.75	6.25	4.41E-06	2.98E-05	cwf18 pre-mRNA splicing factor protein
TGME49_264130	1.50	5.62	4.44E-06	2.99E-05	hypothetical protein
TGME49_316350	-8.71	5.45	4.50E-06	3.04E-05	hypothetical protein
TGME49_267690	-3.85	6.16	4.58E-06	3.09E-05	hypothetical protein
TGME49_270140	-8.70	5.48	4.63E-06	3.11E-05	splicing factor DIM1, putative
TGME49_263040	-1.12	9.58	4.69E-06	3.15E-05	ribosomal protein RPS16
TGME49_283850	-1.94	8.26	4.73E-06	3.17E-05	peptidyl-prolyl cis-trans isomerase
TGME49_210682	3.05	5.90	4.76E-06	3.18E-05	hypothetical protein
TGME49_215070	1.23	6.64	4.78E-06	3.20E-05	ferodoxin FD
TGME49_213115	-8.68	4.95	4.79E-06	3.21E-05	hypothetical protein
TGME49_297420	-3.85	5.82	4.94E-06	3.29E-05	beta-tubulin cofactor D, putative
TGME49_294020	-8.71	5.25	5.03E-06	3.35E-05	zinc finger, C3HC4 type (RING finger) domain-containing
TGME49_205750	-2.43	6.42	5.09E-06	3.38E-05	histone deacetylase complex subunit Sin3
TGME49_248990	1.29	8.43	5.17E-06	3.43E-05	hypothetical protein
TGME49_310802	1.52	5.93	5.33E-06	3.53E-05	CRAL/TRIO domain-containing protein
TGME49_214960	1.13	7.09	5.40E-06	3.57E-05	AP2 domain transcription factor AP2X-8
TGME49_288010	2.59	5.10	5.46E-06	3.61E-05	hypothetical protein
TGME49_215060	-1.54	7.25	5.47E-06	3.62E-05	small GTP-binding protein sar1, putative
TGME49_318575	-8.69	5.22	5.50E-06	3.63E-05	hypothetical protein
TGME49_257340	-8.65	5.20	5.52E-06	3.64E-05	Ras family protein
TGME49_311890	-1.36	7.36	5.53E-06	3.65E-05	hypothetical protein
TGME49_257710	-3.82	5.56	5.56E-06	3.66E-05	actin-like protein ALP 5
TGME49_209210	-1.97	6.84	5.60E-06	3.68E-05	hypothetical protein
TGME49_217740	-1.92	6.97	5.74E-06	3.77E-05	3-ketoacyl-(acyl-carrier-protein) reductase
TGME49_205540	-8.65	5.64	5.79E-06	3.80E-05	DEAD/DEAH box helicase domain-containing protein
TGME49_314280	2.47	5.65	5.85E-06	3.84E-05	AAR2 protein
TGME49_310100	-8.67	5.39	5.86E-06	3.84E-05	mannosyltransferase (pig-m) protein
TGME49_254160	-3.79	4.76	5.89E-06	3.86E-05	hypothetical protein
TGME49_233410	1.48	5.64	5.89E-06	3.86E-05	Sof1 family domain-containing protein
TGME49_228980	-8.67	5.06	6.03E-06	3.94E-05	hypothetical protein
TGME49_228200	1.16	7.28	6.05E-06	3.95E-05	vacuolar (h+)-atpase g subunit protein
TGME49_249740	1.51	6.54	6.06E-06	3.95E-05	translation machinery associated tma7 protein
TGME49_262120	-1.51	7.00	6.10E-06	3.97E-05	IQ calmodulin-binding motif domain-containing protein
TGME49_215260	-1.52	7.22	6.11E-06	3.98E-05	carbamoylphosphate synthetase
TGME49_274130	-8.63	5.27	6.15E-06	4.00E-05	TBC domain-containing protein

TGME49_224580	-1.95	6.71	6.17E-06	4.01E-05	RNA recognition motif-containing protein
TGME49_271930	-1.52	8.59	6.21E-06	4.03E-05	hypothetical protein
TGME49_289670	2.35	4.94	6.22E-06	4.03E-05	DNA repair metallo-beta-lactamase
TGME49_275980	-3.79	5.59	6.24E-06	4.04E-05	coenzyme q (ubiquinone) biosynthesis protein coq4 prot
TGME49_318210	-8.65	5.53	6.24E-06	4.04E-05	hypothetical protein
TGME49_209070	1.56	7.33	6.28E-06	4.06E-05	hypothetical protein
TGME49_245710	-1.51	6.93	6.39E-06	4.13E-05	phosphatidylinositol-4-phosphate 5-kinase, putative
TGME49_262980	-8.65	5.23	6.42E-06	4.14E-05	hypothetical protein
TGME49_232360	-1.82	6.79	6.46E-06	4.17E-05	exonuclease
TGME49_233500	1.23	8.04	6.59E-06	4.24E-05	triose-phosphate isomerase TPI-II
TGME49_318170	1.50	6.11	6.65E-06	4.28E-05	hypothetical protein
TGME49_220175	2.87	4.77	6.66E-06	4.28E-05	hypothetical protein
TGME49_323110	1.42	6.09	6.67E-06	4.28E-05	hypothetical protein
TGME49_258480	-1.89	6.81	6.88E-06	4.41E-05	hypothetical protein
TGME49_249820	-3.04	5.81	6.92E-06	4.43E-05	ATP-binding cassette sub-family B member 5
TGME49_306310	-2.25	6.64	6.96E-06	4.45E-05	RecF/RecN/SMC N terminal domain-containing protein
TGME49_204400	-1.50	8.95	7.09E-06	4.52E-05	ATPase synthase subunit alpha, putative
TGME49_279540	-8.72	4.99	7.11E-06	4.54E-05	hypothetical protein
TGME49_313690	-1.69	6.61	7.19E-06	4.58E-05	Sel1 repeat-containing protein
TGME49_297230	-2.06	6.39	7.23E-06	4.60E-05	Vps53 family, N-terminal protein
TGME49_311250	-3.17	6.06	7.24E-06	4.61E-05	hypothetical protein
TGME49_284780	-1.33	7.47	7.27E-06	4.62E-05	hypothetical protein
TGME49_216680	-2.46	7.27	7.60E-06	4.82E-05	ankyrin repeat-containing protein
TGME49_281570	-3.77	5.67	7.60E-06	4.82E-05	hypothetical protein
TGME49_265220	-3.83	5.88	7.67E-06	4.87E-05	co-chaperone GrpE protein
TGME49_273370	-2.82	6.35	7.76E-06	4.92E-05	coatomer gamma 2-subunit protein, putative
TGME49_275310	-3.07	5.96	7.84E-06	4.96E-05	hypothetical protein
TGME49_215430	1.72	7.91	7.86E-06	4.97E-05	hypothetical protein
TGME49_262060	-3.03	5.97	7.99E-06	5.05E-05	hypothetical protein
TGME49_263610	-1.78	6.91	8.01E-06	5.05E-05	hypothetical protein
TGME49_261600	1.08	7.25	8.05E-06	5.08E-05	creatinase domain-containing protein
TGME49_310850	-8.68	5.31	8.08E-06	5.09E-05	MYND finger domain-containing protein
TGME49_251440	-8.69	5.79	8.11E-06	5.10E-05	troponin c, isotype gamma, putative
TGME49_221690	-3.80	5.62	8.17E-06	5.13E-05	hypothetical protein
TGME49_289510	1.58	5.65	8.22E-06	5.16E-05	hypothetical protein
TGME49_233820	-8.59	4.93	8.26E-06	5.18E-05	DNA polymerase epsilon subunit B protein
TGME49_260620	1.54	8.06	8.33E-06	5.22E-05	hypothetical protein
TGME49_286800	-8.64	5.19	8.63E-06	5.40E-05	hypothetical protein
TGME49_229500	-3.75	5.70	8.65E-06	5.41E-05	hypothetical protein
TGME49_262950	1.05	7.72	8.94E-06	5.58E-05	hypothetical protein
TGME49_278660	-1.24	8.03	8.99E-06	5.60E-05	P-type ATPase4, putative
TGME49_218955	1.82	5.19	9.07E-06	5.65E-05	hypothetical protein
TGME49_253160	1.30	6.54	9.08E-06	5.65E-05	hypothetical protein
TGME49_228490	-2.64	6.14	9.10E-06	5.65E-05	hypothetical protein
TGME49_290940	-1.48	7.21	9.46E-06	5.87E-05	EMP/nonaspanin domain family protein
TGME49_313270	-1.44	7.49	9.58E-06	5.94E-05	hypothetical protein
TGME49_225910	1.48	5.95	9.64E-06	5.97E-05	hypothetical protein
TGME49_261710	-1.75	6.65	9.65E-06	5.98E-05	ankyrin repeat-containing protein
TGME49_238410	-3.02	5.77	9.81E-06	6.07E-05	hypothetical protein
TGME49_225800	-3.04	6.11	1.00E-05	6.18E-05	iron-sulfur assembly ATPase
TGME49_237890	-3.76	6.17	1.01E-05	6.23E-05	calcium-dependent protein kinase CDPK4
TGME49_220870	1.59	5.64	1.02E-05	6.25E-05	hypothetical protein
TGME49_264960	-8.59	5.17	1.02E-05	6.26E-05	hypothetical protein
TGME49_232960	-1.86	6.61	1.03E-05	6.34E-05	oxidoreductase, 2OG-Fe(II) oxygenase family protein
TGME49_223510	-8.57	5.27	1.04E-05	6.41E-05	hypothetical protein
TGME49_270230	-8.58	5.30	1.05E-05	6.41E-05	hypothetical protein
TGME49_212960	-8.61	5.30	1.05E-05	6.42E-05	hypothetical protein

TGME49_291010	2.29	5.14	1.08E-05	6.62E-05	hypothetical protein
TGME49_294740	-2.06	6.49	1.09E-05	6.65E-05	hypothetical protein
TGME49_295420	1.39	7.87	1.10E-05	6.71E-05	hypothetical protein
TGME49_209820	-8.59	5.29	1.10E-05	6.72E-05	syntaxin protein
TGME49_288710	1.81	5.08	1.11E-05	6.75E-05	hypothetical protein
TGME49_201900	-3.75	5.71	1.11E-05	6.75E-05	hypothetical protein
TGME49_268220	6.66	5.12	1.11E-05	6.77E-05	hypothetical protein
TGME49_310810	-2.03	6.36	1.13E-05	6.86E-05	apyrase
TGME49_227600	-1.37	9.30	1.13E-05	6.87E-05	ribosomal protein RPL34
TGME49_213880	2.68	4.99	1.14E-05	6.90E-05	hypothetical protein
TGME49_273815	-8.62	5.51	1.14E-05	6.90E-05	hypothetical protein
TGME49_238230	-3.04	5.73	1.15E-05	6.95E-05	Ser/Thr phosphatase family protein
TGME49_321590	-1.41	7.17	1.16E-05	7.01E-05	hypothetical protein
TGME49_226270	-3.07	5.82	1.17E-05	7.07E-05	hypothetical protein
TGME49_310710	-3.72	5.64	1.18E-05	7.13E-05	small ribosomal subunit Rsm22 protein
TGME49_277010	-8.62	4.90	1.18E-05	7.14E-05	Fe-S metabolism associated domain-containing protein
TGME49_201870	-2.21	6.58	1.18E-05	7.14E-05	tetratricopeptide repeat-containing protein
TGME49_249310	-3.77	5.20	1.19E-05	7.18E-05	hypothetical protein
TGME49_204080	-1.68	6.87	1.20E-05	7.19E-05	histidine acid phosphatase superfamily protein
TGME49_225990	-2.62	6.32	1.20E-05	7.19E-05	acyl transferase domain-containing protein
TGME49_251670	-3.72	5.58	1.20E-05	7.19E-05	werner helicase interacting protein 1, putative
TGME49_274150	-3.79	5.45	1.20E-05	7.19E-05	hypothetical protein
TGME49_232320	-8.59	4.97	1.20E-05	7.19E-05	hypothetical protein
TGME49_253640	1.08	6.73	1.20E-05	7.20E-05	hypothetical protein
TGME49_213240	-8.59	5.32	1.23E-05	7.40E-05	hypothetical protein
TGME49_236210	-1.08	8.23	1.24E-05	7.42E-05	peptidase M16 family potein, putative
TGME49_251410	-1.96	6.67	1.24E-05	7.44E-05	tetratricopeptide repeat-containing protein
TGME49_235150	-1.01	8.45	1.25E-05	7.46E-05	transporter, major facilitator family protein
TGME49_215570	-3.01	6.18	1.25E-05	7.46E-05	AP2 domain transcription factor AP2X-11
TGME49_283830	2.46	5.33	1.26E-05	7.49E-05	type I inorganic pyrophosphatase PPase
TGME49_216120	-2.18	6.42	1.26E-05	7.49E-05	hypothetical protein
TGME49_263150	-8.57	4.96	1.26E-05	7.51E-05	tetratricopeptide repeat-containing protein
TGME49_306520	1.58	7.15	1.27E-05	7.57E-05	tRNA pseudouridine synthase B, putative
TGME49_214770	-1.16	7.90	1.27E-05	7.57E-05	small GTP binding protein rab1a, putative
TGME49_290340	-2.42	6.17	1.27E-05	7.57E-05	HEAT repeat-containing protein
TGME49_221170	-1.40	6.99	1.28E-05	7.60E-05	CAAX metallo endopeptidase
TGME49_269930	2.31	6.51	1.29E-05	7.66E-05	calcium binding egf domain-containing protein
TGME49_264730	-8.53	5.36	1.29E-05	7.66E-05	hypothetical protein
TGME49_246570	1.56	5.60	1.30E-05	7.68E-05	hypothetical protein
TGME49_310490	-1.20	9.63	1.30E-05	7.68E-05	ribosomal protein RPL27A
TGME49_252490	-2.36	5.95	1.30E-05	7.68E-05	vacuolar protein sorting 29, putative
TGME49_257960	-3.03	5.69	1.30E-05	7.68E-05	GDP-D-mannose pyrophosphorylase
TGME49_215040	-2.05	6.64	1.32E-05	7.79E-05	HEAT repeat-containing protein
TGME49_230050	1.85	4.96	1.33E-05	7.83E-05	50S ribosomal protein L3, putative
TGME49_310560	-3.74	5.55	1.34E-05	7.88E-05	hypothetical protein
TGME49_272475	-8.57	5.24	1.34E-05	7.88E-05	protein kinase domain-containing protein
TGME49_294830	-2.72	6.45	1.37E-05	8.03E-05	methyltransferase domain-containing protein
TGME49_307040	-1.59	6.89	1.38E-05	8.09E-05	shikimate dehydrogenase substrate binding domain-con
TGME49_248790	1.96	5.02	1.38E-05	8.09E-05	hypothetical protein
TGME49_296340	3.29	5.35	1.39E-05	8.12E-05	hypothetical protein
TGME49_270720	-8.70	5.34	1.39E-05	8.13E-05	hypothetical protein
TGME49_210390	-8.60	5.21	1.40E-05	8.20E-05	WD domain, G-beta repeat-containing protein
TGME49_253410	2.32	6.01	1.40E-05	8.20E-05	hypothetical protein
TGME49_221460	-1.54	6.68	1.41E-05	8.22E-05	phosphoglycerate mutase family protein
TGME49_266970	-3.02	5.89	1.42E-05	8.30E-05	hypothetical protein
TGME49_253490	2.08	7.37	1.43E-05	8.32E-05	hypothetical protein
TGME49_284660	-8.53	5.27	1.44E-05	8.38E-05	mitochondrial ribosomal protein s6-2, putative

TGME49_223800	-3.73	5.96	1.47E-05	8.52E-05	hypothetical protein
TGME49_246060	-8.64	5.11	1.47E-05	8.52E-05	DNA-dependent RNA polymerase
TGME49_285780	-8.59	5.31	1.48E-05	8.56E-05	hypothetical protein
TGME49_304710	-1.16	7.77	1.48E-05	8.57E-05	eukaryotic peptide chain release factor, putative
TGME49_221260	1.36	6.11	1.48E-05	8.59E-05	Class-II DAHP synthetase family protein
TGME49_248570	-3.77	5.72	1.49E-05	8.65E-05	hypothetical protein
TGME49_203180	-3.69	5.86	1.51E-05	8.75E-05	leucine rich repeat-containing protein
TGME49_288260	-1.70	6.74	1.52E-05	8.78E-05	hypothetical protein
TGME49_283800	-2.23	6.25	1.53E-05	8.85E-05	hypothetical protein
TGME49_288560	-1.87	6.49	1.55E-05	8.92E-05	hypothetical protein
TGME49_228030	2.04	4.77	1.57E-05	9.01E-05	hypothetical protein
TGME49_247240	-1.96	6.78	1.57E-05	9.02E-05	ubiquitin carboxyl-terminal hydrolase, family 1 protein
TGME49_234670	1.81	5.29	1.57E-05	9.03E-05	actin-like family protein
TGME49_283540	1.14	8.26	1.57E-05	9.04E-05	hypothetical protein
TGME49_217750	-3.69	5.56	1.60E-05	9.18E-05	hypothetical protein
TGME49_313240	-3.07	5.84	1.60E-05	9.18E-05	ethylene-responsive RNA helicase, putative
TGME49_224230	1.97	5.03	1.61E-05	9.19E-05	AP2 domain transcription factor AP2X-3
TGME49_268600	-2.96	5.84	1.63E-05	9.33E-05	DNA polymerase epsilon subunit B protein
TGME49_254910	-2.61	6.12	1.66E-05	9.45E-05	hypothetical protein
TGME49_208050	-2.66	5.98	1.67E-05	9.55E-05	ABC transporter, putative
TGME49_233090	-8.51	4.99	1.68E-05	9.56E-05	XPG N-terminal domain-containing protein
TGME49_266900	1.49	7.86	1.69E-05	9.60E-05	cyclin, N-terminal domain-containing protein
TGME49_203450	-2.19	6.40	1.69E-05	9.60E-05	DUF3228 domain-containing protein
TGME49_244000	-8.53	5.35	1.69E-05	9.61E-05	DEAD/DEAH box helicase domain-containing protein
TGME49_270150	-2.98	5.78	1.70E-05	9.62E-05	hypothetical protein
TGME49_315690	-3.70	5.51	1.70E-05	9.62E-05	DnaJ domain-containing protein
TGME49_312860	-3.01	5.92	1.71E-05	9.68E-05	hypothetical protein
TGME49_305330	-1.02	9.74	1.74E-05	9.83E-05	cyclin, N-terminal domain-containing protein
TGME49_271060	-3.68	5.87	1.75E-05	9.89E-05	Sec1 family protein
TGME49_321440	-1.18	7.84	1.77E-05	9.99E-05	SWI2/SNF2 ISWI-like SANT
TGME49_253510	2.46	6.34	1.79E-05	0.000100768	transporter/permease protein
TGME49_299780	1.14	9.06	1.81E-05	0.000102175	hypothetical protein
TGME49_314460	-8.49	4.78	1.82E-05	0.000102317	hypothetical protein
TGME49_305850	-1.10	7.71	1.82E-05	0.000102448	RNA recognition motif-containing protein
TGME49_294390	-8.48	5.02	1.83E-05	0.000102894	myosin light chain MLC4, putative
TGME49_222370	1.49	7.08	1.84E-05	0.000103592	SAG-related sequence SRS13
TGME49_261570	-1.13	10.08	1.85E-05	0.000103706	ribosomal protein RPL7A
TGME49_273850	-2.95	5.77	1.86E-05	0.000104033	hypothetical protein
TGME49_310380	-8.50	5.10	1.86E-05	0.000104033	brix domain containing protein
TGME49_216530	-8.54	4.90	1.86E-05	0.000104226	ribosome recycling factor protein
TGME49_203830	-2.97	6.38	1.86E-05	0.000104245	FHA domain-containing protein
TGME49_285990	-3.77	5.64	1.86E-05	0.000104284	Filamin/ABP280 repeat-containing protein
TGME49_209030	-1.03	11.56	1.88E-05	0.000104814	actin ACT1
TGME49_300260	-1.11	8.27	1.88E-05	0.000104814	threonyl-tRNA synthetase family protein
TGME49_221585	-8.53	5.10	1.90E-05	0.000106202	hypothetical protein
TGME49_226072	-1.13	7.64	1.92E-05	0.000107183	Ser/Thr phosphatase family protein
TGME49_312280	-1.54	6.89	1.92E-05	0.000107183	pre-mRNA-splicing factor ATP-dependent RNA helicase,
TGME49_283810	-3.72	5.42	1.93E-05	0.000107198	hypothetical protein
TGME49_312530	1.08	6.83	1.93E-05	0.000107401	splicing factor, CC1 family protein
TGME49_205160	1.90	5.28	1.93E-05	0.000107449	hypothetical protein
TGME49_221440	-8.53	5.45	1.93E-05	0.000107449	RPGR, putative
TGME49_295040	-1.24	7.94	1.95E-05	0.000108114	HEAT repeat-containing protein
TGME49_253180	1.95	7.48	1.95E-05	0.000108185	hypothetical protein
TGME49_309800	-1.60	6.85	1.95E-05	0.000108185	RNA recognition motif-containing protein
TGME49_224935	1.43	6.20	1.96E-05	0.000108278	hypothetical protein
TGME49_233680	-8.47	5.02	1.96E-05	0.000108278	nuclear movement family protein
TGME49_218250	-2.35	6.10	1.96E-05	0.000108474	TAP42 family protein

TGME49_226560	-3.67	5.49	1.97E-05	0.000108814	zinc finger (CCCH type) motif-containing protein
TGME49_247700	1.32	8.30	1.97E-05	0.000108939	AP2 domain transcription factor AP2XII-4
TGME49_301130	-8.47	5.01	1.97E-05	0.000108939	hypothetical protein
TGME49_297890	1.19	6.67	1.98E-05	0.000109207	hypothetical protein
TGME49_284540	-1.31	7.47	1.99E-05	0.000109645	ATP synthase F1, delta subunit protein
TGME49_269390	1.20	6.73	2.00E-05	0.000109902	CRAL/TRIO domain-containing protein
TGME49_272770	-3.67	5.80	2.00E-05	0.000110075	hypothetical protein
TGME49_318260	-3.67	5.56	2.02E-05	0.000110815	transcription initiation factor TFIID subunit TAF5
TGME49_213370	1.33	6.46	2.02E-05	0.000110945	formin FRM3
TGME49_286750	-1.34	7.53	2.04E-05	0.000112	MA3 domain-containing protein
TGME49_312210	-2.95	5.52	2.04E-05	0.000112074	hypothetical protein
TGME49_306290	-2.04	6.40	2.06E-05	0.000112971	DNA-directed RNA polymerase III RPC1
TGME49_311470	-1.07	9.92	2.08E-05	0.000113753	rhoptry neck protein RON5
TGME49_223668	1.96	5.19	2.08E-05	0.000113872	LYAR-type C2HC zinc finger protein
TGME49_219310	-1.31	8.62	2.11E-05	0.000115315	DnaK family protein
TGME49_314890	-3.65	5.41	2.11E-05	0.00011534	ThiF family protein
TGME49_228230	1.30	9.12	2.14E-05	0.000116509	hypothetical protein
TGME49_223780	-1.76	6.75	2.15E-05	0.000117035	hypothetical protein
TGME49_249460	-8.46	5.36	2.17E-05	0.000118139	WD domain, G-beta repeat-containing protein
TGME49_280720	-8.46	5.01	2.17E-05	0.000118303	hypothetical protein
TGME49_309950	-8.45	5.31	2.18E-05	0.000118429	NLE (NUC135) domain-containing protein
TGME49_240890	-1.27	7.45	2.18E-05	0.000118434	6-phosphofructokinase
TGME49_299900	-8.45	5.26	2.19E-05	0.000118755	hypothetical protein
TGME49_312340	-8.46	5.02	2.20E-05	0.000119496	hypothetical protein
TGME49_243290	-2.13	6.21	2.20E-05	0.000119578	hypothetical protein
TGME49_219770	-8.54	5.44	2.21E-05	0.000119619	30S ribosomal protein S12, putative
TGME49_297460	-8.51	5.02	2.23E-05	0.000120667	hypothetical protein
TGME49_294250	-2.11	6.17	2.26E-05	0.000122221	WD domain, G-beta repeat-containing protein
TGME49_249560	-8.49	5.35	2.26E-05	0.000122376	DNA-directed RNA polymerase alpha chain rpoA
TGME49_288900	1.82	5.12	2.27E-05	0.00012258	Yos1 family protein
TGME49_270900	-3.05	6.04	2.27E-05	0.00012258	ATPase, AAA family protein
TGME49_210778	-1.97	6.36	2.28E-05	0.000123295	hemimethylated DNA binding domain-containing protei
TGME49_229350	-1.98	6.02	2.29E-05	0.000123378	HEAT repeat-containing protein
TGME49_264040	-1.19	7.86	2.31E-05	0.000124258	hypothetical protein
TGME49_254410	1.12	6.78	2.31E-05	0.000124575	protein phosphatase 2C, putative
TGME49_216240	-1.96	6.30	2.33E-05	0.000125462	hypothetical protein
TGME49_258680	-2.11	6.21	2.35E-05	0.000126361	TATA-box binding protein TBP2
TGME49_228730	-8.45	5.41	2.37E-05	0.000127174	hypothetical protein
TGME49_216410	-8.55	5.36	2.45E-05	0.000131168	hypothetical protein
TGME49_311680	-1.45	7.31	2.45E-05	0.000131414	FUN14 family protein
TGME49_230860	-2.33	6.09	2.45E-05	0.000131414	hypothetical protein
TGME49_289680	-1.06	7.66	2.50E-05	0.000133692	Ras-related protein Rab11
TGME49_308880	-8.45	5.26	2.53E-05	0.000135149	ImpB/MucB/SamB family protein
TGME49_246230	-8.49	5.20	2.53E-05	0.000135149	hypothetical protein
TGME49_261000	-1.53	7.03	2.55E-05	0.000135991	MutS domain V domain-containing protein
TGME49_297900	1.37	7.58	2.56E-05	0.000136164	hypothetical protein
TGME49_236800	-8.45	5.06	2.56E-05	0.000136164	hypothetical protein
TGME49_269150	-8.45	5.14	2.56E-05	0.000136164	DHHC zinc finger domain-containing protein
TGME49_213400	-2.32	6.41	2.61E-05	0.000138596	zinc finger (CCCH type) motif-containing protein
TGME49_259950	2.05	7.95	2.62E-05	0.000139061	carbonate dehydratase, eukaryotic-type domain-contain
TGME49_314070	1.30	6.32	2.63E-05	0.000139548	hypothetical protein
TGME49_273920	-2.55	6.00	2.64E-05	0.000139904	aldose reductase, putative
TGME49_201150	-1.77	6.72	2.65E-05	0.000140261	heavy metal translocating P-type ATPase subfamily prote
TGME49_203720	1.89	8.00	2.68E-05	0.000141981	vitamin k epoxide reductase family protein
TGME49_297140	-2.91	5.78	2.70E-05	0.000142776	U6 snRNA-associated sm family protein Lsm2, putative
TGME49_264860	-3.63	5.69	2.71E-05	0.000143204	zinc finger, C3HC4 type (RING finger) domain-containing
TGME49_305030	-2.10	5.98	2.71E-05	0.000143236	kinase, pfkB family protein

TGME49_254380	-8.45	5.12	2.72E-05	0.000143611	ribosomal protein L11, putative
TGME49_305920	-3.62	5.44	2.73E-05	0.000143825	endonuclease III family 1 protein
TGME49_211340	1.29	6.69	2.75E-05	0.00014474	hypothetical protein
TGME49_316200	1.12	6.43	2.76E-05	0.000145164	phosphoglycerate mutase family protein
TGME49_268880	-2.91	6.09	2.78E-05	0.000146036	hypothetical protein
TGME49_321410	1.14	7.54	2.78E-05	0.000146042	hypothetical protein
TGME49_236860	-3.69	6.13	2.80E-05	0.000146746	haloacid dehalogenase family hydrolase domain-contain
TGME49_264720	-3.61	5.63	2.82E-05	0.000147917	hypothetical protein
TGME49_293330	-8.42	5.03	2.89E-05	0.00015113	hypothetical protein
TGME49_256000	-1.01	8.30	2.90E-05	0.000151757	endoplasmic reticulum retention receptor
TGME49_235750	-8.43	5.02	2.90E-05	0.000151779	ULK kinase
TGME49_320750	1.40	5.85	2.93E-05	0.000152887	hypothetical protein
TGME49_248630	-1.79	6.51	2.94E-05	0.000153578	actin-related protein ARP1
TGME49_234390	-8.45	5.43	2.95E-05	0.000153578	hypothetical protein
TGME49_299200	1.21	7.09	2.98E-05	0.000155052	Bet3 transport protein, putative
TGME49_251885	-8.45	5.07	2.99E-05	0.000155555	hypothetical protein
TGME49_253615	1.20	6.39	3.01E-05	0.000156492	hypothetical protein
TGME49_288930	-2.33	6.16	3.04E-05	0.000158209	hypothetical protein
TGME49_300285	-8.56	5.23	3.09E-05	0.000160662	hypothetical protein
TGME49_232020	-1.92	6.60	3.14E-05	0.000162595	hypothetical protein
TGME49_224110	-1.33	6.80	3.17E-05	0.000164218	adhesion regulating molecule region protein, putative
TGME49_226280	1.85	5.02	3.19E-05	0.000165158	ribosomal protein L28, putative
TGME49_226590	1.17	9.66	3.21E-05	0.000166111	cytochrome C oxidase subunit IIa, putative
TGME49_263210	-8.42	5.06	3.26E-05	0.000168644	ubiquitin interaction motif domain-containing protein
TGME49_285210	-8.47	5.26	3.29E-05	0.000169622	hypothetical protein
TGME49_228630	-1.36	7.14	3.30E-05	0.00017007	hypothetical protein
TGME49_291090	-8.45	5.22	3.30E-05	0.00017007	SWI2/SNF2-containing protein
TGME49_319570	-1.90	6.69	3.31E-05	0.000170313	WD domain, G-beta repeat-containing protein
TGME49_291030	-3.67	5.60	3.41E-05	0.000175387	zinc finger, C3HC4 type (RING finger) domain-containing
TGME49_308940	-8.39	4.89	3.42E-05	0.00017559	hypothetical protein
TGME49_308093	1.13	7.72	3.48E-05	0.000178693	rhoptry kinase family protein (incomplete catalytic triad)
TGME49_259850	-8.38	5.08	3.50E-05	0.000179573	hypothetical protein
TGME49_240910	-1.84	6.32	3.51E-05	0.00017989	hypothetical protein
TGME49_239130	1.43	5.73	3.56E-05	0.000182233	Tyrosine kinase-like (TKL) protein
TGME49_209860	-8.38	5.15	3.57E-05	0.000182766	SRP40, C-terminal domain-containing protein
TGME49_206600	-8.37	5.40	3.60E-05	0.000183821	sigma-70, region 3 protein
TGME49_207180	-8.43	5.29	3.64E-05	0.000185678	indole-3-glycerol phosphate synthase domain-containing
TGME49_227115	-8.38	5.08	3.65E-05	0.000186322	hypothetical protein
TGME49_308580	-2.51	5.95	3.69E-05	0.000188101	Lon protease family protein
TGME49_259210	-8.38	5.17	3.73E-05	0.000190304	jmjC domain-containing protein C2orf60
TGME49_268170	-8.46	5.25	3.75E-05	0.000190752	hypothetical protein
TGME49_314660	-1.38	6.48	3.76E-05	0.000191487	TPRX1 protein
TGME49_266800	-1.94	6.24	3.77E-05	0.000191487	integral membrane protein, putative
TGME49_222020	-2.29	6.35	3.86E-05	0.000196109	phosphoglycerate kinase PGKII
TGME49_213480	-2.18	5.67	3.87E-05	0.000196618	hypothetical protein
TGME49_269320	-8.43	5.04	3.87E-05	0.000196618	hypothetical protein
TGME49_208710	1.64	5.63	3.93E-05	0.000199279	DNA/RNA non-specific endonuclease
TGME49_260510	-2.86	5.73	3.94E-05	0.000199519	ubiquitin thioesterase otubain-like family protein
TGME49_297790	-2.08	6.27	4.01E-05	0.000203034	hypothetical protein
TGME49_315600	-8.42	5.50	4.03E-05	0.000203956	MCM2/3/5 family protein
TGME49_223130	1.81	5.12	4.08E-05	0.000206348	hypothetical protein
TGME49_295590	1.89	4.96	4.13E-05	0.000208402	hypothetical protein
TGME49_244720	-3.58	5.51	4.18E-05	0.000210906	hypothetical protein
TGME49_227310	-3.61	5.53	4.22E-05	0.00021262	hypothetical protein
TGME49_243340	-8.35	5.12	4.23E-05	0.000212906	atypical MEK-related kinase (incomplete catalytic triad)
TGME49_212090	1.09	6.61	4.24E-05	0.000213234	hypothetical protein
TGME49_267140	-8.43	4.84	4.25E-05	0.000213234	SAG-related sequence SRS38B

TGME49_260160	2.93	4.70	4.28E-05	0.000214709	hypothetical protein
TGME49_277540	-8.37	4.89	4.29E-05	0.000215336	hypothetical protein
TGME49_223450	-1.66	6.86	4.37E-05	0.000219167	ubiquitin carboxyl-terminal hydrolase
TGME49_275330	-3.59	5.64	4.38E-05	0.000219428	ribosomal protein RPL29
TGME49_299110	-2.10	6.49	4.40E-05	0.000220231	cleft lip and palate transmembrane protein 1 (clptm1) pr
TGME49_313385	1.79	5.03	4.44E-05	0.000221856	hypothetical protein
TGME49_289000	-2.86	5.79	4.46E-05	0.000222826	hypothetical protein
TGME49_287270	-2.49	6.15	4.49E-05	0.000224204	hypothetical protein
TGME49_205170	1.23	8.72	4.56E-05	0.000227273	hypothetical protein
TGME49_257990	-1.53	6.77	4.57E-05	0.000227347	heat shock protein 101, putative
TGME49_312380	-8.34	4.96	4.57E-05	0.000227347	tetratricopeptide repeat-containing protein
TGME49_278470	-3.72	5.56	4.60E-05	0.000228802	hypothetical protein
TGME49_306210	-8.50	5.06	4.66E-05	0.000231222	RNA polymerase II accessory factor CDC73
TGME49_297720	-1.12	7.54	4.73E-05	0.0002344	trehalose-phosphatase
TGME49_289150	1.12	6.22	4.74E-05	0.000234995	hypothetical protein
TGME49_309050	-8.38	4.88	4.75E-05	0.000235139	hypothetical protein
TGME49_229340	-8.33	4.88	4.78E-05	0.000236174	hypothetical protein
TGME49_252400	2.94	5.14	4.80E-05	0.000236912	HIT zinc finger protein
TGME49_250880	-1.64	7.90	4.80E-05	0.000236912	kinase, pfkB family protein
TGME49_269250	-1.29	7.17	4.84E-05	0.00023882	Mov34/MPN/PAD-1 family protein
TGME49_268720	-2.88	5.82	4.88E-05	0.000240507	Hrf1 family protein
TGME49_300010	-8.38	4.88	4.90E-05	0.000241502	hypothetical protein
TGME49_246190	-2.48	6.02	4.93E-05	0.000243042	hypothetical protein
TGME49_239610	-3.56	5.48	4.95E-05	0.000243551	hypothetical protein
TGME49_203390	-1.41	7.14	4.97E-05	0.000244435	CRAL/TRIO domain-containing protein
TGME49_289180	-8.35	5.18	4.98E-05	0.00024457	thioredoxin family redox-active protein, putative
TGME49_205690	1.07	6.66	4.99E-05	0.000244855	hypothetical protein
TGME49_233300	-2.85	5.35	5.06E-05	0.000248176	RhoGAP domain-containing protein
TGME49_277070	1.37	5.84	5.07E-05	0.000248828	SWI2/SNF2-containing protein
TGME49_240300	-8.36	5.18	5.09E-05	0.000249315	zinc finger domain, LSD1 subclass domain-containing pro
TGME49_258625	-8.44	4.74	5.09E-05	0.0002494	peptidyl-prolyl cis-trans isomerase, FKBP-type domain-co
TGME49_249600	-8.35	4.74	5.16E-05	0.000252435	hypothetical protein
TGME49_251790	1.27	6.44	5.20E-05	0.000254231	hypothetical protein
TGME49_240900	1.11	6.85	5.21E-05	0.000254421	AP2 domain transcription factor AP2VI-2
TGME49_259830	1.49	6.46	5.31E-05	0.000259235	diacylglycerol kinase catalytic domain-containing protein
TGME49_278130	-2.05	6.84	5.33E-05	0.000259735	hypothetical protein
TGME49_215775	-1.13	10.26	5.35E-05	0.000260806	rhoptry protein ROP8
TGME49_313360	1.20	6.20	5.44E-05	0.000264592	hypothetical protein
TGME49_239700	-2.87	5.88	5.44E-05	0.000264592	regulator of chromosome condensation (RCC1) repeat-co
TGME49_235460	1.19	6.18	5.54E-05	0.00026941	hypothetical protein
TGME49_267560	1.89	5.89	5.55E-05	0.000269577	folate-binding protein YgfZ protein
TGME49_231640	-1.24	10.99	5.62E-05	0.000272847	alveolin domain containing intermediate filament IMC1
TGME49_222060	-2.32	5.98	5.65E-05	0.000273996	hypothetical protein
TGME49_269075	-8.49	5.19	5.82E-05	0.000281612	hypothetical protein
TGME49_249890	1.71	5.11	5.83E-05	0.00028193	hypothetical protein
TGME49_277870	-2.06	6.10	5.89E-05	0.000284864	hypothetical protein
TGME49_280522	-1.47	6.97	5.92E-05	0.000285703	hypothetical protein
TGME49_202750	-8.29	5.03	5.92E-05	0.000285703	3' exoribonuclease family, domain 1 domain-containing p
TGME49_294705	-8.35	5.03	5.92E-05	0.000285703	hypothetical protein
TGME49_242845	-3.56	5.19	5.94E-05	0.000286428	hypothetical protein
TGME49_233130	1.32	6.19	5.99E-05	0.000288705	nucleoside transporter protein
TGME49_280410	-1.69	6.69	6.06E-05	0.000291559	3'5'-cyclic nucleotide phosphodiesterase domain-contair
TGME49_212920	1.53	5.72	6.08E-05	0.000292344	hypothetical protein
TGME49_223150	-8.29	5.15	6.08E-05	0.000292344	START domain-containing protein
TGME49_212270	1.53	9.40	6.13E-05	0.000294284	hypothetical protein
TGME49_219810	1.25	8.82	6.13E-05	0.000294284	hypothetical protein
TGME49_268210	-8.82	5.75	6.15E-05	0.000295222	AGC kinase

TGME49_233490	-8.38	5.10	6.21E-05	0.000297476	hypothetical protein
TGME49_226660	-1.34	7.06	6.23E-05	0.000298276	hypothetical protein
TGME49_306430	-1.32	6.93	6.27E-05	0.000299915	hypothetical protein
TGME49_200430	-8.30	4.89	6.30E-05	0.000301338	cytidine and deoxycytidylate deaminase zinc-binding reg
TGME49_272680	-8.28	5.08	6.31E-05	0.00030149	hypothetical protein
TGME49_312430	1.25	6.02	6.34E-05	0.000302673	hypothetical protein
TGME49_216390	-2.81	5.89	6.37E-05	0.000303563	RNA methyltransferase, TrmH family protein
TGME49_254606	2.32	5.92	6.37E-05	0.000303593	hypothetical protein
TGME49_213600	-8.28	5.17	6.53E-05	0.000310903	hypothetical protein
TGME49_218280	-1.18	7.67	6.58E-05	0.000312588	eukaryotic porin, putative
TGME49_221350	-3.51	5.68	6.59E-05	0.000313054	Ctr copper transporter family protein
TGME49_239320	-8.28	4.74	6.62E-05	0.000314061	BolA family protein
TGME49_304470	-2.80	5.83	6.63E-05	0.000314213	oxidoreductase, putative
TGME49_248700	-1.40	9.62	6.64E-05	0.000314701	alveolin domain containing intermediate filament IMC12
TGME49_213590	-1.33	6.96	6.66E-05	0.000315256	hypothetical protein
TGME49_233340	-2.23	6.12	6.67E-05	0.000315479	hypothetical protein
TGME49_277760	-1.28	6.96	6.70E-05	0.000316635	adenylosuccinate lyase, putative
TGME49_218370	-2.20	5.98	6.75E-05	0.000318548	hypothetical protein
TGME49_231180	1.22	6.18	6.79E-05	0.000320494	hypothetical protein
TGME49_272670	-8.28	4.99	6.87E-05	0.000323863	peptidase family M3 protein
TGME49_246040	-1.87	6.20	6.89E-05	0.000324437	MIF4G domain-containing protein
TGME49_257370	2.16	5.96	6.92E-05	0.000325525	hypothetical protein
TGME49_265070	-2.87	5.58	6.92E-05	0.000325525	hypothetical protein
TGME49_230010	-1.88	6.26	6.94E-05	0.000326056	hypothetical protein
TGME49_227010	-8.41	5.24	6.96E-05	0.000326628	rhoptry kinase family protein ROP30
TGME49_231030	-3.52	5.64	7.01E-05	0.000329004	hypothetical protein
TGME49_309400	-2.53	5.88	7.02E-05	0.000329135	RecF/RecN/SMC N terminal domain-containing protein
TGME49_320550	-1.82	6.64	7.04E-05	0.00032975	hypothetical protein
TGME49_209740	1.69	5.20	7.06E-05	0.000330755	hypothetical protein
TGME49_222192	-8.27	5.28	7.10E-05	0.000332281	hypothetical protein
TGME49_253300	2.25	4.94	7.13E-05	0.000333408	hypothetical protein
TGME49_315500	-1.57	6.66	7.23E-05	0.000337822	hypothetical protein
TGME49_255635	-3.63	4.83	7.35E-05	0.000343419	hypothetical protein
TGME49_263290	2.69	6.06	7.40E-05	0.000345422	rhomboid protease ROM2
TGME49_299015	-8.68	4.85	7.48E-05	0.000348746	hypothetical protein
TGME49_224235	-8.25	5.14	7.53E-05	0.000350732	translation initiation factor IF-3 protein
TGME49_246030	-8.28	5.02	7.59E-05	0.000353209	mediator complex subunit MED17
TGME49_309390	-8.27	4.72	7.60E-05	0.000353328	hypothetical protein
TGME49_269885	-1.18	8.06	7.61E-05	0.000353557	rhoptry metalloprotease toxolysin TLN1
TGME49_206390	-8.25	4.99	7.61E-05	0.000353557	hypothetical protein
TGME49_249690	-3.64	5.20	7.65E-05	0.000355105	hypothetical protein
TGME49_321170	2.76	5.28	7.69E-05	0.000356687	Toxoplasma gondii family C protein
TGME49_216170	-3.66	5.73	7.71E-05	0.000357169	SufS subfamily cysteine desulfurase
TGME49_289920	1.18	8.09	7.96E-05	0.000368503	hypothetical protein
TGME49_223900	1.89	5.15	8.18E-05	0.000378446	hypothetical protein
TGME49_209160	-1.05	7.51	8.28E-05	0.00038214	myristoyl CoA:protein N-myristoyltransferase
TGME49_214190	1.70	5.29	8.29E-05	0.000382521	SAG-related sequence SRS46
TGME49_209930	-8.28	5.30	8.42E-05	0.000388029	hypothetical protein
TGME49_291140	-1.02	8.46	8.43E-05	0.000388447	CCR4-Not complex component, Not1 protein
TGME49_240540	-2.03	6.41	8.50E-05	0.000391496	hypothetical protein
TGME49_230580	-8.23	5.21	8.61E-05	0.000395889	hypothetical protein
TGME49_318290	-8.23	5.09	8.64E-05	0.000397017	hypothetical protein
TGME49_284170	-8.25	4.98	8.71E-05	0.000400016	DHHC zinc finger domain-containing protein
TGME49_242830	-1.77	6.33	8.77E-05	0.000402353	XRN 5'-3' exonuclease N-terminus protein
TGME49_224600	-3.49	5.60	8.83E-05	0.000404642	GTP binding protein
TGME49_255400	1.27	6.07	8.85E-05	0.000405028	hypothetical protein
TGME49_242435	-3.49	5.43	8.99E-05	0.000411065	hypothetical protein

TGME49_242800	-1.67	6.66	9.05E-05	0.000413271	ribosome biogenesis protein NSA2, putative
TGME49_261510	3.07	5.09	9.06E-05	0.000413368	hypothetical protein
TGME49_269650	-1.46	7.00	9.16E-05	0.000417624	FFD and TFG box motifs protein
TGME49_225690	-1.09	8.01	9.18E-05	0.000418181	hypothetical protein
TGME49_281400	-2.25	6.55	9.22E-05	0.000419646	phosphofructokinase domain-containing protein
TGME49_249020	-2.17	6.04	9.44E-05	0.000429112	kinesin motor domain-containing protein
TGME49_312220	-3.50	5.27	9.46E-05	0.000429727	mitochondrial inner membrane translocase subunit TIM[illegible]
TGME49_306350	1.06	6.97	9.47E-05	0.000430019	variable surface lipoprotein
TGME49_268900	-8.21	5.16	9.56E-05	0.000433991	dense granular protein GRA10
TGME49_221270	-1.47	6.87	9.58E-05	0.000434505	oxidoreductase, 2OG-Fe(II) oxygenase family protein
TGME49_262650	-3.54	5.56	9.58E-05	0.000434505	WD domain, G-beta repeat-containing protein
TGME49_203358	-3.49	5.46	9.76E-05	0.000442123	hypothetical protein
TGME49_213890	-8.36	5.03	9.84E-05	0.000444831	Myb family DNA-binding domain-containing protein
TGME49_209270	-8.29	5.06	9.89E-05	0.000447136	hypothetical protein
TGME49_225300	-1.76	6.43	9.92E-05	0.000447886	hypothetical protein
TGME49_243490	-8.22	5.07	0.00010147	0.000458042	BCS1 family isoform 9, putative
TGME49_260400	1.33	6.04	0.00010197	0.000460017	hypothetical protein
TGME49_262860	-2.77	5.32	0.000102887	0.000463589	ADP-ribosylation factor family protein 1, putative
TGME49_224810	-3.76	5.52	0.000105486	0.000474429	hypothetical protein
TGME49_260370	-1.97	6.27	0.000105842	0.00047574	AtPH1 family protein
TGME49_234950	1.59	6.24	0.00010591	0.000475759	protein kinase (incomplete catalytic triad)
TGME49_305540	-1.64	6.49	0.000106158	0.000476583	hypothetical protein
TGME49_213670	-1.45	6.71	0.000106364	0.000477219	hypothetical protein
TGME49_316900	-1.67	6.55	0.000107698	0.000482912	Sas10 C-terminal domain-containing protein
TGME49_299190	1.52	5.76	0.000108605	0.000486682	B-box zinc finger domain-containing protein
TGME49_295450	-1.26	7.30	0.000108684	0.00048674	sjoegren syndrome nuclear autoantigen 1 family protein
TGME49_219820	-1.03	7.72	0.000109923	0.000491395	polyubiquitin UbC, putative
TGME49_307810	-1.22	7.09	0.000110464	0.000493218	hypothetical protein
TGME49_272570	2.02	5.28	0.000110585	0.000493222	dihydrouridine synthase (dus) protein
TGME49_255890	-2.76	5.76	0.000110598	0.000493222	pyridine nucleotide-disulfide oxidoreductase domain-co[illegible]
TGME49_259700	-1.57	6.48	0.000110873	0.000494152	hypothetical protein
TGME49_216930	-1.54	6.83	0.000112602	0.00050125	cholinephosphate cytidylyltransferase
TGME49_281675	1.83	4.86	0.000113103	0.000503179	protein kinase, putative
TGME49_234520	-2.17	5.92	0.000114717	0.000510053	U2 snRNP auxilliary factor, large subunit, splicing factor [illegible]
TGME49_291050	-1.43	7.12	0.000115076	0.000511342	histone kinase SNF1, putative
TGME49_223070	-1.18	7.72	0.000115276	0.000511921	hypothetical protein
TGME49_236510	1.16	6.37	0.000115578	0.000512955	hypothetical protein
TGME49_209240	1.25	5.74	0.000115726	0.000513306	RNA methyltransferase
TGME49_225310	-1.30	6.93	0.000116482	0.000516347	ARF1-directed GTPase-activating protein, putative
TGME49_233890	-2.17	6.03	0.000117321	0.000519757	hypothetical protein
TGME49_244610	-3.43	5.46	0.000119073	0.000526886	zinc finger, C3HC4 type (RING finger) domain-containing
TGME49_309130	-8.17	5.04	0.000119928	0.000530351	hypothetical protein
TGME49_221200	1.15	7.36	0.000120436	0.000532282	CW-type Zinc Finger protein
TGME49_277550	-8.19	4.87	0.000121209	0.000535376	UvrD/REP helicase domain-containing protein
TGME49_204380	-1.45	6.95	0.00012179	0.000537623	hypothetical protein
TGME49_255740	-2.80	5.77	0.000121982	0.000538146	hypothetical protein
TGME49_240730	-3.44	5.10	0.000122195	0.000538764	hypothetical protein
TGME49_268430	-3.47	5.49	0.000123516	0.000544265	hypothetical protein
TGME49_249780	-1.23	7.43	0.000124124	0.000546618	hypothetical protein
TGME49_267530	1.83	4.71	0.000124567	0.000548244	hypothetical protein
TGME49_225330	-1.38	6.63	0.000126342	0.000555723	hypothetical protein
TGME49_248550	-3.59	5.45	0.000126485	0.000556021	SPX domain-containing protein
TGME49_225680	-2.74	5.66	0.000126613	0.000556253	hypothetical protein
TGME49_268750	-3.47	5.37	0.000127482	0.00055974	peptidyl-prolyl cis-trans isomerase E, putative
TGME49_275750	-1.76	6.27	0.000127982	0.0005616	small nuclear ribonucleoprotein E, putative
TGME49_258920	-8.20	4.81	0.000128069	0.000561649	hypothetical protein
TGME49_264840	-8.15	5.33	0.000128435	0.000562313	ATP-dependent DNA helicase, RecQ family protein

TGME49_258230	-2.70	5.91	0.000128646	0.000562844	rhoptry kinase family protein ROP20
TGME49_252340	1.23	7.56	0.000130347	0.000569949	hypothetical protein
TGME49_264752	-3.52	5.23	0.000130487	0.000570225	HEAT repeat-containing protein
TGME49_294050	1.52	7.02	0.000130669	0.000570424	hypothetical protein
TGME49_309890	-2.78	5.90	0.000130733	0.000570424	hypothetical protein
TGME49_319940	-8.17	4.88	0.000131226	0.000572103	hypothetical protein
TGME49_224150	-8.15	4.83	0.000131901	0.000574705	hypothetical protein
TGME49_258060	-1.13	7.12	0.000132186	0.000575271	myosin heavy chain, putative
TGME49_216380	-2.22	6.35	0.000132464	0.000575802	phospholipid-translocating P-type ATPase, flippase subfa
TGME49_316300	-8.15	5.17	0.000132398	0.000575802	ATP-dependent DNA helicase, RecQ family protein
TGME49_216770	-3.45	5.97	0.000132972	0.00057767	hypothetical protein
TGME49_202080	-8.15	4.81	0.00013325	0.000578536	hypothetical protein
TGME49_261940	-8.16	4.88	0.000134275	0.000582303	hydrolase, alpha/beta fold family protein
TGME49_301120	-1.62	6.22	0.000135466	0.000587127	acetyl-CoA acetyltransferase
TGME49_207960	-1.98	5.90	0.000136174	0.000589846	hypothetical protein
TGME49_297780	-2.36	6.07	0.000138486	0.000598697	ATPase/histidine kinase/DNA gyrase B/HSP90 domain-co
TGME49_208722	-3.48	5.29	0.000138541	0.000598697	hypothetical protein
TGME49_256090	-8.16	4.73	0.000138695	0.000599015	glycerophosphodiester phosphodiesterase family protei
TGME49_245650	2.08	4.74	0.000139062	0.00060025	hypothetical protein
TGME49_207480	-1.95	6.41	0.000139524	0.000601889	GCC2 and GCC3 domain-containing protein
TGME49_216760	-8.19	4.80	0.000139893	0.000603129	RNA pseudouridine synthase superfamily protein
TGME49_272520	-1.36	6.87	0.00014204	0.00061096	hypothetical protein
TGME49_306530	-8.15	5.01	0.000141884	0.00061096	hypothetical protein
TGME49_310350	-1.75	6.39	0.000142496	0.000612565	PGAP1 family protein
TGME49_265460	1.18	6.01	0.000143355	0.0006159	hypothetical protein
TGME49_238180	-1.04	7.48	0.000145183	0.000622613	26s proteasome regulatory complex subunit, putative
TGME49_240810	-1.07	7.26	0.000145138	0.000622613	hypothetical protein
TGME49_223690	-8.14	5.06	0.000145432	0.000623013	hypothetical protein
TGME49_249698	-8.20	4.98	0.000146938	0.000629103	hypothetical protein
TGME49_204420	2.28	6.08	0.000148807	0.000636366	oocyst wall protein OWP1
TGME49_218358	-8.17	4.99	0.000150211	0.000641999	zinc knuckle domain-containing protein
TGME49_262390	-1.70	6.20	0.000150832	0.000644281	TLD protein
TGME49_234490	-2.36	6.15	0.000151801	0.000647742	kelch repeat-containing protein
TGME49_246050	-8.34	4.73	0.000151817	0.000647742	hypothetical protein
TGME49_233310	-3.41	5.31	0.000152368	0.000649715	peptidase D, putative
TGME49_260450	-8.14	5.10	0.000153325	0.000653419	DEAD/DEAH box helicase domain-containing protein
TGME49_234410	-1.04	7.49	0.000154279	0.000657109	transporter, small conductance mechanosensitive ion ch
TGME49_258350	1.50	5.57	0.00015507	0.000659946	hypothetical protein
TGME49_266890	-1.43	6.92	0.000155269	0.000659946	hypothetical protein
TGME49_311160	-1.53	6.69	0.000155302	0.000659946	PWI domain-containing protein
TGME49_305600	-8.12	4.82	0.000155267	0.000659946	hypothetical protein
TGME49_291590	-8.12	4.86	0.000155414	0.00066004	hypothetical protein
TGME49_308070	-2.16	5.86	0.000157262	0.000667506	hypothetical protein
TGME49_288330	-3.41	5.36	0.000157563	0.000668402	histone lysine methyltransferase, SET, putative
TGME49_290740	-8.14	4.96	0.000159221	0.000675048	hypothetical protein
TGME49_240460	-2.70	5.64	0.000160522	0.000679782	AP2 domain transcription factor AP2VI-1
TGME49_265850	1.78	6.27	0.000162229	0.0006865	hypothetical protein
TGME49_238070	-3.39	5.04	0.000162294	0.0006865	glutaredoxin domain-containing protein
TGME49_263590	-2.38	5.74	0.00016266	0.000687656	hypothetical protein
TGME49_316180	-2.10	5.67	0.000163149	0.000689327	hypothetical protein
TGME49_215660	-8.11	5.03	0.000163409	0.000689639	hypothetical protein
TGME49_273550	-3.42	5.26	0.000164884	0.000695069	hypothetical protein
TGME49_226600	-1.80	6.43	0.000167232	0.000704567	syntaxin 5, putative
TGME49_221600	-8.12	5.26	0.000167579	0.000705625	hypothetical protein
TGME49_291980	-1.26	7.41	0.000168847	0.000710562	HECT-domain (ubiquitin-transferase) domain-containing
TGME49_318525	-1.61	6.78	0.000169614	0.000713383	hypothetical protein
TGME49_271200	-1.43	6.92	0.000170511	0.000716066	AP2 domain transcription factor AP2VIII-5

TGME49_212970	3.81	5.08	0.000171671	0.000720394	protein kinase (incomplete catalytic triad)
TGME49_202800	-8.15	4.80	0.000173191	0.00072636	cytochrome c oxidase assembly protein COX11, putative
TGME49_247930	-8.15	4.68	0.000173564	0.000727513	SNARE domain-containing protein
TGME49_320260	1.68	5.77	0.000173906	0.000728533	hypothetical protein
TGME49_310660	-3.40	5.16	0.000174479	0.000730522	Dullard family phosphatase domain-containing protein
TGME49_288800	2.81	6.83	0.000174901	0.000731873	endonuclease/exonuclease/phosphatase family protein
TGME49_204550	-2.74	5.62	0.000175079	0.000732204	hypothetical protein
TGME49_316360	-3.48	5.40	0.000175774	0.000734694	hypothetical protein
TGME49_259000	-8.11	4.76	0.000175939	0.000734968	hypothetical protein
TGME49_300280	-2.41	5.77	0.000176986	0.000738508	LSM domain-containing protein
TGME49_264870	1.55	7.51	0.000177372	0.000739077	Sodium:neurotransmitter symporter family protein
TGME49_240430	1.39	5.61	0.000177353	0.000739077	glyoxalase family protein
TGME49_248400	-1.92	6.09	0.000177423	0.000739077	glyoxalase I, putative
TGME49_315930	-1.08	7.36	0.000178997	0.000744798	integral membrane protein, DUF56 family protein, putat
TGME49_239300	-1.69	6.35	0.00018009	0.000748083	hypothetical protein
TGME49_263330	-8.20	4.96	0.000181618	0.000754006	Adaptin ear-binding coat-associated protein 2 (NECAP-2)
TGME49_253320	-8.09	4.96	0.000181744	0.000754105	hypothetical protein
TGME49_202460	-1.69	6.49	0.000182743	0.000757398	diacylglycerol kinase accessory domain (presumed) dom
TGME49_201230	-2.70	5.61	0.000184408	0.000763874	kinesin motor domain-containing protein
TGME49_257290	-8.16	5.89	0.000185511	0.000768011	RNA 2'-phosphotransferase, Tpt1/KptA family protein
TGME49_307980	1.94	5.22	0.000186563	0.000771504	GTP-binding protein lepA, putative
TGME49_285470	1.85	5.36	0.000191169	0.000790108	patched family protein
TGME49_244700	1.43	7.88	0.000191786	0.000792215	NAD(+)/NADH kinase domain-containing protein
TGME49_208040	-2.08	5.83	0.000193048	0.000796983	aldo-keto reductase
TGME49_255390	-3.38	5.42	0.00019365	0.000798355	HEAT repeat-containing protein
TGME49_258850	-8.11	4.82	0.000193704	0.000798355	hypothetical protein
TGME49_292975	-2.70	6.05	0.000194147	0.000799733	hypothetical protein
TGME49_312660	-2.08	5.79	0.000194942	0.000802562	hypothetical protein
TGME49_269670	-2.67	5.91	0.000199593	0.000820338	hypothetical protein
TGME49_297150	-1.20	7.18	0.000201098	0.000826066	MORN repeat-containing protein
TGME49_304760	1.04	7.95	0.000202607	0.000831801	RNA recognition motif-containing protein
TGME49_244660	-3.40	5.30	0.000203878	0.000836555	hypothetical protein
TGME49_219550	-1.13	7.18	0.00020608	0.000844653	dihydrolipoyllysine-residue succinyltransferase compone
TGME49_295340	-1.12	7.60	0.000206468	0.000845775	UV excision repair protein Rad23 protein
TGME49_234220	-1.77	6.36	0.000208433	0.000852881	hypothetical protein
TGME49_313300	1.28	5.75	0.000209514	0.00085683	YL1 nuclear protein C-terminal domain-containing protei
TGME49_258700	-1.25	6.90	0.000211428	0.000863701	transporter, major facilitator family protein
TGME49_298020	-1.33	6.88	0.000211623	0.000864019	DEAD-family helicase
TGME49_211600	-2.09	6.15	0.000213071	0.000868972	hypothetical protein
TGME49_204100	-1.49	6.50	0.00021343	0.000869899	eIF2 kinase IF2K-C
TGME49_263730	-1.92	6.40	0.000213769	0.000869899	FAD-dependent glycerol-3-phosphate dehydrogenase
TGME49_266390	-8.07	4.86	0.000213942	0.000870125	DNA mismatch repair protein, C-terminal domain-contai
TGME49_232090	-2.06	5.75	0.00021421	0.000870735	3-hydroxyacyl-CoA dehydrogenase, NAD binding domain
TGME49_227300	1.91	4.84	0.000215504	0.000875513	hypothetical protein
TGME49_290180	2.30	5.13	0.000215627	0.000875533	AP2 domain transcription factor AP2IX-6
TGME49_244670	-1.41	6.91	0.000220323	0.000894109	hypothetical protein
TGME49_239630	-3.39	5.29	0.000224066	0.000908802	cytidine and deoxycytidylate deaminase zinc-binding reg
TGME49_235560	-8.05	5.26	0.000227306	0.000921435	hypothetical protein
TGME49_270940	-8.10	4.93	0.00023287	0.000942959	hypothetical protein
TGME49_285950	-8.09	5.02	0.000233511	0.000945037	hypothetical protein
TGME49_244100	-1.43	6.45	0.000233875	0.000945989	snoRNA binding domain-containing protein
TGME49_201220	-1.90	5.76	0.000234539	0.000948159	zinc finger protein
TGME49_228340	-8.07	5.00	0.000235133	0.000950041	hypothetical protein
TGME49_224170	1.95	5.91	0.000236021	0.000953109	SAG-related sequence SRS60A
TGME49_216850	-8.06	4.72	0.000236995	0.000956517	hypothetical protein
TGME49_281500	1.89	4.83	0.000237195	0.000956803	hypothetical protein
TGME49_318400	-1.30	7.52	0.000239265	0.000964626	hypothetical protein

TGME49_235970	-1.02	8.66	0.000240892	0.000970127	eukaryotic initiation factor-2 gamma, putative
TGME49_263840	-2.06	5.67	0.000242403	0.00097356	hypothetical protein
TGME49_260480	-3.42	4.87	0.000242208	0.00097356	leucine rich repeat-containing protein
TGME49_237290	-1.30	7.15	0.000243135	0.000975444	hypothetical protein
TGME49_309250	-1.53	6.68	0.000243058	0.000975444	hypothetical protein
TGME49_218830	1.57	5.33	0.000243427	0.000976082	hypothetical protein
TGME49_202640	-8.04	4.76	0.00024515	0.000982459	RNA pseudouridine synthase superfamily protein
TGME49_225920	-2.30	5.83	0.000246908	0.000988432	hypothetical protein
TGME49_271625	-8.07	4.93	0.0002472	0.000989067	serine--tRNA ligase
TGME49_269770	-2.69	5.57	0.000247906	0.000991357	WD domain, G-beta repeat-containing protein
TGME49_293060	-1.81	6.24	0.000249973	0.000999083	SPRY domain-containing protein
TGME49_240710	-1.71	6.51	0.000253266	0.001011148	RNA recognition motif-containing protein
TGME49_295980	-1.93	5.98	0.000253937	0.001012736	hypothetical protein
TGME49_219530	-3.40	5.05	0.000253895	0.001012736	hypothetical protein
TGME49_298840	1.11	6.20	0.000254627	0.001014939	hypothetical protein
TGME49_309580	-2.64	5.70	0.000258263	0.001028877	transporter, major facilitator family protein
TGME49_249380	1.41	7.53	0.000259787	0.001034392	DHHC zinc finger domain-containing protein
TGME49_208440	1.13	7.83	0.000260577	0.001036421	hypothetical protein
TGME49_208360	1.48	5.38	0.000261017	0.001037612	hypothetical protein
TGME49_264140	-1.45	7.26	0.000262181	0.001041122	hypothetical protein
TGME49_294980	1.58	6.35	0.000263168	0.001044478	hypothetical protein
TGME49_212840	-8.08	5.13	0.000264795	0.001050373	HIT zinc finger protein
TGME49_286270	-1.67	6.58	0.000265125	0.001051117	hypothetical protein
TGME49_273310	-1.66	6.16	0.00026576	0.001053069	hypothetical protein
TGME49_315590	-2.04	5.93	0.000266111	0.001053498	macro domain-containing protein
TGME49_278160	-3.39	5.32	0.000266153	0.001053498	vesicle transport v-snare protein
TGME49_244570	-8.04	4.81	0.000266507	0.001054334	hypothetical protein
TGME49_206640	1.11	6.95	0.000266858	0.001054597	hypothetical protein
TGME49_257360	1.01	6.17	0.000266805	0.001054597	hypothetical protein
TGME49_220920	-8.01	4.78	0.000269623	0.001064427	hypothetical protein
TGME49_278770	-1.25	7.22	0.000274365	0.001081951	hypothetical protein
TGME49_231940	-1.63	6.36	0.000274829	0.001082623	ThiF family protein
TGME49_252065	1.94	5.06	0.000278413	0.001096159	KRUF family protein
TGME49_240660	-8.05	4.90	0.000278991	0.001097849	hypothetical protein
TGME49_207940	-3.35	5.31	0.000281195	0.001105934	ribosomal protein S9, putative
TGME49_257480	-2.41	6.03	0.000283806	0.00111561	NADP-dependent succinate-semialdehyde dehydrogenas
TGME49_202010	-2.20	6.47	0.000289192	0.001135574	hypothetical protein
TGME49_236280	1.75	4.85	0.000290665	0.001140753	hypothetical protein
TGME49_218200	-2.66	5.33	0.000294125	0.00115372	UDP-sugar pyrophospharylase
TGME49_264880	-2.05	6.07	0.00029496	0.001155429	NEDD8-activating enzyme E1 catalytic subunit
TGME49_247280	-2.26	5.61	0.00029503	0.001155429	hypothetical protein
TGME49_207665	-8.04	4.98	0.000294889	0.001155429	kinesin motor domain-containing protein
TGME49_254800	1.43	5.35	0.000297004	0.001161931	hypothetical protein
TGME49_262880	-1.55	6.33	0.000297669	0.001163915	hypothetical protein
TGME49_227860	-8.00	4.75	0.000301027	0.001175802	hypothetical protein
TGME49_235960	-8.01	4.72	0.000302843	0.00118227	hypothetical protein
TGME49_221610	-1.91	6.25	0.000304127	0.001185406	ubiquitin carboxyl-terminal hydrolase
TGME49_286090	1.08	9.78	0.000307256	0.001196971	translation initiation factor SUI1, putative
TGME49_320090	-2.20	6.48	0.000308109	0.001199663	hypothetical protein
TGME49_230500	-3.30	5.36	0.000309171	0.001203163	hypothetical protein
TGME49_266110	1.44	5.29	0.000313903	0.001220292	DEAD/DEAH box helicase domain-containing protein
TGME49_243200	-1.33	9.17	0.000314283	0.001221127	hypothetical protein
TGME49_236780	-3.31	5.28	0.000314748	0.001222294	zinc finger, C3HC4 type (RING finger) domain-containing
TGME49_311220	-1.94	6.31	0.000315309	0.001223828	hypothetical protein
TGME49_222240	-1.27	6.92	0.00031577	0.001224974	hypothetical protein
TGME49_219850	-1.48	8.47	0.000316387	0.001226725	prolyl-tRNA synthetase (ProRS)
TGME49_206415	-1.42	6.70	0.00031863	0.001234775	myosin K

TGME49_287480	1.55	8.12	0.000323223	0.001250943	hypothetical protein
TGME49_309920	-3.29	5.24	0.000324698	0.001255659	hypothetical protein
TGME49_255660	-1.64	6.24	0.000325259	0.001257168	EF hand domain-containing protein
TGME49_214830	-3.30	5.63	0.000326455	0.001261132	hypothetical protein
TGME49_311280	-7.97	4.70	0.000329006	0.001269661	hypothetical protein
TGME49_269130	-7.97	4.83	0.000329566	0.001271159	hypothetical protein
TGME49_270450	-7.98	4.74	0.000333239	0.001283988	MCM2/3/5 family protein
TGME49_318590	-8.02	4.79	0.000333959	0.001285421	MRP family domain-containing protein
TGME49_310300	-2.62	5.65	0.000334471	0.001286723	hypothetical protein
TGME49_277680	-1.63	6.15	0.000334854	0.001287523	hypothetical protein
TGME49_249702	-2.29	5.88	0.000335197	0.001288174	MC family transporter, putative
TGME49_304670	-1.33	6.12	0.000337518	0.001295747	leucine rich repeat-containing protein
TGME49_238170	1.11	6.42	0.000337973	0.001296146	hypothetical protein
TGME49_209170	-1.29	6.83	0.000337919	0.001296146	hypothetical protein
TGME49_202480	-3.28	5.11	0.000344467	0.001319685	hypothetical protein
TGME49_278960	-1.55	6.55	0.000347103	0.001329095	hypothetical protein
TGME49_232680	1.15	6.24	0.000352293	0.001345484	hypothetical protein
TGME49_278930	-1.65	6.40	0.000354291	0.001351925	Tubulin-tyrosine ligase family protein
TGME49_220440	-2.07	5.93	0.000354345	0.001351925	cyclin-dependent kinase regulatory subunit protein
TGME49_228720	-7.96	4.72	0.000354811	0.001353001	hypothetical protein
TGME49_211020	-1.86	5.85	0.000356883	0.001360204	RNA recognition motif-containing protein
TGME49_293050	-2.64	5.54	0.000357816	0.001363055	sybindin family protein
TGME49_204060	-2.62	5.60	0.000358235	0.001363951	SNARE domain-containing protein
TGME49_270760	-7.99	4.79	0.000365189	0.001388282	asparagine synthase
TGME49_208990	1.48	5.29	0.000367232	0.001394613	hypothetical protein
TGME49_264820	1.45	6.38	0.000368041	0.001395338	RbAp48
TGME49_283790	-1.31	7.20	0.000368071	0.001395338	protein kinase, putative
TGME49_221922	-3.32	5.27	0.000368366	0.001395338	NifU family domain-containing protein
TGME49_209460	-7.97	4.82	0.000368108	0.001395338	hypothetical protein
TGME49_257150	-2.60	5.49	0.000369441	0.001398693	NOT2 / NOT3 / NOT5 family protein
TGME49_320005	-3.28	5.20	0.000371246	0.001404808	hypothetical protein
TGME49_268000	-2.63	5.44	0.000372304	0.001407369	hypothetical protein
TGME49_285520	-3.31	5.20	0.000373695	0.001411905	RNA cap guanine-N2 methyltransferase
TGME49_258980	-2.00	5.62	0.00037694	0.001422713	hypothetical protein
TGME49_288720	-1.43	9.52	0.000378282	0.001427048	ribosomal protein RPL10
TGME49_264450	-2.01	5.87	0.000382087	0.001439933	DNA topoisomerase III beta-1, putative
TGME49_275780	-7.94	4.85	0.000383791	0.001444883	hypothetical protein
TGME49_271170	-3.28	5.26	0.000384555	0.001447022	dolichol kinase
TGME49_308090	1.26	10.07	0.000385027	0.001447705	rhoptry protein ROP5
TGME49_255300	-1.56	6.62	0.000385128	0.001447705	hypothetical protein
TGME49_209150	-1.16	6.92	0.00038583	0.001448872	non-proton pumping type-II NADH dehydrogenase I
TGME49_278700	-2.60	5.93	0.000388996	0.001459278	hypothetical protein
TGME49_309100	1.77	5.47	0.000389436	0.001460189	hypothetical protein
TGME49_283820	1.31	5.91	0.000390448	0.001463241	glycine cleavage T-protein (aminomethyl transferase) do
TGME49_215550	-2.32	5.78	0.000391785	0.001467506	hypothetical protein
TGME49_277220	1.36	5.47	0.000401029	0.001498337	hypothetical protein
TGME49_271440	-2.60	5.43	0.000400913	0.001498337	NPL4 family protein
TGME49_246630	-1.70	5.95	0.000413037	0.001541642	DNA-directed RNA polymerase I RPA43
TGME49_205720	-7.98	4.68	0.000413403	0.001542229	Adenosine/AMP deaminase domain-containing protein
TGME49_318480	-2.01	6.16	0.000415858	0.001550607	SWI2/SNF2-containing protein RAD5
TGME49_216910	1.30	6.28	0.000416374	0.001551747	hypothetical protein
TGME49_305800	-1.83	5.93	0.000419267	0.001561743	6-pyruvoyl tetrahydrobiopterin synthase
TGME49_258990	-2.61	5.52	0.000428613	0.001595753	bromodomain-containing protein
TGME49_313860	1.64	7.16	0.000439934	0.001636253	regulator of chromosome condensation (RCC1) repeat-c
TGME49_223640	-7.99	5.20	0.000442061	0.001643338	hypothetical protein
TGME49_212800	-1.48	6.53	0.000444921	0.00165148	hypothetical protein
TGME49_260420	-1.69	6.17	0.000445595	0.001653151	HEC/Ndc80p family protein

TGME49_267760	-1.87	5.83	0.000448967	0.001663156	hypothetical protein
TGME49_213420	-7.90	4.75	0.00046009	0.001703508	RAP domain-containing protein
TGME49_312820	-1.86	5.76	0.000461569	0.00170813	hypothetical protein
TGME49_214800	-7.90	4.74	0.000465179	0.001719765	hypothetical protein
TGME49_242810	-3.27	5.22	0.000466716	0.001724587	hypothetical protein
TGME49_226330	-7.92	4.87	0.000472278	0.001741657	hypothetical protein
TGME49_315670	-1.26	6.74	0.000472559	0.001741825	HEAT repeat-containing protein
TGME49_209950	-1.25	9.03	0.000474064	0.001745634	thioredoxin, putative
TGME49_252320	-7.92	5.00	0.000477266	0.001756549	Sas10/Utp3/C1D family protein
TGME49_221710	-2.21	5.92	0.000479081	0.001762351	TBC domain-containing protein
TGME49_286480	1.10	6.47	0.000480732	0.001767543	hypothetical protein
TGME49_228320	-7.90	4.69	0.000483099	0.001775366	hypothetical protein
TGME49_220208	-7.90	4.78	0.000485104	0.001781846	hypothetical protein
TGME49_244440	-1.06	8.78	0.00048867	0.001793166	nucleoside transporter protein
TGME49_288860	-2.93	6.98	0.000489497	0.001794968	RuvB family 2 protein
TGME49_270160	-7.88	4.78	0.000489647	0.001794968	hypothetical protein
TGME49_215360	1.59	5.74	0.000490001	0.001795263	hypothetical protein
TGME49_223125	-1.82	6.12	0.000495147	0.001811537	ubiquitin family protein
TGME49_293650	1.82	5.03	0.000496326	0.001814953	hypothetical protein
TGME49_262550	-7.88	4.87	0.00049744	0.001818128	hypothetical protein
TGME49_318730	-1.30	6.68	0.000510787	0.001864146	glycosyl transferase
TGME49_306950	-8.04	4.67	0.000515088	0.001877989	RAP domain-containing protein
TGME49_223672	1.31	5.57	0.000517759	0.001885867	3'(2'),5'-bisphosphate nucleotidase
TGME49_312480	1.19	6.37	0.000521321	0.001896974	uracil phosphoribosyltransferase FUR1, putative
TGME49_314295	-3.23	4.70	0.000521153	0.001896974	ribosomal l25 family protein
TGME49_288045	-3.28	5.31	0.000522446	0.001900131	hypothetical protein
TGME49_288840	1.69	7.58	0.000528515	0.001920313	hypothetical protein
TGME49_214470	-2.19	5.61	0.000528827	0.001920503	Ulp1 protease family, C-terminal catalytic domain-contai
TGME49_294860	-1.68	6.13	0.000530747	0.001926531	hypothetical protein
TGME49_246020	-1.58	6.19	0.00053429	0.001938439	SprT domain-containing protein
TGME49_311625	-1.97	5.92	0.000535143	0.001940583	WD domain, G-beta repeat-containing protein
TGME49_257595	-2.65	5.61	0.000536982	0.001946297	hypothetical protein
TGME49_264190	-2.55	5.48	0.000537533	0.001947339	hypothetical protein
TGME49_215380	-3.25	5.25	0.000541669	0.001959443	hypothetical protein
TGME49_235730	1.57	5.14	0.000549242	0.001985867	hypothetical protein
TGME49_236840	-1.26	6.61	0.00055113	0.001990745	zinc finger (C-x8-C-x5-C-x3-H)-2, putative
TGME49_318300	-2.54	5.50	0.000554219	0.002000927	hypothetical protein
TGME49_235680	-1.51	7.23	0.000559823	0.00202017	peptidase M16 inactive domain-containing protein
TGME49_219170	-1.51	6.59	0.00056134	0.002023027	hypothetical protein
TGME49_285810	-7.85	4.79	0.000561435	0.002023027	MYND finger domain-containing protein
TGME49_202430	1.22	6.35	0.000565319	0.00203504	hypothetical protein
TGME49_289890	-2.64	5.61	0.000567043	0.002040253	hypothetical protein
TGME49_251890	-3.22	5.68	0.000568029	0.002042805	hypothetical protein
TGME49_264440	-1.69	6.16	0.000569565	0.002047094	signal recognition particle receptor beta subunit protein
TGME49_320460	-3.20	5.29	0.000569956	0.002047094	ABC transporter transmembrane region domain-contain
TGME49_321610	-7.86	4.89	0.000570259	0.002047094	hypothetical protein
TGME49_307010	1.54	6.89	0.00057146	0.002048287	histone lysine demethylase JMJC1/KDM5D/JARID1D
TGME49_240700	-1.67	5.96	0.000570955	0.002048287	ubiquitin family protein
TGME49_271860	-7.92	4.88	0.000571493	0.002048287	tRNA (Uracil-5-)-methyltransferase
TGME49_278780	-1.56	6.27	0.000577678	0.002068451	hypothetical protein
TGME49_282130	-2.19	5.72	0.000580818	0.002078687	hypothetical protein
TGME49_252270	1.90	6.10	0.000586955	0.002098619	L1P family of ribosomal protein
TGME49_281630	-1.30	6.44	0.000588628	0.002103391	hydroxyacylglutathione hydrolase
TGME49_219460	-2.53	5.68	0.000588858	0.002103391	hypothetical protein
TGME49_229790	-1.96	5.71	0.000589762	0.002105601	hypothetical protein
TGME49_313970	-2.25	5.45	0.000590093	0.002105766	Phytanoyl-CoA dioxygenase (PhyH) superfamily protein
TGME49_214280	-7.97	4.76	0.000591476	0.002109683	phosphoadenosine phosphosulfate reductase family pro

TGME49_227430	2.34	6.17	0.00059333	0.002111698	transmembrane amino acid transporter protein
TGME49_260520	1.26	7.70	0.00059404	0.002111698	hypothetical protein
TGME49_310730	-1.36	6.50	0.000593945	0.002111698	hypothetical protein
TGME49_314840	-1.49	6.47	0.000593145	0.002111698	ubiquitin carboxyl-terminal hydrolase
TGME49_212940	-7.86	5.88	0.00059393	0.002111698	hypothetical protein
TGME49_286180	-7.84	4.90	0.000594962	0.002113959	tRNA ligases class I (M) protein
TGME49_268870	-1.08	7.46	0.000596709	0.002119149	tetratricopeptide repeat-containing protein
TGME49_267400	-1.28	9.30	0.000597264	0.002120102	ribosomal protein RPL32
TGME49_226900	1.11	6.03	0.000599553	0.002127204	hypothetical protein
TGME49_235610	-3.19	5.08	0.000601942	0.002134658	ATPase, AAA family protein
TGME49_290990	1.02	6.58	0.000607624	0.002153773	HEAT repeat-containing protein
TGME49_265400	-1.46	6.69	0.000609775	0.002159329	hypothetical protein
TGME49_243390	-2.56	5.53	0.000610555	0.002161054	hypothetical protein
TGME49_258180	-7.91	4.82	0.000611383	0.002162951	hypothetical protein
TGME49_289720	-1.57	6.27	0.000613703	0.00217012	hypothetical protein
TGME49_233860	-7.90	4.93	0.000615334	0.002174847	DALR anticodon binding domain-containing protein
TGME49_237110	-1.56	6.30	0.000618717	0.002184716	replication factor C subunit 2, putative
TGME49_202950	-1.07	7.30	0.000620432	0.002188679	hypothetical protein
TGME49_248130	-7.87	4.76	0.000621962	0.00219303	hypothetical protein
TGME49_300380	-2.52	5.70	0.000625513	0.002204502	endoplasmic reticulum oxidoreductin, putative
TGME49_270330	1.39	6.26	0.000627878	0.002211782	cell-cycle-associated protein kinase, putative
TGME49_212200	1.28	6.03	0.000639522	0.002247447	hypothetical protein
TGME49_248370	1.12	5.93	0.000639284	0.002247447	prefoldin subunit 6, putative
TGME49_254200	-7.85	4.78	0.00064812	0.002272262	anticodon binding domain-containing protein
TGME49_271430	-3.18	5.38	0.000650132	0.002278234	hypothetical protein
TGME49_289190	-7.82	4.82	0.00065258	0.00228573	tetratricopeptide repeat-containing protein
TGME49_275470	1.49	5.71	0.000653501	0.002287873	dense granule protein GRA15
TGME49_240450	1.81	5.84	0.00066059	0.002307227	Maf family protein
TGME49_218800	-7.86	4.77	0.000670694	0.002339205	hypothetical protein
TGME49_224090	-1.96	5.72	0.000680651	0.002366489	enoyl-CoA hydratase/isomerase family protein
TGME49_250500	-3.24	5.36	0.000690239	0.00239607	hypothetical protein
TGME49_238510	-2.21	5.29	0.000692396	0.002402428	hypothetical protein
TGME49_310770	-1.46	6.43	0.000695442	0.002411866	hypothetical protein
TGME49_306540	1.19	5.76	0.000704317	0.002440148	phosphotransferase enzyme family protein
TGME49_285272	-7.86	4.73	0.000704587	0.002440148	hypothetical protein
TGME49_226320	1.17	6.83	0.000717106	0.002477701	hypothetical protein
TGME49_244250	-1.08	8.20	0.000719875	0.002486108	hypothetical protein
TGME49_254890	1.60	5.23	0.000729626	0.00251391	hypothetical protein
TGME49_221950	-1.25	6.81	0.00072932	0.00251391	splicesome-associated protein, putative
TGME49_272390	-7.80	5.13	0.00073573	0.002531404	hypothetical protein
TGME49_249850	-1.83	10.25	0.000741896	0.002549063	GAP40 protein
TGME49_252220	-1.20	7.73	0.000749732	0.00257479	tetratricopeptide repeat domain containing protein
TGME49_226790	1.95	6.23	0.000750199	0.002575195	ABC transporter, ATP-binding domain-containing protein
TGME49_214380	1.30	5.41	0.000757671	0.002598431	hypothetical protein
TGME49_262130	-1.55	6.15	0.000765562	0.00262306	hypothetical protein
TGME49_229940	-2.14	5.72	0.000766354	0.002624527	cyclophilin, putative
TGME49_315640	1.35	5.20	0.000768528	0.002627137	lipoyl(octanoyl) transferase
TGME49_201670	1.33	5.84	0.000767916	0.002627137	DnaJ domain-containing protein
TGME49_211230	-1.32	6.73	0.000769879	0.002629323	eukaryotic initiation factor-2B, alpha subunit, putative
TGME49_267040	-2.52	5.35	0.00077127	0.002632857	hypothetical protein
TGME49_280450	-7.80	4.79	0.000786559	0.002678867	hypothetical protein
TGME49_226060	1.29	6.99	0.000787337	0.002680282	transmembrane amino acid transporter protein
TGME49_247610	1.39	5.08	0.000794678	0.002702783	small nuclear ribonucleoprotein E, putative
TGME49_276170	1.24	9.19	0.000794374	0.002702783	phosphatidylinositol 3- and 4-kinase
TGME49_267120	-2.50	5.47	0.000797828	0.002712247	hypothetical protein
TGME49_230080	-2.14	5.55	0.000814157	0.002765217	DEAD/DEAH box helicase domain-containing protein
TGME49_210840	-1.09	6.95	0.000817906	0.002776674	arginyl-tRNA synthetase family protein

TGME49_222920	-1.76	6.07	0.000823867	0.002793063	mbp-1 interacting protein-2a family protein
TGME49_205570	-2.49	5.58	0.000825934	0.002798787	hypothetical protein
TGME49_220520	1.04	6.81	0.000828559	0.002806396	hypothetical protein
TGME49_246178	-1.91	5.82	0.000839148	0.002839663	hypothetical protein
TGME49_222910	-1.38	6.45	0.000841033	0.002844739	phosphoglycerate mutase
TGME49_212930	1.87	5.60	0.000845792	0.002858222	NifU family domain-containing protein
TGME49_203420	1.62	5.22	0.000848119	0.002863465	4'-phosphopantetheinyl transferase domain-containing p
TGME49_244860	1.42	5.15	0.000847992	0.002863465	hypothetical protein
TGME49_234300	1.07	6.14	0.000849148	0.002865633	hypothetical protein
TGME49_289140	1.10	6.44	0.000872568	0.002939301	ribosomal protein l22/l43, putative
TGME49_253540	1.31	5.28	0.000876908	0.002952576	hypothetical protein
TGME49_289100	-1.59	6.51	0.000878619	0.002955644	hypothetical protein
TGME49_281920	-1.32	6.37	0.00087955	0.002957432	V-type ATPase, D subunit protein
TGME49_218950	1.33	5.47	0.000894796	0.003005961	hypothetical protein
TGME49_321300	-3.13	5.37	0.000895467	0.003006849	autophagy-related 12 variant 1, putative
TGME49_295060	-7.75	4.82	0.000898982	0.003015913	exonuclease
TGME49_222120	-3.12	5.09	0.00090027	0.003017839	hypothetical protein
TGME49_265650	-3.16	5.20	0.00090078	0.003017839	protein phosphatase 2C domain-containing protein
TGME49_263410	4.20	5.00	0.000917539	0.003069811	scavenger receptor cysteine-rich domain-containing prot
TGME49_276190	-2.20	5.61	0.00093748	0.003130862	hypothetical protein
TGME49_246000	1.66	4.76	0.000944374	0.00315246	large subunit ribosomal protein IMG2
TGME49_217880	-7.75	4.80	0.000950006	0.003169253	RNA-binding protein Nova-1, putative
TGME49_295960	1.15	7.35	0.000951924	0.003171936	hypothetical protein
TGME49_224350	-1.28	8.79	0.00095179	0.003171936	aminopeptidase N, putative
TGME49_263505	-3.14	5.15	0.00095295	0.003172495	hypothetical protein
TGME49_297270	-2.48	5.28	0.000961931	0.003200954	hypothetical protein
TGME49_315360	-1.35	6.61	0.000963503	0.003204742	hypothetical protein
TGME49_224670	-1.89	5.88	0.00096521	0.003208975	DnaJ domain-containing protein
TGME49_245590	1.27	5.36	0.000968408	0.003217131	rhomboid protease ROM6
TGME49_310630	-1.27	6.41	0.000968533	0.003217131	hypothetical protein
TGME49_257060	-2.12	5.49	0.000970221	0.003219846	translation initiation factor sui1 protein
TGME49_312310	-2.50	5.42	0.000969892	0.003219846	ATPase, AAA family protein
TGME49_226740	1.06	7.97	0.000981739	0.003253998	zinc finger, C3HC4 type (RING finger) domain-containing
TGME49_203620	-3.12	5.21	0.000981833	0.003253998	hypothetical protein
TGME49_320015	-7.73	4.68	0.000982929	0.003256172	hypothetical protein
TGME49_217900	-1.50	6.15	0.000990102	0.003276999	hypothetical protein
TGME49_229450	-1.76	5.88	0.000993743	0.003286764	hypothetical protein
TGME49_253020	-2.14	6.02	0.000993942	0.003286764	hypothetical protein
TGME49_301350	-7.79	4.95	0.000997033	0.003295514	SNARE associated protein
TGME49_245475	1.32	5.26	0.000998745	0.003298223	hypothetical protein
TGME49_226000	-1.08	7.07	0.00100035	0.003302049	ATP synthase, putative
TGME49_295610	-1.06	7.21	0.001004683	0.003313393	histone lysine methyltransferase, SET, putative
TGME49_277710	-1.23	6.64	0.00100666	0.003316952	hypothetical protein
TGME49_294812	-3.12	5.19	0.001020245	0.003358724	RNA recognition motif-containing protein
TGME49_237870	-3.13	4.91	0.001024523	0.003368309	FYVE zinc finger domain-containing protein
TGME49_315980	-2.11	5.45	0.00102677	0.003374195	EREBP-4 family protein
TGME49_219300	1.11	5.66	0.001034394	0.003391711	ran binding protein
TGME49_227260	-2.49	5.33	0.001034072	0.003391711	RIO1 family protein
TGME49_255690	-2.46	5.59	0.001046326	0.003423245	2-C-methyl-D-erythritol 2,4-cyclodiphosphate synthase d
TGME49_272270	-7.71	4.79	0.001046194	0.003423245	radical SAM domain-containing protein
TGME49_260650	-3.12	5.40	0.001056956	0.003456495	glycosyltransferase, group 2 family protein
TGME49_273970	-3.09	5.02	0.001060796	0.003467519	CorA family Mg2+ transporter protein
TGME49_226840	-2.45	5.41	0.00106623	0.00348374	hypothetical protein
TGME49_275350	-1.73	6.21	0.001083741	0.003536267	TBC domain-containing protein
TGME49_250680	-2.09	5.77	0.001088171	0.003549155	TBC domain-containing kinase (incomplete catalytic triad
TGME49_216260	-1.03	7.05	0.001090802	0.003553963	eukaryotic initiation factor-2B, gamma subunit, putative
TGME49_287470	-1.60	5.53	0.001090609	0.003553963	hypothetical protein

TGME49_249190	1.62	5.13	0.001093407	0.003559951	AP2 domain transcription factor AP2XII-6
TGME49_270190	-2.57	5.63	0.001095701	0.003565851	protein phosphatase 2C domain-containing protein
TGME49_291690	-3.09	5.09	0.001102163	0.003585302	hypothetical protein
TGME49_319920	-1.01	7.48	0.001110449	0.003610667	2-oxo acid dehydrogenases acyltransferase (catalytic dor
TGME49_306195	2.44	5.14	0.001133504	0.003675933	hypothetical protein
TGME49_268620	-7.69	4.67	0.001132899	0.003675933	blood stage antigen 41-3 precursor, putative
TGME49_276900	-3.11	5.41	0.00113542	0.003680534	hypothetical protein
TGME49_236140	1.53	4.81	0.001148368	0.003711115	hypothetical protein
TGME49_316250	1.34	8.50	0.001150993	0.003717975	hypothetical protein
TGME49_304460	-1.05	7.52	0.001159832	0.003741797	zinc finger, C3HC4 type (RING finger) domain-containing
TGME49_318460	-1.35	6.53	0.001159886	0.003741797	P-type ATPase of unknown pump specificity (type V) pro
TGME49_210380	-1.26	6.73	0.001165867	0.00375945	hypothetical protein
TGME49_205730	-2.44	5.43	0.001171569	0.003776191	hypothetical protein
TGME49_290630	1.17	5.90	0.001173939	0.003778888	AP2 domain transcription factor AP2IX-7
TGME49_255670	-3.09	4.91	0.001178479	0.003785262	methyltransferase domain-containing protein
TGME49_214500	1.38	6.17	0.001179132	0.003785715	ankyrin repeat-containing protein
TGME49_240830	-7.68	4.70	0.001181462	0.003788793	hydrolase, alpha/beta fold family protein
TGME49_209140	-1.18	6.82	0.001185349	0.003799078	anti-silencing protein, ASF1 family protein
TGME49_210360	1.47	6.68	0.001187161	0.003803236	DEAD (Asp-Glu-Ala-Asp) box polypeptide 41 family prote
TGME49_203160	1.08	6.00	0.00122575	0.003921763	hypothetical protein
TGME49_294360	-1.28	6.51	0.001229149	0.003930936	ubiquitin specific protease 39 isoform 2, putative
TGME49_204270	-1.92	6.05	0.001230056	0.003932137	hypothetical protein
TGME49_204140	-7.74	4.77	0.001231177	0.003934021	PHD-finger domain-containing protein
TGME49_313570	-1.33	6.48	0.001236244	0.003946798	regulator of chromosome condensation (RCC1) repeat-c
TGME49_235420	-1.03	7.24	0.001242357	0.003962895	hypothetical protein
TGME49_259230	-2.45	5.32	0.001251458	0.003990204	site-specific recombinase, phage integrase family protei
TGME49_285660	-1.93	5.85	0.001253272	0.003994264	DEAD/DEAH box helicase domain-containing protein
TGME49_301300	-7.66	4.71	0.00128883	0.004095234	hypothetical protein
TGME49_215940	-1.37	6.63	0.001290167	0.004097722	Acetyl-coenzyme A transporter, putative
TGME49_231920	1.51	6.14	0.001305239	0.004142032	oxidoreductase, short chain dehydrogenase/reductase fa
TGME49_269800	-2.08	5.58	0.001309699	0.004154224	glutamine-dependent NAD(+) synthetase protein, putati
TGME49_254365	-1.17	7.32	0.001323288	0.004186731	phosphatidate cytidylyltransferase
TGME49_263530	-1.36	6.47	0.001322745	0.004186731	chaperonin , putative
TGME49_221360	-1.89	5.89	0.001322528	0.004186731	hypothetical protein
TGME49_287980	-1.58	6.10	0.001336699	0.004225547	FHA domain-containing protein
TGME49_281450	-2.46	5.45	0.001341632	0.004239327	cell-cycle-associated protein kinase, putative
TGME49_213020	1.68	5.21	0.001349232	0.004261522	hypothetical protein
TGME49_258080	-1.75	5.91	0.001352458	0.004269888	hypothetical protein
TGME49_246780	-7.65	4.74	0.001361979	0.004298111	hypothetical protein
TGME49_269630	1.47	5.06	0.001367647	0.004314158	hypothetical protein
TGME49_246530	-3.15	5.27	0.001376932	0.004341594	phospholipase D active site domain-containing protein
TGME49_251480	1.76	5.55	0.001382969	0.004356912	DEAD/DEAH box helicase domain-containing protein
TGME49_290960	-1.21	6.72	0.001387075	0.004364268	pyruvate phosphate dikinase, pep/pyruvate binding dom
TGME49_262030	-7.63	4.93	0.001386958	0.004364268	ALG6, ALG8 glycosyltransferase family protein
TGME49_255930	1.41	5.46	0.001392477	0.004379402	hypothetical protein
TGME49_278230	-3.05	5.10	0.001401203	0.004399361	prenyltransferase and squalene oxidase repeat-containi
TGME49_252190	1.56	6.80	0.001425631	0.004474038	KRUF family protein
TGME49_265380	-1.86	5.88	0.001436157	0.004501461	tetratricopeptide repeat (TPR)-/ U-box domain-containi
TGME49_294430	-3.04	5.21	0.001436916	0.00450193	hypothetical protein
TGME49_203480	-3.09	5.11	0.001447385	0.004528973	hypothetical protein
TGME49_313960	-3.09	5.10	0.001448911	0.004529916	ribosomal protein L19 protein
TGME49_310210	1.01	6.10	0.001449669	0.004530369	hypothetical protein
TGME49_242320	-1.31	6.85	0.001462603	0.004566928	B-box zinc finger domain-containing protein
TGME49_266690	-1.12	6.96	0.001481238	0.004621892	hypothetical protein
TGME49_219260	-1.05	6.99	0.001487204	0.004637869	cation-transporting ATPase, putative
TGME49_273840	-2.09	5.63	0.00151589	0.004719363	brix domain-containing protein
TGME49_244130	-1.39	6.36	0.001517217	0.004721508	hypothetical protein

TGME49_261030	-3.23	4.80	0.001521604	0.004733169	pyridine nucleotide-disulfide oxidoreductase domain-co
TGME49_295850	-1.01	7.27	0.001534993	0.004772809	cyclic nucleotide-binding domain-containing protein
TGME49_239080	-7.62	4.71	0.001543195	0.004794278	carrier superfamily protein
TGME49_233760	-2.10	5.69	0.00154869	0.004809328	hypothetical protein
TGME49_314550	-2.38	4.92	0.001556669	0.004828024	hypothetical protein
TGME49_258670	-7.67	5.62	0.001556321	0.004828024	hypothetical protein
TGME49_291350	-3.06	5.02	0.001559587	0.004835046	hypothetical protein
TGME49_234610	-1.31	6.61	0.001581393	0.004892392	WD-40 repeat protein
TGME49_284050	-1.41	6.47	0.001588482	0.004912269	DEAD/DEAH box helicase domain-containing protein
TGME49_213388	1.25	5.45	0.001590128	0.004915303	hypothetical protein
TGME49_269700	1.75	8.27	0.00159665	0.004933403	NLI interacting factor family phosphatase
TGME49_201120	-1.34	6.58	0.001606175	0.004958689	ELMO/CED-12 family protein
TGME49_288640	1.49	5.01	0.001617922	0.004988705	radical SAM domain-containing protein
TGME49_224740	1.13	5.80	0.001618618	0.004988771	hypothetical protein
TGME49_270370	-2.15	5.67	0.001629963	0.005021644	clathrin assembly protein AP19, putative
TGME49_227440	1.14	5.77	0.001633233	0.005029622	WD domain, G-beta repeat-containing protein
TGME49_288210	1.19	6.24	0.001648517	0.005074577	PUL domain-containing protein
TGME49_297360	-1.42	6.33	0.00165056	0.005078751	hypothetical protein
TGME49_268240	1.64	4.85	0.00166286	0.005108096	hypothetical protein
TGME49_284010	-3.01	5.48	0.001672953	0.005134834	5'-3' exonuclease, N-terminal resolvase family domain-co
TGME49_233540	-3.17	5.15	0.001677917	0.005145799	transporter, major facilitator family protein
TGME49_315910	1.00	5.86	0.001682735	0.005158433	hypothetical protein
TGME49_224980	-1.23	6.48	0.00168973	0.005175587	hypothetical protein
TGME49_217610	-1.83	5.34	0.001695087	0.005189843	hypothetical protein
TGME49_209985	1.72	6.45	0.001702261	0.00520965	cAMP-dependent protein kinase
TGME49_244510	-7.58	4.91	0.001703336	0.005210784	AP2 domain transcription factor AP2VI-3
TGME49_288370	1.28	5.46	0.001712611	0.005234828	hypothetical protein
TGME49_221370	-3.01	5.30	0.001719179	0.005248395	hypothetical protein
TGME49_238000	-3.04	5.17	0.00172104	0.005251907	peptidyl-prolyl isomerase
TGME49_203362	-3.06	4.97	0.001721904	0.005252376	hypothetical protein
TGME49_317705	-1.59	6.25	0.001724241	0.005257334	enoyl-CoA hydratase/isomerase family protein
TGME49_286140	-1.68	5.93	0.001730201	0.005273333	hypothetical protein
TGME49_218790	-3.03	4.93	0.00173619	0.005289407	elongation factor G C-terminus domain-containing prote
TGME49_222870	-1.41	6.27	0.001754648	0.005339039	hypothetical protein
TGME49_250090	1.00	6.15	0.001755983	0.005340903	hypothetical protein
TGME49_294720	-2.11	5.83	0.001758178	0.005345382	hypothetical protein
TGME49_239050	1.05	6.44	0.00177224	0.005385919	hypothetical protein
TGME49_267480	1.07	5.89	0.001778263	0.005399785	tRNA (guanine(26)-N(2))-dimethyltransferase, putative
TGME49_267390	-1.31	6.54	0.00180349	0.005471894	DNA-directed RNA polymerase I RPAC1
TGME49_316520	-1.32	6.34	0.00181011	0.005481898	1,4-alpha-glucan-branching enzyme
TGME49_254900	-1.09	6.78	0.001829058	0.005531312	proteasome subunit beta type 2, putative
TGME49_309610	-3.05	4.89	0.001833488	0.00554244	hypothetical protein
TGME49_280790	1.23	5.60	0.001842901	0.005568618	phosphatidylinositol n-acetylglucosaminyltransferase
TGME49_240960	1.75	7.06	0.001843941	0.005569485	AIG2 family protein
TGME49_204410	-1.68	6.09	0.001858048	0.005609802	endonuclease/exonuclease/phosphatase family protein
TGME49_231590	-1.57	5.88	0.001858925	0.005610161	SGS domain-containing protein
TGME49_255440	1.29	5.19	0.001867381	0.005631084	hypothetical protein
TGME49_227920	-1.66	5.69	0.001869363	0.005634761	hypothetical protein
TGME49_216580	-1.57	6.03	0.001879841	0.005661729	hypothetical protein
TGME49_229290	-7.57	4.73	0.001883085	0.005669189	kelch repeat-containing protein
TGME49_258050	-2.35	5.14	0.001915143	0.00575867	actin like protein ALP2a
TGME49_290225	-3.01	5.04	0.001917356	0.00576298	hypothetical protein
TGME49_230210	-1.10	10.35	0.001919079	0.005765814	alveolin domain containing intermediate filament IMC10
TGME49_239270	-1.16	7.56	0.001922268	0.005773049	hypothetical protein
TGME49_234900	-1.13	7.62	0.001936526	0.005813508	PHD-finger domain-containing protein
TGME49_221490	-2.34	5.40	0.001943376	0.005831706	cell cycle regulator protein
TGME49_216700	-3.00	4.75	0.001947915	0.005840585	hypothetical protein

TGME49_200375	-2.41	5.05	0.001954172	0.005852226	hypothetical protein
TGME49_208350	1.00	6.11	0.001956325	0.005856304	hypothetical protein
TGME49_272640	-1.55	6.34	0.001957818	0.005857036	eukaryotic initiation factor-2B, epsilon subunit, putative
TGME49_203970	-2.35	5.54	0.001958154	0.005857036	dolichyl-diphosphooligosaccharide--protein glycosyltrans
TGME49_308990	-2.98	5.30	0.001963221	0.005869815	transporter, solute:sodium symporter (SSS) family protei
TGME49_226400	-1.44	6.16	0.001983379	0.005927689	lipoic acid synthase LIPA
TGME49_261400	-1.02	7.86	0.002016369	0.006014133	hypothetical protein
TGME49_216800	2.12	5.49	0.002026919	0.00604316	flagellar/basal body protein
TGME49_213040	-1.45	6.26	0.002029524	0.006048488	hypothetical protein
TGME49_218740	1.68	5.57	0.002040534	0.006078852	membrane protein, putative
TGME49_202520	1.22	6.53	0.002047255	0.006096417	hypothetical protein
TGME49_269140	-1.49	6.33	0.002089924	0.006213469	transport protein particle component, Bet3 domain-cont
TGME49_297245	-1.09	7.00	0.002091281	0.006215004	transporter, major facilitator family protein
TGME49_281980	-1.12	6.99	0.0020962	0.006227121	phosphatidate cytidylyltransferase
TGME49_208390	1.32	6.10	0.002106785	0.006253541	hypothetical protein
TGME49_221410	-1.11	6.65	0.002107836	0.006254149	actin-like protein ALP4
TGME49_260310	1.21	5.29	0.002110951	0.006260878	ATP-binding cassette transporter ABC.B1
TGME49_308930	-1.52	5.90	0.002119514	0.006281236	50S ribosomal protein L33, putative
TGME49_304720	-1.72	5.94	0.002129888	0.006306925	hypothetical protein
TGME49_271000	-1.88	5.98	0.002134182	0.006317111	hypothetical protein
TGME49_316750	-2.99	4.89	0.002144879	0.006346231	DEAD/DEAH box helicase domain-containing protein
TGME49_318430	-1.22	9.14	0.00214773	0.006352126	malate dehydrogenase MDH
TGME49_289120	1.08	6.55	0.002191582	0.006474054	hypothetical protein
TGME49_237170	-1.72	6.80	0.002193985	0.006478564	hypothetical protein
TGME49_312650	1.34	6.55	0.002200568	0.006492654	hypothetical protein
TGME49_216870	-1.78	5.83	0.002202269	0.006492654	DNA excision repair helicase
TGME49_273885	-2.34	5.11	0.002208323	0.006505314	hypothetical protein
TGME49_290310	-1.79	5.87	0.002223932	0.00654608	hypothetical protein
TGME49_223020	-1.11	6.34	0.002227687	0.006554523	coproporphyrinogen III oxidase
TGME49_257170	-2.95	5.13	0.002233378	0.006560826	hypothetical protein
TGME49_264650	-7.56	4.90	0.002232869	0.006560826	phosphoacetylglucosamine mutase
TGME49_294380	-2.32	5.40	0.002244996	0.006592335	PP-loop domain-containing protein
TGME49_277090	2.09	7.62	0.002260184	0.0066343	carrier superfamily protein
TGME49_233520	-1.09	6.77	0.002282414	0.006694236	ATP-dependent RNA helicase
TGME49_320670	-1.24	6.65	0.002287278	0.006704194	vacuolar protein sorting 16, putative
TGME49_309070	1.55	5.61	0.00228982	0.006705317	hypothetical protein
TGME49_260640	-1.78	5.79	0.002291353	0.006707153	autophagy protein apg9 protein
TGME49_271110	-2.96	5.21	0.002304204	0.006739433	hypothetical protein
TGME49_271990	1.10	6.11	0.002310925	0.006755699	hypothetical protein
TGME49_200330	-1.42	6.65	0.002311593	0.006755699	hypothetical protein
TGME49_202280	-2.99	5.77	0.002316042	0.006766026	WD domain, G-beta repeat-containing protein
TGME49_277970	1.14	5.90	0.002320467	0.006776277	dolichol-phosphate-mannose synthase family protein
TGME49_301170	-7.53	5.92	0.002328567	0.006794564	SAG-related sequence SRS19D
TGME49_315420	1.08	5.70	0.002333409	0.006806006	hypothetical protein
TGME49_270750	-1.69	6.29	0.002370006	0.006899144	hypothetical protein
TGME49_223270	1.19	5.48	0.002381445	0.006927321	hypothetical protein
TGME49_273030	-2.31	5.76	0.002388294	0.00694145	phosphoglycerate mutase family protein
TGME49_262990	-3.00	4.94	0.002391059	0.006946754	hypothetical protein
TGME49_223600	-2.00	5.45	0.002398603	0.006965936	hypothetical protein
TGME49_215920	1.30	5.98	0.00240065	0.006969142	hypothetical protein
TGME49_240050	1.14	7.04	0.002426584	0.007033384	hypothetical protein
TGME49_307860	1.15	8.14	0.002429787	0.007038555	hypothetical protein
TGME49_232970	-2.94	5.07	0.002430272	0.007038555	hypothetical protein
TGME49_220160	-2.94	5.04	0.002438081	0.007058407	WD domain-containing protein
TGME49_321540	-1.00	7.08	0.002457758	0.007109806	hypothetical protein
TGME49_243780	1.03	5.94	0.002481508	0.007170093	hypothetical protein
TGME49_207450	-1.41	5.89	0.002496025	0.007209223	DNA segment, Chr 10, Wayne State University 52, expres

TGME49_319320	-1.36	6.45	0.002504753	0.007228785	hypothetical protein
TGME49_246978	-2.93	5.07	0.002506718	0.007231633	hypothetical protein
TGME49_235402	-7.51	4.70	0.002536297	0.007308409	CorA family Mg2+ transporter protein
TGME49_232035	-1.99	5.23	0.002556069	0.007356782	hypothetical protein
TGME49_278020	-2.94	4.94	0.002583776	0.007422082	hypothetical protein
TGME49_288510	2.14	5.86	0.002592065	0.007439797	ubiquitin carboxyl-terminal hydrolase
TGME49_268730	1.12	5.50	0.002597838	0.007450751	glutaredoxin-related protein
TGME49_207065	-1.77	5.59	0.002601296	0.007455031	hypothetical protein
TGME49_281650	-1.49	5.97	0.002607807	0.007470798	hypothetical protein
TGME49_249320	-1.97	5.46	0.002651257	0.007577657	flavodoxin domain-containing protein
TGME49_255215	2.48	5.93	0.002659869	0.007599332	hypothetical protein
TGME49_293460	-2.94	5.22	0.002678246	0.007642975	ATP-dependent DNA ligase domain-containing protein
TGME49_288700	-1.33	6.08	0.002703951	0.007710378	RecF/RecN/SMC N terminal domain-containing protein
TGME49_220480	-2.94	4.69	0.002724001	0.007764557	hypothetical protein
TGME49_311320	-2.37	5.00	0.00273772	0.007800656	hypothetical protein
TGME49_311750	1.92	5.91	0.002756145	0.007847106	mago binding protein
TGME49_207120	1.11	5.86	0.002770394	0.007881605	Sad1/UNC family protein
TGME49_272000	-1.96	5.52	0.002787736	0.007924844	hypothetical protein
TGME49_311080	-1.63	5.91	0.002792271	0.007934687	transporter, cation channel family protein
TGME49_311510	-1.21	8.65	0.002798641	0.007940581	eIF2 kinase IF2K-B
TGME49_242240	-1.32	6.05	0.002797858	0.007940581	rhoptry kinase family protein ROP19A
TGME49_221480	-1.20	7.89	0.002805296	0.00795641	hypothetical protein
TGME49_216190	1.35	5.21	0.002867932	0.008121595	hypothetical protein
TGME49_227630	2.27	4.86	0.002905243	0.00822096	hypothetical protein
TGME49_221390	-1.59	5.94	0.002921043	0.008259346	hypothetical protein
TGME49_209530	1.41	4.95	0.002946844	0.008329116	hypothetical protein
TGME49_312360	-2.92	4.90	0.002964672	0.008376303	hypothetical protein
TGME49_227900	1.54	6.24	0.002971842	0.008393355	AP2 domain transcription factor AP2X-1
TGME49_258780	1.03	5.89	0.002978508	0.008408968	OTU family cysteine protease
TGME49_249425	-1.13	7.75	0.00297972	0.008409178	hypothetical protein
TGME49_228460	-1.50	6.12	0.003007059	0.008479862	hypothetical protein
TGME49_249950	-2.28	5.46	0.003017795	0.008503649	Mak16 protein
TGME49_255960	-1.25	6.58	0.003021819	0.008511744	hypothetical protein
TGME49_263070	-1.00	8.09	0.003058709	0.008612374	CMGC kinase, CK2 family
TGME49_319720	-2.96	5.17	0.003073161	0.008642923	hypothetical protein
TGME49_257120	-1.20	5.94	0.003084155	0.008664711	sugar transporter ST1
TGME49_224470	1.05	6.39	0.003086922	0.008668706	hypothetical protein
TGME49_305260	-2.89	4.78	0.00309747	0.008691725	hypothetical protein
TGME49_320690	-1.59	5.76	0.003110467	0.008721575	gamma-soluble NSF attachment protein, putative
TGME49_230920	-2.89	4.80	0.003116158	0.008733062	adaptor complexes medium subunit family protein
TGME49_298070	-7.52	5.01	0.003116926	0.008733062	hypothetical protein
TGME49_247670	-2.02	5.41	0.003124156	0.008750003	ribulose-phosphate 3 epimerase family protein
TGME49_210960	-1.36	6.30	0.003139448	0.008784195	replication factor C subunit 4, putative
TGME49_249300	-1.97	7.71	0.003170195	0.008862163	hypothetical protein
TGME49_222270	-1.25	6.62	0.003190304	0.008904911	hypothetical protein
TGME49_265780	-1.16	6.42	0.003244415	0.009042294	flagellar/basal body protein
TGME49_264210	1.09	5.57	0.00325562	0.009070103	hypothetical protein
TGME49_243530	-7.39	4.75	0.00327128	0.009103442	pentatricopeptide repeat domain-containing protein
TGME49_230710	-2.93	5.18	0.003304531	0.009182151	cell division protein CDC48
TGME49_289790	-2.92	4.93	0.00333312	0.009258111	hypothetical protein
TGME49_254610	1.59	5.99	0.003339126	0.00926783	Tim10/DDP family zinc finger superfamily protein
TGME49_217730	1.00	5.67	0.003365116	0.009332958	hypothetical protein
TGME49_223420	1.31	5.26	0.003383171	0.009376	DnaJ domain-containing protein
TGME49_307610	-1.42	6.31	0.003417376	0.009460158	elongation factor TS, putative
TGME49_320620	-1.94	5.26	0.003436156	0.009505027	queuine tRNA ribosyl transferase
TGME49_243790	-1.57	5.96	0.003448795	0.009536421	SAG-related sequence SRS33
TGME49_290640	-1.13	7.98	0.003472934	0.009595992	DNA mismatch repair protein MSH6-1, putative

TGME49_294190	-2.87	5.22	0.003483081	0.009620434	enoyl-CoA hydratase/isomerase family protein
TGME49_245580	1.03	6.12	0.003497051	0.009648207	hypothetical protein
TGME49_297330	1.08	5.60	0.003505416	0.009659131	hypothetical protein
TGME49_295950	1.42	6.77	0.003524077	0.009693833	KRUF family protein
TGME49_203280	-2.28	5.13	0.003604907	0.00989409	hypothetical protein
TGME49_200440	1.32	5.08	0.003614187	0.009915879	hypothetical protein
TGME49_249270	-1.22	9.01	0.003615899	0.009916898	protein disulfide isomerase-related protein (provisional)
TGME49_301460	1.27	5.61	0.00364702	0.009987431	hypothetical protein
TGME49_264890	-2.25	5.24	0.003670702	0.010048563	hypothetical protein
TGME49_267700	-2.87	5.07	0.003705201	0.01013175	hypothetical protein
TGME49_307020	-1.40	6.25	0.003709409	0.010139509	hypothetical protein
TGME49_253800	-2.89	5.03	0.003714537	0.010149772	ribosomal protein L15, putative
TGME49_286050	1.09	5.84	0.003722784	0.010168549	hypothetical protein
TGME49_246990	-2.24	5.31	0.003756518	0.010253117	hypothetical protein
TGME49_258462	-1.26	6.26	0.003828397	0.010426213	hypothetical protein
TGME49_264980	1.18	5.56	0.003864167	0.010512011	hypothetical protein
TGME49_277685	-7.38	5.11	0.003908802	0.010617811	hypothetical protein
TGME49_207950	-1.58	5.76	0.003940754	0.010696747	hypothetical protein
TGME49_233000	-1.07	7.06	0.003994762	0.010827444	KOW motif domain-containing protein
TGME49_219290	1.07	5.81	0.00402714	0.010899222	F-actin-capping protein subunit beta, putative
TGME49_219130	-1.55	5.89	0.004025725	0.010899222	NADPH-glutathione reductase
TGME49_215030	-1.70	5.85	0.004026875	0.010899222	hypothetical protein
TGME49_288945	-1.45	6.28	0.004032307	0.010909214	hypothetical protein
TGME49_222410	-1.18	6.72	0.004042052	0.010931577	hypothetical protein
TGME49_257780	-1.56	5.65	0.004063431	0.010985378	hypothetical protein
TGME49_228060	-2.94	5.12	0.004071245	0.011002481	hypothetical protein
TGME49_231100	-1.00	6.82	0.004175045	0.011265234	hypothetical protein
TGME49_249410	-1.73	6.04	0.004227112	0.011387134	hypothetical protein
TGME49_260790	-1.90	5.27	0.004241726	0.011418647	RAP domain-containing protein
TGME49_216920	-1.94	5.62	0.004242227	0.011418647	mediator complex subunit MED8
TGME49_310620	-1.02	6.77	0.004265158	0.011476194	starch binding domain-containing protein
TGME49_244630	1.36	4.89	0.004274184	0.011496295	hypothetical protein
TGME49_212725	-1.90	5.73	0.004349137	0.011668186	hypothetical protein
TGME49_309370	-1.09	6.46	0.004356357	0.011683317	adaptin n terminal region domain-containing protein
TGME49_312700	1.71	6.57	0.004399414	0.011794514	hypothetical protein
TGME49_309730	-1.23	8.64	0.004409188	0.011816432	thioredoxin reductase
TGME49_222840	1.21	8.70	0.004446317	0.011907304	Ser/Thr phosphatase family protein
TGME49_223040	1.13	7.86	0.004458136	0.011930314	hypothetical protein
TGME49_227050	-2.81	5.12	0.004489445	0.011992441	ATPase/histidine kinase/DNA gyrase B/HSP90 domain-co
TGME49_214120	-2.85	4.78	0.004489461	0.011992441	hypothetical protein
TGME49_243750	2.44	5.73	0.004540686	0.012116142	tetratricopeptide repeat-containing protein
TGME49_289780	-1.42	5.77	0.004563635	0.012172987	ATP-dependent hsl protease ATP-binding subunit hslU, p
TGME49_264240	-7.39	4.81	0.004589883	0.012238584	hypothetical protein
TGME49_245540	-1.11	6.30	0.004644586	0.012357707	hypothetical protein
TGME49_278260	-2.82	4.79	0.004661733	0.012398868	polynucleotide adenylyltransferase
TGME49_251710	1.15	5.03	0.004746225	0.012600928	hypothetical protein
TGME49_250040	-1.57	5.65	0.004869791	0.012910447	hypothetical protein
TGME49_270350	-1.13	6.42	0.004916517	0.013024983	hypothetical protein
TGME49_288990	-2.26	5.24	0.004933307	0.013060104	hypothetical protein
TGME49_206500	-2.88	4.78	0.005084687	0.013425178	hypothetical protein
TGME49_307800	1.30	6.04	0.005141593	0.013553275	GDA1/CD39 (nucleoside phosphatase) family protein
TGME49_269760	1.29	5.00	0.005139836	0.013553275	'chromo' (CHRromatin Organization MOdifier) domain-co
TGME49_263240	-1.31	5.80	0.005151702	0.013575083	hypothetical protein
TGME49_272210	-2.18	4.96	0.00515912	0.013589788	hypothetical protein
TGME49_278170	-1.90	5.42	0.005161593	0.013591459	hypothetical protein
TGME49_299030	1.19	7.26	0.005213278	0.013712906	RNA recognition motif 2 protein
TGME49_255980	-1.31	5.74	0.005222511	0.013727426	hypothetical protein

TGME49_228780	1.08	5.46	0.005229203	0.01373203	Toxoplasma gondii family C protein
TGME49_220450	-7.24	4.82	0.005260697	0.013803262	ribonuclease HI protein
TGME49_205625	-2.80	5.09	0.005329257	0.013959271	hypothetical protein
TGME49_305510	-1.13	7.27	0.005400795	0.014115759	hypothetical protein
TGME49_229160	-2.82	4.89	0.00540026	0.014115759	DHHC zinc finger domain-containing protein
TGME49_251560	-1.03	6.42	0.005445186	0.014211686	hypothetical protein
TGME49_202190	1.15	6.78	0.0054593	0.014242699	hypothetical protein
TGME49_256820	-1.65	5.47	0.005490595	0.014304954	zinc finger (CCCH type) motif-containing protein
TGME49_299060	-1.02	6.76	0.005494999	0.014311384	sodium/hydrogen exchanger NHE2
TGME49_306020	-1.31	6.20	0.005501194	0.014322475	hypothetical protein
TGME49_262450	-1.15	7.29	0.005531671	0.014396752	hypothetical protein
TGME49_204350	-1.51	5.92	0.005566229	0.014481595	hypothetical protein
TGME49_291820	1.27	6.44	0.005600475	0.014565568	RNA helicase (UPF2 interacting domain) protein
TGME49_243600	-1.08	6.49	0.005649076	0.014671328	acetyltransferase, GNAT family protein
TGME49_270520	-1.26	8.13	0.005647379	0.014671328	hypothetical protein
TGME49_212100	-7.23	4.92	0.005669853	0.014720118	ThiF family protein
TGME49_247590	-1.86	5.36	0.005724946	0.01485272	methyltransferase domain-containing protein
TGME49_240500	-1.31	5.85	0.00572915	0.014858413	hypothetical protein
TGME49_226390	2.47	5.45	0.005809574	0.015045881	hypothetical protein
TGME49_300330	1.06	5.61	0.005816375	0.01505295	hypothetical protein
TGME49_202410	-1.34	6.11	0.005820374	0.015058029	hypothetical protein
TGME49_224490	-2.80	5.05	0.005825064	0.015064892	polyprenyl synthetase superfamily protein
TGME49_227350	-2.22	5.75	0.005837065	0.015090649	hypothetical protein
TGME49_267590	-1.10	6.55	0.005840107	0.015093238	hypothetical protein
TGME49_266930	1.09	5.42	0.005851612	0.015117687	general transcription factor IIH polypeptide 3 GTF2H3
TGME49_293490	1.85	4.86	0.005855144	0.015121527	hypothetical protein
TGME49_279390	-1.24	9.03	0.005877146	0.01517305	proliferation-associated protein 2G4, putative
TGME49_311140	-2.15	4.93	0.005887154	0.015193583	hypothetical protein
TGME49_259860	-1.21	6.06	0.005912938	0.015254803	hypothetical protein
TGME49_228380	2.83	5.03	0.005956046	0.01534995	hypothetical protein
TGME49_305010	-1.71	5.59	0.005970247	0.015381187	pre-mRNA branch site protein p14, putative
TGME49_244010	-1.83	5.45	0.006004327	0.015431349	hypothetical protein
TGME49_314800	-1.12	6.52	0.006022312	0.015472195	hypothetical protein
TGME49_255340	-1.08	6.51	0.00604864	0.015519643	tetratricopeptide repeat-containing protein
TGME49_213752	-1.67	5.38	0.006048845	0.015519643	herpesviridae ul52/ul70 dna primase
TGME49_226940	-2.78	5.08	0.006069586	0.015561205	ubiquitin carboxyl-terminal hydrolase
TGME49_273130	1.66	6.10	0.006076517	0.015573575	SAG-related sequence SRS30A
TGME49_300990	1.11	5.28	0.006125993	0.015678641	Toxoplasma gondii family C protein
TGME49_231220	-2.14	4.89	0.006174179	0.015785573	hypothetical protein
TGME49_223710	-1.04	6.49	0.006177889	0.015789599	tetratricopeptide repeat-containing protein
TGME49_285930	-1.73	5.78	0.006215596	0.015874996	hypothetical protein
TGME49_258380	-1.84	5.55	0.006268569	0.016004765	elongation factor p (ef-p) kow family domain-containing
TGME49_264420	3.44	4.97	0.006276742	0.016020101	lipoprotein, putative
TGME49_318360	-1.64	5.27	0.006298951	0.016071236	hypothetical protein
TGME49_263360	-1.63	5.69	0.006307988	0.016083194	WD domain, G-beta repeat-containing protein
TGME49_253600	1.18	6.68	0.006332704	0.016136641	hypothetical protein
TGME49_238140	-1.19	6.57	0.006336645	0.016139565	hypothetical protein
TGME49_219510	1.66	6.27	0.006353167	0.016174852	GTP binding protein 7 isoform 2 family protein, putative
TGME49_258100	-2.78	4.89	0.006354875	0.016174852	TPR repeat region protein
TGME49_221640	1.56	6.98	0.006427024	0.016335997	hypothetical protein
TGME49_217340	-1.32	5.87	0.006450196	0.016383631	hypothetical protein
TGME49_227390	-2.14	5.44	0.006487187	0.016471931	hypothetical protein
TGME49_251510	1.29	6.13	0.006557189	0.01663254	Ulp1 protease family, C-terminal catalytic domain-contai
TGME49_234470	-1.48	5.77	0.006570983	0.016656102	hypothetical protein
TGME49_314530	-2.77	5.09	0.006689001	0.016920449	RPAP1 family, C-terminal protein
TGME49_285480	1.45	5.95	0.006701261	0.016939872	hypothetical protein
TGME49_313530	-1.22	5.98	0.006807498	0.017179058	transmembrane protein 167, putative

TGME49_221280	-1.03	6.77	0.006867607	0.017301221	hypothetical protein
TGME49_314390	-1.37	5.75	0.006867318	0.017301221	hypothetical protein
TGME49_314710	-1.20	6.29	0.006919716	0.017408771	carrier superfamily protein
TGME49_217020	-2.23	6.25	0.006936656	0.017445453	ATPase, AFG1 family protein
TGME49_264670	-1.16	6.15	0.006944447	0.017458664	DNA polymerase family B protein
TGME49_273580	-1.81	5.41	0.006990913	0.017558019	hypothetical protein
TGME49_226690	-1.20	6.27	0.007047946	0.017695251	hypothetical protein
TGME49_267280	1.34	6.02	0.007085035	0.017776297	hypothetical protein
TGME49_236580	-1.15	7.51	0.007220133	0.018072331	Prp31-15.5k-U4 Snrna Complex family protein
TGME49_239070	-1.63	5.50	0.007215595	0.018072331	hypothetical protein
TGME49_238080	1.21	5.19	0.007234928	0.018103234	hypothetical protein
TGME49_203750	-1.83	5.36	0.007276781	0.018201799	hypothetical protein
TGME49_260270	-1.34	6.12	0.007281806	0.018208209	HEAT repeat-containing protein
TGME49_267855	-1.04	6.58	0.00729812	0.018242833	hypothetical protein
TGME49_217000	-1.36	5.87	0.007300814	0.018243399	hypothetical protein
TGME49_309280	-2.10	4.92	0.007313992	0.018270154	zinc finger, C3HC4 type (RING finger) domain-containing
TGME49_307270	-1.37	5.91	0.007326665	0.01829563	hypothetical protein
TGME49_211090	-1.06	6.64	0.007410821	0.018480813	aminotransferase, class V superfamily protein
TGME49_278550	-1.52	6.00	0.007435405	0.018529622	elongation factor Tu GTP binding domain-containing pro
TGME49_224160	1.03	5.52	0.007561617	0.018825116	hypothetical protein
TGME49_322010	-1.61	5.47	0.007572693	0.018840003	myosin-light-chain kinase
TGME49_233260	-1.52	5.63	0.007606155	0.018891472	GTP-binding protein, putative
TGME49_300180	-2.69	4.77	0.007632511	0.018950567	hypothetical protein
TGME49_307575	-2.70	5.16	0.007665618	0.019019993	hypothetical protein
TGME49_306250	1.16	5.14	0.007685984	0.019051347	hypothetical protein
TGME49_293500	1.06	5.56	0.007736005	0.019154644	hypothetical protein
TGME49_257500	1.33	5.05	0.007785241	0.019265084	hypothetical protein
TGME49_257070	-2.10	5.02	0.007800802	0.019297132	hypothetical protein
TGME49_252310	-1.27	6.41	0.007867963	0.019437259	hypothetical protein
TGME49_227820	-1.19	6.10	0.007873814	0.019438725	hypothetical protein
TGME49_249450	-2.70	4.73	0.007944109	0.01958611	hypothetical protein
TGME49_263785	1.23	5.14	0.008017627	0.019734465	phosphatidate cytidylyltransferase
TGME49_231040	-2.71	4.79	0.008020879	0.019735901	3' exoribonuclease family, domain 1 domain-containing
TGME49_270060	-1.61	5.79	0.008045679	0.019777177	hypothetical protein
TGME49_310330	-1.81	5.47	0.008099459	0.019882935	hypothetical protein
TGME49_224190	-1.15	6.40	0.008114431	0.019906471	cation-transporting atpase family protein
TGME49_233245	-2.09	4.98	0.008239428	0.02016628	hypothetical protein
TGME49_209190	-1.05	6.40	0.008275709	0.020239923	ABC transporter transmembrane region domain-contain
TGME49_310500	-1.17	6.13	0.008277729	0.020239923	hypothetical protein
TGME49_206710	2.02	5.64	0.00846752	0.020628943	hypothetical protein
TGME49_312520	-2.69	5.37	0.008726624	0.021225217	tRNA dimethylallyltransferase
TGME49_293680	-1.29	6.24	0.008731977	0.021231253	hypothetical protein
TGME49_228070	1.00	5.71	0.008749592	0.021267088	hypothetical protein
TGME49_258010	-1.64	5.57	0.008795724	0.021365173	calcium signaling protein kinase RAD53, putative
TGME49_239310	-2.12	5.36	0.008848195	0.021483789	ribulose 5-phosphate isomerase
TGME49_305870	-2.08	5.28	0.008862006	0.021497923	DAD family protein
TGME49_253930	-1.03	6.95	0.008874489	0.02151409	GCC2 and GCC3 domain-containing protein
TGME49_261370	1.03	7.02	0.008878339	0.021516367	hypothetical protein
TGME49_283730	-1.10	6.24	0.008897523	0.021555794	endomembrane protein 70 subfamily protein
TGME49_320070	-1.11	6.00	0.008912306	0.021584537	CDK-activating kinase assembly factor MAT1 protein
TGME49_214170	-1.46	5.69	0.008921877	0.021586505	hypothetical protein
TGME49_261960	1.20	5.17	0.008955942	0.021647674	hypothetical protein
TGME49_262590	1.57	5.43	0.008972055	0.021671974	hypothetical protein
TGME49_316760	1.08	5.65	0.008974788	0.021671974	hypothetical protein
TGME49_206700	-2.69	4.86	0.009053551	0.021840769	hypothetical protein
TGME49_308030	-1.36	5.86	0.009072786	0.021880033	hypothetical protein
TGME49_267550	-1.06	5.97	0.009077029	0.021882606	leucine-rich repeat protein LRR1

TGME49_267450	-1.07	6.26	0.009119981	0.021965192	alpha-tubulin suppressor protein
TGME49_232980	1.16	5.06	0.009127071	0.02197511	hypothetical protein
TGME49_242880	-1.76	5.25	0.009143607	0.022007756	flavoprotein
TGME49_283900	-1.47	5.67	0.009188801	0.022094958	ATPase, AAA family protein
TGME49_206490	-1.12	6.09	0.009197298	0.0221082	ICE family protease (caspase) p20 domain-containing pro
TGME49_262170	-1.76	5.41	0.009207199	0.022124408	hypothetical protein
TGME49_225710	-2.09	4.95	0.009354932	0.022457912	mitochondrial inner membrane translocase subunit TIM1
TGME49_320440	-1.56	6.19	0.00941382	0.02256997	hypothetical protein
TGME49_270170	-1.44	5.71	0.009435745	0.022607873	hypothetical protein
TGME49_219738	-1.15	6.44	0.009645081	0.023050709	hypothetical protein
TGME49_289110	1.52	4.69	0.009762676	0.023323176	hypothetical protein
TGME49_221700	-1.11	6.08	0.009795439	0.023393888	hypothetical protein
TGME49_220530	1.44	5.63	0.009890148	0.023589597	AP2 domain transcription factor AP2V-1
TGME49_316240	-1.32	6.03	0.010007351	0.02384607	hypothetical protein
TGME49_258940	1.21	5.05	0.010021846	0.023872916	acylphosphatase family protein
TGME49_225420	-2.04	5.08	0.010130913	0.024109422	histidine triad domain-containing protein
TGME49_238130	-1.24	5.86	0.010238929	0.024327328	hypothetical protein
TGME49_212290	-1.20	9.79	0.010265874	0.024375683	ribosomal protein RPS19
TGME49_288730	1.41	4.73	0.010296543	0.024409311	hypothetical protein
TGME49_313090	-1.77	5.25	0.010389157	0.024605198	hypothetical protein
TGME49_227270	1.71	5.63	0.010507728	0.024846224	hypothetical protein
TGME49_276910	-1.02	6.62	0.010517169	0.024856315	endoplasmic reticulum lumen protein retaining receptor
TGME49_203050	1.66	6.44	0.010541269	0.024892678	AP2 domain transcription factor AP2VIIa-6
TGME49_267460	-2.04	4.87	0.010544207	0.024892678	AP2 domain transcription factor AP2IX-1
TGME49_260840	-2.63	4.88	0.010597015	0.025001381	hypothetical protein
TGME49_315920	-1.75	5.33	0.010702143	0.025217223	DNA-directed RNA polymerase II RPB11A
TGME49_314030	1.50	5.75	0.010812058	0.025468096	hypothetical protein
TGME49_311740	-1.75	5.52	0.010949498	0.025767214	hypothetical protein
TGME49_201810	1.07	7.33	0.010975942	0.025813013	hypothetical protein
TGME49_275770	-1.15	6.31	0.011052409	0.025984583	hypothetical protein
TGME49_205550	-1.54	5.44	0.011063956	0.026003461	AGC kinase
TGME49_277820	-2.05	5.23	0.011069101	0.026007288	hypothetical protein
TGME49_294730	-1.19	6.29	0.011085839	0.02603834	hypothetical protein
TGME49_271770	-2.03	5.22	0.011099819	0.026057766	hypothetical protein
TGME49_232310	-1.73	5.22	0.011134839	0.026111958	endonuclease/exonuclease/phosphatase family protein
TGME49_248180	2.57	4.71	0.011147466	0.02613328	hypothetical protein
TGME49_320720	-2.05	5.10	0.011161357	0.02615755	hypothetical protein
TGME49_278250	1.10	5.20	0.01119186	0.02619694	hypothetical protein
TGME49_284645	-1.55	5.66	0.011211243	0.02622459	hypothetical protein
TGME49_276100	-1.58	5.52	0.011225391	0.02624938	hypothetical protein
TGME49_298010	-1.13	7.06	0.011397131	0.026625715	hypothetical protein
TGME49_253960	1.17	4.75	0.011408628	0.026644156	oxidoreductase, short chain dehydrogenase/reductase fa
TGME49_295680	-1.29	5.74	0.011458157	0.026751377	periodic tryptophan protein PWP2, putative
TGME49_293220	1.85	5.72	0.011659085	0.027177578	DHHC zinc finger domain-containing protein
TGME49_202350	-1.39	5.81	0.011751789	0.02737641	50S ribosomal protein L21, putative
TGME49_217410	-6.96	4.73	0.011761556	0.027390535	hypothetical protein
TGME49_305930	1.10	4.79	0.011792646	0.027445649	hypothetical protein
TGME49_215250	-1.72	5.32	0.011817205	0.027494152	thiamin pyrophosphokinase, catalytic domain-containing
TGME49_220150	-1.72	5.32	0.011848308	0.027549178	50S ribosomal protein L16, putative
TGME49_306590	1.47	6.35	0.011914138	0.027684833	hypothetical protein
TGME49_269050	-1.21	5.62	0.011925212	0.027701858	hypothetical protein
TGME49_257560	1.25	5.00	0.011938884	0.027724907	WD domain, G-beta repeat-containing protein
TGME49_300980	-1.20	5.77	0.012036624	0.027877625	hypothetical protein
TGME49_207720	-1.20	6.17	0.012046618	0.027887506	hypothetical protein
TGME49_297910	-2.63	5.66	0.012067881	0.027927984	hypothetical protein
TGME49_234370	-2.11	5.10	0.012077349	0.027941151	SAG-related sequence SRS42
TGME49_268250	-1.39	5.54	0.012097867	0.027979865	WD domain, G-beta repeat-containing protein

TGME49_278890	-1.74	5.41	0.012141262	0.028045139	hypothetical protein
TGME49_275740	-2.66	4.73	0.012226399	0.028224163	hypothetical protein
TGME49_219620	1.14	5.27	0.012407713	0.028580259	hypothetical protein
TGME49_305040	-1.59	6.83	0.012414354	0.028586651	HEAT repeat-containing protein
TGME49_213930	-1.72	5.15	0.012475984	0.028710683	3' exoribonuclease family, domain 1 domain-containing
TGME49_235905	-2.00	5.09	0.01249267	0.028731198	ribonuclease z, putative
TGME49_234560	-1.08	6.18	0.012504353	0.028749125	hypothetical protein
TGME49_260150	1.15	4.92	0.012522452	0.028772843	tetratricopeptide repeat-containing protein
TGME49_213635	-1.02	5.25	0.012645213	0.029036864	hypothetical protein
TGME49_214310	1.60	5.53	0.012668421	0.029081125	hypothetical protein
TGME49_223725	2.08	5.42	0.012841107	0.029431848	hypothetical protein
TGME49_204040	-2.00	4.98	0.012876316	0.029503403	hypothetical protein
TGME49_320470	1.79	5.10	0.012926985	0.029582832	hypothetical protein
TGME49_295000	-1.23	5.89	0.012945757	0.029607464	hypothetical protein
TGME49_294750	-2.55	4.76	0.013076128	0.029868671	hypothetical protein
TGME49_309880	-1.04	6.92	0.013110544	0.029928794	hypothetical protein
TGME49_232590	1.37	6.36	0.01318822	0.030087535	glutamate-cysteine ligase, catalytic subunit domain-cont
TGME49_246610	-2.04	4.95	0.013234632	0.030174799	hypothetical protein
TGME49_236130	1.59	6.02	0.013314842	0.030306159	signal recognition particle (SRP9) domain-containing pro
TGME49_316770	-1.37	5.68	0.013320938	0.030306159	undecaprenyl diphosphate synthase
TGME49_254480	1.36	5.93	0.013655034	0.030951969	WD domain, G-beta repeat-containing protein
TGME49_287220	1.87	6.36	0.013696759	0.031027523	hypothetical protein
TGME49_297060	-1.30	8.50	0.013715625	0.031059032	phosphoglycerate mutase PGMII
TGME49_202730	-1.71	5.45	0.013858548	0.031346009	hypothetical protein
TGME49_216040	-1.19	6.01	0.013894074	0.031416753	30S ribosomal protein S15, putative
TGME49_202230	-1.18	5.96	0.014010045	0.031640279	histone deacetylase HDAC5
TGME49_226090	-2.55	5.05	0.014045275	0.031700476	DEAD/DEAH box helicase domain-containing protein
TGME49_206300	-1.99	5.15	0.014111333	0.031820432	hypothetical protein
TGME49_243240	1.26	4.74	0.014155343	0.031890496	WD domain, G-beta repeat-containing protein
TGME49_203860	1.40	4.73	0.014210759	0.032005589	hypothetical protein
TGME49_246010	2.37	5.31	0.014232542	0.032035133	hypothetical protein
TGME49_213690	-1.36	5.66	0.014228933	0.032035133	ring box protein 1 family protein
TGME49_305620	-1.31	5.96	0.014246746	0.032047594	hypothetical protein
TGME49_263740	1.97	5.66	0.014339478	0.032236576	ABC transporter transmembrane region domain-contain
TGME49_215450	1.91	6.55	0.014403413	0.03236063	aquaporin 1
TGME49_290840	-1.05	6.35	0.014577841	0.032712021	serine protease
TGME49_251590	1.10	5.99	0.014664576	0.032877465	hypothetical protein
TGME49_261430	-1.36	5.96	0.014698674	0.032933937	hypothetical protein
TGME49_277850	-1.63	5.67	0.014857828	0.033240175	trypsin domain-containing protein
TGME49_309420	-1.37	5.59	0.015145052	0.033821356	ferlin family protein
TGME49_299040	-1.13	5.81	0.015229029	0.033998622	phosphatidylinositol n-acetylglucosaminyltransferase
TGME49_254810	1.22	4.83	0.015403827	0.034347376	hypothetical protein
TGME49_214400	-1.96	5.11	0.015417056	0.03436651	hypothetical protein
TGME49_257980	2.38	4.93	0.015461201	0.034444146	ribosome recycling factor protein
TGME49_209290	-1.03	9.66	0.015585329	0.034647602	ribosomal protein RPS28
TGME49_233770	-2.20	6.27	0.01560608	0.034683305	calcium-translocating P-type ATPase, PMCA-type protein
TGME49_211480	-1.47	5.45	0.01564151	0.034751601	GTP-binding protein engA, putative
TGME49_246690	-1.66	5.40	0.015650476	0.034761075	alpha amylase, catalytic domain-containing protein
TGME49_212735	-1.98	5.29	0.015944788	0.035340457	hypothetical protein
TGME49_232530	-1.66	5.46	0.016152216	0.03576804	hypothetical protein
TGME49_275420	-1.12	6.42	0.016284843	0.036029362	histone lysine-specific demethylase LSD1/BHC110/KDMA
TGME49_291120	-1.24	5.68	0.016369361	0.036205521	trafficking protein mon1 subfamily protein
TGME49_290240	1.41	5.69	0.01645498	0.036373132	hypothetical protein
TGME49_313470	1.15	5.06	0.016453301	0.036373132	helix-hairpin-helix motif domain-containing protein
TGME49_235700	-1.05	6.40	0.01647746	0.036411936	sedoheptulose-1,7-bisphosphatase
TGME49_201240	1.15	5.40	0.016562762	0.036589502	hypothetical protein
TGME49_289130	-1.94	4.92	0.016631801	0.036694228	hypothetical protein

TGME49_309940	-1.26	5.91	0.016716711	0.036841538	phospholipase D active site domain-containing protein
TGME49_205100	-1.46	5.29	0.016715129	0.036841538	hypothetical protein
TGME49_248840	-1.42	5.56	0.01672732	0.036853934	transporter, major facilitator family protein
TGME49_218610	-1.51	5.51	0.016784015	0.036943792	ATPase (DUF699) protein
TGME49_211300	-1.66	5.07	0.01678809	0.036943792	DUF74 family protein, putative
TGME49_268010	-1.15	5.82	0.016880389	0.037113767	hypothetical protein
TGME49_289950	-1.01	6.53	0.016964082	0.037264988	hypothetical protein
TGME49_218230	-1.33	5.66	0.016964289	0.037264988	histone lysine methyltransferase, SET, putative
TGME49_201770	1.42	6.50	0.017120668	0.037563863	cullin 3, putative
TGME49_286640	-2.49	4.82	0.017146195	0.037597558	GTPase
TGME49_231890	-1.25	6.02	0.017190371	0.03768325	beta-ketoacyl-acyl carrier protein synthase III, putative
TGME49_275568	-1.11	6.27	0.017297838	0.037885135	GPI transamidase subunit PIG-U protein
TGME49_308590	-1.13	6.01	0.017441194	0.038153901	Mov34/MPN/PAD-1 family protein
TGME49_249710	-1.46	5.23	0.017557107	0.038384758	Parkinson disease 7 domain containing 1 family protein
TGME49_262700	1.22	6.44	0.017644465	0.038552947	tetratricopeptide repeat-containing protein
TGME49_219230	-1.11	6.00	0.017869789	0.038987671	AMP-binding enzyme domain-containing protein
TGME49_210750	1.78	5.09	0.01798071	0.039194978	Ferredoxin-fold anticodon binding domain-containing pr
TGME49_320700	1.09	5.08	0.018071285	0.039348705	AP2 domain transcription factor AP2IV-1
TGME49_210440	1.04	6.10	0.018230693	0.039634737	polynucleotide adenylyltransferase
TGME49_210310	-1.63	5.16	0.018385376	0.039924073	hypothetical protein
TGME49_286130	1.11	4.91	0.01842217	0.039982594	hypothetical protein
TGME49_249490	-1.08	5.82	0.018423197	0.039982594	hypothetical protein
TGME49_277500	-1.47	7.54	0.018686715	0.040471462	26S proteasome regulatory subunit 7, putative
TGME49_225190	2.25	4.76	0.018718889	0.040509941	hypothetical protein
TGME49_270130	1.08	5.21	0.018720918	0.040509941	hypothetical protein
TGME49_271240	-1.91	5.16	0.018882859	0.040836462	hypothetical protein
TGME49_298610	-1.04	8.23	0.018892573	0.040845523	GYF domain-containing protein
TGME49_237100	1.06	5.16	0.018956085	0.040958882	RAP domain-containing protein
TGME49_250955	1.38	7.35	0.019082296	0.041195474	KRUF family protein
TGME49_258800	1.07	5.25	0.01918682	0.041384873	rhoptry kinase family protein ROP31
TGME49_293700	-1.14	6.01	0.019367187	0.041713074	WD domain, G-beta repeat-containing protein
TGME49_263750	-1.31	5.91	0.019468979	0.041895702	hypothetical protein
TGME49_226350	1.01	5.21	0.01950323	0.041944993	hypothetical protein
TGME49_263190	-2.46	4.74	0.019652068	0.042179214	adenylosuccinate lyase, putative
TGME49_285720	-1.90	4.96	0.019685178	0.042225764	ATP binding protein, putative
TGME49_240950	-2.50	4.75	0.019699653	0.042244557	hypothetical protein
TGME49_235450	1.02	7.07	0.019782783	0.042385946	ubiquitin-conjugating enzyme subfamily protein
TGME49_284040	-1.16	6.15	0.019900935	0.042614398	hypothetical protein
TGME49_313070	-1.21	5.58	0.019995093	0.042803624	hypothetical protein
TGME49_265510	-1.01	7.43	0.020014028	0.042831757	hypothetical protein
TGME49_218000	-1.61	5.14	0.020072461	0.042931957	hypothetical protein
TGME49_258650	-1.90	5.11	0.020100041	0.04296609	protoheme ferro-lyase, putative
TGME49_320680	1.44	4.90	0.020243193	0.043259586	AP2 domain transcription factor AP2IV-2
TGME49_269430	1.14	5.08	0.020259157	0.043281193	polyprenyl synthetase superfamily protein
TGME49_222430	-1.21	5.65	0.020492809	0.043701531	HECT-domain (ubiquitin-transferase) domain-containing
TGME49_255195	1.10	5.32	0.020578477	0.043861995	hypothetical protein
TGME49_272290	-1.01	6.44	0.020672846	0.044012387	pyruvate dehydrogenase complex subunit PD-HE1Beta
TGME49_255170	-2.43	5.01	0.020667469	0.044012387	hypothetical protein
TGME49_202220	1.47	6.78	0.021008272	0.04463654	hypothetical protein
TGME49_238190	-1.01	5.98	0.021064486	0.044743122	DNA-directed RNA polymerase II RPB3
TGME49_319710	-1.07	6.22	0.02108776	0.044779694	kinesin motor domain-containing protein
TGME49_262460	-1.19	5.69	0.021170129	0.044902939	hypothetical protein
TGME49_320610	-1.92	5.08	0.021210211	0.04494135	hypothetical protein
TGME49_293350	-1.06	6.08	0.021529314	0.04558641	mitochondrial carrier superfamily protein
TGME49_239748	-1.39	5.45	0.021566669	0.045639368	hypothetical protein
TGME49_262760	-1.21	5.46	0.021598411	0.045674593	poly(ADP-ribose) glycohydrolase
TGME49_270975	-1.36	5.71	0.021599111	0.045674593	hypothetical protein

TGME49_243590	-1.45	5.49	0.021996256	0.046389137	endonuclease/exonuclease/phosphatase family protein
TGME49_219100	-1.28	5.69	0.022059869	0.04649677	cyclin-dependent kinase regulatory subunit protein
TGME49_288240	1.21	4.81	0.022103032	0.046561201	hypothetical protein
TGME49_231930	-2.46	4.73	0.022200839	0.046716919	hypothetical protein
TGME49_300320	2.19	4.83	0.022255032	0.046814709	dimethyladenosine transferase
TGME49_263440	-1.58	5.39	0.02226183	0.046815692	hypothetical protein
TGME49_212770	-1.33	8.99	0.022369286	0.047001562	hypothetical protein
TGME49_294420	-1.86	4.78	0.022397415	0.047033936	programmed cell death protein 2, c-terminal domain-co
TGME49_238940	1.18	4.73	0.022625175	0.047471777	GDP mannose 4,6-dehydratase, putative
TGME49_200460	1.17	6.56	0.022731589	0.047654484	hypothetical protein
TGME49_215480	-1.40	5.60	0.02278965	0.047749127	Adenosine/AMP deaminase domain-containing protein
TGME49_231870	-1.59	5.18	0.022906131	0.047952415	tetratricopeptide repeat-containing protein
TGME49_269000	-1.62	5.10	0.02293122	0.04797777	ABC transporter family protein
TGME49_218960	-1.07	8.73	0.023137567	0.048382117	AP2 domain transcription factor AP2XII-1
TGME49_219682	-1.11	5.84	0.023252391	0.048581005	pyruvate dehydrogenase kinase, putative
TGME49_230990	1.09	5.02	0.023318609	0.048691836	hypothetical protein
TGME49_285670	1.81	4.94	0.023348288	0.048726288	hypothetical protein
TGME49_244412	-1.85	5.00	0.023357433	0.04873162	hypothetical protein
TGME49_318320	-1.88	5.00	0.023433525	0.048849023	ATP-dependent Clp endopeptidase, proteolytic subunit C
TGME49_313710	-1.22	5.42	0.023562649	0.049076684	hypothetical protein
TGME49_263580	1.27	6.93	0.024014182	0.049876647	bromodomain-containing protein
TGME49_220330	-2.44	5.33	0.024066728	0.049971746	hypothetical protein

Supplementary Table S8A: Differential expression analysis of human miRNA genes from MM6 cells infected with T. gondii EGS strain for 18 hours.

gene_ID	logFC (cont-inf)	logCPM	PValue	FDR
hsa-mir-708-5p	-11.46	5.68	6.11E-22	2.54E-18
hsa-mir-708-3p	-10.28	4.56	7.83E-16	1.63E-12
hsa-mir-29b-3p	-5.46	5.99	1.85E-15	2.56E-12
ENST00000474173	-9.95	4.25	7.09E-15	7.34E-12
hsa-mir-32-5p	-5.48	7.42	8.85E-15	7.34E-12
hsa-mir-142-3p	-4.62	10.47	1.77E-13	1.23E-10
ENST00000500868	-6.60	3.74	5.53E-12	3.28E-09
ENST00000500633	-6.57	3.71	7.75E-12	4.02E-09
hsa-mir-3656-3p	-9.66	4.01	2.28E-11	1.05E-08
ENST00000499697	-5.03	4.03	2.64E-10	9.97E-08
ENST00000498989	-4.61	4.40	3.78E-10	1.25E-07
ENST00000500011	-5.20	4.40	1.88E-09	5.57E-07
hsa-mir-210-5p	-7.79	2.35	3.12E-09	8.55E-07
hsa-mir-34c-5p	-7.76	2.32	3.30E-09	8.55E-07
hsa-mir-218-5p	-5.28	2.58	3.92E-09	9.56E-07
ENST00000501638	-4.61	3.26	4.16E-09	9.59E-07
hsa-mir-212-3p	-5.02	3.17	5.03E-09	1.05E-06
ENST00000500409	-5.57	2.86	5.06E-09	1.05E-06
ENST00000501230	-4.65	3.71	7.25E-09	1.43E-06
hsa-mir-10b-5p	-5.43	2.74	1.33E-08	2.25E-06
ENST00000384148	3.83	1.75	2.20E-08	3.26E-06
ENST00000500340	-4.55	2.74	2.96E-08	3.96E-06
ENST00000487032	-4.88	2.26	3.20E-08	4.15E-06
hsa-mir-3179-3p	3.39	1.65	8.68E-08	9.73E-06
ENST00000363443	-4.61	2.04	9.67E-08	1.06E-05
ENST00000499746	-4.64	2.07	1.12E-07	1.19E-05
ENST00000364915	4.86	11.05	1.24E-07	1.24E-05
ENST00000471237	1.78	16.61	1.29E-07	1.24E-05
ENST00000489413	5.42	1.52	1.41E-07	1.33E-05
hsa-mir-362-3p	-4.94	2.33	2.10E-07	1.92E-05
ENST00000364931	3.72	6.32	2.13E-07	1.92E-05
ENST00000363549	4.96	1.17	2.22E-07	1.96E-05

ENST00000500066	-4.75	2.17	2.66E-07	2.25E-05
ENST00000500656	3.30	4.35	2.75E-07	2.28E-05
ENST00000500845	-4.48	1.94	3.69E-07	2.89E-05
ENST00000363367	2.99	2.77	4.29E-07	3.23E-05
ENST00000501386	-4.68	2.11	5.39E-07	3.92E-05
ENST00000498132	2.95	1.89	5.54E-07	3.96E-05
ENST00000499637	2.82	1.63	8.91E-07	5.87E-05
ENST00000458848	2.94	2.18	1.63E-06	9.93E-05
ENST00000502220	2.83	1.96	2.99E-06	0.0001723
ENST00000470561	2.84	8.18	4.97E-06	0.0002679
ENST00000459424	2.71	2.93	6.26E-06	0.0003248
ENST00000408564	2.78	2.87	6.70E-06	0.0003431
ENST00000365574	2.88	5.75	7.62E-06	0.0003519
ENST00000384314	2.96	3.87	7.63E-06	0.0003519
ENST00000364572	3.27	1.33	8.11E-06	0.0003656
ENST00000384273	3.21	1.72	8.73E-06	0.0003814
ENST00000501131	5.23	5.04	9.32E-06	0.000394
hsa-mir-1304-5p	2.62	2.68	9.93E-06	0.0004078
ENST00000365467	2.57	3.28	1.05E-05	0.0004232
ENST00000463508	1.59	16.21	1.08E-05	0.0004319
ENST00000384084	2.72	3.95	1.11E-05	0.0004391
ENST00000364784	2.55	3.09	1.16E-05	0.0004549
ENST00000384106	3.24	3.11	1.19E-05	0.0004563
ENST00000362808	3.01	11.12	1.20E-05	0.0004563
ENST00000362881	3.14	2.39	1.26E-05	0.0004768
ENST00000363299	2.58	6.05	1.31E-05	0.0004866
ENST00000383873	2.84	3.41	1.31E-05	0.0004866
ENST00000363250	3.36	2.81	1.46E-05	0.0005371
ENST00000384741	2.98	1.58	1.50E-05	0.0005435
ENST00000384743	2.92	4.02	1.51E-05	0.0005435
ENST00000364542	2.73	4.11	1.58E-05	0.0005654
hsa-mir-4301-5p	3.54	2.26	1.59E-05	0.0005654
ENST00000365439	2.70	1.55	1.66E-05	0.0005789
ENST00000384650	2.72	1.87	1.68E-05	0.0005825
ENST00000384547	3.00	1.79	1.77E-05	0.0006058
ENST00000362507	2.88	6.90	1.88E-05	0.0006278
ENST00000384413	2.70	7.24	2.12E-05	0.0007023
hsa-mir-942-5p	2.48	4.42	2.27E-05	0.0007362
ENST00000362423	2.25	6.06	2.49E-05	0.0007891
hsa-mir-33a-3p	-4.35	1.87	2.51E-05	0.0007891
ENST00000499204	-6.41	1.23	2.55E-05	0.0007906
ENST00000500894	2.54	7.69	2.68E-05	0.0008182

ENST00000499768	-6.36	1.19	2.82E-05	0.0008545
ENST00000384172	2.79	3.84	2.90E-05	0.000865
ENST00000476850	3.02	3.81	2.92E-05	0.000865
ENST00000362477	2.74	13.63	3.17E-05	0.0009074
ENST00000384585	3.45	6.77	3.27E-05	0.0009279
ENST00000500576	-6.69	1.43	3.39E-05	0.000958
ENST00000458981	3.08	3.16	3.44E-05	0.0009647
ENST00000408749	2.41	5.01	3.50E-05	0.0009737
ENST00000384007	2.81	4.74	3.52E-05	0.0009749
ENST00000387943	2.38	5.02	3.61E-05	0.0009924
ENST00000364234	2.39	2.77	3.71E-05	0.0010067
ENST00000365537	3.18	2.97	3.80E-05	0.0010144
ENST00000363696	2.36	2.11	4.03E-05	0.0010644
ENST00000365037	-6.31	1.16	4.31E-05	0.0011
ENST00000365606	3.07	1.16	4.32E-05	0.0011
ENST00000365312	3.38	2.98	4.35E-05	0.0011
ENST00000384637	2.86	3.52	4.48E-05	0.0011239
ENST00000365666	3.03	4.03	4.50E-05	0.0011239
ENST00000384178	2.34	5.44	4.67E-05	0.0011609
ENST00000485528	2.82	3.05	4.76E-05	0.0011751
ENST00000362698	2.30	12.33	4.79E-05	0.0011753
ENST00000384245	2.57	3.44	4.98E-05	0.0012036
ENST00000363444	2.65	7.42	4.99E-05	0.0012036
ENST00000363462	2.66	4.23	4.99E-05	0.0012036
ENST00000384205	2.59	3.84	5.48E-05	0.0012915
ENST00000499762	2.79	1.21	5.81E-05	0.0013467
ENST00000459194	2.52	2.10	5.89E-05	0.0013579
ENST00000383948	2.63	3.36	6.08E-05	0.0013926
ENST00000364628	2.67	4.54	6.38E-05	0.0014552
ENST00000384604	2.89	3.49	6.52E-05	0.0014783
ENST00000364358	2.72	3.93	6.81E-05	0.0015182
ENST00000365484	2.58	8.74	6.96E-05	0.0015211
ENST00000501173	2.59	1.48	6.99E-05	0.0015211
ENST00000384619	2.32	9.62	7.04E-05	0.0015211
ENST00000384530	3.09	2.72	7.47E-05	0.001581
ENST00000481391	2.67	1.15	7.63E-05	0.0016026
ENST00000363977	3.09	3.04	7.66E-05	0.0016026
ENST00000501074	5.20	8.31	7.69E-05	0.0016026
ENST00000501998	3.08	1.16	8.08E-05	0.00166
ENST00000365599	2.93	3.79	8.14E-05	0.0016637
hsa-mir-3173-5p	2.67	1.14	8.40E-05	0.0016924
ENST00000384026	2.70	1.36	8.56E-05	0.0017041

ENST00000384385	2.46	2.58	8.58E-05	0.0017041
ENST00000411404	2.64	8.90	9.14E-05	0.0017911
hsa-mir-146b-5p	2.01	12.84	9.34E-05	0.0018116
ENST00000384577	2.97	2.85	9.60E-05	0.001844
hsa-mir-1254-5p	2.22	3.39	0.0001007	0.0018999
ENST00000410462	2.41	1.79	0.0001039	0.0019415
ENST00000384161	2.31	2.87	0.0001054	0.0019618
ENST00000383898	2.46	3.75	0.0001076	0.0019838
ENST00000363435	2.44	2.13	0.0001105	0.0020237
ENST00000363286	2.14	12.35	0.0001112	0.0020238
ENST00000482497	3.89	10.49	0.0001151	0.0020759
ENST00000365604	2.82	2.38	0.0001195	0.0021444
ENST00000362591	2.21	5.66	0.0001205	0.0021444
hsa-mir-31-5p	-6.76	1.45	0.0001209	0.0021444
ENST00000383895	5.35	8.30	0.0001228	0.0021489
ENST00000365341	2.54	4.27	0.0001283	0.0022322
ENST00000365532	2.51	2.05	0.0001291	0.0022322
ENST00000387069	2.28	10.27	0.0001303	0.0022429
ENST00000362352	2.27	5.66	0.0001356	0.0023063
ENST00000364102	2.41	4.33	0.0001427	0.0024074
ENST00000384633	2.71	4.02	0.0001506	0.0025095
ENST00000384630	2.71	1.39	0.0001514	0.0025119
ENST00000384563	2.65	3.93	0.0001636	0.0026718
ENST00000363636	2.17	6.49	0.0001715	0.0027621
ENST00000362353	2.17	5.69	0.0001718	0.0027621
ENST00000362562	2.16	2.20	0.0001719	0.0027621
ENST00000487060	2.35	5.14	0.0001724	0.0027621
ENST00000363624	2.11	6.45	0.0001793	0.0028611
ENST00000384323	2.02	5.69	0.0001815	0.0028761
ENST00000411187	2.82	1.27	0.0001816	0.0028761
ENST00000384589	2.61	1.31	0.0001927	0.0030163
ENST00000365085	2.20	6.38	0.0001934	0.0030167
ENST00000364164	2.44	4.25	0.0001943	0.0030199
hsa-mir-146b-3p	2.32	4.32	0.0001994	0.0030812
ENST00000384781	2.80	3.49	0.0001998	0.0030812
ENST00000363190	2.86	1.22	0.0002153	0.0032843
ENST00000384744	2.04	5.80	0.0002252	0.0034226
ENST00000384776	2.49	3.60	0.000226	0.0034228
ENST00000391154	2.11	4.71	0.00023	0.0034454
ENST00000362862	2.23	5.53	0.0002329	0.0034753
ENST00000391172	2.23	3.69	0.0002345	0.0034753
ENST00000365160	2.64	7.65	0.0002383	0.0035061

ENST00000459189	2.04	6.57	0.0002421	0.0035498
ENST00000497848	1.92	5.70	0.0002484	0.0036284
ENST00000384527	2.48	3.24	0.0002498	0.0036349
ENST00000499508	1.98	6.37	0.0002506	0.0036349
ENST00000496399	1.70	13.26	0.0002687	0.0038713
ENST00000384281	2.15	4.62	0.0002773	0.0039137
ENST00000364641	2.38	4.28	0.000281	0.0039162
ENST00000384534	2.34	2.67	0.0002879	0.0039817
ENST00000383934	2.58	1.80	0.0003333	0.0045491
ENST00000362710	2.40	5.26	0.000339	0.0045843
ENST00000363455	2.11	2.31	0.0003394	0.0045843
ENST00000384793	2.24	3.73	0.0003403	0.0045843
ENST00000363899	2.14	5.53	0.0003746	0.004966
ENST00000383924	1.91	5.24	0.0003812	0.0050364
ENST00000362354	2.22	5.16	0.0003834	0.0050398
ENST00000365075	2.18	1.63	0.0003851	0.0050398
ENST00000384001	2.22	5.03	0.0003899	0.0050717
ENST00000384127	2.63	3.80	0.0003946	0.0051001
ENST00000410624	2.16	5.29	0.0003967	0.0051119
ENST00000364337	2.57	2.66	0.0004074	0.0052336
ENST00000411164	2.03	2.29	0.0004103	0.005249
ENST00000384716	2.18	3.81	0.0004112	0.005249
ENST00000383913	2.74	3.59	0.0004317	0.0054945
ENST00000364432	3.07	7.17	0.0004369	0.0055429
ENST00000459107	2.22	5.01	0.0004416	0.0055862
ENST00000500112	2.04	3.34	0.0004462	0.0056099
ENST00000384341	2.05	5.74	0.000465	0.0058116
ENST00000363372	2.21	1.51	0.000468	0.0058242
ENST00000471506	2.17	5.04	0.0004689	0.0058242
ENST00000362697	2.12	5.23	0.0005105	0.006267
hsa-mir-4521-5p	2.09	1.45	0.0005336	0.0064929
ENST00000384750	2.11	5.05	0.000558	0.0067296
ENST00000486830	2.68	13.86	0.0005653	0.0067989
hsa-mir-4420-3p	1.97	2.91	0.0005922	0.0070811
ENST00000365498	2.16	5.14	0.0006026	0.0071847
ENST00000384238	2.17	3.84	0.0006255	0.0073349
ENST00000500700	1.82	1.81	0.0006288	0.0073495
ENST00000364950	2.12	7.04	0.0006372	0.007385
ENST00000365663	3.48	3.32	0.000653	0.0074961
ENST00000408175	2.02	1.50	0.0006575	0.0075148
ENST00000384483	2.68	3.57	0.0006783	0.0077308
ENST00000364596	2.10	4.66	0.0006801	0.0077308

ENST00000365625	2.13	4.98	0.0006898	0.0078191
ENST00000384388	2.78	2.88	0.0007239	0.0081465
ENST00000384240	2.13	5.08	0.0007291	0.0081465
ENST00000502243	2.40	2.84	0.0007319	0.0081465
ENST00000365462	2.09	4.61	0.000769	0.0085088
ENST00000411366	2.08	5.27	0.0007691	0.0085088
ENST00000383919	2.03	1.72	0.000781	0.0086185
ENST00000459329	2.14	2.25	0.0007854	0.0086438
ENST00000408454	1.87	4.71	0.0008068	0.0088553
hsa-mir-1273h-5p	2.08	2.01	0.0008308	0.0090949
ENST00000363894	2.14	4.38	0.0008678	0.0094501
hsa-mir-92a-5p	1.99	8.24	0.0008703	0.0094528
ENST00000365197	2.56	3.58	0.0008823	0.0095327
ENST00000363640	2.02	6.62	0.0008906	0.0095974
ENST00000365068	1.99	5.32	0.000915	0.0098096
ENST00000362915	3.36	6.13	0.0009176	0.0098123
ENST00000363651	1.99	5.89	0.0009352	0.0099746
ENST00000384504	1.94	1.68	0.0009458	0.0100618
ENST00000498916	3.80	3.58	0.0009858	0.0103811
ENST00000363674	2.04	5.08	0.0009923	0.0103969
ENST00000384254	2.62	2.88	0.0010217	0.0106237
ENST00000499152	1.88	1.55	0.0010301	0.0106851
ENST00000363985	1.92	5.62	0.0010378	0.0107378
ENST00000365487	2.30	3.05	0.0010621	0.0109075
hsa-mir-1273h-3p	1.87	4.25	0.0010801	0.0110455
ENST00000363248	2.04	5.29	0.0010815	0.0110455
hsa-mir-183-5p	1.81	5.24	0.0010872	0.011056
ENST00000384248	2.25	3.49	0.0011232	0.0113386
ENST00000383994	2.09	4.44	0.0011323	0.0114027
ENST00000384677	1.85	2.81	0.0011695	0.0116925
ENST00000500169	2.74	1.37	0.0011887	0.0117984
ENST00000362412	2.38	4.58	0.0012274	0.0121456
ENST00000384376	2.59	2.60	0.0012546	0.0123083
ENST00000384762	2.44	5.43	0.0012549	0.0123083
ENST00000364805	2.04	8.43	0.0012887	0.0125517
ENST00000362918	2.03	5.27	0.0013057	0.0126762
ENST00000384373	2.13	4.43	0.00132	0.0127072
ENST00000364407	2.02	4.57	0.0013457	0.0128945
ENST00000458898	2.05	2.30	0.0013522	0.0129274
ENST00000470693	1.94	1.36	0.0013741	0.0130757
ENST00000390872	1.95	6.00	0.0013855	0.0131543
ENST00000363389	1.84	5.70	0.0014162	0.013385

ENST00000363880	1.92	5.98	0.00145	0.0136418
hsa-mir-191-3p	1.68	5.21	0.0015924	0.0148473
ENST00000384627	2.16	3.82	0.0016033	0.014915
ENST00000410868	1.82	1.74	0.0016209	0.015045
ENST00000384268	1.96	5.34	0.0016406	0.0151301
ENST00000362512	2.63	9.09	0.0016762	0.0154202
ENST00000410494	1.79	2.26	0.0016898	0.0155106
ENST00000384753	1.94	5.31	0.0017086	0.0156489
ENST00000384087	1.79	4.86	0.0017426	0.0158904
ENST00000363963	1.78	5.25	0.001775	0.0160448
ENST00000384708	1.86	2.25	0.0017866	0.0161144
ENST00000483314	3.27	7.01	0.0018051	0.0162462
ENST00000362692	1.94	2.67	0.0018537	0.01654
ENST00000461728	3.29	7.01	0.001865	0.0165924
ENST00000458930	1.80	10.16	0.0018705	0.0165924
ENST00000363872	1.98	4.51	0.0018816	0.0165924
ENST00000408082	1.82	4.86	0.001883	0.0165924
ENST00000364018	1.83	5.45	0.0018913	0.0165924
ENST00000458932	1.93	2.16	0.0018916	0.0165924
ENST00000390904	3.45	4.12	0.0019491	0.0168498
ENST00000384416	1.69	6.58	0.0019807	0.0170142
ENST00000363836	2.07	4.28	0.0020137	0.0172264
ENST00000363171	1.93	5.36	0.0020407	0.0173662
ENST00000384078	1.98	2.65	0.0020465	0.0173662
ENST00000384737	2.39	3.21	0.0020468	0.0173662
ENST00000484776	2.17	7.85	0.0020528	0.0173821
ENST00000468731	2.40	3.88	0.00213	0.017999
ENST00000391033	1.73	5.23	0.002144	0.0180801
ENST00000471029	2.01	4.44	0.0021502	0.0180959
ENST00000384297	1.86	5.39	0.0021792	0.0183023
ENST00000364879	1.71	5.07	0.0021945	0.0183566
ENST00000384653	1.89	5.36	0.0022483	0.018703
ENST00000383874	2.41	2.64	0.0023289	0.0193048
ENST00000363331	2.14	7.90	0.0023727	0.0196103
ENST00000384749	2.15	1.96	0.0023963	0.0197269
ENST00000480363	2.28	4.80	0.0024127	0.019822
ENST00000384067	1.68	6.67	0.0024722	0.0202059
ENST00000384090	2.03	1.43	0.0024985	0.0203663
ENST00000362721	2.02	1.51	0.0025508	0.0207023
hsa-mir-6747-3p	1.66	2.59	0.0025797	0.0208641
ENST00000384097	1.88	5.36	0.0026104	0.0210299
ENST00000384136	1.75	5.01	0.0026468	0.0212751

ENST00000383990	1.88	5.30	0.0026511	0.0212751
ENST00000411192	1.82	8.02	0.0026771	0.0214428
ENST00000383978	1.88	5.32	0.0026935	0.0215321
ENST00000458975	1.80	5.23	0.0026987	0.0215328
ENST00000410344	1.96	7.91	0.0027706	0.0220632
ENST00000384210	2.41	1.34	0.0027812	0.0220632
ENST00000363750	2.33	2.20	0.0029619	0.0231868
ENST00000384419	1.95	4.42	0.0029791	0.0232686
ENST00000384656	1.84	5.38	0.0029836	0.0232686
ENST00000384187	1.86	5.30	0.0030008	0.0233587
ENST00000475628	1.83	9.64	0.0030853	0.0239266
ENST00000419895	2.06	1.12	0.0031066	0.0240471
ENST00000365571	1.36	13.03	0.0031172	0.0240841
ENST00000363548	1.86	1.30	0.0033021	0.0251846
ENST00000363094	1.85	1.60	0.0033264	0.0252807
ENST00000363925	2.23	5.66	0.0034082	0.0257099
ENST00000384119	1.82	5.36	0.0034368	0.0258322
ENST00000384600	2.15	2.57	0.0034567	0.0259347
ENST00000391196	1.71	2.81	0.0034761	0.0260328
ENST00000365672	1.53	1.72	0.0036987	0.027502
ENST00000364916	1.68	5.53	0.003706	0.0275069
ENST00000493125	1.83	2.29	0.0037154	0.0275275
ENST00000502192	1.72	2.49	0.0037232	0.0275355
ENST00000364535	1.75	6.84	0.0037707	0.0278374
ENST00000363660	1.56	4.91	0.0038336	0.0282515
ENST00000363068	1.56	5.71	0.0038435	0.0282744
ENST00000501338	2.54	2.45	0.0038544	0.0282928
ENST00000365063	1.63	4.85	0.0038626	0.0282928
ENST00000384344	2.21	3.12	0.0039043	0.0282928
ENST00000365030	1.56	5.77	0.0040151	0.0289923
ENST00000384567	1.61	5.65	0.004018	0.0289923
ENST00000459367	1.83	4.73	0.0040364	0.0290749
ENST00000362695	2.27	1.60	0.0040782	0.0293252
ENST00000364251	1.62	4.73	0.0041116	0.029514
ENST00000410290	1.57	4.98	0.0041538	0.0297655
ENST00000384010	1.57	10.25	0.0041739	0.0298575
ENST00000410694	1.88	8.00	0.004198	0.029876
ENST00000384782	1.57	10.25	0.0043117	0.0306319
ENST00000383886	2.28	2.48	0.0043887	0.0310759
ENST00000383861	1.56	10.25	0.0043891	0.0310759
ENST00000458811	1.82	9.09	0.0044202	0.0312188
ENST00000383925	1.57	10.25	0.0044282	0.0312188

ENST00000364228	1.40	12.12	0.0044319	0.0312188
ENST00000483592	1.84	2.06	0.004482	0.0313465
ENST00000365208	1.66	4.62	0.0044878	0.0313465
ENST00000384278	1.56	10.25	0.0045088	0.0314407
ENST00000362421	1.72	5.55	0.0045942	0.0319285
ENST00000390910	1.79	3.18	0.0046244	0.0320844
ENST00000363421	1.60	6.86	0.0046552	0.0322445
ENST00000383869	1.55	10.25	0.0047548	0.0328171
ENST00000363271	1.68	9.23	0.0047616	0.0328171
ENST00000410557	1.60	4.84	0.0047902	0.032878
ENST00000384659	1.55	10.25	0.0047935	0.032878
ENST00000384135	1.62	2.39	0.0047942	0.032878
ENST00000411009	2.42	2.04	0.0048193	0.032941
ENST00000364348	1.67	2.14	0.0048353	0.0329961
ENST00000459508	1.72	5.52	0.0049046	0.0333886
ENST00000410794	1.78	7.61	0.0049089	0.0333886
ENST00000383860	2.04	2.93	0.0049234	0.0334323
ENST00000363858	2.61	2.72	0.0050306	0.0340489
ENST00000362931	1.55	5.40	0.0050775	0.0343105
ENST00000501144	2.90	3.74	0.0051282	0.0345323
ENST00000391084	2.07	6.86	0.0051296	0.0345323
ENST00000364308	1.47	5.89	0.0051353	0.0345323
ENST00000363552	1.87	4.23	0.0051716	0.0347198
ENST00000384436	1.56	3.44	0.0051865	0.0347641
ENST00000364113	1.50	6.49	0.0052398	0.0350647
ENST00000390895	1.57	6.87	0.0053223	0.0355379
ENST00000383890	1.49	1.61	0.0053277	0.0355379
ENST00000485481	1.54	5.08	0.0054041	0.0358746
ENST00000384138	1.73	5.40	0.0054267	0.0359672
hsa-mir-4746-5p	1.54	2.84	0.0054503	0.0360662
ENST00000384541	1.49	5.06	0.0056341	0.0371044
hsa-mir-744-5p	1.46	6.27	0.005999	0.0390522
ENST00000364128	1.69	2.51	0.0060266	0.0391303
ENST00000384626	1.82	1.25	0.00607	0.0393508
ENST00000411315	1.62	5.48	0.0061547	0.0397754
ENST00000481967	1.56	5.69	0.0062761	0.0404967
ENST00000495498	1.74	4.72	0.0063703	0.0407248
ENST00000444141	1.56	2.00	0.0063831	0.0407439
hsa-mir-182-5p	1.53	4.70	0.0065326	0.0414432
ENST00000362680	1.62	2.03	0.0066602	0.042124
ENST00000497631	1.71	4.78	0.0068492	0.0429913
ENST00000363186	1.60	5.14	0.0068724	0.0430718

ENST00000459124	1.41	6.03	0.0069123	0.0432119
ENST00000499566	2.03	9.07	0.0069156	0.0432119
hsa-mir-296-3p	1.40	6.09	0.0069758	0.0434574
ENST00000410129	1.49	2.85	0.0070702	0.0439135
ENST00000496990	1.63	2.22	0.007156	0.044314
ENST00000386062	1.62	7.91	0.0071877	0.0444436
hsa-mir-33b-3p	1.49	2.19	0.0072255	0.0446111
ENST00000362540	1.50	5.97	0.007265	0.0447217
ENST00000459577	1.55	4.34	0.0072914	0.0448046
ENST00000459229	1.61	2.43	0.0073067	0.0448046
ENST00000498878	1.50	3.14	0.0073132	0.0448046
ENST00000364938	1.52	6.08	0.0073897	0.0448532
ENST00000459041	1.54	1.74	0.0074958	0.0453353
ENST00000501043	2.98	5.47	0.0075302	0.0453969
ENST00000484771	1.90	10.95	0.0076261	0.0456575
ENST00000410205	1.59	5.03	0.0078392	0.0464505
hsa-mir-1307-3p	1.45	10.13	0.0083312	0.04903
ENST00000362765	1.49	5.89	0.0083873	0.0491509
ENST00000364829	1.74	1.44	0.008468	0.0495541

Supplementary Table S8B: Differential expression analysis of human miRNA genes from neuronal stem cells infected with T. gondii EGS strain for 18 hours.

gene_ID	logFC (cont-inf)	logCPM	PValue	FDR
ENST00000492060	-4.4355792	11.0111788	1.52E-60	4.14E-57
ENST00000498989	-9.2695105	7.00067438	1.29E-54	1.76E-51
ENST00000500340	-10.643292	4.96497887	2.80E-33	2.55E-30
ENST00000461045	-3.0038328	13.0904557	1.02E-32	6.94E-30
ENST00000500868	-8.0484456	4.99815291	3.57E-31	1.95E-28
ENST00000474075	-2.9638692	8.76338968	9.99E-28	4.53E-25
ENST00000362507	5.44517806	5.84437929	1.67E-27	6.51E-25
ENST00000499746	-9.7849844	4.15662322	4.07E-27	1.39E-24
ENST00000500663	-6.3725225	4.22604382	8.17E-27	2.47E-24
ENST00000486682	-7.1880398	4.16983392	2.56E-26	6.97E-24
ENST00000501999	-5.482546	5.21677565	8.44E-26	2.09E-23
ENST00000501119	-5.0419309	5.75012147	1.14E-25	2.58E-23
ENST00000478115	-3.5662645	7.50197233	1.24E-25	2.61E-23
ENST00000474173	-10.135384	4.47863028	3.55E-24	6.90E-22
ENST00000501638	-6.9014743	3.90421218	7.42E-24	1.35E-21
ENST00000501386	-6.828526	3.83771334	3.48E-23	5.92E-21
ENST00000501814	-4.7715281	5.57507361	4.41E-23	7.07E-21
ENST00000387069	2.88000334	7.98544088	1.05E-22	1.59E-20
ENST00000499697	-6.237872	4.12010598	2.61E-22	3.74E-20
ENST00000364931	3.89948308	6.04014189	4.80E-22	6.54E-20
ENST00000500633	-9.2241885	3.63955347	7.29E-22	9.47E-20
ENST00000501902	-4.7969183	4.17051274	3.29E-20	4.07E-18
ENST00000410796	-4.6862754	4.64623249	3.77E-20	4.46E-18
ENST00000499378	-5.4583808	3.92387549	2.04E-19	2.32E-17
ENST00000500409	-9.8624629	4.21691173	4.95E-19	5.40E-17
hsa-mir-155-5p	3.52873956	7.51758678	6.21E-17	6.51E-15
ENST00000501823	-5.5412794	3.46885925	6.64E-17	6.71E-15
ENST00000499246	-4.4128849	3.88553396	1.41E-16	1.37E-14
ENST00000489202	-2.0432881	9.60189313	1.80E-16	1.65E-14
ENST00000487032	-6.1991892	3.26726365	1.81E-16	1.65E-14
ENST00000499572	-6.2197907	3.28847732	1.74E-15	1.53E-13
ENST00000493956	-1.8994194	10.3113906	2.07E-15	1.71E-13

hsa-mir-95-3p	-4.0836044	4.44949291	2.04E-15	1.71E-13
ENST00000500011	-4.7208856	3.6160936	1.26E-14	1.01E-12
ENST00000500207	-4.358338	3.59231343	1.29E-14	1.01E-12
ENST00000479561	-5.1508234	3.11944306	1.40E-14	1.06E-12
ENST00000500974	-6.1617595	3.22220249	4.23E-14	3.11E-12
ENST00000362698	1.91947327	10.1657886	4.38E-14	3.14E-12
ENST00000471114	-2.9486	6.09287896	4.50E-14	3.14E-12
ENST00000463737	-1.840831	9.05938019	2.08E-13	1.41E-11
ENST00000408749	3.03049298	3.9651975	3.24E-13	2.15E-11
ENST00000362477	2.09944223	12.9737847	5.86E-13	3.80E-11
ENST00000480364	-2.9251468	4.06870358	1.41E-12	8.94E-11
ENST00000476674	-1.7606676	9.06129522	2.22E-12	1.38E-10
ENST00000498894	-4.3108507	2.86982444	5.75E-12	3.48E-10
ENST00000387943	2.74251907	3.98372816	8.06E-12	4.72E-10
ENST00000493141	-2.5795824	5.08283812	8.14E-12	4.72E-10
hsa-mir-7974-3p	2.68734105	4.70596784	1.77E-11	1.00E-09
ENST00000501120	-4.0162958	3.27773559	2.21E-11	1.23E-09
ENST00000500692	2.77787533	3.67234095	2.48E-11	1.35E-09
ENST00000500159	-2.5853253	6.21428391	3.12E-11	1.67E-09
ENST00000499784	-3.9924618	3.23547885	3.24E-11	1.70E-09
ENST00000459577	2.82577617	3.53064118	3.80E-11	1.95E-09
ENST00000491379	-1.8511292	11.3457992	4.17E-11	2.10E-09
ENST00000500638	-4.2350374	3.20059877	5.76E-11	2.85E-09
ENST00000479524	-1.5068463	11.6912322	7.37E-11	3.59E-09
hsa-mir-486-5p	-3.2339158	5.77801071	8.19E-11	3.91E-09
ENST00000458546	2.68808286	3.73907334	3.89E-10	1.83E-08
hsa-mir-151a-5p	-1.8772712	8.07772689	4.42E-10	2.04E-08
[truncated: 29,984 more chars]
