# Supplementary material for: New paradigms for understanding and step changes in treating active and chronic, persistent apicomplexan infections
Source: Sci Rep. 2016 Jul 14;6:29179. doi: 10.1038/srep29179 (PMC4944145; doi:10.1038/srep29179)

Supplement A

New paradigms for understanding and step changes in treating active and chronic, persistent apicomplexan infections

Martin McPhillie[1]*, Ying Zhou[2]*, Kamal El Bissati[2], Jitender Dubey[3], Hernan Lorenzi[4], Michael Capper[5], Amanda K Lukens[6,7], Mark Hickman[8], Stephen Muench[1], Shiv Kumar Verma[3] , Christopher R. Weber[2] , Kelsey Wheeler[2], James Gordon[1], Justin Sanders[9], Hong Moulton[9] , Kai Wang[10] , Taek-Kyun Kim[10], Yuqing He[10], Tatiana Santos [11], Stuart Woods[12] , Patty Lee[8], David Donkin[8], Eric Kim [8], Laura Fraczek[2], Joseph Lykins[2], Farida Esaa[2], Fatima Alibana-Clouser[2] , Sarah Dovgin[2], Louis Weiss[11], Gael Brasseur[14], Dyann Wirth[6,7] , Michael Kent [9], Leroy Hood[10], Brigitte Meunieur[13] , Craig W. Roberts[12], S. Samar Hasnain[5], Svetlana V. Antonyuk[5,+], Colin Fishwick[1, +], and Rima McLeod[2+]

[1] University of Leeds, Leeds, UK

[2] University of Chicago, Chicago, USA

[3] USDA, Beltsville, Maryland, USA

[4] J Craig Venter Institute, Rockville Maryland, USA

[5] University of Liverpool, Liverpool, UK

[6] Harvard School of Public Health, Boston, Massachusetts, USA

[7] The Broad Institute, Boston, Massachusetts, USA

[8] Walter Reed Army Institute of Research, Silver Spring, Maryland, USA

[9] Oregon State University, Corvallis, Oregon, USA

[10] Institute for Systems Biology, Seattle, Washington, USA

[11] Albert Einstein College of Medicine, Bronx, New York, USA

[12] Strathclyde University, Glasgow, Scotland, UK

[13] Institute for Integrative Biology of the Cell (12BC), Gif-sur-Yvette, France

[14] CNRS, Marseilles, France

*Equal contributions

[+] To whom correspondence should be addressed: Rima McLeod , rmcleod@uchicago.edu; Colin G. Fishwick, c.w.g-fishwick@leeds.ac.uk; Svetlana V. Antonyuk, antonyuk@liverpool.ac.uk

Competing financial interests: none

Author contribution statement: M.M conceived the idea of the study, contributed to concept and design, interpreted the results and drafted a part of the manuscript. Y.Z contributed to concept and design, performed experiments, interpreted experimental data, drafted and revised her part of the manuscript. K.E.B. contributed to concept and design, performed experiments, interpreted experimental data, drafted and revised his part of the manuscript. J.D. performed experiments and interpreted experimental data. H.L. carried out the statistical and bioinformatics data analyses, interpretation, and drafted his part of the manuscript. M.C. performed experiments and interpreted experimental data. A.L. performed experiments and interpreted experimental data. M.H. performed experiments and interpreted experimental data. S.M performed experiments and interpreted experimental data. S.K.V performed experiments and interpreted experimental data**.** C.W. interpreted experimental. K.WH. performed experiments and

References for online supplement

S1. Holfels E, McAuley J, Mack D, Milhous WK and McLeod R. In vitro effects of artemisinin ether, cycloguanil hydrochloride (alone and in combination with sulfadiazine), quinine sulfate, mefloquine, primaquine phosphate,trifluoperazine hydrochloride, and verapamil on Toxoplasma gondii. Antimicrob. Agents Chemother. , 38:1392-6, 1994. PMID: 8092843

S2. Zuther E, Johnson JJ, Haselkorn R, McLeod R and Gornicki P. Growth of Toxoplasma gondii is inhibited by aryloxyphenoxypropionate herbicides targeting acetyl-CoA carboxylase. Proc. Natl. Acad. Sci. U. S. A. , 96:13387-92, 1999. PMID: 10557330

S3. McLeod R, Muench SP, Rafferty JB, Kyle DE, Mui EJ, Kirisits MJ, Mack DG, Roberts CW, Samuel BU, LyonsRE, Dorris M, Milhous WK and Rice DW. Triclosan inhibits the growth of Plasmodium falciparum andT oxoplasma gondii by inhibition of apicomplexan Fab I. Int. J. Parasitol. , 31:109-13, 2001. PMID: 11239932

S4. Muench SP, Rafferty JB, McLeod R, Rice DW and Prigge ST. Expression, purification and crystallization of the Plasmodium falciparum enoyl reductase. Acta Crystallogr D Biol Crystallogr , 59:1246-8, 2003. PMID: 12832774

S5. Samuel BU, Hearn B, Mack D, Wender P, Rothbard J, Kirisits MJ, Mui E, Wernimont S, Roberts CW, Muench SP, Rice DW, Prigge ST, Law AB and McLeod R. Delivery of antimicrobials into parasites. Proc. Natl. Acad. Sci.U. S. A. , 100:14281-6, 2003. PMID: 14623959

S6. Mui EJ, Jacobus D, Milhous WK, Schiehser G, Hsu H, Roberts CW, Kirisits MJ, and McLeod R. Triazine Inhibits Toxoplasma gondii Tachyzoites in vitro and in vivo. Antimicrob. Agents Chemother. 49:3463-7, 2005. PMID: 16048961.

S7. Muench SP, Prigge ST, Zhu L, Kirisits MJ, Roberts CW, Wernimont S, McLeod R and Rice DW. Expression, purification and preliminary crystallographic analysis of the *Toxoplasma gondii* enoyl reductase. Acta Crystallogr Sect F Struct Biol Cryst Commun , 62:604-6, 2006. PMID: 16754994

S8. Muench SP, Prigge ST, McLeod R, Rafferty JB, Kirisits MJ, Roberts CW, Mui EJ and Rice DW. Studies of Toxoplasma gondii and Plasmodium falciparum enoyl acyl carrier protein reductase and implications for the development of antiparasitic agents. Acta Crystallogr D Biol Crystallogr , 63:328-38, 2007. PMID: 17327670

S9. Mui EJ, Schiehser GA, Milhous WK, Hsu H, Roberts CW, Kirisits M, Muench S, Rice D, Dubey JP, Fowble JW, Rathod PK, Queener SF, Liu SR, Jacobus DP and McLeod R. Novel Triazine JPC-2067-B Inhibits Toxoplasma gondii In Vitro and In Vivo. PLoS Negl Trop Dis , 2:e190, 2008. PMID: 18320016

S10. Tipparaju S, Muench S, Mui E, Ruzheinikov S, Hutson S, Kirisits M, Prigge S, Roberts C, Henriquez F, Kozikowski AP, Rice D, McLeod R. Rational Development of Novel Inhibitors of Toxoplasma gondii Enoyl Reductase. J Med Chem 53: 6287-300, 2010. PMID: 20698542

S11. Kortagere S, Mui E, McLeod R, Welsh WJ. Rapid discovery of inhibitors of Toxoplasma gondii using hybridstructure-based computational approach. J Comput Aided Mol Des 25: 403-411, 2011. PMID: 21359560

S12. Fomovska A, Huang Q, El Bissati K, Mui EJ, Witola WH, Cheng G, Zhou Y, Sommerville C, Roberts CW, BettisS, Prigge ST, Afanador GA, Hickman MR, Lee PJ, Leed SE, Auschwitz JM, Pieroni M, Stec J, Muench SP, Rice DW, Kozikowski AP, McLeod R. Novel N-Benzoyl-2-Hydroxybenzamide Disrupts Unique Parasite Secretory Pathway. Antimicrob Agents Chemother [Internet]. 2012 May [cited 2015 Jul 8];56(5):2666–82. PMID: 22354304

S13. Stec J, Huang Q, Pieroni M, Kaiser M, Fomovska A, Mui E, Witola WH, Bettis S, McLeod R, Brun R,Kozikowski AP. Synthesis, biological evaluation, and structure-activity relationships of N-benzoyl-2-hydroxybenzamides as agents active against P. falciparum (K1 strain), Trypanosomes, and Leishmania. J Med Chem 55: 3088-100, Epub 2012 Mar 20. PMID: 22352841

S14. Lai BS, Witola WH, El Bissati K, Zhou Y, Mui E, Fomovska A, McLeod R. Molecular target validation,antimicrobial delivery, and potential treatment of Toxoplasma gondii infections. Proc Natl Acad Sci U.S.A. 109:14182-14187, 2012. PMID: 22891343

S15. Fomovska A, Wood RD, Mui E, Dubey JP, Ferreira LR, Hickman MR, Lee PJ, Leed SE, Auschwitz JM, WelshWJ, Sommerville C, Woods S, Roberts C, McLeod R. Salicylanilide inhibitors of Toxoplasma gondii. J Med Chem. 55: 8375-91, Epub 2012 Sep 26. PMID: 22970937

S16. Cheng, G, Muench SP, Zhou Y, Afanador G, Mui E, Fomovska A, Lai BS, Prigge S, Woods S, Roberts CW,Hickman M, Lee P, Leed S, Auschwitz J, Rice D, Mcleod R. Design, synthesis, and biological activity of diarylether inhibitors of Toxoplasma gondii enoyl reductase. Bioorganic and Medicinal Chem Letters 23:2035-2043,Epub Feb 2013. PMID: 23453069

S17. Muench SP, Stec J, Zhou Y, Afanador G, McPhillie M, Hickman M, Lee P, Leed S, Auschwitz J, Prigge S, RiceDW, McLeod R. Development of a triclosan scaffold which allows for adaptations on both the A-and B-ring fortransport peptides. Bioorganic and Medicinal Chem Letters 23:3551-3555, Epub Apr 2013. doi: 10.1016/j.bmcl.2013.04.035. Epub 2013 Apr 24.PMID: 23664871

S18. Stec J, Fomovska A, Afanador GA, Muench SP, Zhou Y, Lai BS, El Bissati K, Hickman MR, Lee PJ, Leed SE,Auschwitz JM, Sommervile C, Woods S, Roberts CW, Rice D, Prigge ST, McLeod R, Kozikowski AP.McLeod, R. Modification of triclosan scaffold in search of improved inhibitors for enoyl-acyl carrier protein (ACP) reductase in Toxoplasma gondii. Chem Med Chem . 8: 1138-1160, Epub 2013 June 14. PMID: 23776166

S19. Zhou Y, Fomovska A, Muench S, Lai BS, Mui E, Mcleod R. Spiroindolone that inhibits PfATPase4 is a Potent,Cidal Inhibitor of Toxoplasma gondii tachyzoites in vitro and in vivo. Antimicrobial Agents and Chemistry, 2013. PMID: 24366743

S20. Afanador, GA, Muench SP, McPhillie M, Fomovska A, Schön A, Zhou Y, Cheng G, Stec J,

Freundlich JS, Shieh HM, Anderson JW, Jacobus DP, Fidock DA, Kozikowski AP, Fishwick CW, Rice DW, Freire E, McLeod R, Prigge. Discrimination of Potent Inhibitors of Toxoplasma gondii Enoyl-Acyl Carrier Protein Reductase by Thermal Shift Assay. Biochemistry, 2013 Dec 23;52(51):9155-66. doi: 10.1021/bi400945y. Epub 2013 Dec 13. PMID: 24295325.

S21. Wilkinson C, Muench SP, McPhillie MJ, Zhou Y, Woods S, Afanador GA, Rawson S, Khaliq F, Prigge ST, Roberts CW, Rice DW, McLeod R, Fishwick CW, Muench SP. The benzimidazole based drugs show good activity against T. gondii but poor activity against its proposed enoyl reductase enzyme target.Bioorg Med Chem Lett. 2014 Feb 1;24(3):911-6. doi: 10.1016/j.bmcl.2013.12.066. Epub 2013 Dec 22.PMID: 24398298

<u>Fibroblasts , MonoMacs, Neuronal stem cells</u>

S22. Witola WH, Mui E, Hargrave A, Liu S, Hypolite M, Montpetit A, Cavailles P, Bisanz C, Cesbron-Delauw MF, Fournié GJ, McLeod R. NALP1 influences susceptibility to human congenital toxoplasmosis, proinflammatory cytokine response, and fate of Toxoplasma gondii-infected monocytic cells. Infect Immun 79: 756-66, 2011. PMID: 21098108

S23. Witola W, Liu S, Montpetit A, Hypolite M, Zhou Y, Mui E, Cesbron-Delauw MF, Fournie G, Cavailles P, Bisanz C, Boyer K, Withers S, Noble AG, Swisher C, Heydemann P, Rabiah P, McLeod R. ALOX12 In Human Toxoplasmosis. Infect Immun. 2014 Jul;82(7):2670-9. PMID: 24686056

S24. Siebzehnrubl FA, Steindler DA. Isolating and culturing of precursor cells from the adult human brain.Methods Mol Biol. 2013;1059:79-86. doi: 10.1007/978-1-62703-574-3_7. PMID: 23934835

<u>Inhibitors *in vitro* and *in vivo* in conventional assays</u>

S25. Gubbels M-J, Li C, Striepen B. High-Throughput Growth Assay for Toxoplasma gondii Using Yellow Fluorescent Protein. Antimicrob Agents Chemother [Internet]. 2003 Jan [cited 2015 Jul 8];47(1):309–16.

S26. Vidigal PVT, Santos DVV, Castro FC, Couto JC de F, Vitor RW de A, Brasileiro Filho G. Prenatal toxoplasmosis diagnosis from amniotic fluid by PCR. Rev Soc Bras Med Trop [Internet]. 2002 Feb [cited 2015 Jul 8];35(1):1–6.

S27. Silva LA, Brandão GP, Pinheiro BV, Vitor RWA. Immunosuppression with cyclophosphamide favors reinfection with recombinant Toxoplasma gondii strains. Parasite J Société Fr Parasitol [Internet]. 2012 Aug [cited 2015 Jul 8];19(3):249–57.

S28. Mcleod R, Frenkel JK, Estes RG, Mack DG, Eisenhauer PB, Gibori G. Subcutaneous and intestinal vaccination with tachyzoites of *Toxoplasma gondii* and acquisition of immunity to peroral and congenital toxoplasma challenge. *J Immunol*. 1988;140(5):1632-7. PMID 3346545.

S29. Roberts CW, Brewer JM, Alexander J.Congenital toxoplasmosis in the Balb/c mouse: prevention of vertical disease transmission and fetal death by vaccination. Vaccine. 1994 Nov;12(15):1389-94.

S30. H. Lorenzi, A. Khan, M. Behnke, S. Namasivayam, S. Seshadri, M. Hadjithomas, S. Karamycheva, D. Pinney, B. Brunk, J. Ajioka, D. Azjenberg, J.C. Boothroyd, J. Boyle, M. White, XQ Zhu, J. Parkinson, L. Liu, J.C. Kissinger, D.S. Roos, L.D. Sibley. Virulence determinants in Toxoplasma gondii (In Revision, Nature Communications) 2015.

Omics, Sequencing

S31. Gajria, B. *et al.* ToxoDB: an integrated Toxoplasma gondii database resource. *Nucleic Acids Res* **36**, D553-6 (2008).

S32 . Langmead, B. & Salzberg, S.L. Fast gapped-read alignment with Bowtie 2. *Nat Methods* **9**, 357-9 (2012).

S33 . McKenna, A. *et al.* The Genome Analysis Toolkit: a MapReduce framework for analyzing next-generation DNA sequencing data. *Genome Res* **20**, 1297-303 (2010).

S34. Li, H. *et al.* The Sequence Alignment/Map format and SAMtools. *Bioinformatics* **25**, 2078-9 (2009).

S35. Cingolani, P. *et al.* A program for annotating and predicting the effects of single nucleotide polymorphisms, SnpEff: SNPs in the genome of Drosophila melanogaster strain w1118; iso-2; iso-3. *Fly (Austin)* **6**, 80-92 (2012).

S36. Huson, D.H. & Bryant, D. Application of phylogenetic networks in evolutionary studies. *Mol Biol Evol* **23**, 254-67 (2006).

S37. Robinson, M.D., McCarthy, D.J. & Smyth, G.K. edgeR: a Bioconductor package for differential expression analysis of digital gene expression data. *Bioinformatics* **26**, 139-40 (2010).

S38. Kozomara, A. & Griffiths-Jones, S. miRBase: annotating high confidence microRNAs using deep sequencing data. *Nucleic Acids Res* **42**, D68-73 (2014).

S38a. Binns D, Dimmer E, Huntley R, Barrell D, O'Donovan C, Apweiler R. (2009) QuickGO: a web-based tool for Gene Ontology searching. Bioinformatics. 2009; 25(22):3045-6.

Oocysts

S39. Dubey JP, Ferreira LR, Martins J, McLeod R. Oral oocyst-induced mouse model of toxoplasmosis: effect ofinfection with Toxoplasma gondii strains of different genotypes, dose, and mouse strains (transgenic, out-bred, inbred)on pathogenesis and mortality. Parasitology 139:1-13, Epub 2011. PMID: 22078010

Synthetic methods

S40. Conrad M, Limpach L. Ueber das γ-oxychinaldin und dessen derivate *Ber. Dtsch. Chem. Gesell.* **1887**, 20, 944– 948

S41. Hart ME, Suchland KL, Miyakawa M, Bunzow JR, Grandy DK, Scanlan TS.Trace amine-associated receptor agonists: synthesis and evaluation of thyronamines and related analogues *J. Med. Chem.* **2006**, 49, 1101– 1112 PMID: 16451074

S42. Maiti D, Buchwald SL.Cu-catalyzed arylation of phenols: synthesis of sterically hindered and heteroaryl diaryl ethers *J. Org. Chem.* **2010**, 75, 1791– 1794 PMID: 20141182

ADMET and drug development

S43. Obach RS. Prediction of human clearance of twenty-nine drugs from hepatic microsomal intrinsic clearance data: an examination of *in vitro* half-life approach and nonspecific binding to microsomes *Drug Metab. Dispos.* **1999**, 27, 1350– 1359. PMID: 10534321

S44. Li Y, et al. The relationship between diphenylamine structure and NSAIDs-induced hepatocytes injury. Toxicol Lett. 2009 Apr 25;186:111. PMID: 19429231

S45. Dykens JA, Marroquin LD, Will Y. Strategies to reduce late-stage drug attrition due to mitochondrial toxicity. Expert Rev Mol Diagn. 2007 Mar;7:161. PMID: 17331064

S46. Marroquin LD, Hynes J, Dykens JA, Jamieson JD, Will Y. Circumventing the Crabtree effect: replacing media glucose with galactose increases susceptibility of HepG2 cells to mitochondrial toxicants. Toxicol Sci. 2007 Jun;97:539. PMID: 17361016

S47. Porter, C. J.; Williams, H. D.; Trevaskis, N. L.Recent advances in lipid-based formulation technology *Pharm. Res.* **2013**, 30, 2971– 2975. PMID: 24158727

S48. Friesen, D. T.; Shanker, R.; Crew, M.; Smithey, D. T.; Curatolo, W. J.; Nightingale, J. A.Hydroxypropyl methylcellulose acetate succinate-based spray-dried dispersions: an overview *Mol. Pharmaceutics* **2008**, 5, 1003– 1019. PMID: 19040386

S49. Rautio J, Kumpulainen H, HeimbachT, Oliyai R, Oh D, JarvinenT, Savolainen J. Prodrugs: design and clinical applications *Nature Rev Drug Discovery* **2008**, 7, 255– 270. PMID: 18219308

S50. Davies B, Morris T. Physiological parameters in laboratory animals and humans *Pharm. Res.* **1993**, 10, 1093– 1095. PMID: 8378254

S51. Simmons K.J,; Cunningham F,; McPhillie M.J.; Fishwick C.W.G.; Johnson A.P. Design and preliminary ADMET studies of novel Plasmodium falciparum Dihydroorotate Dehydrogenase Inhibitors; Manuscript in preparation. 2015

S52. weblink to ChemPartner's DMPK section:

http://www.chempartner.cn/a/SERVICES/DMPK/11.html

S53. Sidik SM, Hackett CG, Tran F, Westwood NJ, Lourido S. Efficient genome engineering of Toxoplasma gondii using CRISPR/Cas9. PLoS One. 2014 Jun 27;9(6):e100450. doi: 10.1371/journal.pone.0100450. eCollection 2014. PMID: 24971596 [PubMed - in process] PMCID: PMC4074098

S54. Machida K, Hayashi Y Osada H A Novel Adenine Nucleotide Translocase Inhibitor, MT-21, Induces Cytochrome *c* Release by a Mitochondrial Permeability Transition-independent Mechanism. JBC 277(44) :31242-8. June 12, 2002, DOI 10.1074/jbc.M204564200

S55. Schwab JC, Afifi Afifi m, Pizzorno G, Handschumacherb RE, Keith AJoiner KA, Toxoplasma gondii tachyzoites possess an unusual plasma membrane adenosine transporter. MBP 70:1–2, March 1995, Pp 59–69 doi:10.1016/0166-6851(95)00005-L

S56. Fivelman QL, Adagu IS, Warhurst DC. Modified fixed-ratio isobologram method for studying *in vitro* interactions between atovaquone and proguanil or dihydroartemisinin against drug-resistant strains of *Plasmodium falciparum*. Antimicrob Agents Chemother. 2004 Nov;48:4097. PMID: 15504827

S57. Araujo FG, Suzuki Y, Remington JS (1996) Use of rifabutin in combination with atovaquone, clindamycin, pyrimethamine, or sulfadiazine for treatment of toxoplasmic encephalitis in mice. *Eur J Clin Microbiol Infect Dis* **15**:394–397. PMID:8793398

S58. Kesisoglou F, Panmai S, Wu, Y. Nanosizing—oral formulation development and biopharmaceutical evaluation *Adv. Drug Delivery Rev.* **2007**, 59, 631– 644. PMID: 17601629

S59. Hutson SL, Mui E, Kinsley K, Witola WH, Behnke MS, El Bissati K, Muench SP, Rohrman B, Liu SR, Wollmann R, Ogata Y, Sarkeshik A, Yates JR 3rd, McLeod R. T. gondii RP promoters & knockdown reveal molecular pathways associated with proliferation and cell-cycle arrest. PLoS One. 2010 Nov 22;5(11):e14057 PMID: 21124925

S60. Tan T, Mui E, Cong H, Witola W, Montpetit A, Muench S, Sidney J, Alexander J, Sette A, Grigg M, Reed S, Maewal A, Kim S, McLeod R. Identification of T. gondii epitopes, adjuvants, & host genetic factors that influence protection of mice and humans. Vaccine. 28: 3977-89, 2010 PMID: 20347630

S61. Bissati K, Zhou Y, Dasgupta D, Cobb D, Dubey J, Burkhard P, Lanar D, McLeod R. Effectiveness of a novel immunogenic nanoparticle platform for Toxoplasma peptide vaccine in HLA transgenic mice. Vaccine. 2014 May 30; 32(26); 3243-3248.

S62. Sanders JL, Zhou Y, Moulton HM, McLeod R, Dubey JP, Weiss LM, Kent ML. The zebrafish, Danio rerio, as a model for Toxoplasma gondii: an initial description of infection in fish. J Fish Dis. 2015 Jul; 38(7):675-9.

S63. Mather, M. W.; Morrisey, J. M.; Vaidya, A. B.Hemozoin-free *Plasmodium falciparum* mitochondria for physiological and drug susceptibility studies *Mol. Biochem. Parasitol.* **2010**, 174, 150– 153. PMID: 20674615

S64. Painter HJ, Morrisey JM, Mather MW, Vaidya AB. Specific role of mitochondrial electron transport in blood-stage *Plasmodium falciparum*. Nature. 2007 Mar 1;446:88. PMID: 17330044

Yeast: Mutations to define active site

S65. Hill, P., J. Kessl, N. Fisher, S. Meshnick, B. Trumpower, and B. Meunier. 2003. Recapitulation in *Saccharomyces cerevisiae* of cytochrome *b* mutations conferring resistance to atovaquone in *Pneumocystis jiroveci*. Antimicrob. Agents Chemother. 47:2725-2731. PMID: 12936966

S66. Kessl JJ, Ha KH, Merritt AK, Meshnick SR, Trumpower BL (2006) Molecular basis of Toxoplasma gondii atovaquone resistance modeled in Saccharomyces cerevisiae. *Mol Biochem Parasitol* **146**:255–258. PMID: 16412524

S67. Kessl JJ, Meshnick SR, Trumpower BL (2007) Modeling the molecular basis of atovaquone resistance in parasites and pathogenic fungi. *Trends Parasitol* **23**:494–501. PMID: 17826334

S68. Coppée JY, et al. (1994) Analysis of revertants from respiratory deficient mutants within the center N of cytochrome b in Saccharomyces cerevisiae. *FEBS Lett* **339**:1–6. PMID: 8313954

S69. Rotsaert FA, Covian R, Trumpower BL (2008) Mutations in cytochrome b that affect kinetics of the electron transfer reactions at center N in the yeast cytochrome bc1 complex. *Biochim Biophys Acta* **1777**:239–249. PMID:18328328

S70. Walker DJ, et al. (1998) Sequence polymorphisms in the *Pneumocystis carinii* cytochrome b gene and their association with atovaquone prophylaxis failure. *J Infect Dis* **178**:1767–1775. PMID: 9815231

S71. B. Meunier et al. Resistance to primaquine in Saccharomyces cerevisease. In Press.

S72. Doggett JS, Nilsen A, Forquer I, Wegmann KW, Jones-Brando L, Yolken RH, et al. Endochin-like quinolones are highly efficacious against acute and latent experimental toxoplasmosis. Proc Natl Acad Sci U S A. 2012 Sep 25;109(39):15936–41.

S73. Sanchez-Diaz A, Kanemaki M, Marchesi V, Labib K. Rapid depletion of budding yeast proteins by fusion to a heat-inducible degron. Sci STKE Signal Transduct Knowl Environ. 2004 Mar 9;2004(223):PL8.

Malaria assays

S74. Desjardins RE, Canfield CJ, Haynes JD, Chulay JD. Quantitative assessment of antimalarial activity *in vitro* by a semiautomated microdilution technique. Antimicrob Agents Chemother. 1979 Dec;16:710. PMID: 394674

S75. Johnson JD*, et al.* (2007) Assessment and continued validation of the malaria SYBR green I-based fluorescence assay for use in malaria drug screening. *Antimicrobial agents and chemotherapy* 51(6):1926-1933.

S76. Fivelman QL, Adagu IS, & Warhurst DC (2004) Modified fixed-ratio isobologram method for studying in vitro interactions between atovaquone and proguanil or dihydroartemisinin against drug-resistant strains of Plasmodium falciparum. *Antimicrobial agents and chemotherapy* 48(11):4097-4102.

S77. Lukens AK, et al. (2015) Diversity-Oriented Synthesis Probe Targets Plasmodium falciparum Cytochrome b Ubiquinone Reduction Site and Synergizes With Oxidation Site Inhibitors. *The Journal of Infectious Diseases* 211(7): 1097–1103

S78. Dong CK, et al. (2011) Dong CK, Urgaonkar S, Cortese JF, et al. Identification and Validation of Tetracyclic Benzothiazepines as Plasmodium falciparum Cytochrome bc1 Inhibitors. *Chem Biol.* 18:1602-10.

S79. Trager W, Jensen JB. (1976) Human malaria parasites in continuous culture. *Science* 193:673-5

Modeling Structure

S80. 1KB9 Crystal structure: Lange, C.; Nett, J.H., et al. *EMBO J.* **2001**, 20, 6591-6600

S81. Phyre: Kelley, L.A.; Sternberg M. J. E.; *Nature Protocols* **2009**, 4, 363-371

S82. SPROUT: Gillet, V.; Johnson, A. P., et al. J. Comput.-Aided Mol. Des. 1993, 7, 127-53.

S83. eHitS: Zsoldos, Z.; Reid, D.*, et al. J. Mol. Graph.* 2007, 26, 198-212.

S84. AutoDock: Morris, G. M.; Huey, R.*, et al. J. Comput. Chem.* 2009, 30, 2785-91.

S85. ROCS: Grant, J. A.; Gallardo, M. A.*, et al. J. Comput. Chem.* 1996, 17, 1653-66.

S86. vHTS within Fishwick group: Lolicato, M.; Bucchi, A., *et al*. *Nature Chemical Biology* 2014, 10, 457-462.

S87. ChemAxon: Instant JChem 5.6.0.482, 2008-2009**,** ChemAxon (http://www.chemaxon.com).

S88. Capper MJ, O'Neill PM, Fisher N, Strange RW, Moss D, Ward SA, Berry NG, Lawrenson AS, Hasnain SS, Biagini GA, Antonyuk SV. Antimalarial 4(1H)-pyridones bind to the Qi site of cytochrome bc1.Proc Natl Acad Sci U S A. 2015 Jan 20;112(3):755-60. doi: 10.1073/pnas.1416611112.

Crystallography

S89. J. Foadi, P. Aller, Y. Alguel, A. Cameron, D. Axford, R.L. Owen, W. Armour, D.G. Waterman, S. Iwata and G. Evans, (2013) “Clustering procedures for the optimal selection of data sets from multiple crystals in macromolecular crystallography”, Acta Cryst. D69, 1617-1632, Science 335, 851-855

S90. Murshudov GN, Vagin AA, Dodson EJ (1997) Refinement of macromolecular structures by the maximum-likelihood method. *Acta Crystallogr D Biol Crystallogr* 53:240–55.

S91. Nicholls, Robert a Long, Fei Murshudov, Garib NLow-resolution refinement tools in REFMAC5, (2012) Acta crystallographica Section D: Biological crystallography

S92. Lebedev AA et al. (2012) JLigand: a graphical tool for the CCP4 template-restraint library. *Acta Crystallogr D Biol Crystallogr* 68:431–40.

S93. Emsley P, Cowtan K (2004) Coot: Model-building tools for molecular graphics. *Acta Crystallogr Sect D Biol Crystallogr* 60:2126–2132.

Additional Clinical Background and Pathogenesis:

S94. Swisher CN, Boyer K, and McLeod R. Congenital toxoplasmosis. In JB Bodensteiner, Ed. Sem Ped Neurol , 1:4- 25, 1994.

S95. Roizen N, Swisher CN, Stein MA, Hopkins J, Boyer KM, Holfels E, Mets MB, Stein L, Patel D, Meier P, Withers S, Remington J, Mack D, Heydemann P, Patton D and McLeod R. Neurologic and developmental outcomes in treated congenital toxoplasmosis. Pediatrics , 95:11-20, 1995. PMID: 7770286

S96. Patel DV, Holfels EM, Vogel NP, Boyer KM, Mets MB, Swisher CN, Roizen NJ, Stein LK, Stein MA, Hopkins J, Withers SE, Mack DG, Luciano RA, Meier P, Remington JS and McLeod RL. Resolution of intracranial calcifications in infants with treated congenital toxoplasmosis. Radiology , 199:433-40, 1996. PMID: 8668790

S97. Mets MB, Holfels E, Boyer KM, Swisher CN, Roizen N, Stein L, Stein M, Hopkins J, Withers S, Mack D, Luciano R, Patel D, Remington JS, Meier P and McLeod R. Eye manifestations of congenital toxoplasmosis. Am J Ophthalmol , 122:309-24, 1996. PMID: 8794703

S98. Roberts F and McLeod R. Pathogenesis of toxoplasmic retinochoroiditis. Parasitol Today , 15:51-7, 1999. PMID: 10234186

S99. Roberts F, Mets MB, Ferguson DJ, O'Grady R, O'Grady C, Thulliez P, Brezin AP and McLeod R.Histopathological features of ocular toxoplasmosis in the fetus and infant. Arch. Ophthalmol. , 119:51-8, 2001. PMID: 11146726

S100. Roizen N, Kasza K, Karrison T, Mets M, Noble AG, Boyer K, Swisher C, Meier P, Remington J, Jalbrzikowski J, McLeod R, Kipp M, Rabiah P, Chamot D, Estes R, Cezar S, Mack D, Pfiffner L, Stein M, Danis B, Patel D, Hopkins J, Holfels E, Stein L, Withers S, Cameron A, Perkins J and Heydemann P. Impact of visual impairment on measures of cognitive function for children with congenital toxoplasmosis: implications for compensatory intervention strategies. Pediatrics , 118:e379-90, 2006. PMID: 16864640

S101. Arun V, Noble AG, Latkany P, Troia RN, Jalbrzikowski J, Kasza K, Karrison T, Cezar S, Sautter M, Greenwald MJ, Mieler W, Mets MB, Alam A, Boyer K, Swisher CN, Roizen N, Rabiah P, Del Monte MA and McLeod R. Cataracts in congenital toxoplasmosis. J. AAPOS , 11:551-4, 2007. PMID: 18086432

S102. Benevento JD, Jager RD, Noble AG, Latkany P, Mieler WF, Sautter M, Meyers S, Mets M, Grassi MA, Rabiah P,Boyer K, Swisher C and McLeod R. Toxoplasmosis-associated neovascular lesions treated successfully withranibizumab and antiparasitic therapy. Arch. Ophthalmol. , 126:1152-6, 2008. PMID 18695115

S103. Phan L, Kasza K, Jalbrzikowski J, Noble AG, Latkany P, Kuo A, Mieler W, Meyers S, Rabiah P, Boyer K,Swisher C, Mets M, Roizen N, Cezar S, Remington J, Meier P and McLeod R. Longitudinal study of new eye lesions in treated congenital toxoplasmosis. Ophthalmology , 115:553-59 e8, 2008. PMID: 17825418

S104. Phan L, Kasza K, Jalbrzikowski J, Noble AG, Latkany P, Kuo A, Mieler W, Meyers S, Rabiah P, Boyer K, Swisher C, Mets M, Roizen N, Cezar S, Sautter M, Remington J, Meier P and McLeod R. Longitudinal study of new eye lesions in children with toxoplasmosis who were not treated during the first year of life. Am JOphthalmol , 146:375-84, 2008. PMID: 18619570

S105. Jamieson SE, de Roubaix LA, Cortina-Borja M, Tan HK, Mui EJ, Cordell HJ, Kirisits MJ, Miller EN, Peacock CS, Hargrave AC, Coyne JJ, Boyer K, Bessieres MH, Buffolano W, Ferret N, Franck J, Kieffer F, Meier P, Nowakowska DE, Paul M, Peyron F, Stray-Pedersen B, Prusa AR, Thulliez P, Wallon M, Petersen E, McLeod R,Gilbert RE and Blackwell JM. Genetic and epigenetic factors at COL2A1 and ABCA4 influence clinical outcome in congenital toxoplasmosis. PLoS One , 3:e2285, 2008. PMID:18523590

S106. McLeod R, Kieffer F, Sautter M, Hosten T and Pelloux H. Why prevent, diagnose and treat congenital toxoplasmosis? Mem Inst Oswaldo Cruz , 104:320-44, 2009. PMID: 19430661

S107. Contopoulos-Ioannidis D, Wheeler KM, Ramirez R, Press C, Mui E, Zhou Y, Van Tubbergen C, Prasad S, Maldonado Y, Withers S, Boyer KM, Noble AG, Rabiah P, Swisher CN, Heydemann P, Wroblewski K, Karrison T, Grigg ME, Montoya JG, McLeod R. Clustering of Toxoplasma gondii Infections Within Families of Congenitally Infected Infants. Clin Infect Dis. 2015 Sep 24. civ721. PMID: 26405150

S108. McLeod R, Boyer K, Karrison T, Kasza K, Swisher C, Roizen N, Jalbrzikowski J, Remington J,

Heydemann P, Noble AG, Mets M, Holfels E, Withers S, Latkany P and Meier P. Outcome of treatment for congenital toxoplasmosis, 1981-2004: the National Collaborative Chicago-Based, Congenital Toxoplasmosis Study. Clin Infect Dis , 42:1383-94, 2006. PMID: 16619149

S109. Noble G, Latkany P, Kusmierczyk J, Mets M, Rabiah P, Boyer K, Jalbrzikowski J, Wroblewski K, Karrison T,Swisher C, Mieler W, Meier P, McLeod R. Chorioretinal lesions in mothers of children with congenital toxoplasmosis in the National Collaborative Chicago-based, Congenital Toxoplasmosis Study. Scientia Medica20: 20-26, 2010. PMID: 22577474

S110. Stillwaggon E., Carrier C.S., Sautter M., & McLeod R. Maternal serologic screening to prevent congenital toxoplasmosis: a decision-analytic economic model. PLoS Negl Trop Dis 5: e1333, 2011. PMID: 21980546

S111. Burrowes D, Boyer K, Swisher CN, Noble AG, Sautter M, Heydemann P, Rabiah P, Lee D, McLeod R, and the Toxoplasmosis Study Group. Spinal Cord Lesions in Congenital Toxoplasmosis Demonstrated with Neuroimaging, Including Their Successful Treatment in an Adult. Journal of Neuroparasitology 3: 1-8, 2012. PMID: 23487348

S112. Hutson S, Wheeler K, McLone D, Frim D, Penn R, Swisher CN, Heydemann PT, Boyer KM, Nobe AG, Rabiah P, Withers S, Montoya JG, Wroblewski K, Karrison T, Grigg ME, McLeod R. Patterns of Hydrocephalus Caused by Congenital Toxoplasma gondii Infection Associate with Parasite Genetics. Clinical Infectious Diseases. 24 September 2015. PMID: 26405147

S113. Jones-Brando L, Torrey EF, Yolken R (2003) Drugs used in the treatment of schizophrenia and bipolar disorder inhibit the replication of Toxoplasma gondii. *Schizophr Res* **62**:237–244. PMID: 12837520

References for Figure legend 4.

S114. Tomita T, Bzik DJ, Ma YF, et al. The *Toxoplasma gondii* cyst wall protein CST1 is critical for cyst wall integrity and promotes bradyzoite persistence. PLoS Pathog. 2013;9(12):e1003823. PMID: 24385904

S115. Weiss LM, Kim K. The development and biology of bradyzoites of *Toxoplasma gondii*. Front Biosci. 2000;5:D391-405. PMID: 10762601

S116. Yahiaoui B, Dzierszinski F, Bernigaud A, Slomianny C, Camus D, Tomavo S. Isolation and characterization of a subtractive library enriched for developmentally regulated transcripts expressed during encystation of *Toxoplasma gondii*. Mol Biochem Parasitol. 1999;99(2):223-35. PMID: 10340486

S117. Radke JB, Lucas O, De silva EK, et al. ApiAP2 transcription factor restricts development of the *Toxoplasma* tissue cyst. Proc Natl Acad Sci USA. 2013;110(17):6871-6. PMID: 23572590

Supplement .ƻȄ

Box. Co-crystallization data collection and refinement statistics

	Cytochrome *bc1* –MJM170
Data collection	
Space group	P65
Cell dimensions	
a, b, c (Å)	129.5, 129.5, 720.3
a, b, g (°)	90, 90, 120
Resolution (Å)	50-3.5(3.56-3.50)
Rmerge (%)	15.6(82)
I/sI	5.2(1.4)
Completeness (%)	99.6(99.7)
CC1/2	0.971(0.331)
Redundancy	9.2(9.2)
Refinement	
Resolution (Å)	50 – 3.5
No. reflections	80565
Rwork/ Rfree	24.13(29.13)
No. atoms	31811
Protein	30980
Ligand/ion	831
B-factors	
Protein	93.84
Ligand/ion	87.86
R.m.s deviations	
Bond lengths (Å)	0.010
Bond angles (º)	1.564
PDB access code	5HJ4

Supplemental Figures

Figure S1

Heat Maps for HFF infected with EGS in culture for 2, 18, and 48 hours for host and parasite cells

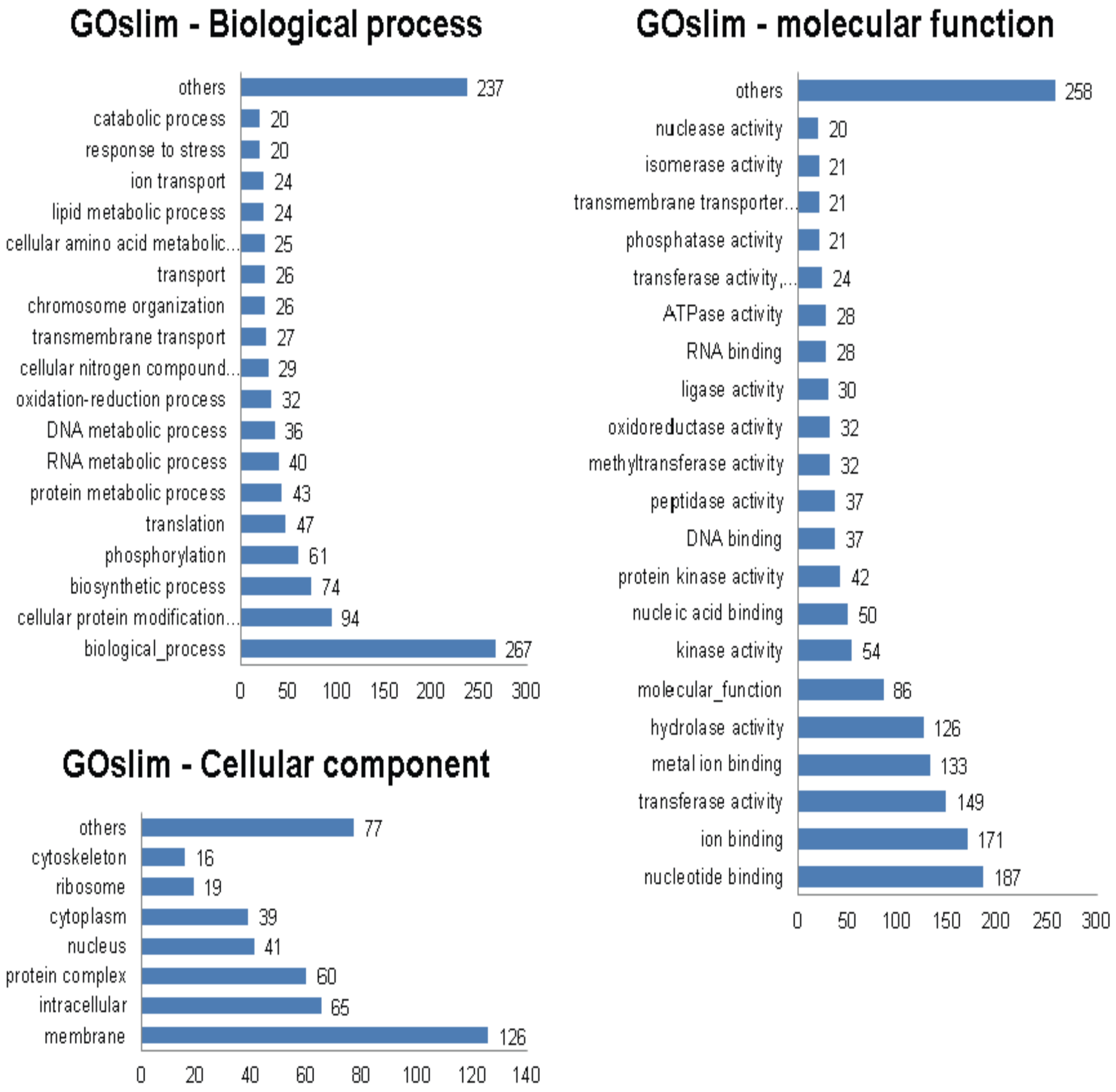

Figure S2

Log2 transformed normalized read count distributions were used to determine transcripts with low read count. The bars represent number of transcripts with different read count. Transcripts with read count below the 25 percentile (dark gray arrow) on the overall read count distribution were removed for further analysis. Yellow and green colors were used for presenting MM6 and NSC data.

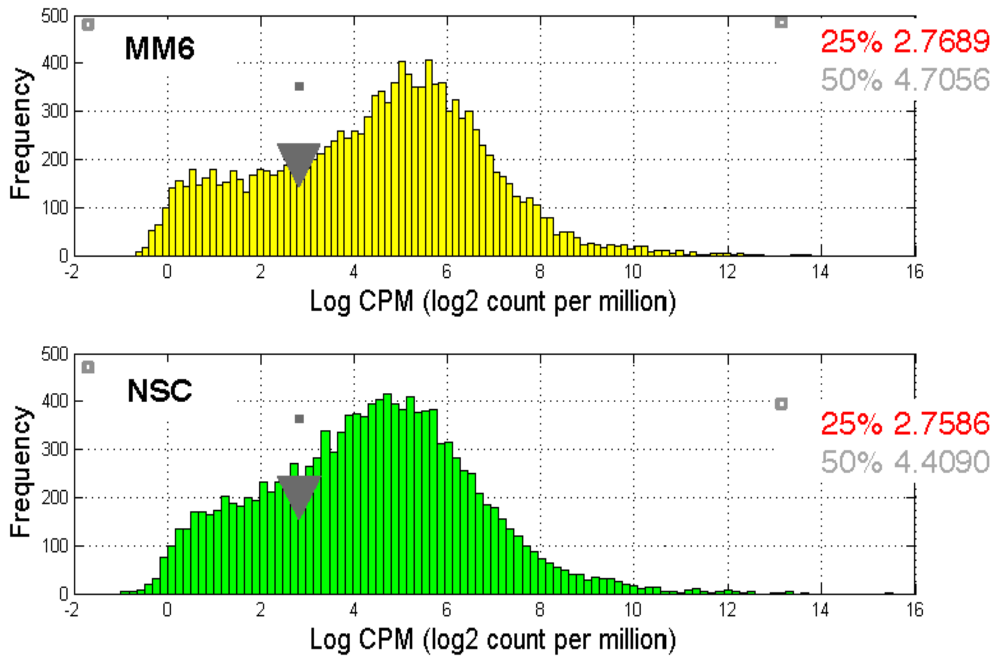

Supplement: Supplement A [file srep29179-s1.pdf]
